# ﻿Flora of Cameroon – Annonaceae Vol 45

**DOI:** 10.3897/phytokeys.207.61432

**Published:** 2022-09-20

**Authors:** Thomas L. P. Couvreur, Leo-Paul M. J. Dagallier, Francoise Crozier, Jean-Paul Ghogue, Paul H. Hoekstra, Narcisse G. Kamdem, David M. Johnson, Nancy A. Murray, Bonaventure Sonké

**Affiliations:** 1 IRD, DIADE, Univ Montpellier, Montpellier, France Naturalis Biodiversity Center Leiden Netherlands; 2 Université de Yaoundé I, Ecole Normale Supérieure, Département des Sciences Biologiques, Laboratoire de Botanique systématique et d’Ecologie, B.P. 047, Yaoundé, Cameroon Univ Montpellier Montpellier France; 3 Naturalis Biodiversity Center, Botany Section, Darwinweg 2, 2333 CR Leiden, Netherlands Université de Yaoundé I Yaoundé Cameroon; 4 Green Connexion, Environmental Group, siège face GP Mélen, à côté de l’immeuble Palais des verres. Yaoundé, Cameroun Green Connexion Yaoundé Cameroon; 5 Department of Botany-Microbiology, Ohio Wesleyan University, Delaware, OH, 43015, USA Ohio Wesleyan University Delaware United States of America

**Keywords:** Africa, botanical identification, conservation, diversity, lectotypification, new species, taxonomy, vascular plants

## Abstract

Annonaceae is a major pantropical family with 113 genera and about 2550 species. Cameroon is one of the most biodiverse countries in Africa but its flora remains incompletely known. In this volume of the Flora of Cameroon, we describe 166 native taxa representing 163 species in 28 native genera within the family Annonaceae. A total of 22 species (about 13%) are endemic to the country. We provide keys to all native genera, species, and infraspecific taxa. For each species a detailed morphological description and a map of its distribution in Cameroon are provided. Distribution maps and diversity analyses are based on a taxonomically verified database of 2073 collections. Across Africa, Cameroon is a center of diversity for Annonaceae harboring one of the highest numbers of species and genera. For example, Cameroon harbors the highest number of African species for the only pantropical genus of Annonaceae, ﻿*Xylopia*. Annonaceae are found across all 10 administrative regions of Cameroon but diversity is concentrated within the tropical rain forest areas situated in the south and South-West. The areas around Bipindi and Mount Cameroon show the highest levels of diversity, but this is correlated with collection effort. Line drawings and/or photographs accompany most species. One species new to science ﻿﻿﻿*Uvariopsisetugeana* Dagallier & Couvreur **sp. nov.** is described. We also undertake a number of nomenclatural changes such as lectotypifications, six new synonymies and two new combinations (﻿﻿﻿*Uvariaanisotricha* (Le Thomas) Couvreur, **comb. nov.**; ﻿﻿﻿﻿﻿Uvariodendronfuscumvar.giganteum (Engl.) Dagallier & Couvreur, **comb. nov.**).

## Abstract (fr)

Les Annonacées sont une grande famille pantropicale avec 113 genres et 2550 espèces. Le Cameroun est l’un des pays les plus riches en biodiversité d’Afrique mais sa flore reste incomplètement connue. Dans ce volume de la Flore du Cameroun, nous décrivons 166 taxons représentant 163 espèces dans 28 genres au sein de la famille des Annonaceae. Au total, 22 espèces (environ 13%) sont endémiques du pays. Nous fournissons une clé de tous les genres et espèces et des infra-espèces au sein des genres. Pour chaque espèce une description morphologique détaillée et une carte de sa répartition au Cameroun sont fournies. Les cartes de distribution et les analyses de diversité sont basées sur une base de données taxonomiquement vérifiée de 2073 collections. À travers l’Afrique, le Cameroun est un centre de diversité pour les Annonacées abritant l’un des plus grands nombres d’espèces et de genres. Par exemple, le Cameroun abrite le plus grand nombre d’espèces africaines pour le seul genre pantropical d’Annonaceae, ﻿*Xylopia*. Les Annonaceae sont présentent dans les 10 régions du Cameroun, mais la plus grande diversité est concentrée dans les régions abritant la forêt tropicale humide située au sud et au sud-ouest. Les zones autour de Bipindi et du Mont Cameroun présentent les niveaux de diversité les plus élevés, mais cela est corrélé à l’effort de collecte. Des dessins et/ou des photographies accompagnent la plupart des espèces. Une espèce nouvelle pour la science ﻿﻿﻿*Uvariopsisetugeana* Dagalier & Couvreur **sp. nov.** est décrite. Nous entreprenons également un certain nombre de changements nomenclaturaux tels que des lectotypifications, six nouvelles synonymies et deux nouvelles combinaisons (﻿﻿﻿*Uvariaanisotricha* (Le Thomas) Couvreur, **comb. nov.**; ﻿﻿﻿﻿﻿Uvariodendronfuscumvar.giganteum (Engl.) Dagalier & Couvreur, **comb. nov.**).

## ﻿Introduction

Annonaceae is a large pantropical family of trees, shrubs and lianas (Keßler 1993; Chatrou et al. 2012). Across Africa, and in Cameroon in particular, Annonaceae play an important role both in terms of species diversity and individual density (Kenfack et al. 2007; Sonké and Couvreur 2014). The famous French botanist André Aubréville (1897–1982) said of Annonaceae that they are among the families that best characterize floristically tropical rain forests in Africa (Aubréville 1970). Just over 50 years ago, Annick Le Thomas published the Annonacées (n°16) in the series Flore du Gabon (Le Thomas 1969b), a foundational treatment that served and continues to serve as the basis for Annonaceae identification across Africa together with other floras from other regions (Boutique 1951b; Robson 1960; Paiva 1966; Verdcourt 1971a; Hawthorne and Jongkind 2006). Our knowledge of African Annonaceae has increased during the last 15 years, with numerous taxonomic revisions being published (Versteegh and Sosef 2007; Couvreur 2009, 2014; Botermans et al. 2011; Fero et al. 2014; Ghogue et al. 2017; Gosline et al. 2018; Johnson and Murray 2018; Hoekstra et al. 2021). This, together with extensive field work across the country over the years, has provided a wealth of information that has allowed the compilation of the present work.

## ﻿Material and methods

### ﻿Morphological data matrix and species descriptions

Morphological species descriptions were automatically generated before manual checking and editing. We used the online collaborative platform PROTEUS (Sauquet 2016). This platform allows data and the source or citation of the data to be entered, permitting traceability of the information. We constructed a list of general morphological characters we wanted to use in the species descriptions. This set of characters works for most species, however, manual editing was needed for some genera, in particular for those with non-bisexual species, where different measurements were needed for male, female and/or bisexual inflorescences and flowers.

Data was gathered from two main sources. First, we used all available taxonomic revisions of African Annonaceae genera (e.g. Chatrou 1998; Couvreur 2009; Johnson and Murray 2018) or floras (e.g. Boutique 1951b; Le Thomas 1969b) to gather data on measurements and morphological characters. In most cases, these data were also checked and re-measured on herbarium specimens. Second, we made additional measurements for species without available taxonomic descriptions and of characters not mentioned in available sources. In several cases, measurements were taken from high quality scans of type material available on JSTOR (https://plants.jstor.org/). For all species, we tried to provide a rough estimate of the total number of stamens. When not explicitly reported in prior studies (e.g. ﻿*Xylopia*, Johnson and Murray 2018), these were counted either using photographic material or on herbarium specimens. Stamen numbers were estimated when species had more than 50 stamens per flower; in a few cases where an estimate was not possible they were termed “numerous”. For some species a detailed count was undertaken by Meinke (2008).

A first draft of descriptions for all species was generated using the package *MonographaR* (Reginato 2016) under the R environment. This assured that descriptions were always parallel. Some characters are repeated across individual species descriptions even if they are constant across the genus (e.g. habit, number of perianth whorls). This deviates from the main approach in taxonomic species descriptions. However, it was adopted here because it allows easier extraction of trait data by outside sources. Indeed, by providing a full description of a species (with all variable or important constant characters) one does not have to search for constant character information in different descriptions (genus or family). For each species, a final description was then prepared. For some genera, we added characters not found in other genera because they were important for identification. For example, the color of the sarcotesta in ﻿*Xylopia* was added because it is a useful character, generally not present or reported in other Annonaceae genera. The same goes for ﻿*Monanthotaxis* or ﻿*Uvaria* (pubescence of young foliate branches). Descriptions for non-bisexual species also deviated slightly from the rest of the descriptions in order to accommodate the description of the male, female and/or bisexual flowers within species.

### ﻿Cameroonian collection database

A database of Annonaceae collections from Cameroon was generated in which we recorded collector number, location, coordinates, region and herbarium where the specimens are deposited. A collection represents a herbarium sample identified by having the same collector and number (when present). It may be composed of one or more specimens, and such duplicates can be deposited in different herbaria. We used several sources as primary data providers to build the database. The initial database was based on data extracted from the “Réseau Informatique des Herbiers d’Afrique” (RIHA). This database contains all specimens held in the Herbier National du Cameroun/National Herbarium of Cameroon. We extracted all Annonaceae from Cameroon. We then supplemented this database with other available databases: BRAHMS (Naturalis Biodiversity Center, Leiden, The Netherlands); TROPICOS (Missouri Botanical Garden, St Louis, USA); Kew Database (Royal Botanical Gardens, Kew, UK) and other databases (T.L.P. Couvreur (IRD); V. Droissart (IRD); D.J. Harris (E); N. Kamdem (Université de Yaoundé I)). As much as possible specimens were checked in herbaria to confirm their identification. Thus, the database contains specimens that we have seen and confirmed, and others which we did not see. For genera with recent taxonomic revisions, determinations were updated (Chatrou 1998; Versteegh and Sosef 2007; Couvreur 2009, 2014; Botermans et al. 2011; Fero et al. 2014; Ghogue et al. 2017; Gosline et al. 2018; Johnson and Murray 2018).

Collections without coordinates were georeferenced using QGIS ver. 3.2.3 (QGIS Development Team 2019) and the IGN maps for Cameroon or the online gazetteer Geo-Locate (http://geo-locate.org/). Distribution maps were then generated using the package ‘MonographaR’ (Reginato 2016) under the R environment using a modified script of the ‘mapBatch’ function. A shapefile containing the outline of the Cameroon border and all ten regions was used (regions are the highest administrative divisions in Cameroon, formerly known as “provinces”, but changed to “regions” in 2008). In addition, we used a shape file of protected areas across Cameroon. These include Faunal Reserves, Flora Sanctuaries, National Parks, Ramsar Sites (wetlands of international importance), UNESCO Biosphere Reserves and Wildlife Sanctuaries. A shape file of these protected areas was downloaded from the https://www.protectedplanet.net/ on the 28^th^ of April 2020 and filtered for the country Cameroon. A list of these protected areas is available in Suppl. material 1: Fig. S1.

### ﻿Herbaria visited and field work

Within this project, numerous herbaria were visited over the course of the last eight years including B, BR, BRLU, G, K, P, YA and WAG. Within the taxonomic revision of certain genera, specimen loans were made available from other herbaria (BM, MO, OWU). In addition, we used specimen scans available online from these different herbaria when possible and needed. Numerous field trips were carried out across Cameroon over a period of eight years (2012–2019), mainly in the regions Central, East, Littoral, South West and West regions. During these field trips, high quality herbarium collections were made and deposited at MPU, YA and WAG. Finally, detailed photographs of the different parts available (leaves, trunk, flowers and fruits) were made and used to illustrate species found within this flora.

### ﻿Diversity maps

The database was used to generate collection, species and genus raw diversity maps. Collection density was log transformed before plotting. After filtering for unidentified species and genera, raw diversity maps were made at 0.25° resolution sampling units (SU) were plotted using the ‘ggplot2’ package (Wickham 2011). Finally, a Shapiro-Wilk’s (for non-normal data) correlation test was done between number of collections and species per 0.25 sampling unit using ‘ggscatter’ function in ‘ggplot2’.

### ﻿Collection citations

Collection citations and the index to numbered collections were generated using the package ‘exsic’ (Simon and Spooner 2013) under the R environment. For species with more than 30 collections, a subsample of these collections is listed after each species (“Selected specimens examined”). In this case, we cited at least one collection per region where the species is known to occur. Alternatively, the section “Specimens examined” lists all specimens seen by at least one of the authors or identified by a known Annonaceae specialist. When several herbaria are cited after each specimen, we do not indicate what individual specimen we saw, but assume they are deposited in the cited herbaria. All collections and our latest identifications are listed in “indexed number of collections” section (see Appendix 1).

### ﻿Line drawings

As much as possible we tried to use the original line drawings drawn by Hélène Lamourdedieu, intended by Annick Le Thomas for this flora. Thanks to Thierry Deroin, we had access to her archives at the Muséum national d’Histoire Naturelle in Paris, where we found numerous line drawings not published in the Flore du Gabon. The numbering of drawings was retained when possible. In some cases, specimens used for drawing a specific species changed (new identification). In other cases drawings came from more recent taxonomic revisions.

### ﻿IUCN Conservation status

The IUCN conservation status of each species was downloaded from the IUCN Red List website (www.iucnredlist.org). Official IUCN published evaluations are provided here, except for the genus ﻿*Monanthotaxis* where the preliminary status were taken from Hoekstra et al. (2021) and cited as “Preliminary”. Otherwise, the mention “not evaluated” is indicated. Most African Annonaceae tree species evaluations were undertaken by Ariane Cosiaux as part of the IUCN SSC Global Tree Specialist Group objectives. Thus, most liana species do not have published conservation statuses yet. For each evaluation the citation is provided. Assessments were downloaded on 29^th^ May 2020 from https://www.iucnredlist.org/ filtering on Annonaceae and Cameroon (and updated on 1^st^ May 2022).

## ﻿Results

### ﻿Diversity

We document a total of 28 native genera, 167 native taxa and 163 native species of Annonaceae in Cameroon (Table 1). One species has two subspecies (﻿*Annonasenegalensis*) and three species (﻿﻿﻿*Artabotrysaurantiacus*, ﻿*A.insignis*, ﻿﻿﻿*Uvariodendronfuscum*) have two varieties. ﻿﻿﻿*Uvariamuricata* is only known to date by the variety *yalingensis* in Cameroon. The most diverse genus is this flora is ﻿*Monanthotaxis* (a genus of lianas) with 31 species, followed by ﻿*Xylopia* (a genus of mainly trees) with 22 species, while eight genera are known by only one species (Table 1). Just over half of all genera have more than 50% of their species diversity in Cameroon, and for five genera Cameroon harbors 100% of the known species (Table 1). Cameroon is a center of diversity for four genera with more than 10 species: ﻿*Monanthotaxis* (39% of the diversity), ﻿*Uvariopsis* (68% of the diversity), ﻿*Piptostigma* (100% of the diversity) and ﻿*Xylopia* (49% of the African diversity). No genus is endemic to Cameroon, but 22 (~13%) species are endemic to the country (Table 2).

**Table 1.** List of number of 28 native genera recorded for Cameroon plus 12 continental African genera not found in Cameroon, with known accepted number of species in Cameroon, for continental Africa (including the Gulf of Guinea Islands, but excluding Madagascar) and percentage of species for each genus found within Cameroon. Total species diversity numbers were taken from Guo et al. (2017b) for endemic continental African genera, and from the African Plant Database for non-endemic genera (*Artabotrys*, *Uvaria*). For *Isolona* continental diversity was taken from Couvreur (2009), *Mischogyne* from Gosline et al. (2018) and *Xylopia* from Johnson and Murray (2018). ^1^*Afroguatteria*, *Monanthotaxis* and *Sphaerocoryne* include species formally placed in *Friesodielsia*; ^2^*Neostenanthera* includes *Boutiquea*.

Genus	Number of species in Cameroon	Number of species in continental Africa	Percentage in Cameroon
*Afroguatteria ^1^*	1	3	33
*Annickia*	4	8	50
*Annona*	1	4	25
*Anonidium*	2	4	50
*Artabotrys*	8	32	25
*Brieya*	1	2	50
*Cleistopholis*	3	3	100
*Dennettia*	1	1	100
*Duguetia*	4	4	100
*Greenwayodendron*	2	5	40
*Hexalobus*	4	5	80
*Isolona*	9	15	60
*Letestudoxa*	2	3	67
*Meiocarpidium*	1	1	100
*Mischogyne*	1	5	20
*Monanthotaxis ^1^*	31	79	39
*Monodora*	6	14	43
*Neostenanthera ^2^*	3	5	60
*Piptostigma*	13	13	100
*Polyceratocarpus*	3	8	38
*Sirdavidia*	1	1	100
*Sphaerocoryne ^1^*	1	2	50
*Toussaintia*	1	4	25
*Uvaria*	17	77	23
*Uvariastrum*	3	5	60
*Uvariodendron*	5	18	28
*Uvariopsis*	13	19	68
*Xylopia*	22	45	49
*Asteranthe* (3), *Cleistochlamys* (1), *Dielsiothamnus* (1), *Huberantha* (4), *Lettowianthus* (1), *Lukea* (2), *Mkilua* (1), *Monocyclanthus* (1), *Mwasumbia* (1), *Ophrypetalum* (1), *Pseudartabotrys* (1), *Sanrafaelia* (1).	0	19	0
***Total***:	**163**	**399**	**41**

**Table 2.** List of the 22 endemic species of Annonaceae recorded for Cameroon.

Genus	Species epithet	Author(s)
*Afroguatteria*	*discostigma*	(Diels) X.Guo & R.M.K.Saunders
*Artabotrys*	*dielsiana*	Le Thomas
*Hexalobus*	*bussei*	Diels
*Monanthotaxis*	*couvreurii*	P.H.Hoekstra
*Monanthotaxis*	*dielsiana*	(Engl.) P.H.Hoekstra
*Monanthotaxis*	*elegans*	(Engl. & Diels) Verdc.
*Monanthotaxis*	*hexamera*	P.H.Hoekstra
*Monanthotaxis*	*submontana*	P.H.Hoekstra
*Monanthotaxis*	*zenkeri*	P.H.Hoekstra
*Monodora*	*zenkeri*	Engl.
*Piptostigma*	*goslineanum*	Ghogue, Sonké & Couvreur
*Piptostigma*	*longepilosum*	Engl.
*Piptostigma*	*macrophyllum*	Ghogue, Sonké & Couvreur
*Piptostigma*	*mayndongtsaeanum*	Ghogue, Sonké & Couvreur
*Piptostigma*	*submontanum*	Ghogue, Sonké & Couvreur
*Uvaria*	*mollis*	Engl. & Diels
*Uvariopsis*	*dicaprio*	Gosline & Cheek
*Uvariopsis*	*etugeana*	Dagallier & Couvreur
*Uvariopsis*	*korupensis*	Gereau & Kenfack
*Uvariopsis*	*sessiliflora*	(Mildbr. & Diels) Robyns & Ghesq.
*Uvariopsis*	*submontana*	Kenfack, Gosline & Gereau
*Uvariopsis*	*zenkeri*	Engl.

### ﻿Annonaceae distribution and diversity in Cameroon

**Figure 1.**
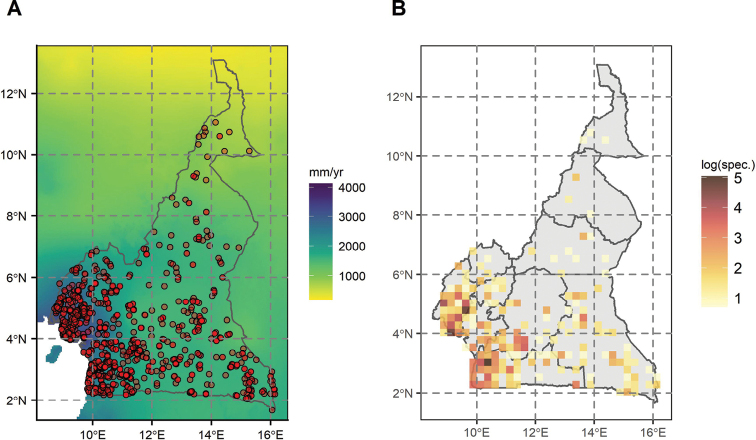
Distribution of Annonaceae collections in Cameroon. Based on 2803 georeferenced collections (out of 2857), including undetermined ones. Map represents Cameroon with regional borders (Admin 1 level). Altitude is given in meters.

A total of 2073 collections were seen for this treatment (Fig. 1A). Of these 1973 were identified to species level or below, while 17 were only identified as Annonaceae sp. These were mainly sterile collections. The dataset used is available on the GBIF platform (https://www.gbif.org/dataset/b738ab95-44a3-4d51-9ac6-3c0971e23a6f and has the DOI: https://doi.org/10.15468/ewp59s).

Annonaceae have been collected from all 10 regions in Cameroon, but most sampling comes from the southern regions of the country (Fig. 1A, B) with the South Region having the highest number of Annonaceae collections while the Far-North had the fewest (most diverse: Littoral Region: 139; Central Region: 264; East Region: 258; South Region: 631; South-West Region: 612; Fig. 1A, B). The highest number of collections for a single SU is 155 (around Bipindi; Fig. 1B) and the mean number of collections per SU is 9.2.

**Figure 2.**
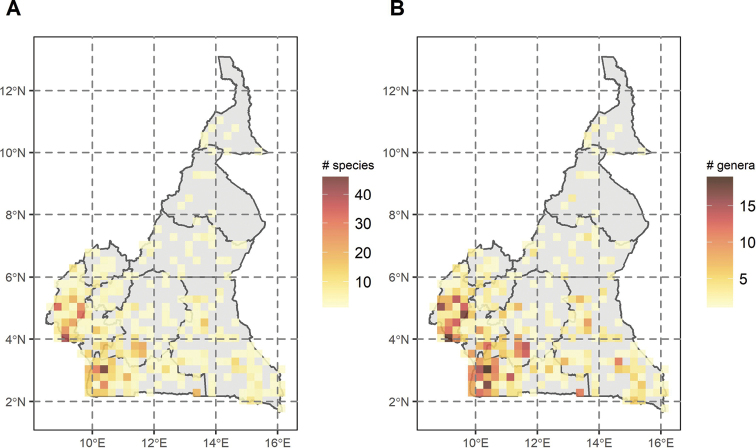
Spatial diversity of Annonaceae in Cameroon **A** log transformed collection density **B** raw species diversity **C** raw generic diversity **D** scatter plot of number of species in function of collections per SU, with correlation parameter R and *p value*. Maps represent Cameroon with limits between regions. Sampling units (SU) are of 0.25.

In terms of species diversity, there are two main hotspots, one located in the SU around Bipindi in the northwestern South Region (with 46 species recorded), and one in the SU around Mount Cameroon in the South-West region (with 40 species recorded) (Fig. 2B). Regions of high Annonaceae species diversity are mainly located in the Atlantic forests (around Yaoundé, southern Cameroon Volcanic Line, and western South region (Bipindi, Kribi, Campo) (Letouzey 1968), and to a lesser level in the southern part of the East region (Fig. 2A, B). Species diversity is significantly correlated with collection density (Spearman’s Rank: Rho_species_ = 0.98, Rho_genus_ = 0.97; *P < 0.001*).

Genus diversity is also concentrated in the Atlantic forests, with hotspots along the Cameroonian Volcanic Line, and towards the western area of the South region. Once again, the SU around Bipindi has the highest generic diversity with 19 (mean: 3.6 genera / SU) recorded genera (Fig. 2A). Genus diversity is also significantly correlated with collection density (Spearman’s Rank: Rho_genus_ = 0.97; *P < 0.001*).

**Figure 3.**
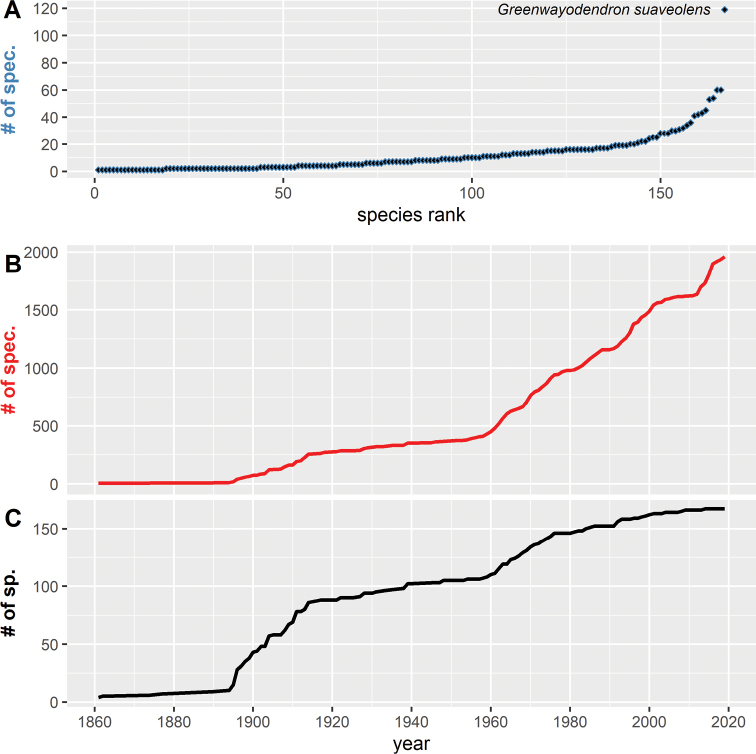
Sampling history of Annonaceae in Cameroon **A** ranked number of collections (specimens) per taxa (including subspecies and varieties) **B** cumulative number of Annonaceae collections (specimens) through time from 1861 to 2019 **C** cumulative number of Annonaceae species through time from 1861 to 2019. This graph is based on 2060 herbarium collections (with known year of collection)

﻿*Xylopia* is the most collected genus across Cameroon (277 collections), followed by ﻿*Monanthotaxis* (196 collections) (Table 3), while the genera ﻿*Sirdavidia* and ﻿*Toussaintia* are only known by a single Cameroonian collection to date. ﻿﻿﻿*Greenwayodendronsuaveolens* is the most collected species (119 collections, Fig. 3A), almost twice as many as the next five most commonly collected species (Table 3, Fig. 3A). Finally, 18 species are known from a single collection, 102 species are known from 10 or fewer collections, and five are known from 50 or more collections (Fig. 3A).

**Table 3.** Number of collections for all 28 genera recorded in Cameroon and for the top 27 species. Values based on collections between 1861 and 2019.

Genus	# specimens	Species	# specimens
*Xylopia*	277	*Greenwayodendronsuaveolens*	119
*Monanthotaxis*	196	*Annickiaaffinis*	60
*Uvariodendron*	165	*Uvariodendronconnivens*	60
*Monodora*	156	*Monodoramyristica*	54
*Uvariopsis*	135	*Anonidiummannii*	53
*Greenwayodendron*	134	*Xylopiaaethiopica*	45
*Piptostigma*	126	*Monanthotaxisenghiana*	43
*Artabotrys*	119	*Meiocarpidiumoliverianum*	42
*Uvaria*	115	*Xylopiathomsonii*	41
*Isolona*	80	*Monodoraundulata*	36
*Annickia*	73	*Monodoratenuifolia*	34
*Neostenanthera*	62	*Xylopiaquintasii*	32
*Anonidium*	59	*Uvariodendronmolundense*	31
*Hexalobus*	53	*Neostenantheraneurosericea*	30
*Cleistopholis*	46	*Xylopiaafricana*	30
*Annona*	44	*Artabotrysaurantiacus*	28
*Uvariastrum*	44	*Neostenantheramyristicifolia*	28
*Meiocarpidium*	42	*Uvariodendroncalophyllum*	28
*Duguetia*	40	Annonasenegalensissubsp.oulotricha	25
*Polyceratocarpus*	33	*Hexalobuscrispiflorus*	25
*Sphaerocoryne*	20	*Uvariodendronfuscum*	24
*Brieya*	15	*Uvariastrumzenkeri*	22
*Dennettia*	7	*Uvariopsisdioica*	22
*Letestudoxa*	6	*Polyceratocarpusparviflorus*	21
*Afroguatteria*	4	*Sphaerocorynegracilipes*	20
*Mischogyne*	3	*Uvariopsisbakeriana*	20
*Sirdavidia*	1	*Artabotrysthomsonii*	19
*Toussaintia*	1	*Duguetiastaudtii*	19

### ﻿IUCN Conservation status

A total of 95 species received a conservation status from the International Union for the Conservation of Nature, IUCN. However, we excluded the species ﻿﻿﻿*Uvariodendronfuscum* which now also includes formerly distinguished taxa (﻿*U.mirabile* and *U.giganteum*) and thus would need to be reassessed. In addition, the name ﻿﻿﻿*Boutiqueaplatypetala* is considered here to be a synonym of ﻿﻿﻿*Neostenantheraneurosericea*. We nevertheless used the available assessment of ﻿*B.platypetala* because ﻿*N.neurosericea* is only known from the type specimen (single location), and this nomenclature change does not affect the assessment. Thus, just over half (94 species) of the Cameroonian species have an official IUCN conservation assessment to date. Of these, only two are liana species (﻿﻿﻿*Uvariaangolensis*, ﻿*U.chamae*). Two species were evaluated using old criteria (version 2.3 and were published before 2000). Of the 94 species considered, 24 (Table 4) are evaluated as Threatened (25.5% of 94 species assessed): none are Critically Endangered, 10 are Endangered (10.6%) and 14 are Vulnerable (15%). Finally, two species are considered as Near Threatened (NT, 2.1%) and one as Data Deficient (DD, ﻿﻿*Xylopiatalbotii*; 1%). ﻿*Piptostigma* has the highest number of threatened species of any genus with seven (and one as NT), followed by ﻿*Xylopia* with five (and one as DD) (Table 4).

**Table 4.** List of the 24 Annonaceae species occurring in Cameroon officially assessed (and published) as Threatened following IUCN criteria. The assessment of *Neostenantheraneurosericea* is based on *Boutiqueaplatypetala* which is now a synonym of the former. *Uvariopsispedunculata* was assessed under the former name *U.vanderystii*.

Genus and species epithet	Red list category	Red list criteria	Year published	Criteria version
*Duguetiadilabens*	Endangered	B2ab(iii,v)	2020	3.1
*Hexalobusbussei*	Endangered	B1ab(ii,iii,iv)+2ab(ii,iii,iv)	2019	3.1
*Isolonapilosa*	Vulnerable	B2ab(iii)	2019	3.1
*Isolonapleurocarpa*	Endangered	B2ab(iii)	2019	3.1
*Mischogynegabonensis*	Endangered	B2ab(i,ii,iii,iv,v)	2021	3.1
*Neostenantheraneurosericea* (as *Boutiqueaplatypetala*)	Vulnerable	B2ab(iii)	2014	3.1
*Piptostigmacalophyllum*	Vulnerable	B2ab(iii)	2019	3.1
*Piptostigmagiganteum*	Vulnerable	B1+2c	1998	2.3
*Piptostigmagoslineanum*	Vulnerable	B1ab(iii)+2ab(iii)	2019	3.1
*Piptostigmalongepilosum*	Endangered	B2ab(iii,v)	2019	3.1
*Piptostigmamacrophyllum*	Vulnerable	B2ab(iii,iv)	2019	3.1
*Piptostigmaoyemense*	Vulnerable	B2ab(iii)	2019	3.1
*Piptostigmasubmontanum*	Endangered	B1ab(iii)+2ab(iii)	2019	3.1
*Sirdavidiasolannona*	Vulnerable	D2	2019	3.1
*Uvariodendrongiganteum*	Vulnerable	B2ab(iii)	2004	3.1
*Uvariopsiskorupensis*	Endangered	B2ab(iii)	2014	3.1
*Uvariopsissubmontana*	Endangered	B1ab(iii)+2ab(iii)	2014	3.1
*Uvariopsispedunculata*	Vulnerable	B2ab(iii)	2014	3.1
*Uvariopsiszenkeri*	Vulnerable	B2ab(i,ii,iii,iv,v).	2021	3.1
*Xylopiaafricana*	Vulnerable	A2c	2014	3.1
*Xylopiacalva*	Endangered	B2ab(iii)	2019	3.1
*Xylopiagilbertii*	Vulnerable	B2ab(iii)	2019	3.1
*Xylopiamildbraedii*	Vulnerable	B2ab(iii)	2019	3.1
*Xylopiapaniculata*	Endangered	B2ab(iii)	2019	3.1

Based on preliminary conservation assessments (Hoekstra et al. 2021), the liana genus ﻿*Monanthotaxis* has more than half of its species assessed as threatened (16/31). Four of these are Critically Endangered (CR), nine are Endangered (EN) and three are Vulnerable (VU).

## ﻿Discussion

### ﻿Diversity and distribution

Cameroon is a diversity hotspot for Annonaceae (Table 1). We recognize 28 native genera out of the 39 known (see Table 1) in continental Africa or ca. 72% of the total (excluding the two endemic genera from Madagascar, *Ambavia* and *Fenerivia*). The total number of genera cited above and in Table 1 includes the newly described genus *Lukea* Gosline & Cheek (with two species) from Tanzania and Kenya (Cheek et al. 2021). We here synonymize the genus ﻿*Boutiquea* with ﻿*Neostenanthera*, while all the African species of ﻿*Friesodielsia* were recently reassigned to three different genera, i.e. ﻿*Afroguatteria*, ﻿*Monanthotaxis* and ﻿*Sphaerocoryne* (Guo et al. 2017b). Most of the African genera not present in Cameroon are monotypic and occur in East Africa (e.g. *Lukea*, *Mkilua*, *Mwasumbia*, *Lettowianthus*, *Sanrafaelia*), Gabon (*Pseudartabotrys*) or West Africa (*Monocyclanthus*). In terms of species diversity, Cameroon harbors about ca. 41% (163 out of ca. 400 species) of the family’ continental species (estimated in Table 1). In terms of Annonaceae species diversity, Cameroon is the most species-rich country of continental Africa. Gabon, for example has 139 recorded species (Sosef et al. 2006) - the Flore du Gabon describes just 119 species (Le Thomas 1969b) but over the last 50 years, several new species or genera have either been described (Jongkind 2002; Couvreur et al. 2015; Couvreur and Niangadouma 2016; Hoekstra et al. 2021) or found to occur in Gabon (Lachenaud et al. 2018), so diversity is higher. Annonaceae represent almost 2% of the Cameroonian flora, which is estimated to harbor around 8000 species (Onana 2011; Sosef et al. 2017).

The increase of Cameroonian Annonaceae specimens or species through time has not been constant (Fig. 3B, C; see also Stropp et al. 2016). This discovery dynamic is similar to the one described for Gabon based on specimens for all plant families (Lachenaud et al. 2018). We can identify two main periods of important Annonaceae discovery: 1895 to 1915 and to a lesser extent 1960 to 1977. The first Annonaceae herbarium specimens documented for Cameroon were collected by the German botanist Gustav Mann (1836–1916) between 1861 and 1862. After that, few collections were made for over 30 years. Then, several German botanists started intensive collecting, in particular Gottfried Wilhelm Johannes Mildbraed (1879–1954) and Georg August Zenker (1855–1922). Zenker arrived in 1889 and was posted in Yaoundé, before moving to the village of Bipindi (South Region), at the base of the Ngovayang mountain range where he spent the rest of his life. This early botanical exploration explains the first strong increase in Cameroonian Annonaceae species starting in 1895 and ending in 1915 (Fig. 3B). The second most important period of Annonaceae discovery started around 1960 and coincided with two main events. First, this was the time when René Letouzey (1918–1989), a French botanist based at the Herbier National du Cameroun in Yaoundé, was active collecting specimens across the country. Second, is the period when Dutch botanists from the Wageningen Herbarium, in particular Jan-Just Bos (1939–2003), Frans Breteler, Anton Leeuwenberg (1930–2010), Willem JJO de Wilde (1936–2021) and Jan JFE de Wilde, made significant contributions to Annonaceae collections. This period also marks a significant increase in Annonaceae specimen collections in general, which continues until today (Fig. 3B). During the end of last century and the start of the present one, important contributions to the exploration and description of Annonaceae diversity in the country were undertaken by Cameroonian botanists such as Martin Etuge, Jean-Paul Ghogue, Narcisse Kamdem, David Kenfack, Moses Sainge, Bonaventure Sonké and Péguy G. Tchouto Mbatchou, together with foreign botanists such as Stuart Cable, Martin Cheek, Thomas Couvreur, George Gosline, David Harris, and Duncan Thomas. This exploration led to the last major increase in specimen collections from 2010 to 2019 but did not result in an increase in new species documented for the country (Fig. 3B, C).

Collecting intensity across the country is highly heterogeneous, with certain regions densely collected (Bipindi region, Mount Cameroon, Yaoundé area), whereas others have few or no collections (Fig. 1B) . This is the case for example in the southern part of the East Region, in the national Parks of Nki and Boumba Bek where no collections have been made yet for Annonaceae or for the flora in general (e.g. Sosef et al. 2017). Species diversity is mainly concentrated along the Atlantic forest of southern Cameroon (Fig. 2A), a diversity pattern also reflected in the overall flora (Cheek et al. 2001; Sosef et al. 2017) and for endemic or rare species (Onana 2013), including endemics within major families such as Orchidaceae or Rubiaceae (Droissart et al. 2012). Bipindi and the Ngovayang mountain range appear as a hotspot of Annonaceae diversity (Fig. 2A), partially related to the intense collecting of Zenker in the area (see above). The Ngovayang range is an important hotspot of plant diversity, but is currently under mining threat (Gonmadje et al. 2012; Droissart et al. 2019). Another center of diversity is found around the Mount Cameroon region, also a hotspot of diversity in general for the African flora (Cable and Cheek 1998; Onana 2013).

### ﻿Conservation

Just over a quarter (25.5%, 24/93) of assessed Annonaceae species occurring in Cameroon have been evaluated as threatened at global levels under IUCN criteria (Table 4). This is below the level of threat across the tropical African flora in general (Brummitt et al. 2015; Stévart et al. 2019). Of the 155 Annonaceae taxa included in the study of Onana (2011), just nine were evaluated as threatened (VU or EN). The available assessments to date also do not include potentially threatened species know from very few collections such as the newly described ﻿﻿﻿*Uvariopsisetugeana* (this flora) and ﻿﻿﻿*Uvariopsisdicaprio* (Gosline et al. 2022). Most other species not assessed belong to diverse liana genera (e.g. ﻿*Artabotrys*, ﻿*Uvaria*) which have yet to be properly assessed. The genus ﻿*Monanthotaxis*, based on a preliminary assessment has more than 50% of its species in a threat category. This underscores once again the importance of taking liana species into account when undertaking assessments.

### ﻿Morphology of Cameroonian Annonaceae

Cameroonian Annonaceae provide an important sample of the morphological variability encountered across the family in general. Below we provide a brief overview of this variability. All these observations concern Cameroonian Annonaceae, unless stated otherwise.

#### Habit

In Cameroon, Annonaceae can be large emergent trees (e.g. several species of ﻿*Xylopia* more than 30 m in height), understory trees (e.g. ﻿*Uvariodendron* sp. between 3 and 15 m in hight), shrubs (﻿﻿﻿*Neostenantheraneurosericea*), scrambling shrubs (i.e. lianas not growing along a tree, but mainly growing on the ground producing long bended stems; some species in ﻿*Uvaria*) and lianas (e.g. ﻿*Artabotrys*, ﻿*Monanthotaxis*). No epiphytic species are known in the family. If we just look at the dichotomy between liana and tree habits, there are 62 liana species (38%) versus 99 tree species (62%). Some species are intermediate between a liana and tree habit. This is the case for example of ﻿﻿﻿*Neostenantheramyristicifolia*, ﻿﻿*Xylopiathomsonii*, or ﻿﻿﻿*Monodoracrispata*, which can appear scandent, leaning on surrounding vegetation (Couvreur 2009; Fero et al. 2014).

Annonaceae exhibit two growth architecture patterns (Hallé et al. 1978). In the genera occurring in Cameroon, spiral arrangement of branches on the primary axis has been documented in ﻿*Annickia*, ﻿*Artabotrys*, ﻿*Duguetia*, ﻿*Greenwayodendron*, and ﻿*Xylopia* (Johnson 2003) and ﻿*Polyceratocarpus* (Marshall et al. 2016), and distichous arrangement of branches on the primary axis occurs in ﻿*Annona*, ﻿*Cleistopholis*, ﻿*Hexalobus*, ﻿*Isolona*, ﻿*Monanthotaxis*, ﻿*Monodora*, ﻿*Sphaerocoryne*, ﻿*Toussaintia*, ﻿*Uvaria*, and ﻿*Uvariodendron* (Johnson 2003). Where known, these patterns are useful for field identification of sterile plants, including seedlings. The spiral versus distichous patterns do not correlate with habit, inflorescence position, or sympodial versus monopodial growth, but are highly conserved in most major clades of the family (Johnson 2003).

#### Trunk

Most Annonaceae species have a smooth and cylindrical trunk with no buttresses or stilt roots. The genus ﻿*Xylopia* is an exception with a range of basal structures from small or large buttresses to stilt roots or no structure at all. This provides a useful character for species identification (Johnson and Murray 2018). The color of the trunk is also a good character with some species having a very white bark with patches of darker grey (﻿﻿﻿*Anonidiummannii*, ﻿﻿﻿*Cleistopholisglauca*, ﻿﻿﻿*Greenwayodendronsuaveolens*, ﻿﻿*Xylopiahypolampra*). Some species are characterized by a deeply furrowed trunk, for example ﻿﻿﻿*Isolonahexaloba*, ﻿*I.zenkeri*, and ﻿﻿﻿*Hexalobuscrispiflorus*. Another character of Annonaceae is the thick and tough bark which easily peels off in a single strip, a useful character to identify Annonaceae. In the genus ﻿*Annickia*, the inner side of the bark is of a characteristic bright yellow colour (Versteegh and Sosef 2007).

#### Leaves

Leaves in Annonaceae are alternate, distichous (in a single plane) and lack stipules. The single plane disposition of the leaves confers a characteristic look to Annonaceae in the forest. Moreover, most species have clearly plagiotropic (horizontally spreading) branches (e.g. ﻿*Greenwayodendron*). Leaves are always entire and the margins are never serrate or dentate. Petiole length varies from 1 to 20 mm, the longer petioles being found in species such as ﻿*Annonasenegalensis*, ﻿﻿﻿*Cleistopholisglauca* and ﻿﻿﻿*Uvariodendroncalophyllum*.

The leaf blade is inserted either on the top or to the side of the petiole (see fig. 2 of Couvreur 2009). When inserted on top of the petiole, the blade appears to “pinch” the petiole. This gives a distinctive appearance to the base of the leaves and can be a useful taxonomic character to identify sterile collections, for example in ﻿*Isolona* (Couvreur 2009). We have added this character in the descriptions although it isn’t always reported in the taxonomic literature.

Within Cameroonian species, leaf size varies more than 20-fold, from 3–4 cm long in species such as ﻿﻿﻿*Uvariaklaineana* or ﻿﻿*Xylopiapynaertii*, to up to more than 60 cm long in species such as ﻿﻿﻿*Piptostigmasubmontanum* or ﻿﻿﻿﻿﻿Uvariodendronfuscumvar.giganteum. However, most species have intermediate-sized leaves between 10 and 30 cm long.

Most Annonaceae species have concolorous leaves, being green on both sides of the leaf blade. Some genera and species, however, can be distinguished by having discolorous leaves, with a much lighter whitish green color of the lower side of the leaf blade, e.g. ﻿*Afroguatteria*, ﻿*Brieya*, ﻿*Cleistopholis*, ﻿*Monanthotaxis*, ﻿*Piptostigma*, ﻿*Polyceratocarpus*, ﻿*Neostenanthera*, ﻿*Sphaerocoryne*, ﻿*Uvaria* or some species of ﻿*Xylopia*.

#### Venation

In most species, the midrib is flat or sunken on the adaxial surface. The genera ﻿*Isolona* and ﻿*Monodora* are unusual in African Annonaceae as they have a raised midrib (Couvreur 2009), and are thus easily distinguished when sterile. The species ﻿﻿﻿*Polyceratocarpuspellegrinii* also has a raised midrib, while the rest of the genus has a flat or sunken one (Le Thomas 1969b).

Secondary venation can provide a useful taxonomic character in Annonaceae. Most species have fewer than 20 pairs of secondary veins. The genus ﻿*Piptostigma* however, is characterized by leaves with a generally high number of parallel secondary veins (Ghogue et al. 2017), with most species having more than 20 pairs. ﻿﻿﻿*Piptostigmasubmontanum* has the highest number of secondary veins in Cameroonian Annonaceae, varying between 58 and 65 pairs. Tertiary venation is also a useful taxonomic character to identify Annonaceae genera or species and is either parallel (percurrent) or reticulate. The best way to see the tertiary venation is looking at the lower side of the leaves with a hand lens. Percurrent venation is less frequent, but is characteristic of the genera ﻿*Monanthotaxis*, ﻿*Neostenanthera*, ﻿*Piptostigma* and ﻿*Polyceratocarpus*. In addition, species within genera can have percurrent venation, for example in ﻿*Uvaria* (e.g. ﻿﻿﻿*Uvariabaumannii*, ﻿*U.poggei*). In some cases, the tertiary venation is indistinct either because of the presence of a thick layer of pubescence (e.g. ﻿﻿﻿*Uvariaklaineana*) or because the veins are not marked enough. In some cases, the venation is termed intermediate when veins appear both parallel and reticulate, for example in ﻿﻿﻿*Artabotrysthomsonii*, ﻿﻿﻿*Polyceratocarpuspellegrinii* or ﻿﻿﻿*Uvariamuricata*. Finally, the tertiary venation of ﻿﻿﻿*Toussaintiahallei* is unique, being very tightly reticulate.

#### Inflorescences

The inflorescences of Annonaceae species are termed monotelic, meaning that the apex of the inflorescence ends with a terminal flower (Weberling 1983; Weberling and Hoppe 1996). Lateral branching from a single prophyll leads to partial inflorescences termed “rhipidia” (Weberling and Hoppe 1996). From this basic structure emerges all the variation in inflorescence types found in Annonaceae, of which we will not provide an in depth review here (see Weberling and Hoppe 1996). In Cameroonian Annonaceae, we encounter a large spectrum of inflorescences, ranging from short single-flowered to very long and many times branched structures with numerous flowers (e.g. ﻿﻿﻿*Piptostigmamultinervium*). In the descriptions below we do not go into detail about inflorescence structure, except for certain genera (e.g. ﻿*Monanthotaxis*, ﻿*Piptostigma*) where inflorescence structure presents a useful taxonomic character.

An important character concerns the position of the inflorescences which are, in the most fundamental sense, either ‘axillary’ that is originating from an axillary meristem, or ‘terminal’ that is originating from a terminal meristem (Fries 1919; Le Thomas 1969b; Chatrou 1998; Maas et al. 2003). In the former case, the inflorescences appear in the axil of the leaves (or leaf scars when fallen), whereas in the latter they are positioned opposite or sub-opposite the leaves (or leaf scars when fallen), and in some cases they can even become extra-axillary (Maas et al. 2003). Thus, in this treatment, we use the terms ‘axillary’ to refer to axillary inflorescences and ‘leaf-opposed’ or ‘extra-axillary’ for terminal inflorescences. Inflorescences can occur on young foliate branches (recent leaf flush of up to 2 years old) or older branches with or without leaves (ramiflory, meaning here flowers on branches young and old). Finally, cauliflory whereby the flowers (and thus the fruits) originate directly from the trunk resulting from retardation of anthesis (Weberling and Hoppe 1996), is also a common character for Annonaceae. Cauliflory is present in 43 species. In some cases cauliflory is pushed to an extreme with the trunk almost completely covered with flowers, for example in ﻿*Uvariopsissubmontanum* (Kenfack et al. 2003) or ﻿﻿﻿*Piptostigmamultinervium* (Ghogue et al. 2017).

#### Flowers

Most species of Annonaceae have a clearly pedicellate flower, with pedicels generally shorter than 10 cm, and in most cases between 0.2 and 2 cm long. ﻿﻿﻿*Uvariopsiscongolana* has (female) pedicels up to 45 cm long that grow from the base of the trunk and along the forest floor (flagelliflory, see Schatz and Wendt 2004). Other species of the genus ﻿*Uvariopsis* can also have long pedicels, and in some cases the length varies between male and female flowers. Female flowers generally have longer and more robust pedicels than male flowers (e.g. ﻿*U.pedunculosa*). ﻿﻿﻿*Monodoramyristica* also has long pedicels reaching up to 27 cm long.

In most genera, the pedicel bears a lower and upper bract conforming to Fries’s type 2, the most common situation across Annonaceae (Fries 1919). The number of basal bracts may vary from 1 to numerous. The upper bract can be inserted at different levels along the pedicel, and we distinguish 3 possible cases: in the lower half, towards the middle or in the upper half of the pedicel. In most cases the bracts are minute (1–3 mm) and soon falling. However, in some species the upper bracts can be large (e.g. in ﻿*Anonidium*, ﻿*Letestudoxa*, ﻿*Monodora*) or leaf-like (e.g. ﻿﻿﻿*Isolonacampanulata*), and thus provide important taxonomic information. In several cases we were not able to observe the bracts and so the information is missing.

Annonaceae flowers are generally bisexual, with stamens and carpels within the same flower. However, some genera are androdioecious (male and bisexual flowers on different individuals, although this state needs to be confirmed with more detailed field observations), dioecious (male and female flowers on different individuals) or monoecious (male and female flowers on the same individual). In ﻿*Monanthotaxis* we can find bisexual and monoecious species. Several ﻿*Monanthotaxis* species with unisexual flowers have female flowers on the trunk while the male flowers are located high in the canopy in axils of the leaves, therefore collections often only contain male or female flowers. Monoecy has not been proven for ﻿*M.cauliflora* or ﻿*M.pynaertii*, but there are collections with both female and male flowers in other species such as *M.bidaultii*, ﻿*M.diclina*, ﻿*M.letouzeyi*. ﻿*Uvariopsis* is also monoecious, while ﻿*Anonidium* (but see under ﻿*A.brieyi*), ﻿*Greenwayodendron* and ﻿*Polyceratocarpus* are androdioecious (Le Thomas 1969b; Couvreur et al. 2015; Lissambou et al. 2018).

The receptacle or torus which bears the stamens on the basal part and/or the carpels towards the central apical part is quite variable within Annonaceae in general (van Heusden 1992). In Cameroon, most species have a generally flat or slightly concave receptacle (e.g. ﻿*Artabotrys*, ﻿*Monanthotaxis*). However, some genera and species are characterized by a strongly conical or extended receptacle such as in ﻿*Mischogyne*, ﻿*Uvariopsis*, ﻿*Toussaintia* or ﻿﻿﻿*Monodoramyristica* (Luke and Deroin 2005; Couvreur 2009; Gosline et al. 2018). In most species of ﻿*Xylopia* a ring or cone formed by the stamen filaments persists in the center of the torus.

The general floral pattern in Annonaceae is actinomorphic, cyclic and trimerous with one whorl of three sepals and two whorls of three petals each (van Heusden 1992). The two whorls of petals are referred to as outer and inner petals in the descriptions. From this general pattern several deviations occur which are useful generic-level characters. In ﻿*Annickia*, the outer petal whorl is absent, and thus the flower only has three inner petals which are opposite the three sepals. In ﻿*Uvariopsis*, most species have only two sepals and a single whorl of four petals, though ﻿*U.congolana* has a single whorl of three petals. ﻿﻿﻿*Monanthotaxistripetala*, ﻿﻿﻿*Uvariopsiscongolana* and ﻿﻿﻿*Dennettiatripetala* have three (or sometimes four) petals (Le Thomas 1969b; Kenfack et al. 2003; Hoekstra et al. 2021). Finally, ﻿*Toussaintia* is unique among Cameroonian Annonaceae in having 9 to 10 petals in 2 or 3 whorls (Le Thomas 1969b; Luke and Deroin 2005).

Sepals are mostly free or are basally fused. Sometimes it can be hard to distinguish between these two states. In ﻿*Letestudoxa* and some species of ﻿*Uvaria*, the sepals are completely fused into a tube or a “cup” and tear open (generally into three parts) during anthesis. In ﻿*Xylopia* the fused sepals often form a cup-shaped calyx. Petals are free in most species, but in the genera ﻿*Hexalobus*, ﻿*Isolona* and ﻿*Monodora* they are basally fused. In this case the petals form either in single whorl (﻿*Hexalobus*, ﻿*Isolona*) or two differentiated whorls (﻿*Monodora*). In the former case, the petals are identical in shape and size (referred to as “not differentiated” in the descriptions) and the petals are clearly fused basally; we refer to the non-fused part as “lobes” and fused part as the “tube” (Couvreur 2009; Botermans et al. 2011). In the latter case, the inner and outer petals have different shapes and sizes (referred to as “differentiated” in the descriptions), retaining the common Annonaceae pattern (Couvreur 2009), at least in appearance; in this case, we used the same terminology as for species with free petals, that is referring to inner and outer petals. Finally, several species within ﻿*Uvariopsis* also have basally fused petals (e.g. ﻿*U.congolana*).

Petal shape and size are very variable across Cameroonian species ranging from 1–2 mm long in male flowers of ﻿﻿﻿*Monanthotaxiscauliflora* (female flowers are larger) to 50 mm in ﻿﻿﻿*Anonidiummannii*, to 79 mm in ﻿﻿*Xylopiamildbraedii*,or even up to 100 mm in ﻿﻿﻿*Monodoramyristica*, and from circular (several species in ﻿*Monanthotaxis*) to linear (﻿*Artabotrys*, ﻿*Xylopia*). In some species, the inner petals are rounded and concave at the base, and form a pollination chamber (e.g. ﻿*Artabotrys*, ﻿*Neostenanthera*, ﻿*Xylopia*). Pollination chambers are also possible by connivance of the inner petal margins (but not fused together), for example in some species of ﻿*Monodora* or ﻿*Uvariodendron*.

#### Stamens

Stamen number varies from six in three species of ﻿*Monanthotaxis* (Hoekstra et al. 2021) to more than 5000 in ﻿﻿﻿*Uvariodendroncalophyllum* (Meinke 2008). The positioning of stamens on the receptacle has generally been referred to as spiral (e.g. Couvreur 2009), but a study indicated that the situation is not that simple with stamen insertion tending towards a complex whorled type pattern (Endress and Armstrong 2011). In any case, it is possible to distinguish stamen “row” numbers which can be useful for species identification (Couvreur 2009), and when possible we provided an estimate of the number of these rows. Stamens are always free, except in ﻿﻿﻿*Monanthotaxiscouvreurii* where the 13 to 15 stamens are basally fused into a single staminal ring (Hoekstra et al. 2016), and in many species of ﻿*Xylopia*, where the filaments are connate at the base to form a cone surrounding the carpels. The stamens are composed of an anther with two thecae containing the pollen joined by a connective. The thecae are septate (i.e. having with many horizontal septa visible with a hand lens; in opposition to aseptate) in ﻿*Neostenanthera* and ﻿*Xylopia* (a usefull generic-level character, Tsou and Johnson 2003) and aseptate in all other genera. The shape of the connective apex is quite variable among genera (van Heusden 1992). In most species, the apical part of the connective completely covers the top of the stamens and is discoid in shape, forming a flat rounded structure, protecting the stamens. However, the connective can also be tongue-shaped (apically prolonged), absent or reduced showing the thecae (e.g. ﻿*Mischogyne*) (Gosline et al. 2018). The genus ﻿*Monanthotaxis* has a wide range of stamen and connective shapes (Hoekstra et al. 2018). The connective apex can be a useful taxonomic character to distinguish certain closely appearing species (e.g. ﻿﻿﻿*Uvariaangolensis* versus ﻿*U.versicolor*). Staminodes (sterile stamens) occur in the flowers of ﻿*Monanthotaxis* and ﻿*Xylopia*.

#### Carpels

In Annonaceae, the carpels are generally free, that is apocarpous, within the flower (van Heusden 1992). Only the genera ﻿*Isolona* and ﻿*Monodora* have truly congenetically fused carpels or syncarpy (Endress 1982; Deroin 1985; Couvreur et al. 2008b; Couvreur 2009). Although there are always several fused carpels (versus the hypothesis that there is just a single carpel (Couvreur et al. 2008b)) in those two latter genera, we do not provide a number because that would necessitate anatomical observation (Deroin 1985; Couvreur 2009).

Carpel number varies from one in ﻿*Sirdavidia* to over 250 in ﻿﻿﻿*Uvariopsisdioica*. We provide a count for each species based on available material, but these numbers remain estimates in most cases. Most species have fewer than 20 carpels. Genera with pseudosyncarpous fruits (see below, ﻿*Annona*, ﻿*Anonidium*, ﻿*Duguetia*, ﻿*Letestudoxa*) generally have more than 50 carpels and up to 120. Other genera such as ﻿*Uvariodendron* or ﻿*Uvariopsis*, ﻿*Monanthotaxis*, ﻿*Neostenanthera* and ﻿*Uvaria* also have species which can have more than 50 carpels. Carpels are generally topped by a stigma which can be variable in shape from bilobed to globose or filiform (van Heusden 1992). Stigma morphology is particularly variable in ﻿*Xylopia* (Johnson and Murray 2018).

The ovules are either numerous and lateral in one or two rows (van Heusden 1992), or basal and one or rarely two in number (e. g., ﻿*Annona*, ﻿*Annickia*, ﻿*Duguetia*, ﻿*Neostenanthera*; (Chatrou 1998; Versteegh and Sosef 2007; Fero et al. 2014)). In this treatment we do not provide details about the ovule number and disposition.

#### Fruits

Most genera have aggregated fruits composed of individual units termed “monocarps” each resulting from the fertilization of a single carpel (van Setten 1990). In the genera ﻿*Isolona* and ﻿*Monodora*, the fruit is syncarpous and forms a single unit, the seeds having no apparent internal order (Couvreur 2009). In a few other genera, the carpels are free in the flower but fuse during fructification, resulting in a single fruiting unit termed a pseudosyncarpous fruit (Chatrou 1998). Fusion between carpels can be complete (e.g. ﻿*Annonasenegalensis*, ﻿﻿﻿*Duguetiabarteri*) or basal (e.g. ﻿﻿﻿*Duguetiadilabens*). In Cameroon, four genera have this type of pseudosyncarpous fruit: ﻿*Annona*, ﻿*Anonidium*, ﻿*Duguetia* and ﻿*Letestudoxa*.

Monocarps are either stipitate or sessile. When present, the stipes can be up to 5 cm long in certain species of ﻿*Annickia*, ﻿*Neostenanthera* or ﻿*Uvaria*, conferring to the characteristic “star shape” look to Annonaceae fruits. In ﻿*Annickia*, ﻿*Cleistopholis*, and ﻿*Neostenanthera*, the stipe is articulated at the apex. In some species, the stipe is short (less than 5 mm) but still present. A number of genera have sessile (no apparent stipe) or short-stipitate (less than 5 mm) monocarps, for example ﻿*Mischogyne*, ﻿*Uvariodendron* or ﻿*Uvariopsis*. Sessile and stipitate monocarps can occur within the same genus, for example in ﻿*Neostenanthera* or ﻿*Uvaria*. Monocarps of most Cameroonian genera are indehiscent, but in ﻿*Xylopia* the monocarps dehisce longitudinally along an abaxial suture.

#### Seeds

Annonaceae seeds are quite variable in size, ranging from 3 mm to more than 40 mm long. Generally in Annonaceae, the seeds are characterized by a ruminate endosperm, resulting from the invasion of the intertegument into the endosperm (van Setten 1990). Seeds generally do not have an aril, but it is present in some genera such as ﻿*Duguetia* and in particular ﻿*Xylopia*. Indeed, African ﻿*Xylopia* have five different aril types: absent, bilobed, brush-like, cupular and fimbriate (Stull et al. 2017; Johnson and Murray 2018). In Cameroon, four character states are present (absent, bilobed, brush-like and fimbriate). In addition, many ﻿*Xylopia* species in Cameroon have a thin pigmented sarcotesta covering the hard inner seed coat.

## ﻿Taxonomic treatment

### ﻿ Annonaceae

Taxon classificationPlantaeMagnolialesAnnonaceae

Juss. Gen. Pl.: 283, 1789 (as “Anoneae”) nom. cons.

862D2E96-2B22-592E-807E-EBC1676AA912

#### Description.

Trees, scrambling shrubs or lianas, up to 50 m tall, monoecious, dioecious or putatively androdioecious. Indumentum, when present, of simple, fasciculate, stellate, or scale-like hairs. Leaves alternate, simple, distichous, margins entire, stipules absent. Inflorescences terminal or axillary, ramiflorous in leaf axils, on young or old leafless branches or cauliflorous, single to many-flowered, pedunculate or subsessile, bracts often present. Flowers bisexual or unisexual, actinomorphic, generally trimerous. Sepals in a single whorl, (2)3(4), valvate or imbricate in bud, free or basally to fully connate. Petals 3, 4 or 6, in 1 or 2(3) whorls, generally differentiated into an inner and outer whorl alternating with the sepals, valvate or imbricate in bud, free, basally or fully connate. Stamens 3 to numerous, inserted onto a flat or convex receptacle; anthers generally exceeded by the connective apex, which forms a a protective cover at the top of the stamen; connective apex flat, extended (tongue shaped) or absent; filaments short or absent, free or rarely fused; staminodes absent or present. Carpels 1 to numerous, free or more rarely fused (syncarpous) in flower; stigma capitate, oblong or variously folded; ovules 1 to numerous, uni- or biseriate, basal or lateral. Fruit generally apocarpous, each carpel producing a single monocarp, or more rarely pseudosyncarpous (carpels fusing during fructification) or syncarpous (unilocular fruits resulting from syncarpous flowers), indehiscent or sometimes dehiscent; monocarps 1 to numerous, sessile to long-stipitate, cylindrical, globose, ovoid, ellipsoid, club-shaped or moniliform, 3 to over 40 mm in length, usually large; seeds 1 to numerous per monocarp, uni- or biseriate, or unordered in syncarpous species, sometimes with arilor sarcotesta; endosperm ruminate, hard.

#### Distribution.

Pantropical, from the Pacific and northern Australia to South East Asia (including southern China), India, Madagascar, tropical Africa, temperate eastern North America south to Central America and South America. 113 genera, and around 2550 species.

In Cameroon, 28 genera and 163 species reported to date.

#### Notes.

Several Annonaceae species have been introduced and are commonly cultivated across the country. *Monoonlongifolium* (Sonn.) B.Xue & R.M.K.Saunders (=*Polyalthialongifolia* (Sonn.) Hook.f. & Thomson) is sold and grown as an ornamental, and planted mainly along roads in major towns and gardens. *Canangaodorata* (Lam.) Hook.f. & Thomson (ylang ylang) is sold and planted as an ornamental, with its large flowers emitting a strong sweet scent especially at night. Several non-native species of ﻿*Annona* are planted in gardens for their large sweet fruits, especially ﻿﻿*A.muricata*L. ﻿﻿*Annonaglabra*L. is naturalized in coastal mangrove regions of West Africa including Cameroon. These non-native species are not treated here.

##### ﻿Key to the genera of Annonaceae in Cameroon

**Table:** 

1	Midrib of leaf blade clearly raised above	**2**
–	Midrib of leaf blade sunken,impressed, or flat above	**4**
2	Petals fused at base (even just shortly); fruits in a single unit (syncarpous)	**3**
–	Petals free; fruits in several independent monocarps (apocarpous)	﻿﻿﻿***Polyceratocarpuspellegrinii***
3	Corolla lobes similar and equal in length, forming a distinct tube at the base, margins generally flat	﻿***Isolona***
–	Corolla lobes clearly differentiated into inner and outer petals; the outer ones longer than inner ones, margins generally undulate or crisped	﻿***Monodora***
4	Liana or scrambling shrub	**5**
–	Tree or shrub	**14**
5	Hook-shaped structures (modified inflorescence) present on branches even in juvenile plants	﻿***Artabotrys***
–	Hook-shaped structures absent	**6**
6	Indumentum of stellate and/or fasciculate hairs	﻿***Uvaria* (pro parte)**
–	Indumentum (if present) of simple hairs	**7**
7	Anthers septate (few species)	**8**
–	Anthers not septate (most species)	**9**
8	Petals subequal; stipe shorter than seeded section of monocarp; indehiscent; several seeds per monocarp	﻿﻿***Xylopiathomsonii***
–	Outer petals longer than inner; stipe at least twice as long as seeded section of monocarp; monocarps indehiscent; seed 1 per monocarp	﻿﻿﻿***Neostenantheramyristicifolia***
9	Leaves bicolored; above green, below glaucous to whitish; monocarps moniliform when more than one seed, ovules uniseriate	**10**
–	Leaves green on both sides; monocarps not moniliform, globose to conical, ovules biseriate	**12**
10	Tertiary venation percurrent when viewed from below, or if venation obscure, then stamens < 35	﻿***Monanthotaxis***
–	Tertiary venation reticulate and stamens > 40	**11**
11	Inflorescences terminal (leaf opposed or extra-axillary)	﻿***Afroguatteria***
–	Inflorescences axillary	﻿***Sphaerocoryne***
12	Receptacle columnar or elongated; petals 6 to 12 in 2 or 3 whorls	﻿***Toussaintia***
–	Receptacle convex but not columnar; petals 6 in two whorls	**13**
13	Sepals entirely fused, enclosing flower in bud, tearing as flower enlarges; fruits pseudosyncarpous	﻿***Letestudoxa***
–	Sepals free or basally fused, not enclosing flower in bud; fruits apocarpous	﻿***Uvaria* (pro parte)**
14	Indumentum of scale-like hairs (easily visible with a hand lens)	﻿***Meiocarpidium***
–	Indumentum (if present) of stellate, fasciculate or simple hairs	**15**
15	Indumentum of stellate and/or fasciculate hairs and fruits pseudosyncarpous	﻿***Duguetia***
–	Indumentum of simple hairs, or glabrous; fruits mostly apocarpous (pseudosyncarpous in ﻿*Annona* and ﻿*Anonidium*)	**16**
16	Sepals 2; petals 4	﻿***Uvariopsis***
–	Sepals 3; petals 3 or 6	**17**
17	Inner bark/slash yellow; petals 3, opposite the 3 sepals	﻿***Annickia***
–	Inner bark/slash cream to reddish; petals 6, or if 3 then only 2 sepals present	**18**
18	Petals fused into a clear tube at the base, plicate (transversely folded) in bud	﻿***Hexalobus***
–	Petals free, petals not plicate (not folded in bud)	**19**
19	Outer petals reduced, sepal like, smaller than inner petals	**20**
–	Outer petals not reduced, subequal to or larger than inner petals	**21**
20	Secondary veins 11 to 17 pairs; inflorescence compact, generally up to than 10(–15) mm long	﻿***Brieya***
–	Secondary veins (15–)22 to 66 pairs; inflorescence compact to lax, but always longer than 16 mm	﻿***Piptostigma***
21	Receptacle cylindrical; anther connective reduced to a tuft of hairs	﻿***Mischogyne***
–	Receptacle convex to flat; connective well developed, discoid to apiculate	**22**
22	Tertiary venation percurrent	**23**
–	Tertiary venation reticulate	**25**
23	Leaf apex obtuse, rounded or emarginate (in Cameroonian species); fruits (pseudo)syncarpous	﻿***Annona***
–	Leaf apex acute, acuminate or caudate; fruits apocarpous	**24**
24	Outer petals much longer than inner petals; inner petals forming a dome over the receptacle; anthers septate; seed 1 per monocarp	﻿***Neostenanthera***
–	Petals sub equal or outer slightly longer; inner petals not forming a dome over the receptacle; anthers not septate; seeds > 1 per monocarp	﻿***Polyceratocarpus* (pro parte)**
25	Sepals reduplicate-valvate, buds ridged	﻿***Uvariastrum***
–	Sepals not reduplicate-valvate, buds not ridged	**26**
26	Petals homogenously red to pink, all reflexed at maturity; anthers bright yellow *in vivo* at maturity; carpel 1; monocarp 1	﻿***Sirdavidia***
–	Petals green, yellow, cream, not reflexed or only curved outward; anthers not bright yellow; carpels > 1; monocarps generally more than one	**27**
28	Anthers septate; monocarps dehiscent	﻿***Xylopia***
–	Anthers not septate; monocarps not dehiscent	**29**
29	Individuals androdioecious or dioecious with separate male, female or bisexual flowers	**30**
–	Individuals with bisexual flowers	**31**
30	Flowering peduncles present, > 50 mm long; stamens more than 30; fruits (pseudo)syncarpous	﻿***Anonidium***
–	Flowering peduncle absent; stamens less than 30; fruits apocarpous	﻿***Greenwayodendron***
31	Flowering pedicels > 15 mm; sepals free; outer petals up to five times longer than inner petals	﻿***Cleistopholis***
–	Flowering pedicels < 15 mm; sepals basally fused; petals subequal	**32**
32	Petioles 2–5 mm long, 1–2 mm wide; petals 3(4), less than 10 mm long, basally fused	﻿***Dennettia***
–	Petioles > 4 mm long, 3–9 mm wide; petals 6, 10 mm or longer, free	﻿***Uvariodendron***

##### ﻿Synoptic key

Genera in parentheses means some but not all species have the indicated trait.

Liana or scrambling to scandent shrub: ﻿*Afroguatteria*; ﻿*Artabotrys*; ﻿*Uvaria*; (﻿*Monodora*); ﻿*Monanthotaxis*; (﻿*Neostenanthera*); ﻿*Sphaerocoryne*; ﻿*Toussaintia*; ﻿*Letestudoxa*; (﻿*Xylopia*).

Tree: ﻿*Annickia*; ﻿*Annona*; ﻿*Anonidium*; ﻿*Brieya*; ﻿*Cleistopholis*; ﻿*Duguetia*; ﻿*Greenwayodendron*; ﻿*Hexalobus*; ﻿*Isolona*; ﻿*Meiocarpidium*; ﻿*Mischogyne*; ﻿*Monodora*; ﻿*Neostenanthera*; ﻿*Piptostigma*; ﻿*Polyceratocarpus*; ﻿*Uvariastrum*; ﻿*Uvariodendron*; ﻿*Uvariopsis*; ﻿*Xylopia*.

Slash of the bark yellow: ﻿*Annickia*.

Stilt roots or buttresses present: (﻿*Xylopia*).

Indumentum of stellate hairs: ﻿*Annickia*, ﻿*Duguetia*; ﻿*Uvaria*.

Indumentum of lepidote hairs: ﻿*Meiocarpidium*.

Hook-like structures on branches: ﻿*Artabotrys*.

Leaves discolorous, light green to whitish below: ﻿*Afroguatteria*; ﻿*Cleistopholis*, ﻿*Monanthotaxis*; ﻿*Piptostigma*, ﻿*Sphaerocoryne*; (﻿*Uvaria*), (﻿*Xylopia*).

Trunk whitish overall: ﻿*Cleistopholis*; ﻿*Greenwayodendron*.

Leaves with many parallel secondary veins (> 25): (﻿*Piptostigma*), (﻿*Uvaria*), (﻿*Uvariodendron*).

Midrib clearly raised above: ﻿*Isolona*; ﻿*Monodora*; (﻿*Polyceratocarpus*).

Sepals 2; petals 4: ﻿*Uvariopsis*; (﻿*Monanthotaxis*).

Sepals reduplicate-valvate (margins folded in bud): ﻿*Uvariastrum*, ﻿*Toussaintia*.

Petals fused into a single whorl with a distinct tube: ﻿*Hexalobus*; ﻿*Isolona*.

Petals 3: ﻿*Annickia*; ﻿*Dennettia*; (﻿*Monanthotaxis*).

Petals 9 to 10 inserted in 2 to 3 whorls: ﻿*Toussaintia*.

Petals plicate in bud, transversely folded when open: ﻿*Hexalobus*.

Inner petals much longer than outer: ﻿*Brieya*; ﻿*Piptostigma*.

Anthers septate: ﻿*Neostenanthera*; ﻿*Xylopia*.

Staminodes present: (﻿*Monanthotaxis*); ﻿*Xylopia*.

Androdioecious, dioecious or monoecious: ﻿*Anonidium*; ﻿*Greenwayodendron*; (﻿*Monanthotaxis*); ﻿*Polyceratocarpus*; ﻿*Uvariopsis*.

Pseudosyncarpous fruits (individual monocarps visible): ﻿*Annona*, ﻿*Anonidium*, ﻿*Duguetia*, ﻿*Letestudoxa*.

Fruits syncarpous (individuals monocarps not visible) with numerous unordered seeds: ﻿*Isolona*; ﻿*Monodora*.

Monocarps moniliform: ﻿*Monanthotaxis*; (﻿*Xylopia*).

Monocarps dehiscent: ﻿*Xylopia*.

Seeds arillate: (﻿*Duguetia*); (﻿*Xylopia*).

Seeds with a sarcotesta: (﻿*Xylopia*).

### ﻿﻿ Afroguatteria

Taxon classificationPlantaeMagnolialesAnnonaceae

Boutique, Bull. Jard. Bot. État Bruxelles 21: 104, 1951

5697CEB7-3642-56C8-9C9C-541EA066D8BB

#### Type species.

﻿﻿*Afroguatteriabequaertii* (De Wild.) Boutique.

#### Description.

Same as species.

A genus of lianas with three species from Central Africa, in the Democratic Republic of the Congo, Cameroon and Angola (Cabinda); one species in Cameroon, endemic.

This genus was phylogenetically validated (Guo et al. 2017b).

#### Taxonomy.

no revision has yet been published, but see Boutique (1951b) and Paiva (1966).

### ﻿﻿﻿ Afroguatteria discostigma

Taxon classificationPlantaeMagnolialesAnnonaceae

(Diels) X.Guo & R.M.K.Saunders, Taxon 66 (1): 13, 2017

5453D833-2321-5BF2-9E37-3F02D26B3B74

Fig. 4 ; Map 1A

≡ ﻿﻿Cleistopholisdiscostigma Diels, Bot. Jahrb. Syst. 39: 474, 1907; ﻿Oxymitradiscostigma (Diels) Ghesq. ex Pellegr., Bull. Soc. Bot. France, 66, 1949; ﻿Richelladiscostigma (Diels) R.E.Fr., in Engler & Prantl Nat. Pflanzenfam., ed. 2, 17a (2): 139, 1959; ﻿﻿Friesodielsiadiscostigma (Diels) Steenis, Blumea 12: 359, 1964.

#### Type.

Cameroon. South Region; Bipindi, *Zenker G.A. 2980*, Apr 1904: holotype: B[B10 0153055]; isotypes: BM[BM001125042]; BR [BR000008800398]; [BR000008800398]; G[G00308361]; GOET[GOET005676]; HBG[HBG-502538]; K[K000198949]; L[L.1754813]; M[M-0107910]; P[P00363341]; S[S03-2239]; WAG[WAG0053550].

#### Description.

Liana, height unknown, d.b.h. unknown. Indumentum of simple hairs; old leafless branches glabrous, young foliate branches pubescent with short sericeous hairs. Leaves: petiole 3–4 mm long, 1–2 mm in diameter, sparsely pubescent to glabrous, grooved, blade inserted on the side of the petiole, 7.5–10 cm long, 4–5 cm wide, elliptic, apex acuminate, acumen ca.1 cm long, base obtuse, subcoriaceous, below sparsely pubescent to glabrous when young, glabrous when old, above glabrous when young and old, **discolorous**, **whitish below (both when fresh and dry)**; midrib flat or sunken, above glabrous when young and old, below sparsely pubescent when young, glabrous when old; secondary veins 8 to 13 pairs, glabrous above; tertiary venation reticulate. Individuals bisexual; inflorescences cauliflorous or ramiflorous on young foliate branches, leaf-opposed or extra-axillary. Flowers with 9 perianth parts in 3 whorls, 1 per inflorescence; **pedicel 18–22 mm long**, ca. 1 mm in diameter, pubescent; in fruit 20–40 mm long, 2–3 mm in diameter, pubescent; bracts not seen; sepals 3, valvate, free, 1–2 mm long, 1–2 mm wide, triangular, apex acute, base truncate, pubescent outside, glabrous inside, margins flat; petals free, subequal; outer petals 3, valvate, 5–6 mm long, 3–4 mm wide, ovate, apex acute, base narrowed, margins flat, pubescent outside, pubescent inside; inner petals 3, valvate, 3–3.5 mm long, 2–3 mm wide, elliptic, apex obtuse, base truncate, margins flat, pubescent outside, pubescent inside; number of stamens not counted, number of rows not seen, 2–3 mm long, oblong; anthers not septate; connective discoid, pubescence not seen; staminodes absent; carpels not seen but free. Monocarps stipitate, stipes 5–10 mm long, 1–2 mm in diameter; monocarps 3 to 7, 10–15 mm long, 5–8 mm in diameter, ellipsoid, apex apiculate, pubescent, smooth, not ribbed, color unknown; **seed 1**, 13–15 mm long, 5–7 mm in diameter, ellipsoid; aril absent.

#### Distribution.

endemic to Cameroon; known from the South Region.

#### Habitat.

A rare species, in primary lowland rain forests. Altitude 100–200(?) m a.s.l.

#### Local and common names known in Cameroon.

None recorded.

#### IUCN conservation status.

NE (Not Evaluated), but probably CR.

#### Uses in Cameroon.

None recorded.

#### Notes.

﻿﻿*Afroguatteriadiscostigma* is only known from four collections by Zenker, all collected close to the type locality in Bipindi (South Region). It remains incompletely known and measurements here are incomplete. The species can be distinguished by its almost glabrous vegetative parts (young foliate branches and underside of young leaves can be pubescent with short hairs), its small leaves that are glaucous below (both when fresh and dry) and branches drying black. The flowers are borne on terminal pedicels that appear leaf-opposed or extra-axillary, but can also be cauliflorous (*Zenker 3023*), the carpels have a single ovule and thus monocarps are single-seeded like in ﻿﻿*Afroguatteriabequaertii* (De Wild.) Boutique) (Boutique 1951a). The altitude range given here is the one around Bipindi, but could be higher given that the mountain range Ngovayang (up to 1000 m) is very close.

A recent molecular phylogenetic study showed that this species (under the name ﻿﻿*Friesodielsiadiscostigma*) clustered in the genus ﻿*Afroguatteria* being sister to the Congolese species ﻿*A.bequaertii* (Guo et al. 2017b). It is thus quite different genetically from the the African species of ﻿*Friesodielsia* (now ﻿*Monanthotaxis*) in which it was placed before based on morphology (van Steenis 1964).

**Figure 4.**
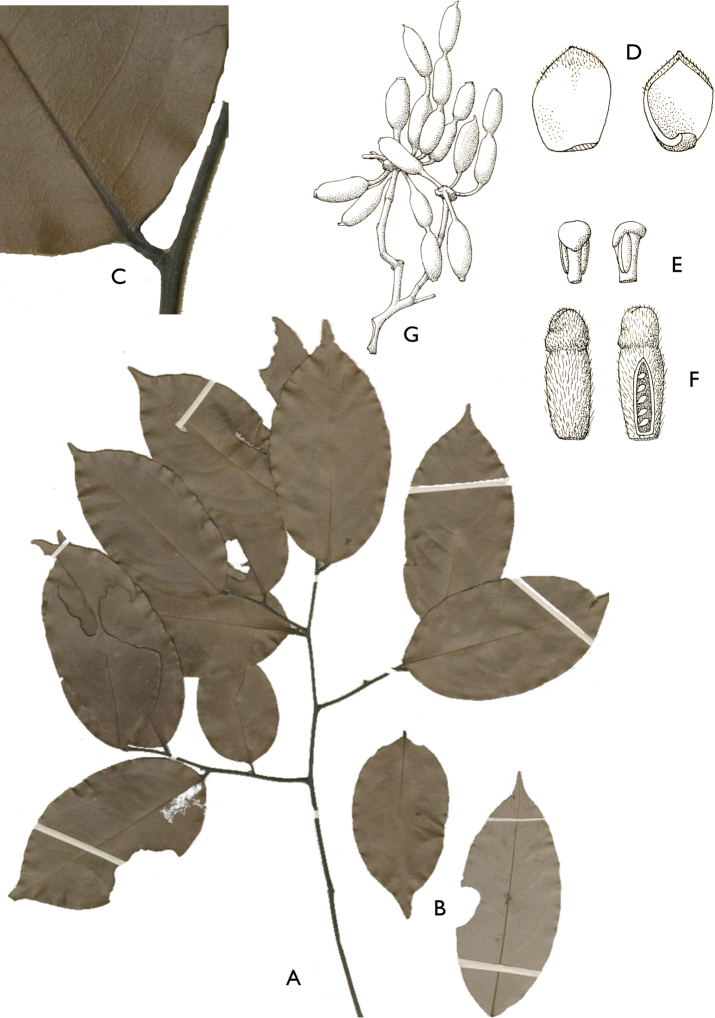
*Afroguatteriadiscostigma***A** branch **B** upper and lower side of leaf, notice glaucous lower side and network like secondary veins **C** detail of upper lower side of leaf and petiole **D** inner (left) and outer petals (right), inner view **E** stamens front and side views **F** carpels side view and view of ovules **G** infructescence with moniliform monocarps **A–C** from *Zenker 2980* [S03-2239] reproduced from Swedish Museum of Natural History Department of Botany (S) https://plants.jstor.org/**D–G** from *Zenker 3023*. Drawings by Hélène Lamourdedieu, Publications Scientifiques du Muséum national d’Histoire naturelle, Paris.

**Map 1.**
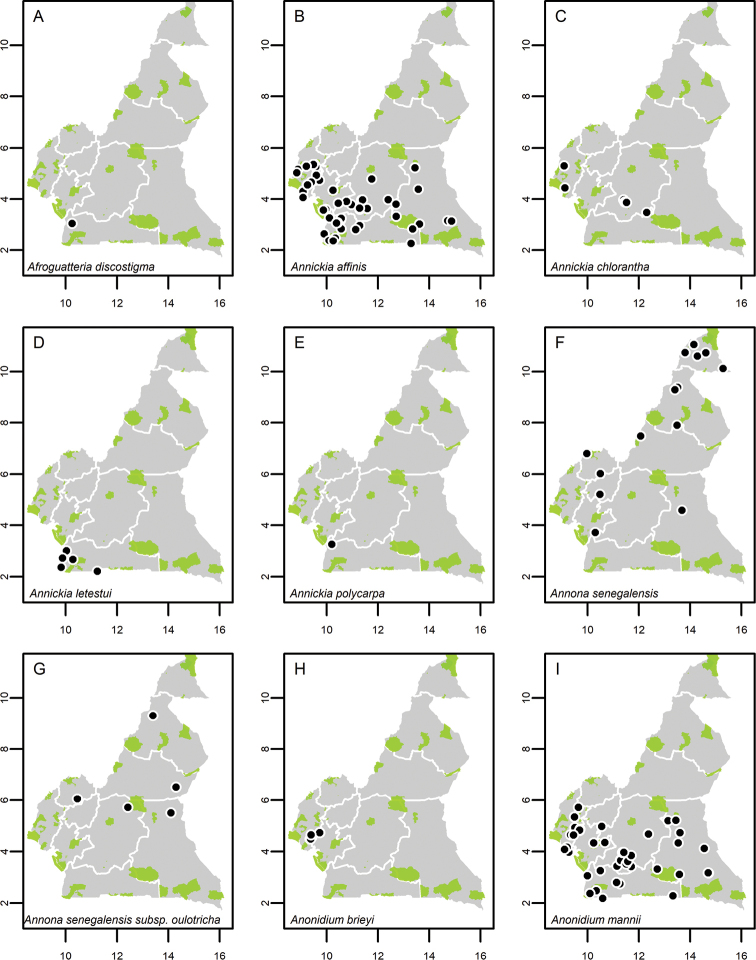
**A***Afroguatteriadiscostigma***B***Annickiaaffinis***C***Annickiachlorantha***D***Annickialetestui***E***Annickiapolycarpa***F***Annonasenegalensis***G**Annonasenegalensissubsp.oulotricha**H***Anonidiumbrieyi***I***Anonidiummannii*. White borders represent region limits in Cameroon; green patches represent protected areas (see methods and Suppl. material 1: Fig. S1).

#### Specimens examined.

**South Region**: Bipindi, 3.05°N, 10.25°E, *01 January 1905*, *Zenker G.A.* 2102a (BM,BR,E,G,K,P); Bipindi, 3.05°N, 10.25°E, *01 January 1904*, *Zenker G.A.* 3023 (BM,G,K,L,P,WAG); Bipindi, 3.08°N, 10.41°E, *01 March 1914*, *Zenker G.A.* 576 (MA).

### ﻿﻿ Annickia

Taxon classificationPlantaeMagnolialesAnnonaceae

Setten & Maas, Taxon 39 (4): 676, 1990

B0D75B82-9733-5BFC-8D09-3D4316698713

= Enantia Oliv. *nom. illeg.*; J. Linn. Soc., Bot. 9: 174–175, 1867.

#### Type species.

﻿﻿*Enantiachlorantha* Oliv. (≡ ﻿﻿﻿*Annickiachlorantha* (Oliv.) Setten & Maas).

#### Description.

Trees, up to 30 m tall, d.b.h. up to 50 cm; stilt roots or buttresses absent, slash yellow. Indumentum of simple, stellate and/or fasciculate hairs. Leaves: petiole 2–9 mm long, 1–2 mm in diameter; blade 3.5–29.5 cm long, 1.5–10.5 cm wide, elliptic to obovate, apex acuminate to acute, base narrowly cuneate to shortly attenuate to rounded; midrib sunken or flat; secondary veins 8 to 13 pairs; tertiary venation reticulate. Individuals bisexual; inflorescences ramiflorous on old or young foliate branches, leaf opposed or extra axillary. Flowers with 6 perianth parts in 2 whorls, 1 per inflorescence; pedicel 4–19 mm long; in fruit 10–27 mm long; bracts 1 to 2, basal and one upper towards the middle or lower half of pedicel, 2–8 mm long; sepals 3, valvate, free, 5–22 mm long, triangular, apex acute, base truncate; petals free; outer petals absent; inner petals 3, valvate, 12–34 mm long, 5–19 mm wide, ovate, margins inversely Y-shaped ridged, apex acute, base broad and concave; stamens 60 to 200, in 5 to 6 rows, 2–4 mm long, linear; connective tongue shaped or flattened, glabrous; staminodes absent; carpels free, 20 to 70, ovary 2–4 mm long, stigma lobed or cylindrical, pubescent. Monocarps stipitate, stipes 6–59 mm long, 5 to 55 monocarps, 18–35 mm long, 4–14 mm in diameter, ellipsoid to obovoid, apex sometimes mucronate, smooth, glossy; seed 1, 20–30 mm long, ca. 10 mm in diameter, ellipsoid; aril absent.

A genus of eight species mostly distributed across west and central Africa, with one endemic species in Tanzania; four species occur in Cameroon, none endemic.

The genus is easily identifiable when sterile by its yellow slash, and when fertile, by its leaf opposed or extra-axillary (terminal) flowers with 3 sepals and 3 petals, and stipitate monocarps with a single seed.

#### Taxonomy.

Versteegh and Sosef (2007).

### ﻿Key to the species of ﻿*Annickia* in Cameroon:

**Table:** 

1	Upper side of midrib pilose	**2**
–	Upper side of midrib glabrous, or pubescent just at the basal part, never pilose	**3**
2	Lower leaf surface with simple, bifid and trifid hairs; petals pubescent at base inside; monocarps with stipes < 20 mm	﻿***A.chlorantha***
–	Lower leaf surface at least with some stellate or fasciculate hairs; petals glabrous at base inside; monocarps with stipes > 20 mm	﻿***A.polycarpa***
3	Pubescence on lower leaf surface simple, bifid, trifid or stellate, pointing in all directions	**4**
–	Pubescence on lower leaf surface simple or bifid, all hairs pointing towards leaf apex	﻿***A.affinis***
4	Petiole and young shoots tomentose	﻿﻿***A.letestui***
–	Petiole and young shoots sparsely pubescent to pubescent	﻿***A.chlorantha***

### ﻿﻿﻿ Annickia affinis

Taxon classificationPlantaeMagnolialesAnnonaceae

(Exell) Versteegh & Sosef, Syst. & Geogr. Pl. 77(1): 95, 2007

**Figure 5.**
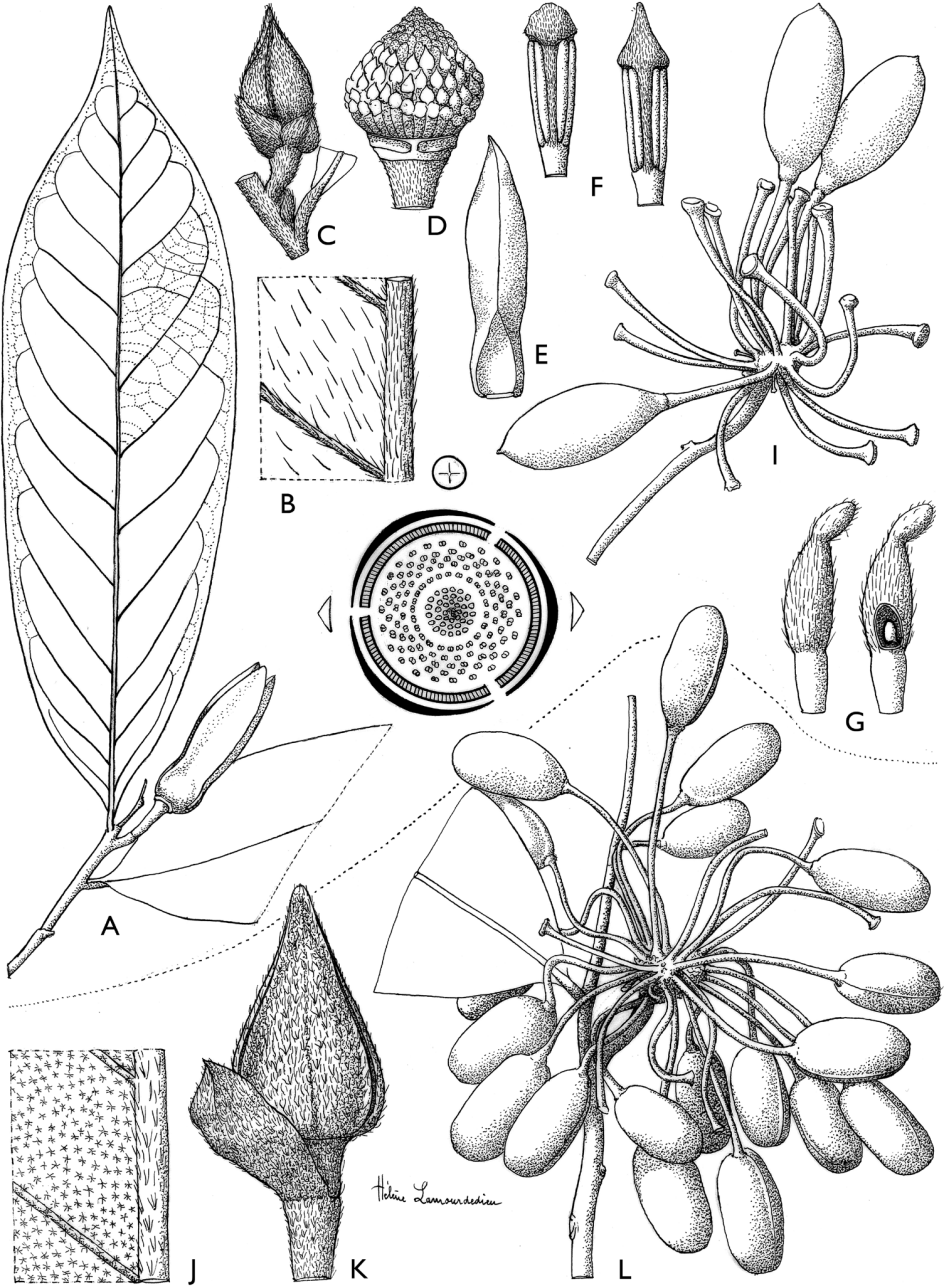
*Annickiaaffinis***A** flowering branch **B** detail of lower side of leaf blade **C** flower bud **D** detail of receptacle with petals removed **E** inner petals, inner view **F** stamens, note different shape of connective (discoid to apiculate) **G** carpel, whole and showing the single basal ovule **H** floral diagram **I** fruit. *Annickiapolycarpa*: **J** details of lower side of leaf blade **K** flower bud **L** fruiting branch **A–C** from *Le Testu 1783***D–I** from *Letouzey 5412***J–L** from *Chevalier 1611*. Drawings by Hélène Lamourdedieu, Publications Scientifiques du Muséum national d’Histoire naturelle, Paris; modified from Le Thomas (1969b; pl. 57, p. 311, pro parte).

8E46DCA6-0ABB-5BF2-BDF4-8A9044C97D1E

Figs 5 , 7 ; Map 1B

≡ Enantia affinis Exell, J. Bot. 64, Suppl.: 9, 1926.
= ﻿﻿Enantiachlorantha(Oliv.)Setten & Maasvar.soyauxii Engl. & Diels, Monogr. Afrik. Pflanzen.-Fam. 6: 70 1901. Type. Gabon. Estuaire, Munda, Sibange Farm, Soyaux H. 125, 21 Sep 1880: lectotype, designated by Versteegh and Sosef (2007), p. 95: B *n.v.*; isolectotypes: K[K001208605]; P[P00267979].

#### Type.

Angola. Cabinda, Munze, ring at Buco Zau, *Gossweiler J. 6675*, 11 Sep 1916: holotype K[not seen]: isotypes: BM[BM000547034]; COI[COI00004913]; LISC[LISC000073, LISC000072, LISC000075, LISC000074].

#### Description.

Tree, 3–30 m tall, d.b.h. 3–50 cm; stilt roots or buttresses absent, **slash yellow**. Indumentum of simple, bifid and fasciculate hairs; old leafless branches glabrous, young foliate branches sparsely pubescent. Leaves: petiole 2–8 mm long, 1–2 mm in diameter, sparsely pubescent, grooved, blade inserted on the side of the petiole; blade 3.5–26 cm long, 1.5–9.5 cm wide, elliptic to obovate, apex acuminate to acute, acumen 1 cm long, base narrowly cuneate to shortly attenuate, coriaceous to subcoriaceous, below pubescent when young and old **with simple or bifid hairs pointing towards the leaf apex**, above sparsely pubescent when young and old, concolorous; midrib sunken or flat, **above sparsely pubescent to glabrous when young and old**, below pubescent when young and old; secondary veins 8 to 13 pairs, sparsely pubescent below; tertiary venation reticulate. Individuals bisexual; inflorescences ramiflorous on old or young foliate branches, leaf opposed or extra axillary. Flowers with 6 perianth parts in 2 whorls, 1 per inflorescence; pedicel 7–14 mm long, 1–2 mm in diameter, pubescent; in fruit 27 mm long, 2–3 mm in diameter, pubescent; bracts 1–2, basal and one upper towards the middle of pedicel, ca. 4 mm long, ca. 2 mm wide; sepals 3, valvate, free, 7 mm long, ca. 4 mm wide, triangular, apex acute, base truncate, green, pubescent outside, glabrous inside, margins flat; petals free; outer petals absent; inner petals 3, valvate, 15–33 mm long, 5–15 mm wide, ovate to inversely Y–shaped ridged, apex acute, base broad and concave, greenish yellow, margins flat, pubescent outside, glabrous inside; stamens 110 to 175, in 5 to 6 rows, 2–4 mm long, linear; **connective tongue shaped**, glabrous, yellow; staminodes absent; carpels free, 35 to 70, ovary 3–4 mm long, stigma lobed, pubescent. Monocarps stipitate, stipes 10–40 mm long, 1–2 mm in diameter; monocarps 3 to 34, 20–35 mm long, 9–14 mm in diameter, ellipsoid to obovoid, apex sometimes mucronate, sparsely pubescent, smooth, glossy, black when ripe; seed 1, ca. 30 mm long, ca. 10 mm in diameter, ellipsoid; aril absent.

#### Distribution.

From Nigeria (one collection) to the Republic of Congo and the extreme west of the Democratic Republic of Congo; in Cameroon known from the East, South, Littoral, Center and South-West regions.

#### Habitat.

A very common species; in lowland rain forests in primary and secondary habitats. Altitude 50–650 m a.s.l.

#### Local and common names known in Cameroon.

Bololo, Bonuke, Bunuku bolobo (dial. Duala); Bululu, Mfo, Pobalo, Ufol, Moabé (dials. Ewondo, Bulu); M’Fo, Mofo, Mpuley (dial. Mab Kwasio, *Foury 113*, *Service Forestier du Cameroun 84*, *Bates 1959*); N’jie (Dials. Duala, Punu); Ogowa (Punguegaloa, *De Wilde 8492*); Moabi jaune (French); évué (dial. Bibaya, Baka).

#### IUCN conservation status.

Least Concern (LC) (Cosiaux et al. 2019a).

#### Uses in Cameroon.

***medicine***: bark as a malaria prophylaxis; ***construction***: house building, furniture; ***dyes and tannins***: as a yellow dye (Versteegh and Sosef 2007).

#### Notes.

﻿﻿*Annickiaaffinis* is distinguished by having overall glabrous branches and petioles and the lower side of the leaf blades which is sparsely pubescent with simple or bifid hairs pointing in the same direction. ﻿﻿*Annickiaaffinis* is morphologically close to ﻿*A.chlorantha* from which it is distinguished by having a glabrous upper midrib surface (versus pilose in ﻿*A.chlorantha*). In addition, ﻿*A.chlorantha* has few simple hairs pointing in different directions combined with smaller bifid or trifid hairs.

﻿﻿*Annickiaaffinis* is the most common species of ﻿*Annickia* and is generally found as a young plant in secondary forest, or as an adult in older secondary or primary forests. For a long time (and still now) ﻿﻿*Annickiaaffinis* was confused with ﻿*A.chlorantha* (or even ﻿﻿*Enantiachlorantha*), but the latter name is attributed to a different and rarer species (Versteegh and Sosef 2007). Thus, most literature refers to the old name *A.* (﻿*Enantia*) *chlorantha* when referring to ﻿*A.affinis* (the common and widespread species). Previous reports of ﻿*A.chlorantha* outside Nigeria and Cameroon (e.g. Gabon) refer to ﻿*A.affinis*.

#### Selected specimens examined.

**Central Region**: near Ebolbom village 3 km est of Ngoumou 2 km north west of Otélé, 3.59°N, 11.28°E, *02 May 2013*, *Couvreur T.L.P.* 426 (WAG,YA); Ottotomo Forest Reserve 3 km after reserve base near small loggers road, 3.66°N, 11.28°E, *02 May 2013*, *Couvreur T.L.P.* 437 (WAG,YA); Mefou Proposed National Park, 3.62°N, 11.57°E, *15 March 2004*, *Etuge M.* 5139 (K,YA); Mbam Minkom, 3.96°N, 11.36°E, *19 September 2013*, *Kamdem N.* 143 (YA); Nguila 1, 4.77°N, 11.75°E, *30 April 2017*, *Kamdem N.* 521 (YA); Colline entre Tcherikoy et Sokelle II (30 km NW Eséka), 3.78°N, 10.96°E, *14 December 1973*, *Letouzey R.* 12361 (P,YA). **East Region**: 77 km south of Yokadouma 30 km after Ngato 15 km after river ALPICAM ‘base de vie’ then 40 km on forestry road starting 4 km before Maséa village, 3.15°N, 14.72°E, *05 March 2019*, *Couvreur T.L.P.* 1203 (MPU,WAG,YA); Deng Deng, 5.21°N, 13.44°E, *19 April 2016*, *Kamdem N.* 422 (YA); 16 km E de Dimako, 4.38°N, 13.57°E, *15 December 1965*, *Leeuwenberg A.J.M.* 7355 (BR,K,MO,P,PHA,WAG,YA); 15 km E of Dimako, 4.38°N, 13.57°E, *08 February 1966*, *Leeuwenberg A.J.M.* 7787 (BR,C,K,MO,P,WAG,YA); Route Mintom I (70 km E de Djoum)-Alati (100 km SE de Djoum)-PK 63, 2.83°N, 13.35°E, *01 January 1973*, *Letouzey R.* 11751 (P,YA). **Littoral Region**: Ebo Wildlife Reserve Djuma permanent camp On Djuma-Djuma trail, 4.33°N, 10.24°E, *14 February 2014*, *Couvreur T.L.P.* 621 (WAG,YA); Mambe Massif above Boga village 100 km along road from Yaoundé to Ed 3.90°N, 10.77°E, *20 June 2014*, *Couvreur T.L.P.* 657 (WAG,YA). **South Region**: Ebolowa, 2.96°N, 11.28°E, *01 January 1925*, *Bates G.L.* 1959 (BM,BR,MO); on road Lolodorf-Bipindi ca half way near Mbiguiligui village (Mbikiliki), 3.16°N, 10.53°E, *26 February 2018*, *Couvreur T.L.P.* 1153 (P,WAG,YA); 22 km east from Lélé village, 3.26°N, 10.10°E, *07 September 2013*, *Couvreur T.L.P.* 469 (WAG,YA); ca 15 km east from Lélé village, 2.26°N, 13.29°E, *09 September 2013*, *Couvreur T.L.P.* 492 (WAG,YA); Campo Ma’an National Park 11 km on trail from Ebinanemeyong village on road 7 km from Nyabessan to Campo town, 2.47°N, 10.33°E, *11 February 2015*, *Couvreur T.L.P.* 671 (WAG,YA); A 6 km à l’ouest de Masea (village situé à 50 km au SSW de Yokadouma), 3.14°N, 14.86°E, *05 July 1963*, *Letouzey R.* 5412 (P,YA); Campo-Ma’an area road Nko-elon-Mvini Akok Beryat rock, 2.36°N, 10.25°E, *30 June 2001*, *van Andel T.R.* 3784 (KRIBI,WAG,YA); Bipindi, 3.08°N, 10.42°E, *01 January 1909*, *Zenker G.A.* 3839 (BM,BR,K,MO,P). **South-West Region**: Ekundu Kundu, 5.15°N, 8.883°E, *30 April 1996*, *Cheek M.* 8297 (K,WAG,YA); Mungo river forest reserve North of Kumba-Tombel road entered ca 05 km West of Mungo bridge, 4.73°N, 9.55°E, *24 October 1998*, *Cheek M.* 9354 (YA); Foot of Nyale Rock, 4.98°N, 9.616°E, *17 November 1998*, *Cheek M.* 9654 (K,YA); on trail through palm oil plantation 3 km before lava flow and Seme Beach hotel when coming from Limbe, 4.05°N, 9.076°E, *18 October 2013*, *Couvreur T.L.P.* 519 (WAG,YA); Kupe village to Loum State Forest, 4.73°N, 9.716°E, *30 May 1996*, *Etuge M.* 2049 (K,WAG,YA); Nyale forest and rock, 5°N, 9.633°E, *15 February 1998*, *Etuge M.* 4235 (K,YA); Edensueh forest, 5.25°N, 9.576°E, *30 November 2000*, *Etuge M.* 4850 (K); Kumba-Mbonge road 500 m W of Meme River bridge between Bole and Mabonji, 4.55°N, 9.25°E, *07 July 1986*, *Thomas D.W.* 6327 (MO); Baro village, 5.27°N, 9.21°E, *03 March 1988*, *Thomas D.W.* 7494 (K,MO,P,WAG).

### ﻿﻿﻿﻿ Annickia chlorantha

Taxon classificationPlantaeMagnolialesAnnonaceae

(Oliv.) Setten & Maas, Taxon 39(4): 676, 1990

EFAA88CC-5722-553C-9C95-00F2B90947C1

Fig. 7 ; Map 1C

≡ Enantia chlorantha Oliv., J. Linn. Soc., Bot. 9: 175, 1867.

#### Type.

Nigeria. Cross River State; Old Calabar, *Thomson W.C 130*, Dec 1863: holotype: K[K000380204].

#### Description.

Tree, 9–25 m tall, d.b.h. to 5 cm; stilt roots or buttresses absent, **slash yellow**. Indumentum of simple, bifid trifid, and fasciculate hairs; old leafless branches glabrous, **young foliate branches sparsely pubescent to pubescent.** Leaves: petiole 2–9 mm long, ca. 2 mm in diameter, pubescent, slightly grooved, blade inserted on top of the petiole; blade 7–28 cm long, 2–9.5 cm wide, elliptic to obovate, apex acuminate, acumen ca. 1 cm long, base narrowly cuneate to shortly attenuate, coriaceous to papyraceous, below densely pubescent when young, sparsely pubescent when old, **hairs simple, bifid or trifid hairs pointing in all directions**, above glabrous when young and old, concolorous; midrib sunken or flat, **above densely pubescent (pilose) to pubescent at least towards base when young**, densely pubescent to pubescent at least towards base when old (rarely glabrous), below pubescent when young and old; secondary veins 8 to 12 pairs, glabrous below; tertiary venation intermediate. Individuals bisexual; inflorescences ramiflorous on young foliate branches, leaf opposed or extra axillary. Flowers bisexual with 6 perianth parts in 2 whorls, 1 per inflorescence; pedicel 5–11 mm long, ca. 1 mm in diameter, densely pubescent; in fruit 10–15 mm long, ca. 2 mm in diameter, densely pubescent; bracts 1–2, one basal and one upper towards the middle of pedicel, basal bract 4–8 mm long; sepals 3, valvate, free, 8–12 mm long, 4–6 mm wide, triangular, apex acute, base truncate, green, pubescent outside, glabrous inside, margins flat; petals free; outer petals absent; inner petals 3, valvate, 15–29 mm long, 6–14 mm wide, elliptic to inversely Y–shaped ridged, apex acute, base broad and concave, greenish yellow, margins flat, pubescent outside, pubescent in a small triangle at the base inside; stamens 145 to 160, in 5 to 6 rows, 2 mm long, linear; **connective flattened**, glabrous, yellow; staminodes absent; carpels free, 20 to 35, ovary ca. 2 mm long, **stigma cylindrical**, pubescent. Monocarps stipitate, stipes 6–20 mm long, ca. 1 mm in diameter; monocarps 3 to 27, 10–16 mm long, 4–9 mm in diameter, ellipsoid to obovoid, apex mucronate, sparsely pubescent, smooth, glossy, green turning red to black when ripe; seed 1, ca. 20 mm long, ca. 10 mm in diameter, ellipsoid; aril absent.

#### Distribution.

Known from Nigeria (one collection); in Cameroon known from the East, South, Center and South-West regions.

#### Habitat.

Locally common when present but rare overall, in lowland and premontane rain forests, mainly in primary habitats. Altitude 150–850 m a.s.l.

#### Local and common names known in Cameroon.

Otou han (dial. bulu, *Bos 6894*), Otoungué (dial. Ewondo, *Chevalier 33132*).

#### IUCN conservation status.

Least Concern (LC) (Cosiaux et al. 2019b)

#### Uses in Cameroon.

***medicine***: bark as an antisepctic, against fever, malaria prophylaxis; ***construction***: house building, furniture; ***dyes and tannins***: as a yellow dye.

#### Notes.

﻿﻿﻿*Annickiachlorantha* is distinguished by having sparsely pubescent branches and petioles and the lower side of the leaf blades pubescent with simple, bifid, trifid or fasciculate hairs pointing in all directions. The midrib is generally densely pubescent (pilose) at least towards the base, but some specimens are reported to be glabrous (Versteegh and Sosef 2007). See notes under ﻿*A.affinis* for confusions surrounding this name. ﻿﻿﻿*Annickiachlorantha* also closely resembles ﻿﻿*A.letestui*, but differs by its sparsely pubescent young branches versus tomentose in ﻿﻿*A.letestui*.

#### Specimens examined.

**Central Region**: Nkolbisson shrubby low forest on summit of Mt Akockdoué Yaoundé, 3.88°N, 11.45°E, *23 May 1970*, *Bos J.J.* 6894 (BR,MO,P,WAG); Mont Mbam Minkon on trail 3 km from Nkol Nyada village, 3.96°N, 11.40°E, *21 March 2013*, *Couvreur T.L.P.* 414 (WAG,YA); Colline Akok Ndoue près Nkolbisson 5 km WSW Yaoundé, 3.88°N, 11.45°E, *23 May 1970*, *Farron C.* 7335 (P,YA); Yaoundé, 3.86°N, 11.51°E, *Feburary 1895*, *Zenker G.A.* 726 (P). **Littoral Region**: Forêt de Ye Youme, 3.48°N, 12.3°E, *01 June 1917*, *Chevalier A.J.B.* 33132 (P). **South-West Region**: Bambuko FR, 4.43°N, 9.116°E, *16 September 1951*, *Olorunfemi J.* 30760 (K); Korup National Park, 5.28°N, 9.083°E, *03 April 1988*, *Thomas D.W.* 7555 (MO).

### ﻿﻿﻿﻿ Annickia letestui

Taxon classificationPlantaeMagnolialesAnnonaceae

(Le Thomas) Setten & Maas, Taxon 39(4): 676, 1990

E71C3949-EE7C-58C4-BCF3-EA1AAA94FE73

Fig. 6 ; Map 1D

≡ Enantia letestui Le Thomas, Adansonia sér. 2, 2: 306, 1962.

#### Type.

Gabon. Ogooué-Lolo, Ikembélé, *Le Testu G.M.P.C. 8432*, Oct 1930: lectotype, here designated: P[P00267987]; isolectotypes: BM[BM000547036]; BR[BR0000006418700]; P[P00362651, P02005895, P02005896].

#### Description.

Tree, 2–8 m tall, d.b.h. unknown; stilt roots or buttresses absent, slash yellow. Indumentum of simple, bifid, fasciculate or stellate hairs; old leafless branches glabrous, **young foliate branches tomentose**. Leaves: petiole 3–8 mm long, 1–2 mm in diameter, **tomentose to sparsely pubescent**, cylindrical, blade inserted on top of the petiole; blade 10–29.5 cm long, 3.5–10.5 cm wide, elliptic to obovate, apex acuminate to mucronate, acumen 1–2 cm long, base cuneate to rounded to acuminate, subcoriaceous, above glabrous when young and old, below pubescent when young and old, **hairs simple, bifid and stellate pointing in all directions**, concolorous; midrib sunken or flat, above glabrous when young and old, below pubescent when young and old; secondary veins 9 to 13 pairs, glabrous below; tertiary venation intermediate. Individuals bisexual; inflorescences ramiflorous on young foliate branches, leaf opposed or extra axillary. Flowers with 6 perianth parts in 2 whorls, 1 per inflorescence; pedicel 4–14 mm long, ca. 2 mm in diameter, sparsely pubescent; in fruit ca. 10 mm long, ca. 2 mm in diameter, pubescent; bracts 2, one basal and one upper towards the lower half of pedicel, basal bract 2–4 mm long, 2–3 mm wide; sepals 3, valvate, free, 5–9 mm long, 3–4 mm wide, triangular, apex acute, base truncate, pubescent outside, glabrous inside, margins flat; petals free; outer petals absent; inner petals 3, valvate, 12–26 mm long, 7–12 mm wide, ovate to inversely Y–shaped ridged, apex acute, base broad and concave, yellow–green, margins flat, pubescent outside, pubescent towards margins inside; stamens 60 to 125, in 5 to 6 rows, ca. 2 mm long, linear; connective flattened, glabrous; staminodes absent; carpels free, 20 to 35, ovary ca. 3 mm long, stigma lobed, pubescent. Monocarps stipitate, stipes 8–19 mm long, ca. 1 mm in diameter; monocarps 8 to 20, 19–25 mm long, 10–14 mm in diameter, ellipsoid, apex mucronate, glabrous, smooth, glossy, green turning red to black when ripe; seeds ca. 20 mm long, ca. 10 mm in diameter, ellipsoid; aril absent.

#### Distribution.

From Cameroon to Gabon, and one collection in northern Republic of Congo; in Cameroon known from the South region.

#### Habitat.

A rare species; in lowland rain forests, mainly in primary habitats. Altitude 300–700 m a.s.l.

#### Local and common names known in Cameroon.

M’Fo, Mofo, Mpuley (dial. Mab Kwasio, *van Andel 4216*); N’jie (Dials. Duala, Punu, *Bos 4962*).

#### IUCN conservation status.

Least Concern (LC) (Cosiaux et al. 2019c).

#### Uses in Cameroon.

None recorded.

#### Notes.

﻿﻿﻿*Annickialetestui* is characterized by having tomentose young foliate branches and petioles, the lower side of the leaf blades are pubescent with appressed or erect hairs that are simple, bifid, fasciculate or stellate, pointing in all directions. Versteegh and Sosef (2007) note that the indumentum is quite variable within this species, even within individuals, varying from short and appressed to erect and longer hairs.

Vernacular names are likely to apply to other species of the genus.

#### Specimens examined.

**South Region**: 15 km from Kribi Lolodorf road, 3.00°N, 10.02°E, *01 July 1969*, *Bos J.J.* 4962 (MO,WAG); Mendoum, 2.22°N, 11.23°E, *13 February 1965*, *Raynal J.* 13392 (P); Campo-Ma’an area Bifa, 2.67°N, 10.28°E, *13 October 2001*, *Tchouto Mbatchou G.P.* BIFAX_150 (WAG); Campo-Ma’an area 2.73°N, 9.873°E, *16 August 2001*, *van Andel T.R.* 3882 (WAG); Campo-Ma’an area near Boussebeliga creek bridge, 2.37°N, 9.822°E, *26 October 2001*, *van Andel T.R.* 4216 (WAG).

**Figure 6.**
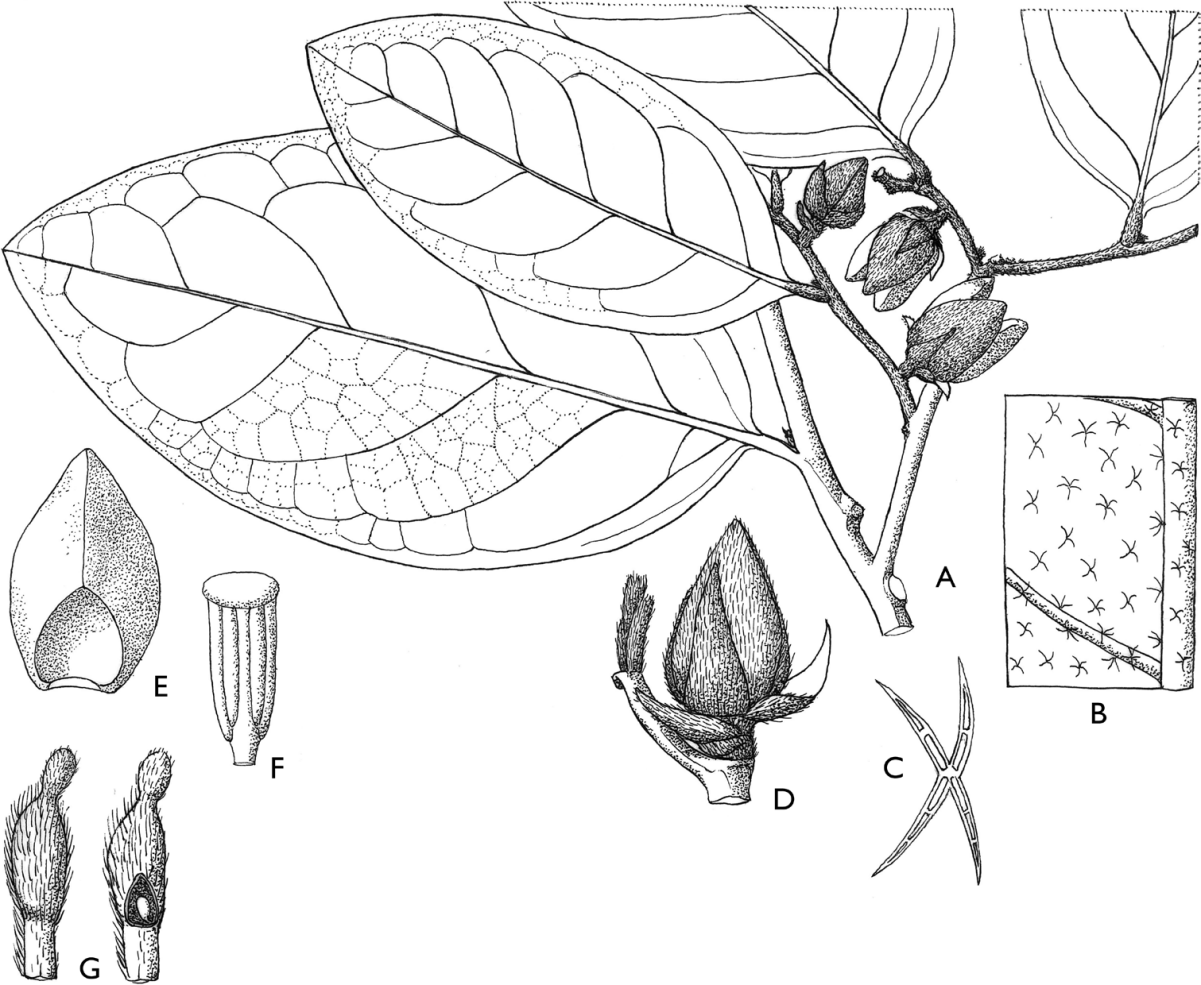
*Annickialetestui***A** flowering branch **B** detail of lower side of leaf blade **C** detail of stellate hair **D** flower bud **E** inner petal, inside view **F** stamen **G** carpel, whole and showing the single basal ovule **A–G** from *Le Testu 8432*. Drawings by Hélène Lamourdedieu, Publications Scientifiques du Muséum national d’Histoire naturelle, Paris; modified from Le Thomas (1969b; pl. 56, p. 307, pro parte).

**Figure 7.**
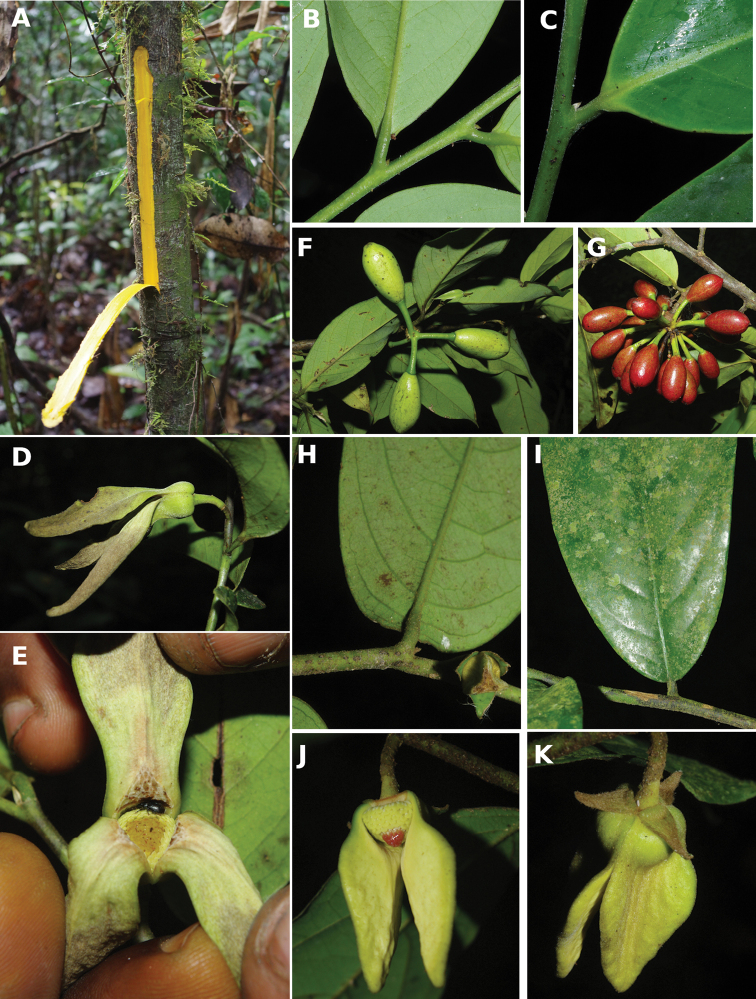
*Annickiaaffinis***A** detail of the yellow bark **B** lower side of leaf, petiole and branch **C** upper side of leaf, petiole and branch **D** flower **E** detail of receptacle **F** and **G** fruits. *Annickiachlorantha***H** lower side of leaf, petiole and branch **I** upper side of leaf, petiole and branch **J** flowers, side view, one petal removed showing receptacle **K** flower, top view showing the three sepals in front of the petals **A** no voucher **B–C***Couvreur 1123*, Gabon **D, E***Couvreur 469*, Lélé, Cameroon **F***Couvreur 519*, Mt Etinde, Cameroon **G***Couvreur 591*, Gabon. Photos Thomas L.P. Couvreur.

### ﻿﻿﻿﻿ Annickia polycarpa

Taxon classificationPlantaeMagnolialesAnnonaceae

(DC.) Setten & Maas ex I.M. Turner, Phytotaxa 32: 52, 2011

ABDAE698-B186-5478-B7C0-4A6621BC7ED9

Fig. 5 ; Map 1E

≡ ﻿Unona polycarpa DC., Syst. Nat. 1: 499, 1817; ﻿﻿Coelocline polycarpa (DC.) A.DC., Mém. Anon.: 33, 1832; ﻿Melodorumpolycarpum (DC.) Benth., Trans. Linn. Soc. London 23: 477–478, 1862; ﻿Xylopia? polycarpa (DC.) Oliv., Fl. Trop. Afr. 1: 32, 1868.

#### Type.

Sierra Leone. no region; no locality, *Afzelius A. s.n.*, no date: holotype: B[B 10 0068937]; isotype: BM[BM000547035].

#### Description.

Tree, 2–20 m tall, d.b.h. unknown; stilt roots or buttresses absent, **slash yellow.** Indumentum of simple, bifid, fasciculate or star hairs; old leafless branches glabrous, **young foliate branches densely pubescent to tomentose.** Leaves: petiole 3–8 mm long, 2 mm in diameter, **densely pubescent to tomentose**, grooved, blade inserted on the side of the petiole; blade 5–27 cm long, 2–8 cm wide, elliptic to obovate, apex acuminate, acumen 1–2 cm long, base cuneate to acuminate, coriaceous, below pubescent when young and old, hairs mainly bifid or stellate but some simple too, pointing in all directions, above pubescent when young and old, concolorous; midrib sunken or flat, above pubescent towards base when young, pubescent towards base when old, below pubescent when young, sparsely pubescent when old; secondary veins 8 to13 pairs, pubescent below; tertiary venation intermediate. Individuals bisexual; inflorescences ramiflorous on old or young foliate branches, leaf opposed or extra axillary. Flowers with 6 perianth parts in 2 whorls, 1 per inflorescence; pedicel 9–19 mm long, ca. 2 mm in diameter, pubescent; in fruit 20 mm long, 4–5 mm in diameter, pubescent; bracts 2, one basal and one upper towards the middle of pedicel, basal bract 8 mm long, 4 mm wide; sepals 3, valvate, free, 9–22 mm long, 4–5 mm wide, triangular, apex acute, base truncate, pubescent outside, glabrous inside, margins flat; petals free; outer petals absent; inner petals 3, valvate, 23–34 mm long, 8–19 mm wide, elliptic to inversely Y–shaped ridged, apex acute, base broad and concave, claw mm long, yellow, margins wavy, densely pubescent outside, glabrous inside; stamens 90 to 200, in 5 to 6 rows, 3–4 mm long, linear; connective flattened, glabrous; staminodes absent; carpels free, ca. 70, ca. ovary 3 mm long, stigma lobed, sparsely pubescent. Monocarps stipitate, **stipes 19–59 mm long**, 1–2 mm in diameter, monocarps 5 to 55, 18–23 mm long, 8–12 mm in diameter, obovoid, apex mucronate, sparsely pubescent, smooth, glossy, green turning red to black when ripe; seed 1, ca. 20 mm long, ca. 10 mm in diameter, ellipsoid; aril absent.

#### Distribution.

A mainly West African species, from Sierra Leone to Cameroon; in Cameroon known from the South region.

#### Habitat.

A rare species in Cameroon, in lowland and pre-montane rain forests mainly in primary habitats. Altitude 110–1400 m a.s.l.

#### Local and common names known in Cameroon.

Pola (Mvaï, Fang, *Annet 174*); African yellow wood, yellow wood (english); Moambe jaune (french).

#### IUCN conservation status.

Least Concern (LC) (Cosiaux et al. 2019d).

#### Uses in Cameroon.

***medicine***: bark as an antisepctic, against fever, malaria; ***construction***: house building, furniture; ***dyes and tannins***: as a yellow dye.

#### Notes.

﻿﻿﻿*Annickiapolycarpa* is distinguished by the densely pubescent to tomentose upper side of the midrib and the petioles, and generally long stipes.

#### Specimens examined.

**South Region**: Bipindi, 3.26°N, 10.20°E, *09 June 1928*, *Annet E.* 174 (P).

### ﻿﻿ Annona

Taxon classificationPlantaeMagnolialesAnnonaceae

L. , Sp. Pl. 1: 536, 1753

85CB4E34-9BAF-5E28-8DE3-DE3494D3CE25

= ﻿Guanabanus Mill. Gard. Dict. Abr., ed. 4: 2, 1754.

#### Type species.

﻿﻿﻿*Annonamuricata* L.

#### Description.

Trees, 1–10 m tall, d.b.h. 2–10 cm; stilt roots or buttresses absent. Indumentum of simple hairs. Leaves: petiole 7–20 mm long, 1–3 mm in diameter, blade 6–25 cm long, 4–19 cm wide, broadly obovate or obovate to broadly elliptic to elliptic, apex rounded or obtuse or shortly emarginated, base subcordate to rounded, discolorous, whitish below or concolorous; midrib sunken or flat; secondary veins 7 to 16 pairs; tertiary venation reticulate. Individuals bisexual; inflorescences ramiflorous on young and old leafless branches, leaf opposed. Flowers with 9 perianth parts in 3 whorls, 1 to 2 per inflorescence; flowering peduncle sometimes present, short; pedicel 10–25 mm long; in fruit 15–50 mm long; bracts 2, all basal, 1–5 mm long; sepals 3, valvate, free, 3–4 mm long, triangular to ovate, apex acute, base truncate; petals free; outer petals longer than inner; outer petals 3, valvate, 10–15 mm long, 8–10 mm wide, ovate, apex acute, base truncate; inner petals 3, valvate, 8–10 mm long, 3–4 mm wide, narrowly oblong or narrowly elliptic, apex acute to obtuse, base truncate; stamens numerous (not counted), in 2 to 3 rows, 2–3 mm long, linear; connective discoid, shortly pubescent; staminodes absent; carpels free, numerous (not counted), ovary 1–2 mm long, stigma capitate, glabrous or pubescent. Fruit pseudosyncarpous, 20–50 mm long, 20–50 mm in diameter, obovoid to globose; monocarps sessile, completely fused between them, numerous (not counted); seed 1, 8–10 mm long, 4–5 mm in diameter, flattened ellipsoid, irregular in shape; aril absent.

A mainly South American genus, one of the largest in Annonaceae with about 170 accepted species (Rainer 2001). In Africa, there are between three or four native species, with numerous subspecies and varieties and of which the taxonomy remains complicated (Robyns and Ghesquière 1934; Sillans 1952; Le Thomas 1969c). ﻿﻿*Annonaglabra*L. is probably of South American origin (Le Thomas 1969b, 1969c) but is naturalized along the coast of West and Central Africa. In Cameroon it is also found in mangrove areas, but is little collected (e.g. *van der Burgt 130* (WAG)) We thus include it in the key, but do not provide a description. In addition, this genus contains the non-native edible species ﻿﻿*Annonasquamosa*L., ﻿﻿*A.muricata*L. and ﻿*A.reticulata*L. (from South America), all of which can be found in cultivation (not included in the descriptions) in Cameroon.

#### Taxonomy.

no recent revision, but see Le Thomas (1969c), Le Thomas (1969b).

### ﻿Key to the species and taxa of ﻿*Annona* in Cameroon

**Table:** 

1	Leaves glabrous, elliptic in shape with an acuminate apex, petiole inserted on the side of the petiole	﻿***A.glabra***
–	Young leaves always pubescent, generally obovate (more rarely elliptic), rounded to emarginated at the apex; petiole inserted on the top of the petiole	**2**
2	Lower side of leaf blade tomentose with short curly hairs covering the whole blade	**A.senegalensissubsp.oulotricha**
–	Lower side of leaf blade glabrescent to densely pubescent with non-curly hairs	**A.senegalensissubsp.senegalensis**

### ﻿﻿ Annona senegalensis Pers. ssp. oulotricha

Taxon classificationPlantaeMagnolialesAnnonaceae

Le Thomas, Hallé, Fl. Gabon, vol. 16: 322, 1969

15594C90-CF5A-54C5-8F9A-DF3B130721CA

Fig. 8 ; Map 1G

= Annona arenaria Thonn. var. obtusa Robyns & Ghesq., Bull. Soc. Roy. Bot. Belge 67: 22 (1934). Type. Republic of the Congo. Pool, Brazzaville, Chevalier A.J.B. 27304, Jul 1912; holotype: P[P00363246].
= ﻿﻿Annona arenaria auct., non Thonn., Robyns & Ghesq., Bull. Soc. Roy. Bot. Belge 67: 22 (1934); Annona senegalensis Pers. var. arenaria (Thonn.) Sillans, Bull. Mus. Natl. Hist. Nat., sér. 2, 24: 581 (1952). Type. Democratic Republic of the Congo. Kongo-Central, Temvo, *Vermoesen F.M.C. 1592*, 20 Fev 1919: neotype, designated by Robyns and Ghesquière (1934, p. 25), sheet here designated: BR[BR0000013871604]; isoneotype: BR[BR0000013871611].

#### Type.

Republic of the Congo. Pool; Bord de la M’Boté, *Bouquet*, *A. 513*, 12 Sep 1964: holotype: P[P00363247].

#### Description.

Tree to shrub, 1–6(8) m tall, d.b.h. unknown; stilt roots or buttresses absent. Indumentum of simple hairs; old leafless branches glabrous, **young foliate branches brown tomentose**. Leaves: petiole 7–20 mm long, 2–3 mm in diameter, brown tomentose, grooved, blade inserted on top of the petiole; blade 6–20 cm long, 5–12 cm wide, obovate to elliptic, apex rounded or obtuse or shortly emarginate, base rounded to subcordate, papyraceous to coriaceous, **below densely pubescent, curly hairs covering the whole leaf blade when young and old**, above sparsely pubescent to glabrous when young, glabrous when old, **discolorous**, **whitish below**; midrib impressed, above glabrous when young and old, below densely pubescent when young and old; secondary veins 8 to 15 pairs, glabrous above; tertiary venation percurrent but also appearing reticulate. Individuals bisexual; inflorescences ramiflorous on old or young foliate branches, leaf opposed or extra axillary. Flowers with 9 perianth parts in 3 whorls, 1 to 2 per inflorescence; pedicel 10–25 mm long, 1–2 mm in diameter, brown tomentose; in fruit 15–30 mm long, 3–4 mm in diameter, pubescent; bracts 2, all basal, 2–5 mm long, 2–3 mm wide; sepals 3, valvate, free, 3–4 mm long, 3–4 mm wide, triangular to ovate, apex acute, base truncate, green, densely pubescent outside, glabrous inside, margins flat; petals free, inner smaller than outer; outer petals 3, 10–15 mm long, 8–10 mm wide, ovate, apex acute, base truncate, yellow to green, margins flat, tomentose outside, glabrous inside; inner petals 3, valvate, 8–10 mm long, 3–4 mm wide, narrowly oblong or narrowly elliptic, apex acute, base truncate, yellow-green, margins flat, glabrous outside, glabrous inside; stamens numerous, rows not counted, 2–3 mm long, linear; connective discoid, shortly pubescent; staminodes absent; carpels free, numerous, ovary 1–2 mm long, stigma capitate, pubescent. Fruit pseudosyncarpous, 20–50 mm long, 20–50 mm in diameter, obovoid to globose, yellow orange at maturity; individual monocarps 20 to 30, sessile, completely fused between them; apex shortly pyramidal, brown tomentose, smooth, yellow to orange when ripe; seed 1, 8–10 mm long, 4–5 mm in diameter, flattened ellipsoid; aril absent.

#### Distribution.

A west and central African subspecies distributed from Guinea to Ivory Coast and from Cameroon to the Democratic Republic of the Congo and the Central African Republic; in Cameroon known from Adamaoua, Central, East, North, North-West, South-West and West regions.

#### Habitat.

A common species; in lowland savanna regions towards the north, at higher altitudes towards the south, sometimes the dominant tree species in the savanna, reported to naturally invade certain areas (Le Thomas 1969c). Altitude 200–1300 m a.s.l.

#### Local and common names known in Cameroon.

None recorded.

#### IUCN conservation status.

Least Concern (LC) (Botanic Gardens Conservation International and IUCN SSC Global Tree Specialist Group 2019a).

#### Uses in Cameroon.

None recorded, but probably same as for var.senegalensis.

#### Notes.

Subsp. oulotricha is distinguished by the pubescence of the lower surface of leaf blades, which is tomentose with short curly hairs. Besides that, it is very close morphologically to subsp. senegalensis. The species (*A.senegalensis*) as a whole is very variable morphologically and widespread across the drier parts of sub-Saharan Africa (west to east), also occurring in northern Madagascar (Le Thomas 1969c). Though we have followed the classification of Le Thomas (1969c), the taxonomic limits in this group would need more in-depth studies.

Cheek et al. (2000, p. 114), in the Check list of plants of Mt Oku, reported the presence of ﻿*A.chrysophylla* Bojer (*Brunt 234*), but this specimen has now been identified as A.senegalensissubsp.oulotricha. Moreover, the former name is now a synonym of A.senegalensissubsp.senegalensis (Le Thomas 1969c).

Robyns and Ghesquière (1934) chose a neotype for the species ﻿*A.arenaria* (now a synonym of A.senegalensissubspeciessenegalensis), thinking that no original material seen by Thonning remained (see under that name for details). However, in doing so, they chose a neotype specimen belonging to A.senegalensissubsp.oulotricha (Le Thomas 1969c). When describing this latter species, Le Thomas chose a different type than the one selected by Robyns and Ghesquière (1934) as not to “bring extra confusion to the situation” (Le Thomas 1969c).

**Figure 8.**
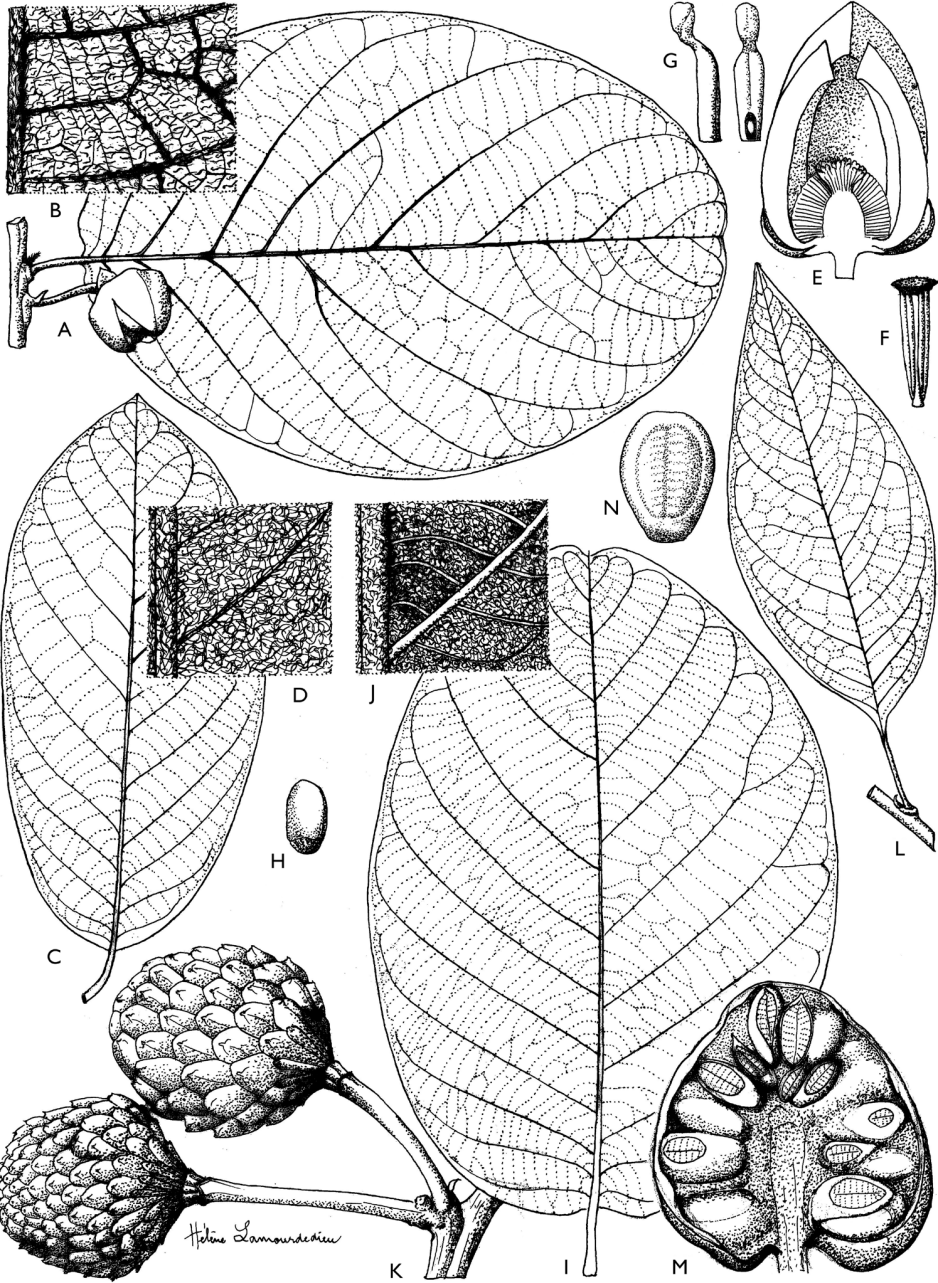
Annonasenegalensissubsp.senegalensis**A** flowering branch **B** detail of pubescence on lower side of leaf blade **C** leaf **D** detail of pubescence on lower side of leaf blade **E** longitudinal section of the flower **F** stamen **G** carpel, side view and view of the single basal ovule **H** seed. Annonasenegalensissubsp.oulotricha**I** leaf **J** detail of pubescence on lower side of leaf blade **K** fruit branch showing two pseudosyncarpous fruits. *Annonaglabra***L** leaf **M** section of fruit. Drawings by Hélène Lamourdedieu, Publications Scientifiques du Muséum national d’Histoire naturelle, Paris; modified from Le Thomas (1969b; pl. 56, p. 321).

#### Specimens examined.

**Adamaoua Region**: Meiganga, 6.52°N, 14.3°E, *06 February 1946*, *Aubréville A.* 729 (P); Tignère, 7.37°N, 12.65°E, *01 March 1939*, *Jacques-Félix H.* 3423 (P); Ngaoundéré, 7.32°N, 13.58°E, *01 June 1939*, *Jacques-Félix H.* 4012 (P); 11 km de Ngaoundéré vers Ngakha, 7.32°N, 13.58°E, *06 March 1958*, *Letouzey R.* 606 (P). **Central Region**: Mont Ngolep massif Ngoro 38 km N de Bafia, 5.09°N, 11.26°E, *21 April 1975*, *Ngameni B.K.* 51 (P); Mont Ngoro à 58 km SW de Linte, 5.09°N, 11.26°E, *17 April 1982*, *Nkongmeneck B.A.* 256 (P). **East Region**: Bétaré Oya, 5.5°N, 14.1°E, *02 March 1961*, *Breteler F.J.* 1185 (P); Piste Moyenam Rivière Konbo, 4.58°N, 13.68°E, *29 February 1960*, *Letouzey R.* 3187 (P); Piste Moyenam-rivière Koubou, 4.58°N, 13.68°E, *29 February 1960*, *Letouzey R.* 3191 (P). **North Region**: Garoua, 9.3°N, 13.4°E, *10 February 1946*, *Aubréville A.* 787 (P); Garoua, 9.3°N, 13.4°E, *11 February 1946*, *Aubréville A.* 804 (P); 17 km N of Banyo along road to Mba, 6.91°N, 11.8°E, *29 February 1972*, *Leeuwenberg A.J.M.* 9440 (WAG). **West Region**: Dschang, 5.45°N, 9.95°E, *13 April 1966*, *CNAD* 317 (P); Nkounden, 5.7°N, 10.67°E, *01 May 1967*, *CNAD* 808 (P); Bangwa ca. 15 km NW of Baganté, 5.2°N, 10.48°E, *30 April 1964*, *de Wilde W.J.J.O* 2359 (P); Between Bangwa and Bangangté ca 8 km NW of Bangangté, 5.16°N, 10.5°E, *12 May 1964*, *de Wilde W.J.J.O* 2589 (P,WAG); Batchingou, 5.13°N, 10.4°E, *01 January 1939*, *Jacques-Félix H.* 3026 (P); Kontchankap, 5.58°N, 10.80°E, *01 February 1939*, *Jacques-Félix H.* 3033 (P); Foumban, 5.72°N, 10.92°E, *01 February 1939*, *Jacques-Félix H.* 3136 (P).

### ﻿﻿ Annona senegalensis Pers. ssp. senegalensis

Taxon classificationPlantaeMagnolialesAnnonaceae

, Syn. Pl. 2: 95, 1807

ECFE7930-118D-5DBD-B175-636E566F2B28

Fig. 8 ; Map 1F

= Annona arenaria Thonn., Beskr. Guin. Pl. 257, 1827. *non* Robyns & Ghesq., Bull. Soc. Roy. Bot. Belge 67: 22, 1934. Type. Ghana. *Thonning s.n.*: holotype: P [Herb. Jussieu, number: 10799].
= ﻿﻿Annona chrysophylla Bojer, Ann. Sci. Nat., Bot. sér. 2, 20: 53, 1943; Annona senegalensis var. chrysophylla (Boj.) Sillans, Bull. Mus. Natl. Hist. Nat., sér. 2, 24: 581 (1952). Type. Comores. Anjouan [Ndzuwani, Nzwani], *Bojer W. s.n.*, s.d.: holotype: We were not able to locate the type specimen. Verdcourt (1971a) suggests it is possibly in P, but it isn’t amongst the scanned specimens, so likely not in P. No specimens were found in W either.
= Annona senegalensis var. latifolia Oliv., Fl. Trop. Africa: 17, 1868. Type. Uganda. Northern region, Madi, *Speke & Grant s.n.*, *s.d.*: holotype: We were not able to locate the type specimen, which should be in Kew (Oliver 1868, p. 17, Robyns and Ghesquière 1934).
﻿﻿Annona porpetac Boiv. ex Baill.; Bull. Mens. Soc. Linn. Paris 1. 341, 1882; Annona senegalensis var. porpetac (Boiv. Ex Baill.) Diels, Notizbl. Konigl. Bot. Gart. Berlin 9: 356, 1934. Type. Madagascar. Antsiranana Province, Nossi Be, *Bovin M. 2115*, 1846: holotype: P[P030360].

#### Type.

Senegal: *Roussillon 69*, 1798: holotype: P[P00363244].

#### Description.

Tree to shrub, 1–10 m tall, d.b.h. 2–10 cm; stilt roots or buttresses absent. Indumentum of simple hairs; old leafless branches glabrous, young foliate branches brown tomentose. Leaves: petiole 10–20 mm long, 1–2 mm in diameter, brown tomentose, grooved, blade inserted on top of the petiole; blade 7–25 cm long, 4–19 cm wide, broadly obovate to broadly elliptic, apex rounded, base subcordate, subcoriaceous to coriaceous, **below densely pubescent with straight hairs to glabrescent when young and old**, above sparsely pubescent to glabrous when young, glabrous when old, **discolorous**, **whitish below**; midrib impressed, above glabrous when young and old, below densely pubescent when young and old; secondary veins 7 to 16 pairs, glabrous above; tertiary venation percurrent but also appearing reticulate, dense. Individuals bisexual; inflorescences ramiflorous on old or young foliate branches, leaf opposed. Flowers with 9 perianth parts in 3 whorls, 1 to 2 per inflorescence; pedicel 10–20 mm long, 1–2 mm in diameter, brown tomentose; in fruit 25–50 mm long, 3–4 mm in diameter, pubescent; bracts 2, all basal, 1–4 mm long, 2–3 mm wide; sepals 3, valvate, free, 3–4 mm long, 3–4 mm wide, broadly triangular to circular, apex acute, base truncate, green, densely pubescent outside, glabrous inside, margins flat; petals free, outer longer than inner; outer petals 3, 10–15 mm long, 8–9 mm wide, ovate, apex acute, base truncate, yellow to green, margins flat, tomentose outside, glabrous inside; inner petals 3, valvate, 8–10 mm long, 3–4 mm wide, narrowly oblong or narrowly elliptic, apex acute, base truncate, yellow-green, margins flat, glabrous outside, glabrous inside; stamens numerous (not counted), 2–3 mm long, linear; connective discoid, shortly pubescent; staminodes absent; carpels free, numerous (not counted), ovary 1–2 mm long, stigma capitate, glabrous. Fruit pseudosyncarpous; 20–50 mm long, 20–30 mm in diameter, obovoid to globose, yellow orange at maturity, monocarps sessile, numerous, apex flat, glabrous, smooth, yellow to orange when ripe; seed 1, 8–10 mm long, 4–5 mm in diameter, flattened ellipsoid; aril absent.

#### Distribution.

A west, central and east African and northern Malagasy subspecies from Senegal to Mozambique; in Cameroon known from the Adamaoua, Central, East, Far North, North, North-West, South-West and West regions.

#### Habitat.

A common species; in lowland savanna regions towards the north, at higher altitudes towards the southern region, may, sometimes be the dominant tree species across the savanna. Altitude 100–1400 m a.s.l.

#### Local and common names known in Cameroon.

Falŏ (dial. Bamileke (Burkill 1985)); pomme-cannelle du Sénégal (French); African custard-apple; wild custard apple, wild soursop (English).

#### Uses in Cameroon.

***food***: fruit is eaten, flower for sauces, condiments, spices, flavourings; ***medicine***: root as pain-killer, against diarrh dysentery, cholera, venereal diseases, bark used as vermifuges, diuretics, genital stimulants/depressants, lactation stimulants; ***construction***: house building, furniture; ***dyes and tannins***: astringents, insecticides, arachnicides; ***products***: wood fire; fuel and lighting; ***social***: religion, superstitions, magic.

#### Notes.

﻿Annonasenegalensissubsp.senegalensis is distinguished by the pubescence of the lower side of the leaf blade which ranges from densely pubescent (but not tomentose) with short but straight hairs (not curly as in subsp. oulotricha) to glabrescent. See notes under subsp. oulotricha and Le Thomas (1969c) for more details. The fruits are edible.

Le Thomas (1969c p. 2) suggested that there exists a specimen of Thonning sent to Jussieu by Vahl in 1804 and present in P (under catalogue number 10779 from Herb. Jussieu) and that this would be the lectotype. However, we were not able to locate this specimen using the online scanned material.

#### Specimens examined.

**Adamaoua Region**: Dodéo, 7.48°N, 12.07°E, *01 March 1939*, *Jacques-Félix H.* 3388 (P); Bountoun Mboun mountains ca 40 km N of Ngaoundere, 7.9°N, 13.48°E, *12 April 1977*, *Nordal I.* 929 (P). **Central Region**: Bibbanga, 3.72°N, 10.3°E, *09 March 1927*, *Hédin L.* 409 (P). **East Region**: Bertoua-Batouri, 4.58°N, 13.68°E, *01 January 1962*, *Vroumsia T.* 116 (P). **Far-North Region**: Douzeye (c Bongor), 10.1°N, 15.28°E, *08 January 1968*, *Achoundong G.* 1385 (P); Plaine de Maroua à 5 km au NO de Maroua, 10.6°N, 14.28°E, *18 August 1964*, *Biholong M.* 28 (P); ca 5 km W of Maroua, 10.6°N, 14.28°E, *02 September 1964*, *de Wilde W.J.J.O* 2966 (MO); Bogo (Maroua), 10.7°N, 14.61°E, *01 May 1939*, *Jacques-Félix H.* 3737 (P); Reserve forestière du Mayo Louti (10 km W de Mokolo), 10.7°N, 13.8°E, *10 September 1964*, *Letouzey R.* 6779 (P); Mora, 11.0°N, 14.14°E, *01 January 1945*, *Vaillant A.* 15 (P). **North Region**: Pitoa, 9.38°N, 13.50°E, *25 March 1974*, *Achoundong G.* 3419 (P); Garoua, 9.3°N, 13.4°E, *04 August 1955*, *de Wit H.C.D* 7182 (WAG); Ecole de faune de Garoua, 9.3°N, 13.4°E, *09 August 2000*, *Dong E.* 391 (P). **North-West Region**: Piste Munka (=Munkep) 45 km NNW Wum, 6.8°N, 9.97°E, *09 July 1975*, *Letouzey R.* 13988 (MO). **South-West Region**: Ndop Plain Hillside above Courtar Ndop Amp ref No 28, 6.02°N, 10.49°E, *01 March 1962*, *Brunt M.A.* 51 (K). **West Region**: Bangwa, 5.2°N, 10.48°E, *12 May 1964*, *de Wilde W.J.J.O* 2389 (P,WAG).

### ﻿﻿ Anonidium

Taxon classificationPlantaeMagnolialesAnnonaceae

Engl. & Diels, Notizbl. Königl. Bot. Gart. Berlin 3: 56, 1900

2CC7D69C-35FA-5769-A152-4DB857B6A5E7

#### Type species.

﻿﻿﻿*Anonidiummannii* Engl. & Diels.

#### Description.

Trees, 4–30 m tall, d.b.h. up to 80 cm; stilt roots or buttresses absent. Indumentum of simple hairs. Leaves: petiole 3–10 mm long, 2–5 mm in diameter, blade 20–50 cm long, 7–18 cm wide, oblong to obovate, apex rounded or abruptly acuminate, base subcordate forming two small lobes on top of the petiole, concolorous; midrib sunken or flat; secondary veins 10 to 20 pairs; tertiary venation reticulate. Individuals androdioecious or dioecious; inflorescences cauliflorous or ramiflorous on old leafless branches, axillary. Flowers with 9 perianth parts in 3 whorls. 5 to 20 or more per inflorescence; flowering peduncle long, up to 2–4 m, woody, hanging or semi erect; pedicel 10–70 mm long; in fruit 25–100 mm long; bracts 2–4, basal or inserted along the pedicel, 1–5 mm long; sepals 3, valvate, free, 3–4 mm long, triangular to ovate, apex acute, base truncate; petals free; outer petals longer than inner; outer petals 3, valvate, 10–15 mm long, 8–10 mm wide, ovate, apex acute, base truncate; inner petals 3, valvate, 8–10 mm long, 3–4 mm wide, narrowly oblong or narrowly elliptic, apex acute to obtuse, base truncate; stamens 65 to 700, in 2 to 3 rows, 2–3 mm long, linear; connective discoid, shortly pubescent; staminodes absent; carpels free, 180 to 260, ovary 1–2 mm long, stigma capitate, glabrous or pubescent. Fruit pseudosyncarpous, 20–50 mm long, 20–50 mm in diameter, obovoid to globose; monocarps sessile, completely fused, 250 to 500; seed 1, 8–10 mm long, 4–5 mm in diameter, flattened ellipsoid; aril absent.

A genus of trees with four species distributed in Central Africa, one widespread and common across its range (﻿﻿*A.mannii*) and three others mainly in Gabon (two endemic); in Cameroon two species, none endemic.

﻿﻿*Anonidiumusambarense* R.E.Fr, endemic to Tanzania, is in fact a ﻿*Polyceratocarpus* species (probably ﻿﻿*Polyceratocarpusscheffleri* Engl. & Diels, Couvreur, pers. obs.). Finally, one study (Focho et al. 2010) reports the presence of ﻿﻿*Anonidiumfloribundum* Pellegr. in the Mount Cameroon area. However, no herbarium collection is available to confirm this and we do not consider it present in Cameroon for now.

#### Taxonomy.

No recent revision has yet been published, but see Le Thomas (1969b) were most species are treated for Gabon.

### ﻿Key to the species of ﻿*Anonidium* in Cameroon:

**Table:** 

1	Leaves obovate to oblong-elliptic, 20–45 cm, upper bract inserted at middle of flowering pedicel; individuals male or hermaphrodite (androdioecious); sepals basally fused; outer petals 25–50 mm long, 20–40 mm wide, elliptic to obovate	﻿﻿***A.mannii***
–	Leaves distinctly oblong, 37–50 cm long; upper bract inserted directly under the calyx, orbicular; individuals female or male (dioecious), sepals free, outer petals 35–60 mm long, 10–18 mm wide; narrowly elliptic to narrowly oblong	﻿***A.brieyi***

### ﻿﻿﻿﻿ Anonidium brieyi

Taxon classificationPlantaeMagnolialesAnnonaceae

De Wild., Repert. Spec. Nov. Regni Veg. 13: 383, 1914

9F1C6D87-5D17-50C2-8262-D746B473B786

**Figure 9.**
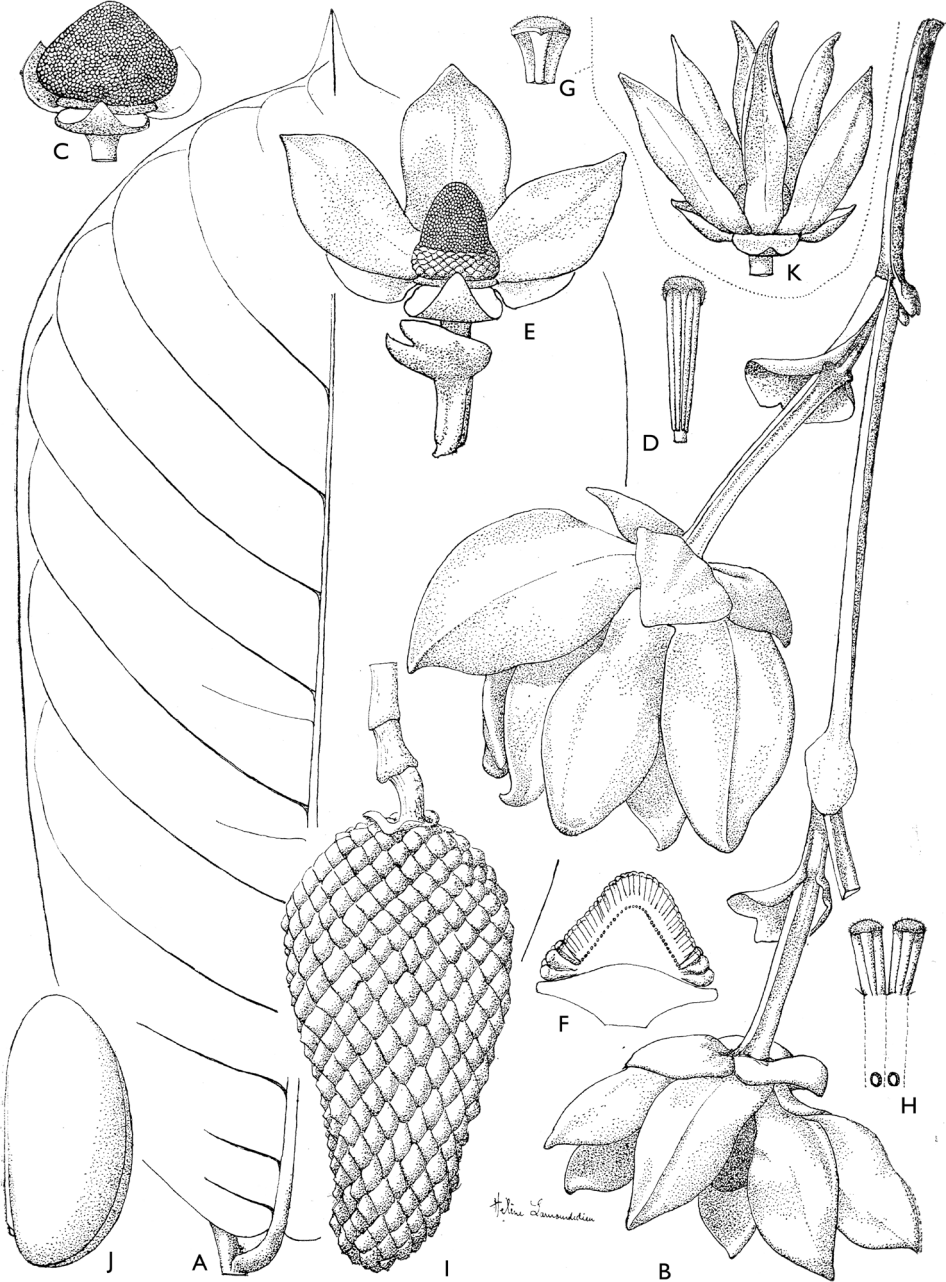
*Anonidiummannii***A** leaf **B** male inflorescence, note upper bract inserted towards lower part of pedicel **C** receptacle of male flower, all petals removed **D** stamen **E** receptacle of bisexual flower, note small row of stamens at base of carpels **F** longitudinal section of bisexual flower **G** stamen of bisexual flower, front view **H** two free carpels, showing basal ovules **I** fruit, note syncarpous nature, referred to as pseudosycarpous **J** seed, side view. *Anonidiumbrieyi***K** flower, note narrow petals **A** from *Le Testu 9169***B–D** from *Le Testu 9509***E–H** from *Le Testu 7269***I** from Nigerian tree photo **J** from *Le Testu s.n.***K** from *Le Testu 1641*. Drawings by Hélène Lamourdedieu, Publications Scientifiques du Muséum national d’Histoire naturelle, Paris; modified from Le Thomas (1969b; pl. 60, p. 331).

**Figure 10.**
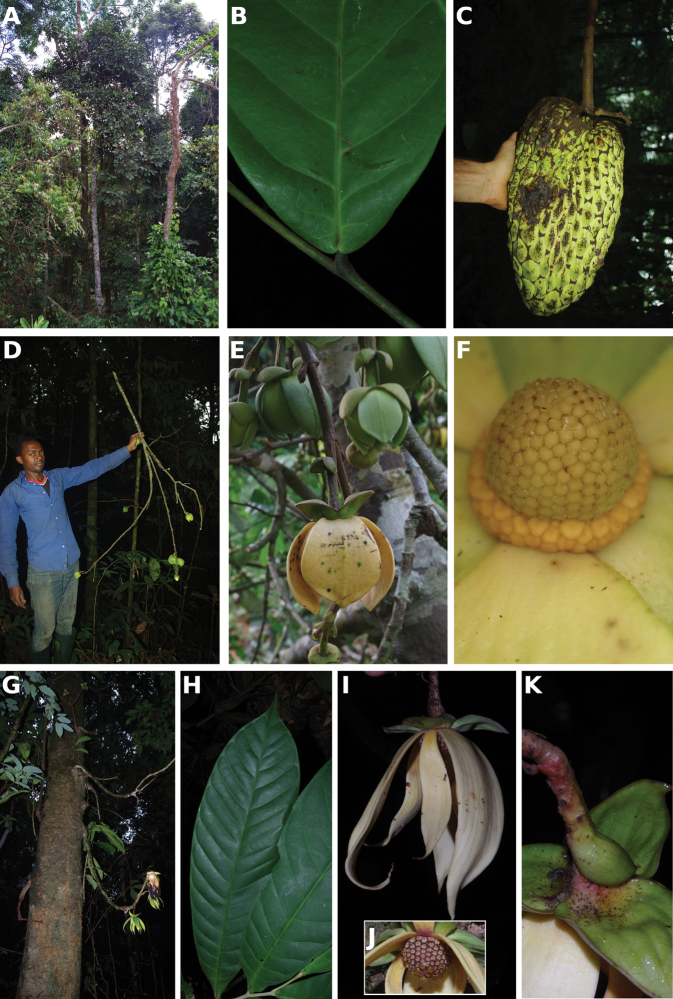
*Anonidiummanni***A** habit, *A.manni* is the species with white trunk in the center **B** base of leaf blade, upper side **C** pseudosyncarpous fruit, note hand for size **D** Narcisse Kamdem holding an inflorescence **E** detail of flower, side view **F** detail of receptacle of bisexual flower, note 2 to 3 rows of stamens at base. *Anonidiumbrieyi***G** trunk with semi erect inflorescences **H** leaf, upper side **I** flower, side view, note narrow petals **J** detail of receptacle in female flower, note absence of stamens at base of carpels **K** flower, top view showing insertion of upper bract just belong the calyx **A** no voucher, Gabon **B, C***Couvreur 449*, Otottomo, Cameroon **D, F***Couvreur 696*, Campo, Cameroon **E***Couvreur 1207*, Maséa, Cameroon **G–K***Couvreur 1132*, Gabon. Photos Thomas L.P. Couvreur.

Figs 9 , 10 ; Map 1H

≡ Anonidium mannii var. brieyi (De Wild.) R.E.Fr., Acta Hort. Berg. 10: 80, 1930.
= Anonidium friesianum Exell, J. Bot. 70, Suppl. Polypet.: 211, 1932. Type. Angola. Mayombe, Buco Zau, *Gossweiler J. 6690*, 16 Sep 1916: lectotype, designated here: LISC[LISC000056]; isolectotypes: COI[COI00004880]; BM[BM000546826, BM000546827]; LISC[LISC000054, LISC000055, LISC000057, LISC000058, LISC000059, LISC000060, LISC000061, LISC000062, LISC000063].

#### Type.

Democratic Republic of the Congo. Bas-Congo; Ganda-Sundi, *de Briey J. 86*, 1911: lectotype, here designated: BR[BR8822635]; isolectotype: BR[BR8822642].

#### Description.

Tree, 15–25 m tall, d.b.h. up to 35 cm; stilt roots or buttresses absent. Indumentum of simple hairs; old leafless branches glabrous, young foliate branches sparsely pubescent to glabrous. Leaves: petiole 3–10 mm long, 2–5 mm in diameter, sparsely pubescent, soon becoming glabrous, slightly grooved, blade inserted on top of the petiole; blade 37–50 cm long, 10–16 cm wide, oblong, apex rounded to abruptly acuminate, acumen 2–3.5 cm long, base rounded, subcordate, subcoriaceous to coriaceous, below sparsely pubescent when young, glabrous when old, above glabrous when young and old, concolorous; midrib impressed, above glabrous when young and old, below sparsely pubescent when young, glabrous when old; secondary veins 14 to 16 pairs, glabrous above; tertiary venation reticulate. **Individuals dioecious**; inflorescences cauliflorous or on leafless branches, axillary; peduncle 50–115 mm long, 3–10 mm in diameter, woody, hanging or semi erect from the trunk, glabrous. Flowers with 9 perianth parts in 3 whorls, **1 to 10 per inflorescence**, male and female inflorescences similar; pedicel 10–35 mm long, 2–4 mm in diameter, glabrous; bracts, 1 to 2 basal and one upper towards the upper of pedicel, basal bracts 5–10 mm long, 5–10 mm wide; upper bract directly under the calyx, amplexicaul, 12–21 mm long, 10–20 mm wide; sepals 3, valvate, free, 20–40 mm long, 14–18 mm wide, triangular, apex long acuminate, gradually tapering into an acute apex, base truncate, green, densely pubescent outside, glabrous inside, margins flat; petals free, sub equal; outer petals 3, 35–60 mm long, 10–18 mm wide, narrowly elliptic to obovate, apex acute to attenuate, base attenuate, green turning yellow, margins flat, pubescent outside, pubescent inside; **inner petals 3, valvate, 30–50 mm long, 9–16 mm wide**, narrowly elliptic, apex acute, base truncate, green turning yellow, margins flat, pubescent outside, pubescent inside; stamens in male flowers: 500 to 700 inserted on a conical receptacle, in 14 to 17 rows, 3.5–5 mm long, oblong, connective truncate, sparsely pubescent, green to cream-yellow; carpels in female flower (see notes) 220 to 260, ovary 2–3 mm long, stigma capitate, glabrous; staminodes absent. Whole fruits not seen, label information: **ca. 20 cm long, ca. 10 cm in diameter** [taken from descriptions on specimens *Hallé & Villiers 4505*, P02032580], white [taken from *Cheek 10240* P00956188]; seed 1, 40–48 mm long, 17–23 mm wide, ellipsoid; aril absent.

#### Distribution.

A central African species, known from Cameroon, Gabon, Republic of the Congo and Angola (Cabinda); in Cameroon known from the Littoral and South-West regions.

#### Habitat.

An uncommon species, in lowland primary or old secondary rain forests. Altitude 100–350 m a.s.l.

#### Local and common names known in Cameroon.

None recorded.

#### IUCN conservation status.

Not evaluated.

#### Uses in Cameroon.

None recorded.

#### Notes.

﻿﻿﻿*Anonidiumbrieyi* differs from ﻿﻿*A.mannii* by its usually larger leaves and the narrower inner (10–18 mm wide versus 15–25 mm in ﻿﻿*A.mannii*), and the upper bract inserted just under the calyx (versus near the middle of the pedicel in ﻿﻿*A.mannii*). Three collections seen have strictly female flowers (*Couvreur 1132*, WAG; *Hallé & Villiers 4505*, [P02032580]; *Sita 712* [MPU411091). The first author’s field observations failed to see any stamens in the thus apparently female flowers (Fig. 10J). A note by Le Testu (*Le Testu 1641* [P02032574]) also indicates that the species is dioecious. This suggests that ﻿*A.brieyi* is a strictly dioecious species (male and female flowers on different individuals) rather than androdioecious as in the other species of ﻿*Anonidium* (Le Thomas 1969b). This is the first time this is suggested to occur in this species as only male flowers were described to date. ﻿﻿*Anonidiumletestui* Pellegr. (endemic to Gabon) has functionally female flowers, but has a small row of sterile stamens at the base (Le Thomas 1969b), something not observed in the two female specimens examined in ﻿*A.brieyi* (Fig. 10J). Variation of sexual systems within genera (e.g. androdioecious and dioecious species) has been reported in African Annonaceae such as ﻿*Monanthotaxis* (Hoekstra et al. 2021) or ﻿*Uvariopsis* (Couvreur and Luke 2010).

Le Thomas (1969b) recognized this species as a variety of ﻿﻿*A.mannii* (﻿﻿A.manniivar.brieyi (De Wild.) Fries), however, the morphological differences in addition to its dioecious nature described above warrant it to be retained at the species level for now. Nevertheless, a phylogeographic study of ﻿﻿*A.mannii* based on hundreds of nuclear markers inferred a nested position of ﻿*A.brieyi* (population collected around reference number *Couvreur 1132*, here identified as ﻿*A.brieyi*) within ﻿﻿*A.mannii* (Helmstetter et al. 2020). This sheds further doubt on the delimitation between these two species and needs further study.

Narrowly elliptic petals are also found in the Gabonese species ﻿*A.floribundum* Pellegr. (not recorded for Cameroon to date). However, this latter species is a small tree up to 4 or 5 m tall, the inflorescences are shorter with fewer flowers, and it is androdioecious (Le Thomas 1969b).

Two herbarium specimens (*Cheek 10240*, Cameroon; *Hallé & Villiers 4505*, Gabon) indicate that the collection had fruits, but we were unable to locate them. *Cheek 10240* states the fruits are white. Collection *Cheek 12040* was identified as ﻿﻿*A.mannii* in the check list of the plants of Mt Kupe (Cheek et al. 2004, p. 236). Four of the type sheets of the name ﻿*A.friesianum* have seeds (*Gossweiler 6690*, LISC000055, LISC000058, LISC000060, LISC000063). On sheets LISC000055 and LISC000061 there are large narrow structures (18 cm by 3.5 cm) which we cannot identify, either being a dried fruit or part of the bark (?).

The sheet *Cheek 10240* from P (P00956188) is a mixed collection. The leaves belong to a species of ﻿*Uvariopsis* (probably *connivens*), and not ﻿*A.brieyi*. The flowers however are from ﻿*A.brieyi.* The other sheets we have seen appear to have the correct leaves (K, WAG [WAG.1378838])

Finally, for the type of ﻿﻿*Anonidiumfriesianum*, among the 10 sheets of *Gossweiler 6690* available at we did not designate one sheet as lectotype because every sheet has important information (leaves, seeds, leaves) and should be considered as a single collection. Among the 10 sheets of *Gossweiler 6690* available at LISC for the type of ﻿﻿*Anonidiumfriesianum*, we selected a single sheet as the lectotype (LISC000056). Indeed, according to the ICBN, different sheets are considered as a single specimen only if they are cross-referenced (e.g. “Sheet I”, “Sheet II”) which is not the case here. The selected sheet (LISC000056) was the only one that had a leaf and a flower (but broken) thus being the most complete. The other sheets only had either just leaves, or inflorescences or flowers or seeds.

#### Specimens examined.

**Littoral Region**: Loum Forest Reserve, 4.73°N, 9.731°E, *16 April 2005*, *Onana J.M.* 3101 (K). **South-West Region**: Banga, 4.55°N, 9.416°E, *01 March 1956*, *Binuyo A.* 35606 (FHO); Southern Bakundu FR, 4.48°N, 9.350°E, *13 March 1948*, *Brenan J.P.M.* 9410 (K,P); Just west of Loum, 4.73°N, 9.729°E, *03 December 1999*, *Cheek M.* 10240 (K,MO,P,WAG,YA); S Bakundu FR, 4.49°N, 9.374°E, *09 April 1951*, *Olorunfemi J.* 30508 (K); 5 km S of Kumba on Buea/Douala Road, 4.65°N, 9.39°E, *21 June 1983*, *Thomas D.W.* 2188 (MO,P,WAG).

### ﻿﻿﻿﻿ Anonidium mannii

Taxon classificationPlantaeMagnolialesAnnonaceae

Engl. & Diels, Notizbl. Königl. Bot. Gart. Berlin 3: 56, 1900

3279AD28-86A7-5000-8196-EF40AE4DD962

Figs 9 , 10 ; Map 1I

≡ Annona mannii Oliv., Hooker’s Icon. Pl. vol. 11: 7–8, 1867.
= Uvaria crassipetala Engl., Notizbl. Königl. Bot. Gart. Berlin 2: 292, 1899. Type. Cameroon. South-West Region, Station Johann-Albrechtshohe, Staudt A. 813, no date: holotype B destroyed, lectotype, here designated: PH[PH28648].

#### Type.

Nigeria. Cross River State; Old Calabar, *Mann G. 2231*, 1863: lectotype, sheet here designated: K[K000198881]; isotypes: B[B100153003]; K[K000198882]; P[P00363249].

#### Description.

Tree, 8–30 m tall, d.b.h. 30–80 cm; stilt roots or buttresses absent. Indumentum of simple hairs; old leafless branches glabrous, young foliate branches sparsely pubescent to glabrous. Leaves: petiole 3–7 mm long, 2–3 mm in diameter, sparsely pubescent to glabrous, slightly grooved, blade inserted on top of the petiole; blade 20–45 cm long, 7–18 cm wide, obovate to oblong-elliptic, apex rounded to abruptly acuminate, acumen 2–3 cm long, **base rounded to shortly attenuate generally covering part of the petiole**, subcoriaceous to coriaceous, below sparsely pubescent when young, glabrous when old, above glabrous when young and old, concolorous; midrib impressed, above glabrous when young and old, below sparsely pubescent when young, glabrous when old; secondary veins 10 to 20 pairs, glabrous above; tertiary venation reticulate, indistinct. **Individuals androdioecious; inflorescences cauliflorous or on old leafless branches, axillary; peduncle 0.3–4 m long, up to 2–3 cm in diameter towards the base, woody, hanging, sparsely pubescent to glabrous.** Flowers with 9 perianth parts in 3 whorls, 10 to 20 per inflorescence, male and female inflorescences similar; pedicel 10–85 mm long, 2–4 mm in diameter, glabrous; in fruit 25–100 mm long, 10–20 mm in diameter, woody, glabrous; bracts 2–4, several basal and one upper towards the middle of pedicel, basal bracts 7–12 mm long, 7–12 mm wide; upper bract 10–12 mm long, 12–25 mm wide, clasping the pedicel; sepals 3, valvate, basally fused, 15–22 mm long, 17–20 mm wide, triangular to ovate, apex acute, base truncate, green, tomentose outside, glabrous inside, margins flat; petals free, sub equal; outer petals 3, 25–50 mm long, 20–40 mm wide, elliptic to obovate, apex acute or rounded, base truncate, yellow to green, margins flat, pubescent outside, pubescent inside; **inner petals 3, valvate, 12–35 mm long, 15–25 mm wide**, elliptic, apex rounded, base attenuate to truncate, yellow-green, margins flat, pubescent outside, pubescent inside; stamens in male flowers: numerous on a conical receptacle, 4–4.5 mm long, oblong, connective truncate, sparsely pubescent, green to cream–yellow; stamens in hermaphrodite flowers: 65 to 100, in 3–4 rows, 2–2.5 mm long, oblong; connective truncate, sparsely pubescent, green to cream - yellow; staminodes absent; carpels free, 180–210, ovary 3–4 mm long, stigma capitate, glabrous. **Fruit pseudosyncarpous, 250–500 mm long, 100–300 mm in diameter**; individual monocarps 150 to 180, sessile, completely fused between them; apex shortly pyramidal, glabrous, smooth, glossy, yellow at maturity with white pulp; seed 1, 30–50 mm long, 17–30 mm in diameter, flattened to oblong; aril absent.

#### Distribution.

A central African species, from southeastern Nigeria to the Republic of Congo, and in the Democratic Republic of the Congo; in Cameroon known from the East, South, Central, Littoral, South-West and West regions.

#### Habitat.

A widespread and very common species across its range; in evergreen or semi-deciduous primary, old or young secondary, lowland or premontane rain forests. Altitude: 0–1600 m a.s.l.

#### Local and common names known in Cameroon.

mbé, nbwé, ombé (dial. Bagali, Baka), ébom, ében, ébon ntangan (dial. Ewondo, *Hochuli 4*).

#### IUCN conservation status.

Least Concern (LC) (Harvey-Brown 2019a).

#### Uses in Cameroon.

***Food***: fruit is eaten; ***medicine***: bark used against arthritis, rheumatism, stomach troubles, diarrho dysentery, menstrual cycle, antidotes, paralysis, epilepsy, convulsions, spasms; ***social***: religion, superstitions, magic.

#### Notes.

﻿﻿﻿*Anonidiummannii* is easily recognizable by its characteristic large leaf blades with a slightly cordate base covering part of the petiole, its long and woody inflorescences hanging from the trunk or old leafless branches and its syncarpous fruits up to 50 cm long and 30 cm large. It is morphologically close to ﻿*A.brieyi* but differs mainly by the wider inner and outer petals, and slightly smaller leaf blades and individuals being androdioecious (see notes under ﻿*A.brieyi*).

#### Selected specimens examined.

**Central Region**: Eastern sector of M’fou Nat Park Footpath running E of bridge SE of Ndanan 1, 3.61°N, 11.58°E, *22 October 2002*, *Cheek M.* 11248 (K,YA); near Otele, 3.43°N, 11.14°E, *25 February 2007*, *Couvreur T.L.P.* 106 (WAG,YA); Mont Mbam Minkon on trail 3 km from Nkol Nyada village, 3.96°N, 11.40°E, *21 March 2013*, *Couvreur T.L.P.* 415 (WAG,YA); Ottotomo Forest Reserve on top of small hill in front of reserve house, 3.65°N, 11.29°E, *25 June 2013*, *Couvreur T.L.P.* 452 (WAG,YA); on trail to Oveng Lodge hotel near parking just behind the village of Oveng 30 km on road from Mbalmayo to Sangmeli 3.41°N, 11.70°E, *09 February 2014*, *Couvreur T.L.P.* 609 (WAG,YA); Ottotomo Forest reserve 7 km north-west from Ngoumou 30 km south west from Yaoundé, 3.65°N, 11.28°E, *24 February 2016*, *Couvreur T.L.P.* 987 (WAG,YA); SSW of M’Balmayo, 3.52°N, 11.5°E, *27 February 1964*, *de Wilde W.J.J.O* 1968 (B,B,BR,K,MO,P,WAG,YA); Mbam-Minkom Village de Nkolniada, 3.96°N, 11.40°E, *26 July 2012*, *Droissart V.* 1416 (MO); Nanga Eboko, 4.68°N, 12.36°E, *17 February 1927*, *Hédin L.* 36 (P); Mbam Minkom, 3.96°N, 11.36°E, *19 September 2013*, *Kamdem N.* 142 (YA); Nkila, 4.68°N, 12.37°E, *12 March 1959*, *Letouzey R.* 1547 (P,YA); Ekom, 3.85°N, 11.7°E, *16 February 1947*, *Letouzey R.* 194 (P). **East Region**: Toungrélo, 4.33°N, 13.53°E, *09 January 1962*, *Breteler F.J.* 2454 (K,P,WAG); 81 km south of Yokadouma 30 km after Ngato 15 km after river ALPICAM ‘base de vie’ then 40 km on forestry road starting 4 km before Maséa village, 3.17°N, 14.69°E, *05 March 2019*, *Couvreur T.L.P.* 1207 (MPU,WAG,YA); Somalomo, 3.32°N, 12.71°E, *18 March 2016*, *Kamdem N.* 415 (YA); Lomié, 3.11°N, 13.58°E, *01 December 2016*, *Kamdem N.* 454 (YA). **Littoral Region**: Nkam, 4.35°N, 10.67°E, *13 June 1927*, *Hédin L.* 1337 (P). **South Region**: Campo Ma’an National Park 11 km on trail from Ebinanemeyong village on road 7 km from Nyabessan to Campo town, 2.48°N, 10.33°E, *13 February 2015*, *Couvreur T.L.P.* 696 (WAG,YA); 17 km On the newly reconstructed road from Ebolowa to Minkok, 2.75°N, 11.25°E, *29 January 1975*, *de Wilde J.J.F.E* 7930 (BR,K,MO,P,U,WAG,YA); Massif de Ngovayang village de Atog Boga, 3.25°N, 10.49°E, *05 September 2015*, *Droissart V.* 2158 (BRLU); Essam, 4.68°N, 12.37°E, *13 February 1959*, *Letouzey R.* 1276 (P); Essam (Nanga Eboko), 4.68°N, 12.37°E, *14 February 1959*, *Letouzey R.* 1385 (P); Campo-Ma’an area Nsengou, 2.18°N, 10.58°E, *05 February 2001*, *Tchouto Mbatchou G.P.* 3129 (KRIBI,WAG). **South-West Region**: Colline de Bokwa 42 km SE Mamfe, 5.71°N, 9.643°E, *07 December 1986*, *Achoundong G.* 1325 (YA); Nyasoso, 4.86°N, 9.7°E, *03 June 1996*, *Cable S.* 2801 (K,YA); Nature trail, 4.81°N, 9.683°E, *15 January 1995*, *Cheek M.* 6009 (K,WAG); Kupe Mount, 4.82°N, 9.683°E, *20 November 1995*, *Cheek M.* 7896 (K,WAG,YA); Nyasoso village at base of My Kupe forest reserve along nature trail, 4.82°N, 9.686°E, *04 April 2016*, *Couvreur T.L.P.* 1053b (WAG,YA); Cameroon Mountain, 4.08°N, 9.1°E, *29 December 1983*, *Thomas D.W.* 2850 (MO,WAG). **West Region**: Près Bandounga à 40 km au NW de Ndikinimeki, 4.98°N, 10.55°E, *12 February 1972*, *Letouzey R.* 11202 (P,YA).

### ﻿﻿ Artabotrys

Taxon classificationPlantaeMagnolialesAnnonaceae

R.Br., Bot. Reg. 5: 423, 1820

A7A55353-C9BB-5496-ADAD-271F4A880D38

= Ropalopetalum Griff. Not. Pl. Asiat. 4: 716, 1854.

#### Description.

Lianas, up to 30 m tall, d.b.h. up to 20 cm; stilt roots or buttresses absent. Indumentum of simple hairs or absent. Leaves: petiole 1–15 mm long, 1–2 mm in diameter; blade 7–26 cm long, 2.5–14 cm wide, elliptic to ovate to obovate to oblong, apex acuminate to acute, base decurrent to subcordate, concolorous; midrib sunken or flat; secondary veins 7 to 16 pairs; tertiary venation reticulate. Individuals bisexual; inflorescences ramiflorous on old or young foliate branches, leaf opposed or extra axillary. Flowers with 9 perianth parts in 3 whorls, 1 to 90 per inflorescence; pedicel 2–25 mm long; in fruit 2–25 mm long; bracts 2, all basal, minute, soon falling; sepals 3, valvate, free, 1–15 mm long, triangular, apex acute, base truncate; petals free, sub equal; outer petals 3, valvate, 5–35 mm long, 1–14 mm wide, ovate to elliptic to linear to tubular, apex acute to rounded, base broad and concave; inner petals 3, valvate, 5–30 mm long, 1–9 mm wide, ovate to elliptic to linear to tubular, apex acute to rounded, base broad and concave, forming a pollination chamber over the receptacle; stamens 15 to 70, in 2 to 5 rows, 2–3 mm long, linear or cuneiform; connective discoid, glabrous or pubescent; staminodes absent; carpels free, 3 to 32, ovary 1–4 mm long, stigma bilobed or cylindrical, pubescent or glabrous. Monocarps sessile or substipitate, stipe, when present 1–25 mm long, 1 to 20 monocarps, 6–60 mm long, 5–25 mm in diameter, ellipsoid to obovoid, apex rounded to apiculate, smooth or verrucose; seed 1 to 2, 5–25 mm long, 5–15 mm in diameter, ellipsoid or flattened ellipsoid; aril absent.

#### Type species.

﻿*Artabotrysodoratissimus* R.Br., *nom. illegit.* (≡ ﻿*Annonahexapetala L.f.*, ≡ ﻿*Artabotryshexapetalus* (L.f.) Bhandari).

A genus of lianas with around 105 species distributed across the paleotropics in South East Asia, Australia, Madagascar and Africa (Chen et al. 2019); eight species occur in Cameroon, one endemic.

Genus easily identifiable by its lianescent habit with the presence of characteristic inflorescences in form of a hook (the peduncle) and flowers that have a broad and concave base.

#### Taxonomy.

To date there are no taxonomic revisions for ﻿*Artabotrys* in Africa, but see Le Thomas (1969b), Boutique (1951b) and Paiva (1966).

### ﻿Key to the species of ﻿*Artabotrys* in Cameroon

**Table:** 

1	Upper side of midrib glabrous, or pubescent just at the basal part, never densely pubescent	**2**
–	Upper side of midrib densely pubescent	﻿***A.thomsonii***
2	Young foliate branches and petioles glabrous or sparsely pubescent	**3**
–	Young foliate branches and petioles densely pubescent to tomentose	**7**
3	Petioles 10–15 mm long	﻿***A.congolensis***
–	Petioles less than 8 mm long	**4**
4	Leaves 10–20 cm m; sepals 10–15 mm long and 5–8 mm wide, apex of monocarps clearly apiculate, apicule curved	﻿**A.insignisvar.insignis**
–	Leaves smaller than 13 cm; sepals < 5 mm long and < 3 mm wide, apex of monocarps rounded	**5**
5	Flowering pedicels 10–25 mm long; sepals minute, ca. 1 mm long and ca. 1 mm wide, petals linear, 1–2 mm wide above the broad base, pubescent; monocarps 20–40 mm 10–20 mm in diameter, warty to verrucose, faintly ribbed	﻿***A.jacquesfelicis***
–	Flowering pedicels 7–10 mm, sepals 3–5 mm long 2–3 mm wide, petals elliptic to ovate, 4–9 mm wide above the broad base, tomentose; monocarps 15–20 mm 7–13 mm in diameter, smooth, not ribbed	**6**
6	Inflorescence pauciflorous, 1 to 4 flowers	﻿**A.aurantiacusvar.aurantiacus**
–	Inflorescence multiflorous, 6 to 15 flowers	﻿﻿**A.aurantiacusvar.multiflorus**
7	Young foliate branches and petioles hirsute with long erect hairs	﻿***A.rufus***
–	Young foliate branches densely pubescent with appressed or shortly erect hairs	**8**
8	Lower side of leaf blade densely pubescent brown, base of leaves subcordate with the leaf base inserted on top of petiole, secondary veins 13 to16 pairs, inflorescences multiflorous, > 15 flowers, generally on leafless branches	﻿***A.dielsiana***
–	Lower side of leaf blade sparsely pubescent to glabrous, base of leaves decurrent to acute with the leaf base inserted on the side of petiole, secondary veins 8 to 12 pairs, inflorescences pauciflorous, < 10 flowers, generally on leafy branches	**9**
9	Leaves 10–20 cm, leaf base acute, sepals 10–15 mm long and 5–8 m wide, petals 30–35 mm long, 7–12 mm wide, elliptic, not tubular	﻿﻿**A.insignisvar.batesii**
–	Leaves 8–12 cm, leaf base usually decurrent (but can also be acute), sepals 2–3 mm long and 2–3 m wide, petals 5–15 mm long, 1–2 mm wide, linear, tubular in shape	﻿***A.velutinus***

### ﻿﻿﻿﻿ Artabotrys aurantiacus

Taxon classificationPlantaeMagnolialesAnnonaceae

Engl. & Diels, Notizbl. Königl. Bot. Gart. Berlin 2: 300, 1899

EABF3B9E-AF5C-5090-9481-0A24FA415A96

Fig. 11 ; Map 2A

= Artabotrys pynaertii De Wild., Ann. Mus. Congo Belge, Bot. sér. 5, 3(1): 78, 1909. Type. Democratic Republic of the Congo. Equateur, Eala, Pynaert L.A. 606, 15 Oct 1906: lectotype, sheet here designated: BR[BR0000008809971]; isotypes: BR[BR0000008809964, BR0000008809988]; S[S07-13416].
= Artabotrys claessensii De Wild., Bull. Jard. Bot. État Brux. 3: 262, 1911. Type. Democratic Republic of the Congo, Orientale, Yangambi, Claessen, *J. 725*, Jul 1910: lectotype, sheet here designated: BR[BR0000008809995]; isotype: BR[BR0000008809988].

#### Type.

Cameroon. Central Region; Yaoundé, *Zenker G.A. 690*, 1896: holotype: B[B 10 0153007]; isotypes: BM[BM000546848]; COI[COI00004927]; P[P00363375, P00363376]; K[K000198859, K000198860].

#### Description.

Liana, height unknown, d.b.h. unknown. Indumentum of simple hairs; old leafless branches glabrous, **young foliate branches sparsely pubescent with short appressed hairs.** Leaves: petiole 3–5 mm long, 1–2 mm in diameter, pubescent with short appressed hairs to glabrous, grooved, blade inserted on the side of the petiole; **blade 7.5–10 cm long, 2.5–6 cm wide, oblong to elliptic**, apex acuminate, acumen 0.5–1 cm long, **base cuneate to rounded**, coriaceous to subcoriaceous, below sparsely pubescent with short appressed hairs to glabrous when young, glabrous when old, above glabrous when young and old, concolorous; midrib sunken or flat, above glabrous when young and old, below pubescent with short appressed hairs when young and old; secondary veins 10 to 12 pairs, glabrous above; tertiary venation reticulate. Individuals bisexual; inflorescences ramiflorous on old leafless branches, leaf opposed. Flowers with 9 perianth parts in 3 whorls, 1 to 4 per inflorescence; hook-shaped peduncle 15–20 mm long, sparsely pubescent; pedicel 7–10 mm long, 1–2 mm in diameter, sparsely pubescent; in fruit 7–13 mm long, 2–3 mm in diameter, glabrous; bracts all basal, minute; sepals 3, valvate, free, 3–5 mm long, 2–3 mm wide, triangular, apex acute, base truncate, green, pubescent outside, glabrous inside, margins flat; petals free, sub equal, green turning red-orange; outer petals 3, 15–30 mm long, 4–9 mm wide, **narrowly elliptic to narrowly ovate**, apex attenuate, base broad and concave, white to light green, margins flat, tomentose outside, tomentose with a glabrous base inside; inner petals 3, valvate, 15–30 mm long, 3–6 mm wide, linear to narrowly elliptic, apex acute, base broad and concave, white to light green, margins flat, tomentose outside, tomentose with a glabrous base inside; stamens 15 to 20, in 2 to 3 rows, 2–3 mm long, cuneiform; connective discoid, glabrous; staminodes absent; carpels free, 8 to 10, ovary ca. 2 mm long, stigma cylindrical, glabrous. Monocarps sessile, 4 to 6, 15–20 mm long, 7–13 mm in diameter, ellipsoid to oblong, **apex rounded, glabrous, smooth, red when ripe, not ribbed**; seeds 1 to 2 per monocarp, 10–13 mm long, 5–8 mm in diameter, flattened ellipsoid; aril absent.

#### Distribution.

A central African species, from Cameroon to the Republic of Congo and the Democratic Republic of the Congo; in Cameroon known from the Central, East, Littoral, South, South-West and West regions.

#### Habitat.

A common species across its range; in sub montane (sometimes lowland) secondary or primary rain forests. Altitude (100)500–1600 m a.s.l.

#### Local and common names known in Cameroon.

None recorded.

#### IUCN conservation status.

Not evaluated.

#### Uses in Cameroon.

None recorded.

#### Notes.

﻿﻿﻿*Artabotrysaurantiacus* is distinguished by its sparsely pubescent to glabrous branches and leaf blades (upper and lower sides), with leaves that are relatively small but wide (less than 10 cm and up to 6 cm wide) and oblong to elliptic in shape with a cuneate to rounded base. The flowers have narrowly elliptic petals and the monocarps are smooth, ellipsoid with a rounded apex.

#### Specimens examined.

**Central Region**: Badjob, 3.68°N, 10.68°E, *21 December 1963*, *de Wilde W.J.J.O* 1602 (BR,MO,P,WAG,YA); Bank Nyong River near the new bridge ca 65 km SSW of Eséka, 3.46°N, 10.5°E, *17 June 1964*, *de Wilde W.J.J.O* 2720 (WAG); Yaoundé, 3.87°N, 11.52°E, *1896*, *Zenker G.A.* 690 (B,K,P). **East Region**: Ebaka (Bertoua), 4.93°N, 13.32°E, *24 May 1961*, *Breteler F.J.* 1429 (BR,K,M,P,WAG,YA); Ndo Riv (Bertoua), 4.58°N, 13.68°E, *12 December 1961*, *Breteler F.J.* 2210 (BR,K,P,WAG,YA); Doumé Riv (Batouri), 4.23°N, 13.45°E, *15 April 1962*, *Breteler F.J.* 2799 (K,P,WAG); Goyoum, 5.22°N, 13.38°E, *29 January 1961*, *Breteler F.J.* 968 (A,BR,K,M,P,WAG); Nguélémendouka, 4.38°N, 12.92°E, *04 April 1962*, *de Bruijn J.* s.n. (WAG[WAG0175010]); Rives du Dja près Ndongo à 40 km WNW de Moloundou, 2.15°N, 14.86°E, *18 March 1973*, *Letouzey R.* 12141 (P,WAG,YA); Berge arbustive et broussailleuse du fleuve Sanaga au Nord de Goyoum, 5.24°N, 13.36°E, *29 January 1961*, *Letouzey R.* 3309 (P,YA); Rives du Dja entre les rivières Meu et Edjune, 3.41°N, 13.33°E, *12 April 1961*, *Letouzey R.* 3772 (P,YA); Rives de la Kadei entre Mindourou et Dongongo (40 km SSE de Batouri), 4.13°N, 14.60°E, *25 April 1962*, *Letouzey R.* 4859 (P,YA); Betare Oya, 5.59°N, 14.08°E, *Tisserant C.* 3651 (P). **Littoral Region**: Manengouba mount base 4 km WNW Of Nkongsamba, 4.96°N, 9.883°E, *09 September 1971*, *Leeuwenberg A.J.M.* 8319 (B,BR,MO,P,U,WAG,YA). **South Region**: Ebom, 3.1°N, 10.73°E, *13 August 1996*, *Elad M.* 510 (WAG); Nyabesan, 2.4°N, 10.4°E, *05 March 1963*, *Raynal J.* 10240 (P,YA). **South-West Region**: Likombe, 4.11°N, 9.183°E, *19 February 1995*, *Cable S.* 1309 (K,MO,WAG,YA); Likombe, 4.11°N, 9.183°E, *19 February 1995*, *Cable S.* 1310 (K,WAG,YA); Road to NLO Mt from Kodmin, 5°N, 9.683°E, *23 January 1998*, *Cheek M.* 9063 (K,YA); Nyasoso, 4.81°N, 9.683°E, *08 February 1995*, *Elad M.* 132 (K,YA); Mt Cameroon south slope Transect 8, 4.07°N, 9.015°E, *16 November 1985*, *Gentry A.H.* 52942 (MO,P); Nzee Mbeng trail from Ngomin to Nzee Mbeng, 5.83°N, 9.716°E, *10 February 1998*, *Gosline W.G.* 99 (K,YA); Ndum, 4.83°N, 9.7°E, *31 January 1995*, *Groves M.* 21 (K,MO,WAG,YA); Nyasoso, 4.81°N, 9.683°E, *08 February 1995*, *Groves M.* 77 (K,YA); South slope of mount north of Mt Etinde Forest, 4.08°N, 9.133°E, *20 March 1988*, *Nemba J.* 953 (MO,P). **West Region**: Bali Ngemba grassland and forest patches northeast of Mantum, 5.82°N, 10.08°E, *12 April 2004*, *Etuge M.* 5373 (K,MO,P,WAG,YA); Dschang, 5.45°N, 9.95°E, *01 May 1960*, *Jacques-Félix H.* 5211 (K,P,WAG); Ngwenfon 35 km NW de Foumban, 5.72°N, 10.92°E, *11 December 1974*, *Letouzey R.* 13495 (P,YA).

**Figure 11.**
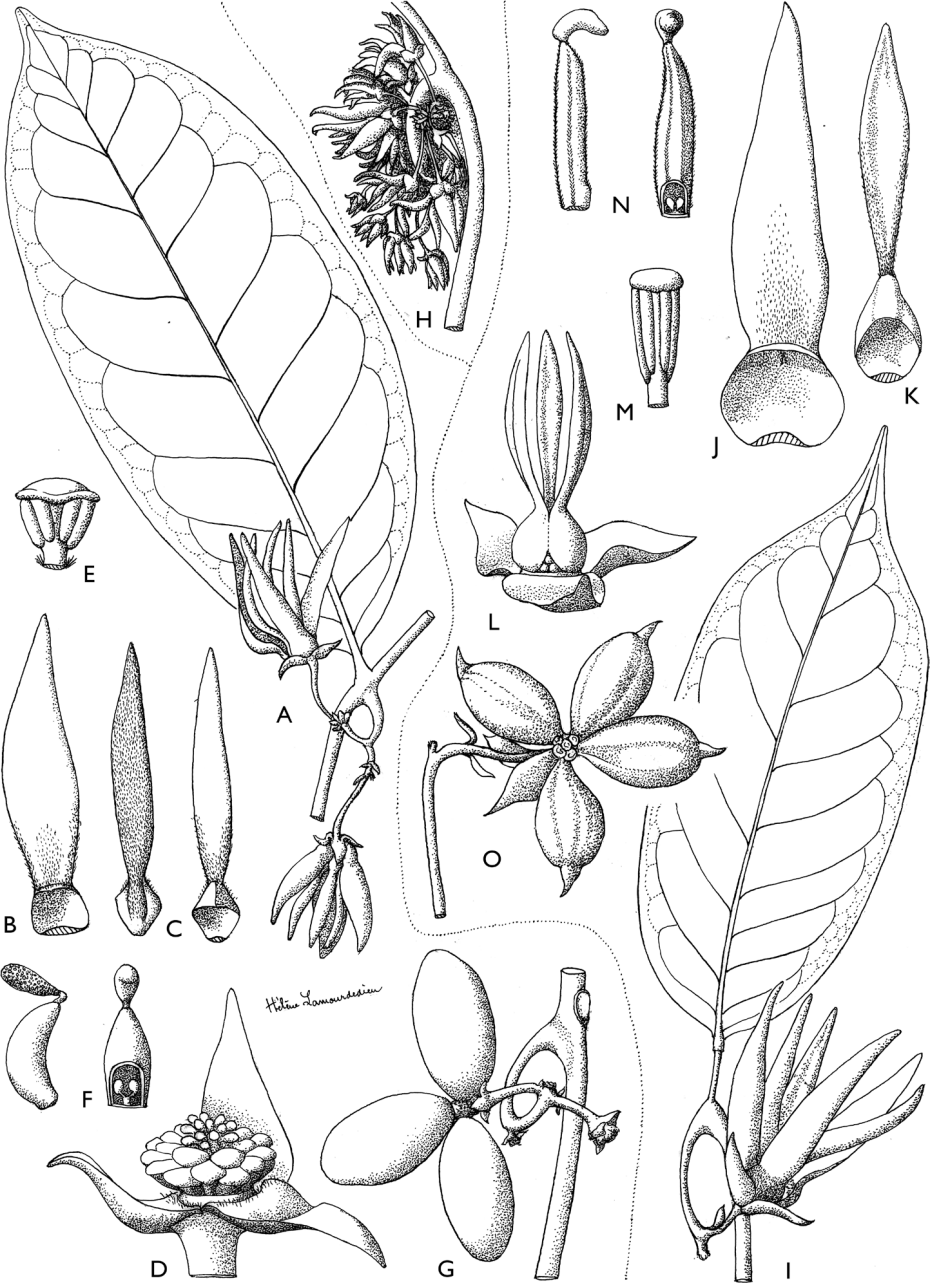
Artabotrysaurantiacusvar.aurantiacus**A** flowering branch **B** outer petal, inner view **C** inner petal, outer and inner views **D** flowering receptacle with petals removed **E** stamen **F** carpel, side view and detail of basal ovules **G** fruiting branch. Var.multiflorus**H** flowering branch, note the numerous flowers. Artabotrysinsignisvar.insignis**I** flowering branch **J** outer petal, inner view **K** inner petal, inner view **L** flower, whole **M** stamen **N** carpel, side view, and detail of ovules **O** fruiting branch, note long apiculate apex of monocarps **A–F** from *Le Testu 8499***G** from *Le Testu 4430***H** from *Le Testu 7116***I–N** from *Le Testu 8674*; 15 from *Berteler 2959*. Drawings by Hélène Lamourdedieu, Publications Scientifiques du Muséum national d’Histoire naturelle, Paris; modified from Le Thomas (1969b; pl. 26, p. 143).

### ﻿﻿﻿﻿ Artabotrys aurantiacus

Taxon classificationPlantaeMagnolialesAnnonaceae

Engl. & Diels var. multiflorus Pellegr. ex Le Thomas, Adansonia, ser. 2, 5: 447, 1965

BED090BA-A01F-55B2-B55A-35EEACA6A2EC

Fig. 11 ; Map 2B

#### Type.

Gabon. Ogooué-Lolo; Lastoursville, *Le Testu G.M.P.C. 7116*, Mar 1929: lectotype, sheet here designated: P[P02034091]; isotypes: BR[BR0000008820792, BR0000008809940, BR0000008809933]; IFAN[IFAN01625]; LISC[LISC000367]; P[P02034088]; K[K000198858].

#### Description.

Differs from the type variety by the presence of numerous densely packed flowers (6–15 versus 1–4).

#### Distribution.

A central African species, from Cameroon to the Republic of Congo and the Democratic Republic of the Congo, the *multiflorus* variety is known from Cameroon and Gabon; in Cameroon known from the Central, East, Littoral, South-West and West regions.

#### Habitat.

A rare variety; in sub montane (sometimes lowland) secondary or primary rain forests. Altitude 700–1600 m a.s.l.

#### Local and common names known in Cameroon.

None recorded.

#### IUCN conservation status.

Not evaluated.

#### Uses in Cameroon.

None recorded.

**Map 2.**
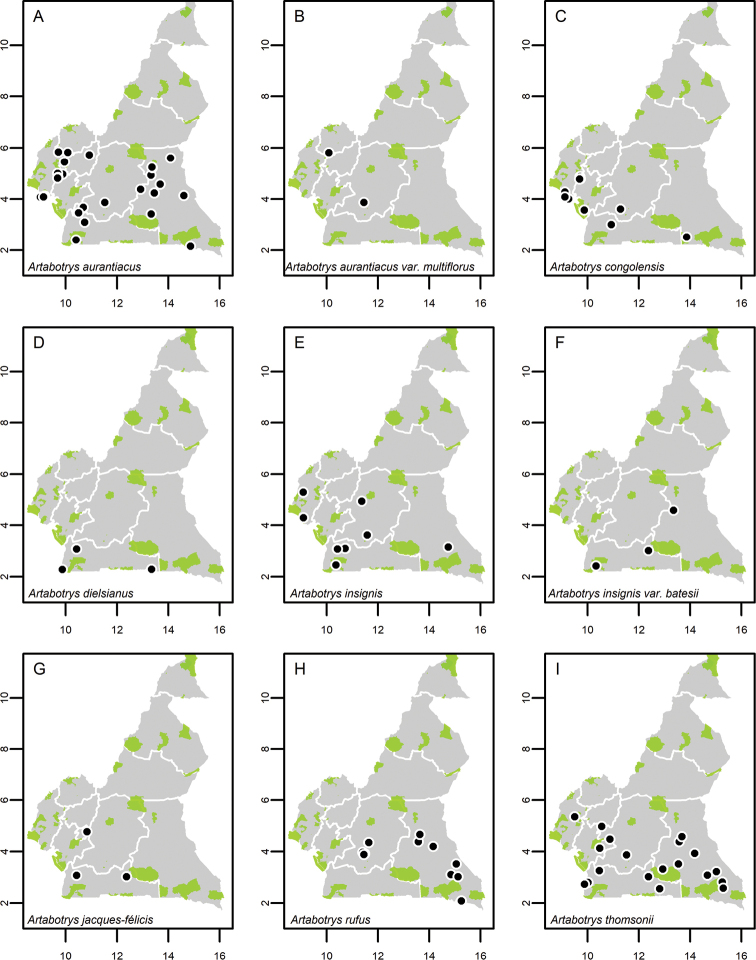
**A***Artabotrysaurantiacus***B**Artabotrysaurantiacusvar.multiflorus**C***Artabotryscongolensis***D***Artabotrysdielsianus***E***Artabotrysinsignis***F**Artabotrysinsignisvar.batesii**G***Artabotrys jacques-félicis***H***Artabotrysrufus***I***Artabotrysthomsonii*. White borders represent region limits in Cameroon; green patches represent protected areas (see methods and Suppl. material 1: Fig. S1).

#### Notes.

The variety status is doubtful, but without further investigation, we shall follow Le Thomas (1969b). The main difference between the two varieties is the number of flowers per inflorescence (see above). Le Thomas (1969b) also mentions that var. multiflorus has smaller flowers (smaller than 13 mm) and pedicels of 1 cm long. However, our measurements were not able to confirm these two latter differences.

#### Specimens examined.

**Central Region**: Près Yaoundé, 3.86°N, 11.45°E, *11 March 1981*, *Meijer D.* 15033 (MO,WAG). **West Region**: Bali- Ngemba FR, 5.81°N, 10.08°E, *13 April 2002*, *Onana J.M.* 2027 (K,WAG,YA).

### ﻿﻿﻿﻿ Artabotrys congolensis

Taxon classificationPlantaeMagnolialesAnnonaceae

De Wild. & T. Durand, Ann. Mus. Congo Belge, Bot. Sér. 2, 1(1): 2, 1899

536EAFDD-706C-5D40-BD27-D1AB7E3785D0

Fig. 12 ; Map 2C

= Artabotrys rhopalocarpus Le Thomas, Adansonia sér. 2, 6: 591, 1966. Type. Central African Republic: Lobaye, Boukoko, *Tisserant C. 2242*, 25 Sep 1951: lectotype, sheet here designated: P[P00364752]; isotypes: BM[BM000546867]; BR[BR0000008820822]; LISC[LISC000373]; P[P00363392, P00363390]; WAG[WAG0392422].

#### Type.

Democratic Republic of the Congo. Equateur; Lukolela, *Dewèvre A.P. 819*, 31 Mar 1896: holotype: BR[BR0000008820808]; isotype: B[B 10 0153012].

#### Description.

Liana, height unknown, d.b.h. ca. 8 cm. Indumentum of simple hairs; **old leafless branches glabrous, young foliate branches glabrous**. Leaves: petiole 10–15 mm long, ca. 2 mm in diameter, glabrous, grooved, blade inserted on the side of the petiole; blade 8–26 cm long, 4–11 cm wide, elliptic to obovate, apex acute to acuminate, acumen 0.5–1 cm long, base acute to decurrent, coriaceous, below glabrous when young and old, above glabrous when young and old, concolorous; midrib impressed, above glabrous when young and old, below glabrous when young and old; secondary veins 8 to 13 pairs, glabrous above; tertiary venation reticulate. Individuals bisexual; inflorescences ramiflorous on old leafless branches, leaf opposed. Flowers with 9 perianth parts in 3 whorls, 1 to 3 per inflorescence, hook-shaped peduncle 10–15 mm long; pedicel 10–25 mm long, 2–3 mm in diameter, glabrous; in fruit 12–20 mm long, 2–3 mm in diameter, glabrous; bracts 1 to 2, all basal, basal bracts 2–3 mm long, 1–2 mm wide; sepals 3, valvate, free, 5–8 mm long, 3–7 mm wide, triangular, apex acute, base truncate, pubescent outside, glabrous inside, margins flat; petals free, inner shorter than outer, green turning yellow; **outer petals 3, 15–25 mm long**, **8–14 mm wide**, ovate, apex acute, base broad and concave, margins flat, tomentose outside, tomentose with a glabrous base inside; inner petals 3, valvate, 8–17 mm long, 5–8 mm wide, elliptic to rhombic, apex acute, base broad and concave forming a chamber over the receptacle, margins flat, tomentose outside, tomentose with a glabrous base inside; stamens numerous, number of rows unknown, 2–3 mm long, oblong; connective discoid, glabrous; staminodes absent; carpels free, 15 to 20, ovary 3–4 mm long, stigma tubular, sparsely pubescent. Monocarps **stipitate, stipes 1–2 mm long, 1–3 mm in diameter, but gradually widening into seed-bearing part; monocarps 15 to 20, to 60 mm long**, ca. 25 mm in diameter, obovoid, apex acute to rounded, **glabrous**, smooth, glossy, green when ripe; seeds 1 to 2 per monocarp, 19–22 mm long, 10–15 mm in diameter, ellipsoid, laterally flattened; aril absent.

**Figure 12.**
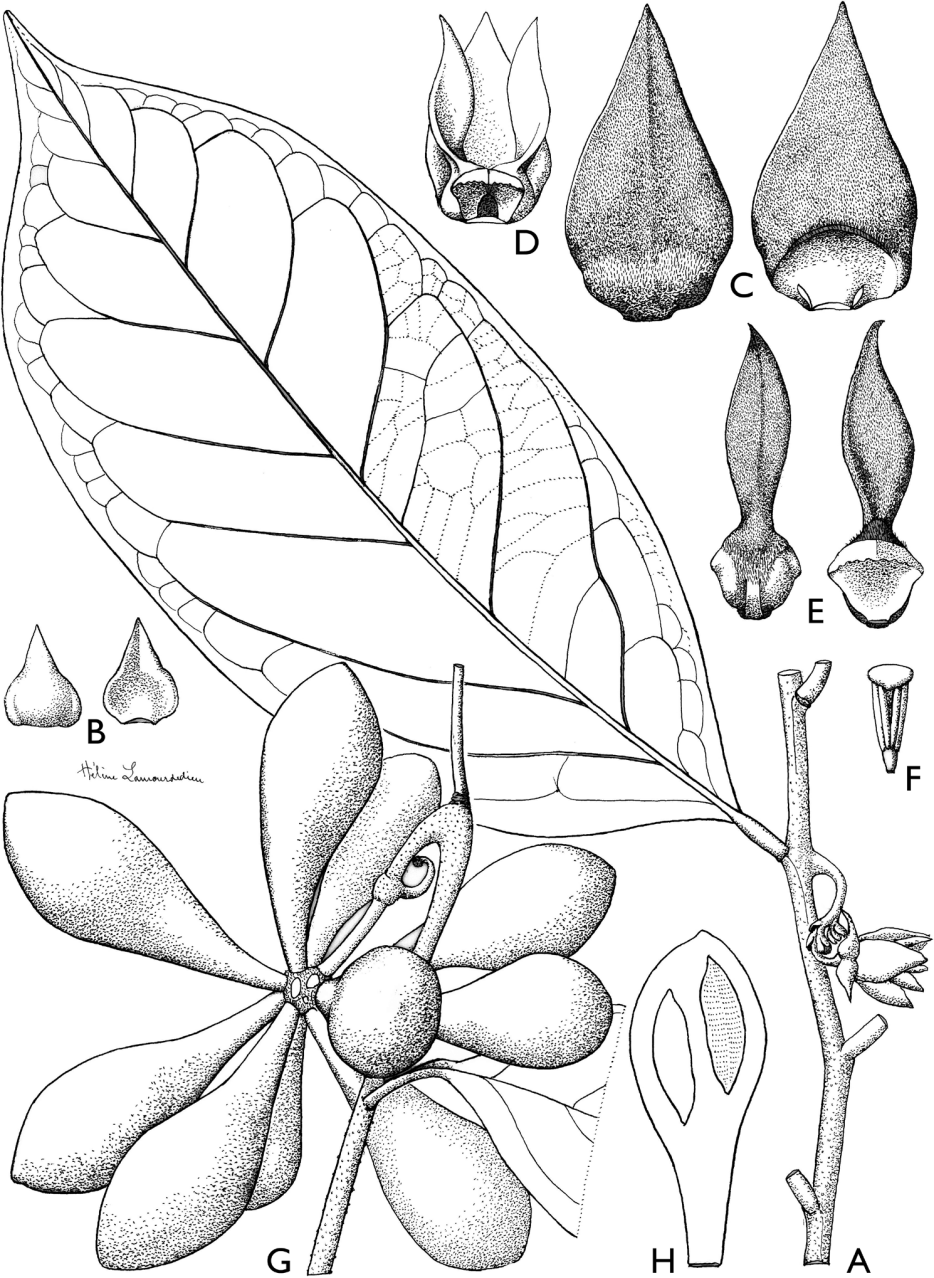
*Artabotryscongolensis***A** flowering branch **B** sepals outer and inner views **C** outer petals, outer and inner views **D** side view of outer and inner petals showing the pollination chamber formed by the inner petals **E** inner petals, outer and inner views **F** stamen **G** fruit, notice the stipes gradually widening into the seed bearing part of the monocarps **H** longitudinal section of a monocarp showing 2 seeds **A–F** from *Tisserant 2242***G, H** from *Hallé 3451*. Drawings by Hélène Lamourdedieu, Publications Scientifiques du Muséum national d’Histoire naturelle, Paris; modified from Le Thomas (1967c).

#### Distribution.

A central African species, from Cameroon to the Republic of Congo and in the Democratic Republic of the Congo; in Cameroon known from the Central, East, Littoral, South and South-West regions.

#### Habitat.

A common species; in lowland or premontane secondary or primary rain forests. Altitude 400–1000 m a.s.l.

#### Local and common names known in Cameroon.

None recorded.

#### IUCN conservation status.

Not evaluated.

#### Uses in Cameroon.

None recorded.

#### Notes.

﻿﻿﻿*Artabotryscongolensis* is distinguished by its completely glabrous branches, petioles and leaf blades, its outer petals that are broad and the inner petals much narrower and its sessile fruits with a stipes gradually widening into the seed-bearing part of the monocarp.

Le Thomas described ﻿﻿*Artabotrysrhopalocarpus* (Le Thomas 1967c) which was later synonymized with ﻿*A.congolensis* (Bamps and Le Thomas 1989).

#### Specimens examined.

**Central Region**: near Ebolbom village 4 km est of Ngoumou 3 km north west of Otélé, 3.60°N, 11.28°E, *02 May 2013*, *Couvreur T.L.P.* 431 (MPU,WAG,YA). **East Region**: Sur axe Lomié-Ngoila-Souanké à 15 km au SSW de Ngola, 2.51°N, 13.86°E, *22 February 1973*, *Letouzey R.* 12016 (P,YA). **Littoral Region**: Tissongo, 3.57°N, 9.869°E, *09 July 1976*, *McKey D.B.* 111 (K). **South Region**: Ebolowa-Yaoundé, 3.00°N, 10.92°E, *12 January 1914*, *Mildbraed G.W.J.* 7727 (B). **South-West Region**: Nyasoso, 4.86°N, 9.7°E, *04 June 1996*, *Cable S.* 2851 (K,YA); Along path from village Mt Etinde summit, 4.05°N, 9.15°E, *02 December 1993*, *Cable S.* 332 (K); Kupe Rock saddle, 4.78°N, 9.716°E, *11 July 1996*, *Cable S.* 3804 (K,YA); Kupe village, 4.78°N, 9.716°E, *17 July 1996*, *Cable S.* 3894 (K,YA); Upper Boando, 4.05°N, 9.153°E, *08 December 1993*, *Cable S.* 475 (K,YA); Mabeta Moliwe reserve 3–5 km east of Limbe, 4.00°N, 9.256°E, *03 July 1992*, *Cheek M.* 3470 (P); Kupe village, 4.77°N, 9.688°E, *29 November 1999*, *Gosline W.G.* 241 (K); Pente E Mont 6 km E Bomana 35 km NW Limbé Alt 950 m, 4.27°N, 9.112°E, *11 December 1984*, *Villiers J.-F.* 2441 (P,YA); Saddle between Mt Etinde and Cameroon, 4.08°N, 9.116°E, *28 October 1992*, *Wheatley J.I.* 644 (K,YA).

### ﻿﻿ Artabotrys dielsianus

Taxon classificationPlantaeMagnolialesAnnonaceae

Le Thomas, Adansonia sér. 2, 9: 442, 1969

7F755F23-B30B-5AAA-A654-327027DA0C4F

**Figure 13.**
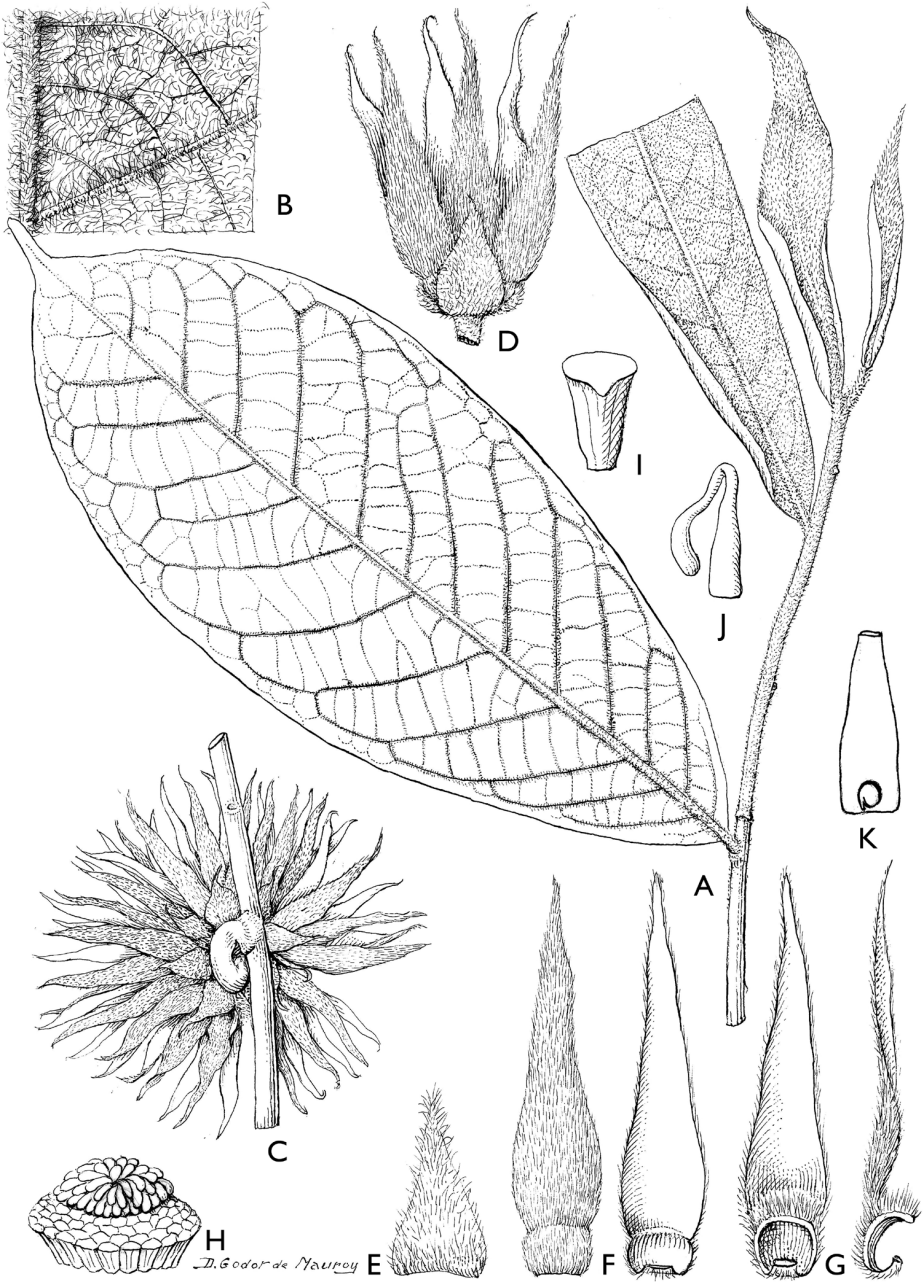
*Artabotrysdielsiana***A** branch **B** detail of the pubescence on the lower side of leaf blade **C** Inflorescence **D** flower **E** sepal, outside side **F** outer petals inner and outside view **G** inner petals inner and lateral side views **H** receptacle with stamens and carpels (stigmas showing) **A–H** from *Zenker 510*. Drawings D. Godor de Mauroy, Publications Scientifiques du Muséum national d’Histoire naturelle, Paris; modified from Le Thomas (1969a, pl. 1, p. 441).

**Figure 14.**
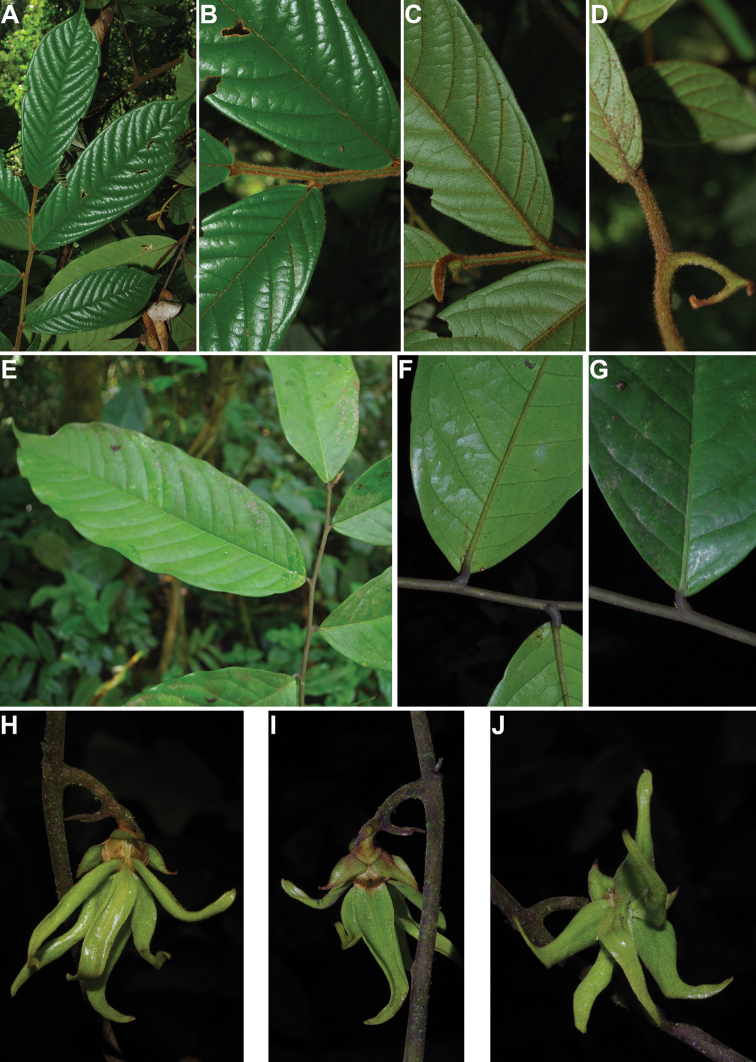
*Artabotrysdielsiana***A** branch **B** base of leaf blade; upper side **C** base of leaf blade; lower side **D** detail of hooked shaped inflorescence (sterile). Artabotrysinsignisvar.batesii**E** branch **F** base of leaf blade; lower side, note tomentose branches **G** base of leaf blade; upper side, note tomentose branches **H** flower, side view **I** flower, top view **J** flower, bottom view **A–D***Couvreur 453*, Lélé, Cameroon **E–J***Couvreur 1044*, Mt Cameroon, Cameroon. Photos Thomas L.P. Couvreur.

Figs 13 , 14 ; Map 2D

#### Type.

Cameroon. South Region; Bipindi, *Zenker G.A. 510*, 1 Jan 1914: lectotype, sheet here designated: P[P00363381]; isotypes: B[B 10 0153019]; BR[BR0000008820693]; P[P00363377]; U[U 0000237]; WAG[WAG0000084,WAG0000085].

#### Description.

Liana, height unknown, d.b.h. unknown. Indumentum of simple hairs; old leafless branches pubescent, **young foliate branches tomentose to densely pubescent with long, up to 2 mm, ferruginous hairs**. Leaves: petiole 2–3 mm long, 1–2 mm in diameter, densely pubescent, grooved, blade inserted on top of the petiole; blade 15–24 cm long, 6–7 cm wide, elliptic, apex acuminate to acute, acumen 0.5–1 cm long, **base subcordate**, coriaceous, below densely pubescent when young and old, above sparsely pubescent when young, glabrous when old, concolorous; midrib impressed, **above glabrous or sparsely pubescent when young, glabrous when old**, below densely pubescent when young and old; secondary veins 13 to 16 pairs, glabrous above; tertiary venation reticulate. Individuals bisexual; inflorescences, ramiflorous on old leafless branches, generally without leaves, leaf opposed. Flowers with 9 perianth parts in 3 whorls, **10 to 15 per inflorescence**, hook-shaped peduncle 12–17 mm long; **pedicel 3–4 mm long**, ca. 1 mm in diameter, densely pubescent; bracts ca. 2, all basal, basal bracts 5 mm long, 4 mm wide; sepals 3, valvate, free, 5–12 mm long, 3–7 mm wide, triangular, apex acute, base truncate, densely pubescent outside, glabrous inside, margins flat; petals free, sub equal; outer petals 3, 20–35 mm long, 6–10 mm wide, elliptic, apex acute, base broad and concave, margins flat, densely pubescent outside, glabrous inside; inner petals 3, valvate, 15–25 mm long, 6–9 mm wide, elliptic, apex acute, base broad and concave, margins flat, densely pubescent outside, pubescent inside; stamens 50 to 70, in 3 to 4 rows, 1–2 mm long, cuneiform; connective discoid, pubescent; staminodes absent; carpels free, 25 to 32, ovary 1–2 mm long, stigma cylindrical, glabrous. Fruits unknown.

#### Distribution.

endemic to Cameroon; known from the South region.

#### Habitat.

Rare species; in primary lowland rain forest. Altitude 0–200 m a.s.l.

#### Local and common names known in Cameroon.

None recorded.

#### IUCN conservation status.

Not evaluated.

#### Uses in Cameroon.

None recorded.

#### Notes.

﻿﻿﻿*Artabotrysdielsiana* is characterized by its densely pubescent young foliate branches with longish ferruginous hairs, leaf blades with a subcordate leaf base and pubescent below but glabrous above (including the midrib), and tightly packed flowers mainly borne on leafless branches (Le Thomas 1969a). This species has only been collected four times.

#### Specimens examined.

**South Region**: Lélé, 2.29°N, 13.34°E, *06 September 2013*, *Couvreur T.L.P.* 453 (WAG,YA); Campo-Ma’an area 2.28°N, 9.866°E, *03 October 2001*, *van Andel T.R.* 4128 (KRIBI,U,WAG); Bipindi, 3.08°N, 10.41°E, *01 January 1914*, *Zenker G.A.* 2087 (WAG); Bipindi, 3.08°N, 10.42°E, *01 January 1914*, *Zenker G.A.* 510 (B,P,U,WAG).

### ﻿﻿﻿﻿ Artabotrys insignis

Taxon classificationPlantaeMagnolialesAnnonaceae

Engl. & Diels, Bot. Jahrb. Syst. 34: 483, 1907

4C73664D-97BB-5E60-A180-CD6E2F8AD358

Fig. 11 ; Map 2E

= Artabotrys malchairi De Wild., Etudes Fl. Bangala & Ubangi: 312, 1911. Type. Democratic Republic of the Congo. Equateur, Environ de Likimi, Malchair L. 282, 20 Apr 1910: lectotype, sheet here designated: BR[BR0000014480478]; isotype: BR[BR0000014480461].
= Artabotrys insignis var. latifolius Pellegr., Bull. Soc. Bot. France 94: 256, 1947. Type. Gabon. Ogooué-Lolo, région de Lastoursville, Moughimba, *Le Testu G.M.P.C. 8474*; 27 Oct 1930: lectotype, here designated: P[P01954179]; isolectotype: BM[BM000546856].
= Artabotrys lucidus A. Chev.; Expl. Bot. Afr. Occ. Franc., 1: 9, 1920, nom. nud.

#### Type.

Cameroon. South Region; Bipindi, *Zenker G.A. 2801*, 1904: lectotype, sheet here designated: B[B 10 0153021]; isotypes: B[B 10 0153022]; COI[COI00004928]; GOET[GOET005674]; HBG[HBG502547]; K[K000198855]; MO[MO-216862]; P[P00363364]; S[S-G-7465]; WAG[WAG0053175]; WU[WU0025886].

#### Description.

Liana, up to 10 m tall, d.b.h. unknown. Indumentum of simple hairs; **old leafless branches glabrous, young foliate branches pubescent with short appressed hairs.** Leaves: petiole 2–3 mm long, 1–2 mm in diameter, **sparsely pubescent to glabrous**, grooved, blade inserted on the side of the petiole; blade 10–20 cm long, 3.5–6 cm wide, ovate to elliptic, apex acuminate to acute, acumen 0.5–1 cm long, base acute, subcoriaceous, below sparsely pubescent when young, glabrous when old, above glabrous when young and old, concolorous; midrib impressed, above sparsely pubescent to glabrous when young, glabrous when old, below sparsely pubescent to pubescent when young, sparsely pubescent when old; secondary veins 9 to 12 pairs, glabrous above; tertiary venation reticulate. Individuals bisexual; inflorescences ramiflorous on old leafless branches. Flowers with 9 perianth parts in 3 whorls, leaf opposed, **1 to 3 per inflorescence**, hook-shaped peduncle 16–25 mm long; pedicel 2–5 mm long, 1–2 mm in diameter, sparsely pubescent with short appressed hairs; in fruit 2–3 mm long, ca. 2 mm in diameter, glabrous; bracts 2(?), all basal, basal bracts not seen; sepals 3, valvate, free, **10–15 mm long, 5–8 mm wide, triangular**, apex acute, base truncate, green turning light reddish, pubescent outside, glabrous inside, margins flat; petals free, sub equal; outer petals 3, 30–35 mm long, 7–12 mm wide, elliptic, apex acute, base attenuate (rounded), green, margins flat but recurved outwards in vivo, pubescent to densely pubescent outside, pubescent to sparsely pubescent inside; inner petals 3, valvate, 15–25 mm long, 2–6 mm wide, elliptic to oblong, apex acute, base broad and concave, margins flat, but recurved outwards in vivo, densely pubescent to pubescent outside, pubescent to sparsely pubescent inside; stamens numerous, number of rows not seen, 2 mm long, cuneiform; connective discoid, glabrous; staminodes absent; carpels free, 12 to 17, ovary 3–4 mm long, stigma cylindrical, pubescent. **Monocarps sessile**, 7 to 9, 20–25 mm long, 10–12 mm in diameter, ellipsoid, **apex long apiculate and slightly curved, glabrous, smooth**, not ribbed; red when ripe, seeds 2 per monocarp, 8–11 mm long, 4–6 mm in diameter, flattened ellipsoid; aril absent.

#### Distribution.

A west and central African species, from Sierra Leone to Benin and from Cameroon to the Democratic Republic of the Congo; in Cameroon known from Central, East, South and South-West regions.

#### Habitat.

A fairly common species in Cameroon, in secondary rain forests a long fringes of forests, in swampy regions too. Altitude 100–800 m a.s.l.

#### Local and common names known in Cameroon.

None recorded.

#### IUCN conservation status.

Not evaluated.

#### Uses in Cameroon.

None recorded.

#### Notes.

﻿﻿﻿Artabotrysinsignisvar.insignis is distinguished by its overall relatively glabrous branches and petioles, long (> 10 cm) elliptic leaves with an acute leaf base (more rarely rounded), long (10–15 mm) triangular sepals, long and wide petals, and glabrous smooth fruits with a distinctive long curved apicule.

#### Specimens examined.

**Central Region**: Route Ndanan I-Ndangan I, 3.62°N, 11.58°E, *10 March 2004*, *Cheek M.* 11641 (K,YA); Yangafok II 25 km ENE de Bafia, 4.93°N, 11.37°E, *26 November 1969*, *Letouzey R.* 9607 (P,YA). **East Region**: 82 km south of Yokadouma 30 km after Ngato 15 km after river ALPICAM ‘base de vie’ then 40 km on forestry road starting 4 km before Maséa village, 3.15°N, 14.73°E, *06 March 2019*, *Couvreur T.L.P.* 1214 (MPU,WAG,YA). **South Region**: Campo Ma’an National Park 11 km on trail from Ebinanemeyong village on road 7 km from Nyabessan to Campo town, 2.46°N, 10.35°E, *14 February 2015*, *Couvreur T.L.P.* 710 (WAG,YA); Ebom, 3.1°N, 10.73°E, *25 February 1997*, *Elad M.* 580 (KRIBI,WAG); Ebom, 3.1°N, 10.71°E, *26 February 1997*, *Parren M.P.E.* 4 (KRIBI,WAG); Bipindi, 3.08°N, 10.41°E, *01 January 1904*, *Zenker G.A.* 2801 (B,BR,K,L,P,WAG); Bipindi, 3.08°N, 10.42°E, *01 January 1907*, *Zenker G.A.* 3320 (P). **South-West Region**: Mount Cameroon National Park on the Bomona trail behind Bomona village 10 km NW from Idenau, 4.29°N, 9.096°E, *03 April 2016*, *Couvreur T.L.P.* 1044 (MPU,WAG,YA); Korup National Park, 5.28°N, 9.083°E, *03 April 1988*, *Thomas D.W.* 7578 (MO,P).

### ﻿﻿﻿﻿﻿﻿ Artabotrys insignis

Taxon classificationPlantaeMagnolialesAnnonaceae

var. batesii Le Thomas Adansonia, ser. 2, 5: 448, 1965

4CF7E1C9-59BA-5690-9D45-CF1E4BDDFE39

Fig. 14 ; Map 2F

#### Type.

Cameroon. East Region; Bitya, near Dja river, *Bates G.L. 1792*, Sep 1922: holotype: P[00363370].

#### Description.

Differs from the type variety by its densely brown tomentose and shortly hirsute branches and petioles, pubescent lower side of leaf blades and tomentose petals.

#### Distribution.

A west and central African species, from Sierra Leone to Benin and from Cameroon to the Democratic Republic of the Congo; in Cameroon known from Central, East, South and South-West regions.

#### Habitat.

A fairly common species in Cameroon, in secondary rain forests a long fringes of forests, in swampy regions too. Altitude 100–800 m a.s.l.

#### Local and common names known in Cameroon.

None recorded.

#### IUCN conservation status.

Not evaluated.

#### Uses in Cameroon.

None recorded.

#### Notes.

Differences between var. insignis and var. batesii are quite small, and are mainly related to the pubescence of the branches, lower side of the leaves and petals (Le Thomas 1965a). There is one more recognized variety within *insignis*: var. concolor (Pellegr.) Le Thomas which differs by having shiny leaves on both sides and longer sepals. We have not seen this variety in Cameroon, but it is present in Gabon (Le Thomas 1965a, 1969b).

#### Specimens examined.

**East Region**: Village Djang 40 km west of Bertoua, 4.58°N, 13.35°E, *15 May 1962*, *Breteler F.J.* 2956 (P,WAG). **South Region**: Bitye near R Ja, 3.02°N, 12.37°E, *01 September 1922*, *Bates G.L.* 1792 (P); Rive du Ntem à Ebianemeyong 60 km east de Campo, 2.42°N, 10.33°E, *12 April 1970*, *Letouzey R.* 10370 (P,WAG,YA).

### ﻿﻿﻿﻿ Artabotrys jacquesfelicis

Taxon classificationPlantaeMagnolialesAnnonaceae

Pellegr., Bull. Soc. Bot. Fr. 97: 15, 1950

933F951F-3EFB-5C13-82C2-215117BF7D79

Fig. 15 ; Map 2G

= Artabotrys robustus Louis ex Boutique Bull. Jard. Bot. Etat Brux. 21: 107, 1950. Type. Democratic Republic of the Congo. Orientale, Yangambi, Louis J.L.P. 6077, 16 Sep 1937: lectotype, sheet here designated: BR[BR0000008820686]; isotypes: BR[BR000000882072]; K[K000795930]; NY[NY00025831]; P[P00363357].

#### Type.

Cameroon. Central Region; Nkidi forest, *Jacques-Félix H. 2490*, Nov 1938: lectotype, sheet here designated: P[P00363361]; isotypes: K; P[P00363359, P00363362].

#### Description.

Liana, height unknown, d.b.h. unknown. Indumentum of simple hairs; old leafless branches glabrous, **young foliate branches glabrous**. Leaves: petiole 4–7 mm long, ca. 1 mm in diameter, glabrous, grooved, blade inserted on the side of the petiole; blade 8–13 cm long, 3–14 cm wide, oblong to elliptic, apex acute, acumen ca. 0.5 cm long, base decurrent to acute, coriaceous, below glabrous when young and old, above glabrous when young and old, **shiny when dried**, concolorous; midrib impressed, above glabrous when young and old, below glabrous when young and old; secondary veins 9 to 12 pairs, glabrous above; tertiary venation reticulate. Flowers bisexual with 9 perianth parts in 3 whorls. Individuals bisexual; inflorescences ramiflorous on old leafless branches, leaf opposed. Flowers with 9 perianth parts in 3 whorls, 3 to 6 per inflorescence, hook-shaped peduncle 15–20 mm long; pedicel 10–25 mm long, ca. 1 mm in diameter, glabrous; in fruit 20–30 mm long, 1–2 mm in diameter, glabrous; bracts 1 to 2, all basal, basal bracts ca. 1 mm long, ca. 1 mm wide; sepals 3, valvate, free, 1–2 mm long, ca. 1 mm wide, triangular, apex acute, base truncate, glabrous outside, glabrous inside, margins flat; petals free, sub equal; **outer petals 3, 20–35 mm long, 1–2 mm wide, linear to narrowly ovate**, apex rounded, base broad and concave, **margins flat**, pubescent outside, glabrous inside; **inner petals 3, valvate, 15–25 mm long, 1–3 mm wide, linear**, apex rounded, base broad and concave, margins flat, densely pubescent outside, glabrous inside; stamens 50 to 70, in 3 to 4 rows, 2–3 mm long, oblong; connective discoid, pubescent; staminodes absent; carpels free, 3 to 4, ovary 3–4 mm long, stigma cylindrical, glabrous. **Monocarps stipitate, stipes 5–10 mm, ca. 6 mm in diameter, 1 to 4 monocarps, 20–40 mm long, 10–20 mm in diameter, ellipsoid, apex rounded, glabrous, warty to verrucose, faintly ribbed**, color when ripe unknown; seeds 1 to 2 per monocarp, 20–25 mm long, 10–15 mm in diameter, flattened ellipsoid; aril absent.

#### Distribution.

A central African species, only known from Cameroon and the Democratic Republic of the Congo; in Cameroon known from the Central and South regions.

**Figure 15.**
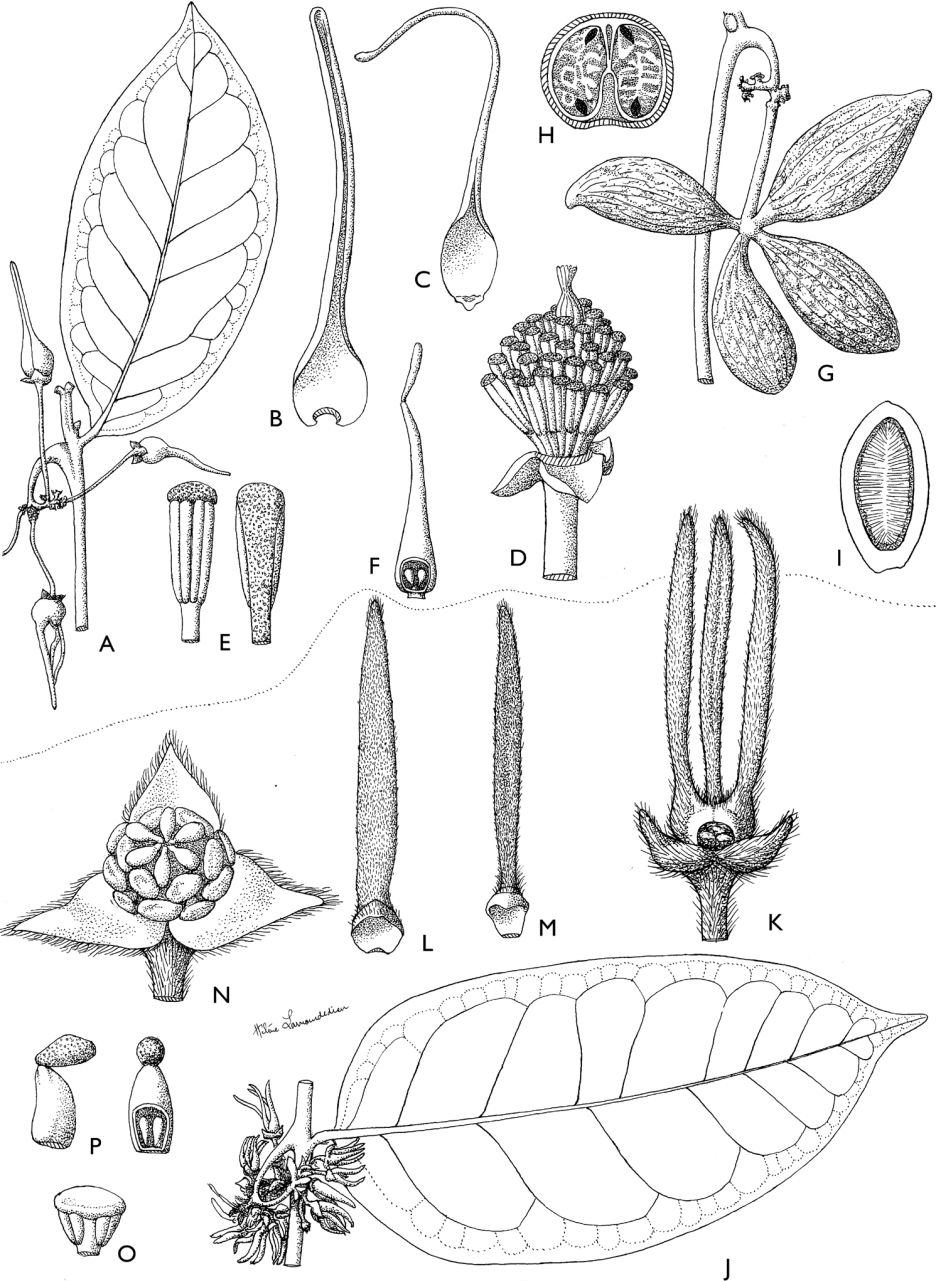
*Artabotrysjacquesfelicis***A** flowering branch **B** outer petal, inner view **C** inter petal, inner view **D** floral receptacle, petals removed **E** stamens, front and back view **F** carpel with detail on ovules **G** fruiting branch **H** seed, section latitudinal section **I** seed, longitudinal section. *Artabotrysvelutinus***J** flowering branch **K** flower receptacle, outer petals removed **L** outer petal, inner view **M** inner petal, inner view **N** flowering receptical, petals removed **O** stamen **P** carpel, side view, detail of ovules **A–F** from *Jacques Felix 2490***G–I** from *Tisserant 2405***J–P** from *Zenker 1222*. Drawings by Hélène Lamourdedieu, Publications Scientifiques du Muséum national d’Histoire naturelle, Paris.

#### Habitat.

A rare species across its range, in primary lowland rain forests. Altitude 500–600 m a.s.l.

#### Local and common names known in Cameroon.

None recorded.

#### IUCN conservation status.

Not evaluated.

#### Uses in Cameroon.

None recorded.

#### Notes.

﻿﻿﻿*Artabotrysjacquesfelicis* is distinguished by its entirely glabrous branches, petioles and leaves that are shiny above in herbarium material, narrowly ellipsoid flower buds, petals with a large concave base abruptly narrowing into an upper linear section, and ellipsoid monocarps with a warty to verrucose surface. In Cameroon, this species is only known from three old collections.

#### Specimens examined.

**South Region**: River Ja Bitya, 3.02°N, 12.37°E, *01 January 1922*, *Bates G.L.* 1699 (K); Bipindi, 3.08°N, 10.42°E, *01 January 1909*, *Zenker G.A.* 3834 (L,P).

### ﻿﻿﻿﻿ Artabotrys rufus

Taxon classificationPlantaeMagnolialesAnnonaceae

De Wild., Bull. Jard. Bot. État Bruxelles 4: 386, 1914

44BAD664-7FE1-5339-A4D2-311300223CC2

**Figure 16.**
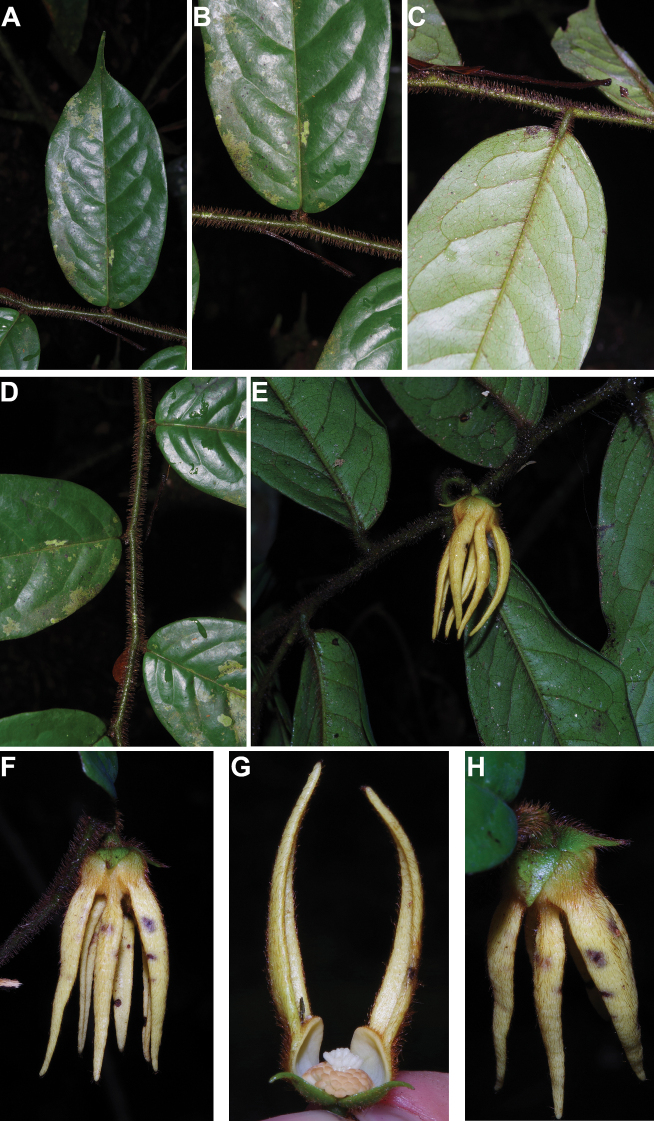
*Artabotrysrufus***A** leaf, upper side **B** base of leaf blade, upper side **C** base of leaf bade, lower side **D** branch, note erect hairs **E** flowering branch **F** flower, side view **G** detail of receptacle, 4 petals removed **H** flower, top view **A–H***Couvreur 854*, Gabon. Photos Thomas L.P. Couvreur.

**Figure 17.**
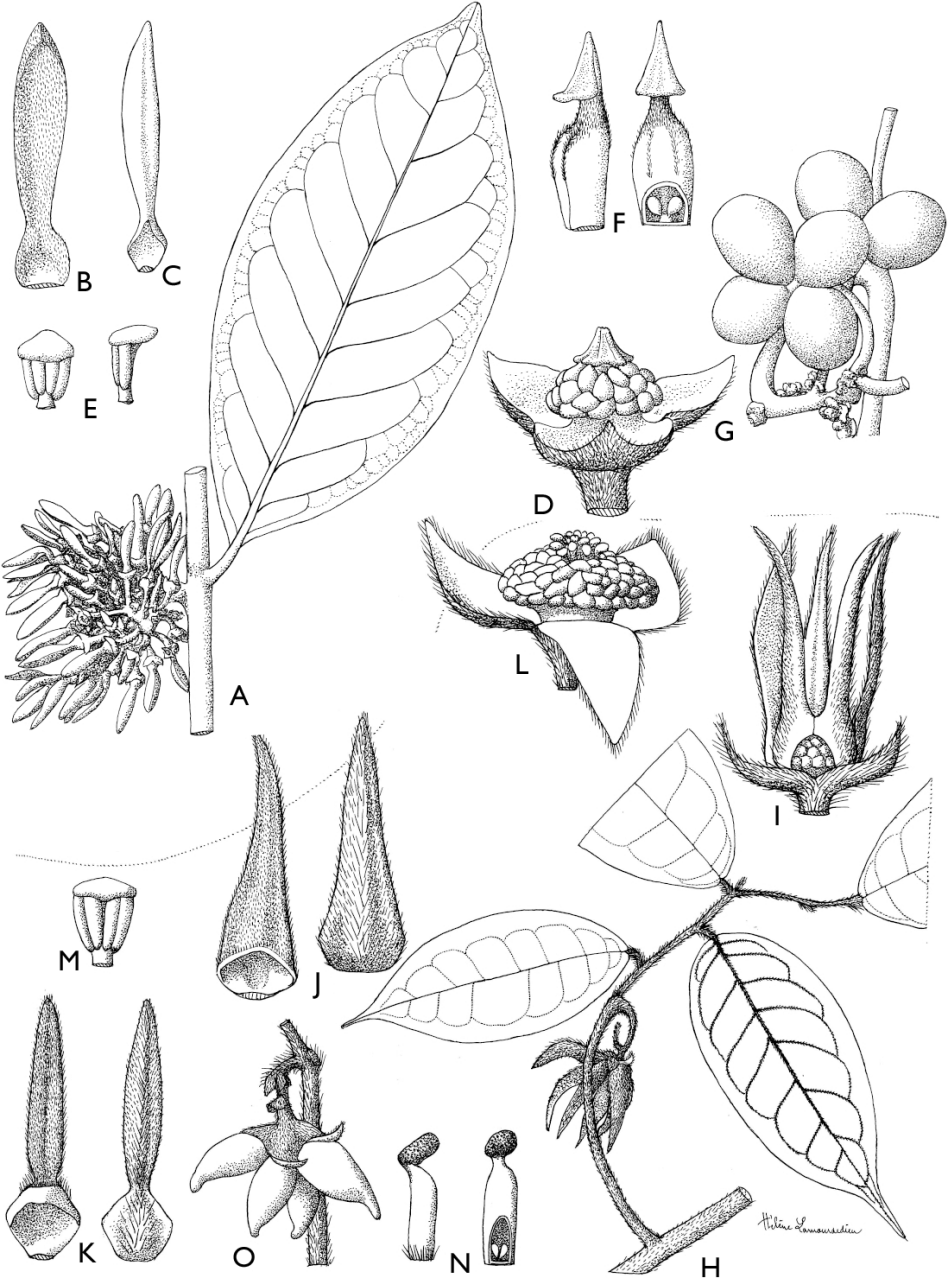
*Artabotrysthomsonii***A** flowering branch **B** outer petal, inner view **C** inner petal, inner view **D** flowering receptacle, petals removed **E** stamens, front and side views **F** carpel, side and detail of ovules **G** fruiting peduncle. *Artabotrysrufus***H** flowering branch **I** flowering receptacle with one outer petal removed **J** outer petals, outer view and inner views **K** inner petals, inner and outer views **L** flowering receptacle, petals removed **M** stamen **N** carpel, side view and detail of ovule **O** fruiting peduncle **A–G** from *Le Testu 9249***H–N** from *Hall 3193*; 15 from *Hall 3528*. Drawings by Hélène Lamourdedieu, Publications Scientifiques du Muséum national d’Histoire naturelle, Paris; modified from Le Thomas (1969b, pl. 23, p. 441 (*A.rufus*)).

Figs 16 , 17 ; Map 2H

= Artabotrys boonei De Wild., Repert. Spec. Nov. Regni Veg. 13: 383, 1914. Syn. nov. Type. Democratic Republic of the Congo. Orientale, Nala, Boone A. 80, 1911: lectotype, sheet here designated: BR[BR0000008820365]; isotype: BR[BR0000008820372].
= Artabotrys dahomensis Engl. & Diels, Notizbl. Königl. Bot. Gart. Berlin 2: 299, 1899. Syn. nov. Type. Benin: Dahome, *Newton s.n.*, 1886: holotype: B[B 10 0153018].
= Artabotrys setulosus Mildbr. & Diels, Bot. Jahrb. Syst. 53. 447, 1915. Type. Cameroon. East Region, Mulundou, *Mildbraed G.W.J. 4999*, 26 Jan 1911: lectotype, here designated: HBG[HBG502545].

#### Type.

Democratic Republic of the Congo. Equateur; Likimi, *Malchair L. 274*, 20 Avr 1910: holotype: BR[BR0000008820297]

#### Description.

Liana, to 20 m tall, d.b.h. 3–5 cm. Indumentum of simple hairs; old leafless branches sparsely pubescent, **young foliate branches hirsute, with erect hairs.** Leaves: petiole 2–4 mm long, 1–2 mm in diameter, **hirsute**, slightly grooved, blade inserted on top of the petiole; **blade 8–14 cm long**, 3.5–5.5 cm wide, oblong to obovate, apex acuminate, acumen 0.5–1 cm long, base rounded to subcordate or obtuse, papyraceous, below pubescent when young and old with long appressed hairs, above glabrous when young and old, concolorous; midrib impressed, above glabrous when young and old, below pubescent when young and old; secondary veins 9 to 12 pairs, glabrous above; tertiary venation reticulate. Individuals bisexual; inflorescences ramiflorous on old leafless branches, leaf opposed. Flowers with 9 perianth parts in 3 whorls, 1 to 3 per inflorescence, hook-shaped peduncle 4–7 mm long; pedicel 3–5 mm long, ca. 1 mm in diameter, **pubescent to sparsely hirsute**; in fruit 3–15 mm long, ca. 2 mm in diameter, pubescent; bracts caduceus, not seen; sepals 3, valvate, free, 5–7 mm long, 2–4 mm wide, triangular, apex acute, base truncate, green, **hirsute outside, glabrous inside**, margins flat; petals free, sub equal, green turning yellow; outer petals 3, 10–20 mm long, 3–4 mm wide, very narrowly elliptic to linear, apex acute, base rounded to broad and concave, green turning bright yellow, **margins flat, recurved inwards *in vivo***, densely appressed-pubescent outside, appressed-pubescent to glabrous inside; inner petals 3, valvate, 12–20 mm long, 2–3 mm wide, very narrowly elliptic to linear, apex acuminate to acute, base broad and concave, green turning bright yellow, margins flat, recurved inwards *in vivo*, appressed-pubescent outside, appressed-pubescent inside; stamens 60 to 70, in ca. 5 rows, ca. 1 mm long, broad; connective discoid, glabrous, green; staminodes absent; carpels free, 8 to 10, ovary ca. 1 mm long, stigma capitate, glabrous. Monocarps sessile, 5 to 11, 12–15 mm long, 5–6 mm in diameter, ellipsoid to fusiform, **apex apiculate, glabrous, smooth**, not ribbed, red when ripe; seeds 1 to 2 per monocarp, 10–12 mm long, 5 mm in diameter, ellipsoid to oblong; aril absent.

#### Distribution.

A mainly central African species, from Benin to Nigeria and Cameroon to the Republic of the Congo and in the Democratic Republic of the Congo; in Cameroon known from the Central and East regions.

#### Habitat.

A fairly common species, in secondary lowland or premontane rain forests. Altitude 400–900 m a.s.l.

#### Local and common names known in Cameroon.

nginda (pygmée–bibaya) (Letouzey 1964).

#### IUCN conservation status.

Not evaluated.

#### Uses in Cameroon.

None recorded.

#### Notes.

﻿﻿﻿*Artabotrysrufus* is distinguished by the hirsute pubescence of the young foliate branches, petioles, peduncles and flowering pedicels, its leaves that are elliptic, apiculate and less than 14 cm long, with long (1–2 mm) appressed hairs on the lower side of the leaf blade and a rounded to subcordate or obtuse base, a short peduncle (generally less than 7 mm long), petals with long dense brown hairs and smooth apiculate monocarps. The tertiary venation is also clearly visible forming clear loops towards the margins of the leaves.

﻿﻿﻿*Artabotrysrufus* resembles ﻿*A.velutinus* being pubescent, but the pubescence of ﻿*A.velutinus* is not hirsute, with shorter hairs and more tomentose on the young foliate branches and petioles. The petals are also very similar being curved inwards, giving them the appearance of a tube. It is possible that both names are synonymous. ﻿﻿﻿*Artabotrysrufus* is also very close morphologically to the west African species *A.hispidus* Sprague & Hutch. by its hirsute pubescence and the shape of the leaves and flowers. It is also very possible that these names are synonymous. Several specimens from Cameroon where identified as *A.hispidus*, but upon closer look we have identified them as belonging to ﻿*A.rufus*. Several authors (Boutique 1951b; Le Thomas 1969b) have suggested that the name ﻿*A.rufus* might be synonym with the east African ﻿*A.rupestris* Diels, although Verdcourt didn’t recognize this synonymy (Verdcourt 1971a) suggesting differences in the leaves.

We here synonymize the names ﻿*A.boonei* and ﻿*A.dahomensis* with ﻿*A.rufus.* The former name was considered a synonym of ﻿*A.velutinus* (Lebrun and Stork 1991), but the type clearly suggests it is a synonym of ﻿*A.rufus* (if the latter is really distinct from ﻿*A.velutinus*).

More detailed studies across the range of these species (*A.hispidus*, ﻿*A.rufus*, ﻿*A.rupestris* and ﻿*A.velutinus*) are needed to determine if there is one single widespread species from west to east Africa (possibly with different infraspecific taxa), or if there are several different species possibly grouped into a species complex (Hawthorne and Jongkind 2006).

#### Specimens examined.

**Central Region**: Fébé Mount, 3.91°N, 11.48°E, *30 March 1962*, *Breteler F.J.* 2717 (A,BR,K,P,WAG,YA); Mont Mbam Minkon on trail 5 km from Nkol Nyada village On top of small hill, 3.97°N, 11.40°E, *21 March 2013*, *Couvreur T.L.P.* 418 (MPU,WAG,YA); Nachtigal, 4.35°N, 11.63°E, *01 July 1964*, *de Wilde W.J.J.O* 2779 (P,WAG,YA); Nkolbison, 3.88°N, 11.45°E, *02 November 1964*, *de Wilde W.J.J.O* 3715 (BR,K,P,WAG); Left bank Sanaga river near Ferry Nachtigal ca 20 km N of Obala, 4.34°N, 11.64°E, *29 April 1965*, *Leeuwenberg A.J.M.* 6011 (B,BR,C,K, MO,P,WAG,YA). **East Region**: Dimako, 4.38°N, 13.57°E, *02 August 1961*, *Breteler F.J.* 1752 (P,YA); Bertoua 15 km along road to Deng Deng, 4.66°N, 13.63°E, *31 August 1961*, *Breteler F.J.* 1825 (WAG); Bamékok (Batouri), 4.2°N, 14.15°E, *16 April 1962*, *Breteler F.J.* 2825 (P,WAG); 60 km south of Yokadouma 5 km south of Maséa village, 3.10°N, 14.84°E, *06 March 2019*, *Couvreur T.L.P.* 1211 (MPU,WAG,YA); A 25 km au NE de Bangé km 75 route Yokadouma-Moloundou, 3.02°N, 15.12°E, *25 May 1963*, *Letouzey R.* 5147 (P,YA); Mbatika-Malen 20 km de Moloundou route Yokadouma 2.03°N, 15.22°E, *22 April 1971*, *Mezili P.* 193 (P,YA); Moloundou near Lokomo Bumba and Bange, 2.08°N, 15.25°E, *26 January 1911*, *Mildbraed G.W.J.* 4350 (B,HBG); Forêt inhabitée entre Yokaduma et Assobam, 3.52°N, 15.05°E, *24 April 1911*, *Mildbraed G.W.J.* 4999 (B,HBG).

### ﻿﻿﻿﻿ Artabotrys thomsonii

Taxon classificationPlantaeMagnolialesAnnonaceae

Oliv., Fl. Trop. Afr. 1: 28, 1868

0668E006-B788-5518-BA7B-410AAED58043

**Figure 18.**
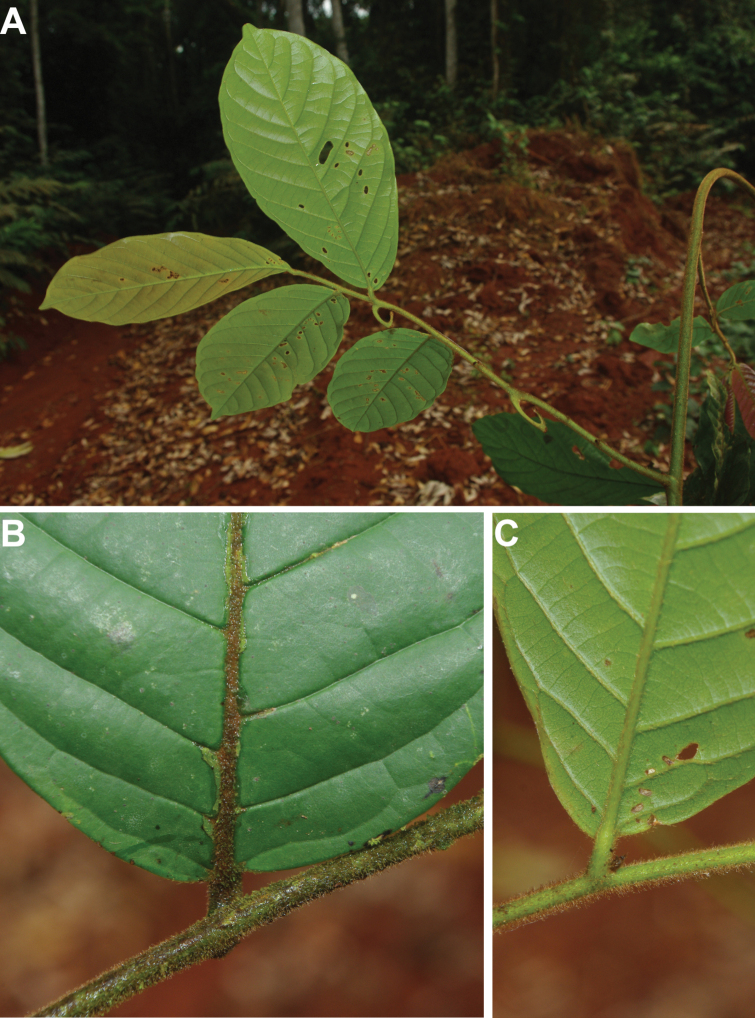
*Artabotrysthomsonii***A** branch **B** base of leaf blade, upper side, note densely pubescent midrib and glabrous blade **C** base of leaf blade, lower side **A–C***Couvreur 751*, Mindourou, Cameroon. Photos Thomas L.P. Couvreur.

Figs 17 , 18 ; Map 2I

#### Type.

Nigeria. Cross River State; Old Calabar, *Thomson W.C 25*, Feb 1863: holotype: K[K000198871].

#### Description.

Liana, 30 m tall, d.b.h. 10–20 cm. Indumentum of simple hairs; old leafless branches sparsely pubescent to glabrous, young foliate branches sparsely pubescent. Leaves: petiole 1–10 mm long, ca. 2 mm in diameter, pubescent to glabrous, slightly grooved, blade inserted on top of the petiole; blade 7–20 cm long, 5–10 cm wide, elliptic to oblong, apex acute to abruptly acuminate, acumen 0.5–1 cm long, base rounded to obtuse, coriaceous, below sparsely pubescent to glabrous when young, sparsely pubescent to glabrous when old, above glabrous when young and old, concolorous; midrib **impressed, above densely pubescent to pubescent when young and old**, below sparsely pubescent to glabrous when young and old; secondary veins 7 to 14 pairs, glabrous above; tertiary venation intermediate. Individuals bisexual; inflorescences ramiflorous on old leafless branches, leaf opposed. Flowers with 9 perianth parts in 3 whorls, **30 to 90 per inflorescence**, hook-shaped peduncle 20–35 mm long; **pedicel 10–20 mm long**, ca. 1 mm in diameter, pubescent; in fruit 10–25 mm long, ca. 2 mm in diameter, pubescent; bracts several, basal with one towards the lower half of pedicel, soon falling, basal bracts ca. 1 mm long, ca. 1 mm wide; upper bract ca. 2 mm long, ca. 2 mm wide; sepals 3, valvate, free, 2–3 mm long, 2–3 mm wide, triangular, apex acute, base truncate, pubescent outside, glabrous inside, margins flat; petals free, sub equal; outer petals 3, 10–20 mm long, 2–3 mm wide, elliptic to narrowly elliptic, apex rounded to obtuse, base broad and concave, margins flat, not folded inwards, pubescent outside, pubescent with a glabrous base inside; inner petals 3, valvate, 12–16 mm long, 2–3 mm wide, narrowly elliptic, apex acute, base broad and concave, margins flat, not folded inwards, pubescent outside, pubescent inside; stamens 30 to 35, in 2 to 3 rows, ca. 1 mm long, cuneiform; connective discoid, glabrous; staminodes absent; carpels free, 4 to 10, ovary ca. 1 mm long, stigma coiled, sparsely pubescent. **Monocarps stipitate, stipes 10–25 mm long**, 2–3 mm in diameter; monocarps 1 to 7, 15–25 mm long, 12–15 mm in diameter, ellipsoid to obovoid, **apex rounded, glabrous, smooth, not ribbed**, green when ripe; seeds 1 (to 2) per monocarp, 10–15 mm long, 8–9 mm in diameter, ellipsoid; aril absent.

#### Distribution.

A central African species; from Nigeria to Angola (Cabinda) and in the Democratic Republic of the Congo; in Cameroon known from the Central, East, Littoral, South, South-West and West regions.

#### Habitat.

A common species, in lowland and premontane secondary or primary rain forests, along forests margins and in logging areas. Altitude 100–1000 m a.s.l.

#### Local and common names known in Cameroon.

None recorded.

#### IUCN conservation status.

Not evaluated.

#### Uses in Cameroon.

***food***: water drinken from stem; ***medicine***: water/sap used for liver, genital stimulant/depressants, pregnancy, antiaborifacients.

#### Notes.

﻿﻿﻿*Artabotrysthomsonii* is easily distinguished by the densely pubescent upper midrib which is not found in any other Cameroonian species of ﻿*Artabotrys*.

There seems to be confusion around the type specimen of the name ﻿﻿﻿*Artabotrysthomsonii*. In the protologue, Oliver (1868, p. 28) indicates “*Thomson* !, Old Calabar” as the type. In the Flore du Gabon, Le Thomas (1969b) indicates that the type is *Thomson s.n.*, and notes that the sheet deposited in Kew is a mixed collection: *pro parte* with the fruits and associated leaves belonging to a different species (see below). This corresponds to specimen K000198872, labeled as *Thomson 2310*. However, this collection belongs to *Mann 2310* (interestingly Le Thomas didn’t mention the number when it is clearly indicated) and not *Thomson 2310* as indicated. The Kew specimen is confusing because it clearly says “collected by Rev W. C. Thomson” followed by the number 2310 with no mention of Mann. However, the duplicate in B [B 10 0154054] says “Old Calabar River, G. Mann 1863” followed by the mention “same as [hard to read, personal interpretation] Rev. W. Thomson”. The specimen in P [P00363393] has an identical handwriting and text as the one in B, but also has the mention “from Rev. W. Thomson”. However, the handwriting (same as in other true Mann collections) and high number (+2000) suggest this is a Mann collection and thus not the type (Thomson W.C. collections are below 150 from what we can see in K).

The collection *Thomson 25* is without doubt a Thomson collection with a printed label indicating “Collected at Old Calabar, by the Rev. W.C. Thomson” followed by “Presented by Professor Balfour, Dec; 1963”. Indeed, some specimens of Thomson were forwarded by Balfour to Kew (Oliver 1865, p. 156).

For what it is worth, the fruit and leaves on *Mann 2310* (K [K000198872] and P [P00363393]) appear to belong to ﻿﻿﻿*Neostenantheramyristicifolia* (Oliv.) Exell also present in Nigeria.

#### Specimens examined.

**Central Region**: Yaoundé, 3.87°N, 11.52°E, *1896*, *Zenker G.A.* 697 (P). **East Region**: Near Dimako 28 km SW of Bertoua, 4.38°N, 13.57°E, *01 August 1961*, *Breteler F.J.* 1725 (BR,K,P,U,WAG,YA); Bertoua 6 km along road to Batouri and Betaré-Oya, 4.58°N, 13.68°E, *30 August 1961*, *Breteler F.J.* 1797 (U,WAG,YA); 67 km south of Yokadouma 30 km after Ngato 15 km after river ALPICAM ‘base de vie’ then 40 km on forestry road starting 4 km before Maséa village, 3.08°N, 14.67°E, *08 March 2019*, *Couvreur T.L.P.* 1230 (MPU,WAG,YA); Palisco forest consession 15 km along main road into consession, 3.52°N, 13.54°E, *27 March 2015*, *Couvreur T.L.P.* 751 (WAG,YA); 20 km environ à l’ENE de Mikel village situé à 85 km au N de Moloundou sur la route de Yokadouma 2.81°N, 15.24°E, *23 February 1971*, *Letouzey R.* 10413 (K,P,YA); Ndongo (Dja-Molundou), 2.58°N, 15.29°E, *18 March 1973*, *Letouzey R.* 12240 (K,P,YA); Djouo (Somalomo), 3.32°N, 12.93°E, *26 February 1962*, *Letouzey R.* 4435 (K,P,YA); A 8 km au SSW de Koso (village situé à 60 km au SSW de Batouri), 3.93°N, 14.17°E, *29 July 1963*, *Letouzey R.* 5533 (P,YA); Entre Badekok et Mpan (50 km ENE de Lomié), 3.22°N, 15.02°E, *05 August 1963*, *Letouzey R.* 5548 (P,YA). **Littoral Region**: 8 km W of Massok, 4.13°N, 10.47°E, *27 March 1965*, *Leeuwenberg A.J.M.* 5201 (B,BR,C,GC,K,MO,P,UC,WAG,YA). **South Region**: Bitya near R Ja, 3.02°N, 12.37°E, *01 November 1922*, *Bates G.L.* 1763 (P); Djoum North East Nkout Base of ridge, 2.55°N, 12.80°E, *05 December 2014*, *Cheek M.* 17781 (K,WAG); Elephant Mont, 2.8°N, 10.01°E, *24 May 2001*, *van Andel T.R.* 3459 (KRIBI,WAG,YA); Campo-Ma’an area 2.73°N, 9.873°E, *16 August 2001*, *van Andel T.R.* 3872 (KRIBI,U,WAG); Nkuambe, 3.26°N, 10.46°E, *01 December 1914*, *Zenker G.A.* 489 (P,WAG). **South-West Region**: Bayang Mbo Wildlife Sanctuary after Mbu river, 5.35°N, 9.497°E, *27 March 2016*, *Couvreur T.L.P.* 1020 (WAG,YA); Ekombe, 4.48°N, 10.87°E, *16 January 1987*, *Etuge M.* 485 (MO,P,WAG). **West Region**: Près Bandounga à 40 km au NW de Ndikinimeki, 4.98°N, 10.55°E, *12 February 1972*, *Letouzey R.* 11200 (P,YA).

### ﻿﻿﻿﻿ Artabotrys velutinus

Taxon classificationPlantaeMagnolialesAnnonaceae

Scott Elliot, J. Linn. Soc., Bot. 30: 71, 1894

4A5B7CB7-80EF-5B6D-9157-BCA5D4FF5A45

Fig. 15 ; Map 3A

= Artabotrys nigericus Hutch., Bull. Misc. Inform. Kew 10: 356, 1921. Type. Nigeria. Jos North, Naraguta, Lely H.V. 541, 17 Aug 1921: holotype: K[K000198866].
= Artabotrys stenopetalus Engl. Notizbl. Königl. Bot. Gart. Berlin 2: 300, 1899. Syn. nov. Type. Cameroon. South Region, Bipindi, Zenker G.A. 1222, 1896: holotype: B[B10 0154052]; isotypes: GOET[GOET005675]; HBG[HBG502541]; K[K000198862]; M[M0107912]; MO[MO-216860]; NY[NY00025832]; P[P00363384, P00363385]; S[S07-13456]; WAG[WAG0053235]; WU[WU0025887, WU0025888].
? Artabotrys stenopetalus var. parviflorus Pellegr., Mém. Soc. Linn. Normandie 26: 7, 1924. Type. Gabon. Nyanga, Tchibanga, *Le Testu G.M.P.C. 1964*, 6 Jan 1915: lectotype, sheet here designated: P[P00363379]; isotypes: EA[EA000002453, EA000002452]; K[K000198861]; LISC[LISC000375]; P[P00363378, P00363380].
= Artabotrys nitidus auct. Exell Jour. of Bot. 73 Supp. Polypet. Add.: 5, 1935 (non Diels) (specimens *Gossweiler 5978* [COI00070298] and *7361* [COI00070297]).

#### Type.

Sierre Leone: Northern Region; Falaba, *Scott Elliot G.F. 5137*, 5 Mar 1892: holotype: K[K000198865]; isotype: B[B 10 0154055].

#### Description.

Liana, up to 10 m tall, d.b.h. up to 20 cm. Indumentum of simple hairs; **old leafless branches sparsely pubescent, young foliate branches pubescent.** Leaves: petiole 3–4 mm long, ca. 1 mm in diameter, pubescent to glabrous, grooved, blade inserted on the side of the petiole; blade 8–13 cm long, 4–5 cm wide, ovate to elliptic, apex acuminate to acute, acumen 1–1.5 cm long, **base decurrent to acute**, papyraceous, below pubescent to sparsely pubescent when young, glabrous when old, above pubescent when young, sparsely pubescent to glabrous when old, concolorous; midrib impressed, above glabrous when young and old, below densely pubescent to pubescent when young, glabrous to pubescent when old; secondary veins 8 to 12 pairs, glabrous above; tertiary venation reticulate. Individuals bisexual; inflorescences ramiflorous on old leafless branches, leaf opposed. Flowers with 9 perianth parts in 3 whorls, 5 to 15 per inflorescence, hook-shaped peduncle 10–16 mm long; pedicel 2–10 mm long, ca. 1 mm in diameter, pubescent with appressed hairs; in fruit 10–20 mm long, ca. 1 mm in diameter, pubescent with appressed hairs; bracts 2, all basal, ca. 1 mm long, ca. 1 mm wide; sepals 3, valvate, free, 2–3 mm long, 2–3 mm wide, triangular, apex acute, base truncate, densely pubescent outside, glabrous inside, yellow and red at the base, margins flat; petals free, sub equal; **outer petals 3, 5–15 mm long, 1–2 mm wide, linear**, apex rounded, base broad and concave, **margins flat but strongly recurved inwards forming a tube**, **densely pubescent outside, densely pubescent inside**; inner petals 3, valvate, 5–15 mm long, 1–2 mm wide, linear, apex acute, base broad and concave, yellow and red at the base, margins flat but strongly recurved inwards forming a tube, tomentose outside, tomentose with a glabrous at base inside; stamens 15 to 22, in 2 rows, 1–2 mm long, oblong; connective discoid, glabrous; staminodes absent; carpels free, 7 to 12, ovary 1–2 mm long, stigma cylindrical, glabrous. Monocarps sessile, 4 to 9, 10–20 mm long, 10 mm in diameter, ellipsoid to obovoid, apex rounded, glabrous, smooth, faintly ribbed, color when ripe not seen; seeds 1 to 2 per monocarp, 5–7 mm long, 5 mm in diameter, flattened ellipsoid; aril absent.

#### Distribution.

A west and central African species, from Sierra Leone to Nigeria and Cameroon to the Republic of the Congo and in the Democratic Republic of the Congo; in Cameroon known from the Adamaoua, Central, East, North, South, South-West, and West regions.

#### Habitat.

A fairly common and widespread species; in secondary lowland premontane and montane rain forests, and in gallery forests occurring in the drier regions of the country, it is one of the few Annonaceae species (e.g. ﻿﻿﻿*Monanthotaxisvulcanica*; ﻿﻿*Xylopiaafricana*) occurring above 2000 m in Cameroon. Altitude 400–2200 m a.s.l.

#### Local and common names known in Cameroon.

None recorded.

#### IUCN conservation status.

Not evaluated.

#### Uses in Cameroon.

***medicine***: water/sap/leaves used for liver, genital stimulant/depressants, pregnancy, antiaborifacients (as ﻿*A.stenopetalus* in Burkill 1985).

**Map 3.**
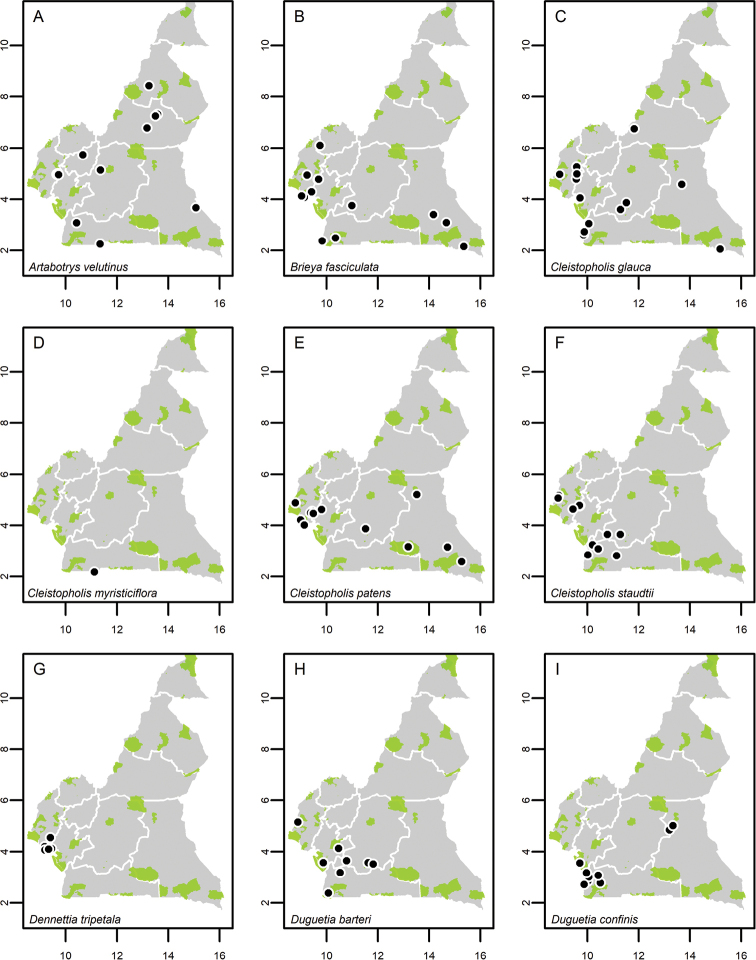
**A***Artabotrysvelutinus***B***Brieyafasciculata***C***Cleistopholisglauca***D***Cleistopholismyristiciflora***E***Cleistopholispatens***F***Cleistopholisstaudtii***G***Dennettiatripetala***H***Duguetiabarteri***I***Duguetiaconfinis*. White borders represent region limits in Cameroon; green patches represent protected areas (see methods and Suppl. material 1: Fig. S1).

#### Notes.

﻿﻿﻿*Artabotrysvelutinus* is characterized by having leaf bases that are decurrent or acute, pubescent branches and petioles with appressed hairs and densely pubescent petals that are tightly recurved forming a tube. This latter character is also found in ﻿*A.rufus*, but in this species the pubescence is hirsute rather than appressed and the base of the leaves is rounded. Nevertheless, both species are very close morphologically.

﻿﻿﻿*Artabotrysvelutinus* belongs to a large species complex of pubescent species with petals that are tightly recurved forming a tube. We synonymize here the name ﻿*A.stenopetalus*. Another species name that could also be synonym is the east African species *A.monteiroae* Oliv. (which would be the older name). More studies are needed across the range of these species to better define the limits of these taxa.

The variety ﻿﻿A.stenopetalusvar.parviflorus is interesting. The type specimen (*Le Testu 1964*) appears to have a raised and grooved midrib on the upper side of the leaf blades (only seen as a scan on jstor), which is quite uncommon for African Annonaceae (Couvreur 2009), and the leaves are narrowly elliptic (versus elliptic for the type variety). The flowers however have the characteristic tubular petals. Pellegrin (1924, p. 7) only cites some minor differences such as smaller flowers, an acute leaf base (but this is also the case for the type variety) and petals that are adnate between them. We leave this name as a synonym of ﻿*A.velutinus* but further studies should be done to properly interpret the status of this variety name.

#### Specimens examined.

**Adamaoua Region**: Mbibol 40 km W de Ngaoundéré, 7.32°N, 13.58°E, *12 June 1977*, *Fotius G.* 2660 (P,YA); Près Tekel (60 km NNO de Bagodo), 6.78°N, 13.17°E, *21 July 1966*, *Letouzey R.* 7481 (P,YA); Boko 14 km Sud-Ouest de Ngaoundéré, 7.25°N, 13.5°E, *06 August 1981*, *van der Zon A.P.M.* 1122 (WAG,YA). **Central Region**: Pentes orientales du mont Yangba (1473 m) près Nyafianga (42 km NNE de Bafia), 5.13°N, 11.35°E, *09 September 1966*, *Letouzey R.* 7826 (K,P,YA). **East Region**: 27 km ENE de Mopwo (village situé au km 22 route Yokadouma-Batouri), 3.67°N, 15.08°E, *06 June 1963*, *Letouzey R.* 5248 (K,P,YA). **North Region**: Mango, 8.42°N, 13.25°E, *21 July 1974*, *Fotius G.* 2144 (YA). **South Region**: Rives du Ntem près du confluent de la Kye 16 km ESE d’Ambam, 2.25°N, 11.34°E, *01 February 1970*, *Letouzey R.* 10040 (P); Bipindi, 3.08°N, 10.42°E, *3 April 1897*, *Zenker G.A.* 1222 (B,P,WAG); Bipindi, 3.08°N, 10.42°E, *01 January 1913*, *Zenker G.A.* 231 (P,U,WAG). **South-West Region**: Edip village to lake edip 2–3 km, 4.96°N, 9.65°E, *11 February 1998*, *Cheek M.* 9143 (K,WAG,YA); Kodmin, 5°N, 9.7°E, *16 November 1998*, *Gosline W.G.* 149 (K,YA); Bank of river Chide, 4.95°N, 9.72°E, *04 February 1998*, *Onana J.M.* 523 (K,P,WAG,YA). **West Region**: Massif du Nkogam (2263 m) 25 km W de Foumban, 5.73°N, 10.67°E, *12 December 1974*, *Letouzey R.* 13501 (P,YA).

### ﻿﻿ Brieya

Taxon classificationPlantaeMagnolialesAnnonaceae

De Wild., Repert. Spec. Nov. Regni Veg. 13: 383, 1914

1B6FC0B4-50C2-561F-9237-4314FE1CD879

#### Type species.

﻿﻿﻿*Brieyafasciculata*De Wild.

#### Description.

Same as species.

A genus with two species, one widespread and one restricted to northern Angola. One species in Cameroun, not endemic.

A genus easily confused with ﻿*Piptostigma* because of the characteristic inner petals being much longer than the outer ones in both genera, a unique feature among Cameroonian Annonaceae (Ghogue et al. 2017). However, sterile, ﻿*Brieya* is mainly distinguished by the lower number of secondary veins (less than 20 versus generally more than 20 in *Pipostigma*) and discolorous leaves being whitish below (versus concolourous in ﻿*Piptostigma*).

#### Taxonomy.

Ghogue et al. (2017).

### ﻿﻿﻿﻿ Brieya fasciculata

Taxon classificationPlantaeMagnolialesAnnonaceae

De Wild., Repert. Spec. Nov. Regni Veg. 13: 384, 1914

CF995930-80B1-5211-807F-27192E5B5418

**Figure 19.**
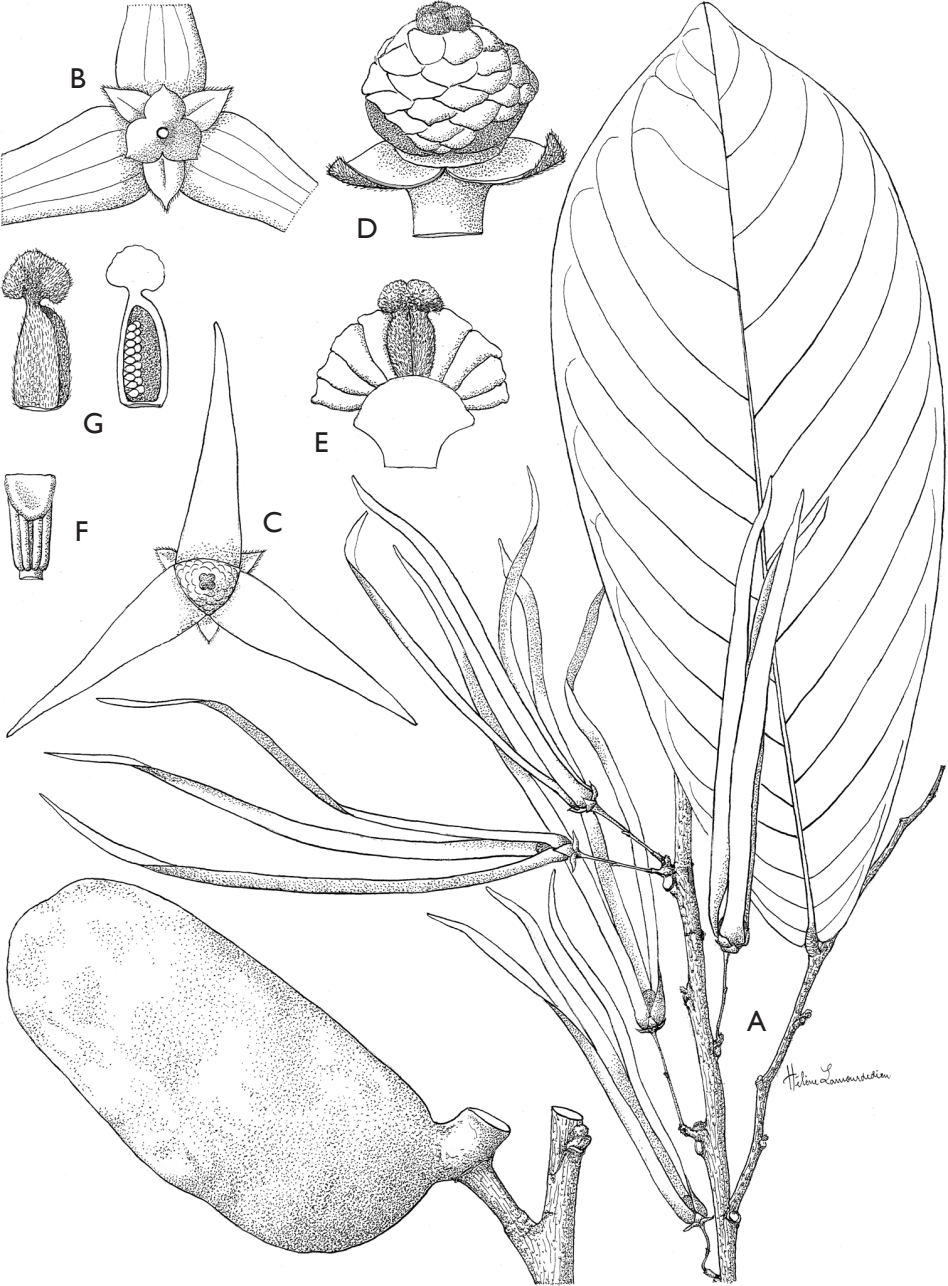
*Brieyafasciculata***A** flowering branch **B** flower, bottom view **C** flower, top view **D** detail of receptacle, all petals removed **E** longitudinal section of receptacle **F** stamen **G** carpel, side view and detail of ovules **A** from *Aubréville 1500***B–G** from *Hallé 3166*; 8 from *Germain 2396*. Drawings by Hélène Lamourdedieu, Publications Scientifiques du Muséum national d’Histoire naturelle, Paris; modified from Le Thomas (1969b, pl. 22, p. 127).

**Figure 20.**
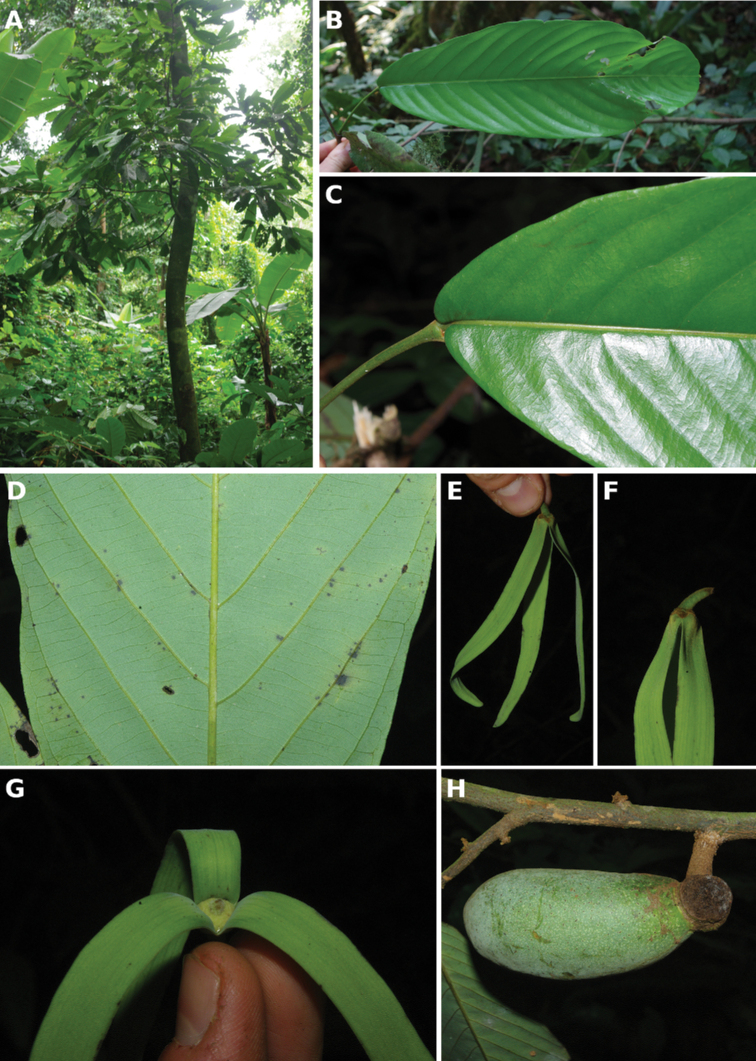
*Brieyafasciculata***A** habit **B** leaf, upper side **C** leaf base, upper side **D** detail of leaf blade and venation; lower side **E** flower **F** detail of minute sepals and outer petal, in contrast to long inner petals **G** detail of receptacle **H** fruit with a single monocarp (others have fallen) **A, H***Couvreur 645*, Mambe, Cameroon **B, C** no voucher, Rumpi Mountains, Cameroon **D–G***Couvreur 510*, Mt Etinde, Cameroon. Photos Thomas L.P. Couvreur.

Figs 19 , 20 ; Map 3B

≡ Piptostigma fasciculatum (De Wild.) Boutique ex Fries, In Engler A., Prantl K. (eds) Die Natürlichen Pflanzenfamilien 17aII: 115–116, 1959.
= Piptostigma aubrevillei Ghesq. ex. Aubrév.; Fl. For. Cote d’Ivoire 1: 98, 1936. Type. Ivory Coast. Mudjika, Aubréville A. 2115, 1932: lectotype, designated by Ghogue et al. (2017), p. 211: P[P02032149].

#### Type.

Democratic Republic of the Congo. Bas-Congo; Kingamu, Ganda sumi, *de Briey J. 66*, 14–16 Oct 1911: lectotype, sheet here designated: BR[BR-S.P.880319]; isotypes: BR[BR0000008803252, BR0000008803245, BR0000008803191].

#### Description.

Tree, 10–25 m tall, d.b.h. 16–50 cm; stilt roots or buttresses absent. Indumentum of simple hairs; old leafless branches glabrous, young foliate branches pubescent. Leaves: petiole 2–5 mm long, ca. 2 mm in diameter, pubescent, grooved, blade inserted on top of the petiole; blade 12–24 cm long, 6–8 cm wide, obovate to oblanceolate, apex acuminate to obtuse, acumen 0.5–0.8 cm long, **base cordate to obtuse**, papyraceous, below glabrous to pubescent when young, glabrous to pubescent when old, above glabrous when young and old, **discolorous**, **whitish below**; midrib impressed, above pubescent when young and old, below pubescent when young and old; **secondary veins 11 to 17 pairs**, glabrous below; **tertiary venation percurrent**. Individuals bisexual; inflorescences ramiflorous on old leafless branches, axillary **occurring on short peduncle-like bases 0–2 mm long**. Flowers with 9 perianth parts in 3 whorls, 1 to 4 per inflorescence; **pedicel 1–2 mm long**, ca. 5 mm in diameter, pubescent; in fruit 15–25 mm long, 4–5 mm in diameter, glabrous; bracts 2, one basal and one upper towards the lower half of pedicel, basal bract 2–3 mm long, ca. 2 mm wide; upper bract ca. 1 mm long, ca. 2 mm wide; sepals 3, valvate, free, ca. 2 mm long, ca. 2 mm wide, ovate, apex acute, base truncate, brown, pubescent outside, glabrous inside, margins flat; petals free, **outer petals shorter than inner; outer petals 3, sepal like, 1.5–2 mm long, 1.5 mm wide**, ovate, apex acuminate, base truncate, light green, margins flat, pubescent outside, glabrous inside; **inner petals 3, valvate, 38–108 mm long, 3–7 mm wide, linear**, apex acute, base truncate, green, margins flat, pubescent inside, pubescent outside; stamens 30 to 40, in 4 to 5 rows, ca. 1 mm long, broad; connective discoid, glabrous, green; staminodes absent; carpels free, ca. 4, ovary ca. 2 mm long, stigma minute, densely pubescent. **Monocarps sessile, 1 to 3, 42–46 mm long, 25–40 mm in diameter, ellipsoid, apex rounded**, glabrous, smooth, fleshy, green when ripe; seeds 18 to 20 per monocarp, ca. 10 mm long, ca. 4 mm in diameter, ellipsoid; aril absent.

#### Distribution.

From Côte d’Ivoire to Democratic Republic of the Congo and Angola; in Cameroon known from East, South, Centre, Littoral and South-West regions.

#### Habitat.

A common species when present, in lowland to submontane rain forests in primary or secondary habitats. Altitude 250–810 m a.s.l.

#### Local and common names known in Cameroon.

baouéfou à grandes feuilles (french) (Burkill 1985).

#### IUCN conservation status.

Least Concern (LC) (Cosiaux et al. 2019e).

#### Uses in Cameroon.

None recorded.

#### Notes.

﻿﻿﻿*Brieyafasciculata* is distinguished by its discolorous leaves, glaucous white below with a percurrent tertiary venation, its flowers occurring on reduced inflorescences with a short peduncle, the inner petals much longer than the outer ones, with the minute sepals and outer petals identical is shape and size, and its long green linear inner petals.

#### Specimens examined.

**Central Region**: Près Ngong (25 km NE d’Edéa), 3.75°N, 10.98°E, *12 December 1973*, *Letouzey R.* 12345 (P,YA). **East Region**: 68 km south of Yokadouma 30 km after Ngato 15 km after river ALPICAM ‘base de vie’ then 40 km on forestry road starting 4 km before Maséa village, 3.08°N, 14.66°E, *08 March 2019*, *Couvreur T.L.P.* 1231 (MPU,WAG,YA); Batéka Malen 20 km NE de Moloundou, 2.15°N, 15.35°E, *23 April 1971*, *Letouzey R.* 10718 (P,YA); Entre Asip et Mang (60 km ENE de Lomié), 3.4°N, 14.17°E, *13 August 1963*, *Letouzey R.* 5605 (P,YA). **North-West Region**: Kagwene, 6.10°N, 9.744°E, *13 June 2009*, *Ashworth J.* 310 (K,YA). **South Region**: Campo Ma’an National Park 11 km on trail from Ebinanemeyong village on road 7 km from Nyabessan to Campo town, 2.48°N, 10.34°E, *11 February 2015*, *Couvreur T.L.P.* 677 (WAG,YA); Abords de la Lobé à 50 km au SSE de Kribi et à 30 km à l’ENE de Campo, 2.51°N, 9.82°E, *23 March 1968*, *Letouzey R.* 9132 (YA). **South-West Region**: Mudjika (Wudjika?), 4.29°N, 9.41°E, *01 January 1933*, *Aubréville A.* 2115 (P); on trail from Ekongo village located 5 km before the entrance to Limbe 7 km on secondary road On flank of Mt Etinde 100 m in Mont Cameroon National Park, 4.07°N, 9.132°E, *16 October 2013*, *Couvreur T.L.P.* 510 (WAG,YA); on trail from Ekongo village located 5 km before the entrance to Limbe 7 km on secondary road On flank of Mt Etinde 100 m in Mont Cameroon National Park, 4.07°N, 9.131°E, *16 October 2013*, *Couvreur T.L.P.* 511 (WAG,YA); Rumpi mountains forest trail ca 5 km after Dikome Balue village ca 40 km north of Kumba, 4.93°N, 9.240°E, *10 January 2016*, *Couvreur T.L.P.* 957 (WAG,YA); Kupe village Muanezum trail = Daniel Ajang’s Earthwatch rented area Mt 4.76°N, 9.666°E, *28 March 1996*, *Etuge M.* 1844 (K); Kupe village, 4.77°N, 9.688°E, *28 November 1999*, *Gosline W.G.* 234 (K); Njonji, 4.11°N, 9.016°E, *21 April 1997*, *Nning J.* 385 (K,YA); Cameroon Mountain, 4.12°N, 9.028°E, *20 June 2001*, *van Andel T.R.* 3732 (U,WAG).

### ﻿﻿ Cleistopholis

Taxon classificationPlantaeMagnolialesAnnonaceae

Pierre ex Engl., Nat. Pflanzenfam. Nachtr. I: 160, 1897

9D8C8AB6-0D0D-5D5E-8DD0-8AEC19D7F313

#### Type species.

﻿﻿﻿*Cleistopholisglauca* Pierre ex Engl. & Diels.

#### Description.

Trees, 15–35 m tall, d.b.h. 30–80 cm; stilt roots or buttresses absent, trunk white or brown. Indumentum of simple hairs when present, but species generally glabrous. Leaves: petiole 3–20 mm long, 1–3 mm in diameter, blade 4.5–31 cm long, 2–6.5 cm wide, elliptic to obovate to oblong, apex acuminate, base acute to rounded, discolorous, whitish below or concolorous; midrib sunken or flat; secondary veins 8 to 24 pairs; tertiary venation reticulate. Individuals bisexual; inflorescences ramiflorous on old or young foliate branches, axillary, occurring or not on a short peduncle. Flowers with 9 perianth parts in 3 whorls, 2 to 9 per inflorescence; pedicel 10–25 mm long; in fruit 15–35 mm long; bracts 2 to 3, all basal, 1–2 mm long; sepals 3, valvate, free, 2–3 mm long, triangular to ovate, apex acute, sometimes rounded, base truncate; petals free; outer petals longer than inner; outer petals 3, valvate, 7–20 mm long, 2–7 mm wide, oblong to elliptic to obovate to linear, apex acute to rounded to obtuse, base truncate; inner petals 3, imbricate, 2–4 mm long, 2–4 mm wide, ovate to suborbicular, apex acute to obtuse, base truncate; stamens 20 to 40, in 3 to 4 rows, ca. 1 mm long, broad; connective discoid, glabrous; staminodes absent; carpels free, 10 to 24, ca. 1 mm long, stigma flat to capitate, glabrous. Monocarps stipitate, stipes 1–50 mm long, 3 to 18 monocarps, 15–30 mm long, 10–25 mm in diameter, globose to ellipsoid to obovoid, apex rounded, smooth, bumpy or constricted around the seeds, glabrous; seeds 1 to 2, 12–25 mm long, 8–12 mm in diameter, ellipsoid; aril absent.

A genus with four accepted species, two widespread, one known only from Cameroon and Gabon and one from Equatorial Guinea and possibly Cameroon. Three (four?) species in Cameroun, none endemic.

#### Taxonomy.

None to date, but partial treated in this present work and Le Thomas (1969b).

### ﻿Key to the species of ﻿*Cleistopholis* in Cameroon (vegetative characters of ﻿*C.myristiciflora* are taken from label information):

**Table:** 

1	Trunk brown, petioles ca. 3 mm long; monocarps with stipes 49–50 mm long, thing, ca. 1 mm in diameter	﻿***C.myristiciflora***
–	Turk white, petioles generally 10–20 mm long (in ﻿*C.patens* can be as short as 3 mm too); monocarps with stipes 1–30 mm long, thick, 2–3 mm in diameter	**2**
2	Inflorescences with a distinct peduncule; monocarps ellipsoid to obovoid, drying smooth; lower leaf side glaucous, at least when fresh	﻿***C.glauca***
–	Inflorescences fasciculate, without a peduncule; monocarps globose to bilobed; lower leaf side not glaucous	**3**
3	Outer petals linear, 15–20 mm long; monocarps drying smooth, not bumpy; petiole 10–15 mm long	﻿***C.staudtii***
–	Outer petals oblong, elliptic or obovate, 7–12 mm long; monocarps bumpy, constricted around the seeds; petiole 3–12 mm long	﻿***C.patens***

### ﻿﻿﻿﻿ Cleistopholis glauca

Taxon classificationPlantaeMagnolialesAnnonaceae

Pierre ex Engl. & Diels, Monogr. Afrik. Pflanzen.-Fam. 6: 35, 1901

8E2E6925-19AD-55AC-B22E-347DC0342454

**Figure 21.**
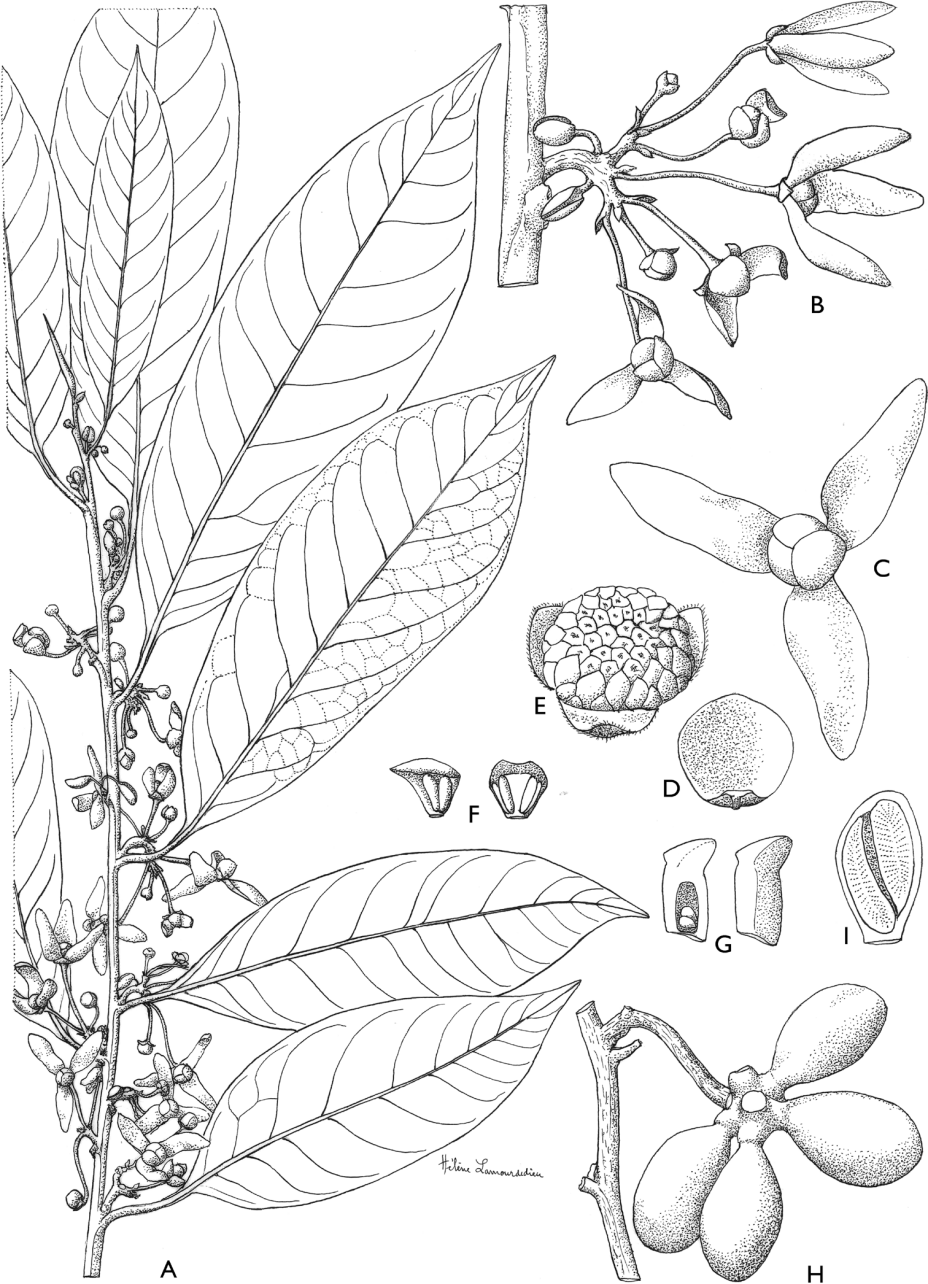
*Cleistopholisglauca***A** flowering branch, note long petioles **B** detail of inflorescence **C** flower, top view, note imbricate inner petals **D** internal petal, inner view **E** detail of receptacle, top view **F** stamen, front and side views **G** carpel, side view and view of ovules **H** fruit, note smooth monocarps **I** longitudinal section of seed **A–G** from *Le Testu 8786*; 8 from *Klaine 41*. Drawings by Hélène Lamourdedieu, Publications Scientifiques du Muséum national d’Histoire naturelle, Paris; modified from Le Thomas (1969b, pl. 15, p. 89).

Figs 21 , 23 ; Map 3C

= Cleistopholis grandiflora De Wild., Ann. Mus. Congo Belge, Bot. sér. 5, 1(1): 39, 1903. Type. Democratic Republic of the Congo. Kinshasa, Région de Kimuenza, *Gérard P. s.n.*, Oct 1900: lectotype, sheet here designated: BR[BR0000008820327]; isotype: BR[BR0000008820655].
= Cleistopholis bequaertii De Wild., Pl. Bequaert. i.; 464, 1922. Type. Democratic Republic of the Congo. Nord-Kivu, Walikale - Lubutu, *Bequaert J.C.C. 6624*, 15 Jan 1915: lectotype, sheet here designated: BR[BR0000008820402]; isotype: BR[BR0000008820396].

#### Type.

Gabon. Estuaire; Libreville, *Klaine T.-J. 376*, Apr 1896: holotype: B[B 10 0154073]; isotypes: K[K000198885, K000198884]; P[P00362650, P00362649, P00362652]; MPU[MPU011662].

#### Description.

Tree, 10–35 m tall, d.b.h. 80 cm; stilt roots or buttresses absent. Indumentum of simple hairs; old leafless branches glabrous, young foliate branches glabrous. Leaves: petiole 10–20 mm long, 1–2 mm in diameter, glabrous, grooved, blade inserted on the side of the petiole; blade 5–15 cm long, 2–5 cm wide, oblong to elliptic, apex acuminate, acumen 0.5–1.5 cm long, base decurrent to cuneate, subcoriaceous, **below glabrous when young and old**, above glabrous when young and old, **discolorous, whitish below**; midrib impressed, above glabrous when young and old, below glabrous when young and old; secondary veins 8 to 15 pairs, glabrous below; tertiary venation reticulate. Individuals bisexual; inflorescences ramiflorous on young and old leafless branches, axillary, **peduncule distinct 2–10 mm long**. Flowers with 9 perianth parts in 3 whorls, 2 to 8 per inflorescence; pedicel 10–18 mm long, ca. 1 mm in diameter, sparsely pubescent; in fruit 15–35 mm long, 2–3 mm in diameter, glabrous; bracts 1 to 3, all basal, basal bracts 1–2 mm long, 2 mm wide; sepals 3, valvate, free, ca. 2 mm long, ca. 2 mm wide, triangular to ovate, apex acute, base truncate, green, glabrous outside, glabrous inside, margins flat; petals free; outer petals 3, 10–15 mm long, 5–7 mm wide, **oblong to elliptic**, apex rounded, base truncate, green, margins flat, glabrous outside, glabrous inside; inner petals 3, imbricate, 2–4 mm long, 2–4 mm wide, **ovate to orbicular**, apex rounded, base truncate, green, margins flat, glabrous outside, glabrous inside; stamens 20 to 30, in 3 to 4 rows, ca. 1 mm long, broad; connective discoid, glabrous, green; staminodes absent; carpels free, 12 to 24, ovary ca. 1 mm long, stigma flat, glabrous. Monocarps stipitate, stipe 18–30 mm long, 3–4 mm in diameter; monocarps 3 to 8, 18–30 mm long, 10–15 mm in diameter, obovoid, apex rounded, **glabrous, finely warty, not bumpy**, green when ripe; seed (1 to) 2 per monocarp, 15–25 mm long, 10–12 mm in diameter, ellipsoid; aril absent.

#### Distribution.

Central Africa; from Cameroon to Democratic Republic of the Congo; in Cameroon known from South, Central, Littoral, South-West and East regions, with one collection from Adamaoua region.

#### Habitat.

A very common species, mainly growing as a pioneer species in disturbed areas and along forest margins. Altitude 100–1200 m a.s.l.

#### Local and common names known in Cameroon.

None recorded.

#### IUCN conservation status.

Least Concern (LC) (Cosiaux et al. 2019f).

#### Uses in Cameroon.

None recorded.

#### Notes.

Closely resembles ﻿*C.patens*, see below for differences.

#### Specimens examined.

**Adamaoua Region**: A 80 km au SO de Banyo-Plaine Tikar, 6.75°N, 11.82°E, *27 June 1969*, *Biholong M.* 219 (P,YA). **Central Region**: Feup (Yaoundé), 3.87°N, 11.52°E, *01 July 1917*, *Chevalier A.J.B.* 33473 (P,WAG); near Ebolbom village 3 km est of Ngoumou 2 km north west of Otélé, 3.59°N, 11.28°E, *02 May 2013*, *Couvreur T.L.P.* 430 (MPU,WAG,YA); Avom, 3.87°N, 11.52°E, *01 January 1935*, *Foury P.* 57 (P). **East Region**: Bertoua, 4.58°N, 13.68°E, *17 February 1960*, *Letouzey R.* 3036 (P,YA); Région de Moloundou, 2.05°N, 15.17°E, *01 August 1949*, *SRFK* 1372 (P,YA). **Littoral Region**: 18 km SEof Douala along road to Ndonga (=old to Edea), 4.05°N, 9.71°E, *20 August 1965*, *Leeuwenberg A.J.M.* 6467 (B,BR,C,GC,K,L,LUAI,MO,P,UC,WAG,YA). **South Region**: 20 km from Kribi Lolodorf road, 3.05°N, 10.05°E, *09 June 1969*, *Bos J.J.* 4784 (B,BR,K,LD,LM,MO,P,POZG,WAG,YA); on road between Campo and Kribi, 2.62°N, 9.847°E, *16 February 2012*, *Couvreur T.L.P.* 389 (WAG,YA); Rocher du Loup km 36 road Kribi-Campo, 2.61°N, 9.85°E, *06 January 1983*, *de Kruif A.P.M.* 1046 (MO,WAG,YA); Campo-Ma’an area 2.73°N, 9.873°E, *13 August 2001*, *van Andel T.R.* 3846 (KRIBI,WAG,YA). **South-West Region**: Mungo River F.R., 4.78°N, 9.566°E, *02 December 1999*, *Cheek M.* 10229 (K,MO,P,WAG,YA); Ntali, 5.25°N, 9.576°E, *01 December 2000*, *Etuge M.* 4873 (K); Nyandong-forest above village, 4.98°N, 9.585°E, *20 March 2003*, *Etuge M.* 4917 (K); Forest and forest relictss near Mundemba, 4.96°N, 8.916°E, *16 January 1985*, *Thomas D.W.* 4200 (P); Near Mundemba town, 4.96°N, 8.916°E, *12 May 1986*, *Thomas D.W.* 6121 (MO,P,WAG,YA).

### ﻿﻿﻿﻿ Cleistopholis myristiciflora

Taxon classificationPlantaeMagnolialesAnnonaceae

Diels & Mildbr., Bot. Jahrb. Syst. 53(3–5): 439 (1915)

601D8560-2174-5D32-A926-365159230195

Map 3D

#### Type.

Cameroon. South Region(?) or Equatorial Guinea. Río Muni; Campo-Gebiet; Bebao[i?], Weg nach Olonga [Manga?], *Tessmann*, *G. 767*, 6 Jan. 1909: holotype: B[B 10 0154074].

#### Description.

Tree, to 19 m tall, d.b.h. to 16 cm; stilt roots or buttresses absent, **trunk brown**. Indumentum of simple hairs (?); old leafless branches glabrous, young foliate branches pubescent (?). Leaves: **petiole ca. 3 mm long, ca. 1 mm in diameter**, pubescent (?), grooved, blade inserted on the side of the petiole; blade 8–12 cm long, ca. 4 cm wide, elliptic to oblong, apex acuminate, acumen ca. 1.5 cm long, base cuneate, papyraceous, below pubescent when young (?), glabrous when old, above glabrous when young and old, concolorous; midrib impressed, above glabrous when young and old, below pubescent when young (?), glabrous when old; secondary veins 10 to 13 pairs, glabrous below; tertiary venation reticulate. Individuals bisexual; inflorescences ramiflorous on young and old leafless branches, axillary, **peduncule generally absent**. Flowers with 9 perianth parts in 3 whorls, 4 to 9 per inflorescence, pedicel 15–27 mm long, ca. 1 mm in diameter, sparsely pubescent (?); in fruit ca. 20 mm long, 2–3 mm in diameter, glabrous (?); bracts not seen (soon falling ?); sepals 3, valvate, free, 1.5–2 mm long, ca. 1.5 mm wide, circular, apex rounded, base rounded, color unknown, pubescence not observed, margins flat; petals free; outer petals 3, valvate, 5–7 mm long, 2–3 mm wide, **broadly elliptic to oblong**, apex rounded, base truncate, color unknown, margins flat or wavy when dry, pubescence not observed; inner petals 3, imbricate (?), dimensions and shape not observed; stamens number not counted, row number not counted, ca. 1 mm long, broad; connective discoid, pubescence not observed, color unknown; staminodes absent (?); carpels free, 10 to 15 (?, possibly more based on the number of monocarps counted), ovary ca. 1.5 mm long, stigma flat, glabrous. Monocarps stipitate, **stipes 49–50 mm long, ca. 1 mm in diameter**; monocarps 17 to 18, 10–15 mm long, 10–15 mm in diameter, globose, apex rounded, **glabrous (?), bumpy**; seeds not seen.

#### Distribution.

Equatorial Guinea and Cameroon (?); if present in Cameroon then from South region.

#### Habitat.

A rare species, in primary submontane tierra firme forest. Altitude 750–850 m a.s.l. (altitude in Equatorial Guinea).

#### Local and common names known in Cameroon.

Akom (Equatorial Guinea) (Guinea López 1946).

#### IUCN conservation status.

No assessed, but probably CR.

#### Uses in Cameroon.

None recorded.

#### Notes.

﻿﻿﻿*Cleistopholismyristiciflora* (initially known only from the type; *Tessmann 767* (B, but see below)) was originally described as being from Cameroon (Diels 1915) with the type locality written as “Kamerun: Campo-Gebiet; Bebao[i?], Weg nach Olonga [Manga?]”. This locality information, however, is also found on several other of Günther Tessmann (1884–1969) specimens (e.g. *779*, *800*), collected between end 1908 and early 1909 but are suggested to be from Equatorial Guinea rather than Cameroon. Tessmann is suggested to have collected around 700 specimens in Equatorial Guinea between the island of Bioko and mainland Río Muni (Fero 2013). Le Thomas (1969b) also suggested this species is from Equatorial Guinea and is cited in the check list of plants in “Ensayo geobotánico de la Guinea continental Española” (Guinea López 1946). It was however not cited in the “Les arbres de la Guinée Équatoriale” (Wilks et al. 2000). Fero indicates that they did not locate any specimens of this species in Equatorial Guinea in the herbaria of BATA, LISU, MA and WAG, but suggest it should be present (Fero 2013). In Tessmann’s book about the Pangwe culture (Tessmann 1913), the name “Bébai” is found several times and is suggested to be at the border between Equatorial Guinea and Cameroon (page XXI). There is a map in the book (page 1) showing a village named Bébai, almost exactly on the border between Equatorial Guinea and Cameroon. Thus, evidence for its presence in Cameroon is still doubtful with no recent collections in Cameroon but two recent ones from Equatorial Guinea (see below). We include it in our taxonomic treatment as tentatively occurring in Cameroon and provide a tentative coordinate for Bebai in the map of this species.

The taxonomic affinities of this species were unclear for some time but suggested to be conspecific with either ﻿*C.patens* or ﻿*C.staudtii*. Le Thomas (1969b) notes that it has morphological characters of both ﻿*C.patens* (shape of the petals and number of carpels) and ﻿*C.staudtii* (leaf shape and venation), the later also suggested by Diels and Mildbrand (1915).

Recently, we located two specimens collected from Monte Alén in Guinea Equatorial (*Senterre & Obiang 2939, 3699*, BRLU) which appear to belong to *C.myristicifolia* (identified as such by B. Senterre). The leaves match the description and the type specimen, especially the shape and the length of the petiole being shorter (ca. 3 mm) than in the other species (> 3 mm). One specimen (*Senterre & Obiang 2939*), is in fruit. This single fruit is morphologically quite different than those of the other species in ﻿*Cleistopholis*. It is partially described here for the first time. The main difference is the length and diameter of the stipes being much longer and thinner than those from the other three species (49–50 mm long and ca. 1 mm in diameter versus 1–30 mm and 2–3 m in diameter). The number of monocarps appears to be higher with 17 to 18 counted in *Senterre & Obiang 2939*, versus 3 to 8 in the other species. These observations strongly support the hypothesis that ﻿*C.myristiciflora* is indeed a distinct and valid species. In terms of its ecology, observations from *Senterre & Obiang* suggest it to occur in primary submontane rain forests, occurring on gentle slopes or top of small mountains.

One specimen collected from southern coastal Gabon (*Bergen 217* [WAG.1379499, WAG.1379500]) at 10 m a.s.l. was identified as ﻿*C.myristiciflora* by M. Fero. It is true that the inflorescences and flowers could potentially match, but the size of the tree (8 m) and leaves are different having a long petiole (> 3 mm). The ecology is also different than described above occurring along the coast on laterite soil. For now we do not consider this specimen as part of ﻿*C.myristiciflora* and *Bergen 217* could potentially represent an undescribed Gabonese coastal species, as has been done recently in other Annonaceae genera such as ﻿﻿*Greenwayodendronlittorale* Lissambou, Dauby & Couvreur (Lissambou et al. 2018).

### ﻿﻿﻿﻿ Cleistopholis patens

Taxon classificationPlantaeMagnolialesAnnonaceae

(Benth.) Engl. & Diels, Monogr. Afrik. Pflanzen.-Fam. 6: 35, 1901

03427571-D5C9-559C-96DD-C5DE2ADFBFF2

**Figure 22.**
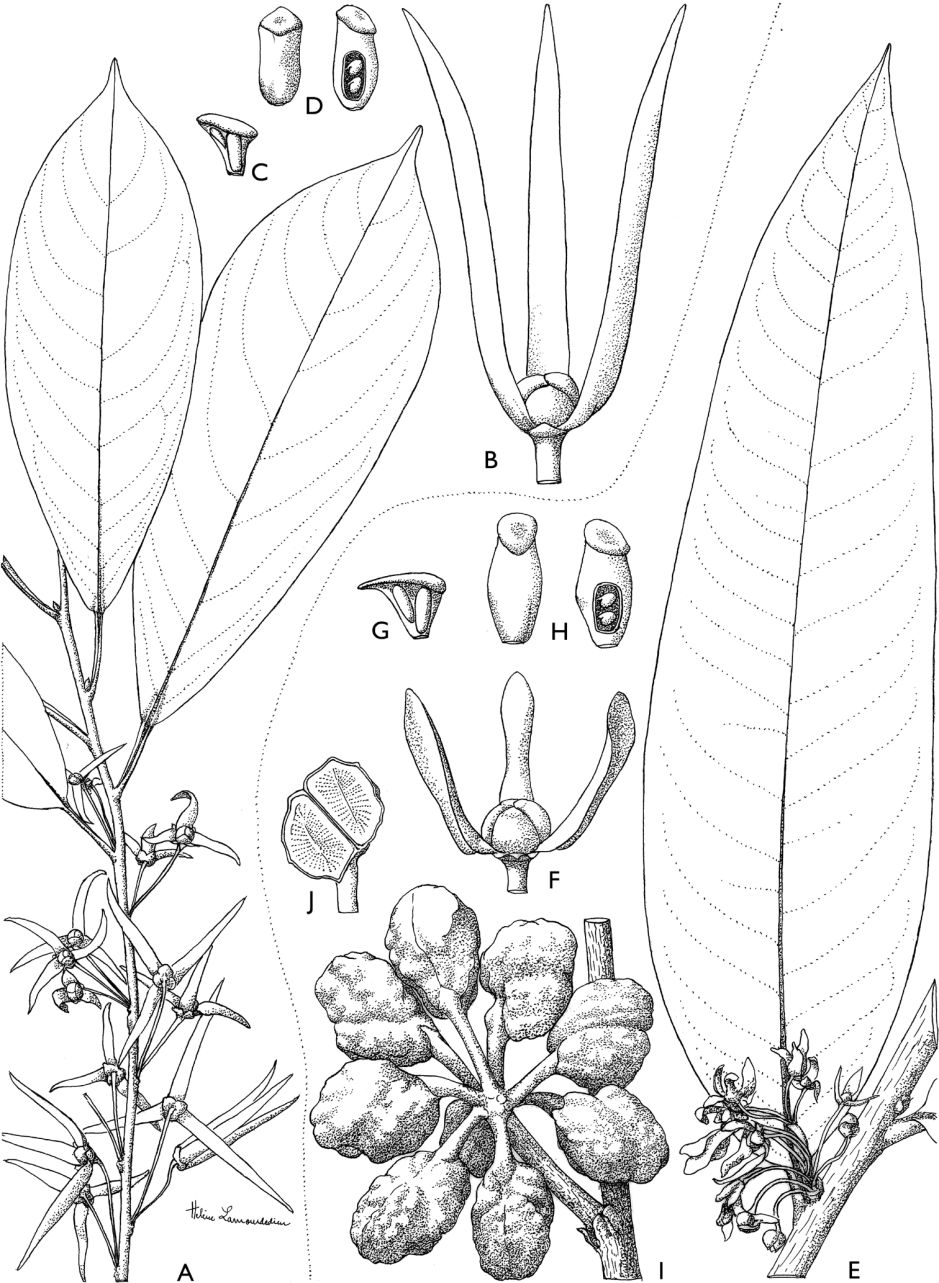
*Cleistopholisstaudtii***A** flowering branch **B** flower, side view, note linear outer petals **C** stamen, side view **D** carpel, side view and detail of ovules. *Cleistopholispatens***E** flowering branch **F** flower, side view **G** stamen, side view **H** carpel, side view and detail of ovules **I** fruit; note bumpy monocarps **A–D** from *Letouzey 4149***E–J** from *Chevalier 22379*. Drawings by Hélène Lamourdedieu, Publications Scientifiques du Muséum national d’Histoire naturelle, Paris; modified from Le Thomas (1969b, pl. 16, p. 93).

**Figure 23.**
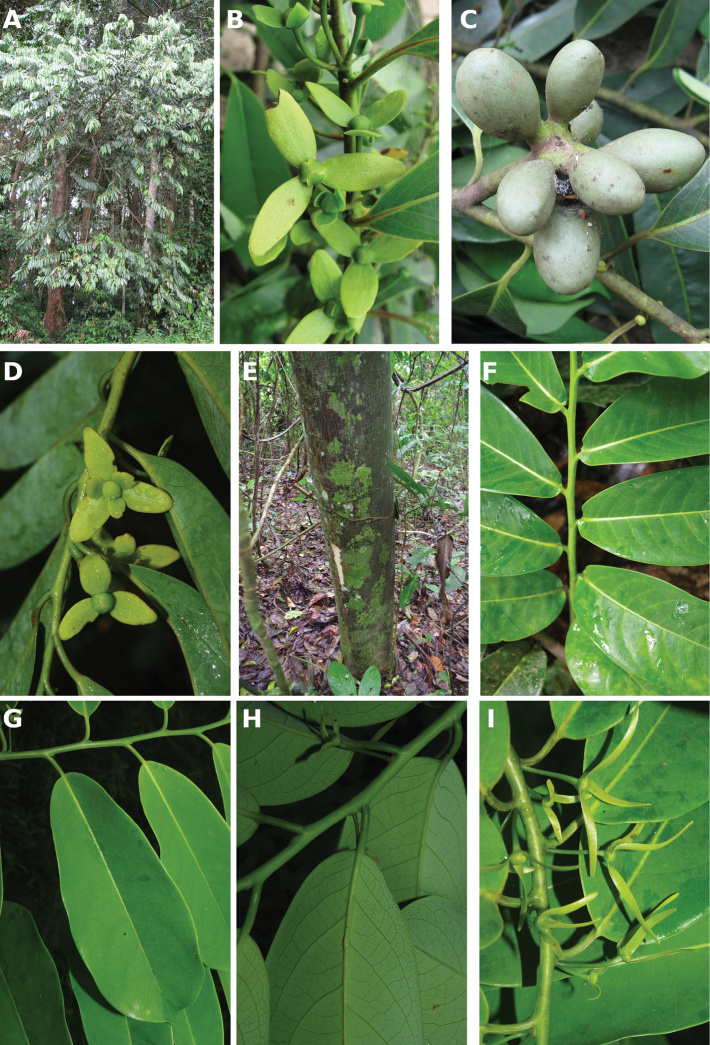
*Cleistopholisglauca***A** habit, note long drooping branches **B** flowers, top view, inner petals imbricate, not opened yet **C** fruit, note smooth monocarps **D** flowering branch, inner petals open revealing receptacle. *Cleistopholispatens***E** trunk **F** base of leaf blades, upper view. *Cleistopholisstaudtii***G** leaf, top view, note long petioles **H** leaf, lower view **I** flowering branch, note long linear outer petals **A–C***Sosef 2036*, Gabon **D***Couvreur 389*, Ebodjé, Cameroon **E, F***Couvreur 1202*, Maséa, Cameroon **G–I***Couvreur 570*, Gabon. Photos Thomas L.P. Couvreur.

Figs 22 , 23 ; Map 3E

≡ Oxymitra patens Benth., Trans. Linn. Soc. London 23(3): 472, 1862.
= Cleistopholis brevipetala Exell, J. Bot. 70 (Suppl. 1): 208, 1932. Type. Angola. Cabinda, *Gossweiler J. 6082*, 31 Dec 1915: holotype: BM[BM000546899]; isotypes: COI[COI00004874]; LISC[LISC000068, LISC000071, LISC000069, LISC000070].
= ﻿﻿Cleistopholis klaineana Pierre ex Engl. & Diels, Monogr. Afrik. Pflanzen.-Fam. 6: 35, 1901, Cleistopholis patens var. klaineana Pellegr., Bull. Soc. Bot. France: 57, 1949.. Type. Gabon. Estuaire, Libreville, Klaine T.J. 345, 1896: holotype: B[B 10 0154075]; isotypes: P[P00362660, P00362656].
= Cleistopholis lucens De Wild., Pl. Bequaert. i. 465, 1922. Type. Democratic Republic of the Congo. Nord-Kivu, entre Walikale et Lubutu, *Bequaert J. 2774*, 22 Fev 1922: holotype: BR[BR0000008820426].
= Cleistopholis pynaertii De Wild., Bull. Jard. Bot. État Bruxelles 4: 387, 1914. Type. Democratic Republic of the Congo. Equateur, Eala, Pynaert L.A. 1083, 1 Fev 1907: lectotype, sheet here designated: BR[BR0000008820419]; isotypes: BR[BR0000008820761, BR0000008820754].
= Cleistopholis verschuereni De Wild., Bull. Jard. Bot. État Brux. 4: 387, 1914. Type. Democratic Republic of the Congo. Manie Malela, *Verschueren R. 358*, Fev 1913: lectotype, sheet here designated: BR[BR0000008820389]; isotype: BR[BR0000008820433].

#### Type.

Sierra Leone. Northern Region; Bagroo River, *Mann G. 828*, Apr 1861: lectotype, sheet here designed: K[K000880416]; isotypes: K[K000880417]; P[P00362653].

#### Description.

Tree, up to 30 m tall, d.b.h. up to 60 cm; stilt roots or buttresses absent. Indumentum of simple hairs; old leafless branches glabrous, young foliate branches glabrous. Leaves: petiole (3–)10–20 mm long, ca. 2 mm in diameter, glabrous, grooved, blade inserted on the side of the petiole; blade 4.5–31 cm long, 2.5–6 cm wide, **oblong to narrowly oblong or oblanceolate to narrowly oblanceolate**, apex acute to acuminate, acumen 1–1.5 cm long, base cuneate to rounded, coriaceous, above glabrous when young and old, **shiny when dry, below glabrous when young and old, green**, concolorous; midrib impressed, above glabrous when young and old, below glabrous when young and old; secondary veins 10 to 24 pairs, glabrous below; tertiary venation reticulate. Individuals bisexual; inflorescences ramiflorous on young or old leafless branches, axillary, **peduncle absent**. Flowers with 9 perianth parts in 3 whorls, 2 to 9 per inflorescence; pedicel 10–25 mm long, ca. 1 mm in diameter, glabrous; in fruit 15–30 mm long, 2–3 mm in diameter, glabrous; bracts 1 to 3, all basal, 1–2 mm long, ca. 2 mm wide; sepals 3, valvate, free, 2–3 mm long, ca. 2 mm wide, triangular to ovate, apex acute, base truncate, green, glabrous outside, glabrous inside, margins flat; petals free; outer petals longer than inner; outer petals 3, 7–12 mm long, 2–4 mm wide, **obovate to oblong**, apex obtuse, base truncate, green, margins flat, glabrous outside, glabrous inside; inner petals 3, imbricate, 3–4 mm long, 2–3 mm wide, **ovate to suborbicular**, apex rounded, base truncate, green, margins flat, glabrous outside, glabrous inside; stamens 25 to 30, in 3 to 4 rows, ca. 1 mm long, broad; connective discoid, pubescent, green; staminodes absent; carpels free, ca. 10, ovary ca. 1 mm long, stigma capitate, glabrous. Monocarps stipitate (sometimes shortly so), **stipes 3–12 mm long**, **3–4 mm in diameter**; monocarps 3 to 6, 15–23 mm long, 11–25 mm in diameter, ellipsoid to globose, apex rounded, glabrous, **finely warty, constricted around seeds, bumpy**; seeds 1 to 2 per monocarp, ca. 12 mm long, 8–9 mm in diameter, ellipsoid; aril absent.

#### Distribution.

In West Africa, Senegal, Sierra Leone to Nigeria, and Central Africa from Cameroon to Uganda; in Cameroon known from South, Central, Littoral, South-West and East regions.

#### Habitat.

A very common species, mainly growing as a pioneer species in disturbed areas and along rain forest margins. Altitude 0–600 m a.s.l.

#### Local and common names known in Cameroon.

avom, sobu (pygmée Bibaya) (Letouzey 1964).

#### IUCN conservation status.

Least Concern (LC) (Cosiaux et al. 2019g).

#### Uses in Cameroon.

***medicine***: bark used as pain-killers, against pulmonary troubles; leaves as vermifuges, fabrifuges; ***construction***: building materials, furniture; ***dyes and tannins***: astringents, insecticides, arachnicides; ***products***: fiber, pulp, wood fire, exudations-gums, resins, farming, forestry, hunting and fishing apparatus, household, domestic and personal items, pastimes-carving, musical instruments; ***social***: religion, superstitions, sayings, aphorisms.

#### Notes.

﻿﻿﻿*Cleistopholispatens* closely resembles ﻿*C.glauca* by the shape and aspect of the flowers and the overall vegetative characters. However, both species differ by their inflorescences being pedunculate in ﻿*C.glauca* versus sessile, the leaves green below versus glaucous in ﻿*C.glauca* and the monocarps being bumpy and constricted around the seeds when dry versus to smooth and not bumpy when dry in ﻿*C.glauca*.

#### Specimens examined.

**Central Region**: Avome, 3.87°N, 11.52°E, *13 August 1945*, *Aubréville A.* 41 (P). **East Region**: 76 km south of Yokadouma 30 km after Ngato 15 km after river ALPICAM ‘base de vie’ then 40 km on forestry road starting 4 km before Maséa village, 3.15°N, 14.72°E, *05 March 2019*, *Couvreur T.L.P.* 1202 (MPU,WAG,YA); Dja Reserve, 3.17°N, 13.18°E, *07 October 1994*, *Fogiel M.K.* 947 (P); Deng Deng, 5.2°N, 13.51°E, *01 July 1939*, *Jacques-Félix H.* 4630 (P,WAG); Rives de la Boumba à 14 km à l’WSW de Kinsassa village situé à 65 km au NNE de Moloundou sur route de Yokadouma 2.58°N, 15.26°E, *07 March 1971*, *Letouzey R.* 10523 (P,YA). **Littoral Region**: km 19 Loum-Yabassi 3 km N of Solé, 4.61°N, 9.8°E, *30 December 1971*, *Leeuwenberg A.J.M.* 9032 (YA,WAG). **South-West Region**: Southern Bakundu Forest Reserve, 4.55°N, 9.433°E, *15 June 1960*, *Adebusuyi J.K.* 44049 (WAG); S Bakundu Forest 3 km from Kindongi Camp (8 km from road), 4.49°N, 9.374°E, *02 May 1972*, *Leeuwenberg A.J.M.* 9785 (B,BR,C,K,M,MO,P,WAG,YA); 2 km W of km 21 Kumba-Victoria road, 4.46°N, 9.483°E, *04 May 1972*, *Leeuwenberg A.J.M.* 9828 (B,BR,C,K,LD,M,MO,P,WAG,YA); Bibundi, 4.21°N, 8.988°E, *08 November 1928*, *Mildbraed G.W.J.* 10640 (K); Korup National Park, 4.88°N, 8.783°E, *22 July 1983*, *Thomas D.W.* 2329 (MO,P,WAG,YA); Limbe (Victoria), 4.01°N, 9.133°E, *25 October 1997*, *van der Burgt X.M.* 219 (KRIBI,WAG).

### ﻿﻿﻿﻿ Cleistopholis staudtii

Taxon classificationPlantaeMagnolialesAnnonaceae

(Engl. & Diels) Engl. & Diels, Monogr. Afrik. Pflanzen.-Fam. 6: 35, 1901

FAFCD672-D1FD-5685-ACA1-A9BE100D4F62

Figs 22 , 23 ; Map 3F

≡ Oxymitra staudtii Engl. & Diels, Notizbl. Königl. Bot. Gart. Berlin 2: 297, 1899.
= Polyalthia (?) *crassipes* Engl. Bot. Jahrb. Syst. 34: 477, 1907. Type. Cameroon. South Region, Bipindi, Zenker G.A. 2454a, 1902: holotype B destroyed, lectotype here designated: P[01988941]; isolectotypes: MO[MO-2500050]; P[P01988940, P01988942].

#### Type.

Cameroon. South-West Region; Johann-Albrechtshöhe [Kumba], *Staudt A. 957*, 1896: holotype: B[B 10 0154076]; isotypes: BM[BM000546890]; K[K000105343]; LE[LE00012452]

#### Description.

Tree, 15–30 m tall, d.b.h. up to 30 cm; stilt roots or buttresses absent. Indumentum of simple hairs; old leafless branches glabrous, young foliate branches glabrous. **Leaves: petiole 10–20 mm long, ca. 3 mm in diameter**, glabrous, grooved, blade inserted on the side of the petiole; blade 9–17 cm long, 3–6.5 cm wide, obovate, oblong to elliptic, apex acuminate, acumen ca. 1 cm long, base rounded to acute, coriaceous, below glabrous when young and old, above glabrous when young and old, discolorous, whitish below, midrib impressed, above glabrous when young and old, below glabrous when young and old; secondary veins 10 to 12 pairs, glabrous below; tertiary venation reticulate. Individuals bisexual; inflorescences ramiflorous on young and old leafless branches, axillary, **peduncle absent**. Flowers with 9 perianth parts in 3 whorls, 2 to 3 per inflorescence; pedicel 12–20 mm long, ca. 1 mm in diameter, glabrous; in fruit 15–35 mm long, ca. 3 mm in diameter, glabrous; bracts 1 to 3, all basal, 1–2 mm long, 2 mm wide; sepals 3, valvate, free, ca. 2 mm long, ca. 2 mm wide, triangular to ovate, apex acute to rounded, base truncate, green, glabrous outside, glabrous inside, margins flat; petals free, outer petals longer than inner; outer petals 3, 15–20 mm long, 2–3 mm wide, **linear**, apex acute, base truncate, green, margins flat, glabrous outside, glabrous inside; inner petals 3, imbricate, 2–2.5 mm long, 3–3.5 mm wide, ovate, apex acute, base truncate, claw mm long, green, margins flat, glabrous outside, glabrous inside; stamens 30 to 40, in 3 to 4 rows, ca. 1 mm long, broad; connective discoid, pubescent, green; staminodes absent; carpels free, 15 to 22, ca. 1 mm long, stigma capitate, glabrous. **Monocarps stipitate to sessile**, stipes when present to 10 mm long, **3–4 mm in diameter**, **3 to 8 monocarps**, 15–20 mm long, 12–15 mm in diameter, globose, apex rounded, glabrous, striate, bumpy, constricted around seeds; seeds 1 to 3 per monocarp, 10–15 mm long, 7–10 mm in diameter, ellipsoid; aril absent.

#### Distribution.

From Cameroon to Gabon; in Cameroon known from South, Central, Littoral and South-West regions.

#### Habitat.

A common species when present, in lowland rain forests in primary or secondary habitats. Altitude 50–1000 m a.s.l.

#### Local and common names known in Cameroon.

None recorded.

#### IUCN conservation status.

Least Concern (LC) (Cosiaux et al. 2019h).

#### Uses in Cameroon.

***construction***: bark for building materials (Tessmann 1913).

#### Notes.

Easily distinguished from ﻿*C.glauca* and ﻿*C.patens* by its linear and acute outer petals, in contrast to short and rounded outer petals in the latter two. It is quite hard to distinguish these species based on sterile material alone.

#### Specimens examined.

**Central Region**: Ca 50 km NW of Eséka W of Yaoundé, 3.65°N, 10.78°E, *25 November 1963*, *de Wilde W.J.J.O* 1342 (B,BR,C,DES,L,LD,MO,P,U,WAG,YA); Ottotomo Forest Reserve, 3.65°N, 11.31°E, *Service Forestier du Cameroun* 32 (P). **South Region**: ca 15 km from Kribi 1 km S of Ebolowa road, 2.85°N, 10.01°E, *20 February 1970*, *Bos J.J.* 6383 (P,WAG); Station de cacaoyer de N’koemvone 14 km On the road from Ebolowa to Ambam, 2.81°N, 11.13°E, *02 February 1975*, *de Wilde J.J.F.E* 7947 (B,BR,K,MO,P,U,WAG,YA); Près de Bella (45 km NE de Kribi), 3.25°N, 10.2°E, *25 January 1962*, *Letouzey R.* 4149 (P,YA); Bipindi, 3.08°N, 10.41°E, *01 January 1900*, *Zenker G.A.* 2264 (L,P,WAG); Bipindi, 3.08°N, 10.41°E, *01 January 1902*, *Zenker G.A.* 2454 (L,P,WAG); Bipindi, 3.08°N, 10.41°E, *01 January 1902*, *Zenker G.A.* 2495 (L,P,WAG); Bipindi, 3.08°N, 10.41°E, *01 January 1913*, *Zenker G.A.* 4669 (L,P); Bipindi, 3.08°N, 10.41°E, *01 January 1913*, *Zenker G.A.* 4880 (L,P). **South-West Region**: Ekundu Kundu, 5.16°N, 8.874°E, *11 April 1996*, *Cable S.* 1825 (K,YA); Kupe Mount Path to Kupe Rock, 4.75°N, 9.686°E, *24 November 1995*, *Cheek M.* 7915 (K,P,WAG); Muanezum trail from Kupe village towards Daniel Ajang’s area 4.77°N, 9.708°E, *18 July 1996*, *Etuge M.* 2884 (K,MO,P,WAG); Just outside Kupe village going north, 4.77°N, 9.688°E, *29 November 1999*, *Gosline W.G.* 240 (K,MO,P,WAG,YA); Korup National Park, 5.06°N, 8.855°E, *05 December 1997*, *Kenfack D.* 984 (MO,P,WAG); Environs of Kumba farmed land and scrub with scattered trees, 4.63°N, 9.433°E, *01 March 1984*, *Thomas D.W.* 3271 (MO,WAG,YA).

### ﻿﻿ Dennettia

Taxon classificationPlantaeMagnolialesAnnonaceae

Baker f., Cat. pl. Oban 5, t. 2. 1913

8AB5BAC0-0D2A-5FB7-AFB3-DFC428576A4C

#### Type species.

﻿﻿﻿*Dennettiatripetala* Baker f.

#### Description.

Same as species.

A genus with a single widespread species from West Africa (Sierra Leone to Nigeria) and in Cameroon. One species in Cameroon, not endemic.

﻿*Dennettia* was first described by Baker (1913) based on the bisexual flowers and inflorescences occurring on foliate branches. However, Kenfack et al. (2003) recombined the name ﻿*Dennettia* into ﻿*Uvariopsis* as ﻿*Uvariopsistripetala* (Baker f.) G.E. Schatz based on a number of morphological characters. A molecular phylogenomic analysis of tribe Monodoreae (where this genus bellows) confirmed that ﻿﻿﻿*Dennettiatripetala* did not cluster with other species of ﻿*Uvariopsis* and should be regarded as a genus of its own (Dagallier et al. in prep).

### ﻿﻿﻿﻿ Dennettia tripetala

Taxon classificationPlantaeMagnolialesAnnonaceae

Baker f., Cat. Pl. Oban: 5, 1913

0854F646-6D0A-5F4A-AA9E-692FD02494E6

Fig. 24 ; Map 3G

≡ Uvariopsis tripetala (Baker f.) G.E.Schatz, Novon 13(4): 447, 2003.

#### Type.

Nigeria. Edo State; Benin City, *Dennett R.E. 44*, 1 Jan 1907: lectotype, designated by Kenfack et al. (2003), p. 447; sheet here designated: K[K000040959]; isolectotypes: K[K000040961]; S[S-G-9774].

#### Description.

Shrub to small tree, 2–5 m tall, d.b.h. unknown; stilt roots or buttresses absent. Indumentum of simple hairs; old leafless branches glabrous, young foliate branches glabrous to sparsely pubescent. Leaves: petiole 2–5 mm long, 1–2 mm in diameter, glabrous, slightly grooved, blade inserted on top of the petiole; **blade 7.2–15.5 cm long, 3–6.8 cm wide**, elliptic, apex attenuate to acuminate, acumen 0.6–1.3 cm long, base acute to decurrent, subcoriaceous, below glabrous when young and old, above glabrous when young and old; midrib sunken or flat, above glabrous when young and old, below glabrous when young and old; secondary veins 5 to 10 pairs per side, glabrous above; tertiary venation reticulate. Individuals bisexual, inflorescences ramiflorous on old leafless branches, axillary. Flowers with **6 perianth parts in 2 whorls**, 1 to 4 per inflorescence; **pedicel 4–9 mm long**, 1–2 mm in diameter, pubescent; in fruit 5–15 mm long, 2–3 mm in diameter, glabrous to pubescent; bracts 1 to 3, all basal 0.5–2 mm long, 1.5–2 mm wide; **sepals 3 (rarely 2)**, valvate, basally fused, 1–3 mm long, 1.5–4 mm wide, triangular, apex acute, base truncate, brown, pubescent outside, glabrous inside, margins flat; **petals 3 (rarely 4, see notes), free, 7–14 mm** long, 6–10 mm wide, broadly ovate, apex obtuse, base truncate, margins flat, pubescent outside, glabrous inside; stamens ca. 150, in 10 to 20 rows, 0.5–1 mm long, oblong; connective reduced or absent, glabrous; staminodes absent; carpels free, 8 to 30, ovary ca. 2–4.5 mm long, stigma globose, pubescent. Monocarps stipitate, stipes 1–3 mm long, ca. 1 mm in diameter; monocarps 1 to 8, 11–32 mm long, 5–15 mm in diameter, ovoid to oblong, apex rounded, glabrous to sparsely pubescent, verrucose, wrinkled; seeds 4 to 12 per monocarp, 4–10 mm long, 11–14 mm in diameter, ellipsoid; aril absent.

**Figure 24.**
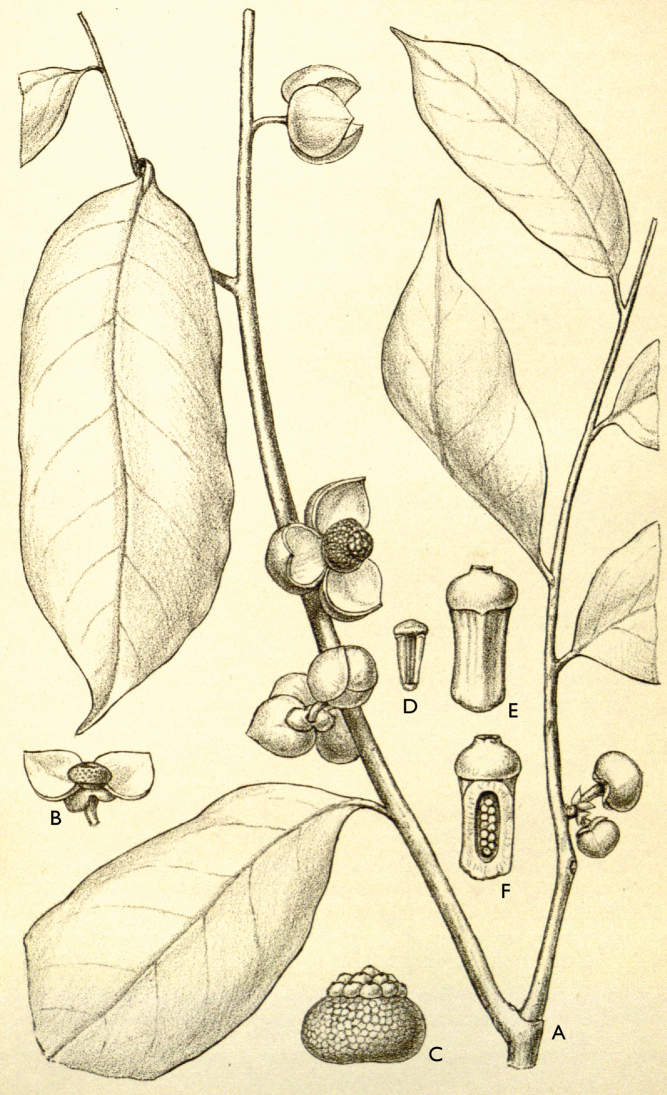
*Dennetiatripetala***A** flowering branch **B** flower, petals remove, 1 sepal removed, showing receptacle **C** receptacle with stamens and stigmas **D** stamen, front view **E** carpel, front view **F** carpel, longitudinal section showing ovules. Material of drawings unknown. Author of drawings unknown, taken from Baker (1913; plate 2).

#### Distribution.

Mainly a West African species from Sierra Leone to Cameroon; in Cameroon known from the South-West region.

#### Habitat.

an uncommon species; in lowland to premontane primary or secondary rain forests. Altitude 0–1000 m a.s.l.

#### Local and common names known in Cameroon.

Bushpèpè (*Westphal 9932*, Pidgin English(?)); Pepperfruit (english)

#### IUCN conservation status.

Least Concern (LC) (Harvey-Brown 2019f) (as ﻿*Uvariopsistripetala*).

#### Uses in Cameroon.

***food***: fruit for sauces, condiments, spices, flavorings (pepper); ***medicine***: cough, fever, toothache, diarrhea, diabetes, nausea (Iseghohi 2015).

#### Notes.

﻿﻿﻿*Dennettiatripetala* is unique in Cameroonian by being a tree with bisexual flowers having three sepals and three petals. This species resembles ﻿﻿﻿*Uvariopsiscongensis* and ﻿﻿*Uvariopsiszenkeri* (﻿*Uvariopsis* being a genus were ﻿*Dennettia* was once part off, see above, Kenfack et al. (2003)) by the smaller dimensions of its leaves (7–18 cm long and 3–6 cm wide), and the small and short pedicellate (< 1 cm) flowers. ﻿﻿﻿*Dennettiatripetala*, however, differs from these two species by its bisexual flowers, whereas all other species of ﻿*Uvariopsis* in Cameroon have unisexual flowers. Only one other species of ﻿*Uvariopsis* is bisexual (*U.bisexualis* Verdc.) which occurs in East Africa (Verdcourt 1971a).

It has been reported that some ﻿*D.tripetala* specimens had 2 sepals and 4 petals (Kenfack et al. 2003), however, a recent morphological study did not find any evidence for that (Dagallier et al. in prep). However, we cannot exclude that it might be a rare event.

#### Specimens examined.

**South-West Region**: Missellele, 4.12°N, 9.448°E, *Box H.E.* 3556 (BM,K); Limbe (Victoria), 4.07°N, 9.189°E, *01 April 1929*, *Maitland T.D.* 626 (K); Buea area 4.2°N, 9.183°E, *01 January 1930*, *Maitland T.D.* s.n. (K[K000105532]); Likomba-Pflanzung 15–35 km NE von Victoria [Limbe], 4.1°N, 9.333°E, *18 October 1928*, *Mildbraed G.W.J.* 10515 (K); Ngandjo on Kumba Mbonge road, 4.55°N, 9.4°E, *25 February 1986*, *Thomas D.W.* 5661 (K); Market of Victoria, 4.01°N, 9.2°E, *04 April 1978*, *Westphal E.* 9932 (WAG).

### ﻿﻿ Duguetia

Taxon classificationPlantaeMagnolialesAnnonaceae

A. St.-Hil., Fl. Bras. Merid. (A. St.-Hil.), 1: 35, 1825

30342911-F062-5BC4-B8AE-E7A9A8F5EE01

= Pachypodanthium Engl. & Diels, Notizbl. Königl. Bot. Gart. Berlin 3: 55, 1900.

#### Type species.

﻿*Duguetialanceolata* A.St.-Hil. (a Brazilian species).

#### Description.

Trees, 8–50 m tall, d.b.h. up to 50 cm; stilt roots or buttresses absent. Indumentum of stellate or fasciculate hairs. Leaves: petiole 1–10 mm long, 2–6 mm in diameter; blade 7–34 cm long, 3–8 cm wide, ovate to elliptic to obovate, apex acuminate to acute, acumen 0.5–1 cm long, base cordate to acute, discolorous, whitish below or concolorous; midrib sunken or flat; secondary veins 8 to 25 pairs; tertiary venation reticulate. Individuals bisexual; inflorescences ramiflorous on young and old leafless branches, leaf opposed or supra-axillary. Flowers with 9 perianth parts in 3 whorls, 2 to 5 per inflorescence; pedicel 3–22 mm long; in fruit 1–50 mm long; bracts 2, one basal and one upper in the lower half of pedicel, basal bract 7–12 mm long, upper bract similar than basal one; sepals 3, valvate, free, 6–15 mm long, apex acute, base truncate; petals free, outer petals longer than inner to sub equal; outer petals 3, valvate, free, 10–30 mm long, 4–10 mm wide, elliptic to ovate, apex acute to acuminate, base truncate; inner petals 3, imbricate, free, 4–20 mm long, 4–9 mm wide, elliptic to ovate to obovate, apex acute to acuminate, base truncate; stamens numerous, 1–2 mm long, broad; connective discoid; staminodes absent; carpels free, 50 to 125, 1.5–3.5 mm long, stigma globose. Fruit pseudosyncarpous; carpels sessile, connate or free, 60 to 125 carpels, 15–55 mm long, 7–30 mm in diameter, globose to ovoid to ellipsoid, apex domed-shaped to acute to apiculate; seed 1, 7–20 mm long, 4–13 mm in diameter, obovoid to ellipsoid; aril present, rudimentary.

A genus of 94 species, with a disjunct distribution, 89 in the Neotropics and 4 in Africa, but absent from Madagascar. All four African species are known from Cameroon, one endemic.

This genus of trees is characterized by stellate hairs on its leaves and pseudosyncarpous fruits. The only other tree genus with stellate hairs in Cameroon is ﻿*Annickia*, but the latter has a yellow slash and apocarpous fruits with clearly stipitate monocarps.

#### Taxonomy.

Maas et al. (2003); present work.

### ﻿Key to the species of ﻿*Duguetia* in Cameroon:

**Table:** 

1	Leaf blabes narrowly elliptic to narrowly oblong, 4 to 6 times longer than wide and leaves verruculose	﻿***D.confinis***
–	Lower side of the leaves sparsely to densely covered with appressed (flattened), stellate hairs; fruit globose or ovoid	**2**
2	Leaf base generally cordate, mid rid furrowed above, secondary veins weakly distinct	﻿***D.staudtii***
–	Leaf base acute, mid rid not furrowed above, secondary veins clearly distinct, forming loops	**3**
3	Inflorescences forming on a short peduncle in leafless parts of branches; fruiting carpels totally fused, areoles domed-shaped; seeds brown	﻿***D.barteri***
–	Inflorescences not forming a short peduncle in leafy part of branches; fruiting carpels basally fused, areoles obovoid to deltoid; seeds black	﻿***D.dilabens***

### ﻿﻿﻿﻿ Duguetia barteri

Taxon classificationPlantaeMagnolialesAnnonaceae

(Benth.) Chatrou, Changing Genera: 66, 1998

4F3DFEA5-7AD3-57A4-92A7-BA5769BF0C14

Fig. 25 ; Map 3H

≡ Annona barteri Benth., Trans. Linn. Soc. London 23(3): 477, 1862.
= Pachypodanthium staudtii (Engl. & Diels) Engl. & Diels var. letestui Pellegr., Bull. Soc. Bot. France 95: 137, 1948. *nom. illeg*.
= Pachypodanthium tessmannii R.E.Fr., *nom. nud.*

#### Type.

Nigeria. Anambra state; Onitsha, *Barter C. 445*, 1858: holotype: K[K000198875].

#### Description.

Tree, 8–40 m tall, d.b.h. 40–60 cm; stilt roots or buttresses absent. Indumentum of stellate or fasciculate hairs; old leafless branches sparsely pubescent to glabrous, young foliate branches sparsely pubescent to densely pubescent. Leaves: petiole 3–7 mm long, 2–3 mm in diameter, densely to sparsely pubescent, grooved, blade inserted on the side of the petiole; blade 10–23 cm long, 3–7 cm wide, ovate to elliptic, apex acuminate to acute, acumen ca. 1 cm long, base cordate to acute, subcoriaceous, below densely pubescent when young and old, above glabrous when young and old, concolorous; midrib **sunken or flat, not grooved**, above glabrous when young and old, below densely pubescent when young and old; secondary veins 9 to 18 pairs, **distinct**, glabrous below; tertiary venation reticulate. Individuals bisexual; **inflorescences ramiflorous on old leafless branches, appearing axillary, forming on a short peduncle 4–10 mm long.** Flowers with 9 perianth parts in 3 whorls, 2 to 5 per inflorescence; pedicel 11–22 mm long, 3–4 mm in diameter, sparsely to densely pubescent; in fruit 1–25 mm long, 1–4 mm in diameter, sparsely to densely pubescent; bracts 2, one basal and one upper in the lower half of pedicel, basal bracts 7–9 mm long; sepals 3, valvate, free, 10–15 mm long, 9–10 mm wide, ovate, apex acute, base truncate, yellowish green to greyish green, densely pubescent outside, glabrous inside, margins flat; petals free, outer petals longer than inner to sub equal; outer petals 3, 10–22 mm long, 5–8 mm wide, elliptic to ovate, apex acute, base truncate, cream to white, margins flat, sparsely pubescent outside, glabrous inside; inner petals 3, imbricate, 15–17 mm long, 4–5 mm wide, elliptic to ovate, apex acute, base truncate, cream, margins flat, pubescent outside, glabrous inside; stamens numerous, ca. 1 mm long, broad; connective discoid, glabrous, red; staminodes absent; carpels free, 50 to 75, ovary ca. 1.5 mm long, stigma globose, glabrous. Fruit pseudosyncarpous, 40–200 mm in diameter, globose to depressed ovoid; individual carpels sessile, **60 to 70 carpels, completely fused**, ca. 15 mm long, ca. 7 mm in diameter, globose to ovoid, apex domed-shaped, densely pubescent, longitudinally ribbed with 5 to 6 main ribs, pinkish red when ripe; seed 1 per monocarp, 7–15 mm long, 4–7 mm in diameter, ellipsoid; **aril present, pale yellow.**

**Figure 25.**
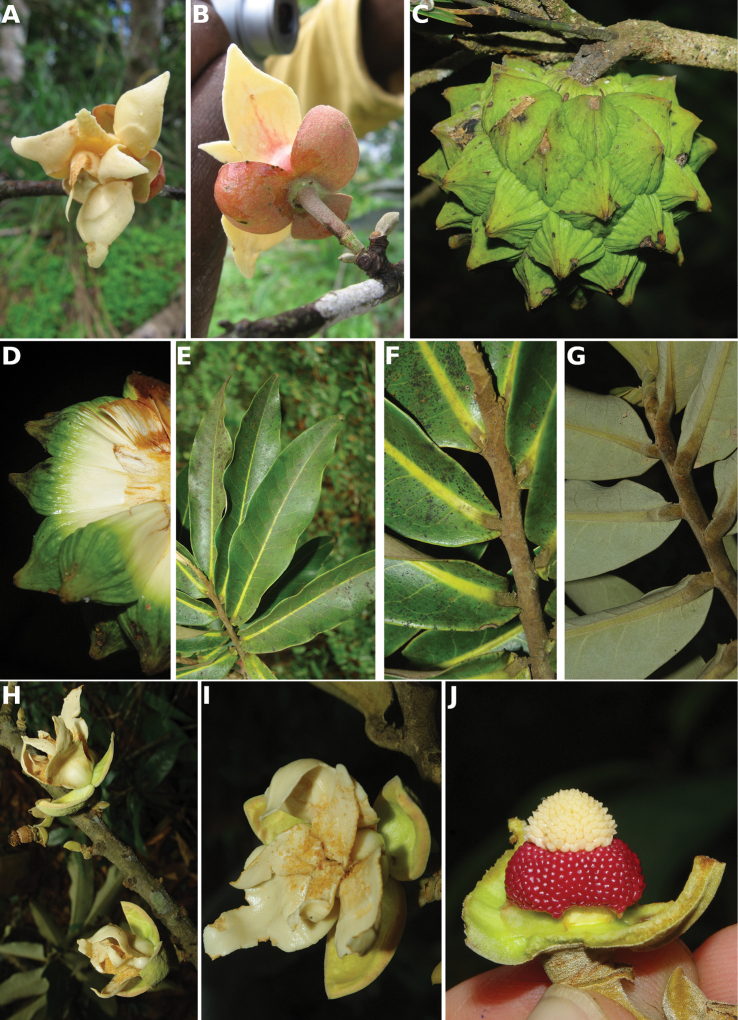
*Duguetiabarteri***A** flower, side view **B** flower, bottom view **C** fruit, note fused monocarps into a syncarpous fruit, referred to as a pseudosyncarp and with dome shaped apex **D** longitudinal section of fruit, note completely fused monocarps. *Duguetiaconfinis***E** leaves, upper side **F** base of leaf blade, upper side **G** base of leaf blade, lower side, note whitish pubescence representing minute stellate hairs completely covering whole lower side of leaf blade **H** flowering branch **I** flower, top view **J** detail of receptacle, all petals removed **A, B***Sosef 2138*, Gabon **C, D***Couvreur 393*, Ngovayang, Cameroon **E–J***Couvreur 527*, Gabon. Photos Thomas L.P. Couvreur.

#### Distribution.

A central African species, from Cameroon to Gabon; in Cameroon known from East, South, Central, Littoral and South-West regions.

#### Habitat.

In periodically or permanently inundated forests. Altitude 350–600 m a.s.l.

#### Local and common names known in Cameroon.

ntom (dial. Bagali) (Letouzey 1964).

#### IUCN conservation status.

Least Concern (LC) (Cosiaux et al. 2019i).

#### Uses in Cameroon.

None recorded.

#### Notes.

﻿﻿﻿*Duguetiabarteri* is distinguished by its elliptic to ovate leaves, the midrib not grooved above, inflorescences occurring on a short peduncle in leafless parts of the branches and its fruits with completely fused monocarps.

#### Specimens examined.

**Central Region**: Bank of Nyong river 40 km SE of Yaoundé, 3.65°N, 10.78°E, *09 November 1961*, *Breteler F.J.* 2013 (BR,K,P,WAG,YA); Abimoa, 3.57°N, 11.62°E, *04 April 1962*, *de Bruijn J.* s.n. (WAG[WAG0175175]); Bordure du Nyong près du lac de Nkolmaka (près route Mbalmayo-Akonolinga), 3.51°N, 11.82°E, *22 April 1954*, *Letouzey R.* 304 (P,YA). **Littoral Region**: Right bank Ouem river near mouth in Sanaga R 6 km SW of Massok, 4.13°N, 10.47°E, *04 April 1965*, *Leeuwenberg A.J.M.* 5377 (BR,K,MO,P,WAG,YA); Tissongo study area 3.57°N, 9.869°E, *01 June 1976*, *Waterman P.G.* 874 (K). **South Region**: mountain chain Ngovoyang 2 km in forest from Bikiliki village situated between Bipindi and Lolodorf, 3.18°N, 10.52°E, *18 February 2012*, *Couvreur T.L.P.* 393 (WAG,YA); Campo-Ma’an National Park, 2.38°N, 10.06°E, *01 July 2001*, *van Andel T.R.* 3810 (KRIBI,WAG,YA). **South-West Region**: Ekundu Kundu, 5.15°N, 8.883°E, *30 April 1996*, *Cheek M.* 8274 (K,WAG,YA).

### ﻿﻿﻿﻿ Duguetia confinis

Taxon classificationPlantaeMagnolialesAnnonaceae

(Engl. & Diels) Chatrou, Changing Genera: 67, 1998

24BF7458-79F0-58E3-A93D-E1214B63D680

**Figure 26.**
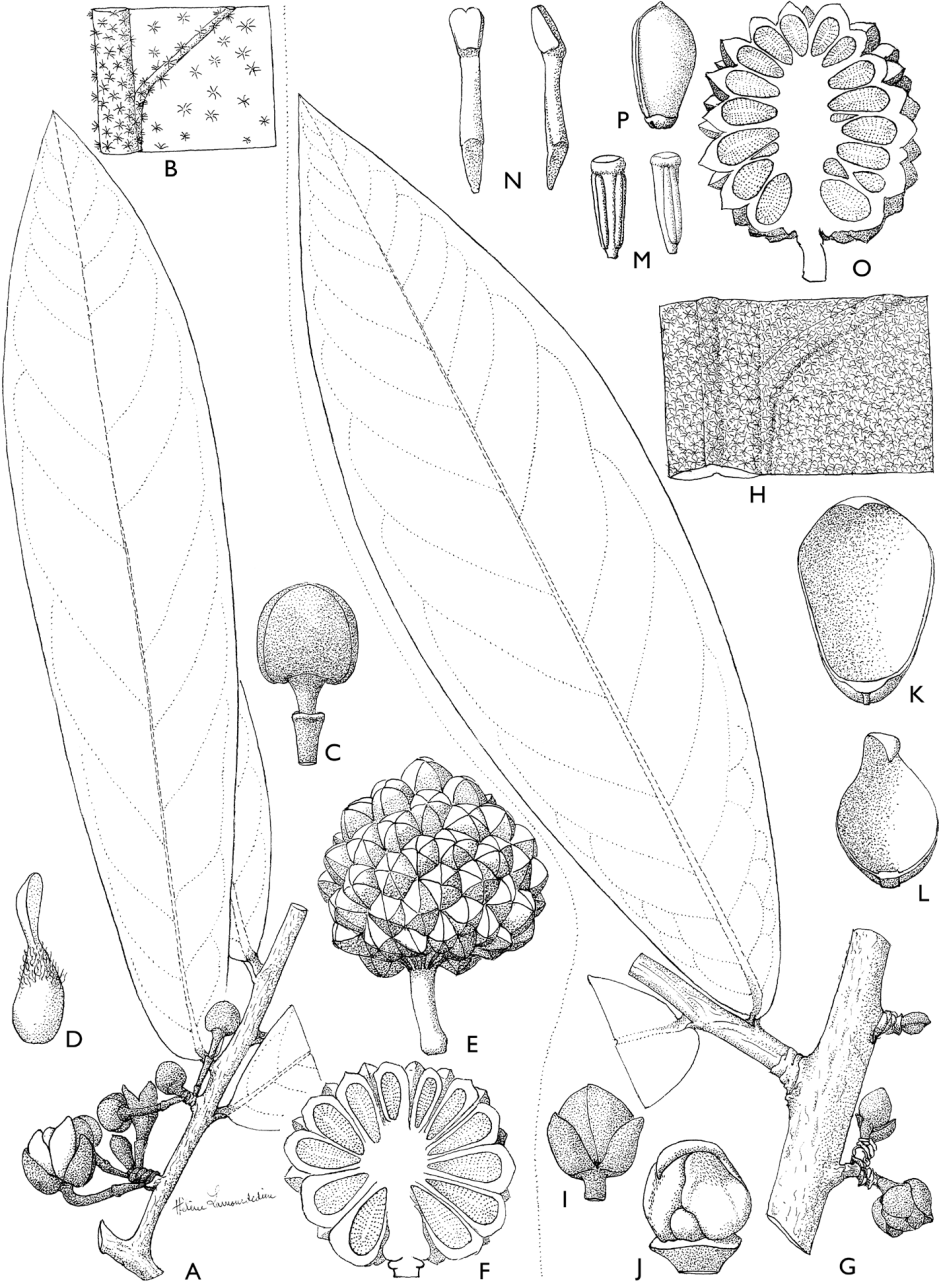
*Duguetiastaudtii***A** flowering branch **B** detail of stellate pubescence on lower side of leaf blade **C** flower bud, side view **D** carpel, side view **E** syncarpous fruit, referred to as a pseudosyncarpous fruit **F** longitudinal section of fruit. *Duguetiaconfinis***G** flowering branch **H** detail of pubescent on lower side of blade, note that it is completely covering the lower surface **I** flower bud, side view **J** detail of flower, sepals removed **K** outer petal, inner view **L** inner petals, inner view **M** stamen, side and front views **N** carpel, side and front views **O** longitudinal section of fruit, note completely fused monocarps **P** seed **A–D** from *Letouzey 4438***E, F** from *Chevalier 16224***G, H** from *Le Testu 1774***I–N, P** from *Klaine 217***O** from *Lecompte s.n.* Drawings by Hélène Lamourdedieu, Publications Scientifiques du Muséum national d’Histoire naturelle, Paris; modified from Le Thomas (1969b, pl. 18, p. 105).

Figs 25 , 26 ; Map 3I

≡ Pachypodanthium confine Engl. & Diels, Notizbl. Konigl. Bot. Gart. Berlin 3: 55, 1900.
= Pachypodanthium sargosii R.E.Fr., Ark. Bot. 3(2): 38, 1955; ﻿﻿﻿Pachypodanthiumconfinevar.sargosii Le Thomas, Fl. Gabon 16: 106, 1969. Type. Republic of Congo: Kouilou, *Sargos R. 29*, 4 Mar 1920: lectotype, sheet here designated: P[P00364784]; isotype: P[P00364785].

#### Type.

Gabon. Estuaire; Libreville, *Klaine T.-J. 217*, 10 Oct 1895: lectotype, sheet here designated: P[P00315819]; isotypes: P[P00315821, P00315815].

#### Description.

Tree, 15–40 m tall, d.b.h. 40–85 cm; stilt roots or buttresses absent. Indumentum of stellate hairs; old leafless branches sparsely pubescent to glabrous, young foliate branches densely pubescent. Leaves: petiole 1–8 mm long, 2–6 mm in diameter, densely pubescent, cylindrical, blade inserted on the side of the petiole; blade 9–31 cm long, 3–8 cm wide, elliptic, apex acute, acumen 0.5–1 cm long, base rounded to acute, coriaceous, **below densely pubescent with white erect stellate hairs covering the whole blade when young and old**, above glabrous when young and old, **discolorous, whitish below**; midrib sunken or flat, above glabrous when young and old, below densely pubescent when young and old; secondary veins 14 to 25 pairs, **indistinct**, glabrous below; tertiary venation reticulate. Individuals bisexual; **inflorescences ramiflorous on old leafless branches, appearing axillary, forming on a short peduncle 2–11 mm long.** Flowers with 9 perianth parts in 3 whorls, 2 to 5 per inflorescence; pedicel 3–20 mm long, 2–5 mm in diameter, densely pubescent; in fruit 3–20 mm long, 2–5 mm in diameter, glabrous to densely pubescent; bracts 2, one basal and one towards the upper half of pedicel, basal bracts 5–9 mm long, 5–10 mm wide; sepals 3, valvate, free, 17–22 mm long, 13–15 mm wide, ovate, apex acute, base truncate, green, densely pubescent outside, glabrous inside, margins flat; petals free, outer petals longer than inner to sub equal; outer petals 3, 11–18 mm long, 5–9 mm wide, elliptic, apex acuminate, base truncate, cream to white, margins wavy, densely pubescent outside, glabrous inside; inner petals 3, imbricate, 11–18 mm long, 5–9 mm wide, elliptic, apex acuminate, base truncate, cream, margins flat, pubescent outside, glabrous inside; stamens 290 to 310, in 7 to 8 rows, 1–2 mm long, broad; connective discoid, glabrous, red; staminodes absent; carpels free, 100 to 125, ovary 1.4–2 mm long, stigma globose, glabrous. Fruit pseudosyncarpous, 25–50 mm in diameter, ellipsoid; individual carpels sessile, 100 to 125 carpels, **completely fused**, 15–12 mm long, 2–5 mm in diameter, obovoid, apex apiculate to acute, densely pubescent, longitudinally ribbed with 5 to 6 main ribs, greyish brown with purplish red or pale brown pulp when ripe; seed 1 per monocarp, 10–14 mm long, 4–8 mm in diameter, ellipsoid; **aril present, red.**

#### Distribution.

From Cameroon to Gabon and Republic of Congo; in Cameroon known from East, South and Littoral regions.

#### Habitat.

In lowland periodically inundated or non-inundated rain forests. Altitude 0–50 m a.s.l.

#### Local and common names known in Cameroon.

None recorded.

#### IUCN conservation status.

Least Concern (LC) (Cosiaux et al. 2019j).

#### Uses in Cameroon.

None recorded.

#### Notes.

﻿﻿﻿*Duguetiaconfinis* is distinguished by its lower leaf surface, which is densely pubescent with erect stellate hairs completely covering the blade, and its carpels completely fused in fruit.

#### Specimens examined.

**Central Region**: Est N of Lom near Sanaga river (Yaundé) 250 km from Deng Deng, 4.84°N, 13.19°E, *01 March 1914*, *Mildbraed G.W.J.* 8558 (K). **Littoral Region**: Au sud de Ngola (8 km E de l’embouchure de la Sanaga), 3.55°N, 9.698°E, *05 January 1974*, *Letouzey R.* 12585 (P); Douala-Edea Reserve 10B, 5.01°N, 13.33°E, *01 April 1978*, *Thomas D.W.* 1237 (K). **South Region**: ca 16 km from Kribi Ebolowa road Bidou plantation Kienke forReserve, 2.85°N, 10.01°E, *03 February 1969*, *Bos J.J.* 3844 (WAG,YA); 20 km From Kribi N of Lolodorf road (SFIA logging road), 3.01°N, 10.05°E, *15 July 1969*, *Bos J.J.* 5048 (BR,BR,K,WAG,YA); Mt Elephant ca 18 km SE of Kribi, 2.78°N, 10.5°E, *14 January 1970*, *Bos J.J.* 6128 (BR,P,WAG); Near mouth of, 3.17°N, 9.961°E, *28 March 1928*, *Hédin L.* 1690 (P,WAG); Campo-Ma’an area 2.71°N, 9.866°E, *26 October 2001*, *van Andel T.R.* 4205 (KRIBI,WAG); Bipindi, 3.08°N, 10.42°E, *01 January 1904*, *Zenker G.A.* 3195 (B,BR,E,G,L,M,MO,P,S).

### ﻿﻿﻿﻿ Duguetia dilabens

Taxon classificationPlantaeMagnolialesAnnonaceae

Chatrou & Repetur, Changing Genera: 69, 1998

9F796C4B-D366-5F29-9496-7CB1AC880BCE

Map 4A

#### Type.

Gabon. Ngounié; new road from Mouila to Yeno, 5 km on either side of Kembele village, *Thomas D.W. & Wilks C.M. 6510*, 20 Jul 1986: lectotype, sheet here designated: WAG[WAG0143388]; isotypes: MO[MO-357359]; P[P00389133]; WAG[WAG0027128].

#### Description.

Tree, up to 30 m tall, d.b.h. unknown; stilt roots or buttresses absent. Indumentum of stellate or fasciculate hairs; old leafless branches sparsely pubescent to glabrous, young foliate branches sparsely pubescent. Leaves: petiole 4–5 mm long, 2–3 mm in diameter, sparsely pubescent, grooved, blade inserted on the side of the petiole; blade 7–16 cm long, 2.5–6 cm wide, **narrowly elliptic to narrowly obovate**, apex acuminate to acute, acumen ca. 1 cm long, base acute, subcoriaceous, below sparsely pubescent when young and old, above glabrous when young and old, concolorous; midrib sunken or flat, above glabrous when young and old, below sparsely pubescent when young and old; secondary veins 8 to 15 pairs, **distinct**, glabrous below; tertiary venation reticulate. Individuals bisexual; **inflorescences ramiflorous on young foliate branches, leaf opposed, not forming a peduncle.** Flowers with 9 perianth parts in 3 whorls, 2 to 4 per inflorescence; pedicel 10–12 mm long, 1–2 mm in diameter, sparsely pubescent to densely pubescent; in fruit 10–50 mm long, 1–15 mm in diameter, sparsely pubescent to densely pubescent; bracts 2, one basal and one towards the lower half of pedicel, basal bracts 5–9 mm long, 7–9 mm wide; sepals 3, valvate, free, 12–15 mm long, 6–9 mm wide, elliptic to ovate, apex acute, base truncate, greyish green, pubescent outside, pubescent inside, margins flat; petals free, outer petals longer than inner to sub equal; outer petals 3, 12–15 mm long, 4–6 mm wide, ovate, apex acute, base truncate, white, margins flat, sparsely pubescent outside, glabrous inside; inner petals 3, imbricate, 13–15 mm long, 4–5 mm wide, elliptic, apex acute, base truncate, margins flat, pubescent outside, glabrous inside; stamens numerous, 1 mm long, broad; connective discoid, glabrous, red; staminodes absent; carpels free, ca. 75, 2–3.5 mm long, stigma globose, glabrous. Fruit pseudosyncarpous, size and shape unknown; carpels sessile, **free to basally fused**, unknown number of carpels, 20–35 mm long, ovary 10–30 mm in diameter, obovoid to deltoid, apex apiculate, pubescent, densely pubescent, longitudinally ribbed with 6 to 7 main ribs, color unknown; seed 1 per monocarp, 12–20 mm long, 10–13 mm in diameter, ellipsoid; aril present, color unknown.

**Map 4.**
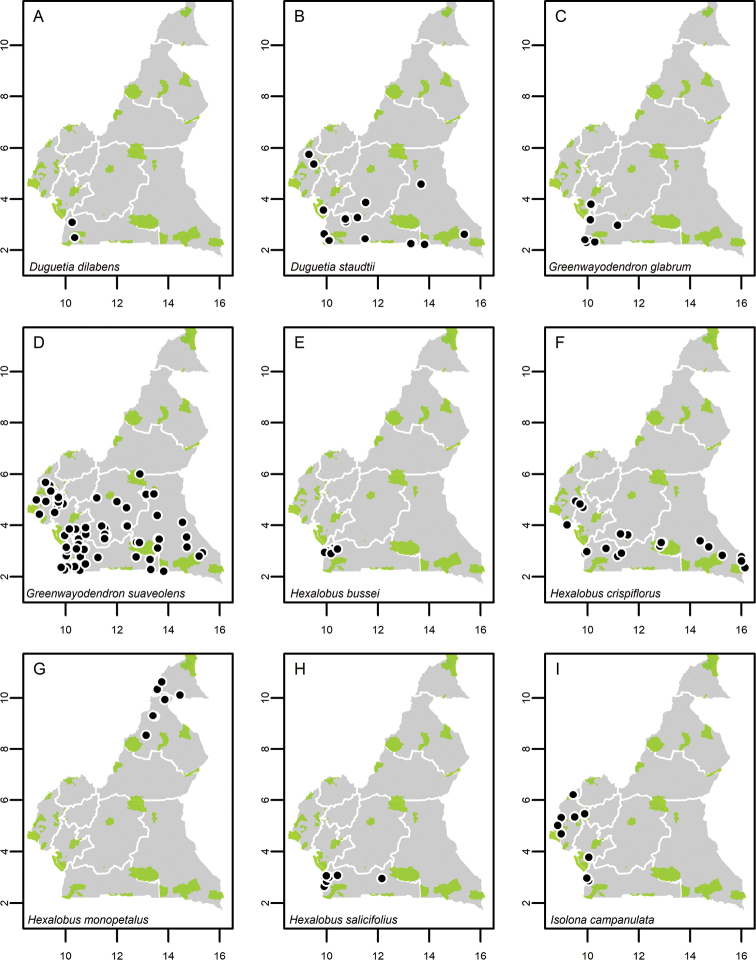
**A***Duguetiadilabens***B***Duguetiastaudtii***C***Greenwayodendronglabrum***D***Greenwayodendronsuaveolens***E***Hexalobusbussei***F***Hexalobuscrispiflorus***G***Hexalobusmonopetalus***H***Hexalobussalicifolius***I***Isolonacampanulata*. White borders represent region limits in Cameroon; green patches represent protected areas (see methods and Suppl. material 1: Fig. S1).

#### Distribution.

Known from Gabon and Cameroon; in Cameroon known from South and Littoral regions.

#### Habitat.

A rare species known from four specimens; in lowland periodically inundated or non-inundated rain forests. Altitude 0–500 m a.s.l.

#### Local and common names known in Cameroon.

None recorded.

#### IUCN conservation status.

Endangered (EN) (Texier and Stévart 2020)

#### Uses in Cameroon.

None recorded.

#### Notes.

﻿﻿﻿*Duguetiadilabens* is distinguished by its leaves that are narrowly elliptic to narrowly obovate, the midrib not grooved above and the carpels only basally fused in fruit. It was recently collected in Campo Ma’an National Park (*Couvreur 692*), but the sample is sterile and the identification remains doubtful, although the leaves do match the type specimen. *Couvreur 692* also notes that the trunk had a bark peeling in smallish flakes.

#### Specimens examined.

**South Region**: Reserve forestière de la Kienké (Kribi-Ebolowa km 16), 3.1°N, 10.25°E, *05 January 1968*, *Bamps P.R.J.* 1679 (BR, YA); Campo Ma’an National Park 11 km on trail from Ebinanemeyong village on road 7 km from Nyabessan to Campo town, 2.49°N, 10.34°E, *12 February 2015*, *Couvreur T.L.P.* 692 (WAG,YA).

### ﻿﻿﻿﻿ Duguetia staudtii

Taxon classificationPlantaeMagnolialesAnnonaceae

(Engl. & Diels) Chatrou, Changing Genera: 70, 1998

23B734AB-F144-5662-BF91-242E3854F2C2

Fig. 26 ; Map 4B

≡ Uvaria staudtii Engl. & Diels, Notizbl. Königl. Bot. Gart. Berlin 2: 292, 1899; ﻿﻿Pachypodanthiumstaudtii Engl. & Diels, Notizbl. Königl. Bot. Gart. Berlin 3: 55, 1900.

#### Type.

Cameroon. South Region; near Lolodorf, *Staudt A. 133*, 1896: holotype: B[B 10 0154084]; isotypes: BM[BM000843984]; K[K000198873, K000198874]; P[P00315814, P00315816]; S[S02-94].

#### Description.

Tree, 15–50 m tall, d.b.h. 20–70 cm; stilt roots or buttresses absent. Indumentum of stellate or fasciculate hairs; old leafless branches sparsely pubescent to glabrous, young foliate branches sparsely pubescent to densely pubescent. Leaves: petiole 2–10 mm long, 2–4 mm in diameter, densely pubescent to sparsely pubescent, grooved, blade inserted on the side of the petiole; blade 13–34 cm long, 3–8 cm wide, **narrowly obovate, narrowly oblong to narrowly elliptic, apex acuminate to acute**, acumen 0.5–1 cm long, **base cordate (more rarely acute)**, coriaceous, below sparsely pubescent when young, pubescent when old, above glabrous when young and old, concolorous; midrib sunken or flat, above glabrous when young and old, below sparsely pubescent when young and old; secondary veins 10 to 22 pairs, **weakly distinct**, glabrous below; tertiary venation reticulate. Individuals bisexual; inflorescences ramiflorous on old leafless or young foliate branches, leaf opposed or extra axillary, **not forming a peduncle.** Flowers with 9 perianth parts in 3 whorls, 2 to 4 per inflorescence; pedicel 6–12 mm long, 2–3 mm in diameter, sparsely pubescent to densely pubescent; in fruit 2–30 mm long, 2–6 mm in diameter, sparsely pubescent to densely pubescent; bracts 2, one basal and one towards the upper half of pedicel, basal bracts 9–12 mm long, 5–7 mm wide; sepals 3, valvate, free, 9–15 mm long, 7–10 mm wide, ovate, apex acute, base truncate, green, densely pubescent outside, glabrescent inside, margins flat; petals free, outer petals longer than inner to sub equal; outer petals 3, 7–30 mm long, 4–10 mm wide, oblong-elliptic to oblong-obovate, apex acute, base truncate, cream to white, margins flat, sparsely pubescent outside, glabrous inside; inner petals 3, imbricate, 4–20 mm long, 4–6 mm wide, elliptic to obovate, apex acute to obtuse, base truncate, cream, margins flat, pubescent outside, glabrous inside; stamens 120 to 150, in 5 to 6 rows, 1–2 mm long, broad; connective discoid, glabrous, red; staminodes absent; carpels free, 50 to 100, ovary 1–1.5 mm long, stigma globose, glabrous. Fruit pseudosyncarpous, 20–55 mm in diameter, globose to depressed ovoid; carpels sessile, **basally fused**, 50 to 100 monocarps, 20–55 mm long, 2–10 mm in diameter, globose to ovoid, apex acute, densely pubescent, longitudinally ribbed with 5 to 6 main ribs, red when ripe; seed 1 per monocarp, 7–13 mm long, 6–8 mm in diameter, ellipsoid; **aril present, pale yellow.**

#### Distribution.

A widespread species with a disjunct distribution in West (Sierra Leone, Liberia, Ivory Coast and Nigeria) and in Central Africa from Cameroon to Democratic Republic of the Congo; in Cameroon known from East, South, Central, Littoral and South-West regions.

#### Habitat.

A common species; in lowland or premontane primary and secondary non-inundated rain forests. Altitude 100–900 m a.s.l.

#### Local and common names known in Cameroon.

ntom (dial. Bagali); nto ntomba (dial. Bagielli) (Letouzey 1964).

#### IUCN conservation status.

Least Concern (LC) (Cosiaux et al. 2019k).

#### Uses in Cameroon.

***medicine***: bark used for pain-killers, pulmonary troubles, vermifuges, dropsy, swellings, oede gout, tumours, cancers; ***constructions***: building materials; ***dyes and tannins***: tannins, astringents, insecticides, arachnicides, arrow-poisons, aromatic substances, alkaloids.

#### Notes.

﻿﻿﻿*Duguetiastaudtii* is distinguished by its narrowly obovate, narrowly oblong to narrowly elliptic leaves, the midrib that is grooved above and monocarps that are only basally fused.

#### Specimens examined.

**Central Region**: Yaoundé, 3.86°N, 11.51°E, *01 January 1935*, *Foury P.* 69 (P,WAG). **East Region**: 17 km along road to Deng Deng, 4.58°N, 13.68°E, *01 September 1961*, *Breteler F.J.* 1841 (WAG); Près Kinsassa 65 km au NNE de Moloundou sur route Yokadouma 2.63°N, 15.37°E, *04 March 1971*, *Letouzey R.* 10509 (P,YA); Colline à l’ENE de Mbalam (140 km ESE de Djoum près Souanké-Congo), 2.22°N, 13.82°E, *20 January 1973*, *Letouzey R.* 11867 (P,YA). **Littoral Region**: Douala-Edéa Reserve Tissongo study area Transect B, 3.57°N, 9.869°E, *01 June 1976*, *Waterman P.G.* 879 (U). **South Region**: Bitye, 3.87°N, 11.52°E, *01 January 1919*, *Bates G.L.* 1199 (BM,MO); 17 km east from Lélé village, 2.28°N, 13.32°E, *07 September 2013*, *Couvreur T.L.P.* 460 (WAG,YA); 25 km east from Lélé village at end of path on Ivindo river, 2.25°N, 13.28°E, *09 September 2013*, *Couvreur T.L.P.* 489 (WAG,YA); Sud TDC, 2.65°N, 9.9°E, *06 November 1991*, *Hallé F.* 4220 (WAG); Ncolbew 3.28°N, 11.2°E, *26 April 1928*, *Hédin L.* 1646 (P); Colline Ebon près Nkobiyo 25 km ENE d’Ambam, 2.45°N, 11.5°E, *21 March 1970*, *Letouzey R.* 10181 (P,YA); Mvini 35 km east of Campo, 2.37°N, 10.09°E, *20 December 1983*, *Mikio K.* 5 (P,YA); ca 7 km NE of Ebom, 3.11°N, 10.75°E, *01 August 1996*, *Parren M.P.E.* 157 (KRIBI,WAG); ca 7 km NE of Ebom, 3.11°N, 10.75°E, *01 August 1996*, *Parren M.P.E.* 212 (KRIBI,WAG); Lolodorf, 3.23°N, 10.73°E, *1896*, *Staudt A.* 133 (P); Lolodorf, 3.23°N, 10.73°E, *March 1895*, *Staudt A.* 138 (B); Campo-Ma’an area 2.4°N, 10.1°E, *02 April 2001*, *van Andel T.R.* 3290 (KRIBI,U,WAG,YA). **South-West Region**: Bayang Mbo Wildlife Sanctuary after Mbu river, 5.35°N, 9.501°E, *26 March 2016*, *Couvreur T.L.P.* 1014 (WAG,YA); Near Mamfe, 5.75°N, 9.31°E, *19 April 1978*, *Thomas D.W.* 384 (K).

### ﻿﻿ Greenwayodendron

Taxon classificationPlantaeMagnolialesAnnonaceae

Verdc., Adansonia sér. 2, 9: 89, 1969

5356C920-BD7C-52A7-9496-D331DAA334F4

≡ Polyalthia sect. Afropolyalthia Engler & Prantl., Leipzig, W. Engelmann.160, 1897.

#### Type species.

﻿﻿﻿*Greenwayodendronsuaveolens* (Engl. & Diels) Verdc.

#### Description.

Trees, 7–45 m tall, d.b.h. 3–125 cm; stilt roots or buttresses absent. Indumentum of simple hairs. Leaves: petiole 2–8 mm long, 1–3 mm in diameter, blade 6.5–16.2 cm long, 2–6.7 cm wide, elliptic to oblong, apex acuminate to caudate, base cuneate to rounded, concolorous; midrib sunken or flat; secondary veins 5 to 18 pairs; tertiary venation reticulate. Individuals androdioecious; male and bisexual inflorescences similar in appearance, ramiflorous on young foliate branches, leaf opposed or extra axillary. Flowers with 9 perianth parts in 3 whorls, 1 to 4 per inflorescence; pedicel 4–6 mm long; in fruit 6–13 mm long; bracts 2, one basal and one upper, 1–2 mm long; sepals 3, valvate, free, 2–4 mm long, ovate, apex acuminate, base truncate; petals free; outer petals longer than inner; outer petals 3, valvate, 8–18 mm long, 2.3–2.6 mm wide, oblong to elliptic, apex acuminate, base rounded; inner petals 3, valvate, 8–18 mm long, 1.3–2.6 mm wide, ovate to elliptic, apex acuminate, base rounded; stamens 15 to 25, in 4 to 5 rows, ovary 1–2 mm long, elongated; connective tongue-shaped, glabrous; staminodes absent; carpels free, 10 to 20, 1–2 mm long, stigma ovoid, pubescent. Fruits apocarpous, monocarps stipitate, stipes 5–10 mm long, monocarps 2 to 8, 8–21 mm long, 7–21 mm in diameter, ellipsoid to globose, apex rounded, smooth, green turning wine red when ripe; seed 1 to 4, 3–13 mm long, 3–13 mm in diameter, ellipsoid to flattened ellipsoid; aril absent.

A genus of six currently described species distributed across Africa. Two species are known from Cameroon, none endemic. Onana (2011) mentions ﻿*Greenwayodendronoliveri* (Engl.) Verdc. from Cameroon, but this is not confirmed here. The latter species is a West African endemic (Lissambou et al. 2018); several specimens from coastal Gabon previously identified as *G.oliveri* are now separated as a different species: ﻿*G.littorale* Lissambou, Dauby & Couvreur (Lissambou et al. 2018, 2019). Neither species is known from Cameroon to date.

#### Taxonomy.

Lissambou et al. (2018).

### ﻿Key to the species of ﻿*Greenwayodendron* in Cameroon

**Table:** 

1	Petiole and midrib glabrous above	﻿***G.glabrum***
–	Petiole and midrib pubescent or sparsely pubescent above	﻿***G.suaveolens***

### ﻿﻿﻿﻿ Greenwayodendron glabrum

Taxon classificationPlantaeMagnolialesAnnonaceae

Lissambou, Hardy & Couvreur, PhytoKeys 114: 66, 2018

3DC2CD7C-0F06-5465-B8F3-7AB4C7571A10

**Figure 27.**
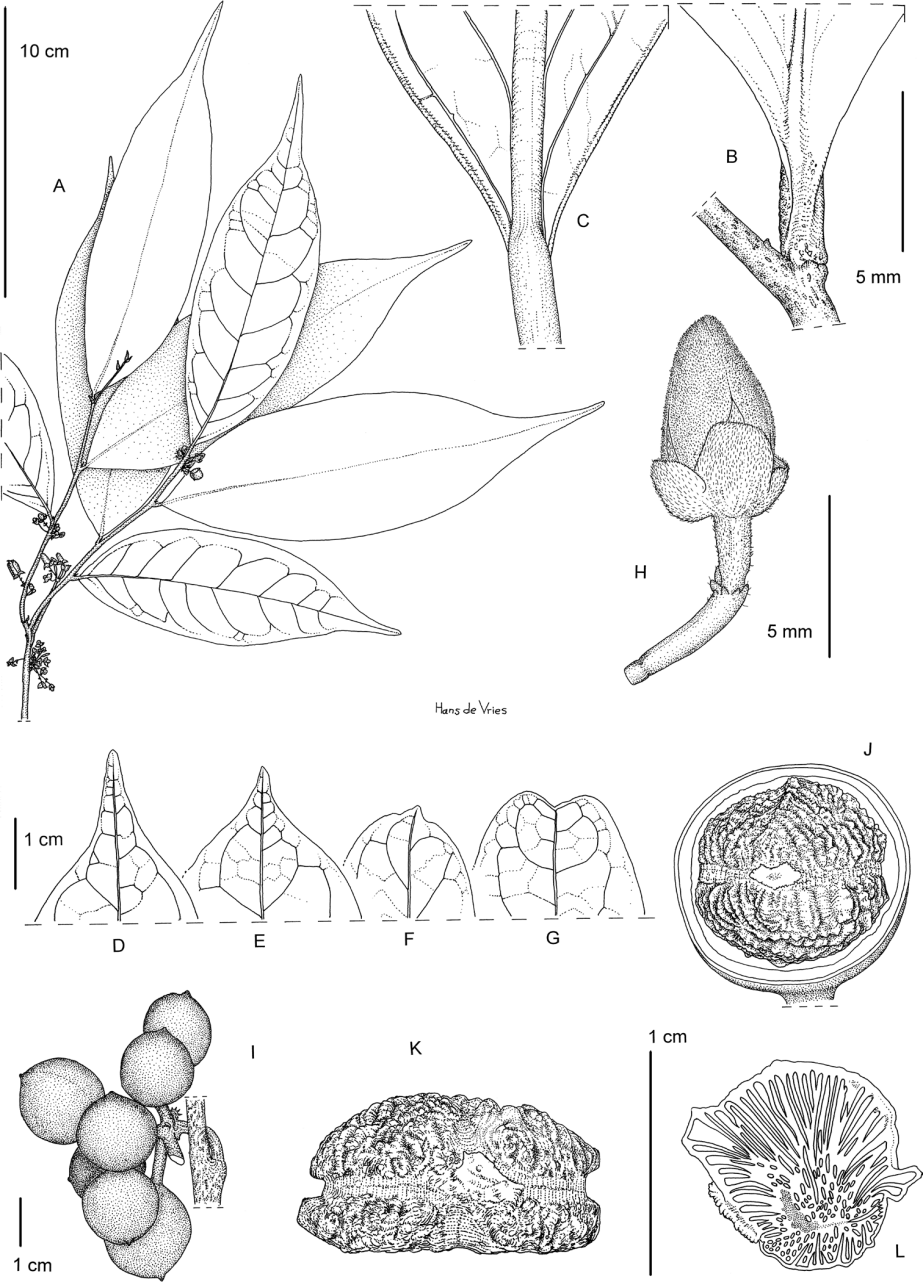
*Greenwayodendronglabrum***A** flowering branch **B** detail of lower leaf surface **C** detail of upper leaf surface **D–G** different types leaf apex **H** flower bud **I** infructescence **J** longitudinal section of fruit revealing seed **K** seed, latitudinal view **L** longitudinal section of seed showing ruminations **A–C, G, H***Letouzey 12869***D–F, I–L***Bos 6267*. Drawings by Hans de Vries (Lissambou et al. 2018, fig. 1, p. 63).

Figs 27 , 29 ; Map 4C

#### Type.

Cameroon. South Region; 40 km from Kribi, 5 km. E. of Edea road, tract of Fifinda-Bella road (SFIA), *Bos J.J. 6267*, 6 Feb 1970: holotype WAG[WAG.1433854]; isotypes BR[BR0000014826399]; YA *n.v.*; WAG[WAG1433855].

#### Description.

Tree, 7–30 m tall, d.b.h. 3–20 cm; stilt roots or buttresses absent. Indumentum of simple hairs; old leafless branches glabrous, young foliate branches pubescent. Leaves: petiole 3–6 mm long, 1–2 mm in diameter, **glabrous**, grooved, blade inserted on the side of the petiole; blade 6.5–16.2 cm long, 2.1–5.8 cm wide, elliptic to oblong, apex acuminate to caudate, acumen 0.4–2 cm long, base cuneate to rounded, papyraceous, below sparsely pubescent to glabrous when young, glabrous when old, **above glabrous when young and old, concolorous; midrib impressed, above completely glabrous when young and old**, below glabrous when young and old; secondary veins 5 to 7 pairs, glabrous below; tertiary venation indistinct. Individuals androdioecious; male and bisexual inflorescences similar, ramiflorous on old leafless and young foliate branches, leaf opposed or extra axillary. Flowers with 9 perianth parts in 3 whorls, 1 to 4 per inflorescence; pedicel ca. 4 mm long, ca. 1 mm in diameter, pubescent to glabrous; in fruit 6–13 mm long, 1–2 mm in diameter, pubescent to glabrous; bracts 2, one basal and one upper towards the upper half of pedicel, basal bracts 1–2 mm long, 2 mm wide; upper bracts 1–3 mm long, 1–3 mm wide; sepals 3, valvate, basally fused to free, 3 mm long, 3–4 mm wide, ovate, apex acuminate, base truncate, green, pubescent outside, glabrous inside, margins flat; petals free, sub equal; outer petals 3, 12–13 mm long, 2–2.5 mm wide, elliptic to ovate, apex acuminate, base rounded, green to light yellow, margins flat, pubescent outside, glabrous inside; inner petals 3, valvate, 12–13 mm long, 2–2.5 mm wide, ovate, apex acuminate, base rounded, green to light yellow, margins flat, pubescent outside, glabrous inside; stamens 10 to 15, in 4 to 5 rows, 1–2 mm long, elongated; connective tongue shaped, glabrous, green; staminodes absent; carpels free, 10 to 15, ovary ca. 1 mm long, stigma ovoid, pubescent. Monocarps stipitate, stipes 5–10 mm long, 1–3 mm in diameter; monocarps 2 to 8, 11–21 mm long, 11–21 mm in diameter, ellipsoid to globose, apex rounded, glabrous, smooth, smooth; seeds 1 to 4 per monocarp, 7–13 mm long, 7–13 mm in diameter, ellipsoid to flattened ellipsoid; aril absent.

#### Distribution.

Known from Cameroon and Gabon; in Cameroon known from the Littoral and South regions.

#### Habitat.

A common species when present and growing in sympatry with ﻿*G.suaveolens* in southern Cameroon; in lowland non-inundated primary or secondary forests. Altitude 20–750 m a.s.l.

#### Local and common names known in Cameroon.

None recorded, but possibly same as ﻿*G.suaveolens* (see below).

#### IUCN conservation status.

Least Concern (LC) (Harvey-Brown 2019b).

#### Uses in Cameroon.

None recorded.

#### Notes.

This species is very close morphologically to ﻿*G.suaveolens.* Both species grow in sympatry in southern Cameroon. However, ﻿*G.glabrum* is distinguished by its glabrous petiole and upper midrib and leaf blades (versus pubescent in ﻿*G.suaveolens*). Studies have shown that these two species are genetically distinct at both the phylogenetic (Couvreur et al. 2019) and population genetic (Lissambou et al. 2019) levels.

#### Specimens examined.

**Central Region**: Left bank Nyong R 30 km S of Edéa near bridge in road to Kribi, 3.8°N, 10.13°E, *26 April 1965*, *Leeuwenberg A.J.M.* 5582 (B,BR,C,GC,K,LUAI,MO,P,UC,WAG,YA). **Littoral Region**: Ndogtima Nyong (Edéa), 3.8°N, 10.13°E, *03 February 1974*, *Letouzey R.* 12869 (BR,P,WAG,YA). **South Region**: 43 kmN of Kribi 5 km E of Edea road forest track Fifinda-Bella old secondary forest, 3.21°N, 10.06°E, *06 February 1970*, *Bos J.J.* 6267 (BR,P,WAG,YA); ca 16 km On the road from Ebolowa to Minkok, 2.98°N, 11.17°E, *12 September 1975*, *de Wilde J.J.F.E* 8465 (B,BR,K,MO,P,WAG,YA); Mvini 35 km East of Campo, 2.39°N, 10.04°E, *19 December 1983*, *Kaji M.* 4 (YA); Campo-Ma’an region, 2.28°N, 9.950°E, *17 January 2016*, *Lissambou B.J.* 1745 (BRLU); Campo-Ma’an region, 2.28°N, 9.949°E, *17 January 2016*, *Lissambou B.J.* 1748 (BRLU); Campo-Ma’an region, 2.28°N, 9.948°E, *17 January 2016*, *Lissambou B.J.* 1775 (BRLU); Campo-Ma’an region, 2.28°N, 9.949°E, *17 January 2016*, *Lissambou B.J.* 1788 (BRLU); Campo-Ma’an region, 2.29°N, 9.945°E, *18 January 2016*, *Lissambou B.J.* 1807 (BRLU); Campo-Ma’an region, 2.40°N, 9.895°E, *18 January 2016*, *Lissambou B.J.* 1828 (BRLU); Campo-Ma’an region, 2.40°N, 9.894°E, *18 January 2016*, *Lissambou B.J.* 1830 (BRLU); Campo-Ma’an region, 3.19°N, 10.10°E, *19 January 2016*, *Lissambou B.J.* 1855 (BRLU); Campo-Ma’an region, 3.19°N, 10.10°E, *19 January 2016*, *Lissambou B.J.* 1856 (BRLU); Cagnon du Ntem 16 km SW de Nyabessan, 2.32°N, 10.28°E, *30 November 1982*, *Nkongmeneck B.A.* 400 (YA).

### ﻿﻿﻿﻿ Greenwayodendron suaveolens

Taxon classificationPlantaeMagnolialesAnnonaceae

(Engl. & Diels) Verdc., Adansonia, n.s. 9: 90, 1969

55A70EAA-835F-5DD8-B69B-B26868F8D3DC

**Figure 28.**
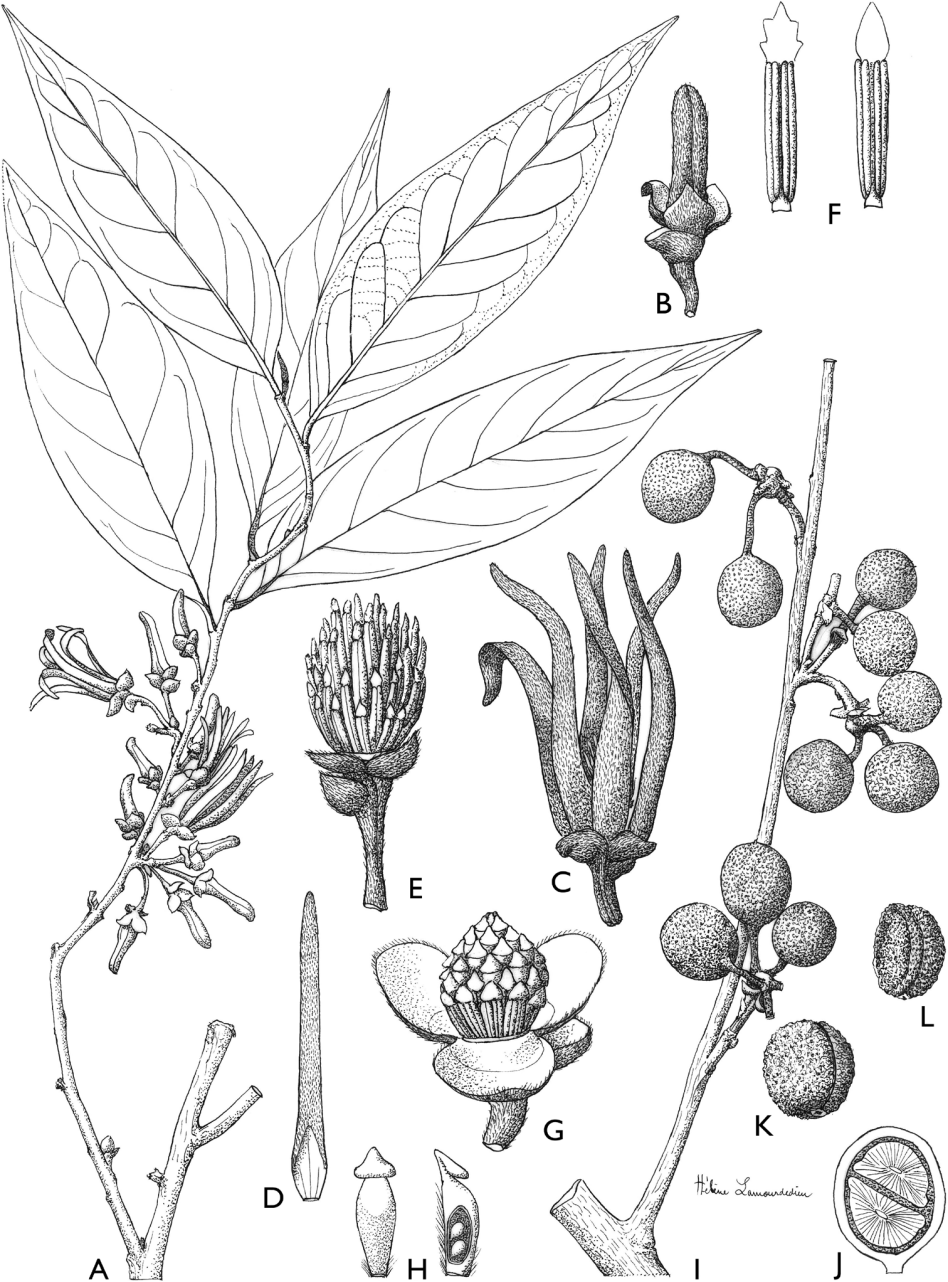
*Greenwayodendronsuaveolens***A** flowering branch **B** flower bud **C** flower at anthesis **D** detail of male receptacle, petals removed **E** detail of hermaphrodite receptacle, petals removed **F** inside view of outer petal **G** stamen **H** stamen **I** carpel **J** longitudinal section of carpel **K** fruiting branch **L** lateral view of seed **M** seed **N** longitudinal section of a single monocarp showing two seeds and their ruminations **A–D, F–H** from *Le Testu 9408*; E, I, J. *Gilbert 936***K–N***Letouzey 5322*. Drawings by Hélène Lamourdedieu, Publications Scientifiques du Muséum national d’Histoire naturelle, Paris; modified from Le Thomas (1969b, pl. 37, p. 205).

Fig. 28 , 29 ; Map 4D

≡ Polyalthia suaveolens Engl. & Diels, Monogr. Afr. Pfl. 6: 42., 1901.
= Polyalthia mortehanii De Wild., Bull. Jard. Bot. État Bruxelles, 4: 384., 1914. Type. Democratic Republic of the Congo. Kasaï-Oriental: Lekimi, *De Giorgi S. 1576*, Dec 1913: lectotype designated by Lissambou et al. (2018), p. 77: BR[BR8804408].
= Polyalthia aubrevillei Ghesquière ex Aubréville, Fl. For. Côte d’Ivoire, i. 114, 1936. Type. Cameroon. South Region: Bipindé, Urwaldgebiet, *Zenker G. 1306*, 1913: lectotype designated by Lissambou et al. (2018), p. 77: P[P01985238]; isolectotypes: L[L.1761577]; MO; P[P01985239]; WAG[WAG.1379971].
= Maba gossweileri Greves., J. Bot. 67 (Suppl. 2): 76., 1929. Type. Angola. Cabinda: Buco Zau - Maiombe, *Gossweiler J. 6923*, 8 Jan 1917: holotype BM[BM000547162]; COI[COI00004858].
= Xylopia otunga Exell., J. Bot. 69: 99, 1931. Type. Cameroon. Central: Bitye Yaoundé, Bates G.L. 1226, 1919: holotype: BM[BM000513697]; isotype LISC[LISC000385].

#### Type.

Gabon. Estuaire; Munda, Sibange Farm, *Soyaux H. 218*, 20 Feb 1881: holotype material presumably destroyed at B; lectotype, designated by Lissambou et al. 2018 (2018), p. 77: P[P00363356]; isolectotype K[K000580898].

#### Description.

Tree, 8–45 m tall, d.b.h. 10–125 cm; stilt roots or buttresses absent. Indumentum of simple hairs; old leafless branches glabrous, young foliate branches pubescent to sparsely pubescent. Leaves: petiole 2–8 mm long, 1–3 mm in diameter, **pubescent to sparsely pubescent**, grooved, blade inserted on the side of the petiole; blade 5.1–15.6 cm long, 2–6.7 cm wide, elliptic to oblong, apex acuminate to caudate, acumen 0.6–1.4 cm long, base cuneate to rounded, papyraceous, below pubescent when young, glabrous to pubescent when old, above densely to sparsely pubescent when young, sparsely pubescent when old, concolorous; midrib impressed, **above pubescent at least basely when young and old**, below densely pubescent when young, sparsely pubescent to densely pubescent when old; secondary veins 5 to 18 pairs, glabrous below; tertiary venation indistinct. Individuals androdioecious; male and bisexual inflorescences similar, ramiflorous on old leafless and young foliate branches, leaf opposed or extra axillary. Flowers with 9 perianth parts in 3 whorls, 1 to 4 per inflorescence, pedicel 3–6 mm long, 1–2 mm in diameter, pubescent; in fruit 6–12 mm long, 2–3 mm in diameter, glabrous; bracts 2, one basal and one upper towards the upper half of pedicel, basal bracts 1–2 mm long, 2 mm wide; upper bracts 1–3 mm long, 1–3 mm wide; sepals 3, valvate, basally fused to free, 2–4 mm long, 2–4 mm wide, ovate, apex acuminate, base truncate, green, pubescent outside, glabrous inside, margins flat; petals free, sub equal; outer petals 3, 8–18 mm long, 1.3–2.6 mm wide, oblong-elliptic to ovate, apex acuminate, base rounded, green to light yellow, margins flat, pubescent outside, glabrous inside; inner petals 3, valvate, 8–18 mm long, 1.3–2.6 mm wide, elliptic to ovate, apex acuminate, base rounded, green to light yellow, margins flat, pubescent outside, glabrous inside; stamens 16 to 25, in 4 to 5 rows, 1–2 mm long, elongated; connective tongue-shaped, glabrous, green; staminodes absent; carpels free, 12 to 20, ovary 1–2 mm long, stigma ovoid, pubescent. Monocarps stipitate, stipes 5–10 mm long, 1–3 mm in diameter; monocarps 2 to 8, 8–18 mm long, 7–16 mm in diameter, ellipsoid to globose, apex rounded, glabrous, smooth, green turning wine red when ripe; seeds 1 to 4 per monocarp, 3–11 mm long, 3–11 mm in diameter, ellipsoid to flattened ellipsoid; aril absent.

#### Distribution.

Known from Nigeria to the Republic of Congo and the Democratic Republic of Congo; in Cameroon known from the East, South, Central, Littoral and South-West regions.

#### Habitat.

A very common species across the forest zone of Cameroon (growing in sympatry with ﻿*G.glabrum* in the south) with a wide ecologically amplitude; in lowland premontane, and sometimes in montane non-inundated primary or secondary forests. Altitude 20–1600 m a.s.l.

#### Local and common names known in Cameroon.

Moabé noir (dial. Nzime), Otunga (dial. Fang), Otungui (dial. Ewondo), Ntoulen (dial. Bassa), Botounga, Botunga (dial. Baka).

#### IUCN conservation status.

Least Concern (LC) (Harvey-Brown 2019c).

**Figure 29.**
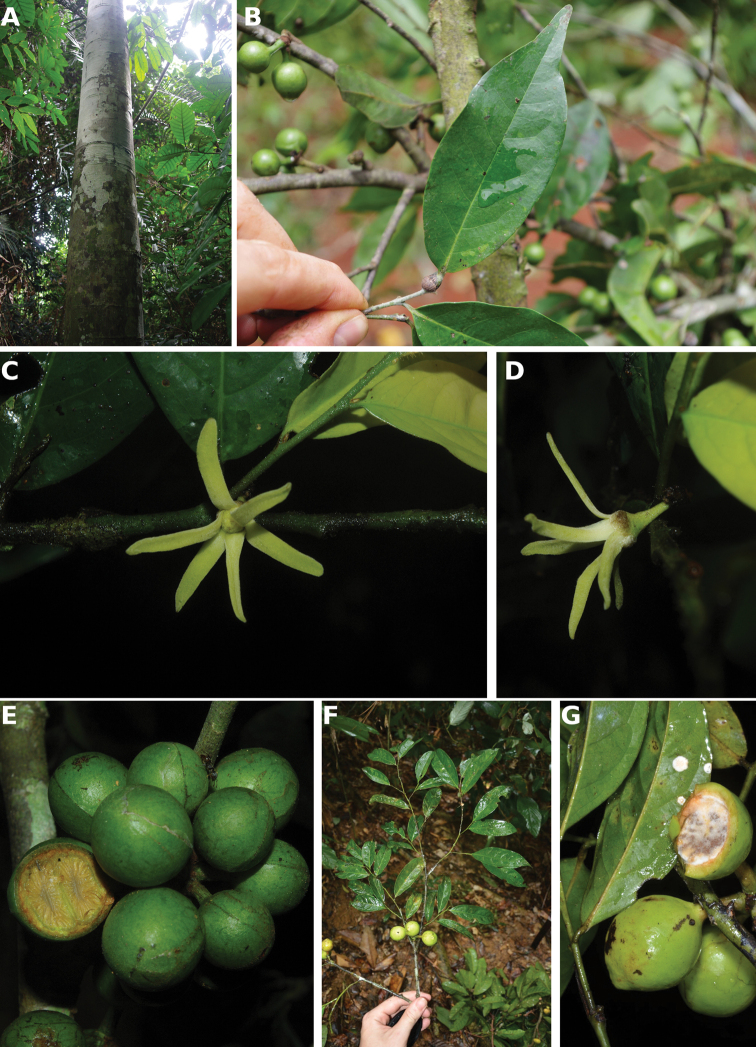
*Greenwayodendronsuaveolens***A** trunk, note light grey color **B** leaf, upper view **C** flower **D** flower, side view **E** detail of fruit, with longitudinal section of one monocarp showing 2 seeds. *Greenwayodendronglabrum***F** branch with fruit **G** fruit with longitudinal section of monocarp showing seed **A, E***Couvreur 476*, Lélé, Cameroon **B***Couvreur 1196*, Maséa, Cameroon **C, D***Couvreur 560*, Gabon **F, G***Bidault 847*, Gabon. Photos **A–E** Thomas L.P. Couvreur **F, G** Ehoarn Bidault, Tropicos.org, Missouri Botanical Garden.

#### Uses in Cameroon.

***medicine***: leaves as pain-killers, against arthritis, rheumatism, fabrifuges, for menstrual cycle; bark for pregnancy, antiaborifacients, root for vermifuges, as genital stimulants/depressants, dropsy, swellings, oede gout; constructions: building materials; ***dyes and tannins***: glycosides, saponims, steroids; ***products***: fibre wood, farming, forestry, hunting and fishing apparatus.

#### Notes.

See under ﻿*G.glabrum*. The species is here treated in the narrow sense; the former varieties *gabonicum* Le Thomas and *usambaricum* Verdc. (not recorded from Cameroon) are now raised to specific rank, as ﻿*G.gabonicum* (Le Thomas) Lissambou & Couvreur and ﻿*G.usambaricum* (Verdc.) Lissambou, Hardy & Couvreur (Lissambou et al. 2019).

#### Selected specimens examined.

**Central Region**: Ndanan 1, 3.62°N, 11.58°E, *21 October 2002*, *Cheek M.* 11224 (K,YA); Mefou National Park, 3.61°N, 11.58°E, *13 March 2004*, *Cheek M.* 27 (YA); Mefou National Park, 3.61°N, 11.58°E, *13 March 2004*, *Cheek M.* 66 (YA); Ca 50 km S of Badjob ca 60 km SW Of Eséka Along the Njong-River, 3.68°N, 10.68°E, *19 March 1964*, *de Wilde W.J.J.O* 2133 (B,BR,K,MO,P,WAG,YA); Yaoundé, 3.87°N, 11.52°E, *01 January 1935*, *Foury P.* 129 (P); Ngoro, 5.06°N, 11.19°E, *29 April 2017*, *Kamdem N.* 510 (YA); AYOS, 3.98°N, 12.36°E, *17 June 2017*, *Kamdem N.* 560 (YA); Ossoéssam (Mbalmayo), 3.52°N, 11.5°E, *01 June 1965*, *Leeuwenberg A.J.M.* 5755 (BR,K,P,WAG). **East Region**: Palisco forest consession 15 km along main road into consession, 3.48°N, 13.59°E, *27 March 2015*, *Couvreur T.L.P.* 756 (WAG,YA); Deng Deng, 5.20°N, 13.13°E, *27 July 2014*, *Kamdem N.* 166 (YA); Mindourou Alpicam, 4.12°N, 14.54°E, *11 December 2016*, *Kamdem N.* 459 (YA); Colline à l’ENE de Mbalam (140 km ESE de Djoum près de Souanke-Congo, 2.22°N, 13.82°E, *20 January 1973*, *Letouzey R.* 11866 (YA); A 6 km au Nord de Mwapak (km 43 piste Yokadouma-Lomié, 3.54°N, 14.71°E, *22 June 1963*, *Letouzey R.* 5322 (YA). **Littoral Region**: Mapubi 30 km before Edea on Yaoundé-Edea road On forestry road 5 km direction to Sanaga river, 3.84°N, 10.38°E, *28 February 2018*, *Couvreur T.L.P.* 1180 (WAG,YA); Mambe Massif above Boga village 100 km along road from Yaoundé to Ed 3.90°N, 10.77°E, *20 June 2014*, *Couvreur T.L.P.* 658 (WAG,YA); Olombé, 3.60°N, 9.959°E, *05 November 2014*, *Kamdem N.* 175 (YA); Chantier Bakaka km 4 Eboné-EkoMtolo road (Eboné situated on km 11 of Nkongsamba-Loum road), 4.83°N, 9.9°E, *20 August 1971*, *Leeuwenberg A.J.M.* 8164 (BR,K,L,MO,P,U,WAG,YA). **South Region**: 20 km from Kribi Lolodorf road, 3.03°N, 10.05°E, *09 June 1969*, *Bos J.J.* 4769 (B,BR,K,LD,LM,MO,P,POZG,WAG,YA); Mt Elephant ca 18 km SE of Kribi, 2.81°N, 10.01°E, *08 January 1970*, *Bos J.J.* 6100 (BR,C,K,LD,P,WAG,YA); hill above Nlonacko near village Ebianemeyong, 2.43°N, 10.35°E, *12 December 1998*, *de Wilde J.J.F.E* 12163 (BR,KRIBI,MO,S,WAG); 16 km on the recently reconstructed road from Ebolowa to Minkok, 2.75°N, 11.25°E, *30 January 1975*, *de Wilde J.J.F.E* 7940 (BR,K,MO,P,U,WAG,YA); Massif de Ngovayang village de Atog Boga, 3.25°N, 10.49°E, *30 August 2015*, *Droissart V.* 2050 (BRLU); Massif de Ngovayang village de Atog Boga, 3.25°N, 10.49°E, *05 September 2015*, *Droissart V.* 2159 (BRLU); A 13 km au N-NW de Djoum (UFA 09-007) vers “la tache verte” Forêt dense inondée et forêt secondaire, 2.77°N, 12.74°E, *25 April 2011*, *Droissart V.* 834 (BRLU); Ebom, 3.1°N, 10.73°E, *20 February 1996*, *Elad M.* 443 (KRIBI,WAG); Campo, 2.28°N, 9.950°E, *05 July 2015*, *Kamdem N.* 329 (YA); Campo, 2.39°N, 10.02°E, *07 July 2015*, *Kamdem N.* 349 (YA); Ma’an, 2.50°N, 10.76°E, *11 July 2015*, *Kamdem N.* 397 (YA); Essam (Nanga Eboko), 4.68°N, 12.37°E, *13 February 1959*, *Letouzey R.* 1106 (P); Essam (Nanga Eboko), 4.68°N, 12.37°E, *13 February 1959*, *Letouzey R.* 1313 (P); 10 km environ à l’ESE de Campo à Kribi, 2.37°N, 9.82°E, *26 March 1968*, *Letouzey R.* 9198 (YA); Campo-Ma’an National Park, 2.38°N, 10.06°E, *01 July 2001*, *van Andel T.R.* 3794 (KRIBI,WAG,YA); Bipindi, 3.08°N, 10.41°E, *1897*, *Zenker G.A.* 1278 (L,P,WAG); Bipindi, 3.08°N, 10.41°E, *1899*, *Zenker G.A.* 2062 (L,P,WAG); Bipindi, 3.08°N, 10.42°E, *01 January 1900*, *Zenker G.A.* 2166 (L,P,WAG). **South-West Region**: Bayang Mbo Wildlife Sanctuary after Mbu river, 5.35°N, 9.501°E, *25 March 2016*, *Couvreur T.L.P.* 1002 (WAG,YA); Mokoko Forest Reserve Boa/Likinge (Bousa forest), 4.42°N, 8.972°E, *05 June 1994*, *Ekema S.N.* 1208 (K,YA); Nguti, 5.34°N, 9.496°E, *03 June 2017*, *Kamdem N.* 537 (YA).

### ﻿﻿ Hexalobus

Taxon classificationPlantaeMagnolialesAnnonaceae

A. DC., Mém. Soc. Phys. Genève 5: 212, 1832

F1AB1C3A-FE22-5C32-83E1-829B0382BB5A

#### Type species.

﻿﻿﻿*Hexalobusmonopetalus* (A. Rich.) Engl. & Diels.

#### Description.

Trees, 10–40 m tall, d.b.h. 35–100 cm; stilt roots or buttresses absent, but trunk strongly fluted. Indumentum of simple hairs. Leaves: petiole 1–8 mm long, 1–4 mm in diameter; blade 3.6–36 cm long, 1.2–10 cm wide, elliptic or obovate or ovate, apex acuminate or rounded to obtuse, base cuneate or cordate, concolorous; midrib sunken or flat; secondary veins 5 to 17 pairs; tertiary venation reticulate. Individuals bisexual; inflorescences ramiflorous on old leafless or young foliate branches, axillary. Flowers with 9 perianth parts in 2 whorls, 1 to 3 per inflorescence; pedicel (0)1–15 mm long, 1–5 mm in diameter; in fruit 2–30 mm long, 1–5 mm in diameter; bracts 5 to 6, several basal and two (sometimes fused) on upper half of pedicel; sepals 3, valvate, free, 4–21 mm long, 3–14 mm wide, ovate, apex acute, base truncate; petals 6, in a single whorl and basally fused, tube 2–10 mm long, inner and outer whorl not differentiated, equal or subequal; lobes 9–80 mm long, 3–21 mm wide, margins plicate (folded in bud) or wavy; stamens numerous, in 10 to 13 rows, 1–8 mm long, elongated; connective discoid or elongated; staminodes absent; carpels free, 2 to 16, ovary 2–5 mm long, stigma bilobed or divided into two lobes with margins coiled inwards. Fruit apocarpous, monocarps stipitate or sessile, stipes 0–3 mm long; monocarps 1 to 8, 22–95 mm long, 13–65 mm in diameter, ellipsoid to cylindrical, apex rounded, smooth or rugose or warty, pubescent, orange-brown to medium brown when ripe; seeds 2 to 36, 10–40 mm long, 7–20 mm in diameter, flattened ellipsoid; aril absent.

A genus of five species, distributed across Africa. Four species are known from Cameroon, one endemic.

This genus of trees is characterized by thin plicate (folded) petals, a unique character for Annonaceae (Botermans et al. 2011). In addition, the petals are fused at the base and form a short tube with 6 lobes, a character otherwise only seen in ﻿*Isolona*. The trunk of adult trees is strongly fluted, a character also seen in the larger species of the genus ﻿*Isolona* (e.g. ﻿*I.hexaloba*).

#### Taxonomy.

Botermans et al. (2011).

### ﻿Key to the species of ﻿*Hexalobus* in Cameroon:

**Table:** 

1	Leaf apex rounded to obtuse; pedicel 0–2 mm long, in drier regions of northern Cameroon	﻿***H.monopetalus***
–	Leaf apex acuminate; pedicel 8–25 mm long, in wetter regions of southern Cameroon	**2**
2	Petiole > 2.5 mm in diameter; stamens 6–8 mm long; monocarps irregularly ribbed, rugose	﻿***H.bussei***
–	Petiole < 2.5 mm in diameter; stamens > 5 mm long; monocarps not ribbed, smooth or verrucose	**3**
3	Leaf blade 5–10 cm long, 1.5–4 cm wide, base cuneate; corolla lobes < 30 mm long; stamens ca. 2 mm long; carpels 3–4; monocarps verrucose	﻿***H.salicifolius***
–	Leaf blade 7–25 cm long, 2.5–8.5 cm wide, base rounded to cordate or occasionally cuneate; corolla lobes > 35 mm long; stamens 3–5 mm long; carpels 7–16; monocarps smooth	﻿***H.crispiflorus***

### ﻿﻿﻿﻿ Hexalobus bussei

Taxon classificationPlantaeMagnolialesAnnonaceae

Diels, Bot. Jahrb. Syst. 39: 479, 1907

1615B5F9-89A6-56BB-887C-BEAD37A23793

Fig. 30 ; Map 4E

= Hexalobus megalophyllus Engl. & Diels, Bot. Jahrb. Syst. 39: 479, 1907. Type. Cameroon. South Region, Bipindi, Zenker G.A. 2889, 1904: holotype: B[B100154197]; isotypes: BR[BR0000015306210]; COI[COI00033178]; F; G[G00011590]; HBG[HBG518920]; K[K000198935]; L[L-0049297]; M[M0089222]; MO[MO-2246481]; P[P00315838]; S[S12-22791]; US[00098767]; WAG[WAG0053629]; WRSL; WU[WU 0025867]; Z[Z-000000827].

#### Type.

Cameroon. South Region; Kribi, *Busse W.C.O. 3216*, 1904: holotype: B[B 10 0154198].

#### Description.

**Tree, 20–30 m tall**, d.b.h. unknown; stilt roots or buttresses absent, **trunk slender, not fluted**. Indumentum of simple hairs; old leafless branches glabrous, young foliate branches pubescent. Leaves: petiole 1–7 mm long, 3–4 mm in diameter, pubescent, grooved, blade inserted on the side of the petiole; **blade 15.5–36 cm long, 5.5–10 cm wide**, obovate, apex acuminate, acumen 0.5–1 cm long, base cordate, coriaceous, below glabrous when young and old, above sparsely pubescent to glabrous when young, sparsely pubescent to glabrous when old, concolorous; midrib sunken or flat, above pubescent when young and old, below pubescent when young and old; secondary veins 12 to 17 pairs; tertiary venation reticulate. Individuals bisexual; inflorescences ramiflorous on old leafless or young foliate branches, axillary. Flowers with 9 perianth parts in 2 whorls, 1 to 2 per inflorescence; pedicel 10–12 mm long, 4–5 mm in diameter, densely pubescent; in fruit 10–13 mm long, 2–3 mm in diameter, glabrous; bracts 5 to 6, several basal and two (sometimes fused) towards the upper half of pedicel, basal bracts 3–9 mm long, 2–5 mm wide; upper bracts 6–13 mm long, 2–3 mm wide; sepals 3, valvate, free, 16–20 mm long, 11–14 mm wide, ovate, apex acute, base truncate, brown, densely pubescent outside, glabrous inside, margins flat; petals basally fused, tube 2–5 mm long, purple, inner and outer whorl not differentiated, equal, lobes 23–40 mm long, 8–10 mm wide, elliptic, apex rounded, pale yellow, margins wavy, densely pubescent outside, densely pubescent inside, plicate; stamens numerous, 7–8 mm long, elongated; connective elongated, glabrous; staminodes absent; carpels free, 3 to 7, ovary 4–5 mm long, stigma elongate, pubescent. Monocarps stipitate, ca. 1 mm long, ca. 3 mm in diameter; monocarps 2 to 4, 53–78 mm long, ca. 40 mm in diameter, ellipsoid, apex rounded, sparsely pubescent, **rugose, irregularly ribbed in reticulate pattern, orange when ripe**; seeds 17 to 19 per monocarp, 23–28 mm long, 17–19 mm in diameter, flattened ellipsoid; aril absent.

**Figure 30.**
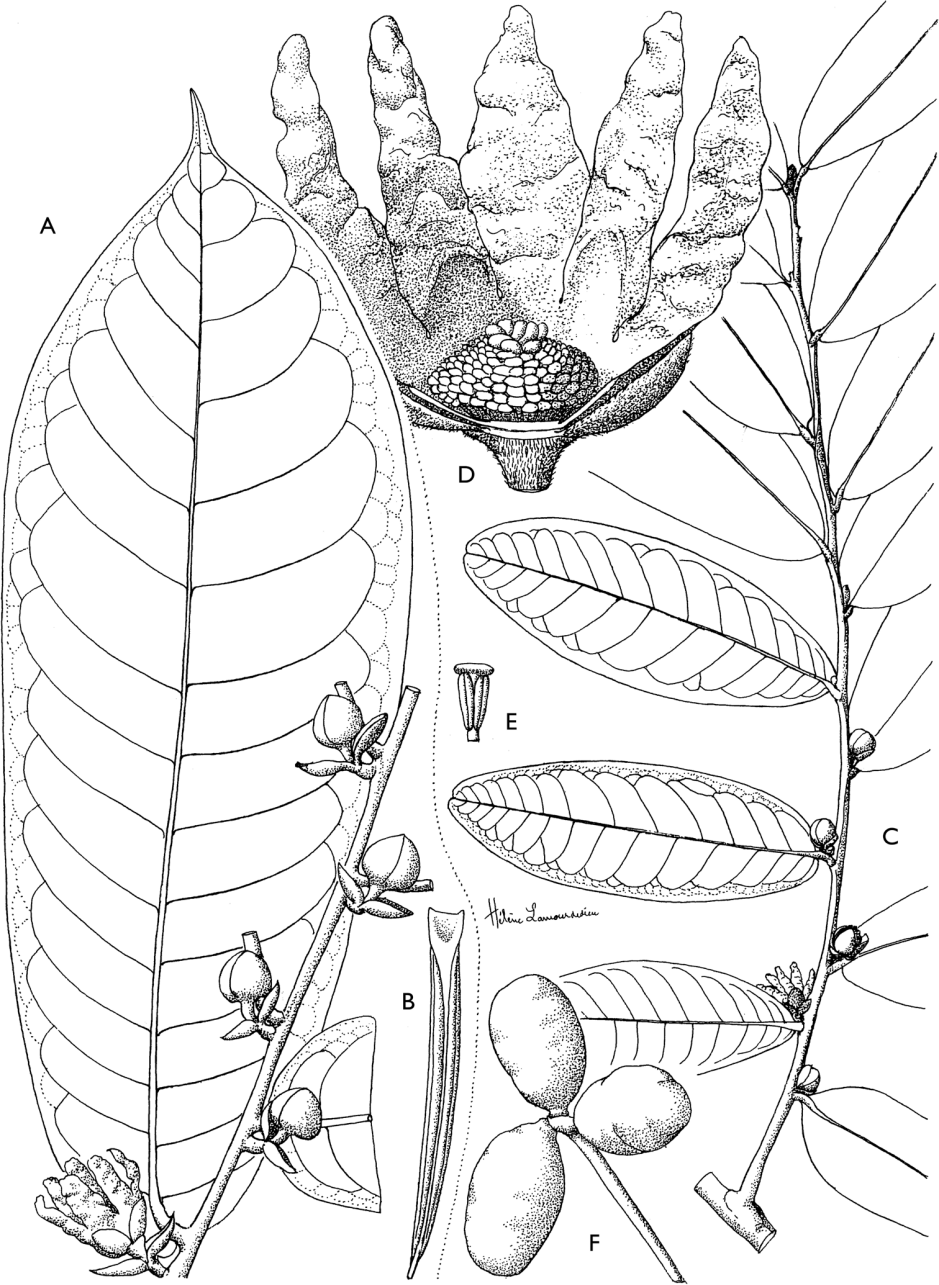
*Hexalobusbussei***A** flowering branch **B** stamen, front view. *Hexalobusmonopetalus***C** flowering branch **D** opened flower, one sepal and one petal removed **E** stamen, front view **F** portion of pedicel with three monocarps **A, B** from *Zenker 3550***C–E** from *Letouzey 7301***F** from *Chevalier 305*. Drawings by Hélène Lamourdedieu, Publications Scientifiques du Muséum national d’Histoire naturelle, Paris; modified from Botermans et al. (2011, fig. 3, p. 35).

#### Distribution.

endemic to Cameroon; known from the South region.

#### Habitat.

A rare species, in primary lowland rain forests, sometimes riverine. Altitude 0–200 m a.s.l.

#### Local and common names known in Cameroon.

None recorded.

#### IUCN conservation status.

Endangered (EN) (Cosiaux et al. 2019l).

#### Uses in Cameroon.

None recorded.

#### Notes.

﻿﻿﻿*Hexalobusbussei* is distinguished by its large leaf blades (15–36 cm long) and the irregularly ridged and strongly rugose surface of its monocarps.

#### Specimens examined.

**South Region**: S bank of Kienke river just E of Kribi, 3.1°N, 10.25°E, *12 May 1969*, *Bos J.J.* 4495 (P,WAG); 21 km from Kribi high forest exploitation N of Lolodorf road, 3.03°N, 10.05°E, *07 August 1969*, *Bos J.J.* 5157 (BR,P,WAG,YA); ca 16 km from Kribi Lolodorf road, 3°N, 10.01°E, *19 September 1969*, *Bos J.J.* 5370 (BR,C,K,LD,LM,MO,P,WAG,YA); Kribi, 2.95°N, 9.916°E, *01 September 1904*, *Busse W.C.O.* 3216 (B); 31 km ESE Kribi N of Kienke River Nyabessan, 2.9°N, 10.16°E, *19 April 1968*, *Letouzey R.* 9387 (P,YA); Bipindi, 3.08°N, 10.42°E, *01 January 1904*, *Zenker G.A.* 2889 (B,BR,COI,F,G,L,M,MO,P,S,WAG); Bipindi, 3.08°N, 10.42°E, *26 October 1908*, *Zenker G.A.* 3550 (BR,COI,G,M,MO,P,S); Bipindi, 3.08°N, 10.41°E, *01 January 1908*, *Zenker G.A.* 3592 (G,K); Bipindi, 3.08°N, 10.41°E, *01 January 1909*, *Zenker G.A.* 3889 (BR,COI,G,K,L,M,MO,P,S); Bipindi, 3.08°N, 10.42°E, *01 January 1913*, *Zenker G.A.* 4831 (BR,G,P,S).

### ﻿﻿﻿﻿ Hexalobus crispiflorus

Taxon classificationPlantaeMagnolialesAnnonaceae

A. Rich., Sagra, Hist. phys. Cuba, Bot. Pl. vasc. 1: 43, 1845

27845401-BCE2-560F-BA3F-7CF57BC93172

**Figure 31.**
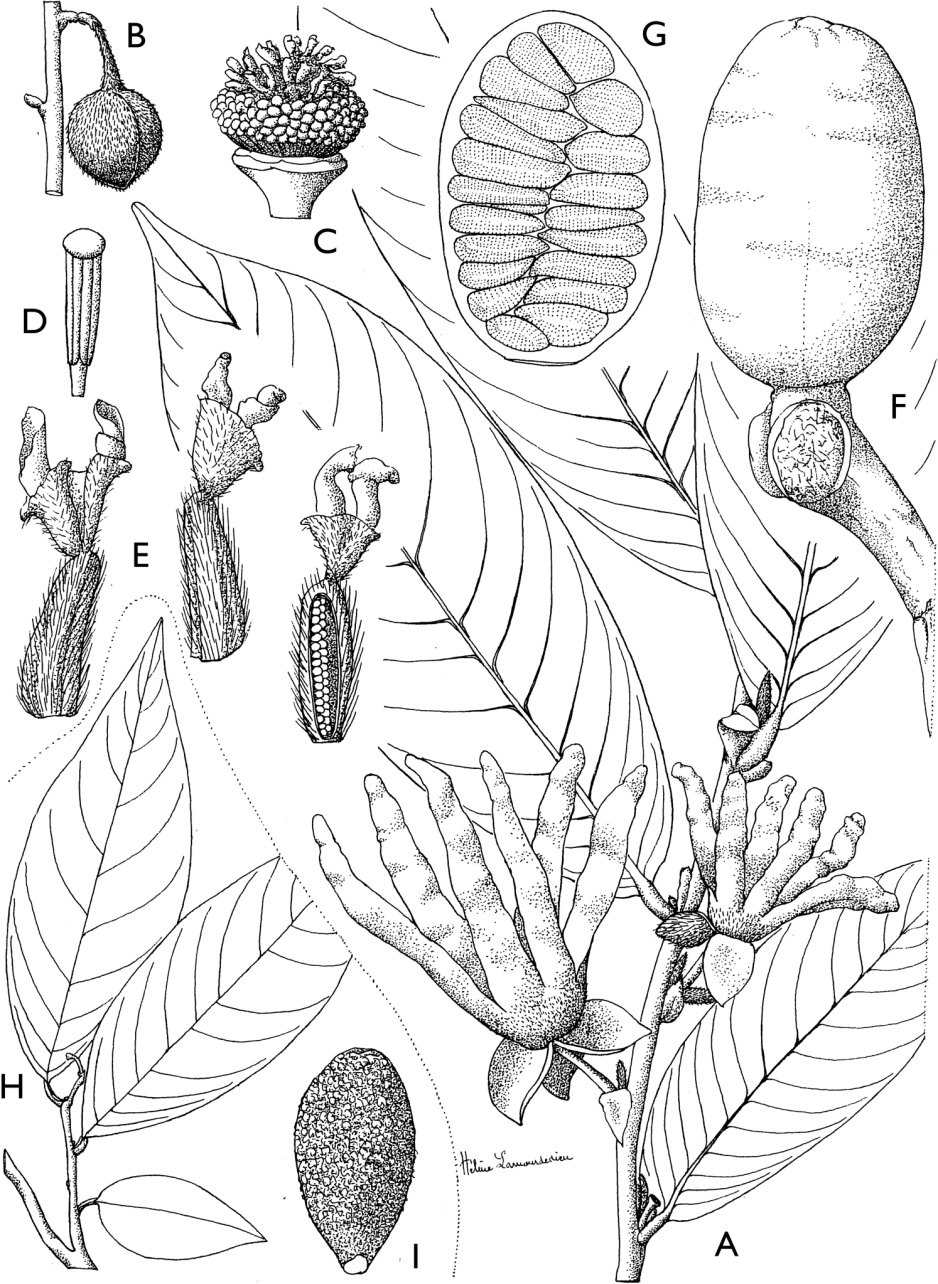
*Hexalobuscrispiflorus***A** flowering branch **B** flower bud **C** receptacle, petals removed **D** stamen, front view **E** three carpels different views, one with longitudinal section showing ovules **F** pedicel with one monocarp, note smooth surface **G** longitudinal section of monocarp showing seeds. *Hexalobussalicifolius***H** branch **I** a single detached monocarp, note verrucose surface **A, C–F** from *Le Testu 8838***B** from *Le Testu 693***G, H** from *Chevalier 7471***I** from *Le Testu 6387***J** from *Zenker 2268*. Drawings by Hélène Lamourdedieu, Publications Scientifiques du Muséum national d’Histoire naturelle, Paris; modified from Le Thomas (1969b, pl. 14, p. 85).

**Figure 32.**
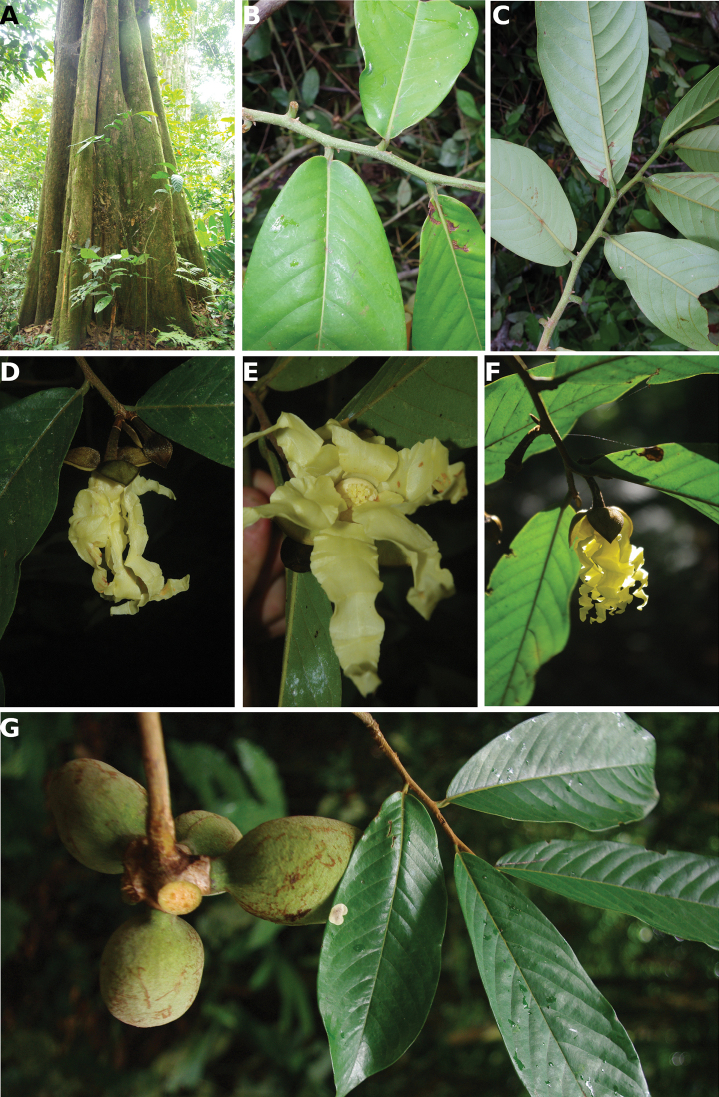
*Hexalobuscrispiflorus***A** base of trunk, note deep furrows **B** base of leaf blade, upper view **C** base of leaf blade, lower view **D** flower, side view **E** flower, top view **F** flowering branch **G** fruit and leaves, note smooth monocarp surface **A***Couvreur 506*, Ottotomo, Cameroon **B, C***Couvreur 1197*, Maséa, Cameroon **D–F***Couvreur 666*, Ottotomo, Cameroon **G***Couvreur 446*, Ottotomo, Cameroon. Photos Thomas L.P. Couvreur.

Figs 31 , 32 ; Map 4F

= Hexalobus grandiflorus Benth., Trans. Linn. Soc. London 23(3): 468, 1862. Type. Cameroon. South-West Region, “Ambas Bay”, *Mann G. 709*, 1861: lectotype, here designated, sheet here designated: K[K000582047]; isolectotypes: GH *n.v.*; K[K000105530, K000105529]; P[P00315844, P00315845].
= Hexalobus grandiflorus Benth. var. inaequilaterifolius Engl., Monogr. Afrik. Pflanzen.-Fam. 6: 57, 1901. Type. Republic of Congo: Cuvette, “Bonga, Sanga”, *Schlechter F.R.R. 12685*, Aug 1899: holotype: B *n.v.*; isotypes: BR[BR0000006915513]; WRSL *n.v.*
= Hexalobus lujae De Wild., Bull. Jard. Bot. État Brux. 4: 389, 1914. Type. Democratic Republic of the Congo. Kasai-Oriental, Sankuru, *Luja E.P. s.n.*, Jun 1910: lectotype, sheet here designated: BR[BR0000008800336]; isotypes: BR[BR0000008800008, BR0000008799906].
= Hexalobus crispiflorus A.Rich. subsp. strigulosus R.E.Fr., Acta Horti Berg. 10: 71, 1930. Type. Cameroon. no location, Deistel H. 99, no date: holotype: B[B 10 0184706]; isotypes: B[B 10 0184707, B 10 0184708, B 10 0184706, B 10 0184709, B 10 0184710, B 10 0184711]; GH; M[M0089315, M0089316]; P[P00486245].
= Hexalobus mbula Exell, J. Bot. 70, suppl. Polypet.: 206, 1932. Type. Angola. Cabinda, Buco Zau, Fazenda Alsyra, *Gossweiler J. 6939*, 20 Jan 1917: lectotype, designated by Botermans et al. (2011), p. 41: BM *n.v.*; isolectotypes: COI[COI00077206]; LISC[LISC000086, LISC000089, LISC000085, LISC000088, LISC000087].

#### Type.

Guinea. Labé; Fouta D’hiallon [Djallon], *Heudelot, J. 865*, Apr 1838: lectotype, sheet here designated: P[P00315839]; isotypes: P[P00315842, P00486270, P00315841]; G[G00011589].

#### Description.

**Tree, 25–40 m tall**, d.b.h. up to 100 cm; stilt roots or buttresses absent, **trunk deeply fluted**. Indumentum of simple hairs; old leafless branches glabrous, young foliate branches densely pubescent. Leaves: petiole 2–8 mm long, 1–3 mm in diameter, densely pubescent, grooved, blade inserted on the side of the petiole; blade **7.2–25 cm long**, 2.5–8.5 cm wide, ovate to obovate, apex acuminate, acumen 0.5–1.5 cm long, base cuneate to cordate, coriaceous, below glabrous when young and old, above sparsely pubescent when young, sparsely pubescent when old, concolorous; midrib sunken or flat, above pubescent when young and old, below pubescent when young and old; secondary veins 9 to 19 pairs; tertiary venation reticulate. Individuals bisexual; inflorescences ramiflorous on old leafless or young foliate branches, axillary. Flowers with 9 perianth parts in 2 whorls, 1 to 3 per inflorescence; **pedicel 12–25 mm long**, 1–2 mm in diameter, glabrous; in fruit 10–30 mm long, 4–5 mm in diameter, glabrous; bracts 5 to 6, several basal and two (sometimes fused) towards the upper half of the pedicel, basal bracts 3–9 mm long, 2–5 mm wide; upper bracts 8–12 mm long, 4–9 mm wide; sepals 3, valvate, free, 12–21 mm long, 9–12 mm wide, ovate, apex acute, base truncate, brown, densely pubescent outside, densely pubescent inside, margins flat; petals basally fused, tube 4–10 mm long, purple, inner and outer whorl not differentiated, sub equal; lobes 37–80 mm long, 6–21 mm wide, elliptic, apex rounded, green to bright yellow, margins wavy, pubescent outside, pubescent with glabrous base inside, plicate; stamens 190 to 210, in 10 to 13 rows, 3–5 mm long, elongated; connective hemispheric, glabrous, cream; staminodes absent; **carpels free, 7 to 16**, ovary 2–5 mm long, stigma bilobed, slightly capitate, pubescent. Monocarps stipitate, ca. 2 mm long, 2–3 mm in diameter; monocarps 1 to 8, (42)50–95 mm long, 35–65 mm in diameter, ellipsoid to oblong, apex rounded, pubescent to glabrous, **smooth, not ribbed**, rusty-brown; seeds 12 to 36 per monocarp, 28–40 mm long, 17–20 mm in diameter, flattened ellipsoid; aril absent.

#### Distribution.

A widespread species, known from Guinea-Bissau to the Democratic Republic of Congo; in Cameroon known from the East, South, Central, Littoral and South-West regions.

#### Habitat.

A common species across the forested region of the country; in lowland or premontane periodically or non-inundated, primary or secondary rain forests, including gallery forests in savanna. Altitude 0–1000 m a.s.l.

#### Local and common names known in Cameroon.

évota, pota (dial. Bibaya, baka), owé (dial. Ewondo, *Letouzey 4433*); Ow (dial. Bulu, *Ndoum 129*); Owoe (South Province, *Mildbraed 5652*); Pota (dial. Bambindjere?, *Harris, Fay 516, 558, 883, 1518*).

#### IUCN conservation status.

Least Concern (LC) (Cosiaux et al. 2019m).

#### Uses in Cameroon.

***dyes and tannins***: lycosides, saponims, steroids.

#### Uses in Cameroon.

None recorded.

#### Notes.

﻿﻿﻿*Hexalobuscrispiflorus* is morphologically close to ﻿*H.salicifolius*, both being tall trees with deeply fluted trunks, similar flowers and growing in similar habitats. ﻿﻿﻿*Hexalobuscrispiflorus* is however distinguished by its larger leaves (7.2–25 cm versus 5–10 cm long), more numerous carpels (7 to 16 versus 2 to 4 in ﻿*H.salicifolius*) and smooth monocarps (versus verrucose in ﻿*H.salicifolius*).

#### Specimens examined.

**Central Region**: Ndanan 2 to Mefou river, 3.62°N, 11.56°E, *13 October 2002*, *Cheek M.* 11064 (K,YA); Ottotomo Forest Reserve 1 km after reserve base near small loggers road, 3.66°N, 11.28°E, *25 June 2013*, *Couvreur T.L.P.* 446 (WAG,YA); Ottotomo Forest Reserve 3 km after reserve base near small loggers road, 3.66°N, 11.28°E, *08 September 2013*, *Couvreur T.L.P.* 506 (WAG,YA); Ottotomo Forest Reserve 45 km South of Yaoundé 5 km on path into reserve, 3.66°N, 11.28°E, *15 January 2015*, *Couvreur T.L.P.* 666 (WAG,YA). **East Region**: 73 km south of Yokadouma 30 km after Ngato 15 km after river ALPICAM ‘base de vie’ then 40 km on forestry road starting 4 km before Maséa village, 3.16°N, 14.71°E, *04 March 2019*, *Couvreur T.L.P.* 1197 (MPU,WAG,YA); Sangha R (Ndakan), 2.78°N, 16°E, *08 March 1988*, *Fay J.M.* 8299 (F,MO,P); West side of Sangha River, 2.35°N, 16.13°E, *01 November 1988*, *Harris D.J.* 1518 (K,MO); West side of Sangha River, 2.35°N, 16.13°E, *02 August 1988*, *Harris D.J.* 883 (MO,P); Rives de la Sangha près Lidjombo 120 km au N de Ouesso, 2.61°N, 16°E, *08 April 1971*, *Letouzey R.* 10614 (P,YA); A 15 km au Sud de Djouo (20 km E de Somalomo dur le Dja), 3.32°N, 12.93°E, *25 February 1962*, *Letouzey R.* 4433 (P,YA); South Cameroon Forest Area Molundu District Bange forest between Lokomo Bumba and Bange, 2.83°N, 15.25°E, *22 February 1911*, *Mildbraed G.W.J.* 4539 (HBG); South Cameroon Forest Area Molundu District between Yokadouma (Post Plehn) and Assobam, 3.4°N, 14.38°E, *21 April 1911*, *Mildbraed G.W.J.* 4996 (HBG); Réserve de Biosphère du Dja vers 500 m de la station de Bouamir, 3.19°N, 12.81°E, *27 May 2001*, *Senterre B.* 1641 (BR); Layon nord-sud à partir de Djolimpoun (entre Somalomo-Malen), 3.33°N, 12.87°E, *13 September 1993*, *Sonké B.* 591 (YA). **Littoral Region**: km 11 Loum-Solé road, 4.7°N, 9.816°E, *24 May 1972*, *Leeuwenberg A.J.M.* 9904 (K,MO,WAG,YA). **South Region**: Hill roughly between Nkolandom and Nkoemvone, 2.8°N, 11.16°E, *17 January 1975*, *de Wilde J.J.F.E* 7909 (B,BR,C,K,MO,P,U,WAG,YA); Ebolowa, 2.91°N, 11.31°E, *01 June 1911*, *Mildbraed G.W.J.* 5652 (HBG); Ebom, 3.1°N, 10.71°E, *24 September 1996*, *Ndoum D.* 129 (KRIBI,WAG); Kribi, 2.92°N, 9.900°E, *01 October 1997*, *van der Burgt X.M.* 232 (KRIBI,WAG); Bisyang, 2.98°N, 9.968°E, *04 June 2006*, *van Velzen R.* 90 (BR,G,MO,WAG). **South-West Region**: Melon to Nyandon ca 2 km, 4.93°N, 9.533°E, *28 November 1998*, *Cheek M.* 9716 (K,WAG,YA); Nyasoso village on max’s trail to Mt 4.82°N, 9.701°E, *05 April 2016*, *Couvreur T.L.P.* 1062 (WAG,YA); Ambas Bay, 4.01°N, 9.2°E, *1861*, *Mann G.* 709 (GH,K,P).

### ﻿﻿﻿﻿ Hexalobus monopetalus

Taxon classificationPlantaeMagnolialesAnnonaceae

(A. Rich.) Engl. & Diels, Monogr. Afrik. Pflanzen.-Fam. 6: 56, 1901

737AB2B7-52AE-582F-9DAB-379137C24196

Fig. 30 ; Map 4G

≡ Uvaria monopetala A.Rich., Guill. & Perr., Fl. Seneg. tent.: 8, 1831. ﻿﻿Hexalobussenegalensis A.DC., Mém. Soc. Phys. Genève 5: 213, 1832, superfluous name.
= Hexalobus monopetalus var. parvifolius Baker.f.; Macleod, Chiefs and cities of Central Africa: 301, 1912. Type. Central African Republic. Environs de Kaga M’bra, Chevalier A.J.B. 6486, 30 Nov 1902: holotype: K[K000582056]; isotypes: G[G00011614]; L[L0049298]; P; WAG[WAG0162940].
= Hexalobus tomentosus A.Chev., Expl. bot. Afr. occ. Énum. pl. 1: 10, 1920. Type. Mali. Ségou, Sansanding, Chevalier A.J.B. 2542, 29 Sep 1899: lectotype, designated by Botermans et al. (2011), p. 42: P[P00486157].
= Hexalobus glabrescens Hutch. & Dalziel, Fl. W. trop. Afr. 1: 52, 1927. Type. Central African Republic: Ouham, Lere to Ham, Talbot P.A. 531, 1911: lectotype, designated by Botermans et al. (2011), p. 42: K; isolectotypes: BM[BM000546380]; Z[Z-000034501].
= Hexalobus monopetalus (A. Rich.) Engl. & Diels var. obovatus Brenan, Mem. New York Bot. Gard. 8, 3: 214, 1953. Type. Zambia. North-Western, E. of Matonchi Farm, Milne-Redhead E.W.B.H. 4536, 12 Feb 1938: holotype: K[K000198933]; isotypes: BM[BM000546381]; BR[BR0000008800664]; PRE[PRE0397001-0].
= Hexalobus huillensis (Engl. & Diels) Engl. & Diels, Monogr. Afrik. Pflanzen.-Fam. 6: 56, 1901; ﻿﻿Uvariahuillensis Engl. & Diels, Notizbl. Königl. Bot. Gart. Berlin. 2: 296, 1899. Type. Angola. Benguela, Benguella, Huilla, *Antunes J.M. 266*, no date: lectotype, designated by Botermans et al. (2011), p. 42: COI[COI00033141]; isolectotype: BM[BM000546379].

#### Type.

Senegal. Tambacounda; Galam prope Bakel, *Leprieur F.M.R. s.n.*, 1828: lectotype, designated by Botermans et al. (2011), p. 42: G[G00011597]; isolectotypes: G[G00011595, G00011593]; P[P00315834, P00315832, P00315836].

#### Description.

**Tree to shrub, 10–15 m tall**, d.b.h. up to 35 cm; stilt roots or buttresses absent, **often several stemmed, not fluted**. Indumentum of simple hairs; old leafless branches glabrous, young foliate branches pubescent. Leaves: petiole 1–4 mm long, 1–2 mm in diameter, densely pubescent, grooved, blade inserted on the side of the petiole; blade 3.6–17.5 cm long, 1.2–6.5 cm wide, ovate to obovate, apex rounded to obtuse, rarely acuminate, acumen 1 cm long, base cuneate to cordate, coriaceous, below pubescent when young, glabrous when old, above pubescent when young, glabrous when old, concolorous; midrib impressed, above pubescent when young, glabrous when old, below pubescent when young, glabrous when old; secondary veins 6 to 14 pairs, below; tertiary venation reticulate. Individuals bisexual; inflorescences ramiflorous on young foliate branches, more rarely cauliflorous, axillary. Flowers with 9 perianth parts in 2 whorls, 1 to 3 per inflorescence; **pedicel sessile or short up to 2 mm long**, ca. 2 mm in diameter when present, densely pubescent; in fruit 2–4 mm long, 1–2 mm in diameter, sparsely pubescent; bracts 5 to 6, several basal and two (sometimes fused) towards the upper half of pedicel, basal bracts ca. 5 mm long, ca. 4 mm wide; upper bracts ca. 5 mm long, ca. 4 mm wide; sepals 3, valvate, free, 4–7 mm long, 3–6 mm wide, ovate, apex acute, base truncate, brown, densely pubescent outside, glabrous inside, margins flat; petals basally fused, tube 3–4 mm long, inner and outer whorl not differentiated, sub equal; lobes 9–27 mm long, 3–7 mm wide, elliptic, apex rounded, cream, margins wavy, pubescent outside, pubescent inside, lobes curving inwards at the base and margins reflexed forming a hollow chamber; stamens numerous, 1–2 mm long, elongated; connective discoid, glabrous, cream-yellow; staminodes absent; carpels free, 2 to 7, ovary 2–3 mm long, stigma divided into two lobes with margins coiled inwards, pubescent. Monocarps stipitate to sessile, stipes < 3 mm long, 3–4 mm in diameter; monocarps1 to 5, **22–46 mm long, 13–22 mm in diameter**, ellipsoid to cylindrical, apex rounded, **sparsely pubescent, warty, constricted around the seeds**, orange when ripe; **seeds 2 to 8 per monocarp**, 10–15 mm long, 7–10 mm in diameter, flattened ellipsoid; aril absent.

#### Distribution.

A widespread species, known from Senegal to northern South Africa, with a disjunct population in southern Angola; in Cameroon known from the North and Far-North regions.

#### Habitat.

A common species in drier regions of Africa; in woodland, savannas or gallery forests, on sandy soils or in rocky places. Altitude 200–1000 m a.s.l.

#### Local and common names known in Cameroon.

Bohili (Fulfuldé) (Malzy 1954).

#### IUCN conservation status.

Least Concern (LC) (Cosiaux et al. 2019n).

#### Uses in Cameroon.

None recorded.

#### Notes.

﻿﻿﻿*Hexalobusmonopetalus* is distinguished from the other Cameroonian species by being a small deciduous shrub or tree (no taller than 15 m) growing in drier areas, with sessile or subsessile flowers and small smooth monocarps (up to 46 mm long versus more than 45 mm generally).

#### Selected specimens examined.

**Far-North Region**: Route Lara-Guidiguis (15 km ENE de Kaele), 10.1°N, 14.45°E, *29 August 1964*, *Letouzey R.* 6540 (P,YA); Près Bourka (65 km SS0 de Mokolo), 10.3°N, 13.56°E, *13 October 1964*, *Letouzey R.* 7301 (P,YA); 9 km SE Guili 10 km NE Bourrah, 10.6°N, 13.74°E, *27 November 1989*, *Villiers J.-F.* 4713 (P,YA). **North Region**: Ecole de faune de Garoua, 9.3°N, 13.4°E, *09 August 2000*, *Dong E.* 393 (YA); Sanguéré (10 km Garoua), 9.27°N, 13.47°E, *01 October 1949*, *Malzy P.* 309 (P,YA); Environs village Boulko au pied Hossere Gode 15 km NW de Poli, 8.53°N, 13.13°E, *24 October 1983*, *Satabié B.* 702 (P,YA); Collines de Tinguelin 10 km N de Garoua, 9.3°N, 13.4°E, *26 November 1984*, *Satabié B.* 781 (P,YA); map # NC 33 VIII Garoua, 9.93°N, 13.86°E, *13 August 1983*, *Thomas D.W.* 2432 (MO,P,WAG,YA).

### ﻿﻿﻿﻿ Hexalobus salicifolius

Taxon classificationPlantaeMagnolialesAnnonaceae

Engl., Monogr. Afrik. Pflanzen.-Fam. 6: 57, 1901

97E02B4A-373A-5B2E-AFF0-35CA4FDE1A97

Fig. 31 ; Map 4H

#### Type.

Cameroon. South Region; Bipindi, *Zenker G.A. 2268*, 1900: holotype: B[B 10 0154199]; isotypes: BM[BM000546383]; COI[COI00033180]; G[G00011622, G00011623]; GEOT[GOET005680]; HBG[HBG518921]; K[K000582046]; L[L 0049299]; M[M0089219]; MO *n.v.*; P[P00363369, P00363367]; S[S12-22767]; WAG[WAG0053744]; WU[WU0025889]; Z[Z-000034502].

#### Description.

**Tree, up to 35 m tall**, d.b.h. up to 100 cm; stilt roots or buttresses absent, trunk fluted. Indumentum of simple hairs; old leafless branches glabrous, young foliate branches densely pubescent. Leaves: petiole 1–3 mm long, 1–2 mm in diameter, sparsely pubescent, slightly grooved, blade inserted on top of the petiole; **blade 5–10 cm long**, 1.5–3.5 cm wide, elliptic, apex acuminate, acumen 0.5–1 cm long, base cuneate, coriaceous, above glabrous when young and old, above sparsely pubescent when young, sparsely pubescent when old, concolorous; midrib sunken or flat, below pubescent when young and old, below pubescent when young and old; secondary veins 5 to 12 pairs; tertiary venation reticulate. Individuals bisexual; inflorescences ramiflorous on young foliate branches, axillary. Flowers with 9 perianth parts in 3 whorls, 1 to 3 per inflorescence; pedicel 8–15 mm long, 1–2 mm in diameter, densely pubescent; in fruit 10–13 mm long, 2–3 mm in diameter, glabrous; bracts 5 to 6, several basal and two (sometimes fused) towards the upper half of pedicel, basal bracts 5 mm long, 3 mm wide; upper bracts 5 mm long, 3 mm wide; sepals 3, valvate, free, 7–11 mm long, 4–7 mm wide, ovate, apex acute, base truncate, brown, pubescent outside, densely pubescent inside, margins flat; petals basally fused, tube 2–4 mm long, purple, inner and outer whorl not differentiated, equal; lobes 17–30 mm long, 4–8 mm wide, elliptic, apex rounded, cream to yellow, margins wavy, pubescent outside, pubescent inside, plicate; stamens numerous, in 11 to 13 rows, ca. 2 mm long, elongated; connective discoid, glabrous, cream; staminodes absent; **carpels free, 3 to 4**, ovary 2–3 mm long, stigma divided into two lobes with margins coiled inwards, pubescent. Monocarps stipitate, 1–2 mm long, ca. 3 mm in diameter; monocarps 2 to 4, 60–93 mm long, 40–55 mm in diameter, ovoid to oblong, apex rounded, pubescent to glabrous, **verrucose**, rusty-brown when ripe; seeds 15 to 15 per monocarp, 22–31 mm long, 11–18 mm in diameter, flattened; aril absent.

#### Distribution.

Known from Cameroon, Gabon and the Republic of Congo (one specimen); in Cameroon known from the East, South and Central regions.

#### Habitat.

A fairly scarce species, in primary and secondary lowland and pre-montane rain forest, occasionally in semi-deciduous forests, in periodically inundated forests and on river banks. Altitude 50–900 m a.s.l.

#### Local and common names known in Cameroon.

Ooué (dial. Jaundi, *Hédin 1663*); Owoé (dial. Yetbou, *Médou 1703*).

#### IUCN conservation status.

Least Concern (LC) (Cosiaux et al. 2019o).

#### Uses in Cameroon.

None recorded.

#### Notes.

﻿﻿﻿*Hexalobussalicifolius* is morphologically close to ﻿*H.crispiflorus* from which it is distinguished by its smaller leaves, fewer carpels (2 to 4 versus 7 to 16 in ﻿*H.crispiflorus*) and verrucose monocarps (versus smooth in ﻿*H.crispiflorus*).

#### Specimens examined.

**South Region**: South Province 15 km from Kribi, 2.96°N, 9.933°E, *28 August 1970*, *Bos J.J.* s.n. (WAG[WAG0162941]); Campakok (Camp Akok), 2.65°N, 9.9°E, *30 October 1991*, *Hallé F.* 4181 (WAG); Colline près Mezese à 17 km ENE de Sangméli 2.95°N, 12.14°E, *19 October 1966*, *Letouzey R.* 8122 (BR,P,YA); Bidou, 2.85°N, 9.991°E, *28 May 1954*, *Médou J. SRFK* 1703 (P,YA); 22 km NE of Kribi Kribi-Bipindi road Bidou I cultivated fields NE of village, 3.00°N, 10.09°E, *04 June 2006*, *van Velzen R.* 98 (WAG); 26 km N of Kribi SE of the toll post for Kribi-Edéa road forest at Bebamboue II, 3.05°N, 9.987°E, *26 May 2006*, *van Velzen R.* 99 (WAG); Bipindi, 3.08°N, 10.42°E, *01 January 1900*, *Zenker G.A.* 2268 (COI,G,G,K,L,M,MO,P,S,WAG); Bipindi, 3.08°N, 10.42°E, *01 January 1907*, *Zenker G.A.* 3330 (BR,G,UPS).

### ﻿﻿ Isolona

Taxon classificationPlantaeMagnolialesAnnonaceae

Engl., Nat. Pflanzenfam. Nachtr. I: 161, 1897

5DC6684E-D8D8-5B48-8623-AD12C576C7F9

#### Type species.

﻿*Isolonamadagascariensis* (A.DC.) Engl. & Diels (a species from Madagascar).

#### Description.

Trees, 3–30 m tall, d.b.h. 5–60 cm; stilt roots or buttresses absent. Indumentum of simple hairs or glabrous. Leaves: petiole 1–15 mm long, 1–4 mm in diameter, blade 8.5–29 cm long, 3–15 cm wide, elliptic or obovate or oblong, apex acuminate, base decurrent to rounded or acute, concolorous; midrib **raised on upper surface**; secondary veins 7 to 20 pairs; tertiary venation reticulate. Inflorescences ramiflorous on old leafless or young foliate branches, axillary. Flowers bisexual with 9 perianth parts in 2 whorls, 1 to 3 per inflorescence; pedicel 1–25 mm long, 1–2 mm in diameter; in fruit 2–29 mm long, 2–5 mm in diameter; bracts 2 to 7, several basal and one upper, lower half of pedicel; sepals 3, valvate, free, 1–9 mm long, 2–5 mm wide, ovate or elliptic, apex acute or acuminate or rounded, base truncate; **petals 6, basally fused**, tube 3–11 mm long, **inner and outer whorl not differentiated**, equal; lobes 6–31 mm long, 2–12 mm wide; stamens numerous, in 3 to 4 rows, 1–2 mm long, broad; connective discoid; staminodes absent; **carpels fused - syncarpous, forming a single visible gynoecium**, 1–3 mm long, stigma bilobed, slightly capitate or capitate. **Fruit syncarpous**, **forming a single visible fruit**, 30–90 mm long, 15–50 mm in diameter, ovoid or ellipsoid, apex apiculate or rounded or cuspidate; seeds numerous not seriate, 8–25 mm long, 5–15 mm in diameter, ellipsoid or flattened ellipsoid; aril absent.

A genus of trees with 20 known species, 15 in Africa and 5 in Madagascar. In Cameroon nine species are known, none endemic.

﻿*Isolona*, together with its sister genus ﻿*Monodora*, are unique in Annonaceae in having truly syncarpous flowers (fused carpels) and fruits. This translates into single fruits with unordered seeds, in contrast to other genera which have either uni- or biseriate placentation. Petals in ﻿*Isolona* are basally fused forming a clearly visible tube, with six equal lobes of equal length in a single whorl. In the vegetative state, ﻿*Isolona* and ﻿*Monodora* (together with ﻿﻿﻿*Polyceratocarpuspellegrinii*) are characterized by a raised midrib, in contrast to a sunken or flat midrib in all other genera found in Cameroon.

#### Taxonomy.

Couvreur (2009).

### ﻿Key to the species of ﻿*Isolona* in Cameroon:

**Table:** 

1	Leaves and/or young foliate branches pubescent	**2**
–	Leaves and young foliate branches completely glabrous	**3**
2	Leaf blade inserted on top of petiole; lobes glabrous outside, pubescent inside	﻿***I.congolana***
–	Leaf blade inserted on side of petiole; lobes pubescent on both sides	﻿***I.pilosa***
3	Leaf blade inserted on top of petiole	**4**
–	Leaf blade inserted on sides of petiole	**5**
4	Flowering pedicels 14–25 mm long, corolla smooth in dried material; corolla lobes 8–15 mm long with flat margins; fruits not ribbed	﻿***I.cooperi***
–	Flowering pedicels 3–7 mm long, corolla clearly verrucose in dried material; corolla lobes 15–25 mm long with margins curving inwards; fruits ribbed longitudinally	﻿***I.zenkeri***
5	Sepals 4–9 mm long, papyraceous; upper bract, when present, halfway up the pedicel or subbasal, sometimes leaf-like; flowers campanulate	﻿***I.campanulata***
–	Sepals 1–3 mm long, coriaceous; upper bract absent or minute; flowers not campanulate	**6**
6	Base of leaf blade acute to obtuse	﻿***I.hexaloba***
–	Base of leaf blade decurrent to narrowly cuneate	**7**
7	Corolla lobes 4–10 times as long as wide; sepal margins glabrous	﻿***I.thonneri***
–	Corolla lobes 1.6–3.5 times as long as wide; sepal margins covered with short appressed hairs	**8**
8	Flowering pedicels 2–7 mm long; corolla lobes with rounded tips, narrowly elliptic to elliptic, the margins sparsely covered with short hairs	﻿***I.dewevrei***
–	Flowering pedicels 10–20 (-23) mm long; corolla lobes with acute tips, narrowly ovate to ovate, the margins glabrous	﻿***I.pleurocarpa***

### ﻿﻿﻿﻿ Isolona campanulata

Taxon classificationPlantaeMagnolialesAnnonaceae

Engl. & Diels, Monogr. Afrik. Pflanzen.-Fam. 6: 83, 1901

5CCBC668-2FFD-5D86-95E5-EE2A007F75D1

Fig. 33 ; Map 4I

= Isolona leonensis Sprague & Hutch., Bull. Misc. Inform. Kew: 151, 1916. Type. Sierra Leone. Northern Province, Yonibana, *Thomas N.W. 4230*, 30 Oct 1914: lectotype, designated by Couvreur (2009), p. 137: K[K000199016]. (this name was erroneously reported as “I.konensis” in Onana (2011)).
= Isolona soubreana A.Chev., Explor. Bot. Afrique Occ. Franc. 1: 12, 1920. Type. Ivory Coast. Sassandra, Chevalier A.J.B. 19088, 23 Jun 1907: lectotype, designated by Couvreur (2009), p. 137, sheet here designated: P[P00363265]; isolectotypes: P[P00363264, P00363266]; WAG[WAG0027026].

#### Type.

Cameroon. Northern Region; Bangwe, *Conrau G. 93*, 17 Oct 1899: holotype: B[B 10 0154200]; isotype: K[K000198841].

#### Description.

Tree to shrub, 10–15 m tall, d.b.h. 10–15 cm; stilt roots or buttresses absent. **Indumentum absent**; old leafless branches glabrous, young foliate branches glabrous. Leaves: petiole 3–6 mm long, 1–2 mm in diameter, glabrous, weakly grooved adaxially, blade inserted on the side of the petiole; blade 10–18 cm long, 3–7 cm wide, elliptic to obovate, apex acuminate, acumen 1 cm long, base decurrent to cuneate, subcoriaceous, below glabrous when young and old, above glabrous when young and old, concolorous; midrib raised, above glabrous when young and old, below glabrous when young and old; secondary veins 7 to 12 pairs, glabrous below; tertiary venation reticulate. Individuals bisexual; inflorescences ramiflorous on old or young foliate branches, axillary. Flowers with 9 perianth parts in 2 whorls, 1 to 2 per inflorescence; pedicel 5–30 mm long, ca. 1 mm in diameter, glabrous; in fruit 2–5 mm long, 2–3 mm in diameter, glabrous; bracts 2 to 5, several basal and one upper towards the lower half of pedicel, basal bracts 2–3 mm long, 1–2 mm wide; **upper bract sometimes leaf-like**, 2–19 mm long, 1–4 mm wide; **sepals 3, valvate, free, 4–9 mm long**, 3–5 mm wide, elliptic to ovate, apex acute, base truncate, green speckled with red and purple, glabrous inside, glabrous outside, margins flat; petals basally fused, tube 4–7 mm long, inner and outer whorl not differentiated, equal; **lobes 6–20 mm long, 2–7 mm wide, triangular, apex acute**, green, turning bright yellow, margins flat, glabrous outside, glabrous inside, spreading horizontally; stamens numerous, in 3 to 4 rows, ca. 1 mm long, broad; connective discoid, glabrous, cream; staminodes absent; carpels fused into a single structure, ca. 2 mm long, stigma capitate, glabrous. Fruit syncarpous, sessile, 40–75 mm long, 20–35 mm in diameter, **ovoid, apex rounded, glabrous, smooth, bumpy**, green turning deep yellow when ripe; seeds not counted, 8–15 mm long, 5–10 mm in diameter, ellipsoid; aril absent.

#### Distribution.

A widespread species with a disjunct distribution in West (Sierra Leone, Liberia, Ivory Coast and Ghana) and Central Africa (Cameroon, Gabon); in Cameroon known from South, Central, Littoral and South-West regions.

#### Habitat.

An infrequent species in Cameroon; in lowland primary and secondary forests, also along rivers. Altitude 0–500 m a.s.l.

**Figure 33.**
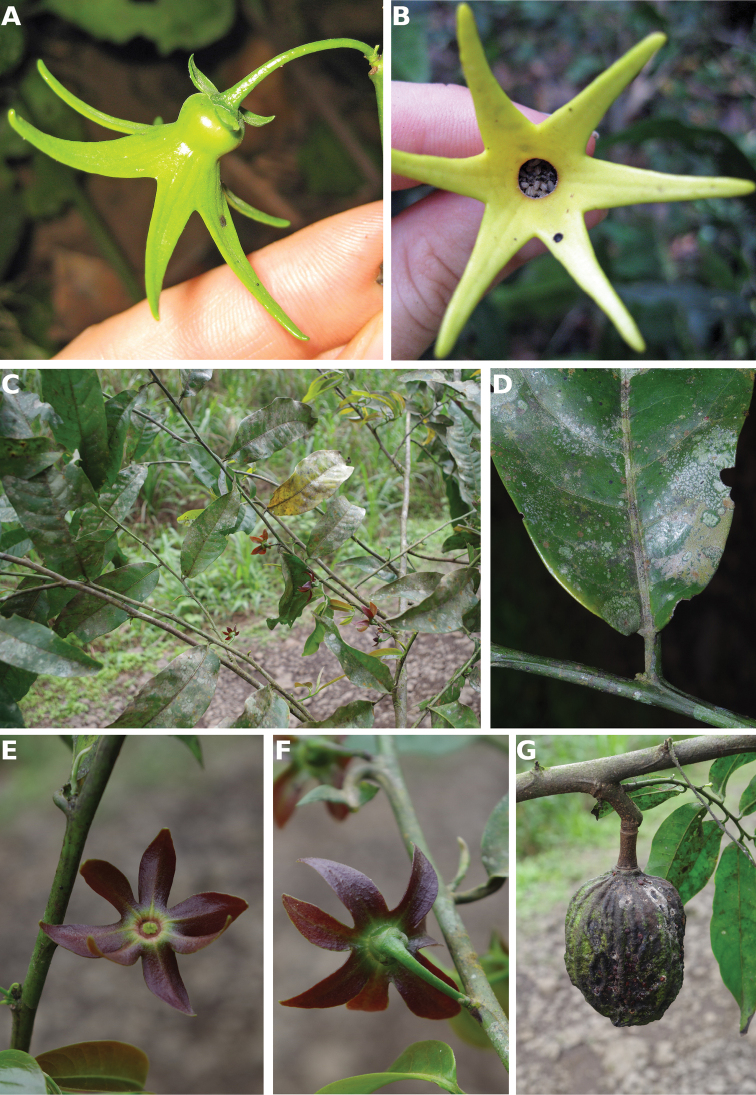
*Isolonacampanulata***A** flower, side view **B** flower, top view. *Isolonacongolana***C** flowering branch **D** base of leaf blade, top view **E** flower, top view **F** flower, bottom view **G** syncarpous fruit **A, B** no voucher, Bayang Mbo, Cameroon **C–G***Couvreur 1054*, Mt Cameroon. Photos **A, B** Sonneck **C–G** Thomas L.P. Couvreur .

#### Local and common names known in Cameroon.

None recorded.

#### IUCN conservation status.

Least Concern (LC) (Cosiaux et al. 2019p).

#### Uses in Cameroon.

***medicine***: root against rheumatism, allay fever.

#### Notes.

This species is characterized by its large, foliaceous, and glabrous sepals, not appressed against the corolla tube, as well as the sometimes leaf-like upper bract. It is also a completely glabrous species, a character only shared with ﻿*I.cooperi* and ﻿*I.hexaloba*. The flower of ﻿*I.campanulata* is very distinct and uniquely campanulate.

#### Specimens examined.

**Littoral Region**: Lombe amp Tissongo Study Area Doula-Edea Reserve, 3.78°N, 10.04°E, *01 June 1976*, *Waterman P.G.* 830 (K). **South Region**: ca 15 km SE of Kribi Kienke Forest Res at Bidou II, 2.86°N, 10.03°E, *30 June 1969*, *Bos J.J.* 4947 (BR,P,WAG,YA); 10 km From Kribi Lolodorf road, 2.96°N, 9.966°E, *09 July 1970*, *Bos J.J.* 7069 (WAG). **South-West Region**: 5.46°N, 9.883°E, *7 October 1899*, *Conrau G.* 93 (B,K); 16 minutes from research station towards rivers, 5.34°N, 9.496°E, *27 November 2000*, *Gosline W.G.* 289 (K); Ente Ekondo Nene et Loe 15 km NW Ekondo Titi, 4.69°N, 8.97°E, *03 June 1976*, *Letouzey R.* 15078 (P,YA); Korup National Park, 5.31°N, 8.966°E, *02 July 1951*, *Olorunfemi J.* 30662 (K); Korup National Park between the Ndian River at PAMOL field and 25 km on transect “ P ”, 5.01°N, 8.833°E, *12 April 1985*, *Thomas D.W.* 4763 (BR,K,MO,P,WAG,YA); Takamanda Forest Reserve, 6.21°N, 9.433°E, *30 April 1987*, *Thomas D.W.* 7354 (MO,P,WAG).

### ﻿﻿﻿﻿ Isolona congolana

Taxon classificationPlantaeMagnolialesAnnonaceae

(De Wild. & T. Durand) Engl. & Diels, Monogr. Afrik. Pflanzen.-Fam. 6: 84, 1901

F54783D0-20D3-58F4-900B-9CED151F1662

**Figure 34.**
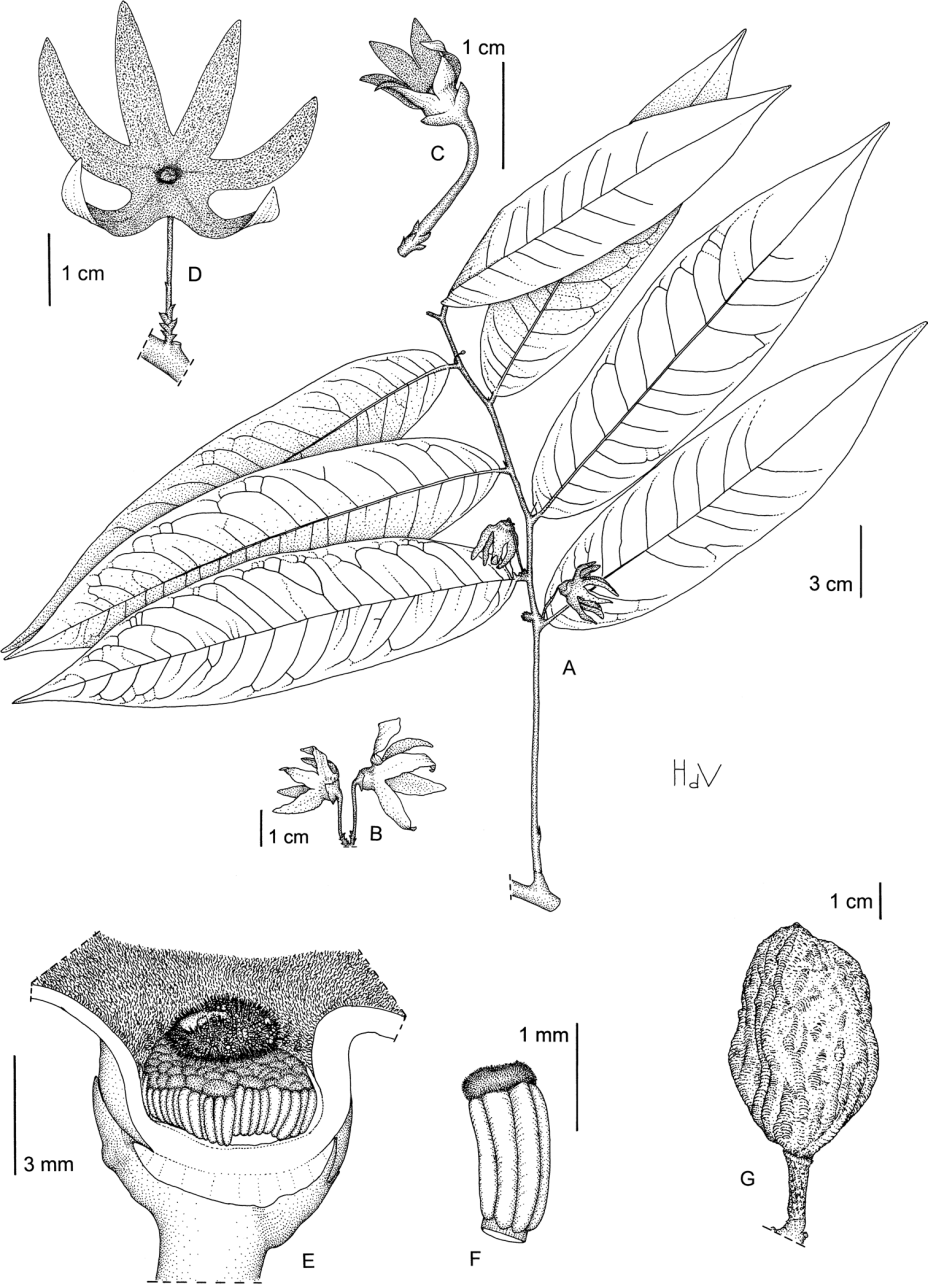
*Isolonacongolana***A** flowering branch **B** Flowers **C** young flower bud **D** flower **E** transversal section of flower showing receptacle, stigma and stamens **F** stamen **G** fruit **A, C–F***Leeuwenberg 9550***B***Wesphal 10012***G***Richardson 234*. Drawings by Hans de Vries (Couvreur 2009, fig. 19, p. 39).

**Map 5.**
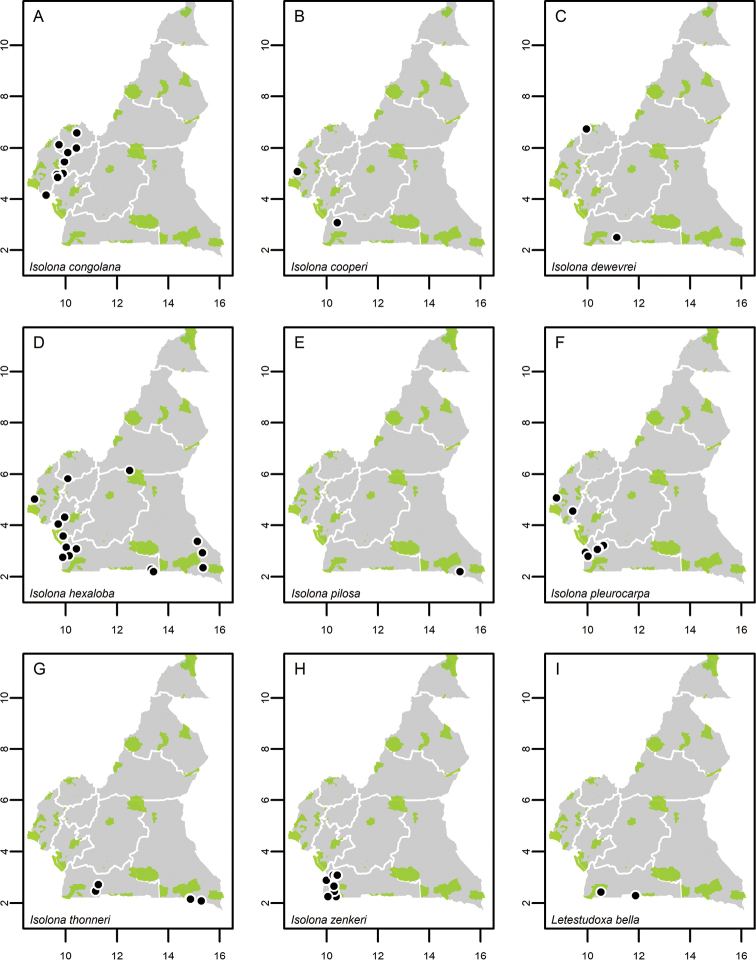
**A***Isolonacongolana***B***Isolonacooperi***C***Isolonadewevrei***D***Isolonahexaloba***E***Isolonapilosa***F***Isolonapleurocarpa***G***Isolonathonneri***H***Isolonazenkeri***I***Letestudoxabella*. White borders represent region limits in Cameroon; green patches represent protected areas (see methods and Suppl. material 1: Fig. S1).

Figs 33 , 34 ; Map 5A

≡ Monodora congolana De Wild. & T. Durand, Bull. Soc. Roy. Bot. Belgique 38: 13, 1899.
= Isolona maitlandii Keay, Kew Bull. 7(2): 155, 1952. Type. Cameroon. North-West Province, Ngong, Maitland T.D. 1555, Jun 1931: holotype: K[K000105576]; isotypes: BM, FHO.

#### Type.

Democratic Republic of the Congo. Equateur; Lukandu, *Dewèvre A.P. 1103*, 19 Nov 1896: lectotype, sheet here designated: BR[BR0000006248932]; isotypes: BR[BR0000006248857, BR0000006249588, BR0000006249250].

#### Description.

Tree, 10–30 m tall, d.b.h. 5–45 cm; stilt roots or buttresses absent. Indumentum of simple hairs; old leafless branches glabrous, young foliate branches densely pubescent. Leaves: petiole 4–7 mm long, 2–3 mm in diameter, densely pubescent, grooved, blade inserted on top of the petiole; blade 13–19 cm long, 4–5 cm wide, **narrowly ovate to narrowly elliptic or oblong**, apex acuminate, acumen 0.5–1 cm long, base cuneate to rounded, subcoriaceous, below sparsely pubescent when young, glabrous when old, above sparsely pubescent when young, glabrous when old, concolorous; midrib raised, above sparsely pubescent when young, glabrous when old, below sparsely pubescent when young, glabrous when old; secondary veins 11 to 14 pairs, sparsely pubescent to glabrous below; tertiary venation reticulate. Individuals bisexual; inflorescences ramiflorous on old leafless or young foliate branches, axillary. Flowers with 9 perianth parts in 2 whorls, 1 to 3 per inflorescence; pedicel 10–23 mm long, 1 mm in diameter, sparsely pubescent to glabrous; in fruit 2–5 mm long, 2–3 mm in diameter, glabrous; bracts 3 to 4, several basal and one upper towards the lower half of pedicel, basal bracts 1 mm long, 1 mm wide; upper bracts 1 mm long, 1 mm wide; sepals 3, valvate, free, 3 mm long, 2 mm wide, ovate, apex acute, base truncate, green, pubescent outside, glabrous inside, margins flat; petals basally fused, tube 4–10 mm long, inner and outer whorl not differentiated, equal; lobes 9–20 mm long, 3–7 mm wide, elliptic to ovate, apex rounded, green turning red, margins wavy, **glabrous outside, densely pubescent inside**, spreading horizontally; stamens numerous, in 3 to 4 rows, 2 mm long, broad; connective discoid, densely pubescent, cream; staminodes absent; carpels fused into a single structure, 2 mm long, stigma bilobed, slightly capitate, densely pubescent. Fruit syncarpous, sessile, 60–80 mm long, 40–50 mm in diameter, ellipsoid, apex rounded, glabrous, smooth to verrucose, irregularly ribbed, green when ripe; seeds not counted, 15–25 mm long, 10–15 mm in diameter, ellipsoid; aril absent.

#### Distribution.

A central African species, with a disjunct distribution between the Cameroon Volcanic Line in Cameroon, and Eastern Democratic Republic of Congo, one collection from Central African Republic; in Cameroon known from the Littoral, North-West and South-West regions.

#### Habitat.

A common species when present, mainly in montane or premontane rain forests, along rivers and bush land. Altitude 800–1700 m a.s.l.

#### Local and common names known in Cameroon.

Ndin (*Westphal 10012*).

#### IUCN conservation status.

Least Concern (LC) (Cosiaux et al. 2019q).

#### Uses in Cameroon.

***food***: fruit for condiments, spices, flavourings (*Westphal 10012*).

#### Notes.

This the only species of ﻿*Isolona* growing above 1000 m in Cameroon. ﻿﻿﻿*Isolonacongolana* is characterized by the densely pubescent inner part of the corolla tube and a glabrous outer part of the tube, a unique feature in this genus (﻿*I.pilosa* is pubescent on both sides of the corolla). The leaves are also characteristic being narrowly ovate to narrowly elliptic or oblong, giving a unique aspect to the foliage.

#### Specimens examined.

**Littoral Region**: Manengouba Mt 4 km WNW Of Nkongsamba, 4.98°N, 9.900°E, *04 April 1972*, *Leeuwenberg A.J.M.* 9550 (B,BR,C,GC,H,K,LD,M,MO,P,WAG,YA). **North-West Region**: Kagwene, 6.12°N, 9.734°E, *20 May 2009*, *Ashworth J.* 196 (K,YA); Bamenda Distr Ngong, 6.58°N, 10.43°E, *01 June 1931*, *Maitland T.D.* 1555 (BM,K); Bamenda Wae, 6°N, 10.41°E, *01 April 1931*, *Maitland T.D.* 1596 (K). **South-West Region**: Nyasoso, 4.86°N, 9.7°E, *04 June 1996*, *Cable S.* 2843 (K,MO,WAG,YA); 2 km north of Nyasoso towards Mpako, 4.84°N, 9.679°E, *04 April 2016*, *Couvreur T.L.P.* 1054 (WAG,YA); Nyasoso, 4.83°N, 9.683°E, *19 March 1996*, *Etuge M.* 1794 (K,WAG); White trail (above Kupe village) towards Madam 4.78°N, 9.716°E, *28 May 1996*, *Etuge M.* 2000 (BR,K,MO,P,WAG,YA); Lake Edip, 4.95°N, 9.65°E, *21 November 1998*, *Etuge M.* 4488 (K,WAG,YA); Etube Tape from Nyasoso, 4.83°N, 9.683°E, *06 February 1995*, *Lane P.* 532 (K,YA); Bu 4.15°N, 9.233°E, *6 June 1898*, *Lehmbach H.* 224 (B) . **West Region**: Bali Ngemba Forest Reserve, 5.82°N, 10.08°E, *13 April 2004*, *Etuge M.* 5431 (K,WAG,YA); Along the road 6 km W of Dschang on road to Fongo Ndeng, 5.45°N, 9.95°E, *15 May 1978*, *Westphal E.* 10012 (P,WAG).

### ﻿﻿﻿﻿ Isolona cooperi

Taxon classificationPlantaeMagnolialesAnnonaceae

Hutch. & Dalziel ex G.P.Cooper & Record, Bull. Yale Univ. School For. No. 31: 15, 1931

5F3DFE65-71A4-5266-873F-6E969E0BD976

Fig. 35 ; Map 5B

#### Type.

Liberia. Montserrado; near Firestone plantations, along Dukwai road, *Cooper G.P. 417*, 7 May 1929: lectotype, designated by Couvreur (2009), p. 148: GH[GH00286760]; isotypes: F[F0093217]; FHO[FHA00095994]; G[GH00286760]; K *n.v.*; NY[NY00026103]; WIS[WIS00000299MAD].

#### Description.

Tree, 6–18 m tall, d.b.h. 20 cm; stilt roots or buttresses absent. **Indumentum absent**; old leafless branches glabrous, young foliate branches glabrous. Leaves: petiole 1–5 mm long, 2–3 mm in diameter, glabrous, grooved, **blade inserted on top of the petiole**; blade 15–29 cm long, 6–15 cm wide, oblong to obovate, apex acuminate, acumen 1–2 cm long, base cuneate to rounded, subcoriaceous, below glabrous when young and old, above glabrous when young and old, concolorous; midrib raised, above glabrous when young and old, below glabrous when young and old; secondary veins 9 to 18 pairs, glabrous below; tertiary venation reticulate. Individuals bisexual; inflorescences cauliflorous or ramiflorous on young foliate branches, axillary. Flowers with 9 perianth parts in 2 whorls, 1 to 2 per inflorescence; pedicel 14–25 mm long, 1 mm in diameter, glabrous; in fruit 16–27 mm long, 2 mm in diameter, glabrous; bracts 2 to 4, all basal, 1 mm long, 1mm wide; sepals 3, valvate, free, 2 mm long, 2 mm wide, ovate, apex rounded, base truncate, green, glabrous outside, glabrous inside, **margins flat**; petals basally fused, tube 6–11 mm long, inner and outer whorl not differentiated, equal; lobes 8–15 mm long, 4–6 mm wide, oblong, apex acute to rounded, green, margins flat, glabrous outside, glabrous inside, spreading horizontally; stamens numerous, in 3 to 4 rows, 2 mm long, broad; connective discoid, glabrous, green; staminodes absent; carpels fused into a single structure, 3 mm long, stigma capitate, glabrous. Fruit syncarpous, sessile, 30–90 mm long, **15–30 mm in diameter, ellipsoid, apex apiculate, glabrous**, **smooth, constricted over seeds in dried material, smooth when fresh**, orange with white spots when ripe; seeds not counted, 10–15 mm long, 5–10 mm in diameter, ellipsoid; aril absent.

#### Distribution.

A mainly West African species, from Liberia to Ghana, with a few specimens from Cameroon and one from Gabon; in Cameroon known from South and South-West regions.

#### Habitat.

A rare species in Cameroon; in lowland primary and secondary forests, also along rivers, on sandy soils. Altitude 0–300 m a.s.l.

#### Local and common names known in Cameroon.

None recorded.

#### IUCN conservation status.

Least Concern (LC) (Cosiaux et al. 2019r).

#### Uses in Cameroon.

None reported.

#### Notes.

﻿﻿﻿*Isolonacooperi* is distinguished by its completely glabrous leaves, young foliate branches and flowers and with the leaf blade inserted on top of the petiole. In addition, the flowers emit a very strong sweet scent, noticeable even in dried material. It has a smooth corolla in dried material and corolla lobes with straight margins. ﻿﻿﻿*Isolonacooperi* is similar to ﻿*I.hexaloba* by the shape of its flowers, but the latter differs by its blade inserted sideways to the petiole and the absence of the strong sweet scent. Finally, ﻿*I.cooperi* resembles ﻿*I.campanulata* by the shape of the fruits.

**Figure 35.**
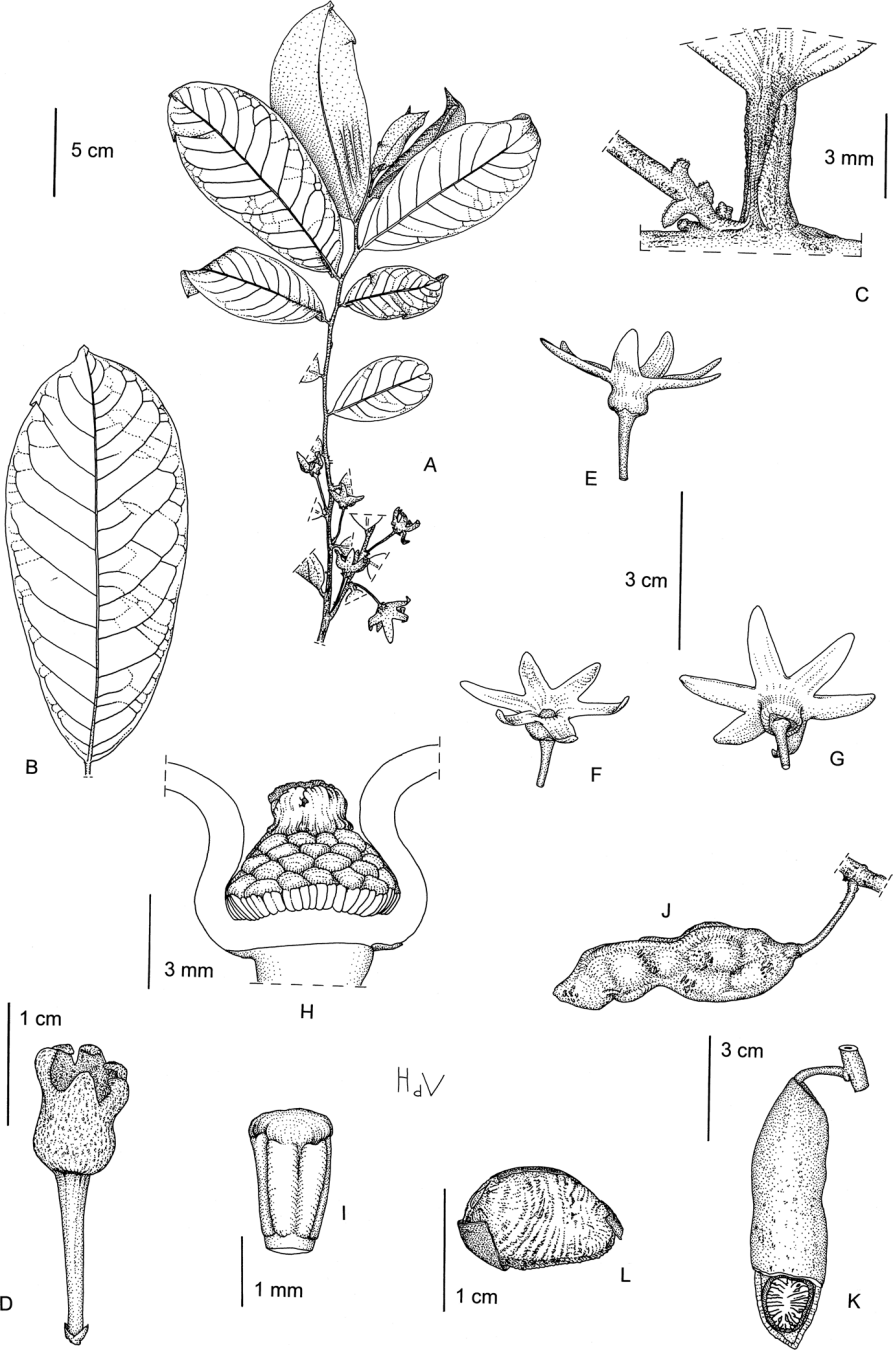
*Isolonacooperi***A** flowering branch **B** leaf, lower view **C** detail of petiole and axillary inflorescence **D** flower bud **E** flower, side view **F** flower, semi side view **G** flower, bottom view **H** transversal section showing androecium and stigma **I** stamen, front view **J** fruit (dried) **K** fruit, part of pericarp removed showing ruminate section of seed (fresh) **L** seed with seed coat partially removed showing ruminations **A–C** from *J.J.F.E. de Wilde 3644***D–I** from fresh material collected at the Utrecht University Botanical Garden **J, L** from de *Koning 149***K** from *Breteler 7458*. Drawings Hans by Vries (Couvreur 2009, fig. 21, p. 42).

#### Specimens examined.

**South Region**: Bipindi, 3.08°N, 10.41°E, *01 February 1910*, *Zenker G.A.* s.n. (F). **South-West Region**: Korup National Park, 5.06°N, 8.855°E, *10 March 1998*, *Kenfack D.* 1063 (MO,WAG).

### ﻿﻿﻿﻿ Isolona dewevrei

Taxon classificationPlantaeMagnolialesAnnonaceae

(De Wild. & T. Durand) Engl. & Diels, Monogr. Afrik. Pflanzen.-Fam. 6: 83, 1901

EDC6B6F7-3CB8-5F22-B2AB-21F7EEFA8A60

Fig. 36 ; Map 5C

≡ Monodora dewevrei De Wild. & T. Durand, Bull. Soc. Roy. Bot. Belg., Compt. Rend. 38: 11, 1899.

#### Type.

Democratic Republic of the Congo. Bas Congo; Lemba-Luki, *Dewèvre A.P. 365*, no date: lectotype, sheet here designated: BR[BR0000008801050]; isotypes: BR[BR0000008799968, BR0000008800725, BR0000008799630].

#### Description.

Tree to shrub, 8–15 m tall, d.b.h. 20 cm; stilt roots or buttresses absent. **Indumentum of simple hairs**; old leafless branches glabrous, young foliate branches glabrous. Leaves: petiole 4–15 mm long, 1–2 mm in diameter, glabrous, grooved, **blade inserted on the side of the petiole**; blade 10–17 cm long, 4–7 cm wide, elliptic to obovate, apex acuminate, acumen ca. 1 cm long, base decurrent to cuneate, papyraceous, below glabrous when young and old, above glabrous when young and old, concolorous; midrib raised, above glabrous when young and old, below glabrous when young and old; secondary veins 9 to 14 pairs, glabrous below; tertiary venation reticulate. Individuals bisexual; inflorescences ramiflorous on old branches, axillary. Flowers with 9 perianth parts in 2 whorls, 1 per inflorescence; pedicel 2–7 mm long, 1 mm in diameter, **sparsely pubescent**; in fruit 9–10 mm long, 2–3 mm in diameter, glabrous; bracts 3 to 5, several basal and one upper towards the lower half of pedicel, basal bracts 1 mm long, 0.5–1 mm wide; upper bract 1 mm long, 1 mm wide; sepals 3, valvate, free, 2–3 mm long, 3–4 mm wide, ovate, apex acuminate, base truncate, glabrous outside, glabrous inside, margins flat; petals basally fused, tube 3–4 mm long, inner and outer whorl not differentiated, equal; lobes 7–17 mm long, 5–7 mm wide, **elliptic, apex rounded, green, margins flat**, overall glabrous but pubescent towards margins outside and inside; **margins curved outwards**; stamens ca. 50, in 3 to 4 rows, 2 mm long, broad; connective discoid, glabrous; staminodes absent; carpels fused into a single structure, ca. 1 mm long, stigma capitate, glabrous. Fruit syncarpous, sessile, 60–70 mm long, 40–50 mm in diameter, **ovoid, apex rounded, glabrous, smooth, not ribbed, green when ripe**; seeds not counted, 10–20 mm long, 10–15 mm in diameter, ellipsoid; aril absent.

#### Distribution.

A widespread species, but with few overall specimens, from Liberia to Nigeria, and from Cameroon to Democratic Republic of Congo; in Cameroon in the South and South-West regions.

**Figure 36.**
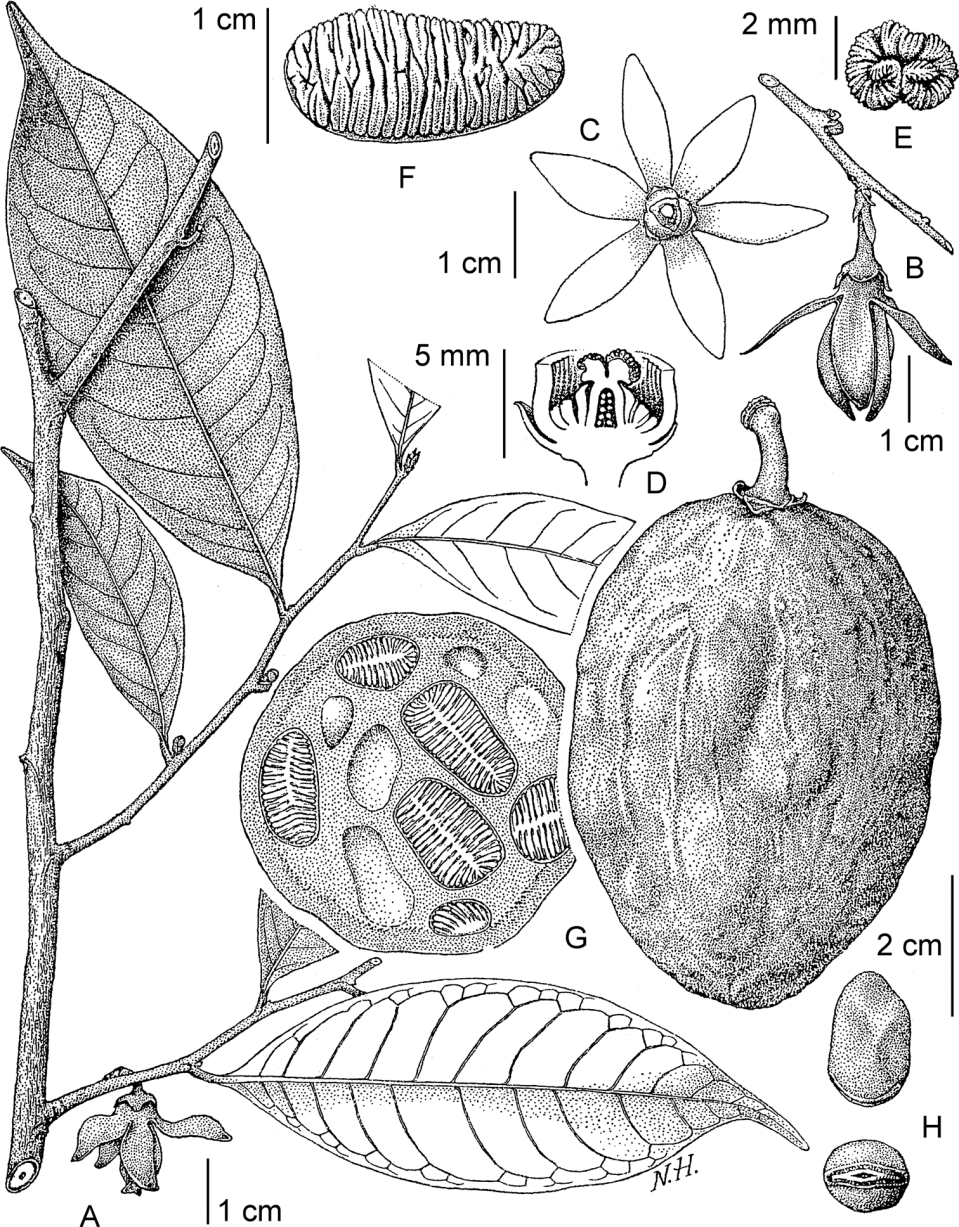
*Isolonadewevrei***A** flowering branch **B** flower (side view) **C** flower top view **D** transversal section of flower **E** detail of stig top view **F** transversal section of seed **G** fruit (left) and longitudinal section of fruit (right) **H** seed, side view (top), detail of hilum (bottom). Modified from Aké Assi (1963, fig. 1, p. 14).

#### Habitat.

A rare species in Cameroon known from two specimens; in lowland primary and secondary forests. Altitude 0–860 m a.s.l.

#### Local and common names known in Cameroon.

None recorded.

#### IUCN conservation status.

Least Concern (LC) (Cosiaux et al. 2019s).

#### Uses in Cameroon.

None reported.

#### Notes.

﻿﻿﻿*Isolonadewevrei* resembles ﻿*I.thonneri* in leaf and fruit shape, but the former can be distinguished by its elliptic and shorter corolla lobes with hairy margins and its flowering pedicel sparsely covered with short hairs. It is, however, very hard to distinguish both species apart based on fruit or vegetative characters alone.

#### Specimens examined.

**South Region**: Ngongondje hill near Akonetye 2 30'S of Ebolowa, 2.5°N, 11.13°E, *28 August 1979*, *Koufani A.* 123 (P,YA). **South-West Region**: Piste Munkep-Gayama 40 km NNW Wum, 6.73°N, 9.95°E, *08 July 1975*, *Letouzey R.* 13984 (K,MO,YA).

### ﻿﻿﻿﻿ Isolona hexaloba

Taxon classificationPlantaeMagnolialesAnnonaceae

(Pierre) Engler, Nat. Pflanzenfam. Nachtr. zu 3(2): 161, 1897

34C312EA-F222-5FAF-9E7D-80BE84B7CF4A

**Figure 37.**
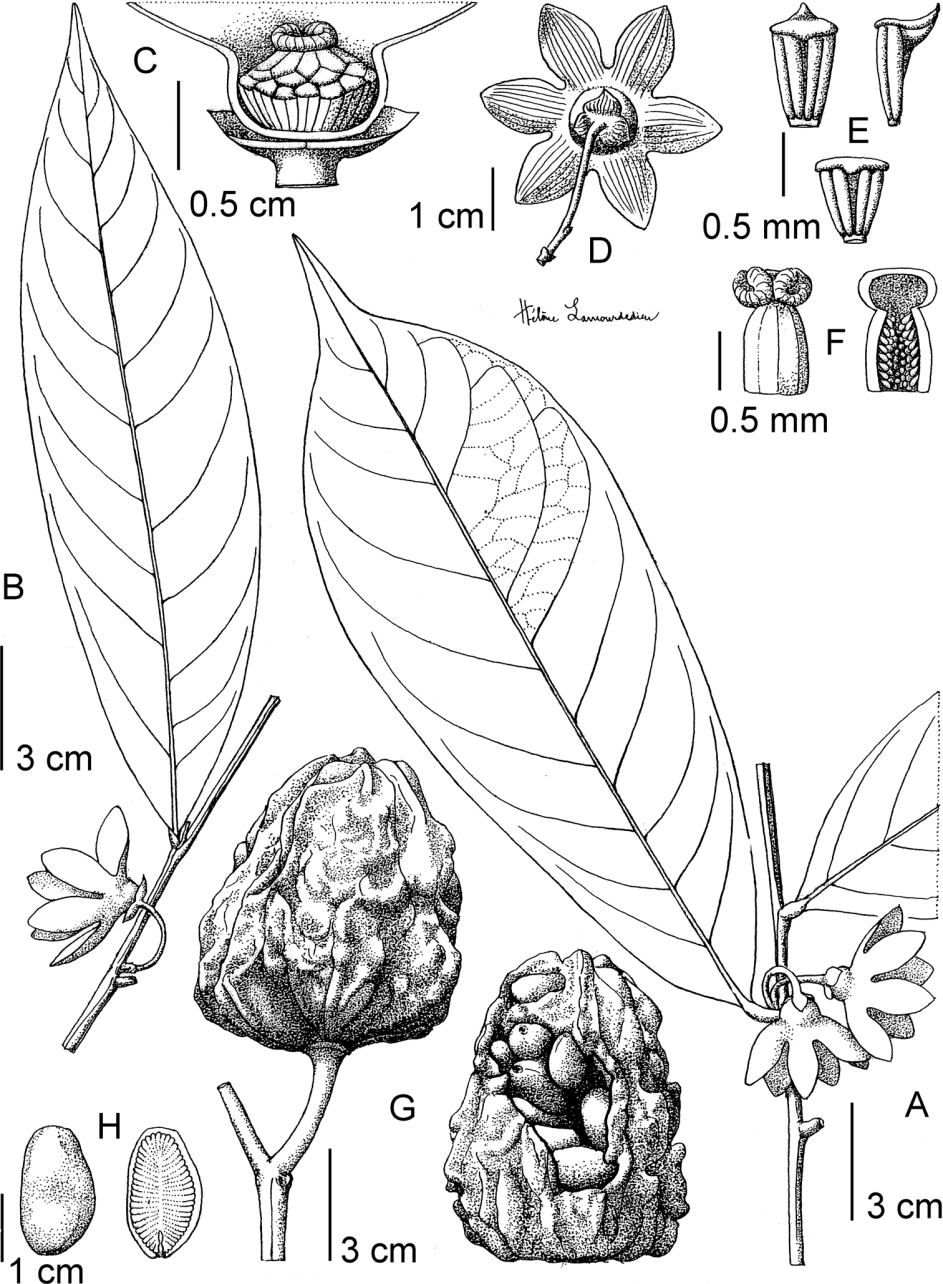
*Isolonahexaloba***A, B** flowering branch **C** transversal section of flower showing androecium and stigma **D** flower (bottom view) **E** stamens inner row (2 top); stamens of inner most row (top) and outermost row (bottom) **F** carpel and detail of ovules **G** fruits, the one on the right opened to show seeds **H** seed (left) and transversal section of seed showing ruminate endosperm (right) **A, C–F** from *Le Testu 5862***B***Le Testu 5836***G, H***Klaine 360*. Drawings by Hélène Lamourdedieu, Publications Scientifiques du Muséum national d’Histoire naturelle, Paris; modified from Le Thomas (1969b, pl. 66, p. 357).

**Figure 38.**
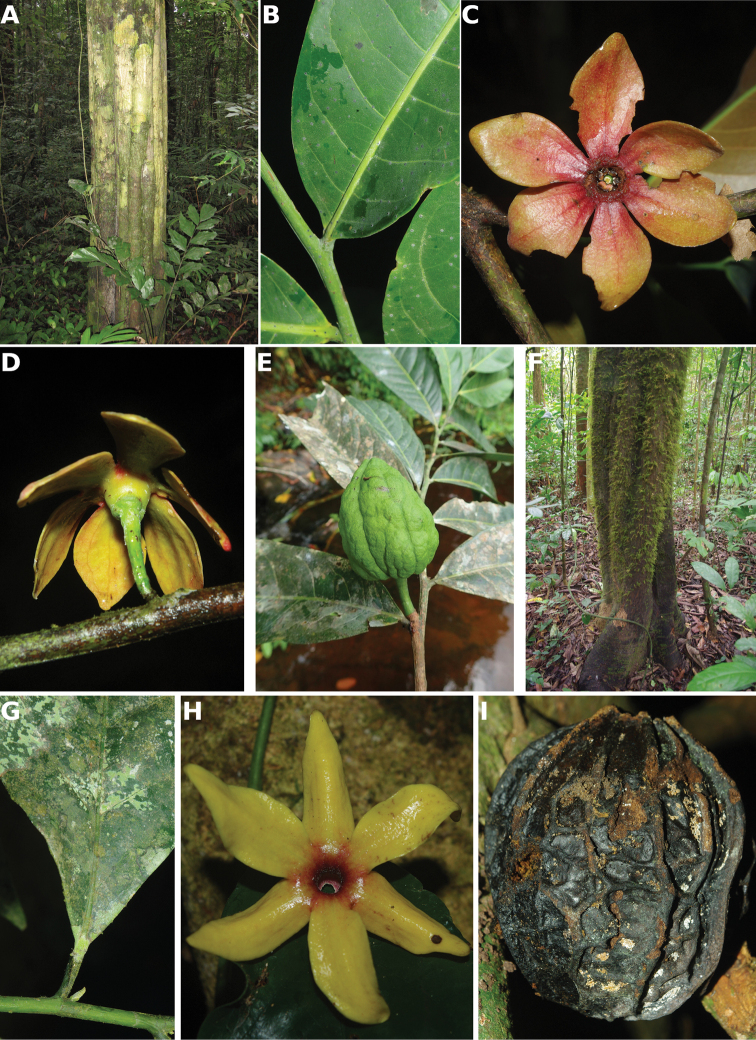
*Isolonahexaloba***A** trunk, note deeply furrowed **B** base of leaf blade, note raise midrib **C** flower, top view **D** flower, side view **E** fruiting branch. *Isolonapleurocarpa***F** trunk **G** bade of leaf blade, notice decurrent base **H** flower, top view, fallen on ground **I** syncarpous fruit; fallen on ground **A***Sosef 2032*, Gabon **B–D***Couvreur 561*, Gabon **E***Texier 2347*, Gabon **F–I***Couvreur 402*, Ngovayang, Cameroon. Photos **A–D, F–I** Thomas L.P. Couvreur **E** Nicolas Texier, Tropicos.org, Missouri Botanical Garden.

Figs 37 , 38 ; Map 5D

≡ ﻿﻿Monodora hexaloba Pierre, Fig. Herb. L. Pierre, del. E. Delpy 5/1896, 1896.
= Isolona bruneelii De Wild., Ann. Mus. Congo Belge, Bot. ser. 5, 3(1): 82, 1909. Type. Democratic Republic of the Congo. Orientale, Dikila, Bruneel A.C.E. s.n., Dec 1906: lectotype, sheet here designated: BR[BR0000008800152] ; isotypes: BR[BR0000008799692, BR0000008799364, BR0000008799302]; S[S10-20956].
= Isolona seretii De Wild., Ann. Mus. Congo Belge, Bot. ser. 5, 3[1]: 82, 1909. Type. Democratic Republic of the Congo. Equateur, near Nala, *Seret F. 792*, Mar 1907: lectotype, sheet here designated: BR[BR0000008800992]; isotype: BR[BR0000008801326].
= Isolona solheidii De Wild., Ann. Mus. Congo Belge, Bot. sér. 5, 3: 83, 1909. Type. Democratic Republic of the Congo. Equateur, surroundings of Yambuya, Solheid A.F. s.n., 1906: lectotype, sheet here designated: BR[BR0000008799395]; isotype: BR[BR0000008799722].
= Isolona seretii var. grandifolia De Wild., Ét. Fl. Bang. Ub.: 313, 1911. Type. Democratic Republic of the Congo. Orientale, Mobwasa, *Claessens J. 615*, Apr 1910: holotype: BR.
= Isolona pleurocarpa subsp. nigerica Keay, Kew Bull. 7: 157, 1952. Type. Nigeria. Ijebu State, Shasha Forest Reserve, *Richards P.W. 3343*, 8 Apr 1935: holotype: BM[BM000546386]; isotypes: BR[BR0000014130090]; G[G00011543, G00011544]; MO[MO-2246487]; S[S10-20983].

#### Type.

Gabon. Estuaire; Environs de Libreville, *Klaine T.-J. 360*, 17 Feb 1896: lectotype, sheet here designated: P[P00363270]; isotypes: B; K[K00198842]; P[P00363269, P00363268]; WAG[WAG0251603].

#### Description.

Tree to shrub, 15–30 m tall, d.b.h. up to 50 cm; stilt roots or buttresses absent, **trunk deeply fluted. Indumentum absent**; old leafless branches glabrous, young foliate branches glabrous. Leaves: petiole 2–4 mm long, 2–3 mm in diameter, glabrous, **grooved, blade inserted on the side of the petiole**; blade 10–28 cm long, 3–11 cm wide, ovate to elliptic, apex acuminate, acumen 1–2 cm long, base obtuse to acute, coriaceous, below glabrous when young and old, above glabrous when young and old, concolorous; midrib raised, above glabrous when young and old, below glabrous when young and old; secondary veins 8 to 16 pairs, glabrous below; tertiary venation reticulate. Individuals bisexual; inflorescences ramiflorous on old or young foliate branches, axillary. Flowers with 9 perianth parts in 2 whorls, 1 to 2 per inflorescence; pedicel 7–30 mm long, ca. 1 mm in diameter, glabrous; in fruit 20–29 mm long, 3–5 mm in diameter, glabrous; bracts 3 to 5, several basal and one upper towards the lower half of pedicel, basal bracts ca. 1 mm long, 0.5 mm wide; upper bract 2–5 mm long, ca. 1 mm wide; sepals 3, valvate, free, 1–3 mm long, 2–4 mm wide, elliptic, apex acuminate, base truncate, green, glabrous outside, glabrous inside, margins flat; petals basally fused, tube 4–10 mm long, inner and outer whorl not differentiated, equal; lobes 6–25 mm long, 4–12 mm wide, elliptic to ovate, apex acute to rounded, dark red, margins flat to wavy, glabrous outside, glabrous inside, spreading horizontally; stamens numerous, in 3 to 4 rows, 2 mm long, broad; connective discoid, glabrous, green; staminodes absent; carpels fused into a single structure, ca. 1 mm long, stigma bilobed, slightly capitate, glabrous. Fruit syncarpous, sessile, 30–70 mm long, 25–40 mm in diameter, **ovoid, apex rounded, glabrous, smooth, bumpy**, **irregularly and transversely ribbed**, light green to dark purple when ripe; seeds not counted, 8–15 mm long, 4–6 mm in diameter, ellipsoid; aril absent.

#### Distribution.

A widespread species from Nigeria to the Democratic Republic of Congo and northern Angola (one specimen); in Cameroon known from Adamaoua, East, South, Central, Littoral and South-West, North-West regions.

#### Habitat.

A relatively rare species in Cameroon; in lowland primary and secondary evergreen forests, but also in semi-deciduous forests, also along rivers. Altitude 0–700 m a.s.l.

#### Local and common names known in Cameroon.

None recorded.

#### IUCN conservation status.

Least Concern (LC) (Cosiaux et al. 2019t).

#### Uses in Cameroon.

None reported.

#### Notes.

﻿﻿﻿*Isolonahexaloba* is distinguished by the short and grooved petiole with the leaf blade inserted on its sides. The corolla lobes are coriaceous and can be quite variable in shape, ranging from elliptic to obovate, with a narrowed base. It is a polymorphic species and has been described under several names, now all reduced to synonymy (Couvreur 2009). ﻿﻿﻿*Isolonahexaloba* resembles ﻿*I.cooperi* by the shape of the corolla lobes, but is distinguished by the insertion of the leaf blade on the side a short petiole and lacks the strong sweet smell. ﻿﻿﻿*Isolonahexaloba* is also similar to ﻿*I.pleurocarpa*, but the later can be distinguished by its decurrent to narrowly cuneate leaf bases, longer petioles, and papyraceous corolla lobes.

#### Specimens examined.

**Central Region**: Tibati près Mbatimbang, 6.14°N, 12.48°E, *04 December 1959*, *Letouzey R.* 2392 (P,YA). **East Region**: A 25 km environ à l’ENE de Mikel village situé à 85 km au N de Moloundou sur la route de Yokadouma 2.93°N, 15.33°E, *24 February 1971*, *Letouzey R.* 10419 (P,WAG,YA); A 14 km à l’Ouest de Yenga Port Gentil village situé à 35 km au NNE de Moloundou, 2.35°N, 15.35°E, *21 April 1971*, *Letouzey R.* 10703 (BR,COI,K,P,WAG,YA); A 20 km au Sud de Mboy I (45 km à l’Est de Yokadouma), 3.38°N, 15.13°E, *16 May 1963*, *Letouzey R.* 5072 (K,P,WAG,YA). **Littoral Region**: Douala (route Razel), 4.05°N, 9.71°E, *01 January 1955*, *Endengle E. SRFK* 2121 (P,YA); Roue forestière SNCB (km 36 vers Ndoksom) environ 25 km Sud Yabassi, 4.31°N, 9.958°E, *11 May 1976*, *Letouzey R.* 14910 (C,K,MO,P,WAG,YA); Tissongo, 3.58°N, 9.9°E, *01 August 1976*, *McKey D.B.* 245 (K). **South Region**: ca 15 km east from Lélé village, 2.28°N, 13.32°E, *10 September 2013*, *Couvreur T.L.P.* 495 (WAG,YA); near Bipaga II km 40 road Kribi-Edéa, 3.15°N, 10.01°E, *30 December 1982*, *de Kruif A.P.M.* 998 (MO,WAG,YA); 17 km S of the Lobe river along the road to Campo, 2.81°N, 10.13°E, *18 March 1975*, *de Wilde J.J.F.E* 8088 (BR,K,MO,P,U,WAG,YA); A l’Ouest d’Alati (100 km SE de Djoum), 2.2°N, 13.42°E, *13 January 1973*, *Letouzey R.* 11840 (K,P,YA); 22 km on road Kribi to Campo 12 km past Gross Batanga, 2.76°N, 9.881°E, *24 February 1994*, *Wieringa J.J.* 2327 (MPU,U,WAG); Bipindi, 3.08°N, 10.41°E, *01 November 1901*, *Zenker G.A.* s.n. (P). **South-West Region**: Korup National Park, 5.01°N, 8.783°E, *31 October 2005*, *van der Burgt X.M.* 791 (BR,G,K,MO,P,WAG,YA). **West Region**: Bali- Ngemba FR, 5.81°N, 10.08°E, *13 April 2002*, *Onana J.M.* 2030 (K,WAG).

### ﻿﻿﻿﻿ Isolona pilosa

Taxon classificationPlantaeMagnolialesAnnonaceae

Diels, Bot. Jahrb. Syst. 41: 328, 1908

A2929938-B889-5C06-A353-EE85112D7E8E

Fig. 39 ; Map 5E

= Isolona theobromina Exell, J. Bot. 64 (Suppl. 1): 10, 1926. Type. Angola. Cabinda, Pango Munga, *Gossweiler J. 6112*, 7 Jan 1916: holotype: BM[BM000889332]; isotypes: COI[COI00077211]; LISJC *n.v.*; LISC[LISC000094, LISC000095, LISC000096].

#### Type.

Democratic Republic of the Congo. Kasai Oriental; Lualaba, *Ledermann C. 11*, Mar 1906: holotype: B[B 10 0154216]; isotype: K *n.v*.

#### Description.

Tree, 13 m tall, d.b.h. up to 50 cm; stilt roots or buttresses absent, trunk not fluted. Indumentum of simple hairs; **old leafless branches glabrous, young foliate branches densely pubescent.** Leaves: petiole 3–12 mm long, 3–4 mm in diameter, **densely pubescent, grooved, blade inserted on the side of the petiole**; blade 19–27 cm long, 6–10 cm wide, obovate, apex acuminate, acumen 1–2 cm long, base rounded to cordate, papyraceous, below densely pubescent when young, densely pubescent when old, above sparsely pubescent to glabrous when young, sparsely pubescent to glabrous when old, concolorous; midrib raised above, above sparsely pubescent when young, sparsely pubescent when old, **below densely pubescent when young, densely pubescent when old**; secondary veins 15 to 20 pairs, glabrous below; tertiary venation reticulate. Individuals bisexual; inflorescences ramiflorous on old branches, axillary. Flowers with 9 perianth parts in 2 whorls, 1 to 2 per inflorescence; pedicel 2–4 mm long, 1–2 mm in diameter, **densely pubescent**; in fruit 2–8 mm long, 2–3 mm in diameter, pubescent; bracts 2 to 4, all basal, 2–4 mm long, 1–2 mm wide; sepals 3, valvate, free, 2–5 mm long, 2–4 mm wide, ovate, apex acuminate, base truncate, **densely pubescent outside**, glabrous inside, margins flat; petals basally fused, tube 5–10 mm long, inner and outer whorl not differentiated, equal; lobes 8–13 mm long, 3–5 mm wide, elliptic, apex acute, yellow, margins flat, **densely pubescent outside, pubescent with base glabrous inside**, curving inwards over the receptacle; stamens numerous, in 3 to 4 rows, 2 mm long, broad; connective discoid, glabrous; staminodes absent; carpels fused into a single structure, 2 mm long, stigma bilobed, glabrous. Fruit syncarpous, sessile, 30–60 mm long, 20–40 mm in diameter, ellipsoid, apex cuspidate, sparsely pubescent, **longitudinally ribbed**, color unknown; seeds not counted, 13–15 mm long, 6–8 mm in diameter, flattened ellipsoid; aril absent.

**Figure 39.**
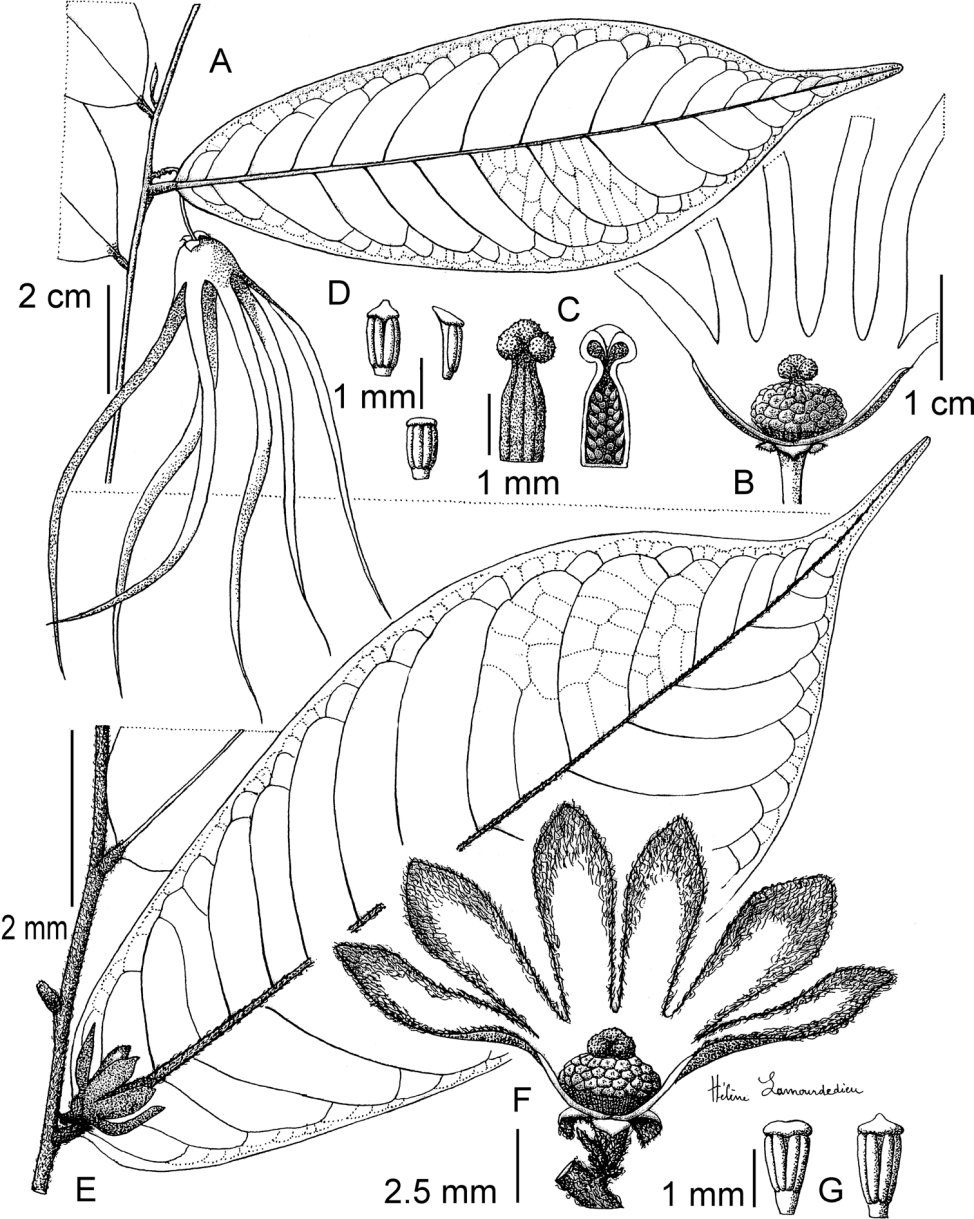
*Isolonaletestui* (not in Cameroon) **A** flowering branch **B** opened flower showing androecium and stigma **C** carpel (left) and transversal section of carpel (right) **D** stamens of innermost row (2 top) and stamen of outermost row (bottom). *Isolonapilosa***E** flowering branch **F** opened flower showing androecium and stigma **G** stamen of outermost row (left) and innermost row (right) **A–D** from *Le Testu 1252*; E from *Le Testu 8740***F, G** from *Le Testu 8602*. Drawings by Hélène Lamourdedieu, Publications Scientifiques du Muséum national d’Histoire naturelle, Paris; modified from Le Thomas (1969b, pl. 65, p. 351).

#### Distribution.

A central African species with a disjunct distribution, from Cameroon to Gabon and the Republic of Congo, also present in the Democratic Republic of Congo; in Cameroon known from the extreme East region.

#### Habitat.

A rare species in Cameroon; in lowland rain forests or swampy areas. Altitude 100–450 m a.s.l.

#### Local and common names known in Cameroon.

None recorded.

#### IUCN conservation status.

Vulnerable B2ab(iii) (Cosiaux et al. 2019u).

#### Uses in Cameroon.

None reported.

#### Notes.

﻿﻿﻿*Isolonapilosa* is the most pubescent species of the genus. It is distinguishable by its densely pubescent leaf midrib on the upper side (even in older leaves), as well as its short and densely hairy flowering pedicels. The corolla lobes are densely hairy on the outside and near the margins on the inside; the inner part of the tube is glabrous, which distinguishes it from *I.congolona* which is pubescent on the inner surface of the tube but glabrous on the outside.

Several specimens collected by Harris DJ are reported from The Lobéké National Park in East Cameroon, but we were not able to see them and verify their identification (e.g. *6408, 6538, 6627*).

#### Specimens examined.

**East Region**: Region near Station Molundu Dscha (Ngoko) Nginda 21 km north Molundu, 2.2°N, 15.2°E, *07 January 1911*, *Mildbraed G.W.J.* 4193 (HBG).

### ﻿﻿﻿﻿ Isolona pleurocarpa

Taxon classificationPlantaeMagnolialesAnnonaceae

Diels, Bot. Jahrb. Syst. 39: 485, 1907

64A31019-68E6-598B-8D76-A2BDD4F5B0D0

Fig. 38 ; Map 5F

= Isolona leucantha Diels, Bot. Jahrb. Syst. 39: 484, 1907. Type. Cameroon. South Region, Bipindi, Zenker G.A. 3038, Apr 1904: holotype: B[B 10 0154212]; isotypes: BM[BM000546385]; BR[BR0000008802644]; COI[COI00077204]; E[E00259306]; G[G00011566]; GOET[GOET005681]; HBG[HBG518922]; K[K000199011]; L[L-0182762]; M[M0089224]; MO[M0089224]; P[P00363267]; S[S-G-7462]; WAG[WAG0000090]; WU[WU0025864].

#### Type.

Cameroon. South Region; Bipindi, *Zenker G.A. 3217*, Jul 1904: holotype: B[B 10 0154211]; isotypes BR[BR0000008498991]; G[G00011576 G00011761]; K[K000198837] ; S[S10-21236]; WU[WU0025885].

#### Description.

Tree, 15–30 m tall, d.b.h. up to 60 cm; stilt roots or buttresses absent, trunk fluted. Indumentum of simple hairs; old leafless branches glabrous, **young foliate branches glabrous.** Leaves: petiole 4–12 mm long, 1 mm in diameter, glabrous, **grooved towards the base, blade inserted on the side of the petiole**; blade 8.5–15.5 cm long, 3–6 cm wide, elliptic to obovate, apex acuminate, acumen 1–1.5 cm long, base decurrent to cuneate, papyraceous to subcoriaceous, below glabrous when young and old, above glabrous when young and old, concolorous; midrib **raised above**, above glabrous when young and old, below glabrous when young and old; secondary veins 9 to 12 pairs, glabrous below; tertiary venation reticulate. Individuals bisexual; inflorescences ramiflorous on old or young foliate branches, axillary. Flowers with 9 perianth parts in 2 whorls, 1 per inflorescence; pedicel 1–2 mm long, 1 mm in diameter, glabrous; in fruit 30 mm long, 3–4 mm in diameter, glabrous; bracts 3 to 7, several basal and one upper towards the lower half of pedicel, basal bracts ca. 1 mm long, ca. 1 mm wide; upper bracts ca. 1 mm long, ca. 1 mm wide; sepals 3, valvate, free, 2–3 mm long, 2–3 mm wide, ovate, apex acuminate, base truncate, green, **glabrous outside, glabrous inside**, margins flat; petals basally fused, tube 6–15 mm long, red, inner and outer whorl not differentiated, equal; lobes 10–23 mm long, 5–10 mm wide, ovate, apex acute, bright green-white to yellow, **margins wavy, glabrous outside, glabrous inside**, spreading horizontally; stamens numerous, in 3 to 4 rows, ca. 2 mm long, broad; connective discoid, glabrous, yellow; staminodes absent; carpels fused into a single structure, ca. 2 mm long, stigma bilobed, slightly capitate, glabrous. Fruit syncarpous, sessile, ca. 50 mm long, ca. 40 mm in diameter, globose, **apex rounded, glabrous, rugulose, longitudinally 6–8 ribbed, green when unripe**; seeds not counted, 8–10 mm long, 5–7 mm in diameter, ellipsoid; aril absent.

#### Distribution.

Known from Nigeria and Cameroon; in Cameroon known from the South and South-West regions.

#### Habitat.

An infrequent species; in lowland rain forests on non-inundated soils. Altitude 0–550 m a.s.l.

#### Local and common names known in Cameroon.

Avom (*van Andel 4177*).

#### IUCN conservation status.

Endangered (EN) (Cosiaux et al. 2019v).

#### Uses in Cameroon.

None reported.

#### Notes.

﻿﻿﻿*Isolonapleurocarpa* is distinguished by the combination of these characters: young leaves glabrous, leaf blade inserted on the side of the petiole and decurrent to narrowly cuneate at base, midrib proximally depressed above, corolla lobes narrowly ovate to ovate with a narrowed base and an acute apex, undulate-wavy on the margins when dried.

#### Specimens examined.

**South Region**: NE of Mt Elephant ca 20 km SE of Kribi, 2.8°N, 10.03°E, *10 February 1970*, *Bos J.J.* 6298 (WAG); mountain chain Ngovoyang 42 km in forest from Bikiliki village situated between Bipindi and Lolodorf, 3.18°N, 10.53°E, *19 February 2012*, *Couvreur T.L.P.* 402 (WAG,YA); Colline Nkolo Manga (20 km SE Kribi), 2.95°N, 9.916°E, *16 April 1968*, *Letouzey R.* 9341 (P,WAG); Elephant Mont, 2.8°N, 10.01°E, *22 October 2001*, *van Andel T.R.* 4177 (KRIBI,WAG,YA); Bipindi, 3.08°N, 10.41°E, *1895*, *Zenker G.A.* 1716 (B,G,M,P,WAG); Bipindi, 3.08°N, 10.41°E, *01 January 1918*, *Zenker G.A.* 22 (P,WAG); Mbiave, 3.21°N, 10.61°E, *01 January 1913*, *Zenker G.A.* 267 (A,B,BR,C,G,M,MO,U,WAG); Bipindi, 3.08°N, 10.41°E, *01 April 1904*, *Zenker G.A.* 3038 (B,COI,G,K,L,M,MO,P,S,WAG); Bipindi, 3.08°N, 10.41°E, *01 July 1904*, *Zenker G.A.* 3217 (B,BR,G,K,L,M,S,WAG); Bipindi, 3.08°N, 10.41°E, *01 January 1908*, *Zenker G.A.* 3433 (BR,COI,G,G,L,M,M,MO,P,S); Bipindi, 3.08°N, 10.41°E, *01 January 1908*, *Zenker G.A.* 3540 (G,K,L,M,M,MO); Bipindi, 3.08°N, 10.41°E, *01 January 1909*, *Zenker G.A.* 3921 (B,BR,COI,COI,G,L,M,M,MO,P,S); Bipindi, 3.08°N, 10.41°E, *01 January 1913*, *Zenker G.A.* 4704 (BM,BR,G,K,L,M,P,S); Bipindi, 3.06°N, 10.38°E, *01 November 1919*, *Zenker G.A.* 95 (BM). **South-West Region**: Southern Bakundu Forest 3 km from Kindongi Camp, 4.55°N, 9.416°E, *02 May 1972*, *Leeuwenberg A.J.M.* 9784 (B,BR,C,H,K,LD,M,MO,P,WAG,YA); Korup National Park, 5.06°N, 8.783°E, *13 April 1978*, *Thomas D.W.* 349 (K).

### ﻿﻿﻿﻿ Isolona thonneri

Taxon classificationPlantaeMagnolialesAnnonaceae

(De Wild. & T. Durand) Engl. & Diels, Monogr. Afr. Pflanzenfam. 6: 83, 1901

A9F81F8C-36C2-56AE-9C37-F454D2E75567

Fig. 40 ; Map 5G

≡ Monodora thonneri De Wild. & T. Durand, Bull. Soc. Roy. Bot. Belg., Compt. Rend. 38: 12, 1899.
= Diospyros oblongicarpa Gürke, Bot. Jahrb. Syst. 43: 200, 1909. Type. Cameroon. South Region, Bipindi, Zenker G.A. 3471, 1908: holotype B *n.v.*: isotype: K[K000199009]; US[US03899523]; WU[WU0040298].

#### Type.

Democratic Republic of the Congo. Equateur; Massanga (près de Monveda), *Thonner F. 104*, 24 Sep 1896: lectotype, designated by Boutique (1951b), p. 263, sheet here designated: BR[BR0000005113330]; isotypes: BR[BR0000005112715, BR0000005113040].

#### Description.

Tree to shrub, 3–10 m tall, d.b.h. up to 25 cm; stilt roots or buttresses absent, trunk not fluted. Indumentum of simple hairs; old leafless branches glabrous, young foliate branches glabrous. Leaves: petiole 3–8 mm long, 1–2 mm in diameter, glabrous, grooved, **blade inserted on the side of the petiole**; blade 11–20 cm long, 4–7.5 cm wide, elliptic to obovate, apex acuminate, acumen 1–2 cm long, base decurrent to cuneate, coriaceous to subcoriaceous, below glabrous when young and old, above glabrous when young and old, concolorous; midrib raised above, above glabrous when young and old, below glabrous when young and old; secondary veins 9 to 12 pairs, glabrous below; tertiary venation reticulate. Individuals bisexual; inflorescences ramiflorous on old or young foliate branches, axillary. Flowers with 9 perianth parts in 2 whorls, 1 to 2 per inflorescence; pedicel 5–18 mm long, ca. 1 mm in diameter, glabrous; in fruit 8–10 mm long, 3–4 mm in diameter, glabrous; bracts 3 to 7, several basal and one upper towards the lower half of pedicel, basal bracts ca. 1 mm long, ca. 1 mm wide; upper bracts ca. 2 mm long, 1 mm wide; sepals 3, valvate, free, 2–3 mm long, 1–2 mm wide, ovate, apex acute, base truncate, dark green, glabrous outside, glabrous inside, **margins flat**; petals basally fused, tube 3–6 mm long, inner and outer whorl not differentiated, equal; **lobes 14–31 mm long, 3–5 mm wide, linear to lorate (strap-shaped)**, apex acute, green, margins flat, glabrous outside, glabrous inside, pendulous; stamens numerous, in 3 to 4 rows, ca. 2 mm long, broad; connective discoid, glabrous; staminodes absent; carpels fused into a single structure, ca. 2 mm long, stigma bilobed, slightly capitate, sparsely pubescent. Fruit syncarpous, sessile, 40–60 mm long, 20–35 mm in diameter, ellipsoid, apex rounded, **glabrous, smooth, not or faintly ribbed**, color unknown; seeds not counted, 15–18 mm long, 8–9 mm in diameter, ellipsoid; aril absent.

**Figure 40.**
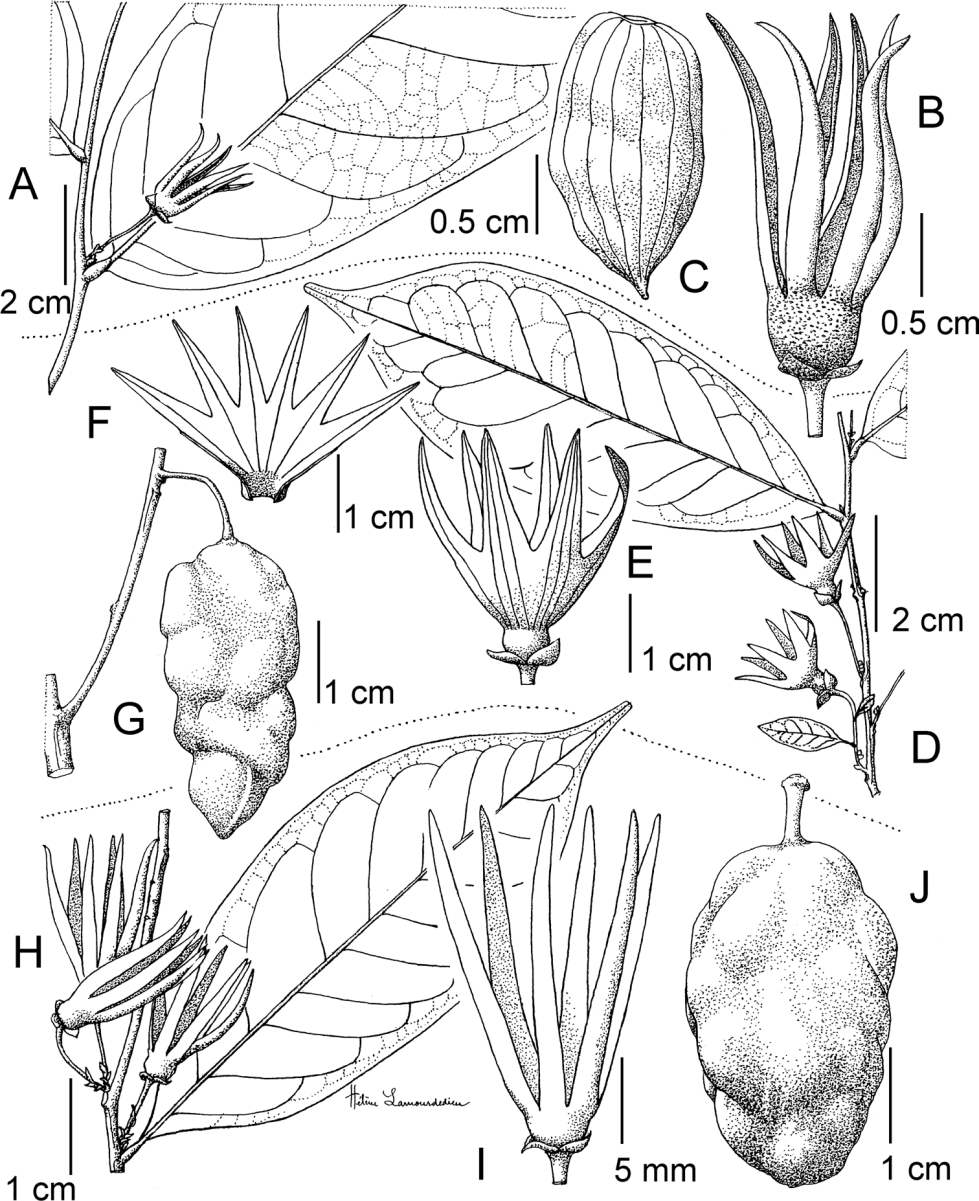
*Isolonazenkeri***A** flowering branch **B** flower **C** fruit. *Isolonacampanulata***D** flowering branch; **E** flower **E** corolla lobe opened **G** fruit. *Isolonathonneri***H** flowering branch **I** flower **J** fruit **A** from *Le Testu 5117***B** from *Le Testu 8001***C** from Klaine 2675 **D–G** from *Aubréville 6***H, I** from *Vrydagh 34***J** from *Lebrun 2032*. Drawings by Hélène Lamourdedieu, Publications Scientifiques du Muséum national d’Histoire naturelle, Paris; modified from Le Thomas (1969b, pl. 67, p. 359).

#### Distribution.

A central African species, known from Cameroon to Gabon and the Democratic Republic of Congo; in Cameroon known from the South and East regions.

#### Habitat.

An infrequent species; in lowland rain forests, near rivers and swamps. Altitude 450–750 m. a.s.l.

#### Local and common names known in Cameroon.

None recorded.

#### IUCN conservation status.

Least Concern (LC) (Cosiaux et al. 2019w).

#### Uses in Cameroon.

None reported.

#### Notes.

﻿﻿﻿*Isolonathonneri* is characterized by long, narrowly elliptic to linear and glabrous corolla lobes. In the absence of flowers, it is hard to distinguish from ﻿*I.dewevrei*.

#### Specimens examined.

**East Region**: Près de Banana 10 km ENE de Moloundou, 2.08°N, 15.28°E, *17 April 1971*, *Letouzey R.* 10682 (P,WAG,YA); near Ndongo ca 45 km WNW of Moloundou, 2.16°N, 14.83°E, *15 March 1973*, *Letouzey R.* 12085 (K,P); Près Ndongo à 45 km WNW de Moloundou, 2.15°N, 14.86°E, *16 March 1973*, *Letouzey R.* 12111 (BR,K,P,WAG,YA); près Ndongo à 40 km WNW de Moloundou, 2.15°N, 14.86°E, *16 March 1973*, *Letouzey R.* 12115 (BR,K,P,WAG,YA). **South Region**: Colline Ongongondje près Akonekye 15 km NW d’Ambam, 2.46°N, 11.16°E, *23 March 1970*, *Letouzey R.* 10205 (BR,COI,K,P,WAG,YA); Inselberg d’Akookas pres du village d’Akookas 38 km au sud est d’Ebolowa, 2.71°N, 11.27°E, *15 March 2001*, *Parmentier I.* 1943 (BRLU,WAG); Inselberg d’Akookas pres du village d’Akookas 38 km au sud est d’Ebolowa, 2.71°N, 11.27°E, *15 March 2001*, *Parmentier I.* 1961 (BRLU,WAG).

### ﻿﻿﻿﻿ Isolona zenkeri

Taxon classificationPlantaeMagnolialesAnnonaceae

Engl., Notizbl. Bot. Gart. Berlin-Dahlem 2: 301, 1899

486E45E6-1E54-53A7-9323-48DA6EF64AA0

**Figure 41.**
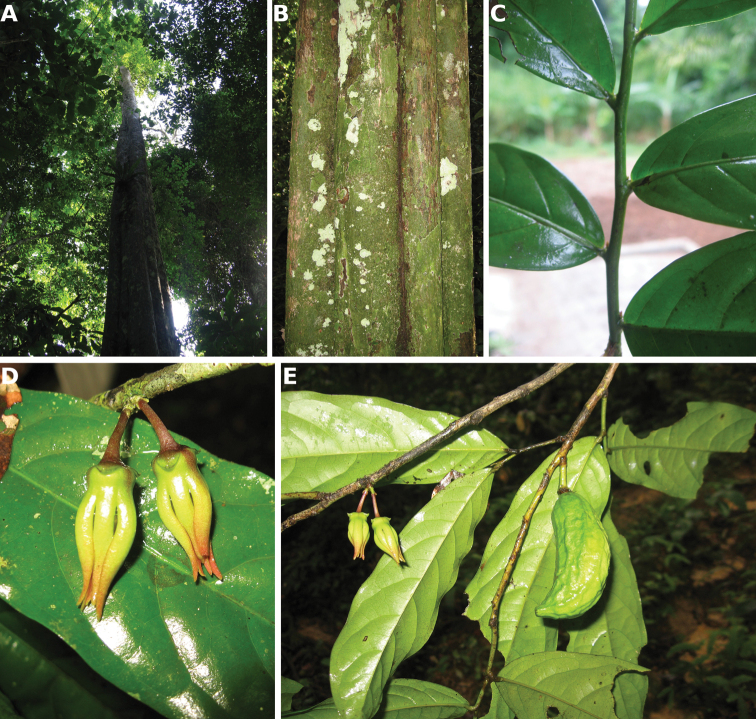
*Isolonazenkeri***A** tree in rain forest **B** detail of funneled trunk **C** detail of leaves, upper side, note raised midrib **D** flowers, side view, note erect lobes **E** flowering and fruiting branch **A, B***Sosef 2291*, Gabon **C***Sosef 2350*, Gabon **D, E***Sosef 2322*, Gabon. Photos Thomas L.P. Couvreur.

Figs 40 , 41 ; Map 5H

#### Type.

Cameroon. South Region; Bipindi, *Zenker G.A. 1186*, 1896: holotype: B[B 10 0154218]; isotypes: BM *n.v.*, G[G00011574]; K[K000199013]; WU[WU0025863].

#### Description.

Tree to shrub, 7–15 m tall, d.b.h. up to 15 cm; stilt roots or buttresses absent, trunk not fluted. Indumentum of simple hairs; old leafless branches glabrous, young foliate branches glabrous. Leaves: petiole 2–6 mm long, ca. 2 mm in diameter, glabrous, **slightly grooved, blade inserted on top of the petiole**; blade 16–23 cm long, 6.5–8.5 cm wide, oblong to oblanceolate, apex abruptly acuminate, acumen 1–2 cm long, base rounded to acute, coriaceous, below glabrous when young and old, above glabrous when young and old, concolorous; midrib raised above, above glabrous when young and old, below glabrous when young and old; secondary veins 11 to 13 pairs, glabrous below; tertiary venation reticulate. Individuals bisexual; inflorescences ramiflorous on young foliate branches, axillary. Flowers with 9 perianth parts in 2 whorls, 1 to 2 per inflorescence; pedicel 3–7 mm long, ca. 1 mm in diameter, glabrous; in fruit 5–15 mm long, 2–3 mm in diameter, glabrous; bracts 2 to 4, all basal, 1 mm long, 1 mm wide; sepals 3, valvate, free, 2–5 mm long, 2–4 mm wide, ovate, apex acute, base truncate, green to brown-red, glabrous outside, glabrous inside, margins flat; petals basally fused, tube 4–7 mm long, inner and outer whorl not differentiated, equal; **lobes 15–25 mm long, 3–4 mm wide, lorate (strap-shaped) to oblong, apex acute, light yellow to light green, margins curved inwards**, glabrous outside, glabrous inside, erect over receptacle, **verrucose when dried**; stamens ca. 40, in 3 to 4 rows, ca. 2 mm long, broad; connective discoid, glabrous, cream; staminodes absent; carpels fused into a single structure, ca. 3 mm long, stigma bilobed, slightly capitate, glabrous. Fruit syncarpous, sessile, 30–65 mm long, 15–30 mm in diameter, ellipsoid to globose, **apex rounded, glabrous, smooth, faintly ribbed to longitudinally ribbed**, green turning yellow when ripe; seeds not counted, 15–20 mm long, 8–10 mm in diameter, ellipsoid; aril absent.

#### Distribution.

Known from Cameroon to the west of Republic of Congo; in Cameroon known from the South and Littoral regions.

#### Habitat.

A fairly infrequent species, growing in lowland rain forests. Altitude 0–800 m a.s.l.

#### Local and common names known in Cameroon.

None recorded.

#### IUCN conservation status.

Least Concern (LC) (Cosiaux et al. 2019x).

#### Uses in Cameroon.

None reported.

#### Notes.

﻿﻿﻿*Isolonazenkeri* is characterized by its coriaceous corolla lobes with incurved margins, erect above the receptacle, and verrucose when dried.

#### Specimens examined.

**South Region**: Bipinde, 3.26°N, 10.20°E, *16 June 1918*, *Annet E.* 359 (WAG); 12 km from Kribi N of Ebolowa road between Kribi aifield and Kienke R, 2.88°N, 9.983°E, *18 June 1969*, *Bos J.J.* 4866 (BR,K,LM,MO,P,POZG,WAG,YA); 6 km N of km Kribi-Lolodorf, 3.08°N, 10.25°E, *12 March 1970*, *Bos J.J.* 6522 (BR,K,P,WAG,YA); Campo-Ma’an area Bibabimvoto, 2.25°N, 10.36°E, *01 February 2000*, *Elad M.* 1269 (WAG); Campo-Ma’an area Ebianemeyong, 2.46°N, 10.29°E, *24 May 2002*, *Elad M.* 1545 (KRIBI,WAG); 3 km S of Kwambo and 6 km WSW of Bipindi, 3.05°N, 10.36°E, *19 January 1987*, *Manning D.* 1453 (MO); Campo-Ma’an area Bifa, 2.65°N, 10.28°E, *12 October 2001*, *Tchouto Mbatchou G.P.* BIFAX_2 (WAG); Campo-Ma’an area Bibabimvoto, 2.21°N, 10.01°E, *13 May 2000*, *Tchouto Mbatchou G.P.* 2855 (KRIBI,WAG,YA); Campo-Ma’an area Bibabimvoto, 2.25°N, 10.04°E, *24 August 2000*, *Tchouto Mbatchou G.P.* 3009 (KRIBI,WAG,YA); Bipindi, 3.08°N, 10.41°E, *1896*, *Zenker G.A.* 1186 (B,BM,G,K); Bipindi, 3.08°N, 10.42°E, *01 January 1907*, *Zenker G.A.* 3375 (P); Bipindi, 3.08°N, 10.41°E, *01 January 1908*, *Zenker G.A.* 3471 (US); Bipindi, 3.08°N, 10.41°E, *01 January 1912*, *Zenker G.A.* 4405 (G,K,MO).

### ﻿﻿ Letestudoxa

Taxon classificationPlantaeMagnolialesAnnonaceae

Pellegr., Bull. Mus. Natl. Hist. Nat. 26: 654, 1920

601954BA-A6B1-5324-AE47-091685DEF224

#### Type species.

﻿﻿﻿*Letestudoxabella* Pellegr.

#### Description.

Lianas, to 40 m tall, d.b.h. up to 4 cm; stilt roots or buttresses absent. Indumentum of simple hairs. Leaves: petiole 3–12 mm long, 1–6 mm in diameter, pubescent to glabrous, slightly grooved, blade inserted on top of the petiole; blade 5–28 cm long, 3–12 cm wide, elliptic to obovate to oblong, apex acuminate to emarginate, base rounded to cordate; secondary veins 11 to 20 pairs; tertiary venation percurrent to indistinct. Individuals bisexual; inflorescences ramiflorous on old leafless branches, extra axillary. Flowers with 9 perianth parts in 3 whorls, 1 per inflorescence; pedicel 3–11 mm long, 1–7 mm in diameter; in fruit 3–7 mm long, 1–3 mm in diameter; bracts 2, one basal and one upper towards the lower half of pedicel; sepals 3, valvate, completely fused forming a nearly closed cup but tearing open at anthesis, 10–20 mm long; petals free, outer petals longer than inner; outer petals 3, imbricate, 30–55 mm long, 15–55 mm wide, ovate, apex acute to rounded, base attenuate; inner petals 3, imbricate, 15–35 mm long, 10–25 mm wide, elliptic to ovate, apex acute, base attenuate to acute; stamens up to 800, in 16 to 20 rows, 2–10 mm long, broad; connective flattened, pubescent, red; staminodes absent; carpels free, 150–175, 2–2.5 mm long, stigma capitate, pubescent. [Fruits only known from ﻿*L.bella*] Fruit pseudosyncarpous ca. 45 mm long, ca. 50 mm in diameter, globose; individual carpels sessile, 125 to 150 carpels, apex rounded to apiculate; seed 1, 15–16 mm long, 4–8 mm in diameter, ellipsoid; aril present.

A genus of lianas with three known species, from Angola (Cabinda), Cameroon, Gabon and Republic of Congo; in Cameroon two species, none endemic.

*Lestestudoxa* is distinguished by the lianescent habit and pseudosyncarpous fruits (carpels fusing after pollination to form a single fruiting unit, similar to those of the genus ﻿*Duguetia*, but the latter being trees) and the sepals completely fused around the floral bud and tearing at anthesis. The only other lianescent Annonaceae liana in Africa with pseudosyncarpous fruits is *Pseudartabotrys*, a monospecific genus endemic to Gabon (Le Thomas 1969b).

#### Taxonomy.

Chatrou (1998).

### ﻿Key to the species of ﻿*Letestudoxa* in Cameroon:

**Table:** 

1	Leaves generally elliptic (sometimes obovate), 5–18 cm long; upper bract not clasping base of flower buds	﻿***L.bella***
–	Leaves generally obovate (sometimes oblong), 15–28 cm long; upper bract clasping (amplexicaul) around the base of flower buds	﻿***L.lanuginosa***

### ﻿﻿﻿﻿ Letestudoxa bella

Taxon classificationPlantaeMagnolialesAnnonaceae

Pellegr., Bull. Mus. Natl. Hist. Nat. 26: 655, 1920

707BB6B8-C8EF-5D9B-B872-92E539DA876E

**Figure 42.**
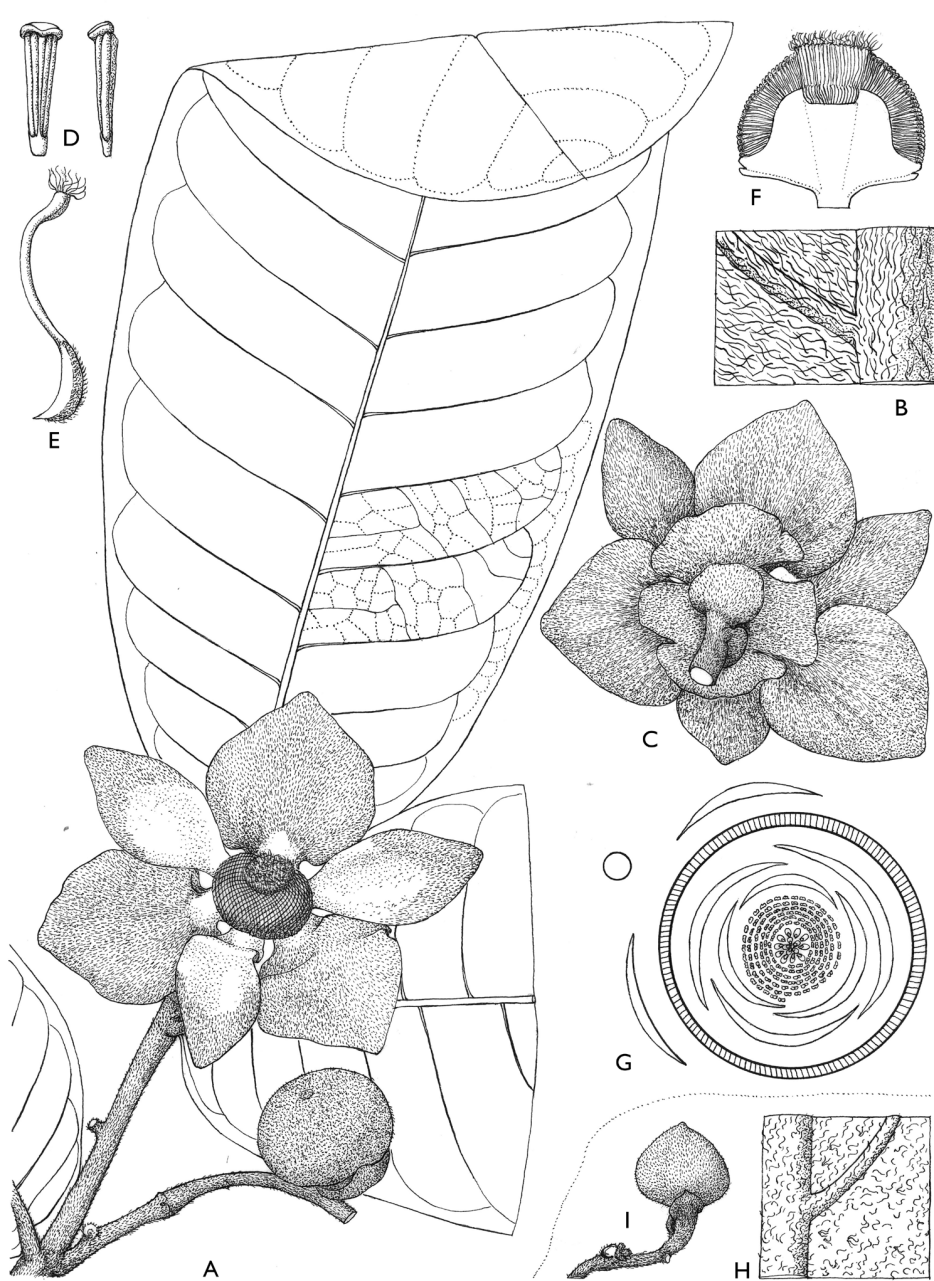
*Letestudoxalanuginosa***A** flowering branch, with one open flower and one flower bud, note sepals completely fused **B** detail of lower side of leaf blade showing dense pubescence **C** flower, bottom view **D** stamens, side and front views **E** carpels, note long elongated stigma **F** longitudinal section of receptacle **G** flower diagram. *Letestudoxabella***H** detail of lower side of leaf blade showing pubescence **I** flower bud, note sepals completely fused **A–G** from *Le Testu 9320*; 8–9 from *Le Testu 8362*. Drawings by Hélène Lamourdedieu, Publications Scientifiques du Muséum national d’Histoire naturelle, Paris; modified from Le Thomas (1969b, pl. 17, p. 99).

**Figure 43.**
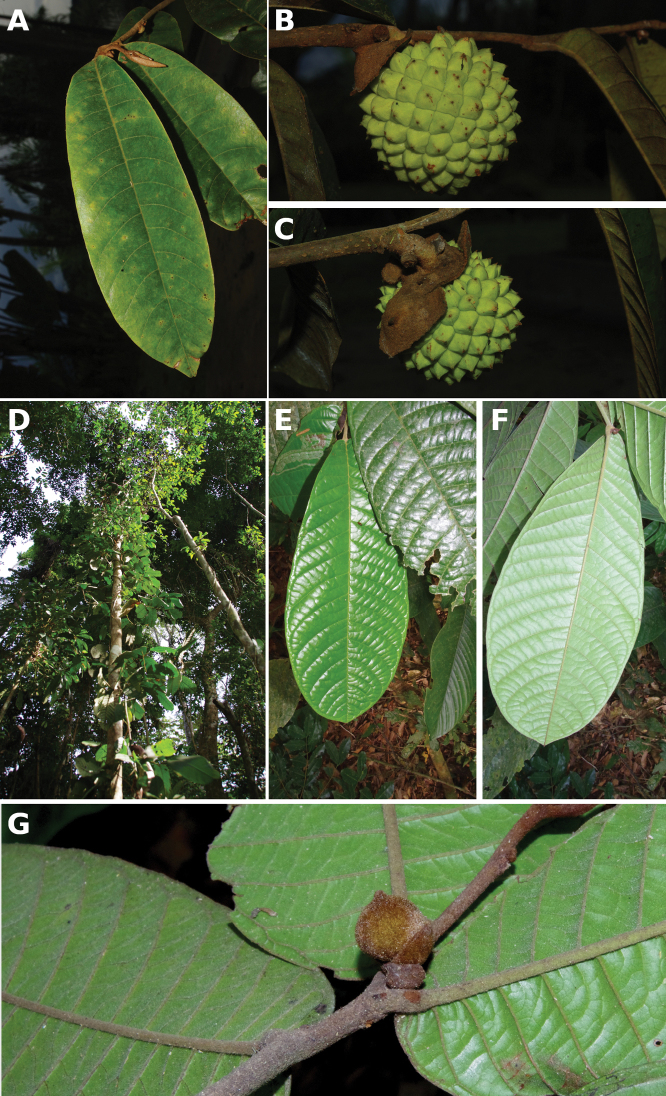
*Letestudoxabella***A** leaf, upper side **B** fruit, a pseudosyncarpous fruit **C** fruit, top view, note the large sepal remains. *Letestudoxalanuginosa***D** habit, liana climbing on tree trunk **E** leaf, upper side **F** leaf, lower side **G** detail of young flower bud, not completely enclosing sepals **A–C***Couvreur 600*, Gabon **D–G***Couvreur 1148*, Ma’an, Cameroon. Photos Thomas L.P. Couvreur.

Figs 42 , 43 ; Map 5I

= Letestudoxa grandifolia Pellegr., Bull. Mus. Natl. Hist. Nat. 26: 656, 1920. Type. Gabon. Nyanga, Ilou Micongo, *Le Testu G.M.P.C. 1442*, 4 Nov 1908: holotype: P[P00364780]; isotype: BM[BM000546387].
= Pachypodanthium simiarum Exell & Mendonça, J. Bot. 74 (Suppl.): 14, 1936. Type. Angola. Maiombe, Belize, *Gossweiler J. 6971*, 16 Fev 1917: holotype: BM[BM000067635]; isotypes: COI[COI00004878]; LISC[LISC000102, LISC000101, LISC000098, LISC000099, LISC000100].

#### Type.

Gabon. Nyanga; Midounga, near Tchibanga, *Le Testu G.M.P.C. 1637*, 24 Oct 1910: holotype: P[P00364779]; isotype: BM[BM000546388].

#### Description.

Liana, 16–40 m tall, d.b.h. up to 4 cm; stilt roots or buttresses absent. Indumentum of simple hairs; old leafless branches glabrous, young foliate branches densely pubescent. Leaves: petiole 3–10 mm long, 1–4 mm in diameter, pubescent to glabrous, slightly grooved, blade inserted on top of the petiole; **blade 5–18 cm long, 3–8 cm wide, elliptic to obovate**, apex acuminate to emarginate, acumen 0.5–1 cm long, base rounded to cordate, coriaceous, below densely pubescent when young, densely pubescent when old, above glabrous when young and old, concolorous; midrib sunken or flat, above glabrous when young and old, below pubescent when young and old; secondary veins 12 to 20 pairs, pubescent below; tertiary venation percurrent to indistinct. Individuals bisexual; inflorescences ramiflorous on old leafless branches, extra axillary. Flowers with 9 perianth parts in 3 whorls, 1 per inflorescence; pedicel 3–6 mm long, 1–3 mm in diameter, pubescent; in fruit 3–7 mm long, 1–3 mm in diameter, pubescent; bracts 2, one basal and one upper towards the lower half of pedicel, basal bract 5–7 mm long, 3–5 mm wide; upper bract 6–12 mm long, 4–7 mm wide, **not clasping the flower bud**; sepals 3, valvate, completely fused, tearing at anthesis, 10–20 mm long, base truncate, brown, densely pubescent outside, densely pubescent inside, margins flat; petals free, outer petals longer than inner; outer petals 3, 30–55 mm long, 15–55 mm wide, ovate, apex acute, base attenuate, yellow to orange with pinkish margins, margins crisped, densely pubescent outside, pubescent inside; inner petals 3, imbricate, 15–30 mm long, 10–25 mm wide, elliptic to ovate, apex acute, base attenuate to acute, yellow to orange with red marginal zone, margins wavy, densely pubescent outside, pubescent inside; stamens numerous, 2–3 mm long, broad; connective flattened, pubescent, red; staminodes absent; carpels free, around 175, ovary ca. 2 mm long, stigma capitate, pubescent. **Fruit pseudosyncarpous**, ca. 45 mm long, ca. 50 mm in diameter in total, globose; individual carpels sessile, 125 to 150 carpels, obovoid to obtrulloid, apex rounded to apiculate, sparsely pubescent, 6 to 7 ribbed, green turning red when ripe; seed 1 per monocarp, 15–16 mm long, 4–8 mm in diameter, ellipsoid; aril present, light brown.

#### Distribution.

Known from Angola, Cameroon to Gabon and in the Republic of Congo; in Cameroon known from the South region.

#### Habitat.

Growing in lowland primary and secondary rain forests. Altitude 50–750 m a.s.l.

#### Local and common names known in Cameroon.

None recorded.

#### IUCN conservation status.

Not evaluated.

#### Uses in Cameroon.

None reported.

#### Notes.

﻿﻿﻿*Letestudoxabella* is characterized by its mostly elliptic leaves (sometimes obovate too) which are generally smaller (5–18 cm) than those of ﻿*L.lanuginosa* (15–28 cm). In addition, in fresh material, ﻿*L.lanuginosa* has a bullate upper surface (versus a more leathery smooth upper surface in ﻿*L.bella*). In flower, ﻿*L.lanuginosa* is characterized by the upper bract clasping the flower bud, which is not the case in ﻿*L.bella*.

#### Specimens examined.

**South Region**: 26 km E of confluent Ntem River and Akom River near Ebolowa, 2.29°N, 11.86°E, *05 March 1970*, *Letouzey R.* 10097 (BR,P,P,YA); Tom (Nyabessan), 2.43°N, 10.52°E, *04 March 1963*, *Raynal J.* 10195 (P,YA).

### ﻿﻿﻿﻿ Letestudoxa lanuginosa

Taxon classificationPlantaeMagnolialesAnnonaceae

Le Thomas, Adansonia sér. 2, 6: 145, 1966

AFF58AEB-F153-58B5-BA5A-7C5BC7188A91

**Map 6.**
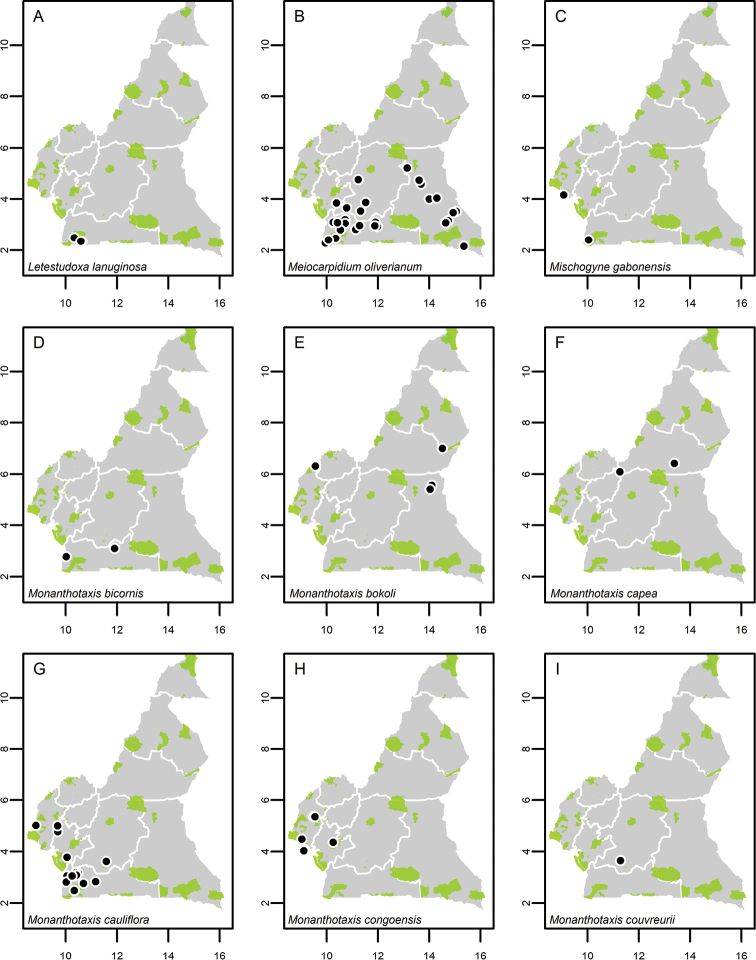
**A***Letestudoxalanuginosa***B***Meiocarpidiumoliverianum***C***Mischogynegabonensis***D***Monanthotaxisbicornis***E***Monanthotaxisbokoli***F***Monanthotaxiscapea***G***Monanthotaxiscauliflora***H***Monanthotaxiscongoensis***I***Monanthotaxiscouvreurii*. White borders represent region limits in Cameroon; green patches represent protected areas (see methods and Suppl. material 1: Fig. S1).

Figs 42 , 43 ; Map 6A

#### Type.

Gabon. Woleu-Ntem; Ncout, *Le Testu G.M.P.C. 9320*, 13 Oct 1938: lectotype, sheet here designated: P[P00364781]; isotypes: P[P00364782, P00364783].

#### Description.

Liana, 20 m tall, d.b.h. 2–3 cm in diameter; stilt roots or buttresses absent. **Indumentum of simple hairs**; **old leafless branches densely pubescent to tomentose, young foliate branches densely pubescent to tomentose.** Leaves: petiole 6–12 mm long, 3–6 mm in diameter, densely pubescent, slightly grooved, blade inserted on top of the petiole; **blade 15–28 cm long, 7–12 cm wide, obovate to sometimes oblong**, apex rounded or emarginate or obcordate or mucronate, acumen 0.1–1.9 cm long, **base cordate**, coriaceous, below densely pubescent when young, densely pubescent when old, above glabrous when young and old, concolorous; midrib sunken or flat, above densely pubescent when young and old, below densely pubescent when young, densely pubescent when old; secondary veins 11 to 18 pairs, densely pubescent below; tertiary venation reticulate. Individuals bisexual; inflorescences ramiflorous on old leafless branches, extra axillary. Flowers with 9 perianth parts in 3 whorls, 1 per inflorescence; pedicel 3–11 mm long, 4–7 mm in diameter, densely pubescent; in fruit unknown; bracts 2, one basal and **one upper towards the upper half of pedicel**, basal bract 8–13 mm long, 10–15 mm wide; upper bract 8–18 mm long, 6–10 mm wide, **clasping the flower bud**; sepals 3, valvate, completely fused, tearing at anthesis, 10–20 mm long, base truncate, brown, pubescent outside, pubescent inside, margins flat; petals free, outer petals longer than inner; outer petals 3, imbricate, 30–50 mm long, 30–40 mm wide, ovate, apex rounded to acute, base attenuate, orange to red, margins crisped, densely pubescent inside, pubescent outside; inner petals 3, imbricate, 25–35 mm long, 15–25 mm wide, elliptic to ovate, apex acute, base attenuate, claw ca. 5 mm long, orange to red, margins wavy, densely pubescent inside, pubescent outside; stamens 750 to 800, in 16 to 20 rows, 7–10 mm long, broad; connective flattened, pubescent; staminodes absent; carpels free, 150, ovary 2–2.5 mm long, stigma capitate, pubescent. Fruits unknown.

#### Distribution.

Known from southern Cameroon to Gabon; in Cameroon known from the South region.

#### Habitat.

A frequent species when present; growing on non-inundated soils, in primary or secondary forests, sometimes near rivers and swampy areas. Altitude 100–600 m a.s.l.

#### Local and common names known in Cameroon.

None recorded.

#### IUCN conservation status.

Not evaluated.

#### Uses in Cameroon.

None reported.

#### Notes.

See under ﻿*L.bella* for differences between the two species. This species can be confused with ﻿﻿﻿*Uvariabipindensis* (also a liana) by the size of its leaves (to 27 cm long) and cordate shape of the leaf base. They differ however but the presence of stellate hairs in ﻿﻿﻿*Uvariabipindensis*.

#### Specimens examined.

**South Region**: Ma’an 3 km on small road towards Ntem river, 2.34°N, 10.63°E, *25 February 2018*, *Couvreur T.L.P.* 1148 (MPU,WAG,YA); Campo Ma’an National Park 11 km on trail from Ebinanemeyong village on road 7 km from Nyabessan to Campo town, 2.48°N, 10.33°E, *13 February 2015*, *Couvreur T.L.P.* 697 (WAG,YA); Campo Ma’an National Park 11 km on trail from Ebinanemeyong village on road 7 km from Nyabessan to Campo town, 2.48°N, 10.33°E, *13 February 2015*, *Couvreur T.L.P.* 698 (WAG,YA); Campo-Ma’an area Boucle du Ntem, 2.34°N, 10.58°E, *16 February 2001*, *Tchouto Mbatchou G.P.* 3166 (KRIBI,WAG).

### ﻿﻿ Meiocarpidium

Taxon classificationPlantaeMagnolialesAnnonaceae

Engl. & Diels, Notizbl. Königl. Bot. Gart. Berlin 3: 54, 1900

1ACB0D2E-C96E-5825-8564-6584C2E2CBCF

#### Type species.

﻿﻿﻿*Meiocarpidiumoliverianum* (Baillon) D.M.Johnson & N.A.Murray

#### Description.

Same as species.

A monotypic genus from the Central Atlantic African region (Lower Guinea).

﻿*Meiocarpidium* is characterized by the presence of peltate scale-like hairs, a character unique among Central African Annonaceae.

#### Taxonomy.

Le Thomas (1969b).

### ﻿﻿﻿﻿ Meiocarpidium oliverianum

Taxon classificationPlantaeMagnolialesAnnonaceae

(Baillon) D.M.Johnson & N.A.Murray, PhytoKeys 97: 221, 2018

A737C488-39EF-525B-83E2-6C6528B4D313

**Figure 44.**
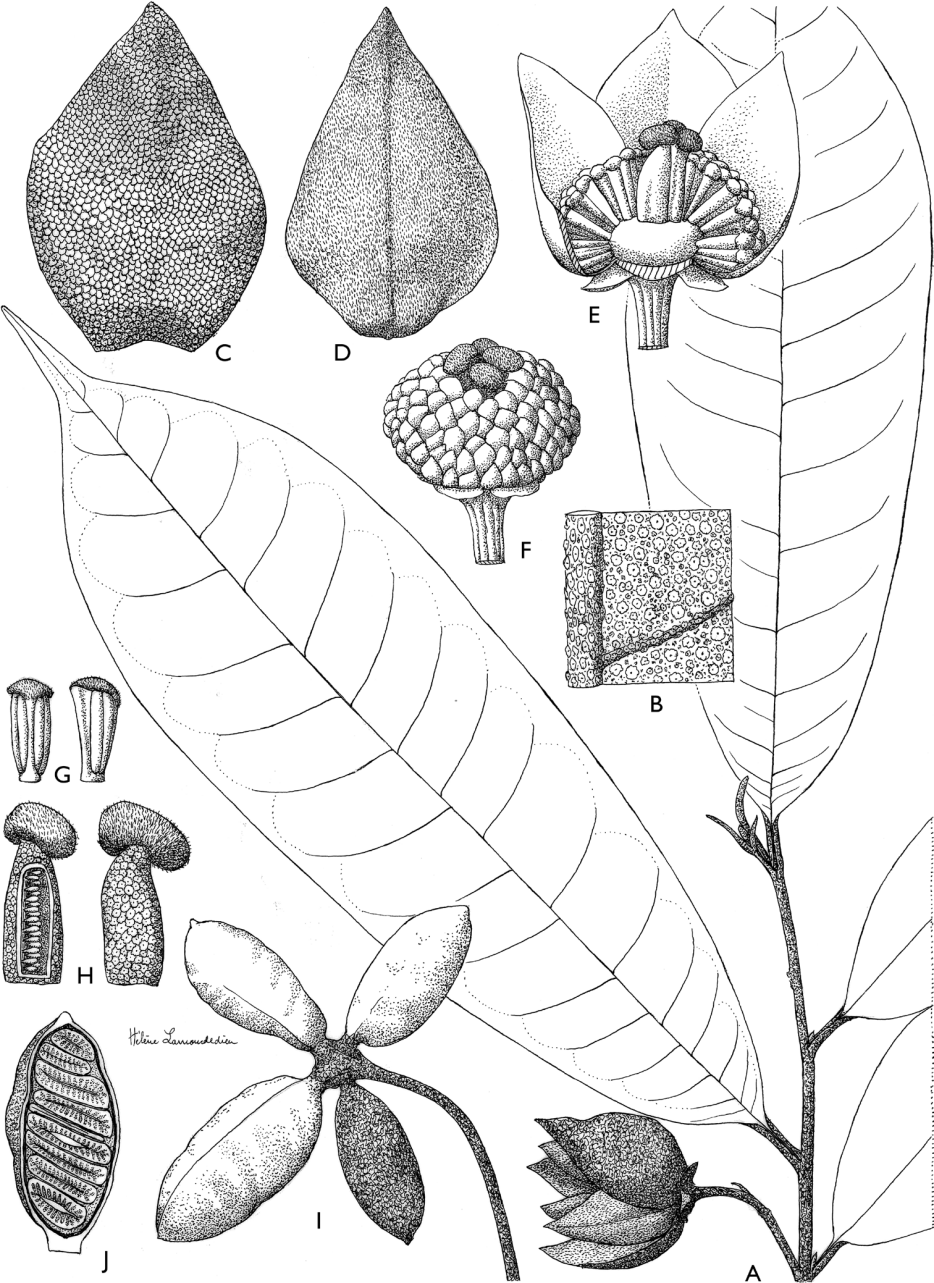
*Meiocarpidiumoliverianum***A** flowering branch **B** detail of lower leaf surface showing lepidote (scale like) hairs **C** outer petal, outer side **D** inner petal, outer side **E** detail of receptacle, side view, 3 petals removed **F** detail of receptacle, semi top view, all petals removed **G** stamens, side and front views **H** carpel, side view, and detail of ovules **I** fruit **J** longitudinal section of a monocarp **A–H** from *Zenker 3027***I–J** from *Letouzey 5473*. Drawings by Hélène Lamourdedieu, Publications Scientifiques du Muséum national d’Histoire naturelle, Paris; modified from Le Thomas (1969b, pl. 48, p. 267).

**Figure 45.**
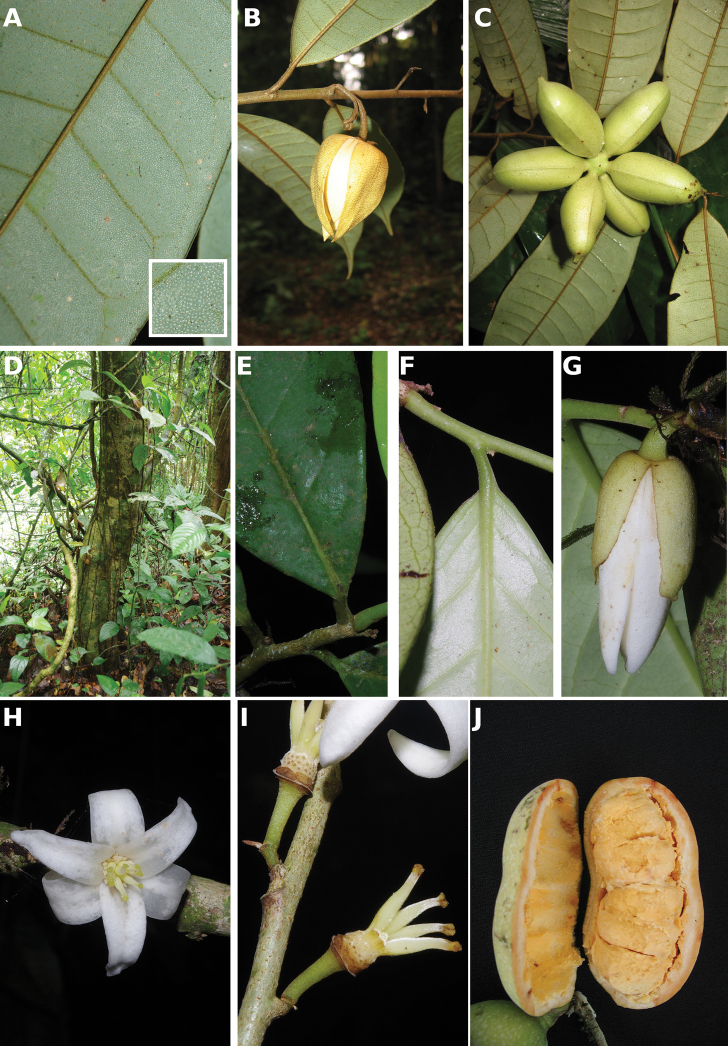
*Meiocarpidiumoliverianum***A** detail of lower side of leaf showing lepidote (scale-like) hairs; white bow zoomed in) **B** flowering branch **C** fruiting branch. *Mischogynegabonensis***D** base of trunk **E** base of leaf blade, upper side **F** base of leaf bade, lower side **G** flower bud, note sepals tearing **H** detail of flower, top view **I** detail of flower, all petals and sepals fallen, note elongated carpels **J** longitudinal section of monocarp, note hairy seeds **A–C***Sosef 2317***D–I***Couvreur 1033*, Mt Cameroon, Cameroon **J***Kenfack 1545*; Mt Cameroon, Cameroon. Photos **A–H** Thomas L.P. Couvreur **I** David Kenfack, Tropicos.org, Missouri Botanical Garden.

Figs 44 , 45 ; Map 6B

≡ Unona oliveriana Baillon, Adansonia 8: 307, 1868; ﻿Unonalepidota Oliv. Fl. Trop. Afr. 1: 36, 1868; ﻿Meiocarpidiumlepidotum (Oliv.) Engl. & Diels, Notizbl. Königl. Bot. Gart. Berlin 3: 55, 1900.
= Uvaria zenkeri Engl., Notizbl. Königl. Bot. Gart. Berlin 2: 293, 1899. Type. Cameroon. South Region, Bipindi, Zenker G.A. 1864, 1896: lectotype, here designated: K[K000198806]; isolectotypes: K[K000198807]; HBG[HBG502509, HBG502510]; WU[WU0025880, WU0025879].

#### Type.

Equatorial Guinea. Rio Muni; Muni River, *Mann G. 1774*, Aug 1862: lectotype, sheet here designated: K[K000795931]; isotypes: K[K000795932]; P[P00362615].

#### Description.

Tree, 8–20 m tall, d.b.h. 20 cm; stilt roots or buttresses absent. **Indumentum of peltate scale-like hairs**; old leafless branches glabrous, young foliate branches pubescent. Leaves: petiole 9–11 mm long, 2 mm in diameter, pubescent, grooved, blade inserted on top of the petiole; blade 15–20 cm long, 5–7 cm wide, oblong to obovate, apex acuminate to acute, acumen 1–2 cm long, base decurrent to acute, coriaceous, **below densely pubescent when young giving a silvery color**, pubescent when old, above glabrous when young and old, **discolorous, whitish below**; midrib impressed above, above glabrous when young and old, below pubescent when young and old; secondary veins 13 to 17 pairs, glabrous below; tertiary venation reticulate. Individuals bisexual; inflorescences ramiflorous on old or young foliate branches, leaf opposed. Flowers with 9 perianth parts in 3 whorls, 1 per inflorescence; pedicel 20–30 mm long, 1–2 mm in diameter, densely pubescent; in fruit 20–30 mm long, 2–3 mm in diameter, pubescent; bracts 2, one basal and one upper towards the upper half of pedicel, basal bract 8–9 mm long, 1 mm wide; upper bract 2–3 mm long, 1 mm wide; sepals 3, valvate, free, 2–3 mm long, 1–2 mm wide, triangular, apex acute, base truncate, green, pubescent outside, densely pubescent inside, margins flat; petals free, sub equal; outer petals 3, valvate, 15–25 mm long, 10–15 mm wide, ovate, apex acute, base truncate, cream, margins flat, densely pubescent outside, pubescent inside; inner petals 3, valvate, 15–25 mm long, 5–10 mm wide, ovate, apex acute, base narrowed into a claw 3–5 mm long, cream, margins flat, pubescent outside, glabrous inside; stamens 90 to 100, in 5 to 6 rows, 3–4 mm long, broad; connective discoid, glabrous, cream; staminodes absent; carpels free, 3 to 5, ovary 7–8 mm long, stigma capitate, pubescent. **Monocarps sessile, 3 to 4**, 40–60 mm long, 20–30 mm in diameter, cylindrical, apex rounded to apiculate, pubescent, smooth, **silver-green when ripe**; seeds 14 to 16 per monocarp, 10–20 mm long, 5–10 mm in diameter, flattened ellipsoid to oblong; aril absent.

#### Distribution.

Known from Cameroon, Equatorial Guinea Gabon and Republic of Congo, one collection for the Democratic Republic of Congo; in Cameroon known from East, South, Littoral and Central regions.

#### Habitat.

A frequent species when present; growing on swampy, periodically inundated or well-drained soils, in primary or secondary forests, sometimes near rivers and swampy areas. Altitude 50–500 m a.s.l.

#### Local and common names known in Cameroon.

mambéléngé, mandongé (dial. Baka bibaya).

#### IUCN conservation status.

Not evaluated (probably LC).

#### Uses in Cameroon.

***medicine***: bark used to allay fever; ***construction***: wood used to make spoons.

#### Notes.

﻿﻿﻿*Meiocarpidiumoliverianum* is easily identifiable by the presence of peltate scale-like hairs on the lower side of the leaf blade, calyx and fruits, giving these parts a silvery aspect and color.

The species was previsously known under the name ﻿*Meiocarpidiumlepidotum* (Oliv.) Engl. & Diels, but the name ﻿﻿﻿*Meiocarpidiumoliverianum* of Baillon (1868) was published earlier and has priority (Johnson and Murray 2018).

#### Selected specimens examined.

**Central Region**: Bank Nyong River near the new bridge ca 65 km SSW of Eséka, 3.65°N, 10.78°E, *17 June 1964*, *de Wilde W.J.J.O* 2735 (B,BR,K,MO,P,WAG,YA); Bafia region, 4.75°N, 11.23°E, *18 December 1957*, *de Wit H.C.D* 7948 (WAG). **East Region**: Berbérati, 4.58°N, 13.68°E, *01 March 1963*, *BPFV* 2664 (P); 47 km NW of Bertoua near road from Mbang to Abaka, 4.58°N, 13.68°E, *22 May 1961*, *Breteler F.J.* 1398 (A,BR,K,M,P,UC,WAG,YA); Bertoua 9 km along road to Deng Deng, 4.58°N, 13.68°E, *31 August 1961*, *Breteler F.J.* 1817 (A,BR,K,M,P,WAG,YA); 70 km south of Yokadouma 30 km after Ngato 15 km after river ALPICAM ‘base de vie’ then 40 km on forestry road starting 4 km before Maséa village, 3.15°N, 14.73°E, *04 March 2019*, *Couvreur T.L.P.* 1193 (MPU,WAG,YA); Yokadouma 3.52°N, 15.05°E, *11 September 1939*, *Jacques-Félix H.* 4904 (P); Deng Deng, 5.20°N, 13.13°E, *27 July 2014*, *Kamdem N.* 169 (YA); sur la route de Esseleké, 4.58°N, 13.68°E, *18 August 1955*, *Nana P.* 213 (P,YA); km 38 de la route de Deng-Deng, 4.58°N, 13.68°E, *17 September 1955*, *Nana P.* 257 (P,YA); Environ de Landjwe 25 km SW Yokadouma 3.47°N, 14.93°E, *16 June 1984*, *Satabié B.* 767 (P,YA). **Littoral Region**: Mapubi 30 km before Edea on Yaoundé-Edea road On forestry road 5 km direction to Sanaga river, 3.84°N, 10.38°E, *28 February 2018*, *Couvreur T.L.P.* 1182 (MPU,WAG,YA). **South Region**: 6 km N of km Kribi-Lolodorf, 3.08°N, 10.25°E, *12 March 1970*, *Bos J.J.* 6523 (WAG); Near village Oveng 27 km from Sangmélima along road to Yaoundé, 3.09°N, 11.90°E, *20 March 1962*, *Breteler F.J.* 2646 (BR,K,L,P,U,WAG,YA); Campo Ma an National Park 26 km after Ntem river, 3.07°N, 14.64°E, *08 March 2019*, *Couvreur T.L.P.* 376 (WAG,YA); on road from Lolodorf to Mekalat just after 1sty village Along the Malange river, 3.19°N, 10.71°E, *20 February 2012*, *Couvreur T.L.P.* 407 (WAG,YA); Campo Ma’an National Park 11 km on trail from Ebinanemeyong village on road 7 km from Nyabessan to Campo town, 2.46°N, 10.35°E, *14 February 2015*, *Couvreur T.L.P.* 709 (WAG,YA); Station de cacaoyer de N’koemvone 14 km On the road from Ebolowa to Ambam, 2.81°N, 11.13°E, *27 September 1974*, *de Wilde J.J.F.E* 7576 (BR,K,MO,P,WAG,YA); Ebolowa SW of Mbalmayo, 2.96°N, 11.28°E, *27 February 1964*, *de Wilde W.J.J.O* 1942 (BR,K,MO,P,WAG,YA); Près Akak 10 km W Sangmeli 2.96°N, 11.88°E, *11 March 1970*, *Letouzey R.* 10153 (P,YA); Ebom, 3.05°N, 10.71°E, *29 August 1996*, *Ndoum D.* 88 (KRIBI,WAG); Campo-Ma’an area 2.39°N, 10.07°E, *12 June 2001*, *van Andel T.R.* 3619 (KRIBI,WAG,YA); Campo-Ma’an area Akom II, 2.8°N, 10.53°E, *18 August 2001*, *van Andel T.R.* 3931 (U,WAG,YA); Bipindi, 3.08°N, 10.42°E, *1898*, *Zenker G.A.* 1864 (P); Bipindi, 3.08°N, 10.42°E, *01 January 1902*, *Zenker G.A.* 2505 (P); Bipindi, 3.08°N, 10.42°E, *01 January 1903*, *Zenker G.A.* 2521 (L,P,WAG); Bipindi, 3.08°N, 10.42°E, *01 January 1904*, *Zenker G.A.* 2947 (L,P,WAG); Bipindi, 3.08°N, 10.42°E, *01 January 1904*, *Zenker G.A.* 3221 (L,P,WAG); Bipindi, 3.08°N, 10.42°E, *01 January 1911*, *Zenker G.A.* 4209 (P,U); Bipindi, 3.08°N, 10.42°E, *01 January 1918*, *Zenker G.A.* 60 (P).

### ﻿﻿ Mischogyne

Taxon classificationPlantaeMagnolialesAnnonaceae

Exell, J. Bot. 70 (Suppl. 1): 213, 1932

797F4F54-4B31-5E71-8511-29F02E468B7E

#### Type species.

﻿*Mischogynemichelioides* Exell.

#### Description.

Genus description for Cameroon same as species.

A genus of trees or shrubs with five known species (Gosline et al. 2018), from West and Central Africa and one from East Africa (endemic to Tanzania). In Cameroon one species is known, not endemic (previously included in ﻿*Mischogyneelliotiana*).

The genus ﻿*Mischogyne* is easily identified when fertile by the presence of a torus, an extended receptacle, and several elongated cylindrical to ovoid carpels, which are divergent from each other.

#### Taxonomy.

Gosline et al. (2018).

### ﻿﻿﻿﻿ Mischogyne gabonensis

Taxon classificationPlantaeMagnolialesAnnonaceae

(Pellegr. ex Le Thomas) Gosline, Kew Bull. 74(2)-28: 13, 2019

9E6EEB3F-B7B2-55EE-9E3A-16D39BDA6573

Fig. 45 ; Map 6C

≡ Mischogyne elliotiana (Engl. & Diels) Le Thomas var. gabonensis Pellegr. ex Le Thomas, Flore du Gabon 16: 291, 1969.

#### Type.

Gabon. Nyanga; Mayumba, *Le Testu G.M.P.C. 1768*, 26 Aug 1914: lectotype, designated by Gosline et al. (2018), p. 28 [P00315820]; isolectotypes BM[BM000547338]; BR[BR0000008801661, BR0000008802330]; LISC[LISC000379]; P[P00315817, P00315818]; WAG[WAG0175098, WAG0175099, WAG0247284, WAG0247285, WAG0247286].

#### Description.

Tree, 3–25 m tall, d.b.h. up to 25 cm; stilt roots or buttresses absent. Indumentum of simple hairs; old leafless branches glabrous, young foliate branches shortly pubescent. Leaves: petiole 5–10 mm long, ca. 2 mm in diameter, sparsely pubescent to glabrous, cylindrical, blade inserted on the side of the petiole; blade 8–16 cm long, 4–7 cm wide, obovate, apex acuminate, acumen 1–3 cm long, base cuneate, papyraceous to subcoriaceous, below glabrous when young and old, above glabrous when young and old, concolorous; midrib impressed, above glabrous when young and old, below glabrous when young and old; secondary veins 6 to 9 pairs, glabrous above; tertiary venation reticulate. Individuals bisexual; inflorescences ramiflorous on old or young foliate branches, extra-axillary. Flowers with 9 perianth parts in 3 whorls, 1 per inflorescence; pedicel 5–12 mm long, 0.5–1 mm in diameter, pubescent; in fruit 7–15 mm long, 2–3 mm in diameter, glabrous; bracts few, reduced to a tuff of hairs, all basal; **sepals, valvate, completely fused in bud, tearing at anthesis into 2 or 3 parts**, 7–13 mm long, 3–5 mm wide, triangular, apex acute, base truncate, green, tomentose on both sides, margins flat; petals free, equal; outer petals 3, valvate, 14–16 mm long, 3–5 mm wide, narrowly elliptic, apex acute, base truncate, bright white, margins flat, densely pubescent outside, tomentose inside; inner petals 3, valvate, 14–16 mm long, 3–5 mm wide, narrowly elliptic, apex acute, base truncate, bright white, margins flat, densely pubescent outside, tomentose inside; stamens 50 to 60, in 3 to 4 rows, 1–3 mm long, elongated; **connective tongue shaped, pubescent**, cream; staminodes absent; **carpels free, 3–7**, ovary 5–6 mm long, stigma bilobed, densely pubescent. Monocarps sessile, 1 to 3, 60–65 mm long, 28–32 mm in diameter, cylindrical, apex rounded, glabrous, smooth, 1-ribbed, yellow when ripe; seeds 6 per monocarp, ca. 20 mm long, ca. 15 mm in diameter, ellipsoid, **covered with an indumentum of fine white hairs**; aril absent.

#### Distribution.

Cameroon, Gabon and Bioko island (Equatorial Guinea); in Cameroon known from the South and South-West regions.

#### Habitat.

A rare species in Cameroon (or at least uncommonly collected); in lowland primary or old secondary rain forests. Altitude 0–500 m a.s.l.

#### Local and common names known in Cameroon.

None recorded.

#### IUCN conservation status.

Endangered (EN) (Texier and Stévart 2021a).

#### Uses in Cameroon.

None reported.

#### Notes.

﻿﻿﻿*Mischogynegabonensis* is morphologically similar to the West African endemic *M.elliotiana* (of which it was formerly regarded as a variety) in the shape and size of its flowers and monocarps (Gosline et al. 2018). However, ﻿*M.gabonensis* is a large tree up to 25 m tall and 25 cm in d.b.h. with sepals completely fused and tearing at anthesis and has 3–7 carpels, whereas *M.elliotiana* is a smaller tree or shrub 3–10 m tall, with 3 sepals that are free with reduplicate-valvate margins, and 7–12 carpels.

#### Specimens examined.

**South Region**: Environs de Nko’olong 30 km E Campo, 2.40°N, 10.03°E, *21 November 1992*, *Satabié B.* 978 (YA). **South-West Region**: Mount Cameroon National Park Bakinguili trail above Bakinguili village, 4.09°N, 9.054°E, *02 April 2016*, *Couvreur T.L.P.* 1033 (WAG,YA); Fako Njonji Njonji along path to the lake Low canal forest, 4.15°N, 9.066°E, *26 September 2001*, *Kenfack D.* 1545 (MO).

### ﻿﻿ Monanthotaxis

Taxon classificationPlantaeMagnolialesAnnonaceae

Baill., Bull. Soc. Linn, Paris 2: 878, 1890

BC65F4F3-DF04-59C3-8438-9F9C23AFB0AA

= Clathrospermum Planch. ex Benth., Gen. Pl. 1(1): 29, 1862.
= Enneastemon Exell J. Bot. 70 (Suppl. 1): 209, 1932.
= Exellia Boutique, Bull. Jard. Bot. État Bruxelles 21: 117, 1951.
= Atopostema Boutique, Bull. Jard. Bot. État Bruxelles 21: 121, 1951.
= Gilbertiella Boutique, Bull. Jard. Bot. État Bruxelles 21: 124, 1951.

#### Description.

Scrambling shrubs or lianas, up to 30 m tall, d.b.h. up to 11 cm; stilt roots or buttresses absent. Indumentum of simple hairs. Leaves: petiole 1–11 mm long, 1–3 mm in diameter; blade 3–35 cm long, 1.4–12.5 cm wide, linear to elliptic to ovate to obovate to oblong, apex acuminate to acute, base decurrent to subcordate, discolorous, whitish below; midrib sunken or flat; secondary veins 6 to 23 pairs; tertiary venation percurrent. Individuals unisexual or bisexual; inflorescences cauliflorous or ramiflorous on old or young foliate branches, axillary, leaf opposed or extra axillary. Flowers with (6-)9 perianth parts in 2 or 3 whorls, 1 to more than 50 per inflorescence with a peduncle from 1 to 70 mm long; pedicel 1–60 mm long; in fruit 2–55 mm long; bracts 2 to 3, one or two basal and one inserted on the pedicel at varying levels; sepals 3, valvate, free, 0.5–10 mm long, ovate to elliptic to triangular, apex acute or obtuse, base truncate; petals free; outer petals 3 or 6 (when petals in one whorl), valvate, 1–50 mm long, 1–25 mm wide, ovate to elliptic, apex acute to rounded, base truncate; inner petals (0-)3, valvate, 0.5–25 mm long, 0.3–10 mm wide, ovate to elliptic to rhombic, apex acute to rounded, base truncate; stamens 1 to more than 125, in 1 to 6 rows, 1–2 mm long, linear or cuneiform to clavate; anthers sometimes connate apically covering connectives, connective discoid to truncate to absent, glabrous or pubescent; staminodes 6 to 12(13) or absent; carpels free, 3 to 150, 1–4 mm long, stigma bilobed or cylindrical or elongate and flattened at top, pubescent or glabrous. Monocarps sessile or stipitate, stipes 1–25 mm long; monocarps 2 to 25, 6–60 mm long, 5–25 mm in diameter, moniliform, cylindrical to ellipsoid, apex rounded to apiculate, smooth or verrucose or weakly torulose; seeds 6–25 mm long, 4–11 mm in diameter, ellipsoid; aril absent.

#### Type species.

﻿﻿﻿*Monanthotaxiscongoensis* Baill.

A genus of scrambling shrubs or lianas with 79 known continental African species, from West, Central and East Africa, plus around 23 endemic species from Madagascar. In Cameroon 26 species are known, six endemic.

The genus ﻿*Monanthotaxis* is easily identified when sterile by its lianescent or scrambling habit, discolorous leaves with very glaucous lower surface, and percurrent (parallel) tertiary venation. Some species are unisexual and have cauliflorous inflorescences; some are confirmed as monoecious, but for others, even though male and female flowers are known, it remains to be confirmed if they occur on the same or different individuals. The stamen number is highly variable within the genus ranging from 1 or 2 to over 100 (Hoekstra et al. 2018); some species have very characteristic large anthers fused towards the apex, hiding the small or absent connective (﻿*M.bicornis*, ﻿*M.pellegrinii* and ﻿﻿*M.zenkeri*). Some species also have staminodes varying in number from 6 to 12.

In the checklist to the plants of Mt Cameroon (Cable and Cheek 1998, p. 11) the species *M.oligandra* Exell is cited as present, but its occurrence in the country is not confirmed by the recent taxonomic revision (Hoekstra et al. 2021). Though we have not seen the specimen, *M.oligandra* is not known to occur north of South-West Democratic Republic of the Congo, so its presence in Cameroon is unlikely.

#### Taxonomy.

African species (excluding Madagascar) are revised in Hoekstra et al. (2021).

### ﻿Key to the Cameroonian species of ﻿*Monanthotaxis*.

The species of ﻿*Monanthotaxis* are variable in their vegetative characters (Hoekstra et al. 2021). Most specimens with at least an indication of the inflorescence position should be identifiable with this key, however if possible always check the floral characters with the descriptions as aberrant specimens may key wrongly if the identification is based on vegetative characters.

**Table:** 

1	Young foliate branches covered with erect hairs longer than 0.5 mm, normally around 1 mm long	**2**
–	Young foliate branches almost glabrous, or covered with appressed to ascending hairs, or with erect hairs shorter than 0.5 mm. (if in doubt choose this option: ﻿*M.diclina* has dense yellow-brown indumentum ascending to erect hairs and ﻿*M.letouzeyi* has ascending to erect hairs just shorter than 0.5 mm)	**7**
2	Inflorescences cauliflorous or axillary	**3**
–	Inflorescences extra–axillary	**4**
3	Petiole 5–7 mm long; flowers unisexual; female inflorescences cauliflorous; petals < 7 mm	﻿***M.pynaertii***
–	Petioles 2.5–6 mm long; flowers bisexual, all axillary; petals > 8 mm	﻿***M.filamentosa***
4	Leaves smaller than 17 cm; stamens 22 to 34; carpels glabrous; monocarps glabrous or at most with few scattered hairs on the stipe	**5**
–	At least several leaves larger than 18 cm; stamens 90 to 120; carpels pubescent; monocarps pubescent	**6**
5	Leaf apex normally obtuse; outer petals 15–19 mm long; carpels 27 to 38; seeds cylindrical, 14–21 mm long; stipes 7–10(–25) mm long	﻿***M.bokoli***
–	Leaf apex acute; outer petals 5.8–6.7 mm long; carpels 12 to 24; seeds ellipsoid, 7–8 mm long; stipes 3–4 (–6.5) mm long	﻿***M.ferruginea***
6	Hairs on young foliate branches around 1.5 mm long; leaves normally oblong to slightly obovate with broad leaf base and acute apex; outer petals 21–50 mm long; thecae large, covering more than half of the stamen length; carpels 22 to 24	﻿***M.hirsuta***
–	Hairs on young foliate branches around 1 mm long; leaves normally oblong–oblanceolate with narrow leaf base and acuminate apex; outer petals 12–22 mm long; thecae small, covering less than half of the stamen length; carpels 40 to 60	﻿***M.enghiana***
7	Leaves linear to narrowly elliptic, at least 5 times longer than wide, widest in middle or lower half of the leaf, secondary veins almost perpendicular to the primary vein (>75)	﻿***M.sterilis***
–	Leaves oblong, elliptic, obovate or oblanceolate, if > 5 times longer than wide then widest in upper half of the leaf and secondary veins forming acute angle with the primary vein (<60)	**8**
8	Inflorescences leaf-opposed or extra–axillary, but not consistently a few mm above the leaf axils	**9**
–	Inflorescences cauliflorous, axillary or supra–axillary and then consistently 1–8 mm above the leaf axils	**15**
9	Upper bract leaf-like, 7–15 mm long; stamens 15	﻿***M.vulcanica***
–	Upper bract not leaf-like, shorter than 5 mm or absent; stamens 9 or more than 5 mm or absent; stamens 9 or more than 23	**10**
10	Young foliate branches with sparse hairs not covering the entire surface; old leafless branches light brown or reddish brown	**11**
–	Young foliate branches with dense hairs completely covering the surface; old branches dark brown to blackish (or grey in ﻿*M.dielsiana*)	**12**
11	Branches light brown; stamens 80 to 125; carpels and monocarps hairy	﻿***M.gracilis***
–	Branches reddish brown; stamens 23 to 24; carpels and monocarps glabrous	﻿***M.laurentii***
12	Inflorescence a 1 to 8 flowered glomerule–like rhipidium; pedicels 1.2–2(–5) mm long; stamens 9; staminodes 6	﻿***M.elegans***
–	Inflorescence a solitary flower or a 2 to 4 flowered fascicle; pedicels 5–33 mm long; stamens more than 35; staminodes absent	**13**
13	Petiole 0.8–1.5 mm diam.; pedicels 0.4–0.5 mm diam.; stamens 36 to 48; carpels 11 to 14	﻿***M.montana***
–	Petiole 1.6–2.8 mm diam.; pedicels 1.4–2.1 mm diam.; stamens >60; carpels 41 to 50	**14**
14	Young foliate branches orange brown, densely covered with appressed, orange brown hairs; stamens c. 65	﻿***M.dielsiana***
–	Young foliate branches brown, densely covered with appressed, pale brown hairs; stamens >100	﻿***M.glaucifolia***
15	Inflorescences cauliflorous, at least most of them (in some species with unisexual flowers the male inflorescences are axillary on foliate branches)	**16**
–	Inflorescences axillary or supra–axillary, rarely some inflorescences cauliflorous; flowers always bisexual	**21**
16	Young foliate branches densely covered with erect or curly hairs 0.4–0.6 mm long	**17**
–	Young foliate branches covered with appressed to ascending hairs 0.1–0.2 mm long	**18**
17	Young foliate branches with reddish brown hairs; male flowers with 31 to 40 stamens in four whorls; 0 staminodes; carpels c. 120; seeds globose	﻿***M.letouzeyi***
–	Young foliate branches with yellow-brown hairs; male flowers with 6 stamens in one whorl and 12 small staminodes in an external whorl; carpels 80 to 100; seeds ellipsoid	﻿***M.diclina***
18	Young foliate branches with very short yellow-brown hairs; petiole long and less than 1 mm in diam.; carpels 65 to 85	﻿***M.submontana***
–	Young foliate branches with very short reddish brown or dark brown hairs; petiole thick, more than 1 mm in diam.; carpels 20 to 60(–76)	**19**
19	Flowers unisexual; female inflorescences cauliflorous in many-flowered panicle–like rhipidia, male inflorescences axillary; stamens 6; staminodes 12; carpels 48 to 60; leaf base rounded to cuneate; secondary veins 11 to 16	﻿***M.cauliflora***
–	Flowers bisexual; inflorescences a solitary flower, a few flowered fascicle or a short glomerule; stamens 9; staminodes 9; carpels 20 to 34; leaf base rounded to subcordate; secondary veins 7 to 14	**20**
20	Leaf base rounded or subcordate; flower buds ovoid, with outer petals and base of the 3 inner petals visible; monocarps with 1 seed; stipes 1–3.5 mm long	***M.klainei***
–	Leaf base narrowly subcordate; flower buds globose, with inner petals entirely covered by outer petals; monocarps with 1 to 3 seeds; stipes 4.5–12 mm long	﻿***M.whytei***
21	Leaf underside with dense appressed hairs more than 1 mm long, giving a silky appearance; inflorescences raceme-like or paniculate rhipidia; all 6 petals in one whorl	**22**
–	Leaf underside with different pubescence, not silky, the hairs either shorter or not appressed; inflorescences 1-flowered or in few-flowered fascicle-like rhipidia; petals in 2 whorls of 3	**23**
22	Leaf underside with yellowish hairs; inflorescence a many–flowered panicle-like rhipidium; flower buds depressed-globose	﻿***M.paniculata***
–	Leaf underside with whitish hairs; inflorescence a 4–10 flowered raceme-like rhipidium; flower buds deltoid–ovoid	﻿***M.congoensis***
23	Young foliate branches covered with white-yellowish hairs; stamens 6; filaments >1 mm long, much longer than half the total length of the stamen	﻿***M.hexamera***
–	Young foliate branches covered with yellowish-brown, reddish brown or dark brown hairs; stamens 8 to 35; filaments <1 mm long, occupying about half or less than the total length of the stamen	**24**
24	Young foliate branches covered with appressed hairs	**25**
–	Young foliate branches covered with ascending to erect hairs	**27**
25	Young foliate branches covered with yellowish–brown hairs, 0.2–0.4 mm long; stamens 15 to 17; thecae converging on top of the stamen, leaving only a small part of connective visible from above, filament wider than connective	﻿***M.bicornis***
–	Young foliate branches covered with reddish–brown hairs 0.1–0.2 mm long; stamens 8 to 12; thecae on sides of the stamen (not on top) with connective clearly visible from above, as wide as or wider than filament	**26**
26	Leaf base cuneate or sometimes rounded, with slightly thickened black margin; secondary veins forming an acute angle with primary vein, straight at their base; petals 6 in 2 whorls; 1 ovule; monocarps with 1 seed	﻿***M.vogelii***
–	Leaf base rounded, sometimes with thick globose glands at the margin; secondary veins curving from base; petals 3 to 4; 3 to 4 ovules; monocarps with 1 to 4 seeds	﻿***M.tripetala***
27	Young foliate branches with dense erect hairs, 0.3–0.4 mm long; stamens 35 in three to four whorls, sparsely hairy; carpels 16	﻿﻿***M.zenkeri***
–	Young foliate branches with ascending or erect hairs 0.1–0.3 mm long; stamens 8 to 24 in one or two whorls, glabrous, papillate or with a few hairs on the inner side of the connective; carpels 6 to 21	**28**
28	Flower bud just before anthesis with only 3 petals visible, inner petals completely covered by outer petals, normally clearly different in shape and smaller; stamens 15 to 24, glabrous, thecae converging on top of the stamen, connective much narrower than width of filament	﻿***M.pellegrinii***
–	Flower bud just before anthesis with inner petals at least partly visible, only slightly differing from outer petals in shape and size; stamens 8 to 15, papillate or with a few hairs on the inner side of the connective, thecae not converging on top of the stamen, connective clearly visible from above and as wide or wider than filament	**29**
29	Petiole 3–5 mm long, 0.8–0.9 mm thick inflorescences cauliflorous, ramiflorous or axillary; flower buds ovoid; stamens 13 to 15, basally connate	﻿***M.couvreurii***
–	Petiole 4.5–8 mm long, 1.2–2.6 mm thick; inflorescences axillary or slightly supra-axillary; flower buds rounded or slightly ovoid; stamens 9, free	**30**
30	Tertiary venation on upper leaf side strongly raised; inflorescences normally 3 to 16 flowered, with a sympodial rachis 3–17 mm long; ovules 2 to 3; monocarps smooth	﻿***M.foliosa***
–	Tertiary venation on upper leaf side flat; inflorescences 1 to 4 flowered, sympodial rachis absent; ovules 4 to 6; monocarps strongly tuberculate-rugulose	﻿***M.capea***

### ﻿﻿﻿﻿ Monanthotaxis bicornis

Taxon classificationPlantaeMagnolialesAnnonaceae

(Boutique) Verdc., Kew Bull. 25(1): 31, 1971

093196A1-6A11-5E8C-9F30-8B7AEEE72ACA

Map 6D

≡ Popowia bicornis Boutique, Bull. Jard. Bot. État Bruxelles 21: 115.

#### Type.

Democratic Republic of the Congo. Orientale; Yamboa, *Louis J.L.P. 8957*, 21 Apr 1938: lectotype, chosen by Hoekstra et al. (2021), p. 129: BR[BR0000008805386]; isolectotypes: BR[BR6102005255172, BR0000008805379]; K[K000913657]; P[P00362792].

#### Description.

Liana, 30 m tall, d.b.h. unknown. Indumentum of simple hairs; old leafless branches glabrescent with lenticels, **young foliate branches yellow-brown, with dense appressed yellow-brown hairs 0.2–0.4 mm long.** Leaves: petiole 3–5 mm long, 0.6–1 mm in diameter, densely pubescent, cylindrical, blade inserted on the side of the petiole; blade 6–12 cm long, 1.9–3.5 cm wide, oblong to elliptic, apex acuminate, acumen 0.5–1.5 cm long, base cuneate, papyraceous, below densely pubescent when young, densely pubescent to sparsely pubescent when old, above glabrous when young and old, discolorous, whitish below; midrib impressed, above sparsely pubescent to glabrous when young and old, below densely pubescent when young and old; secondary veins 11 to 14 pairs, glabrous above; tertiary venation percurrent. Individuals bisexual; inflorescences ramiflorous on young foliate branches, axillary. Flowers with 9 perianth parts in 3 whorls, 1 to 3 per inflorescence; pedicel 15–60 mm long, 0.3–0.6 mm in diameter, sparsely pubescent to densely pubescent; basal bract ca. 1 mm long, ca. 0.5 mm wide; upper bract 0.5–1 mm long, 0.5–1 mm wide; sepals 3, valvate, free, ca. 1 mm long, 2–3 mm wide, ovate, apex acute, base truncate, densely pubescent outside, densely pubescent inside, margins flat; petals free, outer petals longer than inner; inner petals entirely covered in bud; outer petals 3, 4–6.7 mm long, 4.6–6 mm wide, ovate, apex obtuse, base truncate, yellow to white, margins flat, densely pubescent outside, pubescent with a glabrous base inside; inner petals 3, valvate, 2.5–2.8 mm long, 2.2–3 mm wide, rhombic, apex obtuse, base truncate, yellow to white, margins flat, pubescent outside, pubescent, base glabrous inside; **stamens 15 to 17, in 1 row, ca. 1 mm long, oblong**; connective truncate, glabrous, thecae **converging apically**; staminodes absent; carpels free, 6 to 9, ovary ca. 1 mm long, stigma deeply bilobed, glabrous. Fruits unknown.

#### Distribution.

A central African species, known from Cameroon and the Democratic Republic of the Congo; in Cameroon known from the South region.

#### Habitat.

A rare species only known by two collections in Cameroon, in swamp and secondary rain forests. Altitude: 100–650 m a.s.l.

#### Local and common names known in Cameroon.

None recorded.

#### Preliminary IUCN conservation status.

Endangered (EN) (Hoekstra et al. 2021).

#### Uses in Cameroon.

None recorded.

#### Notes.

﻿﻿﻿*Monanthotaxisbicornis* is distinguished by the branches and petioles with appressed yellow-brown hairs, the cuneate leaf base, 15 to 17 stamens in one whorl and the thecae (pollen sacs) converging apically. Converging thecae are also found in ﻿*M.pellegrinii* but this latter species has ascending reddish brown hairs.

#### Specimens examined.

**South Region**: Route minière E of Mt Elephant ca 20 km SE of Kribi, 2.78°N, 10.01°E, *07 April 1970*, *Bos J.J.* 6735 (BR,K,LM,MO,P,WAG,YA); Oveng (Sangmelima), 3.09°N, 11.90°E, *22 March 1962*, *Breteler F.J.* 2692 (B,BR,K,MO,P,U,WAG,YA).

### ﻿﻿﻿﻿ Monanthotaxis bokoli

Taxon classificationPlantaeMagnolialesAnnonaceae

(De Wild. & T. Durand) Verdc., Kew Bull. 25(1): 24, 1971

9D71B8C0-FFA6-5731-9E6C-C4AD6A3F4374

Fig. 46 ; Map 6E

≡ ﻿﻿Xylopia bokoli De Wild. & T. Durand, Ann. Mus. Congo Belge, Bot. sér. 2, 1(2.1): 2, 1900; ﻿Popowia bokoli (De Wild. & T. Durand) Boutique, Fl. Congo Belge & Ruanda-Urundi 2: 349, 1951.
= Popowia iboundjiensis Pellegr., Bull. Soc. Bot. France 96: 212, 1950. Type. Gabon. Ngounié, Ndingui (Mullerville), *Le Testu G.M.P.C. 5729*, 11 nov 1925: lectotype, chosen by Hoekstra et al. (2021), p. 134: P[P00362791]; isolectotypes: BM[BM000553827]; LISC[LISC000388]; P[P00362789, P00362790].

#### Type.

Democratic Republic of the Congo. no region; no location, *Dewèvre A.P. 785*, no date: lectotype, sheet designated in Hoekstra et al. (2021), p. 133: BR[BR0000024941433]; isolectotypes: BR [BR0000008804020, BR0000008804358].

#### Description.

Shrub to liana, 6 m tall, d.b.h unknown. Indumentum of simple hairs; **old leafless branches glabrescent with lenticels, young foliate branches densely pubescent with erect reddish brown hairs 0.4–1.3 mm long**. Leaves: petiole 3–6 mm long, ca. 1 mm in diameter, pubescent, cylindrical, blade inserted on top of the petiole; blade 4.5–12.5 cm long, 2–7 cm wide, oblong to elliptic, **apex obtuse**, base subcordate, papyraceous to subcoriaceous, below sparsely pubescent when young and old, above sparsely pubescent to glabrous when young and old, discolorous, whitish below; midrib impressed, above pubescent when young and old, below pubescent when young and old; secondary veins 7 to 11 pairs, glabrous above; tertiary venation percurrent. Individuals bisexual; inflorescences ramiflorous on young and old leafless branches, leaf opposed or extra axillary. Flowers with 9 perianth parts in 3 whorls, 1 per inflorescence; pedicel 11–24 mm long, ca. 1 mm in diameter, sparsely pubescent to densely pubescent; in fruit 12–22 mm long, ca. 1 mm in diameter; basal bracts when present 1–2 mm long, ca. 1 mm wide; upper bract 1–2 mm long, ca. 1 mm wide; sepals 3, valvate, basally fused, 3–4 mm long, 5–7 mm wide, ovate, apex rounded, base truncate, sparsely pubescent outside, glabrous inside, margins flat; petals free, outer petals longer than inner, inner petals entirely covered in bud; **outer petals 3, 15–19 mm long, 8.4–11.8 mm wide**, ovate, apex rounded to obtuse, base truncate, margins flat, densely pubescent outside, pubescent inside; **inner petals 3, valvate, ca. 13 mm long, 4.5–5.5 mm wide**, linear to elliptic, apex acute, base truncate, margins flat, densely pubescent outside, **glabrous but pubescent towards margins inside**; stamens 24 to 32, in 3 to 4 rows, ca. 1 mm long, obovate; connective truncate, glabrous; staminodes absent; carpels free, 27 to 38, ovary ca. 3 mm long, stigma cylindrical, glabrous. **Monocarps stipitate, stipes 7–25 mm long, 2–3 mm in diameter**; monocarps 1 to 25, 26–65 mm long, 5–8 mm in diameter, moniliform, cylindrical, apex rounded to apiculate, glabrous, verrucose to weakly torulose, constricted around seeds when more than 1, yellow turning orange when ripe; **seeds 1 to 4 per monocarp, 14–21 mm long, 4–6 mm in diameter, subcylindrical**; aril absent.

**Figure 46.**
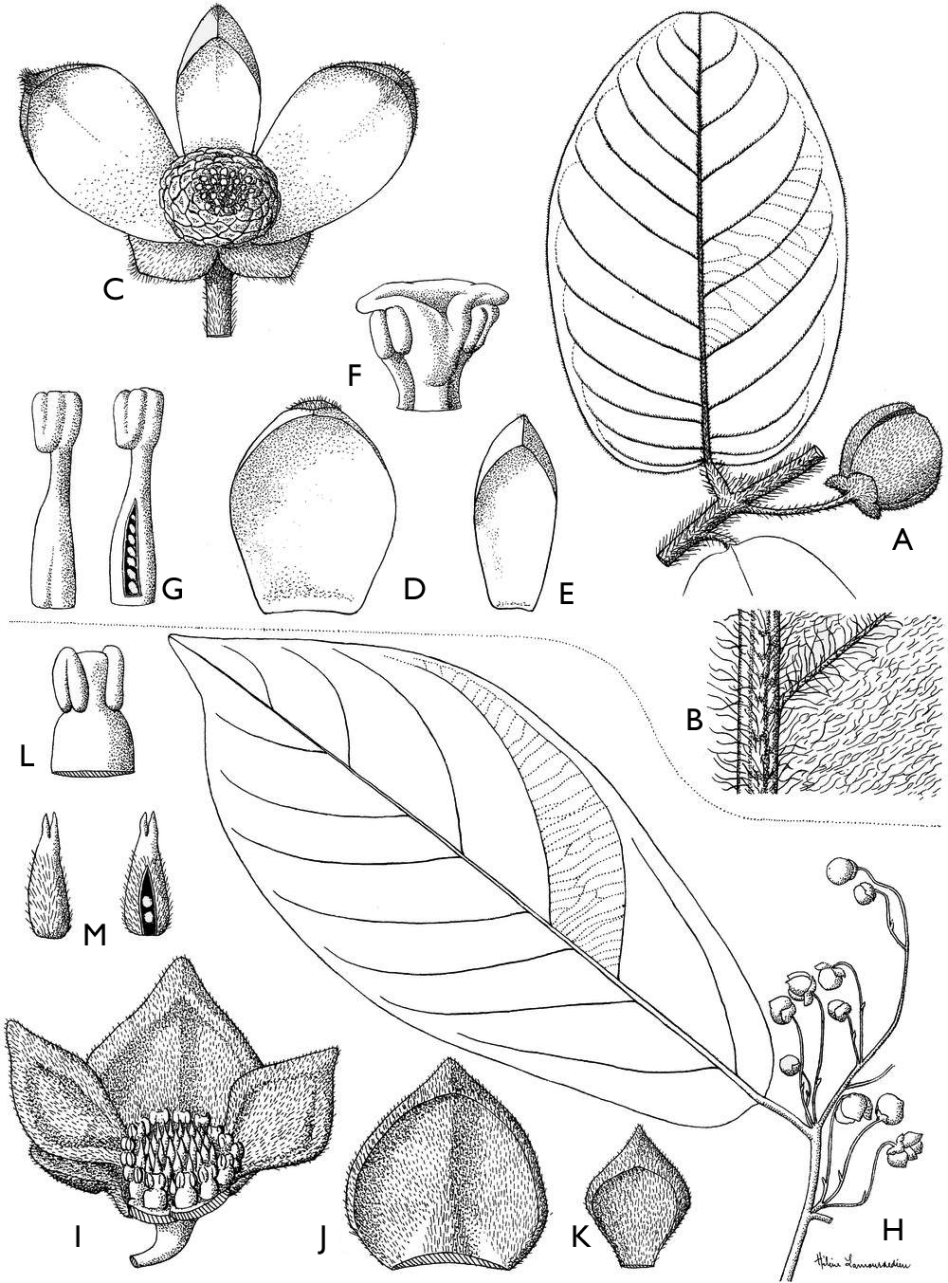
*Monanthotaxisbokoli***A** flowering branch **B** detail of pubescence, lower leaf side **C** flower with three petals removed **D** outer petal, inner side view **E** inner petal, inner side view **F** stamen **G** carpel and longitudinal section of carpel. *Monanthotaxispellegrinii***H** flowering branch **I** flower with three petals removed **J** outer petal, inner side view **K** inner petal inner side view **L** stamen **M** carpel and longitudinal section of carpel **A–G** from *Le Testu 5729***H–M** from *Le Testu 9028*. Drawings by Hélène Lamourdedieu, Publications Scientifiques du Muséum national d’Histoire naturelle, Paris; modified from Le Thomas (1969b, pl. 41, p. 229).

#### Distribution.

A central African species, from Cameroon to Gabon, and the Central African Republic and the Democratic Republic of the Congo; in Cameroon known from the Adamaoua, East and South-West regions.

#### Habitat.

A rare species in Cameroon but with a wide distribution; in swamp forests, gallery forests, premontane primary and secondary rain forests. Altitude: 600–1200 m a.s.l.

#### Local and common names known in Cameroon.

None recorded.

#### Preliminary IUCN conservation status.

Least concern (LC) (Hoekstra et al. 2021).

#### Uses in Cameroon.

None recorded.

#### Notes.

﻿﻿﻿*Monanthotaxisbokoli* is distinguished by its dense erect reddish brown hairs on the branches and leaves, large flowers, almost glabrous inner petals inside and monocarps with long stipes and subcylindrical seeds. In pubescence ﻿*M.bokoli* resembles ﻿*M.ferruginea*.

#### Specimens examined.

**Adamaoua Region**: rocher conglomératique de Mbalarzi dans la vallée de la Mbere (65 km NE de Meiganga), 7°N, 14.51°E, *16 October 1963*, *Letouzey R.* 6195 (P). **East Region**: km 17 of Bétaré Oya-Meiganga road 5 km SE of Ndokayo, 5.55°N, 14.1°E, *03 February 1966*, *Leeuwenberg A.J.M.* 7716 (BR,K,MO,P,WAG,YA); Kongolo, 5.4°N, 14.03°E, *01 April 1914*, *Mildbraed G.W.J.* 9010 (K). **South-West Region**: Piste Akwaya-Mamfe près Makomono 7 km S Akwaya, 6.31°N, 9.557°E, *25 July 1975*, *Letouzey R.* 14081 (MO,P,WAG,YA).

### ﻿﻿﻿﻿ Monanthotaxis capea

Taxon classificationPlantaeMagnolialesAnnonaceae

(E. G. Camus & A. Camus) Verdc., Kew Bull. 25(1): 21, 1971

C7196EDC-2DF7-53C0-B45B-20A73D00CA83

**Figure 47.**
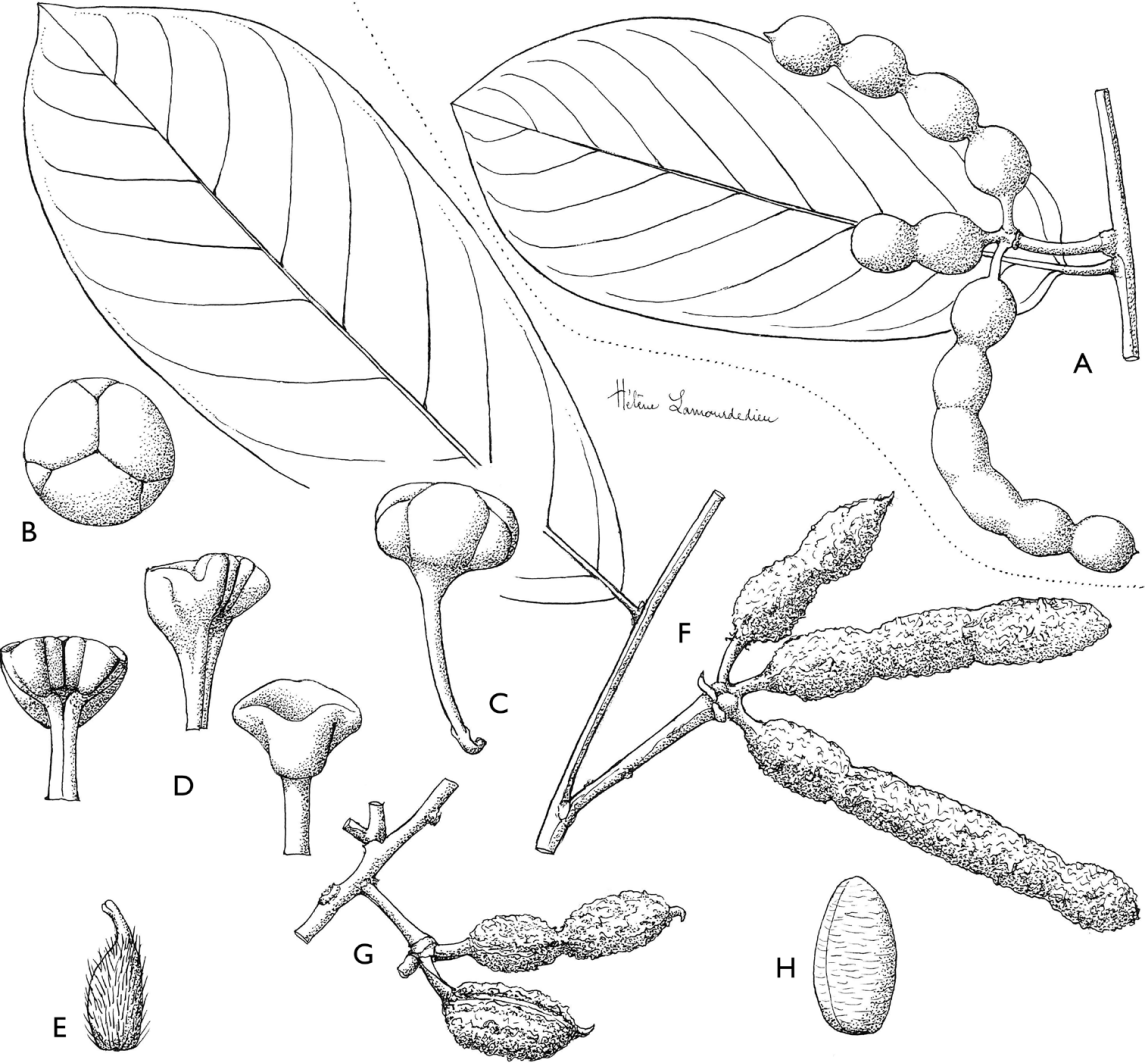
*Monanthotaxisseretii* (De Wild.) P.H.Hoekstra, not in Cameroon **A** fruiting branch. *Monanthotaxiscapea***B** flower bud seen from above **C** flower bud side view **D** stamen, outer, inner and side views **D** carpel **E** fruiting branch **F** fruiting branch **G** seed **A** from *Louis 11405***A–H** from *Hallé 3561*. Drawings by Hélène Lamourdedieu, Publications Scientifiques du Muséum national d’Histoire naturelle, Paris; modified from Le Thomas (1969b, pl. 44, p. 247, pro parte).

**Figure 48.**
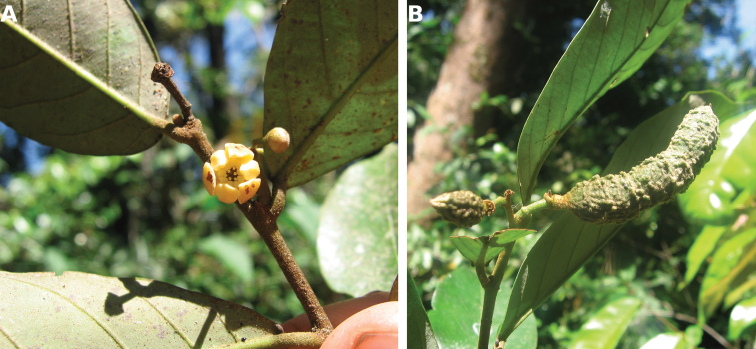
*Monanthoatxiscapea***A** flower, top view **B** fruit, note rugulose/verucose monocarp surface **A, B***Sosef 2238*, Gabon. Photos Thomas L.P. Couvreur.

Figs 47 , 48 ; Map 6F

≡ Popowia capea E.G. Camus & A. Camus, Bull. Sc. & Ind. Maison Roure-Bertrand Fils, Grasse Ser. II. No. 8: 5, 1913; ﻿﻿Enneastemoncapeus (E.G. Camus & A. Camus) Ghesq., Rev. Zool. Bot. Africaines 32: 141, 1939.
= ﻿﻿﻿Monanthotaxis schweinfurthii var. tisserantii (Le Thomas) Verdc., Kew Bull. 25(1): 22, 1971; ﻿Enneastemon seretii (De Wild.) Robyns & Ghesq. var. tisserantii Le Thomas, Andeasonia sér. 2: 292, 1963; ﻿Enneastemonschweinfurthii(Engl. & Diels)Robyns & Ghesq.var.tisserantii Le Thomas, Fl. Gabon 16: 254, 1969. Type. Central African Republic: Lobaye, Boukoko, *Tisserant C. 1710*, 10 Apr 1950: holotype: P[P01982418].

#### Type.

Ivory Coast. no region; “l’est du pays d’Attié”, *Angoulvant G.-L. s.n.*, 1910: lectotype, chosen by Hoekstra et al. (2021), p. 138: P[P00362786]; isotypes: BM[BM001125039]; E[E00624344]; K[K000041008]; P[P00362784, P00362785].

#### Description.

Shrub to liana, height unknown, d.b.h. unknown. Indumentum of simple hairs; old leafless branches glabrescent, young foliate branches densely pubescent with ascending to erect reddish brown hairs 0.2–0.3 mm long. Leaves: petiole 5–8 mm long, 1–3 mm in diameter, densely pubescent, slightly grooved, blade inserted on top of the petiole; blade 7.4–19.7 cm long, 3.4–7.7 cm wide, oblong to oblanceolate, apex acuminate to acute, acumen 1.5 cm long, **base cuneate to rounded**, subcoriaceous, below sparsely pubescent when young, glabrous when old, above glabrous when young and old, discolorous, whitish below; midrib depressed, above sparsely pubescent to glabrous when young and old, below sparsely pubescent when young and old; secondary veins 7 to 12 pairs, glabrous above; tertiary venation percurrent. Individuals bisexual; inflorescences ramiflorous on young and old leafless branches, axillary. Flowers with 9 perianth parts in 3 whorls, 1 to 4 per inflorescence; pedicel 6–9 mm long, 0.5–1 mm in diameter, densely pubescent; in fruit 11–16 mm long, 1–2 mm in diameter; basal bracts not seen, upper bract ca. 1 mm long, ca. 1 mm wide; sepals 3, valvate, basally fused, ca. 1 mm long, ca. 1 mm wide, ovate, apex obtuse, base truncate, densely pubescent outside, glabrous inside, margins flat; petals free, outer petals longer than inner, **inner petals partly covered in bud**; outer petals 3, 3.5–5 mm long, 2–3.8 mm wide, ovate, apex acute, base truncate, margins flat, densely pubescent outside, pubescent inside; inner petals 3, valvate, 2.5–3.5 mm long, 1.5–2.4 mm wide, rhombic, apex acute, base truncate, light yellow to white, margins flat, pubescent outside, pubescent inside; **stamens 8 to 10, in 1 row, 1–2 mm long, clavate, enlarged in the upper part, connective truncate covering the anthers, glabrous**; staminodes absent; **carpels free, 4 to 6**, ovary 7–2 mm long, stigma elongate, glabrous. Monocarps stipitate, stipes 3–6 mm long, 2–3 mm in diameter; monocarps 1 to 6, 20–79 mm long, 8–10 mm in diameter, moniliform, ellipsoid, apex ellipsoid, pubescent, **tuberculate, weakly torulose**, color unknown; seeds 1 to 6 per monocarp, ca. 12 mm long, 7–8 mm in diameter, ellipsoid; aril absent.

#### Distribution.

A west and central African species with a disjunct distribution between Ivory Coast and Cameroon to Gabon, Central African Republic and the Democratic Republic of the Congo; in Cameroon known from Adamaoua and West regions.

#### Habitat.

A rare species in Cameroon known from two collections; in gallery forests in drier regions, swamp forests, in submontane rain forests and old secondary forest. Altitude 700–1000 m a.s.l.

#### Local and common names known in Cameroon.

None recorded.

#### Preliminary IUCN conservation status.

Vulnerable (VU) (Hoekstra et al. 2021).

#### Uses in Cameroon.

None recorded.

#### Notes.

﻿﻿﻿*Monanthotaxiscapea* belongs to the ﻿*M.schweinfurthii* complex (for details see Le Thomas 1969b, p. 248, under ﻿*Enneastemonschweinfurthii*). This complex is mainly characterized by the shape of its stamens being enlarged in the upper part, the connective covering the small anthers and a narrow filament. Several species were initially described, then placed as varieties of ﻿*M.schweinfurthii* and finally re-elevated to the rank of species and now includes these species: *M.aestuaria* P.H.Hoekstra (not in Cameroon), ﻿*M.barteri* (Baill.) Verdc. (not in Cameroon), ﻿*M.foliosa*, ﻿*M.ochroleuca* (Diels) P.H.Hoekstra (not in Cameroon), ﻿*M.schweinfurthii* (Engl. & Diels) Verdc. (not in Cameroon), ﻿*M.seretii* (De Wild.) P.H.Hoekstra (not in Cameroon) following Hoekstra et al. (2021).

In the fruiting stage, ﻿﻿﻿*Monanthotaxiscapea* is easily distinguished by its monocarps that are strongly tuberculate-rugulose, unique within the *schweinfurthii* complex. Flowering material however is difficult to distinguish from other species of the *schweinfurthii* complex, but may be recognized by the combination of ascending to erect reddish brown hairs on the young foliate branches, cuneate to rounded leaf bases, and 4 to 6 ovules per carpel.

#### Specimens examined.

**Adamaoua Region**: A 4 km au SO de Dir près de Bagodo, 6.42°N, 13.38°E, *30 July 1966*, *Letouzey R.* 7570 (P,YA). **West Region**: Près Kongi (10 km au NO de Kimi bankim sur route Fouman-Banyo), 6.09°N, 11.26°E, *27 June 1967*, *Letouzey R.* 8738 (P,YA).

### ﻿﻿﻿﻿ Monanthotaxis cauliflora

Taxon classificationPlantaeMagnolialesAnnonaceae

(Chipp) Verdc., Kew Bull. 25(1): 30, 1971

38F57E34-B1EB-56AD-8656-BCADB13ED1B2

**Figure 49.**
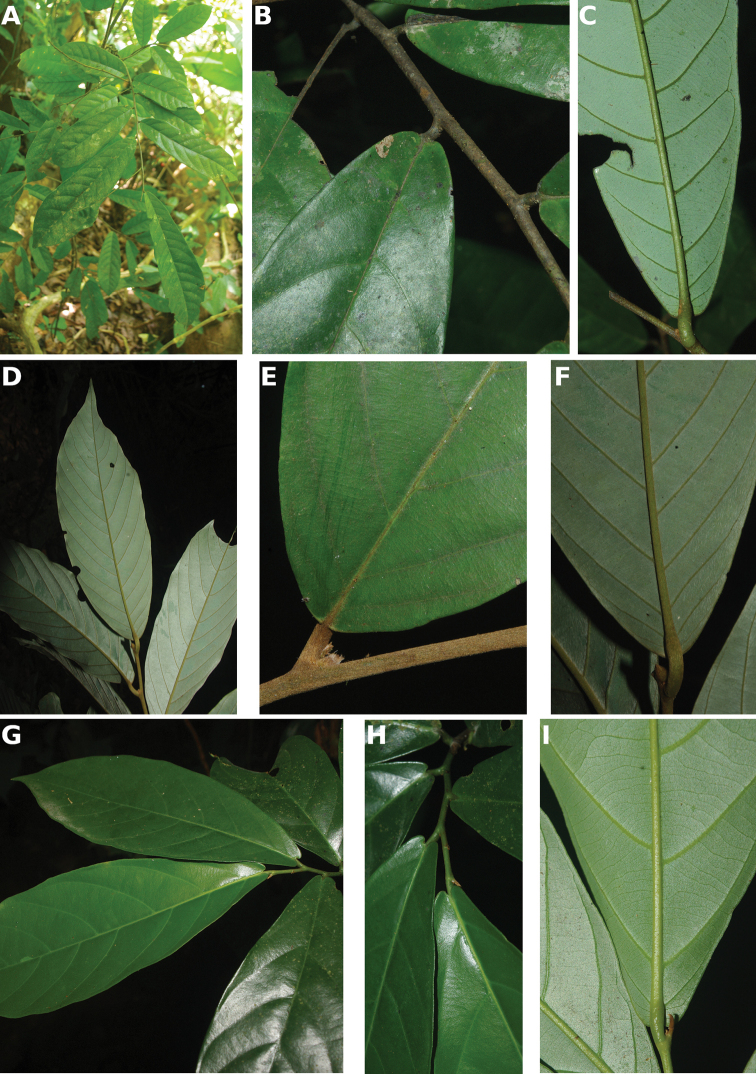
*Monanthotaxiscauliflora***A** habit **B** base of leaf blade, upper side **C** base of leaf blade, lower side. *Monanthotaxiscongoensis***D** leaf, lower side **E** base of leaf blade, upper side **F** base of leaf blade, lower side. *Monanthotaxiselegans***G** leaves, upper side **H** base of leaf blade, upper side, not subcordate base **I** base of leaf blade, lower side **A–C***Couvreur 676*, Campo, Cameroon **D–F***Couvreur 626*, Ebo, Cameroon **G–I***Couvreur 704*, Campo, Cameroon. Photos Thomas L.P. Couvreur.

Figs 49 , 62 ; Map 6G

≡ Popowia cauliflora Chipp, Bull. Misc. Inform. Kew, 5: 182, 1923; ﻿﻿Clathrospermummannii Oliv., Fl. Trop. Afr. 1: 25, 1868, *pro parte*, *quoad specim*. ♀; ﻿Popowiamannii (Oliv.) Engl. & Diels, (non Baill.) in Engl. Monogr. Afr. Pfl. 6: 49, 1901, *pro parte*, *quoad specim*. ♀ *nom. illeg.*; ﻿Popowiadiclina Sprague, Bull. Misc. Inform. Kew 2: 53, 1908, *pro parte*, *quoad specim*. ♀.

#### Type.

Nigeria. Cross River State; Old Calabar, *Thomson W.C. s.n.*, 1963: lectotype, chosen by Le Thomas (1969b), p. 218: K[K000198911].

#### Description.

Liana, 25 m tall, d.b.h. up to 4 cm. Indumentum of simple hairs; old leafless branches glabrescent, **young foliate branches pubescent with very short reddish brown hairs 0.05–0.1 mm long**. Leaves: petiole 5–9 mm long, 1–2 mm in diameter, densely pubescent, weakly grooved, blade inserted on top of the petiole; blade 12.5–15.2 cm long, 4.8–5.8 cm wide, oblong to oblanceolate, apex acuminate to acute, acumen ca. 1 cm long, base cuneate to rounded, papyraceous, below sparsely pubescent when young, glabrous when old, above glabrous when young and old, discolorous, whitish below; midrib sunken or flat, above densely pubescent when young and old, below sparsely pubescent when young, sparsely pubescent to glabrous when old; secondary veins 11 to 16 pairs, sparsely pubescent above; tertiary venation percurrent. **Individuals unisexual, monoecious, dimorphic, female inflorescences cauliflorous, male ones axillary, ramiflorous.** Flowers with 9 perianth parts in 3 whorls, male and female flowers dimorphic, **male inflorescences with 1 to 6 flowers, female inflorescence a condensed panicle to 12 cm in diameter with many flowers**; male flowers: pedicel 5–6 mm long, ca. 1 mm in diameter, densely pubescent; basal bract ca. 1 mm long, ca. 1 mm wide; upper bract ca. 1 mm long, ca. 1 mm wide; sepals 3, valvate, basally fused, ca. 1 mm long, ca. 1 mm wide, triangular, apex acuminate, base truncate, densely pubescent outside, densely pubescent inside, margins flat; petals free, outer petals longer than inner, inner petals entirely covered in bud; outer petals 3, 1.2–2.3 mm long, 2–3 mm wide, broadly ovate, apex obtuse, base truncate, margins flat, densely pubescent outside, pubescent with short papillose hairs inside; inner petals 3, valvate, 0.5–0.7 mm long, 0.3–0.5 mm wide, elliptic, apex obtuse, base truncate, margins flat, pubescent outside, pubescent inside; stamens 6, in 1 row but grouped by 2, ca. 1 mm long, oblong; connective truncate, pubescent; staminodes 12(to 13), in one whorl externally to the stamens, very short 0.2 mm long, sparsely pubescent; female flowers: pedicel 6–20 mm long, ca. 1 mm in diameter, densely pubescent; in fruit 30–40 mm long, 1–2 mm in diameter; basal bract 1–1.5 mm long, ca. 1 mm wide; upper bract ca. 1 mm long, ca. 1 mm wide; sepals 3, valvate, basally fused, ca. 1 mm long, ca. 1 mm wide, ovate to lanceolate, apex acuminate, base truncate, densely pubescent outside, densely pubescent inside, margins flat; petals free, outer petals longer than inner, inner petals entirely covered in bud; **outer petals 3, 3–3.5 mm long, 3.5–4.0(5.0) mm wide**, broadly ovate, apex obtuse, base truncate, margins flat, densely pubescent outside, pubescent with short papillose hairs inside; **inner petals 3, valvate, 1.3–1.4 mm long, 0.5–0.6 mm wide**, elliptic, apex obtuse, base truncate, margins flat, pubescent outside, pubescent inside;, ca. 1 mm long, stigma elongate, glabrous, **carpels free, 48 to 60(76)**, ovary 1–1.5 mm long, densely hairy. Monocarps stipitate, stipes 6–12 mm long, 2–3 mm in diameter; monocarps ca. 8, 15–57 mm long, 6–9 mm in diameter, moniliform, ellipsoid, apex apiculate, pubescent, smooth to verrucose, constricted around seeds when more than 1, color unknown; seeds 1 to 4 per monocarp, 12–15 mm long, 5–8 mm in diameter, ellipsoid; aril absent.

#### Distribution.

A central African species, from Nigeria to Gabon and in Equatorial Guinea; in Cameroon known from the Central, Littoral, South and South-West regions.

#### Habitat.

A fairly common species; in primary rain forest, old secondary forest and gallery forests. Altitude 0–1000 m a.s.l.

#### Local and common names known in Cameroon.

None recorded.

#### Preliminary IUCN conservation status.

Vulnerable (VU) (Hoekstra et al. 2021).

#### Uses in Cameroon.

None recorded.

#### Notes.

﻿﻿﻿*Monanthotaxiscauliflora* is distinguished by its very short pubescence on the young foliate branches and underside of the leaves, cauliflorous inflorescences and smallish female flowers with 48 to 60 (76) carpels. Some specimens of ﻿*M.cauliflora* have a slightly longer pubescence, resembling ﻿*M.diclina*, but their hairs are shorter and ascending, not erect as in ﻿*M.diclina*; both species of which can be found in sympatry in southern Cameroon (Hoekstra et al. 2021).

The collection *Cheek 11657* was tentatively identified as *M.angustifolia* (Exell) Verdc. (now synonym of ﻿*M.vogelii*) in the checklist of the plants of Méfou (Cheek et al. 2011, p. 123), but has now been redetermined as ﻿*M.cauliflora* (Hoekstra et al. 2021).

#### Specimens examined.

**Central Region**: Ndanan I to Ndangan I, 3.62°N, 11.58°E, *10 March 2004*, *Cheek M.* 11657 (K,YA). **Littoral Region**: Douala-Edea Reserve Lake Tissongo study area 3.78°N, 10.04°E, *01 June 1976*, *Waterman P.G.* 840 (E,K). **South Region**: Kribi-Lolodorf, 3.17°N, 10.48°E, *05 March 1969*, *Bos J.J.* 4069 (BR,K,P,WAG,YA); 11 km from Kribi Ebolowa road, 2.88°N, 9.983°E, *02 January 1970*, *Bos J.J.* 6037 (BR,C,K,LD,P,P,WAG,YA); 20 km From Kribi 5 km N of Lolodorf road, 3.05°N, 10.05°E, *09 February 1970*, *Bos J.J.* 6293 (BR,P,WAG); Mt Elephant SE of Kribi, 2.81°N, 10.01°E, *28 April 1970*, *Bos J.J.* 6867 (BR,P,WAG); Campo Ma’an National Park 11 km on trail from Ebinanemeyong village on road 7 km from Nyabessan to Campo town, 2.48°N, 10.33°E, *11 February 2015*, *Couvreur T.L.P.* 676 (WAG,YA); Campo Ma’an National Park 11 km on trail from Ebinanemeyong village on road 7 km from Nyabessan to Campo town, 2.48°N, 10.33°E, *13 February 2015*, *Couvreur T.L.P.* 705 (WAG,YA); C. 15 km South of Ebolowa, 2.49, 11.10, *28 February 1964*, *de Wilde J.J.F.E* 1987 (YA,WAG); Ca 15 km S of Ebolowa, 2.83°N, 11.16°E, *28 February 1964*, *de Wilde W.J.J.O* 1987 (BR,P,WAG,YA); Aloum, 2.76°N, 10.69°E, *13 December 2013*, *Kamdem N.* 164 (YA); Près de Nsola (20 km N Bipindi), 3.16°N, 10.38°E, *29 January 1974*, *Letouzey R.* 12811 (K,P,P,YA); Bipindi, 3.08°N, 10.41°E, *01 January 1913*, *Zenker G.A.* 356 (M,P,U,WAG); Bipindi, 3.05°N, 10.25°E, *01 January 1909*, *Zenker G.A.* 3898 (BR,E,K). **South-West Region**: Kupe village, 4.76°N, 9.691°E, *08 November 1995*, *Cheek M.* 7711 (K,MO,WAG,YA); Summit of Mt Loh, 5°N, 9.683°E, *17 November 1998*, *Gosline W.G.* 175 (K,YA); Korup National Park, 5.01°N, 8.833°E, *01 January 1985*, *Thomas D.W.* 4324 (K,MO,P,YA).

### ﻿﻿﻿﻿ Monanthotaxis congoensis

Taxon classificationPlantaeMagnolialesAnnonaceae

Baill., Bull. Soc. Linn, Paris 2: 879, 1890

6C2DF5F3-F689-585C-B31E-42DA8F8FD897

**Figure 50.**
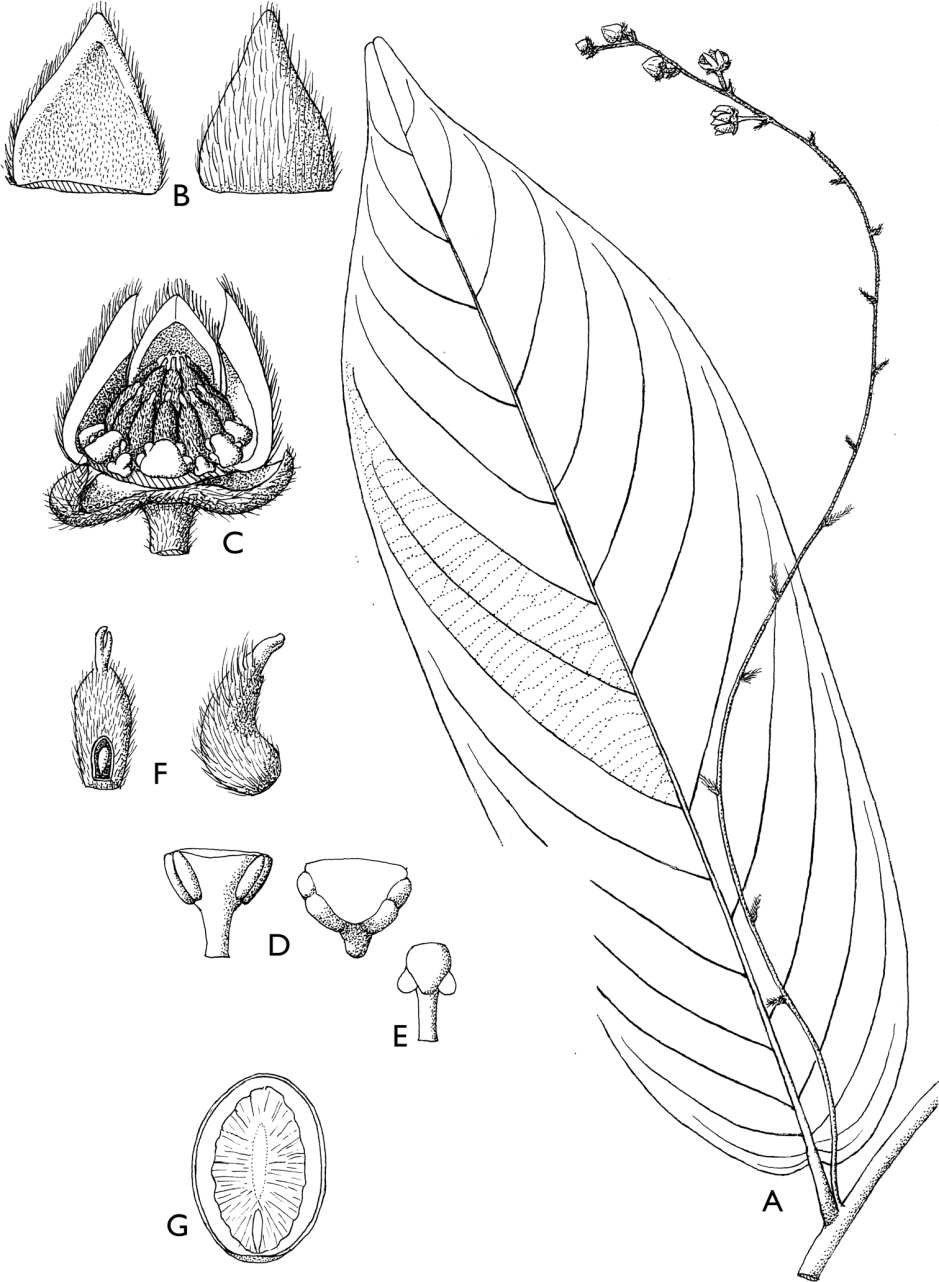
*Monanthotaxiscongoensis***A** flowering branch **B** petal inner and outer side view **C** flower with three petals removed **D** stamen front view and seen from above **E** staminodes **F** carpel and longitudinal section of carpel **G** longitudinal section of seed **A–G** from *Thollon 813*. Drawings by Hélène Lamourdedieu, Publications Scientifiques du Muséum national d’Histoire naturelle, Paris; modified from Le Thomas (1969b, pl. 45, p. 257).

Figs 49 , 50 ; Map 6H

#### Type.

Gabon. no region; Ogooué, *Thollon F.-R. 813*, Jul 1887: lectotype, chosen by Le Thomas (1969b), p. 258: P[P00362762, P00362763, P00362766]; isolectotypes: K[K000198992]; MA[MA630761, MA698356]; WAG[WAG0003586, WAG0003587].

#### Description.

Shrub to liana, 2–5 m tall, d.b.h. unknown. Indumentum of simple hairs; old leafless branches glabrescent, young foliate branches densely pubescent with appressed pale brown hairs 0.7–1.2 mm long. Leaves: petiole 3–ca. 8 mm long, ca. 1 mm in diameter, densely pubescent, grooved, blade inserted on top of the petiole; blade 7.5–25 cm long, 1.9–6 cm wide, oblong to oblanceolate or elliptic, apex acuminate to acute, acumen ca. 1.5 cm long, base cuneate to cordate, papyraceous, **below densely pubescent with appressed silky white hairs when young**, sparsely pubescent when old, above sparsely pubescent to glabrous when young, glabrous when old, discolorous, whitish below; midrib sunken or flat, above densely pubescent when young and old, below densely pubescent when young and old; secondary veins 9 to 16 pairs, pubescent above; tertiary venation percurrent. Individuals bisexual; inflorescences ramiflorous on old leafless branches, terminal or leaf opposed. Flowers with 9 perianth parts in 2 whorls, 4 to 10 per inflorescence, **in 6–13 cm long racemes**; pedicel up to ca. 12 mm long, ca. 1 mm in diameter, sparsely pubescent to densely pubescent; in fruit same as in flower; basal bract 2–3 mm long, ca. 1 mm wide; upper bract ca. 1 mm long, ca. 1 mm wide; sepals 3, valvate, basally fused, 1–3 mm long, 1–2 mm wide, triangular to ovate, apex acute, base truncate, densely pubescent outside, pubescent inside, margins flat; **petals free, 6, in one whorl**, 3–4 mm long, 1–2 mm wide, elliptic to ovate, apex obtuse, base truncate, green to light yellow, margins flat, densely pubescent outside, pubescent inside; stamens 6, in 1 row, opposite with the petals, ca. 1 mm long, obconic; connective truncate, glabrous; staminodes 6, alternating with stamens, ca. 1 mm long; carpels free, 15 to 21, ovary ca. 1 mm long, stigma bilobed, glabrous. Monocarps stipitate, stipes 2–3 mm long, 2–3 mm in diameter; monocarps up to 4, 12–15 mm long, 9–10 mm in diameter, **ellipsoid to subglobose**, apex rounded, densely pubescent, smooth, dull orange to red when ripe; **seed 1 per monocarp**; 8–9 mm long, 7–8 mm in diameter, ellipsoid; aril absent.

#### Distribution.

A central African species, in Cameroon and Gabon; in Cameroon known from the Littoral, South and South-West regions.

#### Habitat.

A uncommon species when present, in primary and old secondary rain forest, periodically inundated rain forests, gallery forests or forest edges, on rocky soil. Altitude 50–700 m a.s.l.

#### Local and common names known in Cameroon.

None recorded.

#### Preliminary IUCN conservation status.

Least Concern (LC) (Hoekstra et al. 2021).

#### Uses in Cameroon.

None recorded.

#### Notes.

﻿﻿﻿*Monanthotaxiscongoensis* is distinguished by its densely pubescent lower leaf surface with appressed silky white hairs and its raceme-like inflorescences.

#### Specimens examined.

**Littoral Region**: Ebo Wildlife Reserve Djuma permanent camp On east trail, 4.36°N, 10.25°E, *15 February 2013*, *Couvreur T.L.P.* 626 (WAG,YA). **South-West Region**: Etinde Upper Boando footpath to west of village, 4.05°N, 9.15°E, *06 December 1993*, *Cable S.* 404 (K); Bayang Mbo Wildlife Sanctuary after Mbu river, 5.35°N, 9.501°E, *26 March 2016*, *Couvreur T.L.P.* 1018 (WAG,YA); on trail leading to top of Mt Etinde after Ekonjo village, 4.06°N, 9.151°E, *01 April 2016*, *Couvreur T.L.P.* 1025 (WAG,YA); Mokoko Forest Reserve Dikome, 4.48°N, 9.033°E, *05 May 1994*, *Ekema S.N.* 939 (K,YA); Banyang-mbo Sanctuary, 5.35°N, 9.541°E, *02 December 2000*, *Gosline W.G.* 300 (K); district Buea Upper Boando, 4.03°N, 9.106°E, *27 March 1992*, *Kwangue A.T.* 22 (K,P,YA).

### ﻿﻿﻿﻿ Monanthotaxis couvreurii

Taxon classificationPlantaeMagnolialesAnnonaceae

P.H.Hoekstra, PhytoKeys 69: 79, 2016

C2595AA3-7C43-5BD9-9CAB-0C72527A9C7E

Fig. 51 ; Map 6I

#### Type.

Cameroon. Central Region; Ottotomo Forest Reserve, *Couvreur T.L.P. 762*, 24 Apr 2015: holotype: WAG[WAG.1576998, WAG.1576999, WAG.1577000]; isotypes: MPU[MPU1374962]; YA *n.v*.

**Figure 51.**
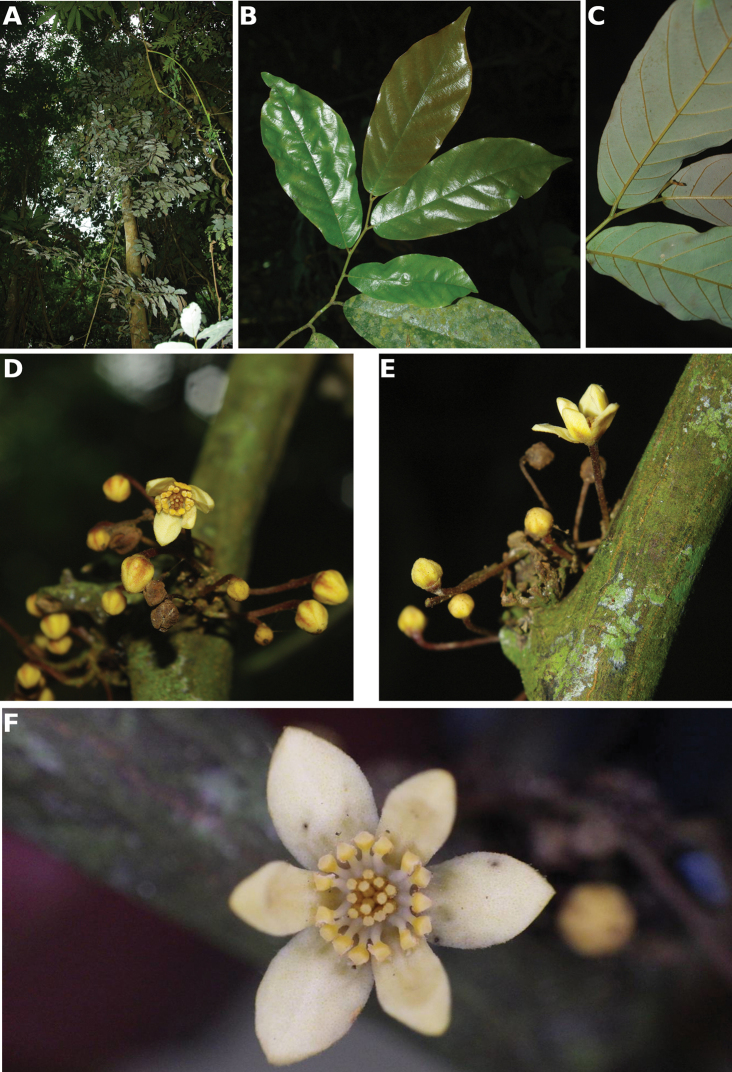
*Monanthotaxiscouvreurii***A** habit, liana growing on tree **B** leaves, upper side **C** base of leaf blade, lower side **D** cauliflorous inflorescence, one flower opened **E** cauliflorous inflorescence, side view **F** detail of flower top view, note the 15 stamens fused at their base forming a ring around the carpels in the center **A–F***Couvreur 762*, Ottotomo, Cameroon. Photos Thomas L.P. Couvreur.

#### Description.

Liana, up to 20 m tall, d.b.h. up to 4 cm. Indumentum of simple hairs; old leafless branches glabrous, young foliate branches densely pubescent with dense ascending reddish brown hairs 0.1–0.2 mm long. Leaves: petiole 3–5 mm long, ca. 1 mm in diameter, densely pubescent, slightly grooved, blade inserted on top of the petiole; blade 4.5–12 cm long, 1.5–4.5 cm wide, oblong to obovate, apex acuminate to acute, acumen ca. 1 cm long, base cuneate to rounded, papyraceous, below sparsely pubescent when young and old, above sparsely pubescent when young, glabrous when old, discolorous, whitish below; midrib sunken or flat, above glabrous when young and old, below pubescent when young and old; secondary veins 7 to 11 pairs, glabrous above; tertiary venation percurrent. Individuals bisexual; **inflorescences cauliflorous** or more rarely ramiflorous on old leafless branches, axillary. Flowers with 9 perianth parts in 3 whorls, 1 to 2(3) per inflorescence when on branches, up to 20 per inflorescence when on main trunk; pedicel 4–20 mm long, ca. 1 mm in diameter, sparsely pubescent; in fruit unknown; basal bract not seen, upper bract minute, ca. 0.5 mm, ca. 1 mm wide; sepals 3, valvate, basally fused, ca. 1 mm long, 1 mm wide, triangular, apex acute, base truncate, brown, densely pubescent outside, glabrous inside, margins flat; petals free, subequal, **inner petals partly covered in bud**; outer petals 3, 3.5–5 mm long, 2–3.5 mm wide, elliptic to ovate, apex obtuse, base truncate, light yellow to white, margins flat, densely pubescent outside, sparsely pubescent inside; inner petals 3, valvate, 3–4.5 mm long, 1.2–1.5 mm wide, elliptic to ovate, apex acute, base truncate, light yellow to white, margins flat, pubescent outside, glabrous inside; **stamens 13 to 15, in 1 row, basally fused between each them**, ca. 1 mm long, linear; connective truncate to rounded, pubescent, cream; staminodes absent; carpels free, 9 to 12, ovary ca. 1 mm long, stigma globose, glabrous. Fruits unknown.

#### Distribution.

endemic to Cameroon; known from the Central Region.

#### Habitat.

A rare species, in lowland old secondary rain forests. Attitude around 700 m a.s.l.

#### Local and common names known in Cameroon.

None recorded.

#### Preliminary IUCN conservation status.

Critically Endangered (CR) (Hoekstra et al. 2021).

#### Uses in Cameroon.

None recorded.

#### Notes.

﻿﻿﻿*Monanthotaxiscouvreurii* is distinguished by its mainly cauliflorous flowers (but may also be ramiflorous) and its stamens in one row that are basally fused, a unique feature in ﻿*Monanthotaxis* (Hoekstra et al. 2016). This species is only known from the Ottotomo Forest reserve, near Yaoundé.

#### Specimens examined.

**Central Region**: Ottotomo Forest Reserve 45 km South of Yaoundé ca 5 km on main path into reserve, 3.65°N, 11.28°E, *24 April 2015*, *Couvreur T.L.P.* 762 (WAG,YA); Reserve Forestière d’Ottotomo 40 km de Yaoundé sur la route de Kribi, 3.64°N, 11.27°E, *05 May 1970*, *Farron C.* 7266 (P); Reserve forestière d’Ottotomo Yaoundé à environ 40 km au SW sur la route de Makak, 3.64°N, 11.27°E, *26 May 1970*, *Farron C.* 7359 (YA).

### ﻿﻿﻿﻿ Monanthotaxis diclina

Taxon classificationPlantaeMagnolialesAnnonaceae

(Sprague) Verdc., Kew Bull. 25(1): 31, 1971

F1B73DF3-D4AD-5EB0-B69A-59D863DF92EA

**Map 7.**
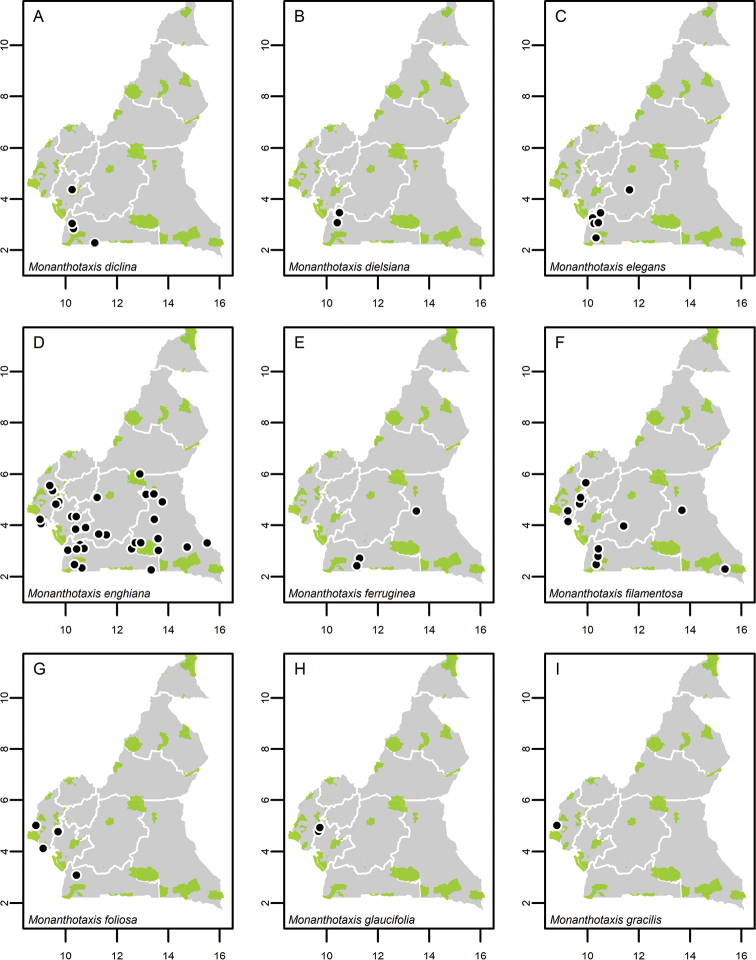
**A***Monanthotaxisdiclina***B***Monanthotaxisdielsiana***C***Monanthotaxiselegans***D***Monanthotaxisenghiana***E***Monanthotaxisferruginea***F***Monanthotaxisfilamentosa***G***Monanthotaxisfoliosa***H***Monanthotaxisglaucifolia***I***Monanthotaxisgracilis*. White borders represent region limits in Cameroon; green patches represent protected areas (see methods and Suppl. material 1: Fig. S1).

**Figure 52.**
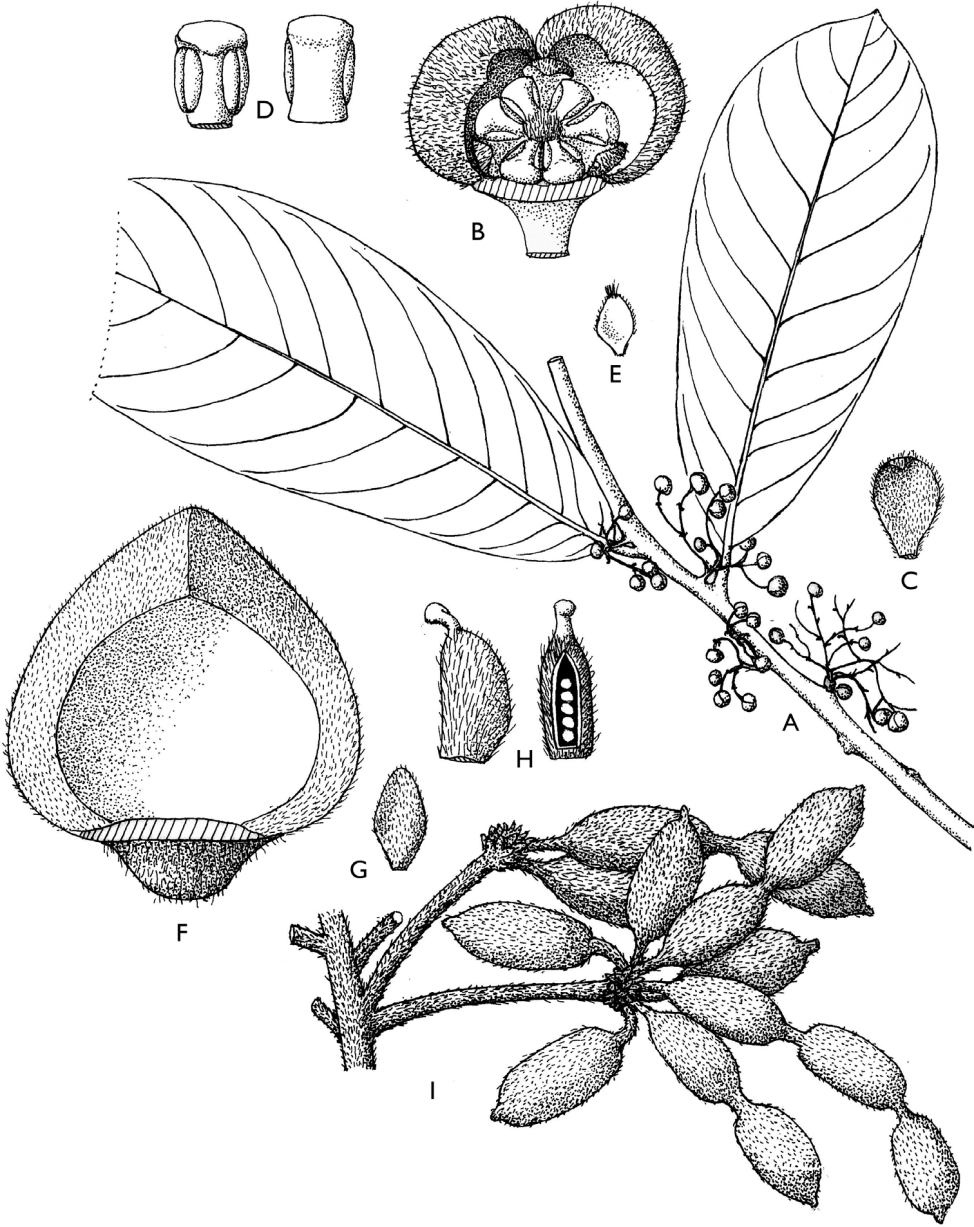
*Monanthotaxisdiclina***A** male flowering branch **B** male flower with one outer petal removed **C** male flower inner petal inner side view **D** stamen, outer and inner side view **E** male flower staminode **F** female flower, outer petal inner side view **G** female flower inner petal, inner side view **H** carpel and longitudinal section of carpel **I** fruits **A–E** from *Klaine 2881***F–H** from *Klaine 1382***I** from *Klaine 404*. Drawings by Hélène Lamourdedieu, Publications Scientifiques du Muséum national d’Histoire naturelle, Paris; modified from Le Thomas (1969b, pl. 38, p. 213).

**Figure 53.**
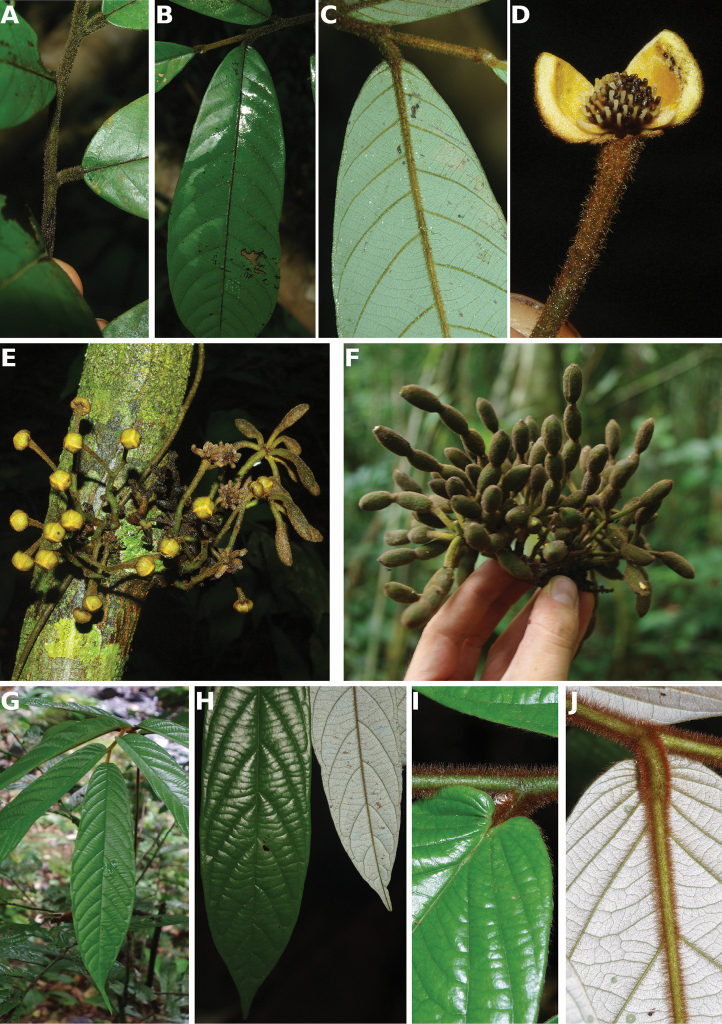
*Monanthotaxisdiclina***A** detail of base of leaf blades, upper view **B** leaf, upper view **C** leaf, lower view **D** detail of female flower receptacle, note the two small inner petals (yellow) at base of carpels, the larger petals are the outer ones (one removed) **E** cauliflorous inflorescences with female flowers and fruits **F** fruits, moniliform in shape. *Monanthotaxisenghiana***G** leaves, top view **H** leaves, upper and lower views, note whitish lower side of leaves **I** detail of base of leaf base with erect pubescence, upper view **J** detail of base of leaf base with erect pubescence, lower view **A–F***Couvreur 537*, Gabon **G–J***Couvreur 1121*, Gabon. Photos Thomas L.P. Couvreur.

Figs 52 , 53 ; Map 7A

≡ Popowia diclina Sprague, Bull. Misc. Inform. Kew 2: 53 1908, *pro parte*, *quoad specim*. ♂; ﻿Popowiadiclina Sprague *emend.* Chipp, Bull. Misc. Inform. Kew, 5: 182, 1923; ﻿﻿Clathrospermummannii Oliv., Fl. Trop. Afr 1: 25, 1868 *pro parte*, *quoad specim*. ♂; ﻿Popowiamannii (Oliv.) Engl. & Diels, Monogr. Afrik. Pflanzen.-Fam. 6: 49, *pro parte*, *quoad specim*. ♂ *non* Baill. Adansonia 8: 320, 1868.
= Popowia caulantha Exell, J. Bot. 70(Suppl. 1): 208, 1932. Type. Angola. Cabinda, BucoZau-Maiombe, *Gossweiler J. 6721*, 29 Sep 1916: holotype: BM[BM000553848]; isotypes: COI[COI00004904]; LISC[LISC000104, LISC000264, LISC000265, LISC000266]; LISU.

#### Type.

Gabon. Estuaire; Gaboon River, *Mann G. 960*, Jul 1861: lectotype, chosen by Le Thomas (1969b), p. 216: K[K000198989].

#### Description.

Liana, 25 m tall, d.b.h. unknown. Indumentum of simple hairs; old leafless branches glabrous, young foliate branches densely pubescent **with dense pubescence of erect to curling yellow-brown hairs 0.4–0.6 mm long**. Leaves: petiole 3–5 mm long, ca. 2 mm in diameter, densely pubescent, weakly grooved adaxially, blade inserted on top of the petiole; blade 8.5–18.5 cm long, 3.5–7 cm wide, oblong to obovate, apex acute to rounded, base rounded to subcordate, papyraceous to subcoriaceous, below densely pubescent when young and old with erect yellow hairs, above sparsely pubescent when young and old, discolorous, whitish below; midrib depressed, above densely pubescent when young and old, below densely pubescent when young and old; secondary veins 15 to 19 pairs, glabrous above; tertiary venation percurrent**. Individuals unisexual, monoecious, dimorphic, male inflorescences ramiflorous and axillary, female ones cauliflorous.** Flowers with 9 perianth parts in 3 whorls, **male inflorescence with a solitary to a few-flowered fascicle, sometimes a rhipidium with up to 25 flowers per inflorescence, female a many-flowered panicle-like rhipidia; male flowers: pedicel 2–5 mm long, ca. 0.5 mm in diameter**, densely pubescent; basal bract ca. 0.5 mm long, 0.5 mm wide; upper bract ca.1 mm long, ca. 0.5 mm wide; sepals 3, valvate, shortly basally fused, ca. 0.5 mm long, ca. 0.5 mm wide, ovate, apex obtuse, base truncate, densely pubescent outside, densely pubescent inside, margins flat; petals free, outer petals longer than inner, inner petals entirely covered in bud; outer petals 3, ca. 1.6 mm long, ca. 2 mm wide, broadly ovate, apex obtuse, base truncate, yellow, margins flat, densely pubescent outside, pubescent inside; inner petals 3, valvate, 0.5–0.8 mm long, 0.3–0.4 mm wide, broadly elliptic, apex rounded, base truncate, margins flat, pubescent outside, pubescent inside; stamens 6, in 1 row, ca. 1 mm long, oblong; connective truncate, pubescent; staminodes 12, in one whorl externally to the stamens, 0.2–0.3 mm long, sparsely pubescent. **Female flowers: pedicel 15–25 mm long, ca. ca. 1 mm in diameter**, densely pubescent with erect yellowish brown hairs; in fruit 15–45 mm long, 1–2 mm in diameter; bracts 2, one basal and one upper towards the lower half of pedicel, basal bract not seen; upper bract 1–2 mm long, ca. 1 mm wide; sepals 3, valvate, basally fused, 1.2–1.7 mm long, 1.3–1.4 mm wide, broadly ovate to broadly elliptic, apex acuminate, base truncate, densely pubescent outside, densely pubescent inside, margins flat; petals free, outer petals longer than inner, inner petals entirely covered in bud; outer petals 3, 3–5 mm long, 3.5–5.4 mm wide, broadly ovate to circular, apex obtuse or rounded, base truncate, margins flat, densely pubescent with brown hairs outside, pubescent with very short yellowish hairs inside; inner petals 3, valvate, 1.3–1.7 mm long, 0.6–0.7 mm wide, elliptic, apex obtuse, base truncate, margins flat, pubescent with very short yellowish hairs outside, pubescent with very short yellowish hairs inside; carpels free, 80 to 100, ovary 1–2 mm long, stigma elongate, glabrous. Monocarps stipitate, stipes 6–14 mm long, 2–3 mm in diameter; monocarps up to 14, 23–55 mm long, 7–8 mm in diameter, moniliform, ellipsoid, apex apiculate, pubescent, verrucose, constricted around seeds when more than 1, green when ripe; seeds 1 to 5 per monocarp, 12–15 mm long, 6–9 mm in diameter, ellipsoid; aril absent.

#### Distribution.

A central African species, from Cameroon to the Republic of the Congo; in Cameroon known from the Littoral and South regions.

#### Habitat.

An uncommon species; in primary or old secondary rain forests, swamp and gallery forests. Altitude 50–300 m a.s.l.

#### Local and common names known in Cameroon.

None recorded.

#### Preliminary IUCN conservation status.

Least Concern (LC) (Hoekstra et al. 2021).

#### Uses in Cameroon.

***cosmestics***: seeds(?) for essential oils and exudates

#### Notes.

﻿﻿﻿*Monanthotaxisdiclina* is distinguished by its dense pubescence of ascending to erect yellowish brown hairs to 0.5 mm long on the young foliate branches and lower side of the leaves, and its unisexual flowers with the female ones being cauliflorous and the male ones ramiflorous.

#### Specimens examined.

**Littoral Region**: Ebo Wildlife Reserve Djuma permanent camp On east trail, 4.36°N, 10.25°E, *15 February 2013*, *Couvreur T.L.P.* 629 (WAG,YA). **South Region**: 27 km on an exploitation track leading from Ipono towards Dipikar island Bongala river, 2.28°N, 11.13°E, *26 June 1975*, *de Wilde J.J.F.E* 8330 (BR,MO,P,WAG,YA); Besou 45 km E of Gross Batanga, 2.85°N, 10.3°E, *22 July 1911*, *Mildbraed G.W.J.* 6059 (HBG); Bipindi, 3.05°N, 10.25°E, *June 1899*, *Zenker G.A.* 2102 (B,G,K).

### ﻿﻿﻿﻿ Monanthotaxis dielsiana

Taxon classificationPlantaeMagnolialesAnnonaceae

(Engl.) P.H.Hoekstra, Taxon 66: 14, 2017

DD18BBB0-1AEB-52D2-9C06-FC0CDF7E4460

Map 7B

≡ Unona dielsiana Engl., Bot. Jahrb. Syst. 39(3–4): 476, 1907. ﻿Oxymitradielsiana (Engl.) Sprague & Hutch.Bull. Misc. Inform. Kew 6: 156, 1916; ﻿Richelladielsiana (Engl.) R.E.Fr., in Engler & Prantl Nat. Pflanzenfam., ed. 2, 17a (2): 139, 1959; ﻿﻿Friesodielsiadielsiana (Engl.) Steenis, Blumea 12: 359, 1964.

#### Type.

Cameroon. South Region; Bipindi, *Zenker G.A. 2473*, Dec 1901: lectotype, chosen by Hoekstra et al. (2021), p. 147: B[B100154098]; isolectotypes: B[B100154096, B100154097, B100154099]; BM[BM001125043]; BR[BR000008801388]; COI[COI00071518]; E; G[G00308364]; GOET[GOET005688, GOET005689]; HBG[HBG502481]; K[K000198948]; KFTA[KFTA 0001554, KFTA 0001555]; L[L 0182291]; M[M-0240178]; P[P00363342, P00363343, P01988326]; S[S07-13404]; WAG[WAG0057970]; WU[WU 0025876].

#### Description.

Liana, height unknown, d.b.h. unknown. Indumentum of simple hairs; old leafless branches glabrescent, **young foliate branches densely pubescent with orange-brown hairs ca. 0.5 mm long**. Leaves: petiole 6–11 mm long, ca. 2 mm in diameter, densely pubescent, grooved, blade inserted on top of the petiole; blade 10.5–21.5 cm long, 3.5–4 cm wide, narrowly oblong to oblanceolate, apex acuminate, acumen 0.5–2.5 cm long, base subcordate, subcoriaceous to membranous, below glabrous when young and old, above glabrous when young and old, discolorous, whitish below; midrib impressed, above sparsely pubescent when young and old, below sparsely pubescent when young and old; secondary veins 7 to 14 pairs, glabrous above; tertiary venation percurrent. Individuals bisexual; inflorescences ramiflorous on old leafless branches, leaf opposed. Flowers with 9 perianth parts in 3 whorls, 1 to 3 per inflorescence, pedicel 15–27 mm long, 1–2 mm in diameter, pubescent; in fruit 15–30 mm long, ca. 3 mm in diameter, pubescent; basal bract ca. 4 mm long, ca. 4 mm wide; upper bract ca. 5 mm long, ca. 4 mm wide; sepals 3, valvate, free, ca. 4 mm long, ca. 4 mm wide, triangular, apex acute, base truncate, densely pubescent outside, glabrous inside, margins flat; petals free, outer petals longer than inner, inner petals entirely covered in bud; outer petals 3, 10–15 mm long, 8–9 mm wide, ovate, apex obtuse, base truncate, margins flat, densely pubescent outside, glabrous inside; inner petals 3, valvate, ca. 8 mm long, ca. 8 mm wide, broadly ovate, apex obtuse, base truncate, margins flat, pubescent outside, glabrous inside; **stamens ca. 65, in 3 rows**, ca. 0.5 mm long, cylindrical; connective rounded, glabrous; staminodes absent; carpels free, ca. 41, ovary ca. 2 mm long, stigma globose, glabrous. Monocarps stipitate, stipes ca. 3 mm long, ca. 2 mm in diameter; monocarps ca. 6, ca. 45 mm long, 10–12 mm in diameter, moniliform, ellipsoid to cylindrical, apex rounded, densely pubescent, rugulose, constricted around seeds when more than 1, brown when unripe; seeds 1 to 2 per monocarp, ca. 15 mm long, ca. 9 mm in diameter, subcylindrical; aril absent.

#### Distribution.

endemic to Cameroon; known from the Central and South regions.

#### Habitat.

A rare species, known from two collections; in primary rain forests. Altitude 0–200 m a.s.l.

#### Local and common names known in Cameroon.

None recorded.

#### Preliminary IUCN conservation status.

Critically Endangered (CR) (Hoekstra et al. 2021).

#### Uses in Cameroon.

None reported.

#### Notes.

﻿﻿﻿*Monanthotaxisdielsiana* is distinguished by its dense yellow to orange-brown appressed pubescence on the young foliate branches, and flowers with around 65 stamens. It resembles ﻿*M.enghiana* and ﻿*M.glaucifolia* by the overall morphology of the flowers and leaves, but ﻿*M.enghiana* has longer erect hairs and ﻿*M.glaucifolia* has denser light-brown appressed hairs; in addition, ﻿*M.enghiana* and ﻿*M.glaucifolia* have many more stamens (more than 90).

#### Specimens examined.

**Central Region**: 60 km SW of Eséka S of Nyong R 12 km W of Songbong, 3.47°N, 10.5°E, *10 March 1965*, *Leeuwenberg A.J.M.* 5088 (BR,K,MO,P,WAG,YA). **South Region**: Bipindi, 3.08°N, 10.41°E, *01 January 1902*, *Zenker G.A.* 2473 (L,P,WAG).

### ﻿﻿﻿﻿ Monanthotaxis elegans

Taxon classificationPlantaeMagnolialesAnnonaceae

(Engl. & Diels) Verdc., Kew Bull. 25(1): 25, 1971

D527C086-04DE-58EA-989A-2D31A091EE1D

Fig. 49 ; Map 7C

≡ Popowia elegans Engl. & Diels, Monogr. Afrik. Pflanzen.-Fam. 6: 45, 1901.

#### Type.

Cameroon. South Region; Bipindi, *Zenker G.A. 1321*, 23 Mar 1897: holotype: B[B100153023]; isotypes: BM[BM000553832, BM000553833]; E[E00181437]; G[G00308366]; HBG[HBG-502506]; K[K000198990, K000198991]; M[M-0198713]; P[P00362605, P00362606].

#### Description.

Liana, up to 4 m tall, d.b.h. unknown. Indumentum of simple hairs; old leafless branches glabrous, young foliate branches densely pubescent with dense appressed to ascending reddish brown hairs 0.1–0.2 mm long. Leaves: petiole 3–5 mm long, ca. 1 mm in diameter, densely pubescent, slightly grooved, blade inserted on top of the petiole; blade 4.6–15.4 cm long, 2.3–4.6 cm wide, **narrowly oblong-elliptic to oblong-oblanceolate (to obovate)**, apex acute to acuminate, acumen ca. 3 cm long, base rounded to subcordate, papyraceous, below sparsely pubescent when young and old, above sparsely pubescent when young and old, discolorous, whitish below; midrib impressed, above glabrous when young and old, below sparsely pubescent when young and old; secondary veins 11 to 14 pairs, glabrous above; tertiary venation percurrent. Individuals bisexual; **inflorescences ramiflorous on foliate branches, leaf opposed to extra axillary**. Flowers with 9 perianth parts in 3 whorls, **1 to 8 per inflorescence, glomerule-like**; pedicel 1–2 mm long, ca. 1 mm in diameter, **densely pubescent with appressed yellowish brown hairs**; in fruit 4–5 mm long, ca. 1 mm in diameter; basal bract ca. 1 mm long, ca. 0.5 mm wide; upper bract ca.1 mm long, ca. 0.5 mm wide; sepals 3, valvate, free, ca. 1 mm long, 1–2 mm wide, triangular to ovate, apex acute, base truncate, densely pubescent outside, glabrous inside, margins flat; petals free, outer petals longer than inner, inner petals entirely covered in bud; outer petals 3, 2.6–4.3 mm long, 2.6–3.5 mm wide, ovate, apex acute, base truncate, yellow to cream, margins flat, densely pubescent outside, glabrous, pubescent towards margins inside; inner petals 3, valvate, ca. 3.5 mm long, ca. 2 mm wide, rhombic, apex acute, base truncate, yellow to cream, margins flat, pubescent towards base outside, pubescent inside; stamens 9, in 1 row, ca. 1 mm long, linear to oblong; connective truncate, glabrous; staminodes 6, alternating with the stamens, minute, glabrous; carpels free, 12 to 20, ovary ca. 1 mm long, stigma globose, glabrous. Monocarps stipitate, stipes ca. 2 mm long, ca. 2 mm in diameter; monocarps 2 to 8, 9–16 mm long, 5–6 mm in diameter, ellipsoid, apex apiculate, glabrous, verrucose, constricted around seeds when more than 1, color unknown; seeds 1 to 2 per monocarp, 7–9 mm long, 5–6 mm in diameter, ellipsoid; aril absent.

#### Distribution.

endemic to Cameroon, known from the Central and South regions.

#### Habitat.

In primary lowland rain forests and gallery forests, open rocky spot on sandy soil. Altitude 50–500 m a.s.l.

#### Local and common names known in Cameroon.

None recorded.

#### Preliminary IUCN conservation status.

Endangered (EN) (Hoekstra et al. 2021).

#### Uses in Cameroon.

None reported.

#### Notes.

﻿﻿﻿*Monanthotaxiselegans* is generally distinguished by its oblong-lanceolate leaves and extra-axillary or terminal glomerule-like inflorescences with yellow-brown pubescence.

The specimen *Annet 348* [P01960095] has obovate instead of oblong-lanceolate leaves, but the inflorescence and flowers are typical for ﻿*M.elegans*.

#### Specimens examined.

**Central Region**: Riverine forest bank Nyong river near the new bridge ca 65 km SSW of Eseka, 3.46°N, 10.5°E, *16 June 1964*, *de Wilde W.J.J.O* 2709 (B,BR,K,MO,P,WAG,YA); Natchigal ca 20 km N of Obala, 4.35°N, 11.63°E, *01 July 1964*, *de Wilde W.J.J.O* 2787 (B,BR,K,MO,P,WAG,YA). **South Region**: Bipinde, 3.26°N, 10.20°E, *20 June 1918*, *Annet E.* 348 (BR,K,P); Campo Ma’an National Park 11 km on trail from Ebinanemeyong village on road 7 km from Nyabessan to Campo town, 2.48°N, 10.33°E, *13 February 2015*, *Couvreur T.L.P.* 704 (WAG,YA); Bipindi, 3.08°N, 10.41°E, *1897*, *Zenker G.A.* 132 (L); Bipindi, 3.08°N, 10.41°E, *1897*, *Zenker G.A.* 1321 (E,M,P); Lokoundjé, 3.08°N, 10.41°E, *01 January 1913*, *Zenker G.A.* 199 (U,WAG); Bipindi, 3.08°N, 10.41°E, *01 January 1904*, *Zenker G.A.* 2693 (E,L,M,P,WAG); Bipindi, 3.08°N, 10.41°E, *01 January 1911*, *Zenker G.A.* 4000 (E,L,M,P); Bipindi, 3.08°N, 10.41°E, *01 January 1912*, *Zenker G.A.* 4477 (E,L,M); Bipindi, 3.05°N, 10.25°E, *Zenker G.A.* s.n. (P).

### ﻿﻿﻿﻿ Monanthotaxis enghiana

Taxon classificationPlantaeMagnolialesAnnonaceae

(Diels) P.H.Hoekstra, Taxon 66: 14, 2017

3C4E99C1-3792-5B31-A0A1-EA7266949039

**Figure 54.**
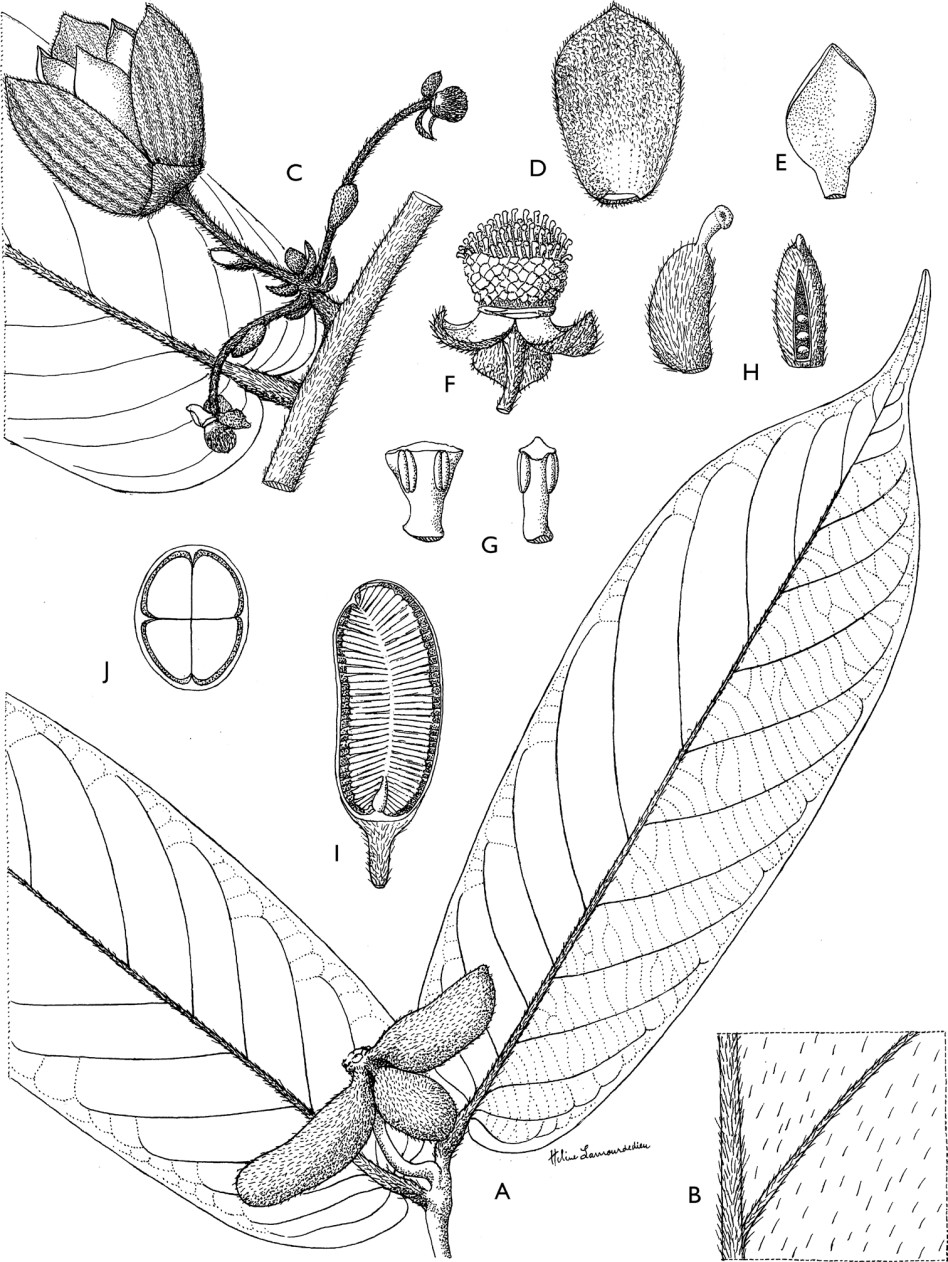
*Monanthotaxisenghiana***A** fruiting branch **B** detail leaf, lower side **C** inflorescence **D** outer petal, inner side **E** inner petal, sinner side **F** flower with petals removed **G** stamen front and side view **H** carpel and longitudinal section of carpel **I** longitudinal section of carpel **J** transverse section of carpel **A, I, J** from *Sillans 1701***A–H** from *Tisserant 1941*. Drawings by Hélène Lamourdedieu, Publications Scientifiques du Muséum national d’Histoire naturelle, Paris; modified from Le Thomas (1969b, pl. 43, p. 241).

Figs 53 , 54 ; Map 7D

≡ Popowia enghiana Diels, Wiss. Ergebn. Deut. Zentr.-Afr. Exped. (1907–1908), Bot. 2: 213, 1911; ﻿﻿Friesodielsiaenghiana (Diels) Verdc. in Le Thomas Fl. Gabon No. 16: 240, 1969.
= ﻿Oxymitra grandiflora Boutique, Bull. Jard. Bot. État Brux. 21: 116, 1951; ﻿Richella grandiflora (Boutique) R.E.Fr., in Engler & Prantl Nat. Pflanzenfam., ed. 2, 17a (2): 139, 1959; Fiesodielsia grandiflora (Boutique) Steenis, Blumea 12: 359, 1964. Type. Democratic Republic of the Congo. Orientale, Yalibutu, 45 km NW of Yangambi, Germain R.G.A. 883, 22 Jan 1948: lectotype, chosen by Hoekstra et al. (2021), p. 150: BR *n.v.*; isolectotypes: K [K000913652, K000913653]; MO *n.v.*
= Unona obanensis Baker f., Cat. Pl. Oban 4, 1913; Oxymitraobanensis (Baker f.) Sprague & Hutch., Bull. Misc. Inform. Kew 6: 156, 1916; Richellaobanensis (Baker f.) R.E.Fr., in Engler & Prantl Nat. Pflanzenfam., ed. 2, 17 a (2): 139, 1959; ﻿Friesodielsiaobanensis (Baker f.) Steenis, Blumea 12 (2): 359, 1964. Type. Nigeria. Cross River State, Oban, Talbot P.A. 1246, 1911: holotype: BM [BM000547069].
= Popowia mangenotii Sillans, Bull. Mus. Natl. Hist. Nat. sér. 2, 24: 578, 1953. Type. Central African Republic: Lobaye, Station de Boukoko, Boukokok, *Tisserant C. (Équipe) 1285*, 14 Dec 1948: lectotype, chosen by Hoekstra et al. (2021), p. 150: P [P00363339]; isolectotypes: BR *n.v.*; K[K000913654]; P[P00363338].
= Popowia mangenotii f. concolor Sillans, Bull. Mus. Natl. Hist. Nat. sér. 2, 24: 580, 1953. Type. Central African Republic, Lobaye, Station de Boukoko, Boukokok, 5 Apr 1951. *C. Tisserant (Équipe) 2062*: lectotype, chosen by Hoekstra et al. (2021), p. 150: P[P00363336]; isolectotypes: BM [BM000547068]; BR *n.v.*, P[P003633385, P01985781].

#### Type.

Type. Democratic Republic of the Congo. Nord Kivu; Fort Beni à Semliki, *Mildbraed G.W.J. 2213*, 1907–1908: holotype: B[B100153056].

#### Description.

Liana, up to 15 m tall or up to canopy, d.b.h. to 6 cm. Indumentum of simple hairs; old leafless branches glabrescent, **young foliate branches densely pubescent with dense erect dark-brown hairs 0.9–1.4 mm long**. Leaves: petiole 3–4 mm long, 1–2 mm in diameter, densely pubescent, slightly grooved, blade inserted on top of the petiole; blade 10.8–35 cm long, 3.3–7.5 cm wide, **narrowly oblong to narrowly oblanceolate**, apex acuminate to acute, acumen up to 5 cm long, base rounded to subcordate, subcoriaceous to membranous, **below whitish blue**, **densely pubescent to pubescent with erect brown hairs when young**, pubescent to glabrous when old, above pubescent when young, sparsely pubescent to glabrous when old, discolorous, whitish below; midrib impressed, above densely pubescent when young and old, below sparsely pubescent when young, glabrous when old; secondary veins 11 to 20 pairs, glabrous above; tertiary venation percurrent. Individuals bisexual; inflorescences ramiflorous on young foliate branches, extra axillary. Flowers with 9 perianth parts in 3 whorls, (1)2 to 5 per inflorescence; pedicel 18–22 mm long, 1–2 mm in diameter, densely pubescent; in fruit 18–22 mm long, 1–2 mm in diameter; basal bract ca. 2 mm long, 2–3 mm wide; upper bract 3–5 mm long, ca. 4 mm wide; sepals 3, valvate, basally fused, 3–5 mm long, 5–8 mm wide, ovate, apex rounded, base truncate, densely pubescent outside, glabrous inside, margins flat; petals free, outer petals longer than inner, inner petals entirely covered in bud; **outer petals 3, 12–22 mm long, 7–14 mm wide**, elliptic to ovate, apex obtuse, base truncate, brown-violet, margins flat, densely pubescent outside, pubescent with a glabrous base inside; inner petals 3, valvate, 9–14 mm long, 8–10 mm wide, ovate to rhombic, apex acute, base truncate, brown-violet, margins flat, glabrous but pubescent towards base outside, glabrous but pubescent towards the base inside; stamens 90 to 110, in 3 to 4 rows, ca. 1 mm long, cuneate; connective truncate, glabrous; staminodes absent; carpels free, 40 to 60, ovary ca. 3 mm long, stigma elongate, glabrous. Monocarps stipitate, stipes 2–5 mm long, 2 mm in diameter; monocarps 5 to 15, 14–18 mm long, 34 mm in diameter, moniliform, ellipsoid, apex rounded to apiculate, densely pubescent, smooth, constricted around seeds when more than 1, glaucous green when ripe; seeds 1 to 2(3) per monocarp, 11–12 mm long, 7–11 mm in diameter, ellipsoid; aril absent.

#### Distribution.

A widespread west and central African species, from Guinea to Ivory Coast, and from Cameroon to the Republic of the Congo, the Democratic Republic of the Congo, Central African Republic and Uganda; in Cameroon recorded from Adamaoua, Central, East, Littoral, South, South-West regions.

#### Habitat.

A very common and widespread species; in primary and young or old secondary rain forests, or semi-deciduous forests, submontane forests, gallery forests and swamp forests. Altitude 0–1200 m a.s.l.

#### Local and common names known in Cameroon.

Mavembegne (Pygmée name, language not specified).

#### Preliminary IUCN conservation status.

Least Concern (LC) (Hoekstra et al. 2021).

#### Uses in Cameroon.

None reported.

#### Notes.

﻿﻿﻿*Monanthotaxisenghiana* is usually distinguished by the narrowly oblong to oblanceolate leaves that are whitish-blue below, and the long dense dark-brown erect hairs on the young foliate branches, petioles and lower side of leaf blades. Some specimens have oblong leaves and can be confused with ﻿*M.hirsuta* when sterile. However, this latter species has much larger flowers (the petals being more than twice as long) and its thecae cover more than half the stamen length, while in ﻿*M.enghiana* the thecae are very short covering less than half the stamen length. ﻿﻿﻿*Monanthotaxisenghiana* is also morphologically close to ﻿*M.dielsiana* and ﻿*M.glaucifolia*, but clearly differs in the pubescence type.

This is one of the most common species of Annonaceae across the forest region of Cameroon. It is generally encountered as a sapling on the forest floor. As for most lianas, it resembles a scrambling shrub when juvenile, sometimes forming large clumps.

#### Selected specimens examined.

**Adamaoua Region**: Mbakaou, 6.00°N, 12.88°E, *12 January 2017*, *Kamdem N.* 465 (YA). **Central Region**: Mefou proposed national park Near Mefou town, 3.62°N, 11.58°E, *08 March 2004*, *Cheek M.* 11499 (K,YA); Ottotomo Forest reserve 7 km north-west from Ngoumou 30 km south west from Yaoundé, 3.65°N, 11.28°E, *24 February 2016*, *Couvreur T.L.P.* 986 (WAG,YA); Ngoro, 5.07°N, 11.22°E, *29 April 2017*, *Kamdem N.* 499 (YA). **East Region**: 18 km NW of Doumé along road to Nguélémendouka, 4.23°N, 13.45°E, *24 November 1961*, *Breteler F.J.* 2137 (BR,P,WAG,YA); 75 km south of Yokadouma 30 km after Ngato 15 km after river ALPICAM ‘base de vie’ then 40 km on forestry road starting 4 km before Maséa village, 3.15°N, 14.73°E, *05 March 2019*, *Couvreur T.L.P.* 1201 (MPU,WAG,YA); Palisco forest consession 15 km along main road into consession, 3.48°N, 13.59°E, *27 March 2015*, *Couvreur T.L.P.* 754 (WAG,YA); Deng Deng, 5.20°N, 13.13°E, *27 July 2014*, *Kamdem N.* 167 (YA); Route Bertoua-Deng Deng à 6 km au Sud de Mambaya, 4.91°N, 13.76°E, *26 January 1961*, *Letouzey R.* 3248 (P,YA); A 15 km au S de Djouo (20 km E de Somalomo sur le Dja), 3.32°N, 12.93°E, *23 February 1962*, *Letouzey R.* 4359 (P,YA); A 20 km au S de Mvoy I (45 km à l’Est de Yokadouma), 3.31°N, 15.51°E, *16 May 1963*, *Letouzey R.* 5071 (P,YA). **Littoral Region**: Mapubi 30 km before Edea on Yaoundé-Edea road On forestry road 5 km direction to Sanaga river, 3.84°N, 10.38°E, *28 February 2018*, *Couvreur T.L.P.* 1176 (MPU,WAG,YA); Ebo Wildlife Reserve Djuma permanent camp On Djashaka trail, 4.35°N, 10.24°E, *13 February 2014*, *Couvreur T.L.P.* 618 (WAG,YA); Mambe Massif above Boga village 100 km along road from Yaoundé to Ed 3.91°N, 10.77°E, *19 June 2014*, *Couvreur T.L.P.* 653 (WAG,YA); Ebo forest reserve ca 2500 m on Dicam trail from Bekob camp, 4.34°N, 10.40°E, *11 March 2007*, *Wieringa J.J.* 5898 (WAG). **South Region**: 20 km from Kribi 2 km N of Lolodorf road, 3.01°N, 10.05°E, *12 December 1969*, *Bos J.J.* 5818 (WAG); 24 km from Kribi ca 3 km N of Lolodorf road, 3.03°N, 10.08°E, *31 March 1970*, *Bos J.J.* 6653 (BR,K,LD,LM,MO,P,WAG,YA); 20 km east from Lélé village, 2.27°N, 13.33°E, *07 September 2013*, *Couvreur T.L.P.* 466 (WAG,YA); Campo Ma’an National Park 11 km on trail from Ebinanemeyong village on road 7 km from Nyabessan to Campo town, 2.47°N, 10.33°E, *11 February 2015*, *Couvreur T.L.P.* 669 (WAG,YA); Campo Ma’an National Park 11 km on trail from Ebinanemeyong village on road 7 km from Nyabessan to Campo town, 2.47°N, 10.33°E, *12 February 2015*, *Couvreur T.L.P.* 691 (WAG,YA); Ebom, 3.1°N, 10.71°E, *27 February 1997*, *Parren M.P.E.* 23 (KRIBI,WAG); Bipindi, 3.08°N, 10.42°E, *01 May 1913*, *Zenker G.A.* 357 (M,P,U,WAG). **South-West Region**: Kupe village, 4.76°N, 9.694°E, *21 May 1996*, *Cable S.* 2523 (K); Gully by Daniel Ajang’s saprophyte site, 4.78°N, 9.716°E, *07 July 1996*, *Cable S.* 3683 (K,YA); Bayang Mbo Wildlife Sanctuary after Mbu river, 5.35°N, 9.502°E, *25 March 2016*, *Couvreur T.L.P.* 1003 (WAG,YA); Mount Cameroon National Park Bakinguili trail above Bakinguili village, 4.09°N, 9.057°E, *02 April 2016*, *Couvreur T.L.P.* 1037 (WAG,YA); on forest trail north of Ngomboku village, 4.91°N, 9.730°E, *06 April 2016*, *Couvreur T.L.P.* 1065 (WAG,YA); Etome, 4.05°N, 9.116°E, *31 January 1997*, *Nning J.* 212 (K,MO,YA); Bakingili, 4.06°N, 9.033°E, *15 February 1997*, *Nning J.* 259 (K,YA); Mahole, 4.81°N, 9.615°E, *29 November 1999*, *Onana J.M.* 947 (K,MO,WAG,YA).

### ﻿﻿﻿﻿ Monanthotaxis ferruginea

Taxon classificationPlantaeMagnolialesAnnonaceae

(Oliv.) Engl. & Diels, Kew Bull. 25(1): 26, 1971

C6B95234-C1D6-5C7B-9436-422A9D80CB7A

Map 7E

≡ Unona ferruginea Oliv., Fl. Trop. Afr. 1: 35, 1868. ﻿Popowiaferruginea (Oliv.) Engl. & Diels: Monogr. Afrik. Pflanzen.-Fam. 6: 46, 1901.
= Unona eminii Engl., Pflanzenw. Ost-Afrikas C: 179, 1895. Syntypes: Stuhlmann F.L. 1556, *n.v.*, Stuhlmann F.L. 4022, n.v.
= Popowia djumaensis De Wild., Ann. Mus. Congo Belge, Bot. sér. 5, 3[1]: 76, 1909. Type. Democratic Republic of the Congo. Bandundu, vallée de la Dju *Gillet J. 2803*, Jul. 1907: holotype: BR[BR0000008803160, BR0000008803962].

#### Type.

Angola. Cuanza Norte; Golungo Alto, *Welwitsch F.M.J. 761*, Jul 1855: lectotype, designated by Paiva (1966), p. 41: LISU[LISU206061]; isolectotypes: B[B100153029]; BM[BM000553834, BM000553835]; BR[BR0000008805324]; COI[COI00004905]; G[G00308369]; K[K000198968]; LISU[LISU206062]; P[P00362602].

#### Description.

Shrub to liana, 3–6 m tall, d.b.h. unknown. Indumentum of simple hairs; old leafless branches glabrescent, **young foliate branches densely pubescent with dense erect reddish brown hairs 0.5–0.9 mm long**. Leaves: petiole 4–8 mm long, 1–2 mm in diameter, densely pubescent, cylindrical, blade inserted on top of the petiole; blade 3.6–17.2 cm long, 1.8–6.8 cm wide, obovate to oblong-elliptic, **apex acute**, base subcordate, papyraceous, below pubescent with erect reddish brown hairs when young, sparsely pubescent when old, above sparsely pubescent when young, glabrous when old, discolorous, whitish below; midrib impressed, above pubescent when young and old, below densely pubescent when young, pubescent when old; secondary veins 7 to 15 pairs, glabrous above; tertiary venation percurrent. Individuals bisexual; inflorescences ramiflorous on old leafless branches, leaf opposed to extra axillary. Flowers with 9 perianth parts in 3 whorls, 1 per inflorescence; pedicel 13–36 mm long, 0.5–1 mm in diameter, pubescent; in fruit 5–27 mm long, 1 mm in diameter; bracts 2, one basal, soon falling, and one upper towards the middle of pedicel or lower half of pedicel, basal bract not seen, upper bract 2–6 mm long, 1–5 mm wide; sepals 3, valvate, free, 2–3 mm long, 3 mm wide, ovate to semiorbicular, apex rounded, base truncate, sparsely pubescent outside, glabrous inside, margins flat; petals free, outer petals longer than inner, inner petals entirely covered in bud; outer petals 3, 5.8–6.7 mm long, 4.5–6 mm wide, ovate, apex obtuse, base truncate, pale yellow, margins flat, densely pubescent outside, pubescent towards the margins inside; inner petals 3, valvate, 3–5.4 mm long, 2.7–2.9 mm wide, elliptic to ovate, apex obtuse, base truncate, pale yellow, margins flat, densely pubescent outside, pubescent with a glabrous base inside; **stamens 22 to 25, in 3 rows**, 1–2 mm long, obovate; connective truncate, glabrous; staminodes absent; **carpels free, 12 to 24, ovary 1–2 mm long, stigma elongate, glabrous.** Monocarps stipitate, stipes 3–7 mm long, 2–3 mm in diameter; monocarps 2 to 17, 12–35 mm long, 6–9 mm in diameter, moniliform, ellipsoid, apex rounded to apiculate, glabrous, verrucose to weakly torulose, constricted around seeds when more than 1, orange to red when ripe; seeds 1 to 3(to 5) per monocarp, 7–8 mm long, 5–6 mm in diameter, ellipsoid; aril absent.

#### Distribution.

A widespread species in Central and East Africa, with a disjunct distribution between Cameroon, Gabon, the Republic of the Congo and western Democratic Republic of the Congo and eastern Democratic Republic of the Congo, Uganda, Burundi, Rwanda, Ethiopia, and western Tanzania and Kenya; in Cameroon known from the East and South regions.

#### Habitat.

A fairly uncommon species in Cameroon, known from three collections to date; in gallery forests, lowland rain forest, brachystegia woodlands, forest edges, old secondary forests, montane forests and rocky plateaus. Altitude 300–700 m a.s.l.

#### Local and common names known in Cameroon.

None recorded.

#### Preliminary IUCN conservation status.

Least Concern (LC) (Hoekstra et al. 2021).

#### Uses in Cameroon.

None reported.

#### Notes.

﻿﻿﻿*Monanthotaxisferruginea* is distinguished by its pubescent branches, petioles and underside of leaf blades with dense erect reddish brown hairs, and its flowers with 22–25 stamens and glabrous carpels. In the vegetative state, it resembles ﻿*M.bokoli*, but ﻿*M.ferruginea* has acute leaf apices (for specimens in Cameroon and Gabon), while these are obtuse in ﻿*M.bokoli*.

#### Specimens examined.

**East Region**: Letta vers Bertoua, 4.55°N, 13.49°E, *07 February 1960*, *Letouzey R.* 2955 (P,P,YA). **South Region**: 25 km on the road from N’Koemvone to Akoakas Akoakas rock, 2.71°N, 11.28°E, *18 July 1975*, *de Wilde J.J.F.E* 8371 (BR,MO,P,WAG); Rocher d’Ako’Akas 25 km SE d’Ebolowa sur piste d’Evindissi, 2.43°N, 11.18°E, *04 February 1970*, *Letouzey R.* 10007 (IFAN,MO,P,YA).

### ﻿﻿﻿﻿ Monanthotaxis filamentosa

Taxon classificationPlantaeMagnolialesAnnonaceae

(Diels) Verdc., Kew Bull. 25(1): 31, 1971

CDFE8722-F233-5600-956D-E5904CF03B7D

**Figure 55.**
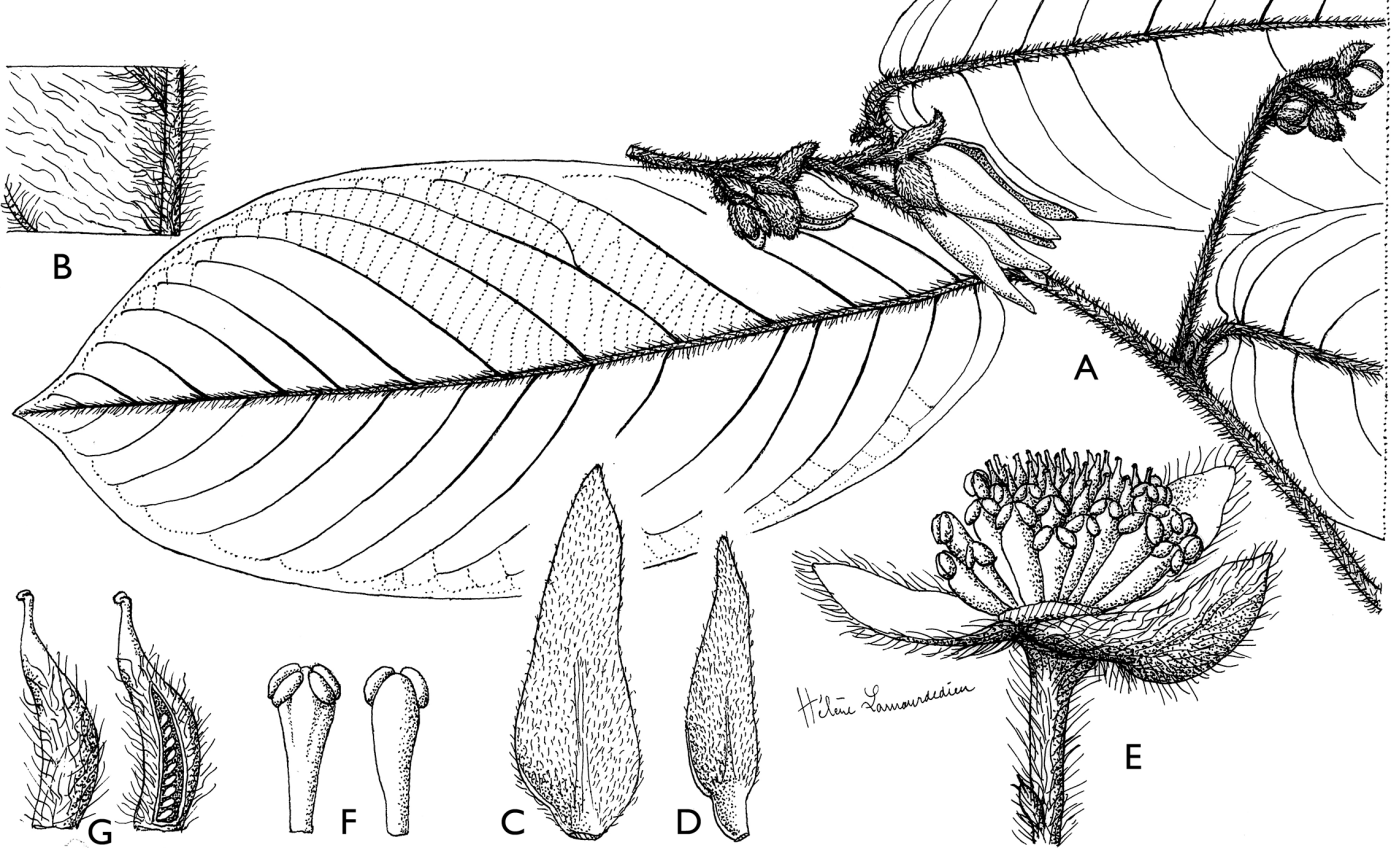
*Monanthotaxisfilamentosa***A** flowering branch **B** detail of leaf pubescence, lower side **C** outer petal, inner view **D** inner petal, inner view **E** detail of flower, all petals removed, side view **F** stamens, front and side views **G** carpel, side view and detail of ovules **A–G** from *Le Testu 3824*. Drawings by Hélène Lamourdedieu, Publications Scientifiques du Muséum national d’Histoire naturelle, Paris.

**Figure 56.**
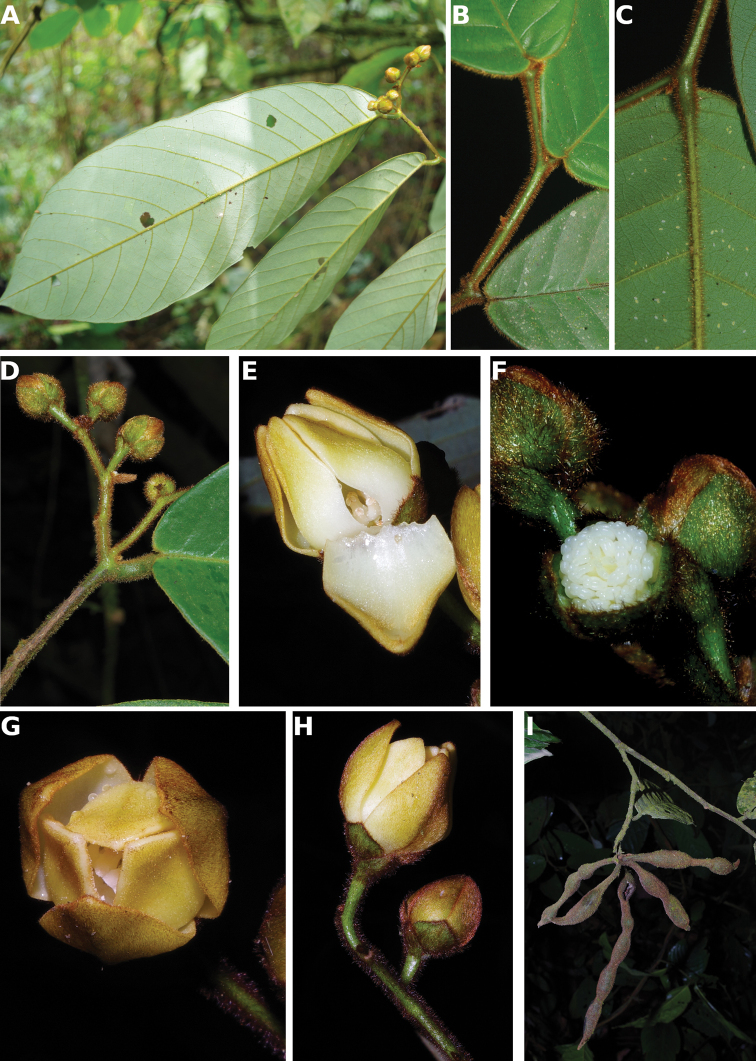
*Monanthotaxisfilamentosa***A** leaf, lower side, and terminal inflorescence **B** base of leaf blade, upper side **C** base of leaf base, lower side **D** inflorescence, leaf opposed **E** flower, one petal folded, revealing inner petals **F** detail of receptacle, all petals removed, showing prominent anthers and no connective **G** flower, top view **H** flowers, side view **I** fruit **A–I***Couvreur 989*, Mt Cameroon, Cameroon. Photos Thomas L.P. Couvreur.

Figs 55 , 56 ; Map 7F

≡ Popowia filamentosa Diels, Bot. Jahrb. Syst. 39(3–4): 478, 1907.
= Popowia malchairii De Wild., Etudes Fl. Bangala & Ubangi: 309, 1911. Type. Democratic Republic of the Congo. Sud-Ubangi, Budjala, environs de Likimi, Malchair L. 295, 25 Apr 1910: holotype: BR[BR0000008804624, BR0000008804952].
= Popowia setosa Diels, Bot. Jahrb. Syst. 53(3–5): 442, 1915. Type. Cameroon. South Region, 58 km E of Kribi, Fenda, *Mildbraed G.W.J. 5989*, 1911: lectotype, chosen by Hoekstra et al. (2021), p. 155: B[B100154095]; isolectotype: HBG[HBG502503]

#### Type.

Cameroon. South Region; Bipindi, *Zenker G.A. 2985*, Apr 1904: holotype: B not seen; isotypes: BR[BR0000008804297]; E[E00624352]; GOET[GOET005686]; HBG[HBG-502505]; K[K000198988]; L[L 0038042]; M[M0107931]; P[P00362600]; S[S07-13262]; WAG[WAG0071434]; WU[WU0025872]..

#### Description.

Liana, 10 m tall, d.b.h. up to 10 cm. Indumentum of simple hairs; old leafless branches glabrous, **young foliate branches densely pubescent with dense erect reddish brown hairs 0.7–1.4 mm long**. Leaves: petiole 3–6 mm long, 1–3 mm in diameter, densely pubescent with erect reddish brown hairs, grooved, blade inserted on top of the petiole; blade 12.5–28.6 cm long, 4.1–12.5 cm wide, obovate to oblanceolate, apex acuminate to acute, acumen 0.5–1 cm long, base rounded to subcordate, subcoriaceous, below sparsely pubescent with erect yellowish hairs when young and old, above sparsely pubescent when young, glabrous when old, discolorous, whitish below; midrib impressed, above pubescent when young and old, below densely pubescent when young and old; secondary veins 12 to 19 pairs, glabrous above; tertiary venation percurrent. Individuals bisexual; inflorescences ramiflorous on old leafless branches, leaf opposed to axillary. Flowers with 9 perianth parts in 3 whorls, 1 to 10 per inflorescence; pedicel 1–18 mm long, ca. 1 mm in diameter, pubescent with erect reddish brown hairs; in fruit 9–31 mm long, 1–2 mm in diameter; basal bract ca. 2 mm long, ca. 1 mm wide; upper bract 1–8 mm long, 1–4 mm wide; sepals 3, valvate, free, 5–10 mm long, 3–6 mm wide, elliptic to ovate, apex acute, base truncate, brown, densely pubescent outside, glabrous inside, margins flat; petals free, outer petals longer than inner, inner petals entirely covered in bud; outer petals 3, 8–22 mm long, 4.7–8.5 mm wide, ovate, apex acute to attenuate, base truncate, yellow-brown bright white towards the base and inside, margins flat, densely pubescent outside, pubescent with a glabrous base inside; inner petals 3, valvate, 6.5–14 mm long, 3–5 mm wide, elliptic to ovate, apex acute to attenuate, base truncate, yellow-brown bright white towards the base and inside, margins flat, densely pubescent outside, pubescent with a glabrous base inside; stamens 17 to 46, in 2 to 4 rows, ca. 2 mm long, linear; **thecae convergent apically hiding the connective apically**, white, **filament 1.3–1.9 mm long**; staminodes absent; carpels free, 8 to 14, ovary 2–3 mm long, stigma elongate, white, glabrous. Monocarps stipitate, stipes 8–15 mm long, 2–3 mm in diameter; monocarps 1 to 7, 110–175 mm long, 7–8 mm in diameter, moniliform, cylindrical, apex apiculate, pubescent to sparsely pubescent, verrucose, constricted around seeds when more than 1, yellow to red when ripe; seeds 1 to 7 per monocarp, 17–25 mm long, 4–5 mm in diameter, ellipsoid; aril absent.

#### Distribution.

A widespread central African species, from Cameroon to Gabon and Equatorial Guinea Democratic Republic of the Congo and Central African Republic; in Cameroon known from Central, East, Littoral, South, and South-West regions.

#### Habitat.

A common species in Cameroon; in lowland or submontane primary and old secondary rain forests, and gallery forests, on rocky soil. Altitude 100–1200 m a.s.l.

#### Local and common names known in Cameroon.

None recorded.

#### Preliminary IUCN conservation status.

Least Concern (Hoekstra et al. 2021).

#### Uses in Cameroon.

None reported.

#### Notes.

﻿﻿﻿*Monanthotaxisfilamentosa* is distinguished by the dense reddish brown erect pubescence on the young foliate branches and leaves, large ovoid flower buds, and stamens with large white thecae covering the apical connective and a long filament (> 1.3 mm versus generally shorter than 0.5 mm).

In the checklist to the plants of Lebialem (Harvey et al. 2010), the collection *Tchiengue 2637* was suggested to be a new species (sp. nov. of Bechati), but it has since been identified as ﻿*M.filamentosa* (Hoekstra et al. 2021).

#### Specimens examined.

**Central Region**: Mont Mbam Minkon on trail 5 km from Nkol Nyada village On top of small hill, 3.97°N, 11.40°E, *21 March 2013*, *Couvreur T.L.P.* 417 (MPU,WAG,YA). **East Region**: 43 km NW of Bertoua Road from Mbang to Ebaka, 4.58°N, 13.68°E, *19 May 1961*, *Breteler F.J.* 1374 (BR,K,P,U,WAG,YA); Village situé à 35 km au NNE de Moloundou, 2.29°N, 15.35°E, *21 April 1971*, *Letouzey R.* 10717 (P,YA). **Littoral Region**: Path above village summit Likombe village Etinde, 4.21°N, 9.166°E, *28 February 1995*, *Etuge M.* 1191 (K); Nsoung-1 km from village towards east river Meduya, 4.96°N, 9.802°E, *21 March 2003*, *Etuge M.* 4919 (K). **South Region**: Campo Ma’an National Park 11 km on trail from Ebinanemeyong village on road 7 km from Nyabessan to Campo town, 2.49°N, 10.34°E, *12 February 2015*, *Couvreur T.L.P.* 690 (WAG,YA); Campo Ma’an National Park 11 km on trail from Ebinanemeyong village on road 7 km from Nyabessan to Campo town, 2.47°N, 10.33°E, *13 February 2015*, *Couvreur T.L.P.* 695 (WAG,YA); 58 km E of Kribi Fenda, 2.8°N, 10.4°E, *01 January 1911*, *Mildbraed G.W.J.* 5989 (B,HBG); Bipindi, 3.08°N, 10.41°E, *01 April 1904*, *Zenker G.A.* 2985 (E,L,P,WAG). **South-West Region**: Nyasoso, 4.81°N, 9.683°E, *06 February 1995*, *Cable S.* 1185 (K,YA); Upper Boando, 4.06°N, 9.15°E, *15 March 1995*, *Cable S.* 1581 (K,YA); Kupe village, 4.78°N, 9.700°E, *23 May 1996*, *Cable S.* 2575 (K,YA); slopes of Mount Cameroon on the Bokwango trail near Bokwango village 4 km south west of Bu 4.12°N, 9.186°E, *23 March 2016*, *Couvreur T.L.P.* 989 (WAG,YA); Nyasoso, 4.83°N, 9.683°E, *27 February 1996*, *Etuge M.* 1729 (K,WAG,YA); Nyasoso, 4.83°N, 9.683°E, *24 June 1996*, *Etuge M.* 2377 (K,WAG,YA); Bime rock face, 5.07°N, 9.727°E, *12 November 2001*, *Etuge M.* 4531 (K); Entre Ndikoko et Ile 35 km N Kumba, 4.56°N, 9.23°E, *26 March 1976*, *Letouzey R.* 14591 (BENIN,GC,IFAN,MO,P,TOGO,YA); Cameroon mountain at Bu 4.15°N, 9.233°E, *01 January 1930*, *Maitland T.D.* 566 (K); Near the path from village Bechati to village Fossimondi, 5.65°N, 9.933°E, *26 September 2006*, *Tchiengue B.* 2816 (K).

### ﻿﻿﻿﻿ Monanthotaxis foliosa

Taxon classificationPlantaeMagnolialesAnnonaceae

(Engl. & Diels) Verdc., Kew Bull. 25(1): 21, 1971

B0200772-7632-5C0E-B7BC-FD34BF125180

Fig. 58 ; Map 7G

≡ Popowia foliosa Engl. & Diels, Monogr. Afrik. Pflanzen.-Fam. 6: 52, 1901. ﻿﻿Enneastemonfoliosus (Engl. & Diels) Robyns & Ghesq., Ann. Soc. Sci. Bruxelles, Ser. B 53: 165, 1933.
= ﻿﻿﻿﻿Monanthotaxis foliosa var. ferruginea (Robyns & Ghesq.) Verdc., Kew Bull., 25(1): 21, 1971; Enneastemon ferrugineus Robyns & Ghesq., Bull. Mus. Natl. Hist. Nat. Sér. 2 (6): 90, 1934; ﻿﻿﻿Enneastemonfoliosusvar.ferrugineus (Robyns & Ghesq.) Le Thomas, Fl. Gabon. 246, 1969. Type. Gabon. Nyanga, Tchibanga area Roungala, *Le Testu G.M.P.C. 2108*, 9 Sep 1915: holotype: P[P00362594, P00362596, P00362597]; isotypes: BM[BM000547358]; BR[BR0000008820235, BR0000008820242]; E[E00624354]; LISC[LISC000377].

#### Type.

Cameroon. South Region; Bipindi, *Zenker G.A. 2050*, 1899: lectotype, chosen by Hoekstra et al. (2021), p. 157: B[B100153030]; isolectotypes: B[B100153031]; BM[BM001125038]; BR[BR0000008820280]; E[E00624353]; G[G00014883, G00014884]; HBG[HBG-502537]; K[K000198987]; L[L.1754335]; M[M-0205486]; MO *n.v.*, P[P00362595, P00362598]; S[S07-13495]; WU[WU0025871].

#### Description.

Shrub when young (?) to liana, 4 m tall, d.b.h. unknown. Indumentum of simple hairs; old leafless branches glabrous, young foliate branches reddish brown to yellowish brown, with dense erect reddish brown hairs 0.2 mm long. Leaves: petiole 5–7 mm long, 1–2 mm in diameter, densely pubescent, slightly grooved, blade inserted on top of the petiole; blade 8.8–20 cm long, 5.2–8.9 cm wide, elliptic to oblong, apex acute to obtuse, base rounded to narrowly cuneate, subcoriaceous, below sparsely to densely pubescent when young, glabrous when old, above sparsely pubescent when young, glabrous when old, discolorous, whitish below; midrib depressed, above densely pubescent when young and old, below densely pubescent to sparsely pubescent when young and old; secondary veins 7 to 10 pairs, glabrous above; **tertiary venation percurrent, very dense**. Individuals bisexual; inflorescences ramiflorous on old leafless branches, **axillary**. Flowers with 9 perianth parts in 3 whorls, 1 to 16 per inflorescence; pedicel 6–11 mm long, ca. 1 mm in diameter, densely pubescent with dense erect reddish brown hairs; in fruit 7–15 mm long, ca. 1 mm in diameter; basal bract ca. 1 mm long, ca. 1 mm wide; upper bract 0.5–1 mm long, ca. 1 mm wide; sepals 3, valvate, basally fused, ca. 1 mm long, ca. 1 mm wide, ovate, apex acute to obtuse, base truncate, densely pubescent outside, glabrous inside, margins flat; petals free, outer petals longer than inner, **inner petals partly covered in bud**; outer petals 3, 2.5–6.5 mm long, 2–4.2 mm wide, ovate, apex obtuse, base truncate, margins flat, densely pubescent outside, pubescent with a glabrous base inside; inner petals 3, valvate, 2.4–6.1 mm long, 1.4–3.4 mm wide, rhombic, apex obtuse, base truncate, margins flat, densely pubescent outside, pubescent with a glabrous base inside; **stamens 8 to 9**, in 1 row, ca. 1 mm long, clavate; connective truncate, glabrous; staminodes absent; carpels free, 6 to 7, ovary 1–2 mm long, stigma elongate, glabrous. Monocarps stipitate, stipes ca. 4 mm long, ca. 2 mm in diameter; monocarps 1 to 3, 16–24 mm long, 9–11 mm in diameter, moniliform, cylindrical, apex apiculate, pubescent to glabrous, **smooth**, constricted around seeds when more than 1, color unknown; seeds 1 to 3 per monocarp, ca. 11 mm long, ca. 8 mm in diameter, ellipsoid; aril absent.

#### Distribution.

A widespread west and central African species, from Ivory Coast to eastern Nigeria and Cameroon to Gabon, with a disjunct distribution in eastern Democratic Republic of the Congo; in Cameroon known from the South and South-West regions.

**Figure 57.**
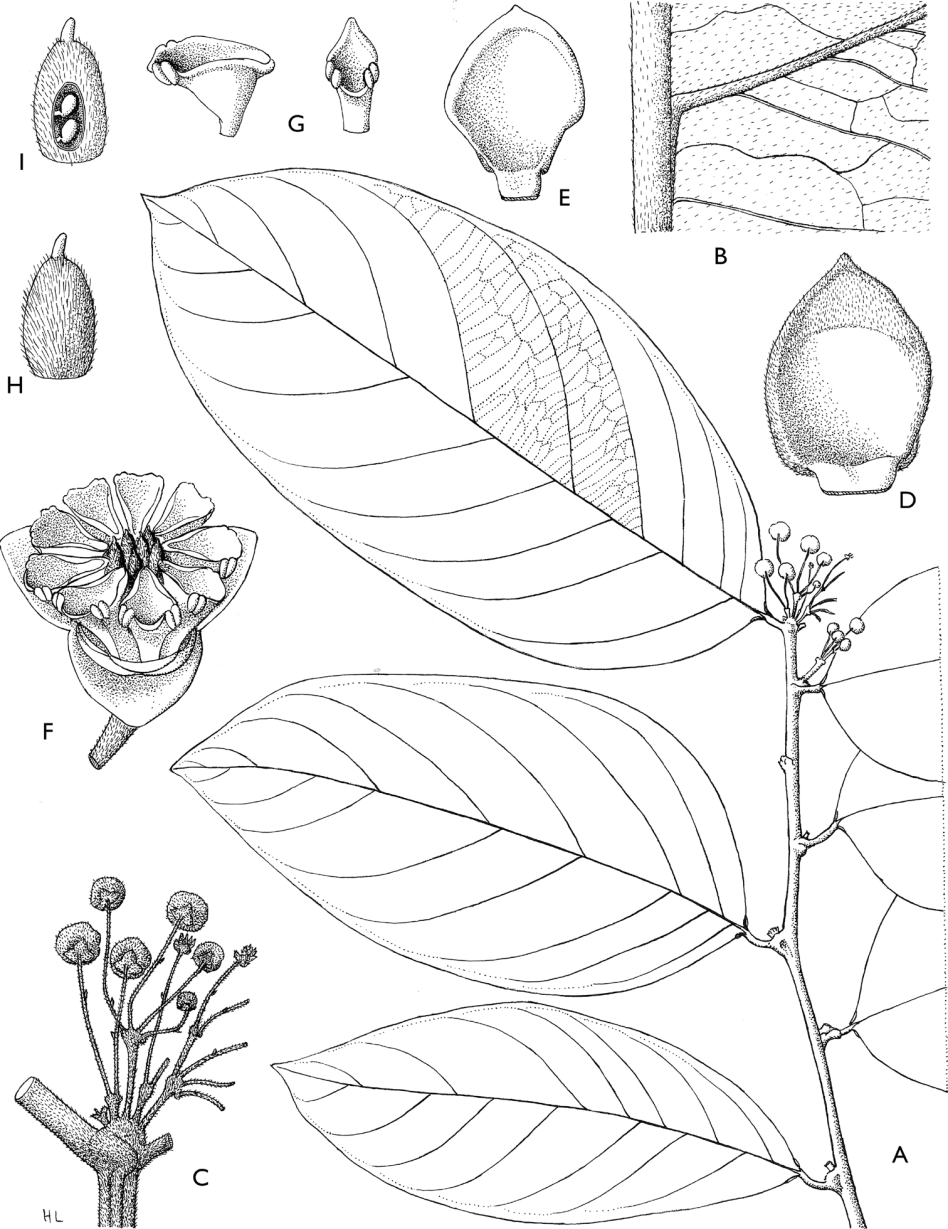
*Monanthotaxisfoliosa***A** flowering branch **B** detail of leaf pubescence, lower side **C** detail of inflorescence **D** outer petal, inner view **E** inner petal, inner view **F** detail of flower, all petals removed, semi top view **G** stamens, front and side views **H** carpel, side view **I** carpel, with detail of ovules **A–I** from *Zenker 3001*. Drawings by Hélène Lamourdedieu, Publications Scientifiques du Muséum national d’Histoire naturelle, Paris.

**Figure 58.**
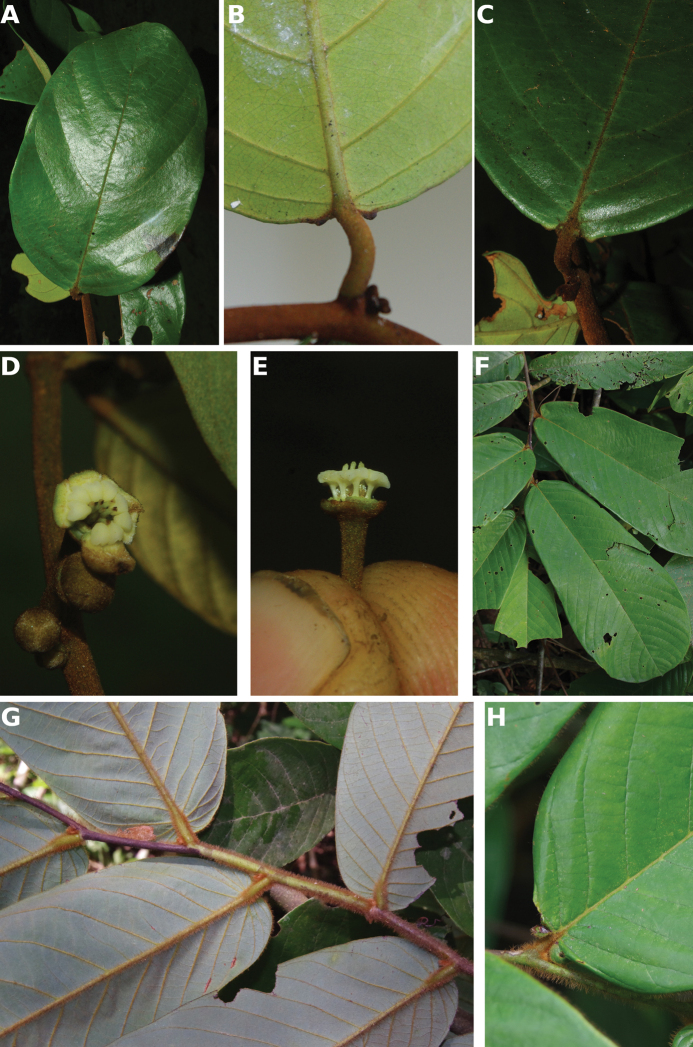
*Monanthotaxisfoliosa***A** leaf, upper view **B** base of leaf blade, lower view, note two small lobes at base **C** base of leaf blade, upper view **D** flower, petals removed, top view **E** detail of stamens, side view; not clearly unguiculate stamens. *Monanthotaxishirsuta***F** leaves, upper view **G** base of leaf blades, lower view **H** Base of leaf blade, upper view **A–E***Couvreur 601*, Gabon **F–H***Couvreur 1175*, Mapubi, Cameroon. Photos Thomas L.P. Couvreur.

#### Habitat.

A, uncommon species when present; in primary or secondary rain forests, and gallery forests. Altitude 300–1500 m a.s.l.

#### Local and common names known in Cameroon.

None recorded.

#### Preliminary IUCN conservation status.

Endangered (EN) (Hoekstra et al. 2021).

#### Uses in Cameroon.

None reported.

#### Notes.

﻿﻿﻿*Monanthotaxisfoliosa* belongs to the *schweinfurthii* complex (see under ﻿*M.capea*) which has axillary inflorescences, rounded flower buds in which the 3 outer petals overlap the 3 inner petals at the top and 8 to 9 stamens per flower. It differs from ﻿*M.capea* (the only other species of this complex present in Cameroon) by the dense tertiary venation raised on the upper side of the leaf blade, the more broadly cordate leaf base and the smooth monocarps (versus tuberculate-rugulose in ﻿*M.capea*).

#### Specimens examined.

**South Region**: Bipindi, 3.08°N, 10.41°E, *1899*, *Zenker G.A.* 2050 (E,L,M,P); Bipindi, 3.08°N, 10.41°E, *01 January 1904*, *Zenker G.A.* 3001 (B,E,G,K,L,M,MO,P). **South-West Region**: Permanent Sample Plot on Shrike trail above Nyasoso, 4.81°N, 9.683°E, *06 February 1995*, *Cable S.* 1169 (K,YA); Saddle of Kupe Rock, 4.79°N, 9.686°E, *14 November 1995*, *Cheek M.* 7794 (K,YA); Kupe village, 4.79°N, 9.701°E, *17 November 1995*, *Cheek M.* 7880 (K,WAG,YA); Nyasoso, 4.81°N, 9.716°E, *27 February 1996*, *Etuge M.* 1748 (K,WAG,YA); Kupe village, 4.76°N, 9.7°E, *03 December 1999*, *Gosline W.G.* 254 (K); Mapanja, 4.12°N, 9.115°E, *01 January 1931*, *Maitland T.D.* 1072 (K); Korup National Park, 5.01°N, 8.833°E, *12 April 1985*, *Thomas D.W.* 4721 (MO,P,WAG).

### ﻿﻿﻿﻿ Monanthotaxis glaucifolia

Taxon classificationPlantaeMagnolialesAnnonaceae

(Hutch. & Dalziel) P.H.Hoekstra, Taxon 66: 14, 2017

62DD3F51-C1B2-50F8-9BA8-7CC94526566D

Map 7H

≡ Oxymitra glaucifolia Hutch. & Dalziel, Kew Bull.: 153, 1927; ﻿Richellaglaucifolia (Hutch. & Dalziel) R.E.Fr., Nat. Pflanzenfam., ed. 2, 17a (2): 139, 1959; ﻿﻿Friesodielsiaglaucifolia (Hutch. & Dalziel) Steenis, Blumea 12: 359, 1964.

#### Type.

Nigeria. Cross River State; Oban, *Talbot P.A. 403*, 1911: holotype: BM[BM000843988].

#### Description.

Liana, height unknown, d.b.h. unknown. Indumentum of simple hairs; old leafless branches pubescent to glabrescent, **young foliate branches densely pubescent with dense appressed to ascending light-brown hairs 0.2–0.5 mm long**. Leaves: petiole 4–7 mm long, 2–3 mm in diameter, densely pubescent with light-brown hairs, slightly grooved, blade inserted on top of the petiole; blade 11–25.8 cm long, 3.7–8.6 cm wide, oblong to obovate, apex acuminate, acumen 0.5–2.5 cm long, base subcordate, subcoriaceous to membranous, below sparsely pubescent when young and old, above glabrous when young and old, discolorous, whitish below; midrib impressed, above glabrous when young and old, below sparsely pubescent when young, glabrous when old; secondary veins 10 to 13 pairs, glabrous above; tertiary venation percurrent. Individuals bisexual; inflorescences ramiflorous on old leafless branches, leaf opposed to extra axillary. Flowers with 9 perianth parts in 3 whorls, **1 per inflorescence**; pedicel 5–21 mm long, ca. 1 mm in diameter, densely pubescent; in fruit 5–21 mm long, 2 mm in diameter; upper bract ca. 3 mm long, ca. 2 mm wide; sepals 3, valvate, free, ca. 5 mm long, ca. 7 mm wide, ovate, apex obtuse, base truncate, densely pubescent outside, glabrous inside, margins flat; petals free, outer petals longer than inner, inner petals entirely covered in bud; outer petals 3, 30–35 mm long, 23–25 mm wide, ovate, apex, base truncate, margins flat, pubescent outside, sparsely pubescent to glabrous inside; inner petals 3, valvate, ca. 21 mm long, ca. 26 mm wide, rhombic, apex acute, base truncate, margins flat, pubescent outside, glabrous inside; **stamens 100 to 150**, in 5 to 6 rows, ca. 1 mm long, cuneate; connective truncate, glabrous; staminodes absent; carpels free, 45 to 50, ovary ca. 2 mm long, stigma globose, glabrous. Monocarps stipitate, stipes 3–4 mm long, ca. 2 mm in diameter; monocarps ca. 8, 15–26 mm long, 9–10 mm in diameter, ellipsoid, apex rounded to apiculate, pubescent, smooth, constricted around seeds when more than 1, color unknown; seeds 1 to 2 per monocarp, ca. 10 mm long, ca. 8 mm in diameter, ellipsoid; aril absent.

#### Distribution.

Known from Nigeria and Cameroon; in Cameroon known from the South-West region.

#### Habitat.

A rare species, in submontane primary forest. Altitude 950 m a.s.l.

#### Local and common names known in Cameroon.

None recorded.

#### Preliminary IUCN conservation status.

Endangered (EN) (Hoekstra et al. 2021).

#### Uses in Cameroon.

None reported.

#### Notes.

﻿﻿﻿*Monanthotaxisglaucifolia* resembles ﻿*M.dielsiana* and ﻿*M.enghiana* by the shape of its leaves (oblong to obovate) and the largish flowers. It differs from ﻿*M.dielsiana* by having more than 100 stamens per flower, and by light brown hairs on the young foliate branches, while ﻿*M.dielsiana* has orange-brown hairs and about 65 stamens per flower. ﻿﻿﻿*Monanthotaxisenghiana* differs from ﻿*M.glaucifolia* by its densely pubescent branches and leaves with long erect hairs; furthermore, ﻿*M.enghiana* generally has 2 to 5 flowers per inflorescence and ﻿*M.glaucifolia* only one.

It is possible that ﻿*M.enghiana* and ﻿*M.glaucifolia* are synonymous, and Hoekstra et al. (2021) suggested that the latter could merely be a higher altitude variant of the former. However for now, both species are retained before more detailed studies are done (Hoekstra et al. 2021).

#### Specimens examined.

**South-West Region**: Mount 4.78°N, 9.683°E, *26 November 1999*, *Cheek M.* 10154 (K,MO,WAG,YA); AyinKeh 3 km north of Ngomboku, 4.93°N, 9.731°E, *17 December 1999*, *Ghogue J.-P.* 500 (K,P,WAG,YA).

### ﻿﻿﻿﻿ Monanthotaxis gracilis

Taxon classificationPlantaeMagnolialesAnnonaceae

(Hook.f.) P.H.Hoekstra, Taxon 66 (1): 14, 2017

3366E5CC-435E-57A7-9D30-C4E695C45F96

Fig. 59 ; Map 7I

≡ ﻿﻿Uvaria gracilis Hook.f., Niger Fl.: 210, 1849. Oxymitra gracilis (Hook.f.) Sprague & Hutch., Bull. Misc. Inform. Kew 6: 154, 1916; ﻿Richellagracilis (Hook.f.) R.E.Fr., Nat. Pflanzenfam., ed. 2, 17a (2): 139, 1959; ﻿﻿Friesodielsiagracilis (Hook.f.) Steenis, Blumea 12: 359, 1964.
= Oxymitra platypetala Benth., Trans. Linn. Soc. London 23(3): 472, 1862; ﻿﻿Cleistopholisplatypetala (Benth.) Engl. & Diels, Monogr. Afrik. Pflanzen.-Fam. 6: 34, 1901. Type. Sierra Leone. Southern Province, Bagroo river, *Mann G. 857*, Apr 1896: holotype: K[K00198952].
= Unona millenii Engl. & Diels, Monogr. Afrik. Pflanzen.-Fam. 6: 40, 1901. Type. Nigeria. Lagos State, Lagos, Millen H. 149, Mar 1896: holotype: K, not seen.
= Oxymitra rosea Sprague & Hutch., Bull. Misc. Inform. Kew 6: 154, 1916; ﻿Richellarosea (Sprague & Hutch.) R.E.Fr., in Engler & Prantl Nat. Pflanzenfam., ed. 2, 17a (2): 139, 1959; ﻿﻿Friesodielsiarosea (Sprague & Hutch.) Steenis, Blumea 12: 361, 1964. Type. Nigeria. Cross River State, Oban, Talbot P.A. 199, 1911: holotype: BM[BM000547067].

#### Type.

Sierra Leone. no region; no location, *Don G. s.n.*, no date: holotype: BM[BM000547066].

#### Description.

Shrub to liana, 4–20 m tall, d.b.h. 10–20 cm. Indumentum of simple hairs; old leafless branches sparsely pubescent to glabrous, **light grey in color**, young foliate branches pubescent with **very short appressed to ascending hairs 0.1 mm long**. Leaves: petiole 2–4 mm long, 0.5–1 mm in diameter, pubescent, grooved, blade inserted on top of the petiole; blade 4–16.5 cm long, 1.4–5.7 cm wide, obovate to oblanceolate, apex acuminate to acute, acumen ca. 2 cm long, base cuneate to subcordate, subcoriaceous, below sparsely pubescent to glabrous when young, glabrous when old, above glabrous when young and old, discolorous, whitish below; midrib depressed, above glabrous when young and old, below sparsely pubescent when young, glabrous when old; secondary veins 7 to 10 pairs, glabrous above; tertiary venation percurrent. Individuals bisexual; inflorescences ramiflorous on old leafless branches, leaf opposed to extra axillary. Flowers with 9 perianth parts in 3 whorls, 1 to 3 per inflorescence; **pedicel 15–45 mm long, ca. 0.5 mm in diameter**, pubescent; in fruit 25–50 mm long, 0.5–1 mm in diameter; basal bract 1–2 mm long, 0.5–1 mm wide; **upper bract 2–3 mm long, ca. 1 mm wide, lanceolate in shape**; sepals 3, valvate, basally fused, 3–6 mm long, 4–5 mm wide, ovate, apex obtuse, base truncate, sparsely pubescent outside, glabrous inside, margins flat; petals free, outer petals longer than inner, inner petals entirely covered in bud; outer petals 3, 12–30 mm long, 7–12 mm wide, **elliptic**, apex obtuse, base truncate, light yellow to light green, margins flat, pubescent outside, pubescent inside; inner petals 3, valvate, 5–9 mm long, 3–5 mm wide, ovate to rhombic, apex acute, base truncate, margins flat, pubescent outside, sparsely pubescent to glabrous inside; stamens 80 to 125, in 3 to 5 rows, ca. 1 mm long, linear to oblong; connective globose, glabrous; staminodes absent; carpels free, 17 to 24, ovary 1–2 mm long, stigma elongate, pubescent. Monocarps stipitate, stipes 4–8 mm long, 2 mm in diameter; monocarps 10 to 20, red when ripe, 13–35 mm long, 4–5 mm in diameter, moniliform, ellipsoid to globose, apex apiculate, densely pubescent, pubescent, constricted around seeds when more than 1, red when ripe; seeds 1 to 5 per monocarp, ca. 6 mm long, ca. 4 mm in diameter, ellipsoid; aril absent.

#### Distribution.

A mainly west African species, from Sierra Leone to Nigeria, just reaching into Cameroon where known from the South-West region.

#### Habitat.

A rare species in Cameroon; in primary and secondary rain forests, swamp forests and gallery forests, on river banks and summit ridges. Altitude: 100 m a.s.l.

**Figure 59.**
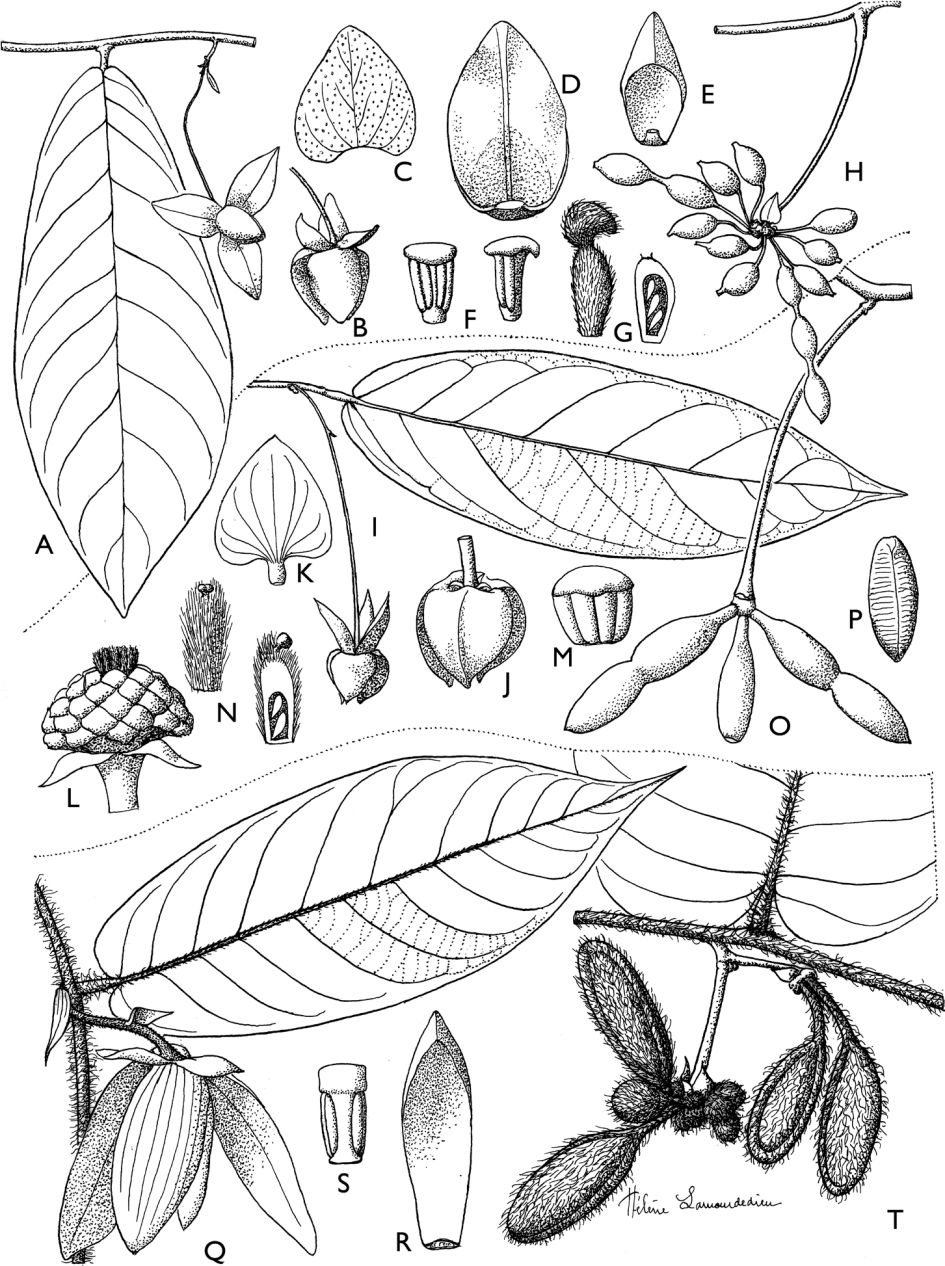
*Monanthotaxisgracilis***A** Flowering branch **B** flower, side view **C** sepal, inner view **D** outer petal, inner view **E** inner petal; inner view **F** stamens, side and front views **G** carpel, side view and detail of ovules **H** fruit, note persistent sepals. *Monanthotaxismontana***I** flowering branch **J** flower, side view **K** inner petal, inner view **L** flower, all petals removed, side view **M** stamen, front view **N** carpel, side view and details of ovules **O** fruiting branch **P** seed, side view. *Monanthotaxishirsuta***Q** flowering branch **R** inner petal, inner view **S** stamen, front view **T** fruiting branch **A** from *Meikle 956***B, C** from *Akpabla 1106***D–G** from *Dalziel 1096***H** from *FHI 28906***I–P** unknown **Q–S** from *Chevalier 14817***T** from *FHI 19738*. Drawings by Hélène Lamourdedieu, Publications Scientifiques du Muséum national d’Histoire naturelle, Paris.

#### Local and common names known in Cameroon.

None recorded.

#### Preliminary IUCN conservation status.

Least Concern (LC) (Hoekstra et al. 2021).

#### Uses in Cameroon.

None reported.

#### Notes.

﻿﻿﻿*Monanthotaxisgracilis* is distinguished by its light-grey branches, very short hairs on the branches and pedicels, a small lanceolate upper bract, its long slender flowering pedicels and ellipsoid flower buds and elliptic outer petals.

This species is only known from a single collection in Cameroon to date.

#### Specimens examined.

**South-West Region**: Korup National Park, 5.01°N, 8.8°E, *15 June 2000*, *van der Burgt X.M.* 614 (WAG).

### ﻿﻿﻿﻿ Monanthotaxis hexamera

Taxon classificationPlantaeMagnolialesAnnonaceae

P.H.Hoekstra, Blumea 66 (1): 163, 2021

1B354818-18F1-5D83-B0C5-2B9E7331E69C

Fig. 60 ; Map 8A

#### Type.

Cameroon. South Region; près de zingui, *Letouzey R.G. 10288*, 5 Apr 1970: holotype: P[P01960096]; isotype: YA[YA0002998].

#### Description.

Liana, up to 3 m tall, d.b.h. unknown. Indumentum of simple hairs; old leafless branches glabrescent, **young foliate branches pubescent with dense appressed to ascending yellowish-white hairs 0.1–0.2 mm long**. Leaves: petiole 3–5 mm long, ca. 1 mm in diameter, densely pubescent, slightly grooved, blade inserted on top of the petiole; blade 9.5–11.5 cm long, 3.3–4.1 cm wide, oblong to elliptic, apex acute, acumen 0.5–1 cm long, base rounded to subcordate, papyraceous, **below sparsely pubescent with yellowish-white hairs when young and old**, above glabrous when young and old, discolorous, whitish below; midrib impressed, above glabrous when young and old, below pubescent when young, sparsely pubescent when old; secondary veins 11 to 13 pairs, glabrous above; tertiary venation percurrent. Individuals bisexual; inflorescences ramiflorous on old leafless branches, leaf opposed to axillary. Flowers with 9 perianth parts in 3 whorls, 1 to 4 per inflorescence; pedicel 15–24 mm long, ca. 0.5 mm in diameter, sparsely pubescent; in fruit unknown; bracts 2, one basal and one upper towards the lower half of pedicel, basal bract ca. 1 mm long, ca. 0.5 mm wide; upper bract ca. 1 mm long, ca. 0.5–1 mm wide; sepals 3, valvate, basally fused, ca. 1 mm long, ca. 1 mm wide, ovate, apex acute, base truncate, sparsely pubescent outside, glabrous inside, margins flat; petals free, outer petals longer than inner, inner petals entirely covered in bud; outer petals 3, 4.5–8 mm long, 3.7–5.8 mm wide, ovate, apex rounded to obtuse, base truncate, white, margins flat, sparsely pubescent outside, pubescent, base glabrous inside; inner petals 3, valvate, ca. 5.1 mm long, 2.7–2.9 mm wide, elliptic, apex obtuse and shortly acuminate, base truncate, margins flat, densely pubescent outside, pubescent with a glabrous base inside; stamens 6, in 1 row, ca. 2 mm long, linear; connective truncate, pubescent, **filament ca. 2 mm long**; staminodes absent; carpels free, 6, ovary ca. 2 mm long, stigma globose, glabrous. Fruits unknown.

#### Distribution.

endemic to Cameroon; known only from the type locality in the South region.

#### Habitat.

In bushy scrub. Altitude ca. 450 m a.s.l.

**Figure 60.**
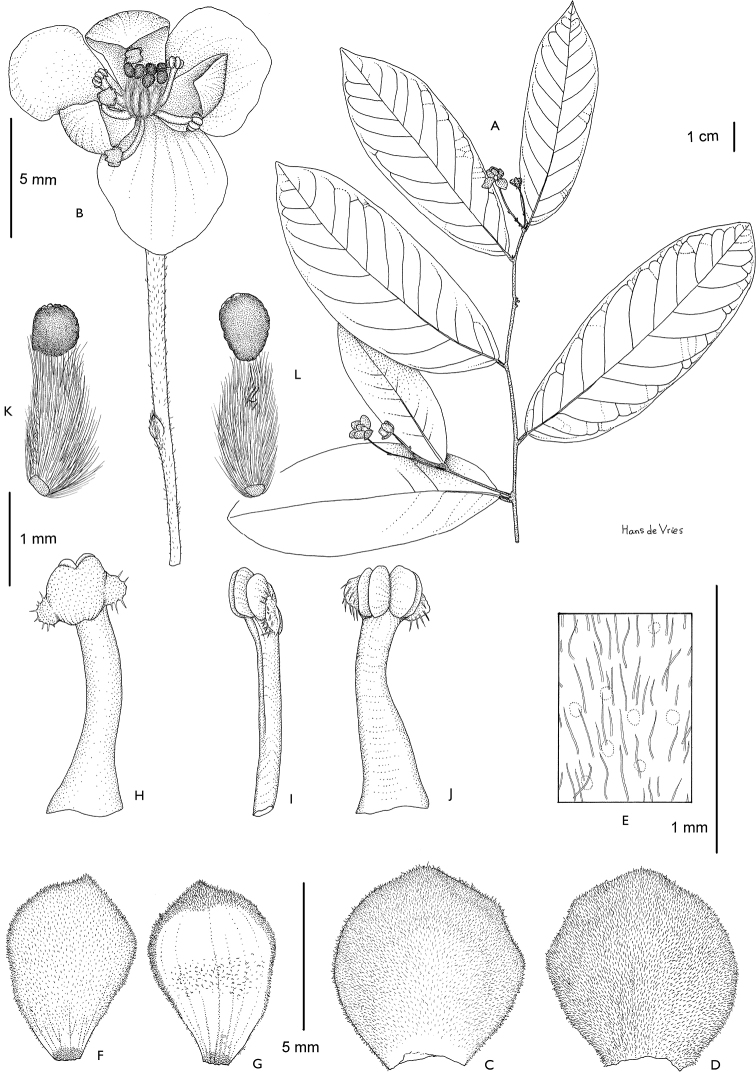
*Monanthotaxishexamera***A** flowering branch **B** flower **C** outer petal, inner view **D** outer petal, outer view **E** outer petal, outer detail **F** inner petal, outer view **G** inner petal, inner view **H** stamen, back view **I** stamen, side view **J** stamen, front view **K** carpel, side view **L** carpel, front view **A–L** from *Letouzey 10288.* Drawing by Hans de Vries (Hoekstra et al. 2021, fig. 16, p. 164).

**Map 8.**
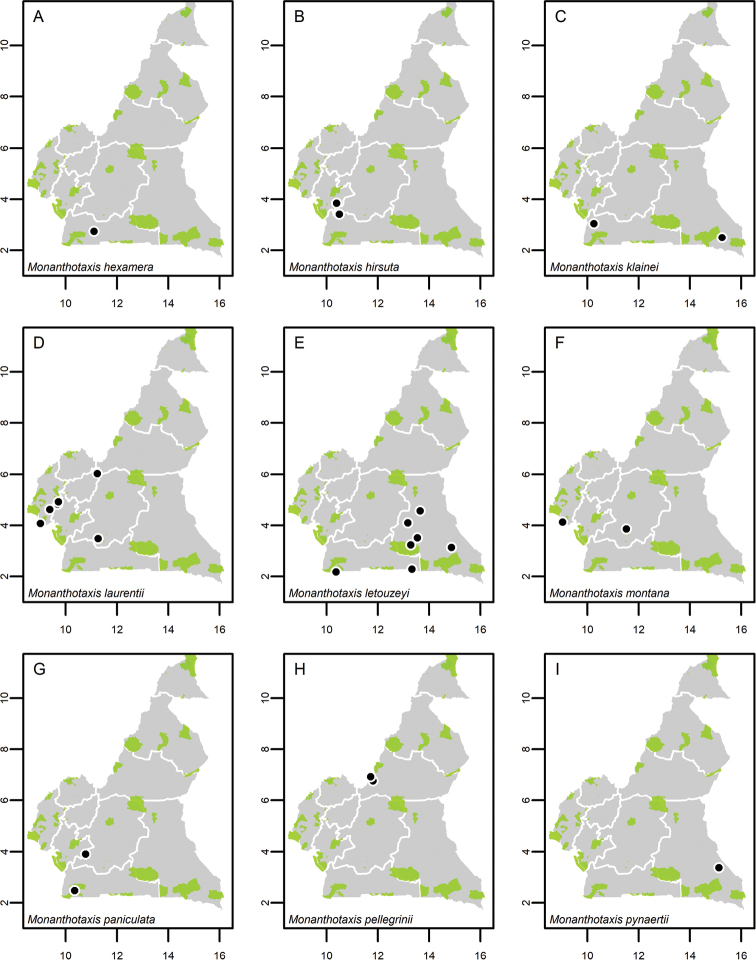
**A***Monanthotaxishexamera***B***Monanthotaxishirsuta***C***Monanthotaxisklainei***D***Monanthotaxislaurentii***E***Monanthotaxisletouzeyi***F***Monanthotaxismontana***G***Monanthotaxispaniculata***H***Monanthotaxispellegrinii***I***Monanthotaxispynaertii*. White borders represent region limits in Cameroon; green patches represent protected areas (see methods and Suppl. material 1: Fig. S1).

#### Local and common names known in Cameroon.

None recorded.

#### Preliminary IUCN conservation status.

Critically Endangered (CR) (Hoekstra et al. 2021).

#### Uses in Cameroon.

None reported.

#### Notes.

﻿﻿﻿*Monanthotaxishexamera* can be distinguished from the other Cameroonian species by the yellowish-white hairs on the branches and leaf underside. Furthermore, the stamens have very long filaments (ca. 2 mm long versus less than 0.5 mm long in other species), a characteristic only shared with ﻿﻿﻿*Monanthotaxisfilamentosa*, which has a very different indumentum consisting of long erect reddish-brown hairs.

### ﻿﻿﻿﻿ Monanthotaxis hirsuta

Taxon classificationPlantaeMagnolialesAnnonaceae

(Benth.) P.H.Hoekstra, Taxon 66: 14, 2017

E7C846FF-217F-50CD-8483-645E33DF2326

Figs 58 , 59 ; Map 8B

≡ Unona hirsuta Benth., Trans. Linn. Soc. London 23(3): 469, 1862; ﻿Oxymitrahirsuta (Benth.) Sprague & Hutch., Bull. Misc. Inform. Kew: 155, 1916; ﻿Richellahirsuta (Benth.) R.E.Fr., Nat. Pflanzenfam., ed. 2, 17 a(2): 139, 1959.
= Uvaria caillei A.Chev. ex Hutch. & Dalziel, Fl. W. Trop. Afr. 1: 49, 1927. Type. Guinea. Mamou, Timbou, Kouria, Chevalier A.J.B. 14817, 28 Nov 1905: lectotype, chosen by Hoekstra et al. (2021), p. 165: P[P00363329]; isolectotypes: G[G00308375]; L[L.1765233]; P[P00363319, P00363320, P01954813].

#### Type.

Equatorial Guinea. Bioko Norte; Fernando Poo, *Mann G. 559*, 1860: holotype: K[K000198950]; isotypes: P[P00363313; P00363314].

#### Description.

Shrub to liana, up to 3 m tall, d.b.h. unknown. Indumentum of simple hairs; old leafless branches glabrescent, **young foliate branches densely pubescent with erect reddish brown 1.2–1.7 mm long hairs**. Leaves: petiole 5–6 mm long, 2–3 mm in diameter, densely pubescent with erect reddish brown hairs, slightly grooved, blade inserted on top of the petiole; blade 8.3–28.5 cm long, 4.9–7.5 cm wide, oblong to obovate, apex acuminate to acute, acumen 0.5–2 cm long, base cordate or subcordate, subcoriaceous to membranous, below pubescent when young and old, above densely pubescent with yellowish hairs when young, sparsely pubescent with yellowish hairs to glabrous when old, discolorous, whitish below; midrib sunken or flat, above sparsely pubescent with erect reddish brown hairs when young, sparsely pubescent with erect reddish brown hairs when old, below densely pubescent when young, pubescent when old; secondary veins 9 to 23 pairs, glabrous above; tertiary venation percurrent. Individuals bisexual; inflorescences ramiflorous on old leafless branches, leaf opposed. Flowers with 9 perianth parts in 3 whorls, 1 to 2 per inflorescence; pedicel 7–12 mm long, 1–3 mm in diameter, densely pubescent with yellow erect hairs; in fruit 13–31 mm long, ca. 2 mm in diameter, pubescent; basal bract ca. 5 mm long, ca. 3 mm wide; upper bract ca. 5 mm long, ca. 5 mm wide; sepals 3, valvate, free, 6–9 mm long, 5–7 mm wide, triangular to ovate, apex acute to obtuse, base truncate, densely pubescent outside, glabrous inside, margins flat; petals free, outer petals longer than inner, inner petals entirely covered in bud; **outer petals 3, 21–50 mm long, 9–17 mm wide**, elliptic to ovate, apex acute, base truncate, red to cream, margins flat, densely pubescent outside, pubescent with a glabrous base inside; inner petals 3, valvate, 16–25 mm long, 6–8 mm wide, linear to elliptic, apex acute, base truncate, margins flat, densely pubescent outside, pubescent inside; stamens 100 to 120, in 4 to 5 rows, 1–2 mm long, cylindrical to obconic; connective truncate, glabrous; staminodes absent; carpels free, 22 to 24, ovary ca. 2 mm long, stigma elongate, glabrous. Monocarps stipitate, stipes 7–11 mm long, 2–3 mm in diameter; monocarps 2 to 9, 23–52 mm long, 9–13 mm in diameter, moniliform, ellipsoid to cylindrical, apex rounded to apiculate, **pubescent, densely pubescent with erect hairs**, slightly constricted around seeds when more than 1, orange when ripe; seeds 1 to 3, 17–27 mm long, 9–10 mm in diameter, ellipsoid; aril absent.

#### Distribution.

A mainly west African species from Guinea to Ivory Coast, and eastern Nigeria to Equatorial Guinea; in Cameroon known from the Central, Littoral, South and South-West regions.

#### Habitat.

A fairly uncommon species in Cameroon; in primary and secondary rain forest, in swamp forests, gallery forests. Altitude 50–200 m a.s.l.

#### Local and common names known in Cameroon.

None recorded.

#### Preliminary IUCN conservation status.

Least Concern (LC) (Hoekstra et al. 2021).

#### Uses in Cameroon.

None reported.

#### Notes.

﻿﻿﻿*Monanthotaxishirsuta* is distinguished by its branches and petioles with long (> 1 mm) erect reddish brown hairs and when in flower by its long petals (> 20 mm) and its monocarps with dense long erect hairs. This species is morphologically close to ﻿*M.enghiana* and ﻿*M.velutina* (the latter not present in Cameroon), but the former generally has narrowly obovate or oblanceolate leaves with a narrow subcordate base and acuminate leaf apex, while the latter generally has more obovate leaves with a broader subcordate base and obtuse to acute leaf apex.

Throughout its range, ﻿*M.hirsuta* is quite variable when sterile (Hoekstra et al. 2021) and can be hard to distinguish from ﻿*M.enghiana*. In Cameroon, however, ﻿*M.hirsuta* has broadly oblong or obovate leaves versus narrowly obovate leaves in ﻿*M.enghiana*.

#### Specimens examined.

**Central Region**: Chantier forestier au sud de Song Bong, 3.41°N, 10.5°E, *08 December 1967*, *Bamps P.R.J.* 1381 (BR,P,YA). **Littoral Region**: Mapubi 30 km before Edea on Yaoundé-Edea road On forestry road 5 km direction to Sanaga river, 3.84°N, 10.39°E, *28 February 2018*, *Couvreur T.L.P.* 1175 (K,MPU,P,WAG,YA).

### ﻿﻿ Monanthotaxis klainei

Taxon classificationPlantaeMagnolialesAnnonaceae

(Engl.) Verdc. var. lastoursvillensis (Pellegr.) Verdc., Kew Bull. 25(1): 30, 1971

5B1235AC-F7D3-57A7-99A5-572DFDA0DCB8

**Figure 61.**
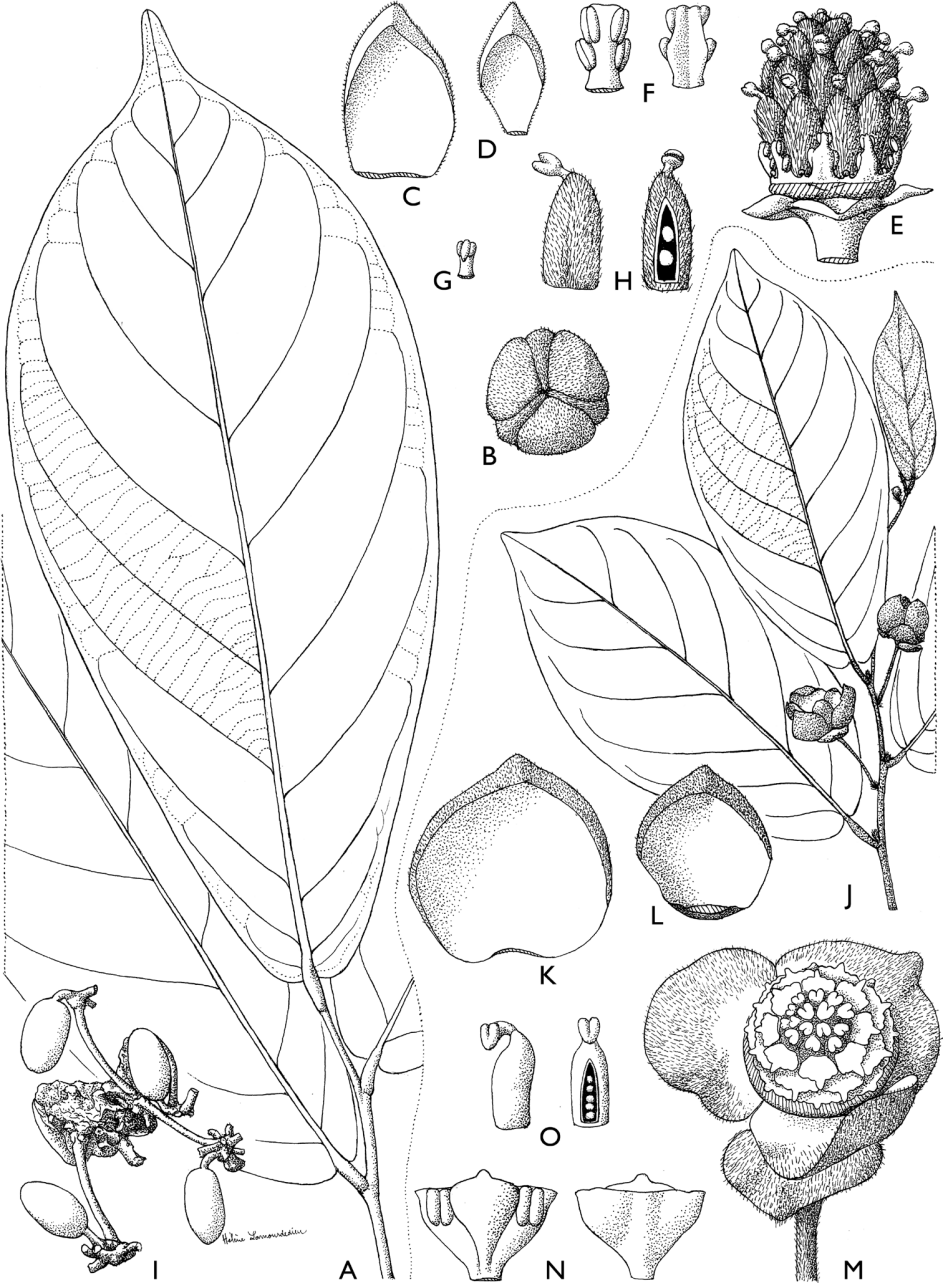
Monanthotaxisklaineivar.lastoursvillensis**A** leaves **B** flower bud seen from above **C** outer petal inner side view **D** inner petal, inner view **E** flower with petals removed **F** stamen, inner outter views **G** staminodes **H** carpel and longitudinal section of carpel. Monanthotaxisklaineivar.klainei (not in Cameroon) **I** fruits. *Monanthotaxislaurentii***J** flowering branch **K** outer petal inner side view **L** inner petal, inner view **M** flower with petals removed **N** stamens outer and inner views **O** carpel and longitudinal section of carpel **A–H** from *Klaine 2662***I** from *Klaine 1539***J–O** from *Le Testu 4512*. Drawings by Hélène Lamourdedieu, Publications Scientifiques du Muséum national d’Histoire naturelle, Paris; modified from Le Thomas (1969b, pl. 39, p. 219).

**Figure 62.**
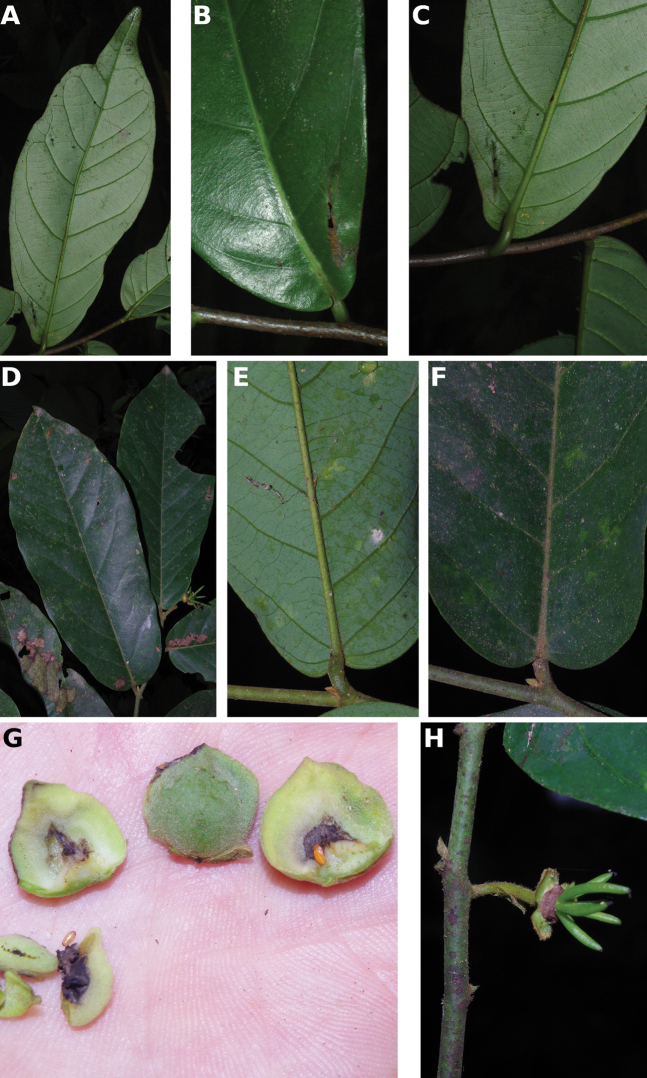
Monanthotaxisklaineivar.lastoursvillensis**A** leaf, lower side **B** base of leaf blade, upper side **C** base of leaf blade, lower side. *Monanthotaxislaurentii***D** Leaf, upper side **E** base of leaf blade, lower side **F** base of leaf blade, upper side **G** outer petals, inner and outer views, and inner petals, lower part of photo **H** young fruit, sepals persistent **A–C***Couvreur 599*, Gabon **D–H***Couvreur 1056*, Mt Cameroon. Photos Thomas L.P. Couvreur.

Figs 61 , 62 ; Map 8C

≡ Popowia lastoursvillensis Pellegr., Bull. Soc. Bot. France 96: 213, 1949; ﻿Popowiaklaineivar.lastoursvillensis (Pellegr.) Le Thomas, Adansonia 3: 290, 1963.

#### Type.

Gabon. Ogooué-Lolo; région de Lastoursville, *Le Testu G.M.P.C. 8595*, 19 Dec 1930: lectotype, chosen by Le Thomas (1969b), p. 221: P[P00362624, P00362625]; isolectotypes: BM[BM000553844]; BR[BR0000008823748, BR0000008823847].

#### Description.

Liana, 7 m tall, d.b.h. up to 4 cm. Indumentum of simple hairs; old leafless branches glabrous, **young foliate branches pubescent with very short appressed yellowish to reddish brown hairs 0.1 mm long to glabrous**. Leaves: petiole 3–9 mm long, 2–3 mm in diameter, glabrous, grooved, blade inserted on top of the petiole; blade 9.8–26.8 cm long, 4.6–10.8 cm wide, **obovate**, apex acuminate to acute, acumen ca. 1.5 cm long, base rounded to subcordate, papyraceous, below sparsely pubescent when young and old, above glabrous when young and old, discolorous, whitish below; midrib sunken or flat, above glabrous when young and old, below sparsely pubescent when young and old; secondary veins 7 to 10 pairs, glabrous above; tertiary venation percurrent. Individuals bisexual; inflorescences cauliflorous or ramiflorous on old leafless branches, axillary. Flowers with 9 perianth parts in 3 whorls, 1 to 3 per inflorescence; pedicel 0.5–3 mm long, ca. 1 mm in diameter, densely pubescent with short appressed reddish brown hairs; in fruit 1–10 mm long, ca. 1 mm in diameter; basal bract 0.5–1 mm long, 0.5–1 mm wide; upper bract 1–1.5 mm long, ca. 0.5 mm wide; sepals 3, valvate, connate, ca. 1 mm long, ca. 1 mm wide, ovate, apex obtuse, base truncate, pubescent outside, glabrous inside, margins flat; petals free, outer petals longer than inner, **inner petals partly covered in bud**; outer petals 3, 4.5 mm long, 2.6–2.9 mm wide, elliptic to ovate, apex acute, base truncate, red to yellow, margins flat, pubescent outside, glabrous inside; inner petals 3, valvate, 3.3–3.7 mm long, 1.2–1.5 mm wide, elliptic, apex acute, base truncate, margins flat, pubescent outside, glabrous inside; **stamens 9, in 1 row**, ca. 1 mm long, oblong; **thecae on the lateral inner side with transversal dehiscence**; connective truncate, glabrous; **staminodes 9**, in one whorl alternating with the stamens, ca. 0.3 mm long, glabrous; **carpels free, 16 to 26**, ovary 0.5–1 mm long, stigma globose, glabrous. Monocarps stipitate, stipes 1–4 mm long, ca. 2 mm in diameter; monocarps 1 to 8, ca. 17 mm long, 7–8 mm in diameter, moniliform, ellipsoid, apex rounded to apiculate, pubescent, verrucose, yellow to red when ripe; **seed 1 per monocarp**, 10–12 mm long, 6–7 mm in diameter, ellipsoid; aril absent.

#### Distribution.

A central African species, from Cameroon to Angola; in Cameroon known from the East and South regions.

#### Habitat.

A rare taxon in Cameroon; in lowland primary rain forests, gallery forests, along river banks and forest edges along savannas. Altitude 100–450 m a.s.l.

#### Local and common names known in Cameroon.

None recorded.

#### Preliminary IUCN conservation status.

Least Concern (LC) (Hoekstra et al. 2021).

#### Uses in Cameroon.

None reported.

#### Notes.

*M.klainei* is distinguished by its shortly and sparsely pubescent or glabrous branches and leaves, clearly obovate leaves which can be quite large (up to 26 cm long) and by having 9 stamens in a single row, with transversally dehiscent thecae. It has more carpels (16 to 26) than closely related species, such as ﻿*M.vogelii* (8 to 12) and *M.aquila* P.H.Hoekstra (12 to 13) and it has more staminodes (9 vs 6). However, *M.aquila* is not present in Cameroon (only known from the type in Ivory Coast)

The variety *klainei* (known from Gabon and the Democratic Republic of the Congo, not found in Cameroon) was distinguished from the variety *lastoursvillensis* by Le Thomas (1969b, as Popowiaklainiivar.lastroursvillensis, p. 218) based on longer pedicels and a larger number of flowers per inflorescences when compared to var. klainei. Based on more material Hoekstra et al. (2021) showed that the number of flowers per inflorescence is not a distinctive character. However, seed shape, globose in Congolese specimens of var. klainei and ellipsoid in var. lastoursvillensis, was added as an important distinction.

#### Specimens examined.

**East Region**: bezirk Molundu, 2.5°N, 15.25°E, *19 January 1911*, *Mildbraed G.W.J.* 4286 (HBG). **South Region**: Bipindi, 3.05°N, 10.25°E, *01 January 1904*, *Zenker G.A.* 2977 (BM,E).

### ﻿﻿﻿﻿ Monanthotaxis laurentii

Taxon classificationPlantaeMagnolialesAnnonaceae

(De Wild.) Verdc., Kew Bull. 25(1): 26, 1971

66647F38-97A5-5AEB-AED3-36B8A7DD72E9

Figs 61 , 62 ; Map 8D

≡ Popowia laurentii De Wild., Enum. Pl. Laurent: 19, 1905.
= Unona congensis Engl. & Diels, Notizbl. Königl. Bot. Gart. Berlin 2: 296, 1899; ﻿Popowiacongensis (Engl. & Diels) Engl. & Diels, Monogr. Afr. Pfl. 6. 44, 1901. Type. Democratic Republic of the Congo. Equateur, Bomongo, Bangala, *Laurent E. s.n.*, 20 Feb 1896: holotype: BR[BR0000008804686].

#### Type.

Democratic Republic of the Congo. Equateur; Bikoro, *Laurent E. 92*, 20 Feb 1896: holotype: BR[BR0000008805010].

#### Description.

Shrub to liana, 20 m tall, d.b.h. unknown. Indumentum of simple hairs; **old leafless branches glabrescent, reddish brown**, **young foliate branches sparsely pubescent with appressed to ascending yellowish hairs 0.2–0.3 mm long**. Leaves: petiole 3–7 mm long, 1 mm in diameter, pubescent, slightly grooved, blade inserted on top of the petiole; blade 4.7–18.3 cm long, 2.4–7.2 cm wide, obovate to oblanceolate, apex acuminate to acute, acumen up to 2 cm long, base rounded to subcordate, papyraceous, below sparsely pubescent when young and old, above sparsely pubescent when young and old, discolorous, whitish below; midrib impressed, above pubescent when young and old, below pubescent when young and old; secondary veins 7 to 14 pairs, sparsely pubescent above; tertiary venation percurrent. Individuals bisexual; inflorescences ramiflorous on old leafless branches, leaf opposed or extra axillary. Flowers with 9 perianth parts in 3 whorls, 1 to 2 per inflorescence; pedicel 5–11 mm long, ca. 1 mm in diameter, sparsely pubescent; in fruit 15–23 mm long, 1–2 mm in diameter; basal bract ca. 1 mm long, ca. 0.5 mm wide; upper bracts 1–2 mm long, 1–2 mm wide; sepals 3, valvate, shortly fused basally or free, 1–2 mm long, ca. 3 mm wide, ovate, apex rounded, base truncate, green, densely pubescent outside, glabrous inside, margins flat; petals free, outer petals longer than inner, inner petals entirely covered in bud; outer petals 3, 6–8 mm long, 5.2–8.1 mm wide, ovate, apex obtuse then shortly acuminate, base truncate, green, margins flat, densely to sparsely pubescent outside, glabrous but pubescent towards the margins inside; inner petals 3, valvate, 4.5–5.3 mm long, 3.5–3.9 mm wide, ovate to rhombic, apex obtuse but then shortly acuminate, base truncate, green, margins flat, densely pubescent outside, pubescent with a glabrous base inside; **stamens 23 to 24, in 3 rows**, ca. 1 mm long, obovate; connective truncate, glabrous; staminodes absent; carpels free, 9 to 12, ovary ca. 2 mm long, stigma elongate, **glabrous**. Monocarps stipitate, stipes 10–23 mm long, 2–3 mm in diameter; monocarps 3 to 11, 25–85 mm long, 5–6 mm in diameter, moniliform, cylindrical, apex apiculate, glabrous, verrucose, constricted around seeds when more than 1, yellow to orange when ripe; **seeds 1 to 6 per monocarp, 14–19 mm long, 5–6 mm in diameter, subcylindrical**; aril absent.

#### Distribution.

A widespread West and Central African species, from Sierra Leone to Nigeria and Cameroon to the Democratic Republic of the Congo and Angola; in Cameroon known from the Central, North and South-West regions.

#### Habitat.

A common species when present; in primary or old secondary lowland or submontane forests, forest pockets in savanna and gallery forests. Altitude 300–1000 m a.s.l.

#### Local and common names known in Cameroon.

None recorded.

#### Preliminary IUCN conservation status.

Least Concern (LC) (Hoekstra et al. 2021).

#### Uses in Cameroon.

None reported.

#### Notes.

﻿﻿﻿*Monanthotaxislaurentii* is distinguished by its reddish brown branches with a pubescence of scattered short yellow hairs, 23 to 24 stamens in 3 whorls, glabrous carpels, and long subcylindrical seeds.

#### Specimens examined.

**Central Region**: Eloumden 10 km SW of Yaoundé, 3.49°N, 11.26°E, *06 February 1996*, *Nkongmeneck B.A.* 1326 (MO). **North Region**: Ngoussou, 6.03°N, 11.23°E, *01 February 1939*, *Jacques-Félix H.* 3226 (P). **South-West Region**: Nyasoso village on max’s trail to Mt 4.82°N, 9.694°E, *05 April 2016*, *Couvreur T.L.P.* 1056 (WAG,YA); on top of hill near Small Ekombe village 3 km after Kumba on road to Ekondo Titi town, 4.62°N, 9.376°E, *13 January 2016*, *Couvreur T.L.P.* 981 (WAG,YA); Ndabekim Hill to west of village, 4.91°N, 9.716°E, *15 December 1999*, *Etuge M.* 4676 (K,MO,P,WAG,YA); Mt Cameroun south slope W of Victoria Transect 5, 4.07°N, 9.015°E, *16 November 1985*, *Gentry A.H.* 52947 (MO).

### ﻿﻿﻿﻿ Monanthotaxis letouzeyi

Taxon classificationPlantaeMagnolialesAnnonaceae

(Le Thomas) Verdc., Kew Bull. 25(1): 31, 1971

DADC3DCF-57FD-5664-9098-FEB1BC4BFD59

**Figure 63.**
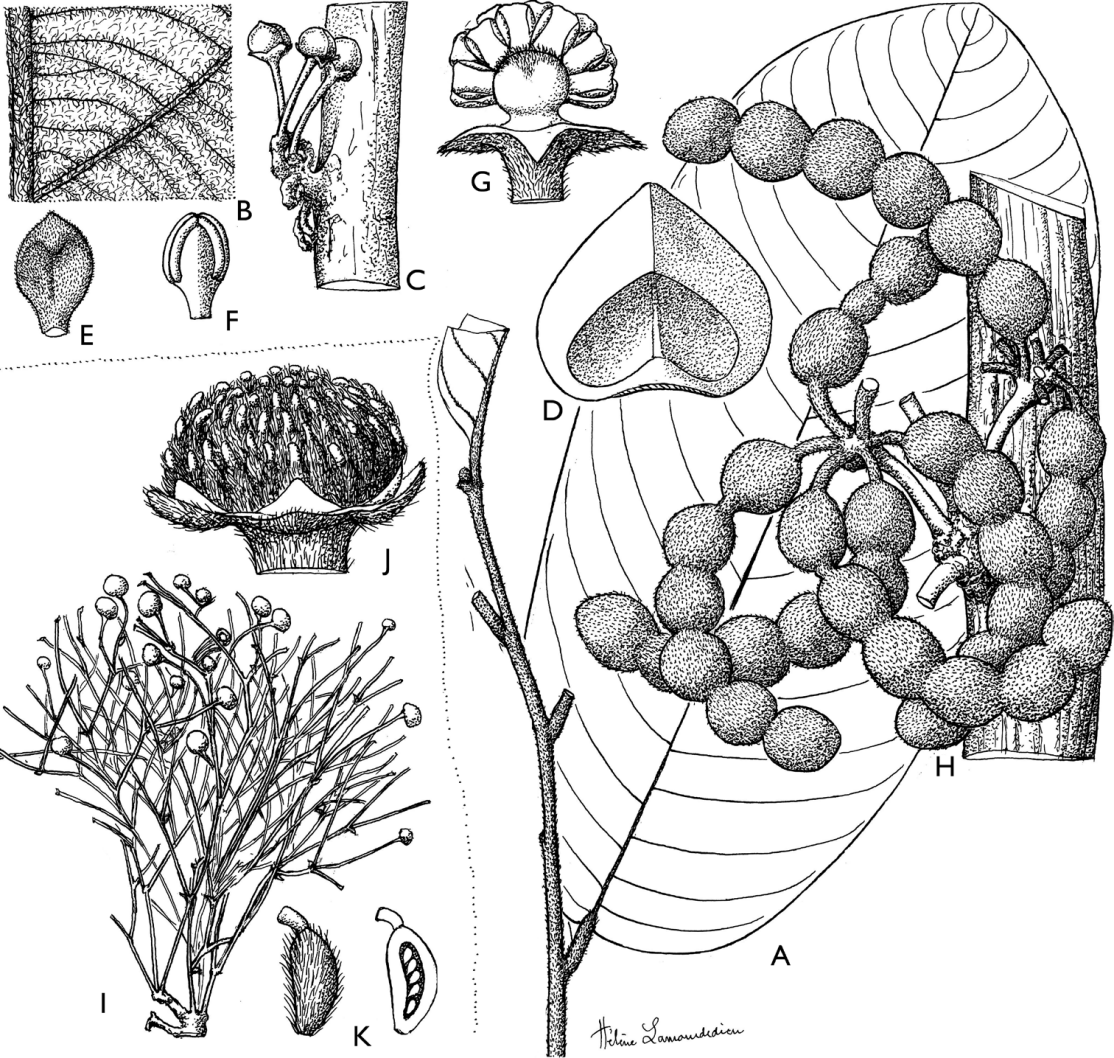
*Monanthotaxisletouzeyi***A** leaf, upper side **B** detail of leaf pubescence, loer side **C** male flowers, cauliflorous **D** outer petal, inner view **E** inner petal, inner view **F** stamen, front view **G** flower, all petals removed, and a few stamens too **H** fruit, cauliflorous. *Monanthotaxiscauliflora***I** female inflorescence **J** female flower, all petals removed **K** carpel, side view and detail of oules **A–G** from *Letouzey 3066***H** from *Letouzey 5403***I–K** from *Zenker 356*. Drawings by Hélène Lamourdedieu, Publications Scientifiques du Muséum national d’Histoire naturelle, Paris. (This drawing originally had sketches from *Lebrun 6134* of a flowering branch with male flowers and detailed drawings of a male flower, however Hoekstra et al. (2021) indicated that *Lebrun 6134* is in fact *Monanthotaxisconfusa* Hoekstra, and so was removed from the drawing).

**Figure 64.**
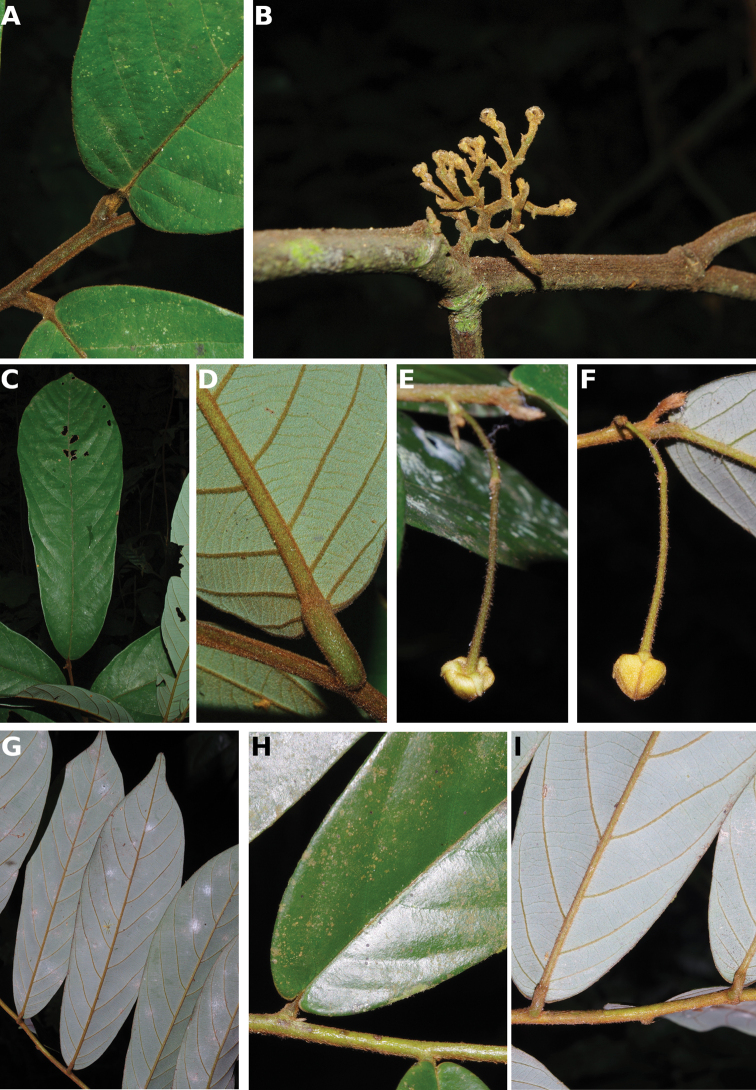
*Monanthotaxisletouzeyi***A** base of leaf blade, upper view **B** cauliflorous inflorescence **C** leaf, upper view **D** base of leaf blade, lower view. *Monanthotaxismontana***E** flower, basal view, note small sepals **F** flower, side view **G** leaves, top view **H** base of leaf blade, upper side **I** base of leaf blade, lower side **A–D***Couvreur 458*, Lélé, Cameroon **E–I***Couvreur 891*, Gabon. Photos Thomas L.P. Couvreur.

Figs 63 , 64 ; Map 8E

≡ Popowia letouzeyi Le Thomas, Adansonia sér. 2, 8: 241, 1968.

#### Type.

Cameroon. East Region; Nkoum, *Letouzey R.G. 3066*, 19 Feb 1960: holotype P[P00362617]; isotype: YA[YA0002635].

#### Description.

Liana, 30 m tall, d.b.h. unknown. Indumentum of simple hairs; old leafless branches glabrescent, **young foliate branches pubescent completely covered with dense erect reddish brown hairs 0.4 mm lon**g. Leaves: petiole 6–15 mm long, 2–3 mm in diameter, densely pubescent with erect reddish brown hairs, weakly grooved adaxially, blade inserted on top of the petiole; blade 6–23 cm long, 6–12.2 cm wide, oblong to oblanceolate, apex rounded to emarginate, acumen ca. 1 cm long, **base cordate** (to rounded), papyraceous, below pubescent when young and old, above sparsely pubescent when young, glabrous when old, discolorous, whitish below; midrib sunken or flat, above densely pubescent when young and old, below densely pubescent when young and old; secondary veins 14 to 19 pairs, sparsely pubescent above; tertiary venation percurrent. **Individuals unisexual, dioecious, male and female inflorescences dimorphic, male inflorescences cauliflorous with 3 to 50 flowers, in a fascicle or glomerule, peduncle 5–25 mm long; female inflorescences cauliflorous, in a condensed panicle with many flowers, peduncle 25–40 mm long**. Flowers with 9 perianth parts in 3 whorls, male and female flowers dimorphic, male flowers: pedicel 6–10 mm long, ca. 1 mm in diameter, densely pubescent; basal bract ca. 1 mm long, ca. 1 mm wide; upper bract ca. 1 mm long, ca. 1 mm wide; sepals 3, valvate, basally fused, ca. 1 mm long, ca. 1 mm wide, broadly ovate, apex rounded, base truncate, brown, densely pubescent outside, glabrous inside, margins flat; petals shortly connate basally, outer petals longer than inner; outer petals 3, ca. 3 mm long, ca. 5 mm wide, ovate, apex acute, base truncate, margins flat, densely pubescent outside, papillose and sparsely pubescent with a few very short hairs inside; inner petals 3, valvate, 1.5–2 mm long, ca. 1 mm wide, elliptic to ovate, apex acute, base truncate, margins flat, papillose, sparsely pubescent towards the apex outside, papillose, sparsely pubescent towards the apex outside inside; **stamens 31 to 40, in ca. 4 rows**, ca. 1 mm long, oblong; connective truncate, pubescent; **staminodes absent**. Female flowers: pedicel 8–15 mm long, 1–2 mm in diameter, densely pubescent; in fruit 10–30 mm long, 2 mm in diameter; bracts 2, one basal and one upper towards the lower half of pedicel, basal bract 1–1.5 mm long, ca. 1 mm wide; upper bract ca. 1 mm long, ca. 1 mm wide; sepals 3, valvate, basally fused, 1–2 mm long, ca. 1–2 mm wide, broadly ovate, apex acuminate, base truncate, densely pubescent outside, densely pubescent inside, margins flat; petals free, outer petals longer than inner, inner petals entirely covered in bud; outer petals 3, 5–6 mm long, 4.8–6 mm wide, broadly ovate, apex obtuse, base truncate, margins flat, densely pubescent with short papillose hairs outside, pubescent with short papillose hairs inside; inner petals 3, valvate, ca. 2 mm long, 1.2–1.4 mm wide, spathulate, apex obtuse, base truncate, margins flat, pubescent with short papillose hairs outside, pubescent with short papillose hairs inside; carpels free, 120, ovary ca. 1 mm long, stigma ellipsoid to globose, glabrous. Monocarps stipitate, stipes 7–10 mm long, 2–3 mm in diameter; monocarps at least 3 (but potentially with many more, up to 100), 20–80 mm long, 9–10 mm in diameter, moniliform, globose, apex rounded, densely pubescent, smooth, constricted around seeds when more than 1, blackish green when ripe; seeds 1 to 6 per monocarp, ca. 9 mm long, c. 9 mm in diameter, ellipsoid; aril absent.

#### Distribution.

A central African species, from Cameroon to the Republic of the Congo; in Cameroon known from the East and South regions.

#### Habitat.

An uncommon species; in primary and old secondary rain forests, gallery forests and swamp forests, on sandy clay. Altitude 300–750 m a.s.l.

#### Local and common names known in Cameroon.

None recorded.

#### Preliminary IUCN conservation status.

Least Concern (LC) (Hoekstra et al. 2021).

#### Uses in Cameroon.

None reported.

#### Notes.

﻿﻿﻿*Monanthotaxisletouzeyi* is distinguished by the dense erect reddish brown hairs on the young foliate branches and petioles, the generally cordate leaf base, unisexual flowers with male and female inflorescences both being cauliflorous. This species is morphologically close to ﻿*M.diclina*, but this latter has yellow-brown hairs, male inflorescences that are axillary (not cauliflorous) and 6 stamens with 12 staminodes (versus 31 to 40 stamens and no staminodes in ﻿﻿﻿*Monanthotaxisletouzeyi*).

#### Specimens.

**East Region**: Bertoua, 4.56°N, 13.65°E, *05 September 1961*, *Breteler F.J.* 1874 (P,U,WAG,YA); Palisco forest consession 15 km along main road into consession, 3.52°N, 13.54°E, *27 March 2015*, *Couvreur T.L.P.* 752 (WAG,YA); Nkoum, 4.1°N, 13.17°E, *19 February 1960*, *Letouzey R.* 3066 (P,YA); A 23 km à l’W de Masea (village situé à 50 km au SSW de Yokadouma), 3.15°N, 14.87°E, *04 July 1963*, *Letouzey R.* 5403 (P,YA); Eden 48 km from Lomie, 3.25°N, 13.28°E, *15 April 1996*, *Nzooh Dongmo Z.L.* 578 (MO). **South Region**: 16 km east from Lélé village, 2.28°N, 13.32°E, *07 September 2013*, *Couvreur T.L.P.* 458 (WAG,YA); Piste Meyo Ntem-Evouzok 75 km W Ambam Entre 1er et 3e bras du Ntem, 2.17°N, 10.37°E, *28 November 1979*, *Letouzey R.* 15270 (P,YA).

### ﻿﻿﻿﻿ Monanthotaxis montana

Taxon classificationPlantaeMagnolialesAnnonaceae

(Engl. & Diels) P.H.Hoekstra, Taxon 66: 15, 2017

7D80F012-9AEB-5BE7-915C-159DF2BCC71D

Figs 59 , 64 ; Map 8F

≡ Unona montana Engl. & Diels, Notizbl. Königl. Bot. Gart. Berlin 2: 296, 1899; ﻿Oxymitramontana (Engl. & Diels) Sprague & Hutch., Bull. Misc. Inform. Kew: 155, 1916; ﻿Richellamontana (Engl. & Diels) R.E.Fr., in Prantl. & Engler Nat. Pflanzenfam. ed. 2, 17a (2): 139, 1959; ﻿﻿Friesodielsiamontana (Engl. & Diels) Steenis, Blumea 12: 360 1964.
= Unona glauca Engl. & Diels (non Zipp.), Notizbl. Königl. Bot. Gart. Berlin 2: 296, 1988; ﻿Oxymitrasoyauxii Sprague & Hutch., Bull. Misc. Inform. Kew 6: 155, 1916; ﻿Richellasoyauxii (Sprague & Hutch.) R.E.Fr., in Prantl. & Engler Nat. Pflanzenfam., ed. 2, 17a (2): 139, 1959. — ﻿﻿Friesodielsiasoyauxii (Sprague & Hutch.) Steenis, Blumea 12: 361, 1964. Type. Gabon. Estuaire, Sibange farm, Soyaux H. 203, 6 Feb 1881: lectotype, designated by Guo et al. (2017b), p. 15: B[B100153059]; isolectotype: K[K000198946].
= Oxymitra mortehanii De Wild., Pl. Bequaert. 1: 472, 1922. Type. Democratic Republic of the Congo. Mongala, Dundusana, *Mortehan M.G. 512*, Sep 1913: holotype: BR[BR000008800459, BR000008800060, BR000008800787].

#### Type.

Cameroon. Central Region; Yaoundé-Station, *Zenker, G.A. 431*, 11 Jan 1894: holotype: B[B100153061].

#### Description.

Shrub to liana, up to 7 m tall, d.b.h. up to 1 cm. Indumentum of simple hairs; old leafless branches glabrescent, young foliate branches densely pubescent with dense appressed to ascending reddish brown hairs 0.2–0.3 mm long. Leaves: petiole 3–6 mm long, 1–2 mm in diameter, densely pubescent, slightly grooved, blade inserted on top of the petiole; blade 5.5–20 cm long, 2.8–6.7 cm wide, elliptic to oblanceolate, apex acuminate to acute, acumen 1.5 cm long, base subcordate, subcoriaceous, below pubescent when young and old, above sparsely pubescent to glabrous when young and old, **grey when dried**, discolorous, whitish below; midrib impressed, above sparsely pubescent when young, glabrous when old, below pubescent when young and old; secondary veins 7 to 13 pairs, glabrous above; tertiary venation percurrent. Individuals bisexual; inflorescences ramiflorous on old leafless branches, extra axillary. Flowers with 9 perianth parts in 3 whorls, 1 to 4 per inflorescence; pedicel 13–33 mm long, 0.5–1 mm in diameter, sparsely pubescent; in fruit 28–38 mm long, 0.5–1 mm in diameter; basal bract ca. 1 mm long, ca. 1 mm wide; upper bract ca. 1 mm long, ca. 1 mm wide; sepals 3, valvate, free, reflexed at anthesis, **1–2 mm long**, 1–2 mm wide, broadly elliptic to broadly ovate, apex acute, base truncate, densely pubescent outside, glabrous inside, margins flat; petals free, outer petals longer than inner, inner petals entirely covered in bud; outer petals 3, 6–15 mm long, 5.4–6.7 mm wide, ovate, apex obtuse, base truncate, yellow-green and red at the base, margins flat, sparsely pubescent outside, pubescent with a glabrous base inside; inner petals 3, valvate, 5.8–7.5 mm long, 4.5–9 mm wide, cordate, apex obtuse, base truncate, margins flat, glabrous outside, glabrous inside; **stamens 36 to 48, in 5 rows**, ca. 1 mm long, linear to oblong; connective truncate, glabrous; staminodes absent; carpels free, 11 to 14, ovary 1–2 mm long, stigma globose, glabrous. Monocarps stipitate, stipes 3–8 mm long, 2–3 mm in diameter; monocarps 4 to 11, 12–33 mm long, 4–9 mm in diameter, moniliform, ellipsoid, apex rounded to apiculate, pubescent to glabrous, verrucose, constricted around seeds when more than 1, yellow with red stripes or dull red when ripe; seeds 1 to 3 per monocarp, 11–12 mm long, 5–6 mm in diameter, ellipsoid; aril absent.

#### Distribution.

A central African species from Cameroon to the Democratic Republic of the Congo, Central African Republic and Angola; in Cameroon known from the Central and South-West regions.

#### Habitat.

A rare species in Cameroon, only known from two collections; in primary or old secondary rain forests, swamp forests, gallery forests, on rocky soil and along river banks. Altitude: 500–700 m a.s.l.

#### Local and common names known in Cameroon.

None recorded.

#### Preliminary IUCN conservation status.

Least Concern (LC) (Hoekstra et al. 2021).

#### Uses in Cameroon.

None reported.

#### Notes.

﻿﻿﻿*Monanthotaxismontana* is distinguished by the upper side of the leaf blade which is grey in dried material, sepals that are small (1–2 mm long) and reflexed at anthesis, and stamens 36 to 48 in 5 rows.

#### Specimens examined.

**Central Region**: Yaoundé, 3.86°N, 11.51°E, *1894*, *Zenker G.A.* 431 (B). **South-West Region**: Njonji, 4.13°N, 9.033°E, *17 April 1997*, *Nning J.* 360 (K,YA).

### ﻿﻿﻿﻿ Monanthotaxis paniculata

Taxon classificationPlantaeMagnolialesAnnonaceae

P.H.Hoekstra, Phytotaxa 186(2): 106, 2014

2ECCFC94-F74E-584B-8031-7722EF20F776

**Figure 65.**
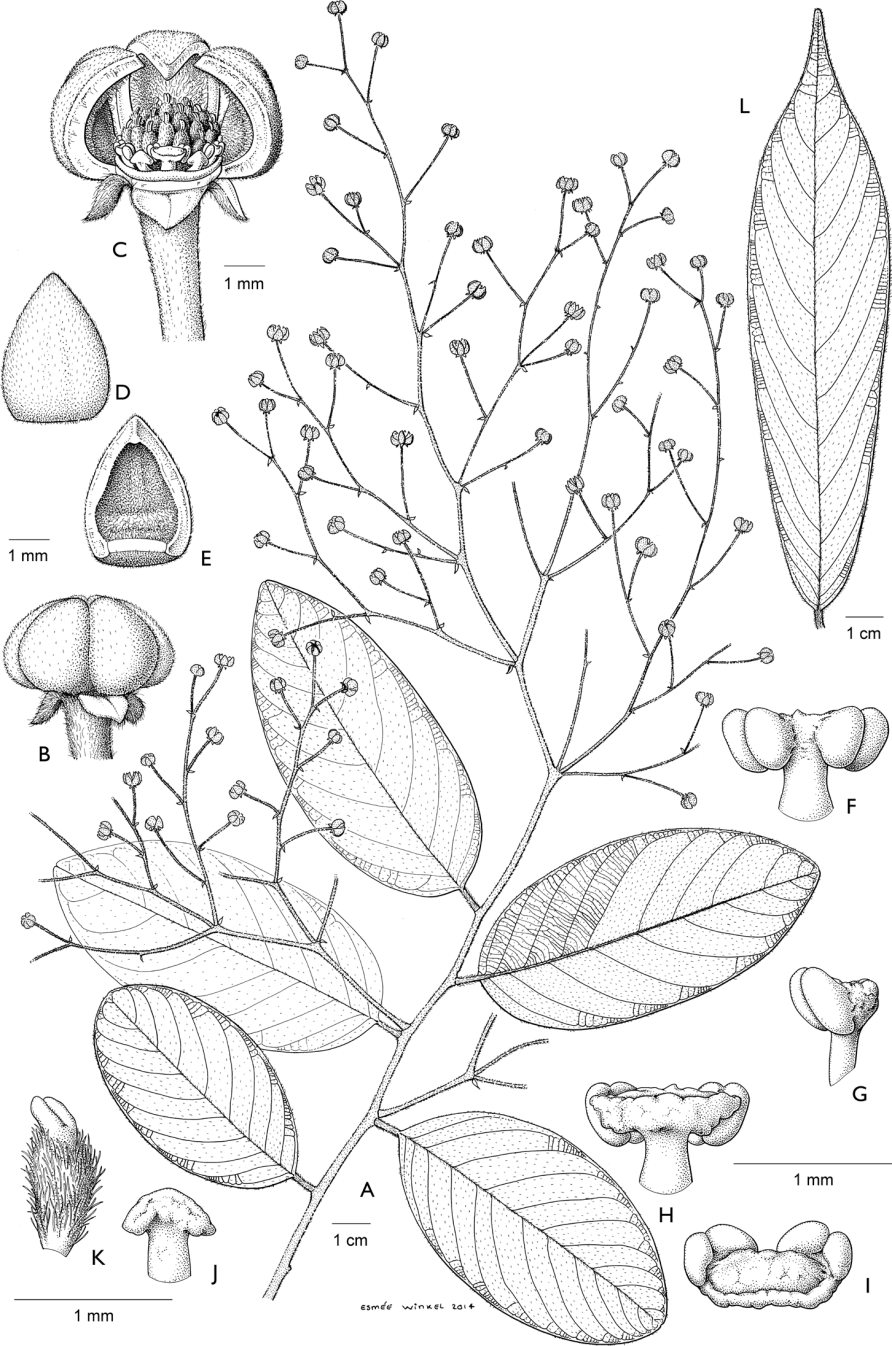
*Monanthotaxispaniculata***A** flowering branch **B** flower bud **C** flower with three petals removed **D** petal outer, side view **E** petal, inner view **F** stamen inner, side view **G** stamen, side view **H** stamen, outer view **I** stamen, top view **J** staminode **K** carpel **A–J** from *G.D. McPherson 16123***K** from *Reitsma 2870*. Drawings by Esmée Winkel (Hoekstra et al. 2021, fig. 23, p. 186).

**Figure 66.**
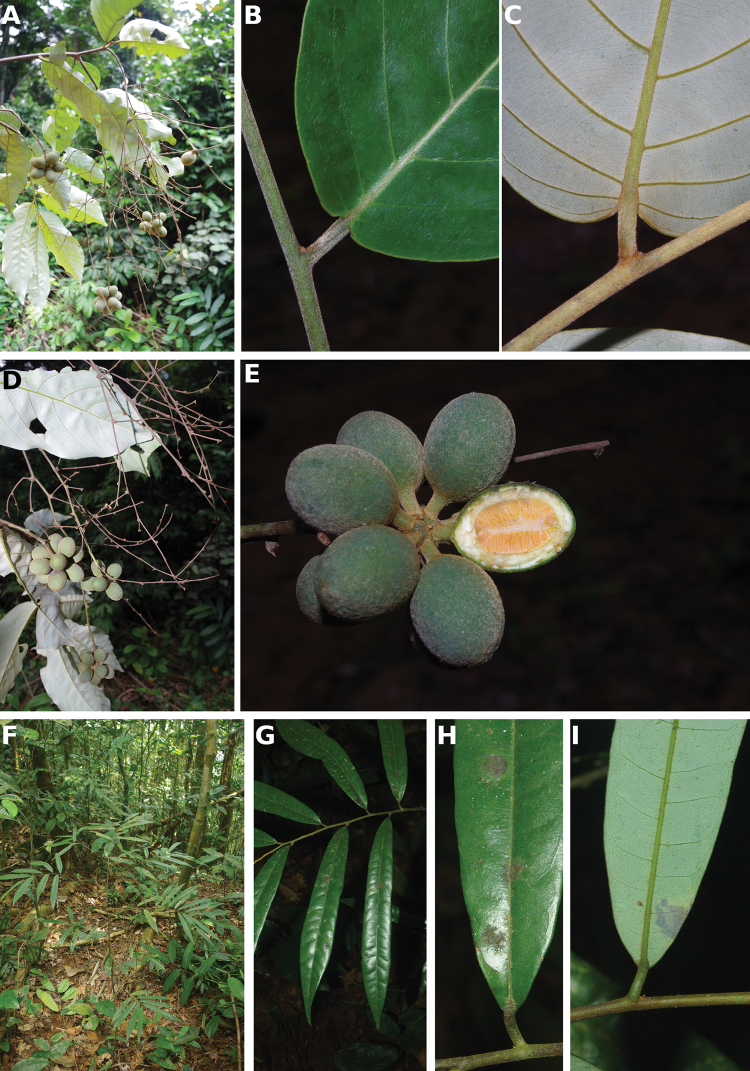
*Monanthotaxispaniculata***A** fruiting branch, note long paniculate inflorescences **B** base of leaf blade, upper view **C** base of leaf blade, lower view **D** fruiting branch, note long paniculate inflorescences **E** fruit, with longitudinal section of one monocarp. *Monanthotaxissterilis***F** habit, juvenile **G** leaves, upper side, note linear blades **H** base of leaf blade, upper view **I** base of leaf blade, lower view **A–E***Couvreur 1108*, Gabon **F–I***Couvreur 628*, Ebo, Cameroon. Photos Thomas L.P. Couvreur.

Figs 65 , 66 ; Map 8G

#### Type.

Gabon. Ogooué-Ivindo; north of Koumameyong along SHM lumber roads, *McPherson G.D. 16123*, 31 Jan 1993: holotype: WAG[WAG0357246, WAG0357247]; isotypes: LBV *n.v.*, MO *n.v.*, P[P01967243].

#### Description.

Liana, 20 m tall, d.b.h. unknown. Indumentum of simple hairs; old leafless branches glabrescent, young foliate branches densely pubescent with appressed reddish-brown hairs 0.5 mm long. Leaves: petiole 4–8 mm long, ca. 1 mm in diameter, densely pubescent, slightly grooved, blade inserted on top of the petiole; blade 8.5–23.5 cm long, 3.3–6.6 cm wide, ovate to oblanceolate, apex acuminate to acute, acumen 0.5–2.7 cm long, base cuneate to broadly cuneate with small linear black glands, chartaceous, **below densely pubescent with yellowish hairs when young**, sparsely pubescent to glabrous when old, above sparsely pubescent when young, glabrous when old, discolorous, whitish below; midrib impressed, above glabrous when young and old, below sparsely pubescent when young and old; secondary veins 10 to 16 pairs, glabrous above; tertiary venation percurrent. Individuals bisexual; inflorescences ramiflorous on old leafless branches, axillary or terminal. Flowers with 9 perianth parts in 2 whorls, **numerous per inflorescence, peduncle 5.5–27 cm long, panicle-like**; pedicel 5–27 mm long, 0.5–1 mm in diameter, densely pubescent with short reddish brown hairs; in fruit 20–30 mm long, 2–3 mm in diameter, pubescent; bracts 2, one basal and one upper towards the middle or lower half of pedicel, basal bract 1–8 mm long, ca. 1 mm wide; upper bract ca. 1 mm long, ca. 1 mm wide; sepals 3, valvate, free, 0.5–1 mm long, 1–1.5 mm wide, broadly ovate, apex obtuse, base truncate, densely pubescent outside, glabrous inside, margins flat; **petals free, petals 6 in one whorl**, ca. 3 mm long, ca 1.5 mm wide, ovate, apex rounded to obtuse, base truncate, margins flat, sparsely pubescent outside, sparsely pubescent inside; **stamens 6, in 1 row, inserted on a black hexagonal disc**, opposite to the petals, free at the base, ca. 1 mm long, obconic; **connective truncate, kidney-shaped in dorsal view**, glabrous; staminodes 6, alternating with the stamens, glabrous; carpels free, 14 to 24, ovary ca. 2 mm long, stigma shortly bilobed, acute, glabrous. Monocarps stipitate, stipes 3–4 mm long, 3–4 mm in diameter; monocarps 2 to10, 15–30 mm long, 10–15 mm in diameter, **not moniliform**, ellipsoid, apex rounded, pubescent, smooth, green when ripe; **seed 1 per monocarp**, ca. 10 mm long, ca. 8 mm in diameter, ellipsoid; aril absent.

#### Distribution.

A central African species from Gabon and Cameroon; in Cameroon known from Littoral and South regions.

#### Habitat.

A rare species in Cameroon; in lowland primary or old secondary rain forests. Altitude 300–350 m a.s.l.

#### Local and common names known in Cameroon.

None recorded.

#### Preliminary IUCN conservation status.

Endangered (EN) (Hoekstra et al. 2021).

#### Uses in Cameroon.

None reported.

#### Notes.

﻿﻿﻿*Monanthotaxispaniculata* is distinguished by the long appressed yellowish pubescence on lower side of the leaf blade, its inflorescence in a large panicle-like rhipidium (unique in the genus) and its flowers with one whorl of 6 petals. It is morphologically close to ﻿*M.congoensis*, but differs by its panicle-like inflorescence, depressed-globose floral buds, stamens inserted on a black hexagonal disc, and connective appendage kidney-shaped in dorsal view.

Fruits were unknown when the species was first published (Hoekstra et al. 2014) but have since been collected (*Couvreur 1108*) and fruits are described here for the first time.

The presence of ﻿﻿﻿*Monanthotaxispaniculata* in Cameroon is only based on two sterile collections, and needs to be confirmed with fertile material.

#### Specimens examined.

**Littoral Region**: Mambe Massif above Boga village 100 km along road from Yaoundé to Ed 3.90°N, 10.77°E, *19 June 2014*, *Couvreur T.L.P.* 651 (WAG,YA). **South Region**: Campo Ma’an National Park 11 km on trail from Ebinanemeyong village on road 7 km from Nyabessan to Campo town, 2.47°N, 10.34°E, *14 February 2015*, *Couvreur T.L.P.* 708 (WAG,YA).

### ﻿﻿﻿﻿ Monanthotaxis pellegrinii

Taxon classificationPlantaeMagnolialesAnnonaceae

Verdc., Kew Bull. 25(1): 28, 1971

1509D09F-A232-53D8-ACAC-087449650338

Fig. 46 ; Map 8H

≡ Popowia letestui Pellegr., Bull. Soc. Bot. France 96: 213, 1950.

#### Type.

Gabon. Woleu-Ntem; region de Bitam, *Le Testu G.M.P.C. 9028*, 12 Mar 1933: lectotype, chosen by Le Thomas (1969b), p. 226: P[P00362618, P00362621, P00362623]; isolectotypes: BM[BM000553843]; BR[BR0000008823779, BR0000008823786, BR0000008823830].

#### Description.

Shrub scrambling when young (?) to liana to ca. 3 m tall, d.b.h. unknown. Indumentum of simple hairs; old leafless branches glabrescent, **young foliate branches densely pubescent with dense ascending reddish brown hairs 0.1–0.3 mm long**. Leaves: petiole 3–9 mm long, ca. 1 mm in diameter, pubescent to glabrous, grooved, blade inserted on the side of the petiole; blade 6.2–22 cm long, 2.8–9 cm wide, elliptic, ovate to obovate, apex acuminate to acute, acumen ca. 1.5 cm long, base cuneate to subcordate, papyraceous, below sparsely pubescent when young and old, above sparsely pubescent when young, glabrous when old, discolorous, whitish below; midrib impressed, above pubescent when young, glabrous to pubescent when old, below pubescent when young and old; secondary veins 6 to 11 pairs, glabrous above; tertiary venation percurrent. Individuals bisexual; inflorescences ramiflorous on old leafless branches, leaf opposed or extra axillary. Flowers with 9 perianth parts in 3 whorls, 1 to 6 per inflorescence; pedicel 16–55 mm long, ca. 1 mm in diameter, sparsely pubescent; in fruit; bracts 2, one basal and one upper towards the lower half of pedicel, basal bract ca. 1 mm long, ca. 0.5 mm wide; upper bract ca. 1 mm long, ca. 1 mm wide; sepals 3, valvate, basally fused, ca. 1 mm long, 2–3 mm wide, ovate, apex acute to rounded, base truncate, sparsely pubescent outside, glabrous inside, margins flat; petals free, outer petals longer than inner, inner petals entirely covered in bud; outer petals 3, 4.7–5.5 mm long, 4.6–5.8 mm wide, ovate, apex acute, base truncate, light yellow, margins flat, densely pubescent outside, pubescent inside; inner petals 3, valvate, 2.5–4 mm long, 2.8–3 mm wide, ovate, apex acute, base truncate, margins flat, densely pubescent outside, pubescent inside; **stamens 14 to 24**, in 1 to 2 rows, 1–2 mm long, oblong; **connective absent or much reduced, filament much wider, thecae convergent towards apex**, glabrous; staminodes absent; carpels free, 10 to 21, ovary ca. 1 mm long, stigma shortly bilobed, acute, glabrous. Fruits unknown.

#### Distribution.

A central African species from Cameroon to Gabon and Central African Republic; in Cameroon known from Adamaoua region.

#### Habitat.

A rare species in Cameroon; in montane forests margins, near roads and on river banks. Altitude 1200–1300 m a.s.l.

#### Local and common names known in Cameroon.

None recorded.

#### Preliminary IUCN conservation status.

Endangered (EN) (Hoekstra et al. 2021).

#### Uses in Cameroon.

None reported.

#### Notes.

﻿﻿﻿*Monanthotaxispellegrinii* is distinguished by the shape of the 14 to 24 stamens with a filament wider than the connective and the thecae converging apically almost hiding the connectives. In this respect, it resembles ﻿﻿﻿*Monanthotaxisbicornis*, but the latter has appressed yellow-brown hairs on the young foliate branches, versus ascending reddish brown hairs young foliate branches in ﻿*M.pellegrinii*.

#### Specimens examined.

**Adamaoua Region**: 21 km NNE de Banyo, 6.77°N, 11.81°E, *08 June 1967*, *Letouzey R.* 8545 (P,YA); Près Gandwa (25 km NNO de Banyo), 6.93°N, 11.72°E, *14 June 1967*, *Letouzey R.* 8648 (P,YA).

### ﻿﻿﻿﻿ Monanthotaxis pynaertii

Taxon classificationPlantaeMagnolialesAnnonaceae

(De Wild.) P.H.Hoekstra, Blumea 66 (1): 191, 2021

EC8CF60F-A385-55FD-BF6B-35640DF341EA

Fig. 67 ; Map 8I

≡ Popowia pynaertii De Wild., Bull. Jard. Bot. État Bruxelles 4: 382, 1914.

#### Type.

Democratic Republic of the Congo. Equateur; Mbandaka, Eala, *Pynaert L.A.E.J. 852*, 20 Dec 1908: lectotype, designated by Hoekstra et al. (2021), p. 191: BR[BR0000008805348, BR0000008805355].

#### Description.

Liana, 20 m tall, d.b.h. up to 2 cm. Indumentum of simple hairs; old leafless branches glabrescent, **young foliate branches pubescent with erect reddish brown hairs 0.6–1.5 mm long**. Leaves: petiole 5–7 mm long, 1–2 mm in diameter, **pubescent with long erect reddish brown hairs**, weakly grooved adaxially, blade inserted on top of the petiole; blade 9.5–23.2 cm long, 2.9–7 cm wide, ovate to oblanceolate, apex acuminate to acute, acumen 0.5–1 cm long, base rounded to subcordate, papyraceous to subcoriaceous, below pubescent with erect yellow hairs when young and old, above sparsely pubescent when young, glabrous when old, discolorous, whitish below; midrib sunken or flat, above densely pubescent when young and old, below pubescent when young and old; secondary veins 11 to 17 pairs, glabrous above; tertiary venation percurrent. **Individuals unisexual, dioecieous, male and female inflorescences dimorphic, male inflorescences axillary, composed of a solitary flower to a few-flowered fascicle with up to 10 flowers; peduncle 4–8 mm long with erect hairs, female inflorescences cauliflorous, a condensed panicle with many flowers; peduncle 35–50 mm long, densely pubescent with erect hairs**; Flowers with 9 perianth parts in 3 whorls, male and female flowers dimorphic. Male flowers: flowering pedicel 2–3 mm long, ca. 1 mm in diameter, pubescent; basal bract ca. 1 mm long, ca. 1 mm wide; upper bract ca. 1 mm long, ca. 1 mm wide; sepals 3, valvate, basally fused, c. 1 mm long, ca. 1 mm wide, triangular to ovate, apex acute, base truncate, pubescent outside, glabrous inside, margins flat; petals free, outer petals longer than inner, inner petals entirely covered in bud; outer petals 3, ca. 3 mm long, 3 mm wide, circular to broadly ovate, apex rounded to obtuse, base truncate, margins flat, densely pubescent outside, pubescent inside; inner petals 3, valvate, ca. 1 mm long, ca. 0.5 mm wide, ovate, apex rounded, base truncate, margins flat, pubescent outside, pubescent inside; stamens 6 (9), in 1 row, ca. 1 mm long, oblong; connective truncate, glabrous; staminodes 12 to 16, in one whorl externally to the stamens, very short, very sparsely pubescent or glabrous. Female flowers: flowering pedicel 20–30 mm long, ca. 1.5 mm in diameter, densely pubescent with erect hairs; in fruit ca. 33 mm long, ca. 2 mm in diameter; basal bract ca. 2–3 mm long, 1.5–2 mm wide; upper bract ca. 1 mm long, ca. 1 mm wide; sepals 3, valvate, basally fused, 1–2 mm long, 1.5–2 mm wide, ovate to broadly ovate, apex acute, base truncate, pubescent outside, glabrous inside, margins flat; petals free, outer petals longer than inner; outer petals 3, 5–6.3 mm long, 5–6.3 mm wide, circular to broadly ovate, apex rounded to obtuse, base truncate, margins flat, densely pubescent outside, pubescent inside; inner petals 3, valvate, 1.7–1.8 mm long, 0.7–0.8 mm wide, ovate, apex rounded, base truncate, margins flat, pubescent outside, pubescent inside; carpels free, 95 to 150, ovary ca. 2 mm long, stigma globose to ellipsoid, glabrous. Monocarps stipitate, stipes 9–12 mm long, ca. 2 mm in diameter; monocarps up to 20, 20–60 mm long, 7–8 mm in diameter, moniliform, ellipsoid to cylindrical, apex apiculate, pubescent, verrucose, constricted around seeds when more than 1, color unknown; seeds 1 to 6 per monocarp, 11–13 mm long, 7–8 mm in diameter, ellipsoid; aril absent.

**Figure 67.**
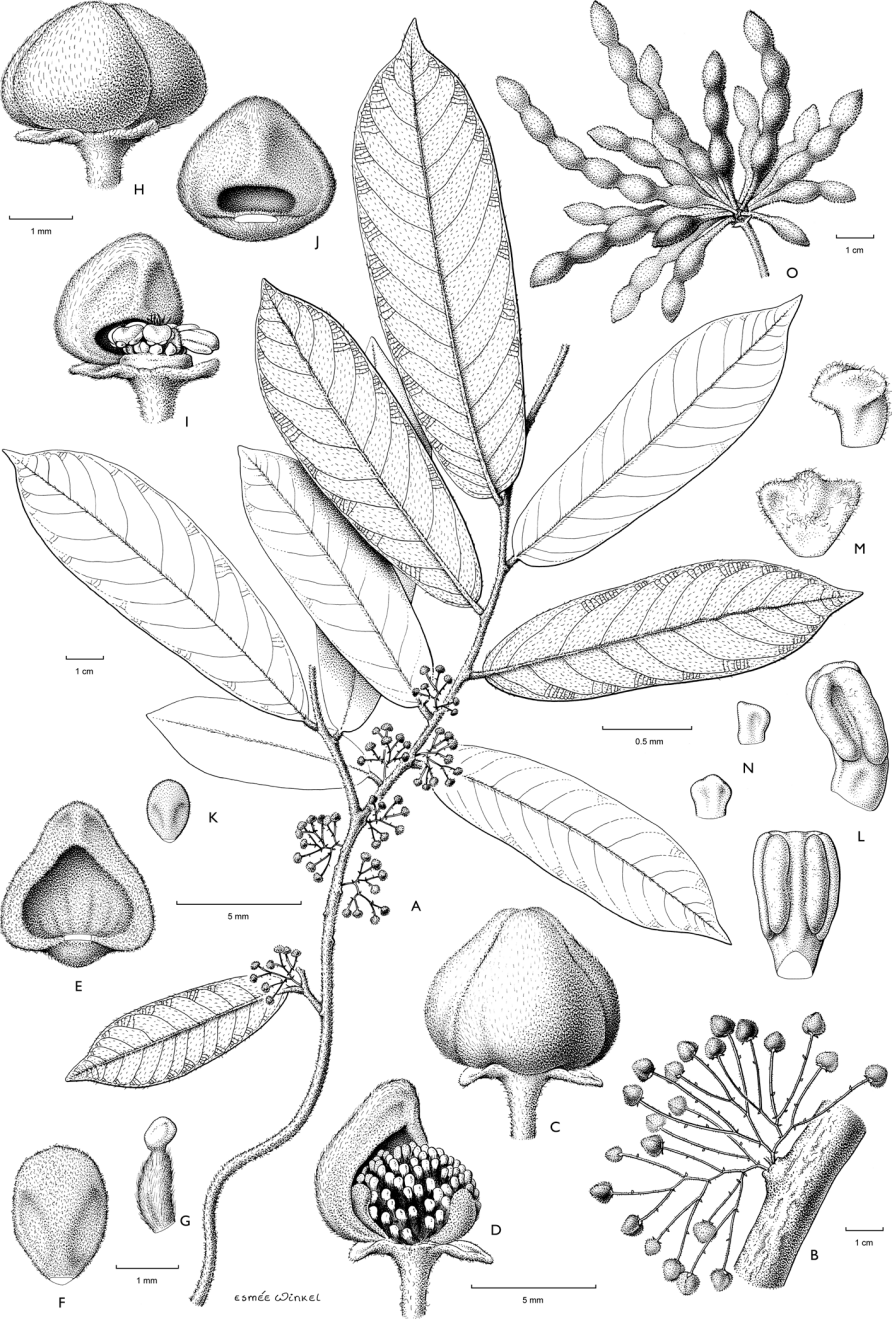
*Monanthotaxispynaertii***A** branch with male inflorescences **B** female inflorescence **C** female flower bud **D** female flower with two outer petals removed **E** outer petal of female flower, inner view **F** inner petal of female flower, inner view **G** carpel **H** male flower bud **I** male flower with two outer petals removed **J** outer petal of male flower, inner view **K** inner petal of male flower, inner view **L** stamen front and side views **M, N** staminodes **O** fruit **A, H–N** from *Evrard 5198***B–G** from *Pynaert 852***O** from *Tisserant 2035*). Drawings by Esmée Winkel (Hoekstra et al. 2021, fig. 25, p. 190).

#### Distribution.

A central African species, from Cameroon to the Democratic Republic of the Congo and the Central African Republic; in Cameroon known from the East region.

#### Habitat.

Very frequent when present (*Letouzey 5049*) but only known in Cameroon from a single collection to date; in swamp forests and gallery forests. Altitude ~500 m a.s.l.

#### Local and common names known in Cameroon.

None recorded.

#### Preliminary IUCN conservation status.

Vulnerable (VU) (Hoekstra et al. 2021).

#### Uses in Cameroon.

None reported.

#### Notes.

﻿﻿﻿*Monanthotaxispynaertii* is distinguished from the other species in the genus with cauliflorous inflorescences by the long (up to 1.5 mm long) erect hairs on the young foliate branches, petioles and leaves. It was previously placed in synonymy of ﻿*M.diclina*, but Hoekstra et al. (2021) reinstated its species status, as it differs by its erect pubescence, larger flowers, and more numerous carpels in the female flowers (95 to 150 versus 80 to 100 in ﻿*M.diclina*).

#### Specimen.

**East Region**: A 25 km au Sud de Mboy I (45 km à l’Est de Yokadouma), 3.38°N, 15.13°E, *15 May 1963*, *Letouzey R.* 5049 (P,YA).

### ﻿﻿﻿﻿ Monanthotaxis sterilis

Taxon classificationPlantaeMagnolialesAnnonaceae

P.H.Hoekstra, Blumea 66 (1): 200, 2021

DEBBC52C-5100-5EEE-A4F6-2ED3439E48A3

Fig. 68 ; Map 9A

#### Type.

Gabon. Woleu-Ntem; on road from Mitzic to Lalara (N2), just after the bridge over the Lara, *Couvreur T.L.P. 869*, 15 Nov 2015: holotype: WAG[WAG.1575982]; isotypes: LBV; YA.

#### Description.

Scrambling shrub to liana, up to 6 m tall, d.b.h. up to 2 cm. Indumentum of simple hairs to glabrous; old leafless branches glabrous, young foliate branches pubescent with dense appressed to ascending reddish brown hairs 0.2–0.4 mm long. Leaves: petiole 2–4 mm long, ca. 1 mm in diameter, pubescent with appressed to ascending reddish brown hairs, slightly grooved, blade inserted on top of the petiole; **blade 9.1–15.2 cm long, 1.4–2.4 cm wide, linear to narrowly elliptic**, apex acuminate, acumen 1–2 cm long, base cuneate, papyraceous, below sparsely pubescent when young, glabrous when old, above glabrous when young and old, discolorous, whitish below; midrib depressed, above sparsely pubescent when young, glabrous when old, below sparsely pubescent when young, glabrous when old; secondary veins 15 to 20 pairs, almost perpendicular with midrib, straight, but curving halfway, glabrous above; tertiary venation percurrent, hardly visible. Inflorescences, flowers and fruits unknown.

**Figure 68.**
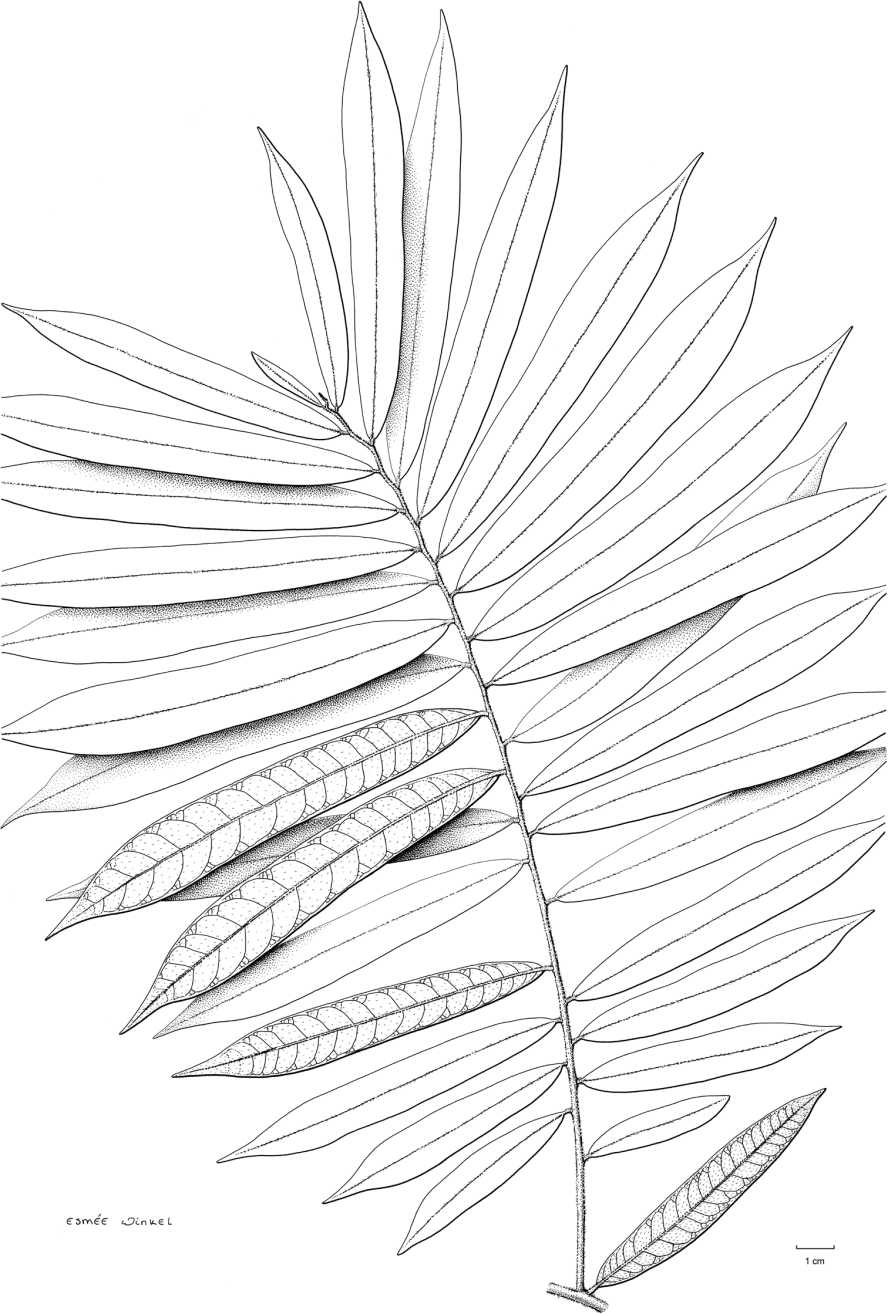
*Monanthotaxissterilis.* Branch. Drawing by Esmée Winkel.

#### Distribution.

A central African species, from Cameroon to the Republic of Congo; in Cameroon known from the Central and Littoral regions.

#### Habitat.

A fairly common species when present, but collected only twice in Cameroon; in primary and old secondary rain forests, along small streams, sometimes on sandy soils. Altitude 100–400 m a.s.l.

#### Local and common names known in Cameroon.

None recorded.

#### Preliminary IUCN conservation status.

Least Concern (LC) (Hoekstra et al. 2021).

#### Uses in Cameroon.

None reported.

#### Notes.

﻿﻿﻿*Monanthotaxissterilis* is distinguished by its linear or narrowly elliptic leaves and the secondary veins which are almost perpendicular with the midrib.

The first author has seen this species numerous times across central Africa, either as a young sapling on the ground or a young liana, but was never able to find any flowering or fruiting material. The DNA analyses indicated that ﻿﻿﻿*Monanthotaxissterilis* is most closely related to ﻿*M.pellegrinii* (see Hoekstra et al. 2018), with which it is very different in the leaf shape and venation.

#### Specimens examined.

**Central Region**: Mefou proposed national park Near Mefou town, 3.62°N, 11.58°E, *08 March 2004*, *Cheek M.* 11504 (K,WAG,YA). **Littoral Region**: Ebo Wildlife Reserve Djuma permanent camp On east trail, 4.36°N, 10.25°E, *15 February 2013*, *Couvreur T.L.P.* 628 (MPU,WAG,YA).

### ﻿﻿﻿﻿ Monanthotaxis submontana

Taxon classificationPlantaeMagnolialesAnnonaceae

P.H.Hoekstra, Blumea 66 (1): 200, 2021

123B7D1A-F487-5502-8A50-7A74B90158D0

Fig. 69 ; Map 9B

= Monanthotaxis cauliflora sensu Cheek et al. (2004): 238.

#### Type.

Cameroon. Littoral Region; Nlonako, *Letouzey R.G. 14476*, 17 Mar 1976: holotype: WAG[WAG0053953]; isotypes: MO[2 sheets]; P[P01982361].

#### Description.

Liana, 6–10 m tall, d.b.h. up to 5 cm. Indumentum of simple hairs; old leafless branches glabrescent, young foliate branches pubescent with short 0.1–0.2 mm long appressed to half-erect yellowish hairs. Leaves: petiole 6–10 mm long, ca. 1 mm in diameter, pubescent, weakly grooved adaxially, blade inserted on the side of the petiole; blade 7.2–14.1 cm long, 2.1–3.5 cm wide, **oblong to narrowly oblong or elliptic to narrowly elliptic**, apex acuminate to acute, acumen ca. 1.5 cm long, **base cuneate**, papyraceous, below sparsely pubescent when young, sparsely pubescent to glabrous when old, above glabrous when young and old, discolorous, whitish below; midrib sunken or flat, above glabrous when young and old, below sparsely pubescent when young and old; secondary veins 11 to 18 pairs, glabrous above; tertiary venation percurrent. Individuals bisexual [although fertile stamens can be absent leading to a female flower, thus possibly individuals gynodioecious], **inflorescences cauliflorous**, **a condensed panicle with many flowers; peduncle up to ca. 70 mm, densely pubescent with appressed to erect reddish brown hairs**; Flowers with 9 perianth parts in 3 whorls, **pedicel 7–55 mm long**, ca. 1 mm in diameter, densely pubescent with reddish hairs; in fruit 17–55 mm long, 1–2 mm in diameter; basal bract 1–2 mm long, 1–2 mm wide; upper bract 1–2 mm long, 1–2 mm wide; sepals 3, valvate, free, 3–2 mm long, ca. 2 mm wide, ovate, apex acute, base truncate, golden brown, densely pubescent outside, glabrous inside, margins flat; petals free, outer petals longer than inner, inner petals entirely covered in bud; outer petals 3, 3.6–5 mm long, 3.6–5.7 mm wide, broadly ovate to circular, apex obtuse, base truncate, golden green outside, yellowish inside, margins flat, densely pubescent outside, pubescent inside; inner petals 3, valvate, 0.4–1.3 mm long, 0.3–1.1 mm wide, broadly elliptic to circular, apex obtuse, base truncate, margins flat, pubescent outside, pubescent inside; stamens 0 to 2, in 1 row near the inner petals, ca. 1 mm long, clavate to oblong; connective reduced hidden by the thecae, pubescent; staminodes 0 to 14; **carpels free, 65 to 85**, ovary ca. 1 mm long, stigma globose, glabrous. Monocarps stipitate, stipes 7–14 mm long, 2 mm in diameter; monocarps up to 18, 23–45 mm long, 7–9 mm in diameter, moniliform, ellipsoid, apex apiculate, pubescent, smooth to slightly verrucose, constricted around seeds when more than 1, green when ripe; seeds 1 to 4 per monocarp, 13–14 mm long, 6–8 mm in diameter, ellipsoid; aril absent.

**Map 9.**
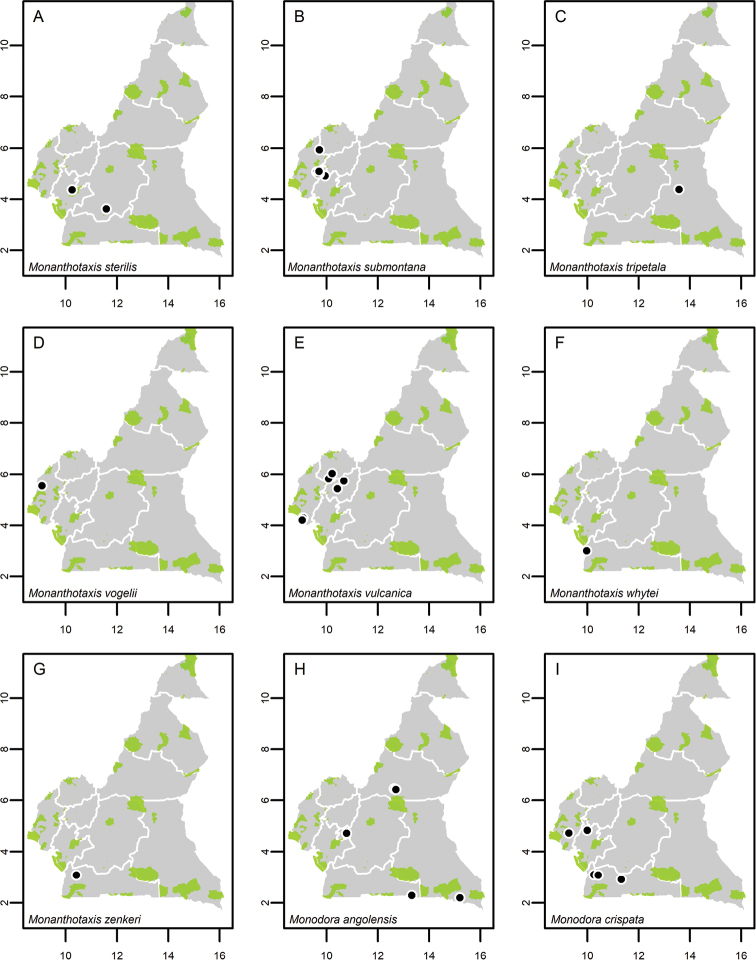
**A***Monanthotaxissterilis***B***Monanthotaxissubmontana***C***Monanthotaxistripetala***D***Monanthotaxisvogelii***E***Monanthotaxisvulcanica***F***Monanthotaxiswhytei***G***Monanthotaxiszenkeri***H***Monodoraangolensis***I***Monodoracrispata*. White borders represent region limits in Cameroon; green patches represent protected areas (see methods and Suppl. material 1: Fig. S1).

#### Distribution.

endemic to Cameroon, known from the South-West and Littoral regions.

#### Habitat.

In sub-montane or montane rain forests and swamp forests. Altitude 800–1700 m a.s.l.

#### Local and common names known in Cameroon.

None recorded.

#### Preliminary IUCN conservation status.

Endangered (EN) (Hoekstra et al. 2021).

#### Uses in Cameroon.

None reported.

#### Notes.

﻿﻿﻿*Monanthotaxissubmontana* is distinguished by its oblong to elliptic leaves with a cuneate base, cauliflorous inflorescences with a peduncle up to 70 mm long, flowering pedicels ranging from 7 to 55 mm long and flowers with 65 to 85 carpels.

Specimens identified as ﻿﻿﻿*Monanthotaxiscauliflora* in Cheek et al. (2004) are in fact redetermined as ﻿*M.submontana* (Hoekstra et al. 2021).

#### Specimens examined.

**Littoral Region**: Nlonako Mt, 4.90°N, 9.943°E, *17 March 1976*, *Letouzey R.* 14476 (MO,P,WAG). **South-West Region**: Ridge on S side of LOH Mt, 5°N, 9.683°E, *23 January 1998*, *Cheek M.* 9067 (K,WAG,YA); Kodmin to Nzee Mbeng trail at N’dib river crossing, 5°N, 9.716°E, *14 February 1998*, *Cheek M.* 9202 (K,WAG,YA); Bakossi Mountains 1–8 km NNE of Menyum Village, 5.05°N, 9.612°E, *22 May 1987*, *Doumenge C.* 554 (MO,P); Nzimbeng road, 5.93°N, 9.716°E, *04 February 1998*, *Etuge M.* 4122 (K,WAG,YA); Kodmin road towards Mahusom, 5°N, 9.683°E, *12 November 1998*, *Etuge M.* 4442 (K,WAG,YA); Bakossi Mountains west of Bangem, 5.08°N, 9.7°E, *01 January 1986*, *Thomas D.W.* 5274 (MO,P,YA).

**Figure 69.**
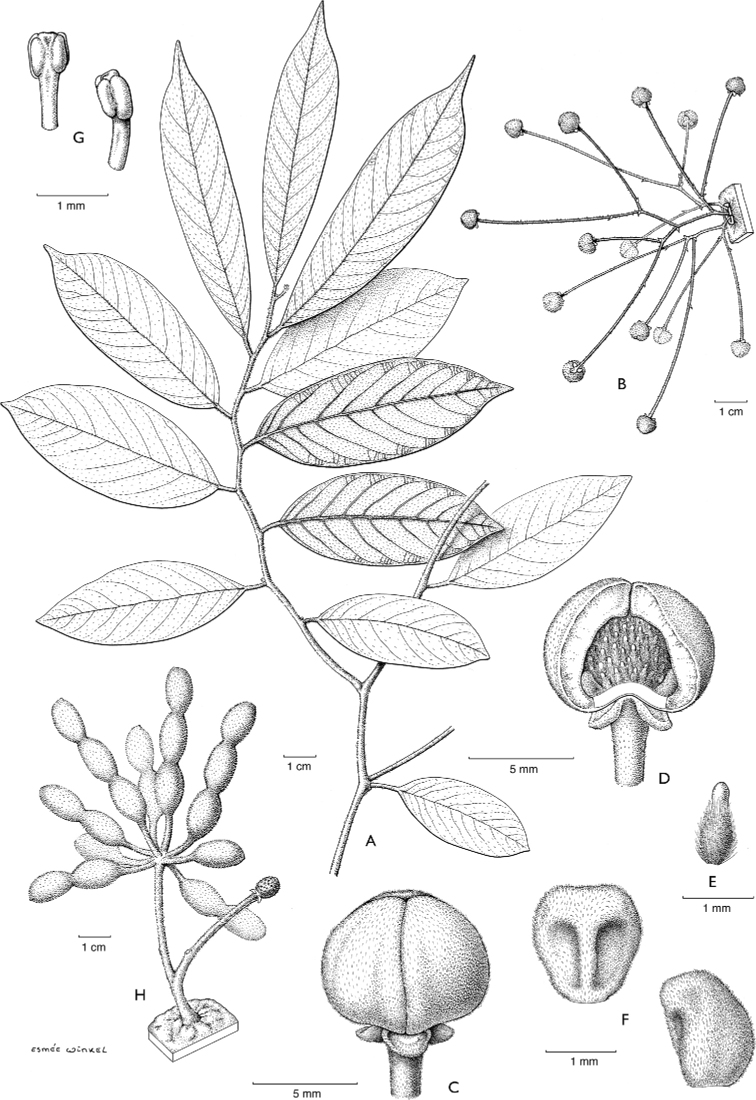
*Monanthotaxissubmontana***A** habit **B** inflorescence **C** flower bud **D** flower bud with one outer petal removed **E** inner petal, inner and outer views **F** carpel **G** stamen front and side views **H** fruiting branch **A–H** from *Letouzey 14476*. Drawings by Esmée Winkel (Hoekstra et al. 2021, fig. 31, p. 204).

### ﻿﻿﻿﻿ Monanthotaxis tripetala

Taxon classificationPlantaeMagnolialesAnnonaceae

P.H.Hoekstra, PhytoKeys 69: 96, 2016

43A2C997-245A-5616-A7FE-64D28914CE33

**Figure 70.**
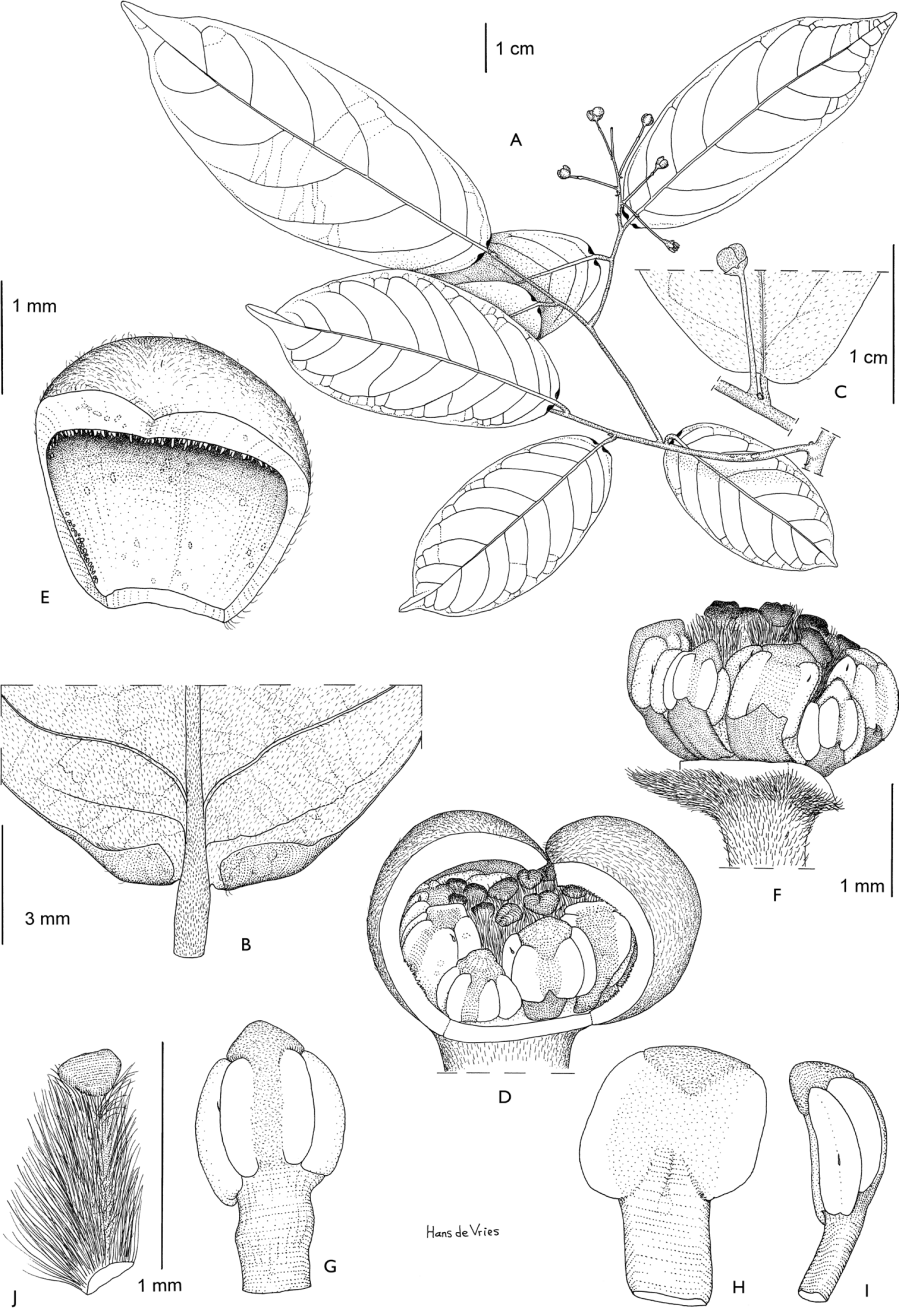
*Monanthotaxistripetala***A** flowering branch **B** leaf base abaxially **C** leaf base and inflorescence **D** flower with one petal removed **E** petal inner side view **F** flower with petals removed **G** stamen outer side view **H** stamen inner side view **I** stamen side view **J** carpel **A–J** from *Leeuwenberg 5828*. Drawings by Hans de Vries (Hoekstra et al. 2021, fig. 30, p. 201).

**Figure 71.**
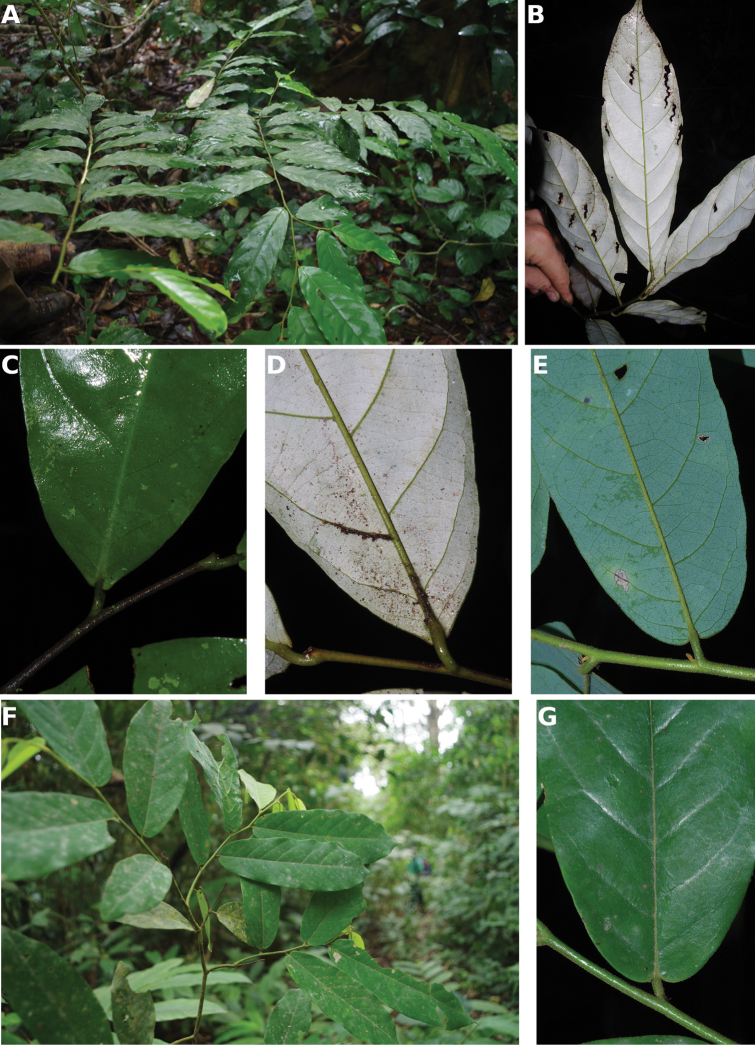
*Monanthotaxistripetala***A** habit, juvenile **B** leaf, lower side **C** base of leaf blade, upper side **D** base of leaf blade, lower side. *Monanthotaxisvulcanica***E** base of leaf blade, lower side **F** habit, juvenile **G** base of leaf blade, upper side **A–D***Couvreur 870*, Gabon **E–G***Couvreur 1049*, Mt Cameroon, Cameroon. Photos Thomas L.P. Couvreur.

Figs 70 , 71 ; Map 9C

#### Type.

Cameroon. East Region; 15 km E of Dimako, village halfway Bertoua-Doumé, *Leeuwenberg A.J.M. 5828*, 11 Jun 1965: holotype: WAG [WAG0110801, WAG0110802]; isotypes: B[B100190273]; BR[BR0000014126253]; C; EA; K; LISC; MO; P[P01967268]; PRE; YA.

#### Description.

Liana, 3 m tall, d.b.h. to 11 cm. Indumentum of simple hairs; old leafless branches glabrescent, young foliate branches pubescent with very short appressed reddish brown hairs 0.1 mm long. Leaves: petiole 2–8 mm long, 1–2 mm in diameter, pubescent, blade inserted on the side of the petiole; blade 4.2–16.2 cm long, 1.8–5.3 cm wide, oblong to elliptic, apex acuminate to acute, acumen 0.5–1 cm long, base rounded, subcoriaceous, below sparsely pubescent when young, glabrous when old, above sparsely pubescent to glabrous when young and old, discolorous, whitish below; midrib impressed, above sparsely pubescent when young, glabrous when old, below pubescent when young, sparsely pubescent when old; secondary veins 7 to 10 pairs, glabrous above; tertiary venation percurrent**. Individuals bisexual; inflorescences ramiflorous on old leafless branches, axillary.** Flowers with 6 to 7 perianth parts in 2 to 3 whorls, 1 to 2 per inflorescence, peduncle 0–6 mm long, pubescent; pedicel 12–20 mm long, 0.5 mm in diameter, pubescent; in fruit 16–29 mm long, ca. 2 mm in diameter; basal bract not seen; upper bracts ca. 1 mm long, ca. 0.5 mm wide; sepals 3, valvate, free, ca. 1 mm long, ca. 1 mm wide, ovate, apex obtuse, base truncate, densely pubescent outside, glabrous inside, margins flat; petals free, outer petals longer than inner (when present), inner petals entirely covered in bud; **outer petals 3, 2–2.2 mm long**, ca. 2.2 mm wide, broadly ovate to circular, apex rounded to obtuse, base truncate, margins flat, pubescent outside, pubescent inside; **inner petals absent or more rarely 1 minute, ca. 1.5 mm long**, ca. 0.5–0.6 mm wide, elliptic, apex acute, base truncate, margins flat, pubescent outside, pubescent and glabrous towards the base inside; stamens 9 to 12, in 1 row, ca. 1 mm long, linear to clavate; connective reduced, slightly acute, glabrous; staminodes absent; carpels free, 9, ovary ca. 1 mm long, stigma flat, glabrous. Monocarps stipitate, stipes 7–22 mm long, ca. 2 mm in diameter; monocarps up to 7, 110–130 mm long, 8–9 mm in diameter, moniliform, ellipsoid, apex apiculate, sparsely pubescent to glabrous, verrucose, constricted around seeds when more than 1, yellow when ripe; seeds 1 to 4 per monocarp, ca. 17 mm long, ca. 7 mm in diameter, ellipsoid; aril absent.

#### Distribution.

A Central African species, from Cameroon to Gabon, in Cameroon known from the East region.

#### Habitat.

A rare species, known from a single collection in Cameroon; in lowland primary or secondary rain forests, on hillsides. Altitude ~650 m a.s.l.

#### Local and common names known in Cameroon.

None recorded.

#### Preliminary IUCN conservation status.

Endangered (EN) (Hoekstra et al. 2021).

#### Uses in Cameroon.

None reported.

#### Notes.

﻿﻿﻿*Monanthotaxistripetala* is distinguished by the small axillary bisexual flowers, of which the inner petals are highly reduced or absent. The latter character also occurs in the male flowers of ﻿﻿﻿*Monanthotaxiscauliflora* and ﻿*M.diclina*, but those have unisexual flowers.

### ﻿﻿﻿﻿ Monanthotaxis vogelii

Taxon classificationPlantaeMagnolialesAnnonaceae

(Hook. f.) Verdc., Kew Bull. 25(1): 23, 1971

8653D4E4-7B62-5572-B09C-2E0D7B75CE21

Map 9D

≡ ﻿﻿Uvaria vogelii Hook.f., in Hooker Icon. Pl. 4: 767, 1848; ﻿﻿Clathrospermum vogelii (Hook.f.) Benth., in Bentham & Hooker 29, 1862; Popowia vogelii (Hook.f.) Baill.; Adansonia 8: 324, 1868.
= Popowia dalzielii Hutch., in Hutch. & Dalziel 55, 1927. Type. Nigeria. Benue State, Abinsi, *Dalziel J.M. 712*, 13 Sep 1972: lectotype, chosen by Hoekstra et al. (2021), p. 208: K [K000198917, K000913659]; isolectotypes: BM[BM001125037]; MO [2 sheets]; P[P00362644, P00362645].
= Monanthotaxis angustifolia (Exell) Verdc., Kew Bull., 25(1): 21, 1971; ﻿Enneastemonangustifolius Exell, J. Bot. 75: 163, 1937. Type. Nigeria. Ogun State, Ijebu, Shasha Forest reserve, *Richards P.W. 3469*, 17 May 1935: holotype: BM[BM000547356, BM001125040, BM001125041]; isotype: MO[MO-1889425].

#### Type.

Nigeria. Benue State; Abinsi, *Dalziel, J.M. 712*, 13 Sep 1972: holotype: K[K000198917, K000913659]; isotypes: BM[BM001125037]; MO [2 sheets]; P[P00362644, P00362645].

#### Description.

Scandent shrub to liana, 3 m tall, d.b.h. unknown. Indumentum of simple hairs; old leafless branches glabrescent, young foliate branches sparsely pubescent with appressed reddish brown hairs 0.1–0.2 mm long. Leaves: petiole 2–5 mm long, 1 mm in diameter, sparsely pubescent with appressed reddish brown hairs, slightly grooved, blade inserted on top of the petiole; blade 9.7–17.5 cm long, 2.5–5.6 cm wide, **obovate to oblanceolate**, apex acuminate to acute, acumen 1.5 cm long, **base cuneate to rounded**, chartaceous, below sparsely pubescent when young and old, above sparsely pubescent with appressed whitish hairs when young, sparsely pubescent to glabrous when old, discolorous, whitish below; midrib impressed, above glabrous when young and old, below sparsely pubescent when young and old; secondary veins 7 to 12 pairs, glabrous above; tertiary venation percurrent. Individual bisexual, inflorescences ramiflorous on old leafless branches, axillary. Flowers with 9 perianth parts in 3 whorls, 2 to 3(4) per inflorescence; pedicel 6–9(14) mm long, ca. 1 mm in diameter, densely pubescent with appressed yellow-brown hairs; in fruit 7–13 mm long, ca. 1 mm in diameter; upper bract soon falling ca. 2 mm long, ca. 0.7 mm wide; sepals 3, valvate, basally fused, 0.5–1 mm long, 1–1.5 mm wide, broadly ovate to broadly triangular, apex acute to rounded, base truncate, sparsely pubescent outside, glabrous inside, margins flat; petals free, outer petals longer than inner, **inner petals partly covered in bud**; outer petals 3, 2.4–3.3 mm long, 2 mm wide, elliptic to ovate, apex obtuse, base truncate, margins flat, sparsely pubescent outside, glabrous inside; inner petals 3, valvate, 2–2.7 mm long, 1.3 mm wide, elliptic, apex obtuse, base truncate, margins flat, sparsely pubescent outside, glabrous and sparsely pubescent towards apex inside; stamens (8)9, in 1 row, 1 mm long, linear to oblong; connective truncate, glabrous; staminodes 6, alternating with the stamens, glabrous; carpels free, 8 to 12, 2 mm long, stigma elongate, glabrous. Monocarps stipitate, stipes 2–4 mm long, ovary ca. 2 mm in diameter; monocarps 2 to 9, 11–15 mm long, 4–6 mm in diameter, **not moniliform, ellipsoid**, apex apiculate, pubescent, smooth, red when ripe; **seed 1 per monocarp**, ca. 10 mm long, 4–5 mm in diameter, ellipsoid; aril absent.

#### Distribution.

Known from Ghana, Ivory Coast, Benin, Nigeria and just reaching into Cameroon; in Cameroon known from the South-West region.

#### Habitat.

An infrequent species in Cameroon known from a single collection; in gallery or swamp forests, savannah open woodland and open high forest. Growing in cracks of schist rocks, on rocky islands, on loamy soil and on black clay. Altitude ~800 m a.s.l.

#### Local and common names known in Cameroon.

None recorded.

#### Preliminary IUCN conservation status.

Least Concern (LC) (Hoekstra et al. 2021).

#### Uses in Cameroon.

None reported.

#### Notes.

﻿﻿﻿*Monanthotaxisvogelii* can be readily recognized among Cameroonian species by its obovate to oblanceolate leaves with a cuneate (or sometimes rounded) base, and secondary veins that form an acute angle with the primary vein. The monocarps are composed of a single seed (developing from a single ovule).

The only specimen of this species from Cameroon was initially identified as *M.angustifolia*, however the name *M.angustifolia* was synonymized by Hoekstra et al. (2021). Indeed, the leaf shape was variable within *M.angustifolia* and intermediates exist with ﻿*M.vogelii*. Besides leaf shape, no other differences were found.

#### Specimen examined.

**South-West Region**: Mamfe Div on Cross river between Mamfe and the Nigerian boundary Abonando, 5.54°N, 9.07°E, *06 May 1902*, *Rudatis H.* 54 (K).

### ﻿﻿﻿﻿ Monanthotaxis vulcanica

Taxon classificationPlantaeMagnolialesAnnonaceae

P.H.Hoekstra, Blumea 66 (1): 208, 2021

0A91EF73-3910-5498-A1F9-56627A68BF18

**Figure 72.**
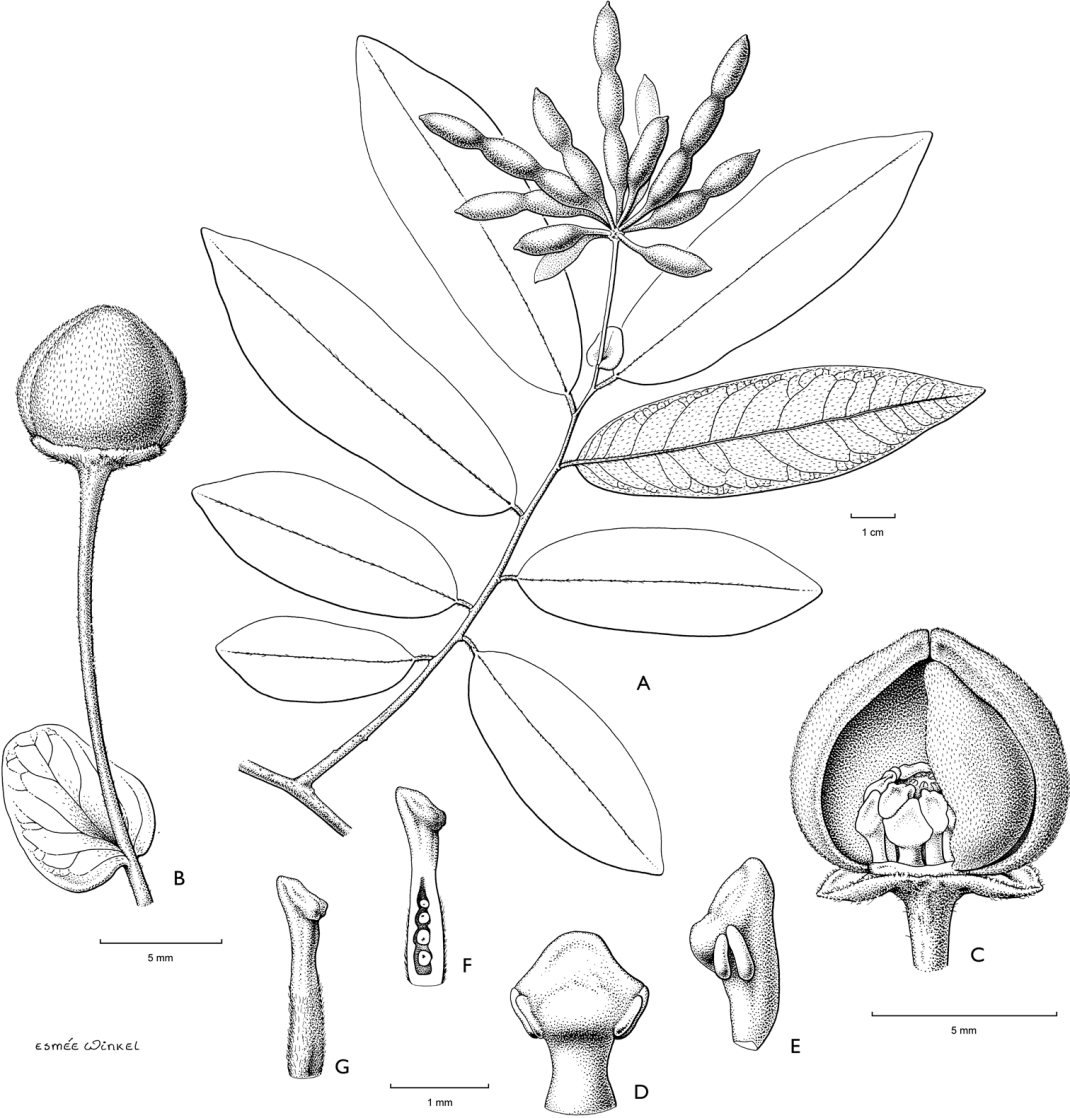
*Monanthotaxisvulcanica***A** fruiting branch **B** inflorescence with flower bud **C** flower bud with one outer and one inner petal removed **D** stamen, outer view **E** stamen, side view **D** carpel **G** longitudinal section of carpel **A** from *Chapman 3675***B–F** from *Letouzey 15050*. Drawings by Esmée Winkel (Hoekstra et al. 2021, fig. 33, p. 209).

Figs 71 , 72 ; Map 9E

#### Type.

Cameroon. South-West Region; NW du Mt Cameroon, *Letouzey R.G. 15050*, 1 Jun 1976: holotype: P[P01982551]; isotypes: K[K001322493]; WAG[WAG.1576469]; YA[YA0003005].

#### Description.

Liana, to 20 m tall, d.b.h. unknown. Indumentum of simple hairs; old leafless branches glabrescent, young foliate branches sparsely pubescent with appressed yellowish hairs 0.2 mm long to almost glabrous. Leaves: petiole 3–6 mm long, ca. 1 mm in diameter, sparsely pubescent with appressed yellowish hairs, slightly grooved, blade inserted on top of the petiole; blade 5.6–11.8 cm long, 2.3–3.9 cm wide, oblong elliptic, apex acuminate to acute, acumen up to 2 cm long, base rounded to narrowly cuneate or cordate, papyraceous, below sparsely pubescent when young, glabrous when old, above glabrous when young and old, discolorous, whitish below; midrib impressed, above sparsely pubescent when young and old, below sparsely pubescent when young and old; secondary veins 8 to 12 pairs, glabrous above; tertiary venation percurrent. Individuals bisexual; inflorescences ramiflorous on old leafless branches, leaf opposed or extra axillary. Flowers with 9 perianth parts in 3 whorls, 1 per inflorescence; pedicel 19–25 mm long, 0.5–1 mm in diameter, sparsely pubescent; in fruit 20–37 mm long, ca. 1 mm in diameter; basal bract ca. 1 mm long, ca. 1 mm wide; upper bract, **leaf-like, 7–15 mm long, 5–10 mm wide**; sepals 3, valvate, basally fused, ca. 2 mm long, ca. 3 mm wide, ovate, apex acute, base truncate, densely pubescent outside, glabrous inside, margins flat; petals free, outer petals longer than inner, inner petals entirely covered in bud; outer petals 3, 6.4–8 mm long, ca. 6 mm wide, ovate, apex obtuse, base truncate, pale orange, margins flat, sparsely pubescent outside, sparsely pubescent inside; inner petals 3, valvate, 5.3–5.7 mm long, ca. 4 mm wide, broadly elliptic, apex acute, base truncate, pale orange, margins flat, pubescent outside, sparsely pubescent inside; **stamens 15, in 2 rows**, ca. 2 mm long, clavate; connective truncate, larger than base, glabrous; staminodes absent; **carpels free, ca. 14**, ovary ca. 2 mm long, stigma elongate, flattened at top, glabrous. **Monocarps stipitate, stipes 6–10 mm long**, ca. 2 mm in diameter; monocarps 2 to 13, 12–50 mm long, 5–7 mm in diameter, moniliform, ellipsoid to cylindrical, apex apiculate, very sparsely pubescent, verrucose, constricted around seeds when more than 1, orange when ripe; seeds 1 to 4 **per monocarp**, 8–15 mm long, 5–6 mm in diameter, ellipsoid; aril absent.

#### Distribution.

From Nigeria to Cameroon; in Cameroon known from the North-West, South-West and West regions.

#### Habitat.

In sub-montane rain forest and on forest edges. Altitude 800–2600 m a.s.l.

#### Local and common names known in Cameroon.

None recorded.

#### Preliminary IUCN conservation status.

Endangered (EN) (Hoekstra et al. 2021).

#### Uses in Cameroon.

None reported.

#### Notes.

﻿﻿﻿*Monanthotaxisvulcanica* is distinguished by its large leaf-like (7–15 mm long and 5–10 mm wide) upper bract on the flowering pedicel (this leafy bract is also found in a few other species such as ﻿*M.obovata* (Benth.) P.H.Hoekstra and ﻿*M.orophila* (Boutique) Verdc., none found in Cameroon however), 15 stamens and ca. 14 pubescent carpels per flower and the monocarps with long stipes (6 to 10 mm long).

In the check list to the plants of Mt Oku (Cheek et al. 2000, p. 114) and in the Flora of West Tropical Africa (Keay 1952), collection *Maitland 1618* was identified as ﻿Monanthotaxis (Popowia) littoralis (Bagsh. & Baker f.) Verdc. but is in fact ﻿*M.vulcanica*. In addition, in the check list to the plants of Bali Ngemba reserve (Harvey et al. 2004), the collection under “﻿*Monanthotaxis* sp. of Bali Ngemba” (*Etuge 4810*) is now identified as ﻿*M.vulcanica*. Finally, specimen Chapman & Chapman 3675 was label as ﻿*Friesodielsia* sp. (Chapman and Chapman 2001), and is also identified here as ﻿*M.vulcanica*.

#### Specimens examined.

**North-West Region**: Mantum, 5.82°N, 10.08°E, *17 November 2000*, *Etuge M.* 4810 (K,YA). **South-West Region**: Mount Cameroon National Park on the Bomona trail behind Bomona village 10 km NW from Idenau, 4.29°N, 9.086°E, *03 April 2016*, *Couvreur T.L.P.* 1049 (WAG,YA); Pentes NW du Mont vers Efolofo 30 km W, 4.21°N, 9.05°E, *01 June 1976*, *Letouzey R.* 15050 (K,P,YA); Bamenda Distr at Bambui, 6.02°N, 10.21°E, *01 June 1931*, *Maitland T.D.* 1618 (K). **West Region**: Megom, 5.43°N, 10.42°E, *01 February 1939*, *Jacques-Félix H.* 3078 (P); Massif du Nkogam (2263 m) 25 km W de Foumban, 5.73°N, 10.67°E, *28 October 1974*, *Letouzey R.* 13046 (P,P,YA).

### ﻿﻿﻿﻿ Monanthotaxis whytei

Taxon classificationPlantaeMagnolialesAnnonaceae

(Stapf) Verdc., Kew Bull. 25(1): 29, 1971

C14CEAEE-D447-52AD-855A-80E18A536529

Map 9F

≡ Popowia whytei Stapf, J. Linn. Soc., Bot. 37: 81, 1905.

#### Type.

Liberia. Sinon; Sinoe Basin, *Whyte A. s.n.*, 1904: holotype: K[K000198908].

#### Description.

Shrub to liana, 2–50 m tall, d.b.h. unknown. Indumentum of simple hairs; old leafless branches glabrescent, young foliate branches densely pubescent with appressed to ascending reddish brown hairs 0.2 mm long. Leaves: petiole 1–7 mm long, 1–2 mm in diameter, densely pubescent with appressed to ascending reddish brown hairs, weakly grooved adaxially, blade inserted on top of the petiole; blade 8.8–19.4 cm long, 3.5–8.5 cm wide, obovate, apex acuminate to acute, acumen 0.5–1 cm long, base subcordate, papyraceous to subcoriaceous, below sparsely pubescent when young, glabrous when old, above sparsely pubescent when young, glabrous when old, discolorous, whitish below; midrib sunken or flat, above sparsely pubescent when young, glabrous when old, below sparsely pubescent when young, glabrous when old; secondary veins 8 to 14 pairs, glabrous above; tertiary venation percurrent, sometimes not visible. Individuals bisexual; **inflorescences cauliflorous or ramiflorous** on old leafless branches, axillary. Flowers with 9 perianth parts in 3 whorls, 1 per inflorescence, peduncle 2–8 mm, densely pubescent with yellowish slightly erect hairs; pedicel 7–11 mm long, ca. 1 mm in diameter, densely pubescent with yellowish slightly erect hairs; in fruit 13–20 mm long, ca. 2 mm in diameter; basal bract 1–2 mm long, ca. 1 mm wide; upper bract 1–2 mm long, ca. 1 mm wide; sepals 3, valvate, basally fused, 1–2 mm long, 1–2 mm wide, broadly ovate, apex obtuse, base truncate, pubescent outside, glabrous inside, margins flat; petals free, outer petals longer than inner, inner petals entirely covered in bud; outer petals 3, 3.4–5 mm long, 3.4–4.5 mm wide, broadly ovate, apex obtuse, base truncate, yellow to light green, margins flat, pubescent outside, glabrous, pubescent towards margins inside; inner petals 3, valvate, 3–4 mm long, 1.5–2.2 mm wide, elliptic to ovate, apex obtuse, base truncate, margins flat, pubescent outside, glabrous inside; **stamens 9, in 1 row**, ca. 1 mm long, obconical; connective truncate, glabrous; **staminodes 9**, **in one external whorl and alternating with the stamens**, glabrous; carpels free, 27 to 36, ovary ca. 1 mm long, stigma elongate, glabrous. Monocarps stipitate, stipes 5–12 mm long, ca. 2 mm in diameter; monocarps up to 10, 15–38 mm long, ca. 8 mm in diameter, moniliform, ellipsoid to globose, apex rounded to apiculate, pubescent to glabrous, verrucose, constricted around seeds, yellow to orange when ripe; seeds 1 to 3 per monocarp, 9–10 mm long, 6–7 mm in diameter, ellipsoid; aril absent.

#### Distribution.

A mainly West African species, from Sierra Leone to Benin, and Nigeria to Cameroon; in Cameroon known from the South region.

#### Habitat.

A rare species just reaching in Cameroon and known by a single collection in Cameroon, in lowland primary or old secondary rain forests, swampy forests, gallery forests and in savanna regions. Altitude 0–50 m a.s.l.

#### Local and common names known in Cameroon.

None recorded.

#### Preliminary IUCN conservation status.

Least Concern (LC) (Hoekstra et al. 2021).

#### Uses in Cameroon.

None reported.

#### Notes.

﻿﻿﻿*Monanthotaxiswhytei* is distinguished by the cauliflorous or ramiflorous inflorescences with rounded floral buds and flowers with 9 stamens and 9 small staminodes.

#### Specimen examined.

**South Region**: ca 15 km N of Kribi Edéa roadLittoral forest behind beach ca 50 m from s 3.01°N, 9.966°E, *04 February 1969*, *Bos J.J.* 3854 (P,WAG,YA).

### ﻿﻿﻿﻿ Monanthotaxis zenkeri

Taxon classificationPlantaeMagnolialesAnnonaceae

P.H.Hoekstra, PhytoKeys 69: 98, 2016

419F47BB-18B3-50CD-B0F7-7CEFDE719043

Fig. 73 ; Map 9G

#### Type.

Cameroon. South Region; Bipindi, *Zenker G.A.3495a*, Oct 1907: holotype: G[G00308331]; isotypes: BR[BR0000013211349]; E[E00624356]; HBG *n.v.*, K *n.v.*, L[L1759466]; MO[3726267].

#### Description.

Liana, height unknown, d.b.h. unknown. Indumentum of simple hairs; old leafless branches glabrous, **young foliate branches densely pubescent with reddish brown, erect hairs 0.3–0.4 mm long**. Leaves: petiole 3–6 mm long, 1–2 mm in diameter, densely pubescent with reddish brown erect hairs, slightly grooved, blade inserted on top of the petiole; blade 4.7–20.1 cm long, 2.3–9.5 cm wide, elliptic to obovate, apex acute to obtuse, base rounded, papyraceous to subcoriaceous, below sparsely pubescent with short erect yellow-brown hairs when young and old, above densely pubescent with erect yellow-brown hairs when young and old, discolorous, whitish below; midrib impressed, above sparsely pubescent with short erect yellow-brown hairs when young and old, below densely pubescent when young and old; secondary veins 8 to 12 pairs, glabrous above; tertiary venation percurrent. Individuals bisexual; inflorescences ramiflorous on old leafless branches, axillary. Flowers with 9 perianth parts in 3 whorls 1 to 3 per inflorescence; pedicel 4–6 mm long, 0.5–1 mm in diameter, densely pubescent; in fruit unknown; basal bract not seen; upper bract ca. 1 mm long, ca. 1 mm wide; sepals 3, valvate, basally fused, ca. 1 mm long, ca.2 mm wide, ovate to broadly triangular, apex obtuse, base truncate, densely pubescent outside, glabrous inside, margins flat; petals free, outer petals longer than inner, inner petals entirely covered in bud; outer petals 3, 2–3.1 mm long, 2.1–2.5 mm wide, ovate, apex obtuse, base truncate, margins flat, pubescent outside, pubescent towards margins to pubescent and glabrous towards center inside; inner petals 3, valvate, 1.8–2.4 mm long, 1.3–1.6 mm wide, rhombic, apex obtuse, base truncate, margins flat, densely pubescent outside, pubescent inside; stamens 35, in 3 to 4 rows, ca. 1 mm long, linear; **anthers apically pubescent and converging apically and hiding the connective which is reduced or absent, glabrous**; staminodes absent; carpels free, ca. 16, ovary ca. 1 mm long, stigma elongate, flattened at top, glabrous. Fruits unknown.

#### Distribution.

endemic to Cameroon, known from the South region.

#### Habitat.

Only known from the type collection, in lowland rain forests. Altitude ca. 200 m a.s.l.

**Figure 73.**
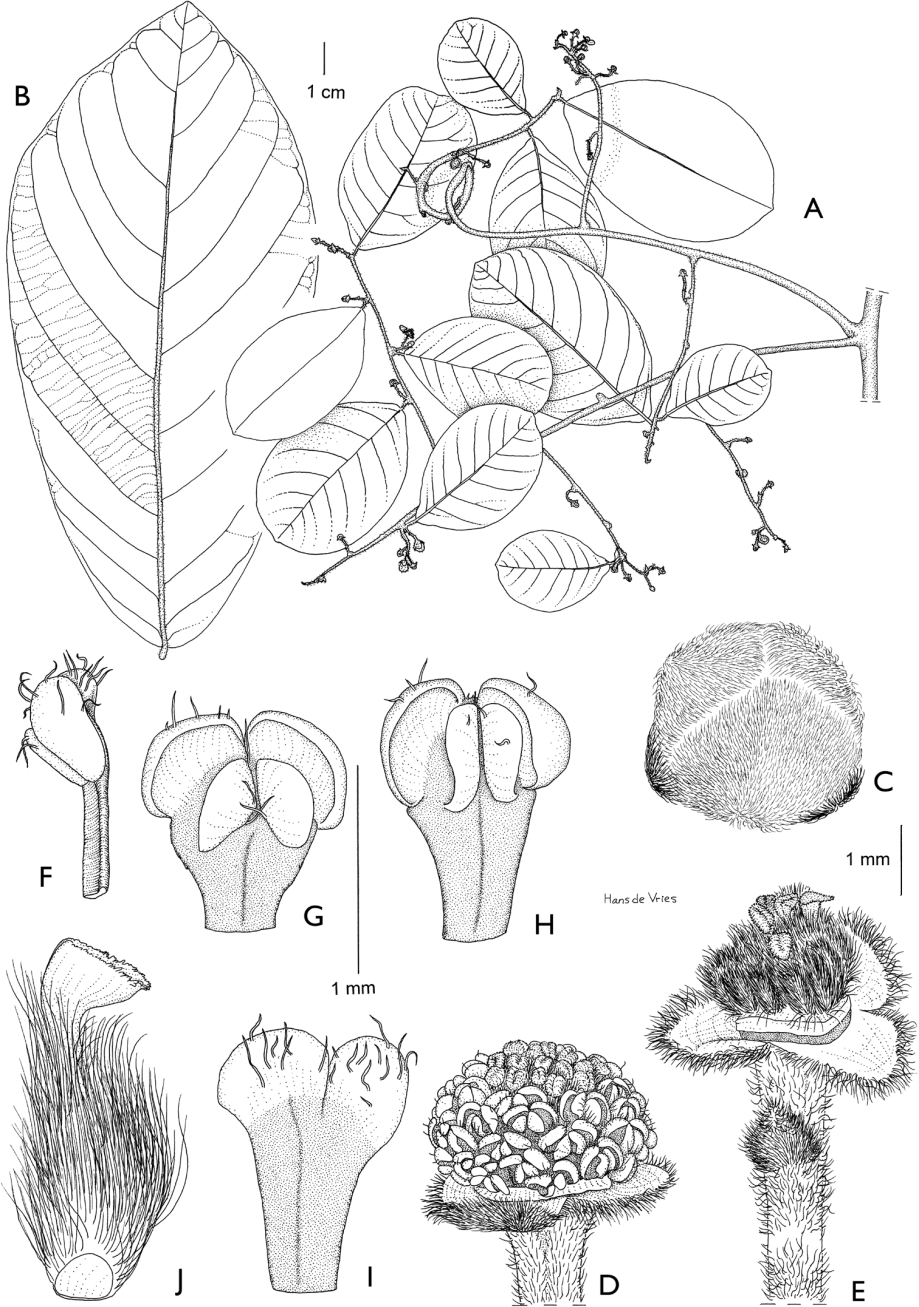
*Monanthotaxiszenkeri***A** habitus **B** leaf, lower side **C** flower bud **D** flower with petals removed **E** old flower **F** stamen side view **G, H** stamen, outer view **I** stamen inner side view **J** carpel **A–J***Zenker 3495a.* Drawings by Hans de Vries (Hoekstra et al. 2021, fig. 35, p. 212).

#### Local and common names known in Cameroon.

None recorded.

#### Preliminary IUCN conservation status.

Critically Endangered (CR) (Hoekstra et al. 2021).

#### Uses in Cameroon.

None reported.

#### Notes.

﻿﻿﻿*Monanthotaxiszenkeri* has unique stamens within the genus (Hoekstra et al. 2021). It is the only species with the combination of apically pubescent anthers and a relatively short filament.

### ﻿﻿ Monodora

Taxon classificationPlantaeMagnolialesAnnonaceae

Dunal, Monogr. Anon. 3: 79, 1817

ECA3897B-27BF-56A6-B4D0-05C7D0C80CDD

#### Type species.

﻿﻿﻿*Monodoramyristica* (Gaertner) Dunal.

#### Description.

Trees, rarely shrubs or rarely lianas, 4–40 m tall, d.b.h. 6–100 cm; stilt roots or buttresses absent. Indumentum of simple hairs, or absent. Leaves: petiole 2–14 mm long, 1–2 mm in diameter, blade inserted on the side of the petiole; blade 4–50 cm long, 2–15 cm wide, elliptic or obovate or oblong or ovate, apex acuminate, acumen ca. 1 cm long; base cuneate to rounded, concolorous; midrib **raised above**; secondary veins 8 to 23 pairs; tertiary venation reticulate. Individuals bisexual; inflorescences ramiflorous on old or young foliate branches, leaf opposed or extra axillary, **1 per inflorescence**; pedicel 8–270 mm long, 1–2 mm in diameter; in fruit 25–350 mm long, 3–15 mm in diameter; bract 1, towards the upper half or middle of pedicel; sepals 3, valvate, free, 2–40 mm long, 2–17 mm wide, ovate or elliptic, apex acute or attenuate or rounded, base truncate, margins flat, undulate, wavy or crisped; **petals basally fused**, tube 2–8 mm long, **inner and outer whorl differentiated**, **outer petals longer than inner**; outer petals 3, 17–105 mm long, 6–40 mm wide, oblong or obovate or ovate, apex truncate, base truncate **or with two small lobes**, margins flat or wavy or crisped; inner petals 3, valvate, 9–35 mm long, 5–30 mm wide, cordate or rhombic or triangular to cochleate, apex acuminate to acute, **base narrowed into a claw**, claw 1–9 mm long; receptacle flat to strongly convex; stamens numerous, in 9 to 20 rows, 1–2 mm long, broad; connective discoid or elongated; staminodes absent; **carpels fused into a syncarpous ovary**, 1–5 mm long, stigma bilobed, slightly capitate. **Fruit syncarpous**, sessile, 35–150 mm long, 20–150 mm in diameter, globose or ovoid or conical, apex apiculate to rounded; seeds numerous, 9–22 mm long, 5–13 mm in diameter, ellipsoid or flattened ellipsoid; aril absent.

A genus of trees with 14 known species, from West, Central and East Africa. In Cameroon six species are known, one endemic.

﻿*Monodora*, together with its sister genus ﻿*Isolona*, are unique in Annonaceae in having truly syncarpous ovaries, producing single fruits with unordered seeds, in contrast to other genera which have either uni- or biseriate placentation. Petals in ﻿*Monodora* are basally fused forming a short (not clearly visible) tube. The petals, however, are differentiated into inner and outer whorls, in contrast to ﻿*Isolona* (which has six equal lobes in a single whorl with a visible tube). In the vegetative state, ﻿*Monodora* and ﻿*Isolona* (together with *Polyceratocarpouspellegrinii*) are characterized by a raised leaf midrib on the upper side, in contrast to a sunken or flat midrib in all other genera found in Cameroon. ﻿*Monodora* species tend to have a whitish-grey wax indumentum on young leaves and fruits, which is especially noticeable in the common and widespread species ﻿*M.myristica*.

#### Taxonomy.

Couvreur (2009).

### ﻿Key to the species of ﻿*Monodora* in Cameroon:

**Table:** 

1	Inner petals with two conspicuously hairy appendages at midpoint on margins of the blade	﻿***M.tenuifolia***
–	Inner petals lacking two conspicuously hairy appendages at midpoint on margins of the blade	**2**
2	Margin of outer petals straight, with two small lobes at the base (rare)	﻿﻿***M.zenkeri***
–	Margin of outer petals undulate or crisped, without two small lobes at the base	**3**
3	Inner petals 20–35 mm long, with claw less than 1/3 of the length of the blade; receptacle strongly convex.	**4**
–	Inner petals 4–17 mm long, claw more than 1/3 of the length of the blade; receptacle slightly convex to flat.	**5**
4	Upper bract with clearly undulate margins, elliptic to obovate; flowering pedicels 7–27 cm long, in fruit 30–35 cm long; fruit generally globose, finely ribbed, glabrous	﻿***M.myristica***
–	Upper bract with straight margins, very broadly ovate; flowering pedicels 3–5.5 cm long, in fruit shorter than 10 cm long; fruit generally ovoid, not finely ribbed, tomentose	﻿***M.undulata***
5	Inner petals with hairs 2–3 mm long on inner surface; outer petals crisped; fruit conspicuously 5 to 7-ribbed, otherwise smooth.	﻿***M.crispata***
–	Inner petals glabrous on inner surface; outer petals undulate; fruit rugose, bumpy, not ribbed	﻿***M.angolensis***

### ﻿﻿﻿﻿ Monodora angolensis

Taxon classificationPlantaeMagnolialesAnnonaceae

Welw., Apont. 587, 1859

5D3B759E-E388-583C-93E2-EE98BB02EF8C

Fig. 74 ; Map 9H

= Monodora angolensis var. decidua Hiern, Cat. Afr. Pl. Welw. 1: 13, 1896. Type. Angola. Malanje, Golungo Alto, *Welwitsch F.M.J. 776*, Dec 1855: holotype: LISC; isotypes: B[B 10 0154049]; BM[BM000889328]; BR[BR0000008802026]; C[C10004780, C10004779]; COI[COI00077210]; G; K[K000198845, *pro parte*]; LISU[LISU206066, LISU206065]; P[P00363309].
= Monodora angolensis var. microphylla Hiern, Cat. Afr. Pl. Welw. 1: 13, 1896. Type. Angola. Malanje, Pungo Andongo, *Welwitsch F.M.J. 775*, Jan 1857: holotype: BM; isotype: K[K000198845, *pro parte*].
= Monodora durieuxii De Wild., Études Fl. Bas-et Moyen-Congo 1: 122, 1903. Type. Democratic Republic of the Congo. Equateur, Wangata, Dewèvre A.P. 613, 14 Jan 1896: lectotype, sheet here designated: BR[BR0000008802354]; isotypes: BR[BR0000008802682, BR0000008802743].
= Monodora letestui Pellegr., Bull. Soc. Bot. France 94: 386, 1947. Type. Gabon. Ogooué-Lolo, Lastoursville, *Le Testu G.M.P.C. 7222*, Apr 1929: lectotype, sheet here designated: P[P00363310]; isotypes: BM[BM000553855]; BR[BR0000008801692]; LBV *n.v.*, LISC[LISC000382]; P[P00363311].
= Monodora louisii Boutique, Bull. Jard. Bot. État Brux. 21: 97, 1951. Type. Democratic Republic of the Congo. Orientale, Yangambi, Louis J.L.P. 6612, 15 Nov 1937: lectotype, sheet here designated: BR[BR0000006246730]; isotypes: BM[BM000553857]; BR[BR0000006247065]; C[C10004782, C10004781]; K[K000199023 K000199024]; NY *n.v.*, P[P00363257] ; PRE[PRE0774858-0] ; S[S10-21167].

#### Type.

Angola. Malanje; Pungo Andongo, *Welwitsch F.M.J. 774*, May 1855: holotype: LISC; isotypes: B[B 10 0190365]; BM[BM000553856]; COI[COI00077209]; G[G00011630]; K[K000198843, K000198844].

#### Description.

Tree, 18–20 m tall, d.b.h. 10–25 cm; stilt roots or buttresses absent. **Indumentum absent; old leafless branches glabrous, young foliate branches glabrous.** Leaves: petiole 2–10 mm long, 1 mm in diameter, glabrous, weakly grooved adaxially, blade inserted on the side of the petiole; blade 4–20 cm long, 2–7.5 cm wide, elliptic, apex acuminate, acumen 0.5–1 cm long, base cuneate to obtuse, coriaceous to papyraceous, below glabrous when young and old, above glabrous when young and old, concolorous; midrib raised above, above glabrous when young and old, below glabrous when young and old; secondary veins 8 to 16 pairs, glabrous below; tertiary venation reticulate. Individuals bisexual; inflorescences ramiflorous on old or young foliate branches, leaf opposed or extra axillary. Flowers with 9 perianth parts in 3 whorls, 1 per inflorescence; pedicel 8–40 mm long, ca. 1 mm in diameter, glabrous; in fruit 25–85 mm long, 5–7 mm in diameter, glabrous; bract 1, towards the upper half of pedicel, 4–17 mm long, 3–12 mm wide; sepals 3, valvate, free, 5–15 mm long, 2–6 mm wide, ovate, apex acute, base truncate, green speckled with red and purple, glabrous outside, glabrous inside, margins undulate; petals basally fused, tube 2–3 mm long, inner and outer whorl differentiated, outer petals longer than inner; outer petals 3, 17–50 mm long, 10–30 mm wide, oblong-obovate, apex truncate, base truncate, red-brown with pale yellow spots towards the apex, **margins wavy**, glabrous outside, glabrous inside; inner petals 3, valvate, 4–11 mm long, 5–16 mm wide, cordate to rhombic, apex acuminate, base narrowed into a claw, claw 3.0–9 mm long, white tinged with yellow, minutely purple-mottled along margins, **margins flat**, glabrous outside, glabrous inside; receptacle convex to flat; stamens numerous, in 9 to 11 rows, 1 mm long, broad; connective discoid, glabrous, cream; staminodes absent; carpels fused into a single structure, 1–2 mm long, stigma bilobed, slightly capitate, glabrous. Fruit syncarpous, 35–80 mm long, 35–50 mm in diameter, globose to ovoid, **apex apiculate, glabrous, rugose, bumpy**, green to brown when ripe; seeds 9–13 mm long, 5–8 mm in diameter, ellipsoid; aril absent.

**Figure 74.**
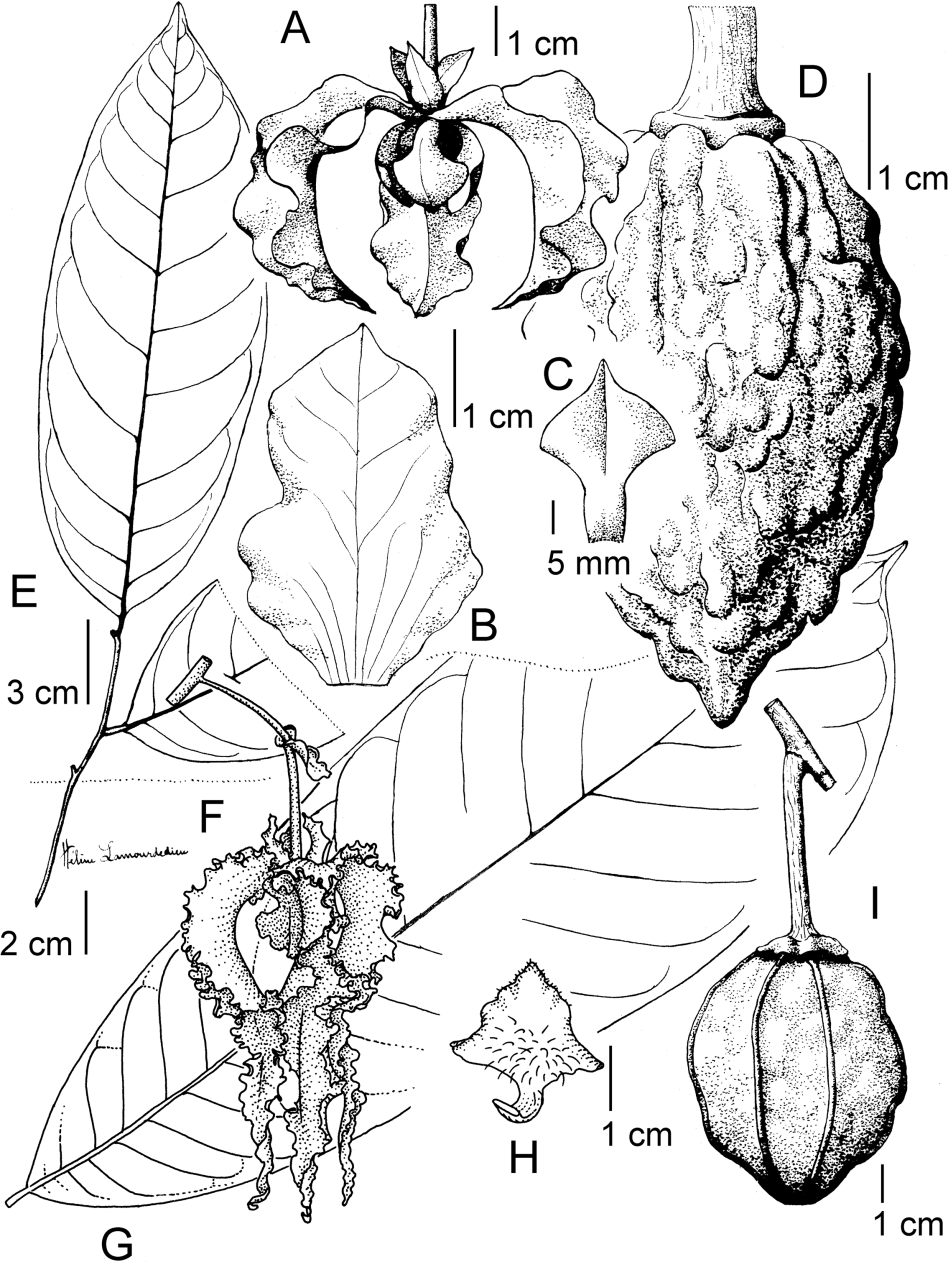
*Monodoraangolensis***A** flower **B** outer petal **C** inner petal, inside view **D** fruit **E** leaf. *Monodoracrispata***F** flower **G** leaf **H** inner petal, inside view **I** fruit **A–E** from *Hallé 2628***F, H***J.J.E.F. de Wilde 867***G, I** from *Klaine 720*. Drawings by Hélène Lamourdedieu, Publications Scientifiques du Muséum national d’Histoire naturelle, Paris; modified from Le Thomas (1969b, pl. 64, p. 347) **F, H** drawn by Hans de Vries (Couvreur 2009, fig. 49, p. 92).

#### Distribution.

A widespread species from eastern Nigeria to northern Angola, Central African Republic, Democratic Republic of Congo, Uganda, western Tanzania and northern Zambia, with one collection from Ivory Coast; in Cameroon known from East, South, Central and Adamaoua regions.

#### Habitat.

Fairly uncommon in Cameroon; in primary, secondary, and montane rain forests, gallery forest, sometimes in dry forests. Altitude 0–1800 m a.s.l.

#### Local and common names known in Cameroon.

None recorded.

#### Uses in Cameroon.

None reported.

#### IUCN conservation status.

Least Concern (LC) (Cosiaux et al. 2019y).

#### Notes.

﻿﻿﻿*Monodoraangolensis* is distinguished from other species by the combination of the following characters: entirely glabrous leaves and branches and clawed non undulated glabrous inner petals.

#### Specimens examined.

**Central Region**: Environs de Ndokalende 10 km SW de Ndikinimeki, 4.72°N, 10.78°E, *04 March 1984*, *Satabié B.* 754 (P,WAG,YA). **East Region**: Molunda Sation Ngoko Nginda 21 km north of Molundu, 2.2°N, 15.2°E, *09 January 1911*, *Mildbraed G.W.J.* 4199 (B,HBG). **North Region**: Tibati, 6.46°N, 12.63°E, *01 February 1909*, *Ledermann C.L.* 2462 (B); km 10 Tibati-Mabouka road, 6.43°N, 12.7°E, *29 June 1972*, *Leeuwenberg A.J.M.* 10034 (BR,K,MO,P,WAG,YA). **South Region**: 18 km east from Lélé village, 2.27°N, 13.32°E, *07 September 2013*, *Couvreur T.L.P.* 461 (WAG,YA); 26 km east from Lélé village, 2.28°N, 13.31°E, *08 September 2013*, *Couvreur T.L.P.* 477 (WAG,YA).

### ﻿﻿﻿﻿ Monodora crispata

Taxon classificationPlantaeMagnolialesAnnonaceae

Engl., Notizbl. Königl. Bot. Gart. Berlin 2: 301, 1899

C1C94E70-9DE7-50CB-8621-655B4E1BC86A

Fig. 74 ; Map 9I

= Monodora crispata var. klaineana Engl., Monogr. Afrik. Pflanzen.-Fam. 6: 90, 1901. Type. Gabon. Estuaire, Libreville, Klaine T.-J. 1435, 14 Jan 1899: lectotype, designated by Couvreur (2009), p. 206: P[P01985660].

#### Type.

Cameroon. South Region; 9 km N. of Kribi, *Bos J.J. 6224*, 2 Feb 1970: neotype, designated by Couvreur 2008, p. 206, sheet here designated: WAG[WAG0158468]; isotypes: BR[BR0000014056789]; C, K, LD, L MO, P, WAG[WAG0158469]; YA.

#### Description.

**Tree or a more often a woody climber**, **lianescent**, up to 20 m tall, d.b.h. 15–30 cm; stilt roots or buttresses absent. Indumentum of simple hairs if present; **old leafless branches glabrous, young foliate branches glabrous**. Leaves: petiole 3–7 mm long, 1–2 mm in diameter, glabrous, grooved, blade inserted on the side of the petiole; blade 5–17 cm long, 2.5–6 cm wide, elliptic to obovate, apex acuminate, acumen 0.3–1 cm long, base rounded, coriaceous to membranous, below glabrous when young and old, above glabrous when young and old, concolorous; midrib raised above, above glabrous when young and old, below glabrous when young and old; secondary veins 9 to 13 pairs, glabrous below; tertiary venation reticulate. Individuals bisexual; inflorescences ramiflorous on old or young foliate branches, leaf opposed or extra axillary. Flowers with 9 perianth parts in 3 whorls, 1 per inflorescence; pedicel 20–50 mm long, ca. 1 mm in diameter, glabrous; in fruit 30–50 mm long, 4–10 mm in diameter, glabrous; bracts 1, upper only, towards the upper half or middle of pedicel, 6–15 mm long, 5–9 mm wide; sepals 3, valvate, free, 5–18 mm long, 3–6 mm wide, ovate, apex acute, base truncate, green, glabrous outside, glabrous inside, **margins wavy to crisped**; petals basally fused, tube 2–3 mm long, inner and outer whorl differentiated, outer petals longer than inner; outer petals 3, 35–70 mm long, 6–20 mm wide, oblong, apex attenuate, base truncate, white or yellow at base and distally yellow with red-brown mottling, **margins crisped**, glabrous outside, glabrous inside; inner petals 3, valvate, 16–25 mm long, 6–20 mm wide, cordate to triangular, apex acute, base narrowed into a claw 3–8 mm long, white to yellow with red streaks towards margins, margins crisped, glabrous outside, sparsely pubescent inside; receptacle convex to flat; stamens numerous, in 9 to 11 rows, 1 mm long; connective discoid, glabrous, cream; staminodes absent; carpels fused into a single structure, ca. 2 mm long, stigma bilobed, slightly capitate, sparsely pubescent. Fruit syncarpous, 35–50 mm long, ca. 20 mm in diameter, **long ellipsoid, apex acute, glabrous, 6–7 ribbed**, otherwise smooth, green when ripe; seeds 10–13 mm long, 5–9 mm in diameter, ellipsoid; aril absent.

#### Distribution.

A widespread species present in West and Central Africa, from Sierra Leone to Ghana, and from southeastern Nigeria to Gabon; in Cameroon known from South and South-West regions.

#### Habitat.

An uncommon species in Cameroon; in primary and secondary rain forests and along streams, generally on sandy soil. Altitude 0–400 m a.s.l.

#### Local and common names known in Cameroon.

None recorded.

#### IUCN conservation status.

Least Concern (LC) (Cosiaux et al. 2019z).

#### Uses in Cameroon.

None reported.

#### Notes.

﻿﻿﻿*Monodoracrispata* is characterized by its generally lianescent habit resembling a woody climber growing on rocks or larger trees, the petals with very crispy margins, entirely glabrous branches and leaves, and ellipsoid and conspicuously 6–7-ribbed fruits.

Cheek and Cable (1998) mention from Mt Cameroon an ﻿*Isolona* sp. nov. (*Watts 687, Wheatley 501*), however these are now identified as ﻿﻿﻿*Monodoracrispata*.

#### Specimens examined.

**South Region**: 9 km N of Kribi, 4.83°N, 9.99°E, *02 February 1970*, *Bos J.J.* 6224 (BR,C,K,LD,MO,P,WAG,YA); Just E of Kribi Seca Regrowth veg On S bank of Kienke R, 3.1°N, 10.25°E, *20 August 1970*, *Bos J.J.* 7199 (BR,P,WAG); bezirk Ebolowa Ekuk 22 km Ebolowa, 2.91°N, 11.31°E, *01 January 1911*, *Mildbraed G.W.J.* 5650 (HBG); Bipindi, 3.08°N, 10.41°E, *01 January 1909*, *Zenker G.A.* 3884 (B); Bipindi, 3.08°N, 10.41°E, *01 January 1909*, *Zenker G.A.* 3935 (BM,BR,G,K,L,MO,P,S). **South-West Region**: Versant extérieur SSE du cratère du lac Dissoni (=SODEN lac) 20 km WNW Kumba, 4.72°N, 9.270°E, *20 March 1976*, *Letouzey R.* 14498 (K,MO,WAG,YA); Mt Bakingili-Nja Keta Path, 4.08°N, 9.05°E, *21 September 1992*, *Wheatley J.I.* 501 (K,YA).

### ﻿﻿﻿﻿ Monodora myristica

Taxon classificationPlantaeMagnolialesAnnonaceae

(Gaertner) Dunal, Monogr. Anon. 3: 80, 1817

9231B8B6-E207-5C9F-9808-A201B1B6DBB4

**Figure 75.**
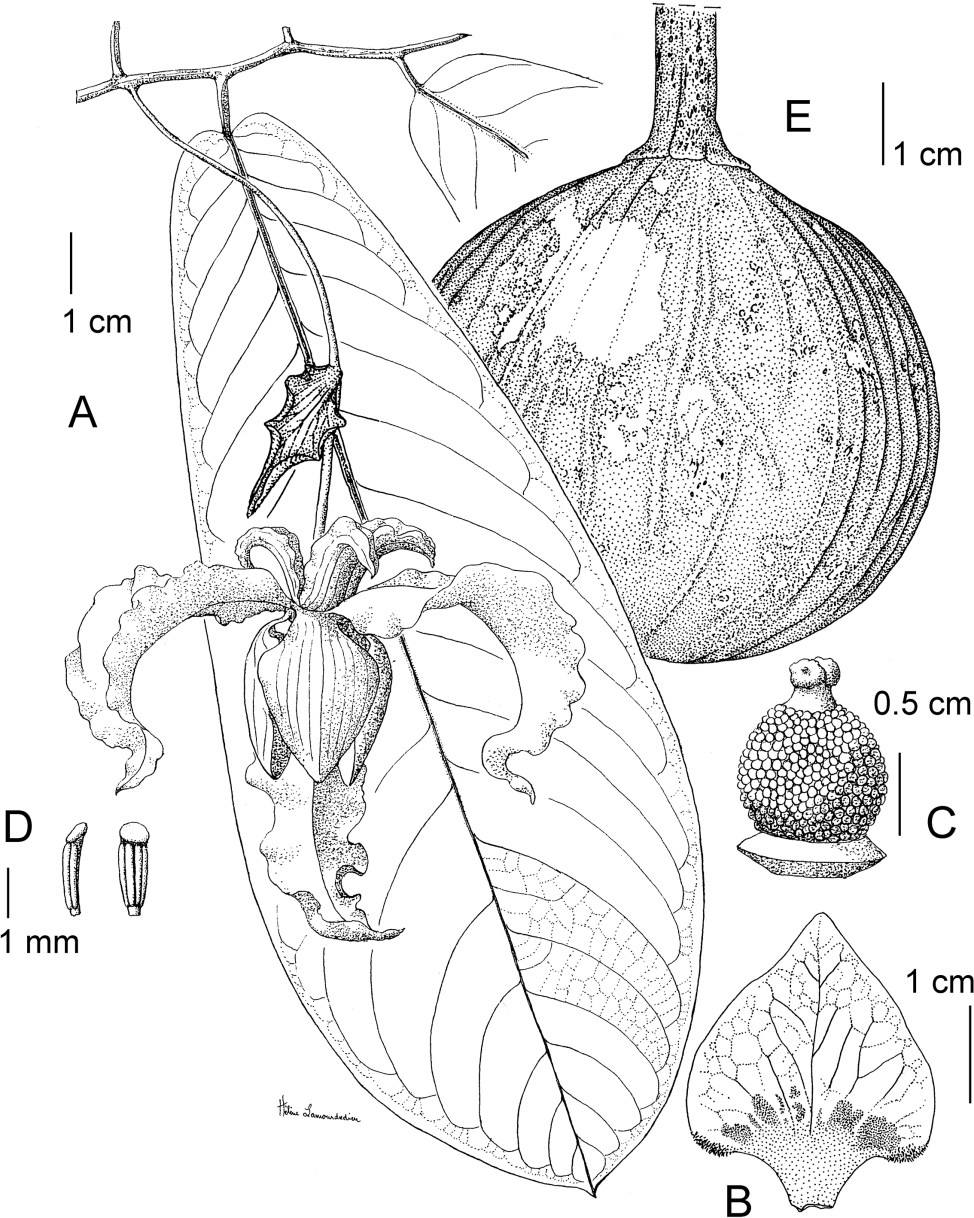
*Monodoramyristica***A** flowering branch **B** inner petal, inner surface **C** androecium and stigma **D** stamens; side view (left), front view (right) **E** fruit **A, C, D** from *Letouzey 3899***B** from *de Koning 1146***E** from *Versteegh 113*. Drawings by Hélène Lamourdedieu, Publications Scientifiques du Muséum national d’Histoire naturelle, Paris; modified from Le Thomas (1969b, pl. 63, p. 343); drawings **A** (bract) **B, E** by Hans de Vries (Couvreur 2009, fig. 64, p. 119).

**Figure 76.**
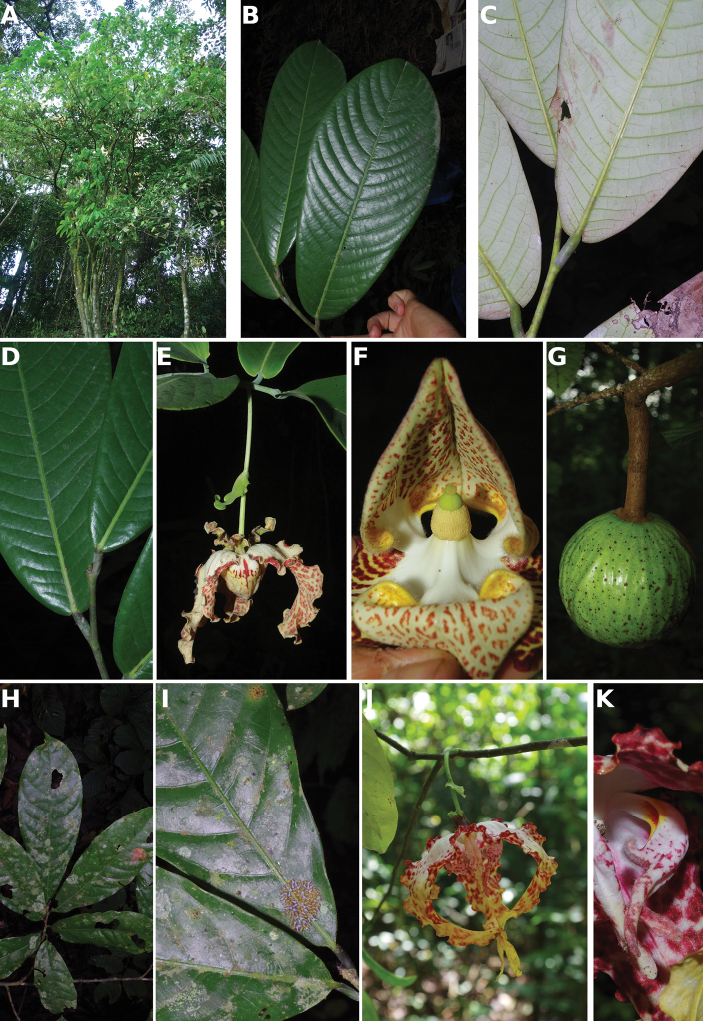
*Monodoramyristica***A** habit **B** Leaf, upper view **C** base of leaf blade, lower side, note pale greyish-green color **D** base of leaf blade, uppper side **E** flower, note long pedicel and undulate bract inserted in the middle of the pedicel **F** detail of flower, inner petal folded, showing petals fused at base and strongly convex receptacle **G** fruit. *Monodoratenuifolia***H** leaf, upper view **I** base of leaf blade, upper view **J** flower **K** detail of inner petal, note small hairy lobe **A***Couvreur 514*, Mt Etinde, Cameroon **B–D***Couvreur 1050*, Mt Cameroon, Cameroon **E, F***Couvreur 608*, Oveng, Cameroon **G***Couvreur 445*, Ottotomo, Cameroon **H, I***Couvreur 1218*, Maséa, Cameroon **J, K***Couvreur 1019*, Mbayang Mbo, Cameroon. Photos Thomas L.P. Couvreur.

**Map 10.**
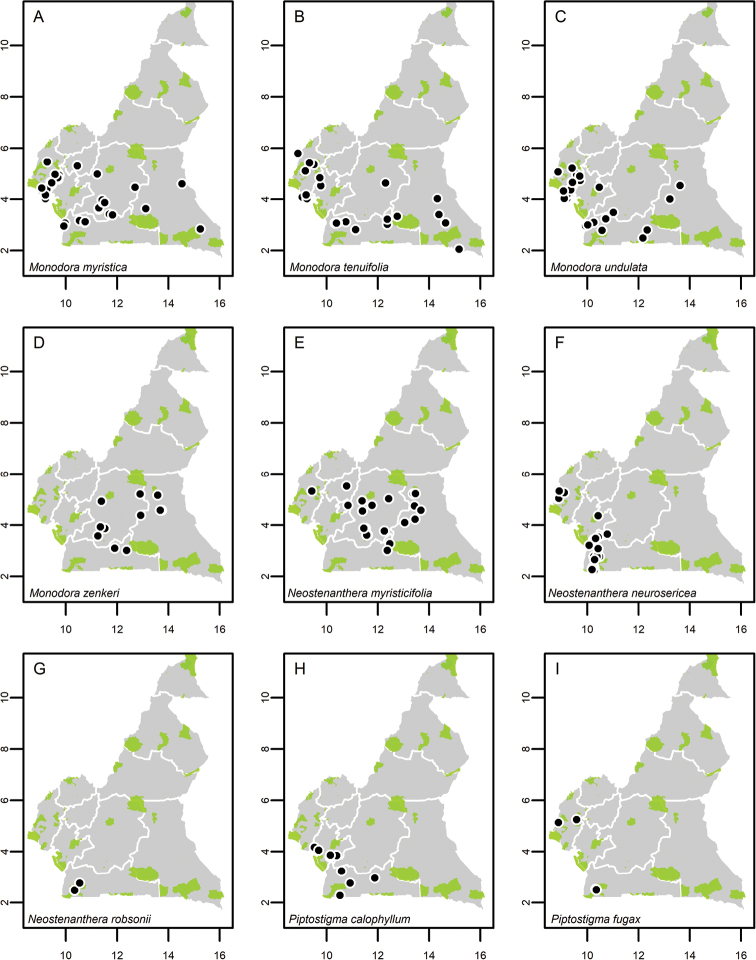
**A***Monodoramyristica***B***Monodoratenuifolia***C***Monodoraundulata***D***Monodorazenkeri***E***Neostenantheramyristicifolia***F***Neostenantheraneurosericea***G***Neostenantherarobsonii***H***Piptostigmacalophyllum***I***Piptostigmafugax*. White borders represent region limits in Cameroon; green patches represent protected areas (see methods and Suppl. material 1: Fig. S1).

Figs 75 , 76 ; Map 10A

≡ Annona myristica Gaertner; Fruct. Sem. Pl. 2: 194, 1791.
= Monodora borealis Scott-Elliot, Journ. Linn. Soc., Bot. 30: 72, 1895. Type. Sierra Leone. Northern Province, Scarcies Rivers, *Scott-Elliot G.F. 4716*, 7 Jan 1892: holotype: K *n.v.*
= Monodora claessensii De Wild., Bull. Jard. Bot. État Brux. 3: 263, 1911. Type. Democratic Republic of the Congo. Manie Kindu, *Claessens, J. 504*, 1910: lectotype, sheet here designated: BR[BR0000008802071]; isotypes: BR[BR0000008802088, BR0000008802408].
= Monodora unwinii Hutch. & Dalziel, Bull. Misc. Inform. Kew: 53, 1927. Type. Nigeria. Southern state, Benin city, Unwin A.H. 45, no date: holotype: K[K000199030].

#### Type.

Jamaica. Cultivated, obtained from Banks: holotype: BM.

#### Description.

Tree, 30–40 m tall, d.b.h. 40–100 cm; stilt roots or buttresses absent. Indumentum of simple hairs if present; old leafless branches glabrous, young foliate branches glabrous, covered with a whitish wax. Leaves: **petiole 8–14 mm long**, 1–2 mm in diameter, glabrous, slightly grooved, blade inserted on the side of the petiole; **blade 11–50 cm long, 4–14 cm wide**, obovate, apex acuminate, acumen 1–1.5 cm long, **base cuneate to cordate**, coriaceous to papyraceous, **below glabrous when young but covered with a whitish wax**, glabrous when old, above glabrous when young and old, concolorous; midrib raised above, above glabrous when young and old, below glabrous when young and old; secondary veins 13 to 23 pairs, glabrous below; tertiary venation reticulate. Individuals bisexual; inflorescences ramiflorous on young foliate branches, leaf opposed or extra axillary. Flowers with 9 perianth parts in 3 whorls, 1 per inflorescence; **pedicel 70–270 mm long, 2 mm in diameter, glabrous; in fruit 300–350 mm long, 10–15 mm in diameter**, glabrous; bracts 1, upper only, towards the middle of pedicel, 15–40 mm long, 8–30 mm wide; sepals 3, valvate, free, 20–40 mm long, 7–17 mm wide, elliptic to ovate, apex attenuate, base truncate, pale yellow with purple to completely dark red, glabrous outside, glabrous inside, margins wavy; petals basally fused, tube 6–8 mm long, inner and outer whorl differentiated, outer petals longer than inner; outer petals 3, 40–105 mm long, 20–40 mm wide, ovate, apex acute, base truncate, deep yellow, base streaked with dark red, pale yellow spotted dark purple when young, margins wavy, glabrous outside, glabrous inside; inner petals 3, valvate, 25–35 mm long, 25–30 mm wide, cordate, apex acute to obtuse, base narrowed into a claw 2.0–5 mm long, white with red central vein abaxially, white and speckled with red-yellow adaxially, margins flat, pubescent towards base on both sides; **receptacle strongly convex**; stamens ca. 460–470, in 16 to 20 rows, 2 mm long, broad; connective elongated, pubescent, yellow; staminodes absent; carpels fused into a single structure, 4–5 mm long, stigma bilobed, slightly capitate, pubescent. Fruit syncarpous, 90–150 mm long, 80–150 mm in diameter, **globose, apex rounded, glabrous, faintly ribbed longitudinally, otherwise smooth**, green when ripe; seeds 15–22 mm long, 10–13 mm in diameter, flattened ellipsoid; aril absent.

#### Distribution.

A widespread species, from Sierra Leone to northern Angola, Democratic Republic of the Congo, Uganda, western Kenya and Tanzania, also on the Island of São Tomé; in Cameroon known from the East, South, Central, Littoral, South-West and Adamaoua (one collection) regions.

#### Habitat.

A common species with a wide ecological amplitude, in primary and secondary rain forests, sometimes along rivers and near marshes, on sandy or rocky soils. Altitude 0–1600 m a.s.l.

#### Local and common names known in Cameroon.

Avom (*Parren 136*, Dial. Ewondo); Ndin (*Westphal, 9149, 9883, 10173, 10204* [these later specimens were seen but not cited or mapped in the flora because they are market collections, thus the orgin of the plants are unknown]); Muscadier de Calabash, fausse noix de muscade (French), Calabash Nutmeg, African nutmeg, false nutmeg (English); muskatnußduftender Orchideenbaum, Kalabassenmuskat (German).

#### IUCN conservation status.

Least Concern (LC) (Cosiaux et al. 2019aa).

#### Uses in Cameroon.

***food***: seeds for condiments, spices, flavourings (*Westphal 10173*).

#### Notes.

﻿﻿﻿*Monodoramyristica* is easily recognizable by its long pedicel (up to 27 cm long in flower and 35 cm in fruit) and its globose generally finely ribbed fruits. It is also characterized by its pale green lower leaf surface and petioles (due to the presence of whitish wax), and a generally cordate leaf base. Sterile it can be confused with ﻿*M.undulata*, but the later generally has smaller leaves lacking the blue whitish indumentum.

#### Selected specimens examined.

**Central Region**: Mont Mbam Minkon on trail 2 km from Nkol Nyada village, 3.97°N, 11.40°E, *21 March 2013*, *Couvreur T.L.P.* 412 (WAG,YA); Ottotomo Forest Reserve 1 km after reserve base near small loggers road, 3.65°N, 11.28°E, *25 June 2013*, *Couvreur T.L.P.* 445 (WAG,YA); on trail to Oveng Lodge hotel near parking just behind the village of Oveng 30 km on road from Mbalmayo to Sangmeli 3.41°N, 11.70°E, *09 February 2014*, *Couvreur T.L.P.* 608 (WAG,YA); Yaoundé, 3.86°N, 11.51°E, *30 July 1976*, *Westphal E.* 9149 (WAG); Market of Messa Yaoundé, 3.87°N, 11.52°E, *14 February 1978*, *Westphal E.* 9883 (YA,P). **East Region**: Colline Nkolandjom près Ngoakélé à 25 km à l’W de Ngoulemakong (route Mbalmayo-Ebolowa), 3.38°N, 11.81°E, *12 July 1972*, *Letouzey R.* 11474 (BR,P,YA); Asia, 3.63°N, 13.11°E, *21 April 1961*, *Letouzey R.* 3899 (P,WAG,YA); Bezirk Molundu Bange-Busch Lokomo Bumba u Bange, 2.83°N, 15.25°E, *21 February 1911*, *Mildbraed G.W.J.* 4530 (HBG). **Littoral Region**: Barombi camp 5 km S of Kumba on Buea-Douala road, 4.58°N, 9.45°E, *20 May 1983*, *Thomas D.W.* 2118 (K,MO,P,YA). **North Region**: Kona, 4.6°N, 14.53°E, *15 December 1960*, *Letouzey R.* 2616 (P,YA). **South Region**: Longii small marsh near seashore, 3.06°N, 9.966°E, *22 March 1969*, *Bos J.J.* 4194 (BR,K,LM,MO,P,WAG,YA); Kribi roadside in New Bell, 2.95°N, 9.916°E, *14 May 1969*, *Bos J.J.* 4526 (BR,K,LD,LM,MO,P,POZG,WAG,YA); on road Lolodorf-Bipindi ca half way near Mbiguiligui village (Mbikiliki), 3.16°N, 10.53°E, *26 February 2018*, *Couvreur T.L.P.* 1155 (MPU,P,WAG,YA); Ebom, 3.1°N, 10.73°E, *20 February 1996*, *Elad M.* 444 (WAG). **South-West Region**: Mungo River F.R., 4.78°N, 9.566°E, *02 December 1999*, *Cheek M.* 10228 (K,MO,P,WAG,YA); Abang road, 4.92°N, 9.733°E, *14 December 1999*, *Cheek M.* 10357 (K,MO,P,WAG,YA); Mount Cameroon National Park on the Bomona trail behind Bomona village 10 km NW from Idenau, 4.29°N, 9.079°E, *03 April 2016*, *Couvreur T.L.P.* 1050 (WAG,YA); 2 km north of Nyasoso towards Mpako, 4.84°N, 9.679°E, *04 April 2016*, *Couvreur T.L.P.* 1055 (WAG,YA); on trail from Ekongo village located 5 km before the entrance to Limbe 7 km on secondary road On flank of Mt Etinde 100 m in Mont Cameroon National Park, 4.06°N, 9.166°E, *16 October 2013*, *Couvreur T.L.P.* 514 (WAG,YA); Ngusi, 4.83°N, 9.683°E, *15 January 1996*, *Etuge M.* 1576 (K,WAG,YA); Nyasoso-Bedume road God-dat trail(opposite Ngusi road), 4.83°N, 9.683°E, *02 July 1996*, *Etuge M.* 2516 (K,MO,P,WAG,YA); Mbolekang 1–2 km N of Nyandong village crossing Ndebessong river, 4.96°N, 9.579°E, *24 March 2003*, *Ghogue J.-P.* 1487 (K); Entre Okoroba et Mbinda 20 km NW Nguti, 5.45°N, 9.272°E, *14 June 1975*, *Letouzey R.* 13825 (P,YA); Limbe (Victoria), 4.01°N, 9.2°E, *Preuss P.R.* 1303 (BR,K,P,S). **West Region**: Bayangam, 5.3°N, 10.45°E, *17 December 1978*, *Westphal E.* 10173 (P,WAG); Bayangam, 5.3°N, 10.45°E, *28 March 1979*, *Westphal E.* 10204 (P,WAG).

### ﻿﻿﻿﻿ Monodora tenuifolia

Taxon classificationPlantaeMagnolialesAnnonaceae

Benth., J. Proc. Linn. Soc., Bot. 5: 72, 1860

CE7EEEB4-50C4-5B02-A987-72433D1EBE5A

**Figure 77.**
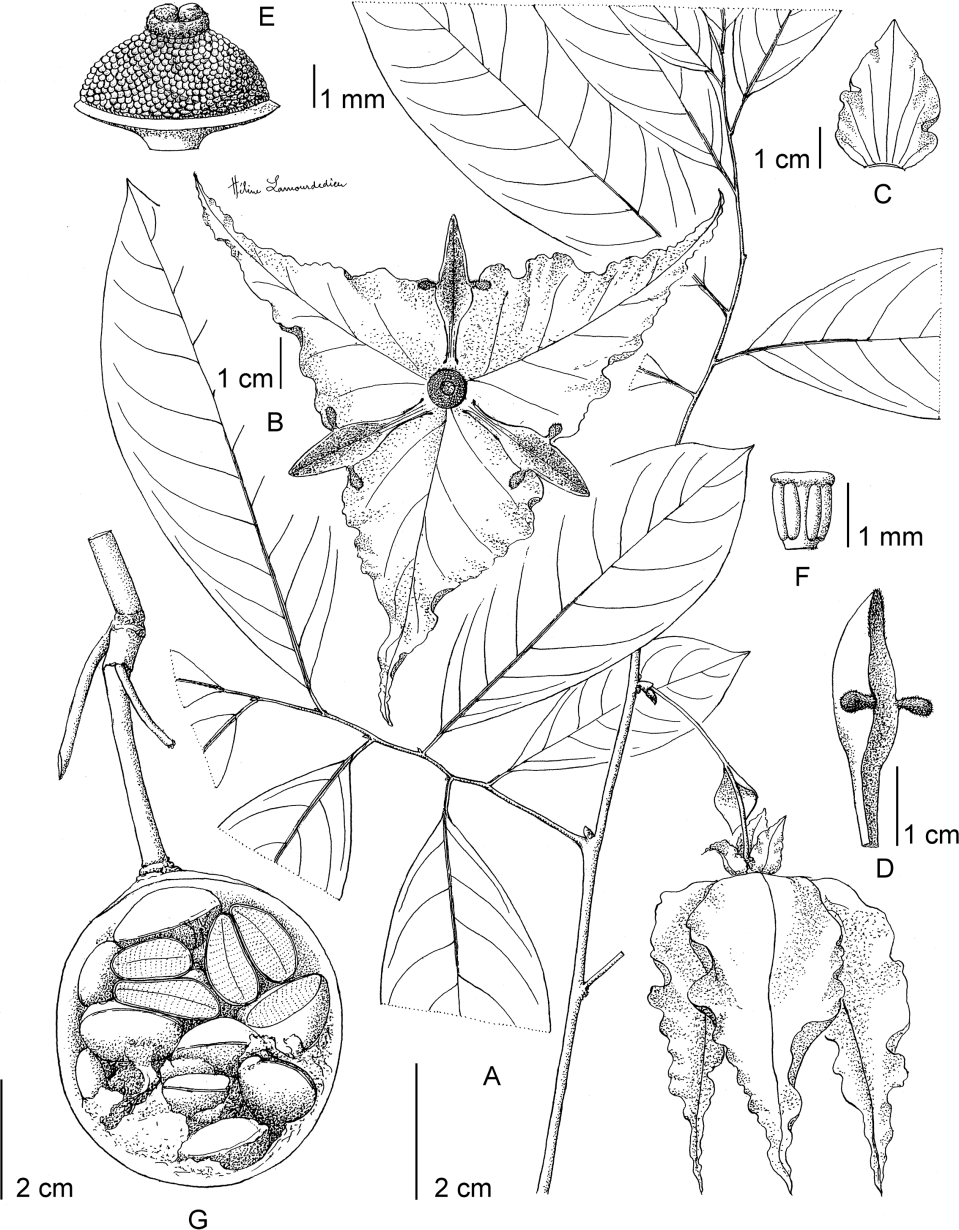
*Monodoratenuifolia***A** flowering branch **B** opened flower **C** sepal **D** inner petal **E** androecium and stigma **F** stamen **G** sectioned fruit, and seeds showing rumination **A–G** from *Chevalier 18342*. Drawings by Hélène Lamourdedieu, Publications Scientifiques du Muséum national d’Histoire naturelle, Paris; modified from Le Thomas (1969a, pl. 62, p. 341).

Figs 76 , 77 ; Map 10B

= Monodora cabrae De Wild., Bull. Soc. Roy. Bot. Belgique 40: 64 1902. Type. Democratic Republic of the Congo. Tchoa, Cabra A.F.F. 2, Dec 1896: holotype: BR[BR0000008801722]; isotypes: BR[BR0000008802385; BR000000880205].

#### Type.

Nigeria. Lagos State; Eppah, *Barter C. 3298*, no date: holotype: K[K000199032].

#### Description.

Tree, 10–20 m tall, d.b.h. up to 60 cm; stilt roots or buttresses absent. Indumentum of simple hairs; old leafless branches glabrous, young foliate branches glabrous. Leaves: petiole 2–7 mm long, 1–2 mm in diameter, glabrous, grooved, blade inserted on the side of the petiole; blade 6–21 cm long, 2–7.5 cm wide, ovate to elliptic, apex acuminate, acumen 0.5–1 cm long, base cuneate, coriaceous to papyraceous, below glabrous when young and old, above glabrous when young and old, concolorous; midrib raised above, above glabrous when young and old, below glabrous when young and old; secondary veins 9 to 15 pairs, glabrous below; tertiary venation intermediate. Individuals bisexual; inflorescences ramiflorous on old leafless branches, leaf opposed or extra axillary. Flowers with 9 perianth parts in 3 whorls, 1 per inflorescence; pedicel 25–75 mm long, 1–2 mm in diameter, glabrous; in fruit 30–70 mm long, 3–4 mm in diameter, glabrous; bracts 1, upper only, towards the upper half of pedicel, 55–65 mm long, 10–30 mm wide; sepals 3, valvate, free, 10–35 mm long, 4–16 mm wide, ovate, apex rounded to attenuate, base truncate, green with red-brown markings, pubescent towards margins outside, pubescent towards margins inside, margins wavy; petals basally fused, tube 2–3 mm long, inner and outer whorl differentiated, outer petals longer than inner; outer petals 3, 30–90 mm long, 25–30 mm wide, ovate, apex acute to rounded, base truncate, yellow-greenish, streaked with red-brown, base shading into bright white, margins wavy, glabrous outside, glabrous inside; inner petals 3, valvate, 10–35 mm long, 6–10 mm wide, **cochleate, with two small oblong and pubescent lateral appendages around mid-height**, apex acute to rounded, base narrowed into a claw 1 mm long, green to white streaked with red brown, margins flat, pubescent towards margins on both sides; receptacle convex to flat; stamens 286 to 337, in 10 to 13 rows, ca. 1 mm long, broad; connective discoid, pubescent, white; staminodes absent; carpels fused into a single structure, 2–3 mm long, stigma bilobed, glabrous. Fruit syncarpous, 45–75 mm long, 20–30 mm in diameter, globose, apex rounded, glabrous, **covered with a greyish wax when young**, faintly ribbed, green when ripe; seeds 12–17 mm long, 10–13 mm in diameter, ellipsoid; aril absent.

#### Distribution.

A widespread species across West and central Africa, from Guinee-Bissau to the Democratic Republic of the Congo; in Cameroon known from Central, East, Littoral, South or South-West regions.

#### Habitat.

A common species with a wide ecological amplitude, in evergreen primary and secondary rain forests, gallery forests, and disturbed and deciduous forests, sometimes in gallery forest (Benin), on sandy soils. Altitude 0–800 m a.s.l.

#### Local and common names known in Cameroon.

Orchid tree, African nutmeg (English) (Burkill 1985).

#### IUCN conservation status.

Least Concern (LC) (Cosiaux et al. 2019ab).

#### Uses in Cameroon.

None reported.

#### Notes.

﻿﻿﻿*Monodoratenuifolia* is characterized by unique cochleate inner petals with two small oblong hairy appendages at the middle. The young fruits are covered by a greyish wax.

#### Specimens examined.

**Central Region**: Etwa 125 km NO Jaunde, 4.63°N, 12.3°E, *01 February 1914*, *Mildbraed G.W.J.* 8301 (K). **East Region**: 60 km south of Yokadouma 30 km after Ngato 15 km after river ALPICAM ‘base de vie’ then 40 km on forestry road starting 4 km before Maséa village, 3.07°N, 14.65°E, *07 March 2019*, *Couvreur T.L.P.* 1218 (MPU,WAG,YA); 69 km south of Yokadouma 30 km after Ngato 15 km after river ALPICAM ‘base de vie’ then 40 km on forestry road starting 4 km before Maséa village, 3.07°N, 14.64°E, *08 March 2019*, *Couvreur T.L.P.* 1233 (MPU,WAG,YA); Somalomo, 3.33°N, 12.75°E, *17 March 2017*, *Kamdem N.* 483 (YA). **Littoral Region**: Piste Sole-Koum 20 km NW de Yabassi, 4.52°N, 9.773°E, *14 March 1976*, *Letouzey R.* 14426 (P,YA). **South Region**: ca 8 km W of Bipindi 59 km from Kribi near Madoungou, 3.06°N, 10.33°E, *16 February 1970*, *Bos J.J.* 6365 (B,BR,L,L,M,MO,P,WAG,YA); N´Koemvone, 2.8°N, 11.13°E, *13 December 1974*, *de Wilde J.J.F.E* 7836 (B,BR,K,MO,P,U,WAG,YA); Nkoemvone, 2.81°N, 11.13°E, *03 July 1975*, *de Wilde J.J.F.E* 8399 (WAG); Près Mekomo (8 km SW confluent Dja & Lobo), 3.21°N, 12.36°E, *19 March 1962*, *Letouzey R.* 4578 (P,WAG,YA); Bipindi, 3.08°N, 10.42°E, *1899*, *Zenker G.A.* 1938 (A,B,BM,BR,G,K,L,P,S,WAG); Bipindi, 3.08°N, 10.42°E, *01 January 1900*, *Zenker G.A.* 2251 (A,B,BM,G,K,L,P,S,WAG); Bipindi, 3.08°N, 10.42°E, *01 January 1904*, *Zenker G.A.* 2727 (BM,BR,K,L,M,P,S,WAG); Bipindi, 3.08°N, 10.41°E, *01 January 1908*, *Zenker G.A.* 3793 (G,K); Bipindi, 3.08°N, 10.41°E, *01 January 1912*, *Zenker G.A.* 4317 (B,BM,BR,G,K,L,MO,P,S). **South-West Region**: between 200–600 m alt above Bakingili, 4.08°N, 9.05°E, *21 December 1993*, *Cheek M.* 5840 (K,YA); West of Kola, 4.83°N, 9.733°E, *27 October 1998*, *Cheek M.* 9399 (K,WAG,YA); Bayang Mbo Wildlife Sanctuary after Mbu river, 5.35°N, 9.503°E, *26 March 2016*, *Couvreur T.L.P.* 1019 (WAG,YA); A 11 km au Sud Est de Molobo (village situé à 50 km au Sud de Batouri), 4.02°N, 14.32°E, *21 July 1963*, *Letouzey R.* 5459 (P,YA); Ambas Bay (Victoria NDLR), 4.01°N, 9.226°E, *1861*, *Mann G.* 111 (K); Around Masaka-Batanga, 5.1°N, 9.17°E, *24 March 1988*, *Thomas D.W.* 7750 (P,YA).

### ﻿﻿﻿﻿ Monodora undulata

Taxon classificationPlantaeMagnolialesAnnonaceae

(P.Beauv.) Couvreur, Reveal. Secrets African Annon.: ﻿Isolona & ﻿Monodora: 246, 2008

2A4245F5-42D7-5076-964D-9CEBC646987A

**Figure 78.**
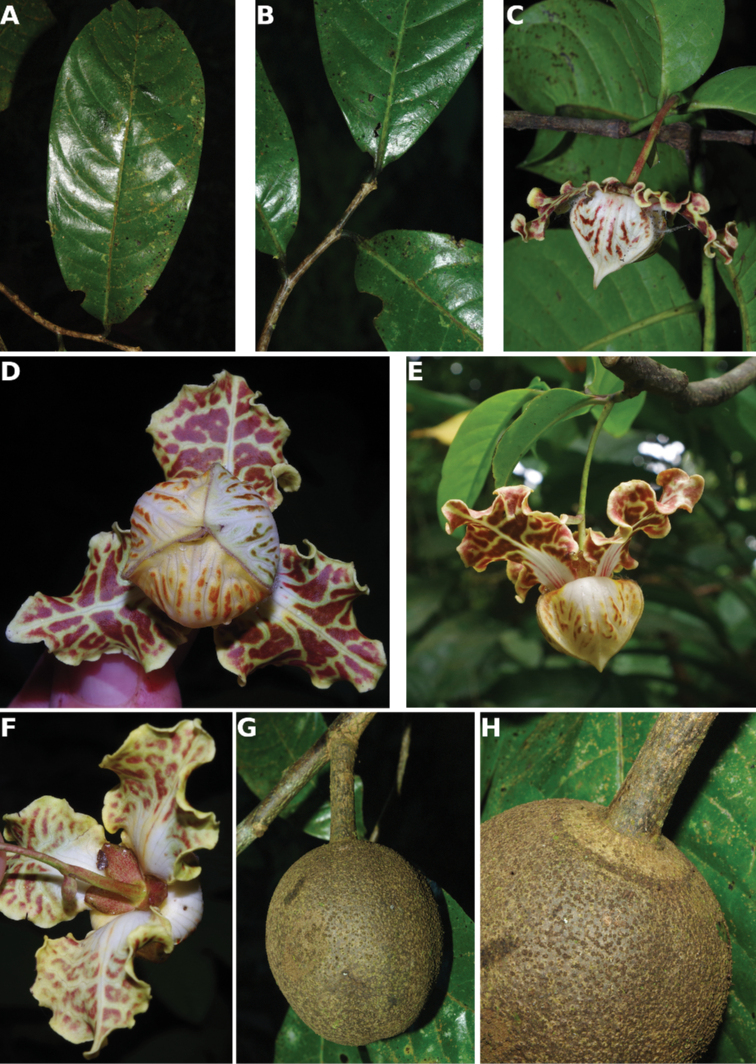
*Monodoraundulata***A** leaf, upper view **B** base of leaf blade, upper view **C** young flower **D** flower, top view, note connivent inner petals forming a floral chamber **E** flower, side view **F** flower, bottom view, note small rounded bract **G** syncarpous fruit, side view **H** detail of rugulose fruit surface **A, B, G, H** Couvreur *s.n.***C** Couvreur 1061, Mt Cameroon **D–F***Couvreur 1042*, Mt Cameroon, Cameroon. Photos Thomas L.P. Couvreur.

**Figure 79.**
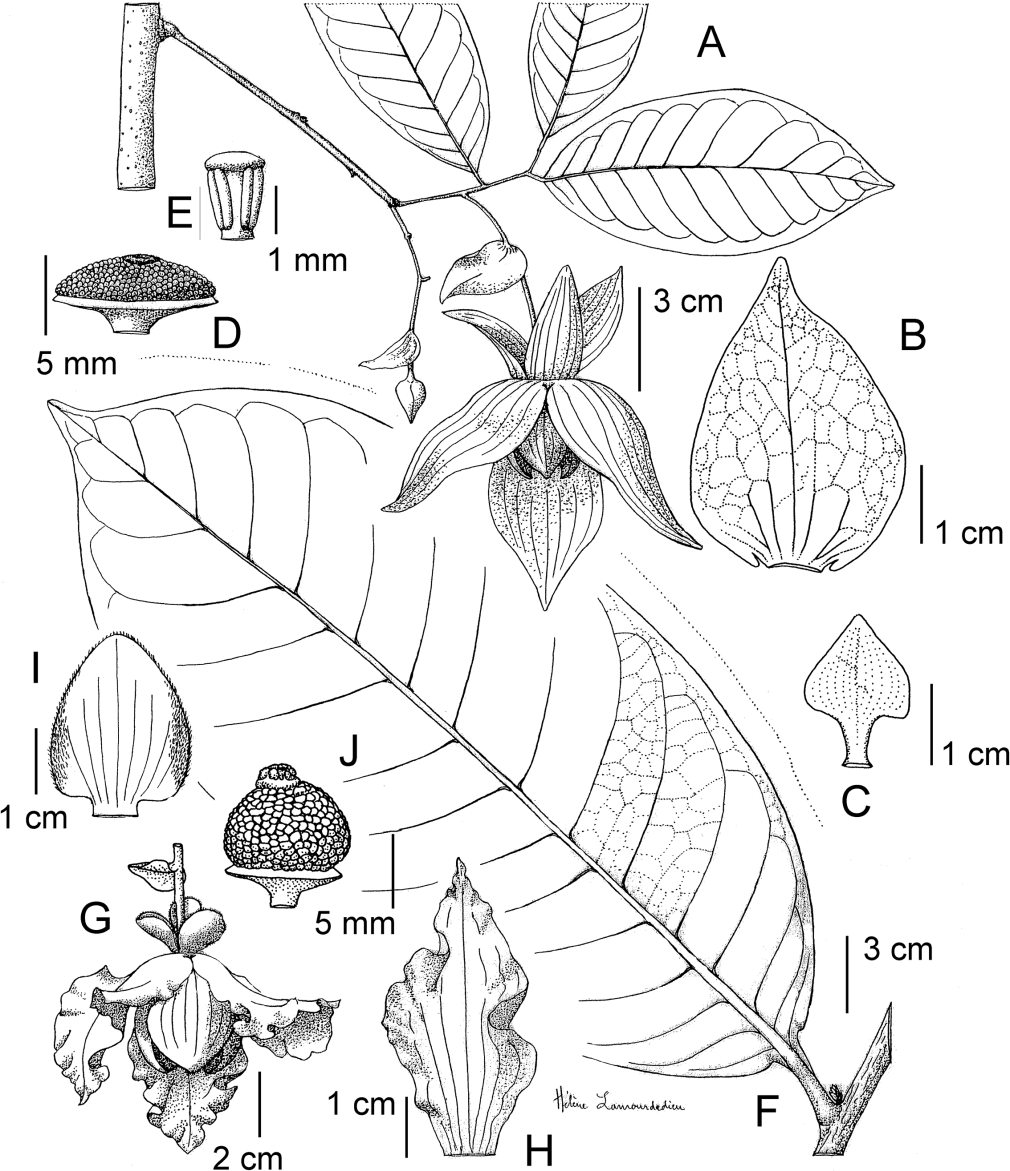
*Monodorazenkeri***A** flowering branch **B** outer petal, inside view **C** inner petal, inside view **D** androecium with missing stigma **E** stamen, front view. *Monodoraundulata***F** leaf **G** flower **H** outer petal, inside view **I** inner petal, inside view **J** androecium and stigma **A** from Zenker 776 **B–E** from *Breteler 2747***F–I** from *Preuss 1314***J** from *Bos 3227*. Drawings by Hélène Lamourdedieu, Publications Scientifiques du Muséum national d’Histoire naturelle, Paris; Drawings **B, C** and **J** Hans de Vries (Couvreur 2009, fig. 69, p. 129).

Figs 78 , 79 ; Map 10C

≡ Unona undulata (Palisot de Beauvois) Dunal, Monogr. Anonac.: 111, 1817.
= Monodora brevipes Benth., Trans. Linn. Soc. London 23(3): 475, 1862. Type. Saõ Tomé & Principe: Principe Island, *Mann G. 1115*, 1861: lectotype, designated by Couvreur (2009), p. 246; sheet here designated: K[K000199026]; isotypes: K[K000199025]; P[P00363308].
= Monodora preussii Engl. & Diels, Notizbl. Königl. Bot. Gart. Berlin 2: 301 1899. Type. Cameroon. South Region, Victoria (Limbe), *Preuss, P.R. 1314*, 1898: lectotype, designated by Couvreur (2009): 246: K[K000105558]; isolectotypes: A[A00295524]; EA; PH[PH00018358]; S[S10-21404]; Z[Z-000034547].

#### Type.

Nigeria. no region; no location, *Palisot de Beauvois A.M.F.J. s.n.*, no date: holotype: G-DC, scanned image[excluding fruits] [G00011671].

#### Description.

Tree, up to 20 m tall, d.b.h. up to 100 cm; stilt roots or buttresses absent. Indumentum of simple hairs; old leafless branches glabrous, young foliate branches glabrous. Leaves: petiole 2–14 mm long, 1–2 mm in diameter, glabrous, slightly grooved, blade inserted on the side of the petiole; blade 10–40 cm long, 8–15 cm wide, oblong to obovate, apex acuminate, acumen 3–9 cm long, base rounded to obtuse, coriaceous to papyraceous, below glabrous when young and old, above glabrous when young and old, concolorous; midrib raised above, above glabrous when young and old, below glabrous when young and old; secondary veins 9 to 17 pairs, glabrous below; tertiary venation reticulate. Individuals bisexual; inflorescences ramiflorous on old or young foliate branches, leaf opposed or extra axillary. Flowers bisexual with 9 perianth parts in 3 whorls, 1 per inflorescence; **pedicel 30–55 mm long**, ca. 1 mm in diameter, glabrous; in fruit 40–50 mm long, 8–10 mm in diameter, glabrous; bracts 1, upper only, towards the upper half or middle of pedicel, 6–10 mm long, 7–11 mm wide; sepals 3, valvate, free, 7–11 mm long, 5–10 mm wide, ovate, apex rounded to obtuse, base truncate, green, glabrous, pubescent towards margins outside, glabrous, pubescent towards margins inside, margins wavy; petals basally fused, tube 2–3 mm long, inner and outer whorl differentiated, outer petals longer than inner; outer petals 3, 25–45 mm long, 15–30 mm wide, ovate, apex acute, base truncate, speckled and streaked yellow and purple, base creamy-white, margins wavy, glabrous, pubescent towards margins outside, glabrous, pubescent towards margins inside; inner petals 3, valvate, 17–27 mm long, 13–20 mm wide, rhombic, apex acute, base narrowed into a claw, claw 2–5 mm long, yellow with brown-purple spots, margins flat, glabrous outside, sparsely pubescent inside; **receptacle strongly convex**; stamens numerous, in 12 to 14 rows, ca. 1 mm long, broad; connective discoid, densely pubescent, cream; staminodes absent; carpels fused into a single structure, 4 mm long, stigma bilobed, slightly capitate, sparsely pubescent. Fruits syncarpous, 60–120 mm long, 40–60 mm in diameter, **ovoid**, apex rounded, **tomentose**, faintly ribbed longitudinally, otherwise **smooth, brown when ripe**; seeds 9–20 mm long, 6–11 mm in diameter, flattened ellipsoid; aril absent.

#### Distribution.

A widespread species, disjunct between West Africa (Sierra Leone to Togo) and Central Africa (Nigeria, Cameroon and São Tome Island); in Cameroon known from East, South, Central, Littoral and South-West regions.

#### Habitat.

A fairly uncommon species even though it has been collected numerous times in Cameroon; in lowland primary and secondary rain forests, along rivers and in swamps. Altitude 0–700 m a.s.l.

#### Local and common names known in Cameroon.

Yellow-Flowering Nutmeg (English) (Cooper and Record 1931).

#### IUCN conservation status.

Least Concern (LC) (Cosiaux et al. 2019ac).

#### Uses in Cameroon.

***food***: seeds used for sauces, as condiment or spice; ***medicine***: bark used against venereal diseases.

#### Notes.

﻿﻿﻿*Monodoraundulata* closely resembles ﻿*M.myristica*; both share a strongly convex receptacle, large leaves, and completely connivent inner petals; but ﻿*M.undulata* is distinguished from ﻿*M.myristica* by its smaller flowers with shorter pedicels, cup-shaped and non-undulate upper bract, and ovoid densely tomentose fruits.

#### Specimens examined.

**Central Region**: Reserve forestière de Makak, 3.48°N, 11.01°E, *14 December 1967*, *Bamps P.R.J.* 1449 (BR,YA). **East Region**: ca 9 km from Bertoua near the road to Doumé, 4.53°N, 13.61°E, *09 December 1961*, *Breteler F.J.* 2182 (K,P,WAG,YA). **Littoral Region**: Ebo Proposed National Park Iboty to Bekob village, 4.46°N, 10.46°E, *14 February 2006*, *Cheek M.* 12928 (K). **South Region**: along main road Kribi-Bipindi, 2.99°N, 10.01°E, *17 February 2012*, *Couvreur T.L.P.* 391 (WAG,YA); Lolodorf, 3.23°N, 10.71°E, *1896*, *Staudt A.* 40 (G,K,P). **South-West Region**: Path above Kupe village, 4.76°N, 9.694°E, *22 May 1996*, *Cable S.* 2544 (K,WAG,YA); Nyangdong volcanic waterfall-whirlpool path, 4.96°N, 9.577°E, *27 March 2003*, *Cheek M.* 11464 (K); Mount Cameroon National Park Bakinguili trail above Bakinguili village, 4.07°N, 9.051°E, *02 April 2016*, *Couvreur T.L.P.* 1042 (WAG,YA); Nyasoso village on max’s trail to Mt 4.82°N, 9.692°E, *05 April 2016*, *Couvreur T.L.P.* 1061 (WAG,YA); Kupe village to Loum State Forest, 4.73°N, 9.716°E, *29 May 1996*, *Etuge M.* 2017 (K,MO,P,WAG,YA); Korup National Park, 5.06°N, 8.855°E, *06 February 1998*, *Kenfack D.* 1027 (MO,WAG); 6 km W of Bota rocky coast with volcanic boulders, 4.01°N, 9.144°E, *31 August 1972*, *Leeuwenberg A.J.M.* 10295 (BR,MO,P,WAG,YA); Limbe (Victoria) Bimbia road, 4.00°N, 13.21°E, *01 February 1929*, *Maitland T.D.* 408 (K); Johann-Albrechtshöhe[Kumba] area 4.63°N, 9.416°E, *1896*, *Staudt A.* 495 (A); Southern slope of Mount above Batoke, 4.03°N, 9.1°E, *25 January 1984*, *Thomas D.W.* 3025 (B,BR,MO,P,YA); Small Koto, 4.31°N, 9.066°E, *07 March 1985*, *Thomas D.W.* 4516 (K,MO,P,YA); Cameroon Mountain, 4.31°N, 9.066°E, *01 June 1985*, *Thomas D.W.* 4814 (BR,MO,P,WAG).

### ﻿﻿﻿﻿ Monodora zenkeri

Taxon classificationPlantaeMagnolialesAnnonaceae

Engl., Notizbl. Königl. Bot. Gart. Berlin 2: 301, 1899

D7164877-10E8-5046-B27C-3319BB4E8202

Fig. 79 ; Map 10D

#### Type.

Cameroon. Central Region; Yaoundé, *Zenker G.A. 776*, 1896: holotype: B[B 10 015406]; isotypes: COI[COI00077202]; G[G00011721]; K[K000199027]; NY[NY00026141]; P[P00363263].

#### Description.

Leaning tree [or liana?], to 6 m tall, d.b.h. up to 6 cm; stilt roots or buttresses absent. Indumentum of simple hairs; old leafless branches glabrous, young foliate branches sparsely pubescent to glabrous. Leaves: petiole 2–4 mm long, 1 mm in diameter, glabrous, grooved, blade inserted on the side of the petiole; blade 10–15 cm long, 8–14 cm wide, elliptic to obovate, apex acuminate, acumen 3–6 cm long, base rounded to obtuse, coriaceous to papyraceous, below glabrous when young and old, above glabrous when young and old, concolorous; midrib raised above, above glabrous when young and old, below glabrous when young and old; secondary veins 10 to 13 pairs, glabrous below; tertiary venation intermediate. Individuals bisexual; inflorescences ramiflorous on young foliate branches, leaf opposed or extra axillary. Flowers bisexual with 9 perianth parts in 3 whorls, 1 per inflorescence; pedicel 28–50 mm long, 1 mm in diameter, sparsely pubescent; in fruit unknown; bracts 1, towards the upper half or middle of pedicel, 11–20 mm long, 10–13 mm wide; sepals 3, valvate, free, 2–5 mm long, 2–4 mm wide, ovate, apex acute, base truncate, green to brown-red, glabrous outside, glabrous inside, margins flat; petals basally fused, tube 4–7 mm long, inner and outer whorl differentiated, outer petals longer than inner; outer petals 3, 35–45 mm long, 20–28 mm wide, ovate, apex acute to obtuse, base truncate flanked by two small lobes, speckled and streaked yellow-purple, base creamy-white, **margins flat**, sparsely pubescent outside, sparsely pubescent to glabrous inside; inner petals 3, valvate, 9–13 mm long, 13–16 mm wide, triangular, apex acute, base narrowed into a claw, claw 4.0–7 mm long, pale green with red spots, margins flat, folded outwards, glabrous inside, pubescent outside; receptacle convex to flat; stamens numerous, in 9 to 10 rows, 1 mm long, broad; connective discoid, glabrous; staminodes absent; carpels fused into a single structure, 1 mm long, stigma bilobed, slightly capitate. Fruits unknown.

#### Distribution.

endemic to Cameroon; known from Central, East, South regions.

#### Habitat.

A rare species, not collected recently; in secondary and disturbed lowland rain forests. Altitude 600–700 m a.s.l.

#### Local and common names known in Cameroon.

None recorded.

#### IUCN conservation status.

Not evaluated (certainly threatened).

#### Uses in Cameroon.

None reported.

#### Notes.

﻿﻿﻿*Monodorazenkeri* is easily distinguished by the presence of two small lobes at the base of the outer petals. Some labels indicate that it is a liana, probably a strongly leaning tree, mainly near large rocks. In this sense it resembles ﻿*M.crispata* in its lianescent habit.

#### Specimens examined.

**Central Region**: ca. 4 km NE of Otélé near the road to Yaoundé, 3.58°N, 11.25°E, *28 March 1964*, *de Wilde J.J.F.E* 2249 (BR,P,WAG,YA); Etwa 195 km NO Jaunde, 5.21°N, 12.9°E, *01 January 1914*, *Mildbraed G.W.J.* 8476 (K); Mt Ngoro à 38 km au N de Bafia (piedmont), 4.93°N, 11.38°E, *01 May 1978*, *Ngameni B.K.* 113 (P,YA); Yaoundé, 3.87°N, 11.52°E, *1896*, *Zenker G.A.* 776 (B,COI,G,K,P). **East Region**: Bertoua, 4.58°N, 13.73°E, *27 April 1961*, *Breteler F.J.* 1319 (WAG); 6 km along road south to Doumé, 4.38°N, 12.91°E, *10 April 1962*, *Breteler F.J.* 2747 (BR,K,P,WAG,YA); Deng Deng, 5.16°N, 13.58°E, *01 April 1914*, *Mildbraed G.W.J.* 8850 (K); Bertoua, 4.58°N, 13.68°E, *27 May 1955*, *Nana P.* 98 (P,YA). **South Region**: Nkoldjobe dans le massif du Mbam-Minkom 18 km Nord-Ouest de Yaoundé, 3.93°N, 11.35°E, *16 April 1984*, *Achoundong G.* 1056 (YA); Bitye river, 3.02°N, 12.37°E, *01 January 1920*, *Bates G.L.* 1244 (COI); Near village Oveng 27 km from Sangmélima along road to Yaoundé, 3.09°N, 11.90°E, *21 March 1962*, *Breteler F.J.* 2683 (BR,K,P,U,WAG,YA).

### ﻿﻿ Neostenanthera

Taxon classificationPlantaeMagnolialesAnnonaceae

Exell, J. Bot. 73 (Suppl.): 5, 1935

825BBC9B-CB20-50C2-BA52-E32916FBB610

= Stenanthera Engl. & Diels, Notizbl. Königl. Bot. Gart. Berlin 3: 57, 1900; ﻿Oxymitra sect. ﻿Stenanthera Oliv., Fl. Trop. Afr. 1: 32. 1868.

#### Type species.

*Oxymitrahamata* Benth. (= ﻿*Neostenantherahamata* (Benth.) Exell).

#### Description.

**Trees to shrubs, 3**–**25 m tall, d.b.h. 3–30 cm**; stilt roots or buttresses absent. Indumentum of simple hairs. Leaves: petiole 2–14 mm long, 1–3 mm in diameter; blade 6–31 cm long, 2–10 cm wide, ovate or elliptic or oblong, apex acuminate or acute, acumen 0.5–2.4 cm long, base rounded or obtuse or cuneate, discolorous, whitish below or concolorous; midrib sunken or flat; secondary veins 10 to 24 pairs; tertiary venation percurrent. Individuals bisexual; inflorescences cauliflorous or ramiflorous on old leafless branches, leaf opposed or extra axillary. Flowers with 9 perianth parts in 3 whorls, 1 to 8 per inflorescence; pedicel 5–46 mm long, ca. 1 mm in diameter; in fruit 20–60 mm long, 2–4 mm in diameter; bract 1, basal, 1–3 mm long, ca. 1 mm wide; sepals 3, valvate, free, 1–4 mm long, 1–3 mm wide, triangular or ovate or semi-circular, apex acute or acuminate, base truncate; petals free, valvate, outer petals longer than inner; outer petals 3, 6–33 mm long, 1–15 mm wide, elliptic or narrowly triangular, apex acute or attenuate, base narrowed and concave; inner petals 3, valvate, 1–10 mm long, 0.6–5 mm wide, triangular, apex acute, base broad and concave; stamens 30 to 170, in 4 to 6 rows, 2–3 mm long, linear or broad; connective tongue-shaped; staminodes absent; carpels free, 20 to 109, ovary 1–2.5 mm long, stigma cylindrical or filiform. **Monocarps sessile or stipitate**, stipes 6–23 mm long; monocarps 7 to 144, 10–30 mm long, 10–20 mm in diameter, ellipsoid, apex apiculate, rounded or pyramidal in shape; **seed 1**, 4–14 mm long, 2–11 mm in diameter, ellipsoid; aril absent.

#### Taxonomy.

Fero et al. (2014) [in part]; Le Thomas (1965b).

A widespread genus with five species (including ﻿﻿﻿*Boutiqueaplatypetala*, see under ﻿*N.neurosericea*), three species known from Cameroon, none endemic (the name ﻿﻿﻿*Boutiqueaplatypetala* is placed in synonymy with ﻿*N.neurosericea* see below).

When sterile, this genus can be confused with ﻿*Monanthotaxis* both being lianas with a whitish lower side of leaf blade and percurrent tertiary venation. They are however unmistakable when in flower or fruit.

﻿﻿﻿*Neostenantheragabonensis* occurs in Gabon and West Africa (Fero et al. 2014). To date it has not been collected from Cameroon as far as we know, but likely does occur there. We have added this species to the key.

### ﻿Key to the species of ﻿*Neostenanthera* in Cameroon

**Table:** 

1	Large trees taller than 8 m and larger than10 cm in d.b.h.; flowering peduncles ramified (arbuscular-like); flowers usually cauliflorous, sometimes ramiflorous on old leafless branches, rarely on young leafy branches; petals brown and thick; monocarps tomentose brown	﻿***N.robsonii***
–	Small trees smaller than 10 m tall and smaller than10 cm in d.b.h.; flowering peduncles not ramified (unbranched); petals flat, yellow to green; monocarps glabrous.	**2**
2	Young foliate branches densely pubescent, lower side of leaf blades covered with erect slightly curly hairs, outer petals 8–15 mm wide, monocarps sessile, pyramidal	﻿***N.neurosericea***
–	Young foliate branches glabrous or sparsely pubescent, lower side of leaf blades covered with appressed short and straight hairs, monocarps stipitate, ellipsoid to fusiform	**3**
3	Inflorescences single or more rarely 2 flowered; monocarps fusiform, longitudinally ribbed (species not observed in Cameroon yet)	﻿***N.gabonensis***
–	Inflorescences 2 to 4 flowered; monocarps ellipsoid, not ribbed	﻿***N.myristicifolia***

### ﻿﻿﻿﻿ Neostenanthera myristicifolia

Taxon classificationPlantaeMagnolialesAnnonaceae

(Oliv.) Exell, J. Bot. 73 (Suppl.): 6, 1935

F6DEAB2F-1ED6-5D6E-BE35-AAEF74D10292

**Figure 80.**
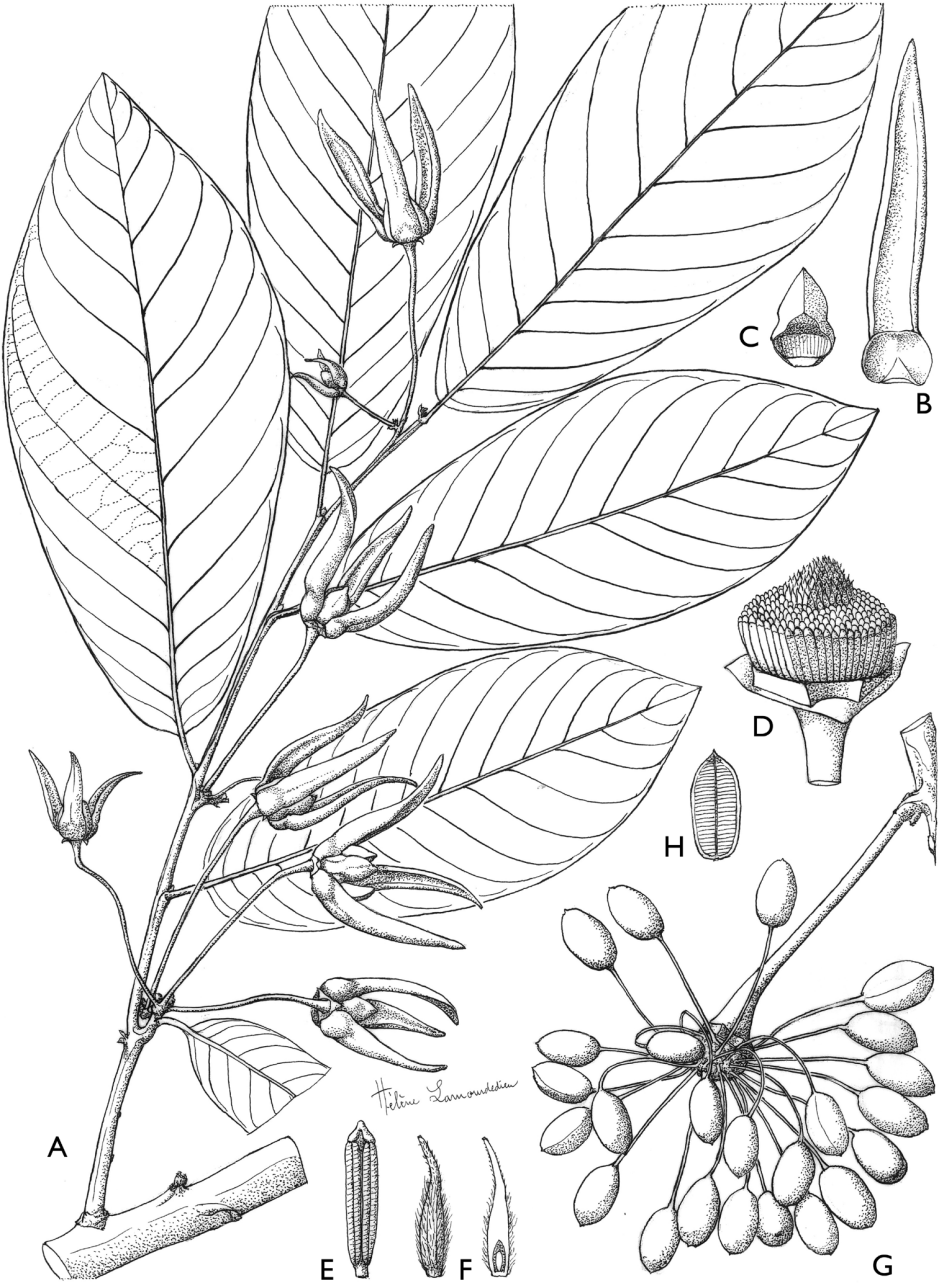
*Neostenantheramyristicifolia***A** flowering branch **B** outer petal, inner view **C** inner petal, inner view **D** receptacle, all petals removed **E** stamen, front view, note septate anthers **F** carpel, side view and detail of single basal ovule **G** fruit **H** longitudinal section of seed **A–H** from *Hallé & Le Thomas 382*. Drawings by Hélène Lamourdedieu, Publications Scientifiques du Muséum national d’Histoire naturelle, Paris; modified from Le Thomas (1969b, pl. 35, p. 191).

**Figure 81.**
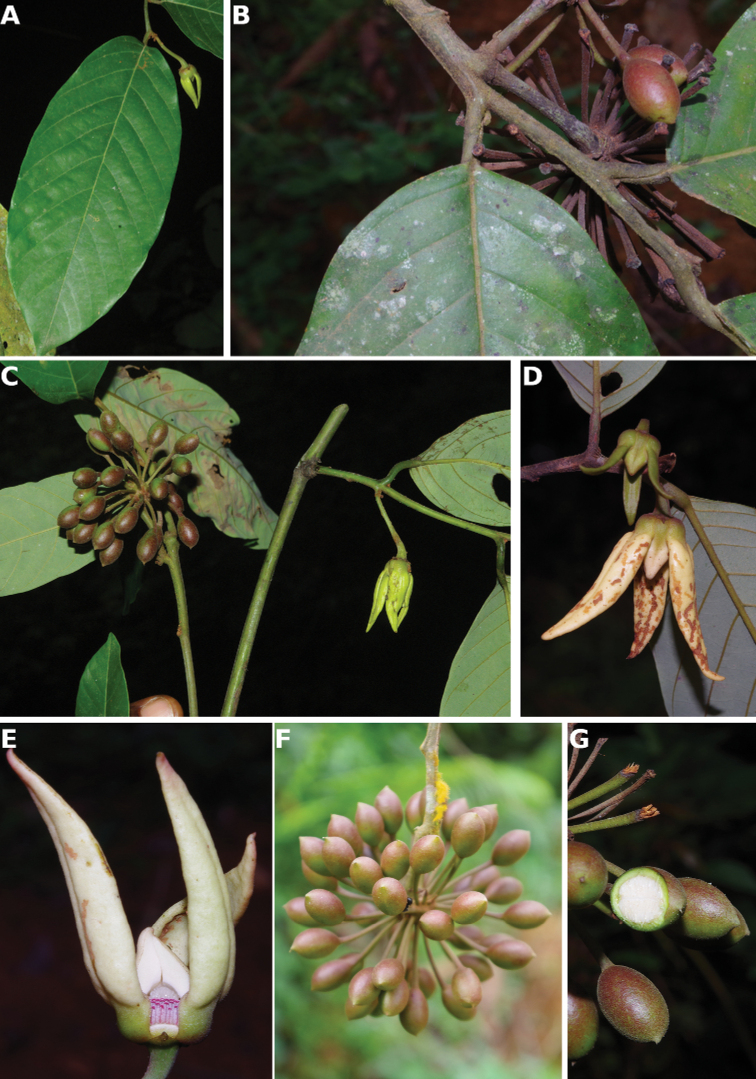
*Neostenantheramyristicifolia***A** leaf with young flower **B** base of leaf blade, upper view **C** flowering and fruiting branches **D** detail of mature flower **E** detail of flower, one outer and one inner petal removed, note blue stamens **F** fruit **G** detail of stipitate monocarp, showing longitudinal section of the single seed **A***Couvreur 422*, Méfou, Cameroon **B–G***Couvreur 841*, Gabon. Photos Thomas L.P. Couvreur.

Figs 80 , 81 ; Map 10E

≡ Oxymitra myristicifolia Oliv., Fl. Trop. Afr. 1: 33, 1868; ﻿﻿Stenantheramyristicifolia (Oliv.) Engl. & Diels, Notizbl. Königl. Bot. Gart. Berlin 3: 57, 1900.
= Stenanthera pluriflora De Wild., Ann. Mus. Congo Belge, Bot. sér. 5, 1(1): 45, 1903. Type. Democratic Republic of the Congo. Bas Congo, Kisantu, *Gillet J. 168*, 1899: lectotype, designated by Boutique (1951b), p. 339, sheet here designated: BR[BR0000008802712]; isotypes: BR[BR0000008802040, BR0000008801715].

#### Type.

Nigeria. Cross River State; Old Calabar, *Thomson W.C. 134*, no date: holotype: K[K000199047].

#### Description.

Tree to shrub or scandent shrub, 3–5 m tall, d.b.h. 6–20 cm; stilt roots or buttresses absent. Indumentum of simple hairs; old leafless branches glabrous, **young foliate branches sparsely pubescent to glabrous**. Leaves: petiole 4–6 mm long, 1–2 mm in diameter, pubescent, slightly grooved to cylindrical, blade inserted on top of the petiole; blade 8.5–25.5 cm long, 3.7–9 cm wide, oblong to elliptic, apex acuminate to acute, acumen 0.2–1.8 cm long, base rounded to obtuse, papyraceous, **below pubescent with short appressed straight hairs when young and old**, above glabrous to pubescent when young, glabrous to pubescent when old, discolorous, whitish below; midrib impressed, above glabrous when young and old, below glabrous when young and old; secondary veins 10 to 18 pairs, glabrous below; tertiary venation percurrent. Individuals bisexual; inflorescences ramiflorous, on foliate or leafless branches, leaf opposed or extra axillary. Flowers with 9 perianth parts in 3 whorls, **(1)2 to 4 per inflorescence**; **peduncle 1–2 mm long, unbranched**; pedicel 12–38 mm long, ca. 1 mm in diameter, pubescent; in fruit 20–40 mm long, 3–4 mm in diameter, sparsely pubescent; bract 1, basal, 1–3 mm long, ca. 1 mm wide; sepals 3, valvate, free, 1–4 mm long, 1–3 mm wide, triangular to ovate, apex acute to acuminate, base acute, brown-green, pubescent outside, pubescent inside, margins flat; petals free, outer petals longer than inner; **outer petals 3, 6.5–29 mm long in total, 2–5(6) mm wide above the claw**, narrowly triangular, apex attenuate, base suborbicular, basal part 4–6 mm wide, light green to light yellow, margins flat, pubescent to glabrous outside, pubescent inside; inner petals 3, valvate, 3.4–10.9 mm long, 1–3 mm wide, triangular, apex acute, base broad and concave, 3–5 mm wide, margins flat, pubescent to glabrous outside, pubescent inside; stamens 135–156, in 4 to 5 rows, ca. 2 mm long, linear; connective tongue shaped, glabrous, red; staminodes absent; carpels free, 67 to 109, ovary ca. 1 mm long, stigma cylindrical, glabrous. **Monocarps stipitate, stipes 17–23 mm long**, ca. 1 mm in diameter; monocarps 15(22) to 48(60), 9–20 mm long, 6–13 mm in diameter, ellipsoid, apex shortly apiculate, sparsely pubescent to glabrous, smooth, not ribbed, brown turning wine red when ripe; seed 1 per monocarp, 4–18 mm long, 2–10 mm in diameter, ellipsoid; aril absent.

#### Distribution.

From Nigeria to Democratic Republic of the Congo; in Cameroon known from East, South, Central, South-West and South West regions.

#### Habitat.

A common species across its range; in secondary forests and along roads and swampy areas. Altitude 300–1000 m a.s.l.

#### Local and common names known in Cameroon.

None recorded.

#### IUCN conservation status.

Least Concern (LC) (Cosiaux et al. 2019ad).

#### Uses in Cameroon.

None reported.

#### Notes.

﻿﻿﻿*Neostenantheramyristicifolia* is distinguishable by its fasciculate inflorescences (rarely solitary flowers), flowers and pedicels not tomentose and long stipitate monocarps.

#### Specimens examined.

**Central Region**: Between Somalomo and Milon 69 km SE Akonolinga, 3.77°N, 12.25°E, *18 June 1981*, *Asonganyi J.N.* 310 (P,YA); NE of Ndangan I on logging trail into Mefou NP, 3.62°N, 11.59°E, *23 March 2004*, *Cheek M.* 11965 (BR,K,K,MO,P,WAG,YA); Méfou National Park just after Ape Action Africa center along road, 3.62°N, 11.58°E, *24 April 2013*, *Couvreur T.L.P.* 422 (WAG,YA); Banlieu de Yaoundé au bord de l’étang Atemengue, 3.87°N, 11.52°E, *14 April 1960*, *Endengle E.* 116 (P,YA); Goura, 4.55°N, 11.4°E, *01 November 1938*, *Jacques-Félix H.* 2340 (P); Yaoundé, 3.87°N, 11.52°E, *01 August 1939*, *Jacques-Félix H.* 4803 (P); Kourou (Yoko), 5.03°N, 12.42°E, *10 July 1959*, *Letouzey R.* 2373 (P,YA); Près de Mendong (25 km N Akonolinga), 3.77°N, 12.25°E, *07 March 1962*, *Letouzey R.* 4486 (P,YA); Issandja (Ntui), 4.77°N, 11.77°E, *19 December 1969*, *Letouzey R.* 9726 (P); Ngolep Mt, 4.95°N, 11.38°E, *27 April 1978*, *Ngameni B.K.* 44 (P,YA); Près rivière Malo non loin de Ndikinimeki, 4.77°N, 10.83°E, *08 November 1983*, *Nkongmeneck B.A.* 518 (P,YA); Mefou National Park, 3.61°N, 11.56°E, *27 March 2004*, *Onana J.M.* 2870 (BR,G,K,YA); Ngoro (28 km NE Bafia Cameroun), 4.95°N, 11.38°E, *29 March 1963*, *Raynal A.* 10569 (P,WAG,YA); Nkolbison (8 km W Yaoundé Cameroun), 3.88°N, 11.45°E, *10 February 1963*, *Raynal J.* 9560 (P,YA). **East Region**: 7 km along road to Batouri and Bétaré Oya, 4.58°N, 13.68°E, *16 March 1961*, *Breteler F.J.* 1220 (A,BR,K,M,P,SL,UC,WAG,YA); Bertoua, 4.58°N, 13.68°E, *13 August 1961*, *Breteler F.J.* 1799 (BR,K,P,WAG,YA); Bertoua, 4.58°N, 13.68°E, *08 November 1960*, *Breteler F.J.* 644 (BR,K,P,WAG,YA); Doumé, 4.23°N, 13.45°E, *12 November 1960*, *Breteler F.J.* 684 (BR,K,M,P,WAG,YA); Piste Mpoundou-Seglendon marécages, 4.1°N, 13.05°E, *16 February 1960*, *Letouzey R.* 3021 (P,YA); Près Goyoum, 5.22°N, 13.38°E, *01 February 1961*, *Letouzey R.* 3350 (P,YA); 70 km route Bertoua-Ndemba I, 4.75°N, 13.43°E, *18 July 1955*, *Nana P.* 171 (P,YA); Bertoua, 4.58°N, 13.68°E, *27 August 1955*, *Nana P.* 240 (P); route Esseleke, 4.58°N, 13.68°E, *13 February 1956*, *Nana P.* 474 (P); 8 km de Deng Deng, 5.23°N, 13.46°E, *10 February 2002*, *Sonké B.* 2671 (BR,BRLU,K,WAG,YA). **South Region**: At Bissombo 59 km SE Akonolinga, 3.28°N, 12.47°E, *12 June 1981*, *Asonganyi J.N.* 275 (YA); Biyi (Ambam), 3.02°N, 12.37°E, *12 February 1963*, *Raynal J.* 9806 (P). **South-West Region**: Korup National Park, 5.33°N, 9.42°E, *18 September 1990*, *Harris D.J.* 2516 (P,YA). **West Region**: Touladen en bas du versant oriental du Mbepit 15 km E Foumbot et 25 km SSW Foumban, 5.52°N, 10.78°E, *26 October 1974*, *Letouzey R.* 13025 (P,WAG,YA).

### ﻿﻿﻿﻿ Neostenanthera neurosericea

Taxon classificationPlantaeMagnolialesAnnonaceae

(Diels) Exell, J. Bot. 73 (Suppl.): 6, 1935

B94F72A2-ADC0-5A84-AEFA-C054A282711F

**Figure 82.**
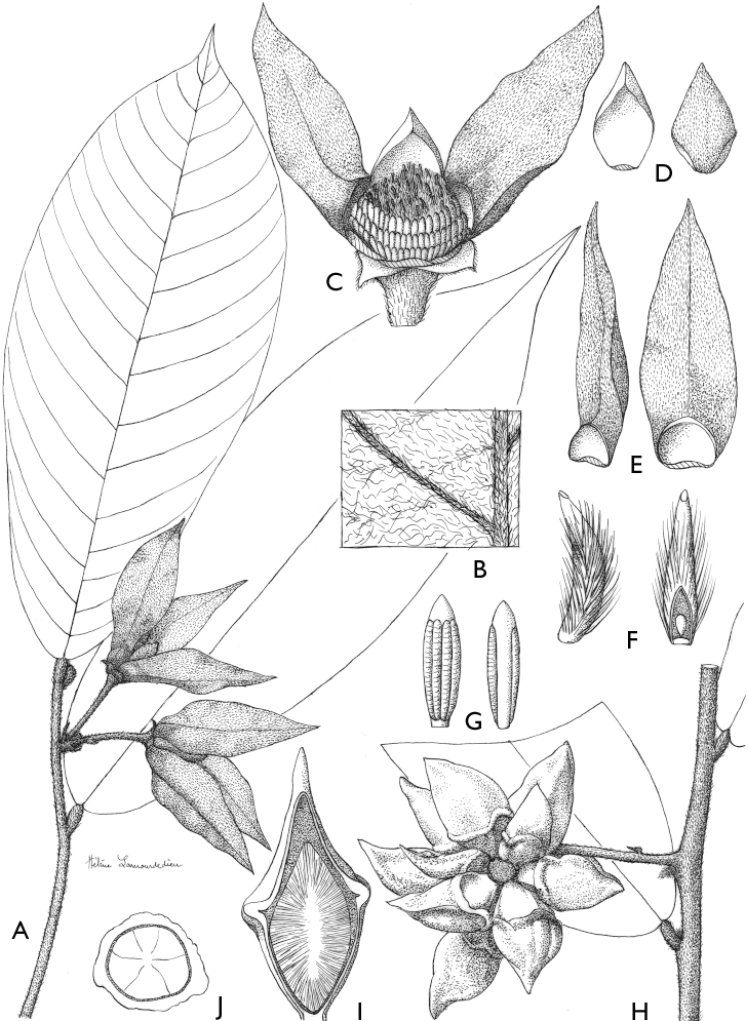
*Neostenantheraneurosericea***A** flowering branch **B** detail of pubescence of leaves, lower side **C** detail of receptacle, 1 outer and 2 inner petals removed **D** inner petals, inner and outer views **E** outer petals, inner and outer views **F** carpel, side view and detail of single basal ovule **G** stamen, front and back views, note septate anthers **H** fruiting branch **I** longitudinal section of a single monocarp showing single seed **J** transversal section of seed **A–C, I, J** from *Letouzey 4092***C–G** from *Raynal 10389*. Drawings by Hélène Lamourdedieu, Publications Scientifiques du Muséum national d’Histoire naturelle, Paris; modified from Le Thomas (1965b, pl. 1, p. 533), originally under *Boutiqueaplatypetala*.

**Figure 83.**
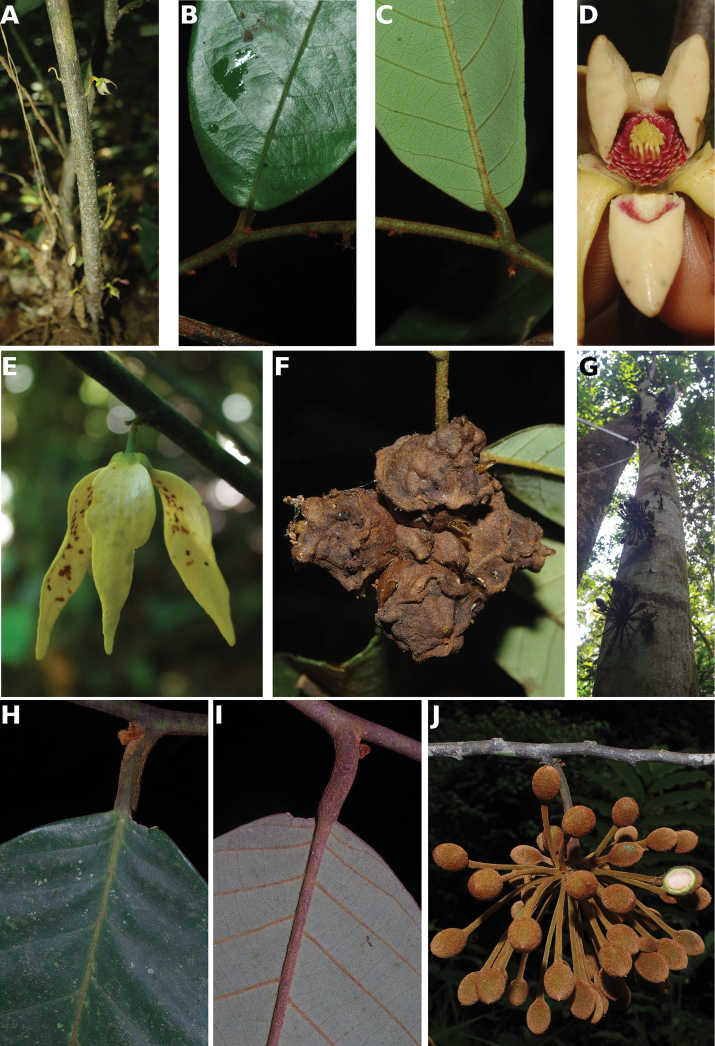
*Neostenantheraneurosericea***A** trunk with flowers **B** base of leaf blade, upper side **C** base of leaf blade, lower side **D** flower, one inner petal folded showing the red staens and yellow carpels **E** flower, side view **F** fruits, note sessile and pyramidal monocarps. *Neostenantherarobsonii***G** trunk with fruits **H** base of leaf blade, upper side **I** base of leaf blade, lower side **J** fruit with numerous monocarps, one monocarp longitudinally sectionned revealing single seed **A–F***Couvreur 603*, Gabon **G***Couvreur 678*, Campo Ma’an, Cameroon **H–J***Couvreur 1077*, Gabon. Photos Thomas L.P. Couvreur.

Figs 82 , 83 ; Map 10F

≡ Stenanthera neurosericea Diels, Bot. Jahrb. Syst. 39. 483, 1907.
= Neostenanthera platypetala (Engl. & Diels) Pellegr., Bull. Soc. Bot. France Mém. 1949: 56. 1950; ≡ ﻿﻿Stenantheraplatypetala Engl. & Diels, Bot. Jahrb. Syst. 39. 482, 1907. ≡ ﻿﻿﻿Boutiqueaplatypetala (Engl. & Diels) Le Thomas. Adansonia sér. 2, 5: 532, 1965. Syn. nov. Type. Cameroon. South Region; Bipindi, Zenker G.A. 2877, Mar 1904: lectotype, sheet here designated: B[B 10 0154069]; isotypes: B[B 10 0154071]; BM[BM000546878]; K[K000199036].
= Stenanthera macrantha Mildbr. & Diels, Bot. Jahrb. Syst. 1111. 445, 1915. Type. Cameroon. South Region, Kribi, *Mildbraed G.W.J. 5886*, Jul 1911: holotype: B[B 10 0154070]; isotype: HBG[HBG502540].

#### Type.

Cameroon. South Region; Bipindi, *Zenker G.A. 3105*, 1904: lectotype, sheet here designated: B[B 10 0154067]; isotypes: B[B 10 0390251]; BR[BR0000008824059]; GOET[GOET005682]; G[G00014887, G00014888]; L[L0038043]; LISC[LISC000393]; HBG[HBG502533]; MO[MO-216971]; M[M0107916]; P[P00363312]; S[S07-13457]; WAG[WAG0000096].

#### Description.

**Tree to shrub, up to 7 m tall, d.b.h. 3–6 cm**; stilt roots or buttresses absent. Indumentum of simple hairs; old leafless branches sparsely pubescent, **young foliate branches densely pubescent.** Leaves: petiole 4–5 mm long, ca. 1 mm in diameter, densely pubescent, grooved, blade inserted on top of the petiole; blade 15–21 cm long, 5–8 cm wide, ovate to elliptic, apex acuminate, acumen 1–2 cm long, base rounded, papyraceous, **below densely pubescent with erect slight curly hairs when young and old**, densely pubescent when old, above glabrous when young and old, concolorous or discolorous, whitish below; midrib sunken or flat, above glabrous when young and old, below densely pubescent when young, densely pubescent when old; secondary veins 15 to 21 pairs, glabrous below; tertiary venation percurrent. Individuals bisexual; inflorescences cauliflorous or ramiflorous on old leafless or young foliate branches, leaf opposed or extra axillary. Flowers with 9 perianth parts in 3 whorls, 1 to 2 per inflorescence; **peduncule ca. 2 mm long, unbranched**; pedicel 10–30 mm long, ca. 1 mm in diameter, densely pubescent; in fruit 20–30 mm long, 2–3 mm in diameter, densely pubescent; bract 1, basal, ca. 1 mm long, ca. 1 mm wide; sepals 3, valvate, free, 2–3 mm long, 1–2 mm wide, triangular, apex acute, base truncate, green, densely pubescent outside, glabrous inside, margins flat; petals free, outer petals longer than inner; **outer petals 3, 15–33 mm long, 8–15 mm wide**, **thick**, elliptic, apex acute, base narrowed and concave, light green to light yellow to cream, margins flat, pubescent outside, pubescent inside; inner petals 3, valvate, 10–15 mm long, 3–5 mm wide, triangular, apex acute, base broad and concave, light green to cream, margins flat, pubescent outside, glabrous inside; stamens 30 to 40, in 5 to 6 rows, 2–3 mm long, linear; connective tongue shaped, glabrous, red; staminodes absent; carpels free, 20 to 25, ovary ca. 2 mm long, stigma cylindrical, glabrous. **Monocarps sessile**, 7 to 15, 20–30 mm long, ca. 20 mm in diameter, ellipsoid, apex apiculate, **pyramidal in shape**, pubescent, wrinkled, brown when ripe; seed 1 per monocarp, 11–14 mm long, 10–11 mm in diameter, ellipsoid; aril absent.

#### Distribution.

From Cameroon to Gabon; in Cameroon known from the South, Central Littoral and South-West regions.

#### Habitat.

A common species when present; in primary or old secondary rain forests, non-inundated soils. Altitude 200–600 m a.s.l.

#### Local and common names known in Cameroon.

None recorded.

#### IUCN conservation status.

Least Concern (LC) (Cheek 2014a) (as ﻿﻿﻿*Boutiqueaplatypetala*).

#### Uses in Cameroon.

None reported.

#### Notes.

This species is closely related to ﻿*N.myristicifolia* but differs by having densely pubescent young foliate branches and sessile and pyramidal monocarps (versus clearly stipitate and ellipsoid monocarps).

We transfer ﻿﻿﻿*Boutiqueaplatypetala* (Engl. & Diels) Le Thomas back into the genus ﻿*Neostenanthera*. This species was initially described in the genus ﻿*Stenanthera* (Diels 1907), then transferred to ﻿*Neostenanthera* (Pellegrin 1949) until Le Thomas (1965b) erected the genus ﻿*Boutiquea* to accommodate it mainly because of its characteristic sessile monocarps (versus long-stipitate in ﻿*Neostenanthera* s.str.). Besides that, all other morphological characters are common with ﻿*Neostenanthera* (Fero et al. 2014), such as overall flower morphology (e.g. broad and concave inner petal bases), septate anthers, carpels with single ovules and consequently single seeded monocarps (rare in Cameroonian Annonaceae, but a character of the tree genus ﻿*Annickia* too). In the latest revision of the genus ﻿*Neostenanthera*, Fero et al. (2014) didn’t consider ﻿*Boutiquea* as congeneric with ﻿*Neostenanthera*. However, recent molecular data (Guo et al. 2017a; Couvreur et al. 2019) revealed that ﻿*B.platypetala* is phylogenetically very close to ﻿*Neostenanthera*, which is coherent with morphology. The occurrence of species with stipitate and sessile monocarps in the same genus is not uncommon in Annonaceae, for example in ﻿*Artabotrys* or ﻿*Uvaria*, and this character thus does not appear sufficient to warrant the separate generic status suggested by Le Thomas (1965b).

In addition, we also consider the name ﻿﻿*Neostenantheraplatypetala* as a synonym of ﻿﻿﻿*Neostenantheraneurosericea* the latter being the older name and thus takes precedence. Both names have already been tentatively suggested to be conspecific based on morphological similarities (Diels 1907; Fero et al. 2014). A close examination of the material of ﻿*N.neurosericea* (only known from the flowering type collection) and *N.platypetala* reveals no major differences, in particular the dense pubescence of young foliate branches, the shape of the leaves (rounded at base, clearly acuminate at apex and obovate in shape), the solitary flowers borne on the branches, and the shape and size of the petals are similar.

#### Specimens examined.

**Central Region**: Bank Nyong River near the new bridge ca 65 km SSW of Eséka, 3.53°N, 10.41°E, *16 July 1964*, *de Wilde W.J.J.O* 2840 (WAG); 16 km SSW d’Eséka, 3.65°N, 10.78°E, *02 January 1974*, *Letouzey R.* 12550 (P,WAG,YA); Route Vetère (Likouk-Lokoundji), 3.48°N, 10.32°E, *14 January 1974*, *Mezili P.* 251 (P,YA). **Littoral Region**: Ebo Bekob abandoned village CRES research station, 4.36°N, 10.41°E, *21 April 2005*, *Etuge M.* 6447 (K). **South Region**: Campo Ma an National Park 5 km after main entrance, 2.34°N, 10.25°E, *15 February 2012*, *Couvreur T.L.P.* 384 (WAG,YA); Campo Ma an National Park 5 km after main entrance, 2.35°N, 10.25°E, *15 February 2012*, *Couvreur T.L.P.* 386 (WAG,YA); Entre Fenda (60 km ESE de Kribi et riviere Kienke), 2.8°N, 10.4°E, *22 January 1962*, *Letouzey R.* 4092 (K,P,YA); Elom (Kribi), 2.78°N, 10.25°E, *25 April 1968*, *Letouzey R.* 9436 (P); 55 km ESE de Kribi 2 km W du village, 3.21°N, 10.06°E, *12 March 1963*, *Raynal A.* 10389 (P,YA); Assok (Nyabessan), 2.77°N, 10.47°E, *18 February 1965*, *Raynal A.* 13457 (P,YA); 44 km N de Nyabesssan Réserve forestière de Kienké-sud 500 m N de la route, 2.78°N, 10.37°E, *18 February 1965*, *Raynal A.* 13472 (P,YA); Campo-Ma’an area Bibambivoto, 2.24°N, 10.26°E, *23 August 2000*, *Tchouto Mbatchou G.P.* 2986 (KRIBI,WAG,YA); Campo-Ma’an area Mvini, 2.33°N, 10.20°E, *25 August 2002*, *Tchouto Mbatchou G.P.* CORIX_3 (WAG); Campo-Ma’an area Mvini, 2.27°N, 10.18°E, *14 September 2000*, *Tchouto Mbatchou G.P.* 3035 (KRIBI,WAG); Campo-Ma’an area Bifa, 2.65°N, 10.28°E, *12 October 2001*, *Tchouto Mbatchou G.P.* 3298 (KRIBI,WAG); Korup National Park Primary rain forest, 5.03°N, 8.833°E, *01 March 1987*, *Thomas D.W.* 6891 (MO); Bipindi, 3.08°N, 10.42°E, *1898*, *Zenker G.A.* 1904 (K); Bipindi, 3.08°N, 10.42°E, *20 June 1904*, *Zenker G.A.* 2877 (**B** ; Bipindi, 3.08°N, 10.42°E, *01 January 1918*, *Zenker G.A.* 29 (P); Bipindi, 3.08°N, 10.42°E, *01 January 1904*, *Zenker G.A.* 3105 (L,P,WAG); Bipindi, 3.08°N, 10.42°E, *01 January 1908*, *Zenker G.A.* 3749 (L,P); Bipindi, 3.08°N, 10.42°E, *01 January 1909*, *Zenker G.A.* 3819 (E,L,M); Bipindi, 3.08°N, 10.41°E, *Zenker G.A.* 3897 (K); Bipindi, 3.08°N, 10.41°E, *01 December 1913*, *Zenker G.A.* 440 (P); Bipindi, 3.08°N, 10.41°E, *01 November 1913*, *Zenker G.A.* 440 (P,U,WAG); Bipindi, 3.08°N, 10.41°E, *01 January 1912*, *Zenker G.A.* 4402 (L). **South-West Region**: Ekundu Kundu, 5.12°N, 8.895°E, *27 April 1996*, *Cable S.* 2279 (K,YA); Mundemba, 5.05°N, 8.883°E, *05 March 1993*, *Gereau R.E.* 5185 (MO,WAG); Between Ikenge ad Esukutang ca 6 km W of Ikenge, 5.27°N, 9.1°E, *03 April 1988*, *Thomas D.W.* 7554 (MO,P,WAG,YA); Korup National Park, 5.33°N, 8.9°E, *22 May 1988*, *Thomas D.W.* 7809 (MO,P).

### ﻿﻿﻿﻿ Neostenanthera robsonii

Taxon classificationPlantaeMagnolialesAnnonaceae

Le Thomas, Fl. Gabon No. 16: 196, 1969

0E81C668-DE69-562A-AC90-34E1050FA42D

**Figure 84.**
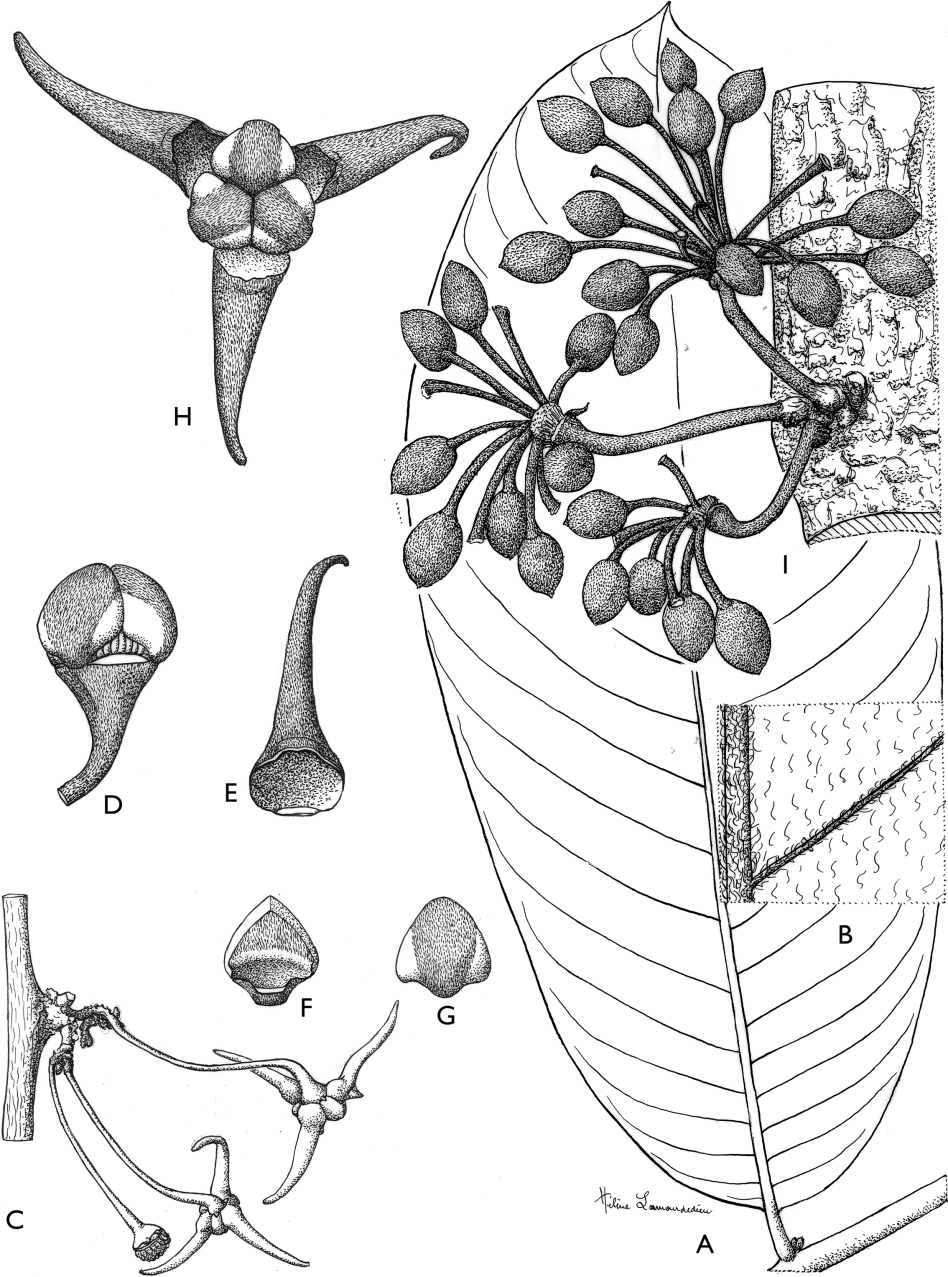
*Neostenantherarobsonii***A** leaf **B** detail of pubescence of leaf, lower side **C** infloresence **D** flower, outer petals removed **E** outer petal, inner view **G** inner petal inner and outer views **H** flower, top view **I** cauliflorous fruits **A, B, J** from *Hallé & Cours* 6094 **C–H** from *Le Testu 8635*. Drawings by Hélène Lamourdedieu, Publications Scientifiques du Muséum national d’Histoire naturelle, Paris; modified from Le Thomas (1969b, pl. 36, p. 195 and fig. 1, p. 197).

Figs 83 , 84 ; Map 10G

#### Type.

Gabon. Ogooué-Lolo; Lastoursville, *Le Testu G.M.P.C. 8635*, 28 Dec 1930: lectotype, sheet here designated: BM[BM000553861]; isotypes: BM[BM000553860, BM000553862]; BR[BR0000008802705, BR0000008802378]; P[P00363316, P00363315].

#### Description.

Tree, 5–25 m tall, d.b.h. 10–30 cm; stilt roots or buttresses absent. Indumentum of simple hairs; old leafless branches glabrous, **young foliate branches tomentose, brown**. Leaves: petiole 7–14 mm long, 1–3 mm in diameter, **tomentose brown**, slightly grooved to cylindrical, blade inserted on the side of the petiole; blade 7.5–31 cm long, 2.8–10.3 cm wide, oblong to elliptic, apex acuminate to acute, acumen 0.3–2.4 cm long, base rounded to obtuse, papyraceous, **below densely pubescent with erect slight curly hairs when young and old**, above glabrous when young and old, discolorous, whitish below; midrib impressed, above pubescent when young, glabrous when old, below pubescent when young and old; secondary veins 15 to 24 pairs, pubescent below; tertiary venation percurrent. Individuals bisexual; inflorescences cauliflorous or ramiflorous on old leafless branches, leaf opposed or extra axillary. Flowers with 9 perianth parts in 3 whorls, **2 to 8 per inflorescence**, **peduncle arbuscular-like, 8–26 mm long**; pedicel 22–46 mm long, ca. 1 mm in diameter, pubescent; in fruit 30–60 mm long, 3–4 mm in diameter, pubescent; bract 1, basal, ca. 1 mm long, ca. 1 mm wide; sepals 3, valvate, free, ca. 1 mm long, 1–2 mm wide, semiorbicular, apex acuminate, base truncate, brown, pubescent outside, pubescent inside, margins flat; petals free, outer petals longer than inner; outer petals 3, 16.2–23.3 mm long, 3–4.6 mm wide, narrowly triangular, very thick, apex attenuate, base suborbicular and concave, brown, margins flat, pubescent to glabrous outside, densely pubescent inside; inner petals 3, valvate, 5.6–6 mm long, 1.4–2.9 mm wide, triangular, apex acute, base broad and concave, margins flat, pubescent outside, glabrous inside; stamens 160 to 170, in 5 to 6 rows, 2 mm long, linear; connective tongue shaped, glabrous, brown; staminodes absent; carpels free, ca. 144, ovary ca. 1 mm long, stigma filiform, glabrous. **Monocarps stipitate**, stipes 10–50 mm long, 1–2 mm in diameter; monocarps 19 to 144, 9–14 mm long, 5–10 mm in diameter, ellipsoid, apex apiculate, **densely pubescent**, smooth, not ribbed, brown when ripe; seed 1 per monocarp, 7–12 mm long, 4–8 mm in diameter, ellipsoid; aril absent.

#### Distribution.

Southern Cameroon and Gabon; in Cameroon known from the South region.

#### Habitat.

A rare species in Cameroon; in primary or old secondary rain forests on non-inundated soils. Altitude 200–800 m a.s.l.

#### Local and common names known in Cameroon.

None recorded.

#### IUCN conservation status.

Least Concern (LC) (Cosiaux et al. 2019ae).

#### Uses in Cameroon.

None reported.

#### Notes.

﻿﻿﻿*Neostenantherarobsonii* is distinguished by the brown tomentose indumentum covering most of its parts, and the usually cauliflorous inflorescences with arbuscular-like peduncles. This species was first considered endemic to Gabon (Le Thomas 1969b), but has since been collected in Cameroon (e.g. *Lachenaud 659*, 2009).

#### Specimens examined.

**South Region**: Campo Ma’an National Park 11 km on trail from Ebinanemeyong village on road 7 km from Nyabessan to Campo town, 2.48°N, 10.33°E, *11 February 2015*, *Couvreur T.L.P.* 675 (WAG,YA); Campo Ma’an National Park 11 km on trail from Ebinanemeyong village on road 7 km from Nyabessan to Campo town, 2.48°N, 10.34°E, *11 February 2015*, *Couvreur T.L.P.* 678 (WAG,YA); Efoulan au sud d’Akom II (mi-chemin entre Ebolowa et Kribi), 2.76°N, 10.53°E, *09 May 2009*, *Lachenaud O.L.* 659 (BR,BR,WAG,YA).

### ﻿﻿ Piptostigma

Taxon classificationPlantaeMagnolialesAnnonaceae

Oliv., J. Linn. Soc., Bot. 8: 158, 1864

15D07D5A-02B2-57BF-BC3C-CAC5B39C11C4

= Phaeanthus Hook. f. & Thoms. sect. ﻿Piptostigma (Oliv.) Baillon Hist. Pl. 4: 287 1878.

#### Type species.

﻿﻿﻿*Piptostigmapilosum* Oliv.

#### Description.

Trees, 1–20 m tall, d.b.h. up to 21 cm; stilt roots or buttresses absent. Indumentum of simple hairs. Leaves: petiole 2–11 mm long, 1–6 mm in diameter; blade 7–66 cm long, 3–3 cm wide, ovate or elliptic or obovate or oblong, apex acuminate to emarginate, acumen 0.2–2.7 cm long, base cordate to decurrent, blade inserted on top of the petiole, **discolorous**, **whitish below**; midrib sunken or flat; secondary veins 14 to 65 pairs; **tertiary venation percurrent**. Inflorescence variable in length from compact to lax, composed of a peduncle like base and axial internodes. Individuals bisexual; inflorescences cauliflorous or ramiflorous on old leafless branches, axillary, 1 to 28 per inflorescence; pedicel 2–20 mm long; in fruit 9–25 mm long; bracts 2, one basal and one upper towards the lower half of pedicel; sepals 3, valvate, free, 2–12 mm long, apex acute or acuminate, base truncate; petals free, valvate, **outer petals shorter than inner**; outer petals 3, 5–28 mm long, 1.5–11 mm wide, elliptic or ovate or obovate, apex acute or acuminate, base truncate; inner petals 3, valvate, 15–80 mm long, 4–20 mm wide, elliptic or ovate, apex acute, base truncate; stamens numerous, 1–2 mm long, broad; connective discoid; staminodes absent; carpels free, 1 to 15, ovary 1–3 mm long, stigma globose or lobed. Monocarps sessile, 1 to 6, 13–65 mm long, 8–40 mm in diameter, globose to oblong, apex acute or rounded or cuspidate; seeds biseriate, 1–15 mm long, 2–10 mm in diameter, ellipsoid; aril absent.

A genus of 13 species from West and Central Africa (no species yet known from East Africa); all species are present in Cameroon, six endemic, making it the center of diversity for this genus.

﻿*Piptostigma* and ﻿*Brieya* are the only Annonaceae genera in Cameroon with longer inner petals than outer. ﻿*Piptostigma* has spectacular inflorescences that are unique among Cameroonian Annonaceae. They appear complex, but are in fact simple and, the different inflorescence types are an important character to identify species. First, inflorescences appear to be “stalked”, that is they have a short woody peduncle at the based on the inflorescence structure and is probably the result of fallen old inflorescences. This peduncle is variable in length. Then follows the indeterminate part or sympodial rachis, which contains axial internodes variable in length. Different lengths between the internodes will lead to different overall aspects of the inflorescence. Following Ghogue et al. (2017) and Maas et al. (2003) we can distinguish three types:

1. compact: internodes are shorter than 1 cm giving a very compact appearance to the inflorescence.
2. lax or sub-lax: internodes are between 1 and 4 cm long, leading to more or less long inflorescences. This is found in most species.
3. panicle-like: internodes between 5 and 10.5 cm long leading to long pendant inflorescences.

We shall refer to these terms in the descriptions below and the key. Finally, we end up with the pedicel and flower *per se*.

#### Taxonomy.

Ghogue et al. (2017).

### ﻿Key to the species of ﻿*Piptostigma* (mainly taken from Ghogue et al. as all species occur in Cameroon)

**Table:** 

1	Petioles, young foliate branches and leaf midribs densely tomentose golden-brown, secondary veins (30)40 to 65 pairs, rarely less than 40	**2**
–	Petioles, young foliate branches and leaf midrib not tomentose, but either glabrous, densely pubescent or hispid; secondary veins less than 40	**3**
2	Leaf base decurrent to cuneate, secondary veins 58 to 65; petiole 2–4 mm long; fruits verrucose, verrucate, very shortly pubescent; above 1000 m elevation.	﻿***P.submontanum***
–	Leaf base acute to obtuse or rarely broadly cordate, secondary veins 30 to 40; petiole 4–11 mm long; fruits smooth or puncticulate, brown tomentose; below 1,000 m elevation	﻿***P.calophyllum***
3	Leaf base rounded.	**4**
–	Leaf base acute, obtuse or cuneate	**6**
4	Leaf blade narrowly oblong to oblong, rarely narrowly obovate, shiny above; carpels 9–12.	﻿***P.macranthum***
–	Leaf blade obovate to narrowly obovate, narrowly elliptic to elliptic, matt above; carpels 3–8	**5**
5	Petiole and young foliate branches hispid, hairs up to 4 mm long; petiole 4–7 mm long; leaf blade 7–24 cm long, glabrous between the veins below	﻿***P.longepilosum***
–	Petiole and young foliate branches densely pubescent, hairs shorter than 4 mm, petiole 2–4 mm long; leaf blade 25–41 cm long, sparsely pubescent between the veins below	﻿***P.pilosum***
6	Upper side of the leaf blade pubescent at least towards the base and the midrib.	**7**
–	Upper side of the leaf blade glabrous even at the base and near the midrib	**8**
7	Leaf blade 11.5–20.5 cm long, petiole 3–4 mm long; young foliate branches hispid; inflorescence 30–270 cm long; carpels 3 to 4; monocarp broadly ovoid, tuberculate and pubescent.	***P.mortehanii***
–	Leaf blade 21–41 cm long, petiole 6–7 mm long; young foliate branches not hispid, but normally pubescent; inflorescence up to 8 cm long; carpels c. 5; monocarps (sub) globose, verrucose and glabrous	﻿***P.macrophyllum***
8	Leaf blade elliptic to narrowly elliptic.	**9**
–	Leaf blade obovate to very narrowly obovate	**10**
9	Petiole 6–8 mm long; leaf base obtuse; inflorescence composed of 2 to 5 rhipidia; sepals 1.8–3 mm long, carpels 4; monocarps shortly pubescent	.	﻿***P.mayndongtsaeanum***
–	Petiole 3–4 mm long; leaf base cuneate; inflorescence composed of 1(2) rhipidia; sepals 5–6 mm long; carpels 3; monocarps glabrous on old fruits (shortly pubescent on young ones)	﻿***P.oyemense***
10	Inflorescence composed of a single flower; carpel generally one, sometimes 3, more rarely 4	﻿***P.fugax***
–	Inflorescence composed of more than 2 multi-flowered rhipidia; carpels 4 or more	**11**
11	Monocarps ovoid or ellipsoid, obtuse at the base, smooth or puncticulate, finely pubescent to glabrous.	﻿***P.goslineanum***
–	Monocarps ellipsoid, aculeate, rarely verrucate, sparsely pubescent to glabrous. Cameroon, Gabon	﻿***P.multinervium***
–	Monocarps oblong, often transversally ribbed, bumpy and puncticulate (blister like), glabrous	﻿***P.glabrescens***

### ﻿﻿﻿﻿ Piptostigma calophyllum

Taxon classificationPlantaeMagnolialesAnnonaceae

Mildbr. & Diels., Bot. Jahrb. Syst. 53(3–5): 443, 1915

8CCA9F5A-03C0-5E72-8BEB-333A132518AA

**Figure 85.**
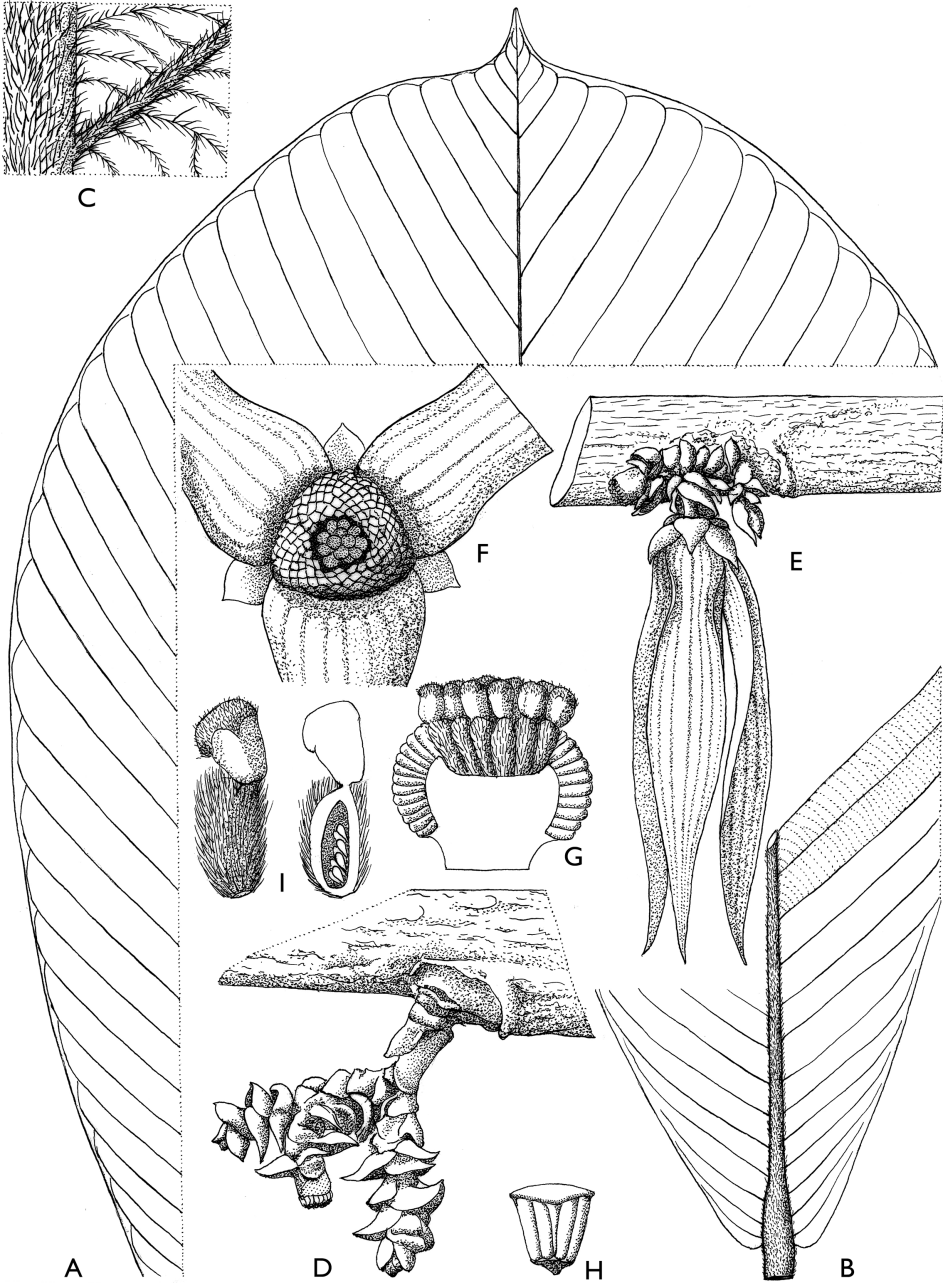
*Piptostigmacalophyllum***A** detail of leaf apex **B** detail of leaf base **C** detail of pubescence (lower portion of leaf) **D** detail of inflorescence with old flower (sepals and petals fallen), note compact nature of the inflorescence **E** flower, whole **F** flower, top view **G** longitudinal section of receptacle **H** stamen, front view **I** carpel side view and detail of ovules **A–I** from *Hallé, N.* 2263. Drawings by Hélène Lamourdedieu, Publications Scientifiques du Muséum national d’Histoire naturelle, Paris; modified from Le Thomas (1969b, pl. 36, p. 195 and fig. 19, p. 115).

**Figure 86.**
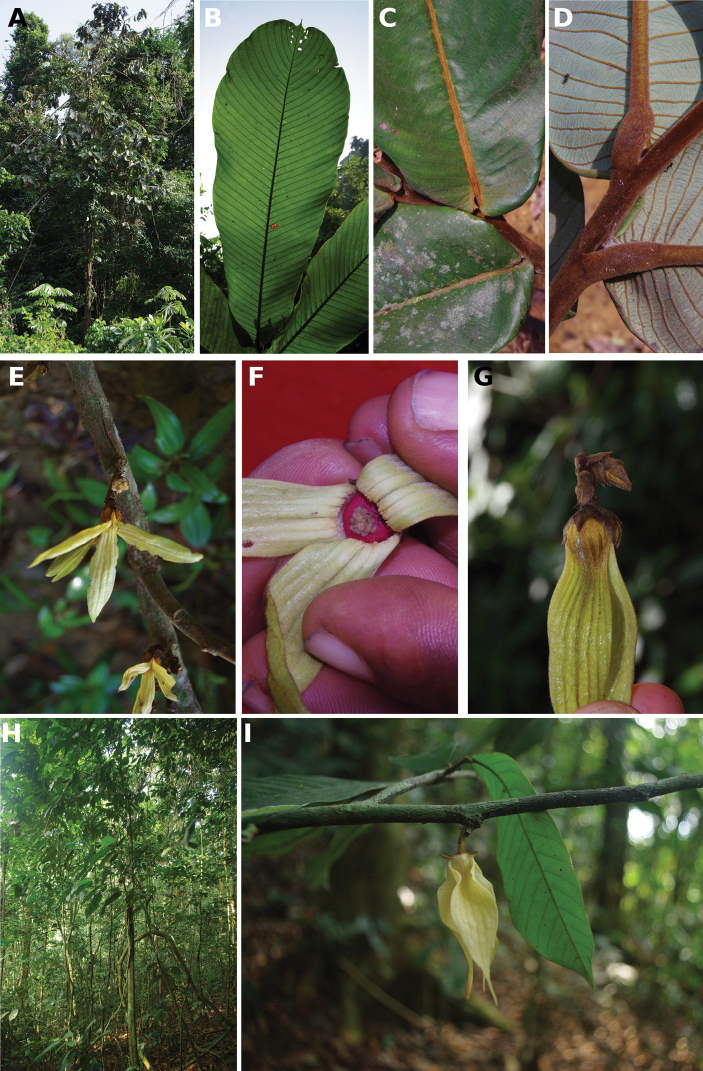
*Piptostigmacalophyllum***A** habit **B** base of leaf blade, lower side, note numerous secondary veins **C** base of leaf blade, upper side **D** base of leaf blade, lower side **E** flowering branch **F** detail of receptacle, note numerous carpels **G** detail of lower part of flower. *Piptostigmafugax***H** habit **I** flower, note single flowered inflorescence **A–G***Couvreur 1167b*, Mapubi, Cameroon **H, I***Couvreur 681*, Campo Ma’an, Cameroon. Photos Thomas L.P. Couvreur.

Figs 85 , 86 ; Map 10H

#### Type.

Cameroon. South Region; Ebolowa, *Mildbraed G.W.J. 5791*, 4 Jul 1911: holotype: B[B100154077]; isotypes: HBG[HBG502525, HBG502527, HBG50252]; L[L0196773]; P[P00363307, P00363307]; YA[YA0002833].

#### Description.

Tree, 8–10 m tall, d.b.h. 6–20 cm; stilt roots or buttresses absent. Indumentum of simple hairs; old leafless branches glabrous, **young foliate branches tomentose**. Leaves: petiole 4–11 mm long, 3–6 mm in diameter, **tomentose**, cylindrical, blade inserted on top of the petiole; **blade 30–66 cm long, 14–34 cm wide**, obovate, apex emarginate to mucronate, acumen 0.7–0.8 cm long, **base cordate to acute**, coriaceous, **below densely pubescent when young, densely pubescent when old**, above glabrous when young and old, discolorous, whitish below; midrib impressed, above pubescent when young, sparsely pubescent when old, below pubescent when young, glabrous when old; **secondary veins 30 to 40 pairs**, sparsely pubescent above; tertiary venation percurrent. Individuals bisexual; inflorescence cauliflorous, 2–2.5 cm long, peduncle like base 5–9 mm, axial internodes 2–4 mm long, **compact, sympodial rachis 10–15 mm long.** Flowers with 9 perianth parts in 3 whorls, 3 to 6 per inflorescence; pedicel ca. 6 mm long, ca. 3 mm in diameter, tomentose; in fruit 9–11 mm long, 3–4 mm in diameter, tomentose; bracts 2, one basal and one upper towards the lower half of pedicel, basal bract ca. 7 mm long, ca. 7 mm wide; upper bract ca. 7 mm long, ca. 7 mm wide; sepals 3, valvate, free, 4–5 mm long, 4 mm wide, ovate, apex acute, base truncate, brown, pubescent outside, glabrous inside, margins flat; petals free, outer petals shorter than inner; outer petals 3, 5–7 mm long, 3–4 mm wide, ovate, apex acuminate to obtuse, base truncate, light yellow, margins flat, pubescent outside, glabrous inside; inner petals 3, valvate, 50–60 mm long, 6–8 mm wide, ovate, apex acute, base truncate, pink to yellow, margins wavy, pubescent outside, pubescent inside; stamens 70 to 150, in 5 to 6 rows, 1 mm long, broad; connective discoid, glabrous, red; staminodes absent; carpels free, 8 to 10, ovary ca. 3 mm long, stigma globose, densely pubescent. Monocarps sessile, 2 to 5, 12–35 mm long, 10–28 mm in diameter, obovoid, apex cuspidate, **tomentose, smooth, not ribbed**, brown when ripe; seeds number not counted, ca. 15 mm long, ca. 10 mm in diameter, ellipsoid; aril absent.

#### Distribution.

From Cameroon to Gabon; in Cameroon known from the Central, South and Littoral regions.

#### Habitat.

An uncommon species; in lowland rain forests on non-inundated soils. Altitude 0–400 m a.s.l.

#### Local and common names known in Cameroon.

None recorded.

#### IUCN conservation status.

Vulnerable B2ab(iii) (Cosiaux et al. 2019af).

#### Uses in Cameroon.

None reported.

#### Notes.

﻿﻿﻿*Piptostigmacalophyllum* is characterized by its large obovate and coriaceous leaf blades (> 30 cm) with a broadly cordate base, tomentose petioles and young foliate branches, compact inflorescences, and tomentose smooth monocarps. It is a lowland species. It is morphologically close to ﻿*P.submontanum*, but the latter has a decurrent to cuneate leaf blade base, muricate and glabrous monocarps, and occurs in submontane areas (> 900 m a.s.l.).

#### Specimens examined.

**Central Region**: Edea (Mangombé), 3.8°N, 10.13°E, *01 January 1963*, *Letouzey R.* s.n. (P). **Littoral Region**: Mapubi 30 km before Edea on Yaoundé-Edea road On forestry road 5 km direction to Sanaga river, 3.84°N, 10.39°E, *28 February 2018*, *Couvreur T.L.P.* 1167b (K,MPU,P,WAG,YA); Bassin du Mungo, 4.17°N, 9.52°E, *01 June 1917*, *Fleury F.* 33400 (P); Edea Mangombe, 3.86°N, 10.14°E, *01 January 1956*, *Letouzey R.* 1936 (P,YA); Route Douala-Edéa près du km 28, 4.05°N, 9.689°E, *18 January 1962*, *Letouzey R.* 4011 (P,YA). **South Region**: Ngovayang, 3.23°N, 10.57°E, *01 June 2015*, *Kamdem N.* 295 (YA); Près Akak 10 km W Sangmeli 2.96°N, 11.88°E, *11 March 1970*, *Letouzey R.* 10157 (P,YA); Piste Meyo Ntem-Evouzok 75 km W Ambam Entre 1er et 3e bras du Ntem, 2.28°N, 10.52°E, *28 November 1979*, *Letouzey R.* 15272 (P,YA); Ebolowa Cameroon, 2.76°N, 10.91°E, *04 July 1911*, *Mildbraed G.W.J.* 5791 (B,L,P).

### ﻿﻿﻿﻿ Piptostigma fugax

Taxon classificationPlantaeMagnolialesAnnonaceae

A. Chev. ex Hutch. & Dalziel, Fl. W. trop. Afr. 1: 52, 1927

0EB7102F-49CC-5535-8965-D2E52FEF78A8

Fig. 86 ; Map 10I

#### Type.

Ivory Coast: Bas-Sassandra; Cavally basin, Tepos country: Grabo and surrounding villages, at the foot of Mount Copé, *Chevallier A.J.B. 19620*, 26–28 Jun 1907: lectotype, designated by Ghogue et al. (2017), p. 190: P[P00363296]; isolectotype: P[P00363297].

#### Description.

Tree to shrub, 1–10 m tall, d.b.h. 6–7 cm; stilt roots or buttresses absent. Indumentum of simple hairs; old leafless branches glabrous, young foliate branches densely pubescent. Leaves: petiole 2–4 mm long, 1–2 mm in diameter, densely pubescent, cylindrical, blade inserted on top of the petiole; blade 7–24 cm long, 3–6.5 cm wide, **obovate**, apex acuminate to acute, acumen 0.3–0.7 cm long, base obtuse to acute, papyraceous, below pubescent when young, glabrous when old, above glabrous when young and old, discolorous, whitish below; midrib impressed, above pubescent when young and old, below densely pubescent when young, densely pubescent when old; secondary veins 15 to 23 pairs, glabrous above; tertiary venation percurrent. Individuals bisexual; inflorescence cauliflorous, axillary, peduncle-like base not apparent, axial internodes absent, **compact in aspect, sympodial rachis 5–20 mm long.** Flowers with 9 perianth parts in 3 whorls; **1 per inflorescence**; pedicel 4–6 mm long, ca. 2 mm in diameter, densely pubescent; in fruit 11–13 mm long, ca. 3 mm in diameter, tomentose; basal bract ca. 3 mm long, ca. 1 mm wide; upper bract ca. 3 mm long, ca. 1 mm wide; sepals 3, valvate, free, ca. 5 mm long, ca. 2 mm wide, ovate, apex acute, base truncate, brown, pubescent outside, glabrous inside, margins flat; petals free, outer petals shorter than inner; outer petals 3, ca. 11 mm long, ca. 1.5 mm wide, ovate, apex acute, base truncate, light yellow, margins flat, pubescent outside, glabrous inside; inner petals 3, valvate, 20–40 mm long, 4–10 mm wide, ovate, apex acute, base truncate, margins wavy, pubescent outside, sparsely pubescent to glabrous inside; stamens 100 to 120, in 6 to 7 rows, 1 mm long, broad; connective discoid, glabrous, red; staminodes absent; **carpels free, 1 to 4**, ovary ca. 2 mm long, stigma globose, sparsely pubescent. **Monocarps sessile, 1 to 3, ca. 45 mm long, ca. 20 mm in diameter, ellipsoid to ovoid, apex cuspidate, pubescent, smooth, fleshy, white when ripe**; seeds 5 per monocarp, ca. 13 mm long, ca. 5 mm in diameter, ellipsoid; aril absent.

#### Distribution.

A mainly West African species from Liberia to Togo and Nigeria; in Cameroon known from the South and South-West regions.

#### Habitat.

In the understory of primary and secondary rain forests on granitic and clay soils. Altitude 100–700(–1300) m a.s.l.

#### Local and common names known in Cameroon.

None recorded.

#### IUCN conservation status.

Least Concern (LC) (Cosiaux et al. 2019ag).

#### Uses in Cameroon.

None reported.

#### Notes.

﻿﻿﻿*Piptostigmafugax* resembles ﻿*P.oyemense* in having a single-flowered inflorescence and flowers with 1 to 3(4) carpels, a unique combination of characters in ﻿*Piptostigma*. However, ﻿*P.fugax* has obovate leaf blades, while ﻿*P.oyemense* has elliptic leaf blades.

#### Specimens examined.

**South Region**: Campo Ma’an National Park 11 km on trail from Ebinanemeyong village on road 7 km from Nyabessan to Campo town, 2.52°N, 10.32°E, *11 February 2015*, *Couvreur T.L.P.* 681 (WAG,YA); Campo Ma’an National Park 11 km on trail from Ebinanemeyong village on road 7 km from Nyabessan to Campo town, 2.49°N, 10.34°E, *12 February 2015*, *Couvreur T.L.P.* 693 (WAG,YA). **South-West Region**: Ekundu Kundu to Erat ca 25 km, 5.13°N, 8.869°E, *26 April 1996*, *Cheek M.* 8198 (K,WAG,YA); Edensueh forest, 5.25°N, 9.576°E, *30 November 2000*, *Etuge M.* 4853 (K,YA).

### ﻿﻿﻿﻿ Piptostigma glabrescens

Taxon classificationPlantaeMagnolialesAnnonaceae

Oliv., J. Linn. Soc., Bot. 8: 159, 1864

39B44A13-50A8-597E-A1FC-7B5AC1151302

**Figure 87.**
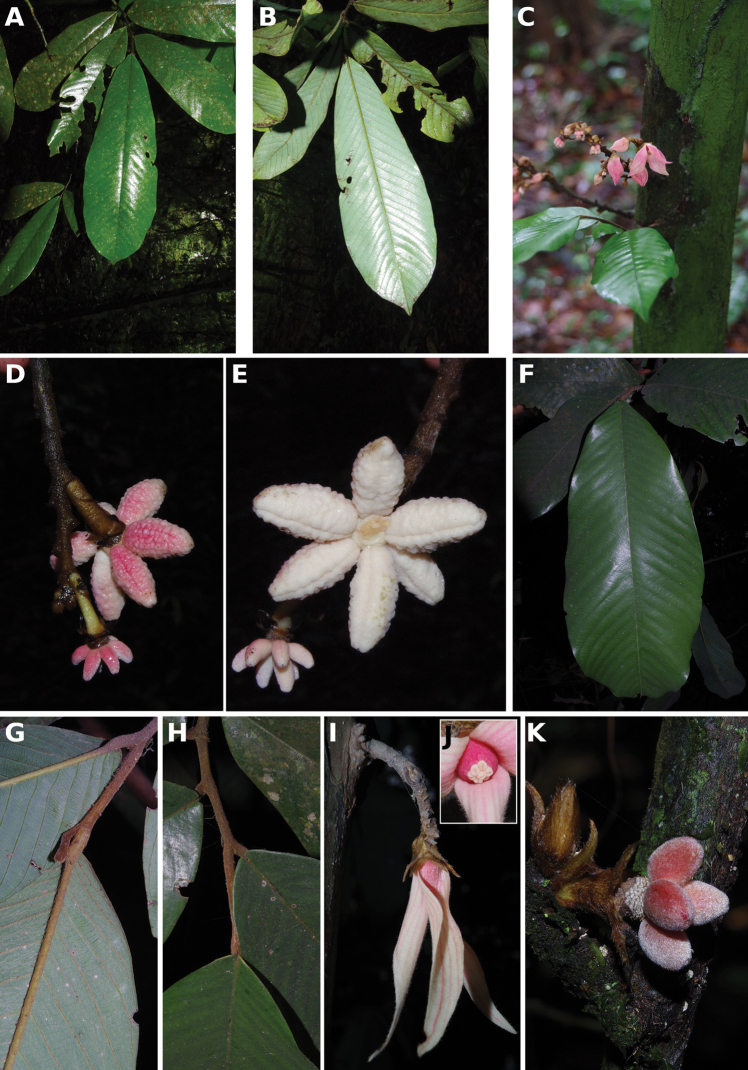
*Piptostigmaglabrescens***A** leaves, upper side **B** leaves, lower side **C** cauliflorous inflorescence **D** fruits, top view **E** fruits, lower view, note verrucose monocarps. *Piptostigmagoslineanum***F** leaf, upper side **G** base of leaf blade, lower side **H** base of leaf blade, upper side **I** inflorescence **J** detail of stamens and few (7) carpels **K** fruit, note hairy smooth monocarps **A–E***Couvreur 1158*, Kribi, Cameroon **F–I***Couvreur 983*, Rumpi Mts, Cameroo; **J***Couvreur 1017*, Mbayang Mbo, Cameroon. Photos Thomas L.P. Couvreur.

**Map 11.**
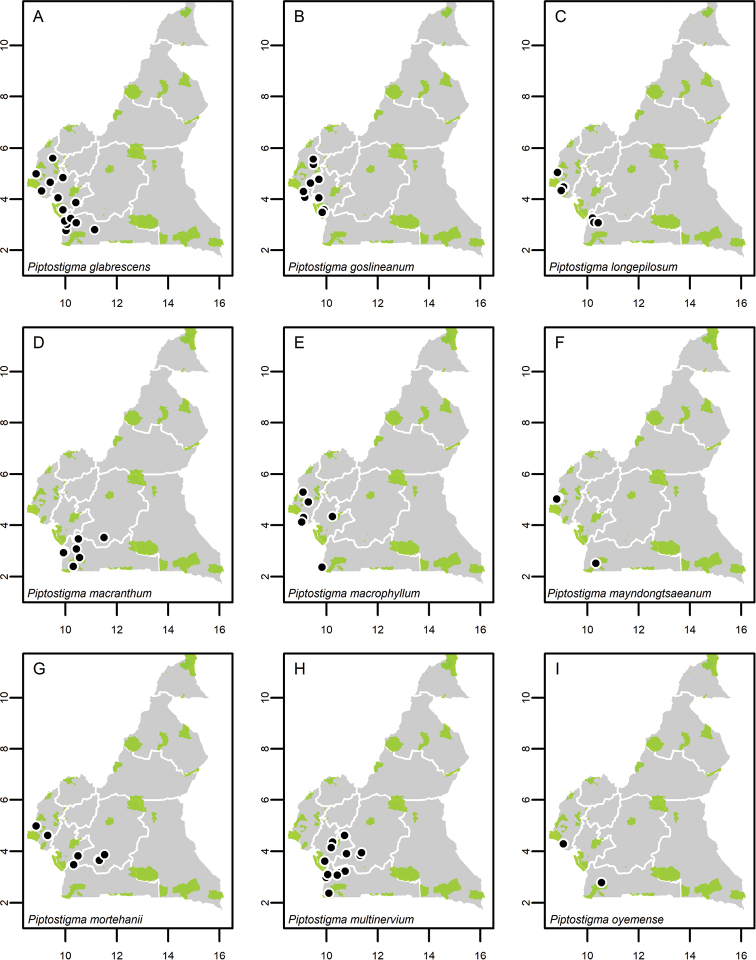
**A***Piptostigmaglabrescens***B***Piptostigmagoslineanum***C***Piptostigmalongepilosum***D***Piptostigmamacranthum***E***Piptostigmamacrophyllum***F***Piptostigmamayndongtsaeanum***G***Piptostigmamortehanii***H***Piptostigmamultinervium***I***Piptostigmaoyemense*. White borders represent region limits in Cameroon; green patches represent protected areas (see methods and Suppl. material 1: Fig. S1).

Figs 87 , 94 ; Map 11A

= Piptostigma preussii Engl. & Diels, Monogr. Afrik. Pflanzen.-Fam. 6: 54, 1901. Type. Cameroon. South-West Region, Barombi Station, *Preuss P.R. 251*, Apr 1890: holotype: B[B100154082]; isotype: K[K000105585].
= Piptostigma glabrescens Oliv. var. lanceolata Le Thomas, Fl. Gabon 16: 120, 1969. Type. Gabon. Ogooué-Ivindo, Bélinga, mines de fer, *Hallé N. 4087*, 12 Aug 1966: holotype: P[P00363300]; isotypes: P[P00323698, P00363299].

#### Type.

Equatorial Guinea. Rio Muni; Kongui River, *Mann G. 1792*, Aug.-Sep 1862: holotype: B[B100154078]; isotype: K[K000199000, K000199706]; P[P00363305].

#### Description.

Tree, 8–10 m tall, d.b.h. 12–15 cm; stilt roots or buttresses absent. Indumentum of simple hairs; old leafless branches glabrous, young foliate branches pubescent. Leaves: petiole 2–3 mm long, 1–2 mm in diameter, pubescent, cylindrical, blade inserted on top of the petiole; blade 12–30 cm long, 4–8.5 cm wide, obovate, apex acuminate, acumen 1.4–1.7 cm long, base obtuse to acute, papyraceous, below pubescent when young, glabrous when old, above glabrous when young and old, discolorous, whitish below; midrib impressed, above glabrous when young and old, below pubescent when young and old; secondary veins 19 to 27 pairs, glabrous above; tertiary venation percurrent. Individuals bisexual; inflorescences cauliflorous, peduncle like base 5–20 mm long, axial internodes 10–150 mm long, **lax to panicle-like, sympodial rachis 20–70 mm long.** Flowers with 9 perianth parts in 3 whorls, 10 to 28 per inflorescence; pedicel 11–13 mm long, 2 mm in diameter, pubescent; in fruit 16–20 mm long, 6 mm in diameter, densely pubescent; bracts 2, one basal and one upper towards the lower half of pedicel, basal bract 4–5 mm long, 3 mm wide; upper bract 2–5 mm long, 1–2 mm wide; sepals 3, valvate, free, 4 mm long, 2 mm wide, triangular, apex acute, base truncate, brown, pubescent outside, glabrous inside, margins flat; petals free, outer petals shorter than inner; outer petals 3, 5–6 mm long, 2 mm wide, ovate, apex acute, base truncate, red, margins flat, pubescent outside, glabrous inside; inner petals 3, valvate, 30–50 mm long, 5–10 mm wide, ovate, apex acute, base truncate, red, margins wavy, pubescent outside, sparsely pubescent inside; stamens 90 to 110, in 6 to 7 rows, ca. 1 mm long, broad; connective discoid, glabrous, red; staminodes absent; carpels free, 5 to 8, ovary ca. 1 mm long, stigma globose, pubescent. Monocarps sessile, ca. 8, 20–65 mm long, 9–21 mm in diameter, **oblong to narrowly oblong**, apex sometimes mucronate, **glabrous or sometimes very sparsely pubescent**, **puncticulate** (covered by small blister-like pimples or wards), **irregularly and longitudinally ribbed**, color unknown; seeds 1 to 7 per monocarp, ca. 15 mm long, ca. 10 mm in diameter, ellipsoid; aril absent.

#### Distribution.

From Cameroon to Gabon, one specimen from Republic of Congo; in Cameroon known from the South, Littoral and South-West regions.

#### Habitat.

A common species when present; in forests and plantations on black volcanic soils. Altitude 200–1000 m a.s.l.

#### Local and common names known in Cameroon.

nom otoungui (Dial. Boulou, *Endengle, E. s.n.*).

#### IUCN conservation status.

Near Threatened B2ab(iii) (Cosiaux et al. 2019ah).

#### Uses in Cameroon.

None reported.

#### Notes.

﻿﻿﻿*Piptostigmaglabrescens* is morphologically similar to ﻿*P.multinervium* (also a common species in Cameroon). ﻿﻿﻿*Piptostigmaglabrescens* however has glabrous older branches which are longitudinally striate (versus shortly or sparsely pubescent and non-striate), and monocarps which are puncticulate, and longitudinally ridged (versus muricate and not longitudinally ridged.

#### Specimens examined.

**Littoral Region**: Douala (route Razel), 4.05°N, 9.71°E, *01 January 1955*, *Endengle E.* 2061 (P); Forêt de Bakaka 3 km E of Eboné a village on km 11 Nkongsamba-Loum Road, 4.83°N, 9.9°E, *13 September 1971*, *Leeuwenberg A.J.M.* 8354 (WAG,YA); Hikoa Mahouda (chaine de la L’Hikoa Mandeng-30 km ENE Edea), 3.87°N, 10.39°E, *17 December 1973*, *Letouzey R.* 12415 (K); Tissongo, 3.58°N, 9.9°E, *07 July 1976*, *McKey D.B.* 103 (K). **South Region**: SE slopes of Mt elephant SE of Kribi, 2.78°N, 10.01°E, *26 February 1970*, *Bos J.J.* 6415 (BR,C,K,LD,P,WAG,YA); ca 3 km N of Lolodorf road, 3.01°N, 10.05°E, *18 March 1970*, *Bos J.J.* 6594 (BR,K,LD,LM,MO,P,WAG,YA); 15 km before Kribi on N7 road from Ed 3.14°N, 9.968°E, *27 February 2018*, *Couvreur T.L.P.* 1158 (MPU,P,WAG,YA); Nkoemvone, 2.81°N, 11.13°E, *09 September 1975*, *de Wilde J.J.F.E* 8453a (BR,K,MO,P,WAG); Près Bella (45 km NE de Kribi), 3.25°N, 10.2°E, *25 January 1962*, *Letouzey R.* 4167 (P,YA); Bipindi, 3.08°N, 10.41°E, *01 January 1901*, *Zenker G.A.* 2396 (B,BR,G,K,L,P,WAG); Bipindi, 3.08°N, 10.42°E, *01 January 1908*, *Zenker G.A.* 3654 (BR,L,P); Bipindi, 3.08°N, 10.41°E, *01 January 1911*, *Zenker G.A.* 4006 (K,P); Bipindi, 3.08°N, 10.41°E, *01 January 1912*, *Zenker G.A.* 4472 (BR,L,P); Bipindi, 3.08°N, 10.41°E, *01 January 1914*, *Zenker G.A.* 505 (B,G,P,WAG). **South-West Region**: mountain circular road S of Koto II and the Onge river, 4.32°N, 9.064°E, *21 October 1993*, *Cheek M.* 5071 (K,YA); Korup National Park nature trail near suspension bridge, 4.98°N, 8.85°E, *01 February 1995*, *Cheek M.* 7230 (K,YA); Pente septentrionale de Nta Ali (1266)m) descente de la cote 1009 à Mbio 30 km SE de Mamfe, 5.59°N, 9.503°E, *21 June 1975*, *Letouzey R.* 13912 (P,YA); Barombi F R Station, 4.65°N, 9.4°E, *1890*, *Preuss P.R.* 251 (K).

### ﻿﻿﻿﻿ Piptostigma goslineanum

Taxon classificationPlantaeMagnolialesAnnonaceae

Ghogue, Sonké & Couvreur, Pl. Ecol. Evol. 150 (2): 193, 2017

2005D255-1A5B-5A6F-81AD-0674FB1D8F56

Fig. 87 ; Map 11B

#### Type.

Cameroon. Littoral Region; around Douala, *Fleury F. 33134*, Jun 1917: holotype: P[P02032174]; isotypes: P[P02032172, P02032173, P02032175].

#### Description.

Tree, 8–30 m tall, d.b.h. 12–15 cm; stilt roots or buttresses absent. Indumentum of simple hairs; old leafless branches glabrous, young foliate branches pubescent. Leaves: petiole 2–4 mm long, 1–2 mm in diameter, pubescent, cylindrical, blade inserted on top of the petiole; blade 9.5–26 cm long, 3.5–10 cm wide, obovate, apex acuminate, acumen 0.5–1.8 cm long, base obtuse to acute, papyraceous, below sparsely pubescent when young and old, above glabrous when young and old, discolorous, whitish below; midrib impressed, above glabrous when young and old, below pubescent to tomentose when young, pubescent to tomentose when old; secondary veins 17 to 33 pairs, glabrous above; tertiary venation percurrent. Individuals bisexual; inflorescence cauliflorous, peduncle like base 15–65 mm long, axial internodes 5–35 mm long, compact to lax, **sympodial rachis 6–38 mm long.** Flowers with 9 perianth parts in 3 whorls, **(1) 2 to 7 per inflorescence**; pedicel ca. 7 mm long, ca. 2 mm in diameter, pubescent; in fruit 10–15 mm long, ca. 4 mm in diameter, tomentose; basal bract ca. 8 mm long, ca. 5 mm wide; upper bract ca. 4 mm long, ca. 2 mm wide; sepals 3, valvate, free, ca. 12 mm long, ca. 4 mm wide, ovate, apex acute, base truncate, brown, pubescent outside, glabrous inside, margins flat; petals free, outer petals shorter than inner; outer petals 3, ca. 15 mm long, ca. 4 mm wide, ovate, apex acute, base truncate, margins flat, tomentose outside, glabrous inside; inner petals 3, valvate, 38–45 mm long, 10–12 mm wide, ovate, apex acute, base truncate; stamens numerous, in 6 to 8 rows, ca. 1 mm long, broad; connective discoid, glabrous, red; staminodes absent; carpels free, 4 to 7, ovary ca. 2 mm long, stigma globose, pubescent. Monocarps sessile, 3 to 4, 20–35 mm long, 10–20 mm in diameter, **ellipsoid to ovoid**, apex rounded, **sparsely pubescent to glabrous, smooth, bumpy when dry, pink reddish when ripe**; seeds 4 to 5 per monocarp, ca. 10 mm long, ca. 6 mm in diameter, ellipsoid; aril absent.

#### Distribution.

endemic to Cameroon, known from the Littoral and South-West regions.

#### Habitat.

A fairly uncommon species; in the understory of submontane and lowland rain forests. Altitude 200–900 m a.s.l.

#### Local and common names known in Cameroon.

Niock (Dial. Yaoundé, *Fleury F. 33134*).

#### IUCN conservation status.

Vulnerable B1ab(iii)+2ab(iii) (Cosiaux et al. 2019ai).

#### Uses in Cameroon.

None reported.

#### Notes.

﻿﻿﻿*Piptostigmagoslineanum* closely resembles ﻿*P.glabrescens* by the shape and the size of their leaf blades, but the inflorescences of ﻿*P.goslineanum* are generally shorter than 12 cm long with 2–7 flowers, while those of ﻿*P.glabrescens* can reach up to 55 cm long with 10–28 flowers. In addition, the monocarps of ﻿*P.goslineanum* are smooth and ovoid to ellipsoid in shape, while those of ﻿*P.glabrescens* are puncticulate and oblong in shape.

In the check list of the plants of Mt. Kupe and Bakossi (Cheek et al. 2004), collections cited under ﻿*Piptostigma* sp. 1 belong to ﻿*P.goslineanum*.

#### Specimens examined.

**Littoral Region**: Aux environs de Douala, 4.05°N, 9.7°E, *01 June 1917*, *Fleury F.* 33134 (P); Tissongo, 3.58°N, 9.9°E, *08 July 1976*, *McKey D.B.* 105 (K); Douala-Edea Reserve Lombe Camp, 3.48°N, 9.833°E, *01 November 1977*, *Thomas D.W.* 510 (K); Lombe Camp Site Douala-Edea Reserve Cameroun, 3.48°N, 9.833°E, *30 May 1976*, *Waterman P.G.* 801 (K). **South-West Region**: Nta Ali SE Mamfe, 5.55°N, 9.521°E, *17 June 1987*, *Achoundong G.* 1267 (YA); Kupe village, 4.78°N, 9.683°E, *24 January 1995*, *Cable S.* 787 (K,WAG,YA); Mungo River F.R., 4.78°N, 9.607°E, *30 November 1999*, *Cheek M.* 10197 (K); Kupe village, 4.77°N, 9.701°E, *16 November 1995*, *Cheek M.* 7849 (K,YA); Kupe village, 4.79°N, 9.701°E, *19 May 1996*, *Cheek M.* 8328 (K,YA); Bayang Mbo Wildlife Sanctuary after Mbu river, 5.35°N, 9.501°E, *26 March 2016*, *Couvreur T.L.P.* 1017 (WAG,YA); Bayang Mbo Wildlife Sanctuary before Mbu river, 5.34°N, 9.487°E, *27 March 2016*, *Couvreur T.L.P.* 1023 (WAG,YA); on trail leading to top of Mt Etinde after Ekonjo village, 4.07°N, 9.152°E, *01 April 2016*, *Couvreur T.L.P.* 1030 (WAG,YA); Mount Cameroon National Park on the Bomona trail behind Bomona village 10 km NW from Idenau, 4.29°N, 9.100°E, *03 April 2016*, *Couvreur T.L.P.* 1047 (WAG,YA); on top of hill near Small Ekombe village 3 km after Kumba on road to Ekondo Titi town, 4.62°N, 9.378°E, *13 January 2016*, *Couvreur T.L.P.* 983 (WAG,YA); Ezeze road Nyasoso between shrike and Max’s trail following the river upwards, 4.82°N, 9.691°E, *25 June 1996*, *Etuge M.* 2420 (K,WAG,YA); Kupe village, 4.76°N, 9.699°E, *09 July 1996*, *Etuge M.* 2698 (K,WAG,YA); Massif Ntali pente NW 30 km SE Mamfé, 5.56°N, 9.482°E, *15 June 1982*, *Villiers J.-F.* 1448 (P,YA).

### ﻿﻿﻿﻿ Piptostigma longepilosum

Taxon classificationPlantaeMagnolialesAnnonaceae

Engl., Notizbl. Königl. Bot. Gart. Berlin 2: 297, 1899

79BFE081-08B2-51CD-8D33-302F77BF66FC

Fig. 88 ; Map 11C

#### Type.

Cameroon. South Region; around Bipinde, *Zenker G.A. 1075*, 1896: holotype: B[B100154079]; isotypes: HBG[HBG502530]; L[L0183458]; M[M0107925]; NY[NY00026195]; P[P00363294, P00363295]; S[S07-13478]; WU[WU0025868].

#### Description.

Tree, 4–16 m tall, d.b.h. unknown; stilt roots or buttresses absent. Indumentum of simple hairs; old leafless branches sparsely pubescent to glabrous, **young foliate branches densely hispid, hairs 4–5 mm long.** Leaves: petiole 4–7 mm long, 2–3 mm in diameter, **hispid, hairs 1–5 mm long**, cylindrical, blade inserted on top of the petiole; blade 7.7–24.3 cm long, 4–10.3 cm wide, obovate, apex acuminate, acumen 0.3–1 cm long, base rounded to obtuse, papyraceous, below glabrous when young and old, above glabrous when young and old, discolorous, whitish below; midrib impressed, above densely pubescent when young and old, below pubescent when young and old; secondary veins 14 to 34 pairs, glabrous above; tertiary venation percurrent. Individuals bisexual; inflorescence cauliflorous, pubescent all over, especially younger parts, with hairs 1–2 mm long, peduncle like base 5–20 mm long, axial internodes 14–40 mm long, lax to sub-lax, sympodial rachis 45–150 mm long. Flowers with 9 perianth parts in 3 whorls, 1 to 4 per inflorescence; pedicel 9–12 mm long, ca. 2 mm in diameter, densely pubescent; in fruit 10–12 mm long, 4 mm in diameter, pubescent; basal bract 6–12 mm long, 4–6 mm wide; upper bract 6–12 mm long, 4–6 mm wide; sepals 3, valvate, free, ca. 6 mm long, 4 mm wide, ovate, apex acute, base truncate, brown, pubescent outside, glabrous inside, margins flat; petals free, outer petals shorter than inner; outer petals 3, 6–10 mm long, 3–4 mm wide, elliptic, apex acute, base truncate, margins flat, pubescent outside, glabrous inside; inner petals 3, valvate, 40–80 mm long, 10–15 mm wide, elliptic, apex acute, base truncate, margins wavy, densely pubescent outside, pubescent towards base inside; stamens 70 to 90, in 5 to 6 rows, 1–2 mm long, broad; connective discoid, glabrous, red; staminodes absent; carpels free, 3 to 5, ovary ca. 2 mm long, stigma globose, pubescent. Monocarps sessile, 1 to 3, ca. 15 mm long, ca. 20 mm in diameter, **ellipsoid, apex rounded, pubescent, warty, bumpy, orange, pink to yellow when ripe**; seeds 10 to 12 per monocarp, ca. 10 mm long, ca. 8 mm in diameter, ellipsoid; aril absent.

**Figure 88.**
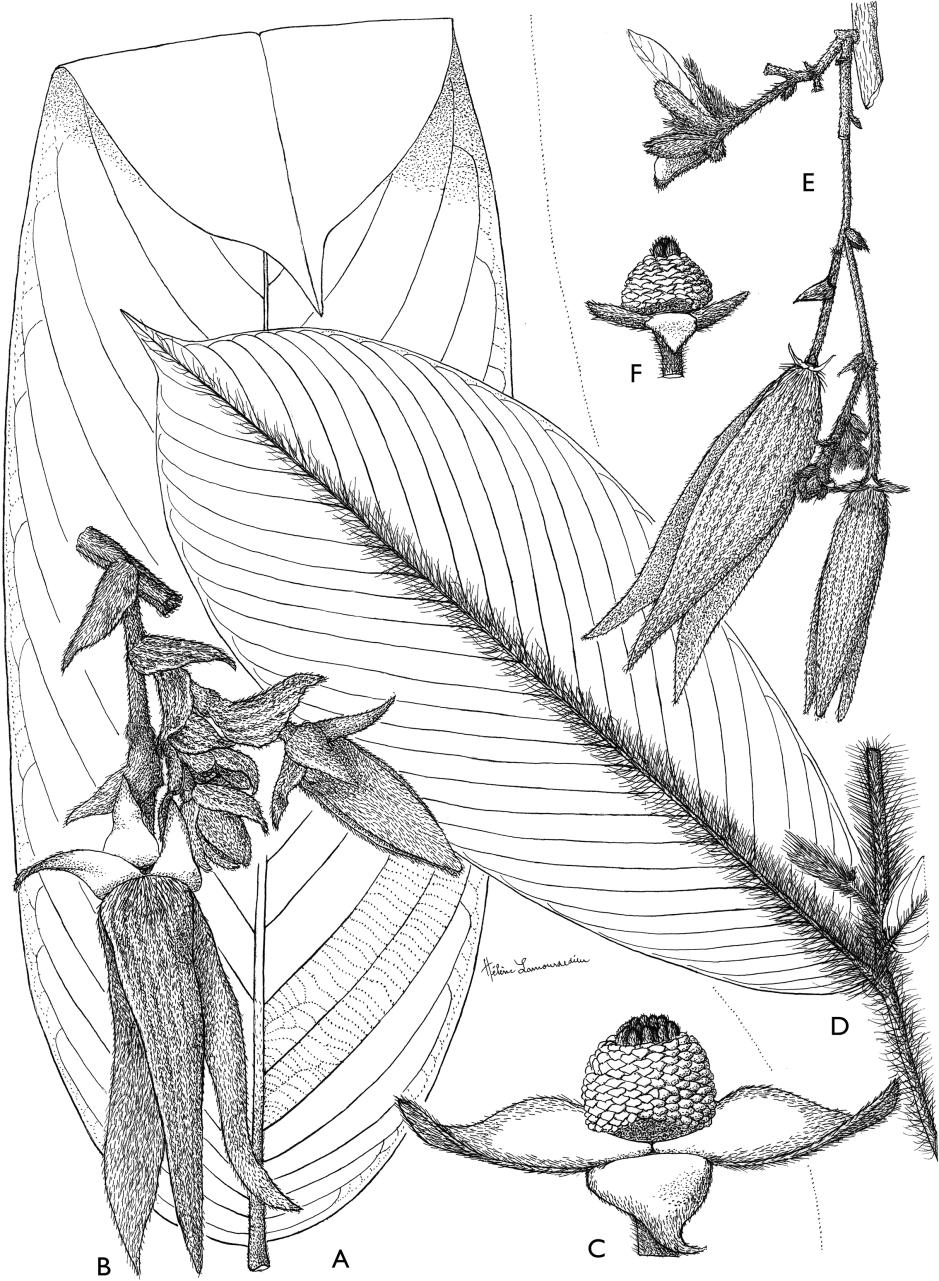
*Piptostigmamacranthum***A** leaf **B** inflorescence **C** receptacle, inner petals removed. *Piptostigmalongepilosum***D** leaf, note long pubescence **E** inflorescence **F** receptacle, outer petals removed **A–C** from *Letouzey 1937***D–F** from *Annet 420*. Drawings by Hélène Lamourdedieu, Publications Scientifiques du Muséum national d’Histoire naturelle, Paris.

#### Distribution.

endemic to Cameroon, known from the South and South-West regions.

#### Habitat.

A rare species; in the understory of lowland primary rain forests. Altitude 0–120 m a.s.l.

#### Local and common names known in Cameroon.

None recorded.

#### IUCN conservation status.

Endangered (EN) (Cosiaux et al. 2019aj).

#### Uses in Cameroon.

None reported.

#### Notes.

﻿﻿﻿*Piptostigmalongepilosum* is easily identified by the long (4–5 mm long) generally erect pubescence on the young foliate branches, petioles and young inflorescences, and rounded to obtuse leaf base. ﻿*Piptostigmamortehanii* also has a hispid pubescence, but its hairs are much shorter (less than 4 mm long) and the leaf blade base is acute. This species has not been recollected since 1994, despite efforts to locate it.

#### Specimens examined.

**South Region**: Route de Bipindi à Dihani, 3.26°N, 10.20°E, *24 June 1928*, *Annet E.* 420 (P); ca 45 k from Kribi ca 8 km N of Lolodorf road, 3.1°N, 10.25°E, *02 April 1970*, *Bos J.J.* 6683 (BR,K,LD,LM,MO,P,WAG,YA); Bipindi, 3.08°N, 10.42°E, *1896*, *Zenker G.A.* 1075 (L,P,WAG). **South-West Region**: Mokoko Forest Reserve Boa/Mokoko, 4.42°N, 8.972°E, *31 May 1994*, *Acworth J.M.* 289 (K,YA); Mokoko Forest Reserve Dikome, 4.48°N, 9.033°E, *02 May 1994*, *Ekema S.N.* 862 (K,YA); Aikoume(?) Banondo(?) forest (Cameroun), 4.45°N, 9.016°E, *03 May 1994*, *Sonké B.* 1173 (BR,WAG); Mokoko Mount above Bonja village, 4.46°N, 9.1°E, *23 March 1993*, *Tchouto Mbatchou G.P.* 613 (K); Korup National Park Primary rain forest, 5.03°N, 8.833°E, *01 March 1987*, *Thomas D.W.* 6906 (MO); Mokoko Forest Reserve Ekombe-Mofako, 4.46°N, 9.066°E, *21 May 1994*, *Watts J.* 1104 (K,YA); Likingi 35 hrs S on Wonge river E hills, 4.33°N, 8.98°E, *23 March 1993*, *Wheatley J.I.* 814 (K).

### ﻿﻿﻿﻿ Piptostigma macranthum

Taxon classificationPlantaeMagnolialesAnnonaceae

Mildbr. & Diels, Bot. Jahrb. Syst. 53(1–2): 142, 1915

F4F271AB-58F2-5745-A2F5-73AD0926851F

**Figure 89.**
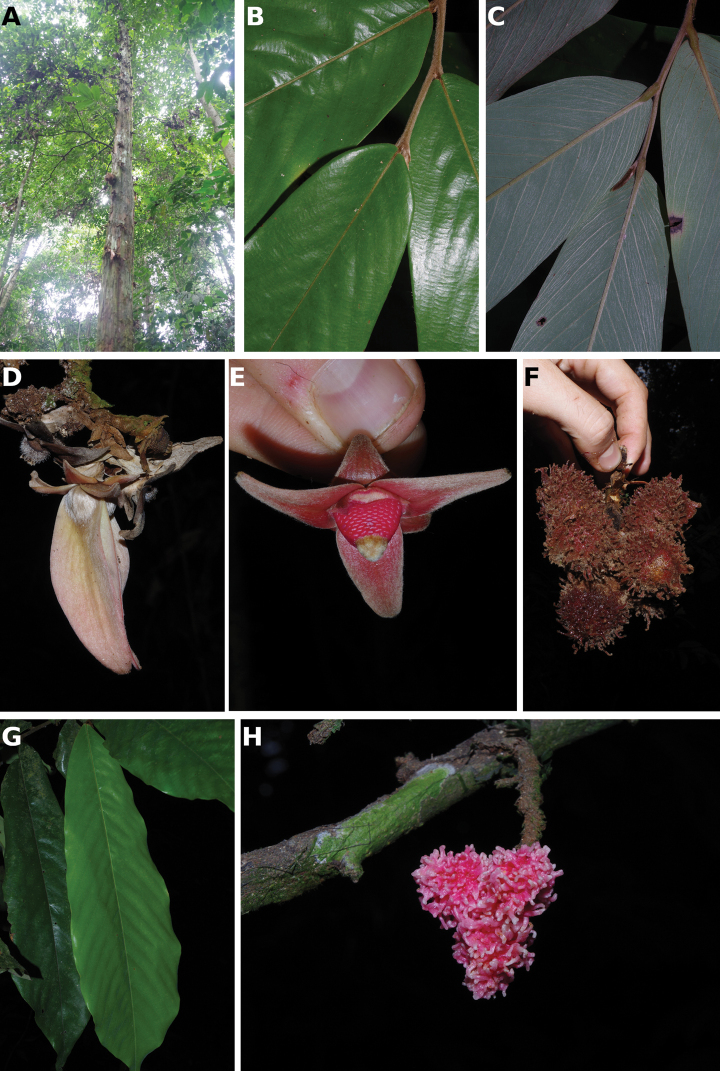
*Piptostigmamacranthum***A** habit with cauliflorous flowers **B** base of leaf blade, upper side **C** base of leaf blade, lower side **D** compact inflorescence with a single flower **E** detail of receptacle, outer petals removed **F** fruit, note brown muricate monocarps. *Piptostigmamacrophyllum***G** leaf, upper side **H** fruit; note smallish red-pink muricate monocarps **A, D–F***Couvreur 1135*, Gabon **B, C***Couvreur 1125*, Gabon **G, H***Couvreur 1034*, Mt Cameroon, Cameroon. Photos Thomas L.P. Couvreur.

Figs 88 , 89 ; Map 11D

= Piptostigma mayumbense Exell, J. Bot. 64 (Suppl. 1): 10, 1926. Type. Angola. Cabinda, M’bulu hills, Mayumbe, *Gossweiler J. 7807*, 15 Feb 1919: holotype: B[B100460898].

#### Type.

 Cameroon. South Region; Mimfia (Bipindi), *Zenker G.A. 2528*, 1902: holotype: B[B100154080]; isotypes:[BR0000013174743]; G[G00442261]; K[K000199001]; P[P00363281, P00363282, P00363283]; S[S07-13471].

#### Description.

Tree, 6–18 m tall, d.b.h. 15–21 cm; stilt roots or buttresses absent. Indumentum of simple hairs; old leafless branches glabrous, young foliate branches pubescent. Leaves: petiole 2–3 mm long, 2–3 mm in diameter, pubescent, cylindrical, blade inserted on top of the petiole; blade 14–31 cm long, 7–9 cm wide, **ovate to oblong**, apex acuminate, acumen 0.5 cm long, **base rounded to cordate**, subcoriaceous, below pubescent when young and old, above glabrous when young and old, discolorous, whitish below; midrib impressed, above pubescent when young and old, below densely pubescent when young, densely pubescent when old; secondary veins 16 to 30 pairs, glabrous above; tertiary venation percurrent. Individuals bisexual; inflorescence cauliflorous, pubescent all over, peduncle like base 6–25 mm long, axial internodes 10–20 mm long, **compact to sub-lax, sympodial rachis 10–70 mm long**. Flowers with 9 perianth parts in 3 whorls, 2 to 4 per inflorescence; pedicel 5–20 mm long, 3–5 mm in diameter, tomentose; in fruit 20–25 mm long, ca. 6 mm in diameter, pubescent; basal bract ca. 10 mm long, ca. 10 mm wide; upper bract ca. 10 mm long, ca. 10 mm wide; sepals 3, valvate, free, ca. 9 mm long, ca. 8 mm wide, triangular, apex acute, base truncate, brown, pubescent outside, glabrous inside, margins flat; petals free, outer petals shorter than inner; outer petals 3, 12–28 mm long, 9–11 mm wide, obovate, apex acute, base truncate, wine-brown, margins flat, pubescent outside, glabrous inside; inner petals 3, valvate, 40–55 mm long, 10–20 mm wide, elliptic, apex acute, base truncate, cream, margins wavy, pubescent outside, pubescent inside; stamens 280 to 320, in 10 to 11 rows, 1–2 mm long, broad; connective discoid, glabrous, red; staminodes absent; carpels free, 9 to 12, ovary ca. 2 mm long, stigma globose, pubescent. Monocarps sessile, 3 to 6, 30–40 mm long, 25–40 mm in diameter, **obovoid, apex acute, pubescent, muricate with projections up to 10 mm long, not ribbed, projections brown otherwise reddish when mature**; seeds 2 to 6 per monocarp, ca. 10 mm long, ca. 5 mm in diameter, ellipsoid; aril absent.

#### Distribution.

From Cameroon to the Republic of Congo; in Cameroon known from the South and Central regions.

#### Habitat.

A rare species in Cameroon; in forest edges or in closed forests along rivers. Altitude 50–500 m a.s.l.

#### Local and common names known in Cameroon.

Nom-owé (Dial. Yaoundé, *Letouzey s.n.*).

#### IUCN conservation status.

Least Concern (LC) (Cosiaux et al. 2019ak).

#### Uses in Cameroon.

None reported.

#### Notes.

﻿﻿﻿*Piptostigmamacranthum* is easily distinguished from other species of ﻿*Piptostigma* by its characteristic narrowly oblong to oblong leaf blade shape, shiny upper side of leaf blade, rounded and cordate leaf blade base and thick compact inflorescences.

#### Specimens examined.

**Central Region**: Ca 50 km S of Badjob ca 60 km SW of Eséka, 3.46°N, 10.5°E, *19 March 1964*, *de Wilde W.J.J.O* 2132 (BR,P,WAG); Akomimbang (Mbalmayo), 3.52°N, 11.5°E, *13 November 1957*, *Letouzey R.* 1937 (P); Akomimbang Mbalmayo, 3.52°N, 11.5°E, *13 November 1957*, *Letouzey R.* s.n. (P,YA). **South Region**: 10 km From Kribi Lolodorf road, 2.98°N, 9.966°E, *27 May 1969*, *Bos J.J.* 4647 (BR,K,MO,P,U,WAG,YA); Bezirk Kribi Vorland mit einzeln Hügeln bei Adjab 25 km Grand batanga near Eduma and Bidue, 2.93°N, 9.92°E, *25 July 1911*, *Mildbraed G.W.J.* 6118 (HBG); 51 SE de Campo Nkolmenbegue, 2.39°N, 10.3°E, *24 November 1992*, *Satabié B.* 1037 (YA); Efoulan, 2.74°N, 10.54°E, *04 December 2000*, *Tchouto Mbatchou G.P.* 3100 (KRIBI,WAG,YA); Bipindi, 3.08°N, 10.42°E, *01 January 1903*, *Zenker G.A.* 2528 (B,BR,G,L,P,WAG); Bipindi, 3.08°N, 10.42°E, *01 May 1902*, *Zenker G.A.* s.n. (P).

### ﻿﻿﻿﻿ Piptostigma macrophyllum

Taxon classificationPlantaeMagnolialesAnnonaceae

Ghogue, Sonké & Couvreur, Pl. Ecol. Evol. 150 (2): 199, 2017

B399F234-4E02-56A7-964B-36F138B4C638

Fig. 89 ; Map 11E

#### Type.

Cameroon. South-West Region; above small Koto village (Mt. Cameroon), *Thomas D.W. 4493*, 6 Mar 1985: holotype: YA[YA0002852]; isotypes: MO[MO3282523]; P[P00284016].

#### Description.

Tree, 5–10 m tall, d.b.h. 10–20 cm; stilt roots or buttresses absent. Indumentum of simple hairs; old leafless branches glabrous, young foliate branches pubescent. Leaves: petiole 6–7 mm long, 3–4 mm in diameter, pubescent, cylindrical, blade inserted on top of the petiole; blade 25–41 cm long, 9–16 cm wide, **obovate**, apex acuminate, acumen ca. 2.5 cm long, base acute, papyraceous, below glabrous when young and old, above sparsely pubescent when young, sparsely pubescent when old, discolorous, whitish below; midrib impressed, above glabrous when young and old, below sparsely pubescent when young and old; secondary veins 21 to 28 pairs, glabrous above; tertiary venation percurrent. Individuals bisexual; inflorescence cauliflorous or ramiflorous on old leafless branches, axillary, pubescent all over, peduncle like base 5–10 mm long, axial internodes 5–15 mm long, compact to sub-lax, sympodial rachis up to 70 mm long. Flowers with 9 perianth parts in 3 whorls, **3 to 6 per inflorescence**; **pedicel 8–10 mm long**, ca. 2 mm in diameter, tomentose; in fruit 10–12 mm long, ca. 4 mm in diameter, glabrous; basal bract ca. 12 mm long, ca. 3 mm wide; upper bract ca. 12 mm long, ca. 3 mm wide; sepals 3, valvate, free, ca. 9 mm long, ca. 3 mm wide, ovate, apex acute, base truncate, brown, pubescent outside, glabrous inside, margins flat; petals free, outer petals shorter than inner; outer petals 3, 9–15 mm long, 2.5–3 mm wide, narrowly elliptic, apex acute, base truncate, margins flat, pubescent outside, glabrous inside; inner petals 3, valvate, 25–35 mm long, 4–5 mm wide, ovate, apex acute, base truncate, margins wavy, pubescent outside, pubescent inside; stamens numerous, in 6 to 8 rows, 1–2 mm long, broad; connective discoid, glabrous, red; staminodes absent; carpels free, ca. 5, ovary ca. 3 mm long, stigma globose, densely pubescent. Monocarps sessile, 1 to 3, ca. 33 mm long, ca. 35 mm in diameter, globose, apex rounded, glabrous, **verrucose to muricate with short projections, not ribbed, light red pink when ripe**; seeds 2 to 4 per monocarp, 10–15 mm long, ca. 12 mm in diameter, ellipsoid; aril absent.

#### Distribution.

endemic to Cameroon, known from the South, Littoral and South-West regions.

#### Habitat.

A rare species; in primary or secondary lowland or premontane rain forests on black volcanic soils, sometimes in plantations. Altitude 550–1000 m a.s.l.

#### Local and common names known in Cameroon.

None recorded.

#### IUCN conservation status.

Vulnerable B2ab(iii,iv) (Cosiaux et al. 2019al).

#### Uses in Cameroon.

None reported.

#### Notes.

﻿﻿﻿*Piptostigmamacrophyllum* is morphologically close to ﻿*P.pilosum* by the large size and papery consistency of its leaf blade. However, the leaf blades of ﻿*P.pilosum* are mostly oblong or elliptic and only exceptionally narrowly obovate like those of ﻿*P.macrophyllum*. The inflorescences of ﻿*P.pilosum* are also less compact and less pubescent than those of ﻿*P.macrophyllum*.

The monocarps of ﻿*P.macrophyllum* strongly resemble those of ﻿*P.multinervium* and ﻿*P.macranthum* being muricate to verrucose in texture with short projections. ﻿﻿﻿*Piptostigmamacrophyllum* is distinguished from ﻿*P.multinervium* by its larger size of the leaf blades (25–41 cm long versus 13–21 cm long in ﻿*P.multinervium*), the lower side of the leaf blades being glabrous between the veins in ﻿*P.macrophyllum* and pubescent in ﻿*P.multinervium*; finally the sepals are narrowly ovate in ﻿*P.macrophyllum* while broadly triangular in ﻿*P.multinervium*. From ﻿*P.macranthum* it is distinguished by the leaves being obovate (versus narrowly oblong to oblong).

#### Specimens examined.

**Littoral Region**: Ebo Forest Reserve Djuma permanent camp on transect 5, 4.33°N, 10.23°E, *16 February 2013*, *Couvreur T.L.P.* 637 (WAG,YA). **South Region**: Bord de la Lobé 25 km E Campo, 2.37°N, 9.82°E, *01 January 1968*, *Letouzey R.* 9156 (P,YA). **South-West Region**: Mount Cameroon National Park Bakinguili trail above Bakinguili village, 4.09°N, 9.054°E, *02 April 2016*, *Couvreur T.L.P.* 1034 (WAG,YA); on trail through palm oil plantation 3 km before lava flow and Seme Beach hotel when coming from Limbe, 4.06°N, 9.079°E, *18 October 2013*, *Couvreur T.L.P.* 518 (WAG,YA); Entre DikomeBalue (1200 m) et Ifanga Nalende (650 m) 35 km NNW-Kumba, 4.9°N, 9.29°E, *25 March 1976*, *Letouzey R.* 14590 (P,YA); Etinde forest reserve Njonji lake, 4.13°N, 9.033°E, *25 January 1993*, *Tchouto Mbatchou G.P.* 1053 (K); Disturbed forest Bomana and Koto II, 4.3°N, 9.05°E, *26 April 1996*, *Tchouto Mbatchou G.P.* 1378 (K,YA); Mount Cameroon above small Koto village, 4.3°N, 9.1°E, *06 March 1985*, *Thomas D.W.* 4493 (YA); Between Ikenge and Esukutang ca 6 kms West of Ikenge, 5.28°N, 9.083°E, *03 April 1988*, *Thomas D.W.* 7645 (YA); Cameroon Mountain, 4.12°N, 9.029°E, *20 June 2001*, *van Andel T.R.* 3728 (U,WAG).

### ﻿﻿﻿﻿ Piptostigma mayndongtsaeanum

Taxon classificationPlantaeMagnolialesAnnonaceae

Ghogue, Sonké & Couvreur, Pl. Ecol. Evol. 150 (2): 201, 2017

6039D1EA-4E4B-55C5-BEE0-A81C6C6FCD3B

Map 11F

#### Type.

Cameroon. South-West Region; Korup National Park, *van der Burgt R. 689*, 25 Aug 2004: holotype: WAG[WAG0204511]; isotypes: BR[BR0000013174750]; P[P06901232].

#### Description.

Tree, up to 10 m tall, d.b.h. 17 cm; stilt roots or buttresses absent. Indumentum of simple hairs; old leafless branches glabrous, young foliate branches sparsely pubescent. Leaves: petiole 6–8 mm long, 2–3 mm in diameter, pubescent, cylindrical, blade inserted on top of the petiole; blade 23.5–36.5 cm long, 6.5–8.5 cm wide, **narrowly to very narrowly elliptic or narrowly obovate (rarely)**, apex obtuse, base obtuse, papyraceous, below sparsely pubescent when young and old, above glabrous when young and old, discolorous, whitish below; midrib impressed, above pubescent when young and old, below pubescent when young, glabrous when old; secondary veins 22 to 32 pairs, glabrous above; tertiary venation percurrent. Individuals bisexual; inflorescence cauliflorous or ramiflorous on old leafless branches, axillary, peduncle like base 15–30 mm long, axial internodes 1–50 mm long, **lax or panicle-like**, sympodial rachis 30–100 mm long. Flowers with 9 perianth parts in 3 whorls, 2 to 5 per inflorescence; pedicel 5–7 mm long, ca. 2 mm in diameter, densely pubescent; in fruit 15 mm long, 3 mm in diameter, pubescent; basal bract ca. 3 mm long, ca. 3 mm wide; upper bract ca. 2 mm long, ca. 2 mm wide; sepals 3, valvate, free, 2–3 mm long, 2–3 mm wide, ovate, apex acute, base truncate, brown, pubescent outside, glabrous inside, margins flat; petals free, outer petals shorter than inner; outer petals 3, 5–6 mm long, ca. 5 mm wide, obovate, apex acute, base truncate, pale yellow to cream, margins flat, pubescent outside, glabrous inside; inner petals 3, valvate, 30–40 mm long, 8–10 mm wide, elliptic, apex acute, base truncate, pale yellow to cream, margins wavy, densely pubescent outside, pubescent inside; stamens numerous, in 6 to 8 rows, 1–2 mm long, broad; connective discoid, glabrous, red; staminodes absent; **carpels free, 4**, ovary ca. 2 mm long, stigma globose, pubescent. Monocarps sessile, 1 to 3, 21–43 mm long, 27–53 mm in diameter, ellipsoid, apex rounded, **pubescent, verrucose with short flat projections, not ribbed, light red all over when ripe**; seeds up to 6 per monocarp, ca. 16 mm long, ca. 10 mm in diameter, **transversely ellipsoid**; aril absent.

#### Distribution.

endemic to Cameroon, known from the South and South-West regions.

#### Habitat.

A very rare species known from five collections; in primary rain forest on well-drained sandy soils, intermixed with crystalline rocks. Altitude c. 100 m a.s.l.

#### Local and common names known in Cameroon.

None recorded.

#### IUCN conservation status.

Least Concern (LC) (Cosiaux et al. 2019am).

#### Uses in Cameroon.

None reported.

#### Notes.

﻿﻿﻿*Piptostigmamayndongtsaeanum* differs from other species of the genus by the narrow shape of its leaf blade (from narrowly to very narrowly elliptic) and the shape of its monocarps (transversely ellipsoid).

#### Specimens examined.

**South Region**: Campo Ma’an National Park 11 km on trail from Ebinanemeyong village on road 7 km from Nyabessan to Campo town, 2.52°N, 10.32°E, *11 February 2015*, *Couvreur T.L.P.* 672 (P,WAG,YA). **South-West Region**: Korup National Park Ndian, 5.06°N, 8.866°E, *19 June 2000*, *Sainge M.* 558 (MO); Korup National Park, 5.01°N, 8.85°E, *10 March 1986*, *Thomas D.W.* 5840 (MO); Korup National Park, 5.01°N, 8.8°E, *25 August 2004*, *van der Burgt X.M.* 689 (BR,G,K,MO,P,WAG,YA); Korup National Park, 5.01°N, 8.8°E, *29 October 2005*, *van der Burgt X.M.* 790 (K,WAG,YA).

### ﻿﻿ Piptostigma mortehanii

Taxon classificationPlantaeMagnolialesAnnonaceae

De Wild., Bull. Jard. Bot. État Bruxelles 4: 383, 1914

C2CD602F-73BF-50DA-9DC0-37E97352C46C

**Figure 90.**
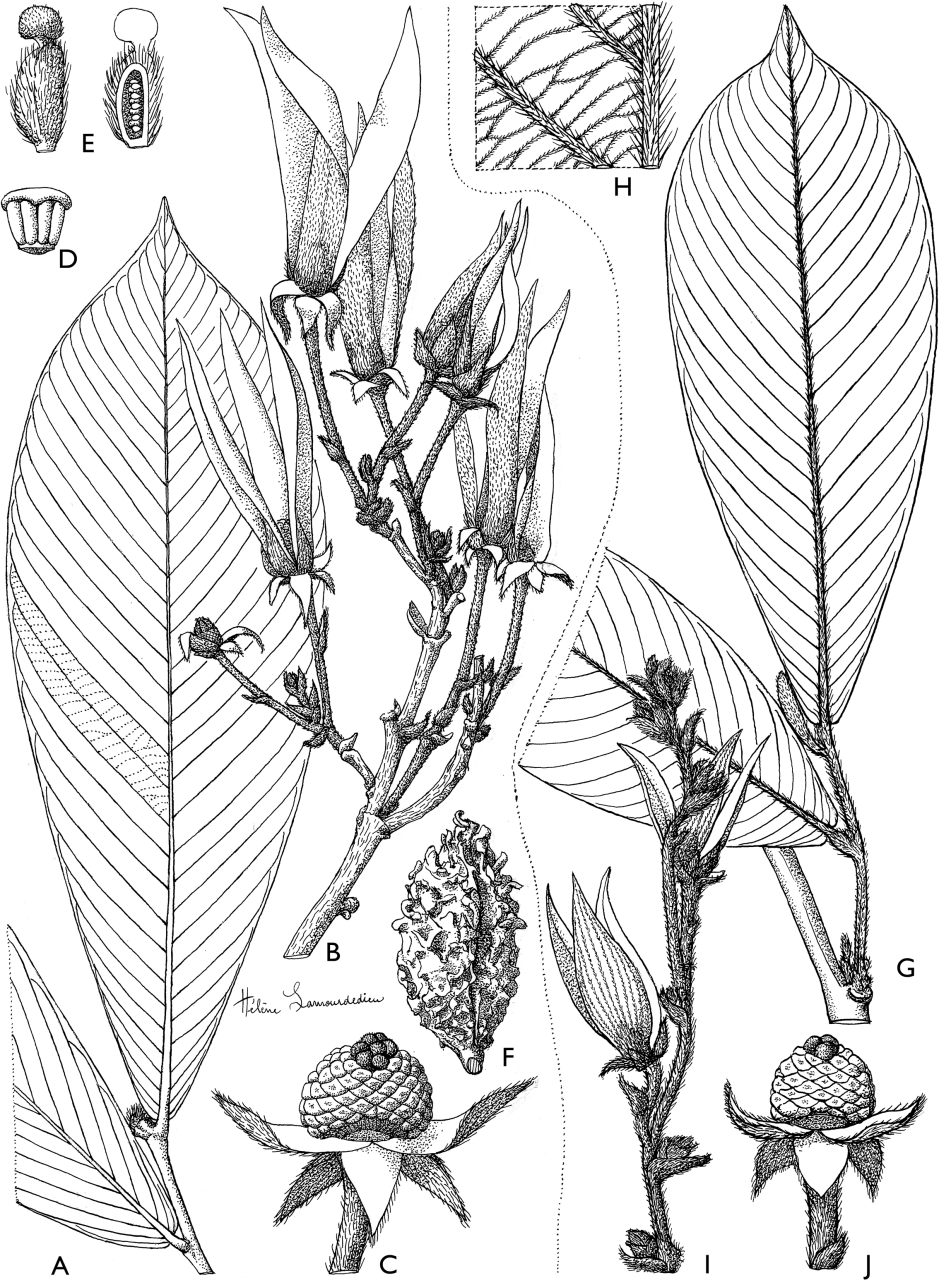
*Piptostigmamultinervium***A** leaf **B** inflorescence **C** receptacle, inner petals removed **D** stamen **E** carpel side view and detail of ovules **F** monocarp. *Piptostigmamortehanii***G** leaves **H** details of leaf, lower side **I** inflorescence **J** flower, inner petals removed **A–F** from *Lolo* 6 **G–J** from *Foury C. 73*. Drawings by Hélène Lamourdedieu, Publications Scientifiques du Muséum national d’Histoire naturelle, Paris; 1–6 modified from Le Thomas (1969b, pl. 21, p. 123).

Figs 90 , 92 ; Map 11G

= Piptostigma fouryi Pellegr., Notul. Syst. (Paris) 14: 75, 1950. Type. Cameroon. Central Region, Otottomo forest reserve, near Yaoundé, *Foury P. 73*, 1935: holotype: P[P00363302].
= ﻿Piptostigma mortehanii De Wild. var. pilosa Sillans, Rev. Int. Bot. Appl. Agric. Trop. 33: 554, 1953. Type. Central African Republic: Lobaye, Oubangui, Région de Boukoko, *Tisserant C. 2335*, 9 Jan 1952: holotype: P[P00363279]; isotypes: P[P00363278, P00363280].
= Piptostigma longipilosum Mildb. & Diels ex. Engl. var. subnudum Tisserant, Not. syst. 15: 327, 1953. Type. Central African Republic: Lobaye, Oubangui, Région de Boukoko, *Tisserant C. 2335*, 9 Jan 1952: holotype: P[P00363279]; isotypes: P[P00363278, P00363280].

#### Type.

Democratic Republic of the Congo. Equateur; Dundusana, *Mortehan M.G. 626*, Oct 1913: lectotype, sheet here designated: BR[BR0000008802118]; isotype: BR[BR0000008802798].

#### Description.

Tree, 8–10 m tall, d.b.h. 18 cm; stilt roots or buttresses absent, trunk funneled. Indumentum of simple hairs; old leafless branches glabrous, **young foliate branches pubescent to hirsute, hairs to 2 mm long**. Leaves: petiole 3–4 mm long, 2–3 mm in diameter, **hirsute**, cylindrical, blade inserted on top of the petiole; blade 11.5–20.1 cm long, 3–12.5 cm wide, obovate, apex acuminate, acumen 0.2–0.6 cm long, base acute, papyraceous, below sparsely pubescent when young and old, above pubescent when young, glabrous when old, discolorous, whitish below; midrib impressed, above pubescent when young and old, **below densely pubescent to tomentose when young and old**; secondary veins 24 to 34 pairs, sparsely pubescent above; tertiary venation percurrent. Individuals bisexual; inflorescence cauliflorous, peduncle like base 18–30 mm long, axial internodes 10–105 mm long, lax to panicle-like, **sympodial rachis 40–320 mm long**. Flowers with 9 perianth parts in 3 whorls, 2 to 8 per inflorescence; pedicel 5–8 mm long, ca. 2 mm in diameter, pubescent; in fruit 10–15 mm long, ca. 3 mm in diameter, pubescent; basal bract ca. 6 mm long, ca. 4 mm wide; upper bract ca. 6 mm long, ca. 4 mm wide; sepals 3, valvate, free, 4–5 mm long, 4–5 mm wide, ovate, apex acute, base truncate, brown, pubescent outside, glabrous inside, margins flat; petals free, outer petals shorter than inner; outer petals 3, 8–9 mm long, 4–5 mm wide, ovate, apex acute, base truncate, yellow, margins flat, pubescent outside, glabrous to pubescent, base glabrous inside; inner petals 3, valvate, 15–20 mm long, 4–7 mm wide, elliptic, apex acute, base truncate, greenish yellow, margins wavy, pubescent outside, tomentose inside; stamens 40, in 6 to 7 rows, ca. 2 mm long, broad; connective discoid, glabrous, red; staminodes absent; carpels free, 4 to 5, ovary ca. 2 mm long, stigma lobed, pubescent. Monocarps sessile, 1 to 2, 18–23 mm long, ca. 18 mm in diameter, ellipsoid, **apex mucronate or rounded, pubescent to tomentose**, **verrucose, irregularly ribbed, white pink turning wine red to dark sepia when ripe**; seeds 2 to 8 per monocarp, ca. 12 mm long, ca. 15 mm in diameter, ellipsoid; aril absent.

#### Distribution.

From Cameroon to the Democratic Republic of the Congo, one specimen from Ghana and two from Gabon; in Cameroon known from the South, Central, Littoral and South-West regions.

#### Habitat.

A common species when present; in lowland rain forest understory, often on inundated or swampy soils and along rivers. Altitude 30–700 m a.s.l.

#### Local and common names known in Cameroon.

None recorded.

#### IUCN conservation status.

Least Concern (LC) (Cosiaux et al. 2019an).

#### Uses in Cameroon.

None reported.

#### Notes.

﻿*Piptostigmamortehanii* is easily distinguished by its densely pubescent or tomentose midrib and secondary veins on the lower side of the leaf blade, which sometimes has a brownish aspect like ﻿*P.calophyllum*. In some specimens, the hairs on the petiole and young foliate branches are long and hispid (i.e. the type specimen of ﻿﻿*Piptostigmafouryi*), resembling ﻿*P.longepilosum*, but in the later species these hairs are significantly longer (1 mm vs. more than 4 mm). In addition, the leaf blade base of the latter is rounded while in *P.mortehanii* it is acute.

﻿*Piptostigmamortehanii* produces the longest inflorescences of the genus, which can be to up 2.7 m long with axial internodes up to 10.5 cm. However, this is quite variable within the species, and the inflorescence ranges from compact to panicle-like.

#### Specimens examined.

**Central Region**: Ottotomo Forest Reserve 3 km after reserve base near small loggers road, 3.66°N, 11.28°E, *02 May 2013*, *Couvreur T.L.P.* 436 (WAG,YA); Ottotomo Forest Reserve 45 km South of Yaoundé 5 km on path into reserve, 3.65°N, 11.32°E, *15 January 2015*, *Couvreur T.L.P.* 667 (WAG,YA); Yaoundé, 3.87°N, 11.52°E, *01 January 1935*, *Foury P.* 73 (P); Route vétère (Likouk-Likoundji), 3.48°N, 10.32°E, *14 January 1974*, *Mezili P.* 250 (P,WAG,YA). **Littoral Region**: Ca 40 km NW of Eséka on the other border of Kele River W of Yaoundé, 3.82°N, 10.48°E, *13 December 1963*, *de Wilde W.J.J.O* 1476 (B,BR,MO,P,WAG,YA). **South-West Region**: Boa, 4.62°N, 9.3°E, *03 May 1994*, *Ndam N.* 1205 (K); Korup National Park, 4.98°N, 8.85°E, *01 April 1979*, *Thomas D.W.* 1110 (K).

### ﻿﻿﻿﻿ Piptostigma multinervium

Taxon classificationPlantaeMagnolialesAnnonaceae

Engl. & Diels, Monogr. Afrik. Pflanzen.-Fam. 6: 55, 1901

A1F5F99F-5426-5391-81EC-A2956389A9DF

**Figure 91.**
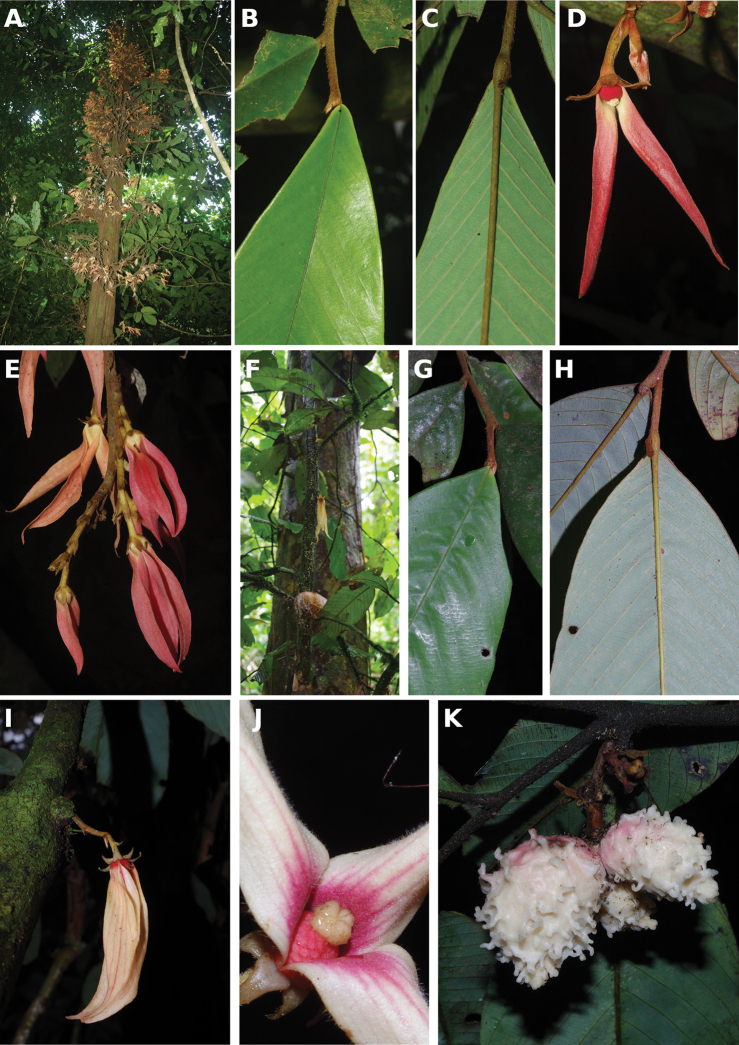
*Piptostigmamultinervium***A** habit, note numerous cauliflorous inflorescences along the trunk **B** base of leaf blade, upper side **C** base of leaf blade, lower side **D** flower, one inner petal removed **E** inflorescence with numerous flowers. *Piptostigmaoyemense***F** habit, note single flowered inflorescence **G** base of leaf blade, upper side **H** base of leaf blade, lower side **I** flower, note single flowered inflorescences **J** detail of receptacle, note 4 carpels **K** fruit, note white echinate to muricate monocarps **A–E***Couvreur 616*, Ebo, Cameroon **F, I–K***Couvreur 917*, Gabon **G, H***Couvreur 1139*, Gabon. Photos Thomas L.P. Couvreur.

Figs 90 , 91 ; Map 11H

#### Type.

Cameroon. South Region; Bipindi, *Zenker G.A. 2263*, Mar 1901: holotype: B[B100460901]; isotypes: BM[BM000553960]; G[G00442259]; GOET[GOET005683]; HBG[HBG502522]; K[K000199004, K000199005]; P[P02031265, P00363277]; WAG[WAG0065102]; WU[WU0038180].

#### Description.

Tree, 8–10 m tall, d.b.h. 10–15 cm; stilt roots or buttresses absent. Indumentum of simple hairs; old leafless branches sparsely pubescent, young foliate branches densely pubescent. Leaves: petiole 2–4 mm long, 2–3 mm in diameter, pubescent, cylindrical, blade inserted on top of the petiole; blade 13–21 cm long, 3–8 cm wide, **obovate**, apex acuminate, acumen 0.5–1 cm long, base cuneate to obtuse, papyraceous, below pubescent when young and old, above glabrous when young and old, concolorous or discolorous, whitish below; midrib impressed, above glabrous when young and old, below pubescent when young and old; secondary veins 15 to 31 pairs, glabrous above; tertiary venation percurrent. Individuals bisexual; inflorescence cauliflorous and ramiflorous on old leafless branches, axillary, peduncle like base 8–20 mm long, axial internodes 5–40 mm long, **lax or sublax**, sympodial rachis 15–70 mm long. Flowers with 9 perianth parts in 3 whorls, up to 6 per inflorescence; pedicel 9–12 mm long, ca. 2 mm in diameter, pubescent; in fruit ca. 20 mm long, ca. 5 mm in diameter, pubescent; basal bract 5–8 mm long, 4–5 mm wide; upper bract 5–7 mm long, 1–2 mm wide; sepals 3, valvate, free, ca. 5 mm long, ca. 4 mm wide, triangular, apex acute, base truncate, brown, pubescent outside, glabrous inside, margins flat; petals free, outer petals shorter than inner; outer petals 3, ca. 8 mm long, ca. 4 mm wide, ovate, apex acute, base truncate, light yellow to red, margins flat, pubescent outside, glabrous inside; inner petals 3, valvate, ca. 35 mm long, ca. 12 mm wide, ovate, apex acute, base truncate, pink, margins wavy, pubescent to pubescent towards base outside, pubescent inside; stamens 70 to 90, in 4 to 5 rows, 1–2 mm long, broad; connective discoid, glabrous, red; staminodes absent; carpels free, 4 to 6, ovary ca. 2 mm long, stigma globose, pubescent. Monocarps sessile, 1 to 4, ca. 40 mm long, ca. 28 mm in diameter, ellipsoid, apex rounded, **glabrous, verrucose to muricate, not ribbed, white when ripe**; seeds 7 to 8(9) per monocarp, ca. 17 mm long, ca. 11 mm in diameter, ellipsoid; aril absent.

#### Distribution.

Known from Cameroon and Gabon, and probably present in Equatorial Guinea; in Cameroon known from South, Central and Littoral regions.

#### Habitat.

A common species within its range; in the understory of old secondary or primary lowland rain forests, often on swampy soils. Altitude 200–900 m a.s.l.

#### Local and common names known in Cameroon.

None recorded.

#### IUCN conservation status.

Least Concern (LC) (Cosiaux et al. 2019ao).

#### Uses in Cameroon.

None reported.

#### Notes.

﻿﻿﻿*Piptostigmamultinervium* is morphologically close to ﻿*P.glabrescens* by the shape and the size of the leaf blade. By the external aspect of the monocarps, it is also close to ﻿*P.macrophyllum*. See notes of both species for more details on the differences.

#### Specimens examined.

**Central Region**: Nkolmylon 20 km route Yaoundé-Douala, 3.84°N, 11.31°E, *30 March 1984*, *Achoundong G.* 893 (YA); Colline Nkoldjobe dans le massif Mbaminkom, 3.95°N, 11.36°E, *15 March 1978*, *Dang D.* 681 (P,YA). **Littoral Region**: Ebo Forest Reserve Djuma permanent camp on Djashaka trail, 4.35°N, 10.23°E, *13 February 2014*, *Couvreur T.L.P.* 616 (WAG,YA); Mambe Massif above Boga village 100 km along road from Yaoundé to Ed 3.90°N, 10.77°E, *19 June 2014*, *Couvreur T.L.P.* 649 (WAG,YA); Ebo proposed national park Hospital trail 1430 m from Ndogbayembe trail, 4.14°N, 10.18°E, *23 March 2006*, *Mackinnon L.E.* 52 (YA); Near Lac Tissongo Mouanko Region, 3.62°N, 9.93°E, *21 February 1975*, *McKey D.B.* 47 (P,YA); Bekob, 4.61°N, 10.70°E, *19 February 2006*, *Tchiengue B.* 2555 (K). **South Region**: 13 km from Kribi S of Lolodorf road, 2.98°N, 9.983°E, *23 May 1969*, *Bos J.J.* 4625 (BR,MO,P,WAG,YA); 21 km from Kribi Lolodorf road, 3.1°N, 10.05°E, *16 June 1969*, *Bos J.J.* 4793 (BR,K,LD,LM,MO,P,POZG,WAG,YA); Kribi-Lolodorf, 3.17°N, 10.48°E, *28 July 1970*, *Bos J.J.* 7163 (BR,P,WAG); Mvini 35 km east of Campo, 2.37°N, 10.09°E, *27 February 1982*, *Hoshino J.* 359 (YA); Lolodorf, 3.23°N, 10.73°E, *18 April 1928*, *Lolo* 6 (P); Bipindi, 3.08°N, 10.41°E, *01 January 1914*, *Zenker G.A.* 2079 (WAG); Bipindi, 3.08°N, 10.41°E, *01 January 1918*, *Zenker G.A.* 21 (U); Bipindi, 3.08°N, 10.42°E, *01 January 1900*, *Zenker G.A.* 2263 (L,P,WAG); Bipindi, 3.08°N, 10.42°E, *01 March 1901*, *Zenker G.A.* s.n. (P).

**Figure 92.**
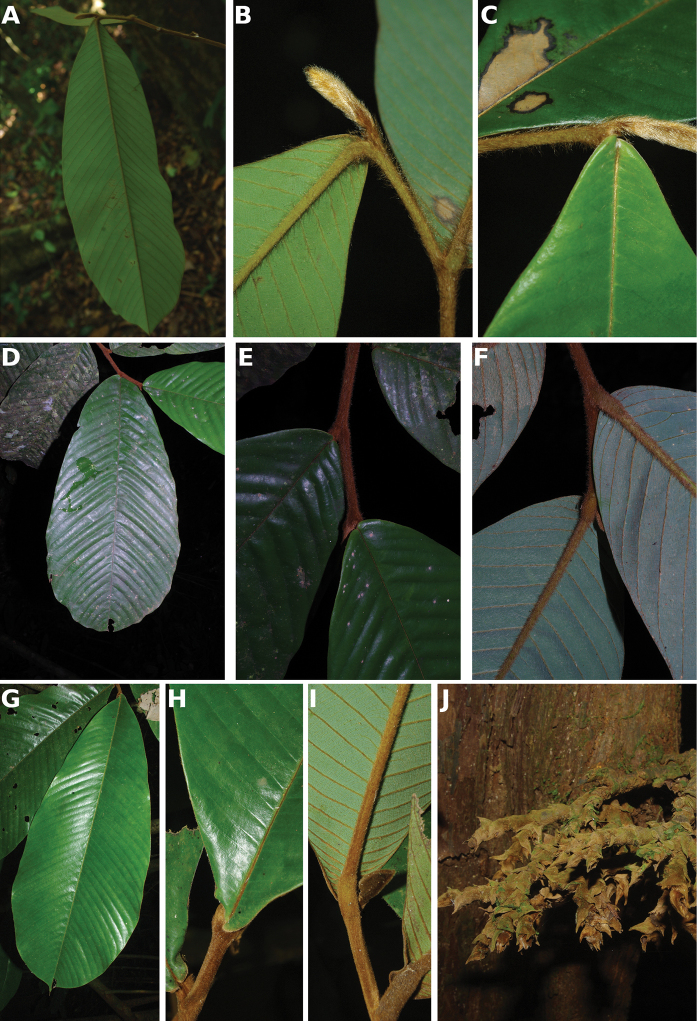
*Piptostigmamortehani***A** single monocarp **B** base of leaf blade, lower side **C** base of leaf blade, upper side. *Piptostigmapilosum***D** leaf, lower side, note numerous secondary veins **E** base of leaf blade, upper side **F** inflorescence with two flowers, side view. *Piptostigmasubmontanum***G** leaf, lower side, note numerous secondary veins **H** base of leaf blade, upper side **I** base of leaf blade, lower side **J** cauliflorous inflorescence, note compact nature **A***Bidault 3633*, Gabon **B, C***Couvreur 436*, Ebolbom, Cameroon **D, E***Couvreur 1070*, Gabon **F***Bidault 1414*, Gabon **G–J***Couvreur 625*, Ebo, Cameroon. Photos **B–E, G–J** Thomas L.P. Couvreur **A, F** Ehoarn Bidault. Tropicos.org, Missouri Botanical Garden.

### ﻿﻿﻿﻿ Piptostigma oyemense

Taxon classificationPlantaeMagnolialesAnnonaceae

Pellegr., Notul. Syst. (Paris) 14: 75, 1950

BF1D3E1E-3486-5D73-8C88-330BD8B5ABA6

**Figure 93.**
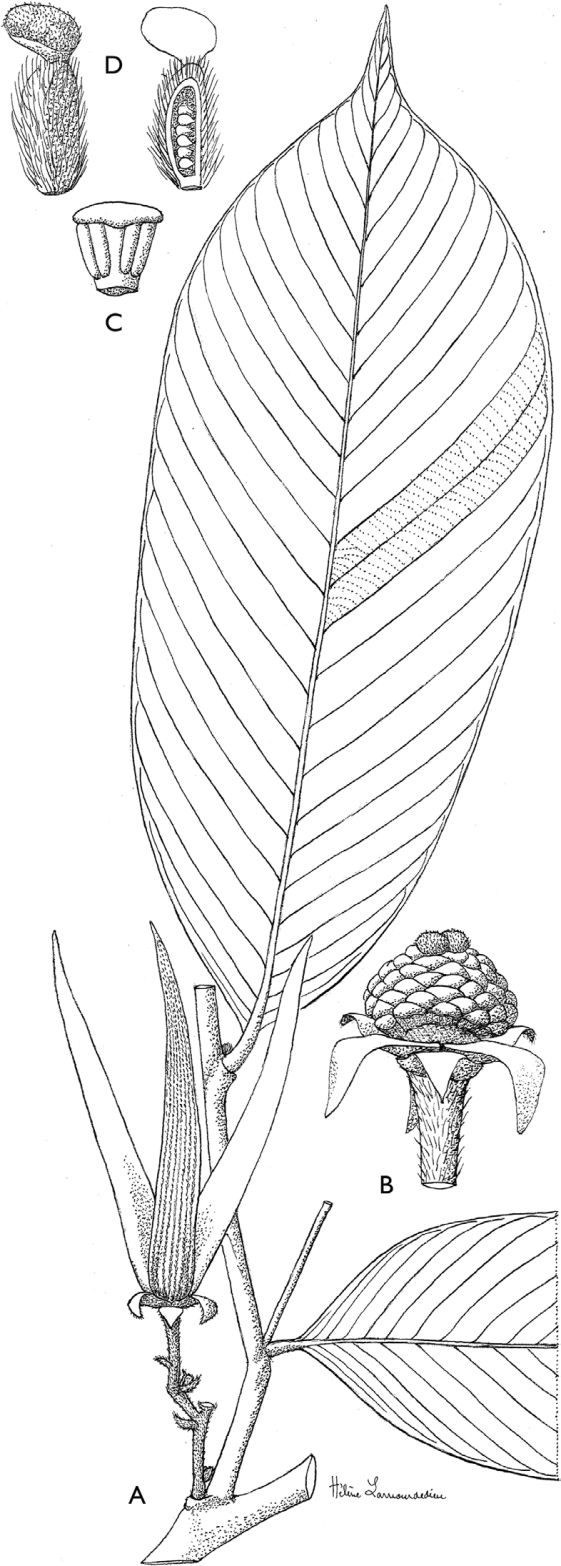
*Piptostigmaoyemense***A** branch (with a leaf and a flower) **B** receptacle **C** stamen **D** carpel side view and detail of ovules **A–D** from *Le Testu 9624*. Drawings by Hélène Lamourdedieu, Publications Scientifiques du Muséum national d’Histoire naturelle, Paris; 1–6 modified from Le Thomas (1969b, pl. 21 (drawings 7–10), p. 123).

Figs 91 , 93 ; Map 11I

#### Type.

Gabon. Woleu-Ntem; Oyem, *Le Testu G.M.P.C. 9624*, 21 Jun 1934: holotype: P[P00363276]; isotypes: BR[BR0000008802774, BR0000008802446]; BM[BM000553961]; P[P00363274, P00363275].

#### Description.

Tree, 2–6 m tall, d.b.h. up to 10 cm; stilt roots or buttresses absent. Indumentum of simple hairs; old leafless branches glabrous, young foliate branches pubescent. Leaves: petiole 3–4 mm long, 1–2 mm in diameter, pubescent, cylindrical, blade inserted on top of the petiole; blade 11–27 cm long, 4–9 cm wide, **elliptic**, apex acuminate, acumen 0.2–2.5 cm long, **base cuneate**, papyraceous, below sparsely pubescent when young, glabrous when old, above glabrous when young and old, discolorous, whitish below; midrib impressed, above glabrous when young and old, below pubescent when young, glabrous when old; secondary veins 21 to 29 pairs, glabrous above; tertiary venation percurrent. Individuals bisexual; inflorescence cauliflorous, peduncle like base 7–10 mm long, axial internodes 3–6 mm long, **compact**, sympodial rachis 27–42 mm long. Flowers with 9 perianth parts in 3 whorls, 1 to 2 per inflorescence; pedicel 11–18 mm long, ca. 1 mm in diameter, pubescent; in fruit ca. 12 mm long, ca. 2 mm in diameter, pubescent; basal bract ca. ca. 4 mm long, 2 mm wide; upper bract ca. 4 mm long, ca. 2 mm wide; sepals 3, valvate, free, 5–6 mm long, 3–4 mm wide, ovate, apex acuminate, base truncate, brown, pubescent outside, glabrous inside, margins flat; petals free, outer petals shorter than inner; outer petals 3, 12–14 mm long, 3–4 mm wide, obovate, apex acuminate, base truncate, light green, margins flat, pubescent outside, glabrous inside; inner petals 3, valvate, **40–50 mm long**, 5–10 mm wide, elliptic, apex acute, base truncate, pink, margins wavy, pubescent outside, pubescent inside; stamens numerous, in 6 to 8 rows, ca. 1 mm long, broad; connective discoid, glabrous, red; staminodes absent; **carpels free, 3**, ovary ca. 2 mm long, stigma globose, pubescent. Monocarps sessile, 1 to 3, 30–40 mm long, 25–30 mm in diameter, ellipsoid, apex rounded, **pubescent when immature, glabrous when mature**, verrucose, not ribbed, white with pink when ripe; seeds 3 to 5 per monocarp, 10–15 mm long, 5–10 mm in diameter, ellipsoid; aril absent.

#### Distribution.

Known from Cameroon to Gabon; in Cameroon known from South and South-West regions.

#### Habitat.

A rarely collected species in Cameroon; in rain forests on the slope of mountains. Altitude 450–900 m a.s.l.

#### Local and common names known in Cameroon.

None recorded.

#### IUCN conservation status.

Vulnerable B2ab(iii) (Cosiaux et al. 2019ap).

#### Uses in Cameroon.

None reported.

#### Notes.

﻿﻿﻿*Piptostigmaoyemense* is characterized by its elliptic leaf blades, glabrous on both sides when old, and its inflorescence usually with a single flower (rarely 2) and 3 carpels. The only other species with a single flower per inflorescence *and* up few carpels (less than 4) is ﻿*P.fugax*, which has obovate to very narrowly obovate leaf blades, a cuneate leaf base (versus obtuse to acute), a pubescent midrib on the upper ide (versus glabrous), the longer inner petals between 20–40 mm long (versus 40–50 mm) and the tomentose monocarps (versus glabrous when mature).

Cheek and Cable (1998, p. 11) mention two specimens as ﻿*Piptostigma* sp. nov. (*Akogo 34*, *Harris 3755*) collected from Mont Cameroon. We identified the former as ﻿*P.oyemense*. We did not see *Harris 3755*.

#### Specimens examined.

**South Region**: Akom II, 2.78°N, 10.55°E, *12 December 2013*, *Kamdem N.* 159 (YA). **South-West Region**: Bomana, 4.28°N, 9.060°E, *05 October 1993*, *Akogo M.* 34 (K,YA).

### ﻿﻿﻿﻿ Piptostigma pilosum

Taxon classificationPlantaeMagnolialesAnnonaceae

Oliv., J. Linn. Soc., Bot. 8: 159, 1964

1A695BC5-7ECF-5FDF-9F33-9812C1F0C56D

**Figure 94.**
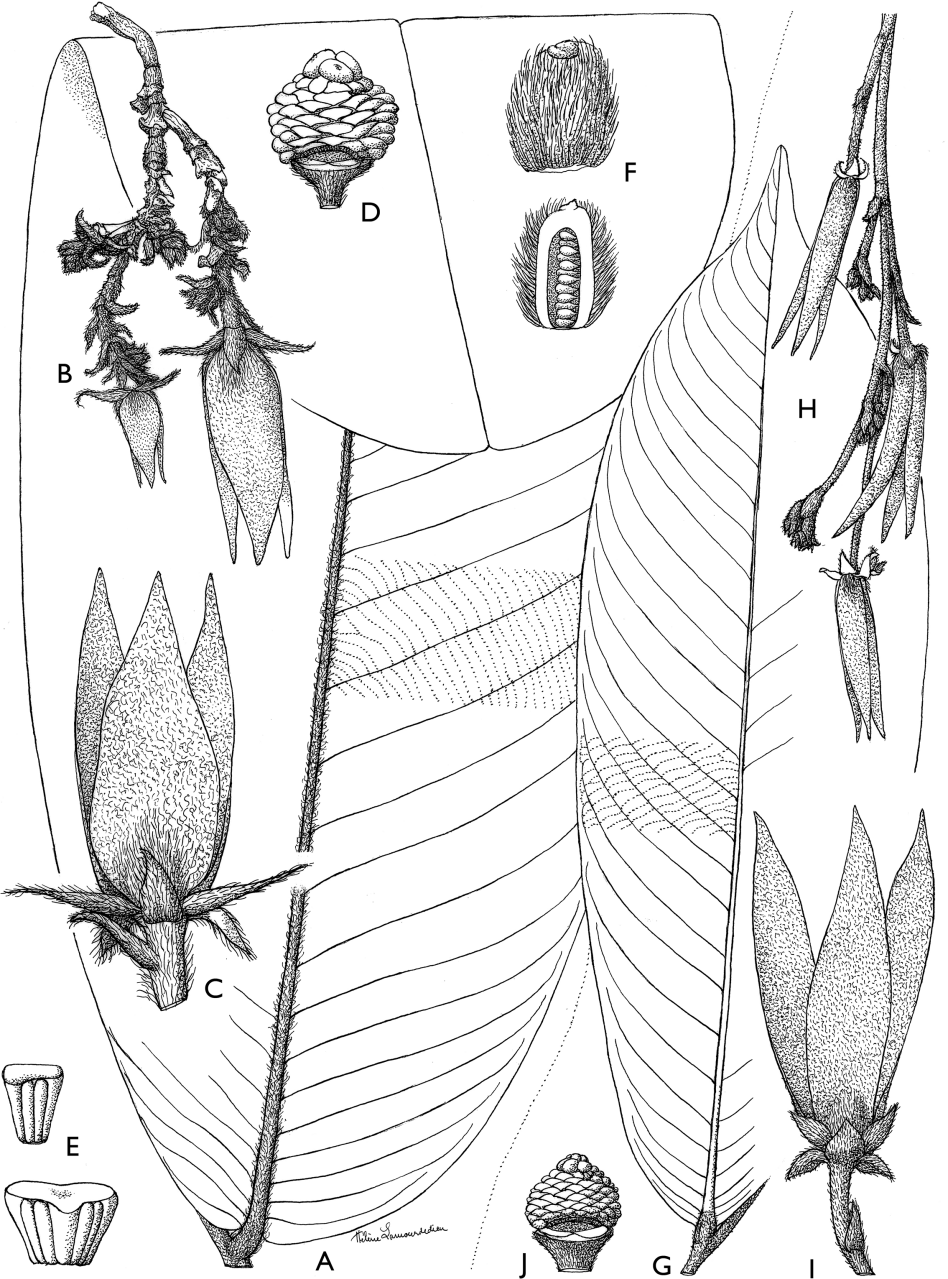
*Piptostigmapilosum***A** leaf **B** inflorescence **C** flower **D** receptacle **E** stamens **F** carpel side view and detail of ovules. *Piptostigmaglabrescens***G** leaf **H** inflorescence **I** flower, side view **J** receptacle, petals and sepals removed **A–F** from *Le Testu 8465***G–J** from *Letouzey 4167*. Drawings by Hélène Lamourdedieu, Publications Scientifiques du Muséum national d’Histoire naturelle, Paris; modified from Le Thomas (1969b, pl. 20, p. 119).

**Map 12.**
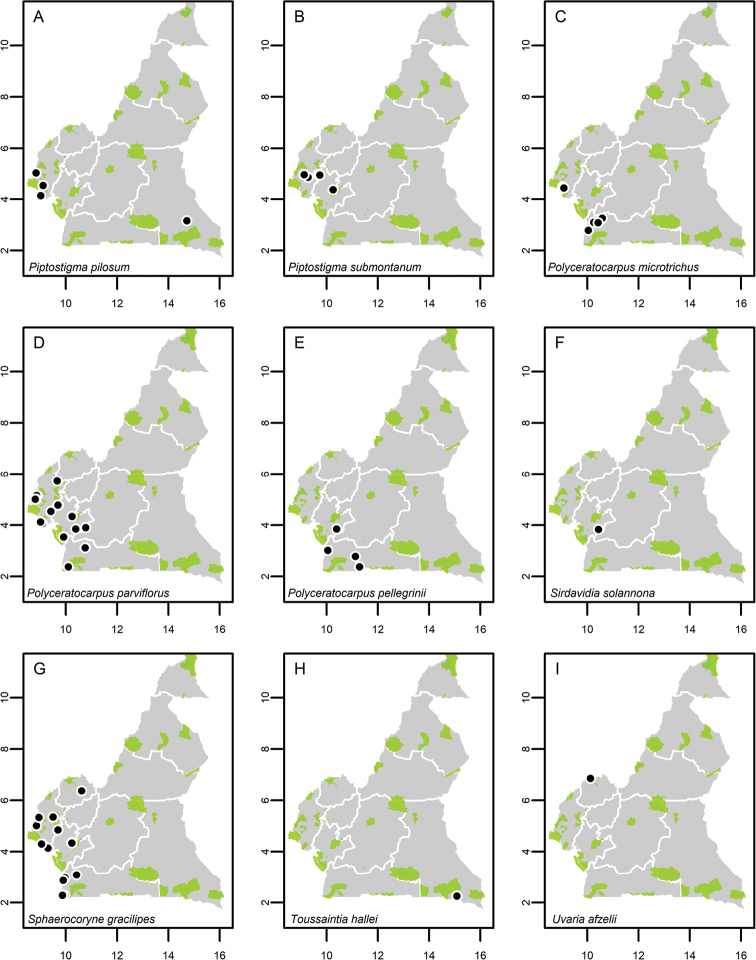
**A***Piptostigmapilosum***B***Piptostigmasubmontanum***C***Polyceratocarpusmicrotrichus***D***Polyceratocarpusparviflorus***E***Polyceratocarpuspellegrinii***F***Sirdavidiasolannona***G***Sphaerocorynegracilipes***H***Toussaintiahallei***I***Uvariaafzelii*. White borders represent region limits in Cameroon; green patches represent protected areas (see methods and Suppl. material 1: Fig. S1).

Figs 92 , 94 ; Map 12A

= Piptostigma giganteum Hutch. & Dalziel, Bull. Misc. Inform. Kew: 151, 1927. Type. Nigeria. Cross River State, Oban District, Talbot P.A. 1336, no date: holotype: K[K000199007]; isotype: K[K000199006].
= Piptostigma latipetalum Baker f.; *nom. nud.*

#### Type.

Nigeria. Cross River State; Old Calabar, *Thomson W.C. 61*, no date: holotype: K[K000199008]; isotype: B[B100154081].

#### Description.

Tree, up to 12 m tall, d.b.h. 8–10 cm; stilt roots or buttresses absent. Indumentum of simple hairs; old leafless branches glabrous, young foliate branches densely pubescent. Leaves: petiole 2–4 mm long, 2–3 mm in diameter, densely pubescent, cylindrical, blade inserted on top of the petiole; blade 25–41 cm long, 9–17.2 cm wide, **narrowly elliptic to elliptic or narrowly obovate to obovate**, apex acuminate, acumen 1.1–2.7 cm long, base rounded to cordate, subcoriaceous, below sparsely pubescent when young and old, above glabrous when young and old, discolorous, whitish below; midrib impressed, above densely pubescent when young and old, below densely pubescent when young, densely pubescent when old; secondary veins 20 to 28 pairs, glabrous above; tertiary venation percurrent. Individuals bisexual; inflorescence cauliflorous or on old leafless branches, axillary, peduncle like base ca. 10 mm long, axial internodes 3–23 mm long, **compact to sublax**, sympodial rachis 35–50 mm long. Flowers with 9 perianth parts in 3 whorls, **1 to 3 per inflorescence**; **pedicel 10–25 mm long**, ca. 3 mm in diameter, tomentose; in fruit 10 mm long, 3 mm in diameter, tomentose; basal bract ca. 12 mm long, ca. 5 mm wide; upper bract ca. 12 mm long, ca. 5 mm wide; sepals 3, valvate, free, 7–8 mm long, 6–7 mm wide, ovate, apex acute, base truncate, brown, pubescent outside, glabrous inside, margins flat; petals free, outer petals shorter than inner; outer petals 3, 7–8 mm long, 3–3.5 mm wide, obovate, apex acuminate, base truncate, light yellow cream to red towards the base, margins flat, pubescent outside, glabrous inside; inner petals 3, valvate, 30–60 mm long, 8–15 mm wide, elliptic, apex acute, base truncate, pink, margins wavy, pubescent to densely pubescent outside, pubescent inside; stamens numerous, in 6 to 8 rows, 1 mm long, broad; connective discoid, glabrous, red; staminodes absent; carpels free, **5 to 8**, ovary ca. 1 mm long, stigma globose, pubescent. Monocarps sessile, 2 to 5, 13–30 mm long, 8–18 mm in diameter, ellipsoid to ovoid, apex cuspidate, **pubescent, finely warty, longitudinally ribbed with ca. 6 ribs, light yellow to orange when ripe**; seeds up to 7 per monocarp, 1.5–3.5 mm long, 2–3 mm in diameter, ellipsoid; aril absent.

#### Distribution.

A central African species, from south eastern Nigeria to Gabon; in Cameroon known from East, South and mainly South-West regions.

#### Habitat.

A fairly uncommon species; in the understory of primary and old secondary rain forests. Altitude 50–390 m a.s.l.

#### Local and common names known in Cameroon.

None recorded.

#### IUCN conservation status.

Least Concern (LC) (Cosiaux et al. 2019aq).

#### Uses in Cameroon.

None reported.

#### Notes.

﻿﻿﻿*Piptostigmapilosum* resembles ﻿*P.macrophyllum* because of the large size of their leaf blades (25–41 cm in both species). However, the leaf blades of ﻿*P.pilosum* are mostly elliptic (sometimes however they can be obovate) with a rounded or cordate base, while those of ﻿*P.macrophyllum* are always obovate with an acute to obtuse base and the flowering pedicel is longer (10–25 mm versus 8–10 mm in *macrophyllum*). The inflorescence of ﻿*P.pilosum* generally presents few flowers per inflorescence (1 to 3) while there are 3 to 6 in ﻿*P.macrophyllum*. The species ﻿*P.fugax* and ﻿*P.oyemense* also have few flowers per inflorescence (1) but these species have fewer carpels (1 to 3 versus 5 to 8 in ﻿*P.pilosum*).

#### Specimens examined.

**East Region**: 78 km south of Yokadouma 30 km after Ngato 15 km after river ALPICAM ‘base de vie’ then 40 km on forestry road starting 4 km before Maséa village, 3.15°N, 14.72°E, *05 March 2019*, *Couvreur T.L.P.* 1204 (MPU,WAG,YA). **South-West Region**: Near Bai Kuke SE of Mbonge, 4.53°N, 9.11°E, *25 January 1958*, *Keay R.W.J.* 37372 (P); Along footpath from Ndian River at PAMOL field 69 and transect P, 5.01°N, 8.833°E, *24 January 1985*, *Thomas D.W.* 4311 (P,YA); Korup National Park, 5.01°N, 8.833°E, *12 April 1985*, *Thomas D.W.* 4755 (MO); Limbe W of Njonji Lake, 4.13°N, 9.016°E, *27 January 1994*, *Wieringa J.J.* 2030 (U,WAG).

### ﻿﻿﻿﻿ Piptostigma submontanum

Taxon classificationPlantaeMagnolialesAnnonaceae

Ghogue, Sonké & Couvreur, Pl. Ecol. Evol. 150 (2): 208, 2017

AD0E629A-025F-5253-A8B4-DC10DBB281C8

Fig. 92 ; Map 12B

#### Type.

Cameroon. South-West Region; Rumpi Mountains, between Lokando and Dikome Balue, 30 km NNW Kumba, *Letouzey R.G. 14535*, 23 Mar 1976: holotype: YA[YA0002870]; isotype: P[P02032181].

#### Description.

Tree, up to 25 m tall, d.b.h. up to 20 cm; stilt roots or buttresses absent. Indumentum of simple hairs; old leafless branches sparsely pubescent, **young foliate branches tomentose**. Leaves: petiole 2–4 mm long, 2 mm in diameter, **tomentose**, cylindrical, blade inserted on top of the petiole; blade 40–49 cm long, 16–23 cm wide, obovate, **apex acuminate to mucronate**, acumen 0.8–1.3 cm long, **base decurrent to cuneate and narrowly cordate, coriaceous**, below densely pubescent when young, densely pubescent when old, above pubescent when young, glabrous when old, discolorous, whitish below; midrib impressed, above pubescent when young and old, below pubescent when young and old; secondary veins **58 to 65 pairs**, sparsely pubescent above; tertiary venation percurrent. Individuals bisexual; inflorescence cauliflorous, peduncle like base 10–18 mm long, axial internodes 2–5 mm long, **compact**, sympodial rachis 20–40 mm long. Flowers with 9 perianth parts in 3 whorls, 1 to 4 per inflorescence; **pedicel 2–6 mm long, 2–3 mm in diameter, tomentose**; in fruit ca. 25 mm long, ca. 4 mm in diameter, tomentose; basal bract 7–8 mm long, ca. 4 mm wide; upper bract 4–6 mm long, ca. 6 mm wide; sepals 3, valvate, free, 5–8 mm long, ca. 5 mm wide, ovate, apex acute, base truncate, brown, pubescent outside, glabrous inside, margins flat; petals free, outer petals shorter than inner; outer petals 3, ca. 5 mm long, ca. 4 mm wide, ovate, apex acute, base truncate, light yellow to red, margins flat, densely pubescent outside, glabrous inside; inner petals 3, valvate, 50–60 mm long, 5–7 mm wide, narrowly elliptic, apex acute, base truncate, margins wavy, densely pubescent outside, glabrous inside; stamens numerous, in 6 to 8 rows, 1 mm long, broad; connective discoid, glabrous, red; staminodes absent; carpels free, 12 to 15, ovary ca. 2 mm long, stigma globose, pubescence not seen. Monocarps sessile, 1 to 2, 20–30 mm long, 10–25 mm in diameter, obovoid, **apex rounded, tomentose, bumpy**, brown when ripe; seeds 6 to 8 per monocarp, 6–12 mm long, 3–5 mm in diameter, ellipsoid; aril absent.

#### Distribution.

Endemic to Cameroon; known from the South-West and Littoral regions.

#### Habitat.

A fairly uncommon species; in submontane rain forests. Altitude 900–1200 m a.s.l.

#### Local and common names known in Cameroon.

None recorded.

#### IUCN conservation status.

Endangered (EN) (Cosiaux et al. 2019ar).

#### Uses in Cameroon.

None reported.

#### Notes.

See under ﻿*P.calophyllum*.

#### Specimens examined.

**Littoral Region**: Ebo Forest Reserve Djuma camp Djashaka trail, 4.36°N, 10.25°E, *15 February 2013*, *Couvreur T.L.P.* 625 (WAG,YA). **South-West Region**: Edip to Kodmin ca 1 hour’s walk, 4.96°N, 9.666°E, *02 December 1998*, *Cheek M.* 9177 (K,P,WAG,YA); Mount Kupe Kodmin, 4.96°N, 9.683°E, *21 November 1998*, *Gosline W.G.* 198 (K,P,WAG,YA); Abang road and then right to forest, 4.93°N, 9.731°E, *11 December 1999*, *Gosline W.G.* 256 (K,MO,WAG,YA); Entre Lokando (900 m) et Dikome Balue (1200 m) 30 km NNW-Kumba, 4.85°N, 9.28°E, *23 March 1976*, *Letouzey R.* 14535 (P,YA); Rumpi Hills near Madie River, 4.94°N, 9.123°E, *22 February 1995*, *Thomas D.W.* 10496 (K).

### ﻿﻿ Polyceratocarpus

Taxon classificationPlantaeMagnolialesAnnonaceae

Engl. & Diels, Notizbl. Königl. Bot. Gart. Berlin 3: 56, 1900

A7EE3A2E-9234-52FE-9A0E-6034235E0942

= Alphonseopsis Baker f., Cat. Pl. Oban: 2, 1913.

#### Type species.

﻿﻿*Polyceratocarpusscheffleri* Engler et Diels.

#### Description.

Trees, 5–12 m tall, d.b.h. up to 10 cm; stilt roots or buttresses absent. Indumentum of simple hairs. Leaves: petiole 2–8 mm long, 1–3 mm in diameter, blade 9–34 cm long, 3–10 cm wide, oblong to obovate to elliptic, apex acuminate to acute, base cuneate to rounded, discolorous, whitish below; midrib sunken or flat or raised; secondary veins 9 to 17 pairs; tertiary venation intermediate to percurrent. Species unisexual and bisexual, androdioecious, inflorescences ramiflorous on young foliate or old leafless branches, axillary. Flowers with 9 perianth parts in 3 whorls, 1 to 4 per inflorescence; male and bisexual flowers similar; pedicel 5–10 mm long; in fruit 5–15 mm long; bract 1, basal, 2–3 mm long; sepals 3, valvate, basally or entirely fused, 1–4 mm long, triangular to ovate, apex acute, base truncate; petals free, petals subequal or outer petals longer than inner; outer petals 3, valvate, 10–25 mm long, 5–9 mm wide, oblong to elliptic to ovate, apex acute, base truncate; inner petals 3, valvate, 5–14 mm long, 3–7 mm wide, oblong or elliptic to obovate, apex acute, base truncate; stamens 90 to 200 (or numerous), in 7 to 20 rows, 1–6 mm long, linear or clavate; connective discoid, glabrous; staminodes absent; carpels free, 2 to 9, ovary 2–4 mm long, stigma cylindrical, glabrous or pubescent. Monocarps sessile, 2 to 7, 20–110 mm long, 10–30 mm in diameter, oblong to obovoid, apex rounded, glabrous, smooth or ribbed; seeds biseriate, 5–20 mm long, 3–10 mm in diameter, ellipsoid; aril absent.

#### Taxonomy.

no recent revision, but see Le Thomas (1969b) for the Gabonese species.

A genus of ca. eight species; in Cameroon three species are reported, none endemic.

### ﻿Key to the Cameroonian species of ﻿*Polyceratocarpus*

**Table:** 

1	Leaf blades glabrous on lower surface, midrib raised above; outer petals 20–25 mm long	﻿***P.pellegrinii***
–	Leaf blades pubescent (sparsely or densely) on lower surface, midrib impressed, not raised; outer petals 10–15 mm long	**2**
2	Petiole 5–8 mm long; petals subequal, inner petals 10–14 mm long; fruits obovoid, 20–30 mm long, 15–25 mm in diameter, smooth not ribbed, white.	﻿***P.microtrichus***
–	Petiole 2–3 mm long; outer petals longer than inner, inner petals 5–7 mm long; fruits oblong, irregularly and transversally ribbed, green	﻿***P.parviflorus***

### ﻿﻿﻿﻿ Polyceratocarpus microtrichus

Taxon classificationPlantaeMagnolialesAnnonaceae

(Engl. & Diels) Ghesq. Ex Pellegr., Bull. Soc. Bot. France Mém: 68, 1950

AB2F8BA8-C6F3-574F-9FBB-F7617854B080

**Figure 95.**
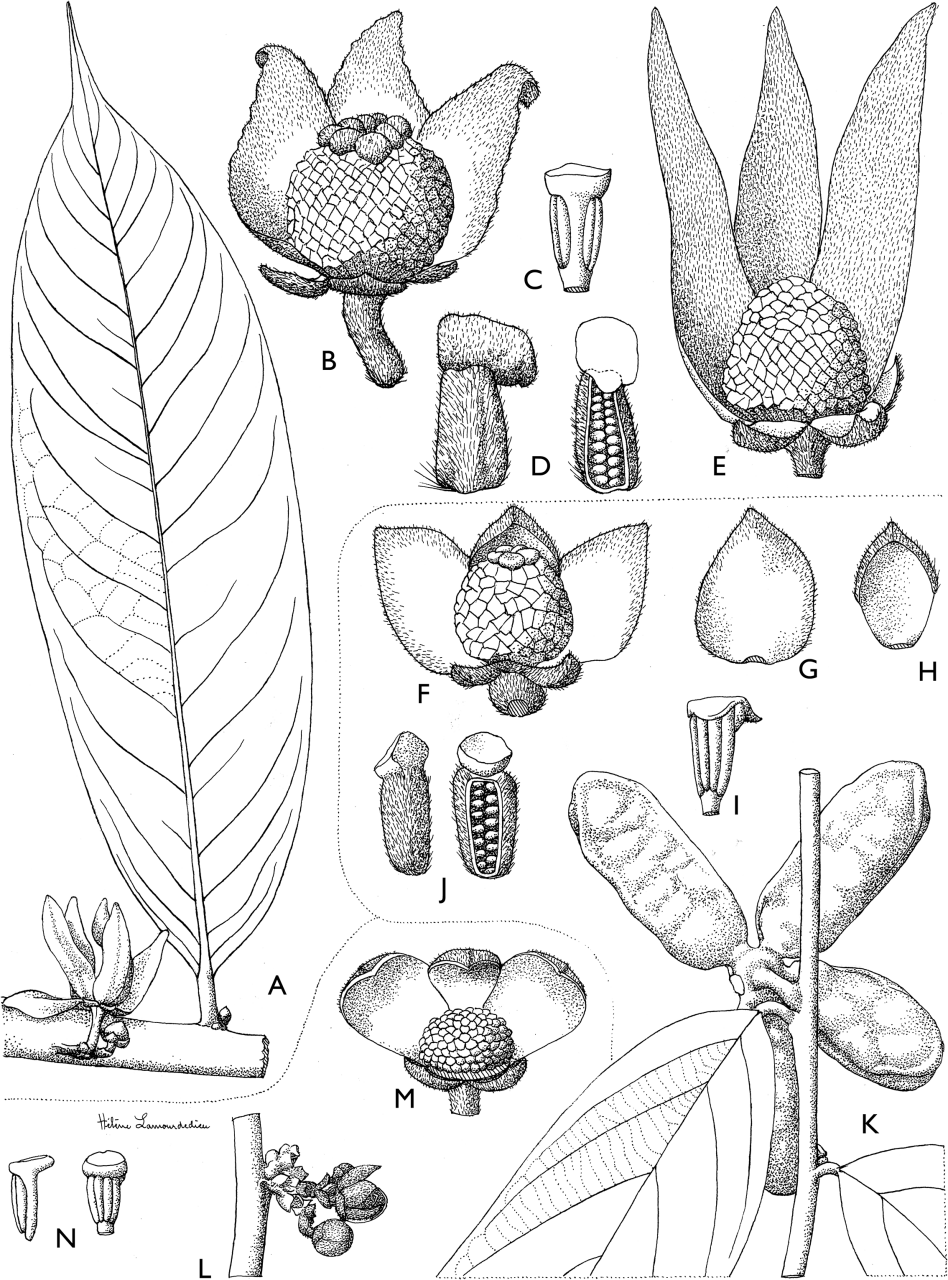
*Polyceratocarpuspellegrinii***A** flowering branch **B** detail of bisexual flower, 6 petals removed **C** stamen **D** carpel, side view, and detail of ovules **E** detail of male flower. *Polyceratocarpusparviflorus***F** bisexual flower, 4 petals removed **G** outer petal, inside view **H** inner petal, inside view **I** stamen **J** carpel, side view, detail of ovules **K** fruiting branch. *Polyceratocarpusmicrotrichus***L** flowering branch **M** bisexual flower, 4 petals removed **N** stamen, side and from view **A–E** from *Le Testu 7754***F–J** from *Le Testu 8549***K** from *Chevalier 21343***L–N** from *Hallé 2279*. Drawings by Hélène Lamourdedieu, Publications Scientifiques du Muséum national d’Histoire naturelle, Paris; modified from Le Thomas (1969b, pl. 49, p. 279).

**Figure 96.**
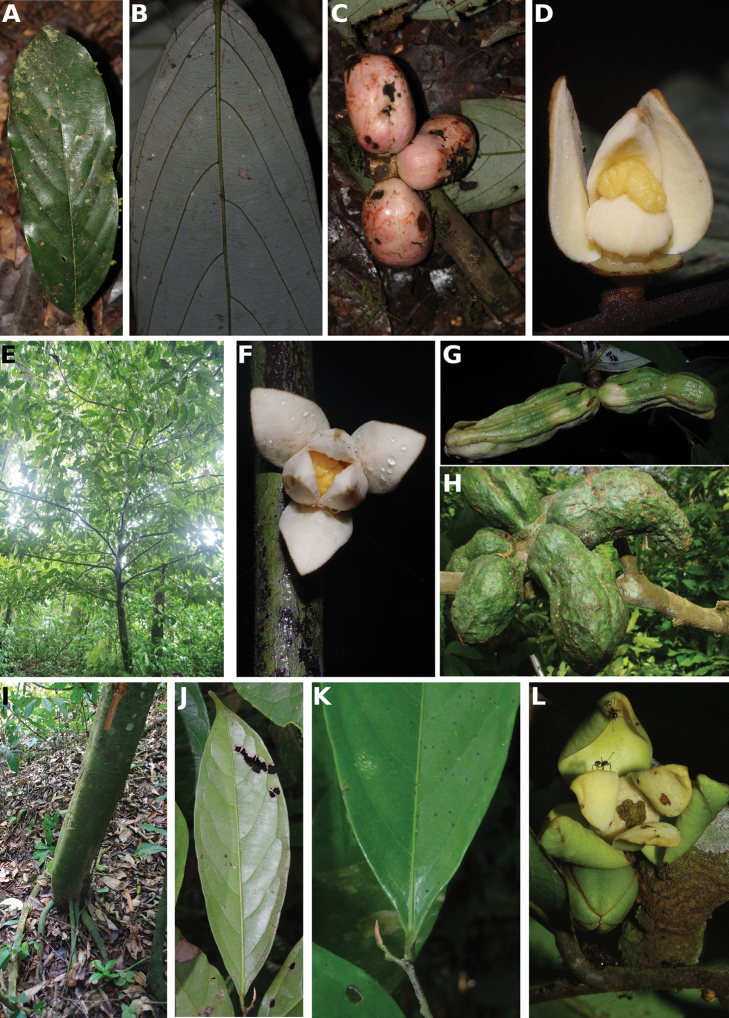
*Polyceratocarpusmicrotrichus***A** leaf, upper side **B** leaf, lower side **C** fruits, note white smooth monocarps. *Polyceratocarpusparviflorus***D** detail of bisexual flower showing receptacle, one outer petals and two inner petals removed **E** habit **F** flower, top view **G** fruits, note ribbed monocarps. *Polyceratocarpuspellegrinii***H** fruit, note curved green and irregularly ribbed monocarps **I** base of trunk **J** leaf, lower side **K** base of leaf, upper side, note raised midrib **L** flower, top view **A–C***Droissart 1881***D–G***Couvreur 1032***H, L***Couvreur 575***I–K***Couvreur1178*. Photos **A–C** Vincent Droissart **D–L** Thomas L.P. Couvreur.

Figs 95 , 96 ; Map 12C

≡ Uvaria microtricha Engl. & Diels, Bot. Jahrb. Syst. 39: 473, 1907.

#### Type.

Cameroon. South Region; Bipindi, *Zenker G.A. 2899*, Mar 1904: holotype: B[B 10 0154089]; isotypes: BR[BR0000008804808]; K[K000199038]; M[M0107917]; P[P00363351]; S[S07-13391].

#### Description.

Tree, up to 8 m tall, d.b.h. up to 10 cm; stilt roots or buttresses absent. Indumentum of simple hairs; old leafless branches glabrous, young foliate branches pubescent. Leaves: **petiole 5–8 mm long**, 1–2 mm in diameter, sparsely pubescent to glabrous, slightly grooved, blade inserted on the side of the petiole; blade 14–34 cm long, 3.5–8 cm wide, oblong to elliptic, apex acuminate to acute, acumen 0.5–1 cm long, base acute, coriaceous, below sparsely pubescent when young, sparsely pubescent to glabrous when old, above glabrous when young and old, discolorous, whitish below; midrib sunken or flat, above glabrous when young and old, below glabrous when young and old; secondary veins 9 to 15 pairs, glabrous above; tertiary venation intermediate. Individuals unisexual (?, only male flowers and fruits seen), inflorescences ramiflorous on young foliate or old leafless branches, axillary; flowers with 9 perianth parts in 3 whorls, 2 to 4 per inflorescence; male flowers: pedicel 6–8 mm long, 3–4 mm in diameter, tomentose; bract 2–3 mm long, ca. 1 mm wide; sepals 3, valvate, basally or entirely fused, cup shaped, ca. 4 mm long, 7–8 mm wide, triangular to ovate, apex acute, base truncate, tomentose outside, glabrous inside, margins flat; petals free, **subequal**; outer petals 3, **10–14 mm long**, 6–9 mm wide, oblong to elliptic, apex acute, base truncate, margins flat, densely pubescent outside, pubescent but glabrous towards the base inside; inner petals 3, valvate, **10–14 mm long**, 5–7 mm wide, obovate to oblong, apex acute, base truncate, margins flat, tomentose outside, pubescent inside; stamens 90 to 110, in 7 to 10 rows, 2–3 mm long, linear to clavate; connective discoid, glabrous; staminodes absent; female flowers only seen in fruit; fruiting pedicel 10–20 mm long, glabrous. Monocarps sessile, 2 to 5, 20–30 mm long, 15–25 mm in diameter, **obovoid**, apex rounded, glabrous, **smooth, slightly bumpy when dry, not ribbed, white when ripe**; seeds 10 to 14 per monocarp, 5–10 mm long, 3–4 mm in diameter, ellipsoid; aril absent.

#### Distribution.

From Cameroon to Gabon, in Cameroon known from the South and South-West regions.

#### Habitat.

A rare species in Cameroon; in premontane primary rain forests. Altitude: 100–700 m a.s.l.

#### Local and common names known in Cameroon.

None recorded.

#### IUCN conservation status.

Not evaluated.

#### Uses in Cameroon.

None reported.

#### Notes.

See under ﻿*P.parviflorus*.

The collection *Watts 627* was suggested to be a new species (Cable and Cheek 1998, p. 11) but having seen the specimen in K, we have identified it as ﻿*P.microtrichus*.

#### Specimens examined.

**South Region**: 20 km SE of Kribi route minière E of Mt Eléphant, 2.78°N, 10.03°E, *01 April 1970*, *Bos J.J.* 6679 (WAG); ca 45 km from Kribi ca 8 km N of Lolodorf road Forest towards Lokoundje R, 3.1°N, 10.25°E, *02 April 1970*, *Bos J.J.* 6684 (BR,K,LD,LM,MO,P,WAG,YA); Ngovayang, 3.25°N, 10.57°E, *08 June 2015*, *Droissart V.* 1881 (MO); Bipindi, 3.08°N, 10.42°E, *01 January 1904*, *Zenker G.A.* 2899 (BR,L,P,WAG); Bipindi, 3.08°N, 10.42°E, *01 January 1907*, *Zenker G.A.* 3270 (L,P); Bipindi, 3.08°N, 10.42°E, *01 December 1913*, *Zenker G.A.* 478 (P,U,WAG). **South-West Region**: Mokoko Forest Reserve Dikome, 4.48°N, 9.033°E, *05 May 1994*, *Ekema S.N.* 950 (K); Mokoko Forest Reserve ca 6 km W of Mundongo, 4.43°N, 9.083°E, *22 March 1993*, *Watts J.* 627 (K).

### ﻿﻿﻿﻿ Polyceratocarpus parviflorus

Taxon classificationPlantaeMagnolialesAnnonaceae

(Baker f.) Ghesq., Rev. Zool. & Bot. Afr. 32: 140, 1939

323725C8-130F-52ED-9D4B-476398DA1545

Figs 95 , 96 ; Map 12D

≡ Alphonseopsis parviflora Baker f., Cat. Pl. Oban: 3, 1913.

#### Type.

Nigeria. Cross River State; Oban, *Talbot P.A. 1607*, no date: holotype: K[K000199039].

#### Description.

Tree, 5–12 m tall, d.b.h. up to 10 cm; stilt roots or buttresses absent. Indumentum of simple hairs; old leafless branches glabrous, young foliate branches pubescent. Leaves: **petiole 2–3 mm long**, 1–2 mm in diameter, glabrous, cylindrical, blade inserted on top of the petiole; blade 9–23 cm long, 3–10 cm wide, oblong to obovate, apex acuminate, acumen 0.5–1 cm long, base acute to rounded, subcoriaceous, below densely pubescent when young, sparsely pubescent when old, above glabrous when young and old, discolorous, whitish below; impressed, above glabrous when young and old, below glabrous when young and old; secondary veins 9 to 11 pairs, glabrous above; tertiary venation percurrent. Individuals andromonoecious, male and hermaphrodite inflorescences similar, ramiflorous on old leafless branches, axillary. Flowers with 9 perianth parts in 3 whorls, 1 to 2 per inflorescence; pedicel 5–6 mm long, 3–4 mm in diameter, pubescent; in fruit 5–15 mm long, 4–7 mm in diameter, glabrous; bract 2–3 mm long, 2–3 mm wide; sepals 3, valvate, basally or entirely fused, cup shaped, 1–2 mm long, 2–3 mm wide, ovate, apex acute, base truncate, green to brown-red, tomentose outside, glabrous inside, margins flat; petals free, **outer petals longer than inne**r; outer petals 3, **10–15 mm long**, 6–8 mm wide, ovate, apex acute, base truncate, **white**, margins flat, densely pubescent outside, pubescent and glabrous towards base inside; inner petals 3, valvate, **5–7 mm long**, 3–4 mm wide, obovate, apex acute, base truncate, white, margins flat, tomentose outside, glabrous inside; male flowers: stamens 80 to 90, in 6 to 10 rows, 2–6 mm long, linear to clavate; connective discoid, glabrous, white; stamens in hermaphrodite flowers: 50 to 60, in 4 to 6 rows, 4–6 mm long, connective elongate, glabrous; staminodes absent; carpels free, 2 to 4, ovary 3–4 mm long, stigma cylindrical, glabrous. Monocarps sessile, 2 to 4, **35–40 mm long, 10–15 mm in diameter**, **oblong**, apex rounded, glabrous, **irregularly and transversally ribbed**, otherwise smooth, light green to white when ripe; seeds 12 to 14 per monocarp, 8–10 mm long, 3–5 mm in diameter, flattened ellipsoid; aril absent.

#### Distribution.

A widespread species with a disjunct distribution in West Africa, and central Africa, from eastern Nigeria to Gabon. In Cameroon known from Littoral, South and South West regions.

#### Habitat.

A common species when present; in lowland or premontane primary rain forests. Altitude: 100–800 m a.s.l.

#### Local and common names known in Cameroon.

None recorded.

#### IUCN conservation status.

Least Concern (LC) (Botanic Gardens Conservation International and IUCN SSC Global Tree Specialist Group 2019b).

#### Uses in Cameroon.

None reported.

#### Notes.

﻿﻿﻿*Polyceratocarpusparviflorus* is hard to distinguish from ﻿*P.microtrichus* based on vegetative or flowering material. It differs by having shorter inner petals (5–7 mm long versus 10–14 mm long) and oblong irregularly ribbed green fruits, in contrast to obovoid and smooth white fruits in ﻿*P.microtrichus*.

#### Specimens examined.

**Littoral Region**: At Tissongo 16 km EES of Mouanko, 3.53°N, 9.909°E, *18 January 1984*, *Asonganyi J.N.* 711 (P,YA); Mapubi 30 km before Edea on Yaoundé-Edea road On forestry road 5 km direction to Sanaga river, 3.84°N, 10.38°E, *28 February 2018*, *Couvreur T.L.P.* 1174 (MPU,P,WAG,YA); Ebo Wildlife Reserve Djuma permanent camp On Djuma-Djuma trail, 4.34°N, 10.23°E, *14 February 2014*, *Couvreur T.L.P.* 623 (WAG,YA); Ebo Wildlife Reserve Djuma permanent camp On transect 5, 4.33°N, 10.23°E, *16 February 2013*, *Couvreur T.L.P.* 632 (WAG,YA); Mambe Massif above Boga village 100 km along road from Yaoundé to Ed 3.90°N, 10.77°E, *19 June 2014*, *Couvreur T.L.P.* 645 (WAG,YA). **South Region**: Mvini à Campo, 2.37°N, 10.09°E, *01 October 1983*, *Mitani M.* 134 (P,YA); ca 7 km NE of Ebom, 3.11°N, 10.75°E, *01 August 1996*, *Parren M.P.E.* 190 (KRIBI,WAG). **South-West Region**: 43 km SE of Mamfe Kendem collines, 5.72°N, 9.66°E, *08 June 1985*, *Achoundong G.* 1332 (YA); Kupe village, 4.78°N, 9.716°E, *30 May 1996*, *Cable S.* 2721 (K,MO,WAG,YA); Ekundu Kundu, 5.15°N, 8.883°E, *27 April 1996*, *Cheek M.* 8244 (K,WAG,YA); Mount Cameroon National Park Bakinguili trail above Bakinguili village, 4.08°N, 9.050°E, *02 April 2016*, *Couvreur T.L.P.* 1032 (WAG,YA); Mount Cameroon National Park Bakinguili trail above Bakinguili village, 4.09°N, 9.057°E, *02 April 2016*, *Couvreur T.L.P.* 1036 (WAG,YA); on trail through palm oil plantation 3 km before lava flow and Seme Beach hotel when coming from Limbe, 4.06°N, 9.077°E, *18 October 2013*, *Couvreur T.L.P.* 515 (WAG,YA); Parc National de Korup Mt Yuhan entre 700 et 850 m Forêt mature de terre ferme transition plaine-submontagnard, 5.15°N, 8.860°E, *06 March 2012*, *Droissart V.* 1221 (BRLU,MO,YA); Below Kupe rock near Esense river, 4.78°N, 9.683°E, *25 January 1995*, *Elad M.* 86 (K,YA); Kupe village, 4.77°N, 9.688°E, *28 November 1999*, *Gosline W.G.* 236 (K); Kupe village, 4.77°N, 9.688°E, *28 November 1999*, *Gosline W.G.* 237 (K,MO,P,WAG,YA); Pres Kendonge (Reserve forestiere de Bakundu) 13 km SSW Kumba, 4.54°N, 9.42°E, *20 April 1976*, *Letouzey R.* 14647 (WAG,YA); Korup National Park collected between the Ndian River at Pamol field and 25 km on transect “P”, 5.01°N, 8.833°E, *12 April 1985*, *Thomas D.W.* 4785 (YA); Cameroon Mountain, 4.12°N, 9.009°E, *20 June 2001*, *van Andel T.R.* 3707 (U,WAG); Korup National Park, 5.01°N, 8.806°E, *22 March 2004*, *van der Burgt X.M.* 675 (WAG,YA).

### ﻿﻿﻿﻿ Polyceratocarpus pellegrinii

Taxon classificationPlantaeMagnolialesAnnonaceae

Le Thomas, Adansonia sér. 2, 5: 451, 1965

573D72EF-FFE7-582B-A872-33D19B7A0073

Figs 95 , 96 ; Map 12E

#### Type.

Gabon. Ogooué-Lolo; Poungui, *Le Testu G.M.P.C. 7754*, 10 Dec 1929: lectotype, sheet here designated: P[P00363348]; isotypes: BR[BR0000008805041]; P[P00363347].

#### Description.

Tree, 5–6 m tall, d.b.h. up to 10 cm; stilt roots or buttresses absent. Indumentum of simple hairs; old leafless branches glabrous, young foliate branches glabrous. Leaves: petiole 5–6 mm long, 2–3 mm in diameter, pubescent, slightly grooved, blade inserted on top of the petiole; blade 13–31 cm long, 3.6–5 cm wide, oblong, apex acuminate, acumen 0.5–1 cm long, base narrowly cuneate to shortly attenuate, coriaceous, below glabrous when young and old, above glabrous when young and old, (concolorous or) discolorous, whitish below; **midrib raised above**, above glabrous when young and old, below glabrous when young and old; secondary veins 13 to 17 pairs, glabrous above; tertiary venation intermediate. Individuals andromonoecious, male and hermaphrodite inflorescences similar, ramiflorous on old leafless branches, axillary. Flowers with 9 perianth parts in 3 whorls, 1 to 2 per inflorescence; pedicel ca. 10 mm long, 3–4 mm in diameter, pubescent; in fruit 5–6 mm long, 5–6 mm in diameter, pubescent; bract 2–3 mm long, ca. 1 mm wide; sepals 3, valvate, basally or entirely fused, cup shaped, 3–4 mm long, ca. 6 mm wide, triangular to ovate, apex acute, base truncate, green to brown-red, densely pubescent outside, glabrous inside, margins flat; petals free, outer petals longer than inner; outer petals 3, **20–25 mm long**, 5–7 mm wide, oblong to elliptic, apex acute, base truncate, **yellow**, margins flat, pubescent outside, glabrous inside; inner petals 3, valvate, 10–14 mm long, 6–7 mm wide, elliptic to oblong, apex acute, base truncate, **yellow**, margins flat, pubescent outside, pubescent inside; stamens 160 to 200, in 8 to 10 rows, 1–2 mm long, linear; connective discoid, glabrous, white; staminodes absent; carpels free, 8 to 9, ovary 3–4 mm long, stigma cylindrical, pubescent. Monocarps sessile, 5 to 7, **50–110 mm long, 20–30 mm in diameter, oblong**, apex rounded, glabrous, **irregularly and transversally ribbed**, otherwise smooth, dark green when ripe; seeds 12 to 14 per monocarp, 15–20 mm long, ca. 10 mm in diameter, ellipsoid; aril absent.

#### Distribution.

From Cameroon to Gabon, and one collection from Democratic Republic of Congo; in Cameroon known from the Littoral and South regions.

#### Habitat.

An uncommon and rarely collected species in Cameroon; in lowland or premontane primary or old secondary rain forests. Altitude: 0–600 m a.s.l.

#### Local and common names known in Cameroon.

None recorded.

#### IUCN conservation status.

Not evaluated.

#### Uses in Cameroon.

None reported.

#### Notes.

﻿﻿﻿*Polyceratocarpuspellegrinii* is distinguished by its conspicuous raised midrib unique within the genus, and yellow flowers (versus white in ﻿*P.parviflorus*, color of ﻿*P.microtrichus* unknown). Le Thomas (1969b) indicates that the fruits are unknown but they have since been collected (e.g. *Couvreur 575* from Gabon) and are here described.

#### Specimens examined.

**Littoral Region**: Mapubi 30 km before Edea on Yaoundé-Edea road On forestry road 5 km direction to Sanaga river, 3.84°N, 10.38°E, *28 February 2018*, *Couvreur T.L.P.* 1178 (MPU,WAG). **South Region**: 20 km From Kribi N of Lolodorf road (SFIA logging road), 3.01°N, 10.05°E, *15 July 1969*, *Bos J.J.* 5049 (WAG); Ongongondjé Hill (Ambam), 2.38°N, 11.28°E, *27 December 1975*, *de Wilde J.J.F.E* 8718 (BR,K,MO,P,U,WAG,YA); Colline au SE de Ndengué 15 km S d’Ebolowa, 2.78°N, 11.12°E, *26 March 1970*, *Letouzey R.* 10248 (P,YA).

### ﻿﻿ Sirdavidia

Taxon classificationPlantaeMagnolialesAnnonaceae

Couvreur & Sauquet, PhytoKeys 46: 4, 2015.

E8A323EE-C1C0-5613-8AA1-7263EE669B92

#### Type species.

﻿﻿﻿*Sirdavidiasolannona* Couvreur & Sauquet.

#### Description.

Same as species.

#### Taxonomy.

Couvreur et al. (2015).

### ﻿﻿﻿﻿ Sirdavidia solannona

Taxon classificationPlantaeMagnolialesAnnonaceae

Couvreur & Sauquet, PhytoKeys 46: 4, 2015

397DA421-6E8C-50F1-B07C-60C8A7BAA788

**Figure 97.**
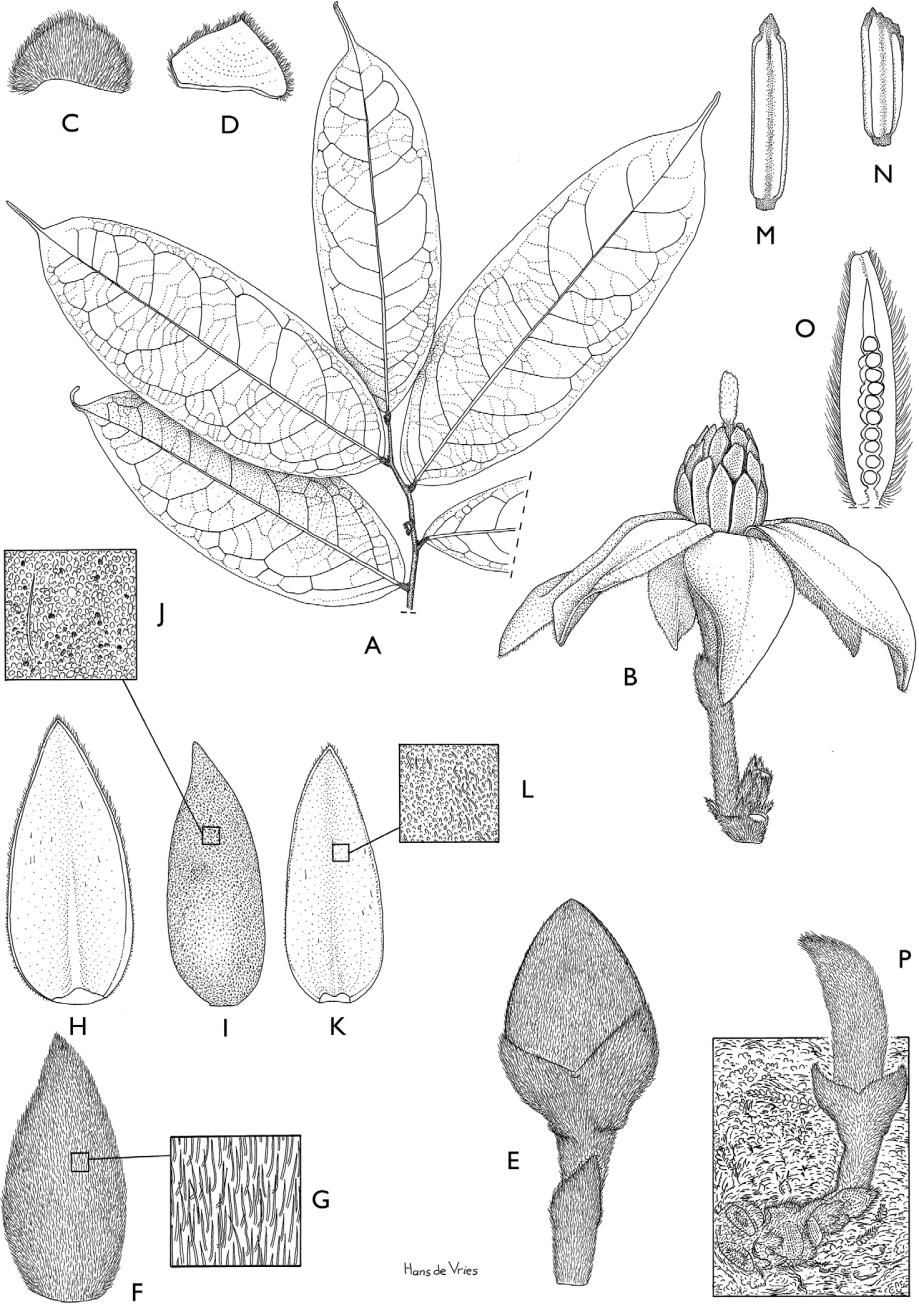
*Sirdavidiasolannona***A** flowering branch **B** flower **C** sepal, outer side view **D** sepal, inner view **E** flower bud **F** outer petal, outer view **G** detail of pubescence of outer petal, outer side **H** outer petal, inner view **I** inner petal, outer view **J** detail of pubescence of inner petal, outer side **K** inner petal, inner view **L** detail of pubescence of inner petal, inner side **M** stamen from inner whorl **N** stamen from outer whorl **O** longitudinal section of carpel showing uniseriate row of ovules (stigma missing) **P** detail of young fruit **A–P** from *Couvreur 596*, *597*. Drawing by Hans de Vries (Couvreur et al. 2015, fig. 3, p. 8).

**Figure 98.**
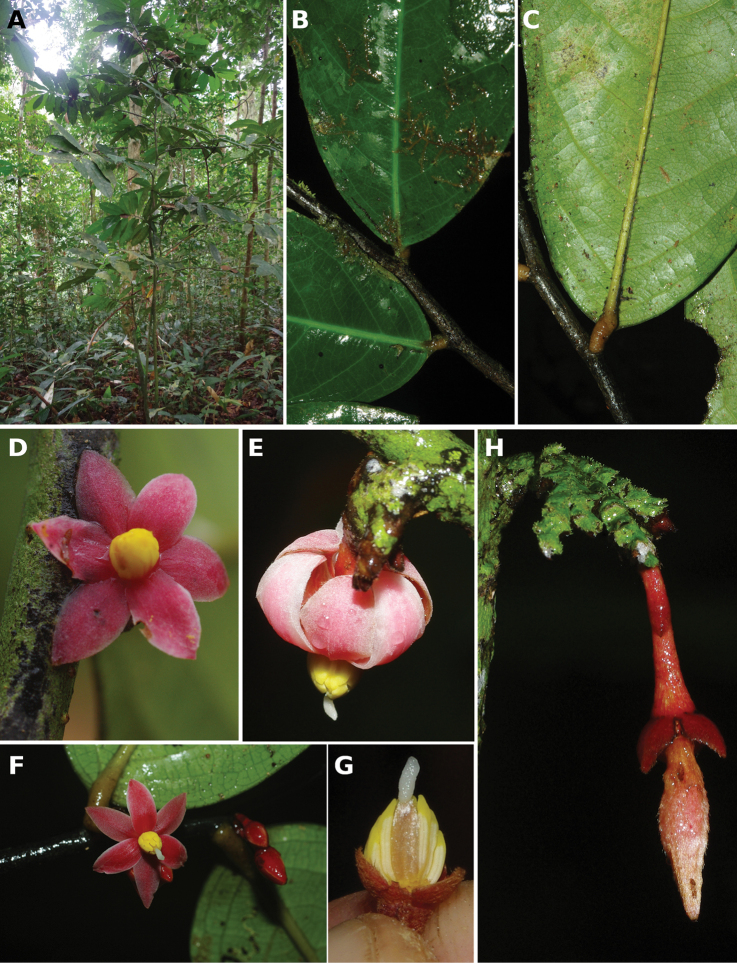
*Sirdavidiasolannona***A** habit **B** base of leaf blade, upper side **C** base of leaf base, lower side **D** flower **E** flower, top view **F** flower **G** detail of receptacle, petals and 1 sepal removed **H** young cauliflorous fruit **A** Couvreur, no voucher **B–H***Couvreur 597, 596*, Gabon. Photos Thomas L.P. Couvreur.

Figs 97 , 98 ; Map 12F

#### Type.

Gabon. Estuaire; Monts de Cristal, near first bridge after Kinguele, *Couvreur T.L.P. 596*, 15 Nov 2013: holotype: WAG[WAG0392343]; isotypes: LBV; P[P04022675]; YA; WAG[WAG0392342].

#### Description.

Tree, 4–6 m tall, d.b.h. 2–4 cm; stilt roots or buttresses absent. Indumentum of simple hairs; old leafless branches glabrous, young foliate branches sparsely pubescent. Leaves: petiole 3–4 mm long, 2–3 mm in diameter, sparsely pubescent to glabrous, slightly grooved, blade inserted on top of the petiole; blade 20–26 cm long, 4.5–9 cm wide, ovate to elliptic or obovate, **apex long acuminate, acumen 2–3 cm long**, base obtuse, coriaceous, below glabrous when young and old, above sparsely pubescent to glabrous when young, glabrous when old, concolorous; midrib sunken or flat, above glabrous when young and old, below sparsely pubescent when young, glabrous when old; secondary veins 9 to 12 pairs, glabrous above; tertiary venation reticulate. Individuals bisexual [possibly androdioecious], inflorescences cauliflorous or ramiflorous on young foliate or old leafless branches, axillary. Flowers with 9 perianth parts in 3 whorls, 1 to 3 per inflorescence; pedicel 2–10 mm long, densely pubescent; in fruit 8–15 mm long, diameter unknown, glabrous; bracts several basal and one upper towards the lower half of pedicel, bracts ca. 1 mm long, ca. 1 mm wide; sepals 3, valvate, free, 2–3 mm long, ca. 2 mm wide, ovate, apex acute, base truncate, pink to light red, densely pubescent outside, glabrous inside, margins flat; petals free, valvate, sub equal; outer petals 3, 4–10 mm long, 2.5–5 mm wide, elliptic, apex acute, base truncate, red, margins flat, densely pubescent outside, pubescent inside, reflexed; inner petals 3, valvate, 4–9 mm long, 2–4 mm wide, elliptic, apex acute, base truncate, pink to light red, margins flat, densely pubescent outside, pubescent at least towards margins inside, reflexed; stamens 16 to 19, in 2 to 3 rows, 3–4 mm long, linear; connective tongue shaped, glabrous, **bright yellow**; staminodes absent; **carpel 1**, ovary 4–5 mm long, stigma cylindrical to coiled, sparsely pubescent. Monocarp sessile, 1, 50–70 mm long, 15–18 mm in diameter, oblong, apex apiculate, sparsely pubescent to glabrous (pubescent when young), **smooth, longitudinally 4 to 6 ribbed**; seeds not counted, biseriate, 12–16 mm long, 6–7 mm in diameter, ellipsoid; aril absent.

#### Distribution.

Cameroon and Gabon; in Cameroon known from the Littoral region.

#### Habitat.

A rare species, only collected once in 1973 in Cameroon, but it is common in the forest understory when present (e.g. Monts de Cristal, Gabon); in lowland primary or old secondary rain forests on non-inundated soils. Altitude 100–600 m a.s.l.

#### Local and common names known in Cameroon.

None recorded.

#### IUCN conservation status.

Vulnerable (VU) (Cosiaux et al. 2019as).

#### Uses in Cameroon.

None reported.

#### Notes.

﻿﻿﻿*Sirdavidiasolannona* is easily distinguished by its long acuminate leaves (acumen 2–3 cm long), flowers with bright yellow stamens and small red reflexed petals, resembling the flower of a Solanaceae nor even *Ardisia* (Primulaceae) (Olivier Lachenaud, pers. com), its single carpel and thus single monocarp with 6 to 8 ribs. The androdioecious nature [flowers unisexual staminate or bisexual] of this species needs to be confirmed. We observed male and bisexual flowers in Gabon. *Letouzey 12405* suggests that male flowers are cauliflorous and hermaphrodite ones are ramiflorous, which needs to be confirmed.

﻿﻿﻿*Sirdavidiasolannona*, the only species of the genus, was thought to be endemic to Gabon (Couvreur et al. 2015). However, after its description, a herbarium collection from Cameroon, *Letouzey 12405* was identified (dating from 1973). We returned to this same locality in 2018, but unfortunately could not locate the species.

In the original description, we didn’t have access to a fruiting specimen. Here, we provide a description of the fruit (*Moungazi 1544, LBV*).

#### Specimen examined.

**Littoral Region**: near Nkak Ndjok (ca Mapubi) 30 km ENE Ed 3.83°N, 10.43°E, *17 December 1973*, *Letouzey R.* 12405 (K,P).

### ﻿﻿ Sphaerocoryne

Taxon classificationPlantaeMagnolialesAnnonaceae

Scheff. Ex Ridl., J. Straits Branch Roy. Asiat. Soc. 75: 8, 1917

683B45AD-3539-5F05-B30D-A0CD441D4562

#### Type species.

*Polyalthiasiamensis* Boerl. (= ﻿***Sphaerocoryneaffinis*** (Teijsm. & Binn.) Ridl.).

#### Description.

Same as species.

A genus with four species having a disjunct distribution between Africa and South East Asia. Two species are known from Africa, one (﻿*S.gracilipes*) in Central Africa (Nigeria, Cameroon, Gabon, Equatorial Guinea) and one (*S.gracilis*) in East Africa (Verdcourt 1971a). In Cameroon one species, not endemic.

The Central African species was initially placed within ﻿*Friesodielsia* (﻿*F.gracilipes* (Benth.) Steenis) (Le Thomas 1969b), but a recent molecular phylogeny of ﻿*Friesodielsia*, ﻿*Monanthotaxis* and associated liana genera showed that this species clusters with ﻿*Sphaerocoryne* (Guo et al. 2017b). Other African species of ﻿*Friesodielsia* have been transferred to ﻿*Monanthotaxis* and ﻿*Afroguatteria*, and thus the genus ﻿*Friesodielsia* does not occur in Africa anymore, being strictly South East Asian.

### ﻿﻿﻿﻿ Sphaerocoryne gracilipes

Taxon classificationPlantaeMagnolialesAnnonaceae

(Benth.) X.Guo & R.M.K.Saunders, Taxon 66(1): 15, 2017

7F10A334-3DE8-510E-8BEF-F33FD7547340

**Figure 99.**
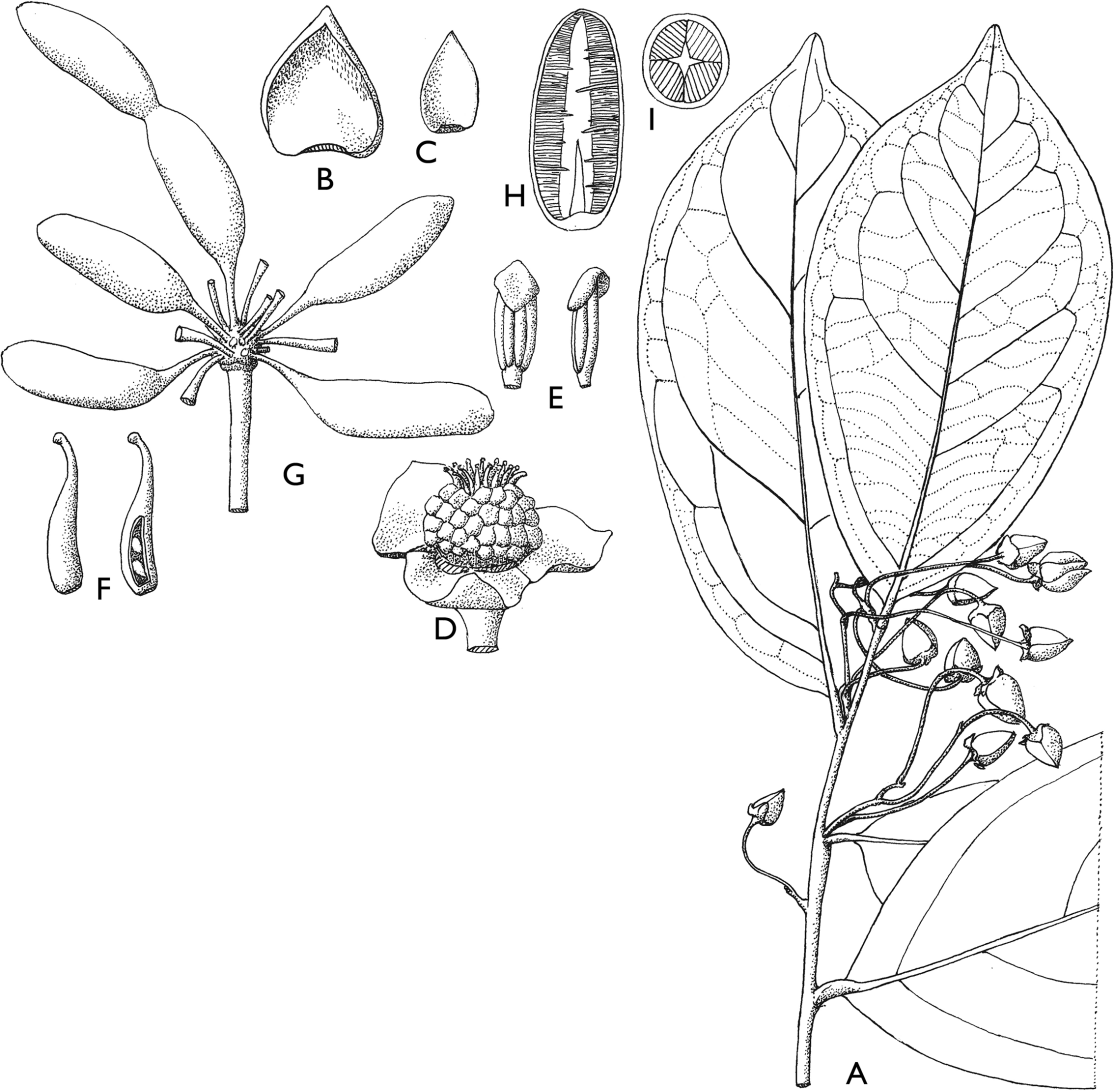
*Sphaerocorynegracilipes***A** flowering branch **B** outer petal, inner view **C** iner petal, inner view **D** detail of receptacle, all petals removed **E** stamen, view and front views **F** carpel, side view and detail of ovules **G** fruit **H** longitudinal section of seed **I** transversal section of seed **A–F** from *Zenker 516***G–I** from *Annet 465*. Drawings by Hélène Lamourdedieu, Publications Scientifiques du Muséum national d’Histoire naturelle, Paris; modified from Le Thomas (1969b, pl. 42, p. 237).

**Figure 100.**
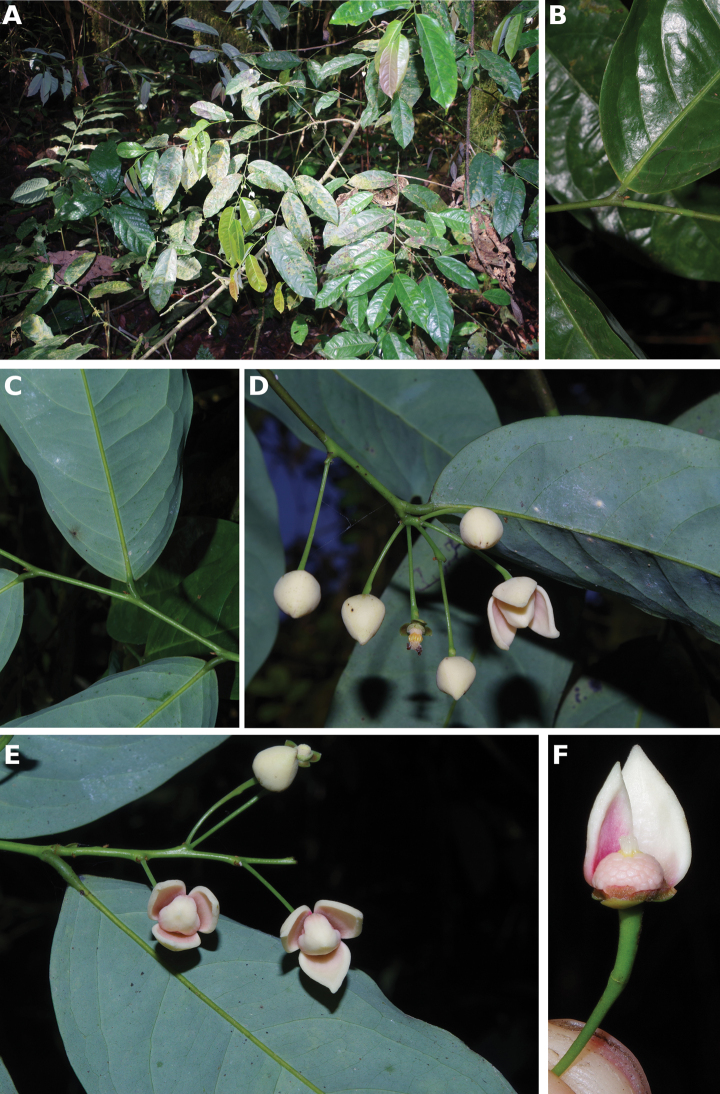
*Sphaerocorynegracilipes***A** habit **B** leaf base, upper side **C** leaf base, lower side, note light green color **D** flowering branch **E** flowering branch, lower view **F** detail of flower, 4 petals removed **A–F***Couvreur 1043*, Mt Cameroon, Cameroon. Photos Thomas L.P. Couvreur.

Figs 99 , 100 ; Map 12G

≡ Oxymitra gracilipes Benth., Trans. Linn. Soc. London 23: 471–472, 1862; ﻿﻿Cleistopholisgracilipes (Benth.) Engl. & Diels, Engler in Monogr. Afrik. PflanzenFam. 6: 34, 1901; ﻿Richellagracilipes (Benth.) R.E.Fr., Engler & Prantl in Nat. Pflanzenfam., ed. 2, 17 a(2): 139, 1959; ﻿﻿Friesodielsiagracilipes (Benth.) Steenis, Blumea 12: 359, 1964.
= Unona albida Engl., Notizbl. Königl. Bot. Gart. Berlin 2: 297, 1899; ﻿﻿Cleistopholisalbida (Engl.) Engl. & Diels, Monogr. Afrik. Pflanzen-Fam. 6: 34, 1901; ﻿Oxymitraalbida (Engl.) Sprague & Hutch., Bull. Misc. Inform. Kew 1916: 153–154. 1916; ﻿Richellaalbida (Engl.) R.E.Fr. in Engler & Prantl, Nat. Pflanzenfam., ed. 2, 17 a(2): 139. 1959; ﻿﻿Friesodielsiaalbida (Engl.) Steenis, Blumea 12: 358. 1964. Type. Cameroon. South Region, Bipinde, Zenker G.A. 1715, 1898: Lectotype, designated by Guo et al. 2017, p. 15: B[B 10 0153057]; isolectotypes: B[B 10 0153058]; BM[BM000547065, BM000843987]; BR[BR000008800121]; E[E00181435]; G[G00308362]; HBG[HBG-502539]; K[K000198947]; L[L 0187107]; M[M-0107909]; NY[NY0026308]; P[P00363331, P00363333]; S; U[U 0269929 (wood sample)]; US; WAG[WAG0061084]; WU[WU025877].
= ﻿﻿﻿Cleistopholis albida var. longipedicellata Baker f., in Rendle & al., Cat. Pl. Oban: 3–4, 1913; Oxymitra longipedicellata (Baker f.) Sprague & Hutch., Bull. Misc. Inform. Kew 1916: 154, 1916; ﻿Richellalongipedicellata (Baker f.) R.E.Fr., in Engler & Prantl, Nat. Pflanzenfam., ed. 2, 17 a(2): 139, 1959; ﻿﻿Friesodielsialongipedicellata (Baker f.) Steenis, Blumea 12: 360, 1964. Type. Nigeria, Oban, Talbot P.A.1677, 1912: Lectotype, designated by Guo et al. (2017b), p. 15: BM *n.v.*; isolectotype: BM *n.v.*

#### Type.

Equatorial Guinea. Bioko, Fernando Po, *Mann G. 251*, 1860: holotype: K[K000198951].

#### Description.

**Liana**, 2–15 m tall, d.b.h. 1–3 cm. Indumentum of simple hairs; **old leafless branches glabrous, drying black**, young foliate branches sparsely pubescent to glabrous. Leaves: petiole up to 5 mm long, ca. 1 mm in diameter, glabrous, slightly grooved, blade inserted on the side of the petiole; blade 4.5–17 cm long, 2–6 cm wide, elliptic to ovate, apex acuminate to acute, acumen ca. 1 cm long, base acute (rarely cordate), papyraceous, **below glabrous when young and old**, above glabrous when young and old, **discolorous**, **whitish below**; midrib sunken or flat, above glabrous when young and old, below glabrous when young and old; secondary veins 9 to 12 pairs, glabrous above; **tertiary venation reticulate**. Individuals bisexual; **inflorescences ramiflorous on young foliate branches, axillary**. Flowers with 9 perianth parts in 3 whorls, 2 to 7 per inflorescence; **pedicel 25–50 mm long**, ca. 1 mm in diameter, glabrous; in fruit 25–40 mm long, 2–3 mm in diameter, glabrous; bracts 2, one basal and one towards the upper half of pedicel, bracts ca. 1 mm long, ca. 1 mm wide; sepals 3, valvate, free, ca. 2 mm long, ca. 2 mm wide, semiorbicular, apex rounded, base truncate, green, glabrous outside, inside pubescent towards margins but otherwise glabrous, margins flat; petals free, valvate, outer petals larger than inner; outer petals 3, 5–15 mm long, 5–10 mm wide, elliptic to ovate, apex acute, base truncate, cream to white, margins flat, glabrous outside, inside pubescent towards margins but otherwise glabrous; inner petals 3, valvate, 5–7 mm long, 2.5–3 mm wide, ovate to oblong, apex acute, base truncate, cream to bright white, margins flat, glabrous outside, glabrous inside; stamens 60 to 80, in 8 to 9 rows, ca. 2 mm long, linear; connective discoid, glabrous, white; staminodes absent; carpels free, 10 to 12, ovary 2–3 mm long, stigma filiform, glabrous. Monocarps stipitate, stipes 8–10 mm long, 2–3 mm in diameter; monocarps 10 to 14, **25–30 mm long, 8–13 mm in diameter, oblong, apex rounded, glabrous, smooth**, sometimes with a prominent rib when dried, red when ripe; seeds 1 to 4 per monocarp, 20–25 mm long, 7–10 mm in diameter, oblong; aril absent.

#### Distribution.

A central African species, from Nigeria to Gabon and Equatorial Guinea; in Cameroon known from the Littoral, South and South-West regions.

#### Habitat.

A fairly common species, in lowland or submontane primary or old secondary rain forests. Altitude 0–800 m a.s.l.

#### Local and common names known in Cameroon.

None recorded.

#### IUCN conservation status.

Not assessed yet.

#### Uses in Cameroon.

None reported.

#### Notes.

﻿﻿﻿*Sphaerocorynegracilipes* is distinguished by its glaucous (light green) lower side of leaf blades, glabrous branches (or sparsely pubescent when very young), leaves and petioles, branches drying black, long pedicellate flowers (25–50 mm long) with bright white petals, and moniliform fruits. This species (and the genus) is easily confused when sterile with ﻿*Monanthotaxis*, both being lianas with a glaucous lower leaf surface and moniliform monocarps. However, ﻿*Monanthotaxis* has percurrent tertiary venation versus reticulate in ﻿*Sphaerocoryne* (Guo et al. 2017b). For the same reason as above, ﻿*Sphaerocoryne* is also very close morphologically to ﻿﻿*Afroguatteriadiscostigma* both being lianas with glaucous lower leaf surface and reticulate tertiary venation, but ﻿*Sphaerocoryne* has axillary inflorescences while ﻿*Afroguatteria* has terminal ones (Guo et al. 2017b). The four liana genera ﻿*Afroguatteria*, ﻿*Monanthotaxis*, ﻿*Sphaerocoryne* and ﻿*Toussaintia* are phylogenetically close (Guo et al. 2017b) but remain separate on morphological grounds.

In the check list to the plants of Mt Kupe and Bakossi (Cheek et al. 2004), the fruiting collection *Cable 3526* (K) is cited as “﻿*Monanthotaxis sp. nov.*” but is identified here as ﻿﻿﻿*Sphaerocorynegracilipes*. The only major difference with the rest of the material is that this specimen has cordate leaf bases, when all other specimens we have seen have acute leaf bases.

#### Specimens examined.

**Littoral Region**: Ebo Wildlife Reserve Djuma permanent camp On Djashaka trail, 4.35°N, 10.24°E, *13 February 2014*, *Couvreur T.L.P.* 617 (WAG,YA); Ebo Wildlife Reserve Djuma permanent camp On transect 5, 4.33°N, 10.23°E, *16 February 2013*, *Couvreur T.L.P.* 633 (WAG,YA). **South Region**: N bank of Lobé river above Gr Batanga ferry ca. 9 km S of Kribi, 2.87°N, 9.893°E, *14 October 1968*, *Bos J.J.* 3077 (P,WAG,YA); ca 9 km S of Kribi Lobe R bank E of Gr Batanga ferry, 2.86°N, 9.9°E, *11 January 1969*, *Bos J.J.* 3602 (P,WAG,YA); Lobé R mouth 7 km S of Kribi, 2.88°N, 9.9°E, *20 March 1969*, *Bos J.J.* 4180 (BR,K,LD,LM,MO,P,WAG,YA); 10 km From Kribi Lolodorf road, 2.96°N, 9.966°E, *27 May 1969*, *Bos J.J.* 4655 (BR,P,WAG); Ndoumalé 11 km S of Kribi, 2.86°N, 9.9°E, *29 August 1969*, *Bos J.J.* 5264 (WAG); Campo-Ma’an area Bibabimvoto, 2.28°N, 9.950°E, *16 August 2002*, *Tchouto Mbatchou G.P.* 3400 (KRIBI,WAG); Campo-Ma’an area 2.28°N, 9.866°E, *02 October 2001*, *van Andel T.R.* 4095 (KRIBI,U,WAG,YA); Bipindi, 3.08°N, 10.41°E, *1898*, *Zenker G.A.* 1715 (E,L,WAG); Bipindi, 3.08°N, 10.41°E, *01 May 1913*, *Zenker G.A.* 360 (U,WAG); Bipindi, 3.08°N, 10.41°E, *01 April 1914*, *Zenker G.A.* 516 (U,WAG). **South-West Region**: Nyasoso, 4.84°N, 9.689°E, *02 July 1996*, *Cable S.* 3526 (K,WAG,YA); Bayang Mbo Wildlife Sanctuary after Mbu river, 5.35°N, 9.501°E, *25 March 2016*, *Couvreur T.L.P.* 1001 (WAG,YA); Mount Cameroon National Park on the Bomona trail behind Bomona village 10 km NW from Idenau, 4.29°N, 9.090°E, *03 April 2016*, *Couvreur T.L.P.* 1043 (WAG,YA); Buea area at Bolifamba, 4.13°N, 9.303°E, *01 March 1929*, *Maitland T.D.* 536 (K,K,YA); Sousi Forest, 6.36°N, 10.61°E, *16 February 2006*, *Onana J.M.* 3612 (K); Disturbed forest Bomana and Koto II, 4.3°N, 9.05°E, *26 April 1996*, *Tchouto Mbatchou G.P.* 1372 (K,YA); Korup National Park, 5.01°N, 8.85°E, *10 March 1986*, *Thomas D.W.* 5727 (MO); Korup National Park, 5.33°N, 8.95°E, *25 May 1988*, *Thomas D.W.* 7858 (P,YA).

### ﻿﻿ Toussaintia

Taxon classificationPlantaeMagnolialesAnnonaceae

Boutique, Bull. Jard. Bot. État Bruxelles 21: 97, 1951

304B9259-C16C-518F-A26D-C3A6C71879BE

#### Type species.

*Tousaintiacongolensis* Boutique.

#### Description.

Same as species.

A small genus of four species (Luke and Deroin 2005) distributed in central and east Africa, one species occurs in Cameroon, not endemic.

﻿*Toussaintia* is a genus of lianas or scandent shrubs, characterized by the presence of an androgynophore (elongated receptacle on which the stamens and carpels are inserted). All species are rare across their range.

#### Taxonomy.

There is no revision available for this genus yet, but Luke and Deroin (2005) provide a key to all four species.

### ﻿﻿﻿﻿ Toussaintia hallei

Taxon classificationPlantaeMagnolialesAnnonaceae

Le Thomas, Adansonia sér. 2, 7: 99, 1967

D530102D-258A-51F2-99AD-7A146A51BB3D

Fig. 101 ; Map 12H

#### Type.

Gabon. Ogooué-Ivindo; Belinga, *Hallé N. 4189*, 2 Jul 1966: lectotype, sheet here designated: P[P00046762]; isotypes: P[P00046760, P00046761].

#### Description.

**Liana**, 10–15 m tall, d.b.h. 4 cm. Indumentum of simple hairs; old leafless branches glabrous, young foliate branches tomentose. Leaves: petiole 4–7 mm long, ca. 1 mm in diameter, pubescent, grooved, blade inserted on top of the petiole; blade 5–14 cm long, 3.5–5 cm wide, oblong to elliptic, apex acuminate, acumen ca. 1 cm long, base rounded, subcoriaceous, below pubescent when young and old, above glabrous when young and old, concolorous; midrib impressed, above glabrous when young and old, below sparsely pubescent when young, glabrous when old; secondary veins 7 to 10 pairs, glabrous above; **tertiary venation reticulate, very dense and tight**. Individuals bisexual; inflorescences ramiflorous on young foliate branches, axillary. **Flowers with 12 to 14 perianth parts in 3 to 4 whorls**, 1 to 2 per inflorescence; peduncle short, ca. 2 mm long; pedicel 10–17 mm long, 1–2 mm in diameter, tomentose; **one large bract at the base of the inflorescence, 7–13 mm long, 5–7 mm wide, elliptic**; floral bracts 2 to 4, one basal and several upper towards the lower half of pedicel, basal bract 5–9 mm long, 4–7 mm wide, elliptic; upper bracts 5–8 mm long, 4–6 mm wide, elliptic; sepals 3, valvate, free, 10–18 mm long, 5–7 mm wide, elliptic to ovate, apex acute, base truncate, green, tomentose outside, glabrous inside, margins flat, slightly reflexed in bud; petals free, valvate, sub equal; petals 15–30 mm long, 5–9 mm wide, narrowly obovate, apex rounded, base truncate, greenish-yellow, streaked with red-brown, base shading into bright white, margins flat, pubescent outside, glabrous inside; staminodes absent; **stamens 300 to 350, in 20 to 30 rows arranged on a long conical receptacle (androgynophore)**, ca.1 mm long, linear; connective discoid, glabrous, yellow; carpels free, 20 to 22, ovary 4–5 mm long, stigma shortly bilobed, glabrous. Fruits unknown.

**Figure 101.**
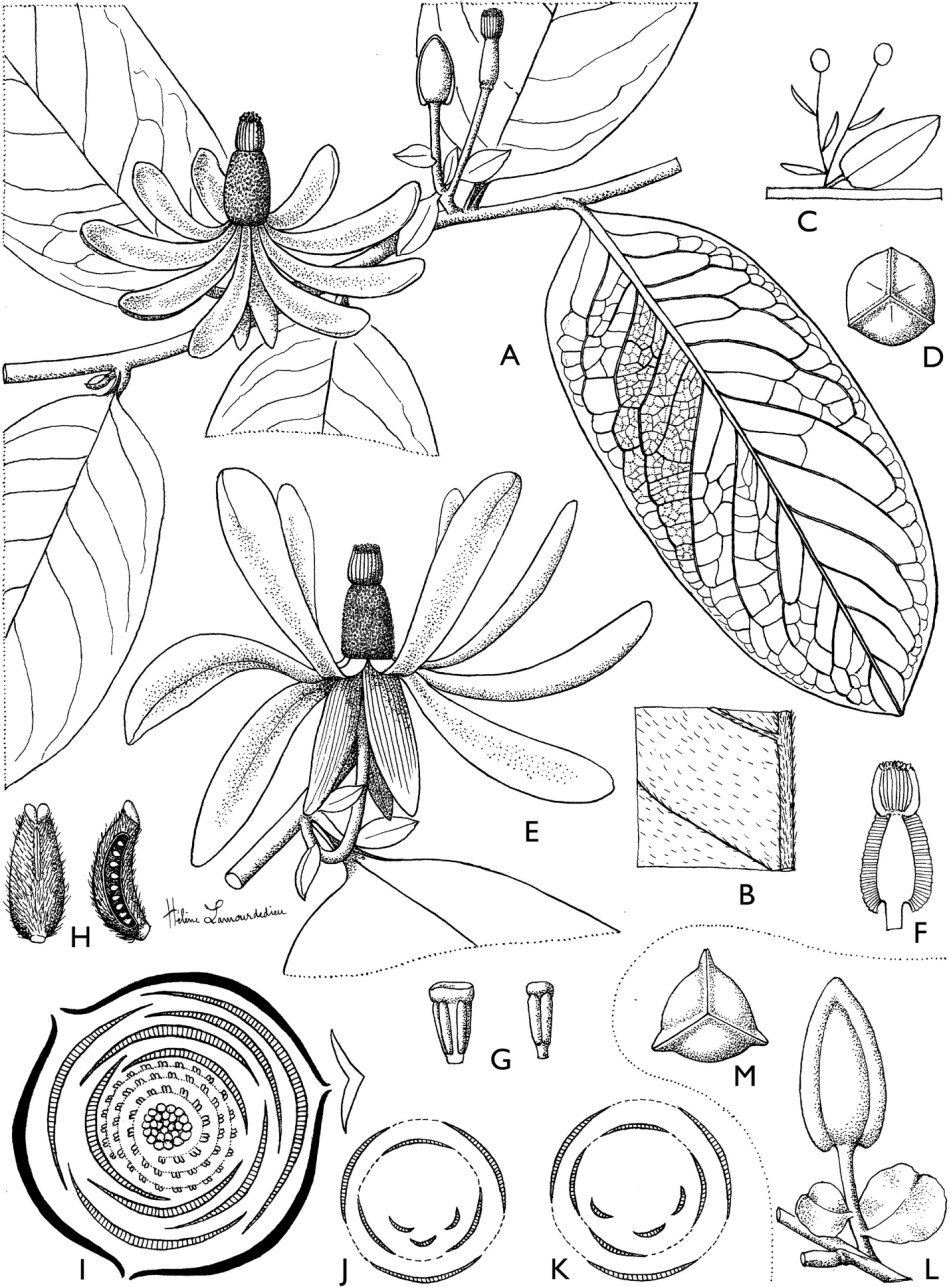
*Toussaintiahallei***A** flowering branch, note the dense network of tertiary veins on levaes **B** detail of pubescence lower leaf side **C** inflorescence diagram with large prophyl at the base **D** flower bud, top view **E** detail of opened flower, note the androgynophore part of receptacle **F** longitudinal cut of flowering receptacle **G** details of stamens, side and front views **H** details of carpels front view, and latitudinal cut **I** floral diagram (black. sepals; dashed petals) **J, K** floral diagrams showing the variable number of petals; *Toussaintiacongolensis* Boutique (not in Cameroon) **L** flower bud **M** flower bud top view **A–K***Hallé 4189*, based on living material **L, M***Wagemans 1677*. Drawings by Hélène Lamourdedieu, Publications Scientifiques du Muséum national d’Histoire naturelle, Paris; modified from Le Thomas (1969b; pl. 3, p. 31).

#### Distribution.

A central African species, known from Gabon, Cameroon and Republic of Congo. In Cameroon known from the East region (one collection).

#### Habitat.

A very rare species across its range. In primary rain forests. Altitude ca. 400 m a.s.l.

#### Local and common names known in Cameroon.

None recorded.

#### IUCN conservation status.

Not evaluated.

#### Uses in Cameroon.

None reported.

#### Notes.

﻿﻿﻿*Toussaintiahallei* is characterized by leaves that are shiny above and have a reticulate and very dense tertiary venation. The flowers have 9 to 10 petals inserted in 2 to 3 whorls and numerous stamens inserted horizontally on a very strongly convex receptacle referred to as an androgynophore, all unusual and unique features among Cameroonian Annonaceae (Le Thomas 1967b, 1969b; Luke and Deroin 2005). This species is rarely collected and is known from a single collection in Cameroon.

#### Specimen examined.

**East Region**: ca 30 km NW of Moloundou, 2.25°N, 15.08°E, *07 December 1982*, *de Kruif A.P.M.* 871 (WAG,YA).

### ﻿﻿ Uvaria

Taxon classificationPlantaeMagnolialesAnnonaceae

L. , Sp. Pl. 1: 536, 1753

41011587-2D9E-5F0C-BF3E-DC459DD04F72

= Uva Brun., Thes. Zeylan.: 231, 1737: *nom. illegit.*, *superfl.*; Narum Adanson, Fam. 2: 365, 1763: *nom. illegit.*, *superfl.*; Xylopiastrum Roberty, Bull. I.F.A.N. 15: 1387, 1953; Melodorum Lour., Fl. Cochinch. 329: 351, 1790; Marenteria Noronha ex Thouars, Gen. Nov. Madagasc.: 18, 1806; Cyathostemma Griff., Not. Pl. Asiat. 4: 707, 1854; Ellipeia Hook.f. & Thomson, Fl. Ind. 104, 1855; Anomianthus Zoll., Linnaea 29: 324, 1858; Tetrapetalum Miq., Ann. Mus. Bot. Lugduno-Batavi 2: 23, 1865; Rauwenhoffia Scheff., Ann. Jard. Bot. Buitenzorg 2: 21, 1881; Uvariella Ridl., Fl. Malay. Penins. 1: 22, 1922; Ellipeiopsis R.E.Fr., Verstreute Beob. Fam. Annon.: 41, 1953; Dasoclema J.Sinclair, Gard. Bull. Singapore 14: 273, 1955; Balonga Le Thomas, Adansonia sér. 2, 8: 106, 1968.

#### Type species.

﻿*Uvariazeylanica*L. (a South East Asian species).

#### Description.

Lianas, up to 20(–30) m tall, d.b.h. up to 20 cm. Indumentum of star, fasciculate and/or simple hairs. Leaves: petiole 2–15 mm long, 1–2 mm in diameter; blade 5–26 cm long, 1–3 cm wide, elliptic, ovate, obovate or oblong, apex acuminate to obtuse, base acute to cordate, discolorous, whitish below or concolorous; midrib sunken or flat; secondary veins 6 to 25 pairs; tertiary venation reticulate or percurrent. Individuals bisexual; inflorescences cauliflorous and ramiflorous on old or young foliate branches, leaf opposed or extra axillary. Flowers with 9 perianth parts in 3 whorls, 1 to 5 per inflorescence; pedicel 2–50 mm long; in fruit 5–60 mm long; bracts 2 (or 1), one basal and one upper, 1–15 mm long; sepals 3, valvate (or imbricate), free or basally fused or completely fused tearing at anthesis, 2–20 mm long, ovate or triangular to semiorbicular, apex acute or acuminate or truncate, base truncate; petals free; outer petals 3, valvate or imbricate, 7–35 mm long, 5–25 mm wide, ovate to elliptic to obovate to semiorbicular, apex acute to rounded, base truncate; inner petals 3, **imbricate**, 7–35 mm long, 6–25 mm wide, ovate to elliptic to obovate to semiorbicular, apex acute to obtuse to rounded, base truncate; stamens 100 to 400, in 5 to 10 rows, 1–3 mm long, linear to cuneiform; connective discoid or tongue-shaped, glabrous or pubescent; staminodes absent; carpels free, 15 to 70 (or numerous), 2–4 mm long, stigma flat or bilobed or coiled or cylindrical, pubescent or glabrous. Monocarps sessile to stipitate, stipes 5–45 mm long; monocarps 8 to 48, 6–70 mm long, 6–40 mm in diameter, globose or ellipsoid or cylindrical, apex rounded to apiculate, smooth, bumpy, ridged, verrucose or echinate, sometimes strongly ornamented; **seeds numerous, bi or uniseriate**, 7–15 mm long, 4–10 mm in diameter, ellipsoid or flattened ellipsoid; aril absent.

A diverse genus of ca. 200 species distributed across Africa (west to east), Madagascar and in South East Asia, 17 species occur in Cameroon, one endemic.

﻿*Uvaria* is a genus of lianas or scrambling shrubs, most of which have stellate hairs and numerous seeds per monocarp. The taxonomy of this genus remains complicated and a recent continental revision is still lacking. Differences between the species are mostly based on fruit and leaf characters.

#### Taxonomy.

There has yet to be a complete taxonomic revision of African and Malagasy ﻿*Uvaria* since Engler and Diels (1901). Taxonomy and keys to species of different regions can be found in: Paiva (1966) and Paiva and Bárrios (2019) for Angola; Le Thomas (1969b) for Gabon; Verdcourt (1971a) for Tropical East Africa, Cavaco et Keraudren (1958) for Madagascar and Hawthorne and Jongkind (2006) for West Africa (Senegal to Ghana). Besides Africa some revisions have been published for South East Asia providing excellent sources for taxonomy and morphological characters within the genus (Utteridge 2000; Zhou et al. 2009, 2010; Meade and Parnell 2018).

In the key below, we tried to use as many vegetative characters as possible, although in some cases flower or fruit characters are needed. Using mainly vegetative characters is an advantage, but also has drawbacks linked to some characters that can be variable (e.g. pubescent density could vary). The user should be aware of that and check the descriptions and illustrations carefully to confirm identification.

We provide here illustrations to the different types of hairs and combinations which will help with the key (Fig. 101).

### ﻿Key to the species of ﻿*Uvaria* in Cameroon

**Table:** 

1	Young foliate branches and petioles sparsely pubescent, glabrescent or glabrous	**2**
–	Young foliate branches and petiole tomentose to densely pubescent	**8**
2	Upper side of leaf blade completely glabrous even along the midrib in old leaves	**3**
–	Upper side of leaf blade pubescent, at least along the midrib	**5**
3	Leaves generally oblong, leaf base largely cordate, inflorescence 1 to 3 flowered, flowering pedicel < 10 mm long, monocarps sessile	﻿***U.obanensis***
–	Leaves elliptic to ovate, leaf base acute to rounded or subcordate, inflorescence one flowered, flowering pedicel > 10 mm long, monocarps stipitate	**4**
4	Sepals valvate; stipes 10–15 mm long, monocarps cylindrical	﻿***U.chamae***
–	Sepals imbricate, stipes 20–30 mm long, monocarps ellipsoid, smooth, two-ribbed	﻿***U.buchholzii***
5	Tertiary veins percurrent (parallel) but not very visible; sepals free in bud not tearing at anthesis, monocarps globose (10–18 × 10–18 mm)	﻿﻿**U.muricatavar.yalingensis**
–	Tertiary veins reticulate (network-like); sepals completely fused in bud and tearing at anthesis; monocarps cylindrical (13–45 × 6–20 mm)	**6**
6	Leaf margin thickened, leaf base acute, secondary veins > 14 and weak	﻿***U.chamae***
–	Leaf base rounded, subcordate to obtuse, secondary veins < 14 and prominent	**7**
7	Lower side of leaf blade densely to sparsely pubescent with simple or fasciculate hairs; blade connective of stamens tongue shaped	﻿***U.angolensis***
–	Lower side of leaf blade glabrous; connective of stamens discoid	﻿***U.versicolor***
8	Leaves strongly discolorous, their lower surface completely covered with minute stellate hairs obscuring the tertiary venation	**9**
–	Leaves green on both sides, their lower surface clearly visible between the hairs and with conspicuous tertiary venation	**12**
9	Lower side of leaf blade with a dense lower layer of minute stellate hairs and a more scattered upper layer of larger stellate hairs, secondary veins > 15, prominent below, tertiary veins percurrent.	**10**
–	Lower side of leaf blade with stellate hairs of two sizes, but not forming conspicuous distinct strata, secondary veins < 15, not prominent below, tertiary veins reticulate	**11**
10	Upper side of leaf blade pubescent with stellate; sepals free, reflexed, flower bud pyramidal	﻿***U.anisotricha***
–	Upper side of leaf blade generally glabrous above; sepals completely fused in bud and tearing at anthesis, flower bud globose	﻿***U.baumannii***
11	Upper side of leaf blade smooth and glabrous; sepals basally fused, flowering pedicel 5–8 mm long, monocarps globose and rounded with stipes 25–40 mm long	﻿***U.klaineana***
–	Upper side of leaf blade pubescent with minute scabrid hairs, sepals fused (but not tearing at anthesis); flowering pedicel 10–30 mm long, monocarps cylindrical and apiculate with stipes 10–15 mm long	﻿***U.osmantha***
12	Tertiary veins reticulate (network)	**13**
–	Tertiary veins percurrent (parallel)	**17**
13	Upper side of leaf blades and midrib pubescent	**14**
–	Upper side of leaf blades glabrous, midrib pubescent or not	**15**
14	Both sides of leaf blade with simple and stellate hairs; monocarps ellipsoid, shortly pubescent	﻿***U.comperei***
–	Lower side of leaf blade with long fasciculate hairs, upper side with sparse non-scabrid simple hairs; monocarps club-shaped, densely pubescent, hispid	﻿***U.afzelii***
15	Twigs and lower side of leaf blade with intermixed simple and stellate hairs; monocarps subglobose and long-stipitate.	﻿***U.heterotricha***
–	Twigs and lower side of leaf blade with fasciculate or simple hairs only; monocarps cylindrical, shortly stipitate (unknown in ﻿*U.mollis*)	**16**
16	Secondary veins > 13 pairs; sepals completely fused in bud and tearing at anthesis, monocarps cylindrical (13–45 × 6–20 mm) (common, widespread)	﻿***U.angolensis***
–	Secondary veins < 13 pairs; sepals not fused, fruits unknown (rare, endemic)	﻿***U.mollis***
17	Upper side of leaf blades usually with simple scabrid (hard) hairs (but sometimes almost glabrous), monocarps sessile forming a compact fruit	﻿﻿***U.scabrida***
–	Upper side of leaf blades usually with simple non-scabrid (hard) hairs, monocarps medium to long stipiate	**18**
18	Lower side of leaf blade with long fasciculate hairs; inflorescence with up to 5 flowers and usually cauliflorous, monocarps cylindrical with up to four ribs	﻿***U.bipindensis***
–	Lower side of leaf blade with minute stellate hairs; inflorescence one flowered and never cauliflorous, monocarps globose, verrucose, not ribbed	**19**
19	Leaf blade with simple hairs above and stipitate stellate hairs below, secondary veins > 16; sepals free, stipes more than twice as long as monocarps, 30 to 35 monocarps	﻿***U.poggei***
–	Leaf blade glabrous above and with sessile stellate hairs below, secondary veins < 16; sepals fused, not tearing at anthesis, stipes as long as the monocarps, 8 to 2 monocarps	﻿***U.anonoides***

### ﻿﻿﻿﻿ Uvaria afzelii

Taxon classificationPlantaeMagnolialesAnnonaceae

Scott Elliot, J. Linn. Soc., Bot. 30(206): 70, 1895

F326C1A0-52E8-5854-8C43-8B46F16D2846

Map 12I

#### Type.

Sierra Leone. Southern Province; between Kahreni and Port Lokko, *Scott Elliot G.F. 5812*, Apr 1891: holotype: K[K000198777].

#### Description.

Liana, ca. 3 m tall, d.b.h. unknown. Indumentum of simple or fasciculate 4 or 3 branched hairs; old leafless branches sparsely pubescent to glabrous, **young foliate branches hirsute**. Leaves: petiole 3–4 mm long, 1 mm in diameter, densely pubescent **erect hairs**. Leaves: petiole 3–4 mm long, 1 mm in diameter, densely hirsute, slightly grooved, blade inserted on top of the petiole; **blade 15–20 cm long, 4–6cm wide**, oblong to ovate, apex acute, **base cordate**, subcoriaceous, **below densely pubescent with fasciculate hairs** when young, pubescent when old, **above pubescent with simple hairs** when young, sparsely pubescent to glabrous when old; midrib sunken or flat, above densely pubescent with simple hairs when young and old, below pubescent with fasciculate hairs when young and old; **secondary veins 11–15** pairs, sparsely pubescent to glabrous above; **tertiary venation reticulate**. Individuals bisexual; inflorescences ramiflorous on old or young foliate branches, leaf opposed or extra axillary. Flowers with 9 perianth parts in 3 whorls, 1 per inflorescence; pedicel 35–50 mm long, 1–2 mm in diameter, densely pubescent; in fruit 40–55 mm long, 2 mm in diameter, densely pubescent; bracts 2, one basal and one upper towards the lower part of the pedicel, basal bract 3–8 mm long, 2–3 mm wide; upper bract 3–7 mm long, 2–3 mm wide; sepals 3, valvate, basally fused, 8–10 mm long, 4–6 mm wide, ovate, apex obtuse, base truncate, pubescent outside, tomentose inside, margins flat; petals free, sub equal; outer petals 3, 17–22 mm long, 13–17 mm wide, ovate, apex obtuse, base truncate, yellow, margins flat, pubescent outside, densely pubescent inside; inner petals 3, imbricate, 13–20 mm long, 10–13 mm wide, ovate, apex obtuse, base attenuate, yellow, margins flat, pubescent outside, tomentose inside, hairs longer towards center; stamens 150 to 200, in 8 to 9 rows, 2–3 mm long, linear; connective tongue shaped, pubescent; staminodes absent; carpels free, 40 to 50, ovary 2–3 mm long, stigma conical, glabrous. Monocarps stipitate, **stipes 35–45 mm long**, 2–3 mm in diameter; monocarps 13 to 25, 13–18 mm long, 10–15 mm in diameter, **club-shaped** with **the stipes inserted laterally**, apex rounded or mucronate, **densely pubescent with erect hairs**, smooth, **constricted around the seeds (2 or 3 bumps)**, yellow brown to pale orange when ripe; seeds 4 to 6 per monocarp, 12–15 mm long, 8–10 mm in diameter, flattened ellipsoid; aril absent.

#### Distribution.

A mainly west African species, just reaching into Cameroon with a single collection to date, in the North-West region.

#### Habitat.

A rare species in Cameroon (a single collection); along scrub vegetation near cultivation. Altitude 10–600 m a.s.l.

#### Local and common names known in Cameroon.

None recorded.

#### IUCN conservation status.

Not evaluated.

#### Uses in Cameroon.

None reported.

#### Notes.

﻿﻿﻿*Uvariaafzelii* is easily recognized by the long golden erect hairs on the young foliate branches and petioles, leaf blades with the combination of simple hairs above and fasciculate hairs below, and especially by its unique club-shaped monocarps that are constricted around the seeds and densely pubescent with erect golden hairs.

#### Specimen examined.

**North-West Region**: Nser et Banji 50 km N Wum, 6.85°N, 10.12°E, *11 July 1975*, *Letouzey R.* 14016 (P,YA).

### ﻿﻿﻿﻿ Uvaria angolensis

Taxon classificationPlantaeMagnolialesAnnonaceae

Welw. ex Oliv., Fl. Trop. Afr. 1: 23, 1868

F1FFF646-66F3-57FC-9C9B-E1E6CA287932

**Map 13.**
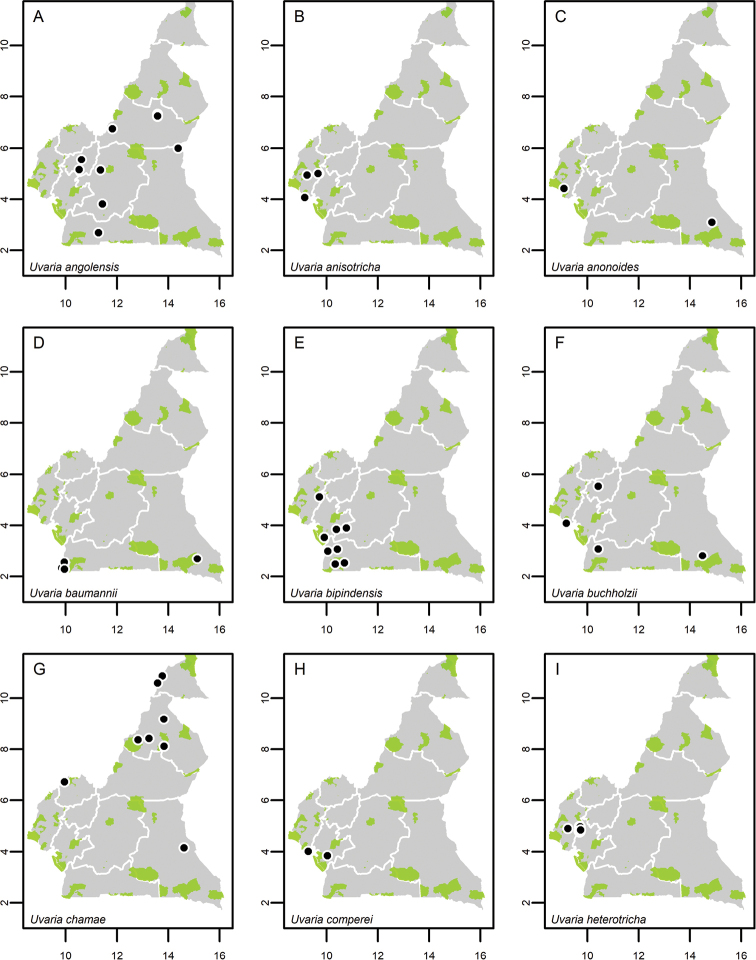
**A***Uvariaangolensis***B***Uvariaanisotricha***C***Uvariaanonoides***D***Uvariabaumannii***E***Uvariabipindensis***F***Uvariabuchholzii***G***Uvariachamae***H***Uvariacomperei***I***Uvariaheterotricha*. White borders represent region limits in Cameroon; green patches represent protected areas (see methods and Suppl. material 1: Fig. S1).

Figs 103 , 113 ; Map 13A

= Uvaria bukobensis Engl., Pflanzenw. Ost-Afrikas C: 178, 1895. Type. Tanzania: Kagera region, Bukoba, Stuhlmann F.L. 1132, no date: lectotype, here designated: K[K000198760].
= Uvaria angolensis var. guineense Keay, Kew Bull. 8: 71, 1953. Type. Cameroon. Kunde, *Mildbraed G.W.J. 9224*, 3 May 1914: holotype: K[K000198772].
= Uvaria variabilis De Wild., Pl. Bequaert. I: 461, 1922. Type. Democratic Republic of the Congo. Orientale, bord de la Semliki, Lesse, *Bequaert J. 4117*, 7 May 1914: lectotype, sheet here designated: BR[BR0000009826274]; isotypes: BR[BR0000009826601]; K[K000198773].

#### Type.

Angola. Malanje; Pungo Andongo, *Welwitsch F.M.J. 754*, Apr 1857: lectotype, sheet here designated: LISU[LISU206054]; isotypes: B[B 10 0153065]; BM[BM000554044]; COI[COI00004859]; K[K000198826]; P[P00046766]; LISU[LISU206055].

#### Description.

Liana, 2–6 m tall, d.b.h. unknown. Indumentum of mixed simple, fasciculate or stellate hairs; old leafless branches sparsely pubescent, **young foliate branches densely to sparsely pubescent**. Leaves: petiole 3–10 mm long, 2 mm in diameter, tomentose to very sparsely pubescent, cylindrical, blade inserted on the side of the petiole; blade 4–17 cm long, 2.5–7 cm wide, oblong to elliptic, apex acuminate, acumen 1–1.5 cm long, **base rounded to subcordate**, subcoriaceous, **below densely to sparsely pubescent** when young and old, above sparsely pubescent when young, sparsely pubescent to glabrous when old; midrib sunken or flat, above densely pubescent when young, densely to sparsely pubescent when old, below sparsely pubescent when young, densely to sparsely pubescent when old; secondary veins **6 to 12** pairs, pubescent above; **tertiary venation reticulate**. Individuals bisexual; inflorescences ramiflorous on young or old foliate branches, extra axillary. Flowers with 9 perianth parts in 3 whorls, 1 to 3 per inflorescence; pedicel 4–6 mm long, 1–2 mm in diameter, pubescent; in fruit 7–10 mm long, 2 mm in diameter, tomentose; bracts 2, one basal and one towards the lower half of pedicel, basal bract 1–2 mm long, 1 mm wide; upper bract 2–3 mm long, 4–5 mm wide; **sepals 3, valvate, completely fused, tearing at anthesis**, 5–9 mm long, 4–7 mm wide, ovate, apex truncate, base truncate, green to yellow, tomentose outside, tomentose inside, margins flat; petals free, sub equal; outer petals 3, 12–20 mm long, 8–11 mm wide, ovate to obovate, apex obtuse, base truncate, green to yellow, margins flat, pubescent outside, pubescent inside; inner petals 3, imbricate, 12–19 mm long, 8–11 mm wide, obovate to ovate, apex rounded, base truncate, green to yellow, margins flat, pubescent outside, tomentose inside; stamens 190 to 210, in 8 to 9 rows, 3–5 mm long, linear; **connective tongue shaped**, pubescent, yellow to orange; staminodes absent; carpels free, 25 to 35, ovary 4–5 mm long, stigma bilobed, slightly capitate, glabrous. Monocarps stipitate, **stipes 8–12 mm long**, 2–5 mm in diameter, **inserted laterally**; monocarps ca. 20, 13–40 mm long, 6–20 mm in diameter, **cylindrical**, apex apiculate, **tomentose, smooth** and slightly constricted around the seeds, brown turning red when ripe; seeds 6 to 16 per monocarp, ca. 10 mm long, 4–5 mm in diameter, ellipsoid; aril absent.

#### Distribution.

A widespread tropical African species, occurring from Sierra Leone to Central African Republic, Sudan and Ethiopia, and from Kenya to Zambia, one of the few Annonaceae species with such a wide continental distribution (Couvreur et al. 2008a); in Cameroon known from the Adamaoua, Central, East, North-west, South and West regions.

#### Habitat.

A common species in Cameroon; mainly occurring in gallery forests in drier regions of the country but also in rain forests. Altitude 500–1400 m a.s.l.

#### Local and common names known in Cameroon.

None recorded.

#### IUCN conservation status.

Least Concern (LC) (Botanic Gardens Conservation International and IUCN SSC Global Tree Specialist Group 2019c).

#### Uses in Cameroon.

None reported.

#### Notes.

﻿﻿﻿*Uvariaangolensis* belongs to a complex of species characterized by a calyx completely fused in bud and tearing into three distinct sepals at anthesis (Verdcourt 1971b). In Cameroon, other species of ﻿*Uvaria* with that character are ﻿*U.baumannii*, ﻿*U.chamae*, and ﻿*U.osmantha*. These species are all quite close morphologically (although *U.baumanii* and ﻿*U.osmantha* are quite different) but can be differentiated by some distinctive characters in particular fruit shape (see Key). ﻿﻿﻿*Uvariaangolensis* is particularly close to ﻿*U.versicolor* (not in Cameroon), but is distinguished by the connective of the stamens being tongue shaped versus discoid in ﻿*U.versicolor*. ﻿﻿﻿*Uvariaangolensis* is also quite variable in its indumentum varying from densely pubescent to glabrous.

#### Specimens examined.

**Adamaoua Region**: Réserve forestière de Ngaoundéré, 7.32°N, 13.58°E, *21 July 1977*, *Fotius G.* 2738 (P,YA); Beleldibi (35 km au SSE de Ngaoundéré), 7.32°N, 13.58°E, *22 July 1966*, *Letouzey R.* 7486 (P,YA); Pentes NO de l’Hoséré Banyo entre 1100 et 1400 m, 6.75°N, 11.82°E, *09 June 1967*, *Letouzey R.* 8565 (L,YA). **Central Region**: Yaoundé Mt Eloumden path from foot of mountain (ca 800 m) on Mendong side, 3.81°N, 11.43°E, *02 May 1996*, *Cheek M.* 8307 (K,WAG,YA); Pentes orientales du mont Yangba (1473 m) près Nyafianga (42 km NNE de Bafia), 5.13°N, 11.35°E, *09 September 1966*, *Letouzey R.* 7829 (P,YA); Près Nyafianga à 46 km SW de Linté, 5.13°N, 11.35°E, *23 April 1982*, *Nkongmeneck B.A.* 310 (P,YA). **East Region**: Kumbe, 6°N, 14.30°E, *01 January 1914*, *Mildbraed G.W.J.* 9224 (COI,K). **North Region**: Wakwa, 7.23°N, 13.58°E, *07 October 1960*, *Breteler F.J.* 432 (WAG); Near craterlake ‘Lac Tison’ ca 12 km SE of Ngaoundéré, 7.25°N, 13.58°E, *30 November 1964*, *de Wilde W.J.J.O* 4361 (WAG). **South Region**: Rocher de Ako’okas 26 km southeast of Ebolowa, 2.7°N, 11.28°E, *21 February 1987*, *Huber H.F.J.* 982 (YA). **West Region**: ca. 6 km NE of Bangangte, 5.15°N, 10.52°E, *11 May 1964*, *de Wilde J.J.F.E* 2579 (B,BR,K,MO,P,WAG,YA); Mme Vilatte plantations caOca 5 km from Foumbot, 5.55°N, 10.61°E, *07 July 1972*, *Leeuwenberg A.J.M.* 10160 (BR,K,MO,P,WAG,YA).

### ﻿﻿﻿﻿ Uvaria anisotricha

Taxon classificationPlantaeMagnolialesAnnonaceae

(Le Thomas) Couvreur, comb. et stat. nov.

6F14A6C7-0A6E-5A47-975E-B25378BD0523

urn:lsid:ipni.org:names:77305098-1

Figs 103 , 108 ; Map 13B

≡ Uvaria poggei var. anisotricha Le Thomas, Adansonia, ser. 2, 8, 2: 247, 1968.

#### Type.

Gabon. Ogooué-Ivindo; Bélinga, *Hallé N. & Le Thomas A. 484*, 14 Aug 1966: holotype: P[P00362742].

#### Description.

Liana, 5–20 m tall, d.b.h. unknown. Indumentum of stellate hairs; old leafless branches sparsely pubescent, **young foliate branches tomentose**. Leaves: petiole 2–4 mm long, 1–2 mm in diameter, tomentose, grooved, blade inserted on top of the petiole; blade 7–12 cm long, 3–5 cm wide, ovate, oblong or elliptic, apex acuminate, acumen 1–1.5 cm long, **base rounded to subcordate**, coriaceous, **below covered with a continuous persistent layer of small stellate hairs intermingled with larger stellate hairs**, above glabrous when young and old; midrib sunken or flat, **above densely pubescent** when young and old, below densely pubescent when young and old; secondary veins **18 to 20** pairs, glabrous above; **tertiary venation percurrent**. Individuals bisexual; inflorescences ramiflorous on young foliate branches or less often on old leafless branches, extra axillary. Flowers with 9 perianth parts in 3 whorls, 1 per inflorescence; pedicel 10–20 mm long, **2–3 mm in diameter**, tomentose; in fruit unknown; bracts 2, one basal and one towards the lower half of pedicel, basal bract 1–2 mm long, 1 mm wide, soon falling; upper bract unknown (soon falling?); sepals 3, valvate, **covering in bud, free**, 8–10 mm long, 8–10 mm wide, triangular, apex acute, base truncate, green to yellow, densely pubescent outside, densely pubescent inside, margins reflexed; petals free, sub equal; outer petals 3, 17–20 mm long, 10–15 mm wide, elliptic to suborbicular, apex obtuse to rounded, base truncate, light yellow, margins flat, pubescent outside, sparsely pubescent inside; inner petals 3, imbricate, 17–20 mm long, 10–15 mm wide, elliptic, apex obtuse to rounded, base truncate, light yellow, margins flat, tomentose outside, sparsely pubescent inside; stamens 150 to 200, in 8 to 9 rows, 2–3 mm long, oblong; connective discoid, sparsely pubescent; staminodes absent; carpels free, 27 to 32, ovary 4–5 mm long, stigma coiled, pubescent. Fruits unknown.

**Figure 102.**
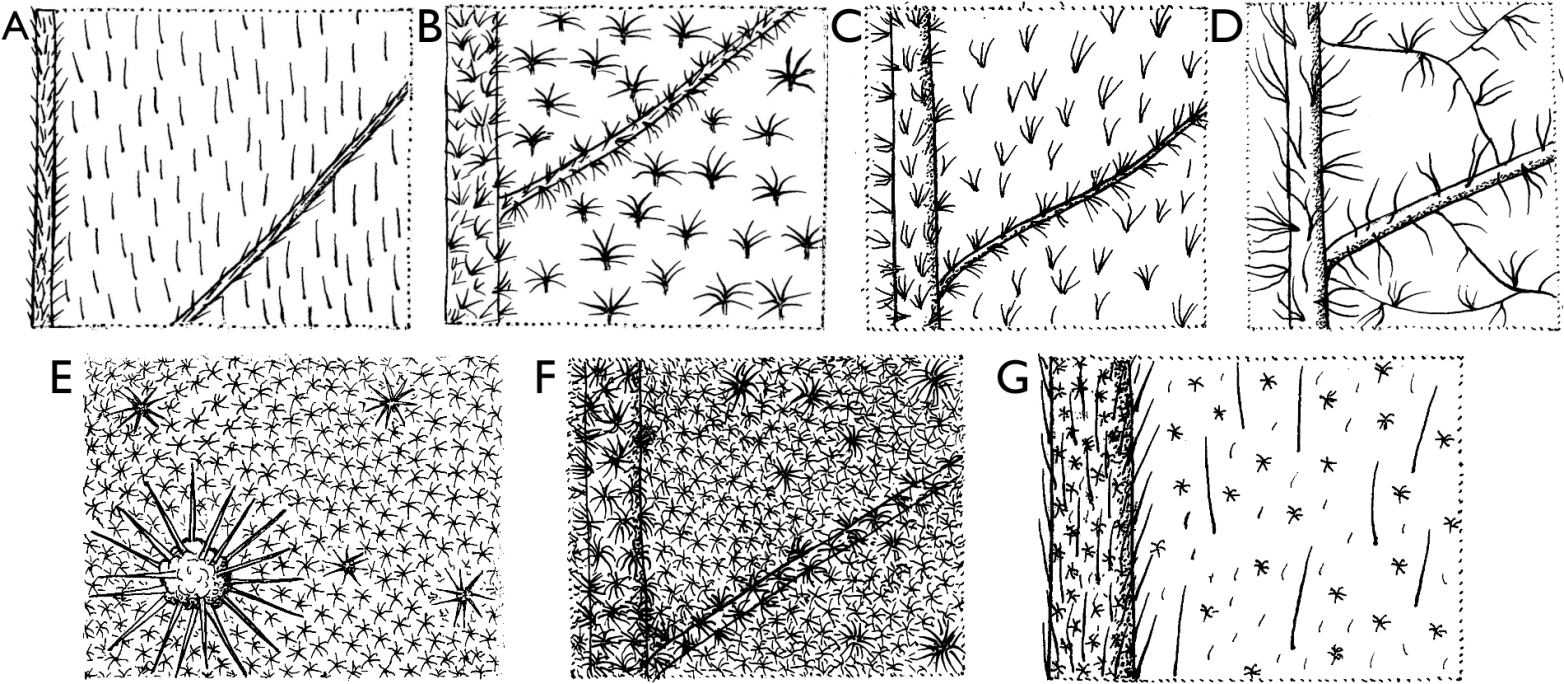
Different type of hairs on lower side of leaf blades in Cameroonian *Uvaria***A** simple **B** stipitate stellate **C** short fasciculate **D** long fasciculate **E, F** minute stellate mixed with larger stellate **G** simple mixed with minute stellate. Drawings by Hélène Lamourdedieu, Publications Scientifiques du Muséum national d’Histoire naturelle, Paris; modified from Le Thomas (1969b, pl. 6, p. 51; pl. 8, p. 57; pl. 9, p. 63; pl. 13, p. 77).

#### Distribution.

A central African species, known from Gabon, Cameroon and Democratic Republic of Congo; in Cameroon only known from the South West region.

#### Habitat.

An uncommon species in Cameroon; mainly occurring in submontane or montane rain forests. Altitude 800–1300 m a.s.l.

#### Local and common names known in Cameroon.

None recorded.

#### IUCN conservation status.

Not evaluated.

**Figure 103.**
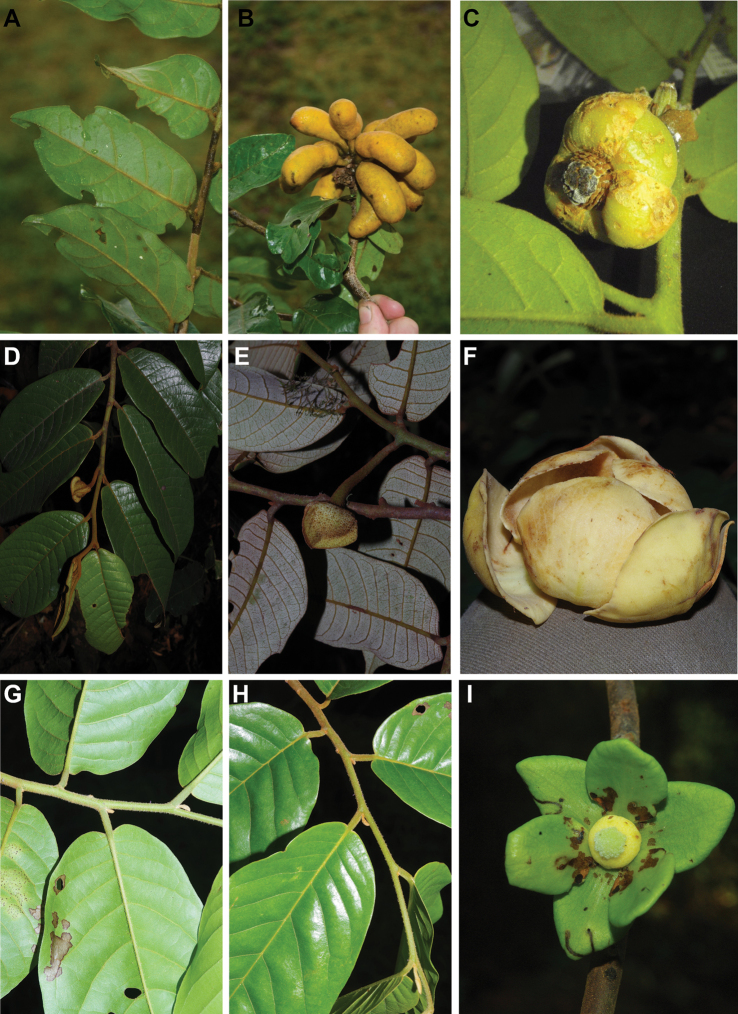
*Uvariaangolensis***A** lower side of foliate branch **B** fruits **C** flower, top view, note reflexed petals and tongue shape stamen connectives. *Uvariaanisotricha***D** leaves, top view with a flower **E** close up of flower bud, note reflexed sepal margins **F** petals of flower (fallen on ground). *Uvariacomperei* (1/2) **G** leaf base, lower side **H** leaf base, upper side **I** flower **A, B***Bidault 3284*, Guinea **C***Mas 1301*, Guinea **D–F***Couvreur 959*, Rumpi Montains, Cameroon **G–I***Couvreur 893*, Gabon. Photos **A, B** Ehoarn Bidault. Tropicos.org, Missouri Botanical Garden **C** Cyrille Mas. Tropicos.org, Missouri Botanical Garden **D–I** Thomas L.P. Couvreur.

#### Uses in Cameroon.

None reported.

#### Notes.

﻿﻿﻿*Uvariaanisotricha* was first described under the name ﻿﻿﻿Uvariapoggeivar.anisotricha by Le Thomas (1968b) distinguishing it from var. ﻿﻿﻿poggei by the presence of two layers of stellate hairs on the lower side of the leaf blades, one minute and another larger. Besides this character, and after close examination, we can add the following differences suggesting the status of species rather than simply a variety: In ﻿*U.anisotricha* the leaves are coriaceous, strongly discolorous, with two types of hairs on lower surface: short buff hairs fully covering the lower leaf surface mixed with sparser longer rufous hairs; and the flowering pedicels are thick (2–3 mm). In ﻿*U.poggei* the leaves are papyraceous, concolourous, with hairs all similar, sparse and not obscuring the surface and the flowering pedicels thin (1 mm). ﻿﻿﻿*Uvariapoggei* is also found in Cameroon but is restricted to the East region towards the border with the Central African Republic.

﻿﻿﻿*Uvariaanisotricha* belongs to a group of species in ﻿*Uvaria* with numerous secondary veins, generally more than 18 pairs and up to 25, whereas the rest of Cameroonian ﻿*Uvaria* have between 6 and 16 pairs of secondary veins. It resembles ﻿*U.baumannii* in this respect in addition to the presence of two layers of stellate hairs on the lower surface of the leaf blades, but differs by the flower buds being pyramidal in shape with the sepals free and reflexed at maturity, versus sepals completely fused and cylindrical in bud in ﻿*U.baumannii* (Le Thomas 1968b, 1969b). Pyramidal flower buds with reflexed margins are also found in other species such as ﻿*U.poggei* and ﻿*U.klainei*, the latter only known from Gabon and differing by its larger leaves glabrous above (except for the midrib) and with a single layer of stipitate stellate hairs below and larger flowers.

Cheek and Cable (1998, p. 12) tentatively named *Tekwe 49* as ﻿*U.poggei*, but this specimen is ﻿*U.anisotricha*.

#### Specimens examined.

**South-West Region**: Monkey forest descending to waterfall at Ndip, 5°N, 9.7°E, *20 January 1998*, *Cheek M.* 8950 (K,YA); Rumpi mountains forest trail ca 5 km after Dikome Balue village ca 40 km north of Kumba, 4.93°N, 9.239°E, *10 January 2016*, *Couvreur T.L.P.* 959 (WAG,YA); Kodmin, 5°N, 9.666°E, *16 November 1998*, *Gosline W.G.* 147 (K,WAG,YA); Above Upper Baondo, 4.06°N, 9.15°E, *07 April 1992*, *Tekwe C.F.* 49 (K,P,YA).

### ﻿﻿﻿﻿ Uvaria anonoides

Taxon classificationPlantaeMagnolialesAnnonaceae

Baker f., Cat. Pl. Oban: 2, 1913

969AF01D-A840-57ED-8A7D-0EDACDB482B4

Fig. 105 ; Map 13C

= Uvaria platyphylla Boutique, Fl. Congo Belge & Ruanda-Urundi ii: 296, 1951; ﻿﻿Annonalatifolia Scott Elliot; J. Linn. Soc., Bot. 30: 69, 1895; ﻿﻿Uvarialatifolia (Scott Elliot) Engl. & Diels (*non* Blume, Fl. Javae Anon., vol. 2, 37, 1828), Monogr. Afrik. Pflanzen.-Fam. 6: 22, 1901. Type. Sierra Leone. Northern Province, near Kafogo in Limba country, *Scott Elliot C.F. 5617*, 06 Apr 1892: lectotype, here designed: K[K000198787]; isolectotype: B[B 10 0153108]

#### Type.

Nigeria. Cross River State; Oban, *Talbot P.A. 1558*, 1912: lectotype, sheet here designated: K[K000198786]; isotypes: K[K000198786].

#### Description.

Liana, unknown height, d.b.h. unknown. Indumentum of stellate, minute hairs; old leafless branches sparsely pubescent, young foliate branches tomentose. Leaves: petiole 4–8 mm long, 1–2 mm in diameter, tomentose, grooved, blade inserted on top of the petiole; blade 7–25 cm long, 5–13 cm wide, obovate to oblong or elliptic, apex acuminate, acumen 1.5–2 cm long, **base rounded to cordate**, coriaceous, below densely pubescent when young, pubescent to sparsely pubescent when old, above densely pubescent quickly becoming glabrous when young, glabrous when old; midrib sunken or flat, **above densely pubescent**, at least towards the base when young and old, below densely pubescent when young and old; secondary veins **10 to 15** pairs, pubescent to glabrous above; **tertiary venation percurrent**. Individuals bisexual; inflorescences ramiflorous on young foliate branches and less often on old leafless branches, leaf opposed or extra axillary. Flowers with 9 perianth parts in 3 whorls, 1 to 2 per inflorescence; pedicel 15–22 mm long, 1–2 mm in diameter, tomentose; in fruit 25–30 mm long, 3–4 mm in diameter, pubescent; bracts 2, one basal and one towards the lower half of pedicel, basal bract not seen (soon falling?); upper bract 2–4 mm long, 3–4 mm wide; sepals 3, valvate, **fused almost completely, but not tearing**, 4–5 mm long, 8–10 mm wide, suborbicular, apex rounded, base truncate, densely pubescent outside, densely pubescent inside, margins flat; petals free, sub equal; outer petals 3, 17–20 mm long, 10–15 mm wide, ovate to oblong, apex rounded, base truncate, margins flat, tomentose outside, densely pubescent inside; inner petals 3, imbricate, 17–20 mm long, 10–15 mm wide, ovate to oblong, apex rounded, base truncate, margins flat, tomentose outside, densely pubescent inside; stamens 150 to 200, in 6 to 7 rows, 1–2 mm long, oblong; connective discoid, sparsely pubescent; staminodes absent; carpels free, 20 to 30, ovary 4–5 mm long, stigma coiled, densely pubescent. Monocarps stipitate, **stipes 20–30 mm** long, 2–3 mm in diameter, centrally inserted; monocarps 8 to 12, **20–22 mm long, 20–22 mm in diameter**, **globose**, apex rounded, **tomentose, verrucose to shortly echinate**, not ribbed; seeds not seen.

#### Distribution.

A West and Central African species, known from Sierra Leone to Nigeria and Cameroon with a disjunct distribution in eastern Democratic Republic of the Congo; in Cameroon only known from the East and South West regions.

#### Habitat.

An uncommon species in Cameroon; mainly occurring in lowland rain forests. Altitude 600–800 m a.s.l.

#### Local and common names known in Cameroon.

None recorded.

#### IUCN conservation status.

Not evaluated.

#### Uses in Cameroon.

None reported.

#### Notes.

﻿﻿﻿*Uvariaanonoides* is characterized by tomentose young branches, obovate leaves with a clearly cordate or rounded base and relatively few secondary veins (less than 16) and sepals almost completely fused but not enclosing the bud and not tearing apart at anthesis. In leaves longer than 5 cm the shape can vary from obovate to oblong or elliptical and with a larger cordate leaf base. ﻿﻿﻿*Uvariaanonoides* resembles ﻿*U.obanensis* in the shape and size of the leaves, but this latter species is almost glabrous, sepals are free and the monocarps are sessile and smooth.

﻿﻿﻿*Uvariaanonoides* was first described by Baker (1913) who stated it to be morphologically similar to ﻿*U.platyphylla* (see below) but differing mainly by leaf size (more than 15 cm long in ﻿*U.platyphylla* versus less than 15 cm in ﻿*U.anonoides*) a character (in addition to monocarp pubescence) also used by Le Thomas (1969b) in her key to the genus in the *Flore du Gabon* (although neither species occurs there). This distinction was also adopted by Hawthorne and Jongkind (2006). However, besides leaf size, there is no reliable distinguishing character between these two taxa, and we consider them as synonyms following previous authors (Hutchinson and Dalziel 1936; Lebrun and Stork 1991). ﻿﻿﻿*Uvariaanonoides* is also close morphologically to *U.mocoli*De Wild. & T. Durand (not found in Cameroon to date) by the shape (obovate) and size of its leaves, as suggested by Le Thomas (1969b). *U.mocoli* however has sessile monocarps.

The name ﻿﻿*Uvarialatifolia* was already published by Blume (1828) (﻿*U.latifolia* (Dunal) Blume) and thus Boutique (1951b, p. 256) provided a new name: ﻿*U.platyphylla* Boutique, which is younger than ﻿*U.anonoides*.

﻿﻿﻿Uvariaplatyphyllavar.luluensis Engl. & Diels (under ﻿﻿U.latifoliavar.luluensis) was also suggested to be synonym with ﻿*U.anonoides* but we have to disagree. After examination of the type (*Pogge 636* (B)) there are a number of differences: young foliate branches, petioles and leaf blades are glabrous or very shortly and sparsely pubescent, the leaf base is acute to decurrent (versus cordate to rounded), and the sepals are free (versus clearly fused), suggesting this is quite different from ﻿*U.anonoides*. Rather, this taxon could belong to the ﻿*U.muricata* complex, possibly conspecific with ﻿﻿U.muricatavar.suaveolens (not in Cameoon; O. Lachenaud, pers. com.).

#### Specimens examined.

**East Region**: 60 km south of Yokadouma 5 km south of Maséa village, 3.10°N, 14.84°E, *06 March 2019*, *Couvreur T.L.P.* 1208 (MPU,WAG,YA). **South-West Region**: Munyenge “trouble” village between liwenyi and Bonja, 4.41°N, 9.083°E, *20 March 1993*, *Tchouto Mbatchou G.P.* 548 (K,YA).

### ﻿﻿﻿﻿ Uvaria baumannii

Taxon classificationPlantaeMagnolialesAnnonaceae

Engl. & Diels, Notizbl. Königl. Bot. Gart. Berlin 2: 294, 1899

0450DF0A-2C6B-570F-BDC9-BA3F45835444

Fig. 104 ; Map 13D

≡ Uva baumannii (Engl. & Diels) Kuntze, Kuntze, Deutsche Bot. Monatsschr. 21: 173, 1903.
= Uvaria verrucosa Eng. & Diels, Notizbl. Königl. Bot. Gart. Berlin 2: 294, 1899 (*non* Scheff. Ann. Jard. Bot. Buitenzorg 2: 3, 1885). 10; ﻿﻿Uvariaengleriana (Engl. & Diels) Exell, J. Bot. 73 (Suppl.): 4, 1935. Type. Democratic Republic of the Congo. Haut-Uélé, “Kambele”, Schweinfurth G.A. 3683, 19 Apr 1870: holotype: B[B 10 0153069]; isotype: K[K000198771].

#### Type.

Togo. Plateaux; Misahöhe, *Baumann E. 527*, 19 Apr 1895: holotype: B[B 10 0153070].

#### Description.

Scrambling shrub to liana, 5–m tall, d.b.h. 6 cm. Indumentum of small stellate hairs; old leafless branches pubescent to glabrous, young foliate branches tomentose to densely pubescent. Leaves: petiole 2–4 mm long, 1–2 mm in diameter, pubescent, cylindrical, blade inserted on top of the petiole; blade 4.5–19 cm long, 2.5–6.5 cm wide, oblong to obovate, apex acuminate, acumen 1–1.5 cm long, base rounded to subcordate, papyraceous to subcoriaceous, **below covered with a persistent continuous layer of small stellate hairs intermingled with sparser larger stellate hairs, whitish**, above sparsely pubescent with stellate hairs when young, sparsely pubescent with stellate hairs to glabrous when old; midrib sunken or flat, above densely pubescent when young and old, below densely pubescent when young and old; secondary veins **17 to 25 pairs**, pubescent above; tertiary venation indistinct or percurrent. Individuals bisexual; inflorescences ramiflorous on young foliate branches and less often on old leafless branches, leaf opposed or extra axillary. Flowers with 9 perianth parts in 3 whorls, 1 per inflorescence; pedicel 10–15 mm long, 1–2 mm in diameter, tomentose; in fruit 20–25 mm long, 2–3 mm in diameter, tomentose; bracts 2, one basal and one towards the lower half of pedicel, soon falling, basal bracts 1–2 mm long, 1 mm wide; upper bracts 2–3 mm long, 3–5 mm wide; sepals 3, valvate, **completely fused, covering in bud, tearing at anthesis**, 5–10 mm long, 8–10 mm wide, ovate, apex acuminate, base truncate, pubescent with two layers of stellate hairs outside, pubescent with a single layer of minute stellate hairs inside, margins flat; petals free, sub equal; outer petals 3, 12–20 mm long, 9–15 mm wide, ovate, apex rounded, base truncate, yellow, margins flat, tomentose outside, tomentose inside; inner petals 3, imbricate, 12–20 mm long, 10–18 mm wide, ovate, apex rounded, base ungulate, yellow, margins flat, tomentose outside, tomentose inside; stamens 100 to 130, in 5 to 6 rows, 2–3 mm long, linear; connective discoid, pubescent; staminodes absent; carpels free, 15 to 20, ovary ca. 6 mm long, stigma conical, pubescent. Monocarps stipitate, **stipes 30–40 mm long**, 2–3 mm in diameter, **inserted laterally**; monocarps 30 to 48, 6–12 mm long, 6–10 mm in diameter, **globose**, apex generally shortly apiculate, **red-tomentose, warty to verrucose**, not ribbed, bluish-green when ripe; seeds 4 to 6 per monocarp, 8–10 mm long, 4–5 mm in diameter, flattened ellipsoid; aril absent.

**Figure 104.**
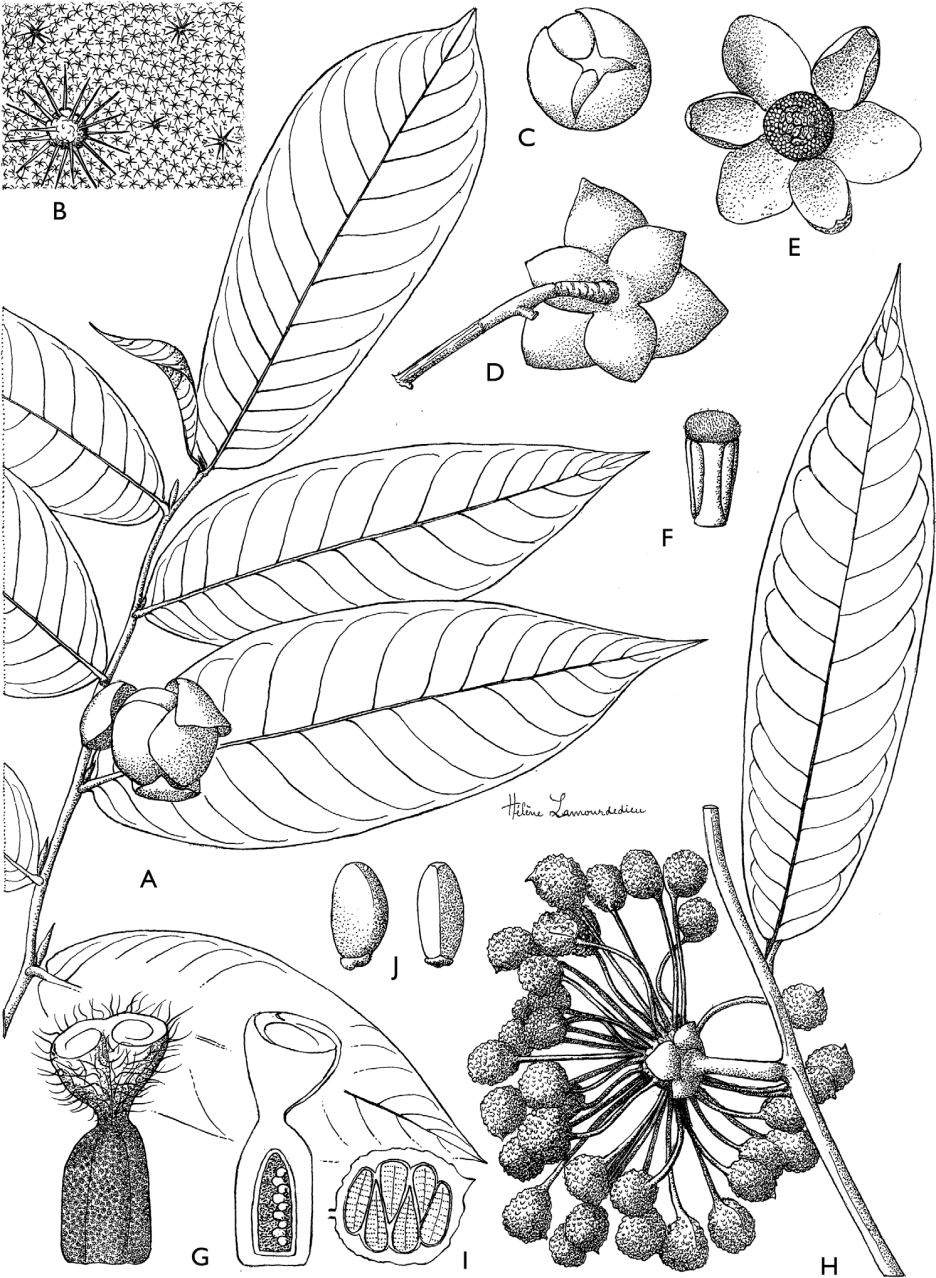
*Uvariabaumannii***A** flowering branch **B** detail of lower side of leaf, note the minute stellate hairs mixed with very large stellate hairs **C** flower bud, top view, note spepals tearing when flower opening **D** flower, bottom view, note sepals three, after flower opened **E** flower, top view **F** stamen **G** carpel, front view and detail of ovules **H** fruit branch **I** longitudinal section of monocarp **J** seeds **A, B** from *Le Testu 1421***C–G** from *Le Testu 8334***H–J** from *Hallé & Le Thomas 11*. Drawings by Hélène Lamourdedieu, Publications Scientifiques du Muséum national d’Histoire naturelle, Paris; modified from Le Thomas (1969b, pl. 9, p. 63).

#### Distribution.

A widely distributed species in West and Central Africa, from Sierra Leone to Benin and from Cameroon to the Democratic Republic of the Congo; in Cameroon known from the East, South and South West (?) regions (see notes).

#### Habitat.

An uncommon species in Cameroon; in swampy regions on sand soils. Altitude 400–750 m a.s.l.

#### Local and common names known in Cameroon.

None recorded.

#### IUCN conservation status.

Not evaluated.

#### Uses in Cameroon.

None reported.

#### Notes.

﻿﻿﻿*Uvariabaumannii* is characterized by having the lower side of the leaf blades completely covered with a layer of small minute stellate hairs together with a second layer of larger stellate hairs more sparsely dispersed (as in ﻿*U.anisotricha*, see notes under that species), sepals that are completely fused in bud and tearing at anthesis (see notes under ﻿*U.angolensis*), and long stipitate monocarps with stipes inserted laterally.

In Cameroon this species is restricted to the East and South regions. One collection (*Mukete W. 47*, K) was identified as ﻿*U.baumannii* from the South West region, but we were unable to see that specimen, and highly doubt it is correct (could probably be ﻿*U.anisotricha*).

**Figure 105.**
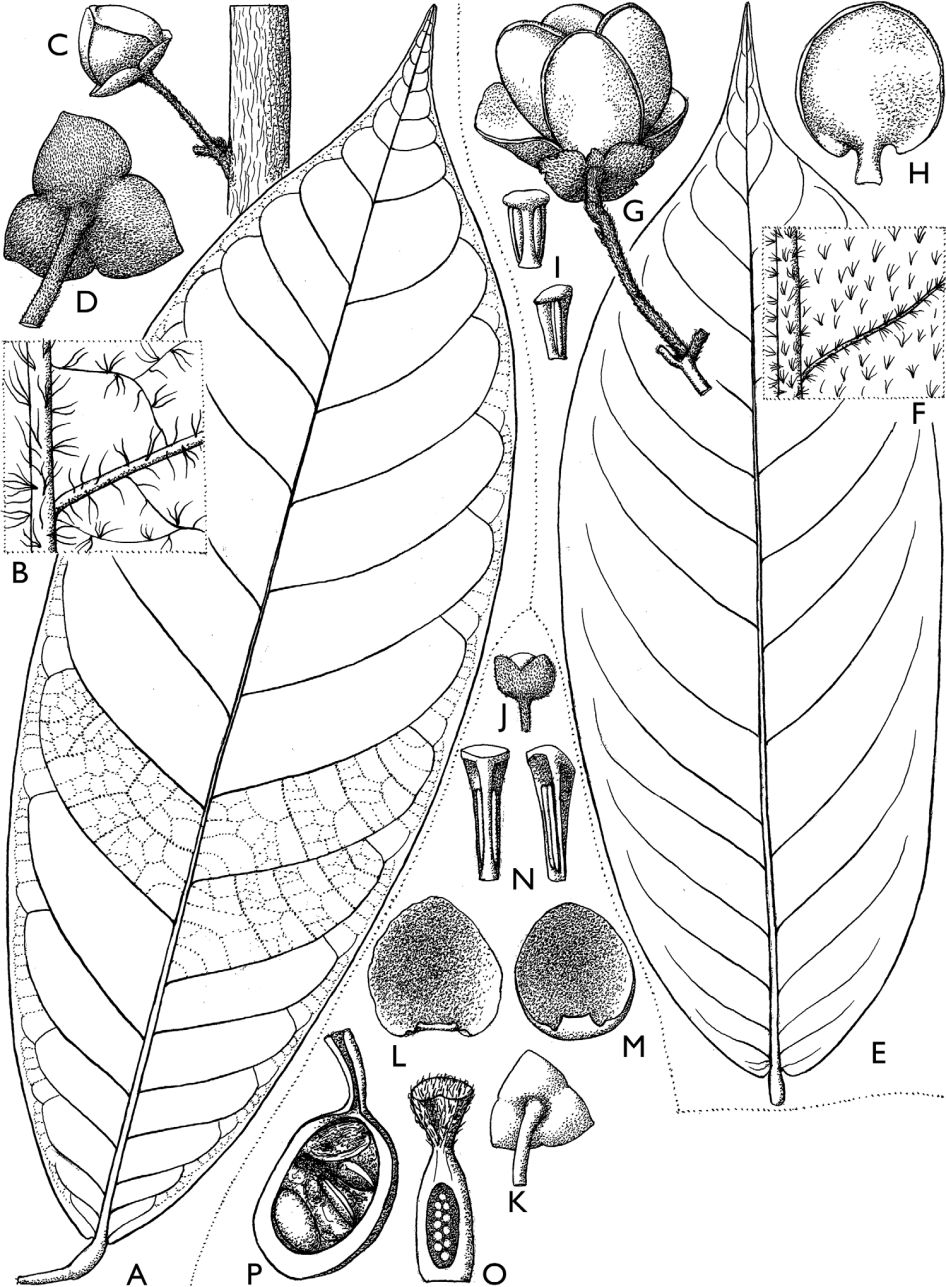
*Uvariabipindensis***A** leaf, upper view **B** detail of pubescence lower side of leaf blade **C** flower, note cauliflorous nature **D** flower, bottom view. *Uvariamollis***E** leaf, lower side **F** detail of pubescence, lower side of leaf blade **G** flower **H** inner petal, note ungulate base **I** stamen, side and front views. *Uvariaanonoides***J** flower bud **K** detail of calyx, bottom view **L** outer petal, inner view **M** inner petal, inner view **N** stamen, front and side views **O** carpel, detail of ovules **P** longitudinal section of monocarp **A–D** from *Brenan 9445***E–I** from *Zenker & Staudt 3***J–O***Jaeger 9876***P** from *Jaeger 8800*. Drawings by Hélène Lamourdedieu, Publications Scientifiques du Muséum national d’Histoire naturelle, Paris.

#### Specimens examined.

**East Region**: A 14 km de Ntan (80 km ENE de Lomie), 2.7°N, 15.13°E, *09 August 1963*, *Letouzey R.* 5584 (P,YA). **South Region**: Campo-Ma’an area Ipono, 2.33°N, 9.841°E, *18 April 2002*, *Elad M.* 1535 (KRIBI,WAG); Campo-Ma’an area Mamelles Massif, 2.55°N, 9.944°E, *23 April 2001*, *Tchouto Mbatchou G.P.* 3238 (KRIBI,WAG); Campo-Ma’an area Bibabimvoto, 2.28°N, 9.950°E, *16 August 2002*, *Tchouto Mbatchou G.P.* 3404 (KRIBI,WAG).

### ﻿﻿﻿﻿ Uvaria bipindensis

Taxon classificationPlantaeMagnolialesAnnonaceae

Engl., Notizbl. Königl. Bot. Gart. Berlin 2: 292, 1899

BF2463D3-4E65-51A1-BD6C-9B193599C2CF

**Figure 106.**
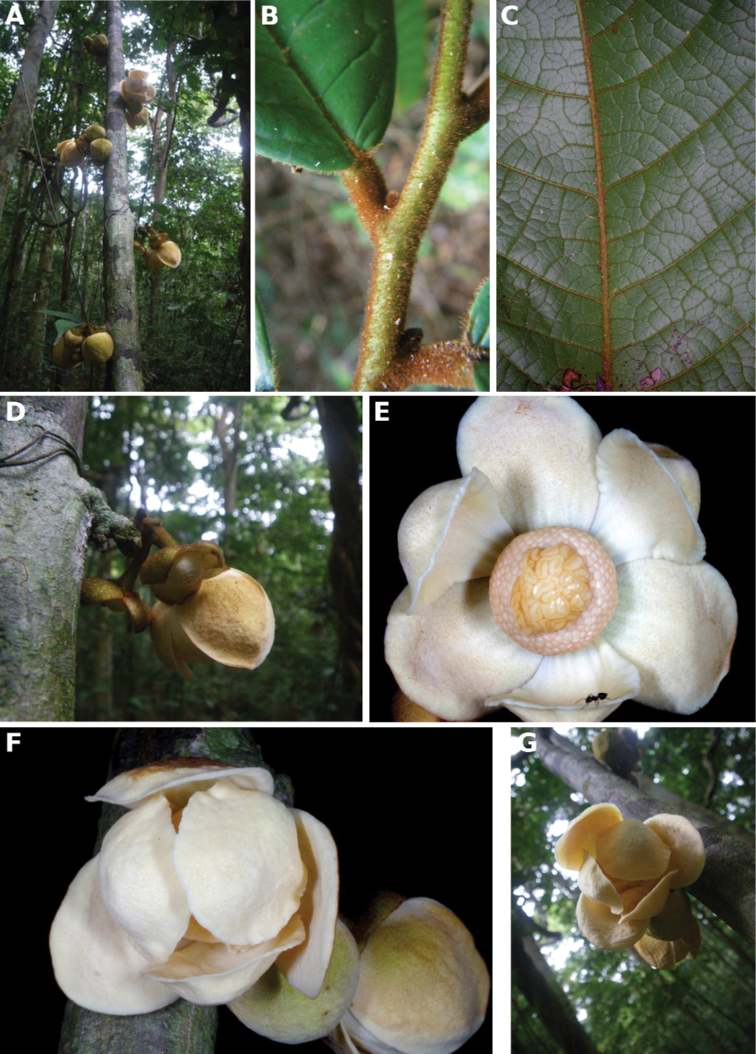
*Uvariabipindensis***A** stem showing flowers **B** young branch with basal part of upper leaf blade **C** detail of lower side of leaf blade, note some percurrent veins, but also some network like **D** detail of cauliflorous flower, side view **E** flower, top view, petals opened for photo **F** flower at anthesis, side view **G** flower, bottom view **A–G***Lachenaud 1979*, Gabon; Photos **A–F** Dietrich I. Lafferty **G** Olivier Lachenaud; both from Tropicos.org, Missouri Botanical Garden.

Figs 105 , 106 ; Map 13E

≡ Uva bipindensis (Engl.) Kuntze, Deutsche Bot. Monatsschr. 21: 173, 1903.
= Uvaria cardiophylla Engl. & Diels, Monogr. Afrik. Pflanzen.-Fam. 6: 13, 1901. Type. Cameroon. South Region, Grand Batanga, *Dinklage M.J. 834*, 22 Oct 1890: holotype: B[B 10 0153071]; isotype: HBG[HBG502501].

#### Type.

Cameroon. South Region; Bipindi, *Zenker G.A. 1116*, 1 Jul 1896: holotype: B[B 10 0153072]; isotypes: E[E00147945]; K[K000198775]; P[P00046767]; WU[WU0025884].

#### Description.

Liana, 5–20 m tall, d.b.h. unknown. Indumentum of fasciculate hairs; old leafless **branches hirsute becoming glabrous, young foliate branches hirsute**. Leaves: petiole (2)4–5 mm long, 2 mm in diameter, densely pubescent, slightly grooved, blade inserted on top of the petiole; blade 9–27 cm long, 4–11 cm wide, obovate to elliptic, apex acuminate, acumen 1.5–2 cm long, **base cordate to acute or subcordate to rounded**, papyraceous, below sparsely hirsute with fasciculate hairs when young and old, above glabrous when young and old; midrib sunken or flat, **above densely pubescent with simple hairs** when young, pubescent to glabrous when old, below densely pubescent when young, pubescent when old; secondary veins **13 to 20** pairs, pubescent above; **tertiary venation percurrent (but some venation network like)**. Individuals bisexual; **inflorescences cauliflorous or occasionally on young foliate branches**, axillary. Flowers with 9 perianth parts in 3 whorls, 1 to 5 per inflorescence; pedicel 10–20 mm long, 1–2 mm in diameter, densely pubescent to tomentose; in fruit unknown; bracts 2, one basal and one towards the upper half of pedicel, basal bract not seen (soon falling?); upper bract 2–3 mm long, 3–5 mm wide; sepals 3, valvate, **free**, 7–8 mm long, 10–11 mm wide, suborbicular, apex obtuse, base truncate, brown tomentose outside, densely pubescent inside, margins flat, green-brown; petals free, sub equal; outer petals 3, **16–20 mm long, 15–16 mm wide**, ovate to suborbicular, apex obtuse, base truncate, cream white to cream yellow, margins flat, tomentose outside, densely pubescent towards margins, glabrous towards center inside; inner petals 3, imbricate, 10–12 mm long, 10–18 mm wide, elliptic to oblong, apex obtuse, base narrowed, cream white to cream yellow, margins flat, tomentose outside, glabrous inside; stamens 150 to 200, in 5 to 7 rows, ca. 4 mm long, linear; connective discoid, glabrous, white; staminodes absent; carpels free, 15 to 25, ovary ca. 4 mm long, stigma coiled, glabrous. **Monocarps stipitate**, **stipes 15–20 mm long**, 3–4 mm in diameter, **laterally inserted**; monocarps 8 to 13, 20–70 mm long, 20–40 mm in diameter, **cylindrical to oblong**, apex rounded, **brown tomentose, 4 to 5 ribbed**, otherwise smooth, orange-brown when ripe; seeds 5 to 12 per monocarp, ca. 20 mm long, ca. 14 mm in diameter, ellipsoid; aril absent.

#### Distribution.

A central African species, from Cameroon to Gabon and Equatorial Guinea (recently collected in those latter two countries); in Cameroon known from the Littoral, South and South-West regions.

#### Habitat.

A locally common species when present; occurring in primary rain forest on drained or swampy regions of sand soils, often in sandy areas. Altitude 0–400 m a.s.l.

#### Local and common names known in Cameroon.

None recorded.

#### IUCN conservation status.

Not evaluated.

#### Uses in Cameroon.

None reported.

#### Notes.

﻿﻿﻿*Uvariabipindensis* can be distinguished by its hirsute indumentum on the young foliate branches, petioles, sepals and petals and its large leaves (15–22 cm) with a clearly cordate or subcordate base. The flowers are cauliflorous, the only species in Cameroon with this character reported to date. The fruits were described for the first time by Lachenaud (2018), and here taken from two specimens (*Reits J.M. 1865* (WAG); *Carvalho M. 6047* (MA)) collected in Gabon and Equatorial Guinea respectively. The monocarps are very characteristic for Cameroonian ﻿*Uvaria* being stipitate oblong to cylindrical in shape having 4 to 5 marked ribs arising from the base. These fruits resemble those of ﻿*U.chamae*, ﻿*U.angolensis* or ﻿*U.versicolor* (not in Cameroon) in the length of the stipes (medium), but in the latter three the monocarps are smaller and narrower and lack ribs.

This species was thought to be endemic to Cameroon, but has now been collected in Gabon several times (e.g. *Bidault 1686*, *1739*; *Couvreur 1092, 521*; *Lachenaud 1979*; *Reitsma 1865*) and Equatorial Guinea (*Carvalho 6047*).

#### Specimens examined.

**Littoral Region**: Mapubi 30 km before Edea on Yaoundé-Edea road On forestry road 5 km direction to Sanaga river, 3.84°N, 10.38°E, *28 February 2018*, *Couvreur T.L.P.* 1179 (K,MPU,WAG,YA); Mambe Massif above Boga village 100 km along road from Yaoundé to Ed 3.90°N, 10.77°E, *20 June 2014*, *Couvreur T.L.P.* 662 (WAG,YA); Au SE du Lac Tisongo (35 km SW Edéa), 3.53°N, 9.909°E, *09 January 1974*, *Letouzey R.* 12660 (P,YA). **South Region**: 20 km from Kribi N of Lolodorf road (SFIA logging road), 3°N, 10.05°E, *10 June 1969*, *Bos J.J.* 4794 (WAG); Campo Ma’an National Park 11 km on trail from Ebinanemeyong village on road 7 km from Nyabessan to Campo town, 2.48°N, 10.33°E, *11 February 2015*, *Couvreur T.L.P.* 673 (WAG,YA); Campo-Ma’an area Onoyong, 2.52°N, 10.69°E, *18 March 2001*, *Tchouto Mbatchou G.P.* ONOX_53 (WAG); Bipindi, 3.08°N, 10.42°E, *1896*, *Zenker G.A.* 1116 (K,P). **South-West Region**: Jocteh Andie-Muen, 5.10°N, 9.716°E, *09 November 2001*, *Etuge M.* 4504 (K).

### ﻿﻿﻿﻿ Uvaria buchholzii

Taxon classificationPlantaeMagnolialesAnnonaceae

Engl. & Diels, Notizbl. Königl. Bot. Gart. Berlin 2: 295, 1899

2E820654-B649-5A88-9CE4-1135C6994BEE

Fig. 107 ; Map 13F

≡ Uva buchholzii (Engl. & Diels) Kuntze, Kuntze, Deutsche Bot. Monatsschr. 21: 173, 1903; Balongabuchholzii (Engl. & Diels) Le Thomas, Adansonia ser. 2, 8, 1: 108, 1968.

#### Type.

Cameroon. West Region; Balong, *Buchholz R.W. 103*, 26 Jun 1874: holotype: B[B 10 0154065].

#### Description.

Scrambling shrub or liana, height unknown, d.b.h. to 25 cm in diameter. Indumentum of simple or fasciculate hairs; old leafless branches glabrous, young foliate branches pubescent becoming quickly glabrous. Leaves: petiole 5–9 mm long, ca. 1 mm in diameter, glabrous, grooved, blade inserted on top of the petiole; blade 10–24 cm long, 3.5–9 cm wide, obovate to oblong, apex acuminate, acumen ca. 1 cm long, base obtuse to subcordate, papyraceous, below sparsely pubescent when young, sparsely pubescent to glabrous when old, above glabrous when young and old; midrib sunken or flat, above glabrous when young and old, below sparsely pubescent when young, glabrous when old; secondary veins 10 to 14 pairs, glabrous above; tertiary venation reticulate. Individuals bisexual; inflorescences ramiflorous on old leafless branches, leaf opposed or extra axillary. Flowers with 9 perianth parts in 3 whorls, 1 per inflorescence; pedicel 20–25 mm long, 1–2 mm in diameter, pubescent; in fruit 25–27 mm long, 2–3 mm in diameter, glabrous; bracts 2, one basal and one upper towards the upper half of pedicel, basal bract 1–2 mm long, 1 mm wide; upper bract 4–6 mm long, 3–5 mm wide; sepals 3, **imbricate**, free, 6–8 mm long, 6–8 mm wide, suborbicular, apex rounded, base truncate, pubescent outside, glabrous inside, margins flat; petals free, inner slight longer than outer; outer petals 3, 10–15 mm long, 7–10 mm wide, ovate to oblong, apex acute, base truncate, margins revolute, pubescent outside, pubescent inside; inner petals 3, imbricate, 20–22 mm long, 14–17 mm wide, obovate, apex rounded, base ungulate, margins flat, pubescent outside, glabrous inside; stamens numerous, in 10 rows, 1–2 mm long, narrowly oblong; connective discoid, glabrous; staminodes absent; carpels free, numerous, ovary ca. 3 mm long, stigma obpyramidal, glabrous. Monocarps stipitate, **stipes 20–30 mm long, laterally inserted**, 1–2 mm in diameter; monocarps 30 to 40, 12–15 mm long, 10–15 mm in diameter, ellipsoid, apex apiculate or rounded, pubescent, smooth, **irregularly ribbed with two main prominent ribs**, greyish green turning orange when ripe; **seed 1 (more rarely 2)** per monocarp, 8–10 mm long, 5–6 mm in diameter, flattened ellipsoid; aril absent.

#### Distribution.

A central African species, from Cameroon to Gabon; in Cameroon known from the East, South, South West and West regions.

#### Habitat.

An uncommon and rarely collected species but is suggested to be frequent when present; in rain forests near rocky outcrops. Altitude 100–800(1000) m a.s.l.

#### Local and common names known in Cameroon.

None recorded.

#### IUCN conservation status.

Not evaluated.

#### Uses in Cameroon.

None reported.

**Figure 107.**
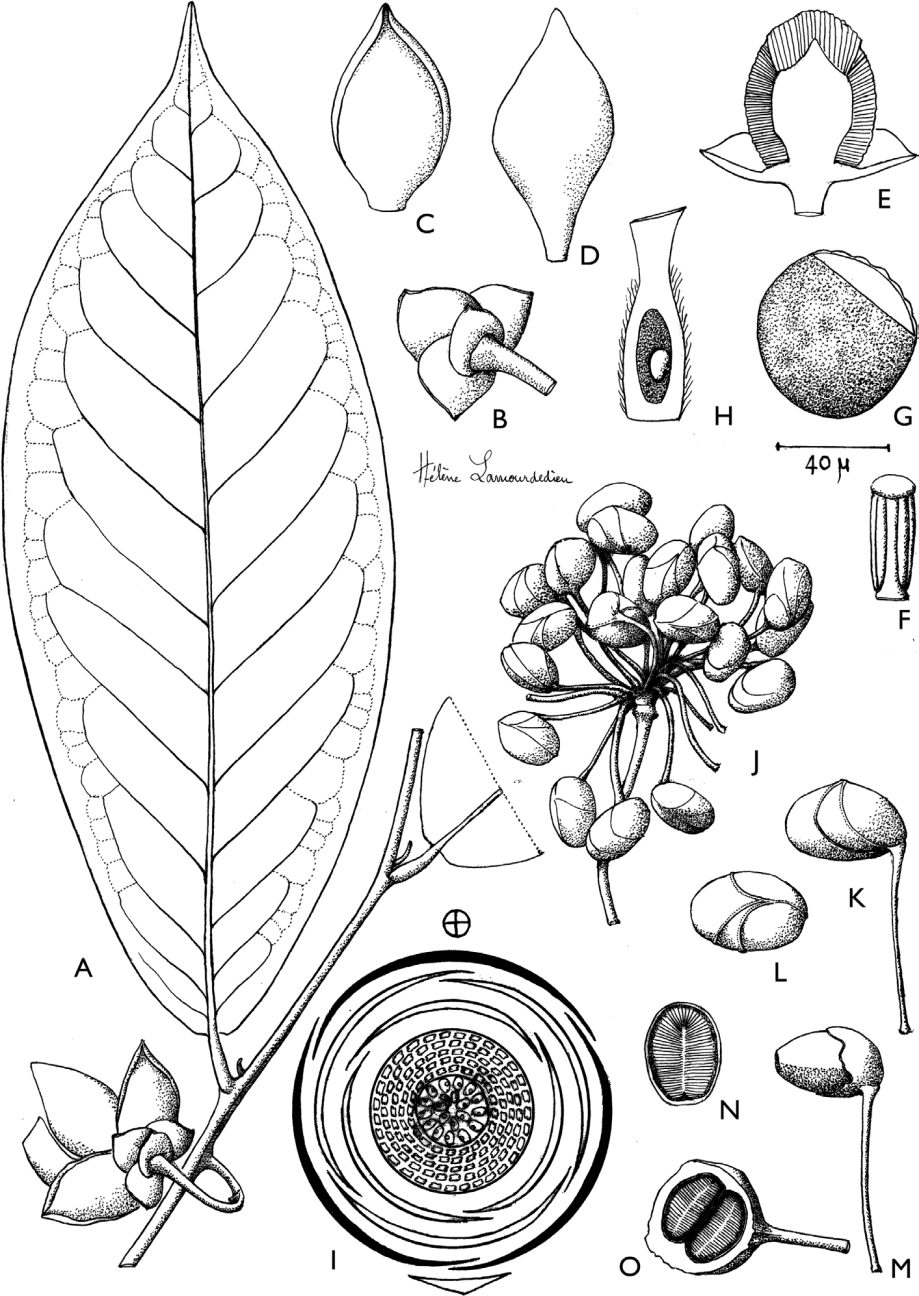
*Uvariabuchholzii***A** flowering branch **B** detail of lower side of flower, showing imbricate sepals and upper bract **C** outer petal, inner view **D** inner petal, inner view **E** longitudinal section of flower (petals removed) showing strongly convex receptacle **F** stamen **G** pollen grain **H** longitudinal section in carpel showing ovule **I** flower diagram **J** fruit **K** detail of stipitate monocarp showing ridges **L** detail of monocarp showing ridges **M** detail of monocarp showing different structure **N** longitudinal section of seed; longitudinal section of monocarp **A–H** from *Zenker 4926***J–N** from *Klaine 2658* bis **O** from *Bulchholz 103*. Drawings by Hélène Lamourdedieu, Publications Scientifiques du Muséum national d’Histoire naturelle, Paris; modified from Le Thomas (1969b, pl. 4, p. 35).

#### Notes.

﻿﻿﻿*Uvariabuchholzii* is mainly distinguished by its imbricate sepals and its 2-ribbed monocarps with few seeds (1 to 2) and laterally inserted stipes. ﻿﻿﻿*Uvariabuchholzii* superficially resembles ﻿*U.welwitschii* Engl. & Diels (not present in Cameroon to date) in the overall leaf morphology, but the latter has smooth monocarps with centrally inserted stipes.

The imbricate aestivation of the sepals led Le Thomas (1968a) to place this species into a new genus, *Balonga* Le Thomas. However, recent molecular data found this species nested within ﻿*Uvaria* and so the name was transferred back into ﻿*Uvaria* (Zhou et al. 2010).

Le Thomas suggested that this species might be a small tree or a shrub (Le Thomas 1968a, 1969b) which would be unusual for ﻿*Uvaria* (generally lianas). However, a collection by Letouzey (*12190*, P) indicates it is a scrambling shrub (“arbuste sarmenteux”) which is a more common habit state for ﻿*Uvaria*.

#### Specimens examined.

**East Region**: Rocher Ekok Edanbawa à 110 km au SW de Yokadouma 2.81°N, 14.48°E, *29 March 1973*, *Letouzey R.* 12190 (P,WAG,YA). **South Region**: Bipindi, 3.08°N, 10.42°E, *01 January 1913*, *Zenker G.A.* 4926 (L,P); Bipindi, 3.08°N, 10.41°E, *01 April 1914*, *Zenker G.A.* 597 (U,WAG). **South-West Region**: Limbe Mt Etinde above Mpanja, 4.08°N, 9.174°E, *11 July 1990*, *Cheek M.* 3014 (K).

### ﻿﻿﻿﻿ Uvaria chamae

Taxon classificationPlantaeMagnolialesAnnonaceae

P.Beauv., Fl. Oware 2: 43, 1816

D1094219-0B5E-56B8-AA16-038E559C91DD

Map 13G

≡ Uva chamae (P.Beauv.) Kuntze, Deutsche Bot. Monatsschr. 21: 173, 1903.
= Uvaria cylindrica Schumach., Beskr. Guin. Pl. 256, 1827. Type. Guinea. South Region, *Thonning P. 44*, no date: lectotype, sheet here designated: C[C10004675]; isotype: C[C10004674].
= Uvaria cristata R.Br. ex Oliv., Fl. Trop. Afr. 1: 23, 1868. Type. Sierra Leone. *Purdie W. s.n.*, no date: holotype: BM[BM000843983].
= Uvaria nigrescens Engl. & Diels, Monogr. Afrik. Pflanzen.-Fam. 6: 15, 1901. Type. Sierra Leone. Afzelius A. s.n., no date: holotype: B[B 10 0153102].
= Uvaria echinata A.Chev., Explor. Bot. Afrique Occ. Franc. I: 6, 1920: *nom. nud.*

#### Type.

Nigeria. no region; no locality, *Palisot de Beauvois A.M.F.J. s.n.*, no date: holotype: G[G00014882].

#### Description.

Scrambling shrub to liana, 3–10 m tall, d.b.h. 3–10 cm. Indumentum of minute stellate hairs; old leafless branches glabrous, **young foliate branches very sparsely pubescent to glabrous**. Leaves: petiole 4–10 mm long, ca. 1 mm in diameter, glabrous, grooved, blade inserted on the side of the petiole; blade 9–12 cm long, 4–6 cm wide, elliptic, apex acuminate, acumen 0.7–0.9 cm long, **base acute (obtuse)**, subcoriaceous, **below** sparsely pubescent to glabrous when young, glabrous when old, above glabrous or sparsely pubescent when young and old; midrib sunken or flat, above pubescent to glabrous when young and old, below glabrous when young and old; secondary veins **12 to 16** pairs, **not prominent above**, glabrous above; **tertiary venation reticulate**. Individuals bisexual; inflorescences ramiflorous on foliate branches, extra axillary or terminal. Flowers with 9 perianth parts in 3 whorls, 1 to 3 per inflorescence; pedicel 14–18 mm long, 1–2 mm in diameter, pubescent; in fruit 15–19 mm long, 2–3 mm in diameter, sparsely pubescent; bracts 2, one basal and one towards the lower half of pedicel, basal bract 2–4 mm long, 3–4 mm wide; upper bract 2–4 mm long, 3–4 mm wide; sepals 3, valvate, **completely fused**, tearing at anthesis, 9–12 mm long, 9–12 mm wide, ovate, apex obtuse or acute, base truncate, light green, densely pubescent outside, sparsely pubescent inside, margins flat; petals free, sub equal, **reflexed when opened**; outer petals 3, 10–17 mm long, 5–10 mm wide, ovate, apex rounded, base truncate, green to yellow, margins flat, densely pubescent outside, densely pubescent towards margins, glabrous towards center inside; inner petals 3, imbricate, 9–15 mm long, 5–10 mm wide, ovate, apex rounded, base unguiculate, green to yellow, margins flat, densely pubescent outside, densely pubescent towards margins and glabrous towards center inside; stamens 150 to 200, in 6 to 7 rows, 2–3 mm long, linear; connective discoid, pubescent, yellow to cream; staminodes absent; carpels free, 20 to 30, ovary ca. 4 mm long, stigma coiled, glabrous. Monocarps stipitate, **stipes 10–15 mm long**, 2–3 mm in diameter, **laterally inserted**; monocarps 10 to 20, **22–27 mm long, 10–13 mm in diameter**, **cylindrical**, apex rounded, **pubescent with small stellate hairs**, slightly constricted over seeds in dried material, otherwise smooth, brown turning orange when ripe; seeds 12 to 16 per monocarp, 8–10 mm long, 4–5 mm in diameter, flattened ellipsoid; aril absent.

#### Distribution.

A mainly west African species from Senegal to Cameroon, Central African Republic, and northern Democratic Republic of the Congo; in Cameroon known from the Central, Far-North, North and North-West regions.

#### Habitat.

Occurring in drier regions of the country; in dry forest, thickets and gallery forests. Altitude 100–800(1000) m a.s.l.

#### Local and common names known in Cameroon.

None recorded.

#### IUCN conservation status.

Least Concern (LC) (Botanic Gardens Conservation International and IUCN SSC Global Tree Specialist Group 2019c).

#### Uses in Cameroon.

None reported.

#### Notes.

﻿﻿﻿*Uvariachamae* is characterized by having (almost) glabrous leaf blades, secondary veins very weak (much more prominent in ﻿*U.angolensis*), with sepals completely fused in bud and tearing at anthesis (see notes under ﻿*U.angolensis*) and the petals clearly reflexed at anthesis. The monocarps are stipitate with the stipes shorter or as long as the monocarps which are cylindrical and pubescent. It is also one of the few species of ﻿*Uvaria* growing in the drier regions of Cameroon, together with e.g. ﻿﻿U.muricatavar.yalingensis.

#### Specimens examined.

**East Region**: Rives de la Kadei près de Moundia (15 km SSE de Batouri), 4.15°N, 14.61°E, *31 March 1962*, *Letouzey R.* 4625 (P,YA). **Far-North Region**: Ziver, 10.8°N, 13.77°E, *17 May 1974*, *Fotius G.* 1995 (P,WAG,YA); Mogode-Rhumsiki, 10.5°N, 13.58°E, *26 August 1976*, *Geerling C.* 5666 (WAG). **North Region**: Koro (Goré), 10.8°N, 13.77°E, *19 January 1946*, *Aubréville A.* 628 (P); Mango, 8.42°N, 13.25°E, *25 July 1974*, *Fotius G.* 2173 (P,YA); Parc National de la Bénoué Près du campement du buffle noir, 8.12°N, 13.83°E, *05 December 1995*, *Letouzey R.* 11695 (P,YA); Parc national de Faro Campement, 8.36°N, 12.81°E, *05 March 1985*, *van der Zon A.P.M.* 2542 (WAG); Benoué National Park near Buffle Noir Camp, 8.11°N, 13.83°E, *04 October 1974*, *Wit P.* 2955 (BR,MO,WAG). **North-West Region**: Piste Munka (=Munkep) 45 km NNW Wum, 6.73°N, 9.95°E, *09 July 1975*, *Letouzey R.* 13994 (K,P,YA); Munka (=Munkep) 45 km NNW Wum, 6.73°N, 9.95°E, *09 July 1975*, *Letouzey R.* 13999 (K,P,YA).

### ﻿﻿﻿﻿ Uvaria comperei

Taxon classificationPlantaeMagnolialesAnnonaceae

Le Thomas, Adansonia sér. 2, 8: 244, 1968

46C34BA4-F1F8-5770-8B1F-A83DEF40E5E5

Figs 103 , 109 , 110 ; Map 13H

#### Type.

Gabon. Ogooué-Ivindo; Belinga, *Hallé N. 3267*, 20 Nov 1964: holotype: P[P00046772].

#### Description.

Liana, 3–5 m tall, d.b.h. 3–5 cm. Indumentum of simple or fasciculate hairs; old leafless branches glabrous, **young foliate branches tomentose to pubescent**. Leaves: petiole 4–6 mm long, 1–2 mm in diameter, tomentose, grooved, blade inserted on the side of the petiole; blade 6–17 cm long, 3–6.5 cm wide, oblong, apex acuminate to obtuse, acumen 1 cm long, **base rounded to broadly cordate**, papyraceous, below pubescent with simple or fasciculate hairs when young and old, above **sparsely pubescent** with short simple and stellate hairs when young, glabrous when old; midrib sunken or flat, above sparsely pubescent when young and old, below pubescent when young and old; secondary veins **9 to 13** pairs, sparsely pubescent above; **tertiary venation reticulate**. Individuals bisexual; inflorescences ramiflorous on young foliate branches, extra axillary. Flowers with 9 perianth parts in 3 whorls, 1 per inflorescence; pedicel 6–11 mm long, 1–2 mm in diameter, pubescent; in fruit 11–16 mm long, 2–3 mm in diameter, sparsely pubescent; bracts 2, one basal and one towards the lower half of pedicel, basal bract 2–4 mm long, 3–4 mm wide; upper bract 2–4 mm long, 3–4 mm wide; sepals 3, valvate, basally fused, 2–3 mm long, 3–4 mm wide, ovate, apex acuminate to attenuate, base truncate, pale green, tomentose outside, glabrous inside, margins flat; petals free, sub equal; outer petals 3, 7–14 mm long, 6–10 mm wide, ovate to oblong, apex obtuse, base truncate, green to light yellow, margins shortly revolute or straight, tomentose outside, pubescent inside; inner petals 3, imbricate, 7–14 mm long, 6–10 mm wide, ovate, apex obtuse, base shortly unguiculate, green to light yellow, margins flat, tomentose outside, pubescent inside; stamens numerous, in 7 to 8 rows, ca. 2 mm long, linear; connective discoid, sparsely pubescent, yellow; staminodes absent; carpels free, numerous (not clounted), ovary ca. 4 mm long, stigma bilobed, densely pubescent. Monocarps stipitate, **stipes 17–22 mm long**, 1–2 mm in diameter, **laterally inserted**; monocarps 30 to 40, 10–15 mm long, 7–9 mm in diameter, ellipsoid to subglobose, apex rounded or very shortly apiculate, **sparsely pubescent**, **slightly constricted over seeds in dried material**, otherwise smooth, green to brown when ripe; seeds 10 to 14 per monocarp, 7–8 mm long, 4–5 mm in diameter, flattened ellipsoid; aril absent.

#### Distribution.

A central African species, known from Cameroon, Gabon and Democratic Republic of the Congo; in Cameroon known from the Littoral and South-West regions.

#### Habitat.

A rare species in Cameroon; in secondary rain forests. Altitude 0–100 m a.s.l.

#### Local and common names known in Cameroon.

None recorded.

#### IUCN conservation status.

Not evaluated.

#### Uses in Cameroon.

None reported.

#### Notes.

﻿﻿﻿*Uvariacomperei* belongs to a group of species characterized by having sepals mostly free or basally fused and long stipitate globose monocarps with laterally inserted stipes. In flower characters, ﻿*U.comperei* is very close to ﻿*U.gabonensis* (not found in Cameroon to date) and Le Thomas (1968b) distinguished the former mainly based on differences of the leaves which has a rounded to broadly cordate leaf base and a long petiole. In addition, in ﻿*U.gabonensis* the lower side of the leaves have short appressed stellate hairs and the monocarps are smooth and densely tomentose versus short appressed stellate hairs intermixed with some longer erect fasciculate hairs (the latter mostly near the midrib) and bumpy and sparsely pubescent in ﻿*U.comperei*.

#### Specimens examined.

**Littoral Region**: Lac Ossa Nord Dizangue, 3.84°N, 10.03°E, *Achoundong G.* 786 (YA); Lac Ossa Nord Dizangue, 3.84°N, 10.03°E, *Achoundong G.* 790 (YA). **South-West Region**: North Base, 4.01°N, 9.283°E, *24 April 1992*, *Tchouto Mbatchou G.P.* 167 (K,YA).

### ﻿﻿﻿﻿ Uvaria heterotricha

Taxon classificationPlantaeMagnolialesAnnonaceae

Pellegr., Bull. Soc. Bot. Fr. 96: 173, 1949

2D9F6ABD-C054-5AAA-B0E2-8004FFCB84BA

Fig. 108 ; Map 13I

#### Type.

Gabon. Woleu-Ntem; Oyem, *Le Testu G.M.P.C. 9481*, 2 Fev 1934: lectotype, sheet here designated: P[P00363327]; isotypes: BM[BM000554061]; BR[BR0000008823953, BR0000008823960]; LISC[LISC000396]; P[P00363326, P00363328].

#### Description.

Liana, 3–10 m tall, d.b.h. up to 20 cm. Indumentum of simple to stellate and fasciculate hairs; old leafless branches sparsely pubescent to glabrous, **young foliate branches densely pubescent, with small stellate hairs intermixed with longer simple hairs**. Leaves: petiole 2–3 mm long, 1–2 mm in diameter, tomentose, grooved, blade inserted on top of the petiole; blade 7–16 cm long, 3–6 cm wide, oblong to elliptic, apex acuminate to acute, acumen 1.5–2 cm long, **base rounded to subcordate**, subcoriaceous, below pubescent with short stellate hairs intermixed with long simple hairs when young, sparsely pubescent with short stellate hairs intermixed with long simple hairs when old, above glabrous when young and old; midrib sunken or flat, **above densely pubescent when young and old**, below pubescent when young and old; **secondary veins 10 to 13** pairs, pubescent above; **tertiary venation reticulate**. Individuals bisexual; inflorescences ramiflorous on young foliate branches, extra axillary. Flowers with 9 perianth parts in 3 whorls, 1 per inflorescence; pedicel 10–12 mm long, 1–2 mm in diameter, pubescent; in fruit 40–60 mm long, 3–4 mm in diameter, sparsely pubescent; bracts 2, one basal and one towards the lower half of pedicel, similar in size, 5–6 mm long, 3–4 mm wide; sepals 3, valvate, **completely fused**, **tearing at anthesis**, 5–10 mm long, 7–11 mm wide, triangular to ovate, apex acute, base truncate, yellow, pubescent outside, glabrous inside, margins flat; petals free, inner slightly longer than outer to sub equal; outer petals 3, 15–25 mm long, 12–20 mm wide, ovate to suborbicular, apex rounded, base truncate, yellow, with dark red-brown base, margins flat or revolute, tomentose outside, glabrous inside; inner petals 3, imbricate, 20–35 mm long, 10–22 mm wide, ovate to suborbicular, apex rounded, base unguiculate, claw 4–5 mm long, yellow with dark red-brown base, margins flat or revolute, tomentose outside, glabrous inside; stamens 300 to 320, in 8 to 10 rows, 2–3 mm long, narrowly oblong; connective discoid, pubescent; staminodes absent; carpels free, 40 to 50, ovary ca. 3 mm long, stigma coiled, densely pubescent. Monocarps stipitate, stipes 30–45 mm long, ca. 1 mm in diameter, **laterally inserted**; monocarps 42 to 46, 9–12 mm long, 7–8 mm in diameter, ellipsoid, apex apiculate or mucronate, **tomentose**, smooth, not ribbed or very faintly ribbed, green to orange when ripe; **seeds 1 to 2 per monocarp**, 7–8 mm long, 4–5 mm in diameter, flattened ellipsoid; aril absent.

**Figure 108.**
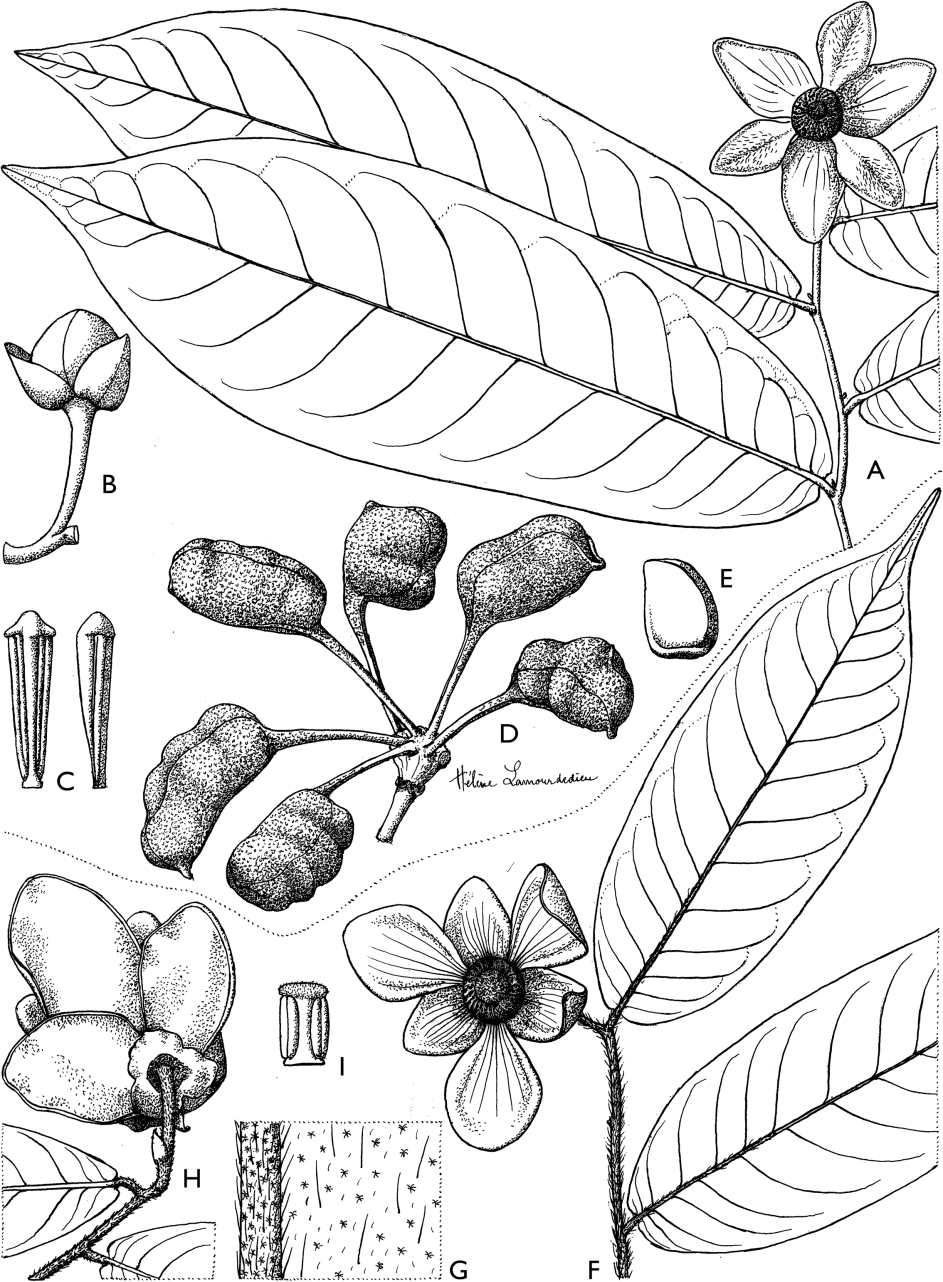
*Uvariaclavata* (not in Cameroon) **A** flowering branch **B** flower **C** stamen, side and front view **D** fruit **E** seed. *Uvariaheterotricha***F** flowering branch **G** detail of pubescence of lower side of leaf blade, note mix between stellate and simple hairs **H** flower, bottom view **I** stamen **A–E** from *Hallé & Villiers 5594***F***Le Testu 8610***G–I** from *Le Testu 9481*. Drawings by Hélène Lamourdedieu, Publications Scientifiques du Muséum national d’Histoire naturelle, Paris; modified from Le Thomas (1969b, pl. 6, p. 51).

**Figure 109.**
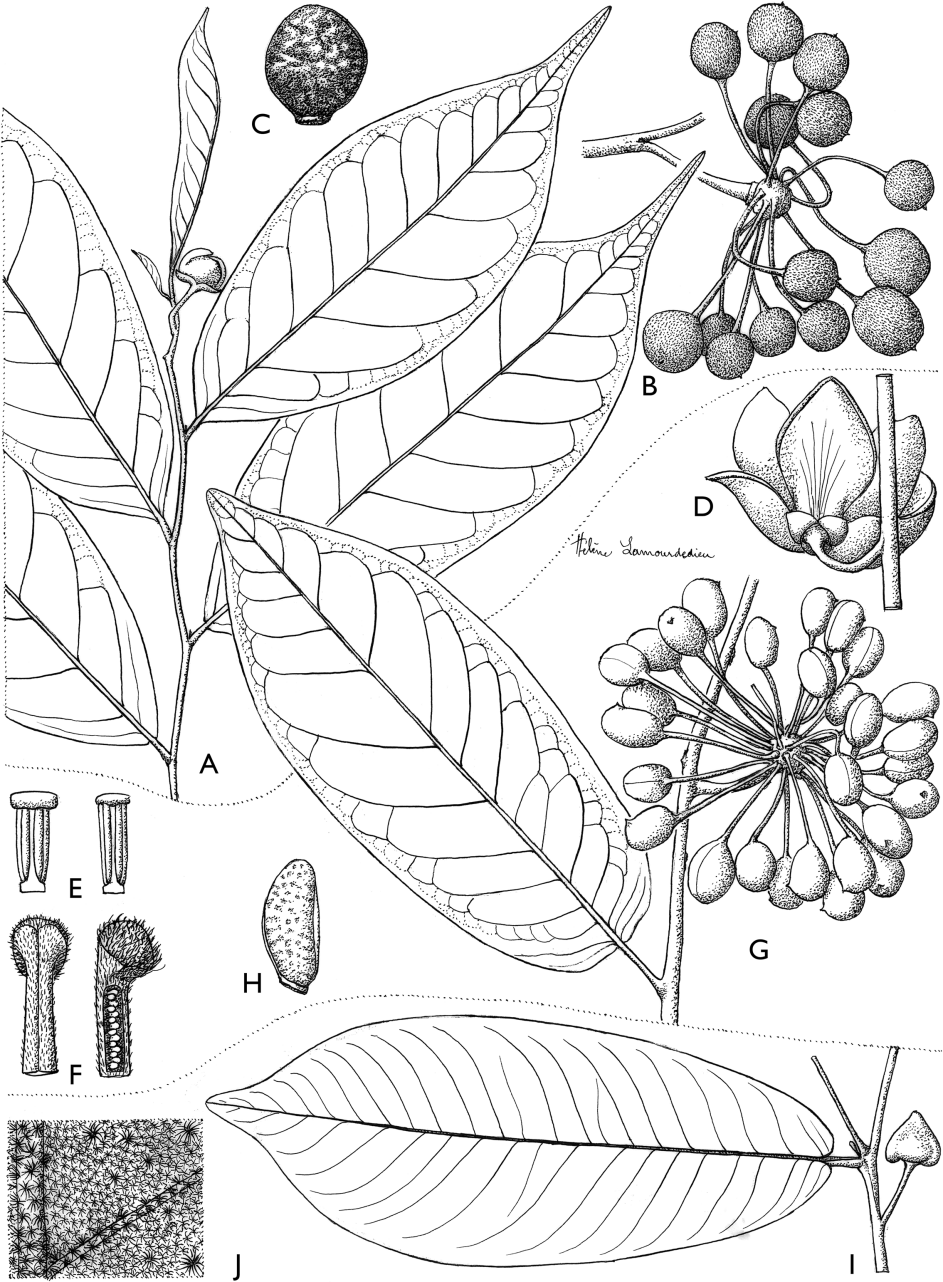
*Uvariagabonensis* (not in Cameroon) **A** flowering branch **B** fruit **C** seed. *Uvariacomperei***D** flower **E** stamen, different shapes **F** carpel, back view, and detail of ovules **G** fruiting branch **H** seed. *Uvariaanisotricha***I** leaf and flower bud **J** detail of pubescence on lower side of leaf, note minute stellate hairs mixed with large stellate hairs **A–C** from *Soyaux 217***D–F** from *Klaine 3070***G, H** from *Hallé 3267***I, J** from *Hallé & Le Thomas 484*. Drawings by Hélène Lamourdedieu, Publications Scientifiques du Muséum national d’Histoire naturelle, Paris; modified from Le Thomas (1969b, pl. 8, p. 57).

**Figure 110.**
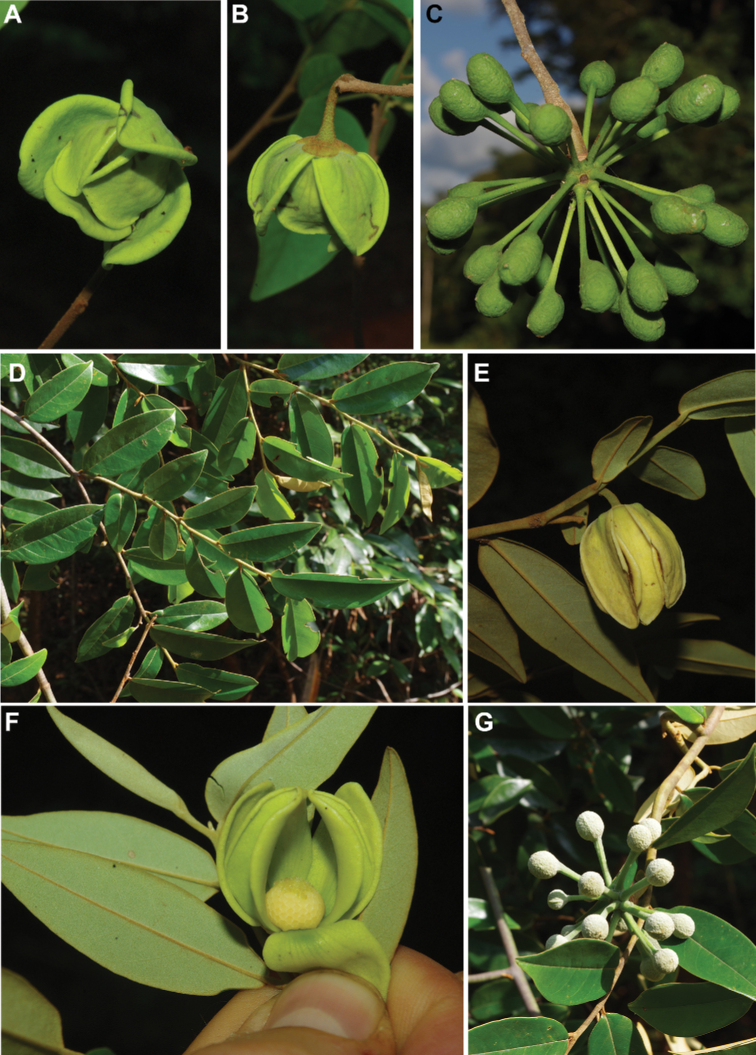
*Uvariacomperei* (2/2) **A** flower, top view **B** flower, side view, note free sepals **C** fruit with long stipitate monocarps. *Uvariaklaineana***D** branch **E** flower **F** detail of flower and receptacle **G** fruit **A, B** Couvreur 545 **C***Couvreur 893*, Gabon **D, G***Couvreur 919*, Gabon **E, F***Couvreur 546*, Gabon. Photos Thomas L.P. Couvreur.

#### Distribution.

A central African species, known from Cameroon and Gabon; in Cameroon known from the South West region.

#### Habitat.

A common species when present; in submontane or montane secondary or primary rain forests. Altitude 700–1400 m a.s.l.

#### Local and common names known in Cameroon.

None recorded.

#### IUCN conservation status.

Not evaluated.

#### Uses in Cameroon.

None reported.

#### Notes.

﻿﻿﻿*Uvariaheterotricha* is easily distinguished by having small stellate hairs intermixed with longer simple hairs on the young foliate branches and lower side of the leaves (use hand lens). Its monocarps are also densely tomentose.

This species was only known from Gabon (Le Thomas 1969b), but has since been collected several times (Cheek et al. 2004) in the South West province (e.g. *Cheek 7457*, *8863*; *Gosline 83*; *Letouzey 14586*). It is interesting that in Cameroon this species appears restricted to submontane or montane regions (above 700 m) near Mont Kupe or Rumpi Mountains (Cheek et al. 2004), whereas in Gabon it is found at lower altitudes (below 600 m).

#### Specimens examined.

**South-West Region**: Kupe village, 4.76°N, 9.694°E, *21 May 1996*, *Cable S.* 2500 (K,MO,P,WAG,YA); Kupe village, 4.78°N, 9.700°E, *28 May 1996*, *Cable S.* 2674 (K,YA); Mbule, 4.80°N, 9.673°E, *26 June 1996*, *Cable S.* 3344 (K,MO,WAG,YA); Nyasoso, 4.86°N, 9.7°E, *01 July 1996*, *Cable S.* 3459 (B,K,MO,P,WAG,YA); Second trail to summit along road to Mbulle from Nyasoso, 4.81°N, 9.681°E, *03 July 1996*, *Cable S.* 3558 (K,YA); Kupe village, 4.78°N, 9.716°E, *15 July 1996*, *Cable S.* 3809 (K,WAG,YA); Nyasoso, 4.83°N, 9.691°E, *23 October 1995*, *Cheek M.* 7457 (K,YA); Bakossi Mt Kodmin 05 km on road to Muawhojun, 4.98°N, 9.683°E, *17 January 1998*, *Cheek M.* 8863 (K,WAG,YA); Nyasoso, 4.83°N, 9.683°E, *26 March 1996*, *Etuge M.* 1819 (K,WAG); Nyasoso, 4.81°N, 9.683°E, *03 June 1996*, *Etuge M.* 2092 (K,WAG,YA); Kupe village, 4.77°N, 9.708°E, *08 July 1996*, *Etuge M.* 2659 (K,WAG,YA); Pool at summit of waterfall, 4.96°N, 9.716°E, *05 February 1998*, *Gosline W.G.* 83 (K,WAG,YA); Kupe village, 4.78°N, 9.716°E, *11 July 1996*, *Keay R.W.J.* 297 (K,YA); km 2 Essosong Estate-Kola N side of Mount Koupé, 4.85°N, 9.733°E, *20 January 1972*, *Leeuwenberg A.J.M.* 9249 (B,BR,K,MO,P,WAG,YA); Monts Rumpi entre Dikome Balue (1200 m) et Ifanga Nalende (650 m), 4.9°N, 9.233°E, *25 March 1976*, *Letouzey R.* 14586 (WAG,YA).

### ﻿﻿﻿﻿ Uvaria klaineana

Taxon classificationPlantaeMagnolialesAnnonaceae

Engl. & Diels, Notizbl. Königl. Bot. Gart. Berlin 2: 294, 1899

6561B5C7-F2F5-5E53-9135-DFC5E27E9820

**Figure 111.**
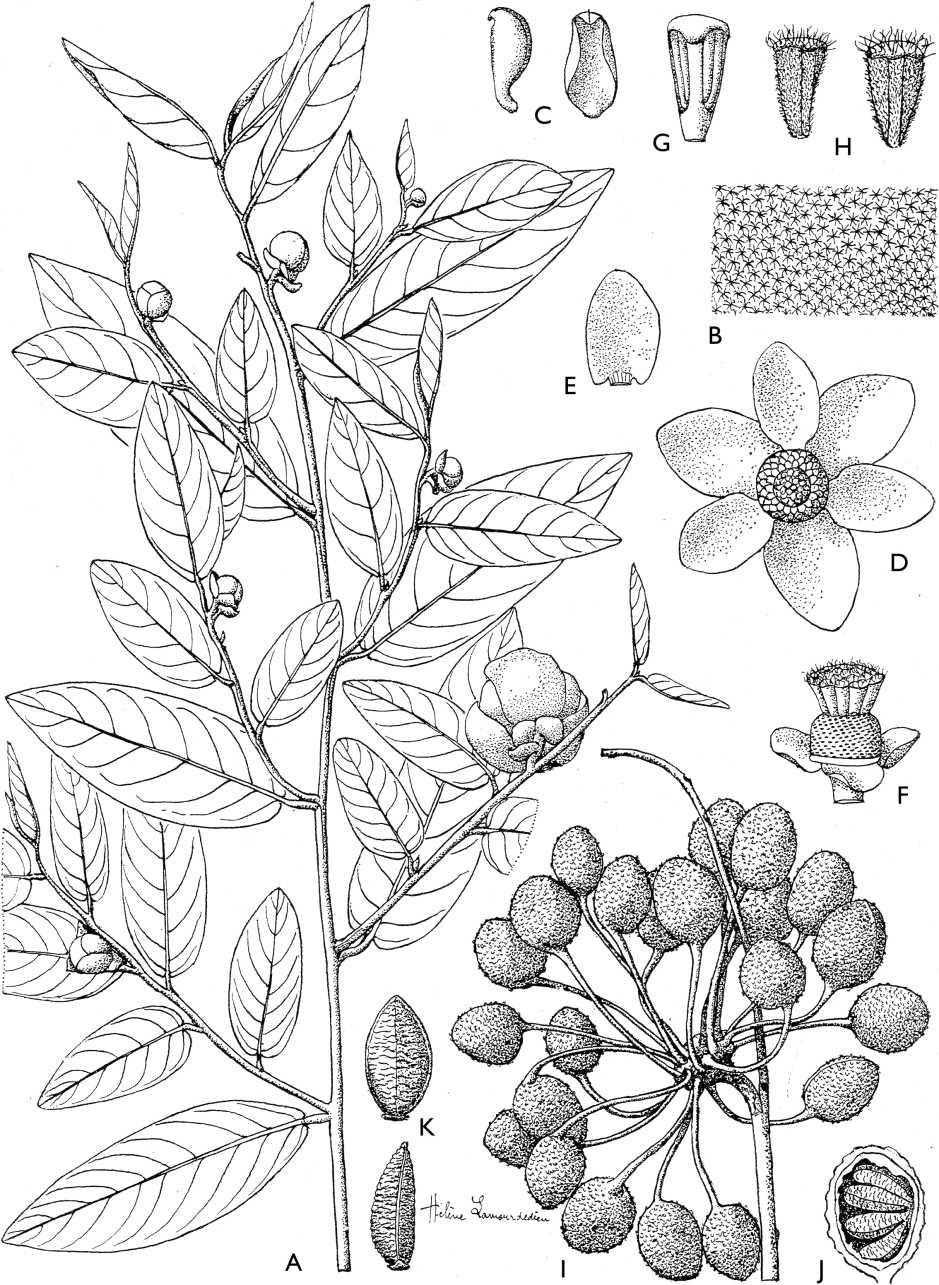
*Uvariaklaineana***A** flowering branch **B** detail of pubescence on lower side of leaf **C** upper bract, side and front views **D** flower, top view **E** outer petal, inner view **F** detail of flower, all petals removed **G** stamen **H** carpel, side and front views **I** fruit **J** longitudinal section of monocarp **K** seeds, side and front views **A–J** from *Klaine 235*. Drawings by Hélène Lamourdedieu, Publications Scientifiques du Muséum national d’Histoire naturelle, Paris; modified from Le Thomas (1969b, pl. 10, p. 65).

**Map 14.**
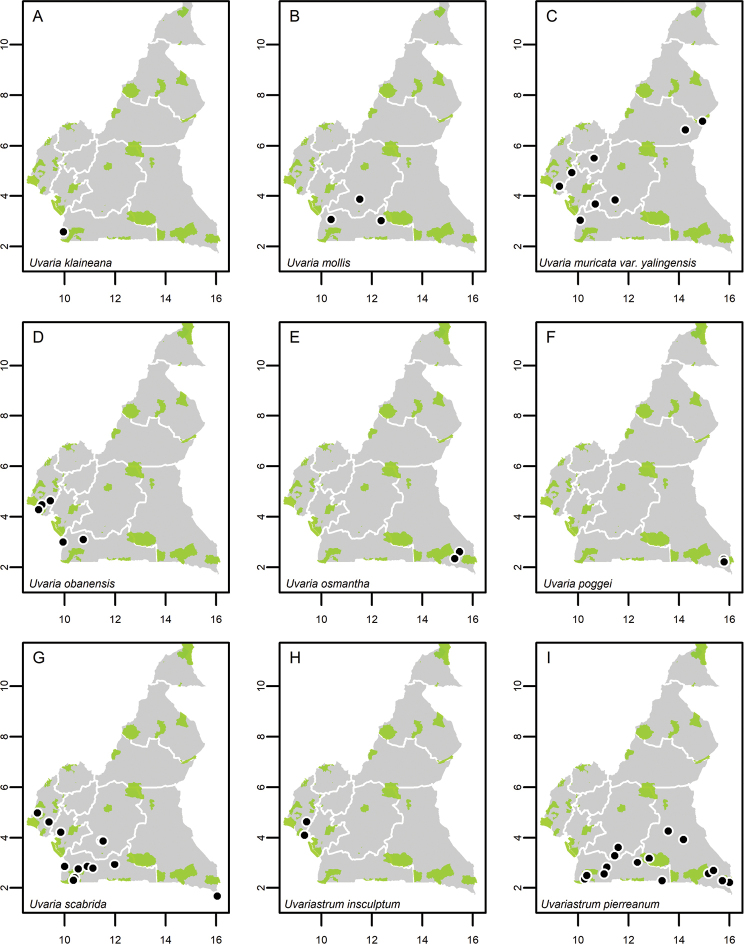
**A***Uvariaklaineana***B***Uvariamollis***C**Uvariamuricatavar.yalingensis**D***Uvariaobanensis***E***Uvariaosmantha***F***Uvariapoggei***G***Uvariascabrida***H***Uvariastruminsculptum***I***Uvariastrumpierreanum*. White borders represent region limits in Cameroon; green patches represent protected areas (see methods and Suppl. material 1: Fig. S1).

Figs 110 , 111 ; Map 14A

≡ Uva klaineana (Engl. & Diels) Kuntze, Kuntze, Deutsche Bot. Monatsschr. 21: 173, 1903.
= Uvaria klaineana var. chrysophylla Pellegr., Bull. Soc. Bot. Fr. Mém. 31: 60, 1949. Type. Gabon. Ngounié, Moucongo, *Le Testu G.M.P.C. 6337*, 20 Oct 1926: lectotype, here designated: BM[BM000554053]; isolectotypes: EA[EA000002481, EA000002480]; MO[MO-247263]; LISC[LISC000398].

#### Type.

Gabon. Estuaire; Libreville, *Klaine T.-J. 235*, 14 Sep 1896: lectotype, sheet here designated: P[P00362663]; isotypes: B[B 10 0153078]; P[P00362707, P00362706, P00362710, P00362712, P00362714, P00362709, P00362708, P00362662].

#### Description.

Scrambling shrub to liana, 3–20(30) m tall, d.b.h. unknown; stilt roots or buttresses absent. Indumentum of **stellate hairs**; old leafless branches glabrous, **young foliate branches tomentose**. Leaves: petiole 2–5 mm long, 1–2 mm in diameter, tomentose covered with minute stellate hairs, grooved, blade inserted on top of the petiole; **blade 2–9 cm long, 1–3 cm wide**, oblong to elliptic, **apex obtuse, base rounded to obtuse**, subcoriaceous, **below densely pubescent with short persistent stellate hairs covering the surface**, above sparsely pubescent when young, glabrous when old; midrib sunken or flat, above densely pubescent with fasciculate hairs when young, pubescent when old, below densely pubescent when young and old; secondary veins **7 to 12** pairs, pubescent above; tertiary venation reticulate, indistinct. Individuals bisexual; inflorescences ramiflorous on young foliate branches, leaf opposed or extra axillary. Flowers with 9 perianth parts in 3 whorls, 1 per inflorescence; pedicel 5–8 mm long, 1–2 mm in diameter, tomentose; in fruit 6–8 mm long, 2–3 mm in diameter, pubescent; bracts 2, one basal and one towards the upper half of pedicel, basal bract 1–2 mm long, 1 mm wide; upper bract 4–5 mm long, 3–4 mm wide; sepals 3, valvate, basally fused, ca. 4 mm long, 5–6 mm wide, ovate, apex obtuse, base truncate, light brown, pubescent outside, glabrous inside, margins flat; petals free, sub equal; outer petals 3, 15–17.5 mm long, 10–12 mm wide, ovate to oblong, apex rounded, base truncate, green to light yellow, margins flat becoming revolute, pubescent outside, pubescent to glabrous inside; inner petals 3, imbricate, 15–16 mm long, 10–12 mm wide, ovate to oblong, apex obtuse, base truncate, green to light yellow, margins flat becoming revolute, pubescent outside, sparsely pubescent to glabrous inside; stamens 130 to 150, in 6 to 7 rows, 2–3 mm long, linear; connective discoid, glabrous, yellow to cream; staminodes absent; carpels free, ca. 100, ovary ca. 2 mm long, stigma flat, densely pubescent. Monocarps stipitate, **stipes 25–40 mm long**, 1–2 mm in diameter, **centrally inserted**; monocarps 30 to 50, 12–18 mm long, 8–15 mm in diameter, **globose to ovoid**, apex rounded, **densely tomentose, verrucose**, faintly-one ribbed, green when immature; seeds 8 to 10 per monocarp, 7–9 mm long, 4–5 mm in diameter, flattened ellipsoid; aril absent.

#### Distribution.

A central African species, known mainly from Gabon, but also from Cameroon, Equatorial Guinea and Republic of the Congo; in Cameroon known from the South region.

#### Habitat.

A rare species in Cameroon; in lowland secondary or primary rain forests. Altitude 0–100 m a.s.l.

#### Local and common names known in Cameroon.

None recorded.

#### IUCN conservation status.

Not evaluated.

#### Uses in Cameroon.

None reported.

#### Notes.

﻿﻿﻿*Uvariaklaineana* is distinguished by its small elliptic leaves (the smallest of Cameroonian species), that are strongly discolorous with an uniform indumentum of whitish stellate hairs on the lower side and weak secondary veins above.

In Cameroon, this species is only known from a single collection in the Massif des Mamelles near Campo (*Satabié 954*).

#### Specimen examined.

**South Region**: Massif des Mamelles 25 km NE Campo Nyabessan, 2.57°N, 9.953°E, *01 January 1992*, *Satabié B.* 954 (P,YA).

### ﻿﻿﻿﻿ Uvaria mollis

Taxon classificationPlantaeMagnolialesAnnonaceae

Engl. & Diels, Notizbl. Königl. Bot. Gart. Berlin 2: 295, 1899

72DC7F79-03E7-5B4B-BB67-2A48B9046DA5

Fig. 105 ; Map 14B

≡ Uva mollis (Engl. & Diels) Kuntze, Kuntze, Deutsche Bot. Monatsschr. 21: 173, 1903.

#### Type.

Cameroon. South Region; Bipindi, *Zenker G.A. & Staudt A. 3*, no date: lectotype, here designated: K[K000198767].

#### Description.

Liana(?), unknown height, d.b.h. unknown. **Indumentum of fasciculate hairs**; old leafless branches sparsely pubescent to glabrous, **young foliate branches tomentose**. Leaves: petiole 2–5 mm long, 1–2 mm in diameter, tomentose, slightly grooved, blade inserted on top of the petiole; blade 13–16 cm long, 5–7 cm wide, oblong to elliptic, apex acuminate, acumen 1.5–2 cm long, **base subcordate**, subcoriaceous, **below pubescent with fasciculate hairs** when young and old, **above glabrous** when young and old; midrib sunken or flat, **above densely pubescent** when young, pubescent when old, below densely pubescent when young or old; secondary veins **14 to 17 pairs**, **prominent** and pubescent above; **tertiary venation reticulate**. Individuals bisexual; inflorescences ramiflorous on young branches, leaf-opposed or extra axillary. Flowers with 9 perianth parts in 3 whorls, **1 per inflorescence**; **pedicel 30–40 mm long**, ca. 2 mm in diameter, tomentose; bracts 2, one basal and one in the upper half of pedicel, basal bract 1–2 mm long, 1–2 mm wide; upper bract 2–3 mm long, 4–7 mm wide; sepals 3, valvate, free, 4–7 mm long, 5–6 mm wide, ovate, apex acute, base truncate, pubescent outside, inside not seen, margins flat; petals free, inner slightly smaller than outer; outer petals 3, 20–23 mm long, 11–14 mm wide, elliptic, apex rounded, base truncate, margins revolute, pubescent outside, inside not seen; inner petals 3, imbricate, 14–16 mm long, 16–17 mm wide, ovate to suborbicular, apex rounded, base ungulate, claw ca. 5 mm long, margins flat or revolute, pubescent outside, glabrous inside; stamens 300 to 400, in 6 to 7 rows, 1–2 mm long, linear; connective discoid, pubescent; staminodes absent; carpels free, number unknown, ovary ca. 3 mm long, stigma coiled, densely pubescent. Fruits unknown.

#### Distribution.

endemic to Cameroon, known from the Central and South regions.

#### Habitat.

A rare species; in lowland primary rain forests. Altitude 200–800 m a.s.l.

#### Local and common names known in Cameroon.

None recorded.

#### IUCN conservation status.

Not evaluated.

#### Uses in Cameroon.

None reported.

#### Notes.

﻿﻿﻿*Uvariamollis* is an imperfectly known species. Morphologically, it resembles ﻿*U.lastoursvillensis* Pellegr., a Gabonese endemic (Pellegrin 1949; Le Thomas 1969b) by its leaves with clearly impressed secondary veins looping towards the margin, the overall dense pubescence in the younger branches and flowers, and the single terminal flower at the end of young foliate branches. However, ﻿*U.mollis* is less pubescent with much shorter hairs leading to a very different aspect, and the inner petals are unguiculate with a long claw (ca. 5 mm), suggesting they are different species. In the absence of more material, we follow Le Thomas (1969b) and keep them separate.

The name ﻿*U.mollis* was first used for a South Asian species (﻿﻿﻿*Uvariamollis* Wall.) given in the “Wallich Catalogue” (Wallich 1832, catalogue number 6475), but this name is a nom. nud. In Flora Indica, Hooker and Thomson (1855 p. 135) considered this name to be a synonym of ﻿*Unonapannosa* Dalzell (Dalzell 1851) (which is now ﻿*Meiogynepannosa* (Dalzell) J.Sinclair). We found no evidence that the Wallich name was used again (e.g. Turner 2018), rendering the name ﻿*U.mollis* Engl. & Diels as described by Engler and Diels (1899) valid.

We chose here the specimen *Zenker & Staudt 3* (K) as the lectotype. It was a hard decision, because according to JSTOR the only two specimens of the syntypes that remain are in NY (*Zenker 475*) and K (*Zenker & Staudt 3*). Neither of these specimens have flowers or fruits, and are thus poor specimens to choose as a lectotype. However, we know that the specimens deposited in B did have flowers, as this was indicated by Engler and Diels (1901, p. 29). It is probable that they got destroyed or have just not been identified as type material. However, we had access to Annick Le Thomas’s archives in P when she was preparing the “Flore du Cameroun” (but never finalized it). In those archives there is an illustration of ﻿*U.mollis* with a flower (see Fig. 105) which was drawn from *Zenker & Staudt 3*. This indicates that Le Thomas had access to a specimen of *Zenker & Staudt 3* with flowers, suggesting that a better specimen might be available and could be located (possibly in P or B). At this point we have no proof that another specimen of *Zenker 475* is available.

#### Specimens examined.

**Central Region**: Yaoundé, 3.87°N, 11.52°E, *1890*, *Zenker G.A.* 249 (B); Yaoundé, 3.87°N, 11.52°E, *Zenker G.A.* 3 (K); Yaoundé, 3.06°N, 10.38°E, *01 January 1913*, *Zenker G.A.* 475 (NY). **East Region**: Bitya near R Ja Nsolo, 3.02°N, 12.37°E, *31 March 1921*, *Bates G.L.* 1818 (K).

### ﻿﻿﻿﻿ Uvaria muricata

Taxon classificationPlantaeMagnolialesAnnonaceae

Pierre ex Engl. & Diels, Monogr. Afrik. Pflanzen.-Fam. 6: 23, 1901

BA035F61-0D12-5A23-B38C-F9459576E0C8

#### Type.

Gabon. Estuaire; Environ de Libreville, *Klaine T.-J. 550*, 1896: holotype: B[B 10 0153099]; isotypes: P[P00362733, P00362731].

Only var. yalingensis is known from Cameroon to date.

### ﻿﻿﻿﻿﻿﻿ Uvaria muricata

Taxon classificationPlantaeMagnolialesAnnonaceae

var. yalingensis (Engl. & Diels) Tisserant, Bull. Soc. Bot. Fr. 103: 468, 1956

9824C584-80A4-5983-9033-1552040B0B2E

**Figure 112.**
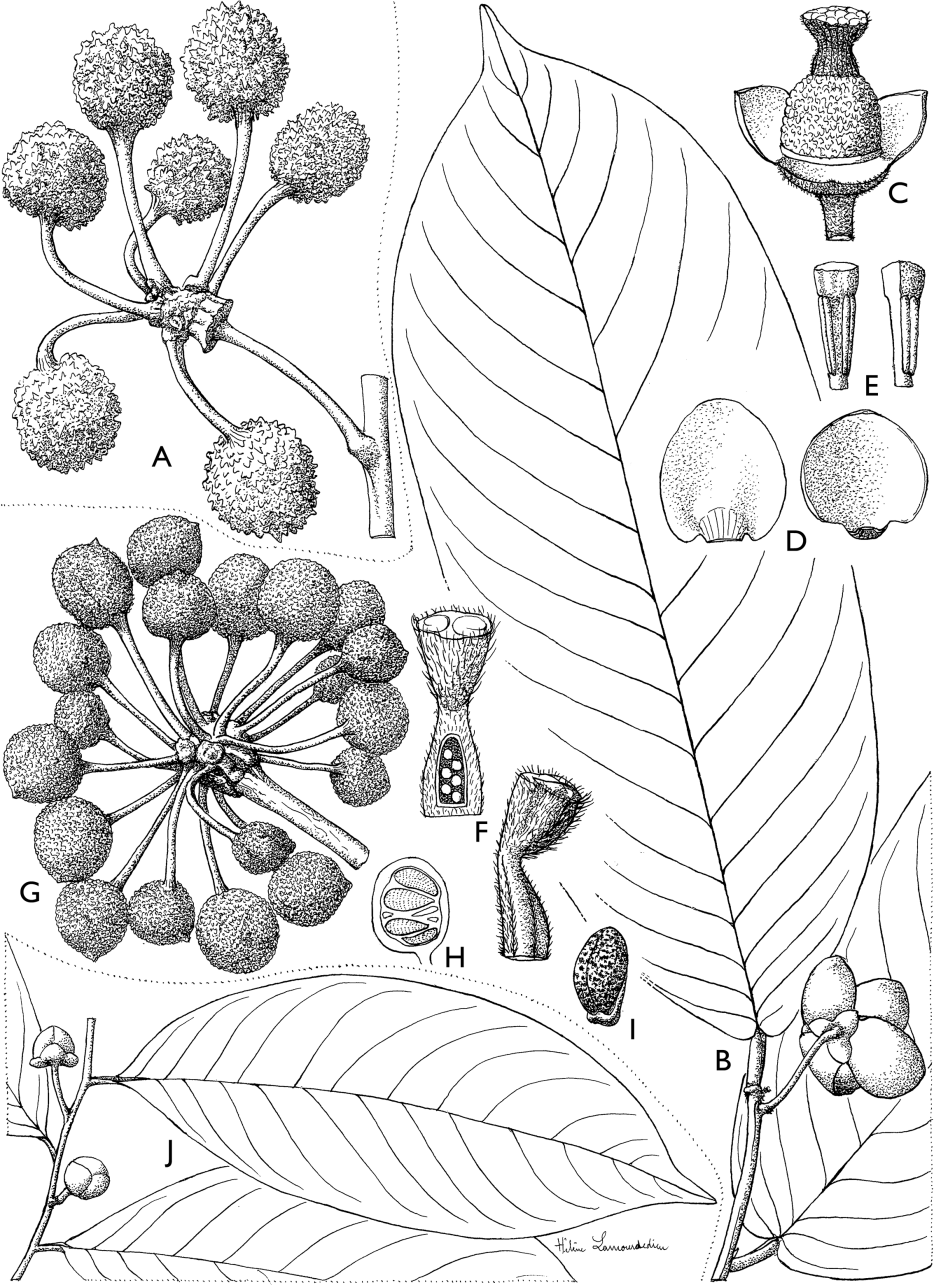
Uvariamuricatavar.muricata (not in Cameroon) **A** fruit. Uvariamuricatavar.yallingensis**B** flowering branch **C** detail of receptacle, petals removed **D** inner (left) and outer petal (right), inner view **E** stamen, side and front views **F** carpel, detail of ovules and side view **G** fruit **H** longitudinal section of monocarp **I** seed. Uvariamuricatavar.suaveolens (not in Cameroon) **J** flowering branch **A** from *Klaine 550***B–F** from *Le Testu 4647***G–I** from *Letouzey 6167***J** from *Le Testu 8486*. Drawings by Hélène Lamourdedieu, Publications Scientifiques du Muséum national d’Histoire naturelle, Paris; modified from Le Thomas (1969b, pl. 7, p. 53).

**Figure 113.**
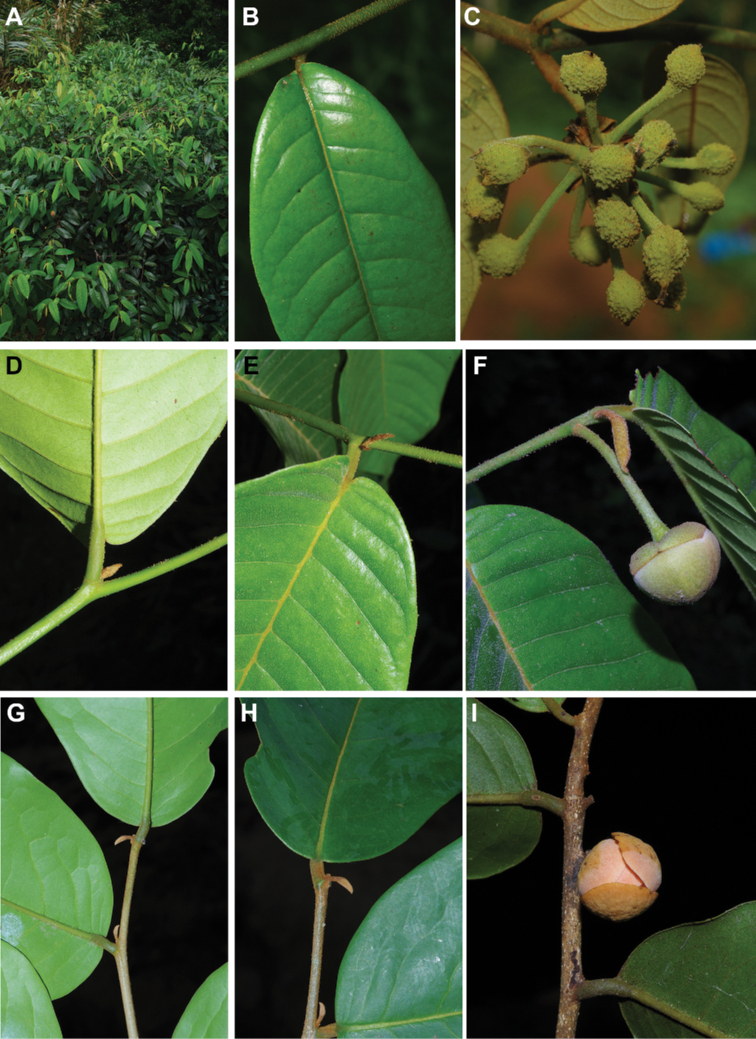
Uvariamuricatavar.muricata (not in Cameroon) **A** habit **B** leaf base, upper side **C** fruit **D** leaf base, lower side **E** leaf base, upper side **F** flower bud. *Uvariaobanensis***G** leaf base, lower side **H** leaf base, upper side **I** flower bud **A–C***Couvreur 571*, Gabon **D–F***Couvreur 892*, Gabon **G–I***Couvreur 1098*, Gabon. Photos Thomas L.P. Couvreur.

Figs 112 , 113 ; Map 14C

#### Type.

Central African Republic: Haute-Kotto; Yalinga, *Le Testu G.M.P.C. 4241*, 25 Oct 1922: holotype: P[P00362713]; isotype: BM[BM000554051].

#### Description.

Scrambling shrub to liana, 3–10 m tall, d.b.h. 6 cm. Indumentum of **stellate hairs**; old leafless branches glabrous, young foliate branches **pubescent to sparsely pubescent**. Leaves: petiole 5–15 mm long, 1–2 mm in diameter, pubescent to glabrous, slightly grooved, blade inserted on top of the petiole; blade 12–19 cm long, 2.5–9.5 cm wide, elliptic to oblong, apex acuminate, acumen 1–1.5 cm long, **base cordate**, papyraceous to subcoriaceous, **below sparsely pubescent when young**, glabrous when old, **above sparsely pubescent to glabrous** when young and old; midrib sunken or flat, **above densely pubescent** when young, pubescent when old, below pubescent when young and old; **secondary veins 12 to 17** pairs, glabrous above; **tertiary venation indistinct, slightly percurrent**. Individuals bisexual; inflorescences ramiflorous on young foliate branches, leaf opposed or extra axillary. Flowers with 9 perianth parts in 3 whorls, 1 per inflorescence; pedicel 10–22 mm long, 1–2 mm in diameter, pubescent to sparsely pubescent; in fruit 20–40 mm long, 2–3 mm in diameter, sparsely pubescent; bracts 2, one basal and one in lower half of pedicel, basal bract 2–4 mm long, 3–4 mm wide; upper bract not seen; sepals 3, valvate, **distinct in the flower bud**, basally **fused**, 3–5 mm long, 4–7 mm wide, triangular to ovate to semiorbicular, apex obtuse, base truncate, tomentose with short stellate hairs outside, tomentose with short stellate hairs inside, margins flat; petals free, sub equal; outer petals 3, 12–15 mm long, 4–7 mm wide, ovate to suborbicular, apex rounded, base truncate, margins flat, tomentose outside, glabrous inside; inner petals 3, imbricate, 12–15 mm long, 4–7 mm wide, ovate to suborbicular, apex rounded, base ungulate, margins flat, tomentose outside, glabrous inside; stamens numerous, number of rows not seen, 2–3 mm long, oblong to cuneiform; connective discoid, sparsely pubescent; staminodes absent; carpels free, 20 to 25, ovary 3–4 mm long, stigma obconical, pubescent. Monocarps stipitate, **stipes 10–15 mm long**, 1–2 mm in diameter, **centrally inserted**; monocarps 20 to 25, 10–18 mm long, 10–18 mm in diameter, **globose**, apex rounded, **pubescent with stellate hairs, finely echinate**, **not ribbed, green to brown when ripe**; seeds 4 to 5 per monocarp, ca. 10 mm long, 5–6 mm in diameter, ellipsoid; aril absent.

#### Distribution.

In its broad sense ﻿﻿﻿*Uvariamuricata* is a widespread mainly central African species (a few specimens collected from Sierra Leone) known from Cameroon to the Republic of the Congo, the Democratic Republic of the Congo and the Central African Republic; the variety *yalingensis* is known from Central African Republic and Cameroon; in Cameroon known from Adamaoua, Central, North, South, South West and West regions.

#### Habitat.

A fairly common species in Cameroon; in lowland secondary or primary rain forests. Altitude 0–1200 m a.s.l.

#### Local and common names known in Cameroon.

None recorded.

#### IUCN conservation status.

Not evaluated.

#### Uses in Cameroon.

None reported.

#### Notes.

To date, of the three varieties of ﻿*U.muricata*, only var. yallingensis is known to occur in Cameroon. The three varieties differ from each other by the pubescence of their leaves, the shape of the leaf base and the texture of the monocarps (Tisserant 1956; Le Thomas 1969b). Var. yalingensis is mainly found in drier parts of the country in the Adamaoua region, but also in the Central, South, South West and West and regions (at higher altitudes).

#### Specimens examined.

**Adamaoua Region**: ca 15 km NE of Meiganga, 6.63°N, 14.25°E, *24 November 1964*, *de Wilde W.J.J.O* 4049 (WAG); Près Dota (route Meiganga-Baïbokoum à 85 km à vol d’oiseau de Meiganga), 6.97°N, 14.93°E, *14 October 1963*, *Letouzey R.* 6167 (P,YA). **Central Region**: Eloumden, 3.83°N, 11.46°E, *20 October 1961*, *Breteler F.J.* 1977 (WAG); Ca 50 km S of Badjob ca 60 km SW Of Eséka Along the Njong-River, 3.68°N, 10.68°E, *20 March 1964*, *de Wilde W.J.J.O* 2164 (BR,K,MO,P,WAG,YA). **South Region**: 24 km from Kribi ca 3 km N of Lolodorf road, 3.03°N, 10.08°E, *31 March 1970*, *Bos J.J.* 6652 (BR,P,WAG,YA). **South-West Region**: Ndile waterfall below mission dispensary, 4.91°N, 9.745°E, *16 December 1999*, *Cheek M.* 10415 (K,MO,P,WAG,YA); Mont versant de Munyenge, 4.37°N, 9.256°E, *21 January 1985*, *Nkongmeneck B.A.* 954 (YA). **West Region**: Massif du Mbapit (1988 m) 30 km SW Foumban Versant Sud, 5.5°N, 10.63°E, *21 October 1974*, *Letouzey R.* 12936 (P,YA).

### ﻿﻿﻿﻿ Uvaria obanensis

Taxon classificationPlantaeMagnolialesAnnonaceae

Baker f., Cat. Pl. Oban: 1, 1913

8590A171-BAC5-5116-99B4-27E832BBBFF1

Fig. 113 ; Map 14D

≡ Richella obanensis (Baker f.) R.E.Fr. Nat. Pflanzenfam., ed. 2, 17a(2): 139, 1959.
= Uvaria marginata Diels, Bot. Jahrb. Syst. 53. 437, 1915. Type. Cameroon. South-West Region, Johann Albrechtshöhe, *Büsgen M. 191*, 1 Dec 1908: holotype: B[B 10 0153104].

#### Type.

Type. Nigeria. Cross River State; Oban, *Talbot P.A. 1603*, 1912: lectotype, sheet here designated: K[K000198779]; isotypes: BM[BM000554066]; K[K000198780, K000198781].

#### Description.

Liana, 3–10 m tall, d.b.h. unknown. Indumentum of simple or fasciculate hairs, **but overall glabrous**; old leafless branches glabrous, **young foliate branches sparsely pubescent becoming quickly glabrous**. Leaves: petiole 4–5 mm long, 2 mm in diameter, glabrous, slightly grooved, blade inserted on top of the petiole; **blade 15–26 cm long, 5–10 cm wide**, oblong, apex acuminate to acute, acumen 1.5–2 cm long, **base rounded to subcordate**, **coriaceous**, below **very sparsely pubescent to glabrous** when young, glabrous when old, **above glabrous** when young and old; midrib sunken or flat, above glabrous when young and old, below glabrous when young and old; secondary veins **7 to 12** pairs, glabrous above; **tertiary venation reticulate**. Individuals bisexual; inflorescences ramiflorous on young foliate branches, extra axillary. Flowers with 9 perianth parts in 3 whorls; 1 to 3(9) per inflorescence; pedicel 6–8 mm long, 2–3 mm in diameter, sparsely pubescent to glabrous; bracts 2, one basal and one towards the lower half of pedicel, basal bract 2–4 mm long, 3–4 mm wide; upper bract 2–3 mm long, 3–5 mm wide; sepals 3, valvate, free, 4–7 mm long, 6–8 mm wide, ovate to semiorbicular, apex obtuse, base truncate, green, tomentose outside, pubescent inside, margins flat; petals free, sub equal; outer petals 3, 20–30 mm long, 17–25 mm wide, ovate to suborbicular, apex rounded, base truncate, green to light yellow, margins flat, tomentose outside, glabrous inside; inner petals 3, imbricate, 20–30 mm long, 17–25 mm wide, ovate to suborbicular, apex rounded, base unguiculate, green to light yellow, margins flat, tomentose outside, glabrous inside; stamens 120 to 150, in 8 to 10 rows, 2–3 mm long, oblong to cuneiform; connective discoid, glabrous; staminodes absent; carpels free, 15 to 20, ovary ca. 3 mm long, stigma coiled, glabrous. **Monocarps sessile**, monocarps possibly 5 to ca. 8 (but only one seen in *McPherson 15524* (LBV)), **ca. 40 mm long, ca. 25 mm in diameter**, **glabrescent to glabrous, ellipsoid**, apex rounded, **smooth**, green when ripe; seeds not seen.

#### Distribution.

Known from Nigeria to Cameroon, and more recently from Gabon (see below); in Cameroon known from South and South West regions.

#### Habitat.

A fairly rare species in Cameroon; in lowland secondary or primary rain forests. Altitude 0–300 m a.s.l.

#### Local and common names known in Cameroon.

None recorded.

#### IUCN conservation status.

Not evaluated.

#### Uses in Cameroon.

None reported.

#### Notes.

﻿﻿﻿*Uvariaobanensis* belongs to a group of species that are almost glabrous on the branches and leaves. It is characterized by its large and shiny (in herbarium material) oblong coriaceous leaves with few secondary veins (less than 13) and a broadly cordate base. The flowers have free sepals not enclosing the petals in bud. The monocarps are sessile, a character only found in one other ﻿*Uvaria* species from Cameroon. ﻿﻿*U.scabrida*. ﻿﻿﻿*Uvariaobanensis* closely resembles ﻿*U.cabrae*De Wild from the Democratic Republic of the Congo in the shape and size of its leaves with a cordate leaf base and being overall glabrous, but differs in its monocarps which are stipitate, ribbed and densely pubescent brown in the latter (versus sessile, smooth and glabrescent to glabrous in ﻿*U.obanensis*).

We recently collected ﻿﻿﻿*Uvariaobanensis* in Gabon (near Koulamoutou, Ogooué-Lolo, *Couvreur 1098*), extending its distribution range south of the equator.

The monocarps are here described for the first time (but we did not see the seeds) based on a collection from Gabon (*McPherson 15524* (LBV)).

#### Specimens examined.

**South Region**: 9 km N of Kribi, 3°N, 9.933°E, *30 October 1969*, *Bos J.J.* 5555 (BR,C,K,LD,P,WAG,YA); Ebom, 3.1°N, 10.73°E, *24 February 1997*, *Elad M.* 564 (KRIBI,WAG). **South-West Region**: Ekombe-Mofako Mokoko Forest Reserve, 4.47°N, 9.092°E, *23 April 1994*, *Acworth J.M.* 182 (K,YA); Kumba, 4.63°N, 9.433°E, *01 December 1908*, *Büsgen M.* 191 (B, K); West bank of the Onge River, 4.28°N, 8.966°E, *07 November 1993*, *Thomas D.W.* 9802 (K,YA).

### ﻿﻿﻿﻿ Uvaria osmantha

Taxon classificationPlantaeMagnolialesAnnonaceae

Diels, Bot. Jahrb. Syst. 53: 436, 1915

951F1407-B9F1-5B99-A214-B061DFAF6058

Map 14E

= Uvaria scaberrima Exell, J. Bot. 73 (Suppl. 1): 3, 1935. Type. Angola. Cuanza Sul Province, Quibanga de Mucende, Libolo, *Gossweiler J. 6311*, 19 May 1915: holotype: BM[BM000554067]; isotypes: COI[COI00004869]; LISC[LISC000302].

#### Type.

Cameroon. East Region; Molundu, *Mildbraed G.W.J. 4715*, 1911: lectotype, sheet here designated: B[B 10 0153106]; isotypes: B[B 10 0153105]; HBG[HBG502489].

#### Description.

Liana, 4–6 m tall, d.b.h. unknown. Indumentum of **stellate or fasciculate hairs**; old leafless branches glabrous, **young foliate branches densely pubescent**. Leaves: petiole 2–3 mm long, 1–2 mm in diameter, densely pubescent to tomentose, slightly grooved, blade inserted on top of the petiole; **blade 4.5–12 cm long, 2–5 cm wide**, elliptic to oblong, apex acuminate to acute, acumen 0.9–1.5 cm long, **base rounded to cordate**, papyraceous to subcoriaceous, **discolorous, below completely covered with short stipitate stellate hairs** when young and old, **above pubescent with very short scabrid fasciculate hairs**; midrib sunken or flat, above densely to sparsely pubescent when young, sparsely pubescent to glabrous when old, below densely pubescent when young and old; secondary veins **9 to 12** pairs, sparsely pubescent to glabrous above; tertiary venation reticulate, but indistinct. Individuals bisexual; inflorescences ramiflorous on young foliate branches, leaf opposed or extra axillary. Flowers with 9 perianth parts in 3 whorls, 1 to 3 per inflorescence; pedicel 10–30 mm long, 1–2 mm in diameter, densely pubescent; in fruit 20–30 mm long, 2–3 mm in diameter, pubescent; bracts 2, one basal and one towards the lower half of pedicel, basal bract 2 mm long, 2 mm wide; upper bract 2–3 mm long, 3–5 mm wide; sepals 3, valvate, **completely fused, tearing at anthesis**, 12–15 mm long, 8–10 mm wide, ovate, apex acute to acuminate, base truncate, pubescent outside, glabrous inside, margins flat; petals free, sub equal; outer petals 3, 25–35 mm long, 10–18 mm wide, oblong-obovate to oblong, apex acute, base truncate, green to light yellow, margins flat, densely pubescent outside, pubescent inside; inner petals 3, imbricate, 25–35 mm long, 10–18 mm wide, obovate to ovate, apex obtuse, base truncate, green to light yellow, margins flat, densely pubescent outside, pubescent inside; stamens 120 to 150, in 6 to 7 rows, 1–2 mm long, elongated; connective discoid, sparsely pubescent; staminodes absent; carpels free, 30 to 45, ovary 3–4 mm long, stigma coiled, glabrous. Monocarps stipitate, **stipes 10–15 mm long**, 1–2 mm in diameter, **inserted laterally**; monocarps 15 to 20, 15–30 mm long, 5–12 mm in diameter, **cylindrical**, apex apiculate, **brown-tomentose, verrucose**, slightly constricted between the seeds in dried material, brown when ripe; seeds 4 to 8 per monocarp, 7 mm long, 4–6 mm in diameter, ellipsoid; aril absent.

#### Distribution.

A central African species, known from Cameroon, Gabon, Democratic Republic of the Congo and Angola; in Cameroon known from the East region.

#### Habitat.

A fairly rare species in Cameroon; in lowland secondary or primary rain forests. Altitude 500–900 m a.s.l.

#### Local and common names known in Cameroon.

None recorded.

#### IUCN conservation status.

Not evaluated.

#### Uses in Cameroon.

None reported.

#### Notes.

﻿﻿﻿*Uvariaosmantha* is distinguished by its strongly discolorous leaves with a rounded to cordate, scabrid above and completely covered with stellate hairs below. In addition, its sepals are completely fused in bud and tearing at anthesis (see notes under ﻿*U.angolensis*) and its monocarps are cylindrical and brown-tomentose, with moderately long stipes (10–15 mm long) inserted laterally. In leaf shape and indumentum, ﻿*U.osmantha* resembles ﻿﻿*U.scabrida* (also found in Cameroon, upper side of leaves also scabrous) and ﻿*U.schweinfurthii* (not found in Cameroon but in the Democratic Republic of the Congo, Central African Republic and East Africa) but the monocarps of the latter two are clearly different being sessile in the former and long (> 20 mm long) stipitate (more than twice as long as the monocarp) in the latter. In addition, ﻿﻿*U.scabrida* has stipitate (not sessile) stellate hairs on lower leaf surface, much more prominent secondary leaf veins, percurrent tertiary venation, and a calyx with distinct sepals. ﻿﻿*Uvariaschweinfurthii* also has a calyx with distinct sepals and monocarps inserted centrally on the stipes.

#### Specimens examined.

**East Region**: Prairies 10 km à l’E de Kinsassa village situé à 65 km au NNE de Moloundou par route Yokadouma 2.61°N, 15.47°E, *04 March 1971*, *Letouzey R.* 10494 (P,YA); Prairie à 30 km WSW de Kinsasa village situé à 65 km NNE de Moloundou sur la route de Yokadouma 2.34°N, 15.28°E, *10 March 1971*, *Letouzey R.* 10556 (P,YA).

### ﻿﻿﻿﻿ Uvaria poggei

Taxon classificationPlantaeMagnolialesAnnonaceae

Engl. & Diels, Notizbl. Königl. Bot. Gart. Berlin 2: 294, 1899

4161E336-8350-582D-81E3-5802C271AB29

Map 14F

≡ Uva poggei (Engl. & Diels) Kuntze, Kuntze, Deutsche Bot. Monatsschr. 21: 173, 1903.

#### Type.

Democratic Republic of the Congo. Kasaï Central; Mukenge, *Pogge P. 622*, 18 Sep 1882: lectotype, designated by Le Thomas (1969b), p. 73: B[B 10 0153109]; isolectotype: K[K000198768].

#### Description.

Liana, 15–18 m tall, d.b.h. 3–10 cm. Indumentum of **minute stellate or simple hairs**; old leafless branches sparsely pubescent, **young foliate branches tomentose**. Leaves: petiole 2–3 mm long, 1–2 mm in diameter, densely pubescent to tomentose, slightly grooved, blade inserted on top of the petiole; blade 8–19 cm long, 5–9 cm wide, obovate to elliptic, apex acuminate, acumen ca. 1 cm long, **base rounded to cordate**, papyraceous to membranaceous, **below rather densely pubescent with stipitate stellate** hairs when young and old, **above sparsely pubescent** with simple hairs (soft to touch) when young and old; midrib sunken or flat, above sparsely pubescent when young and old, below densely pubescent when young and old; **secondary veins 15 to 24** pairs, sparsely pubescent to glabrous above; **tertiary venation percurrent**. Individuals bisexual; inflorescences ramiflorous on young foliate branches, extra axillary. Flowers with 9 perianth parts in 3 whorls, 1 per inflorescence; pedicel 15–20 mm long, 1–2 mm in diameter, densely pubescent; in fruit 25–35 mm long, 2–3 mm in diameter, sparsely pubescent; bracts 2, one basal and one upper towards the lower half of pedicel, basal bract 2 mm long, 2 mm wide; upper bract 2–3 mm long, 3–5 mm wide; sepals 3, valvate, free, not covering in bud, 4–7 mm long, 5–8 mm wide, triangular to ovate, apex obtuse, base truncate, tomentose outside, pubescent inside, **margins reflexed**; petals free, sub equal; outer petals 3, 15–20 mm long, 12–15 mm wide, ovate, apex attenuate, base truncate, margins flat, tomentose outside, sparsely pubescent to glabrous inside; inner petals 3, imbricate, 15–20 mm long, 12–15 mm wide, ovate, apex attenuate, base truncate, margins flat, tomentose outside, sparsely pubescent to glabrous inside; stamens 150 to 200, in 8 to 10 rows, ca. 2 mm long, linear; connective discoid, sparsely pubescent; staminodes absent; carpels free, 15 to 20, ovary 3–4 mm long, stigma coiled, pubescent. Monocarps stipitate, **stipes 25–35 mm long**, 1–2 mm in diameter, **centrally inserted**; monocarps 30 to 35, 7–15 mm long, 7–15 mm in diameter, **globose**, apex apiculate, **densely to sparsely pubescent**, smooth, not ribbed, green when ripe; seeds not counted, ca. 7 mm long, 4–6 mm in diameter, ellipsoid; aril absent.

#### Distribution.

A central African species, known from Cameroon, Democratic Republic of the Congo, Gabon, Republic of the Congo, Central African Republic and Angola; in Cameroon known from the East region.

#### Habitat.

A fairly rare species (or at least rarely collected) in Cameroon; in lowland secondary or primary rain forests. Altitude 400–600 m a.s.l.

#### Local and common names known in Cameroon.

None recorded.

#### IUCN conservation status.

Not evaluated.

#### Uses in Cameroon.

None reported.

#### Notes.

﻿﻿﻿*Uvariapoggei* is distinguished by its leaves with numerous secondary veins (17 to 24), its lower leaf surface covered with an uniform indumentum of stellate hairs, and its globose monocarps with a long central stipes inserted centrally. This species only marginally reaches Cameroon (Harris 2002) where it is known from two collections (*Harris 6200*, *6528*) close to the Central African Republic border. The species is here regarded as monotypic; indeed, ﻿U.poggeivar.anisotricha Le Thomas is raised to species rank, as ﻿*U.anisotricha* (see under that name).

#### Specimens examined.

**East Region**: Lobeke Reserve Terra firma forest 1 km east of Djangi Bai, 2.31°N, 15.76°E, *04 November 1998*, *Harris D.J.* 6200 (E); Lobeke Reserve 3–4 km north of Djembe road 5 km northeast of Bolu Bai, 2.21°N, 15.78°E, *24 November 1998*, *Harris D.J.* 6528 (E).

### ﻿﻿﻿﻿﻿ Uvaria scabrida

Taxon classificationPlantaeMagnolialesAnnonaceae

Oliv., Fl. Trop. Afr. 1: 21, 1868

8B35008D-E76F-571F-9FA1-062FB02572EC

**Figure 114.**
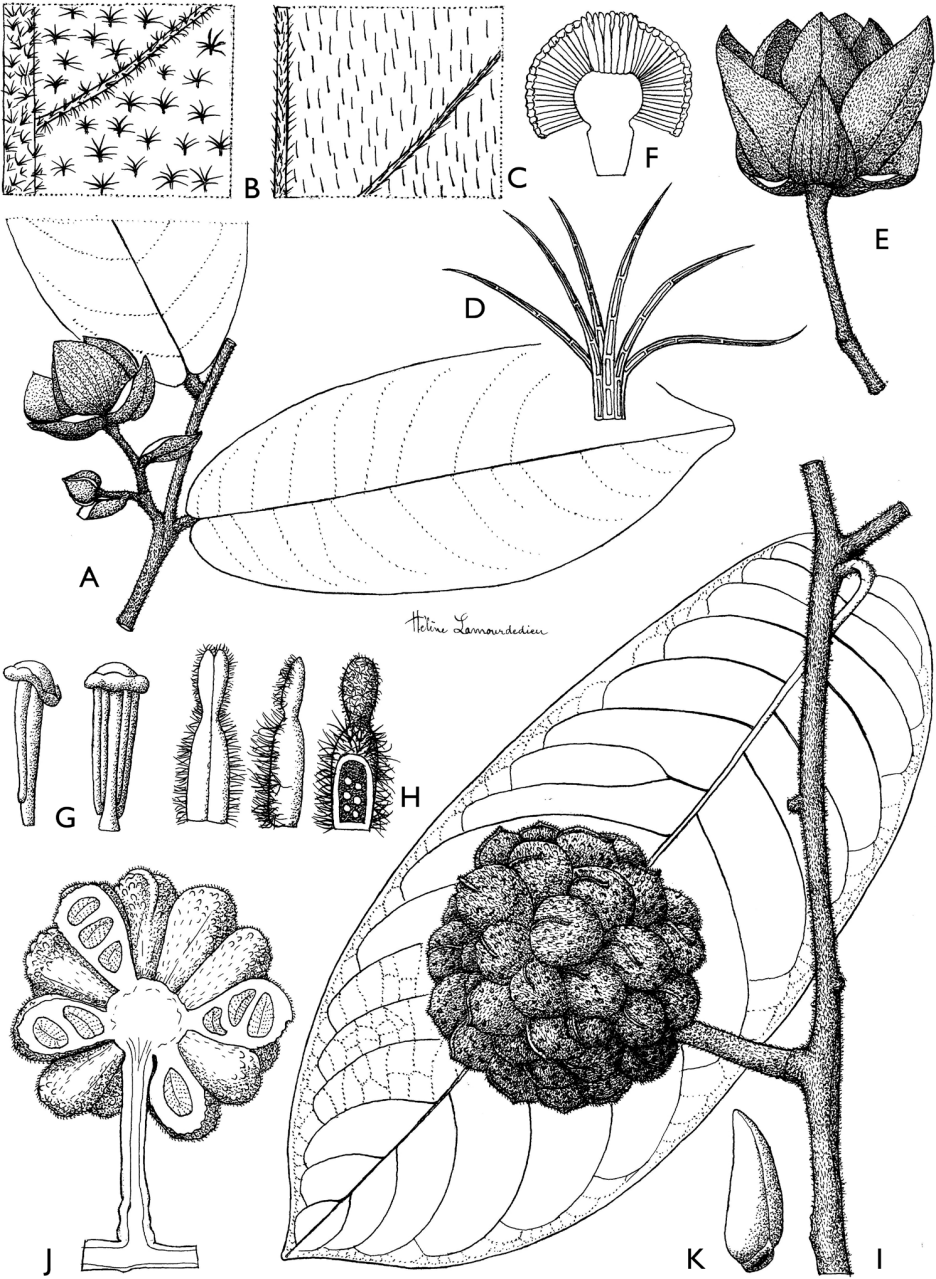
*Uvariascabrida***A** flowering branch **B** detail of pubescence on leaf blade, lower side **C** detail of pubescence of leaf blade, upper side **D** detail of one stellate hair **E** flowering pedicel and flower, side view **F** longitudinal section of receptacle **G** stamen, side and front views **H** carpel, front and side views, detail of ovules **I** fruit branch **J** longitudinal section of fruit and monocarps, showing sessile monocarps **K** seed **A–D** from *Le Testu 9356***E–H** from *Pobéguin 142***I–K** from *Hallé & Le Thomas 30*. Drawings by Hélène Lamourdedieu, Publications Scientifiques du Muséum national d’Histoire naturelle, Paris; modified from Le Thomas (1969b, pl. 13, p. 77).

Figs 114 , 116 ; Map 14G

= Uvaria corynocarpa Diels, Bot. Jahrb. Syst. 53: 436, 1915. Type. Cameroon. East Region, Lomié, *Mildbraed G.W.J. 5300*, 20 May 1911: lectotype, sheet here designated: B[B 10 0153076]; isotypes: B[B 10 0153075]; HBG[HBG502488].
= ﻿﻿﻿﻿﻿Uvariascabridavar.parviflora Pellegr., Fl. Mayombe 1: 12, 1920. Type. Gabon. Nyanga, Mavoundi, *Le Testu G.M.P.C. 1516*, 12 Dec 1908: lectotype, here designated: BM[BM000554040]; isolectotype: LISC[LISC000400].
= Pachypodanthium gossweileri Exell & Mendonça, Journ. Bot 74 (Suppl. Polypet.): 14, 1936. Type. Angola. Uige, Maquela do Zombo, Rio Vogi, Zadi - Inquissi, *Gossweiler J. 10417*, 29 Oct 1935: holotype: LISC[LISC000303]; isotypes: BM[BM000554042]; C[C10000168]; K[K000198774].
= Uvaria glomerulata A. Chev.; Sudania i: 192, No. 11086, 1911. *nom. nud.*

#### Type.

Cameroon. Littoral Region; Cameroons River, *Mann G. 1433*, 1862: lectotype, here designated: K[K000105336].

#### Description.

Liana, 2–20 m tall, d.b.h. unknown. Indumentum of **minute stellate or simple hairs**; old leafless branches pubescent to glabrescent, young foliate branches densely pubescent. Leaves: petiole 2–3 mm long, ca. 1 mm in diameter, pubescent, grooved, blade inserted on top of the petiole; blade 6–23 cm long, 2.5–10 cm wide, elliptic to oblong, apex acuminate to obtuse, acumen 1–1.2 cm long, base rounded to subcordate, papyraceous to coriaceous, **below densely pubescent** covered with minute stipitate stellate hairs when young and old, **above pubescent with short simple scabrid hairs (or glabrous besides veins)** when young and old; midrib sunken or flat, above densely pubescent when young and old, below densely pubescent when young and old; **secondary veins 9 to 16** pairs, densely pubescent above; **tertiary venation percurrent**. Individuals bisexual; inflorescences ramiflorous on young foliate branches, leaf opposed or extra axillary. Flowers with 9 perianth parts in 3 whorls, 1 to 2 per inflorescence; pedicel 10–25 mm long, 1–2 mm in diameter, densely pubescent to tomentose; in fruit 20–40 mm long, 6 mm in diameter, pubescent; bracts 2, soon falling, one basal and one towards the upper half of pedicel, basal bract 10–15 mm long, 7–10 mm wide; upper bract not seen; sepals 3, valvate, basally fused, 15–20 mm long, 7–13 mm wide, ovate, apex acute to obtuse, base truncate, yellow brown, densely pubescent outside, pubescent inside, margins flat; petals free, sub equal; outer petals 3, 18–30 mm long, 13–20 mm wide, elliptic to ovate, apex attenuate, base truncate, yellow-brown, margins flat, densely pubescent outside, pubescent inside; inner petals 3, imbricate, 15–25 mm long, 12–19 mm wide, ovate, apex obtuse, base truncate, yellow brown, margins flat, pubescent outside, sparsely pubescent to glabrous inside; stamens 180 to 200, in 9 to 10 rows, ovary 1–2 mm long, linear; connective discoid, glabrous, cream to yellow; staminodes absent; carpels free, 45 to 70, ca. 3 mm long, stigma coiled, pubescent. **Monocarps sessile**, 20 to 35 **tightly packed together**, 15–20 mm long, 10–13 mm in diameter, pyramidal or four-sided, apex rounded to shortly apiculate, **tomentose, verrucose to shortly echinate**, not ribbed or 1-ribbed, green when ripe; seeds 4 to 6 per monocarp, 8–12 mm long, 4–6 mm in diameter, flattened; aril absent.

#### Distribution.

A widespread species in west and central Africa, in Liberia and Nigeria and in Cameroon, Gabon and Equatorial Guinea to the Democratic Republic of the Congo and Angola; in Cameroon known in the Central, East, Littoral, South and South-West regions.

#### Habitat.

A fairly common species in Cameroon; in lowland secondary or primary rain forests, gallery forests, growing along forest openings. Altitude 0–800 m a.s.l.

#### Local and common names known in Cameroon.

None recorded.

#### IUCN conservation status.

Not evaluated.

#### Uses in Cameroon.

None reported.

#### Notes.

﻿﻿﻿﻿*Uvariascabrida* is distinguished by its leaves covered below with uniform stellate hairs, and above with minute scabrid simple hairs, as well as by its sessile, densely tomentose monocarps. Together with ﻿*U.obanensis*, it is the only species of ﻿*Uvaria* in Cameroon with sessile monocarps. Vegetatively, it resembles ﻿*U.osmantha*, but the latter has shorter (10–15 mm long) and stipitate monocarps, and its leaf blades are strongly discolorous with their lower surface completely obscured by the stellate indumentum, while in ﻿﻿*U.scabrida* it is visible between the hairs.

A few collections from Cameroon (*Bos 3164, 3353, 5011, 5508*) and Gabon (*Wieringa 4387*) have glabrous leaves above apart from the veins, and therefore are not scabrid. Nevertheless, they have the characteristic monocarps and thus match ﻿﻿*U.scabrida*, possibly being a variety (Lachenaud, pers. comm.).

#### Specimens examined.

**Central Region**: Village Nkolbisson 7 km West of Yaoundé, 3.88°N, 11.45°E, *04 May 1962*, *Breteler F.J.* 2880 (U,WAG); Nkolbison, 3.88°N, 11.45°E, *10 November 1963*, *de Wilde W.J.J.O* 1199 (WAG); Etuk Ebé (Yaoundé), 3.87°N, 11.52°E, *04 May 1971*, *Mpom B.* 544 (P). **East Region**: Ngoko (Moloundou), 1.67°N, 16.04°E, *04 April 1971*, *Letouzey R.* 10599 (P,YA). **Littoral Region**: Tonde (Douala), 4.22°N, 9.84°E, *09 May 1976*, *Letouzey R.* 14884 (P,WAG,YA). **South Region**: 65 km S of Kribi Gr Batanga road, 2.89°N, 9.905°E, *28 October 1968*, *Bos J.J.* 3164 (WAG,YA); 75 km from Kribi Ebolowa road, 2.89°N, 9.957°E, *13 May 1969*, *Bos J.J.* 4518 (WAG,YA); 15 km from Kribi Ebolowa road Bidou II, 2.85°N, 10°E, *04 July 1969*, *Bos J.J.* 5011 (BR,P,WAG,YA); 13 km from Kribi Ebolowa road, 2.85°N, 10.9°E, *16 October 1969*, *Bos J.J.* 5508 (BR,C,K,LD,P,WAG,YA); Ndengue (Ebolowa), 2.78°N, 11.12°E, *26 March 1970*, *Letouzey R.* 10251 (P,WAG,YA); Sangméli 2.93°N, 11.98°E, *26 March 1981*, *Meijer D.* 15270 (MO,WAG); 6 km W de Nyabessan, 2.4°N, 10.4°E, *26 November 1982*, *Nkongmeneck B.A.* 348 (P,YA); Campo-Ma’an area Medjivini, 2.29°N, 10.34°E, *31 March 2000*, *Tchouto Mbatchou G.P.* 2721 (KRIBI,WAG,YA); Efoulan, 2.74°N, 10.54°E, *24 April 2000*, *Tchouto Mbatchou G.P.* 2819 (KRIBI,WAG,YA). **South-West Region**: on top of hill near Small Ekombe village 3 km after Kumba on road to Ekondo Titi town, 4.62°N, 9.376°E, *13 January 2016*, *Couvreur T.L.P.* 982 (WAG,YA); Manja village Mundemba, 4.98°N, 8.916°E, *06 May 1996*, *Etuge M.* 1888 (K).

**Figure 115.**
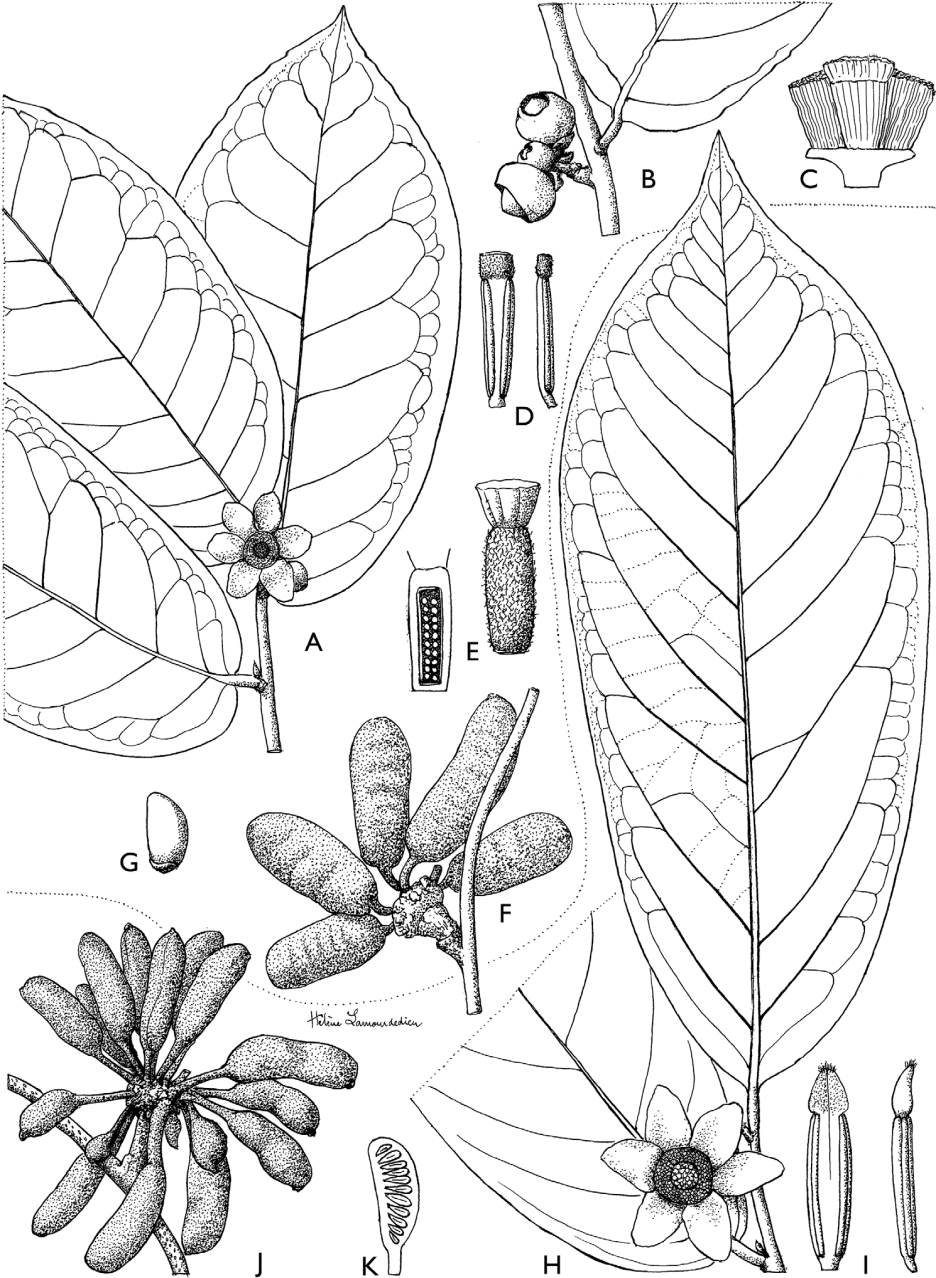
*Uvariaversicolor***A** flowering branch **B** flower buds, note fused cup shaped sepals **C** longitudinal section of receptacle **D** stamen, side and front view, note discoid connective **E** carpel, section showing ovules, and front view **F** fruit, note shorty stipitate and cylindrical monocarps **G** seed. *Uvariaangolensis***H** flowering branch **I** stamen, side and front views, note elongated (tongue shaped) connective **J** fruit, note shorty stipitate and cylindrical monocarps **K** longitudinal section of monocarp **A, B** from *Le Testu 8491***C–E** from *Le Testu 1812***F, G** from *Klaine 680***H–K** from *Letouzey 7486*. Drawings by Hélène Lamourdedieu, Publications Scientifiques du Muséum national d’Histoire naturelle, Paris; modified from Le Thomas (1969b, pl. 5, p. 47).

**Figure 116.**
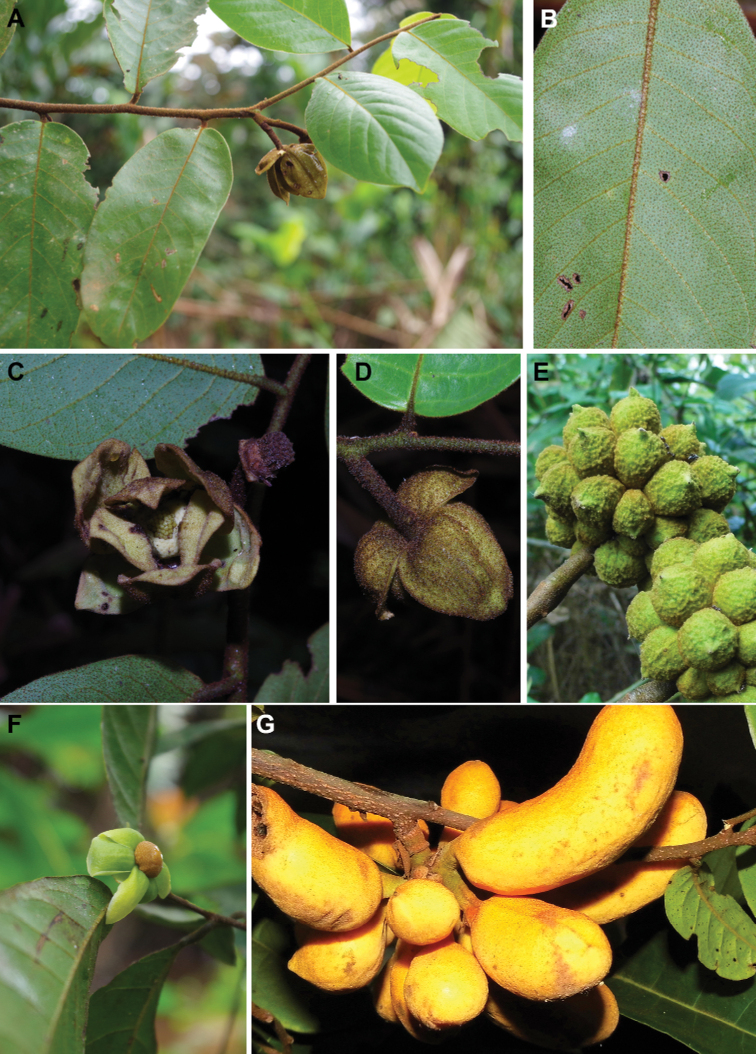
*Uvariascabrida***A** flowering branch **B** detail of lower leaf side **C** flower, top view **D** flower, basal view **E** fruits, note sessile monocarps. *Uvariaversicolor***F** flower **G** fruit, note subsessile monocarps cylindrical in shape **A–E***Couvreur 849*, Gabon **F***Couvreur 897*, Gabon **G***Faye 33*, Republic of the Congo. Photos **A–F** Thomas L.P. Couvreur **G** Adama Faye.

### ﻿﻿ Uvariastrum

Taxon classificationPlantaeMagnolialesAnnonaceae

Engl., Monogr. Afrik. Pflanzen.-Fam. 6: 31, 1901

0259C5C4-D442-5E7E-B4E4-8919CFA9E728

#### Type species.

﻿﻿﻿*Uvariastrumpierreanum* Engl. & Diels.

#### Description.

Trees or shrubs, 4–30 m tall, d.b.h. up to 30 cm; stilt roots or buttresses absent. Indumentum of simple hairs. Leaves: petiole 1–4 mm long, 1–2 mm in diameter; blade 6–22 cm long, 2–5 cm wide, elliptic or obovate, apex acuminate, acumen 0.7–2 cm long, base subcordate to decurrent to rounded to cuneate, concolorous; midrib sunken or flat; secondary veins 7 to 17 pairs, arching well before the margin; tertiary venation reticulate. Individuals bisexual; inflorescences cauliflorous, or ramiflorous young foliate branches and axillary. Flowers with 9 perianth parts in 3 whorls, 1 to 3 per inflorescence; pedicel 8–50 mm long; in fruit 10–50 mm long; bracts 1 to 3, all basal; sepals 3, reduplicate-valvate, free, 10–25 mm long, apex acute, base truncate; petals free, sub equal; outer petals 3, valvate, 20–40 mm long, 5–15 mm wide, ovate to elliptic, apex acute, base truncate or narrowed; inner petals 3, valvate, 10–28 mm long, 6–15 mm wide, ovate or elliptic, apex acute, base cuneate; stamens numerous (not counted), 2–6 mm long, linear; connective elongated to discoid; staminodes absent; carpels free, 5 to 16, 2–6 mm long, stigma bilobed or capitate. Monocarps stipitate or sessile, stipes 1–6 mm long; monocarps 2 to 8, 30–100 mm long, 10–50 mm in diameter, globose or ellipsoid, apex apiculate or acute to rounded; seeds 16 to 27, 10–25 mm long, 7–15 mm in diameter, flattened ellipsoid; aril absent.

A genus of five species from West and Central Africa; one species adapted to the drier regions in southern Central Africa (*U.hexaloboides* (R.E.Fr.) R.E.Fr.); in Cameroon three species, none endemic.

#### Taxonomy.

Couvreur (2014).

### ﻿Key to the species of ﻿*Uvariastrum* in Cameroon

**Table:** 

1	Upper side of midrib glabrous, or sometimes very sparsely pubescent in young leaves	**2**
–	Upper side of midrib conspicuously pubescent, especially in younger leaves	﻿***U.insculptum***
2	Blade inserted on top, pinched, not forming a groove above on the petiole; flowering pedicels and sepals drying black	﻿﻿﻿***U.zenkeri***
–	Blade inserted on the sides, not pinched, forming a groove; flowering pedicels and sepals drying light brown	﻿***U.pierreanum***

### ﻿﻿﻿﻿ Uvariastrum insculptum

Taxon classificationPlantaeMagnolialesAnnonaceae

(Engl. & Diels) Sprague & Hutch., Bull. Misc. Inform. Kew 6: 159, 1916

EA9F3FE8-BB0C-5E2F-AE5A-217B1D8D9417

Fig. 117 ; Map 14H

≡ Uvaria insculpta Engl. & Diels, Notizbl. Königl. Bot. Gart. Berlin 2: 295, 1899.

#### Type.

Cameroon. South-West Region; Johann-Albrechtshöhe[Kumba], *Staudt A. 740*, 1896: lectotype, designated by Couvreur (2014), p. 18: B[B100153111]; isolectotypes: COI[COI00077205]; G[G00011729]; K[K000105338, K000105339, K000105340]; [P00315828, P00315829]; S[S12-22768].

#### Description.

Tree to shrub, 4–15 m tall, d.b.h. 3–5 cm; stilt roots or buttresses absent. Indumentum of simple hairs; old leafless branches glabrous, **young foliate branches densely pubescent.** Leaves: petiole 1–4 mm long, 1 mm in diameter, **densely pubescent**, weakly grooved adaxially, blade inserted on top of the petiole; blade 6–14 cm long, 2–4 cm wide, elliptic to obovate, apex rounded or acuminate, acumen 1–2 cm long, base subcordate, papyraceous to subcoriaceous, below glabrous when young and old, above sparsely pubescent to glabrous when young, sparsely pubescent to glabrous when old, concolorous; midrib sunken or flat, above pubescent when young, glabrous when old, below sparsely pubescent to densely pubescent when young, sparsely pubescent to densely pubescent when old; secondary veins 8 to 12 pairs, sparsely pubescent to glabrous above; tertiary venation reticulate. Individuals bisexual; inflorescences cauliflorous, ramiflorous on young foliate or on old leafless branches, axillary. Flowers with 9 perianth parts in 3 whorls, 1 to 2 per inflorescence; pedicel 8–15 mm long, 1–2 mm in diameter, densely pubescent; in fruit 10–20 mm long, 2–5 mm in diameter, densely pubescent; bracts 3, all basal, 4 mm long, 3–4 mm wide; sepals 3, reduplicate-valvate, free, 10–20 mm long, 5–8 mm wide, ovate, apex acute, base truncate, green to light green or pale yellow with darker margins, densely pubescent outside, densely pubescent inside, margins slightly revolute; petals free, outer petals longer than inner; outer petals 3, 23–35 mm long, 5–10 mm wide, ovate, apex acute, base narrowed, light yellow to white, margins flat, pubescent outside, pubescent (sometimes sparsely) pubescent inside; inner petals 3, valvate, 10–20 mm long, 6–10 mm wide, ovate, apex acute, base cuneate, light yellow, margins flat, densely pubescent outside, sparsely pubescent inside; stamens numerous, 2–3 mm long, linear; connective elongated, densely pubescent; staminodes absent; carpels free, 6 to 7, ovary 2–3 mm long, stigma bilobed, slightly capitate, glabrous. Monocarps shortly stipitate, stipes 2–6 mm long, 2–5 mm in diameter; monocarps 2 to 8, 30–60 mm long, 10–20 mm in diameter, oblong, apex apiculate, **densely pubescent, finely warty, longitudinally 4 to 6 ribbed, resembling that of a peanut**, light brown when ripe; seeds ca. 20 per monocarp, 10–15 mm long, 7–9 mm in diameter, flattened ellipsoid; aril absent.

#### Distribution.

A mainly West African species, but with a disjunct distribution in West and Central Africa, from Liberia to Côte d’Ivoire, and from Nigeria to Gabon. In Cameroon known from the South-West region.

**Figure 117.**
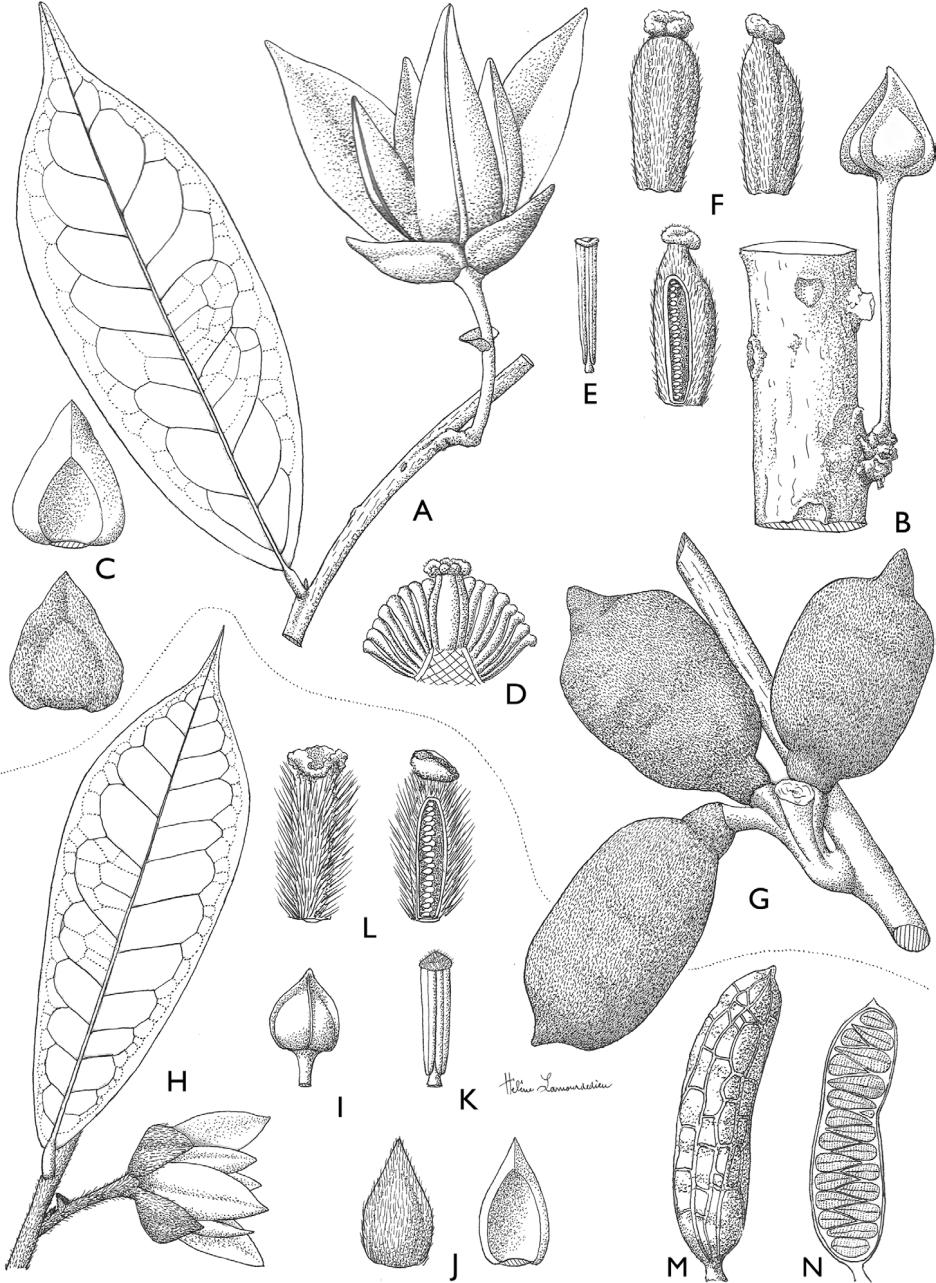
*Uvariastrumpierreanum***A** flowering branch **B** flower, cauliflorous **C** outer petals, inner and outer view **D** longitudinal section of receptacle **E** stamen, front view **F** carpel, front and side view, and detail of ovules **G** fruit. *Uvariastruminsculptum***H** flowering branch **I** flower bud, note reflexed sepal margins **J** outer petals, inner and outer view **K** stamen, front view **L** carpel, front view and detail of ovules **M** detail of monocarp, and **N** longitudinal section of monocarp showing seeds **A, B** from *Letouzey 2670***C–G** from *Le Testu 6083***H** from *Klaine 99***I, J, N** from *Staudt 740***K–M** from *Aubréville 1331*. Drawings by Hélène Lamourdedieu, Publications Scientifiques du Muséum national d’Histoire naturelle, Paris; modified from Le Thomas (1969b, pl. 53, p. 293).

#### Habitat.

A rare species in Cameroon known from three old collections (including the type) plus a more recent one we did not see; in lowland primary and secondary rain forest. Altitude 0–400 m a.s.l.

#### Local and common names known in Cameroon.

None recorded.

#### IUCN conservation status.

Least Concern (LC) (Cosiaux et al. 2019at)

#### Uses in Cameroon.

None reported.

#### Notes.

﻿﻿﻿*Uvariastruminsculptum* is distinguishable by its densely pubescent petioles and upper leaf midrib, and its impressed venation of the upper side of the leaves.

#### Specimens examined.

**South-West Region**: Likomba-Pflanzung 15–35 km NE von Victoria [Limbe], 4.1°N, 9.333°E, *01 December 1928*, *Mildbraed G.W.J.* 10795 (A,K); Johann-Albrechtshöhe[Kumba] area 4.63°N, 9.416°E, *1896*, *Staudt A.* 740 (B,COI,G,K,P,P,S); Johann-Albrechtshöhe[Kumba] area 4.63°N, 9.416°E, *1897*, *Staudt A.* 900 (G).

### ﻿﻿﻿﻿ Uvariastrum pierreanum

Taxon classificationPlantaeMagnolialesAnnonaceae

Engl., Monogr. Afrik. Pflanzen.-Fam. 6: 32, 1901

D37C9FFF-FD24-5E0A-8040-5834197CCDE3

**Figure 118.**
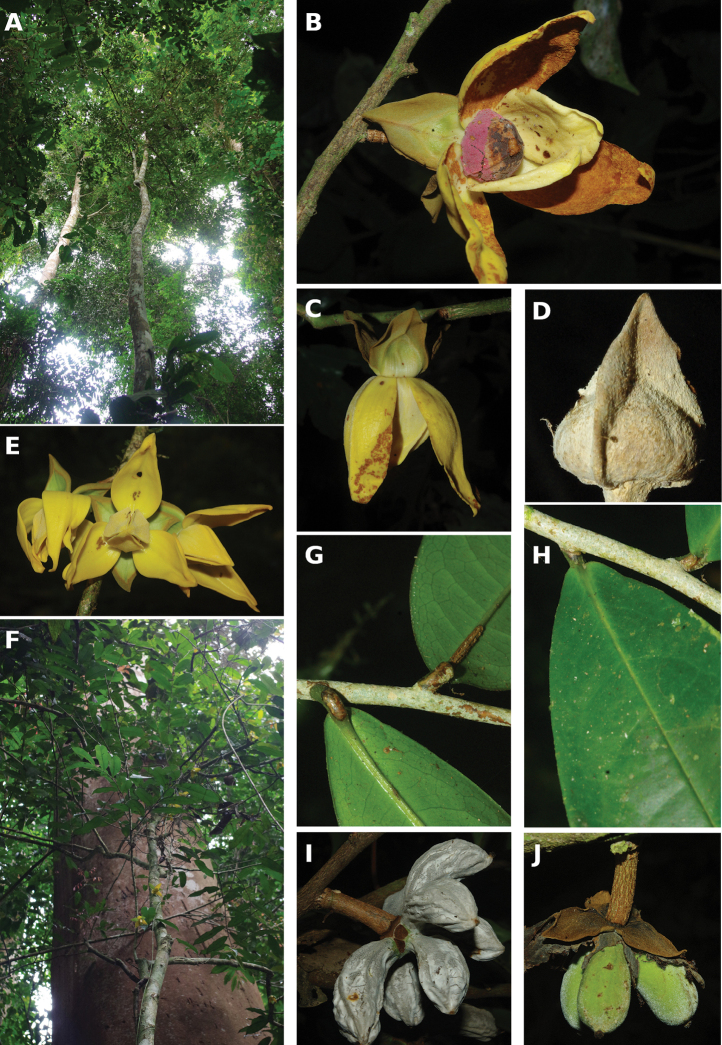
*Uvariastrumpierreanum***A** habit **B** flower, side view, one outer and one inner petal removed **C** flower, side view **D** flower bud, note folded sepal margins **J** young fruits. *Uvariastrumzenkeri***F** habit, *U.zenkeri* is the smaller tree, note yellow flowers **E** flower, top view **G** base of leaf blades, lower side **H** base of leaf blade, upper side, note leaf blade inserted on top of the petiole **I** fruit **A–D***Couvreur 454*, Lélé, Cameroon **E, G, H***Couvreur 624*, Ebo, Cameroon **F***Couvreur 877*, Gabon **I***Couvreur 572*, Gabon **J***Couvreur 590*, Gabon. Photos Thomas L.P. Couvreur.

Figs 117 , 118 ; Map 14I

#### Type.

Gabon. Estuaire; Libreville, *Klaine T.-J. 1091*, Oct 1897: lectotype, designated by Le Thomas (1969b), p. 294: P[P00315822]; isolectotype: B[B1001153112].

#### Description.

Tree to shrub, 4–25 m tall, d.b.h. 40 cm; stilt roots or buttresses absent. Indumentum of simple hairs; **old leafless branches glabrous, young foliate branches sparsely pubescent.** Leaves: petiole 2–4 mm long, 1–2 mm in diameter, sparsely pubescent to glabrous, weakly grooved adaxially, **blade inserted on the side**; blade 6–16 cm long, 2–4.5 cm wide, elliptic to obovate, apex acuminate, acumen 0.7–2 cm long, base decurrent to cuneate, papyraceous to subcoriaceous, below sparsely pubescent to glabrous when young, sparsely pubescent to glabrous when old, above sparsely pubescent to glabrous when young, sparsely pubescent to glabrous when old, concolorous; midrib sunken or flat, above glabrous when young and old, below glabrous when young and old; secondary veins 7 to 12 pairs, glabrous above; tertiary venation reticulate. Individuals bisexual; inflorescences cauliflorous or ramiflorous on young foliate or old leafless branches, axillary. Flowers with 9 perianth parts in 3 whorls, 1 to 3 per inflorescence; pedicel 15–50 mm long, 1–2 mm in diameter, sparsely pubescent to densely pubescent; in fruit 15–50 mm long, 4–6 mm in diameter, sparsely pubescent to glabrous; bract 1, basal, 6 mm long, 6 mm wide; sepals 3, reduplicate-valvate, free, 15–25 mm long, 10–20 mm wide, ovate, apex acute, base truncate, grey-green, pubescent towards margins outside, sparsely pubescent inside, margins revolute; petals free, sub equal; outer petals 3, 25–40 mm long, 10–15 mm wide, elliptic, apex acute, base narrowed, yellow to greyish yellow, margins flat, densely pubescent outside, sparsely pubescent inside; inner petals 3, valvate, 15–28 mm long, 6–15 mm wide, elliptic, apex acute, base cuneate, yellow to greyish yellow, margins flat, densely pubescent outside, sparsely pubescent inside; stamens numerous, 4–6 mm long, linear; connective elongated, pubescent, pinkish red; staminodes absent; carpels free, 5 to 10, ovary 4–6 mm long, stigma bilobed, slightly capitate, densely pubescent. Monocarps shortly stipitate to sessile, stipes 0–4 mm long, 3–5 mm in diameter; monocarps 3 to 5, 90–100 mm long, 40–50 mm in diameter, **ellipsoid to globose, apex rounded, pubescent**, velvety, **smooth, not ribbed**, light green when ripe; seed 16 to 20 per monocarp, 15–25 mm long, 1–2 mm in diameter, flattened ellipsoid; aril absent.

#### Distribution.

A widespread species with a disjunct distribution in west and central Africa, from Guinea and Liberia to Ghana, and from Nigeria to Republic of Congo, and northern Democratic Republic of Congo. In Cameroon known from the East, South, Central and South-West regions.

#### Habitat.

A fairly common species; in primary or secondary lowland rain forest or in gallery forests near savannas, on non-inundated soils or along rivers, occurring on sandy or rocky soils. Altitude 0–600 m a.s.l.

#### Local and common names known in Cameroon.

None recorded.

#### IUCN conservation status.

Least Concern (LC) (Cosiaux et al. 2019au).

#### Uses in Cameroon.

None reported.

#### Notes.

﻿﻿﻿*Uvariastrumpierreanum* is characterized by a combination of light brown sepals and glabrous leaves and smooth monocarps. By the shape of its flowers and glabrous aspect, ﻿*U.pierreanum* resembles ﻿﻿﻿*U.zenkeri*, but the latter has large leathery leaves and its leaf blade is inserted on top instead of on the side of the petiole. In herbarium material, the sepals of ﻿*U.pierreanum* dry brown, whereas those of ﻿﻿﻿*U.zenkeri* dry black.

#### Specimens examined.

**Central Region**: Mefou National Park, 3.61°N, 11.58°E, *13 March 2004*, *Cheek M.* 86 (K,YA). **East Region**: 3 km west of Djembe road head Lobeke Reserve, 2.21°N, 16°E, *16 October 1998*, *Harris D.J.* 5889 (E); Lobeke Reserve Small Bai, 2.28°N, 15.71°E, *01 November 1998*, *Harris D.J.* 6135 (E); A 25 km à l’WSW de Kinsassa village situé à 65 km au NNE de Moloundou sur route de Yokadouma 2.56°N, 15.16°E, *09 March 1971*, *Letouzey R.* 10533 (HGB,P,YA); Forêt au Sud de Dimako rive droite de la rivière Mbonda, 4.26°N, 13.56°E, *18 January 1960*, *Letouzey R.* 2670 (P,YA); A 13 km SSW de Koso (village situé à 60 km au SSW de Batouri), 3.93°N, 14.17°E, *29 July 1963*, *Letouzey R.* 5529 (P,YA); Approximately 10 km N of Welele between Yokadouma and Molundu, 2.68°N, 15.36°E, *18 March 1987*, *Manning D.* 1586 (MO,P,YA); Réserve de Biosphère du Dja vers 1175 m sur la piste reliant la station de Bouamir et l’inselberg de Mbasakok, 3.18°N, 12.81°E, *18 May 2001*, *Senterre B.* 1283 (BR); Réserve de Biosphère du Dja vers 1175 m sur la piste reliant la station de Bouamir et l’inselberg de Mbasakok, 3.18°N, 12.81°E, *19 May 2001*, *Senterre B.* 1370 (BR). **South Region**: Bitye, 3.01°N, 12.35°E, *01 September 1922*, *Bates G.L.* 1764 (K); Campo Ma an National Park 5 km after main entrance, 2.35°N, 10.25°E, *15 February 2012*, *Couvreur T.L.P.* 385 (WAG,YA); 1 km from main camp near Lélé river 16 km East from Lélé village, 2.28°N, 13.32°E, *07 September 2013*, *Couvreur T.L.P.* 454 (WAG,YA); Campo Ma’an National Park 11 km on trail from Ebinanemeyong village on road 7 km from Nyabessan to Campo town, 2.49°N, 10.34°E, *12 February 2015*, *Couvreur T.L.P.* 680 (WAG,YA); Ca 30 km S of Mbalmayo, 3.29°N, 11.44°E, *13 February 1964*, *de Wilde J.J.F.E* 1904 (B,BR,K,MO,P,WAG,YA); Station de cacaoyer de N’koemvone 14 km On the road from Ebolowa to Ambam, 2.81°N, 11.13°E, *18 February 1975*, *de Wilde J.J.F.E* 7972 (B,BR,K,MO,P,U,WAG,YA); Meyo Centre, 2.55°N, 11.03°E, *24 March 1970*, *Letouzey R.* 10225 (P).

### ﻿﻿﻿﻿ Uvariastrum zenkeri

Taxon classificationPlantaeMagnolialesAnnonaceae

Engl. & Diels, Bot. Jahrb. Syst. 34: 473, 1907

D54ADF8D-36B8-5C50-8A6D-7D9EABCBB052

**Figure 119.**
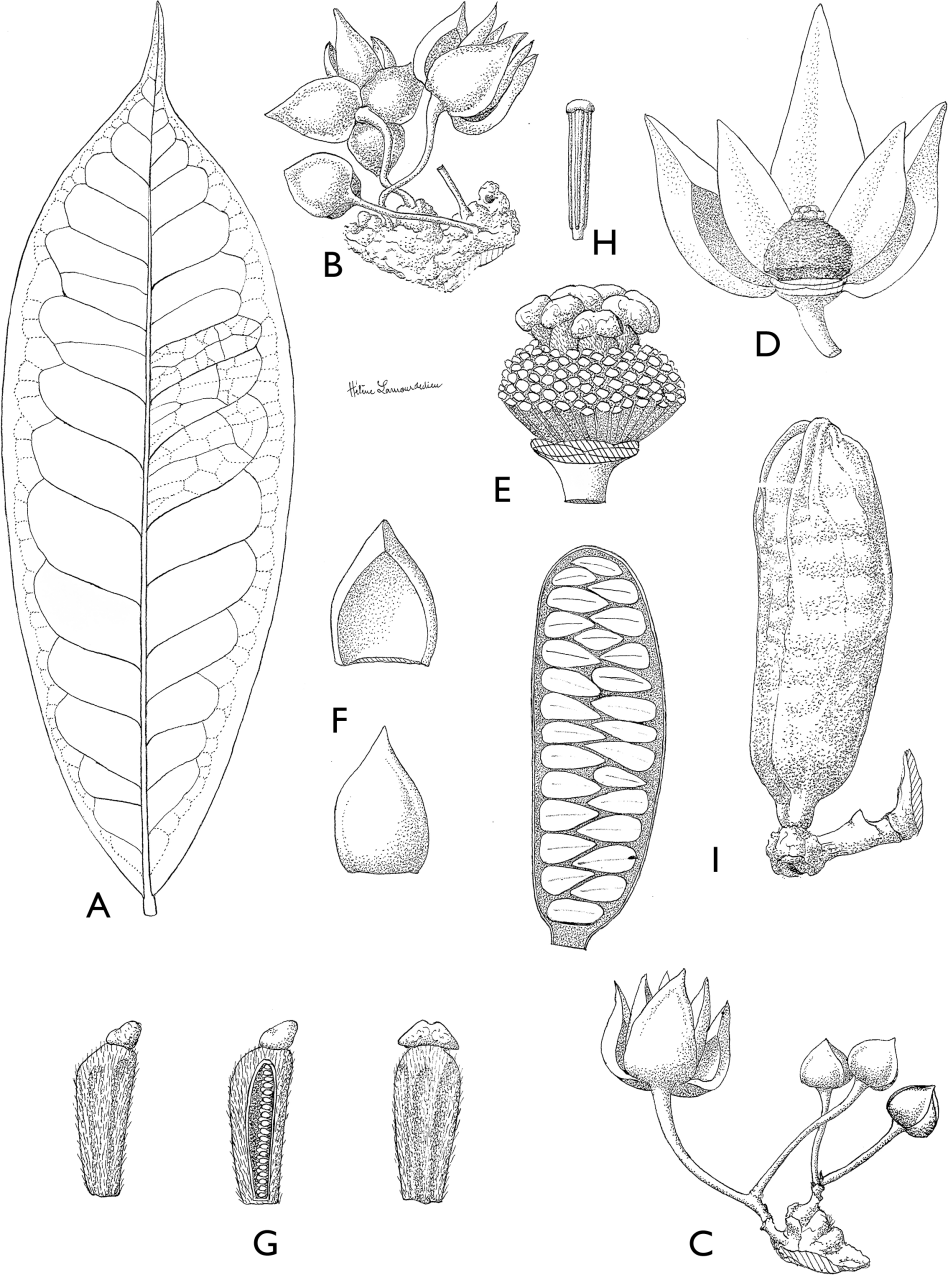
*Uvariastrumzenkeri***A** leaf **B, C** cauliflorous flowers **D** detail of flower with three petals removed **E** detail of receptacle **F** sepals **G** carpels **H** stamens **I** detail of monocarp. Drawings by Hélène Lamourdedieu, Publications Scientifiques du Muséum national d’Histoire naturelle, Paris; modified from Le Thomas (1969b, pl. 52, p. 291, pro parte).

**Map 15.**
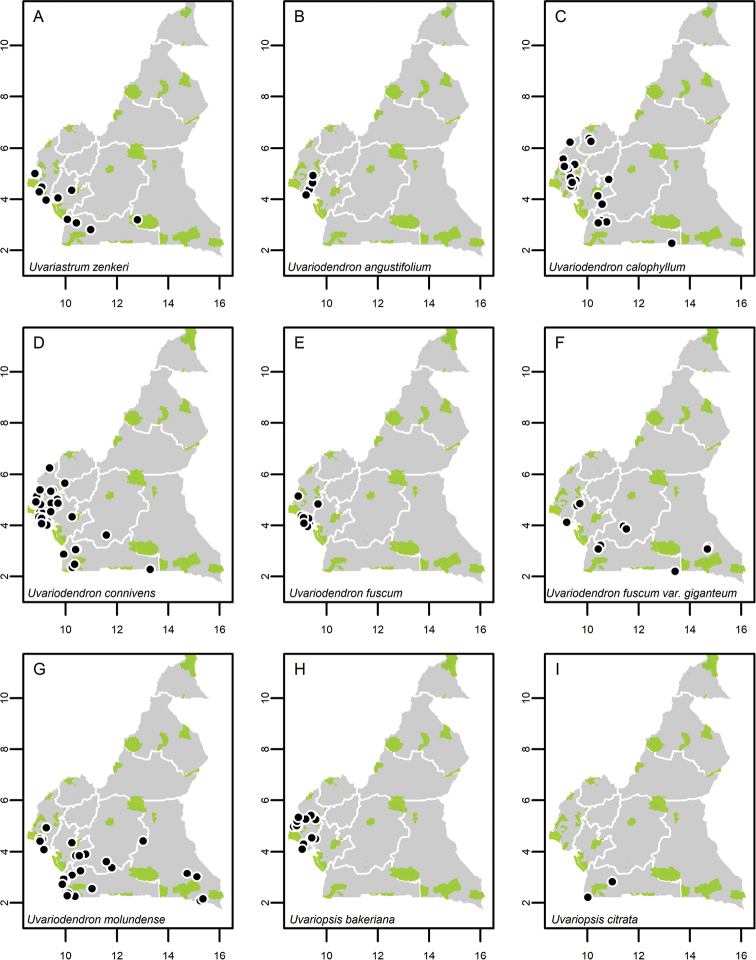
**A***Uvariastrumzenkeri***B***Uvariodendronangustifolium***C***Uvariodendroncalophyllum***D***Uvariodendronconniven*s **E***Uvariodendronfuscum***F**Uvariodendronfuscumvar.giganteum**G***Uvariodendronmolundense***H***Uvariopsisbakeriana***I***Uvariopsiscitrata*. White borders represent region limits in Cameroon; green patches represent protected areas (see methods and Suppl. material 1: Fig. S1).

Figs 118 , 119 ; Map 15A

= Uvariastrum zenkeri Engl. & Diels var. nigritanum Baker f., Cat. Talbot’s Plants 3, 1913. Type. Nigeria. Cross River State: Oban district, recd. at Paris 21 Feb 1912, Talbot P.A. 1341; lectotype, designated by Couvreur (2014), p. 28: K[K001081866]; isotypes: FHO [accession number 15560, barcode 3586]; P [P01983332].
= Uvariastrum pynaertii De Wild., Ann. Mus. Congo Belge, Bot. sér. 5, 3(1): 74, 1909. Type. Democratic Republic of the Congo. Equateur, Eala, Pynaert L.A. 1234, Mar 1907: lectotype designated by Le Thomas (1969b), p. 292, sheet designated by Couvreur (2014), p. 28: BR[BR0000008824288]; isolectotypes: BR[BR0000008824295, BR0000008824301]; S[S12-22788].

#### Type.

Cameroon. South Region; Bipindi, *Zenker G.A. 2935*, 1904: lectotype, designated by Couvreur (2014), p. 28: B[B100153114]; isolectotypes: B[B100190283]; BM,[BM000554069]; BR[BR0000014035722]; COI[COI00077201]; GOET[GOET005731]; G[G00011742, G00011744]; K[K000198808]; L[L0191076]; M[M0089220]; MA[MA215566-3]; P[P00315826]; S[S12-22789]; WAG[WAG0057973]; WU[WU0025789]; Z [Z000034578, Z000034577].

#### Description.

Tree to shrub, 20–30 m tall, d.b.h. 20–30 cm; stilt roots or buttresses absent. Indumentum of simple hairs; old leafless branches glabrous, young foliate branches sparsely pubescent to glabrous. Leaves: petiole 2–3 mm long, ca. 2 mm in diameter, sparsely pubescent, not grooved, **blade inserted on top of the petiole**; blade 12–22 cm long, 3–5 cm wide, elliptic to obovate, apex acuminate, acumen 1–2 cm long, base cuneate to rounded, coriaceous, below sparsely pubescent when young, glabrous when old, above glabrous when young and old, concolorous; midrib sunken or flat, above pubescent when young, glabrous when old, below sparsely pubescent to densely pubescent when young, sparsely pubescent to densely pubescent when old; secondary veins 11 to 17 pairs, glabrous above; tertiary venation reticulate. Individuals bisexual; inflorescences cauliflorous or ramiflorous on young foliate or old leafless branches, axillary. Flowers with 9 perianth parts in 3 whorls, 1 to 2 per inflorescence; pedicel 13–30 mm long, 1–2 mm in diameter, sparsely pubescent to glabrous; in fruit 15–30 mm long, 2–5 mm in diameter, glabrous; bracts 3, all basal, 5 mm long, 3–4 mm wide; sepals 3, reduplicate-valvate, free, 15–25 mm long, 8–15 mm wide, ovate, apex acute, base truncate, light brown, sparsely pubescent to glabrous outside, pubescent and glabrous towards center inside, margins recurved; petals free, sub equal; outer petals 3, 20–35 mm long, 8–15 mm wide, elliptic to ovate, apex acute, base truncate, yellow, margins flat, densely pubescent outside, pubescent except towards center inside; inner petals 3, valvate, 15–25 mm long, 7–10 mm wide, ovate, apex acute, base cuneate, claw mm long, white turning bright yellow to orange, light grey when old, margins flat, densely pubescent outside, pubescent except towards center inside; stamens numerous, 2–4 mm long, linear; connective discoid, pubescent, red; staminodes absent; carpels free, (1–3) 5 to 15, ovary 3–5 mm long, stigma capitate, sparsely pubescent to glabrous. Monocarps shortly stipitate, stipes 2–5 mm long, ca. 5 mm in diameter; monocarps 2 to 5, 80 mm long, 25 mm in diameter, oblong, apex acute, **sparsely pubescent to glabrous, longitudinally 4 to 6 ribbed, sometimes more finely ribbed**, light green-grey with dashed of white when ripe; seeds 20 to 27 per monocarp, 15–20 mm long, 10–15 mm in diameter, flattened ellipsoid; aril absent.

#### Distribution.

A widespread species in Central Africa, from extreme southeast of Nigeria to the Republic of Congo, and Democratic Republic of Congo. In Cameroon known from the East, South, Central, South-West regions.

#### Habitat.

A common species, in primary or secondary lowland rain forest, mainly on non-inundated soils, but also on marshy or sandy soils. Altitude 0–400 m a.s.l.

#### Local and common names known in Cameroon.

None recorded.

#### IUCN conservation status.

Least Concern (LC) (Cosiaux et al. 2019av).

#### Uses in Cameroon.

None reported.

#### Notes.

﻿﻿﻿*Uvariastrumzenkeri* is easily distinguished by the sepals that dry black and the large leaves with the blade inserted on top of the petiole. The black color of the dried sepals is related to their glabrous or sparsely pubescent outer side, whereas it is pubescent in ﻿*U.insculptum* and ﻿*U.pierreanum*.

#### Specimens examined.

**East Region**: Dja reserve, 3.20°N, 12.79°E, *04 November 1994*, *Fogiel M.K.* 1039 (MO,P). **Littoral Region**: Ebo Wildlife Reserve Djuma permanent camp, 4.34°N, 10.23°E, *14 February 2014*, *Couvreur T.L.P.* 624 (WAG,YA); Douala (Route Razel), 4.05°N, 9.7°E, *01 January 1955*, *Endengle E.* s.n. (P,YA). **South Region**: 41 kmN of Kribi 5 km E of Edea road forest track Fifinda-Bella old secondary forest, 3.21°N, 10.06°E, *06 February 1970*, *Bos J.J.* 6266 (BR,C,K,LD,P,WAG,YA); Entre 15 et 25 km au SW de Zingui (soit à 45 km au SSE de Kribi), 2.82°N, 10.97°E, *22 March 1968*, *Letouzey R.* 9121 (P,YA); Bipindi, 3.08°N, 10.42°E, *01 January 1902*, *Zenker G.A.* 2438 (B,COI,G,K,L,MO,P,S,WAG); Bipindi, 3.08°N, 10.42°E, *01 January 1904*, *Zenker G.A.* 2935 (B,BM,COI,G,K,L,M,P,WAG); Bipindi, 3.08°N, 10.41°E, *01 January 1906*, *Zenker G.A.* 3248 (K); Bipindi, 3.08°N, 10.42°E, *01 January 1907*, *Zenker G.A.* 3289 (B,L,P,S); Bipindi, 3.08°N, 10.42°E, *01 January 1908*, *Zenker G.A.* 3409 (COI,G,L,M,MO,P,S); Bipindi, 3.08°N, 10.42°E, *01 January 1912*, *Zenker G.A.* 4473 (B,COI,G,L,M,P,S); Bipindi, 3.08°N, 10.41°E, *01 January 1914*, *Zenker G.A.* 481 (B,BR,C,F,G,GH,M,P,S,U,WAG). **South-West Region**: Mabeta Moliwe, 3.96°N, 9.233°E, *02 April 1992*, *Bongyu J.* 42 (K,P); Korup National Park, 5.06°N, 8.855°E, *12 January 1998*, *Kenfack D.* 1008 (MO,P,WAG); Boa, 4.4°N, 9°E, *04 May 1994*, *Ndam N.* 1248 (K); Mokoko Forest Reserve, 4.46°N, 9.066°E, *19 April 1994*, *Tchouto Mbatchou G.P.* 1108 (K); Ndian River at PAMOL field 69 and transect P, 5.01°N, 8.833°E, *24 January 1985*, *Thomas D.W.* 4334 (K,MO,P,YA); Korup National Park, 4.98°N, 8.85°E, *12 January 1979*, *Thomas D.W.* 604 (K); Korup National Park, 5.05°N, 8.766°E, *10 June 1988*, *Thomas D.W.* 8094 (L,P,WAG,YA); West bank of the Onge River, 4.28°N, 8.966°E, *07 November 1993*, *Thomas D.W.* 9772 (K,MO,P,WAG,YA); Korup National Park, 5°N, 8.8°E, *04 February 2000*, *van der Burgt X.M.* 590 (G,WAG,YA); Korup National Park, 5°N, 8.8°E, *19 March 2004*, *van der Burgt X.M.* 674 (BR,G,K,MO,P,WAG,YA).

### ﻿﻿ Uvariodendron

Taxon classificationPlantaeMagnolialesAnnonaceae

(Engl. & Diels) R.E.Fr., Acta Horti Berg. 10: 51, 1930

1E34C942-2AFD-5784-9234-F5F1EE8C7FDA

= Uvaria L. sect. ﻿Uvariodendron Engl. et Diels in Engl., Monogr. Afr. Pilanzenf. 6: 8, 1901.

#### Type species.

﻿﻿﻿*Uvariodendronfuscum* (Benth.) R.E.Fr. (≡ ﻿﻿*Uvariagigantea* Engl.)

#### Description.

Trees, 3–20 m tall, d.b.h. up to 20 cm; stilt roots or buttresses absent. Indumentum of simple hairs or absent. Leaves: petiole 4–20 mm long, 1–9 mm in diameter; blade 13–70 cm long, 3.6–15 cm wide, ovate or elliptic or obovate or oblong, apex acuminate to emarginate, acumen 0.7–2 cm long, base acute to rounded; midrib sunken or flat; secondary veins 11 to 36 pairs per side; tertiary venation reticulate. Individuals bisexual; inflorescences cauliflorous or ramiflorous on old leafless branches, axillary, 1 to 3 per inflorescence; pedicel 1–19 mm long; in fruit 6–38 mm long; bracts 2 to 6, several basal and one upper towards the middle of the pedicel; sepals 3, valvate, basally fused, 6–35 mm long, apex acuminate, base truncate; petals free, sub equal; outer petals 3, valvate, 12–33 mm long, 8–25 mm wide, ovate, apex acuminate, base truncate; inner petals 3, valvate, 6.5–26.5 mm long, 5–17 mm wide, ovate, apex acute, base truncate; stamens 200 to 5000, 2–5 mm long, linear to narrowly oblong; connective discoid; staminodes absent; carpels free, 6 to 104, 3–7 mm long, stigma bilobed. Monocarps sessile or shortly stipitate; monocarps (1) 5 to 63, 20–70 mm long, 12–30 mm in diameter, globose or ellipsoid or oblong, apex acute or rounded; seeds 6–29 mm long, 4–21 mm in diameter, ellipsoid to flattened ellipsoid or oblong; aril absent.

#### Taxonomy.

Le Thomas (1969b); Dagallier et al. (in prep.).

A genus of 14 species from West, Central and East Africa; in Cameroon five species, none endemic.

### ﻿Key to the species of ﻿*Uvariodendron* in Cameroon:

**Table:** 

1	Crushed leaves with strong lemon scent; leaf blades 10–19.9 cm long; leaves invariably narrowly elliptic	﻿***U.angustifolium***
–	Crushed leaves without strong lemon scent; leaf blades 16–76.5 cm long (largest leaves with blade generally > 20 cm long); leaves narrowly elliptic to narrowly obovate to obovate	**2**
2	Young branches, petioles, and mid rib below the blade covered with a brown tomentum, generally persisting on older branches	﻿***U.calophyllum***
–	Young branches, petioles, and mid rib below the lamina pubescent covered with long soft hairs to glabrous	**3**
3	Young branches invariably glabrous; flowering pedicel ≥ 10 mm, petals wine red outside and inside; monocarps sparsely pubescent to glabrous	﻿﻿***U.connivens***
–	Young branches pubescent to glabrous; flower pedicel ≤ 15 mm, petals wine red cream to light yellow outside, cream with dark red steak inside; monocarps pubescent to sparsely pubescent	**4**
4	Bracts 3–8 mm long and 3–10 mm wide; sepals free and imbricate, 5–9 mm long and 5–10 mm wide	﻿***U.molundense***
–	Bracts 8–22 mm long and 10–50 mm wide; sepals fused at base over 20–50% of their length, 11–30 mm long and 13–26 mm wide	**5**
5	Young branches glabrous to pubescent; leaf blade 15.9–45 cm long, 15 to 24 secondary veins; sepals 11–23 mm long, petals 20–42 mm long and 15–26 mm wide; carpels 20 to 70	﻿**U.fuscumvar.fuscum**
–	Young branches with long whitish hairs, generally falling off with age; leaf blade 30–70 cm long, 22 to 33 secondary veins; sepals 21–30 mm long, petals 25–40 mm long and 20–30 mm wide; carpels 33 to 104	﻿﻿**U.fuscumvar.giganteum**

### ﻿﻿﻿﻿ Uvariodendron angustifolium

Taxon classificationPlantaeMagnolialesAnnonaceae

(Engl. & Diels) R.E.Fr., Acta Horti Berg. 10: 58, 1930

100F39AA-D508-5C87-8F63-BE823BEEC1A0

Fig. 120 ; Map 15B

≡ Uvaria angustifolia Engl. & Diels, Notizbl. Königl. Bot. Gart. Berlin 2: 295, 1899.

#### Type.

Cameroon. South-West Region; Johann-Albrechtshöhe[Kumba], *Staudt A. 742a*, 20 Mar 1896; holotype: B[B 10 0153115].

#### Description.

Tree to shrub, 3–12 m tall, d.b.h. unknown; stilt roots or buttresses absent. Indumentum of simple hairs; old leafless branches glabrous, young foliate branches glabrous to pubescent. Leaves: petiole 3–7.5 mm long, 1–2 mm in diameter, glabrous to pubescent, grooved, blade inserted on top of the petiole; **blade 10–19.9 cm long, 3–5.8 cm wide, narrowly elliptic**, apex acute to acuminate, acumen 1.1–1.4 cm long, base acute to cuneate, subcoriaceous, below glabrous to pubescent at base, glabrous when old, above glabrous when young and old, **lemon-scented when crushed**; midrib sunken or flat, above glabrous when young and old, below glabrous to pubescent at base when young and old; secondary veins 8 to 14 pairs per side, tertiary venation reticulate. Individuals bisexual; inflorescences ramiflorous on old or young foliate branches, axillary. Flowers with 9 perianth parts in 3 whorls, 1 to 2 per inflorescence; **pedicel 0–6 mm long**, 2–3 mm in diameter, densely pubescent; in fruit ca. 5 mm long, ca. 3.5 mm in diameter; bracts 2 to 6, several basal and one upper towards the middle of pedicel, basal bracts 2–3 mm long, 1–3 mm wide; upper bract 6–11 mm long, 9–15 mm wide; sepals 3, valvate, basally fused, imbricate at the middle, 9–13 mm long, 9–13 mm wide, ovate, apex acuminate, base truncate, densely pubescent outside, glabrous inside, margins flat; petals free, sub equal; outer petals 3, 15–21 mm long, 9–15 mm wide, ovate, apex acuminate, base truncate, margins flat, densely pubescent outside, glabrous inside; inner petals 3, valvate, 15–22 mm long, 5–10 mm wide, ovate, apex acuminate, base truncate, margins flat, densely pubescent outside, glabrous inside; stamens 200 to 300, in 10 to 15 rows, 3–3.5 mm long, narrowly oblong; connective discoid; staminodes absent; carpels free, 7 to 30, ovary 3.5–4.5 mm long, stigma bilobed, slightly capitate, densely pubescent. Monocarps shortly stipitate, stipes 1–3 mm long, ca. 3 mm in diameter; monocarps 2 to 10, 23–40 mm long, 17–30 mm in diameter, ellipsoid to globose, yellow when ripe emitting strong lemon smell; seeds 9 to 18 per monocarp, 21–28 mm long, ca. 10 mm in diameter, ellipsoid to oblong; aril absent.

**Figure 120.**
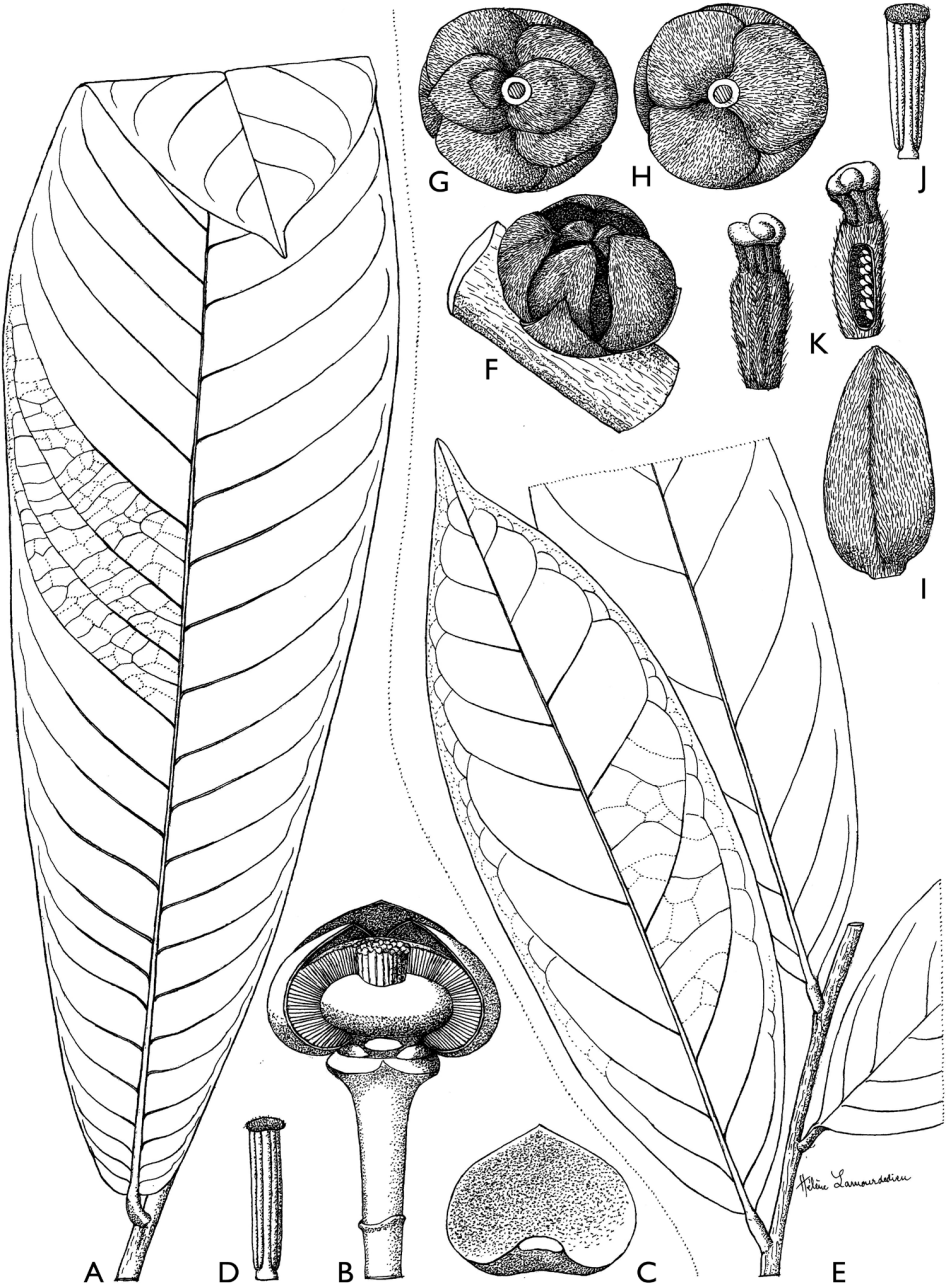
*Uvariodendronconnivens***A** leaf **B** flower, two outer and one inner petal removed **C** outer petal inner view **D** stamen, front view. *Uvariodendronaugustifolium***E** leaves **F** flower, semi top view **G** flower, bottom view showing bracts **H** flower, bottom view, bracts removed **I** outer petal, outer view **J** stamen, front view **K** carpel, front view and detail of ovules **A–D** from *Mann 1159***A–K** from *Vigne 1610*. Drawings by Hélène Lamourdedieu, Publications Scientifiques du Muséum national d’Histoire naturelle, Paris; modified from Le Thomas (1969b, pl. 51, p. 281).

#### Distribution.

A species with a disjunct distribution in West Africa (Ghana, Ivory Coast) and Central Africa (Nigeria, Cameroon); in Cameroon, known from the South-West region.

#### Habitat.

A rare species, only collected five times in Cameroon, the last collection from 1987. In lowland primary or secondary rain forest. Altitude 200–400 m a.s.l.

#### Local and common names known in Cameroon.

None recorded.

#### IUCN conservation status.

Not evaluated.

#### Uses in Cameroon.

None reported.

#### Notes.

﻿﻿﻿*Uvariodendronangustifolium* is distinguished by its narrowly elliptic and relatively small leaves (13–16 cm *versus* > 16 cm) compared to the other species in Cameroon.

Two collections, *Thomas 6087* & *7018*, record that this species emits a strong-lemon scent from the leaves when fresh, which is also recorded in ﻿﻿﻿﻿Uvariodendronmolundensevar.citrata Le Thomas, a variety endemic to Gabon (Le Thomas 1969b) and in ﻿﻿﻿*Uvariopsiscitrata* Couvreur & Niangadouma (Couvreur and Niangadouma 2016).

#### Specimens examined.

**South-West Region**: Between Bafia and Likoko, 4.37°N, 9.324°E, *05 February 1958*, *Keay R.W.J.* 37524 (K); Kumba, 4.63°N, 9.416°E, *1896*, *Staudt A.* 642 (K); Johann-Albrechtshöhe[Kumba] area 4.16°N, 9.2°E, *28 March 1896*, *Staudt A.* 742 (B,K); Lake Barombi Kumba, 4.64°N, 9.45°E, *01 April 1986*, *Thomas D.W.* 6087 (YA); Along the road between Konye and Bakole, 4.91°N, 9.466°E, *25 May 1987*, *Thomas D.W.* 7018 (YA).

### ﻿﻿﻿﻿ Uvariodendron calophyllum

Taxon classificationPlantaeMagnolialesAnnonaceae

R.E.Fr., Acta Horti Berg. 10: 63, 1930

99BEEA15-3DF7-517E-8247-62825A05F29C

Fig. 121 ; Map 15C

#### Type.

Cameroon. South Region; Bipindi, *Zenker G.A. 2344*, 1901: holotype: B[B 10 0153116]; isotypes: BM[BM000636669]; G[G00412241]; GOET[GOET005732]; HBG[HBG502513]; K[K000198797, K000198796]; M[M0107940]; P[P00362661, P00362658, P00362659]; S[S07-13396, S07-13393]; WAG[WAG.1418666].

#### Description.

Tree, 2–20 m tall, d.b.h. 20–35 cm; stilt roots or buttresses absent. Indumentum of simple hairs; old leafless branches sparsely pubescent, **young foliate branches densely pubescent to brown tomentose.** Leaves: petiole 4–25 mm long, 2–9 mm in diameter, **densely pubescent to brown tomentose**, slightly grooved, blade inserted on top of the petiole; **blade 25.8–76.5 cm long, 6.1–24.8 cm** wide, oblong to obovate, apex acuminate to emarginate, acumen 0.6–3.2 cm long, base rounded, coriaceous, below glabrous to pubescent at base when young and old, above glabrous when young and old; midrib sunken or flat, above glabrous when young and old, below densely pubescent when young and old; secondary veins 19 to 40 pairs per side, glabrous above, slightly pubescent to pubescent below; tertiary venation reticulate. Individuals bisexual; inflorescences cauliflorous or ramiflorous on old or young foliate branches, axillary. Flowers with 9 perianth parts in 3 whorls, 1 to 3 per inflorescence; pedicel 0–9 mm long, 4–7 mm in diameter, tomentose; in fruit 1–9 mm long, ca. 4–7 mm in diameter, tomentose; bracts 2–6, several basal and one upper towards the middle of pedicel, basal bracts 2–3 mm long, 1–3 mm wide; upper bract 10–23 mm long, 10–40 mm wide; sepals 3, valvate or imbricate, basally fused, 10–27 mm long, 10–26 mm wide, ovate, apex acuminate, base truncate, brown, **densely pubescent to brown tomentose** outside, pubescent except towards center inside, margins flat; petals free, sub equal; outer petals 3, 15–37 mm long, 10–28 mm wide, ovate, apex acuminate, base truncate, greyish yellow, margins flat, **densely pubescent to brown tomentose** outside, glabrous but pubescent towards margins inside; inner petals 3, valvate, 14–34 mm long, 11–20 mm wide, ovate, apex acuminate, base truncate, light yellow to cream, margins flat, **densely pubescent to brown tomentose** outside, pubescent towards margins inside; stamens 3680 to 5256, in 25 to 35 rows, 3.5–4.5 mm long, linear; connective discoid, glabrous, cream; staminodes absent; carpels free, 23 to ca. 150, ovary 4–5 mm long, stigma bilobed, slightly capitate, densely pubescent. Monocarps sessile to stipitate, stipes 0–5 mm long, 2.5–3 mm in diameter; 3 to 35 monocarps, 27–55 mm long, 9–25 mm in diameter, ellipsoid, apex rounded, tomentose, bumpy, otherwise smooth, brown when ripe; seeds 7 to 13 per monocarp, ca. 13 mm long, 8–10 mm in diameter, ellipsoid to oblong; aril absent.

#### Distribution.

A widespread species with a disjunct distribution in West and Central Africa, from Côte d’Ivoire to Ghana, and from Nigeria to Cameroon. In Cameroon known from the South, Central, Littoral, South-West and North-West regions.

#### Habitat.

A fairly common species; in lowland primary or old secondary rain forests, near streams. Altitude 50–30 m a.s.l.

#### Local and common names known in Cameroon.

None recorded.

#### IUCN conservation status.

Least Concern (LC) (Cosiaux et al. 2019af).

#### Uses in Cameroon.

None reported.

#### Notes.

﻿﻿﻿*Uvariodendroncalophyllum* is distinguished by a dense brown tomentose pubescence on the young foliate branches, petioles, flowering pedicels, and outer side of the sepals and petals. ﻿﻿﻿﻿﻿Uvariodendronfuscumvar.giganteum also has a dense pubescence on the young foliate branches, but the hairs are long and white, and soon disappearing with age. Stamen count was taken from Meinke (2008).

#### Specimens examined.

**Central Region**: Sonossi 26 km W of Ndikinimeki, 4.77°N, 10.83°E, *29 March 1982*, *Asonganyi J.N.* 421 (P,YA); Colline entre Tcherikoy et Sokelle II (30 km NW Eséka), 3.80°N, 10.56°E, *14 December 1973*, *Letouzey R.* 12352 (P,YA). **Littoral Region**: 8 km W of Masok, 4.13°N, 10.4°E, *31 March 1965*, *Leeuwenberg A.J.M.* 5282 (BR,K,MO,P,WAG,YA). **North-West Region**: Baji-Tumbo (Wum), 6.38°N, 10.07°E, *12 July 1975*, *Letouzey R.* 14020 (K,P,WAG,YA); Bamenda Prov Wum Distr Nkom-Wum FR, 6.26°N, 10.13°E, *03 July 1951*, *Ujor E.U.* 29281 (K). **South Region**: on road Lolodorf-Bipindi ca half way near Mbiguiligui village (Mbikiliki), 3.16°N, 10.53°E, *26 February 2018*, *Couvreur T.L.P.* 1157 (K,MPU,P,WAG,YA); 31 km east from Lélé village, 2.27°N, 13.29°E, *09 September 2013*, *Couvreur T.L.P.* 486 (WAG,YA); ca 7 km NE of Ebom, 3.11°N, 10.75°E, *01 August 1996*, *Parren M.P.E.* 223 (KRIBI,WAG); ca 7 km NE of Ebom, 3.11°N, 10.75°E, *01 August 1996*, *Parren M.P.E.* 68 (KRIBI,WAG); Bipindi, 3.08°N, 10.42°E, *1898*, *Zenker G.A.* 1738 (B,M,P,WAG); Bipindi, 3.08°N, 10.41°E, *01 January 1901*, *Zenker G.A.* 2344 (B,L,M,P,WAG); Bipindi, 3.08°N, 10.42°E, *01 April 1903*, *Zenker G.A.* s.n. (P). **South-West Region**: Along the path from Pete to Bopo at the right hand side of the road in S Bakundu FR, 4.46°N, 9.392°E, *23 February 1956*, *Binuyo A.* 35564 (K,WAG); Mungo river forest reserve ca 1 km East of bridge Chained road to S, 4.73°N, 9.55°E, *24 October 1998*, *Cheek M.* 9337 (K,YA); Bayang Mbo Wildlife Sanctuary after Mbu river, 5.35°N, 9.500°E, *26 March 2016*, *Couvreur T.L.P.* 1013 (WAG,YA); on top of hill near Small Ekombe village 3 km after Kumba on road to Ekondo Titi town, 4.62°N, 9.376°E, *13 January 2016*, *Couvreur T.L.P.* 980 (WAG,YA); Bayang Mbo Wildlife Sanctuary after Mbu river, 5.35°N, 9.500°E, *25 March 2016*, *Couvreur T.L.P.* 999 (WAG,YA); South Bakundu, 4.49°N, 9.374°E, *19 February 1946*, *Dundas J.* 13989 (K); Entre Babong et Okurikang 35 km WSW Mamfé, 5.58°N, 9.05°E, *29 May 1975*, *Letouzey R.* 13673 (P,YA); Bolo forest 5 km west of Kumba-Mamfe road near Konye, 4.64°N, 9.45°E, *25 March 1986*, *Nemba J.* 64 (K,MO,P,WAG,YA); Kumba Distr Mumbo-Southern Bakossi, 4.83°N, 9.333°E, *09 May 1951*, *Olorunfemi J.* 30561 (K); S Bakundu FR between Bombe Rest House and Mbalange, 4.46°N, 9.452°E, *19 March 1953*, *Onochie C.F.A.* 30860 (K); Korup National Park, 5.26°N, 9.183°E, *24 March 1984*, *Thomas D.W.* 3322 (K,P,YA); Takamanda Forest Reserve, 6.23°N, 9.316°E, *21 March 1985*, *Thomas D.W.* 4549 (YA); 31 km West of Kumba on Mbonge road, 4.51°N, 9.366°E, *26 March 1986*, *Thomas D.W.* 5965 (K,MO,P,WAG,YA); Lake Barombi Kumba, 4.65°N, 9.4°E, *01 April 1986*, *Thomas D.W.* 6090 (P,YA); Korup National Park, 5.27°N, 9.1°E, *01 April 1988*, *Thomas D.W.* 7499 (P).

**Figure 121.**
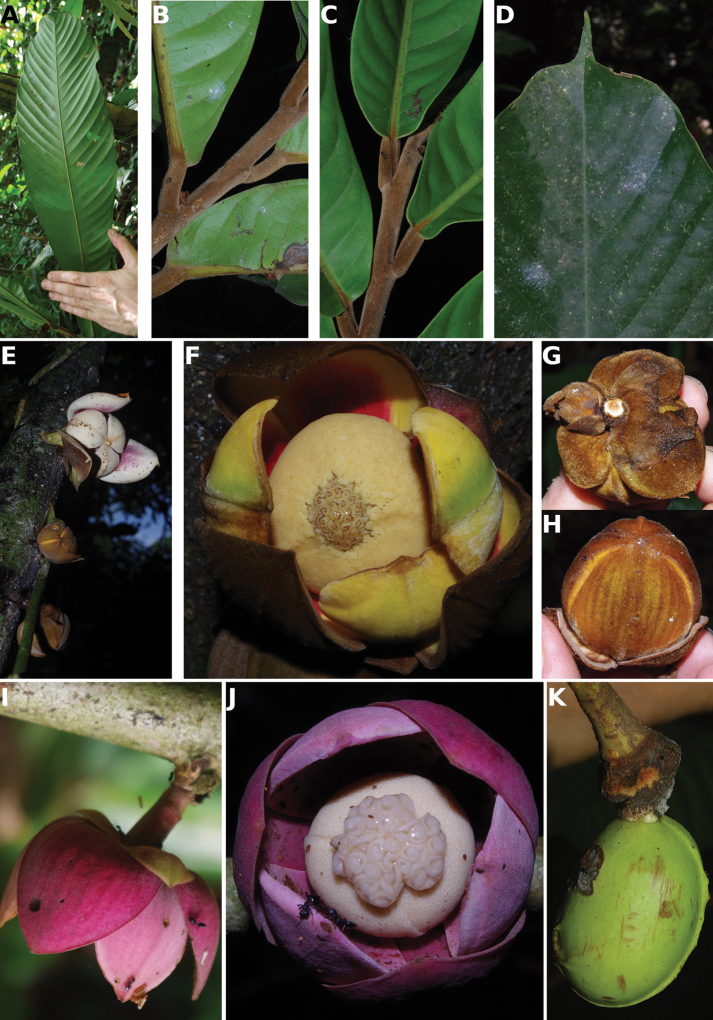
*Uvariodendroncalophyllum***A** leaf, hand for size **B** base of leaf blades, lower side **C** base of leaf blades, upper side, note tomentose pubescence **D** leaf blade apex **E** cauliflorous flowers, buds and one at anthesis **F** detail of flower, stamens and carpels **G** flower, bottom view, note tomentose pubescence **H** flower bud, side view. *Uvariodendronconnivens***I** flower, at anthesis, note long pedicel **J** flower, top view, note wine red purple petals **K** fruit, with a single monocarp **A, D, G, H***Couvreur 1157*, Nogovayang, Cameroon **B, C***Couvreur 999*, Bayang Mbo, Cameroon **E, F***Couvreur 1013*, Bayang Mbo, Cameroon **I, J***Couvreur 1016*, Bayang Mbo, Cameroon **K***Couvreur 620*, Ebo, Cameroon. Photos Thomas L.P. Couvreur.

### ﻿﻿﻿﻿ Uvariodendron connivens

Taxon classificationPlantaeMagnolialesAnnonaceae

(Benth.) R.E.Fr., Acta Horti Berg. 10: 55, 1930

25CC06EF-7F46-5E69-9421-68AE418B1BDC

Figs 120 , 121 ; Map 15D

≡ Uvaria connivens Benth., Trans. Linn. Soc. London 23(3): 465, 1862.
= Uvaria megalantha Diels, Bot. Jahrb. Syst. 39: 472, 1907. Type. Cameroon. South Region, Bipindi, Zenker G.A. 3204, 1904: lectotype, here designated: WAG[WAG0057972]; isolectotypes: BM[BM000636652]; GEOT[GOET005733]; K[K000198800]; M[M0107939]; P[P01982908]; S[S07-13392].

#### Type.

Equatorial Guinea. Bioko Norte; Bioko (Fernando Po), *Mann G. 1159*, 1861: lectotype, here designated: K[K000198803]; isolectotypes: K[K000198804, K000198805]; P[P00362655].

#### Description.

Tree, 3–20 m tall, d.b.h. 2–25 cm; stilt roots or buttresses absent. Indumentum of simple hairs; old leafless branches glabrous, young foliate branches glabrous. Leaves: petiole 4.5–21 mm long, 2–6 mm in diameter, glabrous, grooved, blade inserted on top of the petiole; **blade 25.4–63.6 cm long**, 7–17.7 cm wide, **narrowly elliptic to narrowly oblong**, apex acuminate, acumen 0.7–2.4 cm long, **base rounded** (sometimes acute, truncate or subcordate), subcoriaceous, below glabrous when young and old, above glabrous when young and old; midrib sunken or flat, above glabrous when young and old, below glabrous when young and old; secondary veins 15 to 29 pairs, glabrous above; tertiary venation reticulate. Individuals bisexual; inflorescences cauliflorous or ramiflorous on old or young foliate branches, axillary. Flowers with 9 perianth parts in 3 whorls, 1 per inflorescence; **pedicel 5–40 mm long**, 1.4–4 mm in diameter, glabrous to pubescent; in fruit 13–31 mm long, 3–8 mm in diameter, glabrous; bracts 2 to 5, several basal and one upper towards the upper half of pedicel, basal bracts 2–3 mm long, 1–3 mm wide; upper bract 4–13 mm long, 6–14 mm wide; sepals 3, basally fused, **valvate or slightly imbricate**, 5–14 mm long, 7–17 mm wide, ovate, apex acuminate, base truncate, green, glabrous outside, pubescent and glabrous towards center inside, margins flat; petals free, sub equal; outer petals 3, 11–32 mm long, 4–26 mm wide, ovate, apex acuminate, base truncate, **purple to wine red**, margins flat, glabrous outside, glabrous or pubescent towards the margins inside; inner petals 3, valvate, 9.5–30 mm long, 8–20 mm wide, ovate, apex acuminate, base truncate, **purple to wine red**, margins flat, glabrous outside, glabrous to pubescent with glabrous base inside; stamens 1908 to 2456, in 15 to 25 rows, 2–5 mm long, linear; connective discoid, glabrous, cream; staminodes absent; carpels free, 6 to 20, ovary 3–7 mm long, stigma bilobed, slightly capitate, densely pubescent. Monocarps sessile to stipitate, stipes 0–8 mm long, 3–10 mm in diameter; monocarps 1 to 10, 22–55 mm long, 17–32 mm in diameter, ellipsoid to globose, apex rounded, glabrous, smooth, dumpy; seeds 8 to 23 per monocarp, 13–29 mm long, 3–14 mm in diameter, ellipsoid to oblong; aril absent.

#### Distribution.

A widespread species with a disjunct distribution in West and Central Africa, from Côte d’Ivoire to Ghana, and from Nigeria to Gabon. In Cameroon known from the Littoral, South, and South-West regions.

#### Habitat.

A common species when present; in lowland and premontane primary and old secondary rain forests, can be found in swamp forests. Altitude 50–1000 m a.sl.

#### Local and common names known in Cameroon.

Ikeinju (Bakweri language, *Mbani 14*).

#### IUCN conservation status.

Near Threatened (NT) (Tchouto 1998). This old assessment is certainly wrong, as ﻿﻿*U.connivens* is quite common across Cameroon, and in general across the region.

#### Uses in Cameroon.

None reported.

#### Notes.

﻿﻿﻿*Uvariodendronconnivens* can be distinguished by its long (> 55 cm) narrowly elliptic or narrowly oblong leaves, with a rounded base. When compared to other species with long leaves such as ﻿*U.fuscum* and ﻿*U.calophyllum*, it can be distinguished by its longer flower pedicel 6–40 mm long and slightly imbricate sepals. The mature petals are wine red in color, unique for Cameroonian ﻿*Uvariodendron*. Stamen count was taken from Meinke (2008).

#### Specimens examined.

**South Region**: ca 6 km S of Kribi 2–4 km E of Gr Batanga, 2.88°N, 9.916°E, *26 September 1969*, *Bos J.J.* 5412 (P,WAG); Campo Ma an National Park 5 km after main entrance, 2.35°N, 10.25°E, *15 February 2012*, *Couvreur T.L.P.* 383 (WAG,YA); 29 km east from Lélé village, 2.27°N, 13.29°E, *09 September 2013*, *Couvreur T.L.P.* 484 (WAG,YA); Bipindi, 3.08°N, 10.42°E, *01 January 1903*, *Zenker G.A.* 2624 (B,K,L,M,P,WAG); Bipindi, 3.08°N, 10.42°E, *01 January 1904*, *Zenker G.A.* 3204 (K,L,M,MO,P,P); Mimfia, 3.06°N, 10.38°E, *01 September 1913*, *Zenker G.A.* 358 (M,P,U,WAG). **South-West Region**: Mabeta 6 km SE Limbe SBL, 3.98°N, 9.283°E, *10 August 1993*, *Baker W.J.* 294 (K,YA); Ekundu Kundu, 5.12°N, 8.895°E, *27 April 1996*, *Cable S.* 2230 (K,YA); Dikulu, 3.98°N, 9.233°E, *17 December 1993*, *Cable S.* 611 (K,YA); Liwenyi, 4.37°N, 9.013°E, *28 October 1993*, *Cheek M.* 5180 (K,YA); ca 40 minutes walk N then E from Njonji Hunters path to Lake Njonji, 4.13°N, 8.993°E, *18 November 1993*, *Cheek M.* 5462 (K,YA); Ekundu Kundu, 5.13°N, 8.869°E, *25 April 1996*, *Cheek M.* 8164 (K,YA); Mount Cameroon National Park on the Bomona trail behind Bomona village 10 km NW from Idenau, 4.29°N, 9.078°E, *03 April 2016*, *Couvreur T.L.P.* 1051 (WAG,YA); Mokoko Forest Reserve Boa/Likinge, 4.42°N, 8.972°E, *31 May 1994*, *Ekema S.N.* 1078 (K,YA); Bakolle Bakossi on Kumba-Mamfe road, 5.01°N, 9.666°E, *24 May 1986*, *Etuge M.* 156 (K,MO,WAG,YA); Nyasoso, 4.81°N, 9.683°E, *24 June 1996*, *Etuge M.* 2396 (K,YA); Mungo FR, 4.73°N, 9.560°E, *22 February 2006*, *Etuge M.* 6506 (K); Mahole-Bintulu road, 4.79°N, 9.603°E, *24 November 1999*, *Gosline W.G.* 209 (K,WAG,YA); Mabeta-Moliwe, 3.98°N, 9.25°E, *06 April 1992*, *Jaff B.* 73 (K,YA); Rivières de Mosongosele et de Ndian depuis Mosongosele jusqu’à l’entrée amont de la mangrove environ 20 km SW de Mundemba, 4.83°N, 8.765°E, *13 June 1976*, *Letouzey R.* 15175 (P,YA); Buea are at Bolifamba, 4.13°N, 9.303°E, *01 January 1929*, *Maitland T.D.* 537 (K,P); Bomana-Koto Road c 500 m Bearing 305 deg towards Onge river, 4.31°N, 9.016°E, *18 October 1993*, *Ndam N.* 708 (K,YA); Bolo forest 5 kms west of Kumba-Mamfe road near Konye, 4.86°N, 9.429°E, *25 March 1986*, *Nemba J.* 56 (U); Mont versant de Idenao, 4.24°N, 8.99°E, *23 January 1985*, *Nkongmeneck B.A.* 959 (YA); Bechati-Fossimondi-Besali forest Path leading from Fossimondi to Besali, 5.64°N, 9.966°E, *28 April 2005*, *Tchiengue B.* 2204 (K,YA); Above Isobe, 4.16°N, 9°E, *10 June 1992*, *Tekwe C.F.* 87 (K,YA); Mount above small Koto village, 4.3°N, 9.1°E, *06 March 1985*, *Thomas D.W.* 4447 (K,P,YA); 3 km N of Limbe-Idenao road, 4.05°N, 9.083°E, *10 February 1986*, *Thomas D.W.* 5537 (YA); Matene from Mbilishe, 6.25°N, 9.37°E, *01 March 1987*, *Thomas D.W.* 6928 (P,YA); west of the Onge River and ridges on “Thump Mount”, 4.33°N, 8.95°E, *09 November 1993*, *Thomas D.W.* 9875 (K,YA); ca 5 km North East of Limbe TB +5500 m, 4°N, 9.25°E, *02 June 1992*, *Watts J.* 336 (K,YA); Mabeta-Moliwe TD 5835 m, 4.01°N, 9.266°E, *24 June 1992*, *Wheatley J.I.* 326 (K,YA).

### ﻿﻿﻿﻿ Uvariodendron fuscum

Taxon classificationPlantaeMagnolialesAnnonaceae

(Benth.) R.E.Fr., Acta Horti Berg. 10: 61, 1930

7BAC50D1-A109-5C0B-A50C-B475442C29B5

Fig. 122 ; Map 15E

≡ ﻿﻿Uvaria fusca Benth., Trans. Linn. Soc. London 23(3): 466, 1862; Uva fusca (Benth.) Kuntze, Revis. Gen. Pl. 1: 8, 1891.
= Uvariodendron mirabile R.E.Fr., Acta Horti Berg. 10: 59, 1930 (including ﻿﻿Uvariaconnivens Engl. & Diels, Monogr. Afr. Plf. VI: 12, 1901, *pro parte* specimens *Lehmbach 57* and *178*). Syn. nov. Type. Cameroon. South Region; between Victoria and Bimba, Preuss C.G.T. 1378, 15 Mar 1898: lectotype, here designated: P[P00315830] (B destroyed (Le Thomas 1969b)).

#### Type.

Type. Equatorial Guinea. Bioko Norte; Bioko (Fernando Po), *Mann G. 308*, 1860: holotype: K[K000198801]; isotype: P[P00362657].

#### Description.

Tree, 3–15 (20) m tall, up to 35 cm d.b.h.; stilt roots or buttresses absent. Indumentum of simple hairs; old leafless branches glabrous, **young foliate branches glabrous to slightly pubescent.** Leaves: petiole 4–35 mm long, 2–8 mm in diameter, glabrous to pubescent with long white hairs, slightly grooved, blade inserted on top of the petiole; blade 15.9–70 cm long, 4.3–22.5 cm wide, narrowly elliptic to elliptic to narrowly obovate to obovate, apex acuminate, acumen 0.4–1.5 cm long, base rounded to acute (sometimes slightly truncate), coriaceous, below densely pubescent with long white hairs to glabrous when young, glabrous when old, above glabrous when young and old; midrib sunken or flat, above glabrous when young and old, below glabrous when young, glabrous to slightly pubescent when old; **secondary veins 15 to 33 per side**, glabrous above; tertiary venation reticulate. Individuals bisexual; inflorescences cauliflorous or ramiflorous on old or young foliate branches, axillary. Flowers with 9 perianth parts in 3 whorls, 1 to 3 per inflorescence; **pedicel 0–7.5 mm long**, ca. 4 mm in diameter, pubescent; in fruit 9–15 mm long, 7–8 mm in diameter, densely pubescent; bracts 2 to 6, basal bracts 2–3 mm long, 1–3 mm wide; upper bracts 8–22 mm long, 10–50 mm wide; sepals 3, valvate, **basally fused over 20–50% of the length**, **11–30 mm long, 13–26 mm wide**, ovate, apex acuminate, base truncate, densely pubescent outside, glabrous inside, margins flat; petals free, sub equal; outer petals 3, 20–40 mm long, 17–30 mm wide, ovate, apex acuminate, base truncate, margins flat, densely pubescent outside, glabrous inside, **cream with a red streak inside**; inner petals 3, valvate, 20–42 mm long, 15–29 mm wide, ovate, apex acuminate, base truncate, margins flat, pubescent outside, glabrous inside, cream with a red streak inside; stamens 1000 to 3000, in 19 to 25 rows, 3.4–5 mm long, 0.1–0.5 mm wide, linear; connective discoid, pubescent; staminodes absent; carpels free, 20 to 104, ovary 4–7 mm long, stigma bilobed, slightly capitate, densely pubescent. Monocarps [but see comment in notes] sessile to stipitate, stipes 0–4 mm long, 2–5 mm in diameter; monocarps 6 to 24, 20–50 mm long, 11–25 mm in diameter, obovoid to oblong, apex rounded or shortly narrowed, pubescent, smooth; seeds 6 to 16 per monocarp, 10–15 mm long, 6–11 mm in diameter, flattened ellipsoid; aril absent.

**Figure 122.**
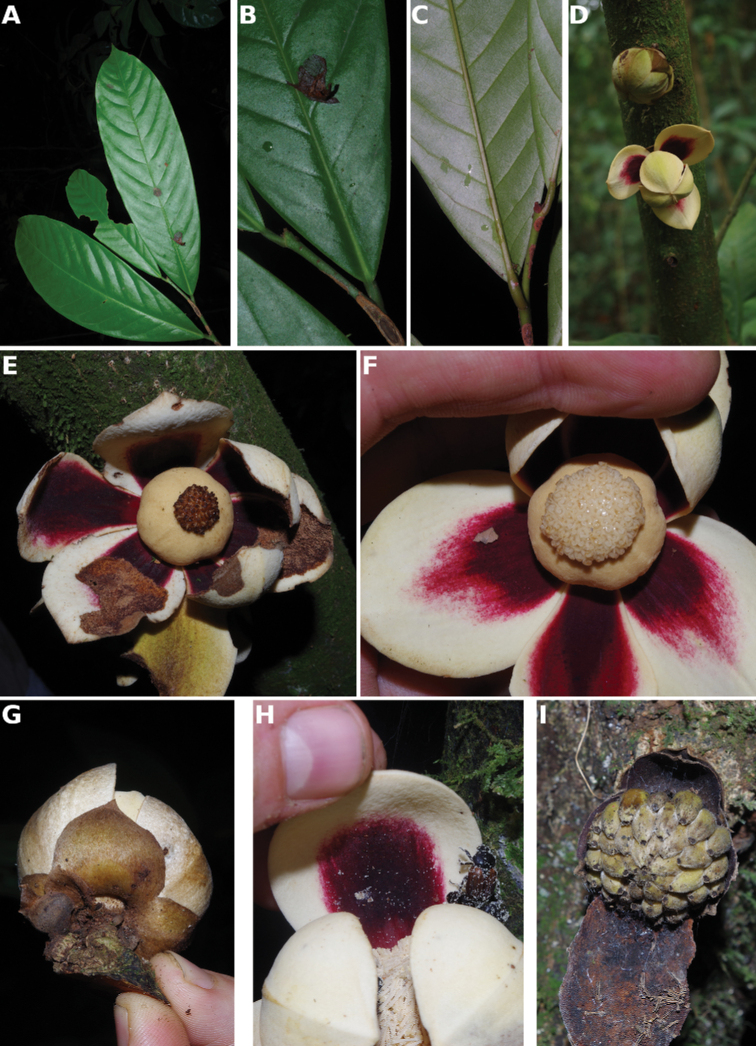
Uvariodendronfuscumvar.fuscum**A** leaf **B** base of leaf blade, upper side side **C** base of leaf blade, lower side **D** cauliflorous flowers **E** detail of flower **F** detail of flower **G** flower, bottom view, showing bracts and sepals, note fused sepal base **H** detail of outer petal and a beetle covered in pollen **I** young fruit with an old petal **A–C***Couvreur 1040*, Mt Cameroon, Cameroon **D–F***Couvreur 1029*; Mt Cameroon, Cameroon **G–I***Couvreur 1046*, Mt Cameroon, Cameroon. Photos Thomas L.P. Couvreur.

#### Distribution.

Nigeria, Cameroon, Equatorial Guinea; in Cameroon known from the South-West region.

#### Habitat.

A common species; mainly in lowland to premontane to mountain primary or old secondary rain forests. Altitude 100–1400 m a.s.l.

#### Local and common names known in Cameroon.

Obom, ossoé (Yaoundé, Biholong 279), Limboto (Bakweri, van Andel 3761).

#### IUCN conservation status.

Not evaluated yet as taxonomically defined here. Cheek and Cable (2000) assessed ﻿*U.fuscum* as Near Threatened (NT), but this didn’t follow the taxonomic concept assigned here (including ﻿*U.mirabile*). Likely Least Concern (LC).

#### Uses in Cameroon.

None reported.

#### Notes.

﻿﻿﻿*Uvariodendronfuscum* resembles ﻿*U.molundense* but has larger sepals that are fused (11–55 mm long vs. imbricate and 5–10 mm long). It also resembles ﻿*U.calophyllum* and ﻿﻿*U.connivens* but differs in having pilose to glabrous young branches and petioles (vs. tomentose in ﻿*U.calophyllum* and completely glabrous in ﻿﻿*U.connivens*). Additionally, it differs from ﻿﻿*U.connivens* in having flowering pedicels 0–7.5 mm long (vs. 10–40 mm long) and in having cream petals with dark red streak within the flower (vs. wine red petals both inside and outside).

The description of the fruits are based on specimens assigned to var. giganteum (see below). Only young immature fruits were seen might be apparent between both varieties with better fruiting material.

We synonymize here the name ﻿*U.mirabile* (Le Thomas 1967a) with ﻿*U.fuscum*. The study of the type specimens together with other specimens led us to consider them as conspecific. The only remaining collection of *Preuss 1378* found in P (the holotype of ﻿*U.mirabile* as defined by Fries 1930 was destroyed in Berlin) has small leaves that appear different from the longer leaves of *Mann 308* (the holotype of ﻿*U.fuscum* as defined by Fries 1930). However, the specimen *Wieringa 2058* presents both types of leaves, as well as leaves of intermediate size, revealing that this character is quite variable within the species. The name ﻿﻿*Uvariodendronoccidentalis* Le Thomas (Le Thomas 1967a) however is retained as a good species, but absent from Cameroon to date (a west African species).

We also recombine the name *U.giganteum* in ﻿*U.fuscum* as a new variety: var. giganteum. Both varieties have elliptic to obovate leaves, sepals which are fused at base over up to half of their length, free and ovate outer petals, and free and obovate inner petals. They differ, however, by the size of their leaves (lamina length, number of secondary veins) and the size of their flowers (sepals and petals dimensions, number of carpels) -see key to varieties below. However, these characters overlap along a continuum, with ﻿U.fuscumvar.fuscum having smaller dimensions than ﻿﻿U.fuscumvar.giganteum and some of the specimens we examined were hard to place in one or the other variety (e.g. *Couvreur 1029*).

#### Specimens examined.

**South-West Region**: Likombe, 4.11°N, 9.183°E, *22 February 1995*, *Cable S.* 1353 (K,YA); Upper Boando, 4.06°N, 9.15°E, *14 March 1995*, *Cable S.* 1524 (K,YA); Upper Boando, 4.06°N, 9.15°E, *16 March 1995*, *Cable S.* 1626 (K,YA); Ekundu Kundu, 5.14°N, 8.893°E, *26 April 1996*, *Cable S.* 2187 (K,WAG,YA); Liwenyi, 4.37°N, 9.013°E, *27 October 1993*, *Cheek M.* 5145 (K,YA); on trail leading to top of Mt Etinde after Ekonjo village, 4.06°N, 9.152°E, *01 April 2016*, *Couvreur T.L.P.* 1026 (WAG,YA); on trail leading to top of Mt Etinde after Ekonjo village, 4.06°N, 9.153°E, *01 April 2016*, *Couvreur T.L.P.* 1029 (WAG,YA); Mount Cameroon National Park Bakinguili trail above Bakinguili village, 4.09°N, 9.056°E, *02 April 2016*, *Couvreur T.L.P.* 1040 (WAG,YA); Mount Cameroon National Park on the Bomona trail behind Bomona village 10 km NW from Idenau, 4.29°N, 9.101°E, *03 April 2016*, *Couvreur T.L.P.* 1046 (WAG,YA); slopes of Mount Cameroon on the Bokwango trail near Bokwango village 4 km south west of Bu 4.12°N, 9.186°E, *23 March 2016*, *Couvreur T.L.P.* 990 (WAG,YA); slopes of Mount Cameroon on the Bokwango trail near Bokwango village 4 km south west of Bu 4.12°N, 9.170°E, *23 March 2016*, *Couvreur T.L.P.* 992 (WAG,YA); Likombe, 4.11°N, 9.183°E, *02 March 1995*, *Dahl A.* 622 (K,YA); Likombe, 4.11°N, 9.183°E, *21 February 1995*, *Groves M.* 122 (K,YA); Kumba Distr eastern boundary of Bambuko FR ca 11 miles SSW of Musome, 4.33°N, 9.166°E, *01 February 1958*, *Keay R.W.J.* 37485 (K); on Shrike Trail leading from Nyasoso to summit, 4.83°N, 9.666°E, *20 June 1994*, *Lane P.* 142 (K,YA); Bu 4.15°N, 9.233°E, *1898*, *Lehmbach H.* 178 (B,K); Bu 4.15°N, 9.233°E, *1898*, *Lehmbach H.* 57 (B,K); Buea above upper farms, 4.15°N, 9.233°E, *01 March 1929*, *Maitland T.D.* 453 (K); Cameroon Mountain Buea area 4.15°N, 9.233°E, *01 January 1930*, *Maitland T.D.* s.n. (K[K000105371]); Likomba-Pflanzung 15–35 km NE von Victoria [Limbe], 4.1°N, 9.333°E, *01 November 1928*, *Mildbraed G.W.J.* 10720 (K); Mt Cameroun flanc d’Ekona Lelu, 4.27°N, 9.3°E, *14 January 1985*, *Nkongmeneck B.A.* 891 (YA); Between Limbe & Bimbia, 3.96°N, 9.25°E, *1895*, *Preuss P.R.* 1378 (P); Mount above small Koto village, 4.3°N, 9.1°E, *06 March 1985*, *Thomas D.W.* 4469 (K,MO,P,YA); Etinde Mont, 4.08°N, 9.116°E, *29 January 1994*, *Wieringa J.J.* 2058 (WAG).

### ﻿﻿﻿﻿﻿﻿ Uvariodendron fuscum

Taxon classificationPlantaeMagnolialesAnnonaceae

var. giganteum (Engl.) Dagallier & Couvreur comb. nov.

26A3D481-7E01-5B92-998E-7DB9726B3F6D

urn:lsid:ipni.org:names:77305099-1

**Figure 123.**
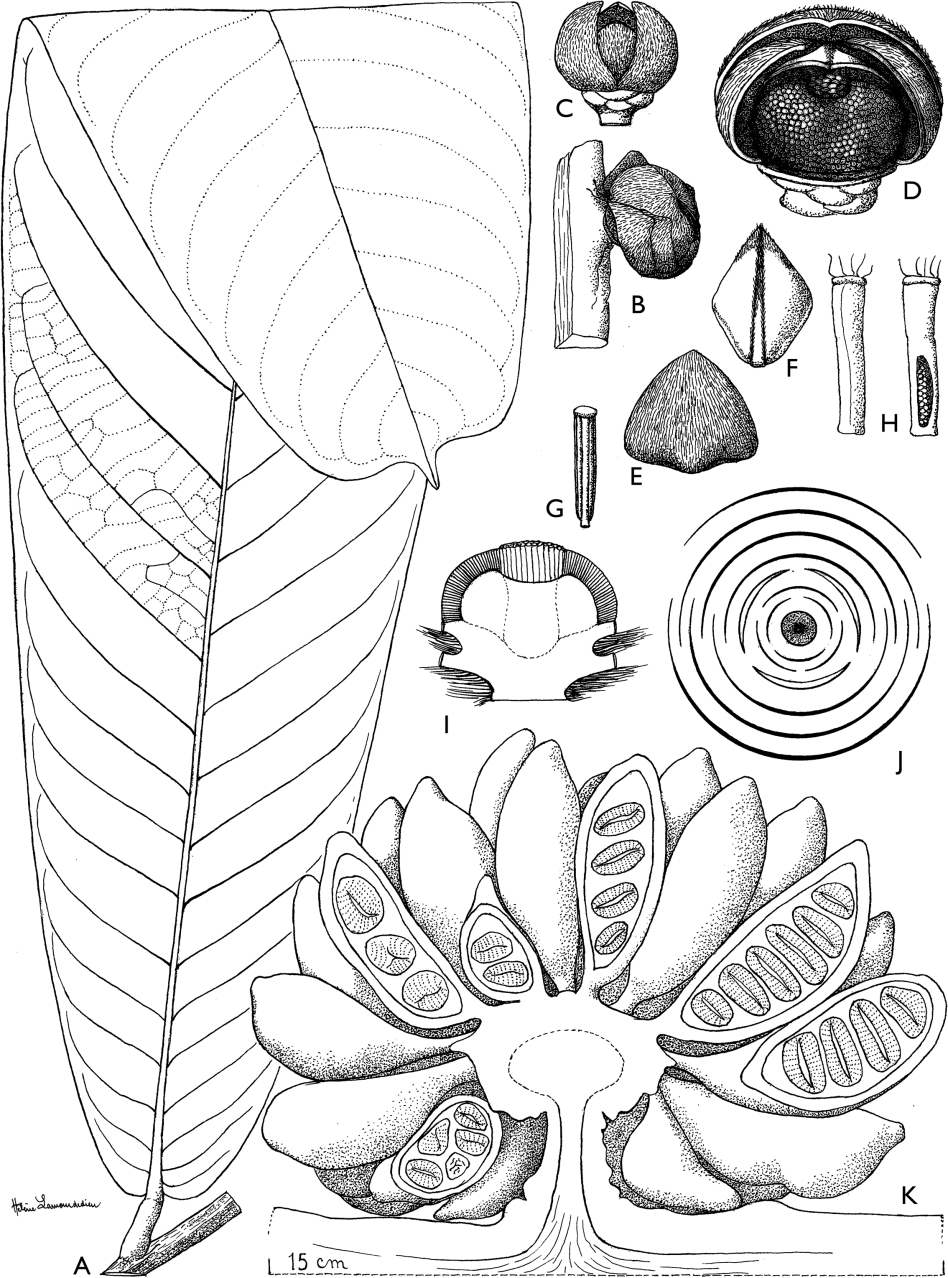
Uvariodendronfuscumvar.giganteum**A** leaf **B** flower bud, side view **C** flower bud, side view **D** detail of receptacle, two outer and one inner petals removed **E** outer petal, outer view **F** inner petal, outer view **G** stamen **H** carpel, side view and detail of ovules **I** longitudinal section of receptacle **J** floral diagram **K** fruit, longitudinal sections of monocarps **A–K** from *Hallé 3156* (as *U.giganteum*). Drawings by Hélène Lamourdedieu, Publications Scientifiques du Muséum national d’Histoire naturelle, Paris; modified from Le Thomas (1969b, pl. 50, p. 279).

**Figure 124.**
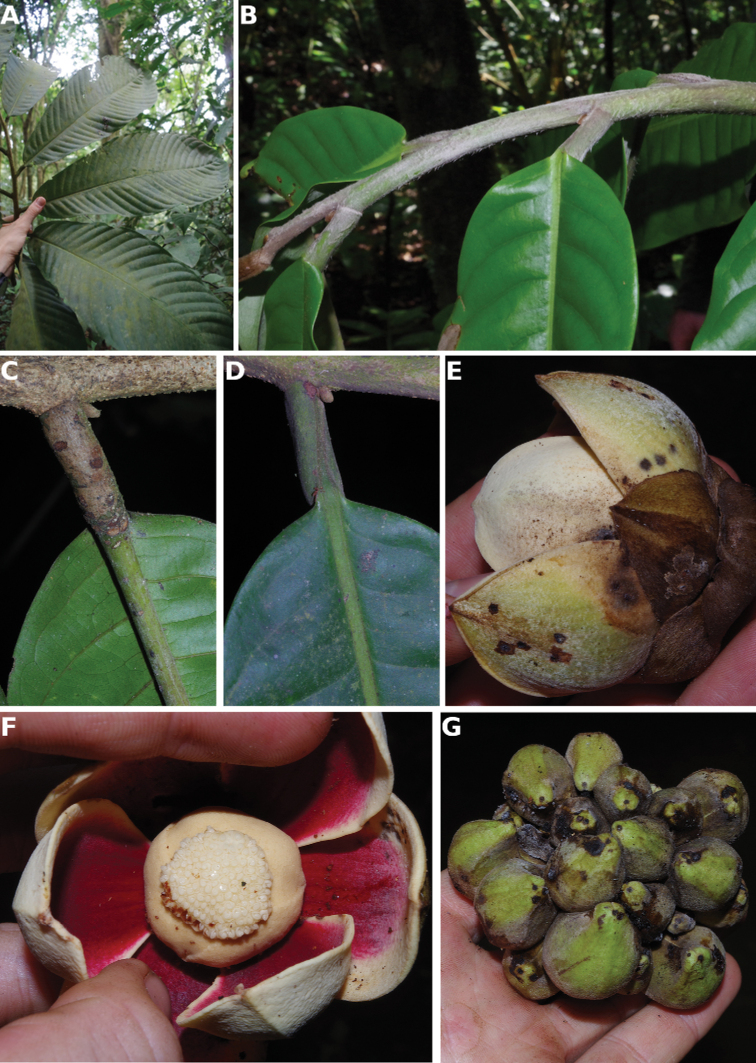
Uvariodendronfuscumvar.giganteum**A** leaf, hand for size **B** detail of young branch, note pubescence of long whitish appressed hairs **C** base of leaf blade, lower side **D** base of leaf base, upper side **E** flower, side view **F** flower, top view **G** young fruit **A, C–G***Couvreur 1057*, Mt Cameroon **B***Couvreur 1206*, Maséa, Cameroon. Photos Thomas L.P. Couvreur.

Figs 123 , 124 ; Map 15F

≡ Uvariodendron giganteum (Engl.) R.E.Fr., Acta Horti Berg. 10: 62, 1930; ﻿﻿Uvariagigantea Engl., Notizbl. Königl. Bot. Gart. Berlin 2: 292, 1899; ﻿Uvagigantea (Engl.) Kuntze, Deutsche Bot. Monatsschr. 21: 173, 1903.

#### Type.

Cameroon. Central Region; Yaoundé, *Zenker G.A. & Staudt A. 108*, 1895: lectotype designated by Fries (1930), p. 62, sheet destroyed at B, sheet here designated: P[P00362654]; isolectotype: COI[COI00004926].

#### Description.

Differs from the type variety in having **young branches and petioles covered with long soft hairs producing a whitish appearance** quickly falling off (vs. young branches and petiole sparsely pubescent to glabrous); leaves **30–70 cm long and 8.2–22.5 cm wide** (vs. 15.9–45 cm long and 4.3–11.8 cm wide), **secondary veins 22 to 33** (vs. 15 to 24), and flowers with **sepals 21–30 mm long** (vs. 11–23 mm long); **carpels 33 to 104** (vs. 20 to 70).

#### Distribution.

From southern Nigeria to Gabon and Democratic Republic of the Congo; in Cameroon known from the East, South, Central and South-West regions.

#### Habitat.

a common variety; in lowland or premontane primary or old secondary rain forests, on inundated soils or along streams or rivers. Altitude: 100–1300 m a.s.l.

Vernacular names (as assigned to *U.giganteum*): Obom, ossoé (Yaoundé, *Biholong 279*), Limboto (Bakweri, *van Andel 3761*).

#### IUCN conservation status.

Least Concern (LC) (Cheek 2014b).

#### Uses in Cameroon.

None reported.

#### Notes.

This variety is more common and widespread than the type variety. Most of these characters provided above overlap with the type variety. Without the young branches covered with long soft hairs it can be hard to identify some specimens as var. giganteum with certainty.

The specimen *Zenker G.A. & Staudt A. 108*, defined to be the type specimen by Fries (1930), was not found in B (lost or destroyed), so we make the duplicate from P as the lectotype and the duplicate from COI as the isolectotype.

#### Selected specimens examined.

**Central Region**: Mont Mbam Minkon on trail 5 km from Nkol Nyada village, 3.96°N, 11.40°E, *21 March 2013*, *Couvreur T.L.P.* 419 (WAG,YA); Yaoundé, 3.87°N, 11.52°E, *1895*, *Zenker G.A.* 108 (P); Yaundé, 3.86°N, 11.51°E, *Feburary 1895*, *Zenker G.A.* 698 (K). **East Region**: 80 km south of Yokadouma 30 km after Ngato 15 km after river ALPICAM ‘base de vie’ then 40 km on forestry road starting 4 km before Maséa village, 3.16°N, 14.70°E, *05 March 2019*, *Couvreur T.L.P.* 1206 (MPU,WAG,YA); 66 km south of Yokadouma 30 km after Ngato 15 km after river ALPICAM ‘base de vie’ then 40 km on forestry road starting 4 km before Maséa village, 3.08°N, 14.67°E, *08 March 2019*, *Couvreur T.L.P.* 1229 (MPU,WAG,YA). **South Region**: Près d’Alati-Ancienne piste Alati-Mintom II, 2.2°N, 13.42°E, *17 January 1973*, *Biholong M.* 279 (P,YA); Massif de Ngovayang village de Atog Boga, 3.22°N, 10.50°E, *04 September 2015*, *Droissart V.* 2125 (BRLU); Bipindi, 3.08°N, 10.41°E, *1898*, *Zenker G.A.* 1438 (L). **South-West Region**: Nyasoso village on max’s trail to Mt 4.82°N, 9.699°E, *05 April 2016*, *Couvreur T.L.P.* 1057 (WAG,YA); on trail from Ekongo village located 5 km before the entrance to Limbe 7 km on secondary road On flank of Mt Etinde 100 m in Mont Cameroon National Park, 4.07°N, 9.133°E, *16 October 2013*, *Couvreur T.L.P.* 512 (WAG,YA); Bakossi Mountains 1–8 km NNE of Menyum Village, 5.05°N, 9.612°E, *22 May 1987*, *Doumenge C.* 473 (L,P); Ebondji, 4.76°N, 9.598°E, *08 June 2017*, *Kamdem N.* 557 (YA); Forest trail 2 km south from Etube-Tape village, 4.85°N, 9.7°E, *02 February 1995*, *Lane P.* 501 (K,WAG,YA); Cameroon Mountain, 4.12°N, 9.187°E, *21 June 2001*, *van Andel T.R.* 3761 (U,WAG).

### ﻿﻿﻿﻿ Uvariodendron molundense

Taxon classificationPlantaeMagnolialesAnnonaceae

(Diels) R.E.Fr., Acta Horti Berg. 10: 61, 1930

FAF017B2-948A-5B4C-AE12-E4E406AA238E

**Figure 125.**
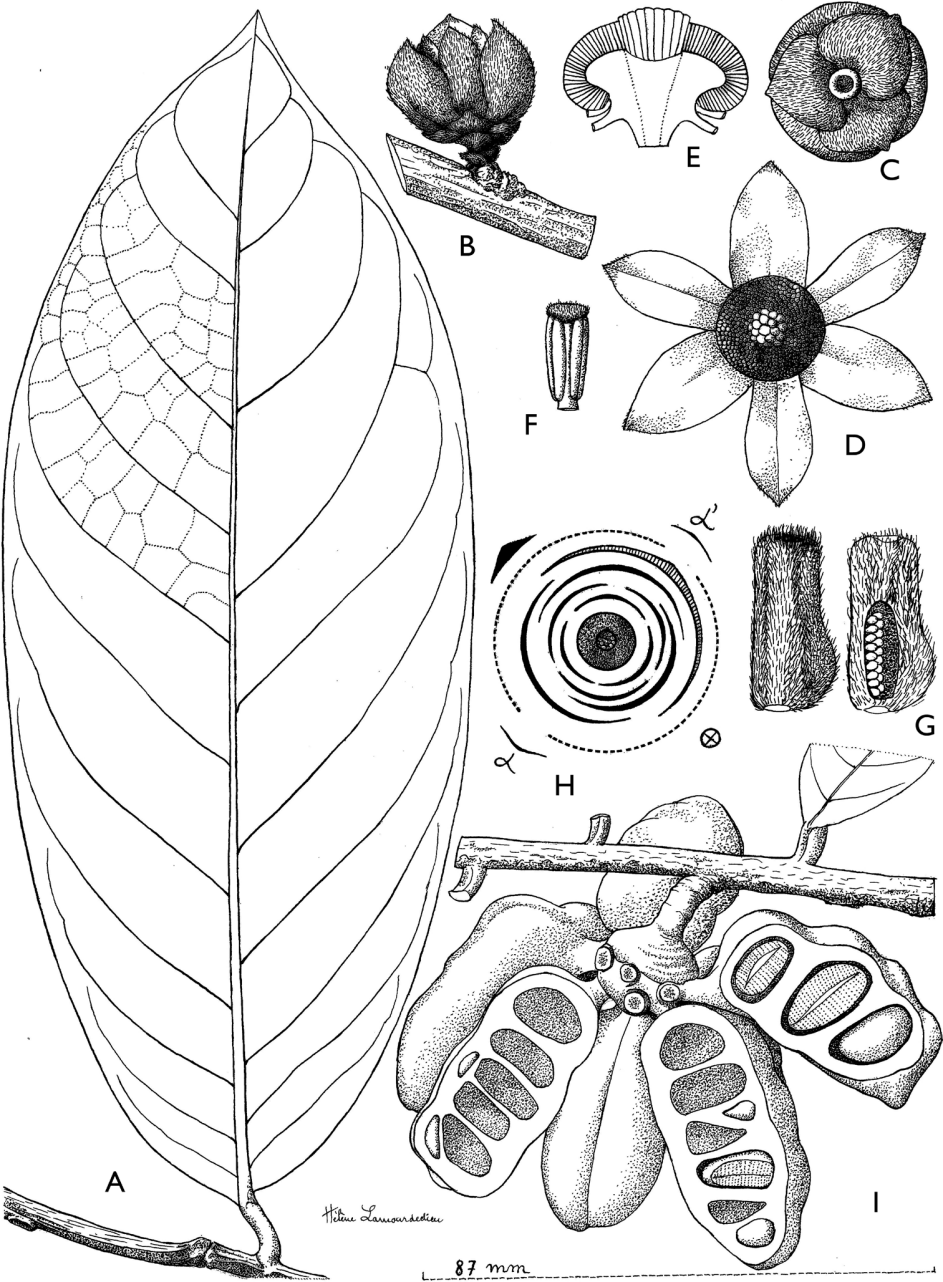
*Uvariodendronmolundense***A** leaf **B** flower on branch, side view **C** flower, bottom view **D** flower, petals open, top view **E** longitudinal section of receptacle **F** stamen, front view **G** carpel, side view and detail of ovules **H** floral diagram **I** fruit, longitudinal sections of monocarps **A–C, E–G** from *Le Testu 9649***D** from *Le Testu 8437***H, I** from *Hallé 3264*. Drawings by Hélène Lamourdedieu, Publications Scientifiques du Muséum national d’Histoire naturelle, Paris; modified from Le Thomas (1969b, pl. 51, p. 281).

**Figure 126.**
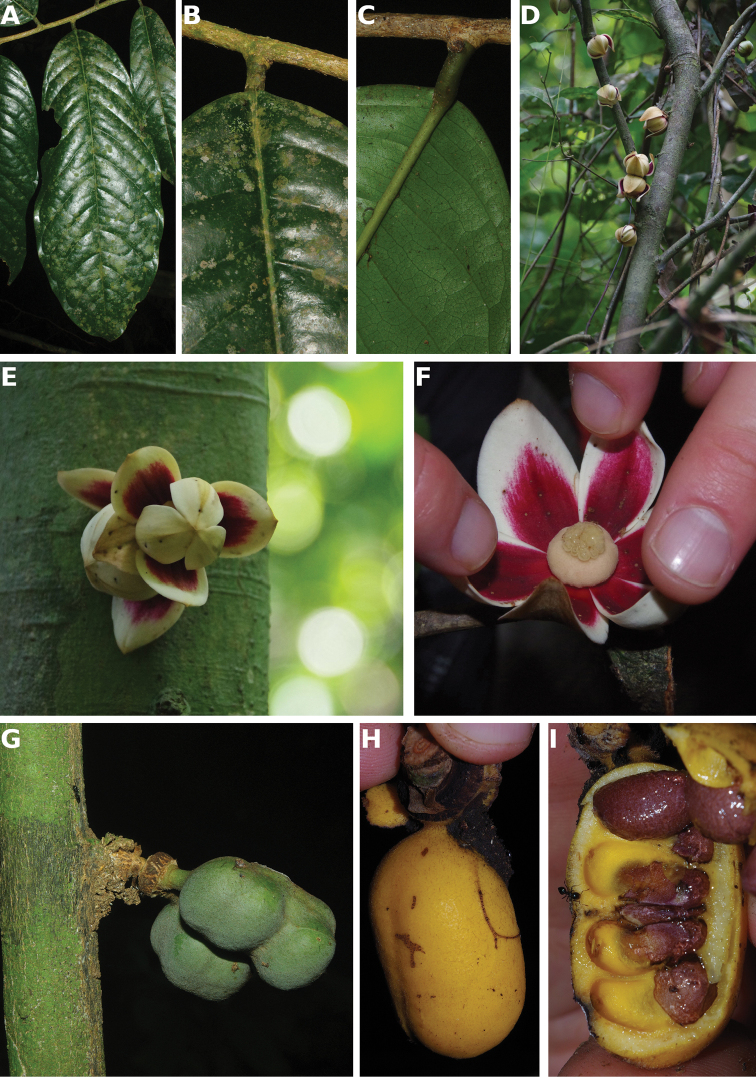
*Uvariodendronmolundense***A** leaf, upper side **B** base of leaf blade, upper side **C** base of leaf blade, lower side **D** trunk with cauliflorous flowers **E** flowers, cauliflorous **F** detail of receptacle showing stamens and carpels **G** fruit, cauliflorous **H** detail of mature monocarp **I** section of monocarp showing seeds **A–C, G***Couvreur 655*, Mambe, Cameroon **D–F***Couvreur 1172*, Mapubi, Cameroon **H, I***Couvreur 932*, Gabon. Photos Thomas L.P. Couvreur.

Figs 125 , 126 ; Map 15G

≡ Uvaria molundensis Diels, Bot. Jahrb. Syst. 53: 435, 1915.
= Uvaria letestui Pellegr., Bull. Mus. Natl. Hist. Nat., 26: 658, 1920; ﻿﻿Uvariodendronletestui (Pellegr.) R.E.Fr., Acta Horti Berg. 10: 60, 1930. Type. Gabon. Nyanga, Tchibanga, *Le Testu G.M.P.C. 1234*, Nov 1907: lectotype, sheet here designated: P[P00315833]; isotypes: BM[BM000554071; BM000554072]; P[P00315835, P00315837].
= Uvariodendron mayumbense (Exell) R.E.Fr., Acta Horti Berg. 10: 57 1930; ﻿﻿Uvariamayumbense Exell, Journ. of Bot. 64 Suppl. Polypet.: 3, 1926. Type. Angola. Cabinda, Pango Munga, *Gossweiler J. 6159*, 17 Jan 1916: holotype: BM[BM000554073].
= Uvaria mannii Hutch. & Dalziel, Kew Bull.: 150, 1927. Type. Equatorial Guinea. Bioko Norte, Bioko (Fernando Po), *Mann G. 257*, 1860: holotype: K[K000198802]; isotype: P[P00315831].

#### Type.

Cameroon. East Region; Südkameruner Waldgebiet: Bezirk Molundu, ‘Bange Busch’, unbewohnter Urwald zwischen Lokomo, Bumba und Bange, *Mildbraed G.W.J. 4373*, 29 Jan 1911: holotype: B[B 10 0153118].

#### Description.

Tree to shrub, 1.2–10 m tall, d.b.h. up to 20 cm; stilt roots or buttresses absent. Indumentum of simple hairs; old leafless branches glabrous, young foliate branches glabrous to slightly pubescent. Leaves: petiole 2–17 mm long, 1.5–5 mm in diameter, glabrous to slightly pubescent, slightly grooved, blade inserted on top of the petiole; blade 14.1–46 cm long, 5.1–16 cm wide, **narrowly elliptic to oblong to obovate**, apex attenuate to acuminate, acumen 0.1–3.8 cm long, base rounded to decurrent, papyraceous, below glabrous when young and old, above glabrous when young and old; midrib sunken or flat, above glabrous when young and old, below glabrous to glabrescent when young, glabrous when old; secondary veins 12 to 22 pairs per side, glabrous above; tertiary venation reticulate. Individuals bisexual; inflorescences cauliflorous or ramiflorous on old or young foliate branches, axillary. Flowers with 9 perianth parts in 3 whorls, 1 to 2 per inflorescence; pedicel 2–14 mm long, 1–4 mm in diameter, pubescent; in fruit 3–15 mm long, 2–6 mm in diameter, pubescent; bracts 2 to 6, several basal and one upper towards the middle of pedicel, basal bracts 2–3 mm long, 1–3 mm wide; upper bract 3–8 mm long, 3–10 mm wide; sepals 3, **imbricate**, free or sometimes fused at base, 5–9 mm long, 5.5–10 mm wide, ovate, apex acuminate, base truncate, green, densely pubescent outside, glabrous inside, margins flat; petals free, sub equal; outer petals 3, 9–30 mm long, 6–17 mm wide, ovate, apex acuminate, base truncate, cream-colored with a purple blotch at the base, margins flat, densely pubescent outside, glabrous inside; inner petals 3, valvate, 9–27 mm long, 5–16 mm wide, ovate, apex acuminate, base truncate, cream-colored with a purple blotch at the base, margins flat, glabrous to densely pubescent outside, glabrous inside; stamens 1000 to 1500, in 15 to 20 rows, 0.8–2 mm long, 0.1–0.5 mm wide, linear; connective discoid, pubescent, cream; staminodes absent; carpels free, **8 to 36**, ovary 2–4 mm long, 0.4–2 mm wide, stigma bilobed, slightly capitate, densely pubescent. Monocarps shortly stipitate, stipes ca. 0–5 mm long, ca. 2–5 mm in diameter; monocarps 1 to 12, 20–60 mm long, 9–30 mm in diameter, ellipsoid to oblong ovoid, apex rounded, bumpy, otherwise smooth, glabrous to sparsely pubescent, greyish green to orange when ripe; seeds 3 to10 per monocarp, 12–15 mm long, 7–11 mm wide, oblong, yellow to pale brown; aril absent.

#### Distribution.

From south Nigeria to Gabon to Central African Republic (southeast), and one collection from Democratic Republic of Congo; in Cameroon known from the East, South, Central Littoral and South-West regions.

#### Habitat.

A common species in Cameroon, locally dominant in the understory; in lowland and premontane primary and old secondary rain forests. Altitude 0–1000 m a.s.l.

#### Local and common names known in Cameroon.

None recorded.

#### IUCN conservation status.

Least Concern (LC) (Botanic Gardens Conservation International and IUCN SSC Global Tree Specialist Group 2019d).

#### Uses in Cameroon.

None reported.

#### Notes.

This species is very variable in terms of leaf size and shape. It is characterized by few carpels (< 40) and imbricate sepals.

In the check list of Mount Cameroon (Cable and Cheek 1998), two collections are identified as ﻿*Uvariodendron* sp. 1 and sp. 2 (*Tchouto 136* and *611*, respectively). However, we have identified these as ﻿*U.molundense*.

#### Selected specimens examined.

**Central Region**: Eastern part of park West of Ndangan 1, 3.62°N, 11.60°E, *18 March 2004*, *Cheek M.* 11839 (K,YA); Colline ‘’Nkom-Benka’a’’ dans l’appellation locale 4–5 km N du village Ekekam, 3.38°N, 11.8°E, *09 March 1978*, *Dang D.* 643 (P,YA); Mefou National park 18 km from MFOU, 3.61°N, 11.58°E, *22 March 2004*, *Tadjouteu F.* 566 (K,YA). **East Region**: A 30 km au NE de Bange (km 75 route Yokadouma-Moloundou), 3.02°N, 15.12°E, *25 May 1963*, *Letouzey R.* 5142 (P,YA); Moloundou near Lokomo Bumba and Bange, 2.08°N, 15.25°E, *29 January 1911*, *Mildbraed G.W.J.* 4373 (B); Camp CFA Bango I Bord de la rivière Bango 36 km SE Bateha Malen village situé à 21 km N de Moloundou, 2.15°N, 15.35°E, *08 April 1971*, *Villiers J.-F.* 625 (P,YA). **Littoral Region**: Mapubi 30 km before Edea on Yaoundé-Edea road On forestry road 5 km direction to Sanaga river, 3.84°N, 10.38°E, *28 February 2018*, *Couvreur T.L.P.* 1172 (MPU,P,WAG,YA); Mambe Massif above Boga village 100 km along road from Yaoundé to Ed 3.91°N, 10.77°E, *19 June 2014*, *Couvreur T.L.P.* 652 (WAG,YA); Mambe Massif above Boga village 100 km along road from Yaoundé to Ed 3.90°N, 10.77°E, *19 June 2014*, *Couvreur T.L.P.* 655 (WAG,YA); Mambe Massif above Boga village 100 km along road from Yaoundé to Ed 3.90°N, 10.77°E, *20 June 2014*, *Couvreur T.L.P.* 656 (WAG,YA). **South Region**: 13 km from Kribi Ebolowa road, 2.87°N, 9.980°E, *13 November 1968*, *Bos J.J.* 3259 (P,WAG,YA); S Bbank Lobé R SE of Grand Batanga ferry riverine forest edge, 2.86°N, 9.9°E, *11 October 1969*, *Bos J.J.* 5474 (BR,MO,P,WAG,YA); 40 km from Kribi 4 km W of Edea road N bank of lokoundje R, 3.08°N, 10.25°E, *12 March 1970*, *Bos J.J.* 6521 (WAG); ca 5 km N of km 7 Kribi-Ebolow, 2.93°N, 9.95°E, *10 July 1970*, *Bos J.J.* 7075 (P,WAG); Massif de Ngovayang village de Ngovayang sommet, 3.25°N, 10.57°E, *08 June 2015*, *Droissart V.* 1880 (BRLU); Campo-Ma’an area Bibabimvoto, 2.25°N, 10.36°E, *01 February 2000*, *Elad M.* 1270 (KRIBI,WAG); Meyo Centre, 2.55°N, 11.03°E, *24 March 1970*, *Letouzey R.* 10222 (P); Region de Kribi, 2.93°N, 9.92°E, *01 January 1911*, *Mildbraed G.W.J.* 5936 (B); Environs de Mvini 34 km east of Campo, 2.37°N, 10.09°E, *24 October 1984*, *Nkongmeneck B.A.* 800 (YA); Campo-Ma’an area Bibabimvoto, 2.27°N, 10.05°E, *26 August 2000*, *Tchouto Mbatchou G.P.* T8X_70 (WAG); Campo-Ma’an area 2.71°N, 9.866°E, *26 October 2001*, *van Andel T.R.* 4228 (KRIBI,U,WAG). **South-West Region**: Mokoko Forest Reserve Ekumbe-Mofako, 4.47°N, 9.092°E, *21 April 1994*, *Akogo M.* 234 (K,YA); Dikome, 4.92°N, 9.240°E, *05 May 1994*, *Sonké B.* 1226 (K); Mapanja, 4.08°N, 9.15°E, *20 April 1992*, *Tchouto Mbatchou G.P.* 136 (K,YA); Mokoko Above Bonja village, 4.41°N, 9°E, *23 March 1993*, *Tchouto Mbatchou G.P.* 611 (WAG).

### ﻿﻿ Uvariopsis

Taxon classificationPlantaeMagnolialesAnnonaceae

Engl., Notizbl. Königl. Bot. Gart. Berlin 2: 298, 1899

79E88FB1-1F00-5140-B884-DF23DB8FEF76

= Tetrastemma Diels Bot. Jahrb. Syst. 38(3): 241 1906; ﻿ThonneraDe Wild., Ann. Mus. Congo Belge, Bot. sér. 5, 3[1]: 86, 1909

#### Type species.

﻿﻿*Uvariopsiszenkeri* Engl.

#### Description.

Trees, 3–25 m tall, d.b.h. up to 40 cm; stilt roots or buttresses absent. Indumentum of simple hairs or glabrous. Leaves: petiole 2–8 mm long, 1–6 mm in diameter; blade 10–52 cm long, 3.5–14 cm wide, ovate, elliptic, obovate or oblong, apex acuminate to abruptly acuminate, acumen 0.7–2 cm long, base cuneate to cordate; midrib sunken or flat; secondary veins 6 to 20 pairs; tertiary venation reticulate. Flowers unisexual, monoecious, male and female flowers similar or dissimilar, or bisexual (in one species not present in Cameroon), with (5) 6 perianth parts in 2 whorls. Inflorescences ramiflorous or cauliflorous, flower buds globose or conical, 1 to 6(50) per inflorescence; pedicel 1–450 mm long; in fruit 3–450 mm long; bracts 1 to 4, one basal and one upper, or all basal, 1–2 mm long; sepals 2, valvate (imbricate), free or basally fused, 1.5–10 mm long, ovate to semi-circular, apex acute, acuminate or attenuate, base truncate; petals free or basally fused, (3)4, valvate, 2–45 mm long, 2.5–17 mm wide, oblong, ovate, elliptic or linear, apex acuminate or attenuate, base truncate; stamens 100 to 1000, in 9 to 30 rows, 1–2 mm long, oblong to elongated; connective reduced or absent, glabrous; staminodes absent; carpels free, 15 to 280, 1–4 mm long, stigma ovoid, coiled or flat, glabrous. Monocarps sessile or stipitate, stipes 1–10 mm long; monocarps 2 to 25, 15–80 mm long, 9–55 mm in diameter, cylindrical, ellipsoid or globose, apex rounded or apiculate, smooth, verrucose or bumpy; seeds 4–25 mm long, 3–15 mm in diameter, ellipsoid; aril absent.

#### Taxonomy.

Le Thomas (1969b); Gereau et Kenfack (2000); Kenfack et al. (2003); Dagallier et al. (in prep.).

A genus with 18 species, 13 of which occur in Cameroon, three being endemic, making Cameroon a center of diversity for this genus.

﻿*Uvariopsis* is distinguished from other Annonaceae genera in Cameroon by having 2 sepals and 4 petals in a single whorl (except for ﻿*U.congolana* which has 3 petals but 2 sepals). ﻿*Uvariopsis* is unisexual and monoecious (male and female flowers on same individual), except for the East African species *U.bisexualis* Verdc. which is bisexual (Verdcourt 1986). Important characters to distinguish the different species of ﻿*Uvariopsis* are the shape of the flower buds (which can be conical or globose) and whether the petals are basally fused or free (Gereau and Kenfack 2000; Kenfack et al. 20﻿﻿﻿﻿﻿﻿﻿﻿﻿03; Gosline et al. 2022). ﻿*Uvariopsis* can be divided in two main groups: species with male or hermaphrodite flowers occurring on branches, and then the flowers are small (petals shorter than 7 mm), and species with large flowers (petals longer than 7 mm) all cauliflorous.

The species ﻿﻿﻿*Dennettiatripetala* Bak. f. was suggested to be congenic with ﻿*Uvariopsis* (Kenfack et al. 2003), but recent phylogenetic analyses showed it should be kept in a genus of its own which is followed here (Dagallier et al. in prep; see under that name).

### ﻿Key to the species of ﻿*Uvariopsis* in Cameroon

**Table:** 

1	Crushed leaves emitting a strong citrus scent when fresh; combination of leaves greater than 30 cm long and flowering pedicels smaller than 2 mm long	﻿***U.citrata***
–	Crushed leaves without citrus scent; leaves smaller than 30 cm long, or leaves greater than 30 cm long but then flowers on pedicels longer than 3 mm long	**2**
2	Leaf blades generally < 15 cm long; pedicel < 7 mm long and petals < 7 mm long	**3**
–	Leaf blades generally > 15 cm long, pedicel > 10 mm long and/or petals > 7 mm long	**6**
3	Young branches glabrous, inflorescences cauliflorous and flowers sessile	﻿***U.sessiliflora***
–	Young branches densely pubescent to glabrous, flowers ramiflorous or cauliflorous and flower pedicels more than 3 mm long; or young branches densely pubescent to pubescent and flower pedicels less than 3 mm long	**4**
4	Young foliate branches glabrous or very sparsely pubescent; petals free; monocarps glabrate to glabrous	﻿﻿***U.congensis***
–	Young foliate branches and petioles densely to sparsely pubescent; petals basally fused; monocarps tomentose	﻿﻿﻿***U.zenkeri***
5	Petals basally fused	**6**
–	Petals free	**9**
6	Petals 3	﻿***U.congolana***
–	Petals 4	**7**
7	Flower buds globose, monocarps verrucose	﻿***U.pedunculosa***
–	Flower buds conical to pyramidal, monocarps smooth	**8**
8	Sepals 5–10 mm long, flowers completely covering base of trunk, generally occurring above 800 m a.s.l.	﻿***U.submontana***
–	Sepals 2–4 mm long, flowers partially covering base of trunk, generally occurring below 800 m a.s.l.	﻿***U.korupensis***
9	Flowering pedicels 2–10 mm long	**10**
–	Flowering pedicels 10–198 mm long	**11**
10	Petals linear, 25–45 mm long, more than 6 times longer than wide	﻿***U.bakeriana***
–	Petals elliptic to ovate, 10–14 mm long, less than 6 times longer than wide	﻿***U.etugeana***
11	Flower buds globose; monocarps not ribbed	﻿***U.dioica***
–	Flower buds conical or narrowly ovoid to pyramidal	**12**
12	Young foliate branches and petioles tomentose; secondary veins 8 to 13 pairs per side; petals wine-brown	﻿***U.solheidii***
–	Young foliate branches and petioles glabrous; secondary veins 5 to 8 (9) pairs per side; petals yellow-green	﻿***U.dicaprio***

### ﻿﻿﻿﻿ Uvariopsis bakeriana

Taxon classificationPlantaeMagnolialesAnnonaceae

(Hutch. & Dalziel) Robyns & Ghesq., Ann. Soc. Sci. Bruxelles, Ser. B 53: 320, 1933

E1E4EEDD-8A8A-5CE2-9F69-1C4E142E12F0

**Figure 127.**
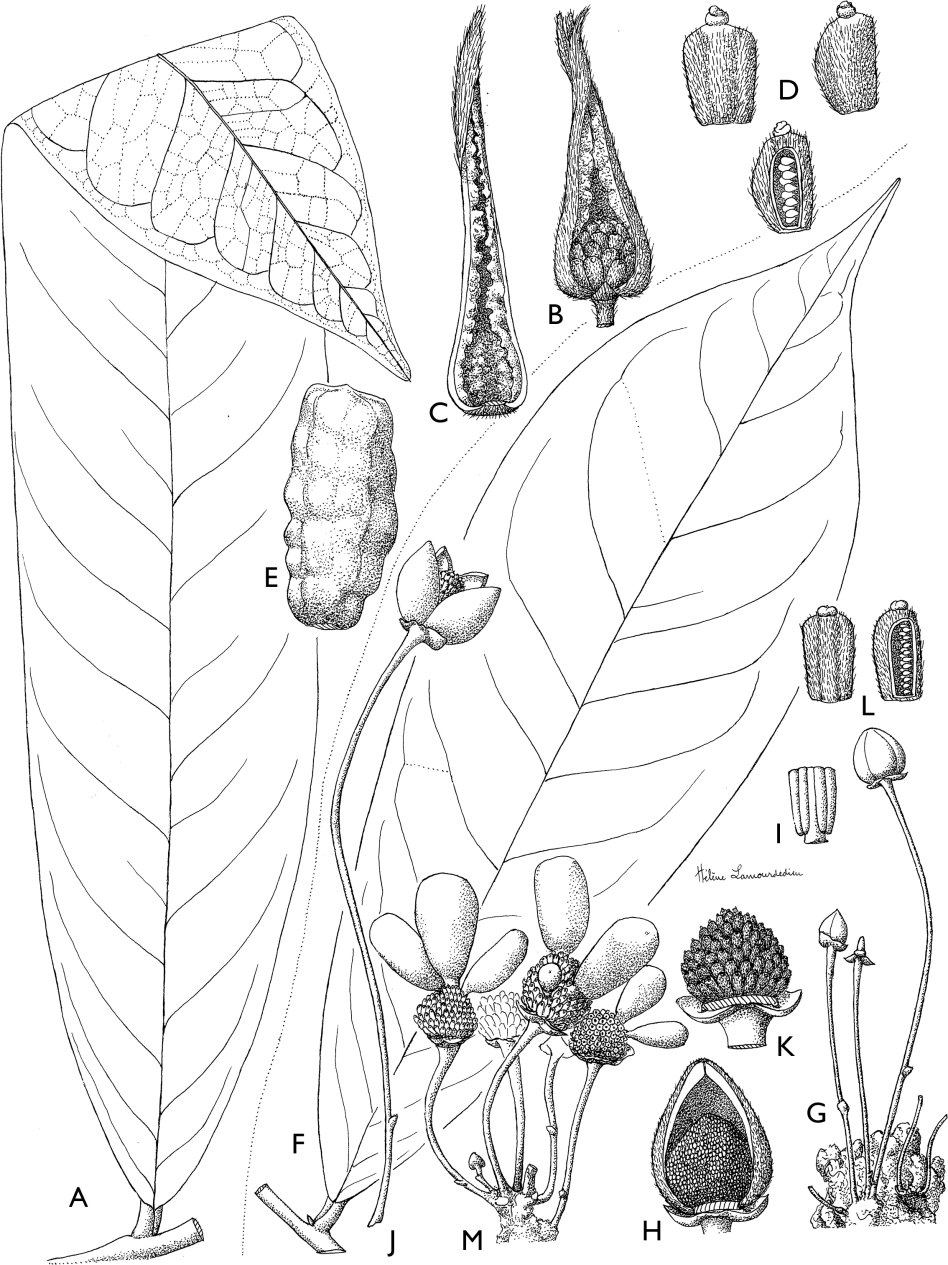
*Uvariopsisbakeriana***A** leaf, top view **B** detail of male flower, 2 petals removed **C** petal, inner view **D** carpel, side view, front view and detail of ovules **E** monocarp. *Uvariopsisdioica***F** leaf, top view **G** male flowering pedicels and flowers **H** detail of male flower, 2 petals removed **I** stamen **J** female flowering pedicel **K** detail of female flower, all 4 petals removed **L** carpel, front view and detail of ovules **M** fruits **A–E** from *Brenan 9409***F–L** from *Keay 28066***M** from *Letouzey 4230*. Drawings by Hélène Lamourdedieu, Publications Scientifiques du Muséum national d’Histoire naturelle, Paris.

**Figure 128.**
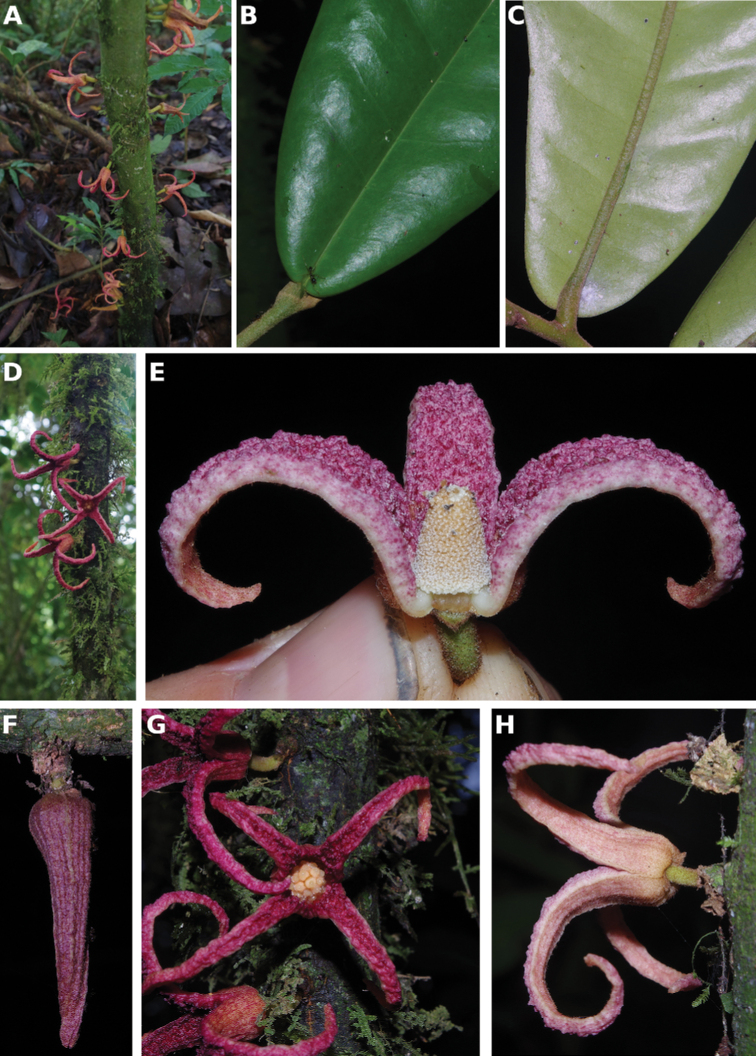
*Uvariopsisbakeriana***A** trunk with flowers **B** leaf base, upper side **C** leaf base, lower side **D** trunk with female flowers **E** detail of male flower, one petal removed **F** flower bud **G** female flower, top view **H** male flower, side view, showing sepals **A, D, G***Couvreur 1045*, Mt Cameroon Cameroon **B, C, E, F, H***Couvreur 1000*, Bayang Mbo, Cameroon. Photos Thomas L.P. Couvreur.

Figs 127 , 128 ; Map 15H

≡ Tetrastemma bakerianum Hutch. & Dalziel, Kew Bull.: 153, 1927.

#### Type.

Nigeria. Cross River State; Oban, *Talbot P. 1517*, 1912: holotype: K[K000199043]; isotype: BM[BM000554076].

#### Description.

Tree, 2–7 m tall, d.b.h. 3–7 cm; stilt roots or buttresses absent. Indumentum of simple hairs; old leafless branches glabrous, young foliate branches pubescent. Leaves: petiole 3–5 mm long, 2–3 mm in diameter, glabrous to pubescent, slightly grooved, blade inserted on top of the petiole; **blade 25–34 cm long, 4.5–9 cm wide, narrowly oblong to narrowly obovate**, apex acuminate, acumen 1–3 cm long, base rounded to subcordate, coriaceous, below sparsely pubescent when young, glabrous when old, above glabrous when young and old; midrib sunken or flat, above glabrous when young and old, below pubescent when young, glabrous when old; secondary veins 11 to 20 pairs per side, glabrous above; tertiary venation reticulate. Individuals unisexual, monoecious, **inflorescences cauliflorous**. Flowers with 6 perianth parts in 2 whorls, **long conical in bud**, 1 to 2 per inflorescence, male and female inflorescences similar, but with a tendency of female flowers being located towards the base of the trunk; pedicel 3–8 mm long, ca. 1 mm in diameter, pubescent; in fruit 4–11 mm long, 2 mm in diameter, pubescent; bracts 2 to 3, all basal or towards the lower half of pedicel, 1–2 mm long, 1–2 mm wide; sepals 2, valvate, free, 3–4 mm long, 2 mm wide, triangular, apex attenuate, base truncate, brown, densely pubescent outside, glabrous inside, margins flat; petals free, 4, 25–45 mm long, 4–8 mm wide, **linear**, apex attenuate, base truncate, **wine-brown to dark red**, margins flat, pubescent outside, **glabrous and highly verrucose inside**; male flowers: stamens 400 to 600, in 15 to 20 rows, ca. 1 mm long, broad; connective reduced or absent, glabrous, cream; staminodes absent; female flowers: carpels free, 24 to 40, ovary 2–3 mm long, 1–1.5 wide, stigma coiled, densely pubescent. Monocarps stipitate, stipes 3–6 mm long, ca. 2 mm in diameter; monocarps 4 to 6, 25–50 mm long, 10–20 mm in diameter, cylindrical, apex rounded, puberulent to glabrous, verrucose, wrinkled, bright red when ripe; seeds 6 to 16 per monocarp, 17–22 mm long, 10–12 mm in diameter, ellipsoid; aril absent.

#### Distribution.

From eastern Nigeria to northern Cameroon; in Cameroon known from South and South-West regions.

#### Habitat.

A common species when present; in lowland primary or old secondary rain forests. Altitude 50–800 m a.s.l.

#### Local and common names known in Cameroon.

None recorded.

#### IUCN conservation status.

Least Concern (LC) (Cosiaux et al. 2019aw).

#### Uses in Cameroon.

None reported.

#### Notes.

Vegetatively, ﻿﻿﻿*Uvariopsisbakeriana* is close to ﻿*U.citrata*, ﻿*U.korupensis* and ﻿*U.submontana*, having generally large leaves (largest leaves > 31 cm) with a rounded to (sub)cordate base. When in flower, ﻿*U.bakeriana* is easily distinguished from other species by its linear to very narrowly elliptic petals (forming a long conical flower bud) which are free at base and deep red in color (when fresh).

#### Specimens examined.

**South-West Region**: Kumba Div S Bakundu FR ca 1 mile west of Bopo village, 4.48°N, 9.372°E, *12 March 1948*, *Brenan J.P.M.* 9305 (K); Banga (S Bakundu), 4.5°N, 9.57°E, *13 March 1948*, *Brenan J.P.M.* 9409 (P); Ekundu Kundu, 5.18°N, 8.859°E, *25 April 1996*, *Cable S.* 2150 (K,YA); Ekundu Kundu, 5.12°N, 8.895°E, *27 April 1996*, *Cable S.* 2256 (K,YA); Korup National Park nature trail near suspension bridge, 4.98°N, 8.85°E, *01 February 1995*, *Cheek M.* 7234 (K,YA); Bayang Mbo Wildlife Sanctuary after Mbu river, 5.35°N, 9.502°E, *25 March 2016*, *Couvreur T.L.P.* 1000 (WAG,YA); Bayang Mbo Wildlife Sanctuary after Mbu river, 5.35°N, 9.501°E, *26 March 2016*, *Couvreur T.L.P.* 1015 (WAG,YA); Mount Cameroon National Park on the Bomona trail behind Bomona village 10 km NW from Idenau, 4.29°N, 9.098°E, *03 April 2016*, *Couvreur T.L.P.* 1045 (WAG,YA); Ntali, 5.25°N, 9.576°E, *30 November 2000*, *Etuge M.* 4860 (K); South Bakundu forest reserve, 4.5°N, 9.57°E, *14 May 1970*, *Farron C.* 6613 (YA); South Bakundu forest reserve, 4.54°N, 9.42°E, *01 May 1970*, *Farron C.* 7297 (P); 3–25 km S of Six cup Garri Creek, 5.03°N, 8.883°E, *06 March 1993*, *Gereau R.E.* 5195 (MO,WAG,YA); Entre Etinkem et Nfaitok 16 10 km N Nguti, 5.41°N, 9.39°E, *15 June 1975*, *Letouzey R.* 13841 (P,YA); Akpasang river camp (Mouanko Region), 4.98°N, 8.71°E, *07 February 1976*, *McKey D.B.* 70 (P,YA); Etome, 4.1°N, 9.05°E, *13 February 1997*, *Tchouto Mbatchou G.P.* 1663 (K,YA); Korup National Park, 4.98°N, 8.85°E, *01 March 1979*, *Thomas D.W.* 1086 (K); Korup National Park, 5.26°N, 9.183°E, *24 March 1984*, *Thomas D.W.* 3336 (YA); Korup National park Forest along footpath from Ndian River at PAMOL Field 69 and transect P, 5.01°N, 8.833°E, *24 January 1985*, *Thomas D.W.* 4300 (P,YA); Korup National Park, 5.18°N, 8.85°E, *16 February 1986*, *Thomas D.W.* 5606 (MO); Korup National Park, 5.33°N, 8.9°E, *22 May 1988*, *Thomas D.W.* 7835 (MO).

### ﻿﻿﻿﻿ Uvariopsis citrata

Taxon classificationPlantaeMagnolialesAnnonaceae

Couvreur & Niangadouma, PhytoKeys 68: 1–8, 2016

1F97C801-FE5B-5EFF-9FF3-0EA5C19B89BD

Fig. 129 ; Map 15I

#### Type.

Gabon. Estuaire; Monts de Cristal, near first bridge after Kinguélé village, 0°46'66"N, 10°27'81"E, *Couvreur T.L.P 1143*, 14 Jun 2016: holotype: WAG; isotypes: LBV, P.

#### Description.

Tree, 4–10 m tall, d.b.h. 3–10 cm; stilt roots or buttresses absent. Indumentum of simple hairs; old leafless branches glabrous, young foliate branches pubescent. Leaves: petiole 4–8 mm long, 3–5 mm in diameter, pubescent when young, pubescent to glabrous when old, grooved on top, blade inserted on top of the petiole, **strong lemon scent when crushed**; **blade 31.2–50 cm long, 8.8–12 cm wide**, elliptic to obovate, apex acuminate, acumen 2–3 cm long, base subcordate to cordate, coriaceous, below glabrous when young and old, above glabrous when young and old; midrib sunken or flat, above glabrous when young and old, below sparsely pubescent when young, glabrous when old; secondary veins 17 to 19 pairs per side, glabrous above; tertiary venation reticulate. Individuals unisexual, monoecious; inflorescences cauliflorous, **sparsely spaced along the trunk mostly towards the lower half of the trunk**. Flowers with 6 perianth parts in 2 whorls, ovoid to conical in bud, 1 to 2 per inflorescence, male and female inflorescences similar; **pedicel 0–2 mm long**, 1–2 mm in diameter, densely pubescent; in fruit unknown; bracts up to 3, all basal, 1–2 mm long, 4 mm wide; sepals 2, valvate, basally fused, enclosing the petals in bud, 9–15 mm long, 4–6 mm wide, narrowly ovate, apex acute, base truncate, densely pubescent with hairs appressed outside, densely pubescent or glabrous towards base inside, margins flat; petals 4, 7–15 mm long, 5–8 mm wide, ovate, apex acute, base truncate, brownish-greenish-yellow, margins flat, pubescent outside, glabrous inside; male flowers: stamen number unknown, 0.5 mm long, oblong, connective truncate, glabrous, pale yellow; female flowers: carpels free, ca. 60, ovary 4–5 mm long, ca. 0.5 mm wide, densely pubescent with long appressed hairs, stigma cylindrical coiled. Fruits unknown.

#### Distribution.

A species only known from southern Cameroon and two localities in Gabon (Monts de Cristal National Park, Mbé sector); in Cameroon known from the South Region.

#### Habitat.

A rare species; in mature or old secondary forests near rivers in periodically flooded soils, in flat valley bottoms or in well-drained forests on slope. Altitude 60–300 m a.s.l.

#### Local and common names known in Cameroon.

Ntala (Yaoundé, *Letouzey 9017*); Kakangula (Bagielli Pygmies, *Letouzey 9017*).

#### IUCN conservation status.

Data deficient (DD) (Cosiaux et al. 2019ax), but this assessment didn’t take in account the two specimens cited here form Cameroon.

#### Uses in Cameroon.

None reported.

#### Notes.

﻿﻿﻿*Uvariopsiscitrata* resembles ﻿*U.sessiliflora* by its (sub)sessile flowers (pedicels 0–2 mm long), but is easily distinguished by its strong lemon scent, longer leaves (31–50 vs 12–18 cm) and (sub)cordate leaf base (vs acute). The strong lemon scent is unique in the genus. This character has also been reported in ﻿﻿﻿*Uvariodendronangustifolium* and in ﻿﻿U.molundensevar.citrata (endemic to Gabon).

This species was suggested to be endemic to Gabon (Couvreur and Niangadouma 2016), however we identified two specimens from southern Cameroon that fit this species morphologically (young foliate branches pubescent; leaves ca. 35 cm long and sessile ovoid flowers). Neither specimen mentions the citrus scent of the leaves. *Letouzey 9017* however does mention that the leaves emit a strong smell when crushed, and the leaves are used to prepare fish dishes which gives them an “aromatic taste” (translated from French, “goût aromatique”).

**Figure 129.**
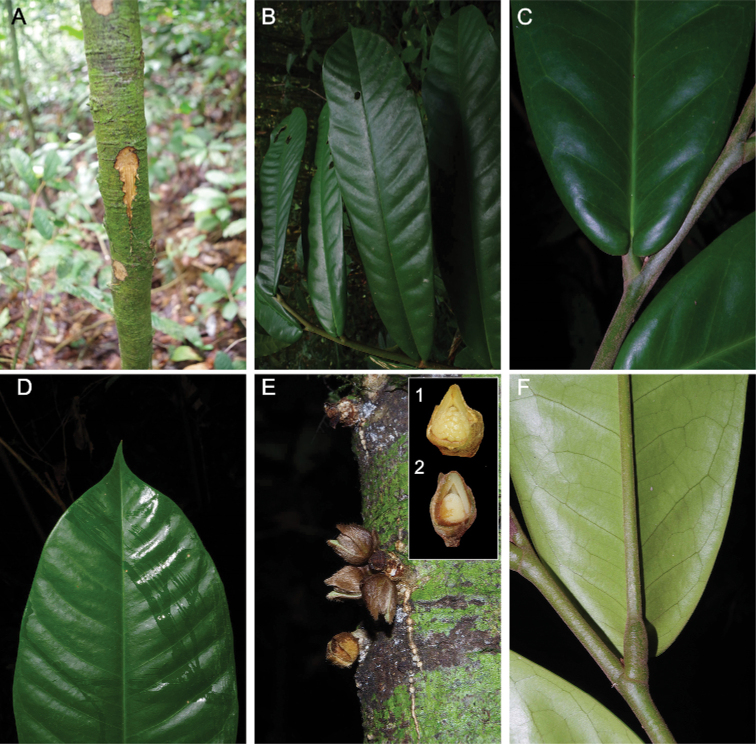
*Uvariopsiscitrata***A** trunk with slash (Couvreur 1126) **B** entire leaf **C** leaf base upper side **D** leaf apex, upper side **E** pre anthetic flower buds on trunk, 1. female flower, 2. male flower **F** leaf base, lower side **A–F***Couvreur 1143*, Gabon. Photos **A–F** Thomas L.P. Couvreur.

#### Specimens examined.

**South Region**: A 15 km au SSE de Zingui (soit à 50 km au SE de Kribi), 2.82°N, 10.97°E, *14 March 1968*, *Letouzey R.* 9017 (P,YA); Campo-Ma’an area Bibabimvoto, 2.21°N, 10.01°E, *13 May 2000*, *Tchouto Mbatchou G.P.* 2869 (KRIBI,WAG,YA).

### ﻿﻿﻿﻿ Uvariopsis congensis

Taxon classificationPlantaeMagnolialesAnnonaceae

Robyns & Ghesq., Ann. Soc. Sci. Bruxelles, Ser. B 53: 322, 1933

F32F99F0-60DD-526D-8C17-AD30D9DE17C0

**Map 16.**
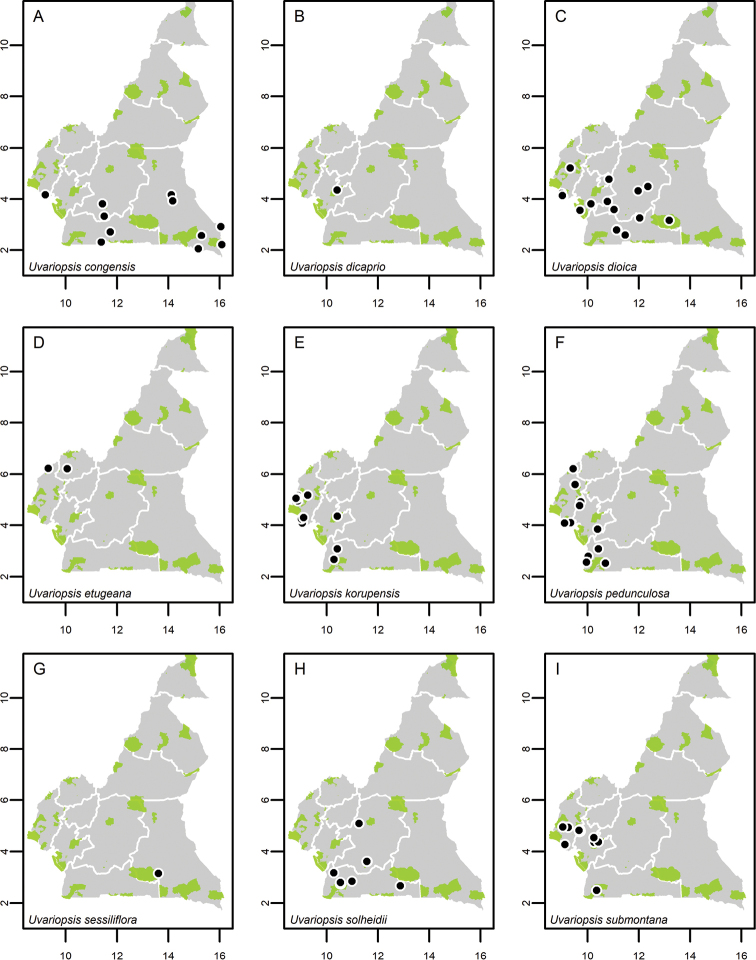
**A***Uvariopsisconge*nsis **B***Uvariopsisdicaprio***C***Uvariopsisdioica***D***Uvariopsisetugeana***E***Uvariopsiskorupensis***F***Uvariopsispedunculosa***G***Uvariopsissessiliflora***H***Uvariopsissolheidii***I***Uvariopsissubmontana*. White borders represent region limits in Cameroon; green patches represent protected areas (see methods and Suppl. material 1: Fig. S1).

**Figure 130.**
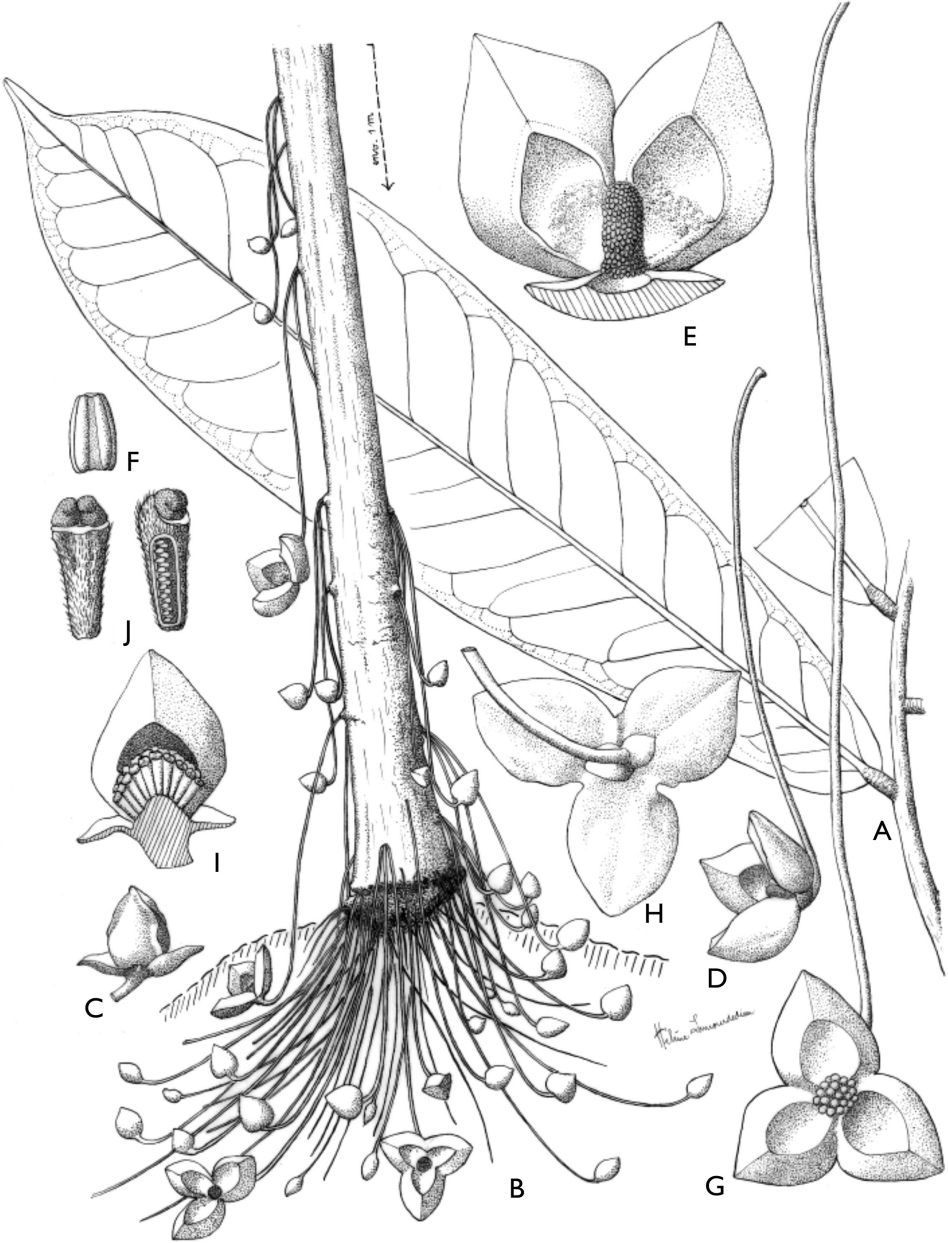
*Uvariopsiscongolana***A** leaves, lower side **B** trunk with long flowering pedicels **C** flower bud **D** male pedicels and flower **E** detail of male flower, 2 petals removed **F** stamen **G** female pedicels and flower **H** lower view of female flower **I** longitudinal section of female flower **J** carpel, front view and detail of ovules **A, G** from *Hallé 3039***B–F, H–J** from *Hallé 3039*. Drawings by Hélène Lamourdedieu, Publications Scientifiques du Muséum national d’Histoire naturelle, Paris; modified from Le Thomas (1969b; pl. 55, p. 305).

Figs 130 , 140 ; Map 16A

#### Type.

Democratic Republic of the Congo. Bandundu; Lusambo, *van Kerkhoven E. s.n.*, 16 Aug 1913: lectotype, here designated: BR[BR0000008824318].

#### Description.

Tree to shrub, 2–6 m tall, d.b.h. up to 6 cm; stilt roots or buttresses absent. Indumentum of simple hairs or glabrous; old leafless branches glabrous, **young foliate branches sparsely pubescent to glabrous.** Leaves: petiole 2–6 mm long, 1–2.5 mm in diameter, **glabrous**, slightly grooved, blade inserted on top of the petiole; blade 8–17.7 cm long, 2.6–7.7 cm wide, elliptic, **apex attenuate to acuminate, acumen 0.5–1.8 cm long**, base acute to decurrent, papyraceous, below glabrous when young and old, above glabrous when young and old; midrib sunken or flat, above glabrous when young and old, below glabrous when young and old; secondary veins 7 to 12 pairs per side, glabrous above; tertiary venation reticulate. Individuals unisexual, monoecious; inflorescences sometimes cauliflorous, mainly ramiflorous on young foliate and old leafless branches, axillary. Flowers with 6 perianth parts in 2 whorls, **globose in bud**, 1 to 2 per inflorescence, male and female inflorescences dimorphic; **male pedicels 2.5–6 mm long**, 0.5–1 mm in diameter, pubescent; **female pedicel: 3.5–8 mm long**, ca. 1 mm in diameter, pubescent; in fruit 5–15 mm long, 1–3 mm in diameter, pubescent to glabrous; male and female bracts 1 to 4, several basal and one upper towards the lower half of pedicel, similar in size, ca. 1 mm long, ca. 1 mm wide. Male and female sepals 2, valvate, free, 0.7–1.5 mm long, 1–2 mm wide, triangular, apex attenuate, base truncate, brown, pubescent outside, glabrous inside, margins flat; **male and female petals free**, 4, 2.5–7 mm long, 3–5 mm wide, ovate, apex acute, base truncate, margins flat, pubescent outside, glabrous, pubescent towards margins inside, light red; male flowers: stamens 300 to 400, in 15 to 20 rows, 0.5 mm long, linear; connective reduced or absent, glabrous, cream; staminodes absent; female flowers: carpels free, 11 to 40, ovary 1.5–3 mm long, stigma globose, densely pubescent. Monocarps stipitate, stipes 0–4 mm long, 1–3 mm in diameter; monocarps 1 to 15, 13–45 mm long, 7–18 mm in diameter, cylindrical, apex rounded, glabrous, smooth, bumpy, green to orange to red when ripe; seeds 2 to 10 per monocarp, 9.5–14 mm long, 7–8 mm in diameter, ellipsoid; aril absent.

#### Distribution.

A widespread species with a disjunct distribution in West Africa from Ivory Coast and Ghana, and in Central Africa from eastern Nigeria to the Democratic Republic of the Congo and South Sudan; in Cameroon known from the Central, South and South west regions.

#### Habitat.

An uncommon species, in lowland and premontane primary or old secondary rain forests, and in seasonally flooded forest along rivers. Altitude 500–1000 m a.s.l.

#### Local and common names known in Cameroon.

None recorded.

#### IUCN conservation status.

Least Concern (LC) (Harvey-Brown 2019d).

#### Uses in Cameroon.

None reported.

#### Notes.

﻿﻿﻿*Uvariopsiscongensis* resembles ﻿﻿﻿*U.zenkeri* in being a small tree with small generally ramiflorous (more rarely cauliflorous, and then few flowered) flowers (in contrast to large mainly cauliflorous flowers in other species). However, ﻿﻿*U.congensis* is glabrous or sparsely pubescent especially in the young foliate branches and petiole (versus very densely pubescent to pubescent in ﻿﻿﻿*U.zenkeri*), has globose flower buds (versus conical), a longer pedicel (2.5–8 mm versus 0–7 mm), petals that are free (versus fused), and glabrous monocarps (versus tomentose).

#### Specimens examined.

**Central Region**: Mt Eloumden 6 km Sud ouest de Yaoundé, 3.81°N, 11.43°E, *Achoundong G.* 872 (YA); Ca 30 km S of Mbalmayo, 3.33°N, 11.5°E, *13 February 1964*, *de Wilde W.J.J.O* 1914 (P,WAG). **East Region**: Village Nkolbisson 7 km West of Yaoundé, 4.16°N, 14.11°E, *15 April 1962*, *Breteler F.J.* 2812 (K,P,WAG); West side of Sangha River, 2.21°N, 16.09°E, *01 November 1988*, *Harris D.J.* 1512 (K,P); Bordure de la Sangha au sud de Lidjombo (près de Libongo) à 110 km au N de Ouesso, 2.93°N, 16.04°E, *10 April 1971*, *Letouzey R.* 10641 (P,YA); Près Ndongo à 45 km WNW de Moloundou, 2.58°N, 15.29°E, *15 March 1973*, *Letouzey R.* 12086 (K,P,WAG,YA); Rive de la Doumé près Bimba (40 km SW de Batouri), 4.16°N, 14.11°E, *15 April 1962*, *Letouzey R.* 4755 (K,P,YA); A 11 km au SSW de Kosso (village situé à 60 km au SSW de Batouri, 3.93°N, 14.17°E, *25 July 1963*, *Letouzey R.* 5491 (K,P,YA); Myko-Malapa(Molundou), 2.05°N, 15.17°E, *21 April 1971*, *Villiers J.-F.* 683 (P,YA). **South Region**: Rives du Ntem près du confluent de la Kye 16 km ESE d’Ambam, 2.30°N, 11.38°E, *07 February 1970*, *Letouzey R.* 10024 (P,YA); Près de Ngomebae 70 km ESE d’Ebolowa sur route de Mvangan, 2.72°N, 11.73°E, *24 January 1970*, *Letouzey R.* 9915 (P,YA). **South-West Region**: Johann-Albrechtshöhe[Kumba] area 4.16°N, 9.2°E, *1896*, *Staudt A.* 556 (K,P).

### ﻿﻿﻿﻿ Uvariopsis congolana

Taxon classificationPlantaeMagnolialesAnnonaceae

(De Wild.) R.E.Fr., Ark. Bot. ser. 2, 3: 42, 1955

80D68B1C-B976-5B3F-A49D-1E04E688CE1E

**Figure 131.**
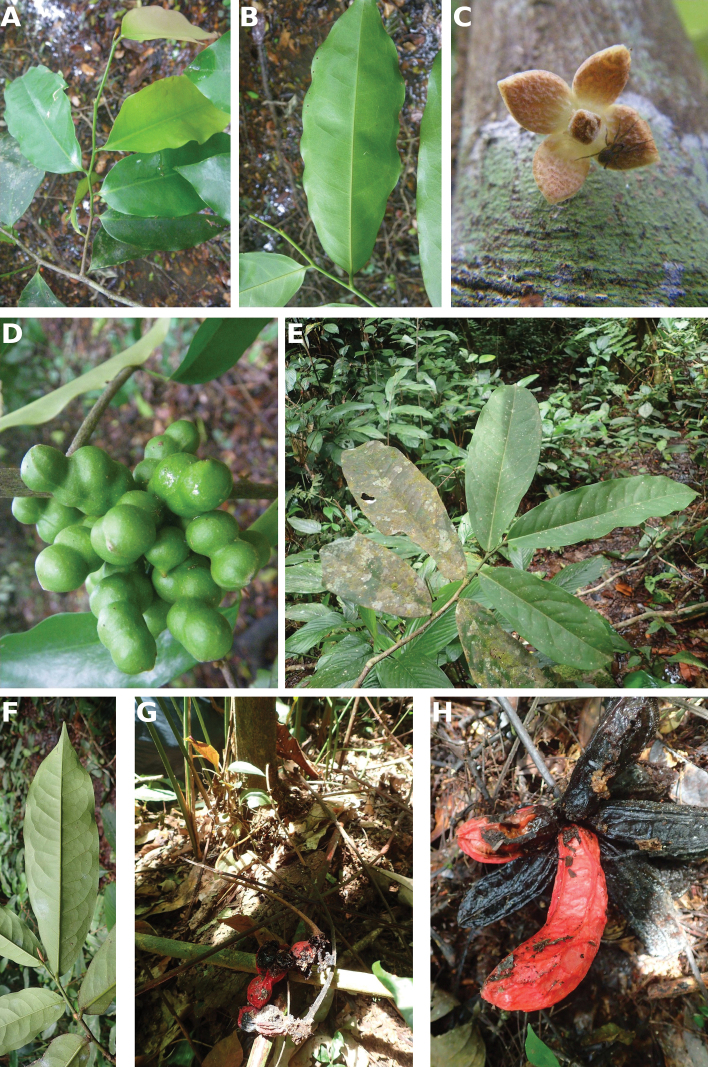
*Uvariopsiscongensis***A** leaves, upper side **B** leaf, lower side **C** male flower **D** fruit, note bumpy smooth monocarps. *Uvariopsiscongolana***E** leaves, upper side **F** leaf, lower side **G** base of trunk, note long fruiting pedicel, with fruit lying on the floor several centimeters away **H** detail of fruit, note finely ribbed monocarp surface **A–D***Lachenaud 1384*, Gabon **E–H***Texier 1144*, Gabon. Photos **A–D** Olivier Lachenaud, Tropicos.org, Missouri Botanical Garden **E–H** Nicolas Texier, Tropicos.org, Missouri Botanical Garden.

Figs 130 , 131 (no map)

≡ Thonnera congolana De Wild., Ann. Mus. Congo Belge, Bot. sér. 5, 3(1): 86.

#### Type.

Democratic Republic of the Congo. Equateur; Liboko, *Thonner F. 100*, 22 Sep 1896: holotype, sheets here designated: BR[BR0000008824394; BR0000008824202]; isotypes: BR[BR0000008824219]

#### Description.

Tree to shrub, 4–10 m tall, d.b.h. 20 cm; stilt roots or buttresses absent. Indumentum of simple hairs; old leafless branches glabrous, young foliate branches pubescent to glabrous. Leaves: petiole 3–8 mm long, 1–4 mm in diameter, sparsely pubescent to glabrous, slightly grooved, blade inserted on top of the petiole; blade 16.3–39.2 cm long, 5–11 cm wide, elliptic to obovate, apex attenuate to acuminate, acumen 1–1.5 cm long, base acute to rounded, papyraceous, below glabrous when young and old, above glabrous when young and old; midrib sunken or flat, above glabrous when young and old, below glabrous when young and old; secondary veins 11 to 22 pairs per side, glabrous above; tertiary venation reticulate. Individuals unisexual, monoecious; inflorescences cauliflorous. Flowers **with 5 perianth parts in 2 whorls**, **conical in bud**, 2 to 3 per inflorescence, male and female inflorescences dimorphic; male and female pedicels 43–450 mm long, 1–2 mm in diameter, sparsely pubescent, **hanging from the base of the trunk and extending on the ground**; in fruit 300–450 mm long, 2–3 mm in diameter, sparsely pubescent to glabrous; bracts 2, one basal and one upper towards the lower half of pedicel, basal bracts 1–2 mm long, 1 mm wide, upper bracts 1–3 mm long, 1–4 mm wide; male sepals 2, valvate, free, ca. 2 mm long, 5–6 mm wide, semiorbicular, apex acute, base truncate, brown, pubescent outside, glabrous inside, margins flat; female sepals 2, valvate, free, ca. 4 mm long, ca. 10 mm wide, semiorbicular, apex acute, base truncate, brown, pubescent outside, glabrous inside, margins flat; **male petals 3**, basally fused, tube 2–3 mm long, ca. 20 mm long, 5–6 mm wide, broadly ovate, apex acute to attenuate, base truncate, margins flat, pubescent outside, glabrous and papillose towards the center, pubescent towards margins inside; **female petals 3**, **basally fused**, tube 2–3 mm long, 20–30 mm long, 8–12 mm wide, ovate, apex acute to attenuate, base truncate, margins flat, pubescent outside, glabrous towards center and pubescent towards margins inside; male flowers: stamens 150 to 200, in 9 to 15 rows, 1 mm long, broad to linear; connective reduced or absent, glabrous, cream; staminodes absent; female flowers: carpels free, 20 to 40, ovary 3–4 mm long, stigma flat, densely pubescent. Monocarps shortly stipitate, stipes 3–4 mm long, 30–80 mm long, 10–35 mm in diameter; monocarps 1 to 5, cylindrical, apex apiculate, sparsely pubescent to glabrous, verrucose, longitudinally 4–6 ribbed, red when ripe; seeds (2)6 to 16 per monocarp, 6–15 mm long, 3–10 mm in diameter, 2 to 16, ellipsoid; aril absent.

#### Distribution.

A central African species, from Cameroon(?) and Gabon to the Democratic Republic of the Congo; unseen and verified specimens were collected in Cameroon from the South-West and South regions.

#### Habitat.

An uncommon(?) species; in lowland or premontane primary or old secondary rain forest. Altitude 200–1000 m a.s.l.

#### Local and common names known in Cameroon.

None recorded.

#### IUCN conservation status.

Least Concern (LC) (Harvey-Brown 2019e).

#### Uses in Cameroon.

None reported.

#### Notes.

﻿﻿﻿*Uvariopsiscongolana* is easily distinguished from other species by the unique combination of 2 sepals and 3 petals. To date in Cameroon its presence has yet to be confirmed, as we were not able to see any of the specimens identified as ﻿﻿﻿*Uvariopsiscongolana*.

### ﻿﻿﻿﻿ Uvariopsis dicaprio

Taxon classificationPlantaeMagnolialesAnnonaceae

Cheek & Gosline, PeerJ 9(e12614): 8, 2022

B02901E3-7F66-5388-A955-368D1DED0E97

Fig. 132 ; Map 16B

= Uvariopsis ebo Cheek & Gosline, https://doi.org/10.1101/2021.03.26.437154; *nom. nud.*

#### Type.

Cameroon. Littoral Region, Yabassi, Ebo Forest, Dicam Trail 2000 m from Bekob camp, *MacKinnon L.E. 51*, 25 March 2008; holotype K [K001381842]; isotypes MO, YA.

#### Description.

**Tree, 3–4 m tall**, d.b.h. 2–3 cm; stilt roots or buttresses absent. Indumentum of simple hairs; old leafless branches glabrous, young foliate glabrous. Leaves: petiole 4(5) mm long, ca. 2 mm in diameter, glabrous, blade inserted on top of the petiole; blade 17–20(–23) cm long, 6–8 cm wide, oblanceolate, apex acuminate, acumen 1–1.5 cm long, broadly acute but minutely cordate, coriaceous, below glabrous when young and old, above glabrous when young and old; midrib sunken, above glabrous when young and old, below glabrous when young and old; secondary veins 5 to 8(9) pairs per side, glabrous above; tertiary venation reticulate. Individuals unisexual [?, female flowers unknown], monoecious [?]; male inflorescences cauliflorous, scattered along the trunk. Male flowers with 6 perianth parts in 2 whorls, narrowly ovoid to pyramidal in bud; **1 to 7 per inflorescence borne on a short peduncle**; **pedicel ca. 18–25 mm long**, ca. 1 mm in diameter, glabrescent to glabrous; bracts (1)2, one basal and one upper towards the lower half of pedicel, similar in size, ca. 1.5 mm long, ca. 0.5 mm wide; sepals 2, valvate, free, ca. 1–1.5 mm long, ca. 2 mm wide, semi-orbicular, apex acute, base truncate, glabrous, margins flat; petals free, 4 in one whorl, 14–16 mm long, ca. 5–9 mm wide, lanceolate-oblong, apex rounded, base truncate, yellow-green, margins flat, sparsely pubescent outside, glabrous inside; stamens ca. 300 in ca. 10 whorls, ca. 0.5 mm long; connective absent; staminodes absent. Fruits unknown.

**Figure 132.**
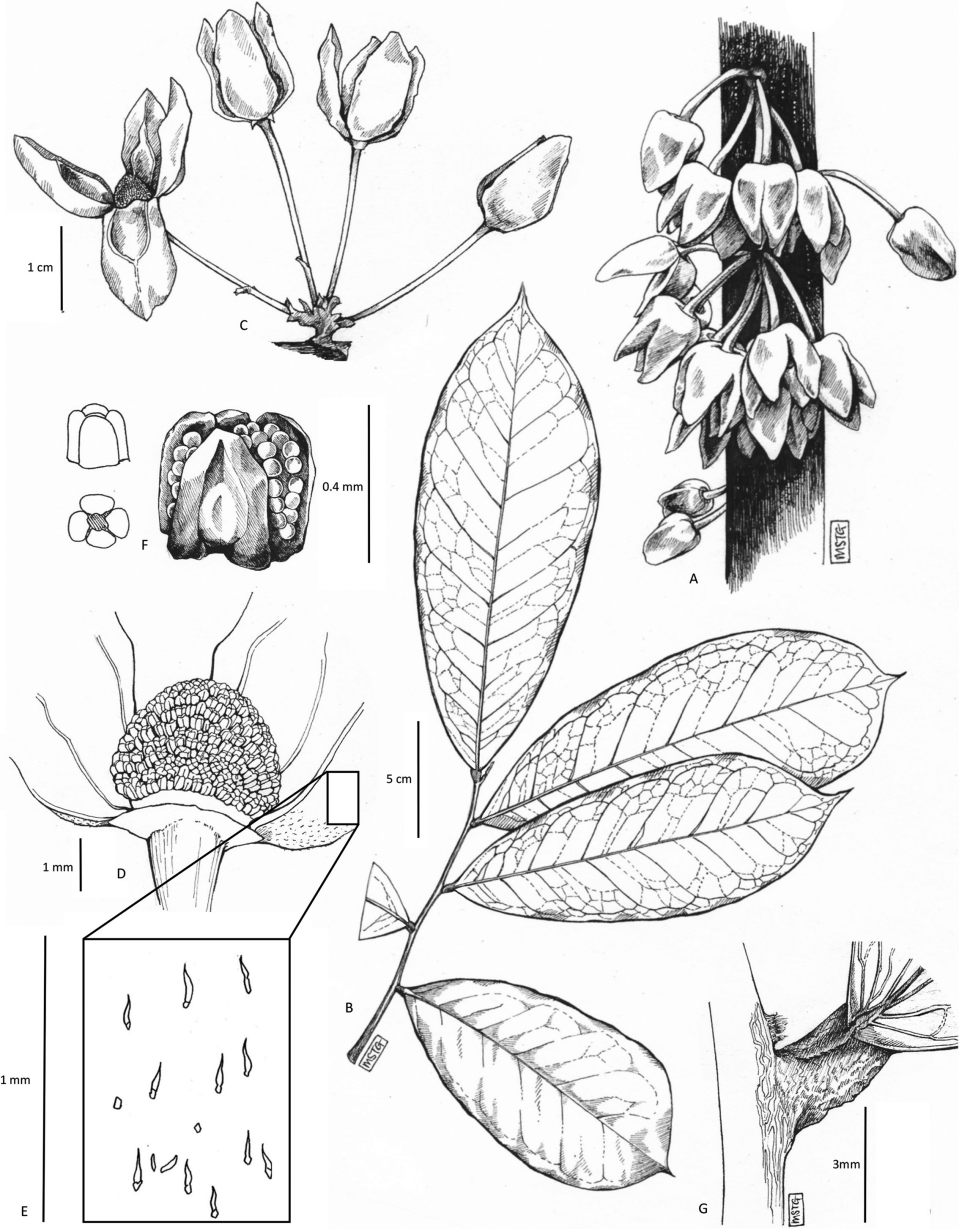
*Uvariopsisdicaprio***A** habit, cauliflorous inflorescences on trunk **B** leafy branch, one season’s growth **C** inflorescence, showing pedicel articulations, bracts and bracteoles **D** flower, with one petal removed to show the staminal dome **E** detail of sparse hairs on abaxial petal surface **F** stamen, different views **G** junction of base of leaf with stem, showing dome-like axillary bud **A–G***MacKinnon 51*, Ebo, Cameroon. Drawings by Meg Griffiths, reproduced with permission from Gosline et al. (2022, fig. 3, page 5) DOI https://doi.org/10.7717/peerj.12614/fig-3.

#### Distribution.

Endemic to Cameroon; known from the Littoral region.

#### Habitat.

A rare species only known by the type, in submontane primary rain forests. Altitude 850 m a.s.l.

#### Local and common names known in Cameroon.

None recorded.

#### IUCN conservation status.

Not officially evaluated, but suggested as CR B1+2ab(iii), D (Gosline et al. 2022).

#### Uses in Cameroon.

None reported.

#### Notes.

﻿﻿﻿*Uvariopsisdicaprio* is only known by the type specimen. It is distinguished from the other species by its flowers with yellow-green petals.

### ﻿﻿﻿﻿ Uvariopsis dioica

Taxon classificationPlantaeMagnolialesAnnonaceae

(Diels) Robyns & Ghesq., Ann. Soc. Sci. Bruxelles, Ser. B 53: 321, 1933

77A237D8-614E-5821-8D03-2409CC8A73A6

**Figure 133.**
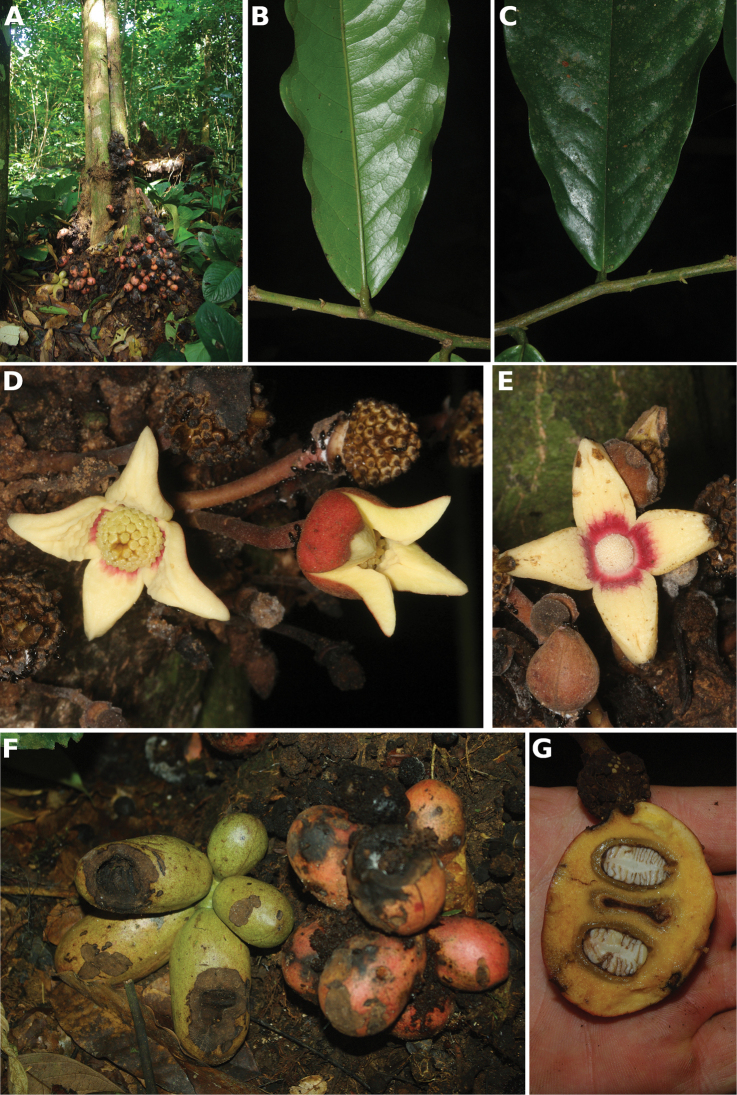
*Uvariopsisdioica***A** habit, note the cluster of fruits at the base of the trunk **B** leaf base, under side **C** leaf base, upper side **D** cluster of female flowers **E** detail of male flower **F** cluster of fruits **G** longitudinal section of monocarp **A–C, F–G***Couvreur 654*, Mambe, Cameroon **D, E***Stévart 4792*, Gabon. Photos **A–C, F, G** Thomas L.P. Couvreur **D, E** Tariq Stévart.

Figs 127 , 133 ; Map 16C

≡ Tetrastemma dioicum Diels, Bot. Jahrb. Syst. 38(3): 241, 1906.

#### Type.

Cameroon. Littoral Region; Ed *Winkler H. 909*, Nov 1904: lectotype, designated here: B[B 10 0153121].

#### Description.

Tree to shrub, 6–20 m tall, d.b.h. 14–40 cm; stilt roots or buttresses absent. Indumentum of simple hairs; old leafless branches glabrous, young foliate branches pubescent to glabrous. Leaves: petiole 2–5 mm long, 1–2.5 mm in diameter, sparsely pubescent to glabrous, narrowly grooved, blade inserted on top of the petiole; **blade 11.1–24.5 cm long, 3.8–9.2 cm** wide, **elliptic to obovate, apex attenuate to acuminate, acumen 0.7–3 cm long, base rounded to acute to decurrent**, coriaceous, below sparsely pubescent to glabrous when young, glabrous when old, above glabrous when young and old; midrib sunken or flat, above glabrous when young and old, below glabrous to pubescent when young, glabrescent when old; secondary veins 6 to 14 pairs per side, glabrous above; tertiary venation reticulate. Individuals unisexual, monoecious, dimorphic. Flowers with 6 perianth parts in 2 whorls. Male inflorescences cauliflorous, **5 to 20-flowered or more, on thick clumps on the lower part of the trunk**; male flowers: pedicel 11–50 mm long, 1–2 mm in diameter, sparsely pubescent to glabrous; bracts 2, one basal and one upper towards the middle or lower half of pedicel, basal bract minute, ca. 1 mm long, ca. 1 mm wide; upper bract 0.5–1.5 mm long, 1–2 mm wide; sepals 2, valvate, basally fused, 1.5–4.5 mm long, 2.5–11 mm wide, circular to broadly ovate, apex acute or rounded, base truncate, purple, pubescent to sparsely pubescent outside, glabrous inside, margins flat; petals free, 4 in one whorl, 6–11 mm long, 3.5–8 mm wide, ovate to elliptic, apex acute, base truncate, wine red to dark brownish-red, margins flat, pubescent to glabrous outside, glabrous inside; stamens ca. 300, in 20 to 25 rows on a convex receptacle, 0.4–0.5 mm long; connective minute, glabrous; staminodes absent. Female inflorescences cauliflorous, axillary, **clumps of 6 to 10 flowers or more, on thick clumps on the lower part of the trunk**; female flowers: pedicel 10–50 mm long; 2–3 mm in diameter, sparsely pubescent to glabrous; in fruit 8–55 mm long, 2–4 mm in diameter, sparsely pubescent to glabrous; bracts 2, one basal and one upper towards the middle or lower half of pedicel, basal bract minute, ca. 1 mm long, ca. 1 mm wide; upper bract 0.5–1.5 mm long, 1–2 mm wide; sepals 2, valvate, basally fused, 2–5 mm long, 2–11 mm wide, circular to broadly ovate, apex acute or rounded, base truncate, purple, pubescent to sparsely pubescent outside, glabrous inside, margins flat; petals free, 4, in one whorl, 7–20 mm long, 4–15 mm wide, ovate to elliptic, apex acute, base truncate, wine red to dark red-brownish outside, margins flat, pubescent to glabrous outside, glabrous inside; **carpels free, 100 to 280**, ovary 1.5–2.5 mm long, stigma coiled bilobed, glabrous. Monocarps stipitate, stipes 0–2 mm long, ca. 4 mm in diameter; monocarps 2 to 5, 21–60 mm long, 30–35 mm in diameter, ovoid to cylindrical, apex rounded, sparsely pubescent to glabrous, smooth, not ribbed, pale brownish grey to yellow to red; seeds 5 to 8 per monocarp, 15–22 mm long, ca. 15 mm in diameter, ellipsoid; aril absent.

#### Distribution.

A central African species, from Nigeria to Republic of the Congo; in Cameroon known from the Central, East, Littoral, South and South-West regions.

#### Habitat.

A common species, in lowland primary or old secondary rain forests. Altitude 50–800 m a.s.l.

#### Local and common names known in Cameroon.

None recorded.

#### IUCN conservation status.

Not evaluated.

#### Uses in Cameroon.

None reported.

#### Notes.

﻿﻿﻿*Uvariopsisdioica* resembles ﻿*U.pedunculosa* and ﻿*U.solheidii* by its elliptic to obovate shape of its leaves and the acute to rounded shape of the leaf base and attenuate to acuminate apex. However, ﻿﻿﻿*Uvariopsisdioica* has flowers borne in thick clumps of 5 to 20 flowers on the lower (less than 3 m) part of the trunk (versus 1 or 2 flowers per inflorescence on think clumps) and with 100 to 240 carpels (versus less than 100 in most other species). Clustered inflorescences on the basal part of the trunk is also found in the Cameroonian endemic species ﻿﻿﻿*Uvariopsiskorupensis*, but ﻿*U.dioica* has smaller leaves (11–25 cm long vs. 28–62 cm long).

Robyns and Ghesquière (1933) indicate two types for ﻿*U.dioica*: *Winkler 908* and *909*. We only located sheet *909* in B, while sheet *908* was not seen (nor is it available online), and is thus probably destroyed.

Contrary to what the name suggests, ﻿﻿﻿*Uvariopsisdioica* is not dioecious but monoecious. The specific epithet probably comes from a misinterpretation of the species based only on the original type specimens composed of a sheet with male flowers (*Winkler 908*) and a sheet with female flowers (*Winkler 909*). Unisexual individuals might exist, but all the specimens we examined have both female and male flowers.

﻿﻿﻿*Uvariopsispedunculosa* (Diels) Robyns & Ghesq. (﻿*Tetrastemmapedunculosum* Diels) was synonymized by Keay under ﻿*U.dioica* (Keay 1952) but is here regarded as a separate species.

#### Specimens examined.

**Central Region**: Reserve forestière de Makak au bord du Nyong, 3.59°N, 11.03°E, *14 December 1967*, *Bamps P.R.J.* 1458 (P,YA); Ndiki, 4.77°N, 10.83°E, *01 November 1938*, *Jacques-Félix H.* 2493 (P); Forêt de Mambé près Boga (30 km N Eseka), 3.9°N, 10.78°E, *08 December 1973*, *Letouzey R.* 12290 (K,P,YA); Mfiki (Ndo par Esse), 4.31°N, 11.96°E, *09 November 1969*, *Letouzey R.* 9541 (P,WAG); Etwa 115 km NO Juande, 4.48°N, 12.35°E, *01 January 1914*, *Mildbraed G.W.J.* 8260 (K). **East Region**: Réserve de faune du Dja Djolimpoun, 3.17°N, 13.18°E, *17 April 1995*, *Sonké B.* 1505 (BR,YA). **Littoral Region**: Mambe Massif above Boga village 100 km along road from Yaoundé to Ed 3.90°N, 10.77°E, *19 June 2014*, *Couvreur T.L.P.* 654 (WAG,YA); Mambe Massif above Boga village 100 km along road from Yaoundé to Ed 3.90°N, 10.77°E, *20 June 2014*, *Couvreur T.L.P.* 659 (WAG,YA); Au sud de Ngola (8 km Est de l’embouchur de la Sanaga), 3.55°N, 9.698°E, *05 January 1974*, *Letouzey R.* 12580 (P,YA); Ed 3.81°N, 10.13°E, *01 January 1904*, *Winkler H.* 909 (B). **South Region**: N’koladom village 4 km on the road (old road) from Nkoemvone to Ambam, 2.8°N, 11.15°E, *27 November 1974*, *de Wilde J.J.F.E* 7754 (K,P,WAG); Nkoemvone, 2.81°N, 11.13°E, *05 June 1975*, *de Wilde J.J.F.E* 8270a (BR,MO,P,WAG); Station de cacaoyer de N’koemvone 14 km On the road from Ebolowa to Ambam, 2.8°N, 11.13°E, *12 December 1975*, *de Wilde J.J.F.E* 8709 (BR,MO,P,WAG,YA); Nkomo près Ngoase au S de la rive Lobe, 3.26°N, 12.02°E, *13 February 1962*, *Letouzey R.* 4219 (K,P,YA); Nkomo près Ngoase au S de la rive Lobe, 3.26°N, 12.02°E, *14 February 1962*, *Letouzey R.* 4230 (BR,P,YA); Près Mevous 50 km SE d’Ebolowa sur piste d’Evindissi, 2.6°N, 11.46°E, *30 January 1970*, *Letouzey R.* 9934 (BR,K,P,YA). **South-West Region**: ca 40 minutes walk N then E from Njonji Hunters path to Lake Njonji, 4.13°N, 8.993°E, *18 November 1993*, *Cheek M.* 5482 (K,YA); ca 40 minutes walk N then E from Njonji Hunters path to Lake Njonji, 4.13°N, 8.993°E, *19 November 1993*, *Cheek M.* 5501 (K,MO,WAG,YA); Entre Ayong et Baro 20 km SW Nguti, 5.2°N, 9.32°E, *10 June 1975*, *Letouzey R.* 13790 (P,YA); Bibundi, 4.21°N, 8.988°E, *01 November 1928*, *Mildbraed G.W.J.* 10647 (K); Pente SW Mt Cameroun ME Bakingili WNW Limbé, 4.07°N, 9.04°E, *09 December 1984*, *Villiers J.-F.* 2429 (P,YA); Limbe W of Njonji Lake, 4.13°N, 9.016°E, *27 January 1994*, *Wieringa J.J.* 2029 (WAG).

### ﻿﻿﻿﻿ Uvariopsis etugeana

Taxon classificationPlantaeMagnolialesAnnonaceae

Dagallier & Couvreur sp. nov.

C6190149-96FA-54BC-BD10-4A98A25BB9B0

urn:lsid:ipni.org:names:77305100-1

Map 16D

#### Diagnosis.

﻿﻿﻿*Uvariopsisetugeana* resembles *U.peduculosa* in being a small tree and in the shape of its leaves, but differs by having a short flowering pedicel (4–10 mm versus 25–130 mm in ﻿*U.pedunculosa*) and glabrous petals on the outside (versus pubescent in both ﻿*U.pedunculosa*).

#### Type.

Cameroon. North-West Region; Wum, *Letouzey R. 13414*, 3 Dec 1974: holotype: P[P01982826].

#### Description.

**Tree, 3–6 m tall**, d.b.h. unknown; stilt roots or buttresses absent. Indumentum of simple hairs; old leafless branches glabrous, young foliate branches slightly pubescent to glabrous. Leaves: petiole 3.5–4 mm long, 2.5–3.5 mm in diameter, glabrous, blade inserted on top of the petiole; blade 19–27 cm long, 7–9.5 cm wide, elliptic, apex attenuate to acuminate, acumen 1–2 cm long, base acute to slightly decurrent, coriaceous, below glabrous when young and old, above glabrous when young and old; midrib sunken, above glabrous when young and old, below glabrous when young and old; secondary veins 8 to 10 pairs per side, glabrous above; tertiary venation reticulate. Individuals unisexual, monoecious, dimorphic; inflorescences cauliflorous and ramiflorous on leafless branches, axillary. Flowers with 6 perianth parts in 2 whorls; 1 to 3 per inflorescence. Male flowers: **pedicel ca. 8 mm long, 1.5 mm in diameter**, glabrescent to glabrous; bracts 2, one basal and one upper towards the middle or lower half of pedicel, basal bract minute, ca. 1 mm long, ca. 1 mm wide; upper bract 1–1.5 mm long, 1–2.5 mm wide; sepals 2, valvate, basally fused, ca. 2 mm long, ca 4. mm wide, broadly ovate, apex acute or rounded, base truncate, sparsely pubescent to glabrous outside, glabrous inside, margins flat; petals free, 4 in one whorl, ca. 10 mm long, ca. 4 mm wide, elliptic to ovate, apex acute, base truncate, color unknown, margins flat, **glabrous outside, glabrous inside**; stamens numerous (exact number unknown), ca. 0.5 mm long; connective minute, glabrous; staminodes absent. Female flowers: **pedicel ca. 4 mm long, ca. 2 mm in diameter**, glabrescent to glabrous; in fruit unknown; bracts 2, one basal and one upper towards the middle or lower half of pedicel, basal bract minute, ca. 1 mm long, ca. 1 mm wide; upper bract ca. 1.5 mm long, ca. 2 mm wide; sepals 2, valvate, **basally fused**, 1.5 mm long, 3.5 mm wide, broadly ovate, apex acute or rounded, base truncate, **sparsely pubescent to glabrous outside, glabrous inside**, margins flat; petals free, 4, in one whorl, ca. 14 mm long, ca. 8 mm wide, elliptic, apex acute, base truncate, color unknown, margins flat, glabrous outside, glabrous inside; sterile stamens, ca. 5, ca. 1 mm long ; carpels free, ca. 20, ovary ca. 3 mm long, ca. 1 mm wide, stigma unknown, glabrescent at base to glabrous. Fruits unknown.

#### Distribution.

Endemic to Cameroon, only known from two localities in North-West and South-West Regions.

#### Habitat.

A rare species, only known from two collections to date; in mature rain forests or semi-deciduous forests. Altitude 170–700 m a.s.l.

#### Local and common names known in Cameroon.

None recorded.

#### IUCN conservation status.

Not evaluated.

#### Uses in Cameroon.

None reported.

#### Etymology.

Named in honor of the late Martin Etuge Ekwoge (1966–2020), a passionate Cameroonian horticulturalist, botanist and parataxonomist from Nyassosso village, South-West Cameroon. He was one of the main collectors for the ‘Plants of Mount Mwanenguba and the Bakossi Mountains’ (Cheek et al. 2004). For a total of 14,538 specimens recorded in the Kew database for Bakossi, 3,170 were collected by him (Cheek et al. 2020) including over 652 (that we have seen) specimens of Annonaceae (representing 28 species).

#### Notes.

﻿﻿﻿*Uvariopsisetugeana* resembles ﻿*U.pedunculosa* in the shape of its leaves, and ﻿*U.solheidii* in the shape of its flowers. However, ﻿*U.etugeana* has a short flowering male or female pedicel (< 10 mm versus 25–130 mm in ﻿*U.pedunculosa* and 22–160 mm in ﻿*U.solheidii*) and petals which are glabrous on the outside versus pubescent in both *U.pedunculosa* and ﻿*U.solheidii*.

#### Specimen examined.

**South-West Region**: Takamanda forest reserve near Matene, 6.23°N, 9.316°E, *21 March 1985*, *Thomas D.W.* 4544 (MO,YA).

### ﻿﻿﻿﻿ Uvariopsis korupensis

Taxon classificationPlantaeMagnolialesAnnonaceae

Gereau & Kenfack, Adansonia sér. 3, 22(1): 41, 2000

F7165758-A9E6-50C6-9F6C-B3F5A11A59AD

**Figure 134.**
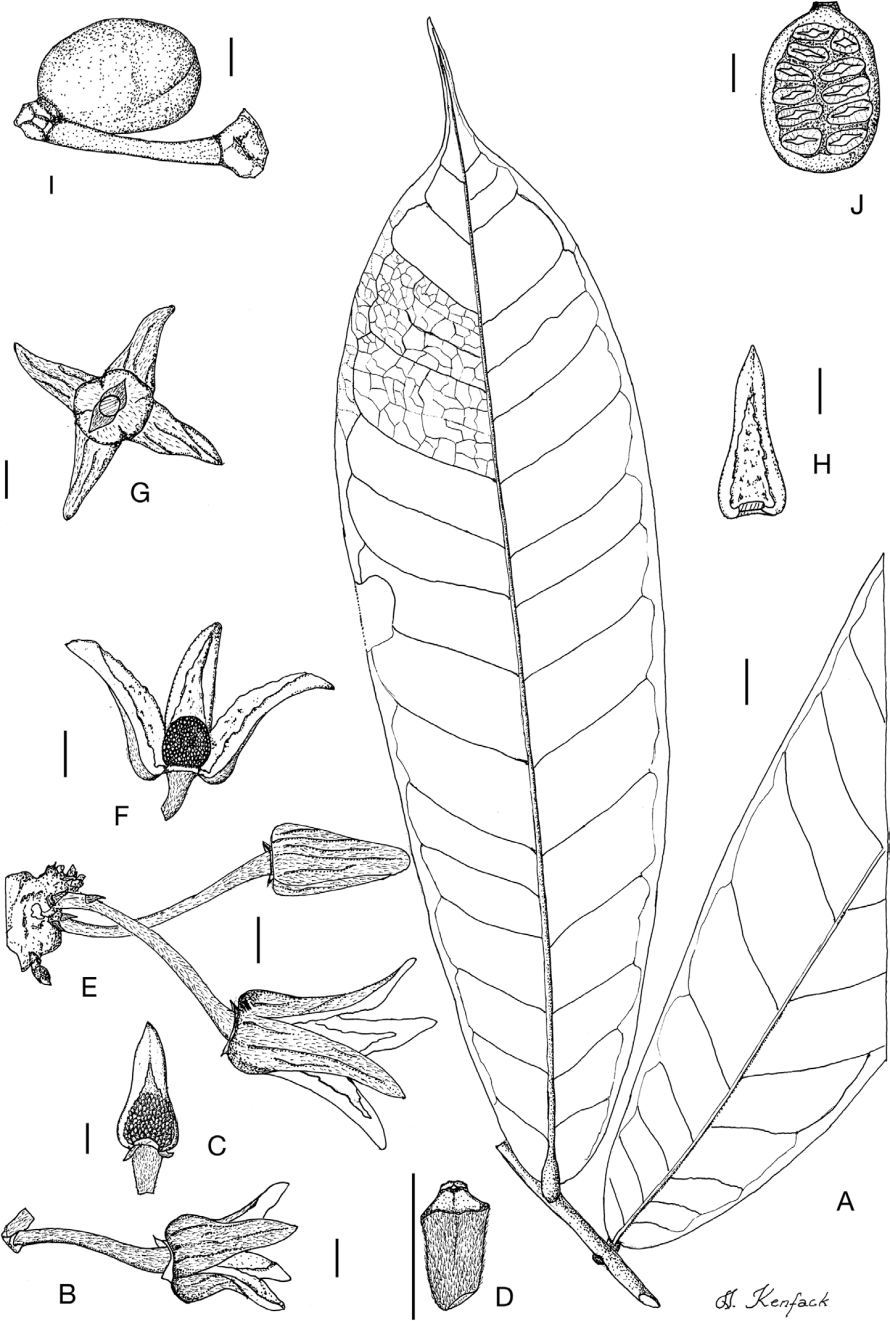
*Uvariopsiskorupensis***A** branch with leaves **B** female flower **C** female flower, three petals removed **D** detail of carpel **E** male flower and male flower bud **F** male flower, one petal removed **G** male flower, bottom view **H** petal of male flower, inner view **I** fruit, with a single monocarp **J** longitudinal section of monocarp **A** from Kenfack 1146 **B–K** from fresh material. Scale bars: 1 cm (**A–C, E–K**); 0.5 cm (**D**). Drawings by David Kenfack. Reproduced with permission from Gereau and Kenfack (2000, page 42).

**Figure 135.**
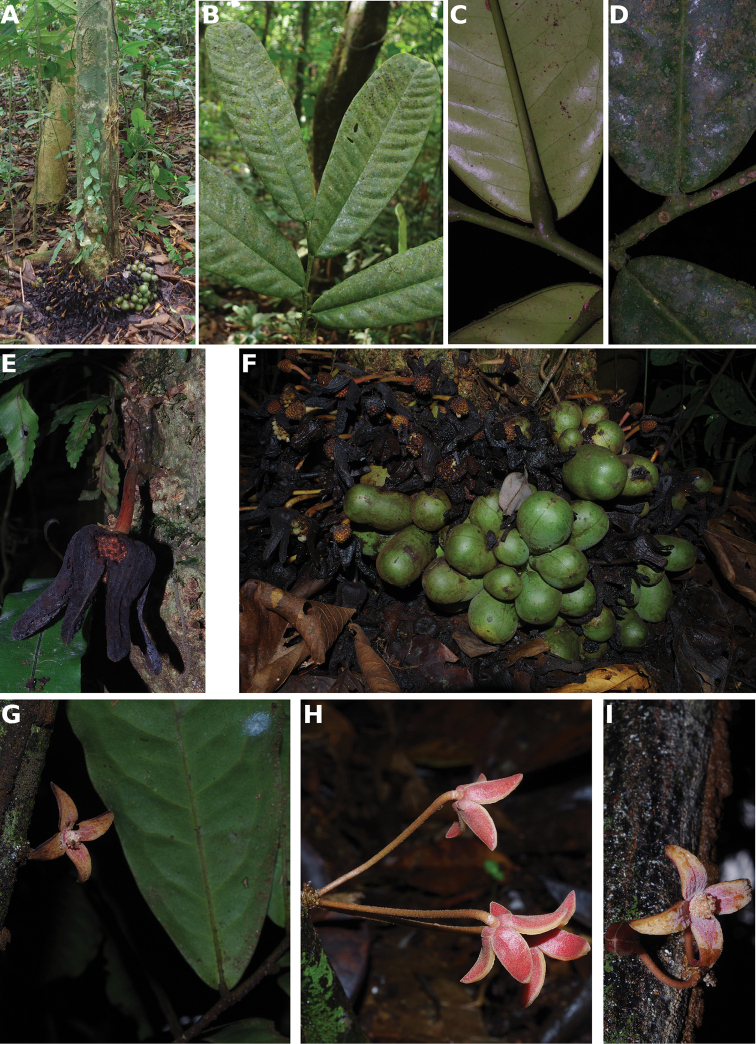
*Uvariopsiskorupensis***A** trunk, note cluster of old flowers and fruits at base **B** leaves **C** base of leaf, lower side **D** base of leaf, upper side **E** old female flower on trunk **F** cluster of fruits at base of trunk. *Uvariopsissolheidii***G** male flower and leaf base, lower side **H** male flowers, side view **I** detail of male flower **A–F***Couvreur 1052*, Mt Cameroon, Cameroon **G–I***Couvreur 855*, Gabon. Photos Thomas L.P. Couvreur.

Figs 134 , 135 ; Map 16E

#### Type.

Cameroon. South Region; Korup National Park, Chimpanzee Camp, 5°04'N, 8°52'E, *Kenfack D. 1026*, 3 Fev 1998: holotype: YA; isotypes: MO[MO-022919]; P[P01817719]; WAG[WAG0358388].

#### Description.

Tree to shrub, 6–15 m tall, d.b.h. 12–14 cm; stilt roots or buttresses absent. Indumentum of simple hairs; old leafless branches glabrous, young foliate branches glabrous to pubescent. Leaves: petiole 2–7 mm long, 2–6 mm in diameter, glabrous to pubescent, slightly grooved, blade inserted on top of the petiole; blade 30–61.5 cm long, 5.9–16.5 cm wide, elliptic to obovate, apex abruptly acuminate, acumen 1.8–3.2 cm long, base rounded to cordate, coriaceous, below glabrous when young and old, above glabrous when young and old; midrib sunken or flat, above glabrous when young and old, below glabrous when young and old; secondary veins 10 to 26 pairs per side, glabrous above; tertiary venation reticulate. Individuals unisexual, monoecious; inflorescences cauliflorous, towards base and up to 3 m on trunk. Flowers with 6 perianth parts in 2 whorls, **ovoid to conical in bud**, 2 to 3 per inflorescence, male and female inflorescences dimorphic; male pedicels 6–35 mm long, 1–2 mm in diameter, tomentose; female pedicels 16–50 mm long, 1–2 mm in diameter, tomentose; in fruit 45–90 mm long, 2 mm in diameter, glabrous; bracts 2 to 4, all basal, 1–2 mm long, ca. 1 mm wide; male sepals 2, valvate, basally fused, 2–7.5 mm long, 3–6.5 mm wide, ovate, apex acute, base truncate, greenish, pubescent outside, glabrous inside, margins flat; female sepals 2, valvate, basally fused, 1.5–5 mm long, 1.5–5 mm wide, ovate, apex acute, base truncate, greenish, pubescent outside, glabrous inside, margins flat; male petals 4, basally fused, tube 1–2 mm long, 10–38 mm long, 5–10 mm wide, elliptic to ovate, apex acute to attenuate, base truncate, greenish-yellow, margins flat, pubescent outside, glabrous inside; female petals 4, basally fused, tube 3–5 mm long, 15–35 mm long, 8–12 mm wide, elliptic, apex acute to attenuate, base truncate, greenish-yellow, margins flat, pubescent outside, glabrous inside; male flowers: stamens 130 to 210, in 9 to 12 rows, 0.5–1 mm long, oblong; connective reduced or absent, glabrous, cream; staminodes absent; female flowers: carpels free, 25 to 40, ovary 3–4 mm long, stigma flat, glabrous. Monocarps stipitate, stipes 2–5 mm long, 2 mm in diameter; monocarps 5 to 9, 30–60 mm long, 18–30 mm in diameter, **ellipsoid to cylindrical, apex rounded, glabrous, smooth**, clear orang yellow when ripe; seeds 8 to 14 per monocarp, 10–22 mm long, 5–14 mm in diameter, ellipsoid to oblong; aril absent.

#### Distribution.

endemic to Cameroon; known from the South, South-West and Littoral regions.

#### Habitat.

In primary or old secondary lowland rain forests. Altitude 100–700 m a.s.l.

#### Local and common names known in Cameroon.

None recorded.

#### IUCN conservation status.

Least Concern (LC) (Cheek 2014c).

#### Uses in Cameroon.

None reported.

#### Notes.

﻿﻿﻿*Uvariopsiskorupensis* is distinguished by its conical flower buds with basally fused petals, and flowers not completely covering the lower part of the trunk; it closely resembles ﻿*U.submontana* (see that species for differences).

In the check list of Mt Cameroon (Cable and Cheek 1998), one collection is identified as ﻿*Uvariopsis* sp. (*Tchouto 675*) which has now been identified as ﻿*U.korupensis*.

#### Specimens examined.

**Littoral Region**: Nkam Yingui Ekem River bank Forest, 4.35°N, 10.41°E, *05 March 2002*, *Kenfack D.* 1620 (MO). **South Region**: Campo-Ma’an area Bifa, 2.67°N, 10.28°E, *13 October 2001*, *Tchouto Mbatchou G.P.* BIFAX_25 (WAG); Bipindi, 3.08°N, 10.41°E, *01 January 1911*, *Zenker G.A.* 3971 (K). **South-West Region**: ca 40 minutes walk N then E from Njonji Hunters path to Lake Njonji, 4.13°N, 8.993°E, *19 November 1993*, *Cheek M.* 5486 (K,WAG,YA); Ekundu Kundu, 5.15°N, 8.883°E, *28 April 1996*, *Cheek M.* 8258 (K,WAG,YA); Ekundu kundu, 5.15°N, 8.89°E, *09 January 1998*, *Cheek M.* 8815 (K,YA); Mount Cameroon National Park Bakinguili trail above Bakinguili village, 4.09°N, 9.056°E, *02 April 2016*, *Couvreur T.L.P.* 1039 (WAG,YA); Mount Cameroon National Park on the Bomona trail behind Bomona village 10 km NW from Idenau, 4.29°N, 9.078°E, *03 April 2016*, *Couvreur T.L.P.* 1052 (WAG,YA); Korup National Park, 4.95°N, 8.87°E, *05 March 1993*, *Gereau R.E.* 5192 (MO,P,WAG); Korup National Park, 5.06°N, 8.866°E, *03 February 1998*, *Kenfack D.* 1026 (MO,P,WAG,YA); Bakingili, 4.08°N, 9.05°E, *16 February 1997*, *Nning J.* 284 (K,YA); Bomana, 4.25°N, 9.016°E, *05 October 1993*, *Tchouto Mbatchou G.P.* 675 (K,YA); Korup National Park, 5.05°N, 8.8°E, *28 April 1984*, *Thomas D.W.* 3182 (L,P,YA); Korup National Park, 5.05°N, 8.8°E, *28 February 1984*, *Thomas D.W.* 3210 (BR,YA); Mount Cameroun above small Koto village, 4.3°N, 9.1°E, *06 March 1985*, *Thomas D.W.* 4477 (MO,YA).

### ﻿﻿﻿﻿ Uvariopsis pedunculosa

Taxon classificationPlantaeMagnolialesAnnonaceae

(Diels) Robyns & Ghesq., Ann. Soc. Sci. Bruxelles, Ser. B 53: 321 1933

15DC421D-DDEF-5D34-B962-B513FC508D15

**Figure 136.**
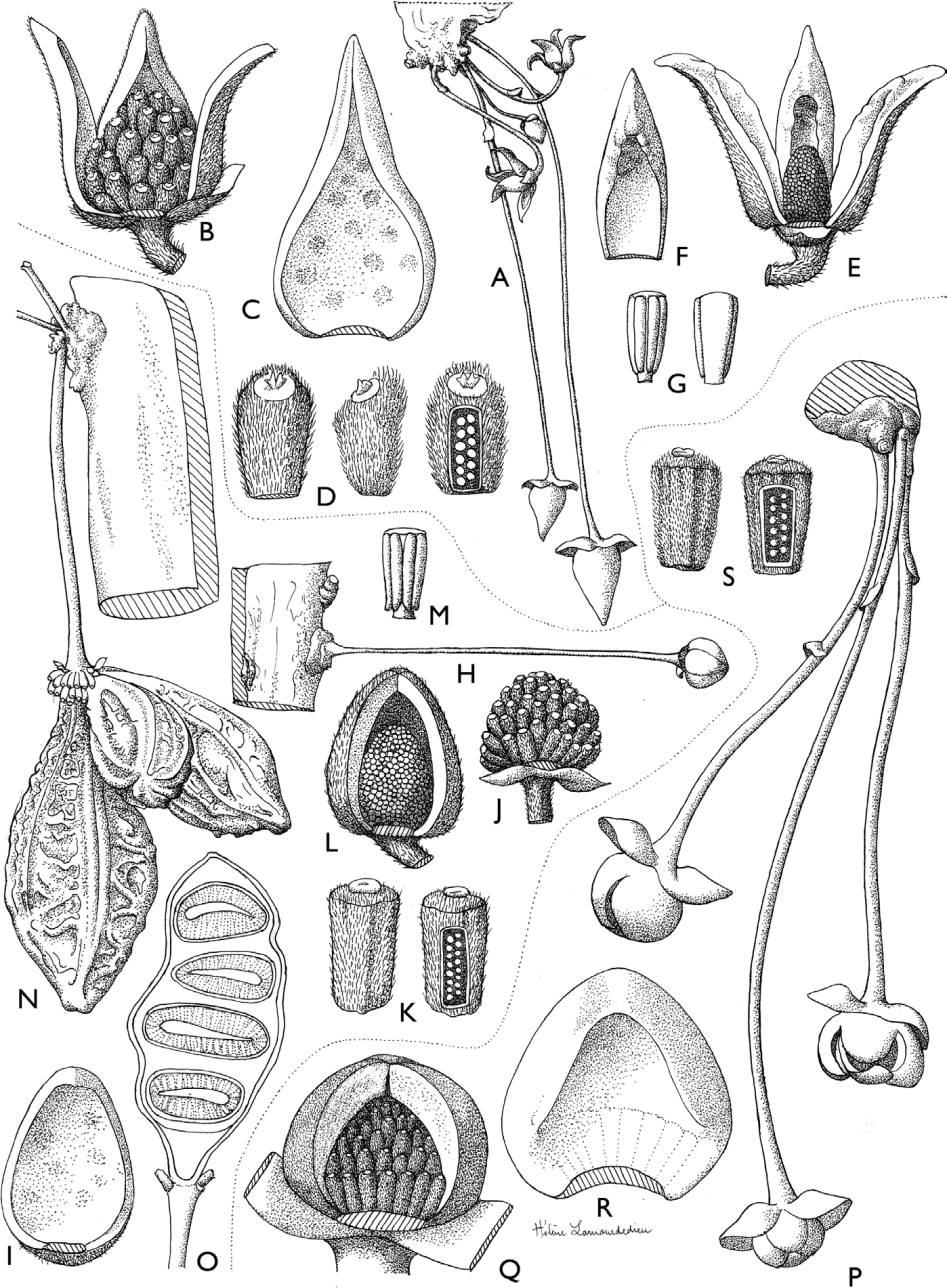
*Uvariopsissolheidii***A** male inflorescence **B** detail of female flower, 1 petal removed **C** petal of female flower, inner view **D** carpel, side and front views, detail of ovules **E** male flower, 1 petal removed **F** petal of male flower, inner view **G** stamen, front and side views. *Uvariopsisletestui* Pellegr. (not present in Cameroon) **H** flower **I** petal of female flower, inner view **J** female flower, all 4 petals removed **K** carpel, front view and detail of ovules **L** male flower, one petal removed **M** stamen, front view **N** fruits **O** longitudinal section of a monocarp. *Uvariopsispedunculosa* (as *U.vanderystii*) **P** female inflorescence **Q** female flower, one petal removed **R** petal of female flower, inner view **S** carpel, side view and detail of ovules **A** from *Tisserant 2242***B–G** from *Tisserant 804***H–M** from *Hallé 3060***N–O** from *Hallé 2975***P–S** from *Le Testu 8525*. Drawings by Hélène Lamourdedieu, Publications Scientifiques du Muséum national d’Histoire naturelle, Paris; modified from Le Thomas (1969b; pl. 54, p. 299).

**Figure 137.**
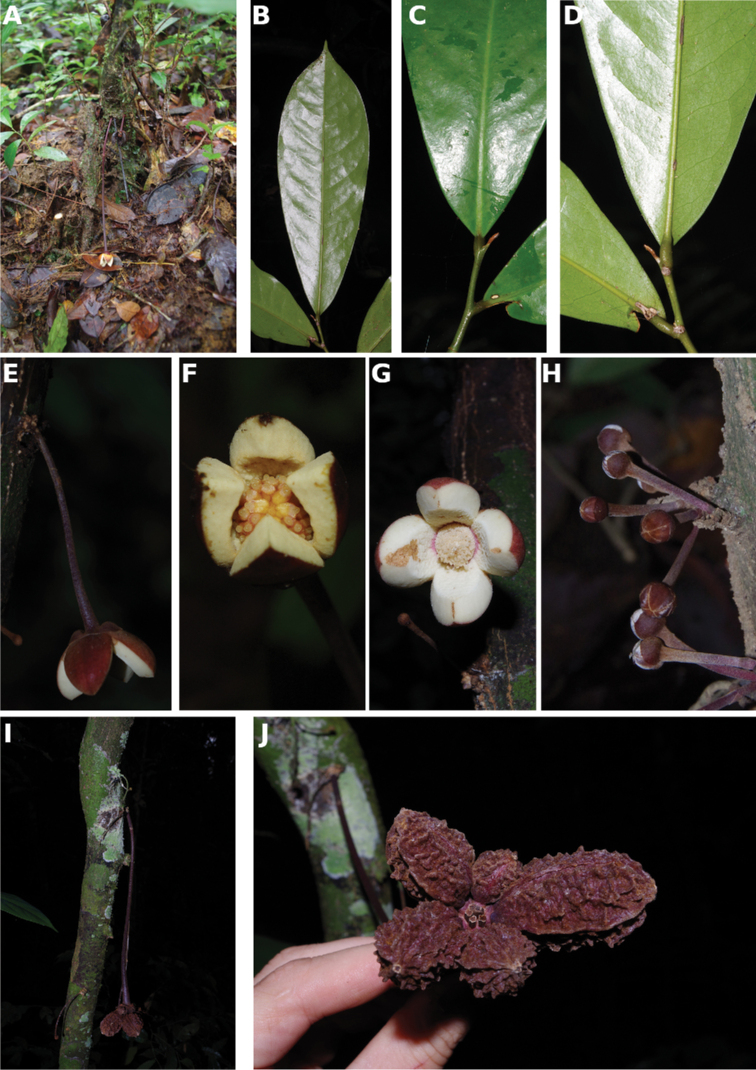
*Uvariopsispedunculosa***A** base of trunk, note flower on long pedicel on ground **B** leaf, upper side **C** leaf base, upper side **D** leaf base, lower side **E** female inflorescence on trunk **F** female flower **G** male flower **H** flower buds **I** fruiting pedicel **J** fruit **A, F***Couvreur 594*, Gabon **B–D***Couvreur 1066*, Cameroon **E, G***Couvreur 878*, Gabon **H***Couvreur 1173*, Mapubi, Cameroon **I, J***Couvreur 885*, Gabon. Photos Thomas L.P. Couvreur.

Figs 136 , 137 ; Map 16F

≡ Tetrastemma pedunculosum Diels, Bot. Jahrb. Syst. 53(3–5): 441. 1915.
= Uvariopsis vanderystii Robyns & Ghesq., Ann. Soc. Sci. Bruxelles, Ser. B 53: 64, 1933. Syn. nov. Type. Democratic Republic of the Congo. Bandundu; Kikwit, Vanderyst H. 9973, 1921: holotype: BR[BR0000008824387].

#### Type.

Cameroon. South Region, Bipindi, *Zenker G.A. 3868*, Mar 1906: B[B 10 0153122]; isotypes: BM[BM000554078]; BR[BR0000008824196, BR0000008824226]; HBG[HBG502486]; K[K000199041]; M [M0107937]; P[P00362599, P00362601]; US[US00098850].

#### Description.

Shrub to tree, 3–8 m tall, d.b.h. 1.5–5 cm; stilt roots or buttresses absent. Indumentum of simple hairs; old leafless branches glabrous, young foliate branches glabrous to pubescent. Leaves: petiole 2–3 mm long, 1–3 mm in diameter, pubescent, slightly grooved, blade inserted on the side of the petiole; blade 17.2–29 cm long, 5.7–11 cm wide, elliptic to obovate, apex acuminate, acumen 0.3–2.3 cm long, base acute, coriaceous, below glabrous when young and old, above glabrous when young and old; midrib sunken or flat, above glabrous when young and old, below glabrous when young and old; secondary veins 8 to 15 pairs per side, glabrous above; tertiary venation reticulate. Individuals unisexual, monoecious; inflorescences cauliflorous. Flowers with 9 perianth parts in 2 whorls, **globose in bud**, 1 to 5 per inflorescence, male and female inflorescences dimorphic; male pedicels 25–130 mm, 1–2 mm in diameter, sparsely pubescent to glabrous; bracts 2, one basal and one upper towards the lower half of pedicel, both bracts 1.5 mm long, ca. 2 mm wide; **female pedicels 80–320 mm** long, 2–4 mm in diameter; in fruit mm long, mm in diameter; bracts 2, one basal and one upper towards the lower half of pedicel, both bracts 2.5–6 mm long, 5–6 mm wide; male sepals 2, valvate, basally fused, 7 mm long, 7 mm wide, very broadly ovate, apex acute, base truncate, brown, pubescent outside, glabrous inside, margins flat; female sepals 2, valvate, basally fused, 7–17 mm long, 13–15 mm wide, very broadly ovate, apex acute, base truncate, fleshy, brown, pubescent outside, glabrous inside, margins flat; male petals 4, **basally fused**, tube ca. 3 mm long, 10–15 mm long, 8–10 mm wide, broadly ovate to broadly elliptic, apex acute, base truncate, wine-brown outside, white cream inside, margins flat, shortly tomentose outside, glabrous inside; female petals 4, **basally fused**, tube 2.5–3 mm long, 14–16 mm long, 8–12 mm wide, broadly ovate, apex acute, base truncate, fleshy, wine-brown outside, white cream inside, margins flat, pubescent outside, glabrous inside; male flowers: stamens 150 to 250, in 10 to 12 rows, ca. 1 mm long, oblong; connective reduced or absent, glabrous, cream; staminodes absent; female flowers: carpels free, 50 to 140, ovary 2–4 mm long, stigma globose, glabrous. Monocarps sessile, 4 to 9, 10–17 mm long, 7–11 mm in diameter, cylindrical, apex rounded, sparsely pubescent, verrucose, longitudinally 1 ribbed, brown when ripe; seeds 6 to 10 per monocarp, 7–10 mm long, 5–7 mm in diameter; aril absent.

#### Distribution.

A central African species from Cameroon, Equatorial Guinea Gabon and Democratic Republic of the Congo; in Cameroon known from South, South-West and Littoral regions.

#### Habitat.

In primary or secondary lowland rain forests. Altitude: 200–1100 m a.s.l.

#### IUCN conservation status.

assessed under the name ﻿*U.vanderystii*, Least Concern (LC) (Cheek 2014e).

#### Uses in Cameroon.

None reported.

#### Notes.

﻿﻿﻿*Uvariopsispedunculosa* is easily distinguished in flower by the combination of globose flower buds and basally fused petals. Le Thomas (1969b) had not seen any fruiting material of the species (as ﻿*U.vanderystii*), but it has since become available.

﻿﻿﻿*Uvariopsispedunculosa* was placed in synonymy with ﻿*U.dioica* in the second edition of the Flora of West Tropical Africa (Keay 1952). However, after careful examination of the type materials of both names, we conclude that ﻿*U.pedunculosa* is in fact conspecific with ﻿*U.vanderystii*. Indeed, the type of ﻿*U.pedunculosa* (*Zenker 3868*) has long flowering pedicels (up to 130 mm) and clearly globose flowers (as seen in the BR specimen [BR0000008824226]), while ﻿*U.dioica* has pedicels up to 50 mm long and ovoid-conical flower buds (Robyns and Ghesquière 1933; Kenfack et al. 2003). Given the priority rule, the name ﻿*U.pedunculosa* (1915, as ﻿*Tetrastemmapedunculosum* Diels) should be retained over ﻿*U.vanderystii* (1933).

#### Specimens examined.

**Littoral Region**: Mapubi 30 km before Edea on Yaoundé-Edea road On forestry road 5 km direction to Sanaga river, 3.84°N, 10.38°E, *28 February 2018*, *Couvreur T.L.P.* 1173 (MPU,P,WAG,YA). **South Region**: Elephant Mont, 2.79°N, 10.02°E, *16 October 2001*, *Tchouto Mbatchou G.P.* ELEX_15 (WAG); Campo-Ma’an area Onoyong, 2.52°N, 10.69°E, *18 March 2001*, *Tchouto Mbatchou G.P.* ONOX_182 (WAG); Campo-Ma’an area Onoyong, 2.52°N, 10.69°E, *18 March 2001*, *Tchouto Mbatchou G.P.* ONOX_274 (WAG); Campo-Ma’an area Mamelles Massif, 2.56°N, 9.949°E, *23 April 2001*, *Tchouto Mbatchou G.P.* 3242 (KRIBI,WAG); Bipindi, 3.08°N, 10.41°E, *01 January 1909*, *Zenker G.A.* 3868 (B,BM,BR,K,L,P). **South-West Region**: on forest trail north of Ngomboku village, 4.91°N, 9.724°E, *06 April 2016*, *Couvreur T.L.P.* 1063 (WAG,YA); on forest trail north of Ngomboku village, 4.91°N, 9.730°E, *06 April 2016*, *Couvreur T.L.P.* 1066 (WAG,YA); on trail through palm oil plantation 3 km before lava flow and Seme Beach hotel when coming from Limbe, 4.07°N, 9.085°E, *18 October 2013*, *Couvreur T.L.P.* 517 (WAG,YA); Ajang saprophyte plot, 4.76°N, 9.683°E, *01 December 1999*, *Gosline W.G.* 244 (K,MO,P,WAG,YA); Crète du Nta Ali (1266 m) entre côtes 1009 et 1202 30 km SE Mamfe, 5.59°N, 9.502°E, *19 June 1975*, *Letouzey R.* 13849 (P,YA); Likomba-Pflanzung 15–35 km NE von Victoria [Limbe], 4.1°N, 9.333°E, *03 December 1928*, *Mildbraed G.W.J.* 10745 (B,K); Environs de Matene RF Takamanda 32 km SW Akwaya, 6.25°N, 9.372°E, *30 April 1987*, *Satabié B.* 873 (YA); Forest on the southern slope of Mount above Batoke, 4.08°N, 9.1°E, *29 December 1983*, *Thomas D.W.* 2756 (MO,YA); Takamanda Forest Reserve, 6.21°N, 9.433°E, *30 April 1987*, *Thomas D.W.* 7364 (P); Massif du Ntali crête sommitale 30 km SE Mamfe, 5.58°N, 9.510°E, *14 June 1982*, *Villiers J.-F.* 1427 (P,YA).

### ﻿﻿﻿ Uvariopsis sessiliflora

Taxon classificationPlantaeMagnolialesAnnonaceae

(Mildbr. & Diels) Robyns & Ghesq., Ann. Soc. Sci. Bruxelles, Ser. B 53: 322, 1933

9340A3EB-9D79-564F-8BE3-38F1D654B93F

Map 16G

≡ Tetrastemma sessiliflorum Mildbr. & Diels, Bot. Jahrb. Syst. 53. 440, 1915.

#### Type.

Cameroon. East Region; Bezirk Lomié, im grossen Dscha-Bogen, *Mildbraed G.W.J. 5239*, 1911: holotype: B[B 10 015312]; isotype: HBG[HBG502485].

#### Description.

Tree, height unknown, d.b.h. unknown; stilt roots or buttresses not observed. Indumentum of simple hairs; old leafless branches glabrous, young foliate branches densely pubescent. Leaves: petiole 2–3 mm long, 1–2 mm in diameter, glabrous, slightly grooved, blade inserted on top of the petiole; **blade 12–18 cm long, 4–6 cm wide**, elliptic to obovate, apex acute, acumen 0.5–0.8 cm long, base acute to obtuse, papyraceous, below densely pubescent when young, densely pubescent when old, above glabrous when young and old; midrib sunken or flat, above densely pubescent when young, glabrous when old, below densely pubescent when young, glabrous when old; secondary veins 10 to 11 pairs per side, glabrous above; tertiary venation reticulate. Individuals unisexual [?, only female flowers known], monoecious [?]; inflorescences cauliflorous. Flowers with 6 perianth parts in 2 whorls, **globose in bud**, 2 to 3 per inflorescence, **female pedicels 1–3 mm long**, **1–2 mm in diameter**, pubescent; in fruit unknown, bracts 2 to 3, all basal or towards the lower half of pedicel, ca. 1 mm long, ca. 1 mm wide; female sepals 2, valvate, free, 2–4 mm long, 2–4 mm wide, ovate, apex acute, base truncate, pubescent outside, glabrous inside, margins flat; female petals 4, free, 7–16 mm long, 6–8 mm wide, ovate, apex acute, base truncate, margins flat, pubescent outside, sparsely pubescent and sericeous towards margins inside; male flowers unknown; staminodes unknown; female flowers: carpels free, number unknown, ovary 2–3 mm long, stigma unknown,. **Monocarps stipitate, 1 seen, ca. 18 mm long**, ca. 13 mm in diameter, globose, apex rounded, glabrous, smooth, color unknown; seeds 3 to 4 per monocarp, ca. 8 mm long, ca. 6 mm in diameter, ellipsoid; aril absent.

#### Distribution.

endemic to Cameroon, known from the South region.

#### Habitat.

Only known from the type specimen; in primary lowland rain forests. Altitude: ca. 600 m a.s.l.

#### Local and common names known in Cameroon.

None recorded.

#### IUCN conservation status.

Not evaluated (but probably Critically Endangered).

#### Uses in Cameroon.

None reported.

#### Notes.

﻿﻿*Uvariopsissessiliflora* is a poorly known species, but is distinguished by its subsessile flowers (pedicel shorter than 3 mm) and globose flower buds. Subsessile flowers are also found in ﻿*U.citrata* (Couvreur and Niangadouma 2016), but this latter species has longer leaves (31–50 vs 12–18 cm) with a characteristic lemon scent (not reported by Mildbraed for ﻿*U.sessiliflora*) and conical flower buds (versus globose)

The latest phylogenetic studies of *Uvariopsis* suggest that *U.sessiliflora* is conspecific with *U.dioica*, and the former name could be synonymized with the later (Dagallier et al. in prep).

### ﻿﻿﻿﻿ Uvariopsis solheidii

Taxon classificationPlantaeMagnolialesAnnonaceae

(De Wild.) Robyns & Ghesq., Ann. Soc. Sci. Bruxelles, Ser. B 53: 321, 1933

11056C98-0D30-5D76-9E05-B481C75B41B6

Figs 135 , 137 ; Map 16H

≡ Tetrastemma solheidii De Wild., Ann. Mus. Congo Belge, Bot. sér. 5, 3(1): 85, 1909.
= Uvariopsis batesii Robyns & Ghesq., Ann. Soc. Sci. Bruxelles, Ser. B 53: 320 1933. Type. Cameroon. South Region, Bitye, Bates G.L. 1367, 1919: holotype: BM[BM000554077].

#### Type.

Type. Democratic Republic of the Congo. Orientale; Yambuya, *Solheid A.F. 96*, 1906: holotype: BR[BR0000008824240).

#### Description.

Shrub to tree, 3–8 m tall, d.b.h. 1.5–8 cm; stilt roots or buttresses absent. Indumentum of simple hairs; old leafless branches glabrous to pubescent, **young foliate branches tomentose to pubescent**. Leaves: petiole 3–4 mm long, 1–2 mm in diameter, **tomentose**, slightly grooved, blade inserted on top of the petiole; blade 16.6–29 cm long, 5–9.5 cm wide, elliptic to obovate, apex acuminate to attenuate, acumen 0.4–2 cm long, base rounded to subcordate, papyraceous, below glabrous when young, sparsely pubescent when old, above glabrous when young and old; midrib sunken or flat, above glabrous when young and old, below sparsely pubescent when young, glabrous when old; secondary veins 8 to 13 pairs per side, glabrous above; tertiary venation reticulate. Individuals unisexual, monoecious; inflorescences cauliflorous. Flowers with 6 perianth parts in 2 whorls, **long conical in bud**, 2 to 3 per inflorescence, male and female inflorescences dimorphic; male pedicels up to 22 mm long, 0.5–1 mm in diameter, densely pubescent; female pedicels 80–160 mm long, 1–2 mm in diameter, densely pubescent; in fruit 53–165 mm long, ca. 2 mm in diameter; bracts 2, one basal and one upper towards the lower half of pedicel, basal bract ca. 1 mm long, ca. 1 mm wide; upper bracts 1–2 mm long, 1.5–3 mm wide, soon falling; male sepals 2, valvate, free, ca. 2 mm long, 2–3 mm wide, ovate, apex acute, base truncate, brown-red, pubescent outside, glabrous inside, margins flat; female sepals 2, valvate, free, 2.5–4 mm long, 3–5 mm wide, ovate to elliptic, apex acute, base truncate, brown-red, pubescent outside, glabrous inside, margins flat; male petals 4 (sometimes 5), free, 7–10 mm long, 2.5–4 mm wide, **narrowly ovate to narrowly elliptic**, apex acute, base truncate, wine-brown, margins flat, pubescent outside, glabrous and finely warty inside; female petals 4, free, 12–17 mm long, 4–7 mm wide, **narrowly ovate to narrowly elliptic**, apex acute, base truncate, wine-brown, margins flat, tomentose outside, glabrous and finely warty inside; male flowers: stamens 150 to 200, in 9 to 15 rows, ca. 0.5 mm long, oblong; connective reduced or absent, glabrous, cream; staminodes absent; female flowers: carpels free, 40 to 65, ovary 1.5–3.5 mm long, stigma coiled, densely pubescent. Monocarps stipitate, stipes 2–4 mm long, ca. 3 mm in diameter; monocarps 5 to 10, 30–70 mm long, 13–30 mm in diameter, ellipsoid to cylindrical, apex rounded to apiculate, sparsely pubescent to glabrous, longitudinally 4 to 6 ribbed, smooth, red when ripe; seeds 2 to 10 per monocarp, ca. 15 mm long, 8–13 mm in diameter, ellipsoid; aril absent.

#### Distribution.

A central African species, from Cameroon to Gabon and Democratic Republic of the Congo; in Cameroon known from the South and Central regions.

#### Habitat.

A fairly uncommon species in Cameroon, in lowland or more rarely submontane, growing in primary or old secondary rain forests. Altitude: 0–1200 m a.s.l.

#### Local and common names known in Cameroon.

None recorded.

#### IUCN conservation status.

Least Concern (LC) (Botanic Gardens Conservation International and IUCN SSC Global Tree Specialist Group 2019e).

#### Uses in Cameroon.

None reported.

#### Notes.

﻿﻿﻿*Uvariopsissolheidii* is distinguished by its young foliate branches being tomentose to pubescent and by its long conical flower buds and narrowly ovate to narrowly elliptic free petals. In the shape of its petals it may resemble ﻿*U.bakeriana*, which has even narrower linear petals that are deep red and very verrucose, and ﻿*U.etugeana*, which has much shorter pedicels.

#### Specimens examined.

**Central Region**: Ndanan 1, 3.62°N, 11.58°E, *10 March 2004*, *Cheek M.* 11606 (K,WAG,YA); Mont Ngoro à 38 km au Nord de Bafia (pied mont), 5.09°N, 11.26°E, *29 April 1978*, *Ngameni B.K.* 109 (P,YA); Mont Ngoro à 58 km SW de Linté, 5.09°N, 11.26°E, *17 April 1982*, *Nkongmeneck B.A.* 273 (P,YA); River N Didoumou, 3.61°N, 11.56°E, *26 March 2004*, *Onana J.M.* 2848 (K). **South Region**: Zingui, 2.85°N, 10.98°E, *21 July 1975*, *de Wilde J.J.F.E* 8373 (WAG); Ngongonjie (Akonetye), 2.67°N, 12.87°E, *30 August 1978*, *Koufani A.* 154 (P); Efoulan, 2.74°N, 10.54°E, *06 December 2000*, *Tchouto Mbatchou G.P.* ONOX_191 (WAG); Efoulan, 2.74°N, 10.53°E, *25 April 2000*, *Tchouto Mbatchou G.P.* 2843 (KRIBI,WAG); Efoulan, 2.74°N, 10.54°E, *04 December 2000*, *Tchouto Mbatchou G.P.* 3089 (KRIBI,WAG,YA); Campo-Ma’an area Akom II, 2.8°N, 10.53°E, *19 August 2001*, *van Andel T.R.* 3951 (MO); Colline de Nkoltsia à 23 km NW de Bipindi, 3.17°N, 10.27°E, *27 April 1974*, *Villiers J.-F.* 893 (P,YA).

### ﻿﻿﻿ Uvariopsis submontana

Taxon classificationPlantaeMagnolialesAnnonaceae

Kenfack, Gosline & Gereau, Novon 13(4): 444, 2003

787DBCA0-E019-554D-8BB5-8FE11B9091C8

**Figure 138.**
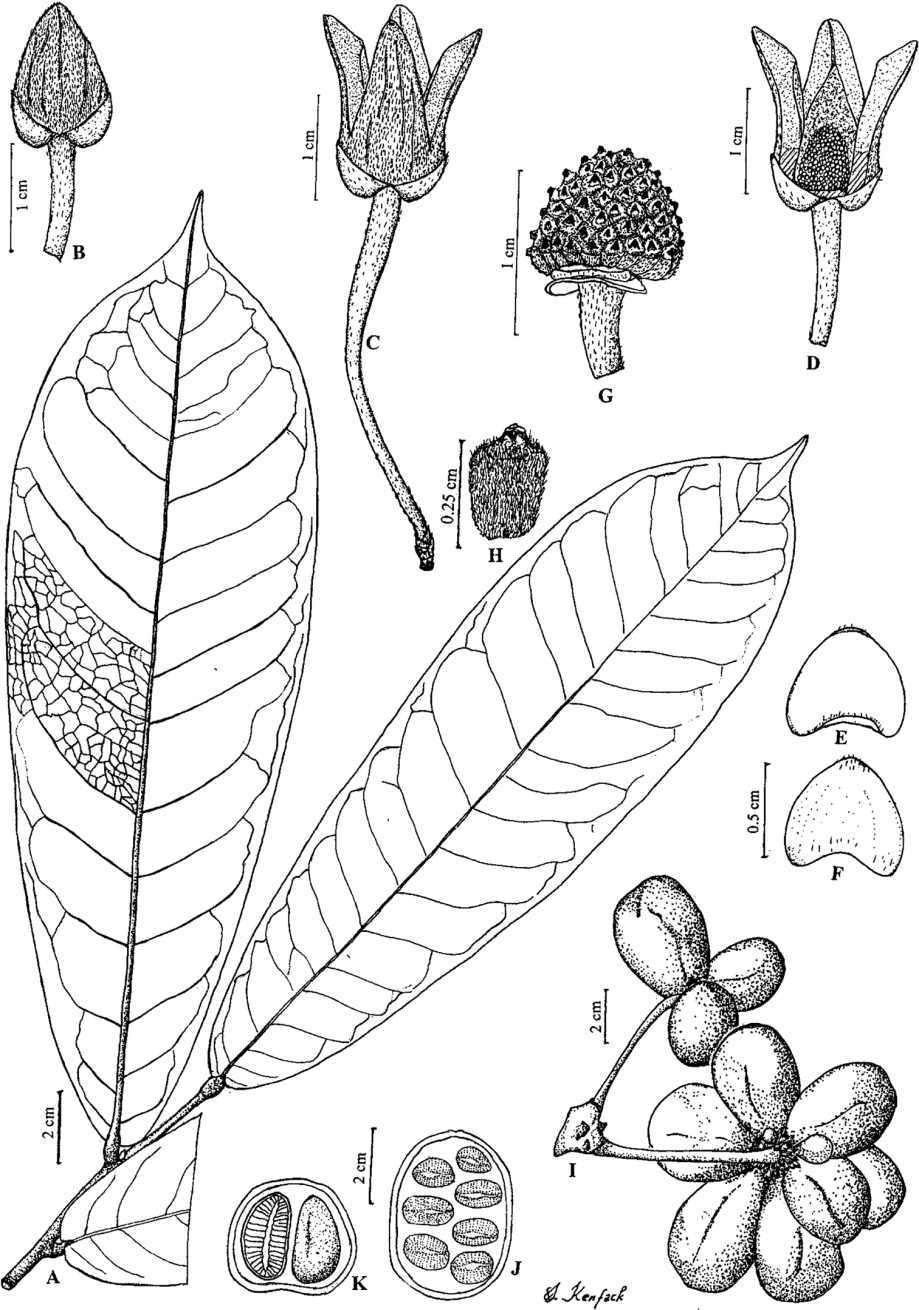
*Uvariopsissubmontana***A** branch with leaves **B** female flower in bud **C** male flower and male flower bud **D** male flower, one petal removed **E** inner surface of sepal **F** outer surface of sepal **G** female receptacle, all petals removed **H** detail of a single carpel, lateral view **I** fruit **J** longitudinal section of a single monocarp **K** transverse section of a single monocarp **A–H** from *Kenfack 1334***I–K** from *Kenfack 1373*. Drawings David Kenfack. Reproduced with permission from Kenfack et al. (2003, page 445).

**Figure 139.**
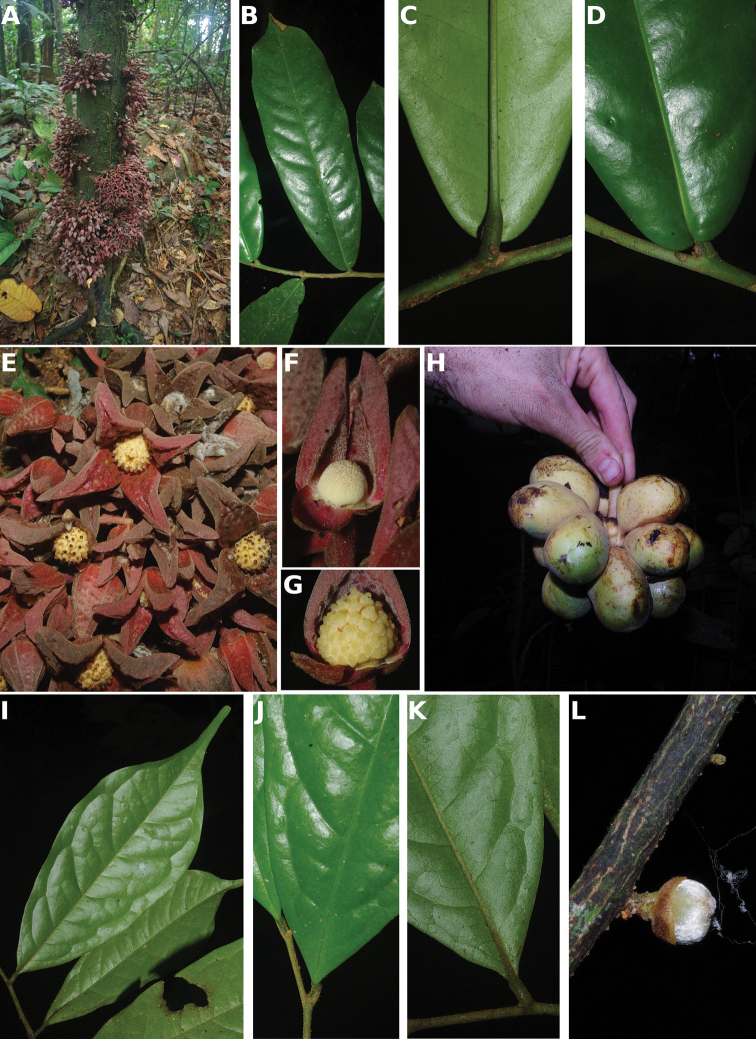
*Uvariopsissubmontana***A** base of trunk, note base completely covered with large clusters of flowers **B** leaf, upper side **C** base of leaf blade, lower side **D** base of leaf blade, upper side **E** cluster of female flowers, note light yellow carpels **F** detail of male flower, one petal removed **G** detail of female flower, one petal removed **H** fruit. *Uvariopsiszenkeri***I** leaf, upper side **J** base of leaf blade, upper side **K** base of leaf blade, lower side **L** flower in bud, note small dimension **A–G***Couvreur 627*, Ebo, Cameroon **H***Couvreur 1052*, Mt Cameroon, Cameroon **I–L***Couvreur 1027*, Mt Cameroon, Cameroon. Photos Thomas L.P. Couvreur.

Figs 138 , 139 ; Map 16I

#### Type.

Cameroon. South-West Region; Rumpi Hills, *Kenfack D. 1334*, 6 Feb 2000: holotype: YA; isotypes: K[000683145]; MO; SCA.

#### Description.

Tree, 8–25 m tall, d.b.h. up to 30 cm; stilt roots or buttresses absent. Indumentum of simple hairs; old leafless branches glabrous, young foliate branches glabrous to pubescent. Leaves: petiole 3–8 mm long, 3–5 mm in diameter, glabrous to sparsely pubescent, grooved, blade inserted on top of the petiole; blade 16–38 cm long, 5–11 cm wide, oblong to obovate, apex attenuate to acuminate, acumen 2–3 cm long, base rounded to subcordate, papyraceous to subcoriaceous, below glabrous when young and old, above glabrous when young and old; midrib sunken or flat, above glabrous when young and old, below sparsely pubescent when young, glabrous when old; secondary veins 9 to 18 pairs per side, glabrous above; tertiary venation reticulate. Individuals unisexual, monoecious; **inflorescences cauliflorous, with hundreds (up to 500) of flowers packed at the base and then more sparsely distributed up to 6 m**. Flowers unisexual, monoecious, with 6 perianth parts in 2 whorls, **conical to pyramidal in bud**, 6 to 50 per inflorescence, male and female inflorescences dimorphic; male pedicel: 25–50 mm, ca. 1 mm in diameter, pubescent; female pedicel: 30–60 mm long, ca. 1 mm in diameter, pubescent; in fruit 25–90 mm long, 3–7 mm in diameter, glabrous; bracts 2 to 4, all basal, ca. 1 mm long, ca. 1 mm wide; male sepals 2, connate, splitting at maturity, 5–10 mm long, 6–12 mm wide, triangular, apex acute, base truncate, brown-red, pubescent outside, glabrous inside, margins flat; female sepals 2, connate, splitting at maturity, 6–8 mm long, 6–9 mm wide, broadly ovate, apex acute, base truncate, brown-red, pubescent outside, glabrous inside, margins flat; male petals, 4, **basally fused**, tube 4–9 mm long, 8–18 mm long, 5–8 mm wide, elliptic to narrowly elliptic, apex acute, base truncate, wine red, margins flat, tomentose outside, pubescent inside; female petals, 4, **basally fused**, tube 3–4 mm long, 15–17 mm long, 5–7 mm wide, elliptic to narrowly elliptic, apex acute, base truncate, wine red, margins flat, tomentose outside, pubescent inside; male flowers: stamens 700 to 1000, in 25 to 30 rows, 0.5–1 mm long, oblong; connective reduced or absent, glabrous, cream; staminodes absent; female flowers: carpels free, 60 to 100, ovary 15–24 mm long, stigma globose, pubescent. Monocarps shortly stipitate, stipes ca. 1 mm long, ca. 1 mm in diameter; monocarps 9 to 25, 17–80 mm long, 13–55 mm in diameter, ovoid to oblong, apex apiculate, sparsely pubescent to glabrous, smooth, slightly constricted over seeds in dried material, pale green turning dark yellow at maturity; seeds 6 to 12 per monocarp, 18–25 mm long, 8–13 mm in diameter, ellipsoid; aril absent.

#### Distribution.

endemic to Cameroon, known from the South, Littoral and South West regions.

#### Habitat.

A species restricted to submontane primary or old secondary rain forests. Altitude: (600) 900–1300 m a.s.l.

#### Local and common names known in Cameroon.

None recorded.

#### IUCN conservation status.

Endangered (EN) (Cheek 2014d).

#### Uses in Cameroon.

None reported.

#### Notes.

﻿﻿*Uvariopsissubmontana* is distinguished by the combination of conical flower buds and basally fused 4 petals, and the trunk base completely covered with hunders of flowers. It is very close to ﻿*U.korupensis*, but differs by its smaller leaves, longer sepals, and submontane habitat (﻿*U.korupensis* being found in the lowlands, generally below 700 m).

#### Specimens examined.

**Littoral Region**: Ebo Wildlife Reserve Djuma permanent camp On east trail, 4.36°N, 10.25°E, *15 February 2013*, *Couvreur T.L.P.* 627 (WAG,YA); Ebo Bekob abandoned village CRES research station, 4.37°N, 10.41°E, *22 April 2005*, *Etuge M.* 6482 (K); Nkam Yingui Bataba, 4.53°N, 10.24°E, *20 February 2002*, *Kenfack D.* 1602 (MO). **South Region**: Campo Ma’an National Park 11 km on trail from Ebinanemeyong village on road 7 km from Nyabessan to Campo town, 2.49°N, 10.34°E, *12 February 2015*, *Couvreur T.L.P.* 682 (WAG,YA). **South-West Region**: Nyasoso, 4.81°N, 9.7°E, *08 February 1995*, *Cable S.* 1221 (K,MO,WAG,YA); Kupe village, 4.78°N, 9.716°E, *30 May 1996*, *Cable S.* 2736 (K,WAG,YA); Esense river near farm of Philip Taza, 4.76°N, 9.683°E, *19 January 1995*, *Cheek M.* 7034 (K,WAG,YA); Kupe village, 4.76°N, 9.683°E, *24 January 1995*, *Cheek M.* 7131 (K,WAG); Nyasoso village on max’s trail to Mt 4.82°N, 9.692°E, *05 April 2016*, *Couvreur T.L.P.* 1059 (WAG,YA); Rumpi mountains forest trail ca 5 km after Dikome Balue village ca 40 km north of Kumba, 4.93°N, 9.241°E, *10 January 2016*, *Couvreur T.L.P.* 965 (WAG,YA); Below Kupe rock near Esense river, 4.78°N, 9.683°E, *23 January 1995*, *Elad M.* 69 (K,YA); South of Nyasoso(end of village) Trail at the end of the village left hand side Nyasoso-Mbulle road, 4.81°N, 9.683°E, *03 July 1996*, *Etuge M.* 2562 (K,YA); Rumpi Hills, 4.95°N, 9.033°E, *06 February 2000*, *Kenfack D.* 1334 (K,MO,YA); Forest trail 2 km south from Etube-Tape village, 4.85°N, 9.7°E, *01 February 1995*, *Lane P.* 490 (K,WAG,YA); Nyasoso, 4.82°N, 9.666°E, *23 October 1995*, *Sebsebe D.* 5035 (K,YA); 7 km WNW of Bomana 34 km NW of Limbé, 4.27°N, 9.112°E, *15 December 1984*, *Villiers J.-F.* 2490 (P,YA).

### ﻿﻿﻿ Uvariopsis zenkeri

Taxon classificationPlantaeMagnolialesAnnonaceae

Engl., Notizbl. Königl. Bot. Gart. Berlin 2: 298, 1899

8E037489-B057-562C-91D6-945DBF12CA4D

**Map 17.**
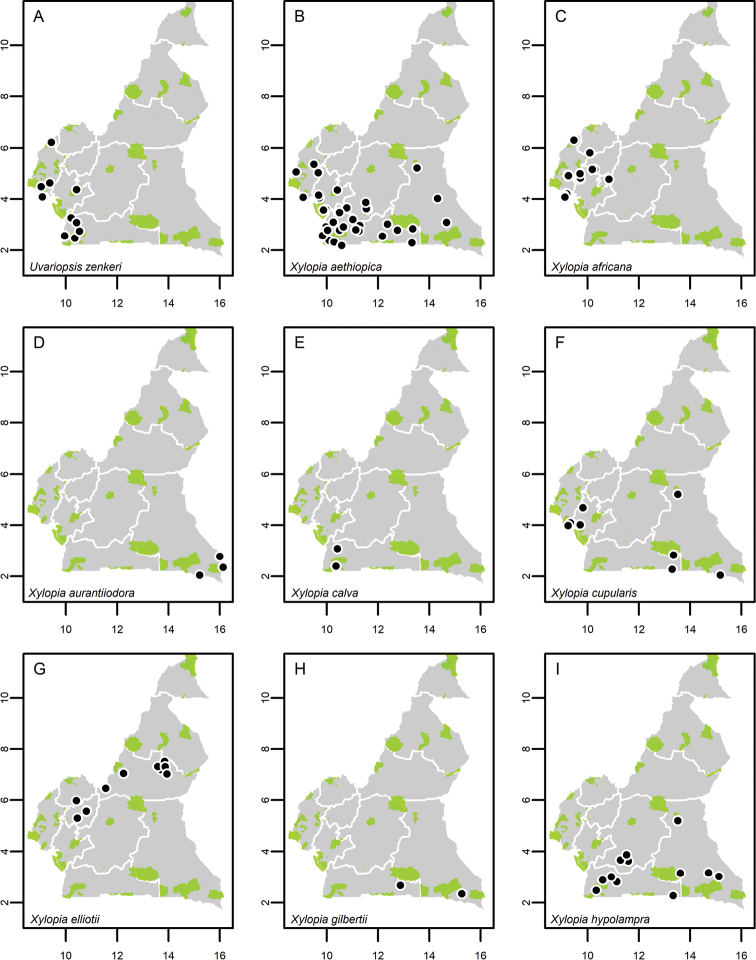
**A***Uvariopsiszenkeri***B***Xylopiaaethiopica***C***Xylopiaafricana***D***Xylopiaaurantiiodora***E***Xylopiacalva***F***Xylopiacupularis***G***Xylopiaelliotii***H***Xylopiagilbertii***I***Xylopiahypolampra*. White borders represent region limits in Cameroon; green patches represent protected areas (see methods and Suppl. material 1: Fig. S1).

**Figure 140.**
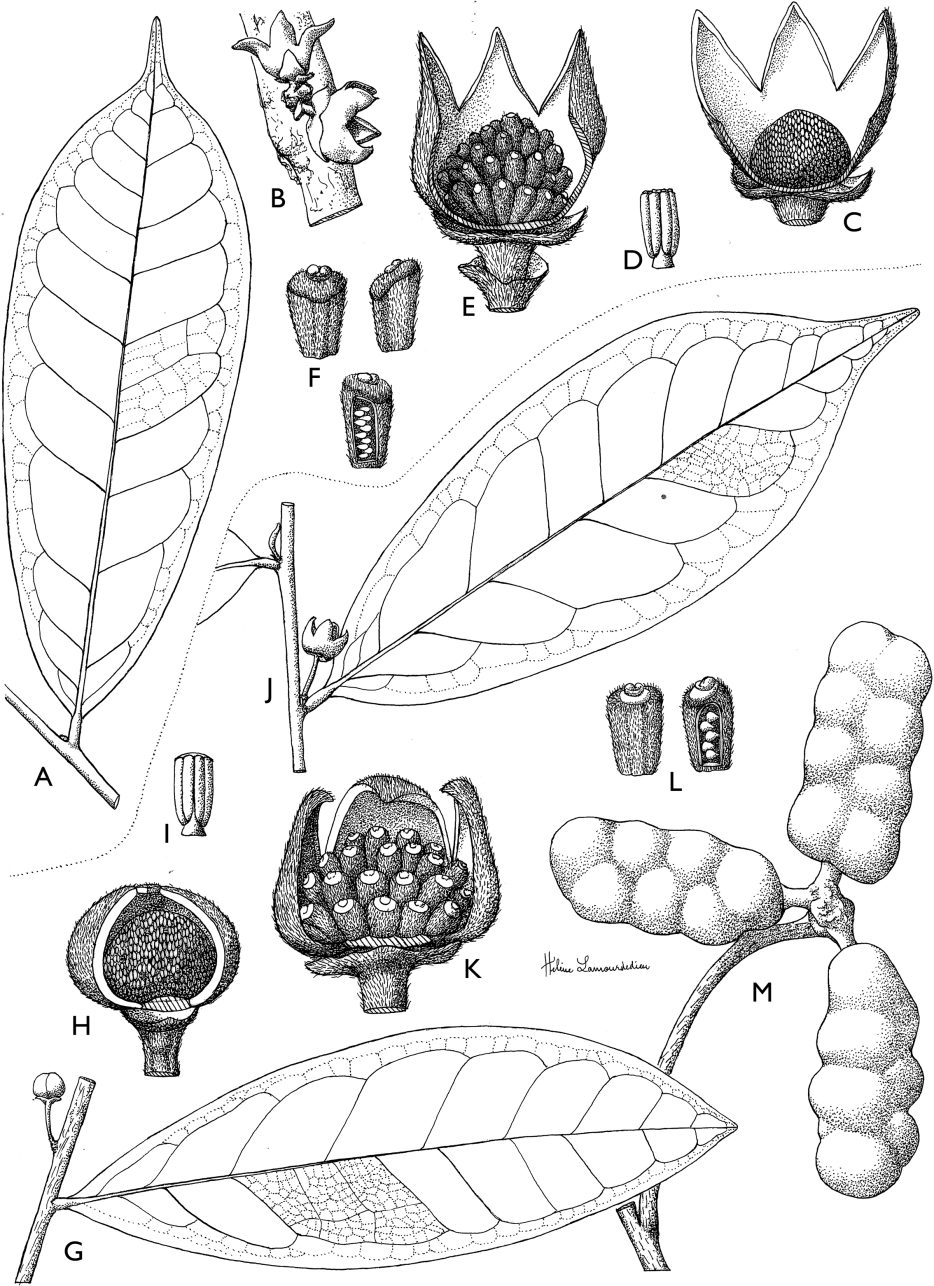
*Uvariopsiszenkeri***A** leaf, upper side **B** male inflorescences **C** male flower, one petal removed **D** stamen **E** female flower, one petal removed **F** carpel, front and side view, detail of ovules. *Uvariopsiscongensis***G** flowering branch with male flower **H** male flower one petal removed **I** stamen **J** flowering branch with female flower **K** female flower, one petal removed **L** carpel, side view and detail of ovules **M** fruit **A–E** from *Zenker 1117***F** from *Zenker 63***G–L** from *Tisserant 1363***M** from *Letouzey 5494*. Drawings by Hélène Lamourdedieu, Publications Scientifiques du Muséum national d’Histoire naturelle, Paris.

Figs 139 , 140 ; Map 17A

#### Type.

Cameroon. South Region; Bipindi, *Zenker G.A. 1117*, 1896: holotype: B[B 10 0153124]; isotypes: BM[BM000554075]; GOET[GOET005734]; HBG[HBG502515]; P[P00362604, P00362603]; K[K000199042]; M[M0107936]; S[S07-11029]; WU[WU0025790]

#### Description.

Shrub to tree, 2–7 m tall, d.b.h. 1.5–11 cm; stilt roots or buttresses absent. Indumentum of simple hairs; old leafless branches glabrous, **young foliate branches densely pubescent**. Leaves: petiole 2–5.5 mm long, 1–2 mm in diameter, **densely to sparsely pubescent**, grooved, blade inserted on top of the petiole; blade 11–15.8 cm long, 3.5–5.8 cm wide, elliptic to obovate, **apex long acuminate, acumen 1–2.4 cm long**, base acute to decurrent, papyraceous, above glabrous when young and old, below glabrous when young and old; midrib sunken or flat, above glabrous when young and old, below pubescent when young, glabrous when old; secondary veins 6 to 13 pairs, glabrous above and below; tertiary venation reticulate. Individuals unisexual, monoecious; inflorescences cauliflorous or ramiflorous on old leafless branches, axillary. Flowers with 6 perianth parts in 2 whorls, globose in bud, 1 per inflorescence, **male and female inflorescences similar; pedicel 0**–**7 mm long**, 1–1.5 mm in diameter, **densely pubescent**; in fruit ca. 10 mm long, 2–3 mm in diameter, glabrous; bracts 1 to 2, all basal 1–4 mm long, 2.5–5 mm wide; sepals 2, valvate, basally fused, 3–7 mm long, 4.5–7 mm wide, circular, apex obtuse, base truncate, brown, densely pubescent outside, glabrous inside, margins flat; petals 4, **basally fused**, tube 1–4 mm long, 6–13 mm long, 4–7 mm wide, triangular, apex acute, base truncate, greenish-white, margins flat, densely pubescent outside, glabrous inside; male flowers: stamens 100 to 150, in 10 to 12 rows, 0.5 mm long, broad; connective reduced or absent, glabrous, pinkish red; staminodes absent; female flowers: carpels free, 13 to 22, ovary 1.5–3 mm long, 1–1.5 wide, stigma coiled, densely pubescent. Monocarps stipitate, stipes ca. 1 mm long, ca. 2 mm in diameter; monocarps 1 to 3, ca. 37 mm long, ca. 17 mm in diameter, cylindrical, apex rounded, **tomentose, bumpy when dry**, green when ripe; seeds 10 to 12 per monocarp, ca. 13 mm long, ca. 7 mm in diameter, ellipsoid; aril absent.

#### Distribution.

endemic to Cameroon; known from the South, Littoral and South-West regions.

#### Habitat.

In lowland primary or old secondary rain forests. Altitude 0–700 m a.s.l.

#### Local and common names known in Cameroon.

None recorded.

#### IUCN conservation status.

Vulnerable (VU). (Texier and Stévart 2021b).

#### Uses in Cameroon.

None reported.

#### Notes.

﻿﻿*Uvariopsiszenkeri* closely resembles ﻿﻿*U.congensis*. See notes under the latter species.

#### Specimens examined.

**Littoral Region**: Ebo Forest proposed National Park Ebo Forest Research Station-Bekob, 4.36°N, 10.41°E, *27 October 2006*, *Osborne J.* 200 (K). **South Region**: Bipindi, 3.26°N, 10.20°E, *20 June 1918*, *Annet E.* 351 (P); Campo Ma’an National Park 11 km on trail from Ebinanemeyong village on road 7 km from Nyabessan to Campo town, 2.47°N, 10.34°E, *14 February 2015*, *Couvreur T.L.P.* 707 (WAG,YA); Efoulan, 2.74°N, 10.53°E, *25 April 2000*, *Tchouto Mbatchou G.P.* 2846 (KRIBI,WAG); Campo-Ma’an area Mamelles Massif, 2.56°N, 9.949°E, *23 April 2001*, *Tchouto Mbatchou G.P.* 3252 (KRIBI,WAG); Bipindi, 3.08°N, 10.42°E, *1896*, *Zenker G.A.* 1117 (K,L,P); Bipindi, 3.08°N, 10.42°E, *01 January 1904*, *Zenker G.A.* 3045 (K,L,P,WAG); Bipindi, 3.08°N, 10.42°E, *01 January 1904*, *Zenker G.A.* 3228 (L); Bipindi, 3.08°N, 10.41°E, *01 March 1914*, *Zenker G.A.* 515 (U,WAG). **South-West Region**: on trail leading to top of Mt Etinde after Ekonjo village, 4.06°N, 9.153°E, *01 April 2016*, *Couvreur T.L.P.* 1027 (WAG,YA); on top of hill near Small Ekombe village 3 km after Kumba on road to Ekondo Titi town, 4.62°N, 9.374°E, *13 January 2016*, *Couvreur T.L.P.* 978 (WAG,YA); Mokoko Forest Reserve Dikome, 4.48°N, 9.033°E, *05 May 1994*, *Ekema S.N.* 944 (K); Mount above Batoke, 4.08°N, 9.083°E, *24 April 1984*, *Thomas D.W.* 3455 (BR,LBV,MO,P,WAG,YA); Takamanda Forest Reserve, 6.21°N, 9.433°E, *30 April 1987*, *Thomas D.W.* 7372 (MO,P,WAG).

### ﻿﻿ Xylopia

Taxon classificationPlantaeMagnolialesAnnonaceae

L. , Systema Naturae ed. 10, 2: 1250, 1759

1D98F7A4-79B8-5FF3-B39B-3EEF1706C877

= Xylopicrum P. Browne; Hist. Jamaic. 250–251 + t. 5, fig. 2, 1756; Xylopicron, orth. mut., Adanson; Fam. 2: 365, 1763; Unona Linnaeus f., Suppl. pl. 270. Apr 1782; ﻿Bulliarda Necker, Elem. bot. 2: 321. 1790, nom. superfl., non Candolle, 1801; ﻿Krockeria Necker, Elem. bot. 2: 317–318. 1790 ; ﻿Coelocline A. de Candolle, Mém. Soc. Phys. Genève 5: 208–209. 1832; Habzelia Hook. f. & Thomson, Fl. Ind. 123. 1855, non A. DC.; ﻿Parartabotrys Miq., Fl. Ned. Ind., Eerste Bijv. 3: 374. 1861 [‘1860’]; ﻿Pseudanona (Baillon) Safford, J. Wash. Acad. Sci. 3: 17. 1913, as “﻿Pseudannona.”

#### Description.

Trees or shrubs, 2–50 m tall, d.b.h. up to 90 cm; stilt roots or buttresses absent or present. Indumentum of simple hairs. Leaves: petiole 1–12 mm long, 1–2 mm wide; blade 3.6–21.3 cm long, 1.2–8.4 cm wide, lanceolate, ovate, elliptic, obovate, oblong, or oblanceolate, apex acuminate or acute or obtuse or cuspidate, acumen 0.2–2.1 cm long, base rounded, cordate, cuneate, obtuse, or truncate; midrib sunken or flat above, rarely slightly raised; secondary veins 7 to 20 pairs; tertiary venation reticulate. Inflorescences axillary, plants ramiflorous on young foliate or older leafless branches, rarely cauliflorous, 1–32-flowered; pedicel 1–12 mm long; in fruit 1–30 mm long; bracts 1–6, basal or inserted along the pedicel. Flowers bisexual with 9 perianth parts in 3 whorls; sepals 3, valvate, free or basally fused, 1–7 mm long, apex acute or acuminate or rounded or apiculate, base truncate; petals free, outer petals longer than inner; outer petals 3, 5.8–64 mm long, 1.2–6 mm wide, linear, lanceolate, ligulate-lanceolate, or ovate, apex acute, rounded, or obtuse, base broad and concave; inner petals 3, valvate, 3.5–48 mm long, 1–5.4 mm wide, linear, ovate, oblong, rhombic, or lanceolate, apex acute, obtuse, or acuminate, base broad and concave,; stamens 40–300, 1–2 mm long, oblong or clavate; connective apex capitate, shieldlike, or conical, filaments connate at base to form a cone surrounding the carpels, or staminal cone absent; staminodes present, in one outer and one inner whorl, the inner whorl absent in ﻿*X.aurantiiodora*, ﻿*X.mildbraedii*, and ﻿*X.quintasii*; carpels free, 3 to 50, 1–3 mm long, stigma filiform, cylindrical, oblong, linear-falcate or ellipsoid. Monocarps dehiscent, stipitate, subsessile, or sessile; monocarps 1 to 36, 19–98 mm long, 6–40 mm wide, narrowly oblong to oblong, sometimes falciform, torulose, or moniliform, apex acute, rounded or mucronate; seeds 5–22 mm long, 3–17 mm wide, ellipsoid to oblong, somewhat flattened, seed coat with a fleshy outer layer (sarcotesta) and hard inner layer, or sarcotesta absent; aril absent or present.

#### Type species.

﻿*Xylopiamuricata*L. (a West Indian species).

A genus of nearly 200 species trees and shrubs, the genus with a pantropical distribution. In Cameroon there are 22 species, comprising over 13% of the Annonaceae flora for the country, but no species is endemic to the country. The dehiscent monocarps of ﻿*Xylopia* are unique among the Annonaceae genera found in Cameroon.

﻿﻿*Xylopiaflamignii* Boutique was reported for Cameroon by Onana (2013), but the voucher specimen (*Harris 3680B*) has been re-identified as ﻿*X.cupularis*. ﻿﻿*Xylopiaflamignii* reaches its northern limit in southeastern Gabon and central Republic of the Congo (Johnson and Murray 2018).

#### Taxonomy.

Johnson and Murray (2018).

### ﻿Key to the species of ﻿*Xylopia* in Cameroon:

(Note: the presence and color of the seed sarcotesta often cannot be discerned in dried material.)

**Table:** 

1	Twigs with longer hairs erect, 1–2 mm long	**2**
–	Twigs with longer hairs < 1 mm long, hairs appressed or rarely erect, or twigs glabrous	**6**
2	Upper bark red, rough, and scaly; leaves obtuse or rarely acute at the apex	﻿***X.pynaertii***
–	Upper bark gray or dark brown; leaves acuminate, the acumen 0.3–1.7 cm long	**3**
3	Leaf blades with sparse to dense tightly appressed gold hairs below	﻿***X.villosa***
–	Leaf blades with loosely appressed to erect rusty, brown, or gray hairs below, or glabrous	**4**
4	Pubescence of twigs dense, rust-colored	﻿***X.talbotii***
–	Pubescence of twigs sparse to moderate, dull gray to brown	**5**
5	Leaf blades with reticulate tertiary venation prominent below; tree of montane habitats	***X.monticola***
–	Leaf blades with reticulate tertiary venation indistinct below; shrub or tree of lowland habitats	﻿﻿***X.thomsonii***
6	Leaf blades appressed-pubescent below, the hairs overlapping and forming a visible indument; seeds in two rows	**7**
–	Leaf blades glabrate to pubescent below, hairs erect or not overlapping; seeds in one or two rows	**9**
7	Leaf blades densely covered with shiny silver hairs below; monocarps with abundant conspicuous lenticels	﻿***X.hypolampra***
–	Leaf blades with shiny gold or dull gray to brown hairs below; monocarps with lenticels small, indistinct, or absent	**8**
8	Leaf blades cuneate to broadly cuneate at base, acuminate at the apex, appressed -pubescent with golden hairs below	﻿***X.cupularis***
–	Leaf blades truncate at the base, acute at apex, appressed-pubescent with dull grayish brown hairs below	﻿***X.letestui***
9	Apex of anther connectives capitate to conical; stigmas separate, narrowly oblong to clavate; aril membranous, fimbriate, covering at least half and often the entire seed	**10**
–	Apex of anther connective shield-like, sometimeswith a slight bump in the center; stigmas more or less connivent, linear; aril covering only the base of seed, or aril absent	**11**
10	Leaf blades elliptic to oblong, rarely oblanceolate; inner petals with a distinct truncate tooth overhanging basal concavity; seeds 13–21 mm long	﻿***X.aurantiiodora***
–	Leaf blades oblanceolate to obovate; inner petals lacking tooth overhanging basal concavity; seeds 10–13 mm long	﻿***X.quintasii***
11	Plants with flowers	**12**
–	Plants with fruits	**24**
12	Outer petals ovate or elliptic, < 3 times as long as wide	**13**
–	Outer petals linear, narrowly elliptic, or lanceolate, > 3 times as long as wide	**15**
13	Twigs pubescent, hairs erect; leaf blades obtuse to acute at the apex	﻿***X.gilbertii***
–	Twigs glabrous or pubescent, hairs appressed; leaf blades acuminate at the apex, the acumen 2–10 mm long	**14**
14	Sepals 5–7 mm long, 5–6 mm wide; aril blood-red	﻿***X.africana***
–	Sepals 2–3 mm long, 3–4 mm wide; aril yellow to orange	﻿﻿***X.staudtii***
15	Inner petals much shorter than outer petals, rhombic, 3.5–6.7 mm long	﻿﻿***X.rubescens***
–	Inner petals subequal to outer petals, linear, > 9 mm long	**16**
16	Carpels 45–50	﻿***X.aethiopica***
–	Carpels < 13	**17**
17	Pedicels 1, rarely 2 per axil, bracts 3–6, usually imbricate and more or less persistent	**18**
–	Pedicels 1–12 per axil, bracts 2, rarely 3–4, not overlapping and with the upper persistent in flower and the lower caducous	**19**
18	Outer petals 45–79 mm long, 3.6–5.5 mm wide at base; inner petals with a tuft of long hairs at the top of concavity on inner base	﻿***X.mildbraedii***
–	Outer petals (14.6–) 22–49 mm long, 2.4–3.8 mm wide at base; inner petals with only uniform fine short hairs on inner base	﻿﻿***X.thomsonii***
19	Leaves glabrous or with a few scattered hairs below	﻿***X.katangensis***
–	Leaves appressed-pubescent below	**20**
20	Sepals reflexed at anthesis; petals lax and crinkled when dried; stigmas 3.8–7 mm long	﻿***X.longipetala***
–	Sepals erect to slightly spreading at anthesis; petals rigid and flat when dried; stigmas 1.3–3 mm long	**21**
21	Outer petals oblong-lanceolate, glabrous on inner surface except at the apex	﻿***X.calva***
–	Outer petals linear to linear-lanceolate uniformly pubescent on inner surface	**22**
22	Inflorescences with up to 32 flowers in a highly branched inflorescence; inner petals 9.7–13.1 mm long;	﻿***X.paniculata***
–	Inflorescences with 12 flowers, often fewer, unbranched or with few branches; inner petals 15–24 mm long	**23**
23	Young branches appressed-pubescent; inflorescences 1–10-flowered, commonly 2–3-flowered; leaves 5.7–17.2 cm long	﻿***X.phloiodora***
–	Young branches erect-pubescent; inflorescences 1(–2)-flowered; leaves 4.5–9.5 cm long	﻿***X.elliotii***
24	Seeds in two rows in the monocarp	**25**
–	Seeds in a single row in the monocarp	**29**
25	Monocarps with abundant conspicuous lenticels, seed sarcotesta orange to red	﻿***X.phloiodora***
–	Monocarps lacking conspicuous lenticels, seed sarcotesta white, pale blue, or green	**26**
26	Monocarps with strong longitudinal ridges when dried	﻿***X.longipetala***
–	Monocarps smooth when dried, or at most obliquely wrinkled	**27**
27	Leaf blades 10–17.2 cm long, 3.6–6.5 cm wide	﻿***X.calva***
–	Leaf blades 4.5–9.5 cm long, 1.8–5.6 cm wide	**28**
28	Leaf blades glabrous below, apex acuminate to acute	﻿***X.katangensis***
–	Leaf blades finely appressed-pubescent below, eventually glabrous, apexobtuse or rarely acute	﻿***X.elliotii***
29	Seeds arillate	**30**
–	Seeds lacking an aril, but with a thin sarcotesta covering the seed (may be indistinct in dried seeds)	**33**
30	Monocarps up to 36 per fruit; seeds 5–6 mm long; aril bilobed	﻿***X.aethiopica***
–	Monocarps up to 15 per fruit; seeds 10–20 mm long; aril brushlike	**31**
31	Monocarps smooth to weakly torulose	﻿﻿***X.staudtii***
–	Monocarps distinctly torulose to moniliform	**32**
32	Apical beak of monocarp 1–1.5 mm long; twigs brown; tree of montane forests	﻿***X.africana***
–	Apical beak of monocarp up to 5 mm long; twigs grayish white; tree of lowland swamp forests	﻿﻿***X.rubescens***
33	Sarcotesta orange to red; leaf blade at the apex obtuse, rarely acute, erect-pubescent below	﻿***X.gilbertii***
–	Sarcotesta white, pale green, gray, or blue; leaf blade at the apex acuminate, rarely acute, glabrous to sparsely appressed-pubescent below	**34**
34	Monocarps 31–40 mm wide, stipe 2–3 mm long; seeds 16–17 mm wide	﻿***X.paniculata***
–	Monocarps 6–15 mm wide, stipe 4–15 mm long; seeds < 10 mm wide	**35**
35	Seeds 13–14 mm long; twigs appressed-pubescent	﻿***X.mildbraedii***
–	Seeds 9–12 mm long; twigs glabrous or erect-pubescent	**36**
36	Monocarps 12–15 mm wide; twigs glabrous or with a few scattered hairs; tree up to 13 m tall	﻿***X.katangensis***
–	Monocarps 6–12 mm wide; twigs persistently erect-pubescent; scandent shrub, rarely a tree to 10 m tall	﻿﻿***X.thomsonii***

### ﻿﻿﻿ Xylopia aethiopica

Taxon classificationPlantaeMagnolialesAnnonaceae

(Dunal) A.Rich., Hist. phys. Cuba, Pl. vasc. 1: 53, 1841

AAFE1603-E0F7-552D-B187-3DEC74D169D1

**Figure 141.**
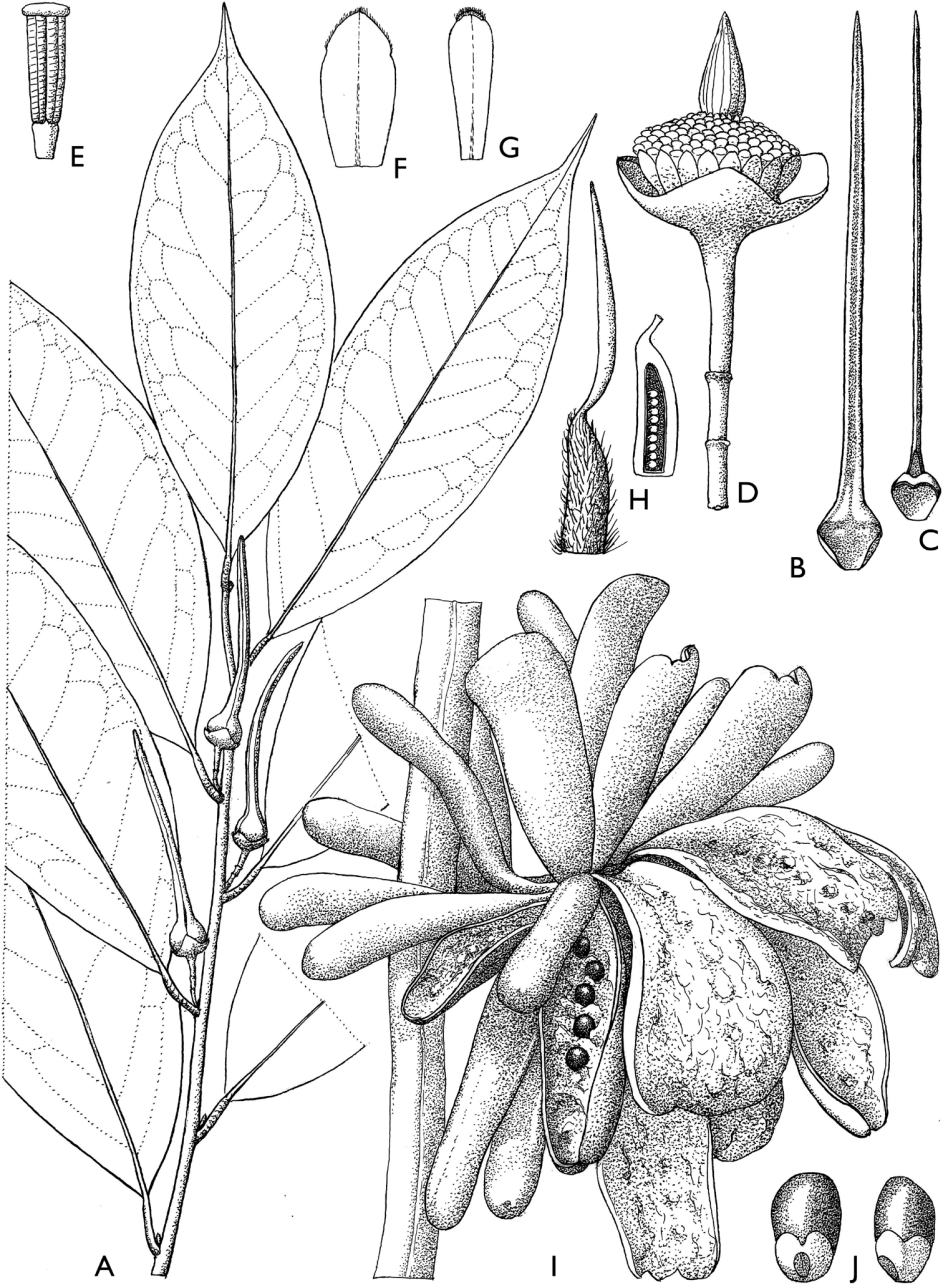
*Xylopiaaethiopica***A** flowering branch **B** outer petal, inner view **C** inner petal, inner view **D** receptacle, all petals removed **E** stamen, front view **F** staminode, outer whorl **G** staminode, inner whorl **H** carpel, side view and detail of ovules **I** fruit, with one monocarp opening via splitting **J** seed, front and side views **A–H** from *Le Testu 7960***I, J** from *Hallé &Le Thomas 581*. Drawings by Hélène Lamourdedieu, Publications Scientifiques du Muséum national d’Histoire naturelle, Paris; modified from Le Thomas (1969b; pl. 30, p. 167).

**Figure 142.**
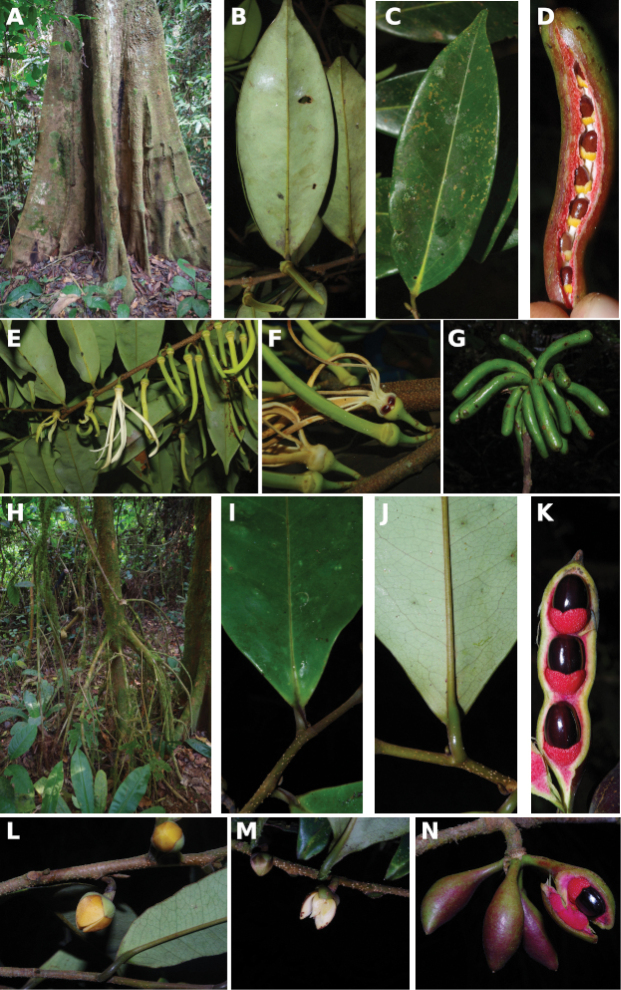
*Xylopiaaethiopica***A** base of trunk, note buttresses **B** leaf, lower side **C** leaf, upper side **D** monocarp longitudinally opening, note yellow aril **E** flowers, side view **F** flower with one outer petal removed, showing the base of inner petals **G** immature fruit. *Xylopiaafricana***H** base of trunk, note the presence of stilt roots **I** base of leaf blade, upper side **J** base of leaf blade, lower side **K** monocarp, longitudinally splitting, note red aril **L** flowers **M** flower, side view, one outer petal removed **N** fruit, side view, one monocarp opened revealing seed with red aril **A, D, G***Couvreur 1223*, Maséa, Cameroon **B***Couvreur 471*, Lélé, Cameroon **C, E, F***Couvreur 543*, Gabon **H–J, M***Couvreur 949***K***Couvreur 967*, Rumpi Mt, Cameroon **L–N***Couvreur 993*, Mt Cameroon, Cameroon. Photos Thomas L.P. Couvreur.

Figs 141 , 142 ; Map 17B

≡ Unona aethiopica Dunal, Monogr. Anonac.: 113, 1817; ﻿﻿Uvariaaethiopica (Dunal) A. Richard; Fl. Senegamb. Tent. 1: 9, 1831; ﻿Habzeliaaethiopica (Dunal) A. DC., Mém. Soc. Phys. Genève 5: 207. 1832, *nom. illeg.*; ﻿Xylopicrumaethiopicum (Dunal) Kuntze, Revis. gen. pl. 1: 8, 1891.
= Xylopia eminii Engl., Pflanzenw. Ost-Afrikas C: 179, 1895. Type. Uganda. Western Province, Bugo, Stuhlmann F.L. 1233: holotype: B[100153132].
= Xylopia dekeyzeriana De Wild., Ann. Mus. Congo Belge, Bot. ser. 5, 3[1]: 4, 1903. Type. Democratic Republic of the Congo. Kongo Central Province, Sanda, *Gillet J. 2258*, 1902: lectotype, designated by Johnson and Murray (2018), p. 76: BR[BR0000024941525]; isolectotypes: BR[BR0000008824257, BR0000008824264].
= Xylopia gilletii De Wild., Ann. Mus. Congo Belge, Bot. sér. 5, 1[1]: 42, 1903. Type. Democratic Republic of the Congo. Kongo Central, Inkisi-Kisantu[“Kisantu”], *Gillet J. 207*, 1899: holotype: BR[BR0000024941532].

#### Type.

Sierre Leone. without definite locality, *Smeathman H. s. n.*, no date: lectotype, designated by Verdcourt (1971a), p. 77: G, secondary lectotype, designated here: G-DC[00201441 on 2 sheets]; isolectotypes: BM[BM000510763, branch on right-hand side of sheet]; FI-W[005603].

#### Description.

Tree, up to 46 m tall, d.b.h. 30–58 cm; **plank buttresses present**. Old branches glabrous, young branches glabrous to sparsely pubescent, the hairs 0.1–0.4 mm long. Leaves: petiole 4–9 mm long, ca. 1 mm wide, glabrous to sparsely pubescent, slightly grooved, blade inserted on the side of the petiole; blade 7.3–16.3 cm long, 2.1–6.6 cm wide, lanceolate-ovate to elliptic, occasionally oblong, narrowly elliptic, oblanceolate, or ovate, apex acuminate, acumen 0.6–2.0 cm long, base cuneate to broadly cuneate, short-decurrent, sometimes asymmetrical, subcoriaceous, below glabrous to sparsely pubescent when young, glabrous when old, above glabrous when young and old, **discolorous, whitish below**; midrib sunken, above glabrous when young and old, below glabrous when young and old; secondary veins 9 to 13 pairs, glabrous above; tertiary venation reticulate. Individuals bisexual; inflorescences ramiflorous on young foliate or old leafless branches, axillary, peduncle 1.5–2.8 (–4) mm long. Flowers with 9 perianth parts in 3 whorls, 1 to 7 per inflorescence; pedicel 5–15 mm long, 1–2 mm in diameter, pubescent; in fruit 10–18 mm long, 2–6 mm in diameter, glabrous; bracts 1 to 2, towards the middle of pedicel, 2–3 mm long, 3 mm wide; sepals 3, valvate, **basally to 2/3 fused, forming a cup**, 2–4 mm long, 3–6 mm wide, triangular, apex acute to rounded, base truncate, greenish, glabrous to sparsely pubescent outside, glabrous inside; petals free, subequal; **outer petals 3, 28–64 mm long, 3.8–6 mm wide at base, linear**, apex obtuse, base broad and concave, yellow to light green or white, pubescent outside, pubescent inside; inner petals 3, valvate, 18.7–51 mm long, 3.8–6 mm wide at base, linear, apex acute, base broad and concave, cream to greenish yellow, pubescent, base glabrous outside, pubescent inside; stamens 140 to 300, in 5 to 6 rows, 1–2 mm long, oblong; connective apex shield-like, pubescent to glabrous, cream; carpels free, 45 to 50, ovary 1–2 mm long, stigmas connivent, linear, 3.5–4.7 mm long, pubescent to glabrous. Monocarps stipitate, stipe 2–8 mm long, 2–4 mm in diameter; monocarps 20 to 30(36), **32–82 mm long, 3–8 mm wide, narrowly oblong, weakly torulose, apex with a blunt beak 1.5–3.5 mm long**, , glabrous, verrucose and wrinkled when dried, green to red outside, endocarp red; seeds 4 to 12 per monocarp, **in a single row**, 5–6 mm long, 3–4 mm wide, ellipsoid; **sarcotesta absent; aril flat, bilobed, orange to pale yellow**.

#### Distribution.

A widespread species in West, Central and East Africa, from Senegal to Kenya and south to Angola and northern Mozambique; in Cameroon known from East, South, Central, Littoral, South-West, and West and regions.

#### Habitat.

A very common species (the most common species of ﻿*Xylopia*) with a broad ecological amplitude, in Cameroon in lowland primary, old secondary or disturbed rain forests; sometimes cultivated or planted. Altitude 0–1600 m a.s.l.

#### Local and common names known in Cameroon.

ngwo (Bibaya); akwi (Yaoundé); Poivre de Guinée (French).

#### IUCN conservation status.

Least Concern (LC) (Harvey-Brown 2019g).

#### Uses in Cameroon.

***Food***: fruits and seeds used as a flavoring in foods and beverages (Johnson and Murray 2018); ***medicine***: fruit against cough, aches, bronchitis, dysentery; ***construction***: bark for building materials (Tessmann 1913).

#### Notes.

This common species is readily distinguished from all other African ﻿*Xylopia* species by the combination of distinctly fused sepals, the large number of narrowly oblong, weakly torulose monocarps, and the bilobed aril of the relatively small seeds. The leaf blades are often pale beneath and with an asymmetrical base. Among Cameroon species of ﻿*Xylopia*, ﻿﻿*X.rubescens* has similar narrow and torulose monocarps, but the monocarps of that species are wider strongly torulose to moniliform, and have larger seeds. ﻿﻿*Xylopiaaethiopica* grows in a range of habitats, and it is difficult to determine from herbarium label data the extent to which its presence in secondary vegetation, and along riverbanks, is spontaneous or the result of deliberate planting and tending.

In Cameroon three species of monkeys eat and defecate the seeds (summarized in Johnson and Murray 2018).

#### Selected specimens examined.

**Central Region**: Ndanan 1 abandoned enclosure on river before bridge, 3.62°N, 11.58°E, *09 March 2004*, *Cheek M.* 11579 (K,YA); NE of Ndangan I on logging trail into Mefou NP, 3.62°N, 11.59°E, *23 March 2004*, *Cheek M.* 11999 (K,YA); East side of park, 3.60°N, 11.59°E, *16 October 2002*, *Gosline W.G.* 423 (K,YA); 60 km S of Edéa S of Mboké 11 km E of km 58 of road Edea-Kribi, 3.47°N, 10.5°E, *22 April 1965*, *Leeuwenberg A.J.M.* 5504 (BR,K,MO,P,WAG,YA). **East Region**: 64 km south of Yokadouma 30 km after Ngato 15 km after river ALPICAM ‘base de vie’ then 40 km on forestry road starting 4 km before Maséa village, 3.09°N, 14.66°E, *07 March 2019*, *Couvreur T.L.P.* 1223 (MPU,WAG,YA); Roue Mintom I (70 km E de Djoum-Alati (100 km SE de Djoum) PK 15, 2.83°N, 13.35°E, *01 January 1973*, *Letouzey R.* 11773 (K,P,WAG,YA); Piste du massif de Fessimi sur la carte de Deng-Deng, 5.2°N, 13.52°E, *09 February 1961*, *Letouzey R.* 3433 (YA); Deng Deng, 5.2°N, 13.52°E, *01 April 1914*, *Mildbraed G.W.J.* 8826 (K). **Littoral Region**: Yabassi, 4.34°N, 10.41°E, *27 October 2007*, *Fenton E.* 163 (K); Duala, 4.05°N, 9.71°E, *01 June 1917*, *Fleury F.* 33338 (P); Olombé, 3.60°N, 9.958°E, *05 November 2014*, *Kamdem N.* 177 (YA). **South Region**: Okala (Batanga), 3.2°N, 11.02°E, *20 September 1945*, *Aubréville A.* 133 (P); Ebolowa, 2.96°N, 11.28°E, *20 November 1968*, *Bos J.J.* 3341 (P,WAG,YA); ca 15 km from Kribi 1 km S of Ebolowa road, 3.1°N, 10.25°E, *20 February 1970*, *Bos J.J.* 6382 (B,BR,C,K,LD,M,P,WAG,YA); 23 km east from Lélé village, 2.28°N, 13.32°E, *08 September 2013*, *Couvreur T.L.P.* 471 (WAG,YA); 16 km on the recently reconstructed road from Ebolowa to Minkok, 2.75°N, 11.25°E, *29 January 1975*, *de Wilde J.J.F.E* 7931 (B,BR,K,MO,P,U,WAG,YA); Station de cacaoyer de N’koemvone 14 km on the road from Ebolowa to Ambam, 2.81°N, 11.13°E, *17 February 1975*, *de Wilde J.J.F.E* 7970 (BR,K,MO,P,U,WAG,YA); 18 km S of the Lobé River along the road to Campo, 2.71°N, 9.866°E, *18 March 1975*, *de Wilde J.J.F.E* 8085 (BR,MO,P,WAG,YA); Station de cacaoyer de N’koemvone 14 km On the road from Ebolowa to Ambam, 2.8°N, 11.13°E, *10 December 1975*, *de Wilde J.J.F.E* 8707 (BR,K,MO,P,U,WAG); Mebemonko (20 km NO d’Oveng), 2.55°N, 12.17°E, *24 October 1966*, *Letouzey R.* 8186 (YA); km 48 route Kribi-Ndjabilobi (village Elone), 3.1°N, 10.25°E, *23 July 1957*, *Mpom B.* 282 (P,YA); Canon du Ntem 16 km SW de Nyabessan, 2.32°N, 10.28°E, *30 November 1982*, *Nkongmeneck B.A.* 399 (P); Campo-Ma’an area Nsengou, 2.18°N, 10.58°E, *05 February 2001*, *Tchouto Mbatchou G.P.* 3126 (KRIBI,WAG); Campo-Ma’an area Ebodje, 2.56°N, 9.833°E, *16 April 2001*, *van Andel T.R.* 3343 (KRIBI,WAG,YA). **South-West Region**: Bayang Mbo Wildlife Sanctuary before Mbu river, 5.34°N, 9.495°E, *27 March 2016*, *Couvreur T.L.P.* 1021 (WAG,YA); on trail through palm oil plantation 3 km before lava flow and Seme Beach hotel when coming from Limbe, 4.06°N, 9.078°E, *18 October 2013*, *Couvreur T.L.P.* 516 (WAG,YA); Bakolle Bakossi on Kumba-Mamfe road, 5.01°N, 9.666°E, *24 May 1986*, *Etuge M.* 148 (MO,WAG,YA); A 15 km au Sud Est de Molobo (village situé à 50 km au Sud de Batouri), 4.02°N, 14.32°E, *21 July 1963*, *Letouzey R.* 5456 (P,WAG,YA).

### ﻿﻿﻿ Xylopia africana

Taxon classificationPlantaeMagnolialesAnnonaceae

Oliv., Fl. Trop. Afr. 1: 30, 1868

3A6B508D-EAC7-5C98-8093-BAF479E2810D

Fig. 142 ; Map 17C

≡ Melodorum africanum Benth., Trans. Linn. Soc. London 23(3): 477, 1862; ﻿Xylopicrumafricanum (Bentham) Kuntze, Revis. gen. pl. 1: 8, 1891. ﻿Fissistigmaafricanum (Bentham) Merrill, Philipp. J. Sci. 15: 130, 1919.

#### Type.

Cameroon. South-West Region; Mount Cameroon, *Mann G. 1193*, Feb 1862: lectotype, designated by Johnson and Murray (2018), p. 42: K[K000105591]; isolectotypes: GH; K[K000105592]; P[P00169118]; U[U0095511].

#### Description.

Tree, up to 20 m tall, d.b.h. up to 30 cm; **stilt roots present, to 2 m above the base**. Old branches glabrous, **brown**, young branches pubescent, with appressed hairs 0.2–0.3 mm long. Leaves: petiole 6–9 mm long, ca. 2 mm wide, pubescent to glabrous, grooved, blade inserted on the side of the petiole; blade 6.3–15.4 cm long, 2.9–7.9 cm wide, oblong to obovate, sometimes elliptic, apex acuminate to cuspidate, acumen 0.2–1.0 cm long, base cuneate and decurrent, coriaceous to subcoriaceous, below pubescent when young, glabrous when old, above glabrous when young and old, discolorous, much paler below; midrib flat to sunken, above glabrous when young and old, below glabrous to sparsely pubescent when young, glabrous to sparsely pubescent when old; secondary veins 10 to 13 pairs, glabrous above; tertiary venation reticulate. Individuals bisexual; inflorescences ramiflorous on young foliate branches, axillary, peduncle not visible. Flowers with 9 perianth parts in 3 whorls, 1 to 2 per inflorescence; pedicel 6–11 mm long, 1–2 mm in diameter, pubescent; in fruit 10–18 mm long, 4–5 mm in diameter, glabrous; bracts 2, towards the middle of pedicel, 1–3 mm long, 1–3 mm wide; sepals 3, valvate, basally fused, 5–7 mm long, 5–6 mm wide, triangular to ovate, apex acute, base truncate, green, pubescent outside, glabrous inside; petals free; **outer petals 3, 8.2–9.5 mm long, 3–4.5 mm wide at base, ovate, apex acute to rounded, base truncate**, yellow, minutely pubescent outside, minutely pubescent inside; inner petals 3, valvate, 5.9–8.3 mm long, 1–2.6 mm wide at base, elliptic to rhombic, apex acuminate to acute, base truncate, yellow, pubescent, nearly glabrous outside, minutely pubescent inside; stamens 100 to 120, in 7 to 8 rows, 2–3 mm long, clavate to oblong; connective apex shield-like, pubescent, cream; carpels 9 to 15, ovary 2 mm long, stigmas connivent, linear, 3.2–4.6 mm long, glabrous. Monocarps stipitate, stipe 10–18 mm long, 3–5 mm in diameter; monocarps 1 to 10, 66–100 mm long, 10–14 mm wide, **narrowly oblong to cylindrical**, somewhat falciform and torulose, apex with a beak 1–1.5 mm long, glabrous, verrucose and longitudinally wrinkled when dried, smooth when fresh, reddish to purplish green outside, endocarp red; seeds 1 to 5 per monocarp, **in a single row**, 13–15 mm long, 9–11 mm wide, ellipsoid; **sarcotesta absent; aril brushlike, unlobed, blood-red.**

#### Distribution.

Eastern Nigeria and Cameroon, also on the Islands of Bioko (Equatorial Guinea) and São Tomé (São Tomé & Principe); in Cameroon known from Central, South-West, Northwest and West regions.

#### Habitat.

A fairly common species, in montane and submontane mossy forests, on non-inundated soils. Altitude 900–2000 m a.s.l.

#### Local and common names known in Cameroon.

Focho et al. (2010) reported the name “hweneta (Ghana)” being used for this species in the Mt. Cameroon area.

#### IUCN conservation status.

Vulnerable (VU) (Cheek 2014f).

#### Uses in Cameroon.

None recorded.

#### Notes.

﻿﻿*Xylopiaafricana* resembles ﻿﻿*X.staudtii* in the short ovate petals (< 10 mm long), shape of the fruit, and brushlike arils; both have stilt roots at the base of the trunk. However, ﻿*X.africana* is mainly a montane (> 900 m) species and is a smaller tree with the arils on the seeds blood-red rather than bright yellow to orange as in ﻿﻿*X.staudtii*. The montane habitat is rare among African ﻿*Xylopia* species.

#### Specimens examined.

**Central Region**: Chaîne de Nkohom à 42 km SSW de Ndiki, 4.77°N, 10.83°E, *14 November 1983*, *Nkongmeneck B.A.* 580 (P). **North-West Region**: High ridge on boundary of Bali-Ngemba, 5.80°N, 10.09°E, *14 November 2000*, *Cheek M.* 10527 (K,MO,P,WAG,YA); Gazette Bali Ngemba F R, 5.81°N, 10.08°E, *05 October 2001*, *Onana J.M.* 1825 (K); Gazette Bali Ngemba F R, 5.81°N, 10.08°E, *05 October 2001*, *Onana J.M.* 1835 (K). **South-West Region**: Nyasoso, 4.86°N, 9.7°E, *04 June 1996*, *Cable S.* 2870 (K,YA); Kupe village, 4.78°N, 9.716°E, *15 July 1996*, *Cable S.* 3814 (K,YA); Mungo River F.R., 4.78°N, 9.607°E, *30 November 1999*, *Cheek M.* 10203 (K); Peak 1, 4.80°N, 9.708°E, *01 November 1995*, *Cheek M.* 7605 (K,YA); Kodmin Mt above the village, 4.99°N, 9.701°E, *13 February 1998*, *Cheek M.* 9192 (K,P,WAG,YA); Rumpi mountains forest trail ca 5 km after Dikome Balue village ca 40 km north of Kumba, 4.93°N, 9.242°E, *10 January 2016*, *Couvreur T.L.P.* 949 (WAG,YA); Rumpi mountains forest trail ca 5 km after Dikome Balue village ca 40 km north of Kumba, 4.93°N, 9.225°E, *11 January 2016*, *Couvreur T.L.P.* 967 (WAG,YA); slopes of Mount Cameroon on the Bokwango trail near Bokwango village 4 km south west of Buea, 4.12°N, 9.183°E, *23 March 2016*, *Couvreur T.L.P.* 993 (WAG,YA); Buea, 4.15°N, 9.24°E, *01 January 1904*, *Deistel H.* 454 (U); Below Kupe rock near Esense river, 4.78°N, 9.683°E, *25 January 1995*, *Elad M.* 90 (K,YA); Likombe, 4.21°N, 9.166°E, *22 February 1995*, *Etuge M.* 1160 (K,YA); Kupe village, 4.78°N, 9.7°E, *08 November 1995*, *Etuge M.* 1441 (K,WAG,YA); Nyasoso, 4.81°N, 9.716°E, *27 February 1996*, *Etuge M.* 1740 (K,WAG,YA); 5 km west of village spring in Kodmin, 4.98°N, 9.7°E, *22 January 1998*, *Gosline W.G.* 75 (K,WAG,YA); Monts Rumpi-Rata Mount (1788 m) 2 km au SW de Dikome Balue, 4.89°N, 9.23°E, *24 March 1976*, *Letouzey R.* 14551 (P,YA); Buea, 4.15°N, 9.233°E, *01 January 1929*, *Maitland T.D.* 233 (K); Cameroon Mountain, Buea area, 4.15°N, 9.233°E, *01 January 1930*, *Maitland T.D.* s.n. (K[K000105602]); Cameroon Mountain, 4.2°N, 9.183°E, *Feburary 1862*, *Mann G.* 1193 (K); southern slope of Mount Cameroon above Batoke, 4.13°N, 9.08°E, *09 January 1984*, *Thomas D.W.* 2981 (K,MO); Forest in the Rumpi Hills near Dikome Balue, 4.90°N, 9.253°E, *01 March 1984*, *Thomas D.W.* 3305 (K,MO,P,WAG,YA); Aguosho 10 km SSW of Akwaya, 6.3°N, 9.466°E, *19 March 1985*, *Thomas D.W.* 4554 (YA); Limbe District Fako Division Mt Etinde N face of N ridge, 4.07°N, 9.11°E, *24 October 1992*, *Wheatley J.I.* 605 (K,P). **West Region**: Banna, 5.15°N, 10.27°E, *01 January 1904*, *Deistel H.* 151 (A,BM,P); Route Batcha-Batchingou (22 km ESE Bafang), 5.15°N, 10.18°E, *23 November 1974*, *Letouzey R.* 13300 (P,YA).

### ﻿﻿﻿ Xylopia aurantiiodora

Taxon classificationPlantaeMagnolialesAnnonaceae

De Wild. & T. Durand, Ann. Mus. Congo Belge, Bot. Sér. 2, 1(1): 4, 1899

148519A8-11F5-5B83-8A6E-A174E00639CB

Map 17D

≡ Artabotrys aurantiiodorus (De Wild. & Th. Durand) Engl., Monogr. Afrik. Pflanzen.-Fam. 6: 76, 1901.
= Xylopia bequaertii De Wild. Pl. Bequaert. 1: 469 1922. Type. Democratic Republic of the Congo. Tshopo, Kisangani, *Bequaert J.C.C. 6994*, 27 Feb 1915: holotype: BR.

#### Type.

Democratic Republic of the Congo. Equateur; Coquilhatville[= Mbandaka], *Dewèvre A.P. 660*, 24 Jan 1896: lectotype, sheet here designated: BR[BR0000008824691]; isotypes: BR[BR0000008824271, BR0000008824271].

#### Description.

Tree to shrub, 2–10 m tall, d.b.h. 30 cm; stilt roots and buttresses absent. Old branches glabrous, young branches glabrous to sparsely pubescent, the hairs 0.2–0.3 mm long. Leaves: petiole 3–7 mm long, ca. 1 mm wide, glabrous to sparsely pubescent, slightly grooved, blade inserted on the side of the petiole; blade 6.3–11.1 cm long, 2.5–4.7 cm wide, **elliptic to oblong, occasionally oblanceolate**, apex acute to obtuse, base cuneate and decurrent, papyraceous to subcoriaceous, below glabrous when young, glabrous to sparsely pubescent when old, above glabrous when young and old, discolorous, tan-colored below; midrib sunken or flat, above glabrous to sparsely pubescent when young, glabrous to sparsely pubescent when old, below glabrous when young, sparsely pubescent when old; secondary veins 10 to 14 pairs, glabrous above; tertiary venation reticulate. Individuals bisexual; inflorescences ramiflorous on young foliate branches, axillary, peduncle 1.5–2 mm long. Flowers with 9 perianth parts in 3 whorls, 1 to 5 per inflorescence; pedicel 4–7 mm long, ca. 1 mm in diameter, sparsely pubescent to glabrous; in fruit 7–10 mm long, 1–2 mm in diameter, sparsely pubescent to glabrous; bracts 2 to 3, basal and towards the upper half of pedicel, all similar, 1–2 mm long, 1–2 mm wide; sepals 3, slightly imbricate at base, free, 2 mm long, 2 mm wide, triangular, apex apiculate, base truncate, pubescent outside, glabrous inside; petals free, subequal; outer petals 3, 8.9–12 mm long, 2.4–3.5 mm wide at base, ligulate-lanceolate, **apex obtuse**, base broad and concave, light yellow to light green, pubescent outside, pubescent but glabrous towards center inside; inner petals 3, valvate, 9.3–11.2 mm long, 1.2–2.3 mm wide at base, narrowly oblong, **apex obtuse, base broad and concave with a pronounced internal tooth overhanging the concavity and fleshy glandlike margins**, light yellow to greenish yellow, densely pubescent outside, pubescent inside; stamens 40 to 60, in 5 to 6 rows, ca. 2 mm long, oblong; connective apex capitate, glabrous; carpels 3 to 5, ovary 1 mm long, stigmas slightly separate, narrowly oblong, ca. 1 mm long, glabrous. Monocarps stipitate, stipe 6–10 mm long, 2–3 mm in diameter; monocarps 1 to 3, 33–65 mm long, 7–9 mm wide, narrowly oblong, sometimes falciform, strongly torulose, apex with an acute beak up to 5 mm long, glabrous, wrinkled or striate when dried, green outside, endocarp color unknown; seeds 1 to 4 per monocarp, **in a single row**, 13–21 mm long, 6–8 mm wide, ellipsoid, pointed at one end; **sarcotesta absent; aril present, fimbriate, extending over the length of the seed, membranous, smooth, red to orange-brown when dried.**

#### Distribution.

From southern Cameroon to the Democratic Republic of the Congo; in Cameroon known from the East region.

#### Habitat.

An uncommon species; in riparian forest, on riverbanks, and in inundated rain forests. Altitude 0–500 m a.s.l.

#### Local and common names known in Cameroon.

None recorded.

#### IUCN conservation status.

Least Concern (LC) (Harvey-Brown 2019h).

#### Uses in Cameroon.

None reported.

#### Notes.

﻿﻿*Xylopiaaurantiiodora* and ﻿*X.quintasii* have short blunt petals and fimbriate orange-red arils. ﻿﻿*Xylopiaaurantiiodora* differs in its smaller stature, the prominent tooth on the inside of the inner petal base, striate monocarps with an acute beak, and larger pointed seeds. The tooth of the inner petal is especially useful for identification, because it appears early in petal development and is visible even in young buds. The two species also occupy different habitats, with ﻿*X.aurantiiodora* in riparian forests and ﻿*X.quintasii* usually in upland forests.

#### Specimens examined.

**East Region**: West bank of Sangha River opposite Ndakan camp and 2 km S, 2.35°N, 16.13°E, *12 February 1989*, *Harris D.J.* 1846 (MO); Sangha R, 2.78°N, 16°E, *22 May 1988*, *Harris D.J.* 752 (P); Mbekou près Moloundou, 2.05°N, 15.22°E, *01 January 1959*, *Letouzey R.* 1374 (P).

### ﻿﻿﻿ Xylopia calva

Taxon classificationPlantaeMagnolialesAnnonaceae

D.M.Johnson & N.A.Murray, PhytoKeys 97: 154, 2018

E1636E20-A45F-5D28-B814-8CF2851D1BB9

Map 17E

#### Type.

Cameroon. South Region; Bipindi, *Zenker G.A. 4747*, 1903: holotype: BM[000511011]; isotypes: BR[BR0000014581892]; G; K; L[0191105]; M; MO[751089]; P.

#### Description.

Tree, height unknown, d.b.h. up to 30 cm; **buttresses present.** Old branches glabrous, young branches pubescent, the hairs 0.3–0.4 mm long. Leaves: petiole 3–10 mm long, 2 mm wide, sparsely pubescent, slightly grooved, blade inserted on top of the petiole; blade 10–17.2 cm long, 3.6–6.5 cm wide, elliptic to oblong, apex acuminate, acumen 0.3–2.1 cm long, base cuneate to rounded, papyraceous to subcoriaceous, below sparsely pubescent when young, sparsely pubescent when old, above glabrous when young, sparsely pubescent when old; concolorous **or slightly glaucous abaxially**; midrib sunken, above sparsely pubescent when young and old, below sparsely pubescent when young and old; secondary veins 8 to 15 pairs, glabrous above; tertiary venation reticulate. Individuals bisexual; inflorescences ramiflorous on young foliate branches, axillary, **peduncle branched, 1–3 mm long**. Flowers with 9 perianth parts in 3 whorls, 1 to 10 per inflorescence; pedicel 5–8 mm long, 1–2 mm in diameter, pubescent; in fruit 6–7 mm long, 2–3 mm in diameter, rusty pubescent; bracts 1 to 2, one basal and one upper towards the middle of pedicel, 2–3 mm long, 2–3 mm wide; sepals 3, valvate, basally fused, 4–5 mm long, 4–5 mm wide, ovate, apex acute to obtuse, base truncate, densely pubescent outside, glabrous inside; petals free, subequal; outer petals 3, 13–23 mm long, **3.8–6 mm wide** at base, oblong-lanceolate, apex obtuse, base broad and concave, light yellow, pubescent, base glabrous outside, pubescent inside; inner petals 3, valvate, 10–17.5 mm long, 2.5–4 mm wide at base, oblong-lanceolate, apex acute, base broad and concave, yellow with red on the inner base, pubescent outside, **glabrous inside except at apex**; stamens ca. 200, in 5 to 6 rows, 1–2 mm long, oblong; connective apex shield-like, glabrous, red; carpels ca. 9, ovaries 2 mm long, stigmas connivent at base, linear, slightly thickened at the midpoint, ca. 2.2 mm long, pubescent. Monocarps stipitate, stipe ca. 3 mm long, ca. 7 mm in diameter; monocarps ca. 8, ca. 32 mm long, ca. 17 mm wide, ellipsoid, apex rounded, pubescent, verrucose and slightly wrinkled when dried, color unknown; seeds 7 to 8 per monocarp, **in two rows**, ca. 9 mm long, ca. 7 mm wide, ellipsoid to flattened ellipsoid, sarcotesta unknown; **aril absent.**

#### Distribution.

From southern Nigeria to Cameroon; in Cameroon known only from the South region.

#### Habitat.

A rare species, known from three collections, two in Cameroon. Altitude 150–200 m a.s.l.

#### Local and common names known in Cameroon.

None recorded.

#### IUCN conservation status.

Endangered (EN) (Cosiaux et al. 2019ay).

#### Uses in Cameroon.

None reported.

#### Notes.

The relatively broad petals of ﻿*X.calva* are distinctive in the absence of hairs on the inner surface except at the apex. ﻿﻿*Xylopiacalva* has a pedunculate and branched inflorescence type similar to that found in ﻿*X.phloiodora* and ﻿*X.paniculata*.

#### Specimens examined.

**South Region**: Près des chutes du Ntem ou de Menvé’élé près Nyabessan (60 km Est de Campo), 2.39°N, 10.35°E, *08 April 1970*, *Letouzey R.* 10306 (P,YA); Bipindi, 3.08°N, 10.41°E, *01 January 1913*, *Zenker G.A.* 4747 (BM,BR,G,K,L,M,MO,P).

### ﻿﻿﻿ Xylopia cupularis

Taxon classificationPlantaeMagnolialesAnnonaceae

Mildbr., Notizbl. Bot. Gart. Berlin-Dahlem 8: 56, 1921

479FFD11-4052-534E-85F0-A5B08D48C83A

Fig. 143 ; Map 17F

= Xylopia gilviflora Exell, J. Bot. 73 (Suppl. 1): 4, 1935. Type. Angola. Cabinda, Buco Zau, Mayumbe, *Gossweiler J. 6933*, 15 Jan 1917: holotype: BM; isotypes: B[100153140]; COI[00004882]; LISC[LISC000308, LISC000309, LISC000310, LISC000311, LISC000312, LISC000313, LISC000314, LISC000315, LISC000316].
= Xylopia chrysophylla Louis ex Boutique, Bull. Jard. Bot. État 21: 108, 1951. Type. Democratic Republic of the Congo. Tshopo, Yangambi, *Louis J. 4309*, Jun 1937: holotype: BR[BR0000024941518]; isotypes: BR[0000008824752]; MO[1639095, 3007016]; NY[00066781]; US[2091336].

#### Type.

Cameroon. East Region; Deng-Deng, *Mildbraed G.W.J. 8649*, Mar 1914: holotype B[B 10 0153141]; isotype: BM[fragment].

#### Description.

Tree, up to 35 m tall, d.b.h. up to 50 cm; **buttresses present, small.** Old branches glabrous, young branches glabrous to pubescent, with fine appressed hairs 0.1–0.3 mm long. Leaves: petiole 2–5 mm long, ca. 1 mm wide, sparsely pubescent, slightly grooved, blade inserted on the side of the petiole; **blade 4.6–7.8 cm long**, 1.3–2.4 cm wide, lanceolate to elliptic or elliptic-oblong, apex acuminate, acumen 0.6–1.1 cm long, base cuneate to broadly cuneate, usually asymmetrical, papyraceous, **below sparsely to densely golden-sericeous (appressed) when young**, sparsely pubescent to densely pubescent when old, above glabrous when young and old, concolorous; midrib slightly raised, above glabrous to sparsely pubescent when young, glabrous when old, below pubescent when young and old; secondary veins 10 to 15 pairs, glabrous above; tertiary venation reticulate. Individuals bisexual; inflorescences ramiflorous on young foliate branches, axillary, peduncle rarely present, up to ca. 4 mm long. Flowers with 9 perianth parts in 3 whorls, 1 to 3 per inflorescence; pedicel 5–12 mm long, ca. 1 mm in diameter, sparsely pubescent; in fruit 6–15 mm long, 3–10 mm in diameter, glabrous; bracts 2, towards the middle of pedicel, ca. 2 mm long, ca. 2 mm wide; sepals 3, valvate, **1/2–3/4 fused, forming a cup, 2–3 mm long, 2–3 mm wide**, broadly ovate, apex acute to obtuse, base truncate, pubescent outside, glabrous inside; petals free, subequal; outer petals 3, 15.5–36 mm long, 2.2–3.4 mm wide at base, linear to linear-lanceolate, apex acute, base broad and concave, yellow to cream, sericeous outside, pubescent inside; inner petals 3, valvate, 13.5–17.7 mm long, 2.4–3.5 mm wide at base, linear, apex acute, base broad and concave, yellow to cream, pubescent with glabrous base on both surfaces; stamens 160 to 200, in 5 to 6 rows, 1–2 mm long, oblong; connective apex shield-like, glabrous; carpels 12 to 20, ovary ca. 1 mm long, 3.5–3.8 mm long, stigmas connivent, filiform, 2.5–2.8 mm long, pubescent. Monocarps stipitate, stipe 5–24 mm long, 2–5 mm in diameter; **monocarps 11 to 18**, 23–54 mm long, 14–19 mm wide, obovoid to oblongoid, apex rounded, glabrous, verrucose and wrinkled when dried, reddish green outside, endocarp pink to dark red; seeds 6 to 8 per monocarp, **in two rows**, 10–13 mm long, 6–10 mm wide, ellipsoid; **sarcotesta glaucous blue or gray; aril absent.**

#### Distribution.

A widespread species across Central Africa, from southeastern Nigeria to northeastern Democratic Republic of the Congo and south to Angola; in Cameroon known from the East, South, Littoral and South-West regions.

#### Habitat.

An uncommon species in lowland rain forest, semi-deciduous forest, forest-savanna edges, and secondary forest. Altitude 50–800 m a.s.l.

**Figure 143.**
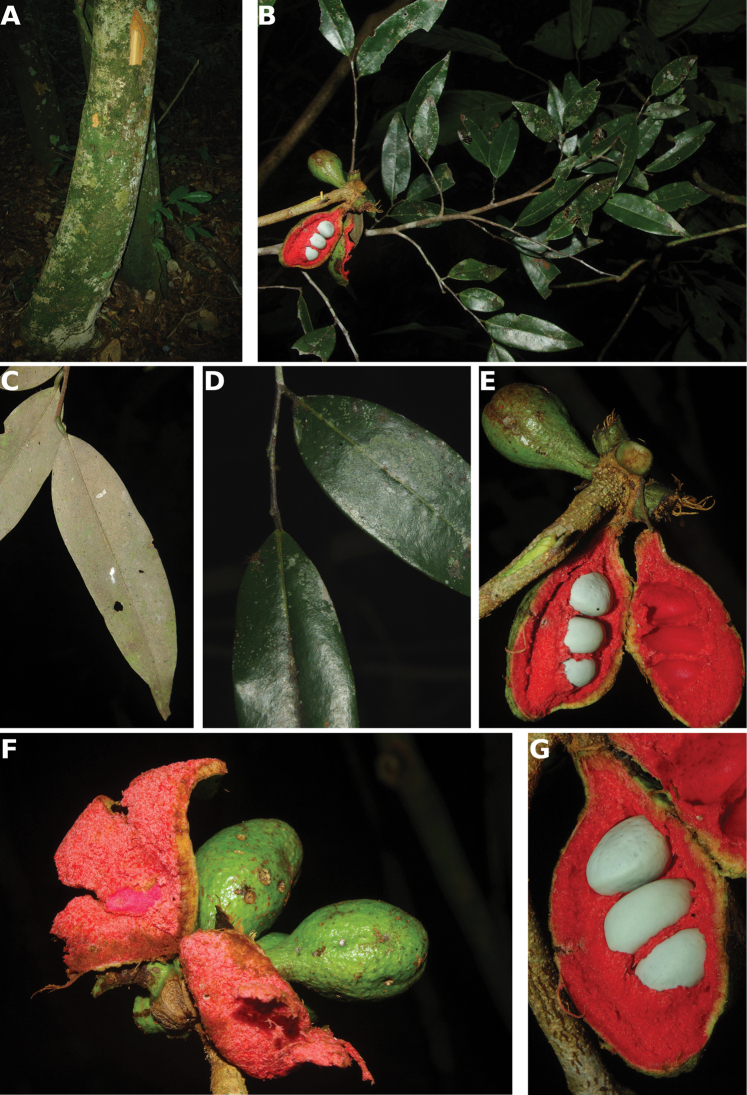
*Xylopiacupularis***A** base of trunk **B** fruiting branch **C** leaf, lower side **D** base of leaf blade, upper side **E** fruit, one monocarp open, note bright white sarcotesta and red inner surface of monocarp **F** mature fruit, monocarps opening longitudinally **G** detail of opened monocarp showing seeds covered with a white sarcotesta nested in a red inner monocarp pulp **A–G** Couvreur 485, Lélé, Cameroon. Photos Thomas L.P. Couvreur.

#### Local and common names known in Cameroon.

odjobi (Yaoundé, *Mbarga 1940*).

#### IUCN conservation status.

Least Concern (LC) (Harvey-Brown 2019i).

#### Uses in Cameroon.

None reported.

#### Notes.

﻿﻿*Xylopiacupularis* is distinctive among African ﻿*Xylopia* species in its combination of small acuminate leaves, often asymmetrical at the base, with golden appressed pubescence below, relatively long pedicels, sepals fused into a cup-shaped calyx, and the numerous monocarps, which are usually distinctly stipitate. It is often a large tree, and thus may be infrequently collected.

#### Specimens examined.

**East Region**: IK 30 Road Mintom 1 (70 km E de Djoum)- Alati (100 km SE de Djoum), 2.83°N, 13.35°E, *08 January 1973*, *Letouzey R.* 11801 (P,WAG); 21 km ENE of Moloundou-Nguilili Chantier, 2.05°N, 15.17°E, *10 March 1975*, *Mbenkum T.F.* 310 (P,YA); Deng Deng, 5.2°N, 13.52°E, *01 March 1914*, *Mildbraed G.W.J.* 8649 (B). **Littoral Region**: 6 km NE of Dibombe a village on km 11 of Loum-Yabassi road, 4.68°N, 9.816°E, *26 May 1972*, *Leeuwenberg A.J.M.* 9915 (WAG,YA); Douala (route Razel), 4.02°N, 9.71°E, *Mbarga A.* 58 (P,YA). **South Region**: 30 km east from Lélé village, 2.27°N, 13.29°E, *09 September 2013*, *Couvreur T.L.P.* 485 (WAG,YA). **South-West Region**: Likomba-Pflanzung 15–35 km NE von Victoria [Limbe], 4.09°N, 9.33°E, *01 November 1928*, *Mildbraed G.W.J.* 10629 (A,K); Mount Mabeta peninsula Mabeta, 3.98°N, 9.233°E, *19 October 1997*, *Tchouto Mbatchou G.P.* 1756 (MO).

### ﻿﻿﻿ Xylopia elliotii

Taxon classificationPlantaeMagnolialesAnnonaceae

Pierre ex Engl. & Diels, Monogr. Afrik. Pflanzen.-Fam. 6: 65, 1901

5BC58269-09B9-59F8-818A-9C355735B9A6

Map 17G

#### Type.

Guinea. Farana Region; Farana, *Scott Elliot G.F. 5325*, Mar 1924: lectotype, designated by Johnson and Murray (2018), p. 165: B[B100153142]; isolectotypes: BM[BM000510796, lower half of sheet]; GH; K[K000199071]; P[P00169156]

#### Description.

Tree, 10–18 m tall, d.b.h. unknown; stilt roots and buttresses absent. Old branches glabrous, **young branches densely pubescent with erect reddish brown hairs 0.2–0.8 mm long**. Leaves: petiole 4–6 mm long, ca. 1 mm wide, pubescent, slightly grooved, blade inserted on the side of the petiole; blade 4.5–9.5 cm long, 1.8–5.6 cm wide, lanceolate-ovate to elliptic, oblong, or oblong-lanceolate, **apex obtuse**, emarginate, or acute, rarely with an acumen ca. 0.5 cm long, base cuneate to rounded, subcoriaceous, below finely appressed-pubescent when young, sparsely pubescent to glabrous when old, above glabrous when young and old, discolorous; midrib sunken, above pubescent when young and old, below pubescent when young and old; secondary veins 10 to 16 pairs, glabrous above; tertiary venation reticulate. Individuals bisexual; inflorescences ramiflorous on young foliate branches, axillary, peduncle absent. Flowers with 9 perianth parts in 3 whorls, 1 to 2 per inflorescence; pedicel 3–6 mm long, 1 mm in diameter, pubescent; in fruit 10–12 mm long, 1–6 mm in diameter, glabrous; **bracts 2**, towards the middle of pedicel, 2–3 mm long, 2–3 mm wide; sepals 3, valvate, basally fused, 2–5 mm long, 3–4 mm wide, ovate, apex acute to apiculate, base truncate, sericeous outside, glabrous inside; petals free, subequal; outer petals 3, 12.6–32 mm long, 1.2–2.5 mm wide at base, linear, apex obtuse, base broad and concave, white, tinged purple at base, densely pubescent outside, pubescent inside; inner petals 3, valvate, 16–24 mm long, 2.3–3.9 mm wide at base, linear, apex acute, base broad and concave, white, tinged with purple at the base, pubescent, base glabrous outside, pubescent and glabrous towards base inside; stamens ca. 120, in 7 to 8 rows, 1–2 mm long, clavate to oblong; connective apex shield-like, glabrous; carpels 9 to 10, ovary 1–2 mm long, stigmas connivent, lanceolate, 1.3–2.3 mm long, pubescent to glabrous. Monocarps **usually sessile** or stipitate, stipe 3–6 mm long, 2–3 mm in diameter; monocarps 3 to 8, 20–38 mm long, 9–13 mm wide, obovoid to oblong, apex rounded, glabrous to sparsely pubescent, verrucose, slightly wrinkled when dried, red to purple tinged outside, endocarp red; seeds up to 9 per monocarp, **in two rows**, 9–12 mm long, 7–8 mm wide, flattened ellipsoid to oblong; **sarcotesta present**, color *in vivo* unknown; **aril absent.**

#### Distribution.

A widespread species with a disjunct distribution in West and Central Africa, from Guinea-Bissau to Togo and from Cameroon to Central African Republic; in Cameroon known from Adamaoua and West regions.

#### Habitat.

A species from the drier forests, in gallery forests along streams and rivers and occasionally extending into drier uplands. Altitude 1100–1400 m a.s.l.

#### Local and common names known in Cameroon.

ké (*Westphal 10047*, *10048*, *10049*, *10172*).

#### IUCN conservation status.

Least Concern (LC) (Harvey-Brown 2019j).

#### Uses in Cameroon.

***Food***: fruits and seeds used as pepper substitute.

#### Notes.

﻿﻿*Xylopiaelliotii* most closely resembles *X.monticola* and ﻿﻿*X.thomsonii* among Cameroon ﻿*Xylopia* species, but has obtuse to acute rather than acuminate leaf apices, young branches densely covered with erect reddish brown hairs of even length, pedicels with only 2 rather than 3–6 bracts. The monocarps of ﻿﻿*Xylopiaelliotii* are sessile to short-stipitate (3–6 mm) with seeds in two rows, in contrast to distinctly stipitate (8–13 mm long, rarely shorter) with seeds in a single row in *X.monticola* and ﻿﻿*X.thomsonii*.

Collections from the area of Bayangam (*Westphal 10047*, *10048*, *10049*, *10172*) document that the plant has been kept around houses and the fruits are used as a condiment. This is the only known report of the use of a ﻿*Xylopia* species in Africa other than ﻿*X.aethiopica*.

#### Specimens examined.

**Adamaoua Region**: Falls in the Vina river ca 15 km SE of Ngaoundéré, 7.2°N, 13.71°E, *01 December 1964*, *de Wilde W.J.J.O* 4447 (K,WAG); Mbalang-16 km E Ngaoundéré, 7.32°N, 13.58°E, *27 January 1978*, *Fotius G.* 2984 (YA); Tchabal-Mbabo, 7.05°N, 12.25°E, *16 March 1978*, *Fotius G.* 3108 (YA); Ngaou Loumou (1700 m), 7.52°N, 13.85°E, *20 October 1967*, *Jacques-Félix H.* 8724 (YA); Mayo Darle, 6.47°N, 11.55°E, *10 November 1967*, *Jacques-Félix H.* 9110 (YA); Sadolkoulay (36 km E Ngaoundéré), 7.31°N, 13.87°E, *05 December 1964*, *Raynal J.* 12228 (P,YA); Près Katil-Foulbe 50 km SE Ngaoundéré, 7.02°N, 13.94°E, *20 October 1983*, *Satabié B.* 687 (P). **West Region**: Ndop Plain road to French Map ref No 23, 5.97°N, 10.40°E, *30 March 1962*, *Brunt M.A.* 261 (K); Bayangam, 5.3°N, 10.45°E, *01 January 1939*, *Jacques-Félix H.* 2965 (P); Kontchankap, 5.58°N, 10.80°E, *01 February 1939*, *Jacques-Félix H.* 3039 (P); a 20 km E de Foumbot, 5.56°N, 10.8°E, *26 October 1974*, *Satabié B.* 15 (MO,P); Bayangam, 5.3°N, 10.45°E, *17 May 1978*, *Westphal E.* 10047 (P,WAG); Bayangam, 5.3°N, 10.45°E, *17 May 1978*, *Westphal E.* 10048 (WAG,YA); Bayangam, 5.3°N, 10.45°E, *17 May 1978*, *Westphal E.* 10049 (K,WAG,YA); Plants collected in the region of Bayangam, 5.3°N, 10.45°E, *17 December 1978*, *Westphal E.* 10172 (WAG).

### ﻿﻿﻿ Xylopia gilbertii

Taxon classificationPlantaeMagnolialesAnnonaceae

Boutique, Bull. Jard. Bot. État Bruxelles 21: 110, 1951

DA5D81BE-A0B4-5E48-8856-15F2DC5F406E

Map 17H

= Xylopia ardua Sillans, Rev. Int. Bot. Appl. Agric. Trop. 33: 555, 1953. Type. Central African Republic: Lobaye, Boukoko, *Tisserant C. 2329*, Dec 1951: lectotype, designated Johnson and Murray (2018), p.110: P[P00169140]; isotypes: BM[000510989]; P[P00169141, P00169142, P00169143]; US[2679729].

#### Type.

Democratic Republic of the Congo. Tshopo Region; Yangambi, plateau de la Luweo, *Louis J.L.P. 6777*, Nov 1937: holotype: BR[BR0000008824738]; isotypes: B[B100249561]; BM K[K000542217]; P[P00169152]; US.

#### Description.

Tree, up to 40 m tall, d.b.h. up to 40 cm; **buttresses present, small.** Old branches sparsely pubescent with erect hairs, **young branches pubescent with persistent erect hairs** 0.3–0.8 mm long. Leaves: petiole 3–4 mm long, ca. 1 mm wide, densely erect pubescent, slightly grooved, blade inserted on the side of the petiole; blade 4.2–9.7 cm long, 2–3.3 cm wide, elliptic-oblong to lanceolate, occasionally oblong or ovate, apex acute to rounded, base cuneate to rounded, papyraceous to subcoriaceous, below sparsely pubescent with erect hairs when young and old, above glabrous when young, glabrous to pubescent when old, slightly discolorous; midrib sunken or flat, above densely pubescent when young and old, below pubescent when young and old; secondary veins 7 to 14 pairs, pubescent above; tertiary venation reticulate. Individuals bisexual; inflorescences ramiflorous on young foliate branches, axillary, peduncle 1.5–2.5 mm long. Flowers with 9 perianth parts in 3 whorls, 1 to 6 per inflorescence; pedicel 1–2 mm long, 1–2 mm in diameter, densely pubescent; in fruit 5–10 mm long, 2–3 mm in diameter, sparsely pubescent; bracts 2, evenly spaced, 2–4 mm long, 2–3 mm wide; sepals 3, valvate, free to basally fused, ca. 2 mm long, 3–4 mm wide, reniform to semicircular, apex acute to rounded, base truncate, densely pubescent outside, glabrous inside; petals free, subequal, fawn-olive, brown-violet, or purple; **outer petals 3, 6.6–11 mm long, 2.4–4.4 mm wide at base, lanceolate to ovate**, apex obtuse, base broad and concave, densely pubescent outside, pubescent but glabrous towards center inside; inner petals 3, valvate, 5.8–9.3 mm long, 3–4 mm wide at base, lanceolate, apex acuminate to obtuse, base broad and concave, **with round glandlike thickenings on margins**, pubescent outside, pubescent but glabrous towards center inside; stamens 80–130, in 6 to 7 rows, ca. 1 mm long, clavate to oblong; connective apex shield-like to capitate, glabrous; carpels 6 to 11, ovary 1–2 mm long, stigmas connivent, linear, 1.5–2.2 mm long, sparsely pubescent. Monocarps stipitate, stipe 5–13 mm long, 1–4 mm in diameter; monocarps 1 to 10, 25–37 mm long, 10–13 mm wide, oblong, apex rounded or with a blunt beak 1–1.5 mm long, glabrous to **sparsely pubescent with erect hairs**, sparsely verrucose and weakly wrinkled when dried, reddish or purplish green outside, endocarp color unknown; seeds up to 6 per monocarp, **in a single row**, 7–10 mm long, 4–7 mm wide, ellipsoid; **sarcotesta red *in vivo*; aril absent.**

#### Distribution.

From southern Cameroon to northern Gabon and Central African Republic and east to the northeastern Democratic Republic of the Congo; in Cameroon known from the East and South regions.

#### Habitat.

An uncommon or infrequently collected species, in submontane rain forests. Altitude 470–900 m. a.s.l.

#### Local and common names known in Cameroon.

None recorded.

#### IUCN conservation status.

Vulnerable (VU) (Cosiaux et al. 2019az).

#### Uses in Cameroon.

None reported.

#### Notes.

﻿﻿*Xylopiagilbertii* is characterized by the sparse but persistent erect hairs of the young branches, leaves, and monocarps, the relatively broad but small flowers on short pedicels, and the rounded marginal glandlike thickenings on the inner petals. The relatively short broad outer petals are shared only with ﻿﻿*X.staudtii* and ﻿*X.africana* in the Cameroon flora.

#### Specimens examined.

**East Region**: A 9 km à l’ouest de Yenga Port Gentil village situé à 25 km au NNE de Moloundou, 2.34°N, 15.26°E, *21 April 1971*, *Letouzey R.* 10701 (P,YA). **South Region**: Près Akonetye PK 95 sur route Mintom I (70 km E de Djoum)-Mbalam (140 km ESE de Djoum), 2.67°N, 12.87°E, *22 January 1973*, *Letouzey R.* 11876 (P,WAG,YA).

### ﻿﻿﻿ Xylopia hypolampra

Taxon classificationPlantaeMagnolialesAnnonaceae

Mildbr., Notizbl. Königl. Bot. Gart. Berlin, Append. 27: 18, 1913

EB4BB415-8367-5CEB-9B99-CAF3218F49E4

**Figure 144.**
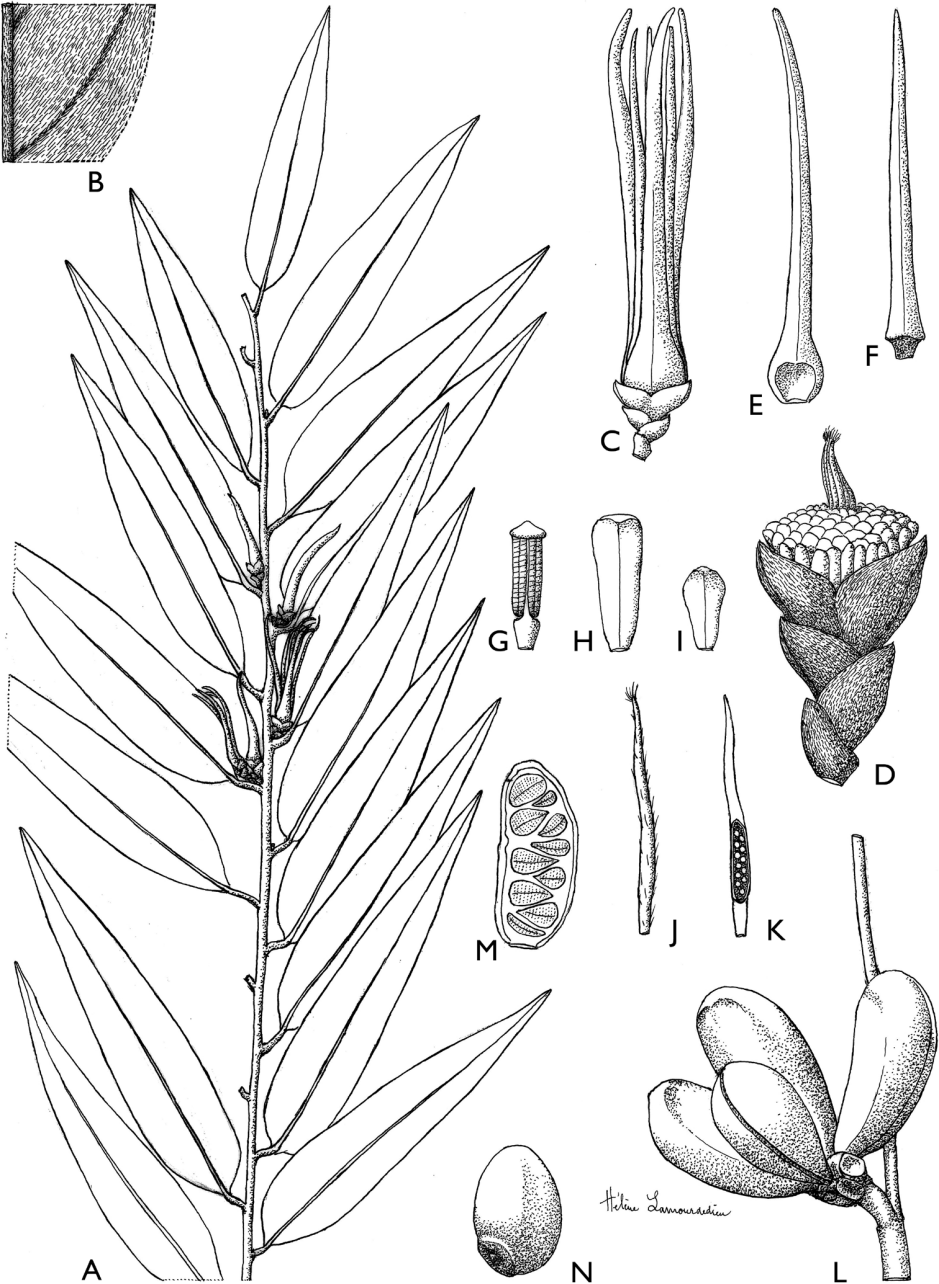
*Xylopiahypolampra***A** flowering branch **B** detail of dense pubescence on lower side of leaves **C** flower **D** detail of receptacle, all petals removed **E** outer petal, inner view **F** inner petal, inner view **G** stamen, front view **H** staminode, outer whorl **I** staminode, inner whorl **J** carpel **K** detail of ovules **L** fruit **M** longitudinal section of monocarp and seeds **N** detail of seed, side view **A–K** from *Le Testu 8094***L–N** from *Tisserant 1385*. Drawings by Hélène Lamourdedieu, Publications Scientifiques du Muséum national d’Histoire naturelle, Paris; modified from Le Thomas (1969b; pl. 34, p. 183).

**Figure 145.**
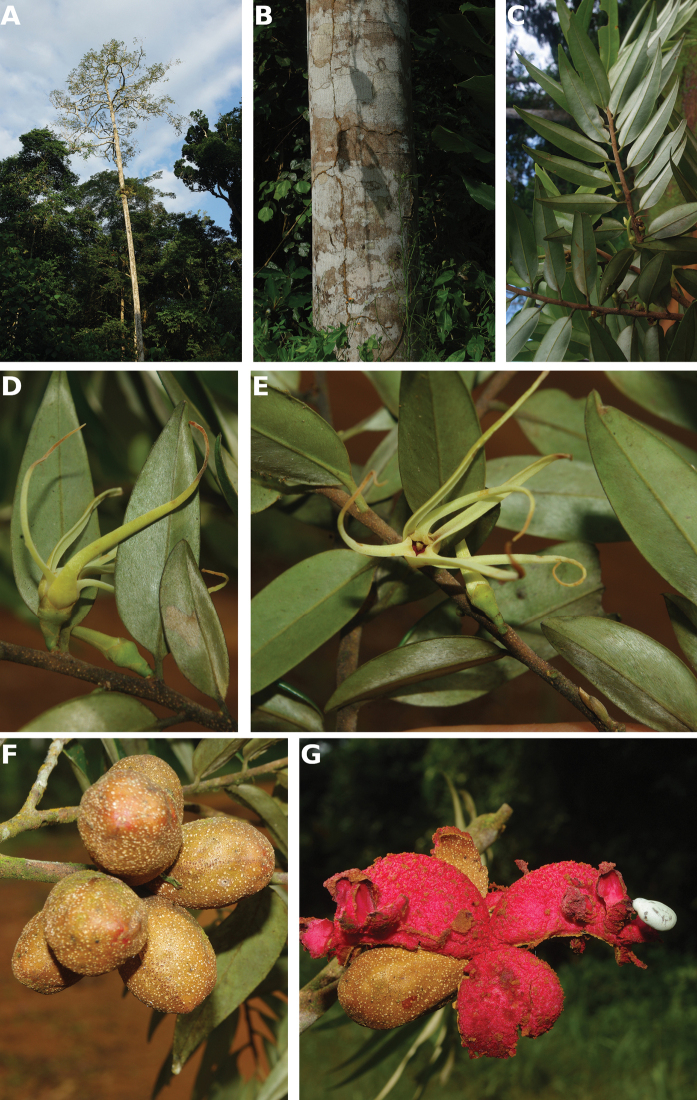
*Xylopiahypolampra***A** tree **B** detail of trunk, note light grey with patches of darker grey **C** flowering branch **D** flower, side view **E** flower, top view **F** immature fruit **G** mature fruit, opened, note seed covered in white sarcotesta **A***Couvreur 483*, Lélé, Cameroon **B–G***Couvreur 568*, Gabon. Photos Thomas L.P. Couvreur.

Figs 144 , 145 ; Map 17I

= Xylopia brieyi De Wild., Bull. Jard. Bot. État 4: 385, 1914. Type. Democratic Republic of the Congo. Kongo Central Province, Ganda-Sundi, *de Briey J. 108*, 8 Oct 1911: lectotype, designated by Johnson and Murray (2018), p. 169: BR[BR 0000008824844]; isolectotypes: BR[BR0000008824790, BR 0000008824806, BR 0000008824813, BR 0000008824837]; US[US1270066].

#### Type.

Cameroon. East Region; Lomié, *Mildbraed G.W.J. 5183*, 13 May 1911: lectotype, designated by Le Thomas (1969b), p. 181: HBG[HBG502479].

#### Description.

Tree, 30–40 m tall, d.b.h. up to 80 cm; stilt roots and buttresses absent. Old branches glabrous, young branches glabrous to pubescent, with fine matted hairs 0.2–0.4 mm long. Leaves: petiole 5 mm long, 1–2 mm wide, pubescent, grooved, blade inserted on the side of the petiole; **blade 5.7–7.5 cm long, 1.4–1.9 cm wide, lanceolate to elliptic**, apex acuminate to acute, acumen 1.2 cm long, base cuneate to rounded, subcoriaceous, **below densely silvery-sericeous when young and old**, above glabrous when young and old, discolorous; midrib sunken or flat, above glabrous when young and old, below densely pubescent when young and old; secondary veins 10 to 18 pairs, glabrous above; tertiary venation reticulate. Individuals bisexual; inflorescences ramiflorous on young foliate branches, axillary, peduncle absent. Flowers with 9 perianth parts in 3 whorls, 1 to 3 per inflorescence; pedicel 2–4 mm long, ca. 1 mm in diameter, pubescent; in fruit 3–6 mm long, 3–5 mm in diameter, glabrous; bracts 3 to 4, evenly spaced, ca. 2 mm long, 1–2 mm wide; sepals 3, valvate, basally fused, 2–3 mm long, 2–3 mm wide, triangular, apex acute, base truncate, green, pubescent outside, glabrous inside; petals free, subequal; outer petals 3, 25–28.7 mm long, 2.5–2.6 mm wide at base, linear, apex obtuse, base broad and concave, yellow to light green, pubescent with base glabrous to sericeous outside, pubescent and glabrous towards base inside; inner petals 3, valvate, 16–31 mm long, 1.9–2.5 mm wide at base, linear, apex acute, base broad and concave, yellow to light green, pubescent outside, pubescent inside; stamens ca. 100, in 7 to 8 rows, 1–2 mm long, oblong; connective apex shield-like, glabrous, red; carpels 7 to 12, ovary ca. 1 mm long, stigmas connivent, sometimes free at tips, filiform, 1.6–2.5 mm long, pubescent. Monocarps **sessile**; monocarps 6 to 8, 26–41 mm long, 11–22 mm wide, obovoid, ellipsoid or oblongoid, becoming slightly ridged and sunken between the ridges when dried, apex rounded, glabrous, greenish brown, **flecked with pale brown lenticels outside**, endocarp pink-red; seeds up to 10 per monocarp, **in two rows**, 7–11 mm long, 6–8 mm wide, ellipsoid; **sarcotesta greenish white *in vivo*; aril absent.**

#### Distribution.

Central Cameroon to southwestern Central African Republic and south to southwestern Democratic Republic of the Congo; in Cameroon known from East, South, Central and South-West regions.

#### Habitat.

A common species, in lowland or submontane evergreen or semi-deciduous rain forest, sometimes along forest edges, and in gallery forest. Altitude 400–900 m. a.s.l.

#### Local and common names known in Cameroon.

abiès (Bulu, *de Wilde 7963*), odjobi (*Foury 101*); moley, munjié, monjié, sangé, sangi (Bibaya); nom akwi, mvomba (Yaoundé).

#### IUCN conservation status.

Least Concern (LC) (Botanic Gardens Conservation International and IUCN SSC Global Tree Specialist Group 2019f).

#### Uses in Cameroon.

None reported.

#### Notes.

﻿﻿*Xylopiahypolampra* is easily distinguished from other species of ﻿*Xylopia* by its narrow, lanceolate to elliptic leaves that are distinctly silvery-sericeous below. The flower pedicels are short, the flowers appearing sessile in the leaf axils. The monocarps are sessile and when fresh are brown with pale brown lenticels. At maturity the monocarps open along 3 lines, and reflex strongly, showing the seeds covered with a greenish white sarcotesta on a red endocarp. Hornbills and monkeys feed on the fruits and seeds; at the Dja Faunal Reserve site in Cameroon hornbills were particularly effective in extending the seed shadow for individual trees (Holbrook and Smith 2000).

#### Specimens examined.

**Central Region**: Mefou proposed national park near Mefou town, 3.62°N, 11.58°E, *08 March 2004*, *Cheek M.* 11487 (K,YA); Méfou National Park just after Ape Action Africa center along road, 3.62°N, 11.58°E, *24 April 2013*, *Couvreur T.L.P.* 420 (WAG,YA); Ottotomo Forest Reserve 1 km after reserve base near small loggers road, 3.66°N, 11.28°E, *25 June 2013*, *Couvreur T.L.P.* 450 (WAG,YA); Yaoundé, 3.87°N, 11.52°E, *01 January 1935*, *Foury P.* 101 (OWU,P); Banlieu de Yaoundé sur une piste après la borne 9, 3.87°N, 11.52°E, *26 November 1959*, *Mpom B.* 362 (P,YA). **East Region**: 74 km south of Yokadouma 30 km after Ngato 15 km after river ALPICAM ‘base de vie’ then 40 km on forestry road starting 4 km before Maséa village, 3.16°N, 14.71°E, *04 March 2019*, *Couvreur T.L.P.* 1198 (MPU,WAG,YA); A 30 km au NE de Bange (km 75 route Yokadouma-Moloundou), 3.02°N, 15.12°E, *25 May 1963*, *Letouzey R.* 5139 (P,YA); entre Bidjum et Dscha-Posten, 3.15°N, 13.61°E, *13 May 1911*, *Mildbraed G.W.J.* 5183 (HBG); Deng Deng, 5.2°N, 13.52°E, *01 January 1914*, *Mildbraed G.W.J.* 8827 (BM,K). **South Region**: 21 km east from Lélé village, 2.27°N, 13.33°E, *07 September 2013*, *Couvreur T.L.P.* 467 (WAG,YA); Campo Ma’an National Park 11 km on trail from Ebinanemeyong village on road 7 km from Nyabessan to Campo town, 2.47°N, 10.33°E, *13 February 2015*, *Couvreur T.L.P.* 694 (WAG,YA); 17 km on the road from Ebolowa to Minkok, 2.81°N, 11.13°E, *06 February 1975*, *de Wilde J.J.F.E* 7963 (B,BR,K,MO,P,U,WAG,YA); Bipindi – Ebolowa, 3.00°N, 10.92°E, *01 December 1913*, *Mildbraed G.W.J.* 7618 (B,K); Mvie (Akom II), 2.88°N, 10.58°E, *28 January 1998*, *van der Burgt X.M.* 364 (P).

### ﻿﻿﻿ Xylopia katangensis

Taxon classificationPlantaeMagnolialesAnnonaceae

De Wild., Ann. Mus. Congo Belge, Bot. Sér. 4, Bot. 1(2): 32–33, 1902

0F0FD50C-1662-5FF8-B44A-D7C0A426D225

Map 18A

#### Type.

Democratic Republic of the Congo. Katanga; Lukafu, *Verdick E. 503*, May 1900: holotype: BR[BR0000024941556].

#### Description.

Tree, up to 13 m tall, d.b.h. 60 cm; **buttresses present.** Old branches glabrous, young branches sparsely pubescent, the hairs ca. 0.2 mm long. Leaves: petiole 4–9 mm long, ca. 1 mm wide, glabrous, slightly grooved, blade inserted on the side of the petiole; blade 7.1–9.4 cm long, 2.4–3.3 cm wide, lanceolate to lanceolate-elliptic, apex acuminate to acute, acumen 0.4–1.1 cm long, base cuneate, subcoriaceous, **below glabrous, rarely sparsely pubescent when young, glabrous when old, above glabrous when young and old**, slightly discolorous; midrib slightly raised or flat, above glabrous when young and old, below glabrous when young, glabrous when old; secondary veins 10 to 15 pairs, glabrous above; tertiary venation reticulate. Individuals bisexual; inflorescences ramiflorous on young foliate or old leafless branches, axillary, peduncle 1–3.5 mm long. Flowers with 9 perianth parts in 3 whorls, **1 to 12 per inflorescence**; pedicel 6–9 mm long, 0.5–1 mm in diameter, pubescent to sparsely pubescent; in fruit 8–14 mm long, 2–3 mm in diameter, glabrous; bracts 2 to 4, several basal and one upper towards the upper half of pedicel, bract 1–2 mm long, 1–2 mm wide; sepals 3, valvate, basally fused, 1–2 mm long, 2–3 mm wide, triangular to ovate, apex acute to obtuse, base truncate, pubescent outside, glabrous inside; petals free, subequal; **outer petals 3, 19–37 mm long, 2.7–3.2 mm wide at base, linear**, apex obtuse, base broad and concave, yellow-green and red at the base, sparsely pubescent outside, pubescent inside; inner petals 3, valvate, 11.4–29 mm long, 2.3–2.9 mm wide at base, linear and rigid, apex acute, base broad and concave, yellow-green and red at the base, pubescent outside, pubescent, base glabrous inside; stamens ca. 90, in 6 to 7 rows, 1 mm long, oblong; connective apex shield-like, glabrous; carpels 3 to 4, ovary 1 mm long, stigmas loosely connivent with tips free, linear, 2.5–4.4 mm long, glabrous. Monocarps stipitate, stipe ca. 4 mm long, 3–4 mm in diameter; monocarps 2 to 4, 19–34 mm long, 12–15 mm wide, oblongoid, apex obtuse, glabrous, weakly torulose, verrucose and longitudinally wrinkled when dried, green outside, endocarp red; seeds 1 to 5 per monocarp, **in a single or two irregular rows**, 9–11 mm long, 7–8 mm wide, ellipsoid; **sarcotesta gray or light green *in vivo*; aril absent.**

**Map 18.**
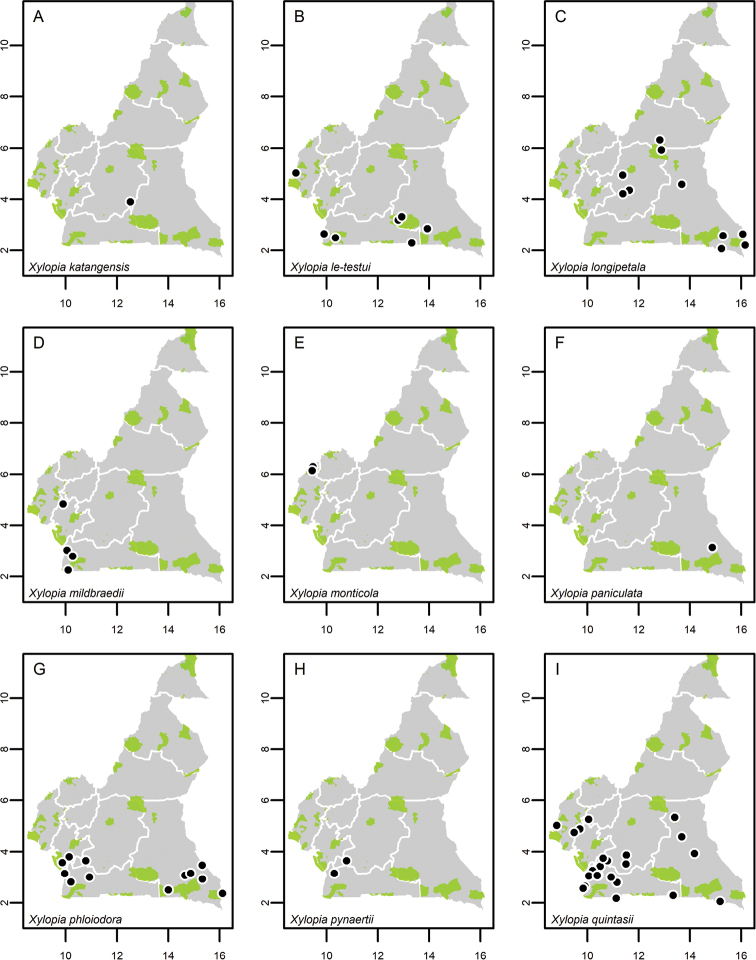
**A***Xylopiakatangensis***B***Xylopialetestui***C***Xylopialongipetala***D***Xylopiamildbraedii***E***Xylopiamonticola***F***Xylopiapaniculata***G***Xylopiaphloiodora***H***Xylopiapynaertii***I***Xylopiaquintasii*. White borders represent region limits in Cameroon; green patches represent protected areas (see methods and Suppl. material 1: Fig. S1).

#### Distribution.

From north-central Nigeria to southern Democratic Republic of the Congo and south to northeastern Zambia; in Cameroon known from a single collection in the Central region.

#### Habitat.

A very rare species in Cameroon, not re-collected since 1962; in riparian inundated habitats and swamp forests (the collection from Cameroon was made along the Nyong). Altitude 470 m a.s.l.

#### Local and common names known in Cameroon.

None recorded.

#### IUCN conservation status.

Least Concern (LC) (Harvey-Brown 2019k).

#### Uses in Cameroon.

None reported.

#### Notes.

In its exceedingly narrow petals and long stigmas ﻿﻿*Xylopiakatangensis* is most similar to ﻿*X.longipetala*, but has proportionately longer petioles, more flowers per inflorescence, and petals that are more rigid and narrower at the base.

#### Specimen examined.

**Central Region**: Près de Mbeuga (entre Ayos et Akonolinga), 3.9°N, 12.52°E, *08 March 1962*, *Letouzey R.* 4498 (P,YA).

### ﻿﻿﻿ Xylopia letestui

Taxon classificationPlantaeMagnolialesAnnonaceae

Pellegr., Bull. Mus. Natl. Hist. Nat. 26: 658, 1920

80360E17-7116-5A63-AB16-F628E2CDEB13

Fig. 146 ; Map 18B

= Xylopia letestui var. longepilosa Le Thomas, Fl. Gabon 16: 178, 1969. Type. Gabon. Ngounié, Moumba, Haute Ngounyé, *Le Testu G.M.P.C. 6046*, 3 Sep 1926: holotype: P[00169154]; isotypes: BM[000511049]; BR[0000008825315]; P[00169153, 00169155].

#### Type.

Gabon. Nyanga; Mayombe Bayaka, Tono-Sanga *Le Testu G.M.P.C. 1760*, 9 Aug 1914: holotype: P[P00169125]; isotypes: BM; K[K000199054]; BR[BR0000008825322]; EA[EA000002492]; LISC[LISC000403]; P[P00169126, P00169127].

#### Description.

Tree, up to 40 m tall, d.b.h. up to 30 cm; **buttresses present, narrow and thin.** Old branches sparsely pubescent to glabrous, **young branches densely pubescent**, the hairs 0.4–1 mm long, **branches sinuous or zigzagging**. Leaves: **petiole 1–2 mm long**, ca. 2 mm wide, pubescent, grooved, blade inserted on top of the petiole; blade 4.7–10.9 cm long, 1.2–2.7 cm wide, lanceolate to lanceolate-oblong, apex acute, **base truncate and often slightly asymmetrical**, subcoriaceous, below densely pubescent when young, sparsely pubescent to densely pubescent when old, the hairs dull grayish brown, above glabrous when young and old, discolorous; midrib flat to slightly raised, above densely pubescent when young, glabrous to densely pubescent when old, below densely appressed-pubescent when young, sparsely appressed-pubescent to densely appressed-pubescent when old; secondary veins 8 to 14 pairs, glabrous above; tertiary venation reticulate. Individuals bisexual; inflorescences ramiflorous on young foliate branches, axillary, peduncle ca. 2.5 mm long. Flowers with 9 perianth parts in 3 whorls, 1 to 4 per inflorescence; pedicel 1–7 mm long, ca. 1 mm in diameter, pubescent; in fruit 8–14 mm long, 2–3 mm in diameter, glabrous; bracts 2, evenly spaced, 2–6 mm long, 2–6 mm wide; sepals 3, valvate, basally to 2/3 fused, forming a cup, 4–6 mm long, ca. 3 mm wide, elliptic to ovate, apex acute to obtuse, base truncate, pubescent outside, glabrous inside; petals free, subequal; outer petals 3, 13–22 mm long, 2.3–3.2 mm wide at base, lanceolate-ligulate, linear-lanceolate, or narrowly triangular, elliptic, apex acute, base broad and concave, cream-colored with a purple blotch at the base, pubescent, base glabrous to densely pubescent outside, pubescent towards margins inside; inner petals 3, valvate, **10.3–18.1 mm long, 2.4–3.2 mm wide at base**, linear-lanceolate, apex acute, base broad and concave **with conspicuous hairy tufts on the margins**, cream, densely pubescent outside, pubescent, and glabrous towards the base inside; stamens numerous, in 5 to 6 rows, 1–2 mm long, oblong; connective apex shield-like, glabrous; carpels 7 to 10, ovary 1 mm long, stigmas loosely connivent with tips free, linear, sinuate, 3–3.5 mm long, pubescent. Monocarps **sessile**; monocarps 4 to 7, 27–45 mm long, 21–34 mm wide, nearly spherical to ellipsoid, apex rounded or obtuse, pubescent, smooth to verrucose, wrinkled when dried, green outside, endocarp bright red; seeds up to 6 per monocarp, **in two rows**, 8–19 mm long, 8–13 mm wide, ellipsoid to flattened ellipsoid; **sarcotesta grayish white *in vivo*; aril absent.**

#### Distribution.

A widespread species with a disjunct distribution between West Africa, from Sierra Leone to Ghana, and Central Africa from eastern Nigeria to western Democratic Republic of the Congo; in Cameroon known from the East, South and South-West regions.

#### Habitat.

A fairly common species, especially visible as a young tree in the understory; in lowland primary and more rarely secondary rain forests, on both clay and well-drained sandy soil, Altitude 50–450 m a.s.l.

#### Local and common names known in Cameroon.

None recorded.

#### IUCN conservation status.

Least Concern (LC) (Harvey-Brown 2019l).

#### Uses in Cameroon.

None reported.

#### Notes.

﻿﻿*Xylopialetestui* is easily distinguished even when sterile by its nearly sessile and densely pubescent leaves with truncate and often slightly asymmetrical bases. In addition, the inner petals are short and bear conspicuous hairy tufts on the margins toward the base. It is similar to ﻿*X.villosa*, but the lower leaf indument is not shiny and there are fewer flowers in the inflorescences. The sinuous young shoots with conduplicate new leaves sometimes seen in ﻿*X.letestui* have not been noted in specimens of ﻿*X.villosa*.

**Figure 146.**
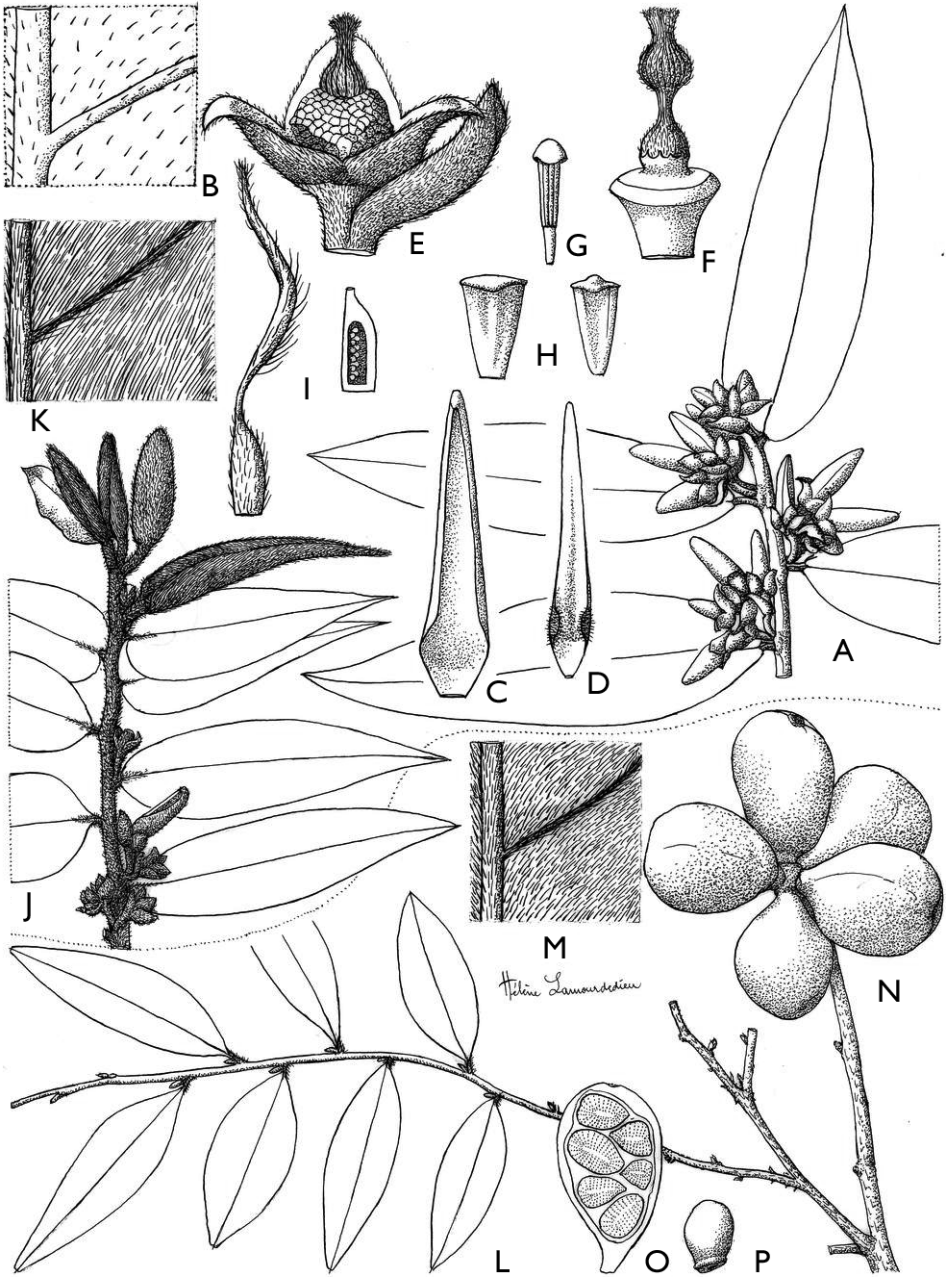
*Xylopialetestui***A** flowering branch **B** detail of pubescence on lower side of leaves **C** outer petal, inner view **D** inner petal, inner view **E** receptacle, petals removed **F** detail of carpels, stamens and petals removed **G** stamen, front view **H** staminodes, inner and outer whorl **I** carpel, side view and longitudinal section showing ovules **J** flowering branch of the more densely pubescent specimen (see notes under this species) **K** detail of dense pubescence on lower side of leaf. *Xylopiapynaertii***L** leaves **M** detail of pubescence on lower side of leaves **N** fruit **O** longitudinal section of monocarp **P** seed, side view **A–I** from *Le Testu 5975***J, K** from *Hallé & Le Thomas 122***L–P** from *Le Thomas 23*. Drawings by Hélène Lamourdedieu, Publications Scientifiques du Muséum national d’Histoire naturelle, Paris; modified from Le Thomas (1969b; pl. 33, p. 179).

#### Specimens examined.

**East Region**: Dja Reserve (Réserve de Faune du Dja) Bouamir Research Area 90 km southeast of Akonolinga, 3.18°N, 12.79°E, *23 May 1997*, *Fogiel M.K.* 2098 (MO,WAG); Entre Kondon I et Mbalam près Ngoila (axe Lomié Souanké), 2.85°N, 13.93°E, *22 December 1972*, *Letouzey R.* 11707 (P,YA); A 15 km au Sud de Djouo (20 km E de Somalomo sur le Dja), 3.32°N, 12.93°E, *23 February 1962*, *Letouzey R.* 4361 (P,YA). **South Region**: 24 km east from Lélé village, 2.28°N, 13.31°E, *08 September 2013*, *Couvreur T.L.P.* 475 (WAG,YA); Campo Ma’an National Park 11 km on trail from Ebinanemeyong village on road 7 km from Nyabessan to Campo town, 2.49°N, 10.34°E, *12 February 2015*, *Couvreur T.L.P.* 688 (WAG,YA); Sud Cameroun TDC, 2.65°N, 9.9°E, *15 November 1991*, *Hallé F.* 4247 (P,WAG). **South-West Region**: Korup National Park P transect P plot subplot 4E, 5.01°N, 8.8°E, *24 February 2008*, *van der Burgt X.M.* 1129 (BR,G,K,MO,P,WAG,YA).

### ﻿﻿﻿ Xylopia longipetala

Taxon classificationPlantaeMagnolialesAnnonaceae

De Wild. & T. Durand, Ann. Mus. Congo Belge, Bot. Sér. 2, Bot. 1(1): 4, 1899

F6C9D657-3A39-5AF6-80E9-B850AE50AB8F

Fig. 147 ; Map 18C

= ﻿﻿Uvaria parviflora A.Rich., in Guillemin, Perrottet, & A. Richard, Fl. Senegamb. tent., part 1: 9, 1831; ﻿﻿Coelocline? parviflora ( A.Rich.,) A.DC., Mém. Soc. Phys. Genève 5: 209, 1832; ﻿Unonaparviflora ( A.Rich.) Steudel, Nomencl. Bot., ed. 2, 2: 730, 1841; ﻿﻿Xylopiaparviflora ( A.Rich.) Benth., Trans. Linn. Soc. 23: 479, 1862, non ﻿X.parviflora Spruce; Xylopicrumparviflorum (A. Richard) Kuntze, Revis. gen. pl. 1: 8. 1891; Xylopiavallotii Chipp ex Hutchinson & Dalziel, Fl. W. Trop. Afr. 1(1): 53. Mar 1927. Type. Senegal. Ziguinchor Region, *Perrottet G.G.-S. s. n.*, 3 or 4 Apr 1829: lectotype, designated by Johnson and Murray (2018), p. 180: P[P00169145]; isotypes: B[B10 0273361, probable]; BM[BM000511054]; G[G00190717]; P[P00169144, plus 4 additional sheets lacking bar codes].

#### Type.

Democratic Republic of the Congo. Equateur; Bangala, *Dewèvre A.P. 876*, May 1896: holotype: BR[BR0000008825360]; isotype: BR[BR0000008825377].

#### Description.

Tree to shrub, up to 15 m tall, d.b.h. up to 40 cm; stilt roots and buttresses absent. Old branches glabrous, young branches glabrous to sparsely pubescent with erect hairs 0.1–0.5 mm long. Leaves: petiole 3–6 mm long, ca. 1 mm wide, pubescent to glabrous, slightly grooved, blade inserted on the side of the petiole; blade 4.2–8.8 cm long, 1.7–3.7 cm wide, oblong or elliptic, occasionally lanceolate, lanceolate-ovate, or ovate-oblong, apex acuminate to obtuse, acumen 0.4–0.8 cm long, base rounded, papyraceous to subcoriaceous, below sparsely pubescent when young, sparsely pubescent to glabrous when old, above glabrous when young and old, slightly discolorous; midrib sunken, above sparsely pubescent when young, glabrous to sparsely pubescent when old, below sparsely pubescent when young, sparsely pubescent when old; secondary veins 9 to 14 pairs, glabrous above; tertiary venation reticulate. Individuals bisexual; inflorescences ramiflorous on young foliate branches, axillary, peduncle 1–1.5 mm long. Flowers with 9 perianth parts in 3 whorls, 1 to 4 per inflorescence; peduncle 1–1.5 mm long; pedicel 2–3 mm long, ca. 2 mm in diameter, sparsely pubescent to glabrous; in fruit 10–17 mm long, 7–11 mm in diameter, sparsely pubescent to glabrous; bracts 2 to 3, towards the middle of pedicel, 1–2 mm long, 1–2 mm wide; sepals 3, valvate, basally fused, **reflexed at anthesis**, 2–3 mm long, ca. 2 mm wide, triangular, apex acute, base truncate, pubescent outside, glabrous inside; petals free, outer petals longer than inner; **outer petals 3, 16–62 mm long, 2.7–5 mm wide at base, linear, lax and ribbonlike**, apex obtuse, base broad and concave, pale yellow to yellow-green with purple to red base, densely pubescent outside, sparsely pubescent inside; inner petals 3, valvate, 19–48 mm long, 3.1–5.2 mm wide at base, linear, lax and ribbonlike, crinkled when dried, apex acute to obtuse, base broad and concave, yellow-green with red base, pubescent at least towards base outside, glabrous to sparsely pubescent inside, densely hairy in the basal concavity; stamens 80 to 100, in 5 to 6 rows, 1–2 mm long, oblong; connective apex shield-like, glabrous; carpels 5 to 7, ovary 1 mm long, stigmas loosely connivent with tips free, linear and sometimes falcate, 3.8–7 mm long, pubescent to glabrous. Monocarps stipitate, stipe 1–5 mm long, 3–8 mm in diameter; monocarps 3 to 6, 30–44 mm long, 11–19 mm wide, oblongoid, apex rounded or with a beak ca. 2 mm long, glabrous, **longitudinally ridged**, verrucose and wrinkled when dried, green, sometimes purple or red tinged outside, endocarp bright red; seeds 7 to 12 per monocarp, **in two rows**, 10–12 mm long, 6–9 mm wide, ellipsoid; **sarcotesta white to green *in vivo*; aril absent.**

**Figure 147.**
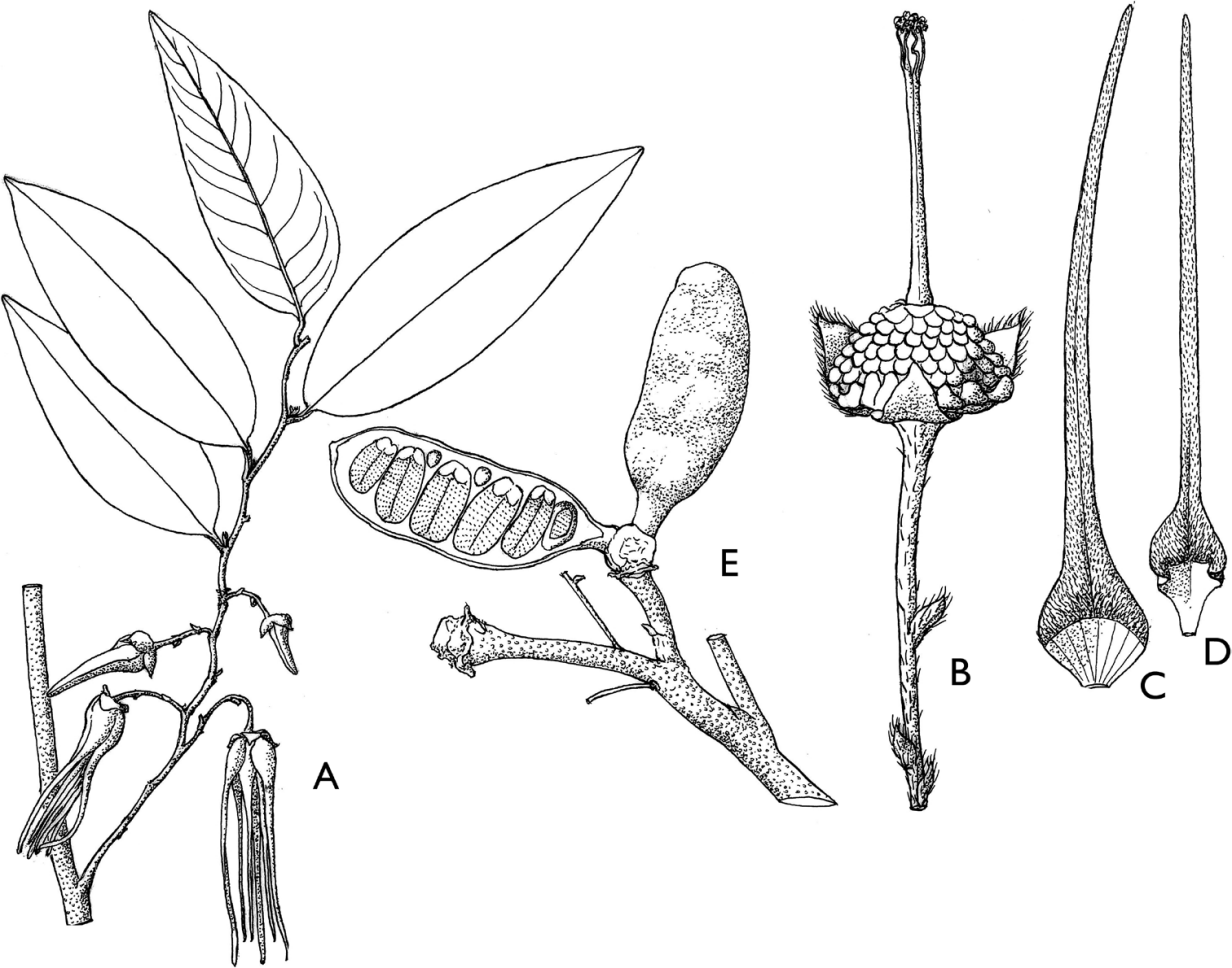
*Xylopialongipetala***A** flowering branch **B** flower with all petals removed **C** outer petal, inner side **D** inner petal, inner side **E** fruiting branch, longitudinal section of one monocarp **A–D** from *Pobéguin 48***E** from *Chevalier 28389*. Drawings by Hélène Lamourdedieu, Publications Scientifiques du Muséum national d’Histoire naturelle, Paris; modified from Le Thomas (1969b; pl. 31, p. 171; 1–5).

#### Distribution.

Occurs from Senegal to southern Chad and south to northern Angola and the northeastern Democratic Republic of the Congo; in Cameroon known from East, Central and Adamaoua regions.

#### Habitat.

A species of inundated riparian forests, sometimes on sandy soils. Altitude 30–700 m a.s.l.

#### Local and common names known in Cameroon.

None recorded.

#### IUCN conservation status.

Not evaluated yet.

#### Uses in Cameroon.

None reported.

#### Notes.

﻿﻿*Xylopialongipetala* was long known by the name ﻿*X.parviflora* (A. Rich.) Benth, which is however an illegitimate later homonym of ﻿*X.parviflora* Spruce, a South American species. The reflexed sepals, lax ribbonlike petals, and ridged monocarps distinguish it from ﻿*X.katangensis*.

#### Specimens examined.

**Adamaoua Region**: Rives du Djerem près Mbakaou, 6.32°N, 12.82°E, *08 December 1959*, *Letouzey R.* 2459 (BR,K,P,YA). **Central Region**: left bank Sanaga River near ferry Nachtigal ca 20 km N of Obala, 4.35°N, 11.63°E, *11 June 1964*, *de Wilde W.J.J.O* 2676 (B,BR,K,MO,P,WAG); Left bank Sanaga R near Ferry Nachtigal ca 20 km N of Obala, 4.21°N, 11.38°E, *19 November 1965*, *Leeuwenberg A.J.M.* 7033 (B,BR,C,GC,K,LUAI,MO,P,UC,WAG,YA); Yangafok II 25 km ENE de Bafia, 4.93°N, 11.37°E, *26 November 1969*, *Letouzey R.* 9614 (P,YA). **East Region**: On island in the Sangha River adjacent to the Ndakan gorilla study site, 2.20°N, 16.15°E, *13 March 1988*, *Fay J.M.* 8307 (MO,WAG); Eastern Province W bank of Sangha River, 2.63°N, 16.06°E, *22 May 1988*, *Harris D.J.* 757 (K); Rives du Dja près Ndongo à 40 km WNW de Moloundou, 2.58°N, 15.29°E, *18 March 1973*, *Letouzey R.* 12138 (K,P,YA); Djerem R (Niadaba), 5.93°N, 12.88°E, *27 June 1959*, *Letouzey R.* 2261 (P,YA); Bertoua-Batouri, 4.58°N, 13.68°E, *01 January 1962*, *Tchinaye V.* 103 (P,YA); 7 km NW du confluent Boumba Dja Ngoba, 2.07°N, 15.23°E, *17 April 1971*, *Villiers J.-F.* 666 (P).

### ﻿﻿﻿ Xylopia mildbraedii

Taxon classificationPlantaeMagnolialesAnnonaceae

Diels, Bot. Jahrb. Syst. 53: 444, 1915

77ADAE28-B238-5007-8321-820D10F83D48

Fig. 148 ; Map 18D

= Xylopia lastoursvillii Pellegr., Mém. Soc. Bot. France 1949: 71, 1950. Type. Gabon. Ogooué-Lolo, région de Lastoursville, Koulamotou, *Le Testu G.M.P.C. 8742*, 13 Apr 1931: lectotype, designated by Johnson and Murray (2018), p. 188: P[P00169128]; isolectotypes: BM[BM000511051]; BR[BR0000008825308]; LISC[000404]; OWU; P[P00169129, P00169130]; WAG[WAG0247282, WAG0247283].

#### Type.

Cameroon. South Region; 45 km west of Grand Batanga, *Mildbraed G.W.J. 6055*, 22 Jul 1911: holotype: B[B 10 0153148]; isotypes: HBG[HBG502478, HBG502477].

#### Description.

Tree up to 9 m tall, rarely a shrub, d.b.h. up to 9 cm; stilt roots and buttresses absent. Old branches glabrous, **young branches pubescent with appressede hairs 0.1–0.5 mm long**. Leaves: petiole 2–5 mm long, 1 mm wide, sparsely pubescent, grooved, blade inserted on the side of the petiole; blade 9.5–17.5 cm long, 3.2–5.6 cm wide, elliptic to oblong, occasionally oblanceolate or lanceolate, apex acuminate, acumen 0.8–1.9 cm long, base cuneate, papyraceous, below pubescent when young, glabrous to sparsely pubescent when old, above glabrous when young and old, discolorous but sometimes concolorous; midrib sunken, above pubescent when young and old, below pubescent when young, glabrous when old; secondary veins 12 to 20 pairs, glabrous above; tertiary venation reticulate. Individuals bisexual; inflorescences ramiflorous on young foliate branches, axillary, peduncle absent. Flowers with 9 perianth parts in 3 whorls, 1 to 2 per inflorescence; pedicel 4–9 mm long, 1–2 mm in diameter, pubescent; in fruit 5–7 mm long, 5–7 mm in diameter, glabrous; bracts 3 to 4, evenly spaced, 2–4 mm long, 2–3 mm wide; sepals 3, valvate, basally fused, 4–7 mm long, 4–5 mm wide, triangular, apex acute, base truncate, pubescent outside, glabrous inside; **petals free, subequal; outer petals 3, 45–79 mm long, 3.6–5.5 mm wide at base**, linear, apex obtuse, base broad and concave, cream, pubescent outside, pubescent towards base inside; inner petals 3, valvate, 35–61 mm long, 4.9–5.4 mm wide at base, linear, apex acute, base broad and concave **with a tuft of long hairs at the apex of the concavity**, cream, pubescent outside, pubescent inside; stamens ca. 100, in 5 to 6 rows, 1–2 mm long, clavate to oblong; connective apex shield-like, glabrous; carpels 10 to 11, ovary 1–2 mm long, stigmas connivent, linear, 4.5–6 mm long, pubescent. Monocarps stipitate, stipe 9–15 mm long, 3–5 mm in diameter; monocarps 1 to 14, 42–65 mm long, 13–15 mm wide, oblong, apex with a curved beak ca. 1 mm long, glabrous to sparsely pubescent, verrucose and wrinkled when dried, pale green to yellowish green outside, endocarp red to pink-red; seeds 5 to 9 per monocarp, **in a single row, 13–14 mm long, 7–9 mm wide**, ellipsoid; **sarcotesta greenish blue, greenish white, or gray; aril absent.**

#### Distribution.

From Cameroon to Gabon; in Cameroon known from the South and Littoral regions.

#### Habitat.

A rare and rarely collected species; in primary and older secondary rain forest Altitude 100–520 m. a.s.l.

#### Local and common names known in Cameroon.

None recorded.

#### IUCN conservation status.

Vulnerable (VU) (Cosiaux et al. 2019ba).

#### Uses in Cameroon.

None reported.

#### Notes.

The petal length of ﻿*X.mildbraedii* surpasses that of all other African ﻿*Xylopia* species. The species most resembles ﻿﻿*X.thomsonii* in overall appearance, but differs in the hairs of the young twigs which are appressed and relatively short, the pronounced tuft of hairs on the inside of the inner petal base, and the distinctly larger seeds.

#### Specimens examined.

**Littoral Region**: Forêt de Bakaka 3 km E of Eboné a village on km 11 Nkongsamba-Loum Road, 4.83°N, 9.9°E, *13 September 1971*, *Leeuwenberg A.J.M.* 8362 (BR,MO,P,WAG,YA). **South Region**: 20 km from Kribi Lolodorf road, 3.03°N, 10.05°E, *09 June 1969*, *Bos J.J.* 4772 (B,BR,K,LD,LM,MO,P,WAG,YA); Campo-Ma’an area Bibabimvoto, 2.25°N, 10.09°E, *01 February 2000*, *Elad M.* 1253 (KRIBI,WAG); Beson 45 km E Kribi, 2.79°N, 10.27°E, *22 July 1911*, *Mildbraed G.W.J.* 6055 (B,HBG).

**Figure 148.**
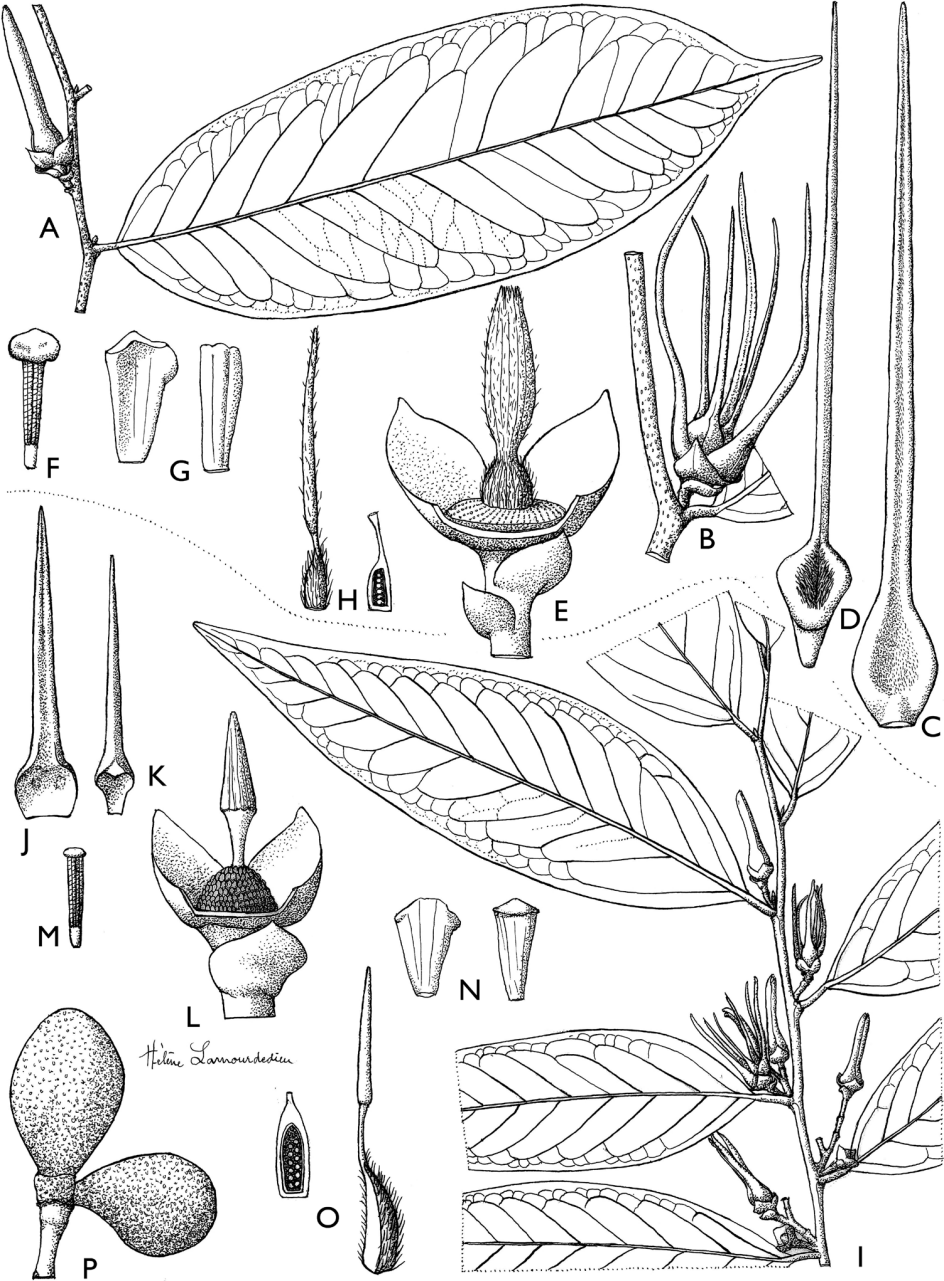
*Xylopiamildbraedii***A** flowering branch **B** flower **C** outer petal, inner view **D** inner petal, inner view **E** detail of carpels, petals and stamens removed **F** stamen, front view **G** staminode, inner and outer whorl **H** carpel, side view and detail of ovules. *Xylopiaphloiodora***I** flowering branch **J** outer petal, inner view **K** inner petal, inner view **L** detail of carpels, petals and stamens removed **M** stamen, front view **N** staminode, inner and outer whorl **O** carpel, side view and detail of ovules **P** fruit **A** from *Mildbread 6055***B–H** from *Le Testu 8742***I–P** from *Letouzey 5027*. Drawings by Hélène Lamourdedieu, Publications Scientifiques du Muséum national d’Histoire naturelle, Paris; modified from Le Thomas (1969b; pl. 32, p. 173).

### ﻿﻿ Xylopia monticola

Taxon classificationPlantaeMagnolialesAnnonaceae

D. M. Johnson & N. A. Murray, PhytoKeys 97: 190–193. 2018

39F388CB-97F0-59D8-BA31-591DC758C59E

Map 18E

#### Type.

Nigeria. Taraba [“N. E. State”] State, Sardauna Province, Kurmin Kugapa, below Kurmin Dodo below the western edge of Cabbal Wade [“Chappal Waddi”], ca. 5500 ft, 28 Feb 1975, *Chapman J.D. 3755* Holotype: K.

#### Description.

Tree up to 10 m (–20 m) tall, d.b.h. unknown; stilt roots and buttresses absent. Old branches glabrate, **young branches pubescent with erect dull gray to brown hairs 0.1–1.2 mm long**. Leaves: petiole 3.5–9 mm long, pubescent, grooved; blades 7.8–10.9 cm long, 2.7–5.1 cm wide, lanceolate, lanceolate-oblong, elliptic to oblong or oblong-oblanceolate, **apex blunt-acuminate**, acumen 0.4–1.6 cm long, base broadly cuneate to nearly rounded, papyraceous to subcoriaceous, below sparsely pubescent to glabrate, above glabrous, concolorous; midrib flat, above pubescent, below pubescent; secondary veins 7–13 pairs; **tertiary venation conspicuously reticulate**. Individuals bisexual; inflorescences ramiflorous on young foliate branches, axillary, peduncle absent. Flowers with 9 perianth parts in 3 whorls, 1 per inflorescence; pedicel 5.2–8.1 mm long, 1–1.3 mm in diameter; in fruit 6–8.7 mm long, 1.5–2.8 mm thick, sparsely pubescent, sometimes with bracts or sepals persistent; **bracts 3–4**, evenly spaced, 2–3.1 mm long, ovate; sepals 3, valvate, basally fused, 2.7–3.5 mm long, 2.9–3.1 mm wide, triangu­lar to ovate, apex acute, pubescent outside; **outer petals 3, (15–) 36–52 mm long**, 2.7–3.6 mm wide at base, linear, apex acute, base concave, cream, pubescent inside, sericeous outside; inner petals 3, valvate, (15.5–) 29–37 mm long, 2–2.6 mm wide at base, linear, apex obtuse, base concave, cream, pubescent on both surfaces except for glabrous base; stamens 160–200, 1.8–2.2 mm long, narrowly oblong, connective apex shield-like, glabrous; carpels 7 to 8, ovary 1.2–1.3 mm long, stigma loosely connivent, linear, 2–3.4 mm long, glabrous except for apical hair tuft. Monocarps stipitate, stipe 8–13 mm long, 2.1–2.6 mm in diameter, monocarps 4 to 8, 34–40 mm long, 6–10 mm wide, narrowly oblong and slightly falciform, torulose, apex obtuse or with a beak 0.5–2 mm long, glabrate, verrucose, green outside, endocarp red; seeds up to 5 per monocarp, **in a single row**, 11–13.1 mm long, 5.8–6.5 mm wide, oblongoid; **sarcotesta “glaucous”; aril absent.**

#### Distribution.

Easternmost Nigeria and adjoining Cameroon (South-West region).

#### Habitat.

A rare species; in gallery forest along streams and in understory of lower montane forests. Altitude 650–1670 m a.s.l.

#### Local and common names known in Cameroon.

None recorded.

#### IUCN conservation status.

Vulnerable (VU) (Cosiaux et al. 2019bb).

#### Uses in Cameroon.

None reported.

#### Notes.

﻿*Xylopiamonticola* is similar to ﻿﻿*X.thomsonii* in having 1-flowered inflorescences and multiple (3 to 4) bracts, but may be distinguished by the more conspicuous abaxial vein reticulum of the leaves, the longer outer petals, and the relatively small monocarps with a proportionally longer stipe. ﻿*Xylopiamonticola* is always a tree, while ﻿﻿*X.thomsonii* is usually a scandent shrub and only rarely an upright tree. ﻿*Xylopiamonticola* seems to be restricted to higher elevations than ﻿﻿*X.thomsonii* but closer comparison of the two taxa in the South-West Region and adjoining areas is needed.

#### Specimens examined.

**South-West Region**: Near Aguosho 10 km SSW of Akwaya, 6.3°N, 9.466°E, *20 March 1985*, *Thomas D.W.* 4558 (MO); Takamanda Forest Reserve along footpath from Malishi to Kalu 6.15°N, 9.433°E, *01 May 1987*, *Thomas D.W.* 7400 (B,K,MO).

### ﻿﻿﻿ Xylopia paniculata

Taxon classificationPlantaeMagnolialesAnnonaceae

Exell, J. Bot. 64 (Suppl.): 8, 1926

97BF1E57-88C7-594B-9B39-1C5448CCB02F

Map 18F

#### Type.

Angola. Cabinda; Belize, Mayumbe, *Gossweiler J. 6988*, no date: holotype: BM; isotypes: COI[COI00004886]; LISC[LISC000323, LISC000324, LISC000322, LISC000321]

#### Description.

Tree, up to 35 m tall, d.b.h. up 35 cm; **stilt roots or small buttresses present.** Old branches glabrous, young branches glabrous to pubescent with loosely appressed hairs 0.4–0.9 mm long. Leaves: petiole 2–3 mm long, 1 mm wide, pubescent, grooved, blade inserted on the side of the petiole; blade 7.2–11.5 cm long, 1.8–2.7 cm wide, narrowly oblong to elliptic, apex acuminate, acumen 0.2–0.7 cm long, base decurrent to cuneate, papyraceous to subcoriaceous, below sparsely pubescent when young, sparsely pubescent when old, above glabrous when young and old, somewhat shiny above, slightly discolorous; midrib raised to slightly sunken, above sparsely pubescent when young, glabrous when old, below pubescent when young, sparsely pubescent when old; secondary veins 8 to 16 pairs, glabrous above; tertiary venation reticulate. Individuals bisexual; inflorescences ramiflorous on young foliate branches, axillary, **peduncle 2–3 per axil, highly branched, 1–4.5 mm long, sometimes with a longer floriferous axis emerging from among the cluster of flowers in an axil.** Flowers with 9 perianth parts in 3 whorls, up to 32 per inflorescence; pedicel 2–3 mm long, ca. 1 mm in diameter, pubescent; in fruit 23–30 mm long, 2–3 mm in diameter, sparsely pubescent; bracts 1 or 2, at or above the middle of pedicel, 2–3 mm long, ca. 1 mm wide; sepals 3, valvate, basally fused, 2–3 mm long, 2–3 mm wide, ovate, apex acute to obtuse, base truncate, pubescent outside, glabrous inside; petals free, inner and outer whorl subequal; outer petals 3, 10–19.7 mm long, 2.3–3.6 mm wide at base, linear-lanceolate, apex obtuse, base broad and concave, yellow-green with red base, densely pubescent outside, pubescent and glabrous towards center inside; inner petals 3, valvate, 9.7–13.1 mm long, 2.2–2.5 mm wide at base, linear, apex acute, base broad and concave, yellow-green with red base, pubescent, base glabrous outside, pubescent, base glabrous inside; stamens 120 to 130, in 5 to 6 rows, 1–2 mm long, narrowly oblong; connective apex capitate, glabrous; carpels 3 to 6, ovary ca. 1 mm long, stigmas connivent, filiform, 2.5–2.7 mm long, sparsely pubescent. Monocarp stipitate, stipe 2–3 mm long, 2–3 mm in diameter; **monocarp 1**, 56–85 mm long, 31–40 mm wide, obovoid to oblongoid, apex rounded, pubescent, longitudinally ridged and wrinkled when dried, green outside, endocarp carmine, red or pink-red; seeds 3 to 4 per monocarp, **in a single row**, 21–22 mm long, 16–17 mm wide, flattened ellipsoid; **sarcotesta grayish blue or greenish blue *in vivo*; aril absent.**

#### Distribution.

A sparsely distributed species from Cameroon to Angola (Cabinda); in Cameroon known from a single collection in the East region.

#### Habitat.

A very rare species in general; in primary rain forests. Altitude unknown, but probably lowland.

#### Local and common names known in Cameroon.

None recorded.

#### IUCN conservation status.

Endangered (EN) (Cosiaux et al. 2019bc).

#### Uses in Cameroon.

None reported.

#### Notes.

﻿﻿*Xylopiapaniculata* is distinguished by its highly branched inflorescences with up to 32 flowers, a unique characteristic for African species. The monocarps and seeds are among the largest of any African ﻿*Xylopia* species. The specimen label of the single Cameroon specimen lists *Baillonellatoxisperma* and *Pentaclethramacrophylla* as associated species.

#### Specimen.

**East Region**: A 23 km à l’Ouest de Maséa (village situé à 50 km au SSW de Yokadouma, 3.15°N, 14.87°E, *04 July 1963*, *Letouzey R.* 5402 (P,YA).

### ﻿﻿﻿ Xylopia phloiodora

Taxon classificationPlantaeMagnolialesAnnonaceae

Mildbr., Notizbl. Bot. Gart. Berlin-Dahlem 8: 55, 1921

CD053639-47F4-5470-A5F6-FFDE900DC940

**Figure 149.**
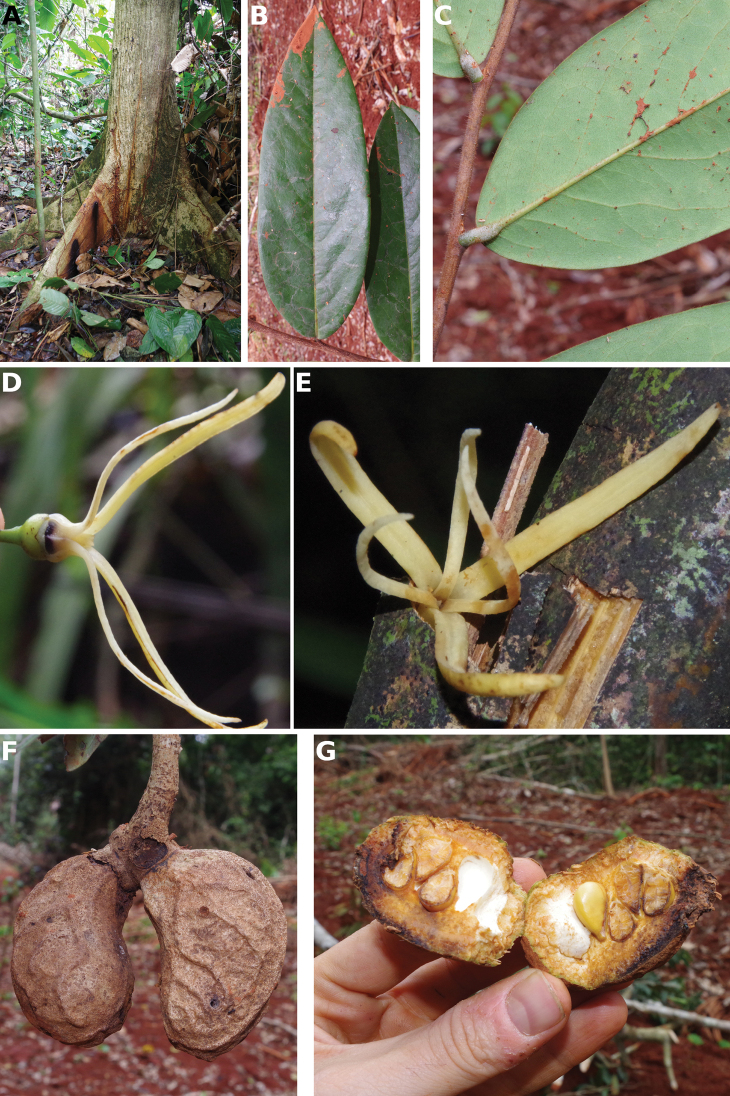
*Xylopiaphloiodora***A** base of trunk, note buttresses **B** leaf, upper side **C** base of leaf blade, lower side **D** flower, side view, 1 outer petal removed **E** flower, top view **F** fruit **G** monocarp opened, immature, the sarcostesta will turn orange at maturity **A–E***Couvreur 1164*, Kribi, Cameroon **F, G***Couvreur 1222*, Maséa, Cameroon. Photos Thomas L.P. Couvreur.

Figs 148 , 149 ; Map 18G

#### Type.

Cameroon. South Region; between Bipindi and Ebolowa near Malakat, *Mildbraed G.W.J. 7592*, Dec 1913: lectotype, designated by Le Thomas (1969b), p. 185: B[B 10 0153152]; isolectotype: K[K000199053].

#### Description.

Tree, 30–35 m tall, d.b.h. up to 80 cm; **buttresses present, up to 1.5 m high, stilt roots rarely present**. Old branches glabrous, young branches glabrous to densely pubescent with appressed hairs 0.1–0.4 mm long. Leaves: petiole 4–8 mm long, ca. 2 mm wide, pubescent, grooved, blade inserted on the side of the petiole; blade 5.7–17.2 cm long, 1.9–5.9 cm wide, elliptic, oblong-elliptic or lanceolate, apex acuminate to obtuse, acumen 0.5–1.0 cm long, base cuneate to subcordate, subcoriaceous, below sparsely pubescent when young, sparsely pubescent when old, above glabrous when young and old, concolorous or slightly discolorous; midrib sunken, above glabrous when young, glabrous to sparsely pubescent when old, below pubescent when young, sparsely pubescent when old; secondary veins 10 to 16 pairs, glabrous above; tertiary venation reticulate. Individuals bisexual; inflorescences ramiflorous on young foliate or old leafless branches, axillary, peduncle 2–4 mm long. Flowers with 9 perianth parts in 3 whorls, **1 to 10 per inflorescence**; pedicel 3–6 mm long, ca. 1 mm in diameter, pubescent; in fruit 5–16 mm long, 5–8 mm in diameter, glabrous; bracts 2, towards or above the middle of pedicel, 2–3 mm long, 2–3 mm wide; sepals 3, valvate, **basally to 2/3 fused, forming a cup**, 2–4 mm long, 3 mm wide, triangular to ovate, apex acute to obtuse, base truncate, pubescent outside, glabrous inside; petals free, subequal; outer petals 3, 19–23 mm long, 3–4.3 mm wide at base, linear-lanceolate, apex obtuse, base broad and concave, cream-colored or pale yellow with a purple blotch at the base, sericeous outside, pubescent with base glabrous inside; inner petals 3, valvate, 12.5–21 mm long, 2–3 mm wide, linear, apex acute, base broad and concave, light yellow to cream, pubescent with glabrous base on both sides; stamens 120, in 5 to 6 rows, 1–2 mm long, oblong; connective apex shield-like, glabrous; carpels 5 to 8, ovary 1–2 mm long, stigmas connivent, linear, slightly widened at midpoint, 2.1–3 mm long, glabrous to sparsely pubescent. Monocarps **sessile**; monocarps 4 to 9, **27–43 mm long, 17–24 mm wide**, ovoid, broadly ellipsoid or oblongoid, apex rounded, glabrous, rugose to verrucose, **conspicuously lenticellate**, wrinkled when dried, brown outside, endocarp light pink; seeds 7 to 12 per monocarp, **in two rows**, 16–21 mm long, 7–10 mm wide, ellipsoid; **sarcotesta orange, fleshy *in vivo*; aril absent.**

#### Distribution.

A central African species, from southern Nigeria to Republic of Congo and northern Democratic Republic of the Congo; in Cameroon known from East, South and Central regions.

#### Habitat.

A fairly common species; in lowland moist forest habitats. Altitude 200–900 m a.s.l.

#### Local and common names known in Cameroon.

odjobbo (Bulu, Mildbraed (1921)), odzobi (*Service Forestier du Cameroun 67*), sange (Bibaya, *Letouzey & Villiers 10418*).

#### IUCN conservation status.

Not assessed yet.

#### Uses in Cameroon.

None reported.

#### Notes.

﻿﻿*Xylopiaphloiodora* exhibits variation in leaf size and shape but is distinguished by the short flower pedicels with the persistent upper bract closely subtending the upright rigid sepals, as well as the narrow petals that are densely pubescent on both surfaces. The staminal cone of ﻿*X.phloiodora* is very well developed and completely encloses the ovaries; only ﻿*X.aethiopica* among Cameroon ﻿*Xylopia* species has a similarly well-developed cone. The sessile thick-walled monocarps are among the largest of any African ﻿*Xylopia* species, and the seeds have an orange sarcotesta. The secondary veins of the leaves are strongly arcuate, unusual in the genus, and often dry pinkish red, contrasting with the gray background color of the blade.

#### Specimens examined.

**Central Region**: Bank Nyong River near the new bridge ca 65 km SSW of Eséka, 3.65°N, 10.78°E, *16 July 1964*, *de Wilde J.J.F.E* 2838A (P); Bank Nyong River near the new bridge ca 65 km SSW of Eséka, 3.65°N, 10.78°E, *16 July 1964*, *de Wilde J.J.F.E* 2838B (B,BR,K,L,MO,P,P,WAG,YA); Edéa, 3.8°N, 10.13°E, *26 August 1955*, *Mpom B.* 121 (P,YA). **East Region**: 63 km south of Yokadouma 30 km after Ngato 15 km after river ALPICAM ‘base de vie’ then 40 km on forestry road starting 4 km before Maséa village, 3.07°N, 14.65°E, *07 March 2019*, *Couvreur T.L.P.* 1222 (MPU,WAG,YA); Ndakan Sango River, 2.36°N, 16.11°E, *10 May 1988*, *Gentry A.H.* 62649 (MO); A 25 km environ à l’ENE de Mikel village situé à environ 85 km au N de Moloundou sur route de Yokadouma, 2.93°N, 15.33°E, *24 February 1971*, *Letouzey R.* 10418 (P,YA); Nkonbong II sur axe Lomie-Ngoila-Swanké à 15 km au SSW de Ngoila, 2.50°N, 14.00°E, *22 February 1973*, *Letouzey R.* 12026 (P,WAG,YA); Près Ngola (30 km à l’Est de Yokadouma), 3.47°N, 15.32°E, *11 May 1963*, *Letouzey R.* 5027 (P,YA); A 24 km à l’Ouest de Masea (village situé à 50 km au SSW de Yokadouma), 3.15°N, 14.87°E, *04 July 1963*, *Letouzey R.* 5401 (P,YA). **Littoral Region**: Tissongo strip, 3.57°N, 9.869°E, *26 July 1976*, *McKey D.B.* 138 (K); Douala-Edéa Reserve Tissongo study area Transect C, 3.57°N, 9.869°E, *21 June 1976*, *Waterman P.G.* 880 (K). **South Region**: 17 km before Kribi on N7 road from Ed 3.14°N, 9.954°E, *27 February 2018*, *Couvreur T.L.P.* 1164 (K,MPU,P,WAG,YA); Bezirk Kribi Vorland mit einzeln Hügeln bei Adjab 35 km östlich Groß-Batanga, 2.82°N, 10.2°E, *24 July 1911*, *Mildbraed G.W.J.* 6090 (B); Bipindi – Ebolowa, 3.00°N, 10.92°E, *01 December 1913*, *Mildbraed G.W.J.* 7592 (B).

### ﻿﻿﻿ Xylopia pynaertii

Taxon classificationPlantaeMagnolialesAnnonaceae

De Wild., Ann. Mus. Congo Belge, Bot. Sér. 5, 3(1): 79, 1909

A9D8752A-F8DD-5121-A422-88428973BBFB

Fig. 146 ; Map 18H

#### Type.

Democratic Republic of the Congo. Equateur; Eala, *Pynaert L.A.E.J. 567*, 15 Oct 1906: lectotype, designated by Johnson and Murray (2018), p. 201: BR[BR0000024941426]; isolectotypes: BR[BR 0000008825339, BR 0000008825346].

#### Description.

Tree, up to 35 m tall, d.b.h. up to 40 cm; **buttresses present, ca. 0.5 m tall, upper bark red, rough, scaly.** Old branches glabrous, **young branches pubescent, the hairs 0.4–1.5 mm long**. Leaves: petiole 1–3 mm long, 1 mm wide, pubescent, grooved, blade inserted on the side of the petiole; blade 3.6–8.7 cm long, 1.2–2.3 cm wide, lanceolate, ovate or elliptic, **apex acute to obtuse**, base cuneate, papyraceous to subcoriaceous, below pubescent when young, sparsely pubescent when old, above glabrous when young and old, discolorous or occasionally concolorous; midrib sunken, above glabrous to pubescent when young, glabrous to pubescent when old, below pubescent when young and old; secondary veins 8 to 13 pairs, glabrous above; tertiary venation reticulate. Individuals bisexual; inflorescences ramiflorous on young foliate branches, axillary, peduncle ca. 1 mm long. Flowers with 9 perianth parts in 3 whorls, 1 to 2 per inflorescence; pedicel 4–5 mm long, ca. 1 mm in diameter, pubescent; in fruit 3–24 mm long, 2–3 mm in diameter, sparsely pubescent; bracts 2, evenly spaced, 1–2 mm long, 1–2 mm wide; sepals 3, valvate, basally to ½ fused, forming a cup, 2–3 mm long, ca. 2 mm wide, triangular to ovate, apex acute, base truncate, pubescent outside, glabrous inside; petals free, subequal; outer petals 3, 15.2–20.5 mm long, 2.4–3 mm wide at base, linear, apex acute to obtuse, base broad and concave, light yellow to white, sericeous outside, pubescent but glabrous towards the base inside; inner petals 3, valvate, 12.3–16.8 mm long, 2.2–3.2 mm wide at base, linear, apex acute, base broad and concave, light yellow to white, pubescent with base glabrous on both sides; stamens ca. 140, in 4 to 5 rows, ca. 1 mm long, oblong; connective apex shield-like, glabrous; carpels 9 to 11, ovary ca. 1 mm long, stigmas connivent, linear, 2.5–3.8 mm long, pubescent. Monocarps sessile, or stipitate with the stipe 2–8 mm long, 4–6 mm in diameter; monocarps 4 to 8, 26–48 mm long, 13–18 mm wide, **obovoid to oblongoid**, apex rounded, glabrous, verrucose, wrinkled when dried, green outside, endocarp red; seeds 5 to 6 per monocarp, **in two rows**, 9–13 mm long, 6–7 mm wide, ellipsoid to flattened ellipsoid; **sarcotesta white to grayish blue *in vivo*; aril absent.**

#### Distribution.

Sparsely occurring from eastern Nigeria to the Democratic Republic of the Congo; in Cameroon known from two collections in the South and Central regions.

#### Habitat.

A rare and little known species; in primary rain forest and semi-deciduous forests. Altitude 20–200 m a.s.l.

#### Local and common names known in Cameroon.

None recorded.

#### IUCN conservation status.

Least Concern (LC) (Cosiaux et al. 2019bd).

#### Uses in Cameroon.

***Materials***: wood for weapons, tools (Tessmann 1913).

#### Notes.

﻿﻿*Xylopiapynaertii* is distinguished by the scaly bright reddish brown upper bark, the long dense hairs of the young branches and lower leaf surfaces, the relatively small leaves (3.6–8.7 cm long), and the short wide monocarps with seeds in two rows.

#### Specimens examined.

**Central Region**: Pont Kelle (20 km N d’Eséka), 3.65°N, 10.78°E, *09 December 1973*, *Letouzey R.* 12317 (K,P,YA). **South Region**: Massif de Ngovayang village de Atog Boga, 3.15°N, 10.29°E, *30 August 2015*, *Droissart V.* 2049 (BRLU,P).

### ﻿﻿﻿ Xylopia quintasii

Taxon classificationPlantaeMagnolialesAnnonaceae

Pierre ex Engl. & Diels, Monogr. Afrik. Pflanzen.-Fam. 6: 62, 1901

156077AC-9D94-5279-9131-6DE0631135C9

Fig. 150 ; Map 18I

= Xylopia striata Engl., Bot. Jahrb. Syst. 34: 160, 1904. Type. Cameroon. South Region, Bipindi, Zenker G.A. 2663, Jan 1903: lectotype, here designated by Johnson and Murray (2018), p. 68: B[B100153156]; isolectotypes: BM[BM000511005, right hand portion of sheet]; K[K001096587]; P[P00169150, P00169151].
= Xylopia lanepoolei Sprague & Hutch., Kew Bull. Misc. Inform.: 160, 1916. Type. Sierra Leone. Western Area, Headquarters District, Heddles Farm, *Lane-Poole C.E. 210*, Apr 1914: lectotype, designated by Johnson and Murray (2018), p. 69: K[K000380211]; isolectotype: K[spirit collection 15057.00].
= Polyalthia mayumbensis Exell, J. Bot. Suppl. 4: 64, 1926. Type. Angola. Cabinda, Buco Zau, Mayumbe, *Gossweiler J. 6845*, 28 Nov 1916: holotype: BM[BM 000511084]; isotype: COI[COI 00004887].

#### Type.

Sao Tome and Principe. Insel St. Thomé, bei Angolares, *Quintas F. 3*, Jan 1886: lectotype, designated by Johnson and Murray (2018), p. 68: K[K000199059]; isolectotype: COI[COI00004887].

#### Description.

Tree, 10–42 m tall, d.b.h. up to 75 cm; **buttresses present, narrow and thin.** Old branches glabrous, young branches glabrous to sparsely pubescent, the hairs 0.1–0.2 mm long. Leaves: petiole 3–7 mm long, ca. 1 mm wide, glabrous to sparsely pubescent, grooved, blade inserted on the side of the petiole; blade 6–11.9 cm long, 2.6–5.5 cm wide, **obovate to oblanceolate, occasionally elliptic**, apex acuminate, acumen 0.2–0.6 cm long, base cuneate and decurrent, papyraceous to subcoriaceous, below glabrous when young, glabrous to sparsely pubescent when old, above glabrous when young and old, discolourous, often tan-colored below; midrib sunken, above glabrous when young and old, below glabrous when young, glabrous when old; secondary veins 7 to 12 pairs, glabrous above; tertiary venation reticulate. Individuals bisexual; inflorescences ramiflorous on young foliate or old leafless branches, axillary, peduncle sometimes present, 2.6–2.8 mm long. Flowers with 9 perianth parts in 3 whorls, 1 to 7 per inflorescence; pedicel 7–9 mm long, ca. 1 mm in diameter, pubescent; in fruit 8–19 mm long, 2 mm in diameter, glabrous; bracts (2)3(4), evenly spaced, 1–2 mm long, 1–2 mm wide; sepals 3, valvate, free to basally fused, ca. 2 mm long, 2–1 mm wide, triangular to ovate, apex acute to obtuse, base truncate, green, pubescent outside, glabrous inside; petals free, subequal; outer petals 3, 8–15 mm long, 1.8–2.8 mm wide at base, ligulate, **apex obtuse**, base broad and concave, cream to light green, pubescent outside, pubescent and glabrous towards the base inside; inner petals 3, valvate, 7.1–13.2 mm long, 0.7–1.5 mm wide, linear, **apex obtuse, base broad and concave, lacking an internal tooth overhanging the concavity, with fleshy glandlike margins**, cream to light green, pubescent outside, pubescent but glabrous towards the base inside; stamens 50 to 80, in 3 to 4 rows, 1–2 mm long, oblong; connective apex conical, glabrous; carpels 3 to 5, ovary 1–2 mm long, stigmas separate, clavate, ca. 0.5 mm long, glabrous. Monocarps stipitate, stipe 7–11 mm long, 2–4 mm in diameter; monocarps 1 to 4, 35–64 mm long, 7–10 mm wide, narrowly oblongoid, apex with a beak 1.5–4 mm long, glabrous, obliquely striate, occasionally verrucose when dried, slightly constricted around seeds, green outside tinged with brown, endocarp green; seeds 3(5) monocarp, **in a single row**, 10–13 mm long, 6–7 mm wide, ellipsoid; **sarcotesta absent; aril present, fimbriate, extending over the length of the seed, membranous, red to deep orange.**

#### Distribution.

A widespread species in West and Central Africa, from Sierra Leone to northwestern Democratic Republic of the Congo and south to northern Angola, also on the island of São Tomé; in Cameroon known from East, South, Central, Littoral, South-West and West regions.

#### Habitat.

A locally common species in primary lowland rain forest on a variety of soils, occasionally in secondary forest or rarely in inundated forest. Altitude: 0–200(680) m a.s.l.

#### Local and common names known in Cameroon.

mbonba (Yaoundé, *Letouzey 5510*), munjié, monjié, sangé, sangi (Bibaya); nom akwi; muomba (Yaoundé, *de Wilde 1320*), mvǔ’ma (Fang, *Tessmann 760*; Tessmann 1913), mwomba.

#### IUCN conservation status.

Least Concern (LC) (Botanic Gardens Conservation International and IUCN SSC Global Tree Specialist Group 2019g).

#### Uses in Cameroon.

None reported.

#### Notes.

The short blunt petals and fimbriate orange aril of ﻿﻿*Xylopiaquintasii* are shared with ﻿*X.aurantiiodora*. ﻿﻿*Xylopiaquintasii*, however,is typically a tall tree of uplands,with a straight bole and compact crown with crowded horizontal branches, while ﻿*X.aurantiiodora* is a shrub or small tree of riverine habitats, reaching eventually a height of up to 10 meters. It also lacks the tooth on the inner petal found in ﻿*X.aurantiiodora*. The new leaf flush in ﻿*X.quintasii* is red or purple, in contrast to the white leaf flush in ﻿*X.congolensis*De Wild., a similar species not yet known from Cameroon.

**Figure 150.**
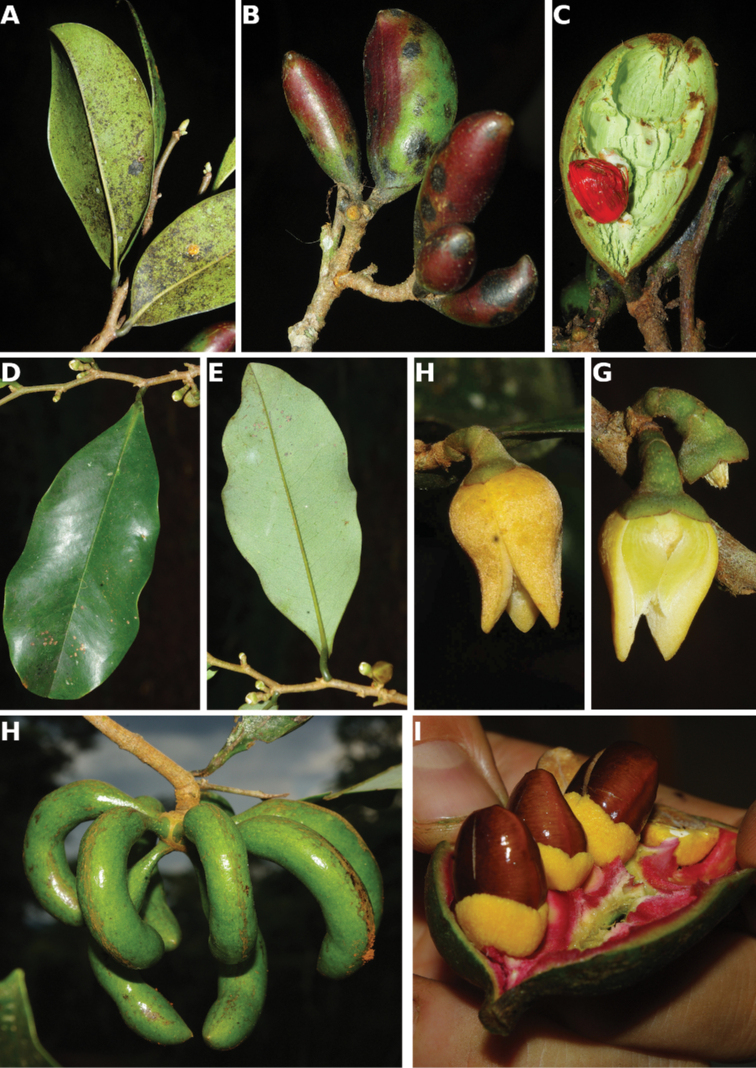
*Xylopiaquintasii***A** leaves, lower side **B** fruits **C** detail of opened monocarp, note fimbriate red aril extending over the whole seed. *Xylopiastaudtii***D** leaf, upper side **E** leaf, lower side **H** flower, side view **G** flower, 1 outer petal removed, side view **H** fruits, immature **I** detail of inside of monocarp, note short bright yellow aril and reddish inside of monocarp **A–C***Couvreur 483*, Lélé, Cameroon **D–I***Couvreur 580*, Gabon. Photos Thomas L.P. Couvreur.

Monkeys of several species have been reported as dispersers of the seeds of ﻿*X.quintasii* (summarized in Johnson and Murray 2018).

#### Selected specimens examined.

**Central Region**: Chantier forestier au sud de Song Bon (SE d’Eseka), 3.42°N, 10.49°E, *08 December 1967*, *Bamps P.R.J.* 1376 (P,YA); Ca 5O km NW of Eseka W of Yaoundé on opposite of the Kele River, 3.65°N, 10.78°E, *23 November 1963*, *de Wilde J.J.F.E* 1320 (BR,K,P,WAG,YA); ca 50 km NW of Eséka W of Yaoundé on opposite [side?] of the Kelè-river, 3.75°N, 10.61°E, *01 November 1963*, *de Wilde W.J.J.O* 1320 (K,P,WAG); Yaoundé, 3.87°N, 11.52°E, *01 January 1935*, *Foury P.* 36 (P); M´balmayo, 3.52°N, 11.5°E, *28 October 1959*, *Mpom B.* 353 (P,YA). **East Region**: 28 km SW of Bertoua near Toungrélo, 4.58°N, 13.68°E, *12 January 1961*, *Breteler F.J.* 2398 (A,BR,G,K,M,P,SL,U,UC,WAG,YA); A 26 km au SSW de Koso (village situé à 60 km au SSW de Batouri), 3.93°N, 14.17°E, *27 July 1963*, *Letouzey R.* 5510 (K,P,YA); Moloundou, 2.05°N, 15.17°E, *06 December 1910*, *Mildbraed G.W.J.* 4003 (P); south of Sanaga between Yaoundé and Deng Deng, between the union of the Lom (Sanaga) and Djerem rivers about, 125 km NE of Yaoundé, 5.33°N, 13.4°E, *01 February 1914*, *Mildbraed G.W.J.* 8294 (K). **South Region**: Bipindi, 3.26°N, 10.20°E, *23 June 1918*, *Annet E.* 319 (P); 22 km from Kribi Lolodorf road, 3.05°N, 10.05°E, *09 June 1969*, *Bos J.J.* 4773 (BR,K,LD,LM,MO,P,POZG,WAG,YA); 28 km east from Lélé village, 2.27°N, 13.29°E, *09 September 2013*, *Couvreur T.L.P.* 483 (WAG,YA); ca 15 km east from Lélé village, 2.29°N, 13.33°E, *10 September 2013*, *Couvreur T.L.P.* 500 (WAG,YA); Hill facing the village of N’koladom, 2.80°N, 11.16°E, *03 January 1975*, *de Wilde J.J.F.E* 7871 (B,BR,K,MO,P,U,WAG,YA); Hill roughly between Nkolandom and Nkoemvone, 2.8°N, 11.15°E, *09 January 1975*, *de Wilde J.J.F.E* 7889 (B,BR,K,MO,P,U,WAG,YA); TDC Sud Cameroun, 2.65°N, 9.9°E, *14 November 1991*, *Hallé F.* 4240 (WAG); Bipindi – Ebolowa, 3.00°N, 10.92°E, *01 December 1913*, *Mildbraed G.W.J.* 7613 (K); [Locality not legible- but should be Bedai], 2.17°N, 11.12°E, *December 1908* [?], *Tessmann G.* 760 (K); Campo-Ma’an area 2.56°N, 9.833°E, *16 April 2001*, *van Andel T.R.* 3345 (KRIBI,WAG,YA); Bipindi, 3.08°N, 10.42°E, *01 January 1900*, *Zenker G.A.* 2080 (L,P,WAG); Bipindi, 3.08°N, 10.42°E, *01 January 1914*, *Zenker G.A.* 2094 (B,GH,M,MO,P,WAG); Bipindi, 3.08°N, 10.41°E, *01 January 1903*, *Zenker G.A.* 2655 (L,WAG); Bipindi, 3.08°N, 10.42°E, *01 January 1903*, *Zenker G.A.* 2663 (P); Mimfia, 3.06°N, 10.38°E, *01 June 1913*, *Zenker G.A.* 359 (P,U); Bipindi, 3.08°N, 10.41°E, *01 October 1913*, *Zenker G.A.* 408 (P,U); Bipindi, 3.08°N, 10.42°E, *01 January 1911*, *Zenker G.A.* 4096 (L,P); Bipindi, 3.08°N, 10.42°E, *01 January 1913*, *Zenker G.A.* 4738 (L,P); Mimfia, 3.06°N, 10.38°E, *01 May 1914*, *Zenker G.A.* 580 (P,U). **South-West Region**: forest near Ngusi village north of Nyassosso, 4.88°N, 9.7°E, *26 April 1986*, *Etuge M.* 56 (B,K,MO,WAG,YA); Korup National Park, 5.02°N, 8.8°E, *01 January 1998*, *Kenfack D.* 1187 (MO); mile 12 Mamfe road between Kumba and Badu 4.75°N, 9.48°E, *01 October 1986*, *Nemba J.* 293 (MO,P,WAG). **West Region**: Piste Santchou-Bale 18 km SSW de Dschang, 5.26°N, 10.04°E, *26 November 1974*, *Letouzey R.* 13327 (P,YA).

### ﻿﻿﻿ Xylopia rubescens

Taxon classificationPlantaeMagnolialesAnnonaceae

Oliv., Fl. Trop. Afr. 1: 30, 1868

C6B602B3-75A6-5C84-A67C-F0BF744D2905

Fig. 151 ; Map 19A

≡ Xylopicrum rubescens (Oliver) Kuntze, Revis. gen. pl. 1: 8, 1891.
= Xylopia klaineana Pierre ex Engler & Diels, Monogr. Afrik. Pflanzen-Fam. 6: 59–60. 1901. ﻿﻿XylopiarubescensOlivervar.klaineana (Engler & Diels) Pellegrin, Bull. Soc. Bot. France, Mém. 31: 70. 1949. Type. Gabon. Without definite locality, Klaine T.-J. 1327, Oct 1898: holotype: P[P00169139]; isotypes: B[B100154150], P[P00169138]).
= Xylopia humilis Engl. & Diels, Monogr. Afrik. Pflanzen.-Fam. 6: 60, 1901. Type. Liberia. Grand Bassa County, Fishtown bei Granbassa, *Dinklage M.J. 2006*, 27 Aug 1898: lectotype, designated by Johnson and Murray (2018), p. 49: B[B10 0154147]; isotypes: A[A00061927, A00062417]; B[B100154145, B100154146, B1001541480]; K[K000199074, K000199075, K000199076].
= Xylopia batesii Engl. & Diels, Monogr. Afrik. Pflanzen.-Fam. 6: 62, 1901. Type. Gabon. Angom, 70 engl. Meilen östlich von Gabun, Bates G.L. 561, Oct 1896: holotype: K[K000199058]; isotypes: BM[BM000510769]; G[G00190711]; P[P00169131, P00169132].
= Xylopia butayei De Wild., Ann. Mus. Congo, Bot. sér. 4, 1: 33, 1902. Type. Democratic Republic of the Congo. Kongo Central, Malela (Bas-Congo), *Butaye R. 2239*, no date: holotype: BR; isotype: BR[BR0000008825391].
= Xylopia zenkeri Engl. & Diels, Bot. Jahrb. Syst. 39: 480, 1907. Type. Cameroon. South Province, Bipindi, Zenker G.A. 2827, Mar 1904: holotype B[B 100154149]; isotypes: BM[BM 000511041]; G [00190712, G 00190713]; GOET[GOET 005735]; HBG[HBG 502474]; K[000199060]; L [0196246]; M[M 0107919]; P[P00169131]; S[S 07-13458]; WAG[WAG 0065882]; WU[WU 0025792].
= Xylopia gossweileri Exell, J. Bot. Suppl. 6: 64, 1926. Type. Angola. Cabinda, Pango Munga, Mayumbe, *Gossweiler J. 6222*, no date: holotype: BM[M000511046].

#### Type.

Nigeria. Rivers state; Old Calabar, *Thomson W.C. 53*, no date: holotype: K[K000199073].

#### Description.

Tree, up to 30 m tall, d.b.h. up to 70 cm; **stilt roots present, up to 2 m on the trunk.** Old branches glabrous, **turning grayish white**, young branches glabrous to pubescent with appressed golden hairs 0.2–0.5 mm long. Leaves: petiole 5–12 mm long, ca. 2 mm wide, glabrous to sparsely pubescent, grooved, blade inserted on the side of the petiole; **blade 7.3–21.3 cm long**, 3.6–8.4 cm wide, oblong, elliptic, or oblanceolate, apex acuminate to cuspidate, acumen 0.2–1.5 cm long, base cuneate to rounded, decurrent, papyraceous to subcoriaceous, below sparsely appressed-pubescent, rarely glabrous, above glabrous, **strongly discolorous**; midrib flat to sunken, above glabrous when young and old, below glabrous to sparsely pubescent when young, glabrous when old; secondary veins 8 to 16 pairs, glabrous above; tertiary venation reticulate. Flowers bisexual with 9 perianth parts in 3 whorls. Individuals bisexual; inflorescences ramiflorous on young foliate or more often **clustered** on older leafless branches, axillary, peduncle 1.5–2.5 mm long. Flowers with 9 perianth parts in 3 whorls, 1 to 3 per inflorescence; pedicel 4–9 mm long, ca. 1 mm in diameter, pubescent; in fruit 9–15 mm long, 2–7 mm in diameter, glabrous; bracts (2)3(5), evenly spaced, 1–3 mm long, 1–3 mm wide; sepals 3, valvate, basally fused, 2–4 mm long, 3–4 mm wide, ovate to broadly triangular, apex acute, base truncate, green, sericeous outside, glabrous inside; petals free, outer petals much longer than inner; outer petals 3, 17.6–35 mm long, 2.5–5.1 mm wide at base, linear, apex acute, base broad and concave, yellow, pubescent outside, pubescent and glabrous towards the base inside; inner petals 3, valvate, 3.5–6.7 mm long, 2.2–4.6 mm wide, ovate to rhombic, apex acuminate, base broad and concave, red with cream base and apex, pubescent outside, pubescent and glabrous towards the base inside; stamens 70 to 77, in 4 to 5 rows, ca. 2 mm long, clavate to oblong; connective apex shield-like, glabrous; carpels 4 to 12, ovary 1–2 mm long, stigmas loosely connivent, linear, 1.4–2.9 mm long, with glandular appendages. Monocarps stipitate, stipe 4–20 mm long, 2–6 mm in diameter; monocarps 1 to 15, 41–16 mm long, 6–12 mm wide, narrowly oblong, **somewhat falciform and strongly torulose to moniliform**, apex with a distinct beak up to 5 mm long, glabrous, verrucose and wrinkled when dried, black or dark purple outside, endocarp pink to scarlet; seeds 1 to 7 per monocarp, **in a single row**, 10–20 mm long, 6–11 mm wide, ellipsoid; **sarcotesta absent; aril present, brushlike, unlobed, red to orange.**

**Map 19.**
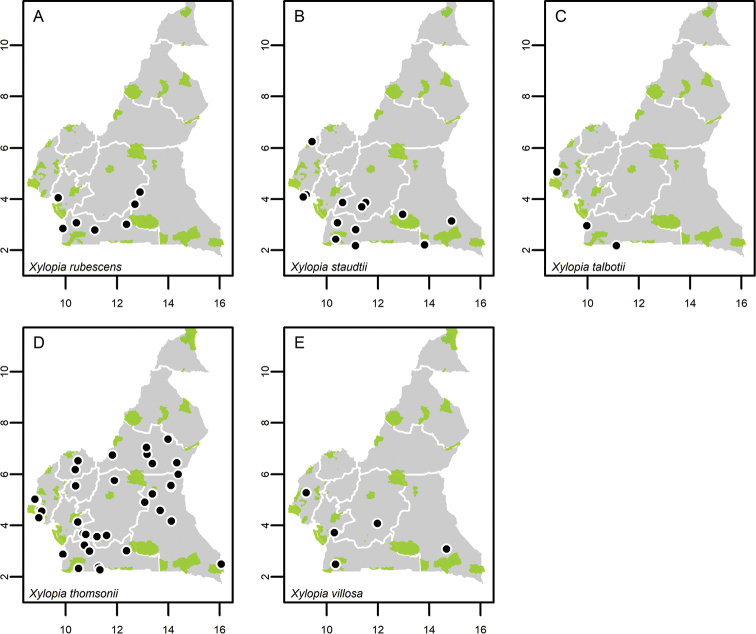
**A***Xylopiarubescens***B***Xylopiastaudtii***C***Xylopiatalbotii***D***Xylopiathomsonii***E***Xylopiavillosa*. White borders represent region limits in Cameroon; green patches represent protected areas (see methods and Suppl. material 1: Fig. S1).

**Figure 151.**
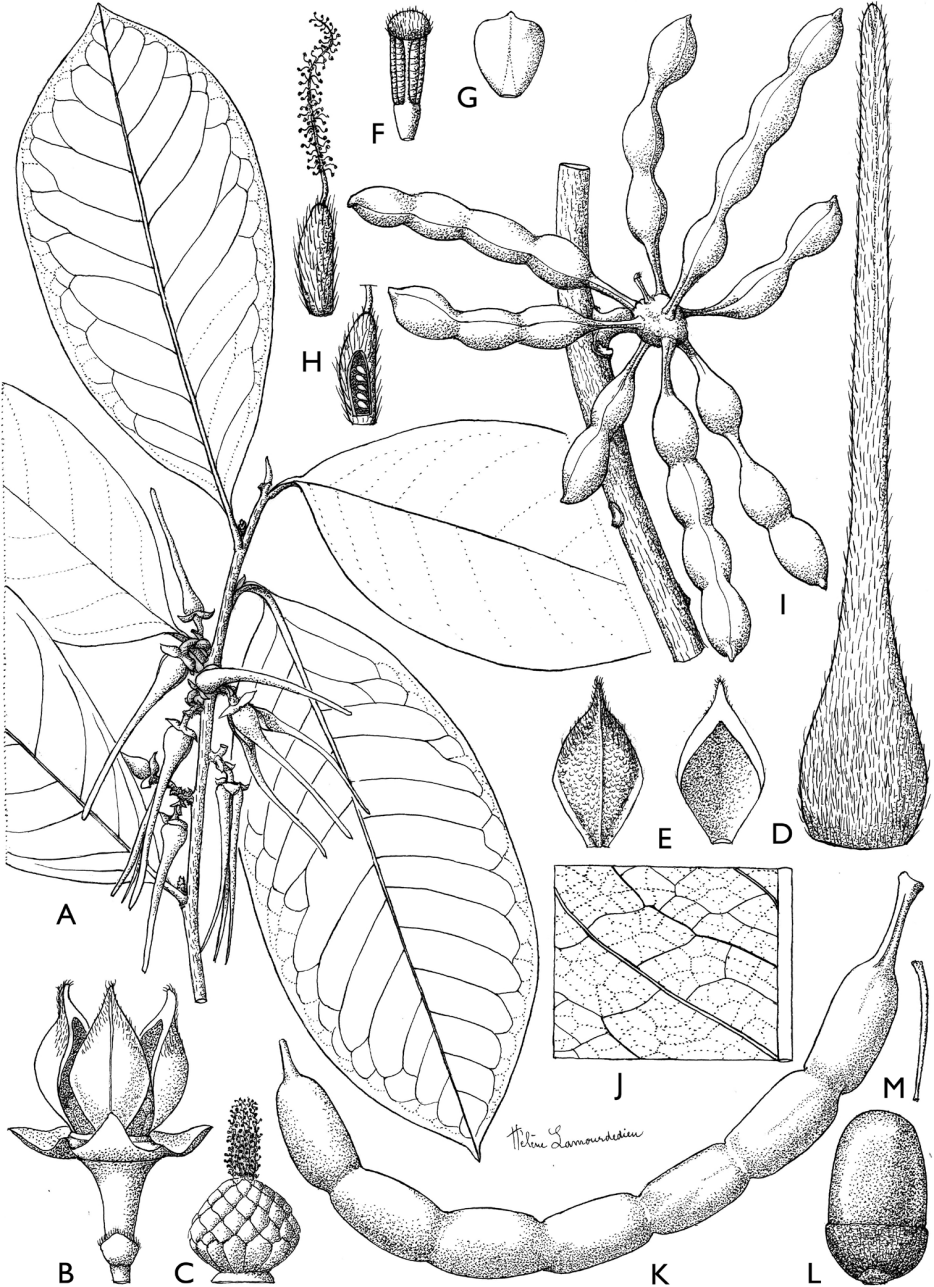
*Xylopiarubescens***A** flowering branch **B** flower, outer petals removed **C** detail of receptacle, petals removed **D** outer petal, outer side **E** inner petal, outer and inner sides **F** stamen, front view **G** staminode **H** carpel, side view and detail of ovules **I** fruit **J** detail of venation lower side of leaf **K** monocarp **L** seed, side view **M** part of aril **A–H** from *Le Testu 9019***I** from *Aubréville 1511***J–M** from *Klaine 1327.* Drawings by Hélène Lamourdedieu, Publications Scientifiques du Muséum national d’Histoire naturelle, Paris; modified from Le Thomas (1969b; pl. 28, p. 161).

#### Distribution.

A widespread species with a disjunct distribution in West Africa, from Guinea-Bissau to Ghana, and in Central Africa from Nigeria to southern South Sudan and south to northern Angola and Mozambique; in Cameroon known from East, South, Littoral and regions.

#### Habitat.

A common species, in a range of wetland habitats, including gallery and other riparian forests, swamp forest, *Raphia* swamps, and pond edges. Altitude 0–900 m a.s.l.

#### Local and common names known in Cameroon.

ntua (*Fleury 33135*), odjobi (*Letouzey 1611*), odjobi nzam (Focho et al. 2010), odzobé (*Fleury 33135*), ôjobi (*Bates 1317*).

#### IUCN conservation status.

Least Concern (LC) (Cosiaux et al. 2019be).

#### Uses in Cameroon.

None reported.

#### Notes.

﻿﻿*Xylopiarubescens* is readily recognized by the combination of relatively large leaves with decurrent bases, which often have an orange-red color on the lower surface of the leaf in dried specimens, branches with grayish white, narrow flowers that are often clustered on leafless porolder branches, and distinctly torulose to moniliform monocarps. Throughout its wide range it is a wetland species, and one of the few ﻿*Xylopia* species with stilt roots. The short inner petals and brushlike arils distinguish ﻿﻿*X.rubescens* from ﻿*X.aethiopica*, with which it is sometimes confused.

#### Specimens examined.

**East Region**: Marécage du Niagoul entre Koumbou et Miambo, 4.27°N, 12.9°E, *13 April 1959*, *Letouzey R.* 1611 (P,YA). **Littoral Region**: Duala, 4.05°N, 9.71°E, *01 June 1917*, *Fleury F.* 33135 (P). **South Region**: Batanga, 2.86°N, 9.889°E, *20 September 1945*, *Aubréville A.* 125 (P); Bitye Yaunde, 3.02°N, 12.37°E, *01 January 1919*, *Bates G.L.* 1317 (BM,MO); N´Koemvone, 2.8°N, 11.13°E, *11 April 1975*, *de Wilde J.J.F.E* 8166 (B,BR,K,MO,P,WAG,YA); Près Akok Bikélé, 3.8°N, 12.7°E, *03 March 1962*, *Letouzey R.* 4464 (YA); Bipindi, 3.08°N, 10.41°E, *01 January 1904*, *Zenker G.A.* 2827 (G,L,M,WAG).**Unknown region**: Vuneli, *01 February 1928*, *Hédin L.* 1668 (P, OWU).

### ﻿﻿﻿ Xylopia staudtii

Taxon classificationPlantaeMagnolialesAnnonaceae

Engl. & Diels, Notizbl. Königl. Bot. Gart. Berlin 2: 298, 1899

7218D70B-AECB-5ABA-A600-60E4875F6A6F

**Figure 152.**
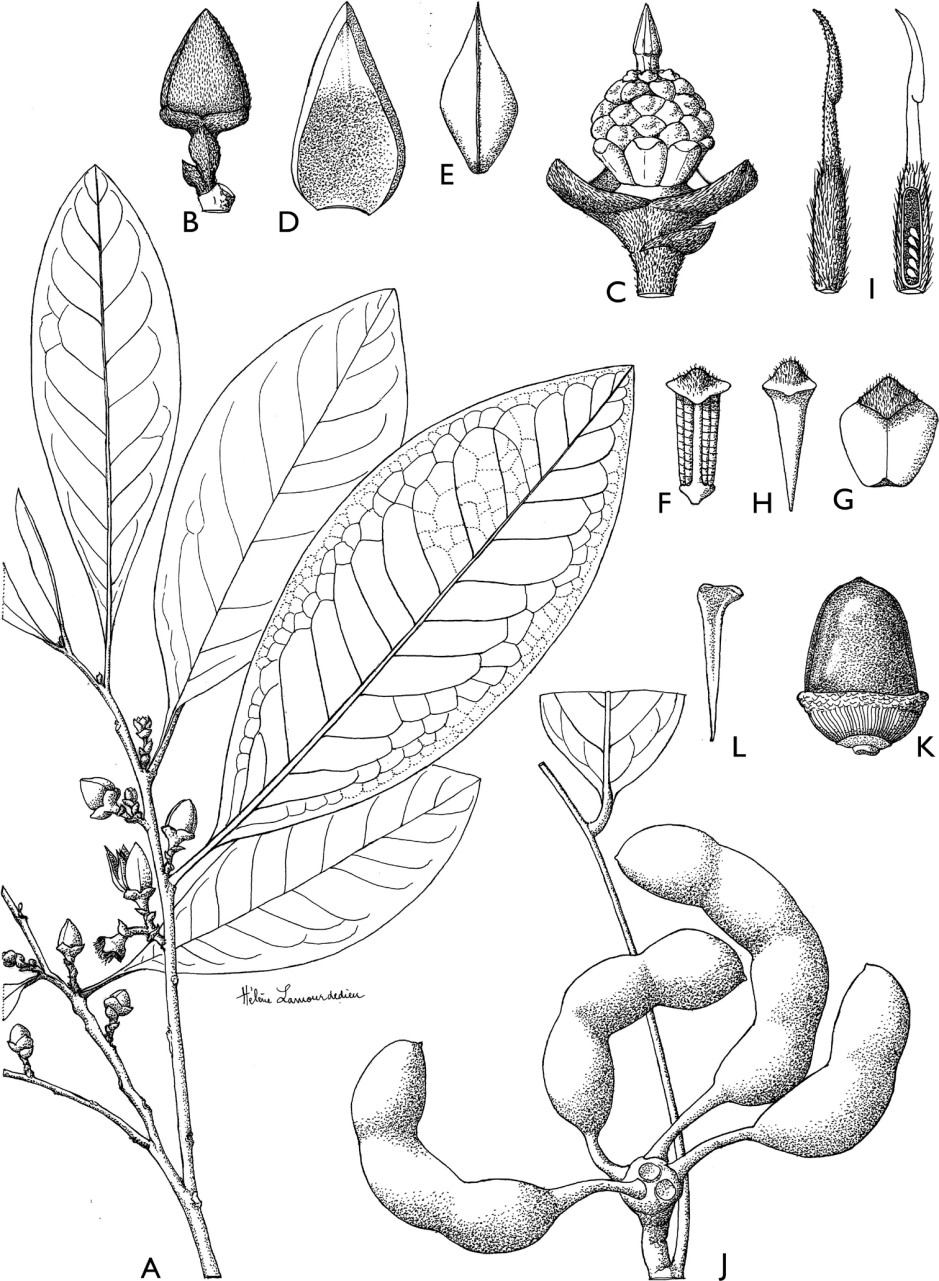
*Xylopiastaudtii***A** flowering branch **B** flower bud, side view **C** detail of receptacle, petals removed **D** outer petal, inner view **E** inner petal, outer view **G** stamen **H** staminode, outer whorl **H** staminode, inner whorl **I** carpel, side view and detail of ovules **J** fruit **K** seed, front view **L** part of the aril. 1 from *Letouzey 5404***B–I** from *Le Testu 9287***J–L** from *Aubréville 1941*. Drawings by Hélène Lamourdedieu, Publications Scientifiques du Muséum national d’Histoire naturelle, Paris; modified from Le Thomas (1969b; pl. 29, p. 163).

Figs 150 , 152 ; Map 19B

≡ Xylopicrum staudtii (Engler) Kuntze, Deutsch. Bot. Monatsschr. 21: 173–174, 1903.
= Xylopia mayombensis De Wild., Bull. Jard. Bot. État 4: 386, 1914. Type. Democratic Republic of the Congo. Kongo Central Province, Ganda-Sundi, *de Briey J. 219*, 1913: holotype: BR[BR0000024941587]; isotypes: BR[BR0000008825421, BR0000008825438, BR0000008825445, BR0000008825506].

#### Type.

Cameroon. South-West Region; Johann-Albrechtshöhe[Kumba], *Staudt A. 530*, 1896: holotype: B; isotypes K[K000105614]; P[00169112, 00169113].

#### Description.

Tree, up to 30(50) m tall, d.b.h. up to 80 cm; **stilt roots and small buttresses present.** Old branches glabrous, young branches glabrous to pubescent with appressed hairs 0.3–0.4 mm long. Leaves: petiole 3–9 mm long, 2 mm wide, glabrous to sparsely pubescent, grooved, blade inserted on the side of the petiole; blade 5.1–11.8 cm long, 2–5.6 cm wide, oblanceolate to obovate, occasionally elliptic, apex acuminate, acumen 0.2–0.3 cm long, base cuneate and decurrent, coriaceous to subcoriaceous, below sparsely pubescent when young, glabrous when old, above glabrous when young and old, discolorous; midrib sunken, above glabrous when young and old, below glabrous when young, glabrous when old; secondary veins 7 to 11 pairs, above; tertiary venation reticulate. Individuals bisexual; inflorescences ramiflorous on young foliate branches, axillary, peduncle 0.5–2.4 mm long. Flowers with 9 perianth parts in 3 whorls, 1 to 3 per inflorescence; pedicel 3–8 mm long, ca. 1 mm in diameter, sparsely pubescent to glabrous; in fruit 7–13 mm long, 3–8 mm in diameter, sparsely pubescent to glabrous; bracts 2 to 4, evenly spaced, 1–2 mm long, 1–2 mm wide; sepals 3, valvate, basally to ½ fused, **forming a cup**, 2–3 mm long, 3–4 mm wide, ovate, apex acute to obtuse, base truncate, green, pubescent outside, glabrous inside; petals free, **subequal; outer petals 3, 5.8–9.6 mm long, 4–5.2 mm wide at base, ovate**, apex acute, base truncate, yellow, pubescent outside, pubescent but glabrous towards the base inside; **inner petals 3, valvate, 4.1–8.4 mm long, 2–3.2 mm wide at base**, elliptic to rhombic, apex acute, base truncate, yellow, pubescent outside, pubescent towards base inside; stamens 100 to 120, in 5 to 6 rows, 2 mm long, clavate; connective apex shield-like with a central conical point, pubescent, bright yellow; carpels 3 to 11, ovary 1–2 mm long, stigmas loosely connivent or separate, linear, 2.6–4.6 mm long, tuberculate and sparsely pubescent. Monocarps stipitate, stipe 7–15 mm long, 3–6 mm in diameter; monocarps 3 to 5, 37–98 mm long, 12–21 mm wide, cylindrical, apex rounded to sometimes with a curved beak 1.3–3 mm long, glabrous to sparsely pubescent, sometimes slightly constricted around seeds and verrucose and wrinkled when dried, green outside, endocarp scarlet; seeds 1 to 5 per monocarp, **in a single row**, 14–19 mm long, 9–13 mm wide, ellipsoid; **sarcotesta absent; aril present, brushlike, bright yellow to orange.**

#### Distribution.

A widespread species with a disjunct distribution in West Africa, from Sierra Leone to Ghana, and in Central Africa from eastern Nigeria to the Democratic Republic of the Congo, western Uganda and Cabinda (Angola); in Cameroon known from East, South, Central, Littoral, South-West and West regions.

#### Habitat.

A fairly common species, in primary and old secondary rain forests. Altitude 0–1350 m a.s.l.

#### Local and common names known in Cameroon.

nkala (Bulu, *de Wilde 7941*), ntom (Ntoumou, Focho et al. (2010)), odjobi (Ntoumou, Focho et al. (2010), *Letouzey 8178*).

#### IUCN conservation status.

Least Concern (LC) (Cosiaux et al. 2019bf).

#### Uses in Cameroon.

None reported.

#### Notes.

﻿﻿*Xylopiastaudtii* reaches the greatest height of any ﻿*Xylopia* species, becoming a canopy tree up to 50 meters tall. The stilt roots of this species were described by Jeník (1970) in detail and consist of adventitious roots up to one meter above the ground and stilted pneumatophores or “peg roots” arising laterally from the roots and expanding up to 10 m from the main trunk. With its broad flower buds and petals, ﻿﻿*Xylopiastaudtii* is most similar to ﻿﻿*Xylopiaafricana*, also sharing with that species the oblong thick-walled monocarps and large seeds with a brushlike aril. ﻿﻿*Xylopiaafricana*,however, is a smaller tree, with larger sepals, obtuse outer petal apices, and red arils rather than bright yellow or orange as in ﻿﻿*X.staudtii*.

The seeds of ﻿﻿*X.staudtii* have been reported as a food item for two hornbill species (Whitney et al. 1998), four species of mangabeys and guenons (Gautier-Hion et al. 1985; Poulsen et al. 2001), and mandrills (Lahm 1986).

#### Specimens examined.

**Central Region**: Yaoundé, 3.87°N, 11.52°E, *01 January 1935*, *Foury P.* 46 (P); 21 km NO d’Oveng, 3.7°N, 11.37°E, *24 October 1966*, *Letouzey R.* 8178 (L,P,YA). **East Region**: Dja Reserve (Réserve de Faune du Dja) Bouamir Research Area 90 km southeast of Akonolinga, 3.41°N, 12.96°E, *24 October 1994*, *Fogiel M.K.* 864 (MO,YA); Colline à l’ENE de Mbalam (140 km ESE de Djoum près Souanké-Congo), 2.22°N, 13.82°E, *20 January 1973*, *Letouzey R.* 11865 (P,YA); 23 km à l’ouest de Masea (village situé à 50 km au SSW de Yokadouma), 3.15°N, 14.87°E, *04 July 1963*, *Letouzey R.* 5404 (P,YA). **Littoral Region**: Makak, 3.87°N, 10.62°E, *22 January 1945*, *Letouzey R.* 1126 (P). **South Region**: hill above Nlonacko near village Ebianemeyong, 2.43°N, 10.35°E, *12 December 1998*, *de Wilde J.J.F.E* 12161 (BR,KRIBI,MO,S,WAG); Station de cacaoyer de N’koemvone 14 km On the road from Ebolowa to Ambam, 2.81°N, 11.13°E, *31 January 1975*, *de Wilde J.J.F.E* 7941 (B,BR,K,MO,P,U,WAG,YA); N´Koemvone, 2.81°N, 11.13°E, *04 March 1975*, *de Wilde J.J.F.E* 8029 (B,BR,K,MO,P,U,WAG,YA); Près Nteigne PK 108 sur route Mintom I (70 km E de Djoum), 2.2°N, 13.81°E, *19 January 1973*, *Letouzey R.* 11854 (P,YA); Bebai- Camposgebiet- Weg u [locality reported as being in Equatorial Guinea], 2.17°N, 11.12°E, *18 November 1908*, *Tessmann G.* 644 (K); Mimfia, 3.06°N, 10.38°E, *01 March 1913*, *Zenker G.A.* 246 (P,U); Bipindi, 3.08°N, 10.41°E, *01 January 1908*, *Zenker G.A.* 3653 (L,P); Bipindi, 3.08°N, 10.42°E, *01 January 1911*, *Zenker G.A.* 3953 (L,P); Bipindi, 3.08°N, 10.42°E, *01 January 1913*, *Zenker G.A.* 4862 (L,P). **South-West Region**: Johann-Albrechtshöhe[Kumba] area 4.16°N, 9.2°E, *1896*, *Staudt A.* 530 (K,P); Mount above Batoke, 4.08°N, 9.083°E, *24 April 1984*, *Thomas D.W.* 3463 (MO,P,WAG,YA); Takamanda Forest Reserve footpath from Mbilishi to Kalu 6.25°N, 9.43°E, *01 May 1987*, *Thomas D.W.* 7401 (MO).

### ﻿﻿﻿ Xylopia talbotii

Taxon classificationPlantaeMagnolialesAnnonaceae

Exell, J. Bot. 69: 98, 1931

CA2F182F-4F63-528D-9B6C-3315C5280F32

Map 19C

#### Type.

Nigeria. Cross River State; Oban, *Talbot P.A. 1601*, 1912: holotype: BM; isotype: K[000199068].

#### Description.

Tree, up to 5 m tall, d.b.h. unknown; stilt roots and buttresses absent. Old branches glabrous to pubescent, **young branches rusty-pubescent**, with sparser erect hairs 1.5–2 mm long mixed with denser erect hairs 0.2–0.4 mm long. Leaves: petiole 3–5 mm long, ca. 1 mm wide, rusty-pubescent, grooved, blade inserted on the side of the petiole; blade 9.2–12.5 cm long, 3–4.2 cm wide, lanceolate-oblong to elliptic, rarely oblanceolate, **apex acuminate, acumen 0.9–1.7 cm long**, base cuneate to rounded, papyraceous, below pubescent when young, densely pubescent to pubescent when old, above glabrous when young and old, concolorous to discolorous; midrib sunken or flat, above densely pubescent when young, pubescent when old, below pubescent when young and old; secondary veins 11 to 15 pairs, glabrous, above; tertiary venation reticulate. Individuals bisexual; inflorescences ramiflorous on young foliate branches, axillary, peduncle absent. Flowers with 9 perianth parts in 3 whorls, 1 per inflorescence; pedicel 3–6 mm long, 1–2 mm in diameter, **rusty-pubescent**; in fruit 3–6 mm long, 1–2 mm in diameter, glabrous; bracts 2 to 3, evenly spaced, 3–4 mm long, 2–3 mm wide; sepals 3, valvate, basally fused, 4–6 mm long, 3–4 mm wide, triangular to ovate, apex acute to acuminate, base truncate, densely rusty-pubescent outside, glabrous inside; petals free, subequal; outer petals 3, 32–38 mm long, 3.1–3.3 mm wide at base, linear, apex acute to obtuse, base broad and concave, color unknown, sericeous outside, pubescent and glabrous towards the base inside; inner petals 3, valvate, 16–33 mm long, 2.1–3.2 mm wide at base, linear, apex acute, base truncate, color unknown, pubescent, base glabrous outside, pubescent and glabrous towards the base inside; stamens ca. 140, in rows, 1–2 mm long, oblong; connective apex shield-like, glabrous; carpels ca. 8, ovary 1 mm long, stigmas connivent, linear, bent at midpoint, 2.5–3 mm long, glabrous. Monocarps (only immature ones seen) sessile; monocarps ca. 8, 10–30 mm long, 9–25 mm wide, apex with a small curved beak, sparsely pubescent; seeds unknown.

#### Distribution.

Eastern Nigeria and Cameroon; in Cameroon known from South and South-West regions.

#### Habitat.

A rare species, in Cameroon known from three collections; in gallery forests near rivers (*Bos 4650*) or lowland rain forests. Altitude: 0–50 m a.s.l.

#### Local and common names known in Cameroon.

None recorded.

#### IUCN conservation status.

Data Deficient (DD) (Cosiaux et al. 2019bg).

#### Uses in Cameroon.

None reported.

#### Notes.

﻿﻿*Xylopiatalbotii* is readily distinguished by the striking and persistent rusty pubescence of the twigs, petioles, pedicels, and sepals, and the long-acuminate leaves. It is similar in flower characteristics to the more widespread ﻿﻿*X.thomsonii*.

#### Specimens examined.

**South Region**: ca 10 km From Kribi S of Lolodorf road, 2.96°N, 9.966°E, *27 May 1969*, *Bos J.J.* 4650 (BR,P,WAG,YA); Bebai - Camposgebiet - Weg u [locality reported as being in Equatorial Guinea], 2.17°N, 11.12°E, *December 1908*, *Tessmann G.* 747 (K). **South-West Region**: Korup National Park, 5.05°N, 8.8°E, *03 April 1984*, *Thomas D.W.* 3204 (K,MO,P).

### ﻿﻿﻿ Xylopia thomsonii

Taxon classificationPlantaeMagnolialesAnnonaceae

Oliv., Fl. trop. Afr. 1: 31, 1868

A52D30AE-100A-5EE6-988E-FB502A9E5F26

Fig. 153 ; Map 19D

≡ Xylopicrum thomsonii (Oliver) Kuntze, Revis. gen. pl. 1: 8, 1891.
= Xylopia pyrifolia Engl., Pflanzenw. Ost-Afrikas C: 179, 1895. Type. Democratic Republic of the Congo. Ituri Province, Bataibo am Duki, Stuhlmann F.L. 2781, 7 Nov 1891: holotype: B[100153155].
= ﻿﻿Xylopia tenuifolia Engl. & Diels, Notizbl. Königl. Bot. Gart. Berlin 2: 298, 1899; Xylopicrum tenuifolium (Engler & Diels) Kuntze, Deutsch. Bot. Monatsschr. 21: 173–174, 1903. Type. Cameroon. South-West Region, Urwald zwischen Mowange und Isongo, Preuss C.G.T. s.n., Mar 1897: B, apparently destroyed.
= Xylopia seretii [“*sereti*”] De Wild., Ann. Mus. Congo Belge, Bot. sér. 5, 3: 79, 1909. Type. Democratic Republic of the Congo. Haut-Uele, bords d’une riviere sur la route de Faradje à Vankerkhovenville, *Seret F. 555*, 12 Apr 1906: holotype: BR[BR0000024941396]; isotype: BR[0000008824370].

#### Type.

Nigeria. Rivers state; Old Calabar, *Thomson W.C. 53*, no date: holotype: K[000199064].

#### Description.

**Scandent shrub up to 10 m tall or rarely an upright tree**, d.b.h. up to 17 cm; stilt roots and buttresses absent. Old branches pubescent, young branches pubescent with erect dull gray to brown hairs 0.1–1.3 mm long. Leaves: petiole 2–6 mm long, ca. 1 mm wide, sparsely pubescent, grooved, blade inserted on the side of the petiole; blade 8.4–13.7 cm long, 2.5–4.7 cm wide, lanceolate, elliptic to oblong, or oblong-oblanceolate, apex acuminate, acumen 0.3–1.6 cm long, base cuneate to rounded, papyraceous, below glabrous to sparsely pubescent when young, glabrous to sparsely pubescent when old, above glabrous when young and old, generally discolorous; midrib sunken, above pubescent when young, glabrous when old, below pubescent when young and old; secondary veins 11 to 17 pairs, glabrous above; tertiary venation reticulate. Individuals bisexual; inflorescences ramiflorous on young foliate or old leafless branches, axillary, peduncle absent. Flowers with 9 perianth parts in 3 whorls, **1 (2) per inflorescence**; pedicel 3–8 mm long, ca. 1 mm in diameter, pubescent; in fruit 5–12 mm long, 2–8 mm in diameter, sparsely pubescent; **bracts 3 to 6, overlapping toward pedicel base**, 2–4 mm long, 2–3 mm wide; sepals 3, valvate, basally fused, 2–4 mm long, 3 mm wide, triangular to ovate, apex acute to obtuse, base truncate, pubescent outside, glabrous inside; petals free, subequal; outer petals 3, 14.6–49 mm long, 2.4–3.8 mm wide at base, linear, apex acute to obtuse, base broad and concave, cream to white, pubescent towards base outside, pubescent with glabrous base inside; inner petals 3, valvate, 16–33 mm long, 2.1–3.2 mm wide at base, linear, apex acute, base broad and concave, cream to white, pubescent with glabrous base on both sides; stamens ca. 200, in 4 to 6 rows, 1–2 mm long, oblong; connective apex shield-like, glabrous; carpels 7 to 13, ovary ca. 1 mm long, stigmas connivent, linear-falcate, 1.8–3.4 mm long, pubescent. Monocarps stipitate, stipe 3–11 mm long, 2–3 mm in diameter; monocarps 5 to 10(12), 21–65 mm long, 6–12 mm wide, narrowly oblong, weakly torulose, apex obtuse or with a beak 1–4 mm long, glabrous, verrucose or lenticellate (in vivo) and smooth to longitudinally wrinkled when dried, green outside, endocarp red to red-purple; seeds up to 9, commonly 4–8 per monocarp, **in a single row**, 9–12 mm long, 6–7 mm wide, ellipsoid; **sarcotesta thin, waxy, green *in vivo*; aril absent**.

#### Distribution.

A widespread species in central Africa, from Nigeria to South Sudan and south to northeastern Angola; in Cameroon known from East, South, Central, Littoral, South-West, West and Adamaoua regions.

#### Habitat.

A common species with a wide ecological amplitude, in the understory of primary or old secondary rain forest or gallery forest, occasionally in marshy forest or forest edges near water. Altitude 0–1300 m a.s.l.

#### Local and common names known in Cameroon.

akwi (Ewondo, *Breteler 931*).

#### IUCN conservation status.

Least Concern (LC) (Harvey-Brown 2019m).

#### Uses in Cameroon.

None reported.

#### Notes.

﻿﻿*Xylopiathomsonii* is a scandent shrub or occasionally a small tree occurring in lowland forests, usually near water. The relatively short pedicels with persistent overlapping bracts, and 1-flowered inflorescences are characteristic. ﻿﻿*Xylopiathomsonii* was formerly included in ﻿﻿*Xylopiaacutiflora* (Dunal) A. Rich., which is now considered to be restricted to West Africa from Sierra Leone to southwestern Ivory Coast. In Cameroon it is most similar to *X.monticola* and ﻿*X.elliotii*; see the key for distinctions among those species.

#### Selected specimens examined.

**Adamaoua Region**: At Sabal Haleo 62 km NE de Tibati, 6.78°N, 13.17°E, *11 April 1983*, *Asonganyi J.N.* 632 (P,YA); A 10 km à l’ouest de Bagodo, 6.42°N, 13.38°E, *28 July 1966*, *Letouzey R.* 7563 (YA); Banyo, 6.75°N, 11.82°E, *08 June 1967*, *Letouzey R.* 8552 (P,YA). **Central Region**: Ndanan I to Ndangan I Forest on path left of road after bridge, site of 25 m × 25 m plot, 3.61°N, 11.59°E, *16 March 2004*, *Cheek M.* 11690 (K,YA); Ca 50 km S of Badjob ca 60 km SW of Eséka Along the Nyong-River, 3.68°N, 10.68°E, *20 March 1964*, *de Wilde W.J.J.O* 2172 (B,BR,K,MO,P,WAG,YA); Syzygeraie près Malandi, 3.57°N, 11.22°E, *06 December 1959*, *Letouzey R.* 2423 (P,YA); Près des lacs de Boubala, 5.75°N, 11.9°E, *13 December 1960*, *Letouzey R.* 2566 (P,YA); Rive boisée de la Sanaga près Mbargue, 4.9°N, 13.08°E, *27 January 1960*, *Letouzey R.* 2824 (P,YA). **East Region**: Bétaré Oya 5 km along road to Bertoua, 5.5°N, 14.1°E, *17 February 1960*, *Breteler F.J.* 1063 (P,WAG,YA); Goyoum, 5.22°N, 13.38°E, *27 January 1961*, *Breteler F.J.* 931 (BR,K,P,WAG); Mbussa, 6°N, 14.38°E, *29 April 1914*, *Mildbraed G.W.J.* 9069 (K); Route Esseleke, 4.58°N, 13.68°E, *15 February 1956*, *Nana P.* 479 (P); Sangha River (international frontier) collected 30 km from Libongo southwards, 2.5°N, 16.06°E, *27 July 1987*, *Thomas D.W.* 7281 (YA). **Littoral Region**: 8 km W of Massok, 4.13°N, 10.47°E, *27 March 1965*, *Leeuwenberg A.J.M.* 5217 (B,BR,C,GC,K,LUAI,MO,P,WAG,YA). **North-West Region**: Anyajua, 6.18°N, 10.36°E, *12 December 1998*, *Cheek M.* 9923 (K,WAG,YA); Bamenda Distr Fonfuka, 6.53°N, 10.48°E, *01 May 1931*, *Maitland T.D.* 1731 (K). **South Region**: Bitya near R Ja, 3.02°N, 12.37°E, *01 January 1921*, *Bates G.L.* 1852 (P); S bank of Lobé R SE of Gr Batanga ferry, 2.88°N, 9.892°E, *11 October 1969*, *Bos J.J.* 5475 (BR,C,K,LD,P,WAG,YA); Ca 10 km SW of Ambam S of Ebolowa On N bank of Ntem-River, 2.38°N, 11.28°E, *02 March 1964*, *de Wilde W.J.J.O* 2043 (B,BR,K,MO,P,WAG,YA); Colline de Mill (5 km NE Lolodorf), 3.23°N, 10.73°E, *26 January 1974*, *Letouzey R.* 12797 (P,YA); Ngoasik (10 km SSE Ambam) rive du Nt, 2.28°N, 11.33°E, *01 March 1963*, *Raynal J.* 10130 (P,YA); Campo-Ma’an National Park, 2.33°N, 10.5°E, *24 August 2001*, *van Andel T.R.* 4023 (KRIBI,WAG). **South-West Region**: Menyum (Bakossi), 4.6°N, 9°E, *28 February 1987*, *Doumenge C.* 313 (MO,P,WAG); Mai Idoanu, 4.24°N, 8.99°E, *12 February 1955*, *Latilo M.G.* 34468 (P); Onge River, 4.3°N, 8.95°E, *20 October 1993*, *Tchouto Mbatchou G.P.* 896 (K,YA); Disturbed forest between Liwenyi and big Koto, 4.3°N, 8.95°E, *22 October 1993*, *Tchouto Mbatchou G.P.* 942 (K,YA); Korup National Park, 5.05°N, 8.8°E, *28 February 1984*, *Thomas D.W.* 3242 (MO,YA); Korup National Park, 4.98°N, 8.85°E, *12 January 1979*, *Thomas D.W.* 595 (K). **West Region**: Baleng, 5.53°N, 10.38°E, *10 May 1964*, *de Wilde W.J.J.O* 2565 (WAG).

**Figure 153.**
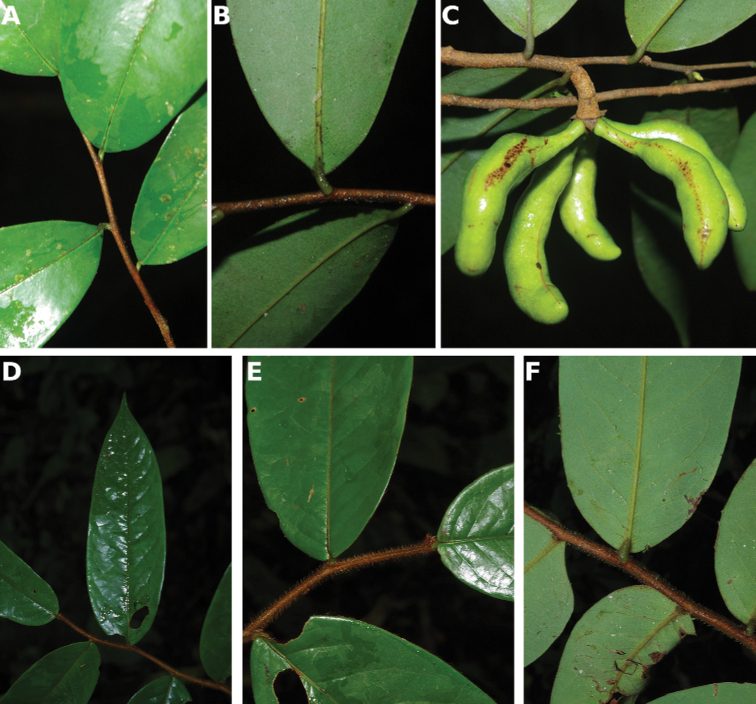
*Xylopiathomsonii***A** base of leaf blade, upper side **B** base of leaf blade, lower side **C** fruit. *Xylopiavillosa* (*cf*, young sterile individual) **D** leaf, upper side **E** base of leaf blade, upper side **F** base of leaf blade, lower side **A–C***Couvreur. 520*, Gabon **D, E***Couvreur 1227*, Maséa, Cameroon. Photos Thomas L.P. Couvreur.

### ﻿﻿﻿ Xylopia villosa

Taxon classificationPlantaeMagnolialesAnnonaceae

Chipp, Bull. Misc. Inform. Kew: 183, 1923

F0F3081D-C67A-58FC-B75B-F61AB5CC9EE0

Fig. 153 ; Map 1E

≡ Xylopiastrum villosum (Chipp) Aubrév., Flor. For. Côte d’Ivoire, ed. 2, 1: 140, 1959.

#### Type.

Nigeria. Lagos State; Ibadan Forest Reserve, Lagos, *Punch C. 119*, Dec 1913: lectotype, designated by Johnson and Murray (2018), p. 215: K[000199069].

#### Description.

Tree, up to 30 m tall, d.b.h. up to 90 cm; **buttresses narrowly concave ca. 1 m high**. Old branches sparsely pubescent to glabrous, **young branches densely villous, with erect orange or reddish brown hairs 0.5–1.3 mm long**. Leaves: petiole 2–4 mm long, ca. 2 mm wide, pubescent, grooved, blade inserted on top of the petiole; blade 8.6–12.6 cm long, 2.6–4.1 cm wide, lanceolate to lanceolate-elliptic or oblong-lanceolate, apex acuminate to acute, acumen 0.4–1.3 cm long, base cuneate to rounded, subcoriaceous, **below golden-sericeous when young, sparsely golden-sericeous to golden-sericeous when old**, above glabrous when young and old, discolorous; midrib impressed, above pubescent when young, glabrous to pubescent when old, below glabrous to pubescent when young, pubescent when old; secondary veins 10 to 15 pairs, glabrous above; tertiary venation reticulate. Individuals bisexual; inflorescences ramiflorous on young foliate branches, axillary, peduncle 8.5–17 mm. Flowers with 9 perianth parts in 3 whorls, 1 to 8 per inflorescence; pedicel 2–5 mm long, ca. 2 mm in diameter, densely pubescent; in fruit 6–42 mm long, 7–11 mm in diameter, glabrous; bracts 2 to 4, evenly spaced, 3–4 mm long, 3–4 mm wide; sepals 3, valvate, **basally to ½ fused, forming a cup**, 4–6 mm long, 3–4 mm wide, ovate, apex acute, base truncate, densely pubescent outside, glabrous inside; petals free, subequal; outer petals 3, 22–34 mm long, 3.5–3.9 mm wide at base, linear, apex acute to obtuse, base broad and concave, light yellow, pubescent, base glabrous outside, pubescent, base glabrous inside; inner petals 3, valvate, 17.4–23 mm long, 2.5–3.6 mm wide at base, linear, apex acute, base broad and concave, light yellow, pubescent with glabrous base outside, pubescent towards base to pubescent and glabrous towards center inside; stamens ca. 200, in 4 to 6 rows, 1–2 mm long, oblong; connective apex shield-like, glabrous; carpels 10 to 12, ovary 1–2 mm long, stigmas connivent with tips free, linear, 2.5–4 mm long, pubescent to sparsely pubescent. Monocarps stipitate, stipe ca. 3 mm long, ca. 9 mm in diameter; monocarps 1 to 10, ca. 46 mm long, ca. 23 mm wide, oblongoid, apex rounded, sparsely pubescent, verrucose and wrinkled when dried, green outside, endocarp color unknown; seeds unknown (sarcotesta blue?).

#### Distribution.

A widespread species in West Africa from Liberia to Ghana, and in Central Africa from southern Nigeria to Cameroon; in Cameroon known from East, South, Central and South-West regions. Given the past confusion with ﻿*X.letestui*, it is difficult to state the full distribution of ﻿*X.villosa* precisely.

#### Habitat.

A large tree species not commonly collected, although locally common westward (O. Lachenaud, personal communication); in evergreen or semi-deciduous rain forests of Sterculiaceae and Ulmaceae, and old secondary forest with *Lophiraalata*, *Coulaedulis*, and *Sacoglottisgabonensis*. Altitude 0–100 m a.s.l.

#### Local and common names known in Cameroon.

oyakwi (*Letouzey 9524*, Yaoundé).

#### IUCN conservation status.

Least Concern (LC) (Botanic Gardens Conservation International and IUCN SSC Global Tree Specialist Group 2019h).

#### Uses in Cameroon.

None reported.

#### Notes.

﻿﻿*Xylopiavillosa* and ﻿*X.letestui* share thick pubescent leaves but the former differs in the broadly cuneate to rounded rather than truncate leaf bases, the longer petioles (2–4 versus 1–1.8 mm), and the longer outer petals (22–34 versus 16.5–19.1 mm). ﻿﻿*Xylopiavillosa* also lacks the marginal hair tufts toward the base of the inner petals. The specimen *Thomas 7703*, which consists of fallen fruits containing seeds, reported the seeds to be “blue,” suggesting the presence of a sarcotesta.

#### Specimens examined.

**Central Region**: près Nkomeyo 10 km d’Esse, 4.07°N, 11.97°E, *07 November 1969*, *Letouzey R.* 9524 (P,YA). **East Region**: 65 km south of Yokadouma 30 km after Ngato 15 km after river ALPICAM ‘base de vie’ then 40 km on forestry road starting 4 km before Maséa village, 3.08°N, 14.67°E, *08 March 2019*, *Couvreur T.L.P.* 1227 (MPU,WAG,YA). **South Region**: Campo Ma’an National Park 11 km on trail from Ebinanemeyong village on road 7 km from Nyabessan to Campo town, 2.49°N, 10.34°E, *12 February 2015*, *Couvreur T.L.P.* 686 (WAG,YA); 3 km E of km 58 of road Edéa-Kribi, 3.72°N, 10.3°E, *05 October 1965*, *Leeuwenberg A.J.M.* 6815 (BR,K,MO,P,WAG,YA). **South-West Region**: Korup National Park, 5.26°N, 9.2°E, *08 April 1988*, *Thomas D.W.* 7703 (MO).

##### ﻿Unresolved names

###### ﻿﻿*Uvariabusgenii* Unwin (non Diels), West African Forests & Forestry: 263, 1920

This species was published by Unwin in his West African Forests & Forestry book (Unwin 1920) suggesting it grows in the region of Johann-Albrechtshöhe (now Kumba, South-West region). Unwin states (p. 263) it is a common “large tree” and wood is used to make “European” houses in the Calabar region of Nigeria. The name is accompanied by the description of the plant (mainly the wood) and its uses. However, no specimen is listed. The species is presumably named in honor of the German collector Moritz Büsgen (1858–1921), who collected in SW Cameroon in 1908. It is possible that Unwin could have seen a specimen collected by Büsgen. Specimens of Büsgen are deposited in Berlin (B) and four collections of Annonaceae species are available online. Only one refers to an ﻿*Uvaria* collected in Johann-Albrechtshöhe [B 10 0153104, *Büsgen 191*]. This specimen is however the holotype (and only specimen) of ﻿﻿*Uvariamarginata* Diels (now synonym of ﻿*U.obanensis*) and does not correspond to a tree as stated in the description.

﻿*Uvaria* species are generally scrambling shrubs or lianas. However, several tree genera were initially included in ﻿*Uvaria* based on flower characters, such as ﻿*Uvariodendron* (“section ﻿*Uvariodendron*” within ﻿*Uvaria* in Engel and Diels (1901)) or ﻿*Hexalobus* (Botermans et al. 2011). These were generally erected to genus status afterwards (Candolle 1832, in the case of ﻿*Hexalobus*, Fries 1930, in the case of ﻿*Uvariodendron*). Thus, ﻿﻿*Uvariabusgenii* certainly refers to a species in a different genus than ﻿*Uvaria*, possibly ﻿*Uvariodendron*. However, without further material it will be hard to confirm this.

###### ﻿﻿*Uvariabusgenii* Diels (non Unwin), nom. nud.

This name was first (?) published in Gilg (1909) page 124, although it doesn’t appear to be the description of a species per se. Only a local name is provided and the indication that it occurs in North Cameroon, and represents a tree. The name has also been used in various other publications (e.g. Wiesner 1918, pages 558, 762). We were however unable to find the original publication describing this name, nor does it appear on IPNI. This name does not refer to the same species as Unwin (see above) as it occurs in North Cameroon. It is probably a manuscript name that was never published, although we have not found any herbarium sheets with this name marked on it yet. Northern Cameroon harbors very few Annonaceae species, and it is hard to see what species or even genus Gilg is referring too here. The common name is “bongele”, but this name is attributed to *Eribromaoblongum* (Mast.). Pierre ex A.Chev.) now a synonym of *Sterculiaoblonga* Mast. (Malvaceae).

## ﻿Appendix 1. Index to numbered collections

﻿*Afroguatteria* = *Af.*; ﻿*Annickia* = *Anki.*; ﻿*Annona* = *Anna.*; ﻿*Anonidium* = *Andi.*; ﻿*Artabotrys* = *Ar.*; ﻿*Brieya* = *B.*; ﻿*Cleistopholis* = *Cl.*; ﻿*Dennettia* = *De.*; ﻿*Duguetia* = *Du.*; ﻿*Greenwayodendron* = *Gr.*; ﻿*Hexalobus* = *H.*; ﻿*Isolona* = *I.*; ﻿*Letestudoxa* = *L.*; ﻿*Meiocarpidium* = *Me.*; ﻿*Mischogyne* = *Mi.*; ﻿*Monanthotaxis* = *Monan.*; ﻿*Monodora* = *Mo.*; ﻿*Neostenanthera* = *Ne.*; ﻿*Piptostigma* = *Pi.*; ﻿*Polyceratocarpus* = *Po.*; ﻿*Sirdavidia* = *Si.*; ﻿*Sphaerocoryne* = *Sp.*; ﻿*Toussaintia* = *T.*; ﻿*Uvaria* = *U.*; ﻿*Uvariastrum* = *Uvtr.*; ﻿*Uvariodendron* = *Uvdr.*; ﻿*Uvariopsis* = *Uvdr.*; ﻿*Xylopia* = *X.*

Achoundong G. 1385, 3419 (*Anna.senegalensis*), 1325 (*Andi.mannii*), 1056 (*Mo.zenkeri*), 1267 (*Pi.goslineanum*), 893 (*Pi.multinervium*), 1332 (*Po.parviflorus*), 786, 790 (﻿*U.comperei*), 872 (*Uvdr.congensis*).

Acworth J.M. 289 (*Pi.longepilosum*), 182 (﻿*U.obanensis*).

Adebusuyi J.K. 44049 (﻿*C.patens*).

Akogo M. 34 (*Pi.oyemense*), 234 (*Uvdr.molundense*).

Annet E. 174 (*Anki.polycarpa*), 359 (﻿*I.zenkeri*), 348 (*Monan.elegans*), 420 (*Pi.longepilosum*), 351 (*Uvdr.zenkeri*), 319 (﻿*X.quintasii*).

Ashworth J. 310 (﻿*B.fasciculata*), 196 (﻿*I.congolana*).

Asonganyi J.N. 275, 310 (﻿*N.myristicifolia*), 711 (*Po.parviflorus*), 421 (*Uvdr.calophyllum*), 632 (﻿﻿*X.thomsonii*).

Aubréville A. 729, 787, 804 (*Anna.senegalensis*), 2115 (﻿*B.fasciculata*), 41 (﻿*C.patens*), 628 (﻿*U.chamae*), 133 (﻿*X.aethiopica*), 125 (﻿﻿*X.rubescens*).

Baker W.J. 294 (*Uvdr.connivens*).

Bamps P.R.J. 1679 (*Du.dilabens*), 1381 (*Monan.hirsuta*), 1449 (*Mo.undulata*), 1458 (*Uvdr.dioica*), 1376 (﻿*X.quintasii*).

Bates G.L. 1959 (*Anki.affinis*), 1792 (*Ar.insignis*), 1699 (*Ar.jacquesfélicis*), 1763 (*Ar.thomsonii*), 1199 (*Du.staudtii*), 1244 (*Mo.zenkeri*), 1818 (﻿*U.mollis*), 1764 (*Uvtr.pierreanum*), 1317 (﻿﻿*X.rubescens*), 1852 (﻿﻿*X.thomsonii*).

Biholong M. 28 (*Anna.senegalensis*), 219 (﻿*C.glauca*), 279 (*Uvdr.fuscum* var. giganteum).

Binuyo A. 35606 (*Andi.brieyi*), 35564 (*Uvdr.calophyllum*).

Bongyu J. 42 (*Uvtr.zenkeri*).

Bos J.J. 6894 (*Anki.chlorantha*), 4962 (*Anki.letestui*), 4784 (﻿*C.glauca*), 6383 (﻿*C.staudtii*), 3844, 5048, 6128 (*Du.confinis*), 6267 (﻿*G.glabrum*), 4769, 6100 (﻿*G.suaveolens*), 4495, 5157, 5370 (﻿*H.bussei*), s.n. (﻿*H.salicifolius*), 4947, 7069 (﻿*I.campanulata*), 6298 (﻿*I.pleurocarpa*), 4866, 6522 (﻿*I.zenkeri*), 6523 (*Me.oliverianum*), 6735 (*Monan.bicornis*), 4069, 6037, 6293, 6867 (*Monan.cauliflora*), 5818, 6653 (*Monan.enghiana*), 3854 (*Monan.whytei*), 6224, 7199 (*Mo.crispata*), 4194, 4526 (*Mo.myristica*), 6365 (*Mo.tenuifolia*), 6415, 6594 (*Pi.glabrescens*), 6683 (*Pi.longepilosum*), 4647 (*Pi.macranthum*), 4625, 4793, 7163 (*Pi.multinervium*), 6679, 6684 (*Po.microtrichus*), 5049 (*Po.pellegrinii*), 3077, 3602, 4180, 4655, 5264 (*Sp.gracilipes*), 4794 (﻿*U.bipindensis*), 6652 (﻿*U.muricata*, 5555 (﻿*U.obanensis*), 3164, 4518, 5011, 5508 (﻿﻿*U.scabrida*), 6266 (*Uvtr.zenkeri*), 5412 (*Uvdr.connivens*), 3259, 5474, 6521, 7075 (*Uvdr.molundense*), 3341, 6382 (﻿*X.aethiopica*), 4772 (﻿*X.mildbraedii*), 4773 (﻿*X.quintasii*), 4650 (﻿*X.talbotii*), 5475.(﻿﻿*X.thomsonii*).

Box H.E. 3556 (*De.tripetala*).

BPFV 2664 (*Me.oliverianum*).

Brenan J.P.M. 9410 (*Andi.brieyi*), 9305, 9409 (*Uvdr.bakeriana*).

Breteler F.J. 1185 (*Anna.senegalensis*, 2454 (*Andi.mannii*), 968, 1429, 2210, 2799 (*Ar.aurantiacus*), 2956 (*Ar.insignis*, 1752, 1825, 2717, 2825 (*Ar.rufus*), 1725, 1797 (*Ar.thomsonii*), 2013 (*Du.barteri*), 1841 (*Du.staudtii*), 1398, 1817, 2646 (*Me.oliverianum*), 2692 (*Monan.bicornis*), 2137 (*Monan.enghiana*), 1374 (*Monan.filamentosa*), 1874 (*Monan.letouzeyi*), 2182 (*Mo.undulata*), 1319, 2683, 2747 (*Mo.zenkeri*), 644, 684, 1220, 1799 (﻿*N.myristicifolia*), 432 (﻿*U.angolensis*), 1977 (﻿*U.muricata*, 2880 (﻿﻿*U.scabrida*), 2812 (*Uvdr.congensis*), 2398 (﻿*X.quintasii*), 931, 1063.(﻿﻿*X.thomsonii*).

Brunt M.A. 51 (*Anna.senegalensis*), 261 (﻿*X.elliotii*).

Buchholz R.W. 103 (﻿*U.buchholzii*).

Büsgen M. 191 (﻿*U.obanensis*).

Busse W.C.O. 3216 (﻿*H.bussei*).

Cable S. 2801 (*Andi.mannii*), 1309, 1310 (*Ar.aurantiacus*), 332, 475, 2851, 3804, 3894 (*Ar.congolensis*), 1825 (﻿*C.staudtii*), 2843 (﻿*I.congolana*), 404 (*Monan.congoensis*), 2523, 3683 (*Monan.enghiana*), 1185, 1581, 2575 (*Monan.filamentosa*), 1169 (*Monan.foliosa*), 2544 (*Mo.undulata*), 2279 (﻿*N.neurosericea*), 787 (*Pi.goslineanum*), 2721 (*Po.parviflorus*), 3526 (*Sp.gracilipes*), 2500, 2674, 3344, 3459, 3558, 3809 (﻿*U.heterotricha*), 611, 2230 (*Uvdr.connivens*), 1353, 1524, 1626, 2187 (*Uvdr.fuscum*), 2150, 2256 (*Uvdr.bakeriana*), 1221, 2736 (*Uvdr.submontana*), 2870, 3814 (﻿*X.africana*).

Cheek M. 8297, 9354, 9654 (*Anki.affinis*), 10240 (*Andi.brieyi*), 6009, 7896, 11248 (*Andi.mannii*), 9063 (*Ar.aurantiacus*), 3470 (*Ar.congolensis*), 11641 (*Ar.insignis*), 17781 (*Ar.thomsonii*), 9143 (*Ar.velutinus*), 10229 (﻿*C.glauca*), 7915 (﻿*C.staudtii*), 8274 (*Du.barteri*), 27, 66, 11224 (﻿*G.suaveolens*), 9716, 11064 (﻿*H.crispiflorus*), 7711, 11657 (*Monan.cauliflora*), 11499 (*Monan.enghiana*), 7794, 7880 (*Monan.foliosa*), 10154 (*Monan.glaucifolia*), 11504 (*Monan.sterilis*), 9067, 9202 (*Monan.submontana*), 10228, 10357 (*Mo.myristica*), 5840, 9399 (*Mo.tenuifolia*), 11464, 12928 (*Mo.undulata*), 11965 (﻿*N.myristicifolia*), 8198 (*Pi.fugax*), 5071, 7230 (*Pi.glabrescens*), 7849, 8328, 10197 (*Pi.goslineanum*), 9177 (*Pi.submontanum*), 8244 (*Po.parviflorus*), 8307 (﻿*U.angolensis*), 8950 (﻿*U.anisotricha*), 3014 (﻿*U.buchholzii*), 7457, 8863 (﻿*U.heterotricha*), 10415 (﻿*U.muricata*, 86 (*Uvtr.pierreanum*), 9337 (*Uvdr.calophyllum*), 5180, 5462, 8164 (*Uvdr.connivens*), 5145 (*Uvdr.fuscum*), 11839 (*Uvdr.molundense*), 7234 (*Uvdr.bakeriana*), 5482, 5501 (*Uvdr.dioica*), 5486, 8258, 8815 (*Uvdr.korupensis*), 11606 (*Uvdr.solheidii*), 7034, 7131 (*Uvdr.submontana*), 11579, 11999 (﻿*X.aethiopica*), 7605, 9192, 10203, 10527 (﻿*X.africana*), 11487 (﻿*X.hypolampra*), 9923, 11690.(﻿﻿*X.thomsonii*).

Chevalier A.J.B. 33132 (*Anki.chlorantha*), 33473 (﻿*C.glauca*).

CNAD 317, 808.(*Anna.senegalensis*).

Conrau G. 93 (﻿*I.campanulata*).

Couvreur T.L.P. 426, 437, 469, 492, 519, 621, 657, 671, 1153, 1203 (*Anki.affinis*), 414 (*Anki.chlorantha*), 106, 415, 452, 609, 696, 987, 1207, 1053b (*Andi.mannii*), 431 (*Ar.congolensis*), 453 (*Ar.dielsianus*), 710, 1044, 1214 (*Ar.insignis*), 418, 1211 (*Ar.rufus*), 751, 1020, 1230 (*Ar.thomsonii*), 510-511, 677, 957, 1231 (﻿*B.fasciculata*), 389, 430 (﻿*C.glauca*), 1202 (﻿*C.patens*), 393 (*Du.barteri*), 692 (*Du.dilabens*), 460, 489, 1014 (*Du.staudtii*), 658, 756, 1002, 1180 (﻿*G.suaveolens*), 446, 506, 666, 1062, 1197 (﻿*H.crispiflorus*), 1054 (﻿*I.congolana*), 495 (﻿*I.hexaloba*), 402 (﻿*I.pleurocarpa*), 697-698, 1148 (﻿*L.lanuginosa*), 376, 407, 709, 1182, 1193 (*Me.oliverianum*), 1033 (*Mi.gabonensis*), 676, 705 (*Monan.cauliflora*), 626, 1018, 1025 (*Monan.congoensis*), 762 (*Monan.couvreurii*), 629 (*Monan.diclina*), 704 (*Monan.elegans*), 466, 618, 653, 669, 691, 754, 986, 1003, 1037, 1065, 1176, 1201 (*Monan.enghiana*), 417, 690, 695, 989 (*Monan.filamentosa*), 1175 (*Monan.hirsuta*), 981, 1056 (*Monan.laurentii*), 458, 752 (*Monan.letouzeyi*), 651, 708 (*Monan.paniculata*), 628 (*Monan.sterilis*), 1049 (*Monan.vulcanica*), 461, 477 (*Mo.angolensis*), 412, 445, 514, 608, 1050, 1055, 1155 (*Mo.myristica*), 1019, 1218, 1233 (*Mo.tenuifolia*), 391, 1042, 1061 (*Mo.undulata*), 422 (﻿*N.myristicifolia*), 384, 386 (﻿*N.neurosericea*), 675, 678 (﻿*N.robsonii*), 1167b (*Pi.calophyllum*), 681, 693 (*Pi.fugax*), 1158 (*Pi.glabrescens*), 983, 1017, 1023, 1030, 1047 (*Pi.goslineanum*), 518, 637, 1034 (*Pi.macrophyllum*), 672 (*Pi.mayndongtsaeanum*), 436, 667 (*Pi.mortehanii*), 616, 649 (*Pi.multinervium*), 1204 (*Pi.pilosum*), 625 (*Pi.submontanum*), 515, 623, 632, 645, 1032, 1036, 1174 (*Po.parviflorus*), 1178 (*Po.pellegrinii*), 617, 633, 1001, 1043 (*Sp.gracilipes*), 959 (﻿*U.anisotricha*), 1208 (﻿*U.anonoides*), 662, 673, 1179 (﻿*U.bipindensis*), 982 (﻿﻿*U.scabrida*), 385, 454, 680 (*Uvtr.pierreanum*), 624 (*Uvtr.zenkeri*), 486, 980, 999, 1013, 1157 (*Uvdr.calophyllum*), 383, 484, 1051 (*Uvdr.connivens*), 990, 992, 1026, 1029, 1040, 1046 (*Uvdr.fuscum*), 419, 512, 1057, 1206, 1229 (*Uvdr.fuscum* var. giganteum), 652, 655-656, 1172 (*Uvdr.molundense*), 1000, 1015, 1045 (*Uvdr.bakeriana*), 654, 659 (*Uvdr.dioica*), 1039, 1052 (*Uvdr.korupensis*), 517, 1063, 1066, 1173 (*Uvdr.pedunculosa*), 627, 682, 965, 1059 (*Uvdr.submontana*), 707, 978, 1027 (*Uvdr.zenkeri*), 471, 516, 1021, 1223 (﻿*X.aethiopica*), 949, 967, 993 (﻿*X.africana*), 485 (﻿*X.cupularis*), 420, 450, 467, 694, 1198 (﻿*X.hypolampra*), 475, 688 (﻿*X.letestui*), 1164, 1222 (﻿*X.phloiodora*), 483, 500 (﻿*X.quintasii*), 686, 1227 (﻿*X.villosa*).

Dahl A. 622 (*Uvdr.fuscum*).

Dang D. 681 (*Pi.multinervium*), 643 (*Uvdr.molundense*).

de Bruijn J. s.n. (*Ar.aurantiacus*), s.n. (*Du.barteri*).

de Kruif A.P.M. 1046 (﻿*C.glauca*), 998 (﻿*I.hexaloba*), 871 (﻿*T.hallei*).

de Wilde J.J.F.E 7930 (*Andi.mannii*), 7947 (﻿*C.staudtii*), 8465 (﻿*G.glabrum*), 7940, 12163 (﻿*G.suaveolens*), 7909 (﻿*H.crispiflorus*), 8088 (﻿*I.hexaloba*), 7576 (*Me.oliverianum*), 1987 (*Monan.cauliflora*), 8330 (*Monan.diclina*), 8371 (*Monan.ferruginea*), 7836, 8399 (*Mo.tenuifolia*), 2249 (*Mo.zenkeri*), 8453a (*Pi.glabrescens*), 8718 (*Po.pellegrinii*), 2579 (﻿*U.angolensis*), 1904, 7972 (*Uvtr.pierreanum*), 7754, 8270a, 8709 (*Uvdr.dioica*), 8373 (*Uvdr.solheidii*), 7931, 7970, 8085, 8707 (﻿*X.aethiopica*), 7963 (﻿*X.hypolampra*), 2838a, 2838b (﻿*X.phloiodora*), 1320, 7871, 7889 (﻿*X.quintasii*), 8166 (﻿﻿*X.rubescens*), 7941, 8029, 12161 (﻿﻿*X.staudtii*).

de Wilde W.J.J.O 2359, 2589 (*Anna.senegalensis*, 2389, 2966 (*Anna.senegalensis*), 1968 (*Andi.mannii*), 1602, 2720 (*Ar.aurantiacus*), 2779, 3715 (*Ar.rufus*), 1342 (﻿*C.staudtii*), 2133 (﻿*G.suaveolens*), 1942, 2735 (*Me.oliverianum*), 1987 (*Monan.cauliflora*), 2709, 2787 (*Monan.elegans*), 2840 (﻿*N.neurosericea*), 2132 (*Pi.macranthum*), 1476 (*Pi.mortehanii*), 4361 (﻿*U.angolensis*), 2164, 4049 (﻿*U.muricata*, 1199 (﻿﻿*U.scabrida*), 1914 (*Uvdr.congensis*), 4447 (﻿*X.elliotii*), 2676 (﻿*X.longipetala*), 1320 (﻿*X.quintasii*), 2043, 2172, 2565.(﻿﻿*X.thomsonii*).

de Wit H.C.D 7182 (*Anna.senegalensis*), 7948 (*Me.oliverianum*).

Deistel H. 151, 454 (﻿*X.africana*).

Dong E. 391 (*Anna.senegalensis*), 393 (﻿*H.monopetalus*).

Doumenge C. 554 (*Monan.submontana*), 473 (*Uvdr.fuscum* var. giganteum), 313 (﻿﻿*X.thomsonii*).

Droissart V. 1416, 2158 (*Andi.mannii*), 834, 2050, 2159 (﻿*G.suaveolens*), 1881 (*Po.microtrichus*), 1221 (*Po.parviflorus*), 2125 (*Uvdr.fuscum* var. giganteum), 1880 (*Uvdr.molundense*), 2049 (﻿*X.pynaertii*).

Dundas J. 13989 (*Uvdr.calophyllum*).

Ekema S.N. 1208 (﻿*G.suaveolens*), 939 (*Monan.congoensis*), 862 (*Pi.longepilosum*), 950 (*Po.microtrichus*), 1078 (*Uvdr.connivens*), 944 (*Uvdr.zenkeri*).

Elad M. 132, 510 (*Ar.aurantiacus*), 580 (*Ar.insignis*), 443 (﻿*G.suaveolens*), 1269, 1545 (﻿*I.zenkeri*), 444 (*Mo.myristica*), 86 (*Po.parviflorus*), 1535 (﻿*U.baumannii*), 564 (﻿*U.obanensis*), 1270 (*Uvdr.molundense*), 69 (*Uvdr.submontana*), 90 (﻿*X.africana*), 1253 (﻿*X.mildbraedii*).

Endengle E. 116 (﻿*N.myristicifolia*), 2061 (*Pi.glabrescens*), s.n. (*Uvtr.zenkeri*).

Endengle E. SRFK 2121 (﻿*I.hexaloba*).

Etuge M. 2049, 4235, 4850, 5139 (*Anki.affinis*), 5373 (*Ar.aurantiacus*), 485 (*Ar.thomsonii*), 1844 (﻿*B.fasciculata*), 4873, 4917 (﻿*C.glauca*), 2884 (﻿*C.staudtii*), 1794, 2000, 4488, 5431 (﻿*I.congolana*), 1191, 1729, 2377, 4531, 4919 (*Monan.filamentosa*), 1748 (*Monan.foliosa*), 4676 (*Monan.laurentii*), 4122, 4442 (*Monan.submontana*), 4810 (*Monan.vulcanica*), 1576, 2516 (*Mo.myristica*), 2017 (*Mo.undulata*), 6447 (﻿*N.neurosericea*), 4853 (*Pi.fugax*), 2420, 2698 (*Pi.goslineanum*), 4504 (﻿*U.bipindensis*), 1819, 2092, 2659 (﻿*U.heterotricha*), 1888 (﻿﻿*U.scabrida*), 156, 2396, 6506 (*Uvdr.connivens*), 4860 (*Uvdr.bakeriana*), 2562, 6482 (*Uvdr.submontana*), 148 (﻿*X.aethiopica*), 1160, 1441, 1740 (﻿*X.africana*), 56 (﻿*X.quintasii*).

Farron C. 7335 (*Anki.chlorantha*), 7266, 7359 (*Monan.couvreurii*), 6613, 7297 (*Uvdr.bakeriana*).

Fay J.M. 8299 (﻿*H.crispiflorus*), 8307 (﻿*X.longipetala*).

Fenton E. 163 (﻿*X.aethiopica*).

Fleury F. 33400 (*Pi.calophyllum*), 33134 (*Pi.goslineanum*), 33338 (﻿*X.aethiopica*), 33135 (﻿﻿*X.rubescens*).

Fogiel M.K. 947 (﻿*C.patens*), 1039 (*Uvtr.zenkeri*), 2098 (﻿*X.letestui*), 864 (﻿﻿*X.staudtii*).

Fotius G. 2144, 2660 (*Ar.velutinus*), 2738 (﻿*U.angolensis*), 1995, 2173 (﻿*U.chamae*), 2984, 3108 (﻿*X.elliotii*).

Foury P. 57 (﻿*C.glauca*), 69 (*Du.staudtii*), 129 (﻿*G.suaveolens*), 73 (*Pi.mortehanii*), 101 (﻿*X.hypolampra*), 36 (﻿*X.quintasii*), 46 (﻿﻿*X.staudtii*).

Geerling C. 5666 (﻿*U.chamae*).

Gentry A.H. 52942 (*Ar.aurantiacus*), 52947 (*Monan.laurentii*), 62649 (﻿*X.phloiodora*).

Gereau R.E. 5185 (﻿*N.neurosericea*), 5195 (*Uvdr.bakeriana*), 5192 (*Uvdr.korupensis*).

Ghogue J.-P. 500 (*Monan.glaucifolia*), 1487 (*Mo.myristica*).

Gosline W.G. 99 (*Ar.aurantiacus*), 241 (*Ar.congolensis*), 149 (*Ar.velutinus*), 234 (﻿*B.fasciculata*), 240 (﻿*C.staudtii*), 289 (﻿*I.campanulata*), 175 (*Monan.cauliflora*), 300 (*Monan.congoensis*), 254 (*Monan.foliosa*), 198, 256 (*Pi.submontanum*), 236, 237 (*Po.parviflorus*), 147 (﻿*U.anisotricha*), 83 (﻿*U.heterotricha*), 209 (*Uvdr.connivens*), 244 (*Uvdr.pedunculosa*), 423 (﻿*X.aethiopica*), 75 (﻿*X.africana*).

Groves M. 21, 77 (*Ar.aurantiacus*), 122 (*Uvdr.fuscum*).

Hallé F. 4220 (*Du.staudtii*), 4181 (﻿*H.salicifolius*), 4247 (﻿*X.letestui*), 4240 (﻿*X.quintasii*).

Harris D.J. 883, 1518 (﻿*H.crispiflorus*), 2516 (﻿*N.myristicifolia*), 6200, 6528 (﻿*U.poggei*), 5889, 6135 (*Uvtr.pierreanum*), 1512 (*Uvdr.congensis*), 752, 1846 (﻿*X.aurantiiodora*), 757 (﻿*X.longipetala*).

Hédin L. 409 (*Anna.senegalensis*), 36, 1337 (*Andi.mannii*), 1690 (*Du.confinis*), 1646 (*Du.staudtii*).

Hoshino J. 359 (*Pi.multinervium*).

Huber H.F.J. 982 (﻿*U.angolensis*).

Jacques-Félix H. 3026, 3033, 3136, 3423, 4012 (*Anna.senegalensis*, 3388, 3737 (*Anna.senegalensis*), 5211 (*Ar.aurantiacus*), 2490 (*Ar.jacquesfélicis*), 4630 (﻿*C.patens*), 4904 (*Me.oliverianum*), 3226 (*Monan.laurentii*), 3078 (*Monan.vulcanica*), 2340, 4803 (﻿*N.myristicifolia*), 2493 (*Uvdr.dioica*), 2965, 3039, 8724, 9110.(﻿*X.elliotii*).

Jaff B. 73 (*Uvdr.connivens*).

Kaji M. 4 (﻿*G.glabrum*).

Kamdem N. 143, 422, 521 (*Anki.affinis*), 142, 415, 454 (*Andi.mannii*), 166, 175, 329, 349, 397, 459, 510, 537, 560 (﻿*G.suaveolens*), 169 (*Me.oliverianum*), 164 (*Monan.cauliflora*), 167, 465, 499 (*Monan.enghiana*), 483 (*Mo.tenuifolia*), 295 (*Pi.calophyllum*), 159 (*Pi.oyemense*), 557 (*Uvdr.fuscum* var. giganteum), 177 (﻿*X.aethiopica*).

Keay R.W.J. 37372 (*Pi.pilosum*), 297 (﻿*U.heterotricha*), 37524 (*Uvdr.angustifolium*), 37485 (*Uvdr.fuscum*).

Kenfack D. 984 (﻿*C.staudtii*), 1063 (﻿*I.cooperi*), 1545 (*Mi.gabonensis*), 1027 (*Mo.undulata*), 1008 (*Uvtr.zenkeri*), 1026, 1620 (*Uvdr.korupensis*), 1334, 1602 (*Uvdr.submontana*), 1187 (﻿*X.quintasii*).

Koufani A. 123 (﻿*I.dewevrei*), 154 (*Uvdr.solheidii*).

Kwangue A.T. 22 (*Monan.congoensis*).

Lachenaud O.L. 659 (﻿*N.robsonii*).

Lane P. 532 (﻿*I.congolana*), 142 (*Uvdr.fuscum*), 501 (*Uvdr.fuscum* var. giganteum), 490 (*Uvdr.submontana*).

Latilo M.G. 34468 (﻿﻿*X.thomsonii*).

Ledermann C.L. 2462 (*Mo.angolensis*).

Leeuwenberg A.J.M. 7355, 7787 (*Anki.affinis*), 9440 (*Anna.senegalensis*, 8319 (*Ar.aurantiacus*), 6011 (*Ar.rufus*), 5201 (*Ar.thomsonii*), 6467 (﻿*C.glauca*), 9032, 9785, 9828 (﻿*C.patens*), 5377 (*Du.barteri*), 5582 (﻿*G.glabrum*), 5755, 8164 (﻿*G.suaveolens*), 9904 (﻿*H.crispiflorus*), 9550 (﻿*I.congolana*), 9784 (﻿*I.pleurocarpa*), 7716 (*Monan.bokoli*), 5088 (*Monan.dielsiana*), 5828 (*Monan.tripetala*), 10034 (*Mo.angolensis*), 10295 (*Mo.undulata*), 8354 (*Pi.glabrescens*), 10160 (﻿*U.angolensis*), 9249 (﻿*U.heterotricha*), 5282 (*Uvdr.calophyllum*), 5504 (﻿*X.aethiopica*), 9915 (﻿*X.cupularis*), 7033 (﻿*X.longipetala*), 8362 (﻿*X.mildbraedii*), 5217 (﻿﻿*X.thomsonii*), 6815.(﻿*X.villosa*).

Lehmbach H. 224 (﻿*I.congolana*), 57, 178 (*Uvdr.fuscum*).

Letouzey R. 5412, 11751, 12361 (*Anki.affinis*), 606, 3187, 3191 (*Anna.senegalensis*, 6779, 13988 (*Anna.senegalensis*), 194, 1276, 1385, 1547, 11202 (*Andi.mannii*), 3309, 3772, 4859, 12141, 13495 (*Ar.aurantiacus*), 12016 (*Ar.congolensis*), 10370 (*Ar.insignis*, 9607 (*Ar.insignis*), 5147 (*Ar.rufus*), 4435, 5533, 5548, 10413, 11200, 12240 (*Ar.thomsonii*), 5248, 7481, 7826, 10040, 13501 (*Ar.velutinus*), 5605, 9132, 10718, 12345 (﻿*B.fasciculata*), 3036 (﻿*C.glauca*), 10523 (﻿*C.patens*), 4149 (﻿*C.staudtii*), 304 (*Du.barteri*), 12585 (*Du.confinis*), 10181, 10509, 11867 (*Du.staudtii*), 12869 (﻿*G.glabrum*), 1106, 1313, 5322, 9198, 11866 (﻿*G.suaveolens*), 9387 (﻿*H.bussei*), 4433, 10614 (﻿*H.crispiflorus*), 6540, 7301 (﻿*H.monopetalus*), 8122 (﻿*H.salicifolius*), 15078 (﻿*I.campanulata*), 13984 (﻿*I.dewevrei*), 2392, 5072, 10419, 10703, 11840, 14910 (﻿*I.hexaloba*), 9341 (﻿*I.pleurocarpa*), 10205, 10682, 12085, 12111, 12115 (﻿*I.thonneri*), 10097 (﻿*L.bella*), 10153 (*Me.oliverianum*), 6195, 14081 (*Monan.bokoli*), 7570, 8738 (*Monan.capea*), 12811 (*Monan.cauliflora*), 3248, 4359, 5071 (*Monan.enghiana*), 2955, 10007 (*Monan.ferruginea*), 10717, 14591 (*Monan.filamentosa*), 10288 (*Monan.hexamera*), 3066, 5403, 15270 (*Monan.letouzeyi*), 8545, 8648 (*Monan.pellegrinii*), 5049 (*Monan.pynaertii*), 14476 (*Monan.submontana*), 13046, 15050 (*Monan.vulcanica*), 14498 (*Mo.crispata*), 2616, 3899, 11474, 13825 (*Mo.myristica*), 4578, 5459, 14426 (*Mo.tenuifolia*), 2373, 3021, 3350, 4486, 9726, 13025 (﻿*N.myristicifolia*), 4092, 9436, 12550 (﻿*N.neurosericea*), 1936, 4011, 10157, 15272, s.n. (*Pi.calophyllum*), 4167, 12415, 13912 (*Pi.glabrescens*), 1937, s.n. (*Pi.macranthum*), 9156, 14590 (*Pi.macrophyllum*), 14535 (*Pi.submontanum*), 14647 (*Po.parviflorus*), 10248 (*Po.pellegrinii*), 12405 (﻿*S.solannona*), 14016 (﻿*U.afzelii*), 7486, 7829, 8565 (﻿*U.angolensis*), 5584 (﻿*U.baumannii*), 12660 (﻿*U.bipindensis*), 12190 (﻿*U.buchholzii*), 4625, 11695, 13994, 13999 (﻿*U.chamae*), 14586 (﻿*U.heterotricha*), 6167, 12936 (﻿*U.muricata*, 10494, 10556 (﻿*U.osmantha*), 10251, 10599, 14884 (﻿﻿*U.scabrida*), 2670, 5529, 10225, 10533 (*Uvtr.pierreanum*), 9121 (*Uvtr.zenkeri*), 12352, 13673, 14020 (*Uvdr.calophyllum*), 15175 (*Uvdr.connivens*), 5142, 10222 (*Uvdr.molundense*), 13841 (*Uvdr.bakeriana*), 9017 (*Uvdr.citrata*), 4755, 5491, 9915, 10024, 10641, 12086 (*Uvdr.congensis*), 4219, 4230, 9541, 9934, 12290, 12580, 13790 (*Uvdr.dioica*), 13414 (*Uvdr.etugiana*), 13849 (*Uvdr.pedunculosa*), 3433, 5456, 8186, 11773 (﻿*X.aethiopica*), 13300, 14551 (﻿*X.africana*), 1374 (﻿*X.aurantiiodora*), 10306 (﻿*X.calva*), 11801 (﻿*X.cupularis*), 10701, 11876 (﻿*X.gilbertii*), 5139 (﻿*X.hypolampra*), 4498 (﻿*X.katangensis*), 4361, 11707 (﻿*X.letestui*), 2261, 2459, 9614, 12138 (﻿*X.longipetala*), 5402 (﻿*X.paniculata*), 5027, 5401, 10418, 12026 (﻿*X.phloiodora*), 12317 (﻿*X.pynaertii*), 5510, 13327 (﻿*X.quintasii*), 1611, 4464 (﻿﻿*X.rubescens*), 1126, 5404, 8178, 11854, 11865 (﻿﻿*X.staudtii*), 2423, 2566, 2824, 7563, 8552, 12797 (﻿﻿*X.thomsonii*), 9524.(﻿*X.villosa*).

Lissambou B.J. 1745, 1748, 1775, 1788, 1807, 1828, 1830, 1855-1856 (﻿*G.glabrum*).

Lolo 6 (*Pi.multinervium*).

Mackinnon L.E. 52 (*Pi.multinervium*), 51 (*Uvdr.dicaprio*).

Maitland T.D. 626, s.n. (*De.tripetala*), 1555, 1596 (﻿*I.congolana*), 566 (*Monan.filamentosa*), 1072 (*Monan.foliosa*), 1618 (*Monan.vulcanica*), 408 (*Mo.undulata*), 536 (*Sp.gracilipes*), 537 (*Uvdr.connivens*), 453, s.n. (*Uvdr.fuscum*), 233, s.n. (﻿*X.africana*), 1731 (﻿﻿*X.thomsonii*).

Malzy P. 309 (﻿*H.monopetalus*).

Mann G. 709 (﻿*H.crispiflorus*), 111 (*Mo.tenuifolia*), 1193 (﻿*X.africana*).

Manning D. 1453 (﻿*I.zenkeri*), 1586 (*Uvtr.pierreanum*).

Mbarga A. 58 (﻿*X.cupularis*).

Mbenkum T.F. 310 (﻿*X.cupularis*).

McKey D.B. 111 (*Ar.congolensis*), 245 (﻿*I.hexaloba*), 103 (*Pi.glabrescens*), 105 (*Pi.goslineanum*), 47 (*Pi.multinervium*), 70 (*Uvdr.bakeriana*), 138 (﻿*X.phloiodora*).

Médou J. SRFK 1703 (﻿*H.salicifolius*).

Meijer D. 15033 (*Ar.aurantiacus*, 15270.(﻿﻿*U.scabrida*).

Mezili P. 193 (*Ar.rufus*), 251 (﻿*N.neurosericea*), 250 (*Pi.mortehanii*).

Mikio K. 5 (*Du.staudtii*).

Mildbraed G.W.J. 7727 (*Ar.congolensis*), 4350, 4999 (*Ar.rufus*), 10640 (﻿*C.patens*), 10515 (*De.tripetala*), 8558 (*Du.confinis*), 4539, 4996, 5652 (﻿*H.crispiflorus*), 4193 (﻿*I.pilosa*), 9010 (*Monan.bokoli*), 6059 (*Monan.diclina*), 5989 (*Monan.filamentosa*), 4286 (*Monan.klainei*), 4199 (*Mo.angolensis*), 5650 (*Mo.crispata*), 4530 (*Mo.myristica*), 8301 (*Mo.tenuifolia*), 8476, 8850 (*Mo.zenkeri*), 5791 (*Pi.calophyllum*), 6118 (*Pi.macranthum*), 9224 (﻿*U.angolensis*), 10795 (*Uvtr.insculptum*), 10720 (*Uvdr.fuscum*), 4373, 5936 (*Uvdr.molundense*), 8260, 10647 (*Uvdr.dioica*), 10745 (*Uvdr.pedunculosa*), 5239 (*Uvdr.sessiliflora*), 8826 (﻿*X.aethiopica*), 8649, 10629 (﻿*X.cupularis*), 5183, 7618, 8827 (﻿*X.hypolampra*), 6055 (﻿*X.mildbraedii*), 6090, 7592 (﻿*X.phloiodora*), 4003, 7613, 8294 (﻿*X.quintasii*), 9069 (﻿﻿*X.thomsonii*).

Mitani M. 134 (*Po.parviflorus*).

Mpom B. 544 (﻿﻿*U.scabrida*), 282 (﻿*X.aethiopica*), 362 (﻿*X.hypolampra*), 121 (﻿*X.phloiodora*), 353 (﻿*X.quintasii*).

Nana P. 213, 257 (*Me.oliverianum*), 98 (*Mo.zenkeri*), 171, 240, 474 (﻿*N.myristicifolia*), 479 (﻿﻿*X.thomsonii*).

Ndam N. 1205 (*Pi.mortehanii*), 1248 (*Uvtr.zenkeri*), 708 (*Uvdr.connivens*).

Ndoum D. 129 (﻿*H.crispiflorus*), 88 (*Me.oliverianum*).

Nemba J. 953 (*Ar.aurantiacus*), 64 (*Uvdr.calophyllum*), 56 (*Uvdr.connivens*), 293 (﻿*X.quintasii*).

Ngameni B.K. 51 (*Anna.senegalensis*), 113 (*Mo.zenkeri*), 44 (﻿*N.myristicifolia*), 109.(*Uvdr.solheidii*).

Nkongmeneck B.A. 256 (*Anna.senegalensis*, 400 (﻿*G.glabrum*), 1326 (*Monan.laurentii*), 518 (﻿*N.myristicifolia*), 310 (﻿*U.angolensis*), 954 (﻿*U.muricata*), 348 (﻿﻿*U.scabrida*), 959 (*Uvdr.connivens*), 891 (*Uvdr.fuscum*), 800 (*Uvdr.molundense*), 273 (*Uvdr.solheidii*), 399 (﻿*X.aethiopica*), 580.(﻿*X.africana*).

Nning J. 385 (﻿*B.fasciculata*), 212, 259 (*Monan.enghiana*), 360 (*Monan.montana*), 284 (*Uvdr.korupensis*).

Nordal I. 929 (*Anna.senegalensis*).

Nzooh Dongmo Z.L. 578 (*Monan.letouzeyi*).

Olorunfemi J. 30760 (*Anki.chlorantha*), 30508 (*Andi.brieyi*), 30662 (﻿*I.campanulata*), 30561 (*Uvdr.calophyllum*).

Onana J.M. 3101 (*Andi.brieyi*), 2027 (*Ar.aurantiacus*, 523 (*Ar.velutinus*), 2030 (﻿*I.hexaloba*), 947 (*Monan.enghiana*), 2870 (﻿*N.myristicifolia*), 3612 (*Sp.gracilipes*), 2848 (*Uvdr.solheidii*), 1825, 1835.(﻿*X.africana*).

Onochie C.F.A. 30860 (*Uvdr.calophyllum*).

Osborne J. 200 (*Uvdr.zenkeri*).

Parmentier I. 1943, 1961 (﻿*I.thonneri*).

Parren M.P.E. 4 (*Ar.insignis*), 157, 212 (*Du.staudtii*), 23 (*Monan.enghiana*), 190 (*Po.parviflorus*), 68, 223 (*Uvdr.calophyllum*).

Preuss P.R. 1303 (*Mo.myristica*), 251 (*Pi.glabrescens*), 1378 (*Uvdr.fuscum*).

Raynal A. 10569 (﻿*N.myristicifolia*), 10389, 13457, 13472 (﻿*N.neurosericea*).

Raynal J. 13392 (*Anki.letestui*), 10240 (*Ar.aurantiacus*), 10195 (﻿*L.bella*), 9560, 9806 (﻿*N.myristicifolia*), 12228 (﻿*X.elliotii*), 10130 (﻿﻿*X.thomsonii*).

Rudatis H. 54 (*Monan.vogelii*).

Sainge M. 558 (*Pi.mayndongtsaeanum*).

Satabié B. 702, 781 (﻿*H.monopetalus*), 767 (*Me.oliverianum*), 978 (*Mi.gabonensis*), 754 (*Mo.angolensis*), 1037 (*Pi.macranthum*), 954 (﻿*U.klaineana*), 873 (*Uvdr.pedunculosa*), 15, 687 (﻿*X.elliotii*).

Sebsebe D. 5035 (*Uvdr.submontana*).

Senterre B. 1641 (﻿*H.crispiflorus*), 1283, 1370 (*Uvtr.pierreanum*).

Service Forestier du Cameroun 32 (﻿*C.staudtii*).

Sonké B. 591 (﻿*H.crispiflorus*), 2671 (﻿*N.myristicifolia*), 1173 (*Pi.longepilosum*), 1226 (*Uvdr.molundense*), 1505 (*Uvdr.dioica*).

SRFK 1372 (﻿*C.glauca*).

Staudt A. 133, 138 (*Du.staudtii*), 40, 495 (*Mo.undulata*), 740, 900 (*Uvtr.insculptum*), 642, 742 (*Uvdr.angustifolium*), 556 (*Uvdr.congensis*), 530 (﻿﻿*X.staudtii*).

Tadjouteu F. 566 (*Uvdr.molundense*).

Tchiengue B. 2816 (*Monan.filamentosa*), 2555 (*Pi.multinervium*), 2204 (*Uvdr.connivens*).

Tchinaye V. 103 (﻿*X.longipetala*).

Tchouto Mbatchou G.P. BIFAX_150 (*Anki.letestui*), 3129 (*Andi.mannii*), BIFAX_2, 2855, 3009 (﻿*I.zenkeri*), 3166 (﻿*L.lanuginosa*), CORIX_3, 2986, 3035, 3298 (﻿*N.neurosericea*), 613 (*Pi.longepilosum*), 3100 (*Pi.macranthum*), 1053, 1378 (*Pi.macrophyllum*), 1372, 3400 (*Sp.gracilipes*), 548 (﻿*U.anonoides*), 3238, 3404 (﻿*U.baumannii*), ONOX_53 (﻿*U.bipindensis*), 167 (﻿*U.comperei*), 2721, 2819 (﻿﻿*U.scabrida*), 1108 (*Uvtr.zenkeri*), 70, 136, 611 (*Uvdr.molundense*), 1663 (*Uvdr.bakeriana*), 2869 (*Uvdr.citrata*), BIFAX_25, 675 (*Uvdr.korupensis*), ELEX_15, ONOX_182, ONOX_274, 3242 (*Uvdr.pedunculosa*), ONOX_191, 2843, 3089 (*Uvdr.solheidii*), 2846, 3252 (*Uvdr.zenkeri*), 3126 (﻿*X.aethiopica*), 1756 (﻿*X.cupularis*), 896, 942 (﻿﻿*X.thomsonii*).

Tekwe C.F. 49 (﻿*U.anisotricha*), 87 (*Uvdr.connivens*).

Tessmann G. 767 (﻿*C.myristiciflora*), 760 (﻿*X.quintasii*), 644 (﻿﻿*X.staudtii*), 747 (﻿*X.talbotii*).

Thomas D.W. 6327, 7494 (*Anki.affinis*), 7555 (*Anki.chlorantha*), 2188 (*Andi.brieyi*), 2850 (*Andi.mannii*), 7578 (*Ar.insignis*), 4200, 6121 (﻿*C.glauca*), 2329 (﻿*C.patens*), 3271 (﻿*C.staudtii*), 5661 (*De.tripetala*), 1237 (*Du.confinis*), 384 (*Du.staudtii*), 2432 (﻿*H.monopetalus*), 4763, 7354 (﻿*I.campanulata*), 349 (﻿*I.pleurocarpa*), 4324 (*Monan.cauliflora*), 4721 (*Monan.foliosa*), 5274 (*Monan.submontana*), 2118 (*Mo.myristica*), 7750 (*Mo.tenuifolia*), 3025, 4516, 4814 (*Mo.undulata*), 6891, 7554, 7809 (﻿*N.neurosericea*), 510 (*Pi.goslineanum*), 6906 (*Pi.longepilosum*), 4493, 7645 (*Pi.macrophyllum*), 5840 (*Pi.mayndongtsaeanum*), 1110 (*Pi.mortehanii*), 4311, 4755 (*Pi.pilosum*), 10496 (*Pi.submontanum*), 4785 (*Po.parviflorus*), 5727, 7858 (*Sp.gracilipes*), 9802 (﻿*U.obanensis*), 604, 4334, 8094, 9772 (*Uvtr.zenkeri*), 6087, 7018 (*Uvdr.angustifolium*), 3322, 4549, 5965, 6090, 7499 (*Uvdr.calophyllum*), 4447, 5537, 6928, 9875 (*Uvdr.connivens*), 4469 (*Uvdr.fuscum*), 1086, 3336, 4300, 5606, 7835 (*Uvdr.bakeriana*), 4544 (*Uvdr.etugiana*), 3182, 3210, 4477 (*Uvdr.korupensis*), 2756, 7364 (*Uvdr.pedunculosa*), 3455, 7372 (*Uvdr.zenkeri*), 2981, 3305, 4554 (﻿*X.africana*), 4558, 7400 (*X.monticola*), 3463, 7401 (﻿﻿*X.staudtii*), 3204 (﻿*X.talbotii*), 595, 3242, 7281 (﻿﻿*X.thomsonii*), 7703 (﻿*X.villosa*).

Tisserant C. 3651 (*Ar.aurantiacus*).

Ujor E.U. 29281 (*Uvdr.calophyllum*).

Vaillant A. 15 (*Anna.senegalensis*).

van Andel T.R. 3784 (*Anki.affinis*), 3882, 4216 (*Anki.letestui*), 4128 (*Ar.dielsianus*), 3459, 3872 (*Ar.thomsonii*), 3732 (﻿*B.fasciculata*), 3846 (﻿*C.glauca*), 3810 (*Du.barteri*), 4205 (*Du.confinis*), 3290 (*Du.staudtii*), 3794 (﻿*G.suaveolens*), 4177 (﻿*I.pleurocarpa*), 3619, 3931 (*Me.oliverianum*), 3728 (*Pi.macrophyllum*), 3707 (*Po.parviflorus*), 4095 (*Sp.gracilipes*), 3761 (*Uvdr.fuscum* var. giganteum), 4228 (*Uvdr.molundense*), 3951 (*Uvdr.solheidii*), 3343 (﻿*X.aethiopica*), 3345 (﻿*X.quintasii*), 4023 (﻿﻿*X.thomsonii*).

van der Burgt X.M. 219 (﻿*C.patens*), 232 (﻿*H.crispiflorus*), 791 (﻿*I.hexaloba*), 614 (*Monan.gracilis*), 689, 790 (*Pi.mayndongtsaeanum*), 675 (*Po.parviflorus*), 590, 674 (*Uvtr.zenkeri*), 364 (﻿*X.hypolampra*), 1129 (﻿*X.letestui*).

van der Zon A.P.M. 1122 (*Ar.velutinus*), 2542 (﻿*U.chamae*).

van Velzen R. 90 (﻿*H.crispiflorus*), 98, 99 (﻿*H.salicifolius*).

Villiers J.-F. 2441 (*Ar.congolensis*), 4713 (﻿*H.monopetalus*), 1448 (*Pi.goslineanum*), 625 (*Uvdr.molundense*), 683 (*Uvdr.congensis*), 2429 (*Uvdr.dioica*), 1427 (*Uvdr.pedunculosa*), 893 (*Uvdr.solheidii*), 2490 (*Uvdr.submontana*), 666 (﻿*X.longipetala*).

Vroumsia T. 116 (*Anna.senegalensis*).

Waterman P.G. 874 (*Du.barteri*), 879 (*Du.staudtii*), 830 (﻿*I.campanulata*), 840 (*Monan.cauliflora*), 801 (*Pi.goslineanum*), 880 (﻿*X.phloiodora*).

Watts J. 1104 (*Pi.longepilosum*), 627 (*Po.microtrichus*), 336 (*Uvdr.connivens*).

Westphal E. 9932 (*De.tripetala*), 10012 (﻿*I.congolana*), 9149, 9883, 10173, 10204 (*Mo.myristica*), 10047-10049, 10172 (﻿*X.elliotii*).

Wheatley J.I. 644 (*Ar.congolensis*), 501 (*Mo.crispata*), 814 (*Pi.longepilosum*), 326 (*Uvdr.connivens*), 605 (﻿*X.africana*).

Wieringa J.J. 2327 (﻿*I.hexaloba*), 5898 (*Monan.enghiana*), 2030 (*Pi.pilosum*), 2058 (*Uvdr.fuscum*), 2029 (*Uvdr.dioica*).

Winkler H. 909 (*Uvdr.dioica*).

Wit P. 2955 (﻿*U.chamae*).

Zenker G.A. 576, 2980, 3023, 2102a (*Af.discostigma*), 3839 (*Anki.affinis*), 726 (*Anki.chlorantha*), 690 (*Ar.aurantiacus*), 510, 2087 (*Ar.dielsianus*), 2801, 3320 (*Ar.insignis*), 3834 (*Ar.jacquesfélicis*), 489, 697 (*Ar.thomsonii*), 231, 1222 (*Ar.velutinus*), 2264, 2454, 2495, 4669, 4880 (﻿*C.staudtii*), 3195 (*Du.confinis*), 1278, 2062, 2166 (﻿*G.suaveolens*), 2889, 3550, 3592, 3889, 4831 (﻿*H.bussei*), 2268, 3330 (﻿*H.salicifolius*), s.n. (﻿*I.cooperi*), s.n. (﻿*I.hexaloba*), 22, 95, 267, 1716, 3038, 3217, 3433, 3540, 3921, 4704 (﻿*I.pleurocarpa*), 1186, 3375, 3471, 4405 (﻿*I.zenkeri*), 60, 1864, 2505, 2521, 2947, 3221, 4209 (*Me.oliverianum*), 356, 3898 (*Monan.cauliflora*), 2102 (*Monan.diclina*), 2473 (*Monan.dielsiana*), 132, 199, 1321, 2693, 4000, 4477, s.n. (*Monan.elegans*), 357 (*Monan.enghiana*), 2985 (*Monan.filamentosa*), 2050, 3001 (*Monan.foliosa*), 2977 (*Monan.klainei*), 431a (*Monan.montana*), 3495a (*Monan.zenkeri*), 3884, 3935c (*Mo.crispata*), 1938, 2251, 2727, 3793, 4317 (*Mo.tenuifolia*), 776 (*Mo.zenkeri*), 29, 440, 440, 1904, 2877, 3105, 3749, 3819, 3897, 4402 (﻿*N.neurosericea*), 505, 2396, 3654, 4006, 4472 (*Pi.glabrescens*), 1075 (*Pi.longepilosum*), 2528, s.n. (*Pi.macranthum*), 21, 2079, 2263, s.n. (*Pi.multinervium*), 478, 2899, 3270 (*Po.microtrichus*), 360, 516, 1715 (*Sp.gracilipes*), 1116 (﻿*U.bipindensis*), 597, 4926 (﻿*U.buchholzii*), 3, 249, 475 (﻿*U.mollis*), 481, 2438, 2935, 3248, 3289, 3409, 4473 (*Uvtr.zenkeri*), 1738, 2344, s.n. (*Uvdr.calophyllum*), 358, 2624, 3204 (*Uvdr.connivens*), 108, 698, 1438 (*Uvdr.fuscum* var. giganteum), 3971 (*Uvdr.korupensis*), 3868 (*Uvdr.pedunculosa*), 515, 1117, 3045, 3228 (*Uvdr.zenkeri*), 4747 (﻿*X.calva*), 359, 408, 580, 2080, 2094, 2655, 2663, 4096, 4738 (﻿*X.quintasii*), 2827 (﻿﻿*X.rubescens*), 246, 3653, 3953, 4862 (﻿﻿*X.staudtii*).

## Supplementary materials

Supplementary material 1 Figure S1 Data type Figure (tiff. image) Explanation note Figure S1. Distribution of protected areas in Cameroon, and represented across the species distribution maps (green). 1. Bakossi (NP); 2. Barombi Mbo Crater Lake (Ramsar Site, WII); 3. Bayang-Mbo (Wildlife Sanctuary); 4. Bouba Ndjida (NP); 5. Boumba Bek (NP); 6. Bénoué (NP); 7. Campo-Ma’an (NP); 8. Deng Deng (NP); 9. Dja (UNESCO-MAB Biosphere Reserve); 10. Douala Edea (NP); 11. Ebo (NP); 11. Estuaire du Rio Del Rey (RS-WII); 13. Faro (NP); 14. Kagwene (WS); 15. Kalamaloué (NP); 16. Kilum Ijim, Mont Oku (FS); 17. Kimbi-Fungom (NP); 18. Kom (NP); 19. Korup (NP); 20. Lac Ossa (Faunal Reserve); 21. Lobéké (NP); 22. Ma Mbed Mbed (NP); 23. Mbam et Djerem (NP); 24. Mefou (NP); 25. Mengame (WS); 26. Mont Cameroun (NP); 27. Mozogo Gokoro (NP); 28. Mpem et Djim (NP); 29. Ngoyla (Faunal Reserve); 30. Nki (NP); 31. Parc National de Waza (UNESCO-MAB Biosphere Reserve); 32. Fleuve Ntem (RS-WII); 33. Rumpi Hills (WS); 34. Santchou (Faunal Reserve); 35. Takamanda (NP); 36. Tchabal Mbabo (NP); 37. Tofala Hill (WS); 38. Vallée du Mbéé (NP); 39. Waza (NP); 40. Waza Logone Floodplain (RS-WII); 41. Zone Humide d’Ebogo (RS-WII). Abreviations. Ramsar Site, Wetland of International ImportancE RS-WII; NP. National Park; Wildlife Sanctuary. WS; Flora Sanctuary. FS. https://binary.pensoft.net/file/745621This dataset is made available under the Open Database License (http://opendatacommons.org/licenses/odbl/1.0/). The Open Database License (ODbL) is a license agreement intended to allow users to freely share, modify, and use this Dataset while maintaining this same freedom for others, provided that the original source and author(s) are credited.Thomas L. P. Couvreur

Supplementary material 2 Distribution data used for the flora of Cameroon - Annonaceae Data type Occurrences (excel file) Explanation note List of herbarium specimens with identification and geolocalization. https://binary.pensoft.net/file/745622This dataset is made available under the Open Database License (http://opendatacommons.org/licenses/odbl/1.0/). The Open Database License (ODbL) is a license agreement intended to allow users to freely share, modify, and use this Dataset while maintaining this same freedom for others, provided that the original source and author(s) are credited.Thomas L. P. Couvreur, Jean-Paul Ghogue, Leo-Paul M. J. Dagallier
