# Supplementary figures and images for: ﻿Flora of Cameroon – Annonaceae Vol 45
Source: PhytoKeys. 2022 Sep 20;207:1–532. doi: 10.3897/phytokeys.207.61432 (PMC9849070; doi:10.3897/phytokeys.207.61432)

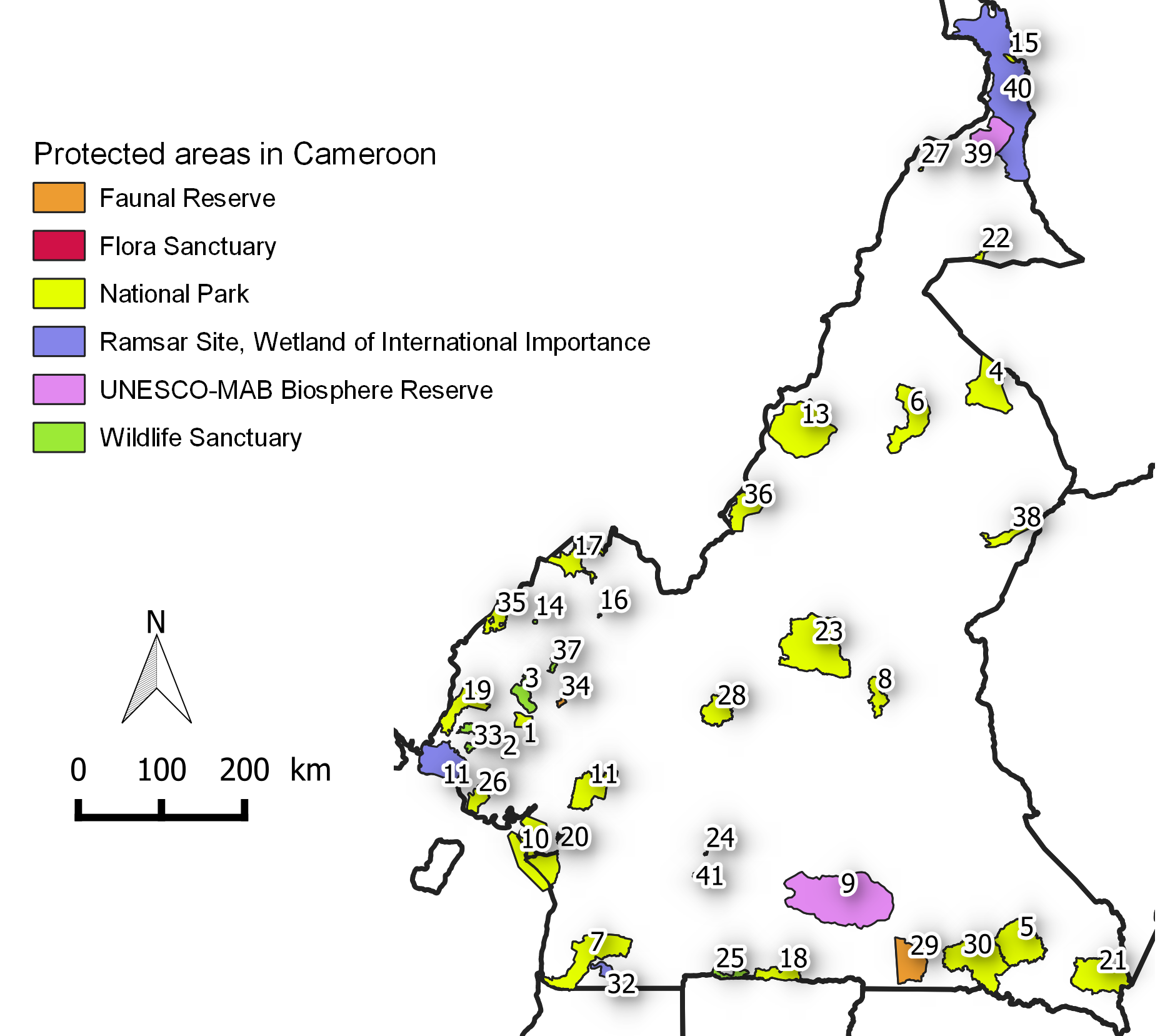

Supplement: Supplementary material 1 — Figure S1 [file phytokeys-207-001_article-61432__-s001.tiff]
